# Molecular Interactions Associated with Coagulation of Organic Pollutants by 2S Albumin of Plant Proteins: A Computational Approach

**DOI:** 10.3390/molecules27051685

**Published:** 2022-03-04

**Authors:** Victoria T. Adeleke, Nkosinathi E. Madlala, Adebayo A. Adeniyi, David Lokhat

**Affiliations:** 1 Discipline of Chemical Engineering, University of KwaZulu-Natal, Howard Campus, Durban 4041, South Africa; nkosinathiemmanuelmadlala@gmail.com (N.E.M.); lokhat@ukzn.ac.za (D.L.); 2 Department of Chemistry, Faculty of Natural and Agricultural Sciences, University of the Free State, Bloemfontein 9301, South Africa; ade4krist@yahoo.com; 3 Department of Industrial Chemistry, Federal University, Oye Ekiti 370111, Nigeria

**Keywords:** molecular dynamic simulations, organic molecules, protein quality, mechanisms, protein-ligand interaction, bioremediation

## Abstract

The removal of organic pollutants is a major challenge in wastewater treatment technologies. Coagulation by plant proteins is a promising technique for this purpose. The use of these proteins has been experimentally investigated and reported in the literature. However, the determination of the molecular interactions of these species is experimentally challenging and the computational approach offers a suitable alternative in gathering useful information for this system. The present study used a molecular dynamic simulation approach to predict the potentials of using *Moringa oleifera* (MO), *Arachis hypogaea*, *Bertholletia excelsa*, *Brassica napus,* and *Helianthus annuus* plant proteins for the coagulation of organic pollutants and the possible mechanisms of coagulation of these proteins. The results showed that the physicochemical and structural properties of the proteins are linked to their performance. Maximum coagulation of organic molecules to the proteins is between 50–100%. Among five proteins studied for coagulation, *Brassica napus* and *Helianthus annuus* performed better than the well-known MO protein. The amino acid residues interacting with the organic molecules play a significant role in the coagulation and this is peculiar with each plant protein. Hydrogen bond and π—interactions dominate throughout the protein–pollutants molecular interactions. The reusability of the proteins after coagulation derived from their structural quality analysis along with the complexes looks promising and most of them are better than that of the MO. The results showed that the seed proteins studied have good prediction potentials to be used for the coagulation of organic pollutants from the environment, as well as the insights into their molecular activities for bioremediation.

## 1. Introduction

Waste treatment technologies are crucial to the modern world as population growth poses a serious problem to the environment, particularly in terms of pollution. According to the UN sustainable development goals [1], every human being is responsible for the environment surrounding him/her. The increase in industrialization and development of new technologies correlates with the complexity of waste streams reaching the environment, contaminating the soil, rivers, and lakes. Coagulation mechanisms are well established in water purification studies to remove organic and inorganic contaminants [2,3]. Different biocoagulants are used in water purification based on their economic importance and effectiveness. Organic pollutant removal requires coagulants with better exploitation of bio-molecular properties, e.g., natural coagulants derived from plants. As an example, the 2S albumin protein derived from certain plant species has shown potential in flocculation/coagulation studies [4,5]. The 2S albumins are protein entities commonly found in edible seeds of mono and dicotyledonous plants, comprising amino acids [6]. Several components of protein such as 2S, 7S, and 11S are present in most plants [7]. The solubility of albumin protein in water, the flexibility of structure, its flocculation, electrostatic and bridging behavior distinguish it amongst other proteins [8]. Approximately 20% to 60% of the soluble proteins are 2S albumin protein if the protein content is not expressed in total seed protein [6]. Most literature favors the 2S albumin protein as the preferred protein for water quality improvement [9]. *Moringa oleifera* proteins have been extensively studied for flocculation/coagulation activities in wastewater treatment [10]. There are other natural plants that are widely used due to their high nutritional attributes [11] and also widely distributed due to their economic importance. For instance, *Brassicas napus* is one of the economic crops in China, Europe, Australia, and North America [12]. Unfortunately, most of these plants have not been fully utilized as agents of wastewater treatment though they have the tendencies [13].

Sunflower (*Helianthus annuus*) and Mustard (*Brassica nigra*) possess a high protein content of 2S, approximately 62% than other protein fractions [8]. In *Helianthus annuus* (crude protein 18.40–21.70% [14]), the presence of high phenolic compounds and the development of disulfide bonds enables its use as a flocculant [15]. *Brassicas napus*, the edible protein from oilseed consists of polypeptide chains with protein content ranging from 24% to 45% are regarded as low molecular weight proteins [16]. *Arachis hypogaea L*. (commonly known as the groundnut) protein fraction consists of lysine, glycine, methionine with a very low content of aspartic and glutamic acid [8]. It is also mentioned in the literature that the overall protein content is 25.73–48.78% [17]. *Bertholletia excelsa*, the Brazilian nut, which is rich in protein as well (16.27%) [18], has been shown through extraction and fractionation to contain 70% lipid and 17% protein content [8]. The high lipid composition of *Bertholletia excelsa* limits its applicability as a coagulant but this can be resolved through dilapidation prior to use [13].

Computational chemistry and molecular modeling are widely used to predict the structure and properties of bio-molecules behavior. During food protein processing under certain techniques, such as X-ray diffraction and nuclear magnetic resonance, there are challenges in observing the protein conformational changes during the process [19]. Molecular dynamic simulation is a notable tool for visualization and analyzing the process [20] and also to analyze the aptitudes of the protein molecule to transform in a given type of environment. With computational chemistry and its ability to represent complex interactions of adsorbate with adsorbent at an atomic level, it is possible to visualize and analyze the dynamic transformation during the process [21]. Through molecular mechanisms, the interaction of atoms is defined by the bond angle and torsions of the atoms or molecules [22]. Natural coagulants such as proteins from vegetables and legumes have the potential for wastewater remediation since they are cost-effective and safe [9]. These plant proteins exhibit much greater potential as coagulants for the removal of organic contaminants than the simple use of biomass waste-derived sorbents [23]. They can be cultivated at a low cost, extracted, and used [11]. The residue potentially contains sufficient nutritional value for further use as well [24]. Experimental evidence of coagulating activities of some of these plant proteins are available [25,26,27] but the information on the mechanism and molecular interactions involved are not fully documented. This paper will be exploring the power of molecular dynamic simulation in predicting the behavior and mechanism of 2S albumin plant proteins in the coagulation of organic molecules discarded into water streams. The 2S albumin proteins from *Moringa oleifera* and other underexploited plants such as *Arachis hypogaea*, *Bertholletia excelsa*, *Brassica napus,* and *Helianthus annuus* will be investigated for their efficiency as a coagulant in contaminant removal. The organic pollutants selected for this study represent different classes of organic molecules that are widely used and detrimental to the environment [28,29,30,31]. They are dichlorvos (pesticide), naphthol blue black (dye), sodium lauryl sulphate (surfactant), and sulfamethoxazole (antibiotic).

## 2. Materials and Methods

**Figure 1:**
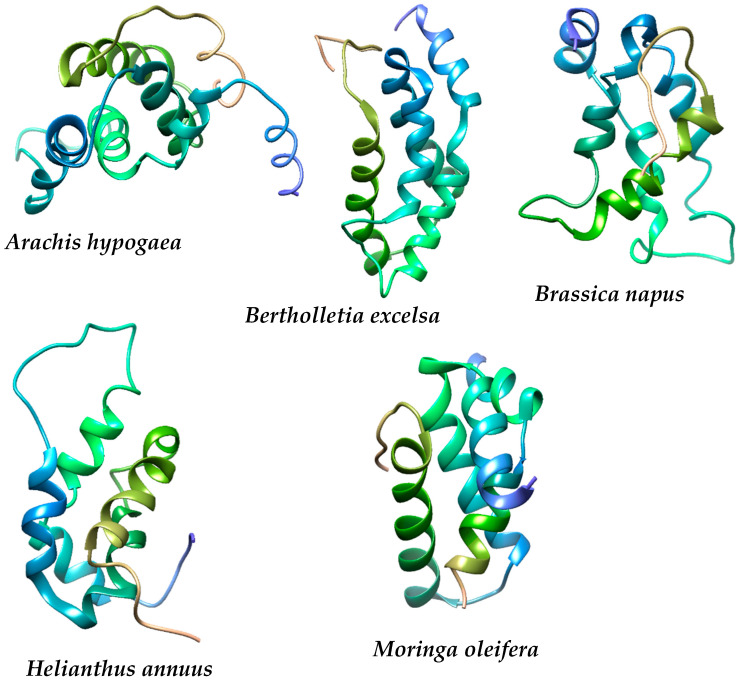
The 2S albumin of plant proteins for coagulation study.

The structures of dichlorvos (Dic), naphthol blue black (Nbb), sodium lauryl sulphate (Sls), and sulfamethoxazole (Sum) were retrieved from PubChem. The retrieved structures were optimized in the Gaussian 16 package of programs [32] using the DFT method B3LYP and basis set 6–311G.(d,p). The crystal structure (Figure 1) of the 2S albumin plant seed proteins were retrieved from protein data bank with PDB ID 6S3F for *Moringa oleifera* (MO) [33], 1W2Q for *Arachis hypogaea* (AH) [34], 2LVF for *Bertholletia excelsa* (BE) [35], 1SM7 for *Brassica napus* (BN) [36] and 1S6D for *Helianthus annuus* (HA) [37]. The physicochemical properties of the proteins were determined by the ProtParam tool [38], structural composition by SOPMA [39], Z score by ProsA-web [40], Errat [41], and Ramachandran plot by PROCHECK [42]. The proteins were prepared using UCSF Chimera [43] by selecting only the protein and every other molecule was stripped off and then prepared using the Dock Prep tool in Chimera.

## 3. Molecular Dynamics Simulation

The previously described method [44] was used and detailed as follows. Prior to the MD simulation, 10 molecules of each of the pollutants were added to each protein molecule using the “AddToBox” function available in AMBER. The number of atoms subjected to MD simulation by each system is presented in Supplementary Tables A–T. The AMBER 18 package [45] with the Antechamber module using the general AMBER force field (GAFF) [46] was used for the ligand preparation, while FF14SB [47] was used for protein preparation. The initial preparation was achieved by generating topologies for the systems in which the TIP3P water box at 8 Å was used as the explicit solvent medium [48]. Following this, each system was subjected to 500 steepest descents with 9500 conjugate gradient initial minimization steps with strong constrains on both the ligands and the protein. The full minimization of 2000 steps without constraint was thereafter followed. The heating was set for the systems at temperature 0–300 K in a canonical ensemble (NVT) of MD simulation for 2 ns via Langevin thermostat allowing a collision frequency of 1.0 ps^−1^ [49] followed by the density of the water system controlled with 4 ns of NVT simulation. The entire system was equilibrated at 300K for another 2 ns at a pressure of 1 bar. The MD production was run for 100 ns of NPT (constant number N, pressure P, and temperature T) using the GPU version of the PMEMD and CUDA provided with the AMBER 18 package. The analyses of the generated MD trajectories were carried out using PTRAJ and CPPTRAJ modules in AMBER 14 [50]. The structural stability and flexibility were analyzed by root mean square deviation (RMSD), root mean square fluctuations (RMSF), solvent accessible surface area (SASA), and a number of hydrogen bonds. Binding free energy was calculated for the protein-ligand complexes using molecular mechanics/generalized born surface area (MM/GBSA) with a total of 100,000 snapshots from 100 ns. Per residue energy decomposition analyses were performed to know the contribution of individual amino acid residue. The binding free energy calculation was based on the Formula (1):

(1) $$\Delta G_{\mathrm{bind}}={\;G}_{\mathrm{complex}}-(G_{\mathrm{receptor}}+{\;G}_{\mathrm{ligand}})$$

## 4. Results and Discussion

### 4.1. Physicochemical Properties, Structural Composition, Quality and Stability of the Proteins under Study

**Figure 2:**
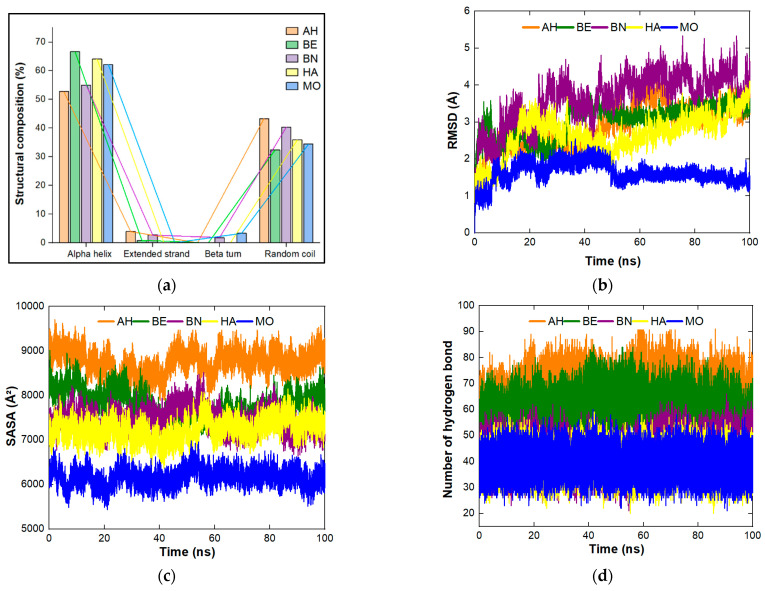
(**a**) Structural composition of the proteins under study. The stability of the proteins without the ligands at 100 ns simulation time derived from the plot of (**b**) RMSD (Å), (**c**) SASA (Å^2^), and (**d**) number of hydrogen bonds.

The analysis of properties was carried out to get more insights into the behaviors of the proteins and how this can influence their performance. The results presented in Table 1 showed that the amino acid length of the proteins ranged between 90 to 127 and their molecular weights between 10.39 and 14.96 kDa. Glutamic and aspartic acid were reported to be the two dominant amino acid residues of legumes [51] with isoelectric point (PI) value below 3.3 while lysine, arginine, and histidine were reported to be the charge neutralization side chains amino acid residues with PI > 7.6 [13]. The composition of these amino acid residues by the five proteins and their PI are presented in Table 2. The theoretical PI is between 5.24 and 11.61. The calculated PI of *Arachis* *hypogaea* (AH), *Helianthus annuus* (HA), and *Bertholletia excelsa* (BE) are towards the acidic side while that of *Brassica napus* (BN) and *Moringa oleifera* (MO) are towards the basic side. The least number of negatively charged residues (Asp + Glu), which is zero, is found in MO; the maximum (21) is found in AH. The least number of positively charged residues (Arg + Lys), which is 11, is found with BN and HA, and the maximum (18) is found with AH. The composition of amino acid residues plays a major in the isoelectric points of proteins [52]. It was reported that the leguminous proteins function maximumly at a pH of 7.0–8.5 for coagulation activities [13]. As observed in Table 2, MO protein lack residues Asp, Glu, and Lys that could be used to balance the isoelectric point to attain the optimum value fitted for coagulation activities. The instability index (a value < 40 predicts protein stable and >40 predicts protein unstable in vitro) is ranged between 59.38 and 81.42 indicating the proteins may be unstable and the most favorable protein is *Brassica napus* (59.38). Moreover, since the instability index values show that the proteins may be unstable, there will be a need to determine the suitable conditions for the protein preparation and storage during experimental investigation and application [53,54]. The aliphatic index (which measures the thermostability, the more positive a protein, the more thermostable it is) is between 27.46 and 61.74 indicating that the proteins may be thermodynamically stable and *Brassica napus* (61.74) is still the most favorable one. There are other factors, such as the amino acid structure, as well as the position of the aliphatic amino acid, that need to be considered concerning protein stability [55,56]. The grand average of hydropathicity, GRAVY (positive values mean hydrophobic while negative values mean hydrophilic) showed that the proteins are hydrophilic with values within −1.216 and −0.586, which shows that the proteins will be soluble in water. The secondary structural composition of the proteins is presented in Figure 2a. The major composition identified for the proteins is alpha helix, extended strand, beta turn, and random coil. The decreasing order for alpha helix follows BE > HA > MO > BN > AH. The highest composition for extended strand was found with AH (3.94%) while the least was found with BE (0.88%). HA and MO do not contain extended strands. Moreover, beta turn was not recorded for AH, BE, and HA while 1.83 and 3.33% were reported for BN and MO respectively. The decreasing order for random coil follows AH > BN > HA > MO> BE. The quality of the proteins alone without the pollutants was analyzed after the simulation and this was based on the Z score, Errat, and Ramachandran plot. The decreasing order of protein quality (Table 1) follows MO > HA > BN > BE > AH. The stability of protein alone without the pollutants after simulation was analyzed for root mean square deviation (RMSD), solvent accessible surface area (SASA), and hydrogen bonding (Figure 2a–d and Figure S1a–c for individual plots). As presented in Figure 2b, the RMSD result showed that MO attained stability after 50 ns, AH after 70 ns, BE after 50 ns, BN after 50 ns, and HA was not able to attain stability at the end of 100 ns. The increasing order of fluctuations throughout the simulation time follows MO < HA < BE < AH < BN. This means that MO is more stable than the other proteins. For SASA (Figure 2c), the decreasing order follows AH > BE > BN > HA > MO. The SASA trend follows the decreasing order of amino acid length displayed in Table 1. The decreasing order for the number of hydrogen bonds (Figure 2d) follows AH > BE > BN > HA ≥ MO.

### 4.2. Coagulation of the Organic Molecules to the Proteins under Study: Binding Affinity, Stability and Structural Quality of the Complexes

Experimental studies on the coagulation ability of extracted proteins from some promising plants have been investigated [27] while many other plants have not been exploited. Moreover, limited information is available on the molecular interactions taking place during coagulation study using theoretical modeling. Moreover, the information on the mechanistic understanding of coagulation on experimentally available ones is limited. In addition, the coagulation potentials of not yet exploited proteins such as BE, BN, and HA, as well as AH that has limited information are predicted through the power of computational modeling. In the present investigation, MD simulation was employed to obtain detailed information on the coagulation mechanism of 2S albumin of five plant proteins interacting with four organic molecules as model pollutants. In this regard, 10 molecules of each of these organic molecules were randomly placed around each of the proteins under investigation in an aqueous medium to study their interactions with the organic molecules as detailed in the method section. The 100 ns simulation time was used to explain the interactions between the proteins and the organic molecules. The amino acid residues of the proteins that interacted with the organic molecules are represented in Table S1 and the types of interaction involved with the percentage of the ligands coagulated are shown in Table 3.

### 4.3. Moringa Oleifera

The interactions taking place between *Moringa oleifera* (MO) and the organic molecules give rise to MO-Dic, MO-Nbb, MO-Sls, and MO-Sum complexes. According to Table 3, the observed percentage of the ligands coagulated to MO is 100% (10 out of 10) for Dic, 90% (9 out of 10) for Nbb, 50% (5 out of 10) for Sls and 70% (7 out of 10) for Sum. The number of coagulated Sls and Sum to MO is low compared to the number of coagulated Dic and Nbb. From energy contributions in Table 4, polar solvation and solvation free energies seem to favor the coagulation of Dic by MO while van der Waals, electrostatic, non-polar solvation and gas phase energies favor the coagulation of Nbb. The observed percentages of coagulated ligands in this study are similar to the one reported theoretically for some organic pollutants around the same simulation period (100 ns) for MO [44]. A percentage of 50–70% at 90–120 ns was recorded for hexazinone which was lower than the ones recorded for the other organic molecules in the same study. An experimental study reported 80% coagulation for Sls [25], there different factors such as concentrations of both the coagulant and the organic molecules were put into consideration. Initial Sls concentrations had an influence on the Sls removal by MO. Another study was carried out on the removal of multiclass antibiotics by MO in which Sum was included [57]. Removal of Sum up to 90% at a concentration of 0.1 mg/L was observed whereas, at the higher concentrations of 1.0 mg/L, less than 60% removal was observed. A higher percentage removal was experimentally also observed for Nbb by MO though the seed pods were used in this case [26]. The experimental reports of coagulation of Dic to MO are not available, it seems this is the first time of predicting its coagulation to MO proteins. In general, the levels of coagulation of the organic molecules to the MO seed protein are not too far from the previous experimental reported ones. The types of interaction taking place are presented in Table S1, as well as the specific amino acid residues associated with the types of interaction observed. The results of binding energy analysis (Table 4) calculated through MM/GBSA, which represent the average total values over 100 ns showed the decreasing order as MO-Sls > MO-Nbb > MO-Dic > MO-Sum. The binding energy range (−112.60–−75.27 kcal/mol) recorded here is within the range (−149.88–−51.24 kcal/mol) recorded for organic pollutants interacting with MO seed proteins [44]. The binding energy from the energy decomposition profile for the amino acid residues for all the complexes ranged between -5.160 to −1.039 kcal/mol (binding energy cut-off of ≤−1.0 kcal/mol). Amino acid residues TRP 88 (−2.884 kcal/mol), PHE 65 (−5.072 kcal/mol), TRP 88 (−3.605 kcal/mol), and ARG 83 (−5.160 kcal/mol) showed the best binding affinity for Dic, Nbb, Sls, and Sum, respectively.

The RSMD result (Figures S2a and S3a) showed that the presence of the organic molecules caused more fluctuation in the system during the simulation except for the MO-Sls complex which showed a level of stability even more than MO alone without the ligand. The decreasing order of stability follows MO-Sls > MO > MO-Sum > MO-Nbb > MO-Dic, which means MO-Sls is the most stable complex in terms of RMSD. The RMSF of the C alpha atom calculated across all the residues showed that the overall protein flexibility of MO was altered by the presence of the pollutants (Figures S2b and S3b). The plot of the number of hydrogen bonds (Figures S2c and S3c) showed a higher number for the complexes compared to that of the MO alone except for MO-Dic which is almost the same as that of MO without the ligands. The increasing order follows MO ≤ MO-Dic < MO-Nbb ≤ MO-Sls ≤ MO-Sum. The compactness of the hydrophobic core which was measured by SASA (Figures S2d and S3d) showed the increasing order of MO-Nbb < MO-Sls < MO-Sum < MO-Dic < MO. The SASA result agrees to some extent with the result of amino acid depicted in Table S2. The levels of stability reported here for MO alone without the ligands in comparison with those of the complexes are similar to those observed previously [44]. The structural quality of the complexes derived from the Z score (which measures the deviation of the total energy, the more negative the better the protein structure), Errat (the overall quality factor of a protein structure), and Ramachandran plot is depicted in Table 1. The MO without the pollutants showed good structural quality compared to the complexes, which implies the possibility of reusing the protein after coagulation is low. The decreasing order of the quality of the complexes follows MO-Dic > MO-Sls > MO-Nbb > MO-Sum.

One of the major concerns in MD simulation methods is the importance of the replicas [58,59]. Some of the factors that are known to cause differences in the molecular dynamics simulation results are the initial random velocities, the number and type of processors, compiling options, dynamic linking to different versions of shared libraries, difference in force fields and some others [59,60,61]. In the present study, the reproducibility of the method was carried out by running MO-Dic in duplicate with the results presented in Figures S4a–d, S5 and Table S7. The values of the binding free energy of the first (−88.68 kcal/mol) and replicated run (−85.60 kcal/mol) show a deviation of −3.08 kcal/mol. Due to the large sample size in the present study, only MO-Dic was used in this context. The reason for choosing MO-Dic for our replica study is basically due to its lower number of residues compare to other systems which simply means it will be less computationally expensive and save time. There is no appreciable difference observed in the results presented for the MO-Dic_1 (first run) and MO-Dic_2 (second run). Therefore, it can be concluded that the results of the MD simulations are consistent with minimal difference in the values.

### 4.4. Arachis Hypogaea

The possibility of using *Arachis hypogaea* (AH) for the treatment of wastewater has been investigated experimentally though the performance was very low compared to that of MO [27]. The present study used a computational approach to investigate the potentials of AH for the removal of organic pollutants. The molecular interactions between AH and the organic molecules give rise to AH-Dic, AH-Nbb, AH-Sls, and AH-Sum complexes. Table 3 presented the observed predicted maximum removal of the ligands by the AH as 70% (7 out of 10) for Dic, 90% (9 out of 10) for Nbb, and 60% (6 out of 10) for Sls and Sum. Nbb is the most coagulated molecule to AH which might be attributed to the stronger energy contributions (Table 4) from van der Waals, electrostatic, non-polar solvation, and gas phase energies of AH-Nbb. As depicted in Table S1, a hydrogen bond is observed to be the dominating interaction for AH-Dic, AH-Nbb, AH-Sls, and AH-Sum complexes with the existence of alkyl majorly in the AH-Dic complex. Table 4 presented binding energy analysis calculated through MM/GBSA which represent the average total values over 100 ns with the decreasing order as AH-Nbb > AH-Sls > AH-Dic > AH-Sum. The range of the binding energy from the energy decomposition profile for the amino acid residues for all the complexes is between −6.601 and −1.004 kcal/mol (the binding energy cut-off of ≤ −1.0 kcal/mol). Amino acid residues MET 33 (−2.156 kcal/mol), GLU 68 (−6.601 kcal/mol), VAL 121 (−3.153 kcal/mol), and ARG 59 (−2.735 kcal/mol) show the best binding affinity for Dic, Nbb, Sls, and Sum, respectively.

Observing the RSMD result (Figures S6a and S7a), followed the increasing order of fluctuation as AH < AH-Sls < AH-Dic < AH-Sum < AH-Nbb. The overall protein flexibility of AH is altered by the presence of the pollutants as displayed for the RMSF of C alpha atom calculated across all the residues (Figures S6b and S7b). The effect of hydrogen bond interaction is presented in Figures S6c and S7c which shows a higher number for the complexes compared to that of the AH alone. The results for SASA (Figures S6d and S7d) showed the increasing order of AH-Nbb < AH-Sls < AH-Sum < AH-Dic < AH. The structural quality of the complexes derived from the Z score, Errat, and Ramachandran plot is depicted in Table 1. The complexes showed good structural quality compared to the AH alone without the pollutants, which implies the reusability of the protein after coagulation. The decreasing order follows AH-Sls > AH-Sum > AH-Dic > AH-Nbb.

### 4.5. Bertholletia Excelsa

The coagulation studies of *Bertholletia excelsa* (BE) seed proteins have not been presented in the literature, but the information on the use of shells as activated carbon for the purification of water is available [62]. From the present study, the results showed that the coagulation capacity of BE is relatively similar to that of AH earlier reported. The interaction that occurred between AH and the organic molecules led to the formation of BE-Dic, BE-Nbb, BE-Sls, and BE-Sum complexes. As presented in Table 3, the observed predicted percentage removal of the ligands by BE is 90% (9 out of 10) for Dic, 70% (7 out of 10) for Nbb and Sls, and 50% (5 out of 10) for Sum. Energy contributions (Table 4) from polar solvation and solvation free energies might favor the coagulation of Dic by BE than others. The free binding energy analysis (Table 4) calculated through MM/GBSA represents the average total values over 100 ns and the decreasing order follows BE-Dic > BE-Sls > BE-Nbb > BE-Sum. Per residue energy decomposition was analyzed to gain more insight into the role of each participating amino acid residue (Table S4). The energy values with cut-off ≤−1.0 kcal/mol ranged between −4.982 to −1.037 kcal/mol. Amino acid residues that showed the strongest binding affinity are those of MET 88 (−2.499 kcal/mol), GLU 76 (−4.982 kcal/mol), GLU 92 (−4.134 kcal/mol), and ARG 14 (−2.782 kcal/mol) for Dic, Nbb, Sls, and Sum, respectively.

The stability of the complexes according to the RSMD result (Figures S8a and S9a) is expressed as decreasing order of BE > BE-Sum > BE-Nbb > BE-Dic > BE-Sls. The changes of residues with the fluctuations within the complex were observed through RMSF (Figures S8b and S9b) and the maximum displacement observed for all is around 5 Å. The plot of the number of hydrogen bonds (Figures S8c and S9c) showed no appreciable difference for the complexes compared to that of the BE alone. The SASA result (Figures S8d and S9d) shows the increasing order as BE-Nbb ≤ BE-Dic ≤ BE-Sls ≤ BE-Sum < BE. The structural quality determined by the Z score, Errat, and Ramachandran plot is depicted in Table 1. The reusability of the BE protein after coagulation is demonstrated with the complexes showing good structural quality than the protein alone. The decreasing order of the structural quality of the complexes follows BE-Nbb > BE-Sls > BE-Dic > BE-Sum.

### 4.6. Brassica napus

There is literature evidence that *Brassica napus* (BN) straw modified by tartaric acid adsorbed methylene blue [63] but the information on the use of the seed protein for organic removal from the environment is limited. In the present study, coagulation of the organic molecules to BN is relatively higher than the ones observed for the rest of the proteins (Table 3). The complexes formed are BN-Dic, BN-Nbb, BN-Sls, and BN-Sum. According to Table 3, BN coagulated the pollutants as high as 100% (10 out of 10) for Dic and Nbb, 90% (90 out of 10) for Sls, and 80% (8 out of 10) for Sum. Energy contributions (Table 4) from polar solvation and solvation free energies may be the reason for the higher coagulation of Dic by BN. The net binding energy value for the complexes is displayed in Table 4 and the decreasing order follows BN-Sls > BN-Nbb > BN-Dic > BN-Sum. A similar order is also observed for interactions between MO and the pollutants. Per residue amino acid residues profile (Table S5) showed that BN-Sls has the highest number of amino acid residues. The binding energy cut-off for the profile is ≤ −1.0 kcal/mol and the value ranged between −4.948 to −1.006 kcal/mol. The key residues with the best binding energy are ILE 88 (−3.292 kcal/mol), GLU 46 (−4.948 kcal/mol), and TRP 36 (−3.429 and −4.011 kcal/mol) for Dic, Nbb, Sls, and Sum, respectively.

From the RSMD result (Figures S10a and S11a), the maximum displacement observed for all is around 5 Å except for BN-Sls at 6 Å. As observed from the RMSF of the C alpha atom calculated across all the residues, the presence of the pollutants altered the overall protein flexibility of BN (Figures S10b and S11b). The plot of the number of hydrogen bonds (Figures S10c and S11c) showed a higher number for the complexes except for BN-Sum when compared to that of the BN alone. The result of SASA (Figures S10d and S11d) showed the surfaces of the complexes are more reduced than that of the BN alone without the ligands. The overall quality of both the BN protein and the complexes was checked by Z score, Errat, and Ramachandran plot as depicted in Table 1. The good structural quality observed for the complexes compared to the BN alone without the pollutants indicated the reusability of the protein after coagulation. The decreasing order follows BN-Nbb > BN-Sls > BN-Dic > BN-Sum.

### 4.7. Helianthus annuus

The seed shell of *Helianthus annuus* (HA) has been investigated for effective removal of malachite green [64] but limited information is available on its seed proteins in the environmental bioremediation. The present study used a computational approach to elucidate the potentials of HA protein in the removal of organic pollutants. After 100 ns simulation time, the interactions taking place between HA protein and the organic molecules give rise to HA-Dic, HA-Nbb, HA-Sls, and HA-Sum complexes. According to Table 3, the highest coagulation of the ligands by HA is 100% (10 out of 10) for Dic, 90% (9 out of 10) for Nbb and Sls, and 60% (6 out of 10) for Sum. The highest coagulation is observed for Dic than the other three organic molecules which may be due to the stronger polar solvation and solvation free energies observed for HA-Dic than the other three complexes in Table 4. The binding energy (Table 4) showed decreasing order as HA-Dic > HA-Sls > HA-Nbb > HA-Sum. A similar order was observed for BE-pollutants complexes. A very strong affinity was shown for HA-Dic which might be resulted from the percentage of the ligand coagulated to the protein (100%) and strong contributions from polar solvation and solvation free energies than the rest of the complexes (Table 4). The binding energy from the energy decomposition profile (with the binding energy cut-off of ≤ −1.0 kcal/mol.) for the amino acid residues for all the complexes ranged between −5.476 to −1.015 kcal/mol. The best binding affinities are found with amino acid residues MET 86 (−3.034 kcal/mol), GLU 19 (−5.476 kcal/mol), MET 75 (−2.174 kcal/mol), and TYR 2 (−2.098 kcal/mol) for Dic, Nbb, Sls, and Sum, respectively.

The RSMD result (Figures S12a and S13a) showed the increasing order of fluctuation as HA < HA-Sum ≤ HA-Nbb < HA-Sls < HA-Dic. The RMSF (Figures S12b and S13b) of the C alpha atom calculated across all the residues measures the difference in the flexibility of amino acid residues during the simulation. The increasing order of hydrogen bonds follows HA < HA-Dic < HA-Nbb ≤ HA-Sls ≤ HA-Sum. The protein flexibility in terms of SASA (Figures S12d and S13d) showed the increasing order of HA-Nbb < HA-Sum < HA- Sls < HA-Dic ≤ HA. The SASA result is in the agreement with the result of amino acid depicted in Table S6 for HA-Nbb with the highest number of amino acids participating which has caused a reduction in the surface of the complex. The structural quality of both the protein and the complexes was analyzed using the Z score, Errat, and Ramachandran plot (Table 1). The structural quality for the complexes is better than that of HA alone without the pollutants. This is an indication of the reusability of the protein after coagulation. The decreasing order follows HA-Sum > HA-Dic > HA-Nbb > HA-Sls.

### 4.8. The Cross Performance of the Protein Molecules under Study for Dynamic Coagulation of Organic Molecules

#### 4.8.1. Coagulation of Organic Pollutants by the Proteins

**Figure 3:**
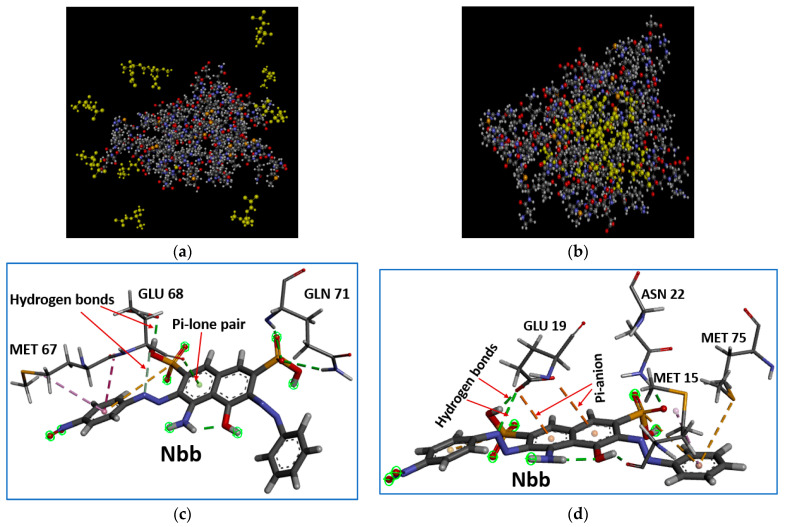
The representative snapshots of the clustering of the Dic pollutant around HA protein (100% coagulation with −124.80 kcal/mol binding energy) (**a**) 0 ns and (**b**) 100 ns. The typical interactions and the bonds between Nbb and the two best binding affinities (**c**) Glu 68 and (**d**) GLU 19.

The coagulation of four organic pollutants by the five proteins was studied computationally and presented in Table 3. Maximum coagulations were predicted at 90% (9 out 10 molecules of Nbb), 90% (9 out 10 molecules of Dic), 100% (10 out 10 molecules of Dic and Nbb), 100% (10 out 10 molecules of Dic), and 100% (10 out 10 molecules of Dic) for AH, BE, BN, and MO, respectively. The average coagulation observed for each of the proteins across the four organic molecules follows the decreasing order of BN > HA > MO > AH = BE. In this case, BN and HA have the potentials for higher coagulation ability than MO. An experimental study reported a similar case in which the seed extracted protein from mustard seed (60% coagulating activity) had higher turbidity removal than that of MO (50% coagulating activity) [65]. In another study, *Panda oleosa* had 92% turbidity removal whereas MO had 89% [66]. These are indications that underexploited plants other than MO can also be utilized as bio-coagulants for water purification. It was also observed that all four organic molecules have their maximum coagulation by BN. This means that BN has a tendency of coagulating organic pollutants when employed in the treatment of contaminated environments than the other proteins under study. This might be associated with the isoelectric point of BN calculated to be 8.71 (Table 2) as it has been reported that the leguminous proteins as natural coagulants serve best around pH of 7.0–8.5 [13]. The result presented in Table 3 showed that there is a high affinity towards Dic by the proteins as four of them have their maximum coagulation with Dic. This may have resulted from solvation effects as the energy contributions (Table 4) from polar solvation and solvation free energy favor the binding affinity of Dic to the proteins. The values of the mentioned solvation energies for the protein-Dic complex were much lower (ranged between 135.21–409.71 kcal/mol for polar solvation and 23.95–43.30 kcal/mol) than other complexes (ranged between 37.42–57.0 kcal/mol for polar solvation and 118.26–386.32 kcal/mol). Though these energies are also low for AH-Dic, Nbb is the most coagulated by AH (Table 3). The reason may also be attributed to the contributions from van der Waals (−154.12 kcal/mol), electrostatic (−349.35 kcal/mol), non-polar solvation (−22.14 kcal/mol), and gas phase energies (−503.47 kcal/mol) which are much higher for AH-Nbb than the other three complexes (ranged between −94.80–−101.87, −110.70–−157.86, −13.47–−17.53, and −105.60–−252.26 kcal/mol for van der Waals, electrostatic, non-polar solvation and gas phase energies respectively). The representative snapshots of the clustering of the Dic pollutant around HA protein (100% coagulation with −124.80 kcal/mol binding energy) are shown in Figure 3a,b. The reusability of these proteins after coagulation was investigated and this was based on the quality of the protein after coagulation as depicted in Table 1. The reusability of MO after coagulation is poor as the qualities of MO-pollutant complexes are below that of the MO alone without the pollutants.

#### 4.8.2. The Binding Affinity of the Proteins with the Organic Molecules

The binding energy recorded for the five proteins with the four organic molecules is generally high (−147.02–−45.68 kcal/mol) as depicted in Table 4. The best binding energy for all the complexes is found with BN-pollutants complexes except for the BE-Dic complex. This result may be associated with the best maximum coagulation of the organic molecules observed for BN. Among all the complexes formed between the proteins and the organic molecules, BN-Sls exhibited the best binding affinity binding energy of −147.02 kcal/mol. Tables S2–S6 depicted amino acid decomposition analysis for the protein-pollutants interactions. A large number of amino acid residue methionine, MET (>50%) was found common with AH-Dic, BE-Dic, and BE-Sls and formed mostly hydrophobic bonds (Table S1) with the three complexes. The amino acid residues with the best binding affinity across the proteins and the organic molecules are found to be GLU 68 (−6.601 kcal/mol), GLU 76 (−4.982 kcal/mol), GLU 46 (−4.948 kcal/mol), GLU 19 (−5.476 kcal/mol) and ARG 83 (−5.160 kcal/mol) with AH-Nbb, BE-Nbb, BN-Nbb, HA-Nbb, and MO-Sum, respectively. It is observed that MO protein contained no glutamic acid which is supported by the number of negatively charged residues (Asp + Glu) recorded to be zero in Table 1. The complex that attracted the larger number of amino acid residues is the one that formed between the proteins and Nbb except for BN-Sls. This might happen as it contained more functional groups in its structure. As it is observed that glutamic acid in the different positions formed the best binding affinity with Nbb. The typical interactions and the bonds between Nbb and Glu 68 and 19 (the two best binding affinities) are represented in Figure 3c,d. It showed that glutamic acid interacts mostly with Nbb through hydrogen bonding and π-lone pair. It is also observed from Table S1 that π-interactions exist majorly between Nbb and the proteins. Some key and common residues were discovered for each of the proteins interacting with the organic molecules and they are:

MO: LUE 4, ARG 24, PRO 39, GLN 40, TYR 46, ARG 47, ARG 51, ILE 55, PHE 65, ARG 79, ARG 83, and TRP 88.

AH: MET 33, ILE 36, MET 37, TYR 46, ARG 59, GLU 68, MET 94, ARG 112, and VAL 121.

BE: ARG 14, MET 23, ARG 26, MET 29, MET 37, GLU 76, MET 77, MET 84, MET 87, MET 88, LEU 90, GLU 92, ILE 111, ALA 112, and PHE 114.

BN: GLN 20, TRP 21, ARG 23, GLN 25, LEU 26, PRO 30, PHE 31, TRP 36, GLU 46, CYS 49, TYR 53, VAL 60, VAL 71, ARG 72, GLN 74, ARG 84, ILE 88, LEU 92, PRO 93, VAL 95, MET 98, and ILE 101.

HA: TYR 2, TYR 12, GLU 19, MET 26, TYR 27, LEU 50, CYS 64, ILE 67, MET 68, PRO 74, MET 75, MET 86, LEU 90, and MET 103.

The strong interaction between organic pollutants and proteins results in floc formation [67,68]. Therefore, a high shear force is required to break down the flocs [69]. Possible methods of separating and recovering the proteins include chromatography and electrophoresis [70,71]. The large-scale implementation of these methods would require further development.

#### 4.8.3. Stability Studies of the Complexes Formed between the Proteins and the Organic Molecules

**Figure 4:**
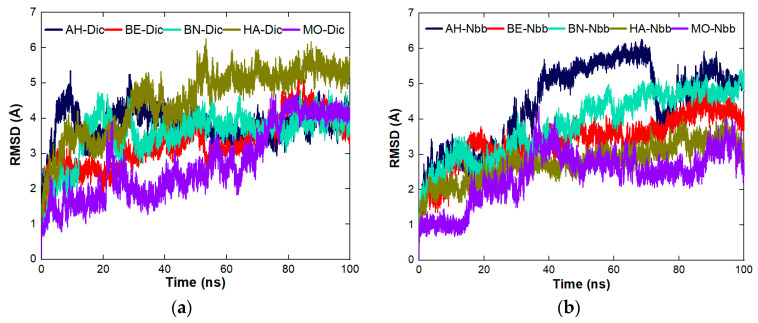

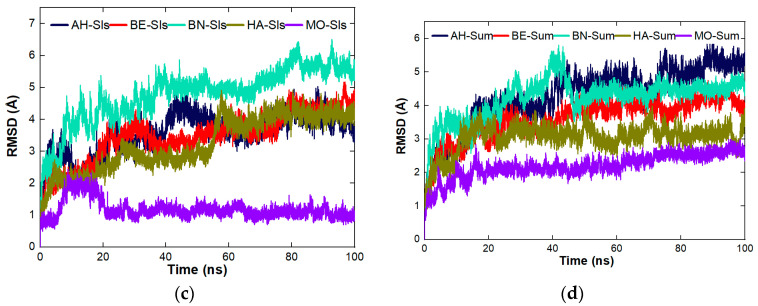
RMSD plots of the complexes across the five proteins (**a**) protein-Dic (**b**) protein-Nbb (**c**) protein-Sls and (**d**) protein-Sum.

**Figure 5:**
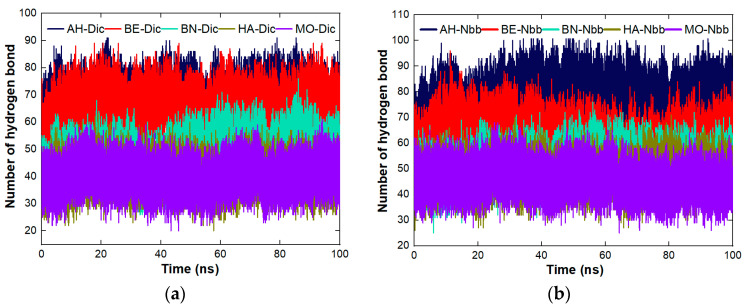

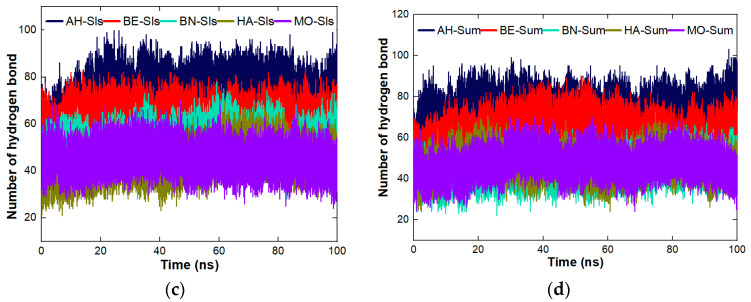
Number of hydrogen bond plots of the complexes across the five proteins (**a**) protein-Dic (**b**) protein-Nbb (**c**) protein-Sls and (**d**) protein-Sum.

**Figure 6:**
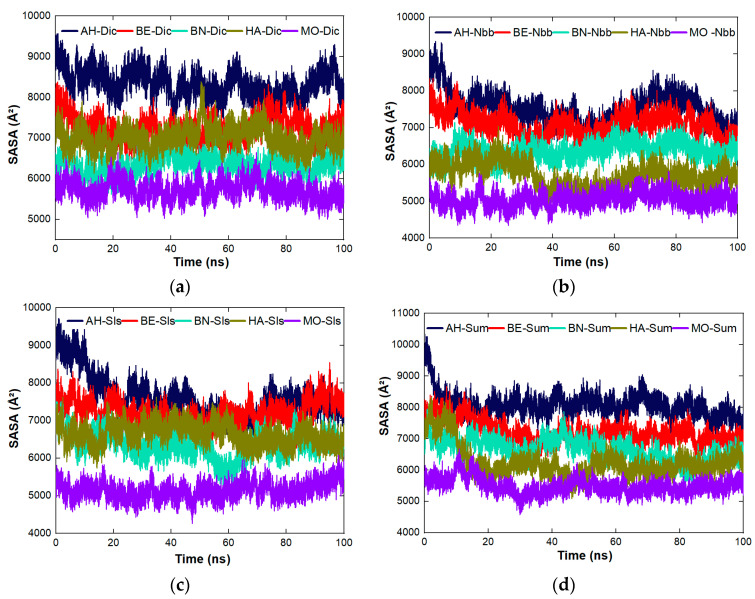
SASA plots of the complexes across the five proteins (**a**) protein-Dic (**b**) protein-Nbb (**c**) protein-Sls, and (**d**) protein-Sum.

The stability of the complexes across the five proteins was studied for RMSD, the number of hydrogen bonds, and SASA. The figures used in this section were pulled from the individual plots presented in the supplementary information. RMSD was used to study the dynamic behavior of the systems of the complexes as represented in Figure 4a–d. The most stable complex between Dic and the proteins (Figure 4a) is MO-Dic and the decreasing order of stability is MO-Dic > BE-Dic > BN-Dic > AH-Dic > HA-Dic. For Nbb and the proteins (Figure 4b), the most stable complex is the one between MO and Nbb, the decreasing order of stability is MO-Nbb > HA-Nbb > BE-Nbb > BN-Nbb > AH-Nbb. The complex MO-Sls is the most stable for protein-Sls complexes (Figure 4c) and the decreasing order follows MO-Sls > HA-Sls > BE-Sls > AH-Sls > BN-Sls. Figure 4d showed that MO-Sum is the most stable complex between the proteins and Sum molecules with the decreasing order of MO-Sum > HA-Sum > BE-Sum > BN-Sum > AH-Sum. In all, the complexes formed between MO protein and the pollutants are the most stable ones and MO-Sls took preeminently. The stability of MO complexes may also be a result of the fact that the MO protein itself is the most stable protein studied among others (Figure 2b). The trend of the stability observed through RMSD does not follow the trend of the instability index listed in Table 1. The trend of the number of hydrogen bonds (Figure 5a–c) is the same for all the five proteins across the four organic molecules. The increasing order of the number of hydrogen bonds follows MO ≤ HA ≤ BN < BE < AH. This implies that all the complexes involving AH protein contain the highest number of hydrogen bonds than the rest complexes. The increasing trend of SASA (Figure 6a–c) for all the proteins interacting with Dic and Sls follows the same order as MO < BN < HA < BE < AH while the increasing trend of SASA for all the proteins interacting with Nbb and Sum follows the same order as MO < HA < BN < BE < AH. MO protein having the least SASA values means that surface of the protein is less available to coagulate more pollutants compare to others. The SASA plots for all the complexes are still much related to the one plotted for the proteins alone without the ligands linking the SASA values to the number of amino acid lengths.

## 5. Conclusions

*Moringa oleifera* (MO) has been extensively studied for its coagulating activities for wastewater treatment whereas there are other edible seed plants that can also serve the same purpose. The present study used a computational approach to investigate the potentials of using underexploited leguminous seed proteins for the coagulation of organic pollutants from the environment. Among such seed proteins are *Arachis hypogaea* (AH), *Bertholletia excelsa* (BE), *Brassica napus* (BN), and *Helianthus annuus* (HA). The percentages of coagulation of the pollutants by the proteins depend on the types of pollutants involved. Considering each of the proteins under study for coagulation of the four organic pollutants, *Arachis hypogaea* is the most suitable for the removal of Nbb but in terms of stability and reusability, it is most suitable for Sls. *Bertholletia excelsa*, *Brassica napus,* and *Moringa oleifera* are relatively good for the coagulation of Nbb while *Helianthus annuus* is recommended for the removal of Dic. The prediction made in the present theoretical study for the coagulation of organic molecules by MO proteins correlates well with the previous experimental reports.

The performance of the proteins in terms of the coagulation of organic molecules and their binding affinities follows the decreasing order BN > HA > MO > AH ≥ BE. The most coagulated pollutant is Dic (polar solvation and solvation free energies favor the coagulation of Dic) and the maximum coagulation of Dic is to HA protein (highest percentage coagulation with relatively higher binding energy). Energy contributions from polar solvation and solvation free energies favor the coagulation of dichlorvos by the proteins than the other three organic molecules while van der Waals, electrostatic, non-polar solvation, and gas phase energies favor the coagulation of naphthol blue black. The coagulation of Sum to the proteins and its binding affinities are generally low compared to the other three organic molecules under investigation. The reusability of the other four proteins after coagulation looks promising than that of MO as most of their complexes have their qualities above the quality of the proteins alone without the pollutants. The coagulation and binding mode are influenced by the types of amino acids participating in the interaction with organic molecules and this is peculiar with each plant protein. Glutamic acid in different forms (GLU 68 and 41 in case of AH, GLU 30, 76, and 82 in case of BE, GLU 33 and 46 in case of BN and GLU 19, 73, and 34 in case of HA) exhibited strong binding affinity in all the complexes involving Nbb as pollutants except for Moringa *oleifera* protein which does not contain glutamic acid. The general types of interaction between the participating amino acid residues and the ligands are halogen bond, hydrogen bond, π-alkyl, π-lone pair, amide-π-stacked, π-π-stacked, van der Waals, π-cation, π-anion, π-sigma, and π-sulfur. The strong hydrophobic interactions formed by all the complexes influenced positively the contributions of van der Waals to the total binding affinity energy observed for all.

The stability in terms of RMSD indicated that complexes formed between MO and the organic molecules are the most stable ones and the MO itself showed more stability than the rest of the proteins. The MO-Sls was also observed to be the most stable complex. The stability of the complexes is strengthened through hydrogen bonding and hydrophobic interactions exhibited by all. The alteration in the RMSF of the C alpha atom observed for all the complexes showed the participation and the interaction of the amino acid residues. The low values of SASA for the complexes when compared to that of the proteins alone without the ligands indicated that the solvent-accessible surfaces of many of the residues of the proteins under study are no longer accessible to solvent because of the coagulated ligands. From the cross-study of the proteins, it can be concluded that the accessibility of the surfaces of AH, BE, BN, and HA proteins are more available to coagulate more pollutants from the environment than MO protein. Moreover, apart from MO protein and its complexes being more stable than the rest of proteins, BN and HA seed proteins have a higher tendency of performing better than MO for coagulation and binding of organic pollutants from the environment including their reusability after coagulation. The present study has demonstrated a computational approach towards the coagulation of organic pollutants by plant proteins which can be utilized in the selection of the most promising ones for environmental bioremediation most especially in wastewater treatment. Further study is recommended for the investigation on the effects of increase or decrease in the number of pollutants used for the simulation as well as the use of the system containing a combination of two proteins for simulation. Experimental investigation cannot be left out in all the predicted and proposed computational studies.

## Figures and Tables

**Table 1:** Physicochemical properties of the proteins that include amino acid length (aa), molecular weight (MW), instability index, aliphatic index, Gravy, and their structural qualities with those of the complexes that include Z score, Errat, and Ramachandran plot.

Plant	Complex Formed	aa	MW (kDa)	Instability Index	Aliphatic Index	GRAVY	Z Score	Errat (%)	Ramachandran (Favoured Region) (%)
*Arachishypogaea* (AH)		127	14.96	68.66	44.49	−1.216	−3.40	88.34	87.0
	AH-Dic						−3.91	86.67	87.0
	AH-Nbb						−3.61	89.69	85.2
	AH-Sls						−4.54	95.23	88.7
	AH-Sum						−3.80	95.24	90.4
*Bertholletia excelsa* (BE)		114	13.63	77.74	27.46	−1.016	−3.7	91.25	88.9
	BE-Dic						−4.33	90.24	90.90
	BE-Nbb						−4.69	92.21	92.9
	BE-Sls						−4.35	100.0	90.9
	BE-Sum						−4.49	87.34	87.9
*Brassica napus* (BN)		109	12.53	59.38	61.74	−0.761	−3.98	93.83	87.1
	BN-Dic						−3.94	96.43	86.0
	BN-Nbb						−5.84	100.0	87.1
	BN-Sls						−4.72	94.87	87.1
	BN-Sum						−4.95	80.7	80.6
*Helianthus annuus* (HA)		103	12.16	67.95	53.98	−0.586	−4.78	92.19	84.6
	HA-Dic						−4.52	100.00	83.5
	HA-Nbb						−5.56	87.10	90.1
	HA-Sls						−4.87	90.00	85.7
	HA-Sum						−5.66	94.74	91.2
*Moringa oleifera* (MO)		90	10.59	81.42	59.56	−0.768	−6.35	100.00	93.4
	MO-Dic						−5.41	100.00	93.4
	MO-Nbb						−5.17	98.21	88.2
	MO-Sls						−5.32	100.00	88.2
	MO-Sum						−4.20	98.33	85.5

**Table 2:** The predominant (glutamic and aspartic acid) and basic side chains (lysine, arginine, and histidine) amino acid present in the seed legumes, theoretical isoelectric point (PI), number of negatively charged residues, and number of positively charged residues.

Plant Proteins	Amino Acid Composition (%)					Isoelectric Point (PI)	Negatively Charged Residues (Asp + Glu)	Positively Charged Residues (Arg + Lys)
	Glutamic Acid (GLU)	Aspartic Acid (ASP)	Lysine (LYS)	Arginine (ARG)	Histidine (HIS)			
AH	8.7	7.9	1.6	12.6	0.8	5.24	21.0	18.0
BE	14.0	0.9	0.9	13.2	1.8	6.20	17.0	16.0
BN	5.5	0.9	4.6	5.5	1.8	8.71	7.0	11.0
HA	9.7	2.9	3.9	6.8	2.9	5.91	13.0	11.0
MO	0.0	0.0	0.0	15.6	2.2	11.61	0.0	14.0

**Table 3:** The percentage of coagulation of organic molecules by the proteins with individual organic molecules together with the types of interactions involved.

Protein	Parameters	Organic Molecules				Average Coagulation (%)
		Dic	Nbb	Sls	Sum	
AH	% of the ligands coagulated	70	90	60	60	70.00
	Types of bonds/interactions	Halogen, hydrogen, alkyl, π-alkyl	Hydrogen, π-alkyl, π-lone pair, Amide-π-stacked, π-π-stacked, van der Waals, π-cation, π-anion	Hydrogen, alkyl	Hydrogen, alkyl, π-alkyl, π-lone pair, Amide-π -stacked, π-anion	
BE	% of the ligands coagulated	90	70	70	50	70.00
	Types of interaction	Hydrogen, alkyl, π-alkyl, π-sigma	Hydrogen, π-alkyl, Amide-π-stacked, π-cation, π-anion, π-sulfur	Hydrogen, alkyl	Hydrogen, alkyl, π-alkyl, π-lone pair	
BN	% of the ligands coagulated	100	100	90	80	92.50
	Types of interaction	Hydrogen, alkyl, π -alkyl	Hydrogen, alkyl, π-alkyl, π-lone pair, Amide- π-stacked, π-π-stacked, π-sulfur, π-anion	Hydrogen, alkyl, π -alkyl	Hydrogen, alkyl, π-alkyl, π-cation, π-sulfur, π-π -stacked	
HA	% of the ligands coagulated	100	90	90	60	85.00
	Types of interaction	Hydrogen, alkyl, π-alkyl	Hydrogen, π-alkyl, π-lone pair, Amide- π-stacked, π-π -stacked, π-cation, π-sulfur, π-anion	Hydrogen, alkyl, π-alkyl	Hydrogen, alkyl, π-alkyl, π-cation, π-sulfur, π-anion	
MO	% of the ligands coagulated	100	90	50	70	77.50
	Types of interaction	Hydrogen, alkyl, π-alkyl	Hydrogen, π-alkyl, π-lone pair, Amide- π-stacked, π-cation, π-anion	Hydrogen, alkyl, π-alkyl, π-sigma	Hydrogen, alkyl, π-alkyl, π-π -stacked, π-cation, π-sulfur	

**Table 4:** Energy composition profile (kcal/mol) based on MM/GBSA for protein–pollutants complexes. ∆E_VDW_ = van der Waals interaction energies, ∆E_ELE_ = electrostatic contribution, ∆E_EGB_ = polar solvation contribution, ∆E_SURF_ = non-polar solvation energy, ∆G_gas_ = gas phase energy, ∆G_solv_ = solvation free energy, and ∆E_bind_ = binding free energy.

Complex	Energy Component (kcal/mol)						
	∆E_VDW_	∆E_ELE_	∆E_EGB_	∆E_SURF_	∆G_gas_	∆G_solv_	∆E_bind_
AH-Dic	−94.90 ± 0.67	−10.70 ± 0.17	37.42 ± 0.25	−13.47 ± 0.09	−105.60 ± 0.75	23.95 ± 0.19	−81.65 ± 0.64
AH-Nbb	−154.12 ± 0.71	−349.35 ± 1.66	401.03 ± 1.77	−22.144 ± 0.09	−503.47 ± 2.30	378.89 ± 1.69	−124.58 ± 0.65
AH-Sls	−101.87 ± 0.52	−140.45 ± 1.05	167.04 ± 1.00	−17.53 ± 0.09	−242.32 ± 1.39	149.50 ± 0.94	−92.81 ± 0.54
AH-Sum	−94.80 ± 0.41	−157.86 ± 1.01	194.04 ± 0.99	−14.27 ± 0.06	−252.66 ± 1.27	179.77 ± 0.94	−72.99 ± 0.39
BE-Dic	−142.58 ± 0.69	−19.39 ± 0.19	54.88 ± 0.24	−20.02 ± 0.09	−161.97 ± 0.75	34.86 ± 0.20	−127.10 ± 0.68
BE-Nbb	−121.69 ± 0.65	−236.03 ± 0.98	284.50 ± 0.09	−17.72 ± 0.08	−357.72 ± 1.44	266.78 ± 0.98	−90.94 ± 0.50
BE-Sls	−102.86 ± 0.60	−117.03 ± 1.27	135.21 ± 1.09	−16.95 ± 0.10	−219.90 ± 1.75	118.26 ± 1.01	−101.63 ± 0.79
BE-Sum	−60.88 ± 0.39	−121.11 ± 1.32	145.87 ± 1.25	−9.56 ± 0.06	−181.99 ± 1.55	136.31 ± 1.20	−45.68 ± 0.39
BN-Dic	−124.29 ± 0.47	−21.99 ± 0.19	53.90 ± 0.22	−17.07 ± 0.06	−146.29 ± 0.56	36.83 ± 0.19	−109.46 ± 0.44
BN-Nbb	−167.18 ± 0.38	−349.56 ± 1.48	409.71 ± 1.31	−23.39 ± 0.05	−516.74 ± 1.74	386.32 ± 1.26	−130.42 ± 0.52
BN-Sls	−170.54 ± 0.86	−95.42 ± 0.87	145.10 ± 0.86	−26.14 ± 0.13	−265.97 ± 1.42	118.95 ± 0.78	−147.02 ± 0.87
BN-Sum	−109.00 ± 0.59	−189.34 ± 1.35	237.59 ± 1.39	−15.77 ± 0.9	−298.34 ± 1.76	221.82 ± 1.32	−76.52 ± 0.53
HA-Dic	−138.76 ± 0.50	−15.30 ± 0.19	48.69 ± 0.24	−19.44 ± 0.07	−154.06 ± 0.59	29.26 ± 0.20	−124.80 ± 0.50
HA-Nbb	−139.63 ± 0.34	−251.35 ± 1.01	316.15 ± 0.99	−20.90 ± 0.04	−390.98 ± 1.17	295.26 ± 0.96	−95.72 ± 0.28
HA-Sls	−114.55 ± 0.57	−155.87 ± 1.64	177.59 ± 1.49	−19.00 ± 0.10	−270.43 ± 2.08	158.59 ± 1.41	−111.84 ± 0.72
HA-Sum	−97.87 ± 0.52	−153.42 ± 0.89	193.44 ± 0.91	−14.40 ± 0.07	−251.29 ± 1.17	179.04 ± 0.87	−72.25 ± 0.41
MO-Dic	−100.04 ± 0.40	−28.87 ± 0.25	57.30 ± 0.29	−13.99 ± 0.06	−128.91 ± 0.55	43.30 ± 0.26	−85.60 ± 0.36
MO-Nbb	−169.10 ± 0.47	−160.33 ± 0.85	248.02 ± 0.92	−23.00 ± 0.06	−329.43 ± 1.11	225.02 ± 0.87	−104.41 ± 0.32
MO-Sls	−123.97 ± 0.47	−135.99 ± 1.29	167.30 ± 1.23	−19.93 ± 0.08	−259.97 ± 1.56	147.37 ± 1.17	−112.60 ± 0.50
MO-Sum	−97.12 ± 0.35	−221.33 ± 1.54	257.84 ± 1.43	−14.66 ± 0.05	−318.44 ± 1.64	243.18 ± 1.41	−75.27 ± 0.37

## Supplementary Materials

The following supporting information can be downloaded online. Table S1: The amino acid residues of the proteins that interacted with the organic molecules and the types of interaction involved, Table S2: Prominent residues for MO-pollutants interactions based on decomposed binding energies (kcal/mol), Table S3: Prominent residues for AH-pollutants interactions based on decomposed binding energies (kcal/mol), Table S4: Prominent residues for BE-pollutants interactions based on decomposed binding energies (kcal/mol), Table S5: Prominent residues for BN-pollutants interactions based on decomposed binding energies (kcal/mol), Table S6: Prominent residues for HA-pollutants interactions based on decomposed binding energies (kcal/mol), Figure S1(a): The stability of the proteins without the ligands at 100 ns simulation time derived from the plot of RMSD. Individual plots are presented, Table S7: Prominent residues for MO-pollutants interactions based on decomposed binding energies (kcal/mol), Figure S1(b): The stability of the proteins without the ligands at 100 ns simulation time derived from the plot of SASA. Individual plots are presented, Figure S1(c): The stability of the proteins without the ligands at 100 ns simulation time derived from the plot of number of hydrogen bond. Individual plots are presented, Figure S2: The stability of the MO protein without the ligands and the various complexes formed at 100 ns simulation time derived from the plot of (a) RMSD (Å), (b) RMSF (Å), (c) Number of hydrogen bond and (d) SASA (Å^2^), Figure S3(a): The stability of the various MO-pollutant complexes at 100 ns simulation time derived from the plot of RMSD, Figure S3(b): The stability of the various MO-pollutant complexes at 100 ns simulation time derived from the plot of RMSF, Figure S3(c): The stability of the various MO-pollutant complexes at 100 ns simulation time derived from the plot of number of hydrogen bond, Figure S3(d): The stability of the various MO-pollutant complexes at 100 ns simulation time derived from the plot of SASA, Figure S4(a): The RMSD of MO-Dic complex run in duplicate. MO-Dic_1 as first run, MO-Dic_2 as second run and mean as the average of the two runs, Figure S4(b): The RMSF of MO-Dic complex run in duplicate. MO-Dic_1 as first run, MO-Dic_2 as second run and mean as the average of the two runs, Figure S4(c): The plots of number of hydrogen bond of MO-Dic complex run in duplicate. MO-Dic_1 as first run, MO-Dic_2 as second run and mean as the average of the two runs, Figure S4(d): The plots of SASA for MO-Dic complex run in duplicate. MO-Dic_1 as first run, MO-Dic_2 as second run and mean as the average of the two runs, Figure S5: The representative snapshots of the clustering of the Dic pollutant around MO protein with 100% coagulation. MO-Dic_1 and MO-Dic_2 represent first and second run respectively, Table S7: Prominent residues for MO-pollutants interactions based on decomposed binding energies (kcal/mol), Figure S6: The stability of the AH protein without the ligands and the various complexes formed at 100 ns simulation time derived from the plot of (a) RMSD (Å), (b) RMSF (Å), (c) Number of hydrogen bond and (d) SASA (Å^2^), Figure S7(a): The stability of the various AH-pollutant complexes at 100 ns simulation time derived from the plot of RMSD, Figure S7(b): The stability of the various AH-pollutant complexes at 100 ns simulation time derived from the plot of RMSF, Figure S7(c): The stability of the various AH-pollutant complexes at 100 ns simulation time derived from the plot of number of hydrogen bond, Figure S7(d): The stability of the various AH-pollutant complexes at 100 ns simulation time derived from the plot of SASA, Figure S8: The stability of the BE protein without the ligands and the various complexes formed at 100 ns simulation time derived from the plot of (a) RMSD (Å), (b) RMSF (Å), (c) Number of hydrogen bond and (d) SASA (Å^2^), Figure S9(a): The stability of the various BE-pollutant complexes at 100 ns simulation time derived from the plot of RMSD, Figure S9(b): The stability of the various BE-pollutant complexes at 100 ns simulation time derived from the plot of RMSF, Figure S9(c): The stability of the various BE-pollutant complexes at 100 ns simulation time derived from the plot of number of hydrogen bond, Figure S9(d): The stability of the various BE-pollutant complexes at 100 ns simulation time derived from the plot of SASA, Figure S10: The stability of the BN protein without the ligands and the various complexes formed at 100 ns simulation time derived from the plot of (a) RMSD (Å), (b) RMSF (Å), (c) Number of hydrogen bond and (d) SASA (Å^2^), Figure S11(a): The stability of the various BN-pollutant complexes at 100 ns simulation time derived from the plot of RMSD, Figure S11(b): The stability of the various BN-pollutant complexes at 100 ns simulation time derived from the plot of RMSF, Figure S11(c): The stability of the various BN-pollutant complexes at 100 ns simulation time derived from the plot of number of hydrogen bond, Figure S11(d): The stability of the various BN-pollutant complexes at 100 ns simulation time derived from the plot of SASA, Figure S12: The stability of the HA protein without the ligands and the various complexes formed at 100 ns simulation time derived from the plot of (a) RMSD (Å), (b) RMSF (Å), (c) Number of hydrogen bond and (d) SASA (Å^2^), Figure S13(a): The stability of the various HA-pollutant complexes at 100 ns simulation time derived from the plot of RMSD, Figure S13(b): The stability of the various HA-pollutant complexes at 100 ns simulation time derived from the plot of RMSF, Figure S13(c): The stability of the various HA-pollutant complexes at 100 ns simulation time derived from the plot of number of hydrogen bond and Figure S13(d): The stability of the various HA-pollutant complexes at 100 ns simulation time derived from the plot of SASA, Supplementary Tables A–T (The number of atoms present in each system).
