# Supplementary material for: Molecular Interactions Associated with Coagulation of Organic Pollutants by 2S Albumin of Plant Proteins: A Computational Approach
Source: Molecules. 2022 Mar 4;27(5):1685. doi: 10.3390/molecules27051685 (PMC8912086; doi:10.3390/molecules27051685)
Supplement: Supplementary file 1 [file molecules-27-01685-s001.zip › molecules-1526729-supplementary.pdf]

Table S1: The amino acid residues of the proteins that interacted with the organic molecules and the types of interaction involved

	Halogen bonds	Hydrogen bonds	Alkyl	π-alkyl	π-lone pair	Amide-π-stacked	van der Waals	π-cation	π-π-stacked	π-sulfur	π-sigma	π-anion
AH-Dic	CYS 75	MET 103, ARG 116, MET 37, MET 33, ARG 88, CYS 28, GLN 57, PRO 27, CYS 109, CYS 61, ARG 52, PRO 114, GLY 72, GLN 71, SER 15, ARG 22, ASN 24, MET 103, LYS 99,	LEU 119, MET 103, MET 37, MET 33, ARG 88, MET 94, LEU 89, ILE 32, LEU 105, LEU 64, PRO 27, PRO 114, ALA 113, MET 103, LYS 99, PRO 106, LUE 78, LEU 119, LEU 10,	TYR 43, TYR 46								

AH-Nbb		ARG 112, ASN 65, GLU 66, ASN 69, ALA 113, GLN 71, CYS 75, ARG 116, CYS 117, GLN 107, ALA 113, GLN 115, GLN 71, GLU 68, GLU 41, ARG 112, ASP 47, ARG 52, ARG 100, GLN 42		ALA 113, MET 67, ILE 36, MET 94,	GLU 68	MET 67, PRO 114, GLN 115, GLN 96	GLN 115, GLN 97	ARG 52, ARG 100	TYR 46			GLU 41, ASP 47, GLU 39, ASP 44

AH-Sls		SER 127, ARG 9, GLY 8, GLU 17, GLU 76, ARG 4, ARG 18, ASP 21, ASP 91, GLN 40, GLN 93, ASP 118	ARG 5, ARG 125, MET 3, ARG 9, MET 94, MET 37, MET 33, VAL 121									
AH-Sum		SER 104, ARG 49,GLY 124, GLY 123, ARG 92, CYS 60, CYS 28, PRO 27, ASN 24, ARG 59, ASP 62, SER 127, ARG 5, ASP 87, GLY 11	VAL 95, ARG 9, ARG 125	MET 67, PRO 27	SER 104	GLY 123						ASP 62, ASP 87

BE-Dic		CYS 60, GLY 64, MET 56, ASP 57, GLU 7, MET 25, CYS 49, CYS 62, ARG 66	ALA 4, MET 68, MET 67, MET 71, MET 107, MET 69, MET 88, MET 84, MET 87, ALA 91, MET 87, ALA 91, MET 42, ARG 97, MET 72, ILE 94, LEU 90, MET 25, MET 12, MET 68, MET 17, MET 56, PRO 95, ILE 94, MET 69, LEU 65, ARG 66, LEU 65, MET 46, MET 69, MET 17, MET 56, LEU 53, MET 68, MET 88, MET 69, MET 72, MET 25, MET 84, ARG 66, LEU 65, PRO 102, PRO 95, LEU 100	PHE 114,							PHE 114	

BE-Nbb		SER 101, LEU 100, GLU 30, ARG 85, GLU 82, GLN 74, ARG 26, GLY 41, ARG 40, PRO 38, ASN 93, ARG 97, SER 19, GLU 76, GLU 30, ARG 26,		ILE 111, ALA 112, LEU 100, ALA 112, MET 23, ARG 26		GLU 92		ARG 85, ARG 97, ARG 22		MET 37, CYS 98		GLU 82, GLU 76, GLU 30
BE-Sls		ARG 70, ARG 66, GLU 92, GLU 92, ARG 85, ARG 39, ARG 40, ARG 22, ARG 26	MET 68, MET 46, MET 42, ILE 94, LEU 90, ARG 89, LEU 90, MET 37, LEU 18, MET 71, MET 25, MET 29, ARG 22, ARG 26									

BE-Sum		LYS 75, MET 103, SER 110, ARG 26, GLN 78, GLN 27	ILE 111, ARG 66, LEU 65, MET 69	LEU 18, PRO 106, PRO 102, ARG 70, ARG 66, MET 25, ARG 22, MET 23	CYS 105, GLN 27									

BN-Dic		THR 103, CYS 61, PRO 105, ALA 89, GLU 12, TRP 21, TRP 36, ASN 91, GLN 81, ARG 72, ILE 85	PRO 93, LEU 92, ILE 101, MET 98, VAL 60, LEU 52, ARG 84, ILE 88, ILE 55, VAL 95, PRO 38, ILE 22, ILE 85, ALA 89, ILE 88, VAL 95, PRO 38, ILE 88, VAL 95, ILE 88, ILE 85, VAL 71, ARG 72, ARG 84	TYR 53, TRP 21, HIS 14, TRP 21, TRP 36, TRP 36								

BN-Nbb		CYS 49, ASN 50, LYS 65, GLY 102, THR 103, ASP 56, CYS 96, GLU 46, HIS 77, GLN 76, GLY 78, SER 82, LYS 65, THR 83, GLU 33, GLN 74, ARG 23, GLN 24, ASN 34, SER 29, GLY 102, GLN 100, GLN 100, GLN 100	LYS 69, PRO 79	ILE 101, ARG 23	ARG 72, VAL 71	GLY 78, SER 82			TYR 53	CYS 49, CYS 49, CYS 96,		ASP 56, GLU 46, GLU 33

BN-Sls		SER 29, GLY 28, GLN 25, ASN 97, ARG 45, ARG 84, LEU 26, GLN 25, ASN 97	ARG 16, ALA 17, LEU 64, LYS 65, VAL 60, ILE 101, LEU 92, VAL 95, ARG 45, VAL 95, MET 98, VAL 60, LEU 52, LEU 64	PHE 31, PHE 106, TRP 21, PHE 31, TRP 36, PHE 31, TRP 21, PHE 31								
BN-Sum		CYS 61, LYS 65, GLN 57, ARG 72, SER 29, ASN 34, ARG 72, GLN 19, ARG 72, GLN 74, GLN 10, GLY 75, HIS 14, HIS 77, ARG 72	LYS 65, PRO 93, MET 98, ARG 23, LEU 26, ARG 23	LYS 65, TYR 86, PRO 30, LEU 26				ARG 72	TRP 36	MET 98, HIS 77		

HA-Dic		CYS 94, LYS 56, HIS 23, HIS 47, LEU 28, PRO 65, PRO 91, GLN 82	LEU 58, LEU 55, MET 63, LEU 90, MET 86, LEU 55, LEU 90, LEU 55, MET 86, LEU 55, MET 20, LYS 48, HIS 47, LEU 28, ILE 65, MET 70, MET 20, LEU 55, LEU 21, ARG 4, LEU 96, PRO 65, LEU 71, ILE 67, ALA 87, MET 68, MET 86, MET 70, VAL 83, LEU 28	TYR 27, TYR 27									

HA-Nbb		ARG 43, ASN 57, GLU 73, CYS 11, GLU 19, ASN 22, MET 15, GLU 34, ARG 78, MET 53, CYS 51, LYS 30, MET 42		PRO 40, ARG 43, MET 20, ALA 18, MET 69, MET 15, MET 53, LEU 50, VAL 48, LYS 30	MET 42	ARG 78		ARG 35	HIS 23, HIS 47	MET 75, MET 79		GLU 73, GLU 19
HA-Sls		ARG 35, GLU 73, TYR 12, GLU 16, GLY 3, GLY 5, ARG 35, TYR 27, ASN 31, HIS 23, GLN 54, GLU 19	MET 29, ARG 78, MET 79, MET 75, MET 97, ALA 87, ILE 67, MET 75, LYS 48, MET 29, MET 26	TYR 12, HIS 23								

HA-Sum		ARG 43, GLN 54, MET 20, GLN 13, ARG 35, GLU 16, MET 103, ARG 4, MET 103, THR 7, LEU 71, ARG 80	LEU 50, LEU 32, MET 29, MET 29, ARG 6	LEU 50, LEU 32, LEU 28, MET 75, MET 26, TRP 76, ARG 6, TYR 2, LEU 71				ARG 80		CYS 51, MET 29, MET 15, MET 103, CYS 64, CYS 11, MET 103		ASP 81, GLU 16
MO-Dic		PRO 64, GLN 75, ARG 77, GLY 43, ARG 51, ILE 55, GLN 5, ARG 47, GLY 43, GLN 40, GLN 31, TYR 46, ALA 54, GLN 25, SER 22, HIS 44, ARG 51,	ARG 24, PRO 31, VAL 14, ARG 47, PRO 39, ARG 51, ALA 54, ARG 47, LEU 4, ARG 4, VAL 42, PRO 39, ALA 54, ILE 55, MET 61, PRO 21, VAL 48,	PHE 65, TYR 46, TRP 88, HIS 44								

MO-Nbb		MET 61, ARG 62, SER 50, GLN 40, ARG 79, ASN 57, ARG 51, ASN 57, CYS 8, GLN 40, ARG 47, GLN 41, SER 89, GLN 90, ARG 12		ARG 47, ILE 55, ALA 54, PRO 16	ILE 55	TYR 46, PRO 39		ARG 79, ARG 62, ARG 47				GLN 90
MO-Sls		GLN 90, GLN 5, GLN 40, PHE 65	ARG 87, ARG 83, LEU 4, ILE 55, ILE 55, LEU 4, PRO 39	TRP 88, TRP 88, TRP 88, TYR 46, PHE 65							TRP 88	
MO-Sum		ASN 57, ARG 47, ARG 87, ARG 83, GLN 33, GLN 90, ARG 77, ARG 24	ALA 54, ARG 83, PRO 64	ARG 47, TRP 88, PRO 1, VAL 84, PRO 64				ARG 79, ARG 83, ARG 87, ARG 77	TYR 46	CYS 8		

Table S2: Prominent residues for MO-pollutants interactions based on decomposed binding energies (kcal/mol)

MO-Dic	MO-Nbb	MO-Sls	MO-Sum
TRP 88 (-2.884 ± 1.331)	PHE 65 (-5.072 ± 2.508)	TRP 88 (-3.605 ± 1.666)	ARG 83 (-5.160 ± 1.928)
ILE 55 (-2.031 ± 1.173)	ARG 62 (-3.571 ± 1.776)	ILE 55 (-2.933 ± 1.275)	ARG 24 (-3.375 ± 2.553)
PRO 21 (-1.981 ± 1.341)	TYR 46 (-3.137 ± 0.865)	TYR 46 (-2.713 ± 0.860)	TYR 46 (-2.056 ± 1.576)
LEU 4 (-1.727 ± 1.286)	ARG 47 (-2.892 ± 1.553)	ARG 47 (-2.567 ± 1.718)	TRP 88 (-1.993 ± 1.948)
ARG 51 (-1.586 ± 0.987)	ARG 12(-2.787 ± 2.256)	LEU 4 (-2.298 ± 1.323)	ARG 51 (-1.825 ± 2.770)
VAL 48 (-1.414 ± 0.689)	GLN 59 (-2.628 ± 1.392)	VAL 84(-1.846 ± 0.906)	GLN 33 (-1.352 ± 0.872)
GLN 25 (-1.329 ± 1.013)	ASN 13 (-2.522 ± 1.462)	PHE 65 (-1.791 ± 1.446)	ARG 79 (-1.211 ± 0.762)
PHE 17 (-1.214 ± 1.203)	THR 74 (-2.357 ± 1.865)	PRO 39 (-1.726 ± 1.295)	ILE 55 (-1.039 ± 1.202)
PRO 64 (-1.138 ± 1.412)	PRO 39 (-2.337 ± 0.938)	ARG 87 (-1.592 ± 1.485)	
ARG 24 (-1.094 ± 1.022)	GLN 73 (-2.019 ± 1.892)	ARG 51 (-1.532 ± 1.778)	
	PRO 16 (-1.966 ± 0.602)	GLY 43 (-1.428 ± 0.656)	
	MET 61(-1.897 ± 0.849)	VAL 42 (-1.391 ± 0.705)	
	ARG 69 (-1.868 ± 1.949)	ARG 79 (-1.291 ± 2.183)	
	GLN 75 (-1.825 ± 1.360)	GLN 40 (-1.280 ± 1.305)	
	ARG 51 (-1.555 ± 0.966)	ARG 83 (-1.219 ± 1.608)	
	ALA 54 (-1.507 ± 0.564)		
	ILE 55 (-1.468 ± 1.142)		
	PRO 60 (-1.281 ± 0.623)		
	GLN 40 (-1.226 ± 1.385)		
	GLN 76 (-1.089 ± 1.027)		

Table S3: Prominent residues for AH-pollutants interactions based on decomposed binding energies (kcal/mol)

AH-Dic	AH-Nbb	AH-Sls	AH-Sum
MET 33 (-2.156 ± 1.078)	GLU 68 (-6.601 ± 4.192)	VAL 121 (-3.153 ± 1.068)	ARG 59 (-2.735 ± 1.871)
ILE 36 (-1.475 ± 0.749)	ASP 47 (-5.626 ± 2.262)	ARG 125 (-2.815 ± 2.507)	PHE 111 (-2.444 ± 1.630)
MET 37 (-1.327 ± 0.850)	GLN 71 (-5.208 ± 2.106)	ASP 21 (-2.811 ± 2.829)	ASP 62 (-2.008 ± 1.502)
ARG 88 (-1.247 ± 0.787)	ASN 69 (-3.957 ± 1.860)	PRO 2 (-1.633 ± 1.880)	LYS 99 (-1.646 ± 1.419)
MET 103 (-1.162 ± 1.092)	GLU 41 (-3.822 ± 1.702)	MET 3 (-1.431 ± 1.505)	ASN 110 (-1.628 ± 1.335)
LEU 119 (-1.121 ± 1.197)	TYR 46 (-3.159 ± 1.777)	MET 33 (-1.347 ± 1.050)	ARG 92 (-1.496 ± 2.121)
LEU 102 (-1.060 ± 1.040)	ARG 112 (-3.159 ± 1.222)	MET 94 (-1.247 ± 0.977)	SER 127 (-1.284 ± 1.771)
MET 94 (-1.004 ± 0.503)	PRO 114 (-2.955 ± 0.620)	MET 37 (-1.209 ± 0.746)	GLY 123 (-1.196 ± 0.635)
	ARG 52 (-2.942 ± 1.872)	ASP 118 (-1.194 ± 1.672)	GLN 96 (-1.146 ± 1.188)
	ARG 116 (-2.562 ± 1.707)	ARG 4 (-1.186 ± 1.622)	TYR 46 (-1.090 ± 1.733)
	ILE 48 (-2.257 ± 1.311)	ILE 36 (-1.178 ± 0.782)	ARG 112 (-1.037 ± 0.970)
	TYR 43 (-1.940 ± 1.170)	TYR 46 (-1.076 ± 1.528)	
	MET 67 (-1.908 ± 1.363)		
	ARG 49 (-1.876 ± 1.680)		
	MET 37 (-1.582 ± 0.493)		
	MET 94 (-1.341 ± 0.613)		
	THR 70 (-1.259 ± 1.585)		
	SER 53 (-1.236 ± 1.224)		

Table S4: Prominent residues for BE-pollutants interactions based on decomposed binding energies (kcal/mol)

BE-Dic	BE-Nbb	BE-Sls	BE-Sum
MET 88 (-2.499 ± 1.115)	GLU 76 (-4.982 ± 4.353	GLU 92 (-4.134 ± 4.377)	ARG 14 (-2.782 ± 3.154)
MET 69 (-2.266 ± 1.627)	GLU 30 (-4.755 ± 1.638)	LEU 90 (-2.339 ± 1.135)	ARG 26 (-2.491 ± 2.301)
MET 84 (-1.914 ± 0.819)	ARG 26 (-4.128 ± 1.662)	ARG 89 (-2.033 ± 1.672)	GLN 27 (-1.370 ± 1.343)
ILE 94 (-1.589 ± 1.420)	GLU 82 (-2.027 ± 3.580)	ILE 111 (-2.017 ± 2.472)	MET 23 (-1.171 ± 0.984)
LEU 90 (-1.516 ± 0.751)	GLN 74 (-1.885 ± 2.302)	PHE 114 (-1.664 ± 1.850)	
GLN 73 (-1.400 ± 0.889)	MET 29 (-1.586 ± 0.496)	MET 84 (-1.363 ± 1.133)	
MET 42 (-1.268 ± 1.035)	GLN 78 (-1.483 ± 1.396)	PRO 79 (-1.238 ± 1.562)	
MET 72 (-1.256 ± 0.725)	LEU 90 (-1.469 ± 0.654)	MET 87 (-1.189 ± 0.805)	
ALA 91 (-1.209 ± 0.904)	ALA 112 (-1.372 ± 0.966)	MET 37 (-1.175 ± 0.879)	
LEU 65 (-1.176 ± 1.324)	PHE 114 (-1.309 ± 1.782)	ALA 112 (-1.167 ± 1.489)	
MET 77 (-1.167 ± 0.990)	ILE 111 (-1.262 ± 0.768)	MET 29 (-1.090 ± 0.727)	
MET 87 (-1.091 ± 0.690)	ARG 40 (-1.223 ± 1.541)	MET 25 (-1.084 ± 0.564)	
MET 23 (-1.058 ± 0.926)	MET 77 (-1.216 ± 0.709)		
MET 46 (-1.037 ± 0.772)	ARG 80 (-1.215 ± 1.584)		
	MET 23 (-1.123 ± 0.803)		
	MET 37 (-1.080 ± 0.709)		

Table S5: Prominent residues for BN-pollutants interactions based on decomposed binding energies (kcal/mol)

BN-Dic	BN-Nbb	BN-Sls	BN-Sum
ILE 88 (-3.292 ± 2.045)	GLU 46 (-4.948 ± 1.384)	TRP 36 (-3.429 ± 1.811)	TRP 36 (-4.011 ± 1.479)
LEU 26 (-2.260 ± 1.275)	GLU 33 (-4.607 ± 3.142)	ILE 101 (-2.613 ± 1.576)	ARG 72 (-2.748 ± 3.495)
VAL 95 (-2.172 ± 1.210)	ASP 56 (-3.904 ± 1.215)	PHE 106 (-2.606 ± 1.549)	HIS 14 (-2.307 ± 0.945)
ILE 85 (-1.952 ± 0.983)	TYR 53 (-3.900 ± 0.846)	PHE 31 (-2.345 ± 1.921)	PHE 31 (-2.026 ± 1.483)
TRP 36 (-1.689 ± 1.422)	ILE 101 (-3.857 ± 1.315)	ILE 88 (-2.141 ± 0.838)	GLN 74 (-1.898 ± 1.534)
ARG 72 (-1.532 ± 0.963)	ARG 23 (-3.504 ± 1.338)	MET 98 (-1.919 ± 1.278)	GLN 10 (-1.644 ± 1.672)
TRP 21 (-1.486 ± 0.821)	ILE 109 (-3.178 ± 3.597)	VAL 95 (-1.909 ± 1.643)	PRO 30 (-1.458 ± 1.045)
ILE 22 (-1.455 ± 1.052)	GLY 102 (-2.940 ± 1.091)	ARG 84 (-1.892 ± 1.586)	ARG 23 (-1.351 ± 2.116)
LEU 92 (-1.439 ± 1.077)	GLN 100 (-2.747 ± 2.631)	GLN 25 (-1.730 ± 1.819)	LEU 26 (-1.265 ± 1.128)
GLN 25 (-1.396 ± 1.149)	THR 103 (-2.263 ± 1.587)	VAL 58 (-1.677 ± 1.059)	GLY 75 (-1.245 ± 1.203)
LEU 52 (-1.316 ± 0.450)	VAL 73 (-1.959 ± 1.182)	VAL 60 (-1.640 ± 1.056)	ARG 16 (-1.093 ± 1.855)
MET 98 (-1.298 ± 0.454)	PRO 79 (-1.757 ± 1.223)	CYS 61 (-1.477 ± 0.934)	LYS 65 (-1.073 ± 1.240)
ARG 84 (-1.202 ± 1.078)	VAL 71 (-1.738 ± 1.411)	ARG 45 (-1.349 ± 1.652)	GLN 3 (-1.027 ± 1.435)
VAL 71 (-1.095 ± 0.690)	GLN 1 (1.598 ± 1.603)	GLN 87 (-1.340 ± 0.921)	
PRO 93 (-1.059 ± 0.589)	CYS 49 (-1.349 ± 0.568)	TYR 53 (-1.307 ± 1.405)	
GLN 20 (-1.021 ± 0.895)	ARG 72 (-1.308 ± 1.142)	PRO 93 (-1.280 ± 0.792)	
VAL 60 (-1.018 ± 0.363)	GLN 74 (-1.292 ± 1.079)	LEU 92 (-1.230 ± 0.972)	
GLN 57 (-1.006 ± 0.528)	LEU 26 (-1.234 ± 1.093)	ASN 91 (-1.116 ± 0.825)	
	PRO 62 (-1.229 ± 1.006)	LEU 26 (-1.099 ± 0.821)	
	PRO 42 (-1.154 ± 0.609)	GLN 20 (-1.096 ± 0.845)	
	LYS 99 (-1.118 ± 1.016)	CYS 104 (-1.094 ± 0.824)	
	PRO 30 (-1.061 ± 0.611)	TRP 21 (-1.087 ± 0.584)	
		LEU 64 (-1.046 ± 0.623)	
		CYS 49 (-1.028 ± 0.932)	

Table S6: Prominent residues for HA-pollutants interactions based on decomposed binding energies (kcal/mol)

HA-Dic	HA-Nbb	HA-Sls	HA-Sum
MET 86 (-3.034 ± 1.226)	GLU 19 (-5.476 ± 2.033)	MET 75 (-2.174 ± 1.455)	TYR 2 (-2.098 ± 1.277)
TYR 27 (-2.288 ± 1.084)	GLU 73 (-4.671 ± 1.184)	ARG 35 (-1.960 ± 2.360)	TYR 12 (-1.929 ± 1.809)
LEU 28 (-2.262 ± 1.419)	ASN 31 (-2.244 ± 1.839)	GLU 73 (-1.660 ± 2.664)	ASN 72 (-1.826 ± 1.510)
LEU 90 (-2.169 ± 0.969)	ARG 4 (-2.038 ± 1.643)	TYR 12 (-1.614 ± 1.665)	LEU 71 (-1.399 ± 0.971)
LEU 55 (-2.093 ± 1.243)	LEU 32 (-2.009 ± 0.723)	MET 86 (-1.481 ± 0.428)	LYS 61 (-1.387 ± 1.033)
CYS 24 (-1.951 ± 1.007)	ARG 43 (-1.881 ± 1.765)	MET 103 (-1.463 ± 1.555)	MET 75 (-1.370 ± 1.430)
CYS 51 (-1.767 ± 0.981)	MET 15 (-1.729 ± 0.690)	GLU 16 (-1.377 ± 1.955)	GLN 102 (-1.317 ± 1.445)
MET 68 (-1.731 ± 0.778)	LEU 50 (-1.670 ± 0.999)	MET 26 (-1.280 ± 1.067)	ARG 80 (-1.097 ± 1.983)
LYS 48 (-1.631 ± 1.011)	GLN 54 (-1.608 ± 1.234)	CYS 64 (-1.262 ± 0.593)	MET 103 (-1.015 ± 0.975)
CYS 52 (-1.623 ± 0.812	ARG 78 (-1.607 ± 1.654)	PRO 74 (-1.213 ± 1.030)	
CYS 64 (-1.537 ± 0.690)	MET 69 (-1.572 ± 0.554)	MET 68 (-1.163 ± 0.615)	
ILE 67 (-1.521 ± 0.969)	TYR 12 (-1.534 ± 0.662)	LEU 90 (-1.158 ± 0.565)	
HIS 47 (-1.474 ± 1.101)	MET 53 (-1.435 ± 0.841)	TYR 27 (-1.104 ± 0.845)	
HIS 23 (-1.260 ± 0.837)	MET 75 (-1.298 ± 0.878)	ILE 67 (-1.078 ± 0.607)	
LEU 50 (-1.236 ± 0.864)	ASN 57 (-1.273 ± 1.305)		
MET 26 (-1.091 ± 0.964)	GLN 82 (-1.176 ± 1.132)		
VAL 83 (-1.068 ± 0.760)	GLU 34 (-1.118 ± 1.968)		
	MET 42 (-1.083 ± 1.411)		
	MET 20 (-1.033 ± 0.606)		
	PRO 74 (-1.025 ± 0.592)		

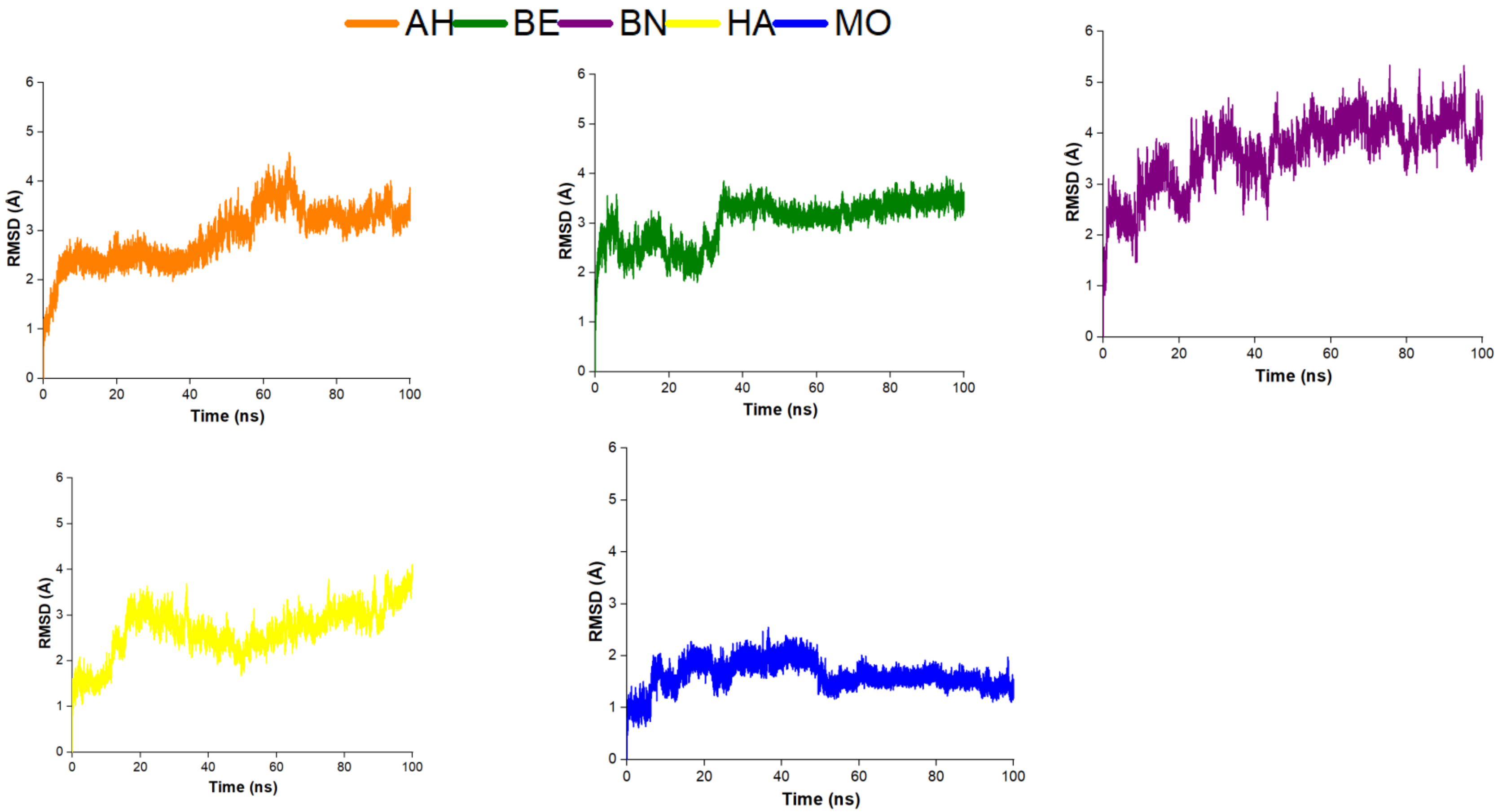

Figure S1(a): The stability of the proteins without the ligands at 100 ns simulation time derived from the plot of RMSD. Individual plots are presented.

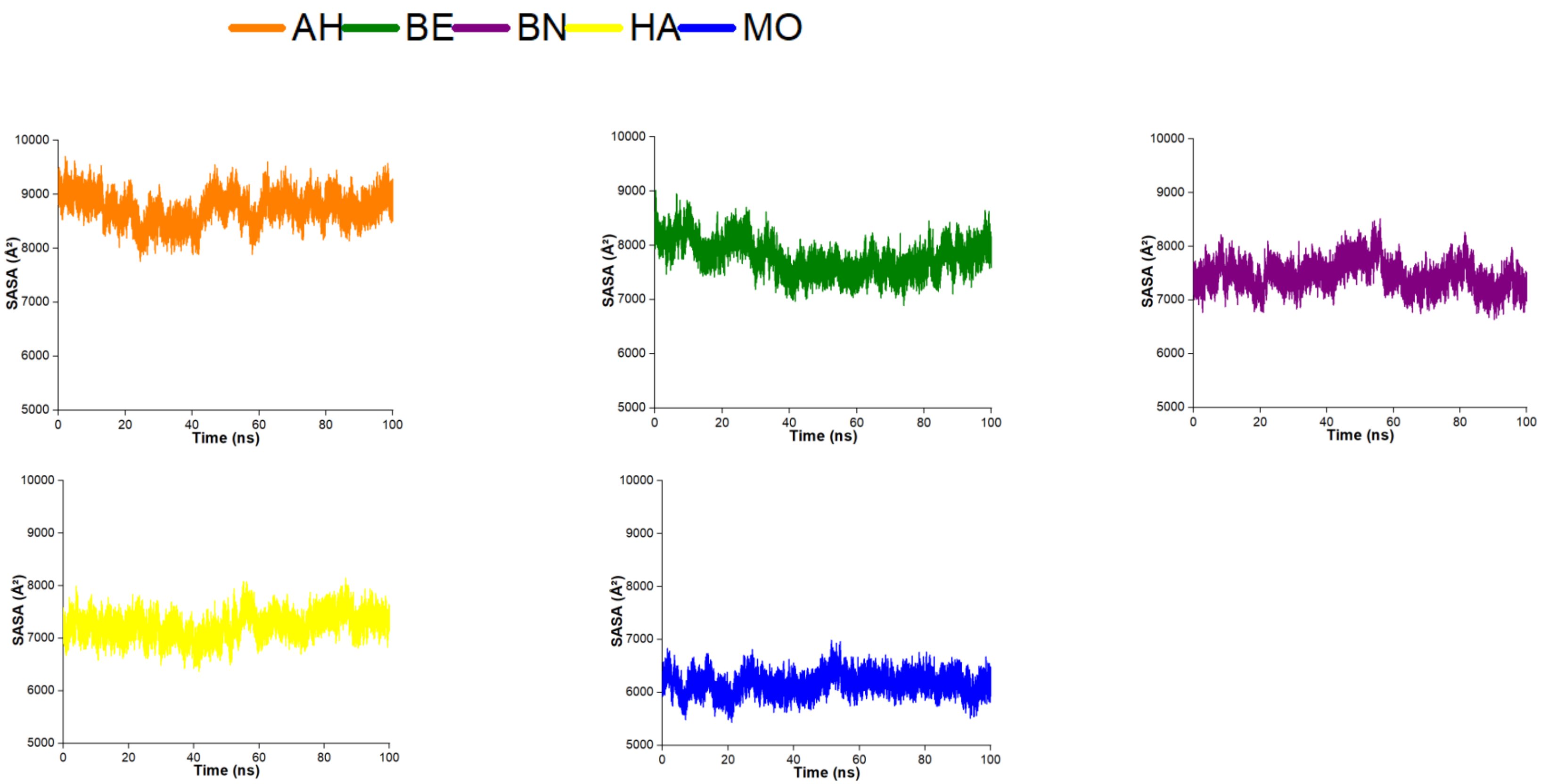

Figure S1(b): The stability of the proteins without the ligands at 100 ns simulation time derived from the plot of SASA. Individual plots are presented.

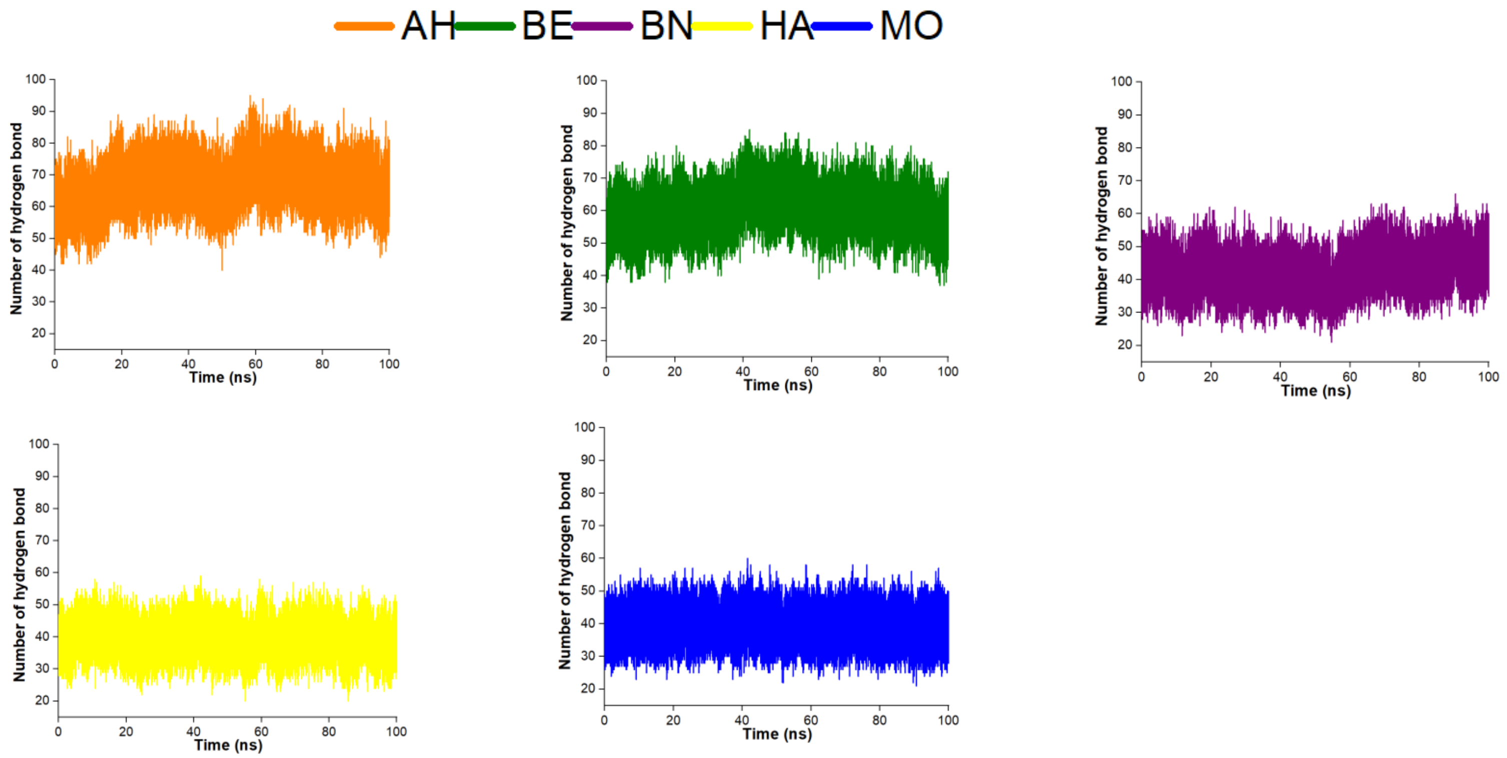

Figure S1(c): The stability of the proteins without the ligands at 100 ns simulation time derived from the plot of number of hydrogen bond. Individual plots are presented.

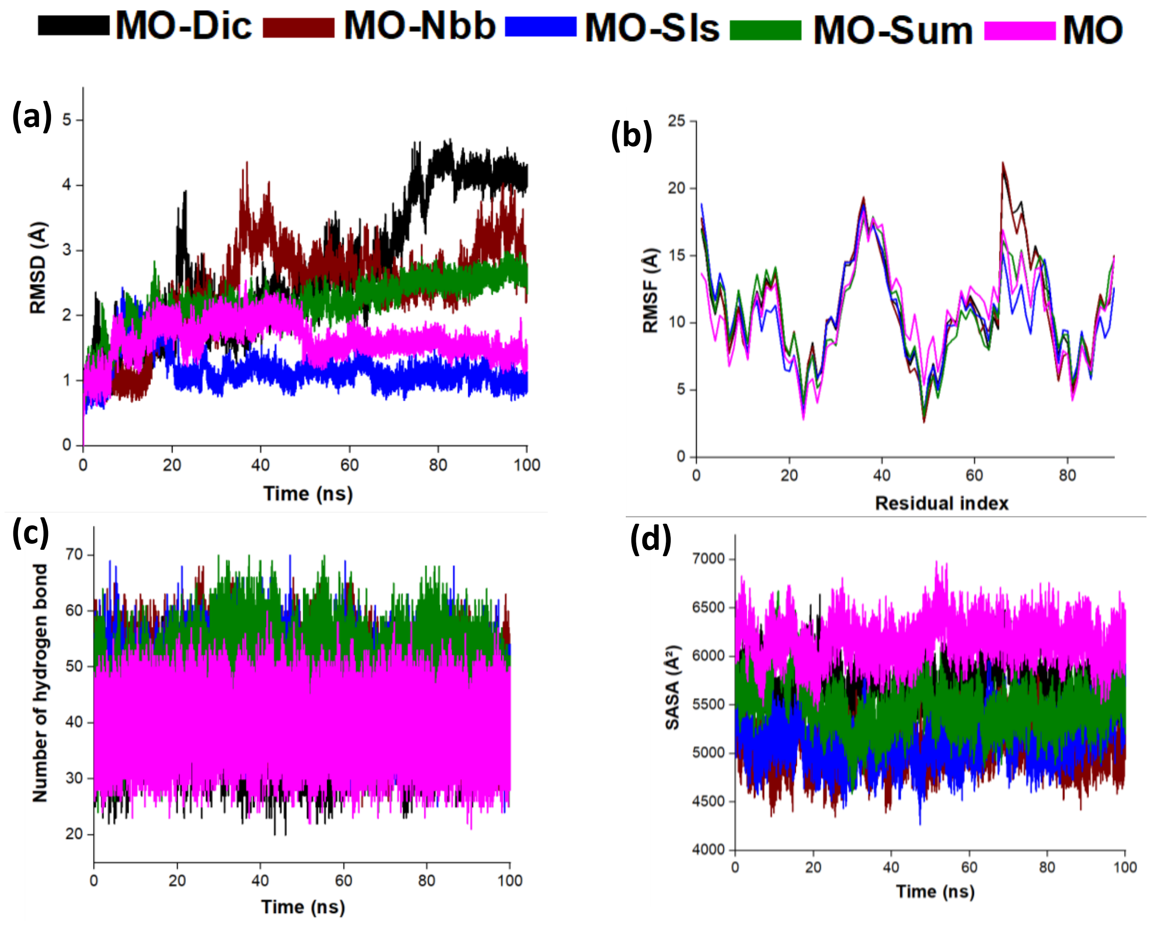

Figure S2: The stability of the MO protein without the ligands and the various complexes formed at 100 ns simulation time derived from the plot of (a) RMSD (Å), (b) RMSF (Å), (c) Number of hydrogen bond and (d) SASA (Å^2)

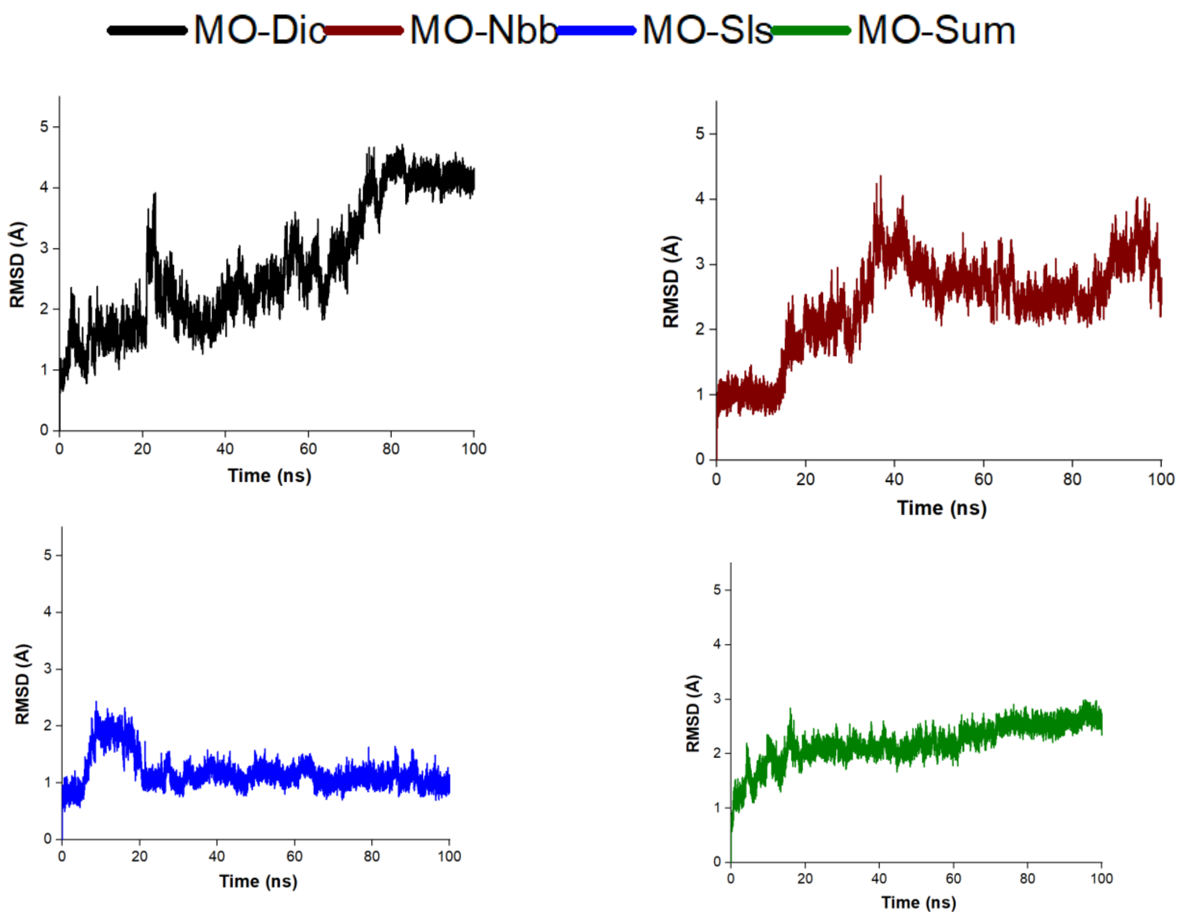

Figure S3(a): The stability of the various MO-pollutant complexes at 100 ns simulation time derived from the plot of RMSD

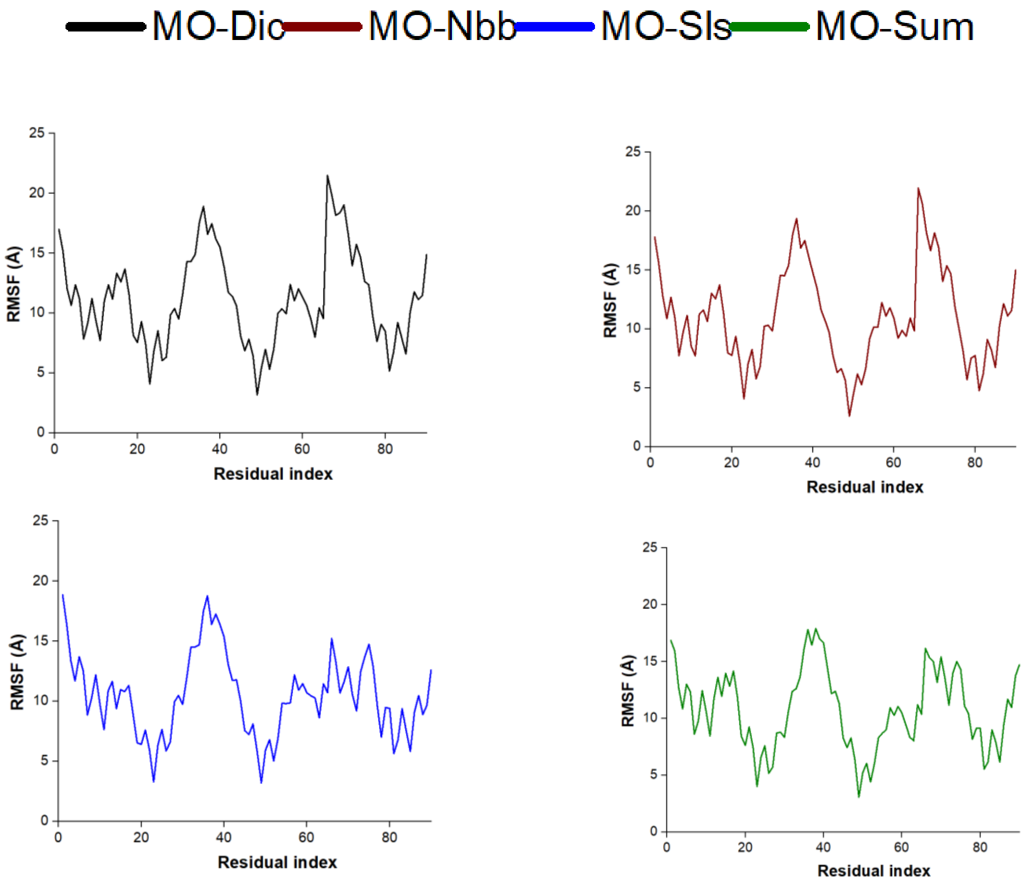

Figure S3(b): The stability of the various MO-pollutant complexes at 100 ns simulation time derived from the plot of RMSF

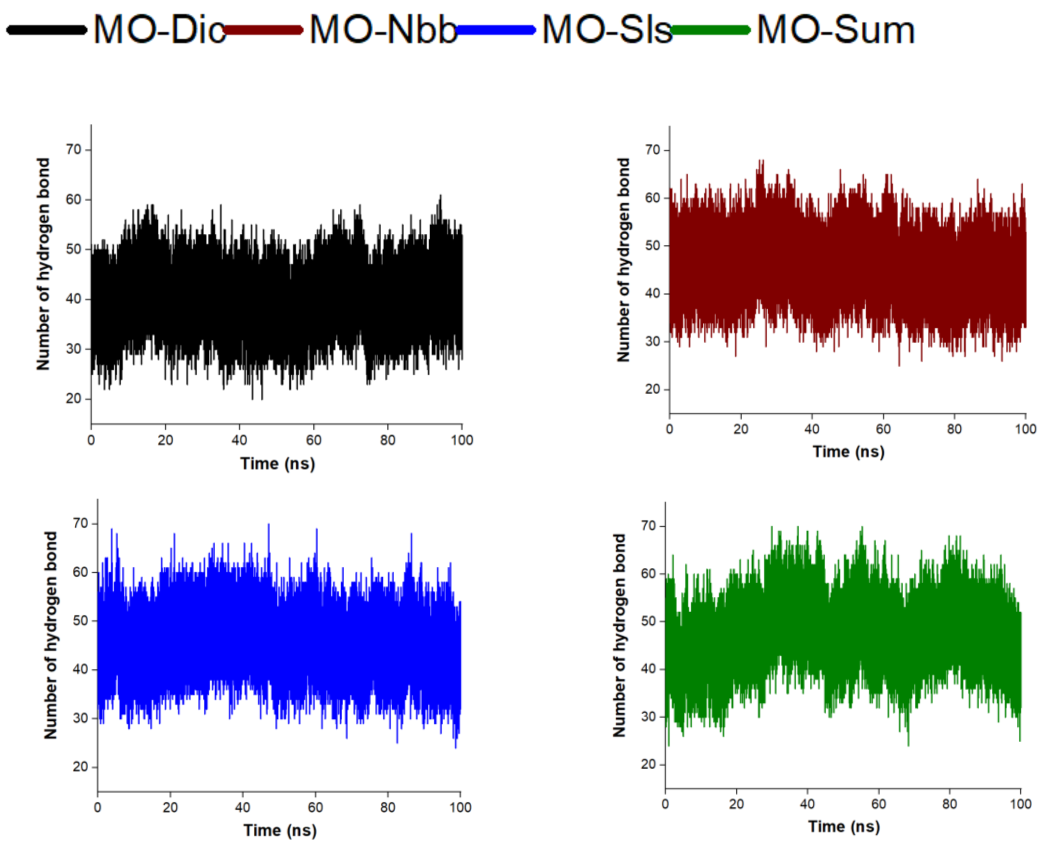

Figure S3(c): The stability of the various MO-pollutant complexes at 100 ns simulation time derived from the plot of number of hydrogen bond

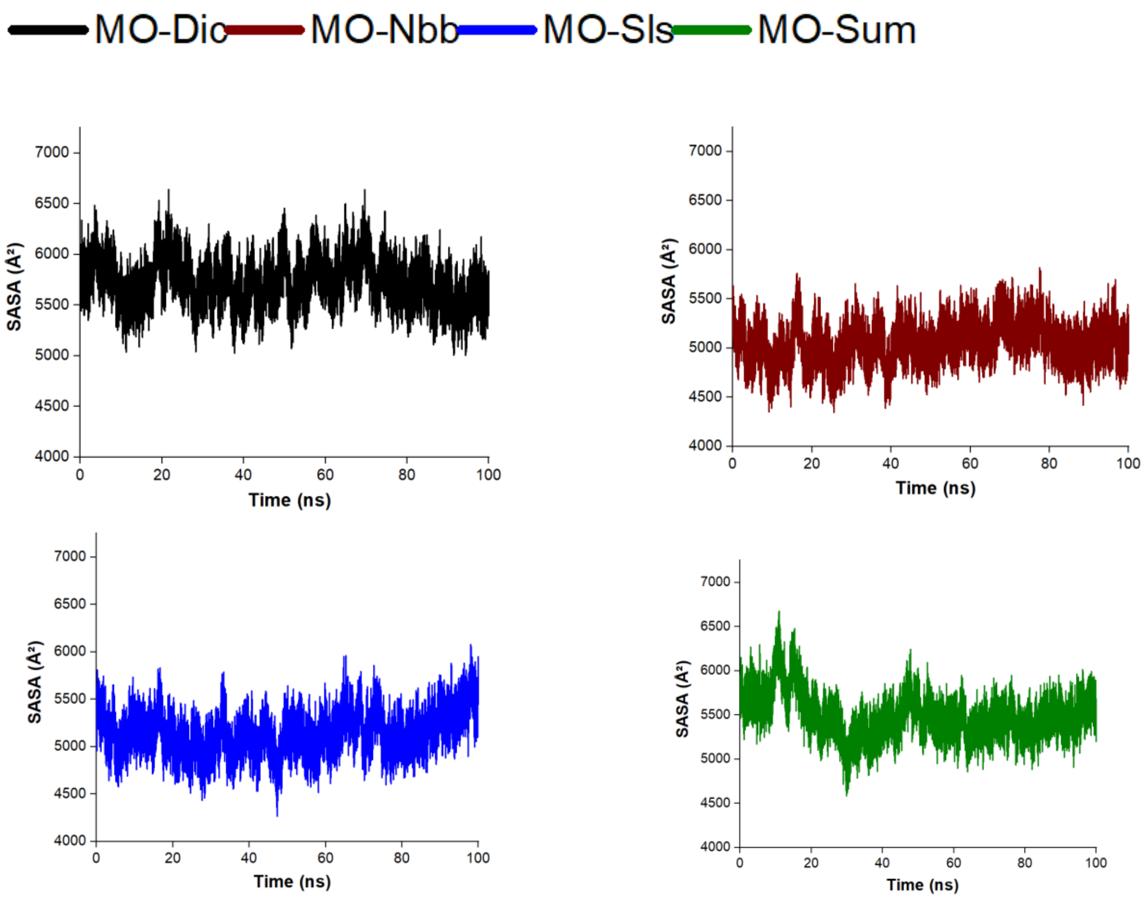

Figure S3(d): The stability of the various MO-pollutant complexes at 100 ns simulation time derived from the plot of SASA

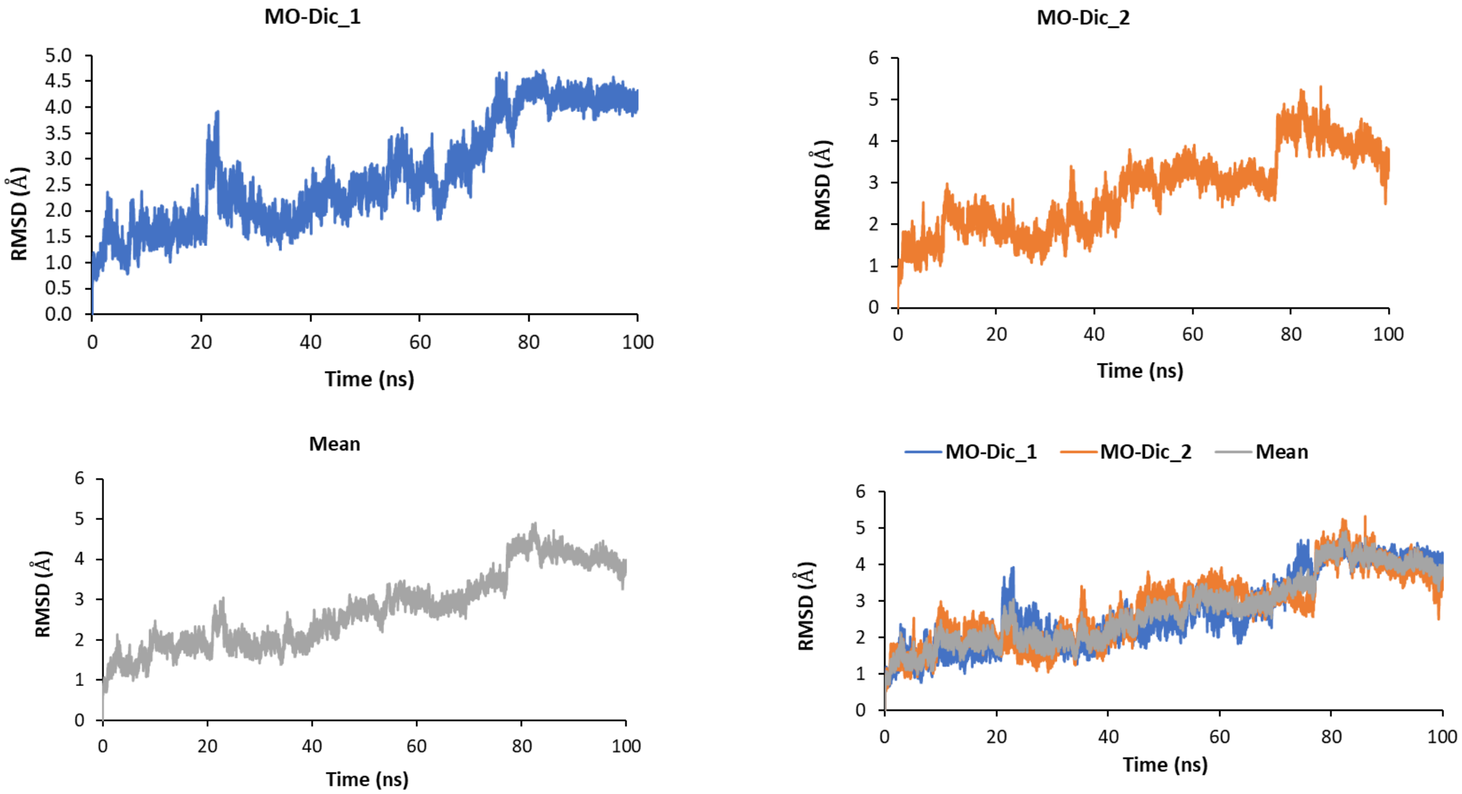

Figure S4(a): The RMSD of MO-Dic complex run in duplicate. MO-Dic_1 as first run, MO-Dic_2 as second run and mean as the average of the two runs.

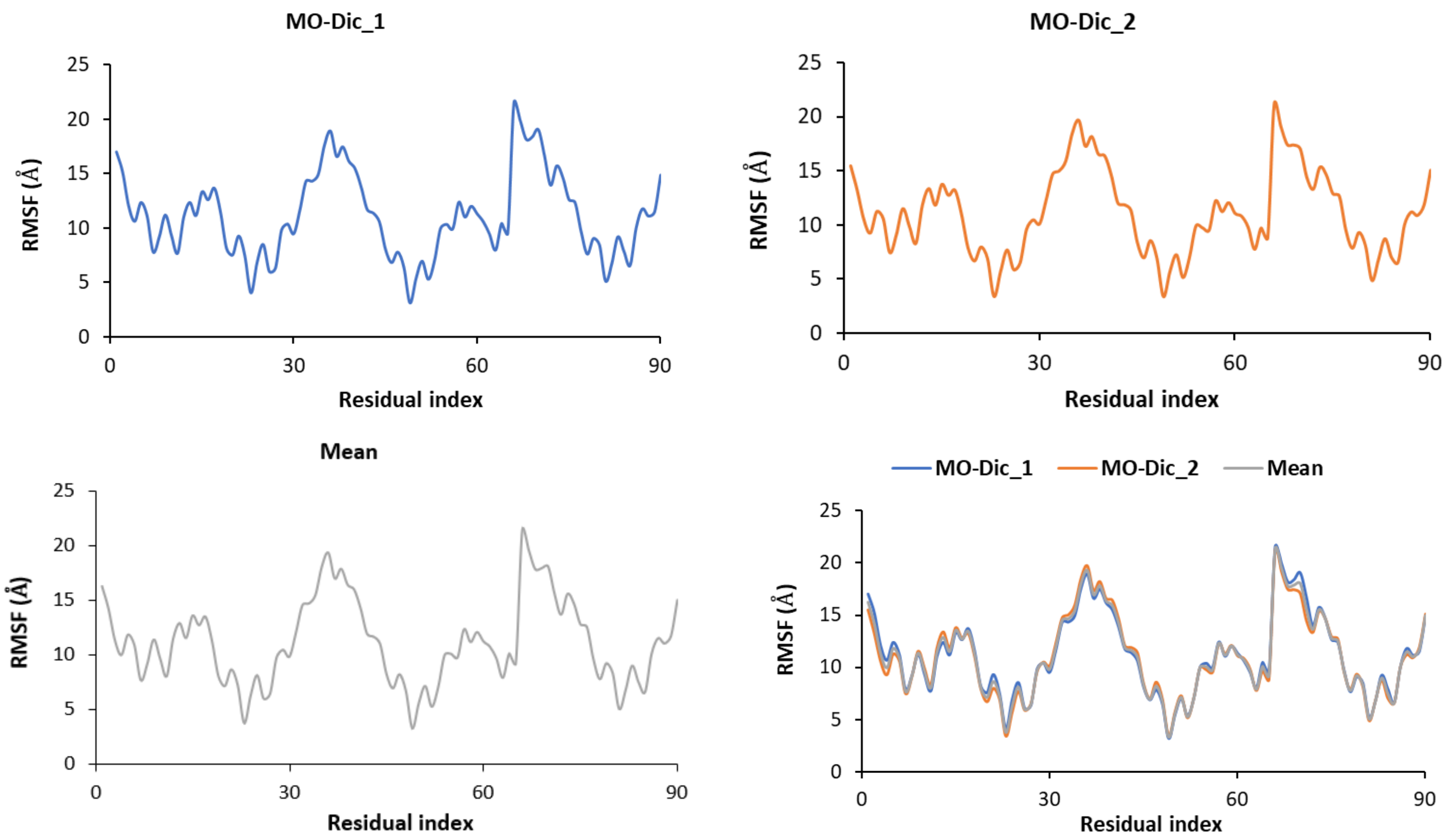

Figure S4(b): The RMSF of MO-Dic complex run in duplicate. MO-Dic_1 as first run, MO-Dic_2 as second run and mean as the average of the two runs.

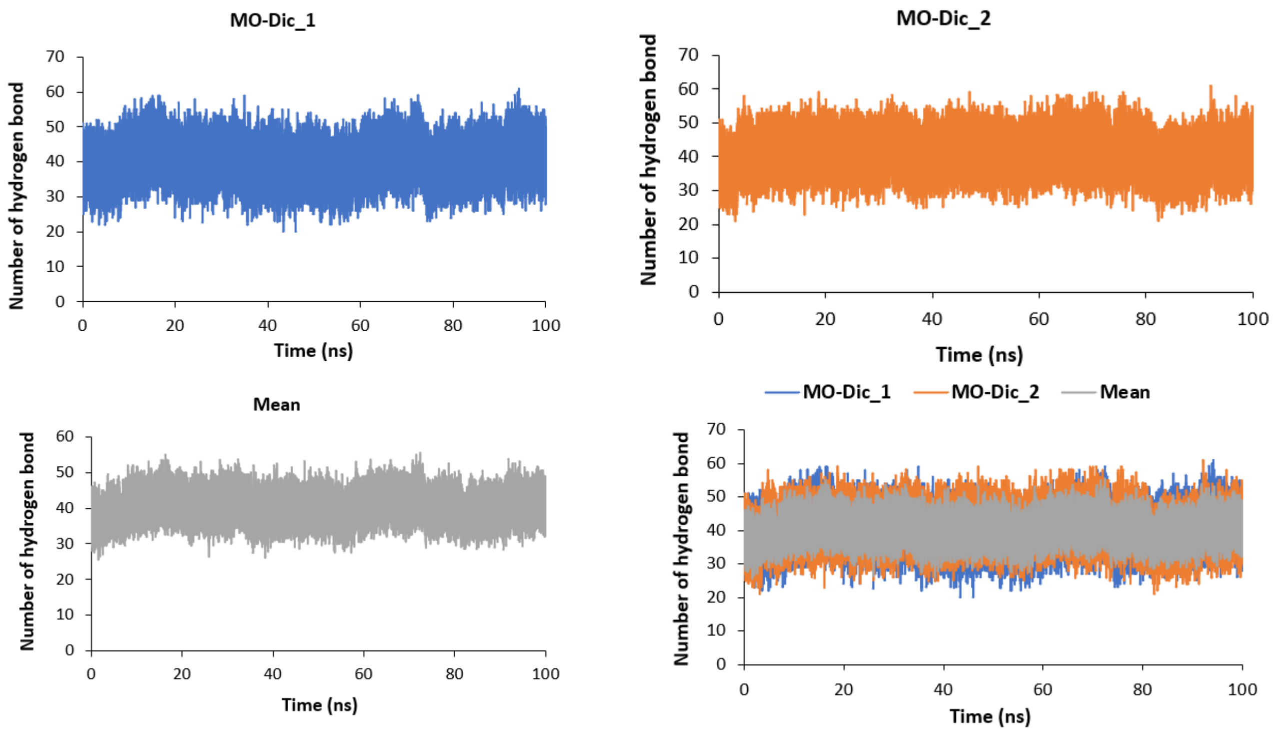

Figure S4(c): The plots of number of hydrogen bond of MO-Dic complex run in duplicate. MO-Dic_1 as first run, MO-Dic_2 as second run and mean as the average of the two runs.

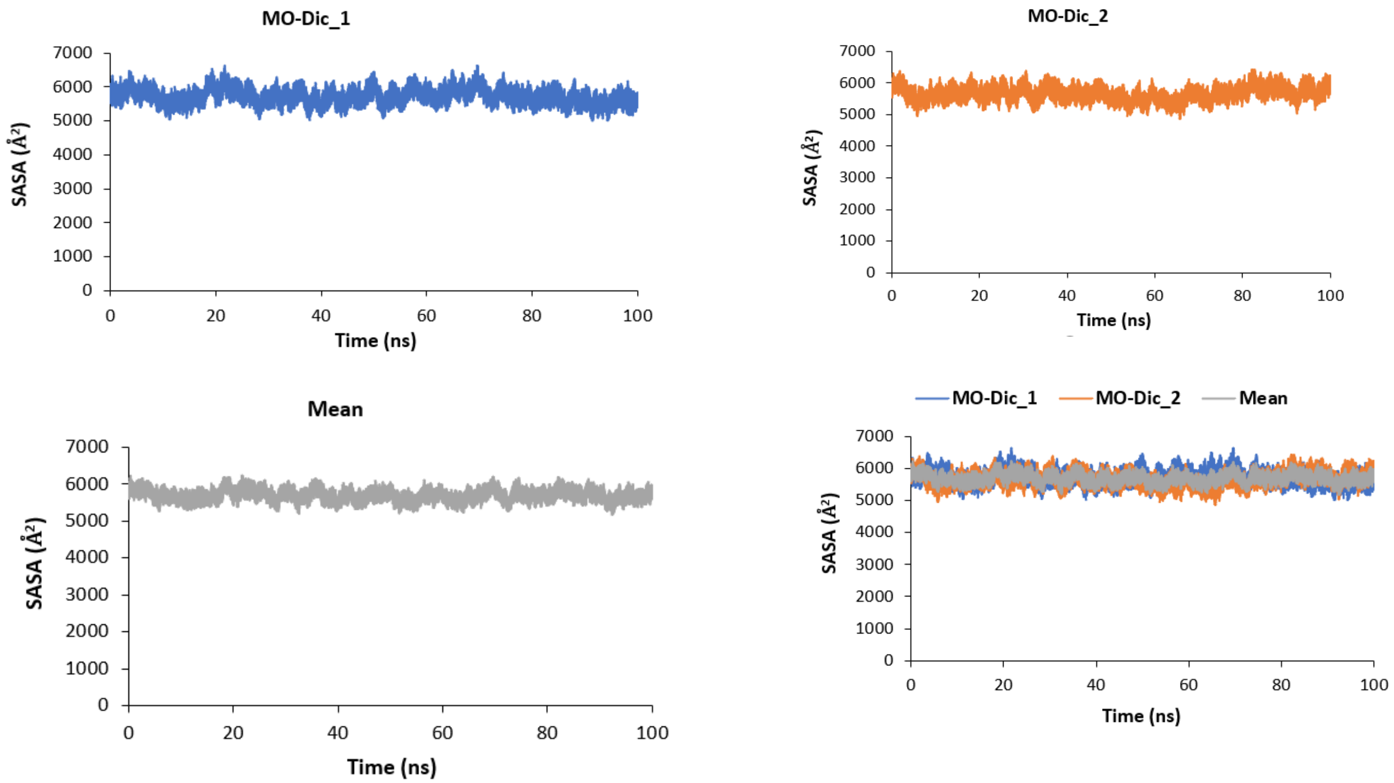

Figure S4(d): The plots of SASA for MO-Dic complex run in duplicate. MO-Dic_1 as first run, MO-Dic_2 as second run and mean as the average of the two runs.

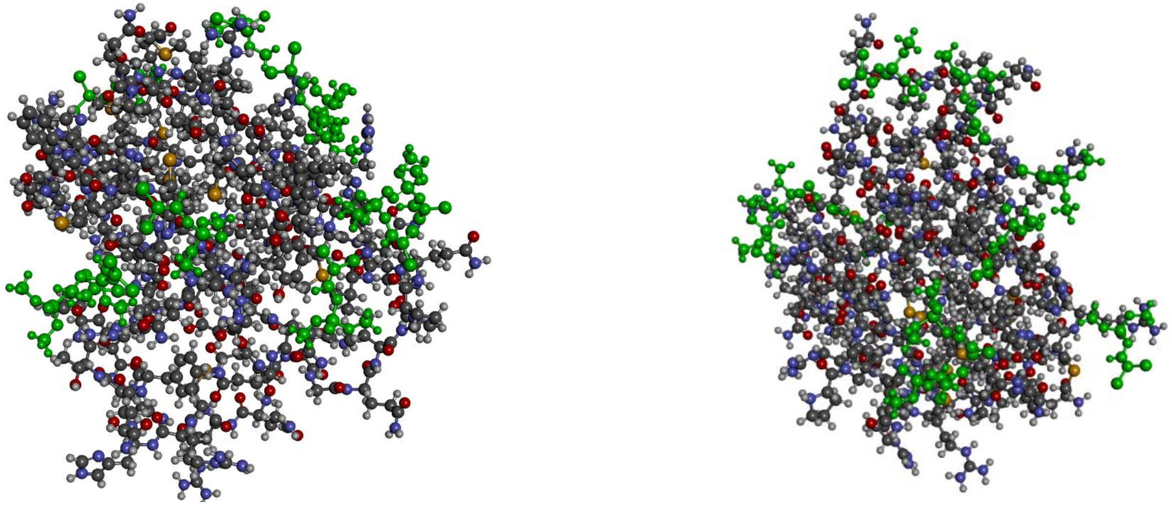

MO-Dic_1 **MO-Dic_2**

Figure S5: The representative snapshots of the clustering of the Dic pollutant around MO protein with 100% coagulation. MO-Dic_1 (binding energy of -85.60 kcal/mol) and MO-Dic_2 (binding energy of -88.68 kcal/mol) represent first and second run respectively.

Table S7: Prominent residues for MO-pollutants interactions based on decomposed binding energies (kcal/mol)

MO-Dic_2
TRP 88 (-2.967 +/- 0.984)
LEU 4 (-1.985 +/- 1.254)
VAL 48 (-1.600 +/- 0.551)
ILE 55 (-1.583 +/- 0.790)
ARG 51 (-1.512 +/- 0.784)
GLN 25 (-1.298 +/- 0.777)
PHE 17 (-1.039 +/- 1.218)
PRO 21 (-0.810 +/- 0.930)
PRO 64 (-0.602 +/- 0.866)
ARG 24 (-0.514 +/- 0.875)

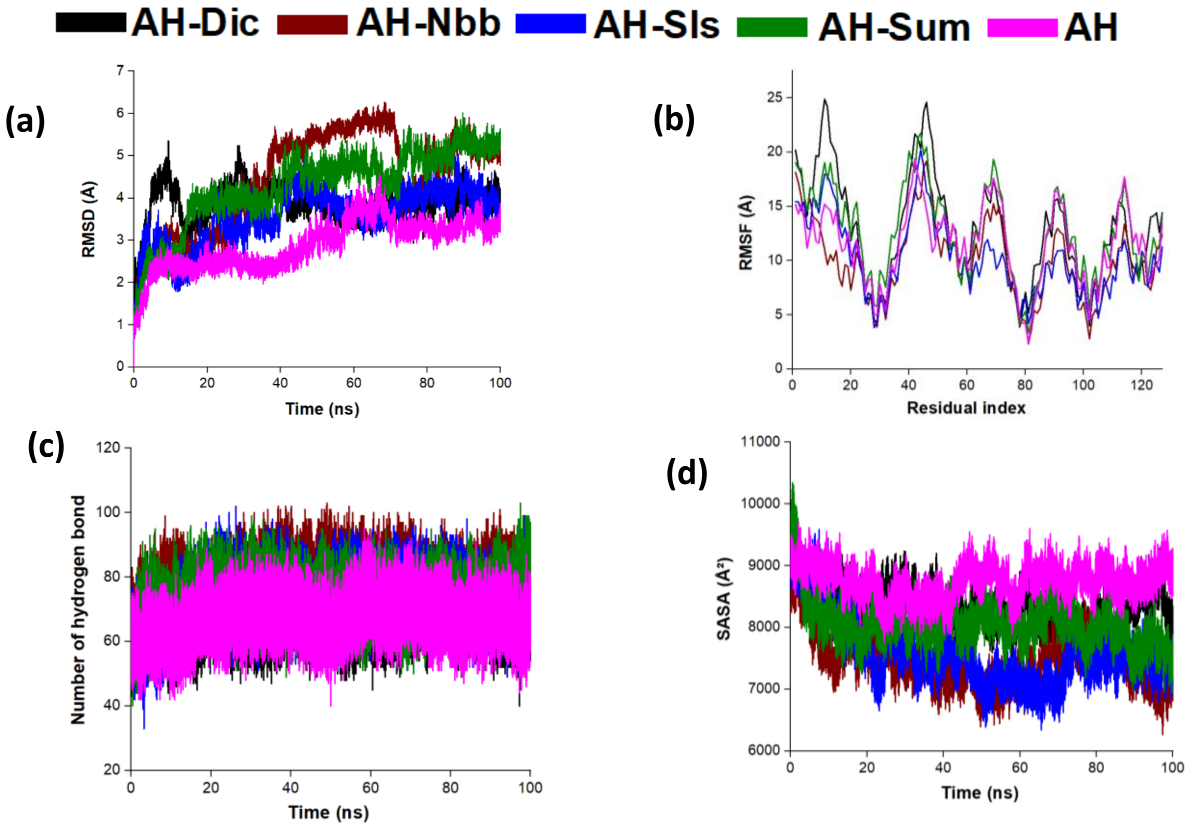

Figure S6: The stability of the AH protein without the ligands and the various complexes formed at 100 ns simulation time derived from the plot of (a) RMSD (Å), (b) RMSF (Å), (c) Number of hydrogen bond and (d) SASA (Å^2)

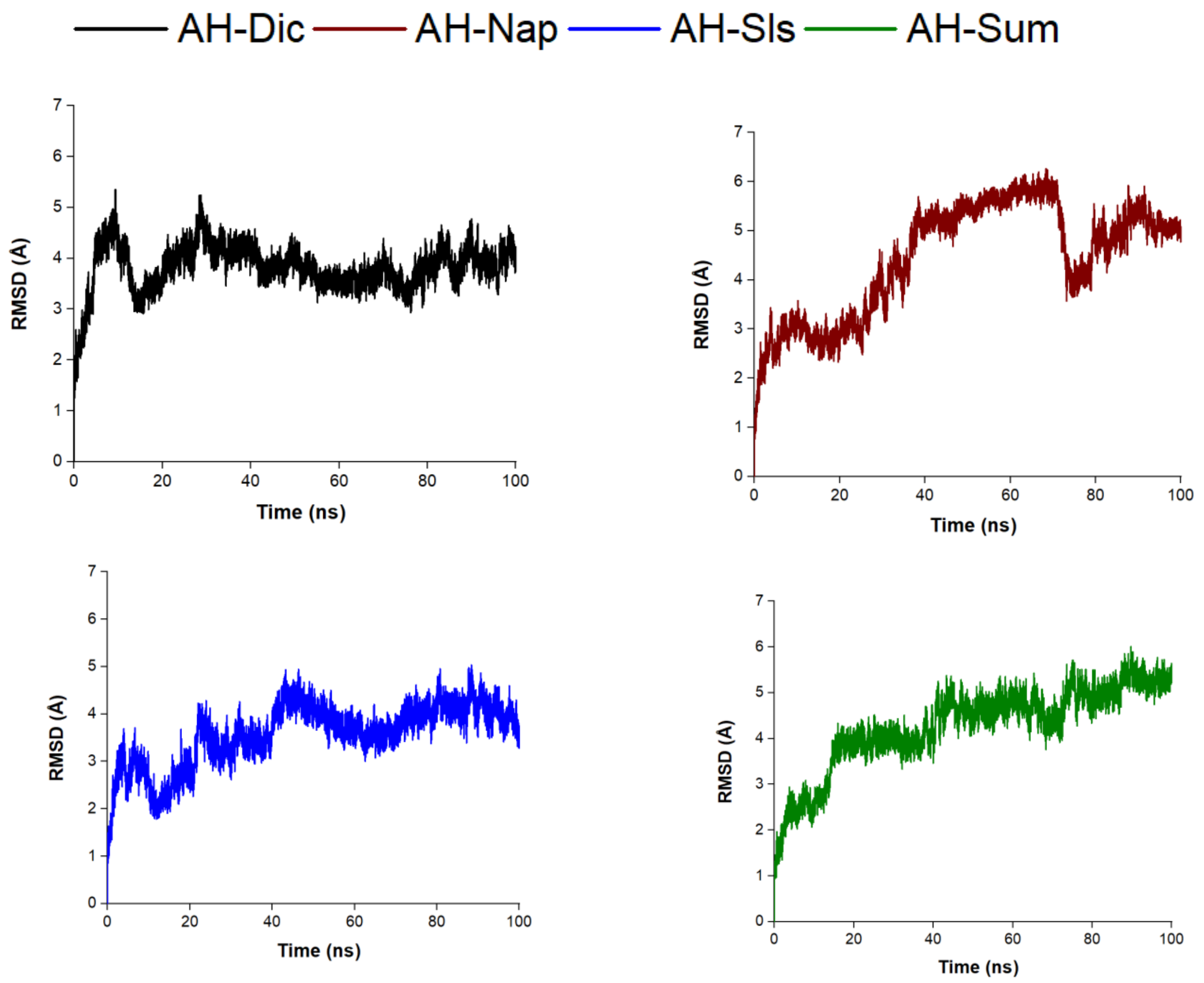

Figure S7(a): The stability of the various AH-pollutant complexes at 100 ns simulation time derived from the plot of RMSD

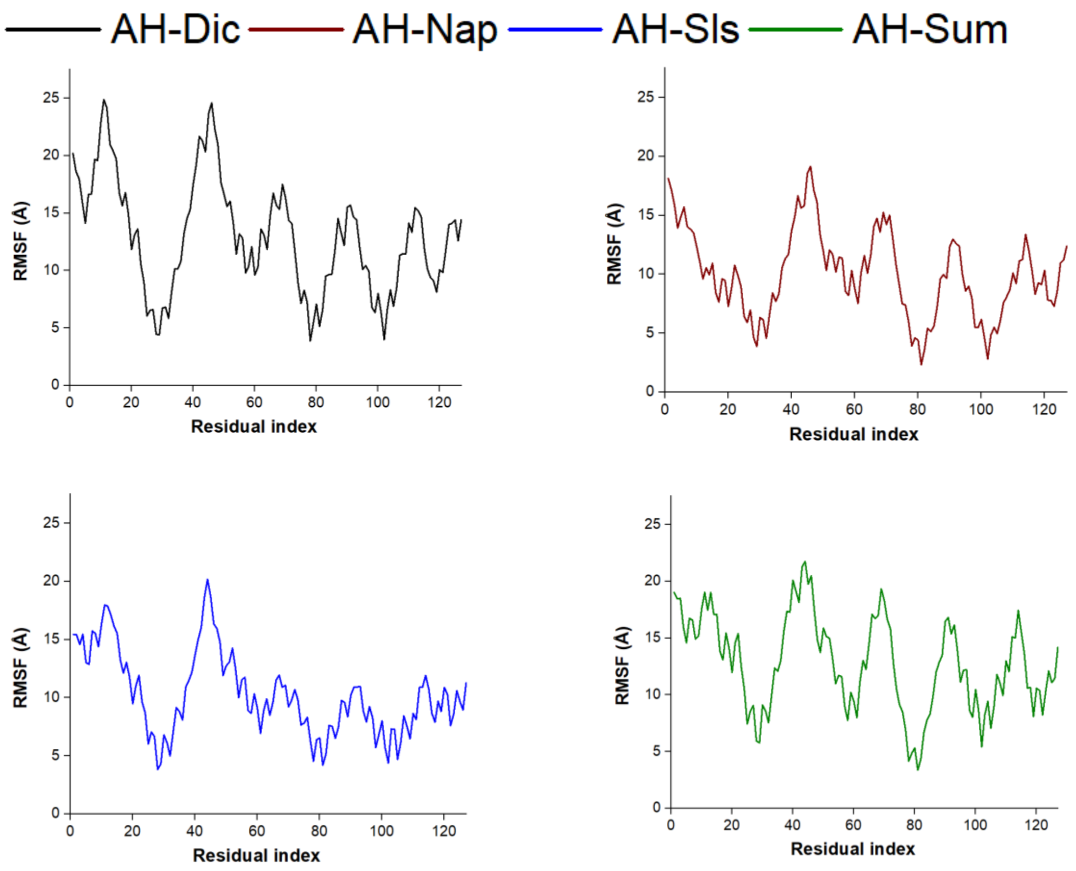

Figure S7(b): The stability of the various AH-pollutant complexes at 100 ns simulation time derived from the plot of RMSF

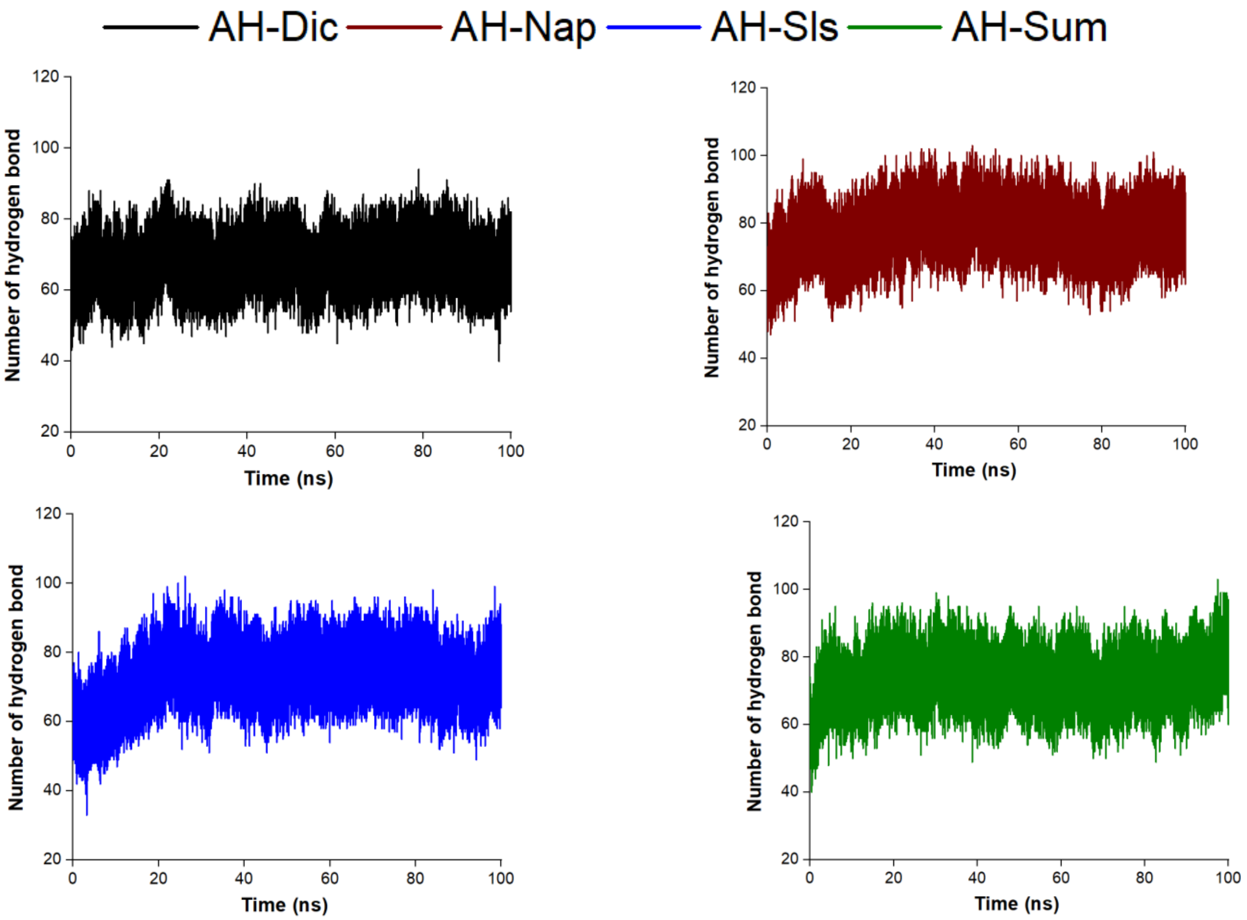

Figure S7(c): The stability of the various AH-pollutant complexes at 100 ns simulation time derived from the plot of number of hydrogen bond

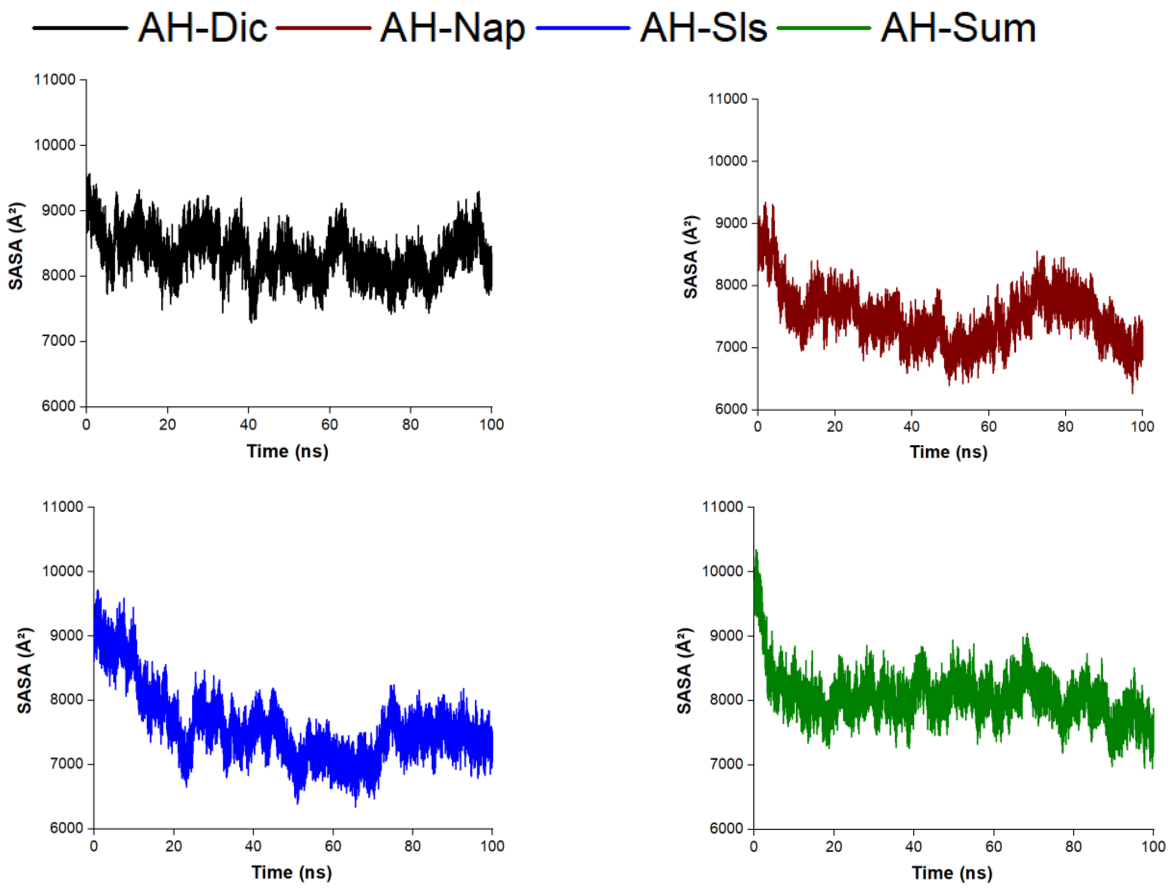

Figure S7(d): The stability of the various AH-pollutant complexes at 100 ns simulation time derived from the plot of SASA

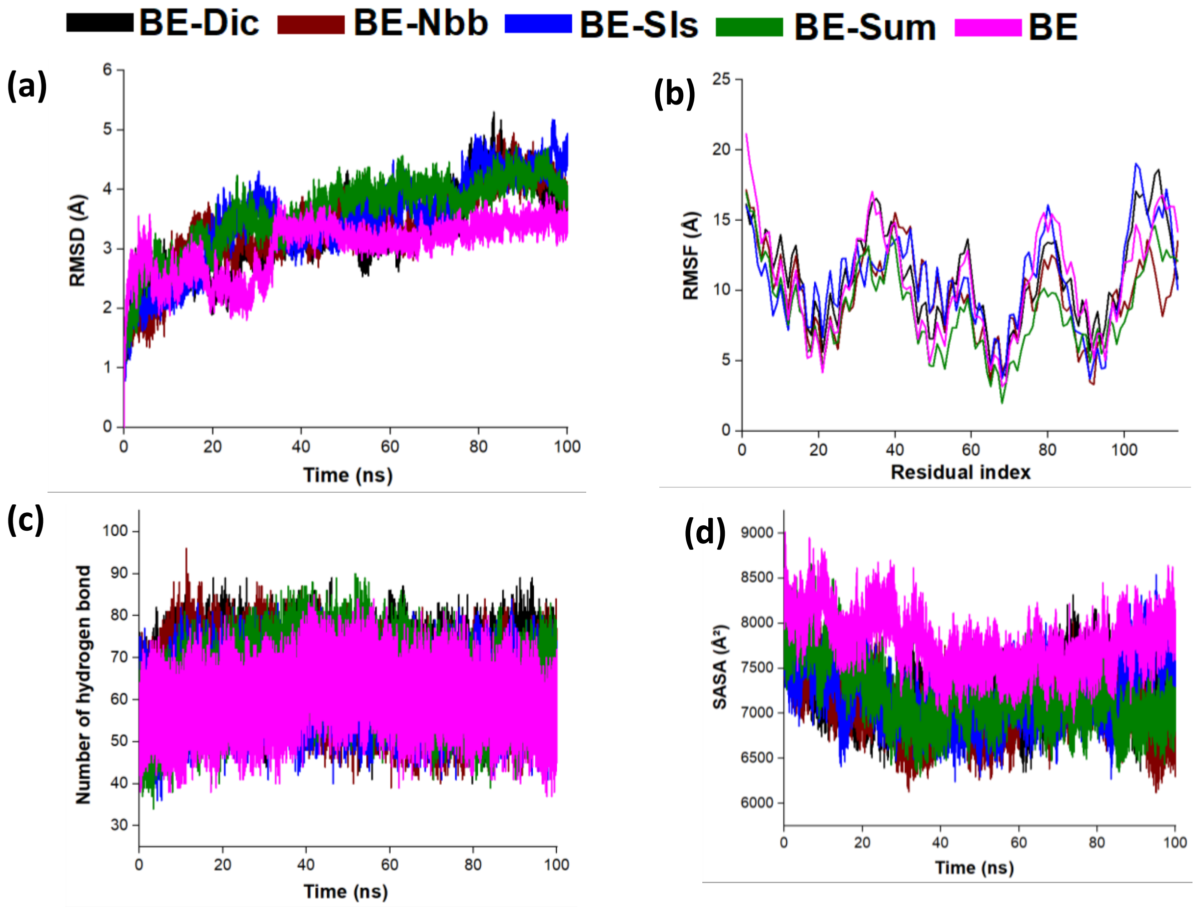

Figure S8: The stability of the BE protein without the ligands and the various complexes formed at 100 ns simulation time derived from the plot of (a) RMSD (Å), (b) RMSF (Å), (c) Number of hydrogen bond and (d) SASA (Å^2)

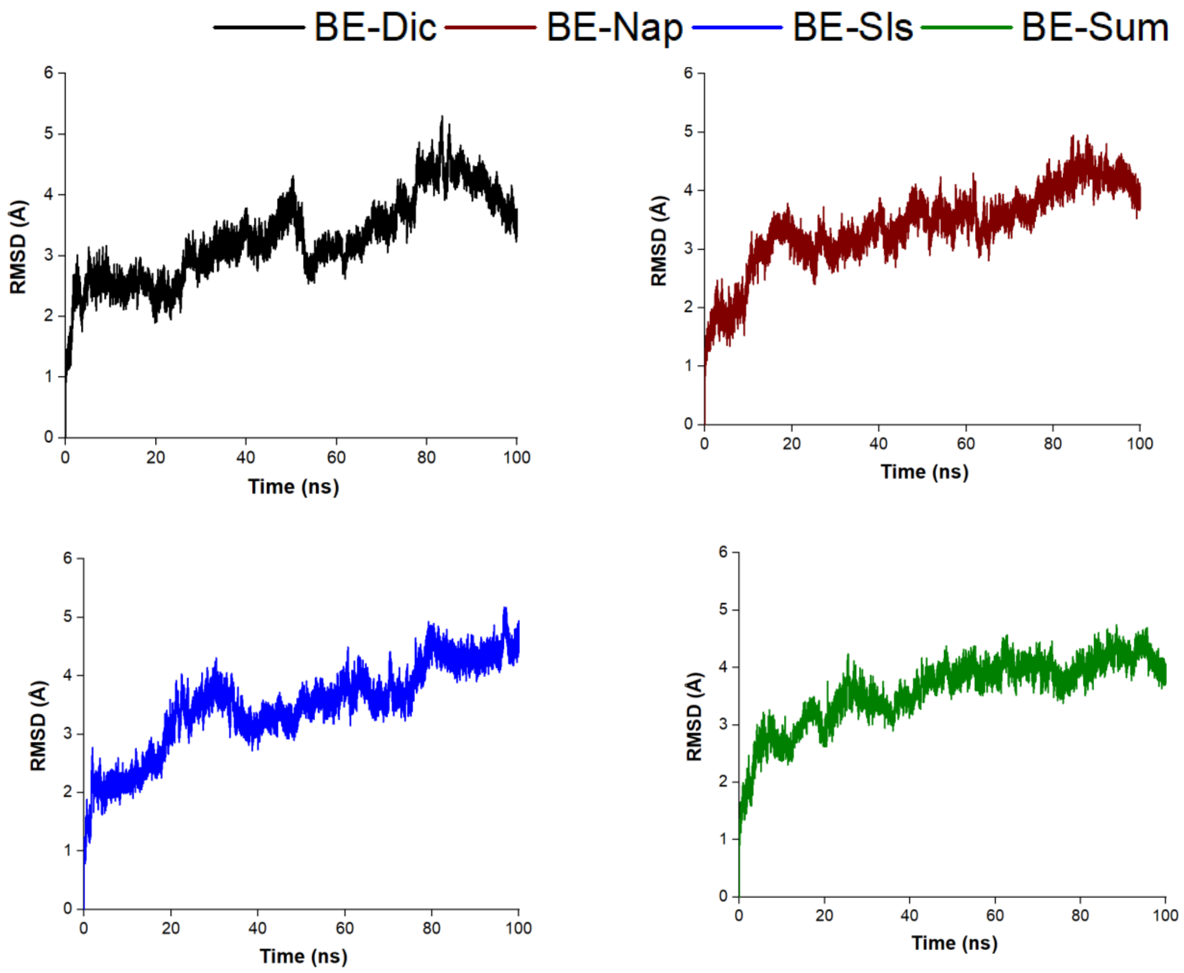

Figure S9(a): The stability of the various BE-pollutant complexes at 100 ns simulation time derived from the plot of RMSD

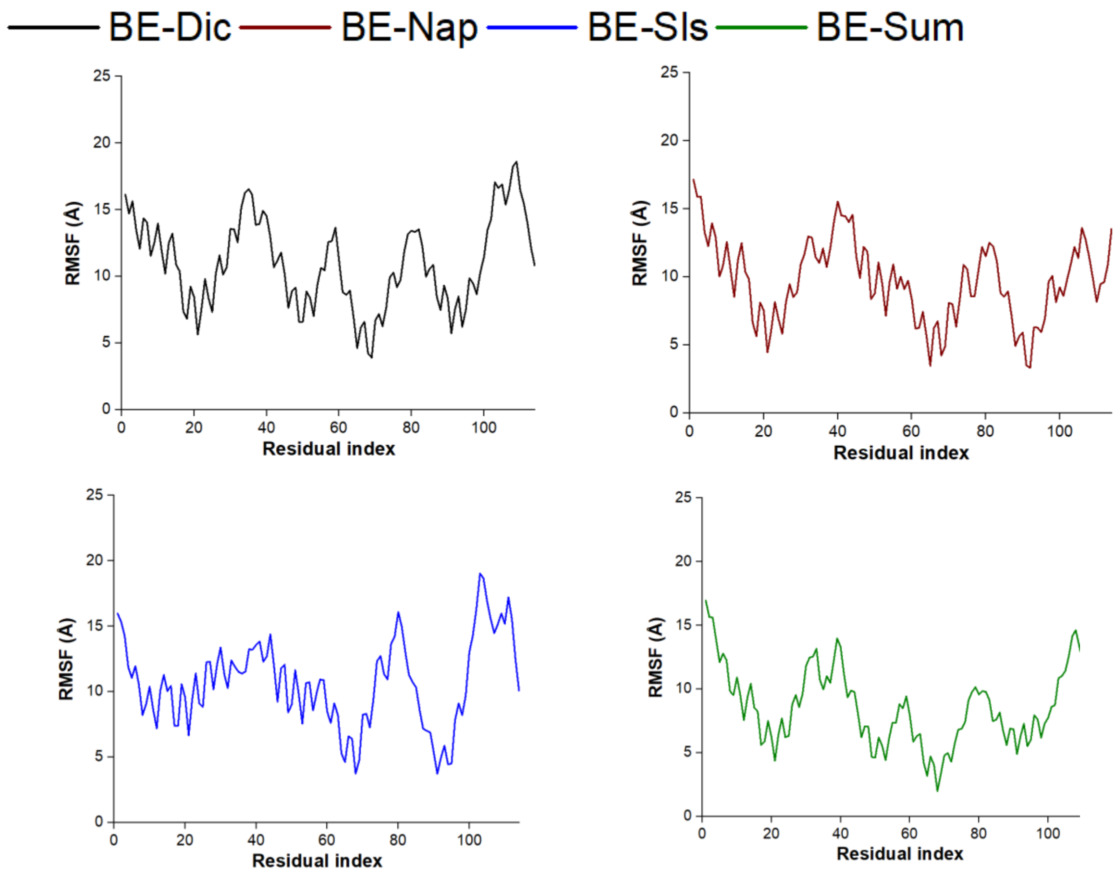

Figure S9(b): The stability of the various BE-pollutant complexes at 100 ns simulation time derived from the plot of RMSF

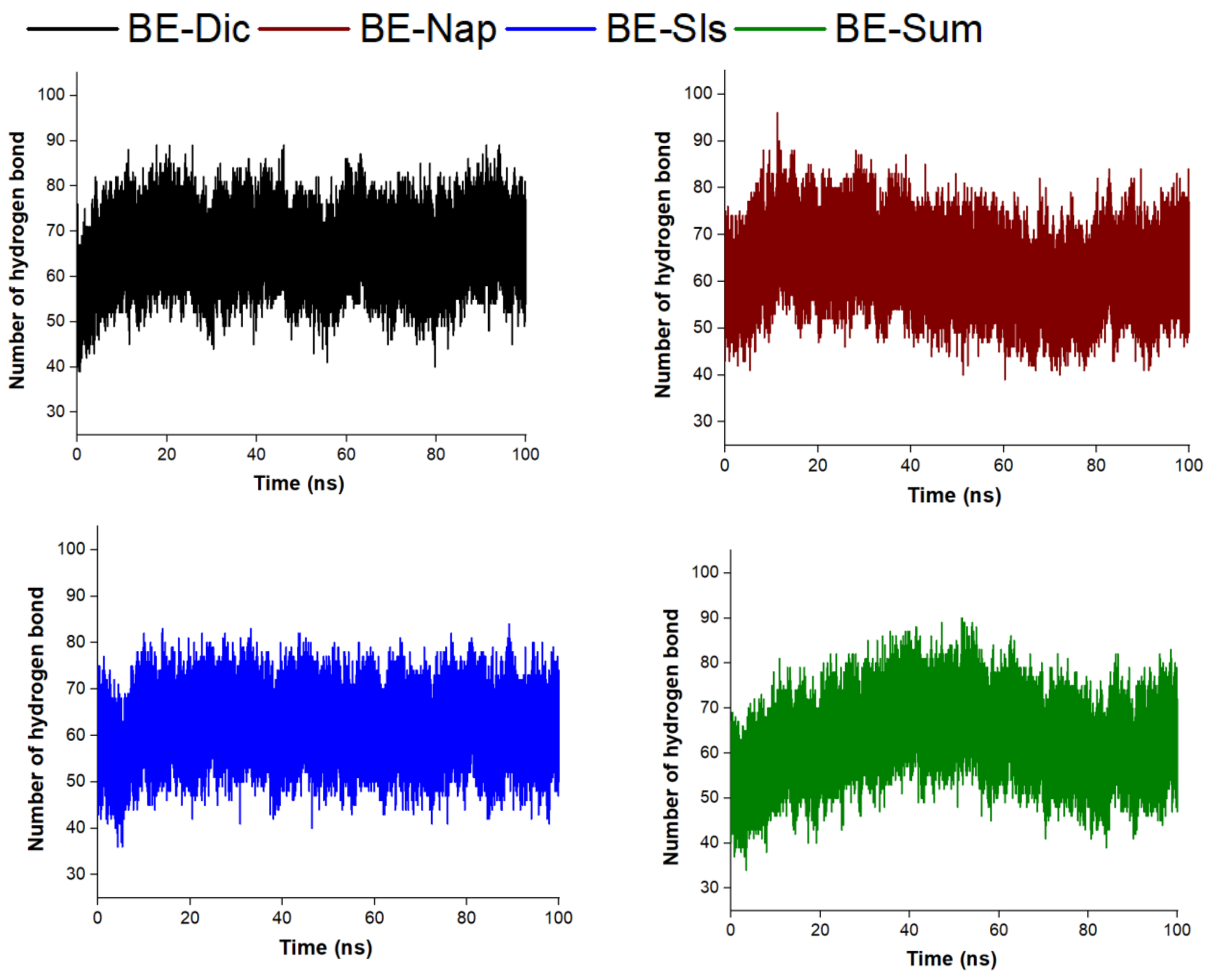

Figure S9(c): The stability of the various BE-pollutant complexes at 100 ns simulation time derived from the plot of number of hydrogen bond.

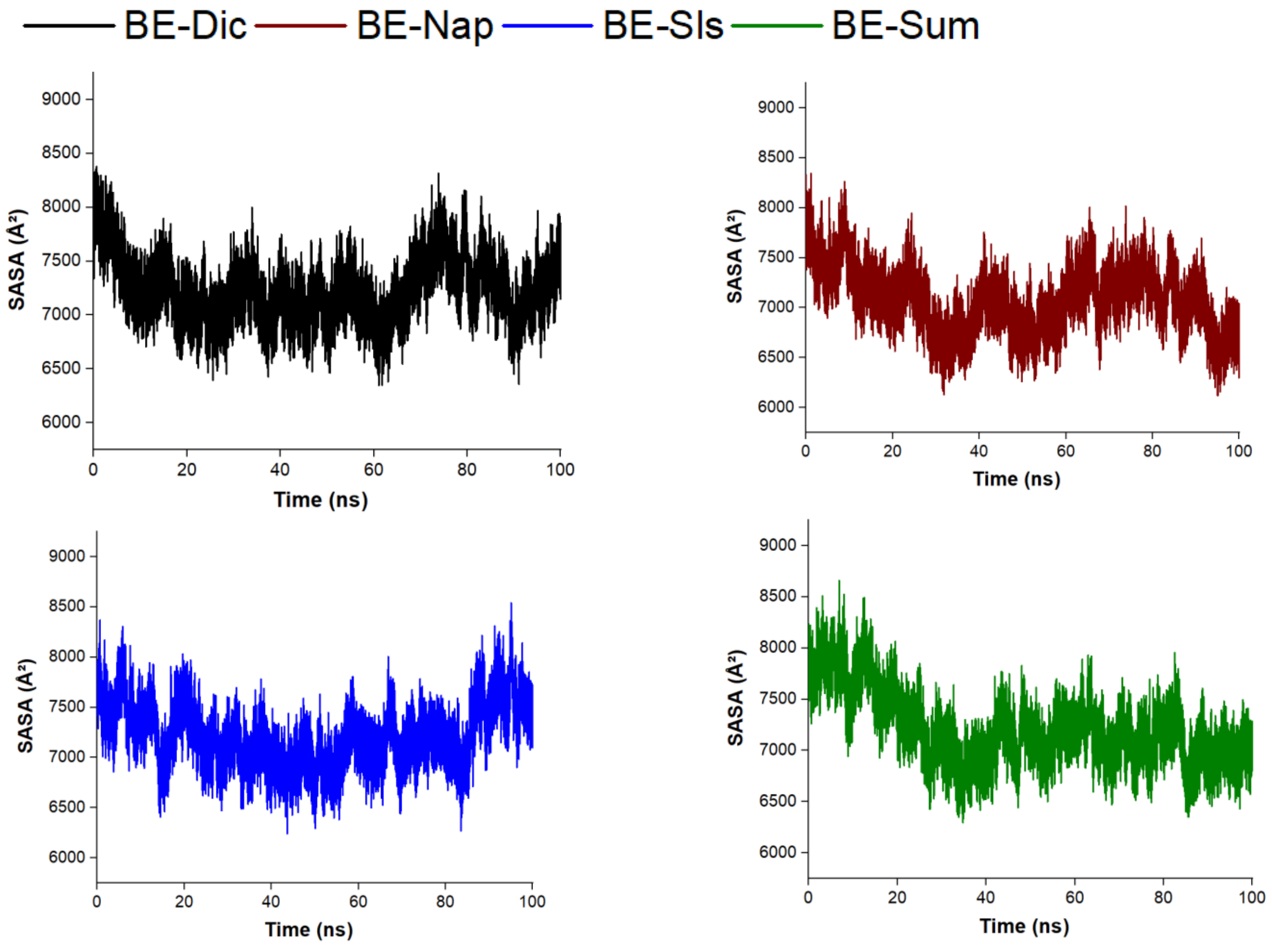

Figure S9(d): The stability of the various BE-pollutant complexes at 100 ns simulation time derived from the plot of SASA.

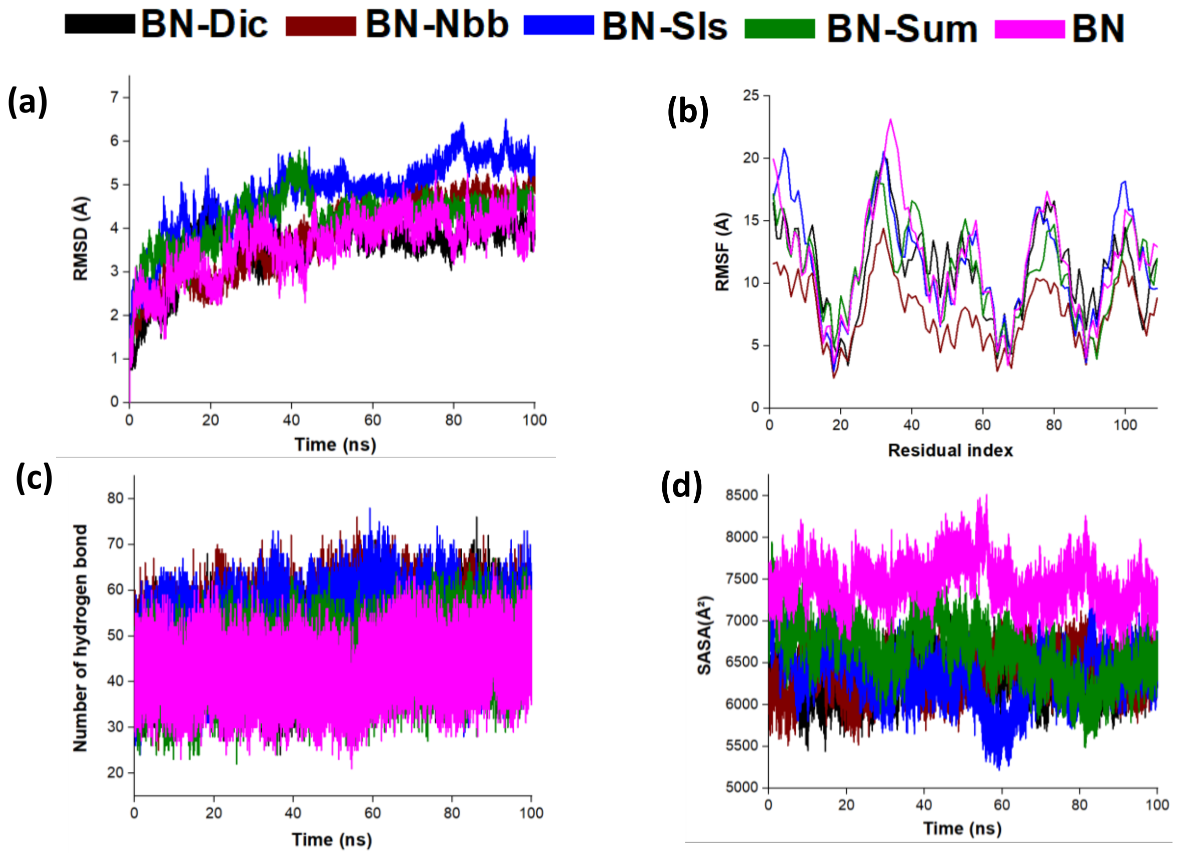

Figure S10: The stability of the BN protein without the ligands and the various complexes formed at 100 ns simulation time derived from the plot of (a) RMSD (Å), (b) RMSF (Å), (c) Number of hydrogen bond and (d) SASA (Å^2)

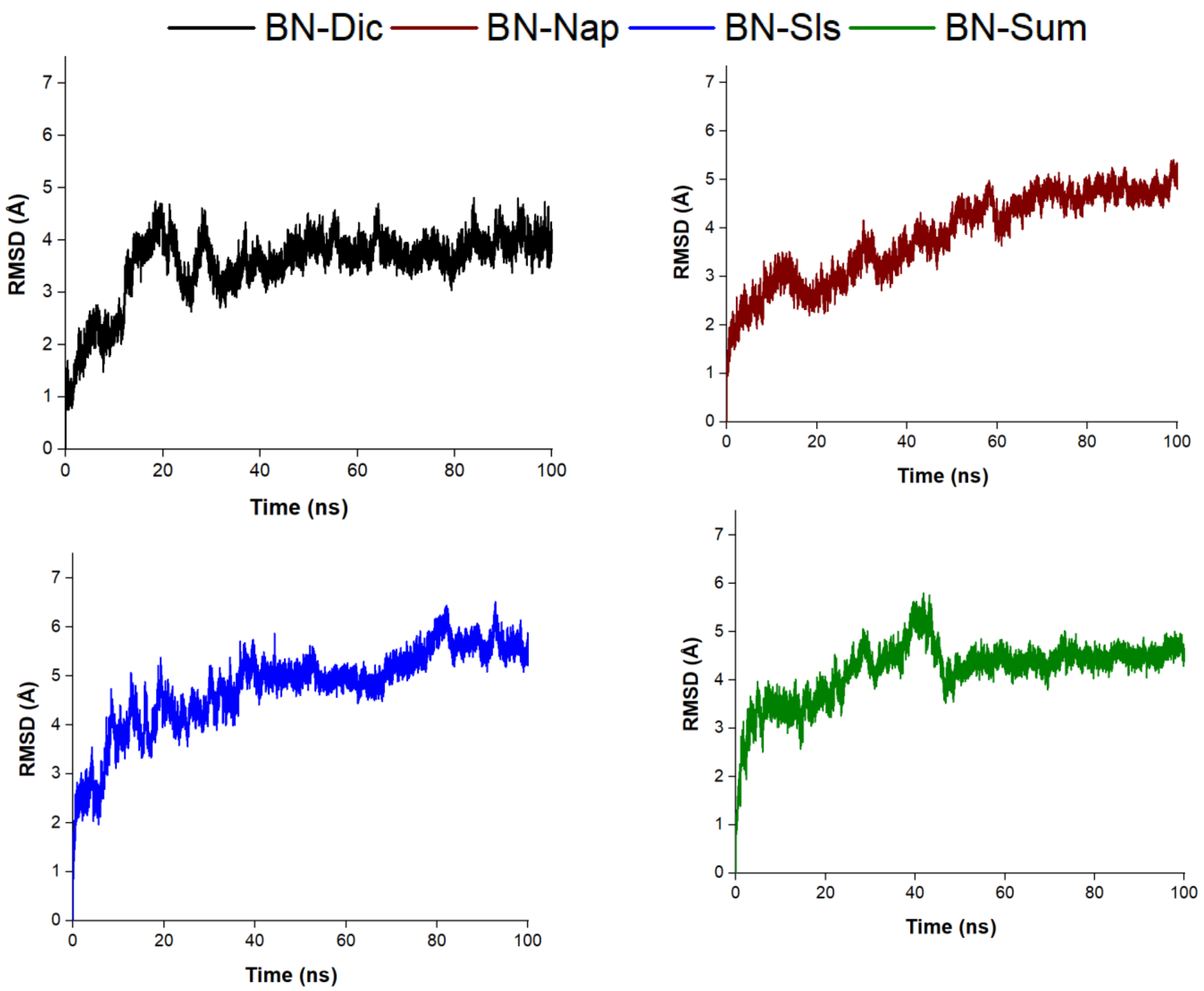

Figure S11(a): The stability of the various BN-pollutant complexes at 100 ns simulation time derived from the plot of RMSD.

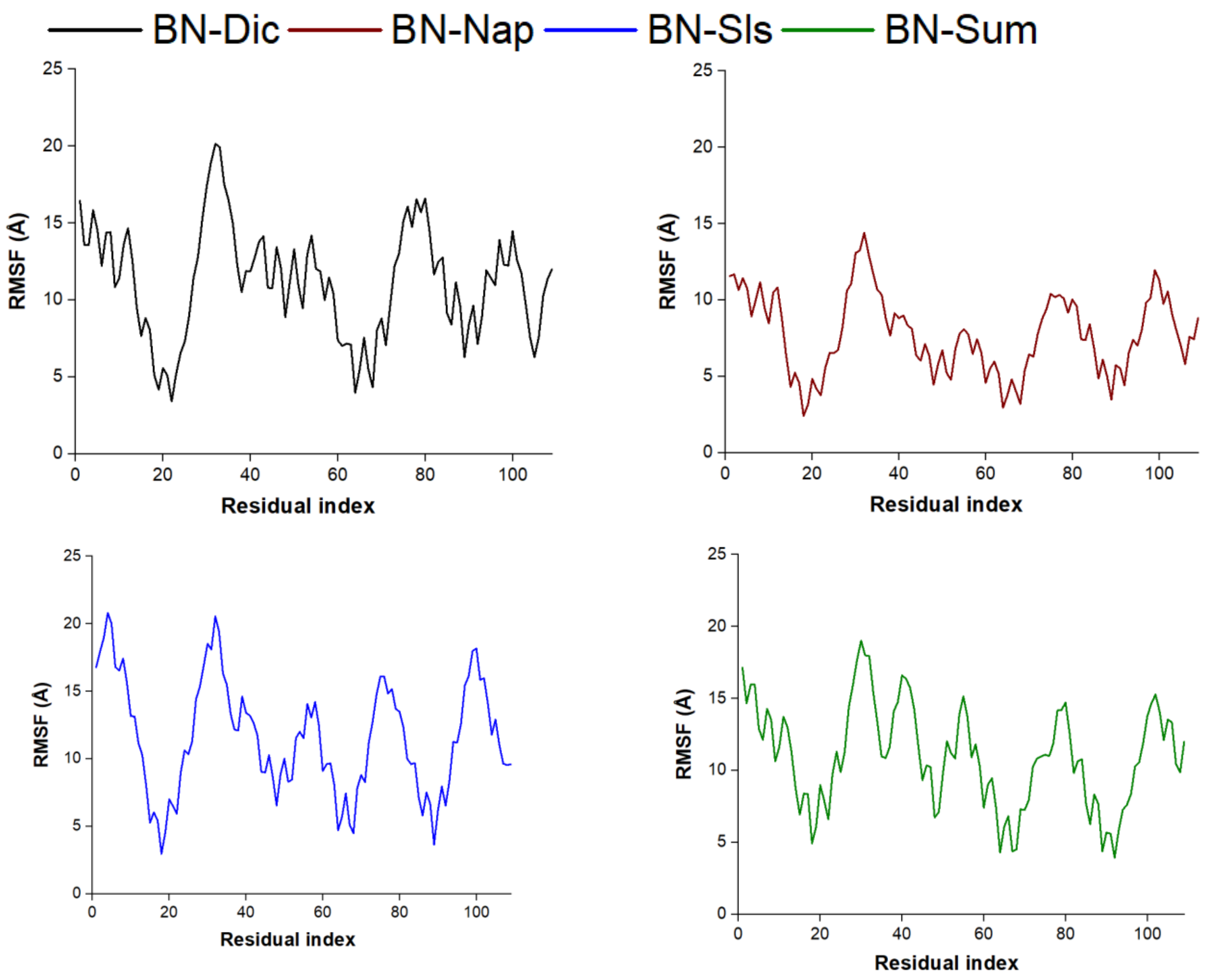

Figure S11(b): The stability of the various BN-pollutant complexes at 100 ns simulation time derived from the plot of RMSF.

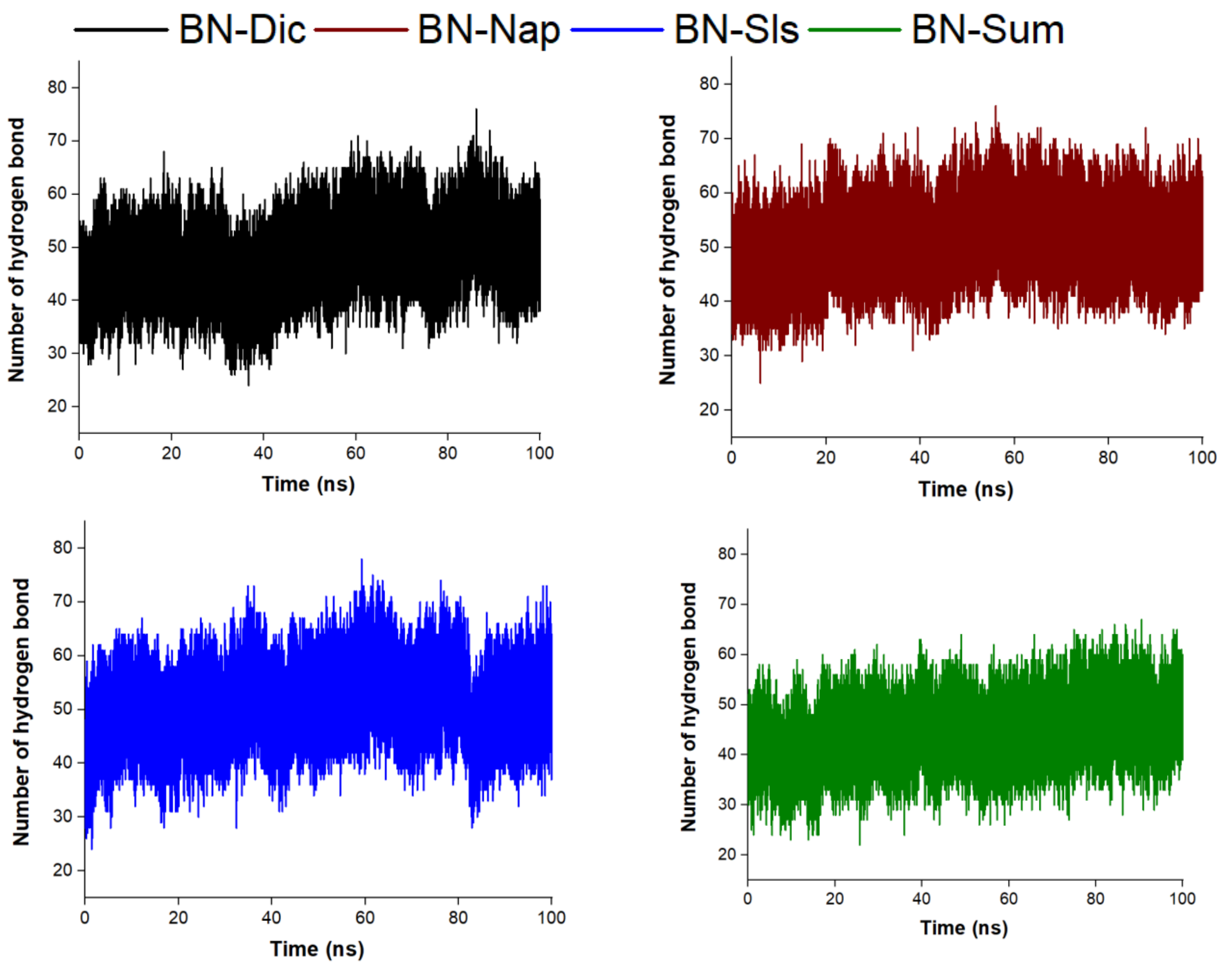

Figure S11(c): The stability of the various BN-pollutant complexes at 100 ns simulation time derived from the plot of number of hydrogen bond.

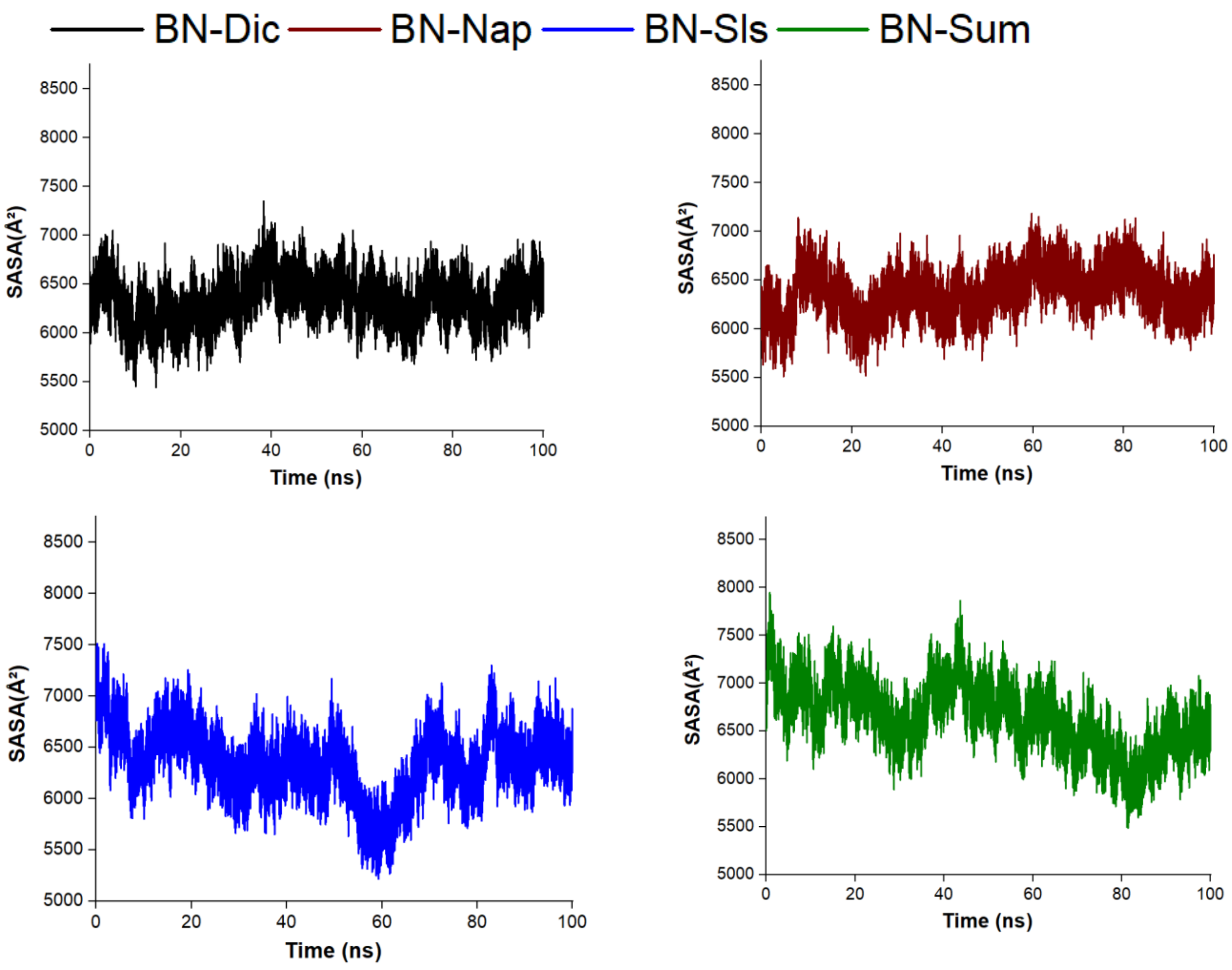

Figure S11(d): The stability of the various BN-pollutant complexes at 100 ns simulation time derived from the plot of SASA.

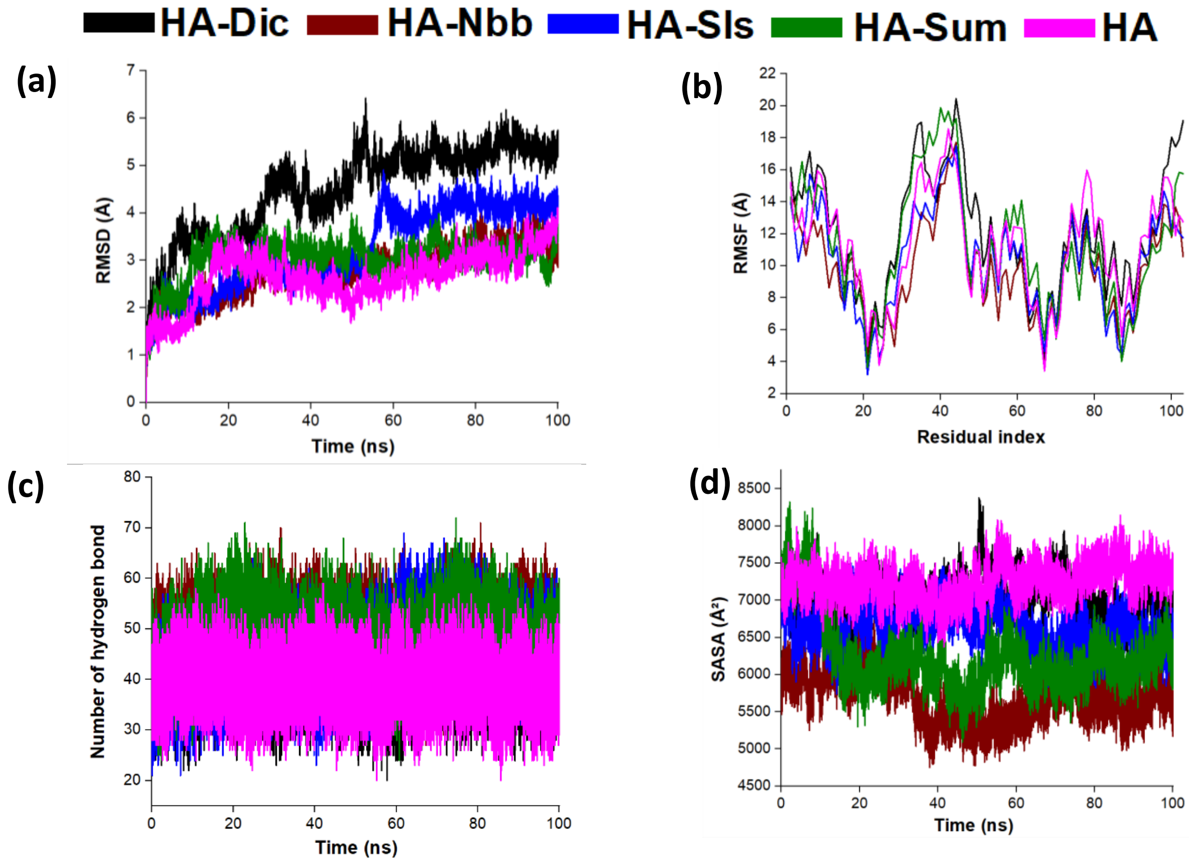

Figure S12: The stability of the HA protein without the ligands and the various complexes formed at 100 ns simulation time derived from the plot of (a) RMSD (Å), (b) RMSF (Å), (c) Number of hydrogen bond and (d) SASA (Å^2)

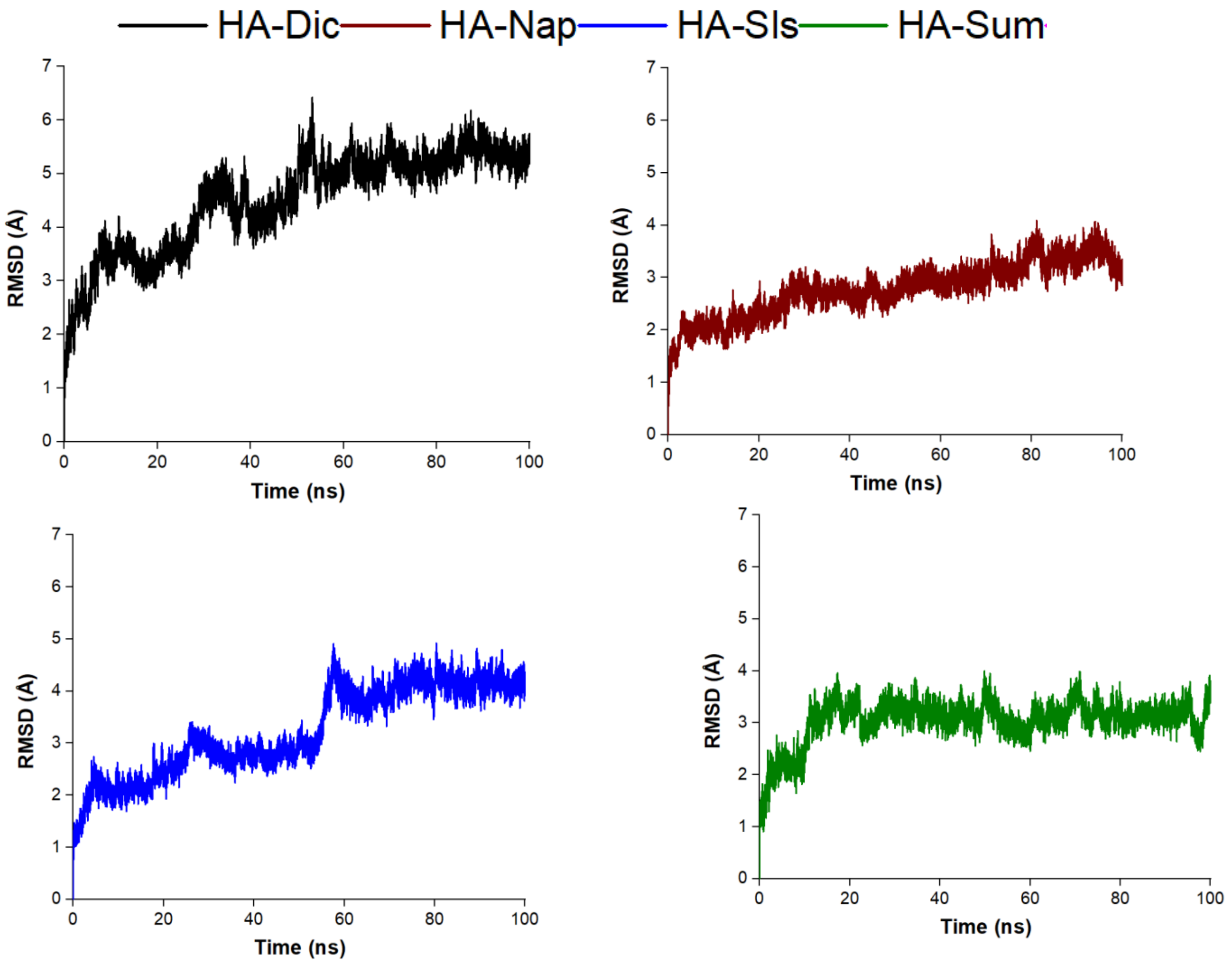

Figure S13(a): The stability of the various HA-pollutant complexes at 100 ns simulation time derived from the plot of RMSD.

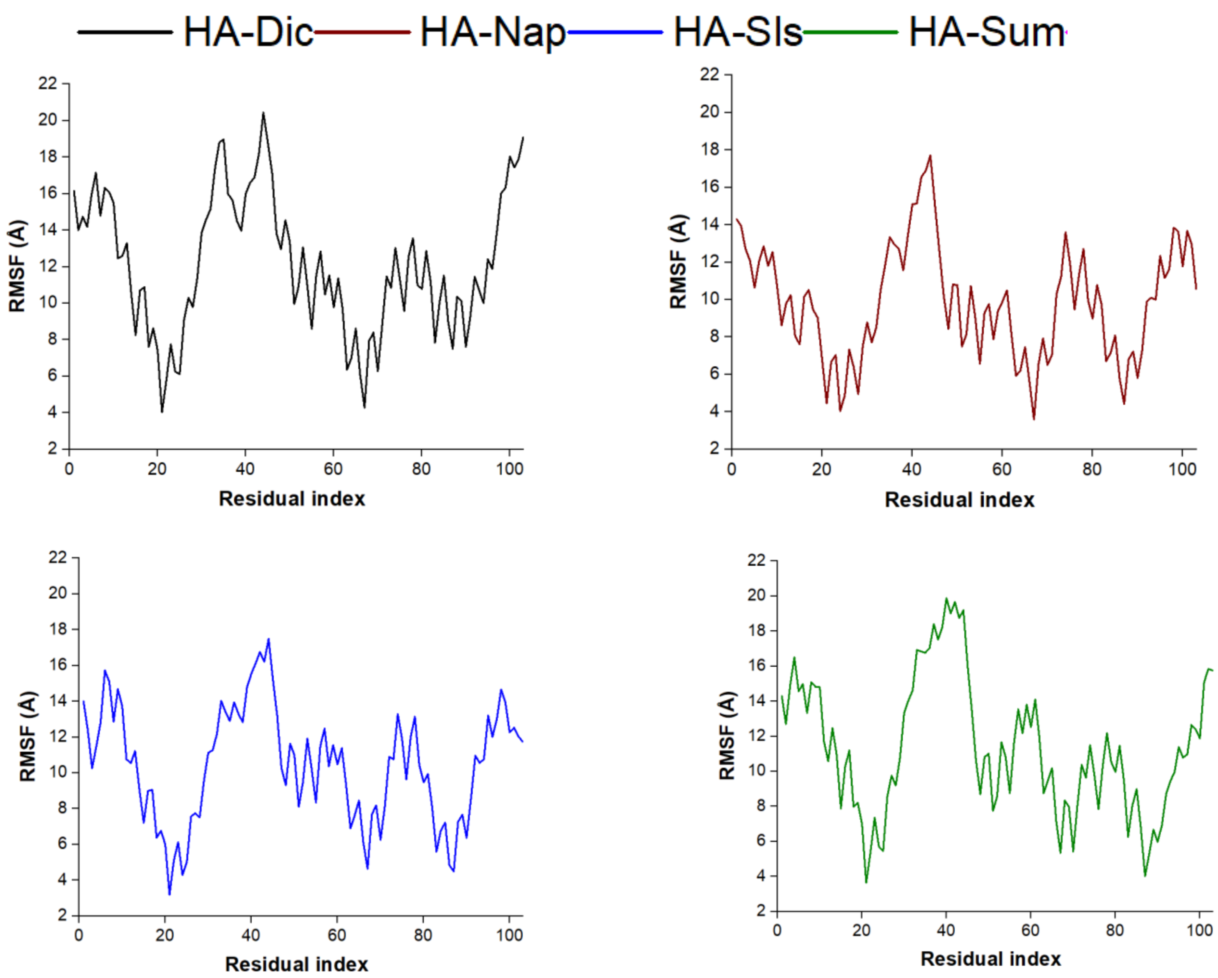

Figure S13(b): The stability of the various HA-pollutant complexes at 100 ns simulation time derived from the plot of RMSF.

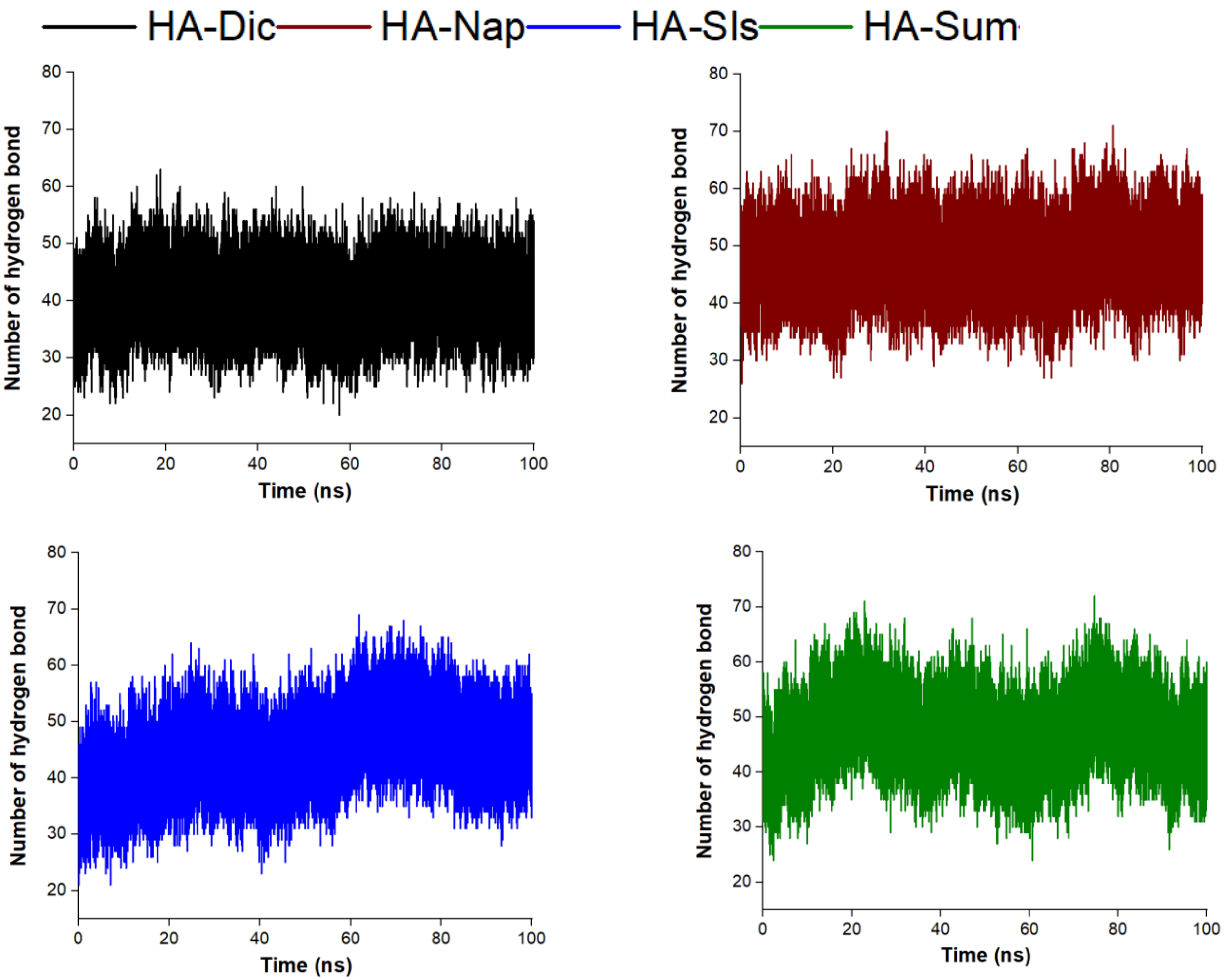

Figure S13(c): The stability of the various HA-pollutant complexes at 100 ns simulation time derived from the plot of number of hydrogen bond.

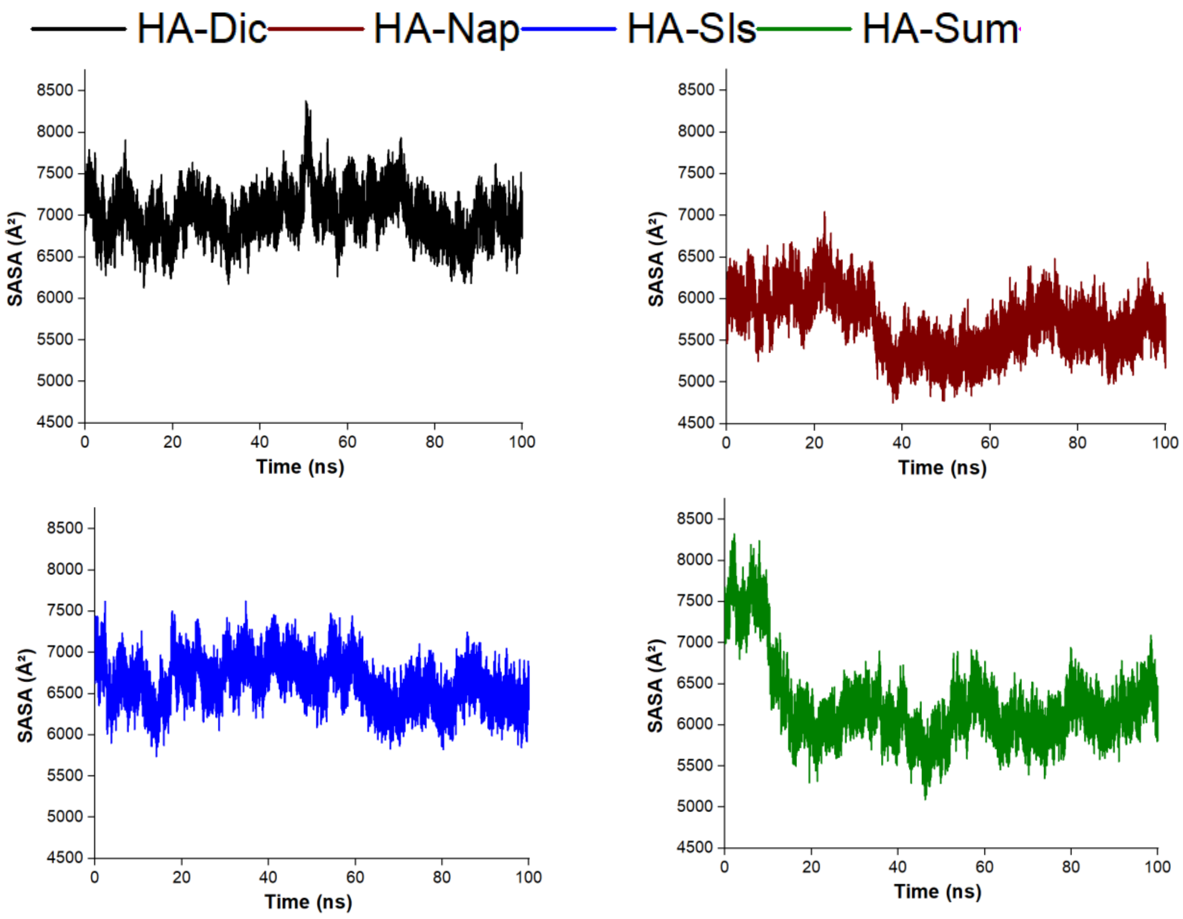

Figure S13(d): The stability of the various HA-pollutant complexes at 100 ns simulation time derived from the plot of SASA.

Table A: The number of atoms present in the system of AH-Dic

ATOM	1	N	GLY	1	21.027	-11.862	-0.185	1.00	0.00
ATOM	2	H1	GLY	1	20.326	-11.147	-0.309	1.00	0.00
ATOM	3	H2	GLY	1	20.642	-12.749	-0.477	1.00	0.00
ATOM	4	H3	GLY	1	21.898	-11.536	-0.580	1.00	0.00
ATOM	5	CA	GLY	1	21.287	-12.007	1.275	1.00	0.00
ATOM	6	HA2	GLY	1	21.055	-13.023	1.596	1.00	0.00
ATOM	7	HA3	GLY	1	22.333	-11.789	1.491	1.00	0.00
ATOM	8	C	GLY	1	20.403	-11.026	2.049	1.00	0.00
ATOM	9	O	GLY	1	20.874	-10.037	2.576	1.00	0.00
ATOM	10	N	PRO	2	19.096	-11.311	2.115	1.00	0.00
ATOM	11	CD	PRO	2	18.453	-12.487	1.503	1.00	0.00
ATOM	12	HD2	PRO	2	19.014	-13.385	1.764	1.00	0.00
ATOM	13	HD3	PRO	2	18.439	-12.371	0.419	1.00	0.00
ATOM	14	CG	PRO	2	17.074	-12.474	2.098	1.00	0.00
ATOM	15	HG2	PRO	2	17.058	-13.108	2.985	1.00	0.00
ATOM	16	HG3	PRO	2	16.362	-12.856	1.367	1.00	0.00
ATOM	17	CB	PRO	2	16.778	-11.053	2.438	1.00	0.00
ATOM	18	HB2	PRO	2	16.109	-11.039	3.298	1.00	0.00
ATOM	19	HB3	PRO	2	16.337	-10.476	1.625	1.00	0.00

```
ATOM    20  CA  PRO     2      18.132 -10.459   2.824  1.00  0.00
ATOM    21  HA  PRO     2      18.271  -9.432   2.488  1.00  0.00
ATOM    22  C   PRO     2      18.321 -10.529   4.342  1.00  0.00
ATOM    23  O   PRO     2      17.876  -9.666   5.073  1.00  0.00
ATOM    24  N   MET     3      18.977 -11.550   4.821  1.00  0.00
ATOM    25  H   MET     3      19.350 -12.272   4.220  1.00  0.00
ATOM    26  CA  MET     3      19.192 -11.673   6.291  1.00  0.00
ATOM    27  HA  MET     3      18.236 -11.761   6.807  1.00  0.00
ATOM    28  CB  MET     3      20.021 -12.926   6.581  1.00  0.00
ATOM    29  HB2 MET     3      20.954 -12.884   6.018  1.00  0.00
ATOM    30  HB3 MET     3      20.243 -12.978   7.647  1.00  0.00
ATOM    31  CG  MET     3      19.230 -14.168   6.164  1.00  0.00
ATOM    32  HG2 MET     3      19.043 -14.143   5.090  1.00  0.00
ATOM    33  HG3 MET     3      19.796 -15.066   6.413  1.00  0.00
ATOM    34  SD  MET     3      17.648 -14.199   7.043  1.00  0.00
ATOM    35  CE  MET     3      18.322 -14.277   8.721  1.00  0.00
ATOM    36  HE1 MET     3      18.939 -13.398   8.907  1.00  0.00
ATOM    37  HE2 MET     3      17.504 -14.305   9.441  1.00  0.00
ATOM    38  HE3 MET     3      18.930 -15.176   8.828  1.00  0.00
ATOM    39  C   MET     3      19.936 -10.438   6.802  1.00  0.00
```

```
ATOM    40  O   MET    3     19.690  -9.960   7.892  1.00  0.00
ATOM    41  N   ARG    4     20.846  -9.917   6.024  1.00  0.00
ATOM    42  H   ARG    4     21.058 -10.310   5.118  1.00  0.00
ATOM    43  CA  ARG    4     21.603  -8.713   6.466  1.00  0.00
ATOM    44  HA  ARG    4     22.187  -8.941   7.358  1.00  0.00
ATOM    45  CB  ARG    4     22.558  -8.273   5.355  1.00  0.00
ATOM    46  HB2 ARG    4     21.995  -8.106   4.437  1.00  0.00
ATOM    47  HB3 ARG    4     23.056  -7.349   5.649  1.00  0.00
ATOM    48  CG  ARG    4     23.605  -9.363   5.119  1.00  0.00
ATOM    49  HG2 ARG    4     24.110  -9.592   6.057  1.00  0.00
ATOM    50  HG3 ARG    4     23.117 -10.262   4.742  1.00  0.00
ATOM    51  CD  ARG    4     24.630  -8.872   4.095  1.00  0.00
ATOM    52  HD2 ARG    4     25.152  -7.994   4.475  1.00  0.00
ATOM    53  HD3 ARG    4     25.353  -9.659   3.878  1.00  0.00
ATOM    54  NE  ARG    4     23.933  -8.503   2.832  1.00  0.00
ATOM    55  HE  ARG    4     23.586  -9.208   2.197  1.00  0.00
ATOM    56  CZ  ARG    4     23.759  -7.246   2.524  1.00  0.00
ATOM    57  NH1 ARG    4     24.758  -6.410   2.608  1.00  0.00
ATOM    58 HH11 ARG    4     25.664  -6.739   2.911  1.00  0.00
ATOM    59 HH12 ARG    4     24.621  -5.439   2.369  1.00  0.00
```

ATOM 60 NH2 ARG 4 22.588 -6.825 2.133 1.00 0.00

ATOM 61 HH21 ARG 4 21.817 -7.475 2.069 1.00 0.00

ATOM 62 HH22 ARG 4 22.458 -5.852 1.896 1.00 0.00

ATOM 63 C ARG 4 20.624 -7.579 6.774 1.00 0.00

ATOM 64 O ARG 4 20.819 -6.809 7.693 1.00 0.00

ATOM 65 N ARG 5 19.570 -7.470 6.011 1.00 0.00

ATOM 66 H ARG 5 19.402 -8.106 5.244 1.00 0.00

ATOM 67 CA ARG 5 18.579 -6.386 6.261 1.00 0.00

ATOM 68 HA ARG 5 19.051 -5.410 6.154 1.00 0.00

ATOM 69 CB ARG 5 17.440 -6.493 5.244 1.00 0.00

ATOM 70 HB2 ARG 5 16.988 -7.483 5.307 1.00 0.00

ATOM 71 HB3 ARG 5 16.687 -5.735 5.460 1.00 0.00

ATOM 72 CG ARG 5 17.993 -6.275 3.834 1.00 0.00

ATOM 73 HG2 ARG 5 18.505 -5.314 3.788 1.00 0.00

ATOM 74 HG3 ARG 5 18.696 -7.073 3.591 1.00 0.00

ATOM 75 CD ARG 5 16.841 -6.287 2.828 1.00 0.00

ATOM 76 HD2 ARG 5 16.243 -7.190 2.950 1.00 0.00

ATOM 77 HD3 ARG 5 16.209 -5.411 2.968 1.00 0.00

ATOM 78 NE ARG 5 17.391 -6.263 1.443 1.00 0.00

ATOM 79 HE ARG 5 17.918 -5.474 1.098 1.00 0.00

ATOM 80 CZ ARG 5 17.196 -7.278 0.646 1.00 0.00

ATOM 81 NH1 ARG 5 15.995 -7.765 0.490 1.00 0.00

ATOM 82 HH11 ARG 5 15.218 -7.354 0.988 1.00 0.00

ATOM 83 HH12 ARG 5 15.848 -8.551 -0.128 1.00 0.00

ATOM 84 NH2 ARG 5 18.202 -7.806 0.005 1.00 0.00

ATOM 85 HH21 ARG 5 19.130 -7.427 0.128 1.00 0.00

ATOM 86 HH22 ARG 5 18.048 -8.592 -0.611 1.00 0.00

ATOM 87 C ARG 5 18.015 -6.524 7.676 1.00 0.00

ATOM 88 O ARG 5 17.663 -5.550 8.311 1.00 0.00

ATOM 89 N GLU 6 17.925 -7.727 8.174 1.00 0.00

ATOM 90 H GLU 6 18.215 -8.542 7.651 1.00 0.00

ATOM 91 CA GLU 6 17.383 -7.925 9.548 1.00 0.00

ATOM 92 HA GLU 6 16.359 -7.557 9.607 1.00 0.00

ATOM 93 CB GLU 6 17.387 -9.417 9.889 1.00 0.00

ATOM 94 HB2 GLU 6 18.398 -9.812 9.784 1.00 0.00

ATOM 95 HB3 GLU 6 17.048 -9.557 10.916 1.00 0.00

ATOM 96 CG GLU 6 16.448 -10.160 8.937 1.00 0.00

ATOM 97 HG2 GLU 6 15.443 -9.747 9.018 1.00 0.00

ATOM 98 HG3 GLU 6 16.805 -10.047 7.913 1.00 0.00

ATOM 99 CD GLU 6 16.420 -11.644 9.305 1.00 0.00

ATOM 100 OE1 GLU 6 17.218 -12.045 10.136 1.00 0.00

ATOM 101 OE2 GLU 6 15.598 -12.356 8.750 1.00 0.00

ATOM 102 C GLU 6 18.255 -7.169 10.553 1.00 0.00

ATOM 103 O GLU 6 17.780 -6.692 11.565 1.00 0.00

ATOM 104 N ARG 7 19.526 -7.055 10.283 1.00 0.00

ATOM 105 H ARG 7 19.928 -7.450 9.444 1.00 0.00

ATOM 106 CA ARG 7 20.425 -6.329 11.223 1.00 0.00

ATOM 107 HA ARG 7 20.527 -6.886 12.154 1.00 0.00

ATOM 108 CB ARG 7 21.810 -6.176 10.591 1.00 0.00

ATOM 109 HB2 ARG 7 22.181 -7.155 10.287 1.00 0.00

ATOM 110 HB3 ARG 7 21.742 -5.527 9.718 1.00 0.00

ATOM 111 CG ARG 7 22.771 -5.561 11.610 1.00 0.00

ATOM 112 HG2 ARG 7 22.383 -4.598 11.941 1.00 0.00

ATOM 113 HG3 ARG 7 22.868 -6.228 12.467 1.00 0.00

ATOM 114 CD ARG 7 24.143 -5.362 10.962 1.00 0.00

ATOM 115 HD2 ARG 7 24.505 -6.303 10.547 1.00 0.00

ATOM 116 HD3 ARG 7 24.082 -4.616 10.170 1.00 0.00

ATOM 117 NE ARG 7 25.113 -4.889 11.990 1.00 0.00

ATOM 118 HE ARG 7 25.541 -5.528 12.646 1.00 0.00

ATOM 119 CZ ARG 7 25.427 -3.624 12.057 1.00 0.00

ATOM 120 NH1 ARG 7 24.836 -2.851 12.926 1.00 0.00

ATOM 121 HH11 ARG 7 24.137 -3.235 13.545 1.00 0.00

ATOM 122 HH12 ARG 7 25.081 -1.873 12.976 1.00 0.00

ATOM 123 NH2 ARG 7 26.332 -3.133 11.255 1.00 0.00

ATOM 124 HH21 ARG 7 26.788 -3.735 10.584 1.00 0.00

ATOM 125 HH22 ARG 7 26.573 -2.154 11.309 1.00 0.00

ATOM 126 C ARG 7 19.844 -4.945 11.518 1.00 0.00

ATOM 127 O ARG 7 19.865 -4.476 12.639 1.00 0.00

ATOM 128 N GLY 8 19.323 -4.286 10.519 1.00 0.00

ATOM 129 H GLY 8 19.301 -4.669 9.585 1.00 0.00

ATOM 130 CA GLY 8 18.741 -2.932 10.742 1.00 0.00

ATOM 131 HA2 GLY 8 19.517 -2.244 11.076 1.00 0.00

ATOM 132 HA3 GLY 8 18.299 -2.560 9.818 1.00 0.00

ATOM 133 C GLY 8 17.654 -3.016 11.815 1.00 0.00

ATOM 134 O GLY 8 17.440 -2.087 12.568 1.00 0.00

ATOM 135 N ARG 9 16.963 -4.121 11.890 1.00 0.00

ATOM 136 H ARG 9 17.133 -4.897 11.266 1.00 0.00

ATOM 137 CA ARG 9 15.891 -4.261 12.916 1.00 0.00

ATOM 138 HA ARG 9 15.091 -3.546 12.727 1.00 0.00

ATOM 139 CB ARG 9 15.309 -5.674 12.855 1.00 0.00

ATOM 140 HB2 ARG 9 14.946 -5.876 11.847 1.00 0.00

ATOM 141 HB3 ARG 9 16.082 -6.398 13.113 1.00 0.00

ATOM 142 CG ARG 9 14.150 -5.790 13.848 1.00 0.00

ATOM 143 HG2 ARG 9 14.505 -5.552 14.851 1.00 0.00

ATOM 144 HG3 ARG 9 13.360 -5.094 13.567 1.00 0.00

ATOM 145 CD ARG 9 13.602 -7.219 13.828 1.00 0.00

ATOM 146 HD2 ARG 9 13.319 -7.501 12.814 1.00 0.00

ATOM 147 HD3 ARG 9 14.352 -7.917 14.202 1.00 0.00

ATOM 148 NE ARG 9 12.397 -7.301 14.701 1.00 0.00

ATOM 149 HE ARG 9 11.544 -6.813 14.471 1.00 0.00

ATOM 150 CZ ARG 9 12.430 -8.017 15.791 1.00 0.00

ATOM 151 NH1 ARG 9 12.904 -9.232 15.761 1.00 0.00

ATOM 152 HH11 ARG 9 13.245 -9.617 14.891 1.00 0.00

ATOM 153 HH12 ARG 9 12.928 -9.783 16.607 1.00 0.00

ATOM 154 NH2 ARG 9 11.989 -7.516 16.913 1.00 0.00

ATOM 155 HH21 ARG 9 11.624 -6.575 16.933 1.00 0.00

ATOM 156 HH22 ARG 9 12.016 -8.072 17.756 1.00 0.00

ATOM 157 C ARG 9 16.481 -4.010 14.305 1.00 0.00

ATOM 158 O ARG 9 15.832 -3.465 15.176 1.00 0.00

ATOM 159 N GLN 10 17.707 -4.405 14.520 1.00 0.00

ATOM 160 H GLN 10 18.253 -4.859 13.801 1.00 0.00

ATOM 161 CA GLN 10 18.336 -4.189 15.853 1.00 0.00

ATOM 162 HA GLN 10 17.821 -4.775 16.615 1.00 0.00

ATOM 163 CB GLN 10 19.799 -4.633 15.802 1.00 0.00

ATOM 164 HB2 GLN 10 20.306 -4.123 14.983 1.00 0.00

ATOM 165 HB3 GLN 10 20.289 -4.383 16.743 1.00 0.00

ATOM 166 CG GLN 10 19.866 -6.146 15.580 1.00 0.00

ATOM 167 HG2 GLN 10 19.286 -6.666 16.343 1.00 0.00

ATOM 168 HG3 GLN 10 19.479 -6.402 14.593 1.00 0.00

ATOM 169 CD GLN 10 21.320 -6.611 15.670 1.00 0.00

ATOM 170 OE1 GLN 10 21.981 -6.863 14.573 1.00 0.00

ATOM 171 NE2 GLN 10 21.861 -6.747 16.749 1.00 0.00

ATOM 172 HE21 GLN 10 22.822 -7.055 16.793 1.00 0.00

ATOM 173 HE22 GLN 10 21.347 -6.552 17.596 1.00 0.00

ATOM 174 C GLN 10 18.266 -2.704 16.215 1.00 0.00

ATOM 175 O GLN 10 18.108 -2.343 17.364 1.00 0.00

ATOM 176 N GLY 11 18.383 -1.841 15.244 1.00 0.00

ATOM 177 H GLY 11 18.515 -2.135 14.287 1.00 0.00

ATOM 178 CA GLY 11 18.324 -0.380 15.533 1.00 0.00

ATOM 179 HA2 GLY 11 19.194 -0.081 16.118 1.00 0.00

ATOM 180 HA3 GLY 11 18.302 0.183 14.600 1.00 0.00

ATOM 181 C GLY 11 17.056 -0.070 16.332 1.00 0.00

ATOM 182 O GLY 11 17.049 0.785 17.194 1.00 0.00

ATOM 183 N ASP 12 15.984 -0.759 16.051 1.00 0.00

ATOM 184 H ASP 12 15.982 -1.471 15.334 1.00 0.00

ATOM 185 CA ASP 12 14.719 -0.502 16.796 1.00 0.00

ATOM 186 HA ASP 12 13.909 -1.105 16.384 1.00 0.00

ATOM 187 CB ASP 12 14.907 -0.876 18.267 1.00 0.00

ATOM 188 HB2 ASP 12 15.760 -0.333 18.675 1.00 0.00

ATOM 189 HB3 ASP 12 14.009 -0.615 18.826 1.00 0.00

ATOM 190 CG ASP 12 15.159 -2.381 18.382 1.00 0.00

ATOM 191 OD1 ASP 12 14.921 -3.077 17.409 1.00 0.00

ATOM 192 OD2 ASP 12 15.586 -2.811 19.441 1.00 0.00

ATOM 193 C ASP 12 14.355 0.980 16.689 1.00 0.00

ATOM 194 O ASP 12 14.037 1.623 17.669 1.00 0.00

ATOM 195 N SER 13 14.400 1.528 15.505 1.00 0.00

ATOM 196 H SER 13 14.664 1.000 14.685 1.00 0.00

ATOM 197 CA SER 13 14.056 2.968 15.338 1.00 0.00

ATOM 198 HA SER 13 14.769 3.592 15.877 1.00 0.00

ATOM 199 CB SER 13 14.109 3.336 13.854 1.00 0.00

ATOM	200	HB2	SER	13	13.347	2.775	13.312	1.00	0.00
ATOM	201	HB3	SER	13	13.926	4.404	13.737	1.00	0.00
ATOM	202	OG	SER	13	15.391	3.016	13.333	1.00	0.00
ATOM	203	HG	SER	13	15.425	3.246	12.402	1.00	0.00
ATOM	204	C	SER	13	12.646	3.219	15.876	1.00	0.00
ATOM	205	O	SER	13	12.373	4.240	16.476	1.00	0.00
ATOM	206	N	SER	14	11.749	2.295	15.666	1.00	0.00
ATOM	207	H	SER	14	11.971	1.443	15.169	1.00	0.00
ATOM	208	CA	SER	14	10.356	2.480	16.166	1.00	0.00
ATOM	209	HA	SER	14	10.359	3.062	17.087	1.00	0.00
ATOM	210	CB	SER	14	9.533	3.229	15.116	1.00	0.00
ATOM	211	HB2	SER	14	9.950	4.225	14.970	1.00	0.00
ATOM	212	HB3	SER	14	9.561	2.682	14.173	1.00	0.00
ATOM	213	OG	SER	14	8.188	3.339	15.560	1.00	0.00
ATOM	214	HG	SER	14	7.671	3.808	14.901	1.00	0.00
ATOM	215	C	SER	14	9.726	1.110	16.426	1.00	0.00
ATOM	216	O	SER	14	9.815	0.210	15.616	1.00	0.00
ATOM	217	N	SER	15	9.090	0.945	17.555	1.00	0.00
ATOM	218	H	SER	15	9.013	1.687	18.236	1.00	0.00
ATOM	219	CA	SER	15	8.457	-0.367	17.866	1.00	0.00

ATOM	220	HA	SER	15	9.141	-1.183	17.631	1.00	0.00
ATOM	221	CB	SER	15	8.117	-0.428	19.357	1.00	0.00
ATOM	222	HB2	SER	15	7.780	-1.433	19.613	1.00	0.00
ATOM	223	HB3	SER	15	9.003	-0.183	19.943	1.00	0.00
ATOM	224	OG	SER	15	7.086	0.505	19.645	1.00	0.00
ATOM	225	HG	SER	15	6.873	0.467	20.580	1.00	0.00
ATOM	226	C	SER	15	7.177	-0.528	17.043	1.00	0.00
ATOM	227	O	SER	15	6.757	-1.627	16.741	1.00	0.00
ATOM	228	N	CYX	16	6.554	0.560	16.676	1.00	0.00
ATOM	229	H	CYX	16	6.896	1.477	16.923	1.00	0.00
ATOM	230	CA	CYX	16	5.304	0.465	15.870	1.00	0.00
ATOM	231	HA	CYX	16	4.543	-0.091	16.418	1.00	0.00
ATOM	232	CB	CYX	16	4.772	1.870	15.583	1.00	0.00
ATOM	233	HB2	CYX	16	4.795	2.461	16.498	1.00	0.00
ATOM	234	HB3	CYX	16	5.395	2.347	14.827	1.00	0.00
ATOM	235	SG	CYX	16	3.049	1.764	15.036	1.00	0.00
ATOM	236	C	CYX	16	5.605	-0.246	14.549	1.00	0.00
ATOM	237	O	CYX	16	4.735	-0.832	13.936	1.00	0.00
ATOM	238	N	GLU	17	6.831	-0.199	14.106	1.00	0.00
ATOM	239	H	GLU	17	7.559	0.286	14.610	1.00	0.00

ATOM	240	CA	GLU	17	7.187	-0.872	12.826	1.00	0.00
ATOM	241	HA	GLU	17	6.587	-0.473	12.008	1.00	0.00
ATOM	242	CB	GLU	17	8.664	-0.624	12.515	1.00	0.00
ATOM	243	HB2	GLU	17	9.255	-0.757	13.422	1.00	0.00
ATOM	244	HB3	GLU	17	9.000	-1.331	11.756	1.00	0.00
ATOM	245	CG	GLU	17	8.842	0.805	11.995	1.00	0.00
ATOM	246	HG2	GLU	17	8.216	0.952	11.115	1.00	0.00
ATOM	247	HG3	GLU	17	8.551	1.513	12.770	1.00	0.00
ATOM	248	CD	GLU	17	10.308	1.032	11.621	1.00	0.00
ATOM	249	OE1	GLU	17	11.079	0.094	11.729	1.00	0.00
ATOM	250	OE2	GLU	17	10.634	2.142	11.233	1.00	0.00
ATOM	251	C	GLU	17	6.937	-2.376	12.955	1.00	0.00
ATOM	252	O	GLU	17	6.321	-2.987	12.104	1.00	0.00
ATOM	253	N	ARG	18	7.410	-2.978	14.012	1.00	0.00
ATOM	254	H	ARG	18	7.922	-2.477	14.724	1.00	0.00
ATOM	255	CA	ARG	18	7.199	-4.443	14.193	1.00	0.00
ATOM	256	HA	ARG	18	7.780	-5.002	13.459	1.00	0.00
ATOM	257	CB	ARG	18	7.655	-4.856	15.594	1.00	0.00
ATOM	258	HB2	ARG	18	8.710	-4.612	15.721	1.00	0.00
ATOM	259	HB3	ARG	18	7.067	-4.323	16.341	1.00	0.00

ATOM 260 CG ARG 18 7.457 -6.363 15.770 1.00 0.00

ATOM 261 HG2 ARG 18 6.409 -6.614 15.609 1.00 0.00

ATOM 262 HG3 ARG 18 8.073 -6.898 15.047 1.00 0.00

ATOM 263 CD ARG 18 7.866 -6.769 17.188 1.00 0.00

ATOM 264 HD2 ARG 18 7.937 -7.854 17.264 1.00 0.00

ATOM 265 HD3 ARG 18 8.827 -6.324 17.445 1.00 0.00

ATOM 266 NE ARG 18 6.842 -6.288 18.158 1.00 0.00

ATOM 267 HE ARG 18 6.084 -5.687 17.869 1.00 0.00

ATOM 268 CZ ARG 18 6.914 -6.641 19.413 1.00 0.00

ATOM 269 NH1 ARG 18 7.414 -5.819 20.295 1.00 0.00

ATOM 270 HH11 ARG 18 7.745 -4.911 20.003 1.00 0.00

ATOM 271 HH12 ARG 18 7.468 -6.096 21.265 1.00 0.00

ATOM 272 NH2 ARG 18 6.487 -7.817 19.784 1.00 0.00

ATOM 273 HH21 ARG 18 6.102 -8.451 19.098 1.00 0.00

ATOM 274 HH22 ARG 18 6.544 -8.089 20.755 1.00 0.00

ATOM 275 C ARG 18 5.713 -4.768 14.025 1.00 0.00

ATOM 276 O ARG 18 5.341 -5.894 13.758 1.00 0.00

ATOM 277 N GLN 19 4.860 -3.793 14.182 1.00 0.00

ATOM 278 H GLN 19 5.161 -2.856 14.406 1.00 0.00

ATOM 279 CA GLN 19 3.401 -4.050 14.034 1.00 0.00

ATOM	280	HA	GLN	19	3.129	-4.982	14.530	1.00	0.00
ATOM	281	CB	GLN	19	2.614	-2.906	14.676	1.00	0.00
ATOM	282	HB2	GLN	19	3.011	-1.952	14.330	1.00	0.00
ATOM	283	HB3	GLN	19	1.564	-2.986	14.397	1.00	0.00
ATOM	284	CG	GLN	19	2.745	-2.990	16.198	1.00	0.00
ATOM	285	HG2	GLN	19	2.486	-3.991	16.544	1.00	0.00
ATOM	286	HG3	GLN	19	3.764	-2.753	16.504	1.00	0.00
ATOM	287	CD	GLN	19	1.793	-1.986	16.850	1.00	0.00
ATOM	288	OE1	GLN	19	1.314	-1.076	16.203	1.00	0.00
ATOM	289	NE2	GLN	19	1.496	-2.112	18.115	1.00	0.00
ATOM	290	HE21	GLN	19	0.868	-1.453	18.551	1.00	0.00
ATOM	291	HE22	GLN	19	1.896	-2.869	18.651	1.00	0.00
ATOM	292	C	GLN	19	3.045	-4.145	12.548	1.00	0.00
ATOM	293	O	GLN	19	2.014	-4.673	12.181	1.00	0.00
ATOM	294	N	VAL	20	3.889	-3.641	11.689	1.00	0.00
ATOM	295	H	VAL	20	4.748	-3.199	11.984	1.00	0.00
ATOM	296	CA	VAL	20	3.593	-3.707	10.230	1.00	0.00
ATOM	297	HA	VAL	20	2.555	-3.434	10.043	1.00	0.00
ATOM	298	CB	VAL	20	4.500	-2.730	9.477	1.00	0.00
ATOM	299	HB	VAL	20	4.508	-1.772	9.995	1.00	0.00

ATOM 300 CG1 VAL 20 5.923 -3.289 9.417 1.00 0.00

ATOM 301 HG11 VAL 20 5.916 -4.248 8.898 1.00 0.00

ATOM 302 HG12 VAL 20 6.566 -2.592 8.880 1.00 0.00

ATOM 303 HG13 VAL 20 6.303 -3.427 10.429 1.00 0.00

ATOM 304 CG2 VAL 20 3.970 -2.536 8.055 1.00 0.00

ATOM 305 HG21 VAL 20 2.958 -2.135 8.096 1.00 0.00

ATOM 306 HG22 VAL 20 4.615 -1.841 7.518 1.00 0.00

ATOM 307 HG23 VAL 20 3.959 -3.495 7.537 1.00 0.00

ATOM 308 C VAL 20 3.842 -5.131 9.726 1.00 0.00

ATOM 309 O VAL 20 3.187 -5.600 8.815 1.00 0.00

ATOM 310 N ASP 21 4.782 -5.823 10.311 1.00 0.00

ATOM 311 H ASP 21 5.331 -5.439 11.067 1.00 0.00

ATOM 312 CA ASP 21 5.070 -7.215 9.866 1.00 0.00

ATOM 313 HA ASP 21 5.483 -7.212 8.857 1.00 0.00

ATOM 314 CB ASP 21 6.091 -7.851 10.809 1.00 0.00

ATOM 315 HB2 ASP 21 5.781 -7.694 11.842 1.00 0.00

ATOM 316 HB3 ASP 21 6.155 -8.921 10.608 1.00 0.00

ATOM 317 CG ASP 21 7.462 -7.208 10.585 1.00 0.00

ATOM 318 OD1 ASP 21 7.581 -6.424 9.657 1.00 0.00

ATOM 319 OD2 ASP 21 8.367 -7.510 11.344 1.00 0.00

ATOM 320 C ASP 21 3.778 -8.035 9.884 1.00 0.00

ATOM 321 O ASP 21 3.596 -8.942 9.097 1.00 0.00

ATOM 322 N ARG 22 2.880 -7.721 10.776 1.00 0.00

ATOM 323 H ARG 22 3.025 -6.969 11.434 1.00 0.00

ATOM 324 CA ARG 22 1.600 -8.482 10.843 1.00 0.00

ATOM 325 HA ARG 22 1.787 -9.549 10.715 1.00 0.00

ATOM 326 CB ARG 22 0.945 -8.260 12.209 1.00 0.00

ATOM 327 HB2 ARG 22 1.665 -8.479 12.998 1.00 0.00

ATOM 328 HB3 ARG 22 0.620 -7.224 12.292 1.00 0.00

ATOM 329 CG ARG 22 -0.263 -9.186 12.351 1.00 0.00

ATOM 330 HG2 ARG 22 -0.937 -9.037 11.508 1.00 0.00

ATOM 331 HG3 ARG 22 0.073 -10.223 12.367 1.00 0.00

ATOM 332 CD ARG 22 -0.999 -8.868 13.655 1.00 0.00

ATOM 333 HD2 ARG 22 -0.301 -8.869 14.492 1.00 0.00

ATOM 334 HD3 ARG 22 -1.483 -7.894 13.587 1.00 0.00

ATOM 335 NE ARG 22 -2.042 -9.904 13.902 1.00 0.00

ATOM 336 HE ARG 22 -2.167 -10.322 14.813 1.00 0.00

ATOM 337 CZ ARG 22 -2.819 -10.291 12.927 1.00 0.00

ATOM 338 NH1 ARG 22 -4.053 -9.868 12.869 1.00 0.00

ATOM 339 HH11 ARG 22 -4.403 -9.242 13.580 1.00 0.00

ATOM 340 HH12 ARG 22 -4.652 -10.170 12.113 1.00 0.00

ATOM 341 NH2 ARG 22 -2.364 -11.101 12.011 1.00 0.00

ATOM 342 HH21 ARG 22 -1.409 -11.428 12.059 1.00 0.00

ATOM 343 HH22 ARG 22 -2.968 -11.398 11.258 1.00 0.00

ATOM 344 C ARG 22 0.662 -7.995 9.740 1.00 0.00

ATOM 345 O ARG 22 -0.292 -8.658 9.384 1.00 0.00

ATOM 346 N VAL 23 0.924 -6.839 9.194 1.00 0.00

ATOM 347 H VAL 23 1.715 -6.283 9.486 1.00 0.00

ATOM 348 CA VAL 23 0.048 -6.308 8.112 1.00 0.00

ATOM 349 HA VAL 23 -0.874 -5.909 8.533 1.00 0.00

ATOM 350 CB VAL 23 0.776 -5.183 7.373 1.00 0.00

ATOM 351 HB VAL 23 1.783 -5.510 7.113 1.00 0.00

ATOM 352 CG1 VAL 23 0.009 -4.832 6.096 1.00 0.00

ATOM 353 HG11 VAL 23 -0.998 -4.505 6.355 1.00 0.00

ATOM 354 HG12 VAL 23 0.527 -4.031 5.569 1.00 0.00

ATOM 355 HG13 VAL 23 -0.049 -5.711 5.453 1.00 0.00

ATOM 356 CG2 VAL 23 0.858 -3.950 8.275 1.00 0.00

ATOM 357 HG21 VAL 23 1.405 -4.201 9.184 1.00 0.00

ATOM 358 HG22 VAL 23 1.377 -3.149 7.749 1.00 0.00

ATOM 359 HG23 VAL 23 -0.148 -3.622 8.535 1.00 0.00

ATOM 360 C VAL 23 -0.286 -7.431 7.129 1.00 0.00

ATOM 361 O VAL 23 -1.324 -8.056 7.214 1.00 0.00

ATOM 362 N ASN 24 0.587 -7.691 6.197 1.00 0.00

ATOM 363 H ASN 24 1.452 -7.176 6.122 1.00 0.00

ATOM 364 CA ASN 24 0.321 -8.772 5.208 1.00 0.00

ATOM 365 HA ASN 24 1.091 -8.778 4.437 1.00 0.00

ATOM 366 CB ASN 24 0.332 -10.126 5.920 1.00 0.00

ATOM 367 HB2 ASN 24 -0.437 -10.154 6.691 1.00 0.00

ATOM 368 HB3 ASN 24 0.159 -10.931 5.205 1.00 0.00

ATOM 369 CG ASN 24 1.695 -10.344 6.581 1.00 0.00

ATOM 370 OD1 ASN 24 2.663 -9.697 6.231 1.00 0.00

ATOM 371 ND2 ASN 24 1.813 -11.234 7.528 1.00 0.00

ATOM 372 HD21 ASN 24 2.710 -11.383 7.968 1.00 0.00

ATOM 373 HD22 ASN 24 1.007 -11.770 7.816 1.00 0.00

ATOM 374 C ASN 24 -1.048 -8.544 4.562 1.00 0.00

ATOM 375 O ASN 24 -2.010 -9.218 4.870 1.00 0.00

ATOM 376 N LEU 25 -1.142 -7.599 3.668 1.00 0.00

ATOM 377 H LEU 25 -0.345 -7.037 3.406 1.00 0.00

ATOM 378 CA LEU 25 -2.448 -7.328 3.003 1.00 0.00

ATOM 379 HA LEU 25 -3.243 -7.899 3.483 1.00 0.00

ATOM	380	CB	LEU	25	-2.778	-5.838	3.118	1.00	0.00
ATOM	381	HB2	LEU	25	-1.886	-5.251	2.900	1.00	0.00
ATOM	382	HB3	LEU	25	-3.563	-5.584	2.406	1.00	0.00
ATOM	383	CG	LEU	25	-3.258	-5.529	4.538	1.00	0.00
ATOM	384	HG	LEU	25	-2.618	-6.041	5.257	1.00	0.00
ATOM	385	CD1	LEU	25	-3.195	-4.021	4.782	1.00	0.00
ATOM	386	HD11	LEU	25	-3.835	-3.510	4.063	1.00	0.00
ATOM	387	HD12	LEU	25	-3.537	-3.802	5.793	1.00	0.00
ATOM	388	HD13	LEU	25	-2.168	-3.676	4.663	1.00	0.00
ATOM	389	CD2	LEU	25	-4.701	-6.013	4.705	1.00	0.00
ATOM	390	HD21	LEU	25	-4.746	-7.089	4.532	1.00	0.00
ATOM	391	HD22	LEU	25	-5.043	-5.794	5.716	1.00	0.00
ATOM	392	HD23	LEU	25	-5.341	-5.503	3.986	1.00	0.00
ATOM	393	C	LEU	25	-2.356	-7.717	1.526	1.00	0.00
ATOM	394	O	LEU	25	-2.384	-6.876	0.649	1.00	0.00
ATOM	395	N	LYS	26	-2.247	-8.987	1.244	1.00	0.00
ATOM	396	H	LYS	26	-2.219	-9.688	1.971	1.00	0.00
ATOM	397	CA	LYS	26	-2.151	-9.435	-0.174	1.00	0.00
ATOM	398	HA	LYS	26	-1.362	-8.890	-0.692	1.00	0.00
ATOM	399	CB	LYS	26	-1.819	-10.928	-0.212	1.00	0.00

ATOM 400 HB2 LYS 26 -0.962 -11.127 0.431 1.00 0.00

ATOM 401 HB3 LYS 26 -2.677 -11.501 0.139 1.00 0.00

ATOM 402 CG LYS 26 -1.485 -11.339 -1.648 1.00 0.00

ATOM 403 HG2 LYS 26 -2.052 -12.232 -1.912 1.00 0.00

ATOM 404 HG3 LYS 26 -1.746 -10.529 -2.328 1.00 0.00

ATOM 405 CD LYS 26 0.012 -11.635 -1.759 1.00 0.00

ATOM 406 HD2 LYS 26 0.407 -11.180 -2.667 1.00 0.00

ATOM 407 HD3 LYS 26 0.529 -11.224 -0.892 1.00 0.00

ATOM 408 CE LYS 26 0.231 -13.149 -1.812 1.00 0.00

ATOM 409 HE2 LYS 26 0.546 -13.506 -0.831 1.00 0.00

ATOM 410 HE3 LYS 26 -0.699 -13.642 -2.096 1.00 0.00

ATOM 411 NZ LYS 26 1.286 -13.464 -2.816 1.00 0.00

ATOM 412 HZ1 LYS 26 2.148 -13.009 -2.553 1.00 0.00

ATOM 413 HZ2 LYS 26 1.430 -14.463 -2.851 1.00 0.00

ATOM 414 HZ3 LYS 26 0.995 -13.135 -3.725 1.00 0.00

ATOM 415 C LYS 26 -3.478 -9.190 -0.904 1.00 0.00

ATOM 416 O LYS 26 -3.489 -8.836 -2.066 1.00 0.00

ATOM 417 N PRO 27 -4.623 -9.387 -0.222 1.00 0.00

ATOM 418 CD PRO 27 -4.732 -9.812 1.188 1.00 0.00

ATOM 419 HD2 PRO 27 -4.043 -9.230 1.800 1.00 0.00

ATOM 420 HD3 PRO 27 -4.484 -10.871 1.271 1.00 0.00

ATOM 421 CG PRO 27 -6.171 -9.533 1.515 1.00 0.00

ATOM 422 HG2 PRO 27 -6.264 -8.525 1.917 1.00 0.00

ATOM 423 HG3 PRO 27 -6.522 -10.253 2.255 1.00 0.00

ATOM 424 CB PRO 27 -6.918 -9.679 0.235 1.00 0.00

ATOM 425 HB2 PRO 27 -7.792 -9.030 0.265 1.00 0.00

ATOM 426 HB3 PRO 27 -7.231 -10.700 0.013 1.00 0.00

ATOM 427 CA PRO 27 -5.944 -9.189 -0.835 1.00 0.00

ATOM 428 HA PRO 27 -5.988 -9.777 -1.752 1.00 0.00

ATOM 429 C PRO 27 -6.205 -7.715 -1.161 1.00 0.00

ATOM 430 O PRO 27 -6.752 -7.388 -2.196 1.00 0.00

ATOM 431 N CYX 28 -5.809 -6.822 -0.296 1.00 0.00

ATOM 432 H CYX 28 -5.347 -7.080 0.565 1.00 0.00

ATOM 433 CA CYX 28 -6.030 -5.375 -0.574 1.00 0.00

ATOM 434 HA CYX 28 -7.084 -5.186 -0.774 1.00 0.00

ATOM 435 CB CYX 28 -5.607 -4.549 0.643 1.00 0.00

ATOM 436 HB2 CYX 28 -4.946 -5.144 1.274 1.00 0.00

ATOM 437 HB3 CYX 28 -5.082 -3.654 0.310 1.00 0.00

ATOM 438 SG CYX 28 -7.067 -4.145 1.634 1.00 0.00

ATOM 439 C CYX 28 -5.184 -4.977 -1.784 1.00 0.00

ATOM 440 O CYX 28 -5.688 -4.492 -2.782 1.00 0.00

ATOM 441 N GLU 29 -3.898 -5.189 -1.711 1.00 0.00

ATOM 442 H GLU 29 -3.471 -5.595 -0.890 1.00 0.00

ATOM 443 CA GLU 29 -3.023 -4.838 -2.859 1.00 0.00

ATOM 444 HA GLU 29 -2.971 -3.756 -2.976 1.00 0.00

ATOM 445 CB GLU 29 -1.612 -5.371 -2.611 1.00 0.00

ATOM 446 HB2 GLU 29 -1.373 -5.289 -1.551 1.00 0.00

ATOM 447 HB3 GLU 29 -1.559 -6.417 -2.915 1.00 0.00

ATOM 448 CG GLU 29 -0.608 -4.554 -3.424 1.00 0.00

ATOM 449 HG2 GLU 29 -0.882 -4.586 -4.479 1.00 0.00

ATOM 450 HG3 GLU 29 -0.617 -3.521 -3.078 1.00 0.00

ATOM 451 CD GLU 29 0.794 -5.139 -3.244 1.00 0.00

ATOM 452 OE1 GLU 29 0.925 -6.089 -2.488 1.00 0.00

ATOM 453 OE2 GLU 29 1.712 -4.630 -3.864 1.00 0.00

ATOM 454 C GLU 29 -3.592 -5.476 -4.126 1.00 0.00

ATOM 455 O GLU 29 -3.500 -4.930 -5.208 1.00 0.00

ATOM 456 N GLN 30 -4.176 -6.636 -4.001 1.00 0.00

ATOM 457 H GLN 30 -4.250 -7.099 -3.106 1.00 0.00

ATOM 458 CA GLN 30 -4.748 -7.314 -5.195 1.00 0.00

ATOM 459 HA GLN 30 -4.015 -7.341 -6.002 1.00 0.00

ATOM 460 CB GLN 30 -5.128 -8.751 -4.833 1.00 0.00

ATOM 461 HB2 GLN 30 -5.547 -8.775 -3.827 1.00 0.00

ATOM 462 HB3 GLN 30 -5.867 -9.122 -5.543 1.00 0.00

ATOM 463 CG GLN 30 -3.880 -9.637 -4.886 1.00 0.00

ATOM 464 HG2 GLN 30 -3.560 -9.777 -5.919 1.00 0.00

ATOM 465 HG3 GLN 30 -3.069 -9.186 -4.314 1.00 0.00

ATOM 466 CD GLN 30 -4.200 -11.005 -4.284 1.00 0.00

ATOM 467 OE1 GLN 30 -5.297 -11.235 -3.814 1.00 0.00

ATOM 468 NE2 GLN 30 -3.281 -11.932 -4.276 1.00 0.00

ATOM 469 HE21 GLN 30 -3.485 -12.839 -3.880 1.00 0.00

ATOM 470 HE22 GLN 30 -2.369 -11.739 -4.666 1.00 0.00

ATOM 471 C GLN 30 -5.992 -6.559 -5.670 1.00 0.00

ATOM 472 O GLN 30 -6.368 -6.636 -6.823 1.00 0.00

ATOM 473 N HID 31 -6.634 -5.825 -4.801 1.00 0.00

ATOM 474 H HID 31 -6.333 -5.751 -3.840 1.00 0.00

ATOM 475 CA HID 31 -7.844 -5.069 -5.222 1.00 0.00

ATOM 476 HA HID 31 -8.457 -5.677 -5.887 1.00 0.00

ATOM 477 CB HID 31 -8.674 -4.706 -3.986 1.00 0.00

ATOM 478 HB2 HID 31 -9.347 -5.514 -3.699 1.00 0.00

ATOM 479 HB3 HID 31 -8.049 -4.436 -3.135 1.00 0.00

ATOM 480 CG HID 31 -9.538 -3.509 -4.283 1.00 0.00

ATOM 481 ND1 HID 31 -10.609 -3.569 -5.161 1.00 0.00

ATOM 482 HD1 HID 31 -10.916 -4.381 -5.678 1.00 0.00

ATOM 483 CE1 HID 31 -11.169 -2.346 -5.202 1.00 0.00

ATOM 484 HE1 HID 31 -12.034 -2.216 -5.853 1.00 0.00

ATOM 485 NE2 HID 31 -10.534 -1.483 -4.404 1.00 0.00

ATOM 486 CD2 HID 31 -9.503 -2.215 -3.822 1.00 0.00

ATOM 487 HD2 HID 31 -8.824 -1.735 -3.117 1.00 0.00

ATOM 488 C HID 31 -7.391 -3.801 -5.937 1.00 0.00

ATOM 489 O HID 31 -8.124 -3.203 -6.700 1.00 0.00

ATOM 490 N ILE 32 -6.177 -3.393 -5.696 1.00 0.00

ATOM 491 H ILE 32 -5.567 -3.887 -5.060 1.00 0.00

ATOM 492 CA ILE 32 -5.658 -2.167 -6.362 1.00 0.00

ATOM 493 HA ILE 32 -6.494 -1.512 -6.606 1.00 0.00

ATOM 494 CB ILE 32 -4.688 -1.433 -5.430 1.00 0.00

ATOM 495 HB ILE 32 -3.725 -1.311 -5.926 1.00 0.00

ATOM 496 CG2 ILE 32 -5.261 -0.061 -5.088 1.00 0.00

ATOM 497 HG21 ILE 32 -6.223 -0.183 -4.591 1.00 0.00

ATOM 498 HG22 ILE 32 -4.574 0.465 -4.425 1.00 0.00

ATOM 499 HG23 ILE 32 -5.395 0.515 -6.003 1.00 0.00

ATOM 500 CG1 ILE 32 -4.493 -2.236 -4.140 1.00 0.00

ATOM 501 HG12 ILE 32 -5.466 -2.479 -3.714 1.00 0.00

ATOM 502 HG13 ILE 32 -3.955 -3.158 -4.365 1.00 0.00

ATOM 503 CD1 ILE 32 -3.689 -1.409 -3.135 1.00 0.00

ATOM 504 HD11 ILE 32 -4.226 -0.488 -2.909 1.00 0.00

ATOM 505 HD12 ILE 32 -3.552 -1.983 -2.219 1.00 0.00

ATOM 506 HD13 ILE 32 -2.715 -1.167 -3.560 1.00 0.00

ATOM 507 C ILE 32 -4.930 -2.561 -7.646 1.00 0.00

ATOM 508 O ILE 32 -5.203 -2.040 -8.709 1.00 0.00

ATOM 509 N MET 33 -4.011 -3.482 -7.560 1.00 0.00

ATOM 510 H MET 33 -3.778 -3.922 -6.681 1.00 0.00

ATOM 511 CA MET 33 -3.274 -3.911 -8.781 1.00 0.00

ATOM 512 HA MET 33 -2.762 -3.061 -9.232 1.00 0.00

ATOM 513 CB MET 33 -2.231 -4.965 -8.403 1.00 0.00

ATOM 514 HB2 MET 33 -2.735 -5.864 -8.047 1.00 0.00

ATOM 515 HB3 MET 33 -1.628 -5.210 -9.277 1.00 0.00

ATOM 516 CG MET 33 -1.327 -4.416 -7.298 1.00 0.00

ATOM 517 HG2 MET 33 -0.927 -3.447 -7.596 1.00 0.00

ATOM 518 HG3 MET 33 -1.898 -4.305 -6.376 1.00 0.00

ATOM 519 SD MET 33 0.044 -5.563 -7.014 1.00 0.00

ATOM 520 CE MET 33 1.175 -4.871 -8.245 1.00 0.00

ATOM 521 HE1 MET 33 1.384 -3.829 -8.003 1.00 0.00

ATOM 522 HE2 MET 33 2.107 -5.438 -8.242 1.00 0.00

ATOM 523 HE3 MET 33 0.718 -4.930 -9.233 1.00 0.00

ATOM 524 C MET 33 -4.264 -4.506 -9.782 1.00 0.00

ATOM 525 O MET 33 -4.170 -4.282 -10.971 1.00 0.00

ATOM 526 N GLN 34 -5.214 -5.262 -9.307 1.00 0.00

ATOM 527 H GLN 34 -5.297 -5.450 -8.318 1.00 0.00

ATOM 528 CA GLN 34 -6.212 -5.875 -10.229 1.00 0.00

ATOM 529 HA GLN 34 -5.708 -6.473 -10.989 1.00 0.00

ATOM 530 CB GLN 34 -7.149 -6.785 -9.432 1.00 0.00

ATOM 531 HB2 GLN 34 -7.501 -6.258 -8.546 1.00 0.00

ATOM 532 HB3 GLN 34 -8.001 -7.062 -10.052 1.00 0.00

ATOM 533 CG GLN 34 -6.396 -8.047 -9.008 1.00 0.00

ATOM 534 HG2 GLN 34 -6.115 -8.633 -9.883 1.00 0.00

ATOM 535 HG3 GLN 34 -5.500 -7.782 -8.446 1.00 0.00

ATOM 536 CD GLN 34 -7.297 -8.903 -8.116 1.00 0.00

ATOM 537 OE1 GLN 34 -8.352 -8.467 -7.701 1.00 0.00

ATOM 538 NE2 GLN 34 -6.923 -10.113 -7.802 1.00 0.00

ATOM 539 HE21 GLN 34 -7.513 -10.684 -7.214 1.00 0.00

```
ATOM    540 HE22 GLN    34      -6.045 -10.474  -8.149  1.00  0.00
ATOM    541  C   GLN    34      -7.029  -4.776 -10.911  1.00  0.00
ATOM    542  O   GLN    34      -7.264  -4.817 -12.102  1.00  0.00
ATOM    543  N   ARG    35      -7.472  -3.798 -10.169  1.00  0.00
ATOM    544  H   ARG    35      -7.284  -3.756  -9.178  1.00  0.00
ATOM    545  CA  ARG    35      -8.281  -2.706 -10.783  1.00  0.00
ATOM    546  HA  ARG    35      -9.143  -3.121 -11.304  1.00  0.00
ATOM    547  CB  ARG    35      -8.779  -1.765  -9.686  1.00  0.00
ATOM    548  HB2 ARG    35      -7.998  -1.633  -8.937  1.00  0.00
ATOM    549  HB3 ARG    35      -9.030  -0.799 -10.122  1.00  0.00
ATOM    550  CG  ARG    35     -10.023  -2.366  -9.025  1.00  0.00
ATOM    551  HG2 ARG    35     -10.727  -2.684  -9.795  1.00  0.00
ATOM    552  HG3 ARG    35      -9.734  -3.226  -8.421  1.00  0.00
ATOM    553  CD  ARG    35     -10.684  -1.314  -8.132  1.00  0.00
ATOM    554  HD2 ARG    35     -11.094  -0.508  -8.739  1.00  0.00
ATOM    555  HD3 ARG    35     -11.481  -1.767  -7.543  1.00  0.00
ATOM    556  NE  ARG    35      -9.669  -0.743  -7.203  1.00  0.00
ATOM    557  HE  ARG    35      -8.821  -1.243  -6.976  1.00  0.00
ATOM    558  CZ  ARG    35      -9.866   0.427  -6.660  1.00  0.00
ATOM    559  NH1 ARG    35     -10.381   1.396  -7.367  1.00  0.00
```

ATOM 560 HH11 ARG 35 -10.626 1.236 -8.334 1.00 0.00

ATOM 561 HH12 ARG 35 -10.532 2.300 -6.944 1.00 0.00

ATOM 562 NH2 ARG 35 -9.546 0.629 -5.412 1.00 0.00

ATOM 563 HH21 ARG 35 -9.146 -0.123 -4.869 1.00 0.00

ATOM 564 HH22 ARG 35 -9.700 1.535 -4.995 1.00 0.00

ATOM 565 C ARG 35 -7.423 -1.919 -11.777 1.00 0.00

ATOM 566 O ARG 35 -7.781 -1.757 -12.926 1.00 0.00

ATOM 567 N ILE 36 -6.298 -1.424 -11.343 1.00 0.00

ATOM 568 H ILE 36 -5.995 -1.552 -10.388 1.00 0.00

ATOM 569 CA ILE 36 -5.424 -0.642 -12.263 1.00 0.00

ATOM 570 HA ILE 36 -5.998 0.164 -12.719 1.00 0.00

ATOM 571 CB ILE 36 -4.256 -0.051 -11.471 1.00 0.00

ATOM 572 HB ILE 36 -3.622 0.533 -12.138 1.00 0.00

ATOM 573 CG2 ILE 36 -4.800 0.853 -10.362 1.00 0.00

ATOM 574 HG21 ILE 36 -5.433 0.269 -9.695 1.00 0.00

ATOM 575 HG22 ILE 36 -3.969 1.275 -9.796 1.00 0.00

ATOM 576 HG23 ILE 36 -5.385 1.659 -10.804 1.00 0.00

ATOM 577 CG1 ILE 36 -3.433 -1.185 -10.854 1.00 0.00

ATOM 578 HG12 ILE 36 -4.105 -1.943 -10.452 1.00 0.00

ATOM 579 HG13 ILE 36 -2.799 -1.632 -11.621 1.00 0.00

ATOM 580 CD1 ILE 36 -2.557 -0.631 -9.728 1.00 0.00

ATOM 581 HD11 ILE 36 -3.189 -0.185 -8.961 1.00 0.00

ATOM 582 HD12 ILE 36 -1.972 -1.440 -9.290 1.00 0.00

ATOM 583 HD13 ILE 36 -1.884 0.126 -10.130 1.00 0.00

ATOM 584 C ILE 36 -4.883 -1.552 -13.368 1.00 0.00

ATOM 585 O ILE 36 -4.368 -1.090 -14.366 1.00 0.00

ATOM 586 N MET 37 -4.995 -2.842 -13.202 1.00 0.00

ATOM 587 H MET 37 -5.419 -3.240 -12.376 1.00 0.00

ATOM 588 CA MET 37 -4.483 -3.771 -14.251 1.00 0.00

ATOM 589 HA MET 37 -3.832 -3.237 -14.943 1.00 0.00

ATOM 590 CB MET 37 -3.680 -4.895 -13.594 1.00 0.00

ATOM 591 HB2 MET 37 -4.247 -5.309 -12.760 1.00 0.00

ATOM 592 HB3 MET 37 -3.485 -5.680 -14.326 1.00 0.00

ATOM 593 CG MET 37 -2.351 -4.337 -13.079 1.00 0.00

ATOM 594 HG2 MET 37 -1.935 -3.643 -13.809 1.00 0.00

ATOM 595 HG3 MET 37 -2.510 -3.818 -12.134 1.00 0.00

ATOM 596 SD MET 37 -1.186 -5.697 -12.820 1.00 0.00

ATOM 597 CE MET 37 -2.181 -6.626 -11.628 1.00 0.00

ATOM 598 HE1 MET 37 -3.128 -6.909 -12.087 1.00 0.00

ATOM 599 HE2 MET 37 -1.642 -7.525 -11.326 1.00 0.00

ATOM 600 HE3 MET 37 -2.372 -6.006 -10.752 1.00 0.00

ATOM 601 C MET 37 -5.661 -4.367 -15.023 1.00 0.00

ATOM 602 O MET 37 -5.510 -4.847 -16.130 1.00 0.00

ATOM 603 N GLY 38 -6.834 -4.342 -14.452 1.00 0.00

ATOM 604 H GLY 38 -6.968 -3.946 -13.533 1.00 0.00

ATOM 605 CA GLY 38 -8.018 -4.908 -15.157 1.00 0.00

ATOM 606 HA2 GLY 38 -7.809 -5.929 -15.479 1.00 0.00

ATOM 607 HA3 GLY 38 -8.883 -4.907 -14.495 1.00 0.00

ATOM 608 C GLY 38 -8.330 -4.052 -16.387 1.00 0.00

ATOM 609 O GLY 38 -8.909 -4.518 -17.349 1.00 0.00

ATOM 610 N GLU 39 -7.951 -2.804 -16.362 1.00 0.00

ATOM 611 H GLU 39 -7.471 -2.411 -15.565 1.00 0.00

ATOM 612 CA GLU 39 -8.225 -1.919 -17.529 1.00 0.00

ATOM 613 HA GLU 39 -9.293 -1.901 -17.747 1.00 0.00

ATOM 614 CB GLU 39 -7.766 -0.495 -17.209 1.00 0.00

ATOM 615 HB2 GLU 39 -8.227 -0.165 -16.278 1.00 0.00

ATOM 616 HB3 GLU 39 -6.681 -0.478 -17.103 1.00 0.00

ATOM 617 CG GLU 39 -8.182 0.442 -18.344 1.00 0.00

ATOM 618 HG2 GLU 39 -7.805 0.055 -19.291 1.00 0.00

ATOM 619 HG3 GLU 39 -9.269 0.504 -18.384 1.00 0.00

ATOM 620 CD GLU 39 -7.601 1.835 -18.095 1.00 0.00

ATOM 621 OE1 GLU 39 -6.890 1.992 -17.115 1.00 0.00

ATOM 622 OE2 GLU 39 -7.876 2.721 -18.887 1.00 0.00

ATOM 623 C GLU 39 -7.467 -2.438 -18.752 1.00 0.00

ATOM 624 O GLU 39 -7.851 -2.199 -19.880 1.00 0.00

ATOM 625 N GLN 40 -6.391 -3.145 -18.539 1.00 0.00

ATOM 626 H GLN 40 -6.063 -3.347 -17.605 1.00 0.00

ATOM 627 CA GLN 40 -5.611 -3.678 -19.691 1.00 0.00

ATOM 628 HA GLN 40 -5.337 -2.870 -20.369 1.00 0.00

ATOM 629 CB GLN 40 -4.331 -4.341 -19.178 1.00 0.00

ATOM 630 HB2 GLN 40 -4.580 -5.051 -18.389 1.00 0.00

ATOM 631 HB3 GLN 40 -3.840 -4.868 -19.997 1.00 0.00

ATOM 632 CG GLN 40 -3.389 -3.271 -18.623 1.00 0.00

ATOM 633 HG2 GLN 40 -3.132 -2.552 -19.401 1.00 0.00

ATOM 634 HG3 GLN 40 -3.859 -2.751 -17.789 1.00 0.00

ATOM 635 CD GLN 40 -2.103 -3.932 -18.124 1.00 0.00

ATOM 636 OE1 GLN 40 -2.041 -5.138 -17.985 1.00 0.00

ATOM 637 NE2 GLN 40 -1.065 -3.190 -17.848 1.00 0.00

ATOM 638 HE21 GLN 40 -0.213 -3.622 -17.518 1.00 0.00

ATOM 639 HE22 GLN 40 -1.118 -2.189 -17.966 1.00 0.00

ATOM 640 C GLN 40 -6.455 -4.709 -20.442 1.00 0.00

ATOM 641 O GLN 40 -6.352 -4.854 -21.643 1.00 0.00

ATOM 642 N GLU 41 -7.290 -5.427 -19.741 1.00 0.00

ATOM 643 H GLU 41 -7.379 -5.315 -18.741 1.00 0.00

ATOM 644 CA GLU 41 -8.142 -6.447 -20.416 1.00 0.00

ATOM 645 HA GLU 41 -7.520 -7.218 -20.872 1.00 0.00

ATOM 646 CB GLU 41 -9.061 -7.106 -19.385 1.00 0.00

ATOM 647 HB2 GLU 41 -9.672 -6.344 -18.903 1.00 0.00

ATOM 648 HB3 GLU 41 -9.708 -7.828 -19.884 1.00 0.00

ATOM 649 CG GLU 41 -8.215 -7.823 -18.331 1.00 0.00

ATOM 650 HG2 GLU 41 -7.581 -8.566 -18.816 1.00 0.00

ATOM 651 HG3 GLU 41 -7.590 -7.097 -17.811 1.00 0.00

ATOM 652 CD GLU 41 -9.135 -8.517 -17.325 1.00 0.00

ATOM 653 OE1 GLU 41 -10.330 -8.284 -17.387 1.00 0.00

ATOM 654 OE2 GLU 41 -8.628 -9.270 -16.511 1.00 0.00

ATOM 655 C GLU 41 -8.987 -5.770 -21.497 1.00 0.00

ATOM 656 O GLU 41 -9.490 -4.681 -21.311 1.00 0.00

ATOM 657 N GLN 42 -9.147 -6.408 -22.624 1.00 0.00

ATOM 658 H GLN 42 -8.730 -7.314 -22.787 1.00 0.00

ATOM 659 CA GLN 42 -9.960 -5.799 -23.713 1.00 0.00

ATOM 660 HA GLN 42 -9.560 -4.822 -23.983 1.00 0.00

ATOM 661 CB GLN 42 -9.917 -6.703 -24.947 1.00 0.00

ATOM 662 HB2 GLN 42 -10.262 -7.702 -24.679 1.00 0.00

ATOM 663 HB3 GLN 42 -10.563 -6.292 -25.722 1.00 0.00

ATOM 664 CG GLN 42 -8.482 -6.783 -25.470 1.00 0.00

ATOM 665 HG2 GLN 42 -7.828 -7.225 -24.718 1.00 0.00

ATOM 666 HG3 GLN 42 -8.444 -7.382 -26.380 1.00 0.00

ATOM 667 CD GLN 42 -7.977 -5.374 -25.790 1.00 0.00

ATOM 668 OE1 GLN 42 -8.628 -4.627 -26.492 1.00 0.00

ATOM 669 NE2 GLN 42 -6.835 -4.977 -25.300 1.00 0.00

ATOM 670 HE21 GLN 42 -6.495 -4.049 -25.507 1.00 0.00

ATOM 671 HE22 GLN 42 -6.295 -5.600 -24.716 1.00 0.00

ATOM 672 C GLN 42 -11.407 -5.642 -23.243 1.00 0.00

ATOM 673 O GLN 42 -12.078 -4.686 -23.575 1.00 0.00

ATOM 674 N TYR 43 -11.894 -6.575 -22.470 1.00 0.00

ATOM 675 H TYR 43 -11.342 -7.373 -22.188 1.00 0.00

ATOM 676 CA TYR 43 -13.298 -6.478 -21.979 1.00 0.00

ATOM 677 HA TYR 43 -13.782 -5.593 -22.392 1.00 0.00

ATOM 678 CB TYR 43 -14.082 -7.716 -22.422 1.00 0.00

ATOM 679 HB2 TYR 43 -13.620 -8.608 -21.998 1.00 0.00

```
ATOM    680  HB3 TYR    43     -15.112  -7.641 -22.074  1.00  0.00
ATOM    681  CG  TYR    43     -14.066  -7.809 -23.929  1.00  0.00
ATOM    682  CD1 TYR    43     -14.941  -7.022 -24.688  1.00  0.00
ATOM    683  HD1 TYR    43     -15.632  -6.342 -24.192  1.00  0.00
ATOM    684  CE1 TYR    43     -14.927  -7.110 -26.085  1.00  0.00
ATOM    685  HE1 TYR    43     -15.608  -6.498 -26.676  1.00  0.00
ATOM    686  CZ  TYR    43     -14.040  -7.985 -26.723  1.00  0.00
ATOM    687  OH  TYR    43     -14.027  -8.071 -28.101  1.00  0.00
ATOM    688  HH  TYR    43     -14.665  -7.489 -28.519  1.00  0.00
ATOM    689  CE2 TYR    43     -13.165  -8.771 -25.964  1.00  0.00
ATOM    690  HE2 TYR    43     -12.473  -9.452 -26.460  1.00  0.00
ATOM    691  CD2 TYR    43     -13.179  -8.683 -24.568  1.00  0.00
ATOM    692  HD2 TYR    43     -12.498  -9.296 -23.977  1.00  0.00
ATOM    693  C   TYR    43     -13.298  -6.397 -20.451  1.00  0.00
ATOM    694  O   TYR    43     -12.351  -6.789 -19.799  1.00  0.00
ATOM    695  N   ASP    44     -14.354  -5.891 -19.875  1.00  0.00
ATOM    696  H   ASP    44     -15.145  -5.564 -20.410  1.00  0.00
ATOM    697  CA  ASP    44     -14.413  -5.785 -18.390  1.00  0.00
ATOM    698  HA  ASP    44     -14.419  -6.778 -17.941  1.00  0.00
ATOM    699  CB  ASP    44     -13.185  -5.026 -17.881  1.00  0.00
```

ATOM 700 HB2 ASP 44 -13.310 -4.802 -16.822 1.00 0.00

ATOM 701 HB3 ASP 44 -12.294 -5.639 -18.019 1.00 0.00

ATOM 702 CG ASP 44 -13.032 -3.720 -18.664 1.00 0.00

ATOM 703 OD1 ASP 44 -13.748 -3.547 -19.636 1.00 0.00

ATOM 704 OD2 ASP 44 -12.199 -2.917 -18.277 1.00 0.00

ATOM 705 C ASP 44 -15.682 -5.033 -17.983 1.00 0.00

ATOM 706 O ASP 44 -16.389 -5.430 -17.079 1.00 0.00

ATOM 707 N SER 45 -15.977 -3.947 -18.645 1.00 0.00

ATOM 708 H SER 45 -15.394 -3.609 -19.397 1.00 0.00

ATOM 709 CA SER 45 -17.201 -3.172 -18.297 1.00 0.00

ATOM 710 HA SER 45 -17.079 -2.686 -17.329 1.00 0.00

ATOM 711 CB SER 45 -17.440 -2.096 -19.358 1.00 0.00

ATOM 712 HB2 SER 45 -18.246 -1.438 -19.034 1.00 0.00

ATOM 713 HB3 SER 45 -16.529 -1.514 -19.496 1.00 0.00

ATOM 714 OG SER 45 -17.798 -2.713 -20.586 1.00 0.00

ATOM 715 HG SER 45 -17.948 -2.038 -21.252 1.00 0.00

ATOM 716 C SER 45 -18.404 -4.114 -18.245 1.00 0.00

ATOM 717 O SER 45 -19.302 -3.947 -17.444 1.00 0.00

ATOM 718 N TYR 46 -18.431 -5.104 -19.096 1.00 0.00

ATOM 719 H TYR 46 -17.687 -5.250 -19.764 1.00 0.00

ATOM 720 CA TYR 46 -19.577 -6.055 -19.097 1.00 0.00

ATOM 721 HA TYR 46 -20.510 -5.523 -19.283 1.00 0.00

ATOM 722 CB TYR 46 -19.377 -7.093 -20.203 1.00 0.00

ATOM 723 HB2 TYR 46 -18.411 -7.583 -20.075 1.00 0.00

ATOM 724 HB3 TYR 46 -20.171 -7.838 -20.150 1.00 0.00

ATOM 725 CG TYR 46 -19.419 -6.408 -21.548 1.00 0.00

ATOM 726 CD1 TYR 46 -20.611 -5.834 -22.004 1.00 0.00

ATOM 727 HD1 TYR 46 -21.509 -5.883 -21.388 1.00 0.00

ATOM 728 CE1 TYR 46 -20.651 -5.198 -23.251 1.00 0.00

ATOM 729 HE1 TYR 46 -21.579 -4.752 -23.606 1.00 0.00

ATOM 730 CZ TYR 46 -19.497 -5.135 -24.041 1.00 0.00

ATOM 731 OH TYR 46 -19.536 -4.509 -25.270 1.00 0.00

ATOM 732 HH TYR 46 -20.402 -4.154 -25.483 1.00 0.00

ATOM 733 CE2 TYR 46 -18.304 -5.708 -23.585 1.00 0.00

ATOM 734 HE2 TYR 46 -17.406 -5.659 -24.201 1.00 0.00

ATOM 735 CD2 TYR 46 -18.264 -6.345 -22.338 1.00 0.00

ATOM 736 HD2 TYR 46 -17.335 -6.791 -21.983 1.00 0.00

ATOM 737 C TYR 46 -19.654 -6.763 -17.742 1.00 0.00

ATOM 738 O TYR 46 -20.719 -6.942 -17.185 1.00 0.00

ATOM 739 N ASP 47 -18.534 -7.168 -17.208 1.00 0.00

ATOM 740 H ASP 47 -17.644 -7.023 -17.664 1.00 0.00

ATOM 741 CA ASP 47 -18.547 -7.864 -15.891 1.00 0.00

ATOM 742 HA ASP 47 -19.558 -8.188 -15.644 1.00 0.00

ATOM 743 CB ASP 47 -17.642 -9.096 -15.956 1.00 0.00

ATOM 744 HB2 ASP 47 -16.648 -8.804 -16.296 1.00 0.00

ATOM 745 HB3 ASP 47 -17.569 -9.545 -14.965 1.00 0.00

ATOM 746 CG ASP 47 -18.236 -10.113 -16.933 1.00 0.00

ATOM 747 OD1 ASP 47 -19.396 -9.964 -17.280 1.00 0.00

ATOM 748 OD2 ASP 47 -17.520 -11.022 -17.320 1.00 0.00

ATOM 749 C ASP 47 -18.035 -6.914 -14.806 1.00 0.00

ATOM 750 O ASP 47 -16.912 -6.450 -14.853 1.00 0.00

ATOM 751 N ILE 48 -18.848 -6.619 -13.829 1.00 0.00

ATOM 752 H ILE 48 -19.782 -7.001 -13.782 1.00 0.00

ATOM 753 CA ILE 48 -18.406 -5.698 -12.744 1.00 0.00

ATOM 754 HA ILE 48 -18.158 -4.726 -13.170 1.00 0.00

ATOM 755 CB ILE 48 -19.534 -5.535 -11.723 1.00 0.00

ATOM 756 HB ILE 48 -19.770 -6.505 -11.284 1.00 0.00

ATOM 757 CG2 ILE 48 -19.089 -4.572 -10.621 1.00 0.00

ATOM 758 HG21 ILE 48 -18.852 -3.603 -11.059 1.00 0.00

ATOM 759 HG22 ILE 48 -19.892 -4.456 -9.893 1.00 0.00

```
ATOM    760 HG23 ILE    48      -18.204  -4.972 -10.124  1.00  0.00
ATOM    761  CG1 ILE    48      -20.776  -4.974 -12.419  1.00  0.00
ATOM    762 HG12 ILE    48      -20.576  -3.957 -12.756  1.00  0.00
ATOM    763 HG13 ILE    48      -21.025  -5.598 -13.278  1.00  0.00
ATOM    764  CD1 ILE    48      -21.950  -4.965 -11.438  1.00  0.00
ATOM    765 HD11 ILE    48      -21.702  -4.341 -10.579  1.00  0.00
ATOM    766 HD12 ILE    48      -22.835  -4.566 -11.933  1.00  0.00
ATOM    767 HD13 ILE    48      -22.150  -5.983 -11.101  1.00  0.00
ATOM    768  C   ILE    48      -17.173  -6.282 -12.053  1.00  0.00
ATOM    769  O   ILE    48      -16.218  -5.586 -11.773  1.00  0.00
ATOM    770  N   ARG    49      -17.186  -7.558 -11.774  1.00  0.00
ATOM    771  H   ARG    49      -17.976  -8.144 -12.004  1.00  0.00
ATOM    772  CA  ARG    49      -16.015  -8.185 -11.101  1.00  0.00
ATOM    773  HA  ARG    49      -15.155  -7.516 -11.136  1.00  0.00
ATOM    774  CB  ARG    49      -16.357  -8.465  -9.636  1.00  0.00
ATOM    775  HB2 ARG    49      -17.242  -9.100  -9.583  1.00  0.00
ATOM    776  HB3 ARG    49      -15.519  -8.971  -9.157  1.00  0.00
ATOM    777  CG  ARG    49      -16.634  -7.143  -8.916  1.00  0.00
ATOM    778  HG2 ARG    49      -17.392  -6.583  -9.463  1.00  0.00
ATOM    779  HG3 ARG    49      -16.990  -7.347  -7.906  1.00  0.00
```

ATOM 780 CD ARG 49 -15.345 -6.321 -8.846 1.00 0.00

ATOM 781 HD2 ARG 49 -14.548 -6.907 -8.386 1.00 0.00

ATOM 782 HD3 ARG 49 -15.038 -6.014 -9.846 1.00 0.00

ATOM 783 NE ARG 49 -15.578 -5.102 -8.022 1.00 0.00

ATOM 784 HE ARG 49 -16.489 -4.888 -7.643 1.00 0.00

ATOM 785 CZ ARG 49 -14.593 -4.283 -7.775 1.00 0.00

ATOM 786 NH1 ARG 49 -13.363 -4.654 -8.003 1.00 0.00

ATOM 787 HH11 ARG 49 -13.177 -5.576 -8.371 1.00 0.00

ATOM 788 HH12 ARG 49 -12.603 -4.018 -7.811 1.00 0.00

ATOM 789 NH2 ARG 49 -14.838 -3.092 -7.300 1.00 0.00

ATOM 790 HH21 ARG 49 -15.791 -2.808 -7.125 1.00 0.00

ATOM 791 HH22 ARG 49 -14.073 -2.461 -7.110 1.00 0.00

ATOM 792 C ARG 49 -15.667 -9.500 -11.803 1.00 0.00

ATOM 793 O ARG 49 -16.534 -10.218 -12.260 1.00 0.00

ATOM 794 N SER 50 -14.405 -9.819 -11.894 1.00 0.00

ATOM 795 H SER 50 -13.679 -9.227 -11.518 1.00 0.00

ATOM 796 CA SER 50 -14.004 -11.086 -12.567 1.00 0.00

ATOM 797 HA SER 50 -14.255 -11.048 -13.627 1.00 0.00

ATOM 798 CB SER 50 -12.492 -11.279 -12.429 1.00 0.00

ATOM 799 HB2 SER 50 -12.235 -11.399 -11.377 1.00 0.00

ATOM 800 HB3 SER 50 -12.186 -12.169 -12.981 1.00 0.00

ATOM 801 OG SER 50 -11.821 -10.144 -12.953 1.00 0.00

ATOM 802 HG SER 50 -10.872 -10.265 -12.867 1.00 0.00

ATOM 803 C SER 50 -14.731 -12.264 -11.914 1.00 0.00

ATOM 804 O SER 50 -15.173 -13.179 -12.581 1.00 0.00

ATOM 805 N THR 51 -14.858 -12.249 -10.616 1.00 0.00

ATOM 806 H THR 51 -14.493 -11.493 -10.056 1.00 0.00

ATOM 807 CA THR 51 -15.556 -13.368 -9.923 1.00 0.00

ATOM 808 HA THR 51 -16.254 -13.860 -10.600 1.00 0.00

ATOM 809 CB THR 51 -14.527 -14.397 -9.451 1.00 0.00

ATOM 810 HB THR 51 -13.864 -14.653 -10.278 1.00 0.00

ATOM 811 CG2 THR 51 -13.706 -13.812 -8.302 1.00 0.00

ATOM 812 HG21 THR 51 -14.368 -13.557 -7.475 1.00 0.00

ATOM 813 HG22 THR 51 -12.973 -14.547 -7.967 1.00 0.00

ATOM 814 HG23 THR 51 -13.190 -12.915 -8.644 1.00 0.00

ATOM 815 OG1 THR 51 -15.198 -15.569 -9.008 1.00 0.00

ATOM 816 HG1 THR 51 -14.552 -16.215 -8.712 1.00 0.00

ATOM 817 C THR 51 -16.322 -12.823 -8.716 1.00 0.00

ATOM 818 O THR 51 -15.996 -11.782 -8.181 1.00 0.00

ATOM 819 N ARG 52 -17.338 -13.518 -8.283 1.00 0.00

ATOM 820 H ARG 52 -17.614 -14.385 -8.722 1.00 0.00

ATOM 821 CA ARG 52 -18.124 -13.038 -7.111 1.00 0.00

ATOM 822 HA ARG 52 -18.538 -12.051 -7.313 1.00 0.00

ATOM 823 CB ARG 52 -19.277 -14.006 -6.839 1.00 0.00

ATOM 824 HB2 ARG 52 -18.883 -15.015 -6.710 1.00 0.00

ATOM 825 HB3 ARG 52 -19.801 -13.703 -5.933 1.00 0.00

ATOM 826 CG ARG 52 -20.248 -13.986 -8.021 1.00 0.00

ATOM 827 HG2 ARG 52 -20.564 -12.961 -8.215 1.00 0.00

ATOM 828 HG3 ARG 52 -19.753 -14.388 -8.906 1.00 0.00

ATOM 829 CD ARG 52 -21.473 -14.843 -7.689 1.00 0.00

ATOM 830 HD2 ARG 52 -22.005 -14.427 -6.834 1.00 0.00

ATOM 831 HD3 ARG 52 -22.144 -14.891 -8.546 1.00 0.00

ATOM 832 NE ARG 52 -21.033 -16.225 -7.350 1.00 0.00

ATOM 833 HE ARG 52 -20.116 -16.410 -6.969 1.00 0.00

ATOM 834 CZ ARG 52 -21.841 -17.232 -7.544 1.00 0.00

ATOM 835 NH1 ARG 52 -22.245 -17.949 -6.531 1.00 0.00

ATOM 836 HH11 ARG 52 -21.930 -17.723 -5.598 1.00 0.00

ATOM 837 HH12 ARG 52 -22.870 -18.728 -6.685 1.00 0.00

ATOM 838 NH2 ARG 52 -22.244 -17.521 -8.750 1.00 0.00

ATOM 839 HH21 ARG 52 -21.929 -16.964 -9.531 1.00 0.00

```
ATOM    840 HH22 ARG    52     -22.869 -18.301  -8.897  1.00  0.00
ATOM    841  C   ARG    52     -17.217 -12.969  -5.881  1.00  0.00
ATOM    842  O   ARG    52     -17.380 -12.121  -5.026  1.00  0.00
ATOM    843  N   SER    53     -16.263 -13.853  -5.784  1.00  0.00
ATOM    844  H   SER    53     -16.123 -14.561  -6.491  1.00  0.00
ATOM    845  CA  SER    53     -15.347 -13.838  -4.609  1.00  0.00
ATOM    846  HA  SER    53     -15.910 -13.991  -3.689  1.00  0.00
ATOM    847  CB  SER    53     -14.321 -14.965  -4.746  1.00  0.00
ATOM    848  HB2 SER    53     -13.761 -15.064  -3.816  1.00  0.00
ATOM    849  HB3 SER    53     -14.835 -15.902  -4.961  1.00  0.00
ATOM    850  OG  SER    53     -13.426 -14.663  -5.807  1.00  0.00
ATOM    851  HG  SER    53     -12.782 -15.370  -5.893  1.00  0.00
ATOM    852  C   SER    53     -14.623 -12.492  -4.544  1.00  0.00
ATOM    853  O   SER    53     -14.124 -12.094  -3.509  1.00  0.00
ATOM    854  N   SER    54     -14.559 -11.787  -5.640  1.00  0.00
ATOM    855  H   SER    54     -14.971 -12.112  -6.503  1.00  0.00
ATOM    856  CA  SER    54     -13.866 -10.468  -5.640  1.00  0.00
ATOM    857  HA  SER    54     -12.799 -10.602  -5.460  1.00  0.00
ATOM    858  CB  SER    54     -14.052  -9.793  -7.000  1.00  0.00
ATOM    859  HB2 SER    54     -13.733 -10.473  -7.790  1.00  0.00
```

```
ATOM 860 HB3 SER 54 -15.103 -9.541 -7.140 1.00 0.00
ATOM 861 OG SER 54 -13.271 -8.609 -7.052 1.00 0.00
ATOM 862 HG SER 54 -13.388 -8.186 -7.906 1.00 0.00
ATOM 863 C SER 54 -14.460 -9.580 -4.545 1.00 0.00
ATOM 864 O SER 54 -13.777 -8.769 -3.952 1.00 0.00
ATOM 865 N ASP 55 -15.727 -9.725 -4.273 1.00 0.00
ATOM 866 H ASP 55 -16.301 -10.396 -4.763 1.00 0.00
ATOM 867 CA ASP 55 -16.363 -8.888 -3.216 1.00 0.00
ATOM 868 HA ASP 55 -16.261 -7.831 -3.459 1.00 0.00
ATOM 869 CB ASP 55 -17.852 -9.228 -3.124 1.00 0.00
ATOM 870 HB2 ASP 55 -17.972 -10.305 -3.005 1.00 0.00
ATOM 871 HB3 ASP 55 -18.289 -8.718 -2.266 1.00 0.00
ATOM 872 CG ASP 55 -18.560 -8.774 -4.402 1.00 0.00
ATOM 873 OD1 ASP 55 -17.947 -8.051 -5.170 1.00 0.00
ATOM 874 OD2 ASP 55 -19.703 -9.157 -4.591 1.00 0.00
ATOM 875 C ASP 55 -15.692 -9.167 -1.870 1.00 0.00
ATOM 876 O ASP 55 -15.690 -8.337 -0.983 1.00 0.00
ATOM 877 N GLN 56 -15.121 -10.330 -1.711 1.00 0.00
ATOM 878 H GLN 56 -15.118 -11.025 -2.444 1.00 0.00
ATOM 879 CA GLN 56 -14.450 -10.660 -0.422 1.00 0.00
```

ATOM 880 HA GLN 56 -15.152 -10.564 0.406 1.00 0.00

ATOM 881 CB GLN 56 -13.942 -12.102 -0.467 1.00 0.00

ATOM 882 HB2 GLN 56 -13.224 -12.210 -1.281 1.00 0.00

ATOM 883 HB3 GLN 56 -13.457 -12.347 0.478 1.00 0.00

ATOM 884 CG GLN 56 -15.119 -13.052 -0.697 1.00 0.00

ATOM 885 HG2 GLN 56 -15.959 -12.776 -0.060 1.00 0.00

ATOM 886 HG3 GLN 56 -15.430 -13.022 -1.741 1.00 0.00

ATOM 887 CD GLN 56 -14.695 -14.482 -0.355 1.00 0.00

ATOM 888 OE1 GLN 56 -15.478 -15.253 0.162 1.00 0.00

ATOM 889 NE2 GLN 56 -13.479 -14.871 -0.625 1.00 0.00

ATOM 890 HE21 GLN 56 -13.192 -15.814 -0.402 1.00 0.00

ATOM 891 HE22 GLN 56 -12.830 -14.228 -1.056 1.00 0.00

ATOM 892 C GLN 56 -13.270 -9.712 -0.201 1.00 0.00

ATOM 893 O GLN 56 -12.967 -9.332 0.912 1.00 0.00

ATOM 894 N GLN 57 -12.602 -9.327 -1.254 1.00 0.00

ATOM 895 H GLN 57 -12.848 -9.640 -2.182 1.00 0.00

ATOM 896 CA GLN 57 -11.441 -8.405 -1.104 1.00 0.00

ATOM 897 HA GLN 57 -10.595 -8.931 -0.660 1.00 0.00

ATOM 898 CB GLN 57 -11.024 -7.882 -2.480 1.00 0.00

ATOM 899 HB2 GLN 57 -11.870 -7.385 -2.953 1.00 0.00

ATOM 900 HB3 GLN 57 -10.203 -7.174 -2.368 1.00 0.00

ATOM 901 CG GLN 57 -10.570 -9.054 -3.352 1.00 0.00

ATOM 902 HG2 GLN 57 -9.682 -9.521 -2.926 1.00 0.00

ATOM 903 HG3 GLN 57 -11.365 -9.795 -3.434 1.00 0.00

ATOM 904 CD GLN 57 -10.230 -8.545 -4.754 1.00 0.00

ATOM 905 OE1 GLN 57 -10.624 -7.460 -5.132 1.00 0.00

ATOM 906 NE2 GLN 57 -9.508 -9.290 -5.547 1.00 0.00

ATOM 907 HE21 GLN 57 -9.279 -8.962 -6.474 1.00 0.00

ATOM 908 HE22 GLN 57 -9.181 -10.192 -5.230 1.00 0.00

ATOM 909 C GLN 57 -11.836 -7.229 -0.207 1.00 0.00

ATOM 910 O GLN 57 -11.107 -6.844 0.685 1.00 0.00

ATOM 911 N GLN 58 -12.985 -6.654 -0.437 1.00 0.00

ATOM 912 H GLN 58 -13.597 -6.969 -1.176 1.00 0.00

ATOM 913 CA GLN 58 -13.424 -5.505 0.402 1.00 0.00

ATOM 914 HA GLN 58 -12.842 -4.616 0.159 1.00 0.00

ATOM 915 CB GLN 58 -14.902 -5.211 0.134 1.00 0.00

ATOM 916 HB2 GLN 58 -15.503 -6.072 0.428 1.00 0.00

ATOM 917 HB3 GLN 58 -15.210 -4.340 0.711 1.00 0.00

ATOM 918 CG GLN 58 -15.105 -4.933 -1.357 1.00 0.00

ATOM 919 HG2 GLN 58 -14.322 -4.273 -1.731 1.00 0.00

ATOM 920 HG3 GLN 58 -15.092 -5.866 -1.921 1.00 0.00

ATOM 921 CD GLN 58 -16.460 -4.253 -1.567 1.00 0.00

ATOM 922 OE1 GLN 58 -16.602 -3.411 -2.431 1.00 0.00

ATOM 923 NE2 GLN 58 -17.468 -4.586 -0.809 1.00 0.00

ATOM 924 HE21 GLN 58 -18.365 -4.142 -0.942 1.00 0.00

ATOM 925 HE22 GLN 58 -17.347 -5.288 -0.092 1.00 0.00

ATOM 926 C GLN 58 -13.236 -5.852 1.880 1.00 0.00

ATOM 927 O GLN 58 -12.804 -5.035 2.669 1.00 0.00

ATOM 928 N ARG 59 -13.558 -7.058 2.262 1.00 0.00

ATOM 929 H ARG 59 -13.918 -7.743 1.612 1.00 0.00

ATOM 930 CA ARG 59 -13.400 -7.454 3.689 1.00 0.00

ATOM 931 HA ARG 59 -14.136 -6.941 4.308 1.00 0.00

ATOM 932 CB ARG 59 -13.613 -8.963 3.826 1.00 0.00

ATOM 933 HB2 ARG 59 -12.884 -9.492 3.211 1.00 0.00

ATOM 934 HB3 ARG 59 -13.488 -9.256 4.869 1.00 0.00

ATOM 935 CG ARG 59 -15.026 -9.321 3.362 1.00 0.00

ATOM 936 HG2 ARG 59 -15.755 -8.767 3.953 1.00 0.00

ATOM 937 HG3 ARG 59 -15.140 -9.062 2.309 1.00 0.00

ATOM 938 CD ARG 59 -15.257 -10.823 3.545 1.00 0.00

ATOM 939 HD2 ARG 59 -14.468 -11.390 3.051 1.00 0.00

ATOM 940 HD3 ARG 59 -15.276 -11.077 4.605 1.00 0.00

ATOM 941 NE ARG 59 -16.566 -11.200 2.940 1.00 0.00

ATOM 942 HE ARG 59 -16.659 -11.378 1.950 1.00 0.00

ATOM 943 CZ ARG 59 -17.626 -11.308 3.695 1.00 0.00

ATOM 944 NH1 ARG 59 -17.925 -12.455 4.240 1.00 0.00

ATOM 945 HH11 ARG 59 -17.334 -13.258 4.076 1.00 0.00

ATOM 946 HH12 ARG 59 -18.745 -12.535 4.823 1.00 0.00

ATOM 947 NH2 ARG 59 -18.386 -10.267 3.904 1.00 0.00

ATOM 948 HH21 ARG 59 -18.151 -9.381 3.480 1.00 0.00

ATOM 949 HH22 ARG 59 -19.205 -10.353 4.488 1.00 0.00

ATOM 950 C ARG 59 -11.995 -7.090 4.168 1.00 0.00

ATOM 951 O ARG 59 -11.781 -6.797 5.327 1.00 0.00

ATOM 952 N CYX 60 -11.035 -7.099 3.286 1.00 0.00

ATOM 953 H CYX 60 -11.202 -7.339 2.319 1.00 0.00

ATOM 954 CA CYX 60 -9.647 -6.747 3.696 1.00 0.00

ATOM 955 HA CYX 60 -9.197 -7.567 4.257 1.00 0.00

ATOM 956 CB CYX 60 -8.800 -6.486 2.447 1.00 0.00

ATOM 957 HB2 CYX 60 -8.549 -7.435 1.972 1.00 0.00

ATOM 958 HB3 CYX 60 -9.363 -5.868 1.748 1.00 0.00

ATOM 959 SG CYX 60 -7.242 -5.694 2.919 1.00 0.00

ATOM	960	C	CYX	60	-9.689	-5.487	4.563	1.00	0.00
ATOM	961	O	CYX	60	-9.777	-5.556	5.774	1.00	0.00
ATOM	962	N	CYX	61	-9.627	-4.339	3.951	1.00	0.00
ATOM	963	H	CYX	61	-9.550	-4.276	2.946	1.00	0.00
ATOM	964	CA	CYX	61	-9.665	-3.074	4.738	1.00	0.00
ATOM	965	HA	CYX	61	-8.693	-2.887	5.196	1.00	0.00
ATOM	966	CB	CYX	61	-10.005	-1.903	3.815	1.00	0.00
ATOM	967	HB2	CYX	61	-10.083	-2.260	2.788	1.00	0.00
ATOM	968	HB3	CYX	61	-10.954	-1.464	4.120	1.00	0.00
ATOM	969	SG	CYX	61	-8.671	-0.680	3.878	1.00	0.00
ATOM	970	C	CYX	61	-10.729	-3.191	5.832	1.00	0.00
ATOM	971	O	CYX	61	-10.542	-2.740	6.944	1.00	0.00
ATOM	972	N	ASP	62	-11.843	-3.795	5.523	1.00	0.00
ATOM	973	H	ASP	62	-12.004	-4.173	4.600	1.00	0.00
ATOM	974	CA	ASP	62	-12.919	-3.941	6.545	1.00	0.00
ATOM	975	HA	ASP	62	-13.249	-2.961	6.888	1.00	0.00
ATOM	976	CB	ASP	62	-14.113	-4.671	5.929	1.00	0.00
ATOM	977	HB2	ASP	62	-13.782	-5.621	5.509	1.00	0.00
ATOM	978	HB3	ASP	62	-14.862	-4.856	6.698	1.00	0.00
ATOM	979	CG	ASP	62	-14.722	-3.809	4.821	1.00	0.00

ATOM 980 OD1 ASP 62 -14.368 -2.643 4.743 1.00 0.00

ATOM 981 OD2 ASP 62 -15.531 -4.327 4.069 1.00 0.00

ATOM 982 C ASP 62 -12.388 -4.744 7.732 1.00 0.00

ATOM 983 O ASP 62 -12.718 -4.480 8.872 1.00 0.00

ATOM 984 N GLU 63 -11.564 -5.720 7.476 1.00 0.00

ATOM 985 H GLU 63 -11.285 -5.947 6.532 1.00 0.00

ATOM 986 CA GLU 63 -11.007 -6.535 8.591 1.00 0.00

ATOM 987 HA GLU 63 -11.814 -6.967 9.183 1.00 0.00

ATOM 988 CB GLU 63 -10.158 -7.672 8.019 1.00 0.00

ATOM 989 HB2 GLU 63 -9.554 -7.295 7.193 1.00 0.00

ATOM 990 HB3 GLU 63 -9.503 -8.064 8.797 1.00 0.00

ATOM 991 CG GLU 63 -11.073 -8.788 7.513 1.00 0.00

ATOM 992 HG2 GLU 63 -11.750 -9.093 8.311 1.00 0.00

ATOM 993 HG3 GLU 63 -11.653 -8.426 6.664 1.00 0.00

ATOM 994 CD GLU 63 -10.226 -9.985 7.078 1.00 0.00

ATOM 995 OE1 GLU 63 -9.014 -9.903 7.198 1.00 0.00

ATOM 996 OE2 GLU 63 -10.803 -10.964 6.632 1.00 0.00

ATOM 997 C GLU 63 -10.139 -5.642 9.478 1.00 0.00

ATOM 998 O GLU 63 -10.281 -5.620 10.684 1.00 0.00

ATOM 999 N LEU 64 -9.240 -4.904 8.887 1.00 0.00

ATOM 1000 H LEU 64 -9.119 -4.920 7.884 1.00 0.00

ATOM 1001 CA LEU 64 -8.360 -4.010 9.690 1.00 0.00

ATOM 1002 HA LEU 64 -7.885 -4.570 10.496 1.00 0.00

ATOM 1003 CB LEU 64 -7.268 -3.428 8.789 1.00 0.00

ATOM 1004 HB2 LEU 64 -7.718 -2.762 8.054 1.00 0.00

ATOM 1005 HB3 LEU 64 -6.555 -2.870 9.397 1.00 0.00

ATOM 1006 CG LEU 64 -6.542 -4.565 8.068 1.00 0.00

ATOM 1007 HG LEU 64 -7.233 -5.068 7.391 1.00 0.00

ATOM 1008 CD1 LEU 64 -5.370 -3.993 7.267 1.00 0.00

ATOM 1009 HD11 LEU 64 -4.678 -3.491 7.943 1.00 0.00

ATOM 1010 HD12 LEU 64 -4.851 -4.803 6.753 1.00 0.00

ATOM 1011 HD13 LEU 64 -5.744 -3.279 6.534 1.00 0.00

ATOM 1012 CD2 LEU 64 -6.014 -5.568 9.095 1.00 0.00

ATOM 1013 HD21 LEU 64 -6.847 -5.976 9.667 1.00 0.00

ATOM 1014 HD22 LEU 64 -5.497 -6.378 8.580 1.00 0.00

ATOM 1015 HD23 LEU 64 -5.321 -5.067 9.770 1.00 0.00

ATOM 1016 C LEU 64 -9.187 -2.867 10.287 1.00 0.00

ATOM 1017 O LEU 64 -8.676 -2.030 11.003 1.00 0.00

ATOM 1018 N ASN 65 -10.461 -2.823 10.001 1.00 0.00

ATOM 1019 H ASN 65 -10.897 -3.515 9.409 1.00 0.00

ATOM 1020 CA ASN 65 -11.311 -1.731 10.558 1.00 0.00

ATOM 1021 HA ASN 65 -10.686 -0.919 10.930 1.00 0.00

ATOM 1022 CB ASN 65 -12.223 -1.182 9.459 1.00 0.00

ATOM 1023 HB2 ASN 65 -12.909 -1.958 9.118 1.00 0.00

ATOM 1024 HB3 ASN 65 -12.793 -0.331 9.831 1.00 0.00

ATOM 1025 CG ASN 65 -11.377 -0.722 8.273 1.00 0.00

ATOM 1026 OD1 ASN 65 -11.893 -0.735 7.074 1.00 0.00

ATOM 1027 ND2 ASN 65 -10.232 -0.347 8.436 1.00 0.00

ATOM 1028 HD21 ASN 65 -9.684 -0.046 7.642 1.00 0.00

ATOM 1029 HD22 ASN 65 -9.834 -0.339 9.364 1.00 0.00

ATOM 1030 C ASN 65 -12.170 -2.281 11.699 1.00 0.00

ATOM 1031 O ASN 65 -12.810 -1.540 12.417 1.00 0.00

ATOM 1032 N GLU 66 -12.192 -3.575 11.871 1.00 0.00

ATOM 1033 H GLU 66 -11.663 -4.201 11.280 1.00 0.00

ATOM 1034 CA GLU 66 -13.016 -4.164 12.966 1.00 0.00

ATOM 1035 HA GLU 66 -13.191 -3.426 13.748 1.00 0.00

ATOM 1036 CB GLU 66 -14.367 -4.607 12.401 1.00 0.00

ATOM 1037 HB2 GLU 66 -14.216 -5.428 11.700 1.00 0.00

ATOM 1038 HB3 GLU 66 -15.010 -4.940 13.216 1.00 0.00

ATOM 1039 CG GLU 66 -15.028 -3.433 11.677 1.00 0.00

ATOM 1040 HG2 GLU 66 -15.284 -2.659 12.400 1.00 0.00

ATOM 1041 HG3 GLU 66 -14.338 -3.026 10.938 1.00 0.00

ATOM 1042 CD GLU 66 -16.299 -3.916 10.976 1.00 0.00

ATOM 1043 OE1 GLU 66 -16.487 -5.120 10.898 1.00 0.00

ATOM 1044 OE2 GLU 66 -17.062 -3.076 10.529 1.00 0.00

ATOM 1045 C GLU 66 -12.296 -5.375 13.567 1.00 0.00

ATOM 1046 O GLU 66 -12.417 -5.659 14.741 1.00 0.00

ATOM 1047 N MET 67 -11.552 -6.094 12.772 1.00 0.00

ATOM 1048 H MET 67 -11.444 -5.869 11.793 1.00 0.00

ATOM 1049 CA MET 67 -10.834 -7.287 13.305 1.00 0.00

ATOM 1050 HA MET 67 -11.499 -7.880 13.933 1.00 0.00

ATOM 1051 CB MET 67 -10.358 -8.157 12.140 1.00 0.00

ATOM 1052 HB2 MET 67 -10.528 -7.633 11.200 1.00 0.00

ATOM 1053 HB3 MET 67 -9.294 -8.365 12.252 1.00 0.00

ATOM 1054 CG MET 67 -11.137 -9.474 12.137 1.00 0.00

ATOM 1055 HG2 MET 67 -11.557 -9.657 13.126 1.00 0.00

ATOM 1056 HG3 MET 67 -11.942 -9.425 11.404 1.00 0.00

ATOM 1057 SD MET 67 -10.022 -10.832 11.704 1.00 0.00

ATOM 1058 CE MET 67 -9.912 -10.478 9.933 1.00 0.00

ATOM 1059 HE1 MET 67 -9.504 -9.478 9.786 1.00 0.00

ATOM 1060 HE2 MET 67 -9.259 -11.210 9.456 1.00 0.00

ATOM 1061 HE3 MET 67 -10.905 -10.534 9.488 1.00 0.00

ATOM 1062 C MET 67 -9.629 -6.835 14.129 1.00 0.00

ATOM 1063 O MET 67 -9.647 -6.865 15.344 1.00 0.00

ATOM 1064 N GLU 68 -8.581 -6.419 13.478 1.00 0.00

ATOM 1065 H GLU 68 -8.560 -6.395 12.468 1.00 0.00

ATOM 1066 CA GLU 68 -7.371 -5.967 14.220 1.00 0.00

ATOM 1067 HA GLU 68 -7.165 -6.639 15.054 1.00 0.00

ATOM 1068 CB GLU 68 -6.165 -5.973 13.279 1.00 0.00

ATOM 1069 HB2 GLU 68 -6.256 -5.155 12.565 1.00 0.00

ATOM 1070 HB3 GLU 68 -5.250 -5.849 13.859 1.00 0.00

ATOM 1071 CG GLU 68 -6.114 -7.303 12.525 1.00 0.00

ATOM 1072 HG2 GLU 68 -5.909 -8.112 13.227 1.00 0.00

ATOM 1073 HG3 GLU 68 -7.072 -7.481 12.037 1.00 0.00

ATOM 1074 CD GLU 68 -5.007 -7.251 11.470 1.00 0.00

ATOM 1075 OE1 GLU 68 -4.438 -6.187 11.288 1.00 0.00

ATOM 1076 OE2 GLU 68 -4.746 -8.277 10.862 1.00 0.00

ATOM 1077 C GLU 68 -7.594 -4.551 14.756 1.00 0.00

ATOM 1078 O GLU 68 -6.712 -3.953 15.339 1.00 0.00

ATOM 1079 N ASN 69 -8.766 -4.010 14.565 1.00 0.00

```
ATOM   1080  H   ASN    69      -9.506  -4.500  14.083  1.00  0.00
ATOM   1081  CA  ASN    69      -9.039  -2.632  15.065  1.00  0.00
ATOM   1082  HA  ASN    69      -9.963  -2.249  14.633  1.00  0.00
ATOM   1083  CB  ASN    69      -9.187  -2.663  16.588  1.00  0.00
ATOM   1084  HB2 ASN    69      -8.253  -2.982  17.052  1.00  0.00
ATOM   1085  HB3 ASN    69      -9.459  -1.676  16.961  1.00  0.00
ATOM   1086  CG  ASN    69     -10.287  -3.652  16.974  1.00  0.00
ATOM   1087  OD1 ASN    69     -11.222  -3.864  16.226  1.00  0.00
ATOM   1088  ND2 ASN    69     -10.216  -4.273  18.120  1.00  0.00
ATOM   1089 HD21 ASN    69     -10.939  -4.928  18.382  1.00  0.00
ATOM   1090 HD22 ASN    69      -9.438  -4.096  18.740  1.00  0.00
ATOM   1091  C   ASN    69      -7.876  -1.714  14.682  1.00  0.00
ATOM   1092  O   ASN    69      -7.619  -0.720  15.332  1.00  0.00
ATOM   1093  N   THR    70      -7.173  -2.041  13.634  1.00  0.00
ATOM   1094  H   THR    70      -7.384  -2.867  13.091  1.00  0.00
ATOM   1095  CA  THR    70      -6.025  -1.189  13.208  1.00  0.00
ATOM   1096  HA  THR    70      -5.158  -1.373  13.842  1.00  0.00
ATOM   1097  CB  THR    70      -5.652  -1.523  11.761  1.00  0.00
ATOM   1098  HB  THR    70      -4.643  -1.165  11.555  1.00  0.00
ATOM   1099  CG2 THR    70      -5.707  -3.038  11.553  1.00  0.00
```

```
ATOM   1100 HG21 THR    70      -6.715  -3.398  11.759  1.00  0.00
ATOM   1101 HG22 THR    70      -5.441  -3.274  10.522  1.00  0.00
ATOM   1102 HG23 THR    70      -5.003  -3.524  12.229  1.00  0.00
ATOM   1103  OG1 THR    70      -6.566  -0.888  10.878  1.00  0.00
ATOM   1104  HG1 THR    70      -6.332  -1.098   9.971  1.00  0.00
ATOM   1105  C   THR    70      -6.420   0.286  13.305  1.00  0.00
ATOM   1106  O   THR    70      -7.586   0.628  13.312  1.00  0.00
ATOM   1107  N   GLN    71      -5.457   1.165  13.381  1.00  0.00
ATOM   1108  H   GLN    71      -4.485   0.889  13.376  1.00  0.00
ATOM   1109  CA  GLN    71      -5.780   2.617  13.478  1.00  0.00
ATOM   1110  HA  GLN    71      -6.399   2.923  12.635  1.00  0.00
ATOM   1111  CB  GLN    71      -6.550   2.883  14.772  1.00  0.00
ATOM   1112  HB2 GLN    71      -7.490   2.332  14.756  1.00  0.00
ATOM   1113  HB3 GLN    71      -5.953   2.558  15.624  1.00  0.00
ATOM   1114  CG  GLN    71      -6.839   4.382  14.893  1.00  0.00
ATOM   1115  HG2 GLN    71      -5.927   4.956  14.730  1.00  0.00
ATOM   1116  HG3 GLN    71      -7.592   4.682  14.164  1.00  0.00
ATOM   1117  CD  GLN    71      -7.367   4.689  16.297  1.00  0.00
ATOM   1118  OE1 GLN    71      -6.766   4.309  17.282  1.00  0.00
ATOM   1119  NE2 GLN    71      -8.475   5.366  16.429  1.00  0.00
```

ATOM 1120 HE21 GLN 71 -8.830 5.572 17.352 1.00 0.00

ATOM 1121 HE22 GLN 71 -8.972 5.680 15.608 1.00 0.00

ATOM 1122 C GLN 71 -4.483 3.430 13.480 1.00 0.00

ATOM 1123 O GLN 71 -4.427 4.527 12.960 1.00 0.00

ATOM 1124 N GLY 72 -3.441 2.904 14.065 1.00 0.00

ATOM 1125 H GLY 72 -3.479 1.993 14.501 1.00 0.00

ATOM 1126 CA GLY 72 -2.152 3.651 14.101 1.00 0.00

ATOM 1127 HA2 GLY 72 -2.310 4.679 13.775 1.00 0.00

ATOM 1128 HA3 GLY 72 -1.750 3.648 15.114 1.00 0.00

ATOM 1129 C GLY 72 -1.144 2.981 13.164 1.00 0.00

ATOM 1130 O GLY 72 -1.282 1.826 12.811 1.00 0.00

ATOM 1131 N CYX 73 -0.130 3.696 12.761 1.00 0.00

ATOM 1132 H CYX 73 -0.010 4.656 13.051 1.00 0.00

ATOM 1133 CA CYX 73 0.886 3.102 11.848 1.00 0.00

ATOM 1134 HA CYX 73 1.708 3.800 11.691 1.00 0.00

ATOM 1135 CB CYX 73 1.445 1.821 12.470 1.00 0.00

ATOM 1136 HB2 CYX 73 0.840 1.540 13.332 1.00 0.00

ATOM 1137 HB3 CYX 73 1.421 1.018 11.733 1.00 0.00

ATOM 1138 SG CYX 73 3.135 2.116 13.049 1.00 0.00

ATOM 1139 C CYX 73 0.235 2.775 10.501 1.00 0.00

ATOM 1140 O CYX 73 0.820 2.123 9.659 1.00 0.00

ATOM 1141 N MET 74 -0.972 3.224 10.292 1.00 0.00

ATOM 1142 H MET 74 -1.463 3.767 10.987 1.00 0.00

ATOM 1143 CA MET 74 -1.660 2.939 9.002 1.00 0.00

ATOM 1144 HA MET 74 -2.024 1.911 8.986 1.00 0.00

ATOM 1145 CB MET 74 -2.854 3.884 8.841 1.00 0.00

ATOM 1146 HB2 MET 74 -2.497 4.909 8.744 1.00 0.00

ATOM 1147 HB3 MET 74 -3.418 3.610 7.949 1.00 0.00

ATOM 1148 CG MET 74 -3.758 3.772 10.071 1.00 0.00

ATOM 1149 HG2 MET 74 -3.899 2.722 10.330 1.00 0.00

ATOM 1150 HG3 MET 74 -3.303 4.295 10.912 1.00 0.00

ATOM 1151 SD MET 74 -5.367 4.517 9.707 1.00 0.00

ATOM 1152 CE MET 74 -6.237 2.987 9.285 1.00 0.00

ATOM 1153 HE1 MET 74 -6.221 2.311 10.141 1.00 0.00

ATOM 1154 HE2 MET 74 -7.269 3.216 9.022 1.00 0.00

ATOM 1155 HE3 MET 74 -5.744 2.510 8.438 1.00 0.00

ATOM 1156 C MET 74 -0.679 3.152 7.847 1.00 0.00

ATOM 1157 O MET 74 -0.453 2.270 7.042 1.00 0.00

ATOM 1158 N CYX 75 -0.093 4.315 7.760 1.00 0.00

ATOM 1159 H CYX 75 -0.274 5.054 8.424 1.00 0.00

ATOM 1160 CA CYX 75 0.873 4.578 6.657 1.00 0.00

ATOM 1161 HA CYX 75 0.344 4.701 5.712 1.00 0.00

ATOM 1162 CB CYX 75 1.649 5.863 6.954 1.00 0.00

ATOM 1163 HB2 CYX 75 0.956 6.641 7.272 1.00 0.00

ATOM 1164 HB3 CYX 75 2.373 5.675 7.747 1.00 0.00

ATOM 1165 SG CYX 75 2.471 6.432 5.446 1.00 0.00

ATOM 1166 C CYX 75 1.849 3.407 6.544 1.00 0.00

ATOM 1167 O CYX 75 2.058 2.861 5.478 1.00 0.00

ATOM 1168 N GLU 76 2.448 3.011 7.635 1.00 0.00

ATOM 1169 H GLU 76 2.282 3.456 8.526 1.00 0.00

ATOM 1170 CA GLU 76 3.406 1.871 7.583 1.00 0.00

ATOM 1171 HA GLU 76 4.249 2.115 6.937 1.00 0.00

ATOM 1172 CB GLU 76 3.936 1.581 8.989 1.00 0.00

ATOM 1173 HB2 GLU 76 3.109 1.293 9.638 1.00 0.00

ATOM 1174 HB3 GLU 76 4.661 0.768 8.944 1.00 0.00

ATOM 1175 CG GLU 76 4.610 2.835 9.548 1.00 0.00

ATOM 1176 HG2 GLU 76 5.362 3.187 8.842 1.00 0.00

ATOM 1177 HG3 GLU 76 3.862 3.612 9.701 1.00 0.00

ATOM 1178 CD GLU 76 5.281 2.504 10.883 1.00 0.00

ATOM 1179 OE1 GLU 76 5.274 1.341 11.255 1.00 0.00

ATOM	1180	OE2	GLU	76	5.789	3.417	11.511	1.00	0.00
ATOM	1181	C	GLU	76	2.685	0.635	7.042	1.00	0.00
ATOM	1182	O	GLU	76	3.301	-0.306	6.580	1.00	0.00
ATOM	1183	N	ALA	77	1.380	0.636	7.087	1.00	0.00
ATOM	1184	H	ALA	77	0.862	1.414	7.468	1.00	0.00
ATOM	1185	CA	ALA	77	0.618	-0.533	6.568	1.00	0.00
ATOM	1186	HA	ALA	77	1.087	-1.481	6.835	1.00	0.00
ATOM	1187	CB	ALA	77	-0.803	-0.513	7.136	1.00	0.00
ATOM	1188	HB1	ALA	77	-1.288	0.425	6.869	1.00	0.00
ATOM	1189	HB2	ALA	77	-1.371	-1.347	6.723	1.00	0.00
ATOM	1190	HB3	ALA	77	-0.762	-0.605	8.221	1.00	0.00
ATOM	1191	C	ALA	77	0.562	-0.454	5.043	1.00	0.00
ATOM	1192	O	ALA	77	1.020	-1.341	4.349	1.00	0.00
ATOM	1193	N	LEU	78	0.012	0.603	4.513	1.00	0.00
ATOM	1194	H	LEU	78	-0.370	1.346	5.080	1.00	0.00
ATOM	1195	CA	LEU	78	-0.060	0.737	3.032	1.00	0.00
ATOM	1196	HA	LEU	78	-0.706	-0.036	2.615	1.00	0.00
ATOM	1197	CB	LEU	78	-0.636	2.106	2.666	1.00	0.00
ATOM	1198	HB2	LEU	78	-1.594	2.243	3.167	1.00	0.00
ATOM	1199	HB3	LEU	78	0.054	2.888	2.983	1.00	0.00

ATOM 1200 CG LEU 78 -0.836 2.186 1.153 1.00 0.00

ATOM 1201 HG LEU 78 0.074 1.861 0.648 1.00 0.00

ATOM 1202 CD1 LEU 78 -1.998 1.279 0.743 1.00 0.00

ATOM 1203 HD11 LEU 78 -2.908 1.603 1.247 1.00 0.00

ATOM 1204 HD12 LEU 78 -2.141 1.336 -0.336 1.00 0.00

ATOM 1205 HD13 LEU 78 -1.774 0.250 1.026 1.00 0.00

ATOM 1206 CD2 LEU 78 -1.151 3.629 0.754 1.00 0.00

ATOM 1207 HD21 LEU 78 -0.323 4.275 1.045 1.00 0.00

ATOM 1208 HD22 LEU 78 -1.293 3.685 -0.325 1.00 0.00

ATOM 1209 HD23 LEU 78 -2.061 3.955 1.258 1.00 0.00

ATOM 1210 C LEU 78 1.350 0.604 2.456 1.00 0.00

ATOM 1211 O LEU 78 1.573 -0.096 1.489 1.00 0.00

ATOM 1212 N GLN 79 2.304 1.269 3.049 1.00 0.00

ATOM 1213 H GLN 79 2.124 1.852 3.854 1.00 0.00

ATOM 1214 CA GLN 79 3.700 1.178 2.542 1.00 0.00

ATOM 1215 HA GLN 79 3.771 1.624 1.550 1.00 0.00

ATOM 1216 CB GLN 79 4.640 1.933 3.486 1.00 0.00

ATOM 1217 HB2 GLN 79 4.511 1.560 4.502 1.00 0.00

ATOM 1218 HB3 GLN 79 5.672 1.779 3.171 1.00 0.00

ATOM 1219 CG GLN 79 4.312 3.427 3.445 1.00 0.00

ATOM 1220 HG2 GLN 79 4.483 3.823 2.444 1.00 0.00

ATOM 1221 HG3 GLN 79 3.274 3.595 3.729 1.00 0.00

ATOM 1222 CD GLN 79 5.216 4.174 4.428 1.00 0.00

ATOM 1223 OE1 GLN 79 5.439 5.448 4.252 1.00 0.00

ATOM 1224 NE2 GLN 79 5.726 3.592 5.365 1.00 0.00

ATOM 1225 HE21 GLN 79 6.321 4.097 6.006 1.00 0.00

ATOM 1226 HE22 GLN 79 5.553 2.605 5.498 1.00 0.00

ATOM 1227 C GLN 79 4.115 -0.290 2.476 1.00 0.00

ATOM 1228 O GLN 79 4.679 -0.744 1.501 1.00 0.00

ATOM 1229 N GLN 80 3.832 -1.040 3.505 1.00 0.00

ATOM 1230 H GLN 80 3.360 -0.673 4.318 1.00 0.00

ATOM 1231 CA GLN 80 4.205 -2.482 3.497 1.00 0.00

ATOM 1232 HA GLN 80 5.290 -2.591 3.478 1.00 0.00

ATOM 1233 CB GLN 80 3.668 -3.158 4.760 1.00 0.00

ATOM 1234 HB2 GLN 80 3.992 -2.599 5.638 1.00 0.00

ATOM 1235 HB3 GLN 80 2.579 -3.181 4.726 1.00 0.00

ATOM 1236 CG GLN 80 4.205 -4.588 4.840 1.00 0.00

ATOM 1237 HG2 GLN 80 3.745 -5.118 5.674 1.00 0.00

ATOM 1238 HG3 GLN 80 4.000 -5.123 3.913 1.00 0.00

ATOM 1239 CD GLN 80 5.719 -4.553 5.059 1.00 0.00

ATOM 1240 OE1 GLN 80 6.206 -3.826 5.902 1.00 0.00

ATOM 1241 NE2 GLN 80 6.489 -5.313 4.329 1.00 0.00

ATOM 1242 HE21 GLN 80 7.489 -5.296 4.468 1.00 0.00

ATOM 1243 HE22 GLN 80 6.081 -5.916 3.628 1.00 0.00

ATOM 1244 C GLN 80 3.595 -3.149 2.263 1.00 0.00

ATOM 1245 O GLN 80 4.260 -3.868 1.543 1.00 0.00

ATOM 1246 N ILE 81 2.338 -2.910 2.010 1.00 0.00

ATOM 1247 H ILE 81 1.780 -2.312 2.602 1.00 0.00

ATOM 1248 CA ILE 81 1.691 -3.525 0.818 1.00 0.00

ATOM 1249 HA ILE 81 1.560 -4.594 0.986 1.00 0.00

ATOM 1250 CB ILE 81 0.326 -2.874 0.588 1.00 0.00

ATOM 1251 HB ILE 81 0.450 -1.796 0.492 1.00 0.00

ATOM 1252 CG2 ILE 81 -0.297 -3.431 -0.693 1.00 0.00

ATOM 1253 HG21 ILE 81 -0.421 -4.510 -0.598 1.00 0.00

ATOM 1254 HG22 ILE 81 -1.269 -2.967 -0.856 1.00 0.00

ATOM 1255 HG23 ILE 81 0.355 -3.215 -1.539 1.00 0.00

ATOM 1256 CG1 ILE 81 -0.593 -3.178 1.773 1.00 0.00

ATOM 1257 HG12 ILE 81 -0.635 -4.256 1.932 1.00 0.00

ATOM 1258 HG13 ILE 81 -0.203 -2.694 2.668 1.00 0.00

ATOM 1259 CD1 ILE 81 -1.999 -2.650 1.479 1.00 0.00

ATOM 1260 HD11 ILE 81 -2.389 -3.134 0.584 1.00 0.00

ATOM 1261 HD12 ILE 81 -2.653 -2.867 2.323 1.00 0.00

ATOM 1262 HD13 ILE 81 -1.957 -1.573 1.320 1.00 0.00

ATOM 1263 C ILE 81 2.581 -3.298 -0.404 1.00 0.00

ATOM 1264 O ILE 81 3.073 -4.231 -1.009 1.00 0.00

ATOM 1265 N MET 82 2.798 -2.064 -0.769 1.00 0.00

ATOM 1266 H MET 82 2.394 -1.285 -0.270 1.00 0.00

ATOM 1267 CA MET 82 3.662 -1.777 -1.947 1.00 0.00

ATOM 1268 HA MET 82 3.132 -2.007 -2.871 1.00 0.00

ATOM 1269 CB MET 82 4.036 -0.293 -1.954 1.00 0.00

ATOM 1270 HB2 MET 82 4.691 -0.079 -1.109 1.00 0.00

ATOM 1271 HB3 MET 82 4.553 -0.053 -2.883 1.00 0.00

ATOM 1272 CG MET 82 2.767 0.554 -1.843 1.00 0.00

ATOM 1273 HG2 MET 82 2.324 0.428 -0.855 1.00 0.00

ATOM 1274 HG3 MET 82 3.010 1.604 -2.001 1.00 0.00

ATOM 1275 SD MET 82 1.578 0.023 -3.100 1.00 0.00

ATOM 1276 CE MET 82 2.563 0.479 -4.549 1.00 0.00

ATOM 1277 HE1 MET 82 3.504 -0.072 -4.538 1.00 0.00

ATOM 1278 HE2 MET 82 2.011 0.234 -5.456 1.00 0.00

ATOM 1279 HE3 MET 82 2.769 1.549 -4.527 1.00 0.00

ATOM 1280 C MET 82 4.932 -2.622 -1.861 1.00 0.00

ATOM 1281 O MET 82 5.293 -3.317 -2.788 1.00 0.00

ATOM 1282 N GLU 83 5.608 -2.573 -0.749 1.00 0.00

ATOM 1283 H GLU 83 5.312 -2.001 0.028 1.00 0.00

ATOM 1284 CA GLU 83 6.853 -3.376 -0.600 1.00 0.00

ATOM 1285 HA GLU 83 7.532 -3.180 -1.430 1.00 0.00

ATOM 1286 CB GLU 83 7.554 -2.993 0.706 1.00 0.00

ATOM 1287 HB2 GLU 83 6.861 -3.111 1.539 1.00 0.00

ATOM 1288 HB3 GLU 83 8.418 -3.640 0.859 1.00 0.00

ATOM 1289 CG GLU 83 8.015 -1.536 0.628 1.00 0.00

ATOM 1290 HG2 GLU 83 8.646 -1.400 -0.251 1.00 0.00

ATOM 1291 HG3 GLU 83 7.145 -0.884 0.555 1.00 0.00

ATOM 1292 CD GLU 83 8.811 -1.185 1.886 1.00 0.00

ATOM 1293 OE1 GLU 83 8.879 -2.018 2.773 1.00 0.00

ATOM 1294 OE2 GLU 83 9.341 -0.087 1.940 1.00 0.00

ATOM 1295 C GLU 83 6.493 -4.862 -0.571 1.00 0.00

ATOM 1296 O GLU 83 7.341 -5.720 -0.713 1.00 0.00

ATOM 1297 N ASN 84 5.238 -5.169 -0.392 1.00 0.00

ATOM 1298 H ASN 84 4.530 -4.460 -0.273 1.00 0.00

ATOM 1299 CA ASN 84 4.819 -6.597 -0.358 1.00 0.00

ATOM 1300 HA ASN 84 5.557 -7.195 0.178 1.00 0.00

ATOM 1301 CB ASN 84 3.474 -6.719 0.362 1.00 0.00

ATOM 1302 HB2 ASN 84 3.517 -6.220 1.330 1.00 0.00

ATOM 1303 HB3 ASN 84 2.681 -6.276 -0.240 1.00 0.00

ATOM 1304 CG ASN 84 3.149 -8.196 0.588 1.00 0.00

ATOM 1305 OD1 ASN 84 3.770 -8.850 1.402 1.00 0.00

ATOM 1306 ND2 ASN 84 2.194 -8.755 -0.104 1.00 0.00

ATOM 1307 HD21 ASN 84 1.974 -9.731 0.040 1.00 0.00

ATOM 1308 HD22 ASN 84 1.679 -8.210 -0.781 1.00 0.00

ATOM 1309 C ASN 84 4.682 -7.120 -1.789 1.00 0.00

ATOM 1310 O ASN 84 4.731 -8.310 -2.031 1.00 0.00

ATOM 1311 N GLN 85 4.509 -6.242 -2.740 1.00 0.00

ATOM 1312 H GLN 85 4.464 -5.251 -2.550 1.00 0.00

ATOM 1313 CA GLN 85 4.371 -6.698 -4.152 1.00 0.00

ATOM 1314 HA GLN 85 5.177 -7.386 -4.409 1.00 0.00

ATOM 1315 CB GLN 85 3.035 -7.425 -4.324 1.00 0.00

ATOM 1316 HB2 GLN 85 2.915 -8.160 -3.528 1.00 0.00

ATOM 1317 HB3 GLN 85 2.219 -6.704 -4.278 1.00 0.00

ATOM 1318 CG GLN 85 3.012 -8.134 -5.680 1.00 0.00

ATOM 1319 HG2 GLN 85 3.118 -7.410 -6.488 1.00 0.00

ATOM 1320 HG3 GLN 85 3.820 -8.864 -5.740 1.00 0.00

ATOM 1321 CD GLN 85 1.679 -8.865 -5.850 1.00 0.00

ATOM 1322 OE1 GLN 85 0.977 -9.105 -4.888 1.00 0.00

ATOM 1323 NE2 GLN 85 1.298 -9.232 -7.043 1.00 0.00

ATOM 1324 HE21 GLN 85 0.419 -9.715 -7.163 1.00 0.00

ATOM 1325 HE22 GLN 85 1.884 -9.032 -7.841 1.00 0.00

ATOM 1326 C GLN 85 4.417 -5.491 -5.095 1.00 0.00

ATOM 1327 O GLN 85 3.599 -5.356 -5.982 1.00 0.00

ATOM 1328 N CYX 86 5.371 -4.616 -4.916 1.00 0.00

ATOM 1329 H CYX 86 6.058 -4.718 -4.182 1.00 0.00

ATOM 1330 CA CYX 86 5.464 -3.427 -5.812 1.00 0.00

ATOM 1331 HA CYX 86 4.482 -3.184 -6.218 1.00 0.00

ATOM 1332 CB CYX 86 5.977 -2.223 -5.019 1.00 0.00

ATOM 1333 HB2 CYX 86 6.181 -1.398 -5.702 1.00 0.00

ATOM 1334 HB3 CYX 86 5.223 -1.917 -4.295 1.00 0.00

ATOM 1335 SG CYX 86 7.529 -2.657 -4.195 1.00 0.00

ATOM 1336 C CYX 86 6.430 -3.729 -6.958 1.00 0.00

ATOM 1337 O CYX 86 6.543 -2.974 -7.903 1.00 0.00

ATOM 1338 N ASP 87 7.132 -4.828 -6.881 1.00 0.00

ATOM 1339 H ASP 87 7.045 -5.460 -6.098 1.00 0.00

ATOM	1340	CA	ASP	87	8.091	-5.177	-7.966	1.00	0.00
ATOM	1341	HA	ASP	87	8.979	-4.547	-7.904	1.00	0.00
ATOM	1342	CB	ASP	87	8.518	-6.639	-7.817	1.00	0.00
ATOM	1343	HB2	ASP	87	7.647	-7.251	-7.581	1.00	0.00
ATOM	1344	HB3	ASP	87	8.962	-6.985	-8.751	1.00	0.00
ATOM	1345	CG	ASP	87	9.545	-6.759	-6.689	1.00	0.00
ATOM	1346	OD1	ASP	87	9.978	-5.730	-6.196	1.00	0.00
ATOM	1347	OD2	ASP	87	9.881	-7.877	-6.337	1.00	0.00
ATOM	1348	C	ASP	87	7.419	-4.979	-9.326	1.00	0.00
ATOM	1349	O	ASP	87	7.977	-4.379	-10.224	1.00	0.00
ATOM	1350	N	ARG	88	6.225	-5.481	-9.487	1.00	0.00
ATOM	1351	H	ARG	88	5.756	-5.982	-8.746	1.00	0.00
ATOM	1352	CA	ARG	88	5.520	-5.323	-10.790	1.00	0.00
ATOM	1353	HA	ARG	88	6.123	-5.737	-11.599	1.00	0.00
ATOM	1354	CB	ARG	88	4.186	-6.072	-10.742	1.00	0.00
ATOM	1355	HB2	ARG	88	3.601	-5.719	-9.893	1.00	0.00
ATOM	1356	HB3	ARG	88	3.634	-5.891	-11.664	1.00	0.00
ATOM	1357	CG	ARG	88	4.448	-7.572	-10.591	1.00	0.00
ATOM	1358	HG2	ARG	88	5.038	-7.925	-11.437	1.00	0.00
ATOM	1359	HG3	ARG	88	4.995	-7.754	-9.666	1.00	0.00

ATOM 1360 CD ARG 88 3.116 -8.322 -10.551 1.00 0.00

ATOM 1361 HD2 ARG 88 2.455 -7.877 -9.808 1.00 0.00

ATOM 1362 HD3 ARG 88 2.636 -8.290 -11.529 1.00 0.00

ATOM 1363 NE ARG 88 3.356 -9.745 -10.184 1.00 0.00

ATOM 1364 HE ARG 88 3.068 -10.116 -9.289 1.00 0.00

ATOM 1365 CZ ARG 88 3.950 -10.546 -11.028 1.00 0.00

ATOM 1366 NH1 ARG 88 3.243 -11.357 -11.765 1.00 0.00

ATOM 1367 HH11 ARG 88 2.237 -11.364 -11.681 1.00 0.00

ATOM 1368 HH12 ARG 88 3.705 -11.975 -12.417 1.00 0.00

ATOM 1369 NH2 ARG 88 5.251 -10.533 -11.134 1.00 0.00

ATOM 1370 HH21 ARG 88 5.795 -9.903 -10.562 1.00 0.00

ATOM 1371 HH22 ARG 88 5.708 -11.154 -11.787 1.00 0.00

ATOM 1372 C ARG 88 5.261 -3.838 -11.058 1.00 0.00

ATOM 1373 O ARG 88 4.905 -3.449 -12.152 1.00 0.00

ATOM 1374 N LEU 89 5.440 -3.007 -10.069 1.00 0.00

ATOM 1375 H LEU 89 5.736 -3.323 -9.156 1.00 0.00

ATOM 1376 CA LEU 89 5.205 -1.549 -10.271 1.00 0.00

ATOM 1377 HA LEU 89 4.178 -1.373 -10.589 1.00 0.00

ATOM 1378 CB LEU 89 5.445 -0.804 -8.956 1.00 0.00

ATOM 1379 HB2 LEU 89 4.958 -1.338 -8.140 1.00 0.00

ATOM 1380 HB3 LEU 89 6.516 -0.745 -8.763 1.00 0.00

ATOM 1381 CG LEU 89 4.867 0.608 -9.058 1.00 0.00

ATOM 1382 HG LEU 89 5.281 1.108 -9.934 1.00 0.00

ATOM 1383 CD1 LEU 89 3.345 0.527 -9.189 1.00 0.00

ATOM 1384 HD11 LEU 89 2.931 0.027 -8.314 1.00 0.00

ATOM 1385 HD12 LEU 89 2.932 1.533 -9.262 1.00 0.00

ATOM 1386 HD13 LEU 89 3.087 -0.037 -10.085 1.00 0.00

ATOM 1387 CD2 LEU 89 5.226 1.401 -7.801 1.00 0.00

ATOM 1388 HD21 LEU 89 6.310 1.459 -7.705 1.00 0.00

ATOM 1389 HD22 LEU 89 4.814 2.407 -7.875 1.00 0.00

ATOM 1390 HD23 LEU 89 4.811 0.902 -6.925 1.00 0.00

ATOM 1391 C LEU 89 6.165 -1.020 -11.339 1.00 0.00

ATOM 1392 O LEU 89 5.982 0.055 -11.874 1.00 0.00

ATOM 1393 N GLN 90 7.188 -1.768 -11.652 1.00 0.00

ATOM 1394 H GLN 90 7.347 -2.664 -11.212 1.00 0.00

ATOM 1395 CA GLN 90 8.158 -1.307 -12.685 1.00 0.00

ATOM 1396 HA GLN 90 8.801 -0.526 -12.279 1.00 0.00

ATOM 1397 CB GLN 90 9.034 -2.483 -13.122 1.00 0.00

ATOM 1398 HB2 GLN 90 8.406 -3.352 -13.319 1.00 0.00

ATOM 1399 HB3 GLN 90 9.577 -2.216 -14.029 1.00 0.00

ATOM	1400	CG	GLN	90	10.032	-2.817	-12.010	1.00	0.00
ATOM	1401	HG2	GLN	90	10.604	-1.930	-11.737	1.00	0.00
ATOM	1402	HG3	GLN	90	9.510	-3.197	-11.132	1.00	0.00
ATOM	1403	CD	GLN	90	11.003	-3.891	-12.504	1.00	0.00
ATOM	1404	OE1	GLN	90	10.778	-4.505	-13.528	1.00	0.00
ATOM	1405	NE2	GLN	90	12.080	-4.147	-11.813	1.00	0.00
ATOM	1406	HE21	GLN	90	12.726	-4.855	-12.133	1.00	0.00
ATOM	1407	HE22	GLN	90	12.263	-3.637	-10.961	1.00	0.00
ATOM	1408	C	GLN	90	7.396	-0.764	-13.894	1.00	0.00
ATOM	1409	O	GLN	90	7.894	0.057	-14.638	1.00	0.00
ATOM	1410	N	ASP	91	6.188	-1.216	-14.098	1.00	0.00
ATOM	1411	H	ASP	91	5.767	-1.898	-13.483	1.00	0.00
ATOM	1412	CA	ASP	91	5.394	-0.727	-15.259	1.00	0.00
ATOM	1413	HA	ASP	91	5.953	-0.865	-16.185	1.00	0.00
ATOM	1414	CB	ASP	91	4.089	-1.518	-15.354	1.00	0.00
ATOM	1415	HB2	ASP	91	3.556	-1.455	-14.405	1.00	0.00
ATOM	1416	HB3	ASP	91	3.468	-1.102	-16.147	1.00	0.00
ATOM	1417	CG	ASP	91	4.401	-2.982	-15.666	1.00	0.00
ATOM	1418	OD1	ASP	91	5.531	-3.261	-16.033	1.00	0.00
ATOM	1419	OD2	ASP	91	3.506	-3.801	-15.534	1.00	0.00

ATOM 1420 C ASP 91 5.077 0.758 -15.070 1.00 0.00

ATOM 1421 O ASP 91 4.807 1.212 -13.975 1.00 0.00

ATOM 1422 N ARG 92 5.110 1.521 -16.129 1.00 0.00

ATOM 1423 H ARG 92 5.336 1.152 -17.042 1.00 0.00

ATOM 1424 CA ARG 92 4.811 2.976 -16.009 1.00 0.00

ATOM 1425 HA ARG 92 5.387 3.413 -15.193 1.00 0.00

ATOM 1426 CB ARG 92 5.190 3.685 -17.310 1.00 0.00

ATOM 1427 HB2 ARG 92 6.266 3.606 -17.466 1.00 0.00

ATOM 1428 HB3 ARG 92 4.668 3.216 -18.144 1.00 0.00

ATOM 1429 CG ARG 92 4.794 5.159 -17.224 1.00 0.00

ATOM 1430 HG2 ARG 92 3.751 5.237 -16.918 1.00 0.00

ATOM 1431 HG3 ARG 92 5.426 5.663 -16.493 1.00 0.00

ATOM 1432 CD ARG 92 4.974 5.817 -18.593 1.00 0.00

ATOM 1433 HD2 ARG 92 4.262 5.403 -19.307 1.00 0.00

ATOM 1434 HD3 ARG 92 4.826 6.894 -18.516 1.00 0.00

ATOM 1435 NE ARG 92 6.354 5.559 -19.091 1.00 0.00

ATOM 1436 HE ARG 92 6.952 4.870 -18.656 1.00 0.00

ATOM 1437 CZ ARG 92 6.815 6.227 -20.112 1.00 0.00

ATOM 1438 NH1 ARG 92 6.550 7.499 -20.232 1.00 0.00

ATOM 1439 HH11 ARG 92 5.988 7.962 -19.533 1.00 0.00

ATOM 1440 HH12 ARG 92 6.909 8.013 -21.024 1.00 0.00

ATOM 1441 NH2 ARG 92 7.543 5.624 -21.012 1.00 0.00

ATOM 1442 HH21 ARG 92 7.748 4.639 -20.915 1.00 0.00

ATOM 1443 HH22 ARG 92 7.899 6.144 -21.801 1.00 0.00

ATOM 1444 C ARG 92 3.317 3.169 -15.740 1.00 0.00

ATOM 1445 O ARG 92 2.884 4.219 -15.307 1.00 0.00

ATOM 1446 N GLN 93 2.524 2.165 -15.994 1.00 0.00

ATOM 1447 H GLN 93 2.876 1.289 -16.355 1.00 0.00

ATOM 1448 CA GLN 93 1.060 2.292 -15.754 1.00 0.00

ATOM 1449 HA GLN 93 0.703 3.263 -16.094 1.00 0.00

ATOM 1450 CB GLN 93 0.321 1.199 -16.532 1.00 0.00

ATOM 1451 HB2 GLN 93 0.681 1.178 -17.561 1.00 0.00

ATOM 1452 HB3 GLN 93 0.505 0.231 -16.064 1.00 0.00

ATOM 1453 CG GLN 93 -1.181 1.493 -16.523 1.00 0.00

ATOM 1454 HG2 GLN 93 -1.579 1.403 -15.512 1.00 0.00

ATOM 1455 HG3 GLN 93 -1.371 2.497 -16.900 1.00 0.00

ATOM 1456 CD GLN 93 -1.902 0.488 -17.424 1.00 0.00

ATOM 1457 OE1 GLN 93 -1.273 -0.250 -18.156 1.00 0.00

ATOM 1458 NE2 GLN 93 -3.205 0.427 -17.400 1.00 0.00

ATOM 1459 HE21 GLN 93 -3.687 -0.234 -17.992 1.00 0.00

```
ATOM   1460 HE22 GLN    93      -3.725   1.041 -16.790  1.00  0.00
ATOM   1461  C   GLN    93       0.769   2.140 -14.259  1.00  0.00
ATOM   1462  O   GLN    93       0.093   2.956 -13.664  1.00  0.00
ATOM   1463  N   MET    94       1.273   1.103 -13.648  1.00  0.00
ATOM   1464  H   MET    94       1.838   0.422 -14.136  1.00  0.00
ATOM   1465  CA  MET    94       1.023   0.900 -12.193  1.00  0.00
ATOM   1466  HA  MET    94      -0.042   0.756 -12.009  1.00  0.00
ATOM   1467  CB  MET    94       1.773  -0.343 -11.713  1.00  0.00
ATOM   1468  HB2 MET    94       2.802  -0.308 -12.073  1.00  0.00
ATOM   1469  HB3 MET    94       1.771  -0.370 -10.623  1.00  0.00
ATOM   1470  CG  MET    94       1.087  -1.598 -12.255  1.00  0.00
ATOM   1471  HG2 MET    94       0.767  -1.428 -13.283  1.00  0.00
ATOM   1472  HG3 MET    94       1.781  -2.439 -12.226  1.00  0.00
ATOM   1473  SD  MET    94      -0.359  -1.984 -11.237  1.00  0.00
ATOM   1474  CE  MET    94       0.490  -2.043  -9.639  1.00  0.00
ATOM   1475  HE1 MET    94       0.954  -1.078  -9.437  1.00  0.00
ATOM   1476  HE2 MET    94      -0.229  -2.272  -8.853  1.00  0.00
ATOM   1477  HE3 MET    94       1.258  -2.816  -9.663  1.00  0.00
ATOM   1478  C   MET    94       1.516   2.121 -11.412  1.00  0.00
ATOM   1479  O   MET    94       0.886   2.565 -10.474  1.00  0.00
```

ATOM 1480 N VAL 95 2.644 2.661 -11.788 1.00 0.00

ATOM 1481 H VAL 95 3.175 2.292 -12.564 1.00 0.00

ATOM 1482 CA VAL 95 3.183 3.849 -11.064 1.00 0.00

ATOM 1483 HA VAL 95 3.680 3.537 -10.145 1.00 0.00

ATOM 1484 CB VAL 95 4.201 4.568 -11.951 1.00 0.00

ATOM 1485 HB VAL 95 3.746 4.799 -12.914 1.00 0.00

ATOM 1486 CG1 VAL 95 4.643 5.865 -11.272 1.00 0.00

ATOM 1487 HG11 VAL 95 5.099 5.634 -10.309 1.00 0.00

ATOM 1488 HG12 VAL 95 5.369 6.377 -11.904 1.00 0.00

ATOM 1489 HG13 VAL 95 3.777 6.508 -11.118 1.00 0.00

ATOM 1490 CG2 VAL 95 5.418 3.665 -12.165 1.00 0.00

ATOM 1491 HG21 VAL 95 5.104 2.739 -12.649 1.00 0.00

ATOM 1492 HG22 VAL 95 6.144 4.176 -12.797 1.00 0.00

ATOM 1493 HG23 VAL 95 5.874 3.434 -11.202 1.00 0.00

ATOM 1494 C VAL 95 2.041 4.808 -10.717 1.00 0.00

ATOM 1495 O VAL 95 1.414 4.691 -9.683 1.00 0.00

ATOM 1496 N GLN 96 1.769 5.756 -11.570 1.00 0.00

ATOM 1497 H GLN 96 2.288 5.862 -12.430 1.00 0.00

ATOM 1498 CA GLN 96 0.670 6.721 -11.284 1.00 0.00

ATOM 1499 HA GLN 96 0.909 7.315 -10.402 1.00 0.00

ATOM 1500 CB GLN 96 0.495 7.664 -12.477 1.00 0.00

ATOM 1501 HB2 GLN 96 0.211 7.087 -13.357 1.00 0.00

ATOM 1502 HB3 GLN 96 -0.284 8.392 -12.253 1.00 0.00

ATOM 1503 CG GLN 96 1.812 8.393 -12.749 1.00 0.00

ATOM 1504 HG2 GLN 96 2.149 8.917 -11.854 1.00 0.00

ATOM 1505 HG3 GLN 96 2.578 7.683 -13.063 1.00 0.00

ATOM 1506 CD GLN 96 1.606 9.417 -13.867 1.00 0.00

ATOM 1507 OE1 GLN 96 0.886 10.382 -13.699 1.00 0.00

ATOM 1508 NE2 GLN 96 2.214 9.249 -15.010 1.00 0.00

ATOM 1509 HE21 GLN 96 2.083 9.921 -15.752 1.00 0.00

ATOM 1510 HE22 GLN 96 2.813 8.447 -15.147 1.00 0.00

ATOM 1511 C GLN 96 -0.631 5.959 -11.040 1.00 0.00

ATOM 1512 O GLN 96 -1.271 6.110 -10.018 1.00 0.00

ATOM 1513 N GLN 97 -1.027 5.142 -11.972 1.00 0.00

ATOM 1514 H GLN 97 -0.500 5.017 -12.824 1.00 0.00

ATOM 1515 CA GLN 97 -2.287 4.366 -11.801 1.00 0.00

ATOM 1516 HA GLN 97 -3.152 5.011 -11.951 1.00 0.00

ATOM 1517 CB GLN 97 -2.333 3.238 -12.834 1.00 0.00

ATOM 1518 HB2 GLN 97 -2.007 3.617 -13.803 1.00 0.00

ATOM 1519 HB3 GLN 97 -1.672 2.429 -12.521 1.00 0.00

ATOM 1520 CG GLN 97 -3.765 2.711 -12.948 1.00 0.00

ATOM 1521 HG2 GLN 97 -3.801 1.852 -13.619 1.00 0.00

ATOM 1522 HG3 GLN 97 -4.139 2.420 -11.966 1.00 0.00

ATOM 1523 CD GLN 97 -4.669 3.809 -13.510 1.00 0.00

ATOM 1524 OE1 GLN 97 -4.547 4.185 -14.659 1.00 0.00

ATOM 1525 NE2 GLN 97 -5.580 4.342 -12.742 1.00 0.00

ATOM 1526 HE21 GLN 97 -6.181 5.067 -13.106 1.00 0.00

ATOM 1527 HE22 GLN 97 -5.679 4.026 -11.788 1.00 0.00

ATOM 1528 C GLN 97 -2.338 3.774 -10.391 1.00 0.00

ATOM 1529 O GLN 97 -3.136 4.176 -9.567 1.00 0.00

ATOM 1530 N PHE 98 -1.491 2.823 -10.105 1.00 0.00

ATOM 1531 H PHE 98 -0.824 2.477 -10.780 1.00 0.00

ATOM 1532 CA PHE 98 -1.499 2.214 -8.744 1.00 0.00

ATOM 1533 HA PHE 98 -2.421 1.653 -8.592 1.00 0.00

ATOM 1534 CB PHE 98 -0.313 1.257 -8.588 1.00 0.00

ATOM 1535 HB2 PHE 98 -0.266 0.527 -9.397 1.00 0.00

ATOM 1536 HB3 PHE 98 0.638 1.786 -8.522 1.00 0.00

ATOM 1537 CG PHE 98 -0.461 0.470 -7.303 1.00 0.00

ATOM 1538 CD1 PHE 98 -1.661 0.518 -6.578 1.00 0.00

ATOM 1539 HD1 PHE 98 -2.491 1.123 -6.941 1.00 0.00

ATOM	1540	CE1	PHE	98	-1.792	-0.210	-5.391	1.00	0.00
ATOM	1541	HE1	PHE	98	-2.726	-0.172	-4.830	1.00	0.00
ATOM	1542	CZ	PHE	98	-0.727	-0.987	-4.923	1.00	0.00
ATOM	1543	HZ	PHE	98	-0.830	-1.554	-3.997	1.00	0.00
ATOM	1544	CE2	PHE	98	0.472	-1.037	-5.644	1.00	0.00
ATOM	1545	HE2	PHE	98	1.302	-1.643	-5.280	1.00	0.00
ATOM	1546	CD2	PHE	98	0.605	-0.309	-6.833	1.00	0.00
ATOM	1547	HD2	PHE	98	1.539	-0.348	-7.394	1.00	0.00
ATOM	1548	C	PHE	98	-1.400	3.323	-7.698	1.00	0.00
ATOM	1549	O	PHE	98	-2.030	3.271	-6.661	1.00	0.00
ATOM	1550	N	LYS	99	-0.615	4.329	-7.966	1.00	0.00
ATOM	1551	H	LYS	99	-0.089	4.377	-8.827	1.00	0.00
ATOM	1552	CA	LYS	99	-0.478	5.446	-6.991	1.00	0.00
ATOM	1553	HA	LYS	99	-0.184	5.060	-6.015	1.00	0.00
ATOM	1554	CB	LYS	99	0.597	6.420	-7.475	1.00	0.00
ATOM	1555	HB2	LYS	99	1.510	5.870	-7.703	1.00	0.00
ATOM	1556	HB3	LYS	99	0.248	6.930	-8.373	1.00	0.00
ATOM	1557	CG	LYS	99	0.882	7.451	-6.380	1.00	0.00
ATOM	1558	HG2	LYS	99	-0.045	7.952	-6.101	1.00	0.00
ATOM	1559	HG3	LYS	99	1.301	6.949	-5.507	1.00	0.00

```
ATOM 1560 CD LYS 99 1.882 8.484 -6.903 1.00 0.00
ATOM 1561 HD2 LYS 99 2.829 7.993 -7.127 1.00 0.00
ATOM 1562 HD3 LYS 99 1.489 8.944 -7.809 1.00 0.00
ATOM 1563 CE LYS 99 2.105 9.561 -5.839 1.00 0.00
ATOM 1564 HE2 LYS 99 1.925 10.544 -6.273 1.00 0.00
ATOM 1565 HE3 LYS 99 1.418 9.399 -5.008 1.00 0.00
ATOM 1566 NZ LYS 99 3.508 9.487 -5.344 1.00 0.00
ATOM 1567 HZ1 LYS 99 4.145 9.637 -6.114 1.00 0.00
ATOM 1568 HZ2 LYS 99 3.655 10.197 -4.642 1.00 0.00
ATOM 1569 HZ3 LYS 99 3.676 8.576 -4.941 1.00 0.00
ATOM 1570 C LYS 99 -1.816 6.178 -6.876 1.00 0.00
ATOM 1571 O LYS 99 -2.180 6.668 -5.826 1.00 0.00
ATOM 1572 N ARG 100 -2.553 6.249 -7.949 1.00 0.00
ATOM 1573 H ARG 100 -2.255 5.843 -8.825 1.00 0.00
ATOM 1574 CA ARG 100 -3.871 6.941 -7.903 1.00 0.00
ATOM 1575 HA ARG 100 -3.759 7.937 -7.475 1.00 0.00
ATOM 1576 CB ARG 100 -4.429 7.078 -9.321 1.00 0.00
ATOM 1577 HB2 ARG 100 -4.247 6.157 -9.875 1.00 0.00
ATOM 1578 HB3 ARG 100 -5.501 7.267 -9.274 1.00 0.00
ATOM 1579 CG ARG 100 -3.736 8.244 -10.029 1.00 0.00
```

ATOM 1580 HG2 ARG 100 -3.883 9.157 -9.453 1.00 0.00

ATOM 1581 HG3 ARG 100 -2.669 8.035 -10.113 1.00 0.00

ATOM 1582 CD ARG 100 -4.333 8.421 -11.427 1.00 0.00

ATOM 1583 HD2 ARG 100 -4.426 7.454 -11.923 1.00 0.00

ATOM 1584 HD3 ARG 100 -5.314 8.891 -11.362 1.00 0.00

ATOM 1585 NE ARG 100 -3.439 9.292 -12.242 1.00 0.00

ATOM 1586 HE ARG 100 -2.533 8.973 -12.555 1.00 0.00

ATOM 1587 CZ ARG 100 -3.821 10.496 -12.567 1.00 0.00

ATOM 1588 NH1 ARG 100 -3.195 11.533 -12.081 1.00 0.00

ATOM 1589 HH11 ARG 100 -2.415 11.399 -11.453 1.00 0.00

ATOM 1590 HH12 ARG 100 -3.493 12.463 -12.335 1.00 0.00

ATOM 1591 NH2 ARG 100 -4.829 10.664 -13.378 1.00 0.00

ATOM 1592 HH21 ARG 100 -5.311 9.859 -13.753 1.00 0.00

ATOM 1593 HH22 ARG 100 -5.122 11.597 -13.628 1.00 0.00

ATOM 1594 C ARG 100 -4.835 6.119 -7.049 1.00 0.00

ATOM 1595 O ARG 100 -5.436 6.618 -6.118 1.00 0.00

ATOM 1596 N GLU 101 -4.979 4.856 -7.348 1.00 0.00

ATOM 1597 H GLU 101 -4.480 4.430 -8.116 1.00 0.00

ATOM 1598 CA GLU 101 -5.894 4.002 -6.542 1.00 0.00

ATOM 1599 HA GLU 101 -6.908 4.398 -6.577 1.00 0.00

ATOM 1600 CB GLU 101 -5.904 2.582 -7.111 1.00 0.00

ATOM 1601 HB2 GLU 101 -4.883 2.202 -7.158 1.00 0.00

ATOM 1602 HB3 GLU 101 -6.501 1.937 -6.467 1.00 0.00

ATOM 1603 CG GLU 101 -6.507 2.597 -8.517 1.00 0.00

ATOM 1604 HG2 GLU 101 -5.836 3.125 -9.194 1.00 0.00

ATOM 1605 HG3 GLU 101 -6.643 1.573 -8.867 1.00 0.00

ATOM 1606 CD GLU 101 -7.861 3.309 -8.483 1.00 0.00

ATOM 1607 OE1 GLU 101 -8.571 3.145 -7.505 1.00 0.00

ATOM 1608 OE2 GLU 101 -8.166 4.006 -9.438 1.00 0.00

ATOM 1609 C GLU 101 -5.392 3.971 -5.100 1.00 0.00

ATOM 1610 O GLU 101 -6.161 3.987 -4.159 1.00 0.00

ATOM 1611 N LEU 102 -4.099 3.928 -4.921 1.00 0.00

ATOM 1612 H LEU 102 -3.457 3.912 -5.701 1.00 0.00

ATOM 1613 CA LEU 102 -3.536 3.899 -3.544 1.00 0.00

ATOM 1614 HA LEU 102 -3.839 2.987 -3.030 1.00 0.00

ATOM 1615 CB LEU 102 -2.008 3.933 -3.619 1.00 0.00

ATOM 1616 HB2 LEU 102 -1.695 3.875 -4.662 1.00 0.00

ATOM 1617 HB3 LEU 102 -1.645 4.863 -3.183 1.00 0.00

ATOM 1618 CG LEU 102 -1.426 2.746 -2.845 1.00 0.00

ATOM 1619 HG LEU 102 -1.509 2.935 -1.775 1.00 0.00

ATOM 1620 CD1 LEU 102 -2.197 1.472 -3.199 1.00 0.00

ATOM 1621 HD11 LEU 102 -2.113 1.281 -4.269 1.00 0.00

ATOM 1622 HD12 LEU 102 -1.780 0.629 -2.647 1.00 0.00

ATOM 1623 HD13 LEU 102 -3.247 1.596 -2.934 1.00 0.00

ATOM 1624 CD2 LEU 102 0.047 2.569 -3.219 1.00 0.00

ATOM 1625 HD21 LEU 102 0.598 3.474 -2.965 1.00 0.00

ATOM 1626 HD22 LEU 102 0.463 1.724 -2.669 1.00 0.00

ATOM 1627 HD23 LEU 102 0.131 2.381 -4.290 1.00 0.00

ATOM 1628 C LEU 102 -4.037 5.114 -2.762 1.00 0.00

ATOM 1629 O LEU 102 -4.326 5.030 -1.587 1.00 0.00

ATOM 1630 N MET 103 -4.149 6.244 -3.407 1.00 0.00

ATOM 1631 H MET 103 -3.915 6.325 -4.386 1.00 0.00

ATOM 1632 CA MET 103 -4.638 7.457 -2.694 1.00 0.00

ATOM 1633 HA MET 103 -4.005 7.669 -1.832 1.00 0.00

ATOM 1634 CB MET 103 -4.596 8.659 -3.639 1.00 0.00

ATOM 1635 HB2 MET 103 -5.048 8.388 -4.593 1.00 0.00

ATOM 1636 HB3 MET 103 -5.148 9.488 -3.198 1.00 0.00

ATOM 1637 CG MET 103 -3.141 9.078 -3.865 1.00 0.00

ATOM 1638 HG2 MET 103 -2.662 9.279 -2.907 1.00 0.00

ATOM 1639 HG3 MET 103 -2.603 8.281 -4.379 1.00 0.00

ATOM	1640	SD	MET	103	-3.096	10.577	-4.879	1.00	0.00
ATOM	1641	CE	MET	103	-3.355	11.765	-3.540	1.00	0.00
ATOM	1642	HE1	MET	103	-2.549	11.673	-2.811	1.00	0.00
ATOM	1643	HE2	MET	103	-3.362	12.776	-3.947	1.00	0.00
ATOM	1644	HE3	MET	103	-4.309	11.562	-3.053	1.00	0.00
ATOM	1645	C	MET	103	-6.077	7.219	-2.234	1.00	0.00
ATOM	1646	O	MET	103	-6.506	7.722	-1.215	1.00	0.00
ATOM	1647	N	SER	104	-6.824	6.449	-2.978	1.00	0.00
ATOM	1648	H	SER	104	-6.471	6.027	-3.825	1.00	0.00
ATOM	1649	CA	SER	104	-8.233	6.172	-2.586	1.00	0.00
ATOM	1650	HA	SER	104	-8.697	7.071	-2.183	1.00	0.00
ATOM	1651	CB	SER	104	-9.025	5.722	-3.815	1.00	0.00
ATOM	1652	HB2	SER	104	-10.066	5.559	-3.538	1.00	0.00
ATOM	1653	HB3	SER	104	-8.970	6.491	-4.585	1.00	0.00
ATOM	1654	OG	SER	104	-8.474	4.512	-4.315	1.00	0.00
ATOM	1655	HG	SER	104	-8.970	4.229	-5.086	1.00	0.00
ATOM	1656	C	SER	104	-8.257	5.065	-1.530	1.00	0.00
ATOM	1657	O	SER	104	-9.115	5.031	-0.671	1.00	0.00
ATOM	1658	N	LEU	105	-7.323	4.155	-1.591	1.00	0.00
ATOM	1659	H	LEU	105	-6.612	4.175	-2.308	1.00	0.00

ATOM 1660 CA LEU 105 -7.291 3.047 -0.596 1.00 0.00

ATOM 1661 HA LEU 105 -8.039 2.294 -0.845 1.00 0.00

ATOM 1662 CB LEU 105 -5.910 2.386 -0.608 1.00 0.00

ATOM 1663 HB2 LEU 105 -5.248 2.950 -1.265 1.00 0.00

ATOM 1664 HB3 LEU 105 -5.500 2.380 0.402 1.00 0.00

ATOM 1665 CG LEU 105 -6.030 0.947 -1.118 1.00 0.00

ATOM 1666 HG LEU 105 -5.092 0.420 -0.941 1.00 0.00

ATOM 1667 CD1 LEU 105 -7.164 0.231 -0.380 1.00 0.00

ATOM 1668 HD11 LEU 105 -8.102 0.756 -0.558 1.00 0.00

ATOM 1669 HD12 LEU 105 -7.246 -0.793 -0.745 1.00 0.00

ATOM 1670 HD13 LEU 105 -6.951 0.218 0.689 1.00 0.00

ATOM 1671 CD2 LEU 105 -6.326 0.961 -2.618 1.00 0.00

ATOM 1672 HD21 LEU 105 -5.516 1.466 -3.144 1.00 0.00

ATOM 1673 HD22 LEU 105 -6.411 -0.063 -2.982 1.00 0.00

ATOM 1674 HD23 LEU 105 -7.262 1.489 -2.799 1.00 0.00

ATOM 1675 C LEU 105 -7.583 3.592 0.804 1.00 0.00

ATOM 1676 O LEU 105 -8.426 3.077 1.511 1.00 0.00

ATOM 1677 N PRO 106 -6.875 4.658 1.215 1.00 0.00

ATOM 1678 CD PRO 106 -5.839 5.356 0.433 1.00 0.00

ATOM 1679 HD2 PRO 106 -6.189 5.509 -0.588 1.00 0.00

ATOM 1680 HD3 PRO 106 -4.926 4.760 0.417 1.00 0.00

ATOM 1681 CG PRO 106 -5.668 6.646 1.185 1.00 0.00

ATOM 1682 HG2 PRO 106 -6.355 7.394 0.789 1.00 0.00

ATOM 1683 HG3 PRO 106 -4.643 7.000 1.075 1.00 0.00

ATOM 1684 CB PRO 106 -5.971 6.328 2.608 1.00 0.00

ATOM 1685 HB2 PRO 106 -6.357 7.230 3.082 1.00 0.00

ATOM 1686 HB3 PRO 106 -5.126 5.948 3.184 1.00 0.00

ATOM 1687 CA PRO 106 -7.070 5.268 2.534 1.00 0.00

ATOM 1688 HA PRO 106 -6.972 4.493 3.294 1.00 0.00

ATOM 1689 C PRO 106 -8.449 5.917 2.644 1.00 0.00

ATOM 1690 O PRO 106 -8.854 6.376 3.694 1.00 0.00

ATOM 1691 N GLN 107 -9.178 5.947 1.563 1.00 0.00

ATOM 1692 H GLN 107 -8.841 5.565 0.690 1.00 0.00

ATOM 1693 CA GLN 107 -10.536 6.551 1.591 1.00 0.00

ATOM 1694 HA GLN 107 -10.636 7.213 2.451 1.00 0.00

ATOM 1695 CB GLN 107 -10.762 7.367 0.317 1.00 0.00

ATOM 1696 HB2 GLN 107 -9.979 8.119 0.223 1.00 0.00

ATOM 1697 HB3 GLN 107 -10.734 6.705 -0.549 1.00 0.00

ATOM 1698 CG GLN 107 -12.126 8.057 0.388 1.00 0.00

ATOM 1699 HG2 GLN 107 -12.918 7.316 0.498 1.00 0.00

ATOM	1700	HG3	GLN	107	-12.157	8.748	1.230	1.00	0.00
ATOM	1701	CD	GLN	107	-12.368	8.844	-0.901	1.00	0.00
ATOM	1702	OE1	GLN	107	-11.481	8.976	-1.722	1.00	0.00
ATOM	1703	NE2	GLN	107	-13.539	9.378	-1.116	1.00	0.00
ATOM	1704	HE21	GLN	107	-13.705	9.898	-1.965	1.00	0.00
ATOM	1705	HE22	GLN	107	-14.274	9.268	-0.432	1.00	0.00
ATOM	1706	C	GLN	107	-11.576	5.436	1.677	1.00	0.00
ATOM	1707	O	GLN	107	-12.479	5.475	2.488	1.00	0.00
ATOM	1708	N	GLN	108	-11.458	4.441	0.841	1.00	0.00
ATOM	1709	H	GLN	108	-10.711	4.404	0.162	1.00	0.00
ATOM	1710	CA	GLN	108	-12.440	3.321	0.872	1.00	0.00
ATOM	1711	HA	GLN	108	-13.405	3.652	0.489	1.00	0.00
ATOM	1712	CB	GLN	108	-11.933	2.175	-0.006	1.00	0.00
ATOM	1713	HB2	GLN	108	-11.733	2.547	-1.011	1.00	0.00
ATOM	1714	HB3	GLN	108	-11.015	1.769	0.420	1.00	0.00
ATOM	1715	CG	GLN	108	-12.994	1.075	-0.072	1.00	0.00
ATOM	1716	HG2	GLN	108	-13.274	0.756	0.932	1.00	0.00
ATOM	1717	HG3	GLN	108	-13.878	1.434	-0.599	1.00	0.00
ATOM	1718	CD	GLN	108	-12.430	-0.130	-0.828	1.00	0.00
ATOM	1719	OE1	GLN	108	-11.521	-0.785	-0.360	1.00	0.00

ATOM 1720 NE2 GLN 108 -12.937 -0.453 -1.987 1.00 0.00

ATOM 1721 HE21 GLN 108 -12.570 -1.248 -2.492 1.00 0.00

ATOM 1722 HE22 GLN 108 -13.694 0.092 -2.373 1.00 0.00

ATOM 1723 C GLN 108 -12.607 2.827 2.309 1.00 0.00

ATOM 1724 O GLN 108 -13.651 2.336 2.691 1.00 0.00

ATOM 1725 N CYX 109 -11.586 2.953 3.111 1.00 0.00

ATOM 1726 H CYX 109 -10.714 3.358 2.801 1.00 0.00

ATOM 1727 CA CYX 109 -11.686 2.490 4.524 1.00 0.00

ATOM 1728 HA CYX 109 -12.729 2.326 4.794 1.00 0.00

ATOM 1729 CB CYX 109 -10.925 1.170 4.690 1.00 0.00

ATOM 1730 HB2 CYX 109 -10.477 1.130 5.683 1.00 0.00

ATOM 1731 HB3 CYX 109 -11.614 0.334 4.569 1.00 0.00

ATOM 1732 SG CYX 109 -9.583 1.077 3.476 1.00 0.00

ATOM 1733 C CYX 109 -11.081 3.549 5.450 1.00 0.00

ATOM 1734 O CYX 109 -10.611 3.246 6.529 1.00 0.00

ATOM 1735 N ASN 110 -11.093 4.786 5.037 1.00 0.00

ATOM 1736 H ASN 110 -11.483 5.041 4.141 1.00 0.00

ATOM 1737 CA ASN 110 -10.522 5.865 5.892 1.00 0.00

ATOM 1738 HA ASN 110 -10.334 6.759 5.297 1.00 0.00

ATOM 1739 CB ASN 110 -11.515 6.216 7.002 1.00 0.00

ATOM 1740 HB2 ASN 110 -11.611 5.383 7.699 1.00 0.00

ATOM 1741 HB3 ASN 110 -11.182 7.103 7.540 1.00 0.00

ATOM 1742 CG ASN 110 -12.887 6.502 6.389 1.00 0.00

ATOM 1743 OD1 ASN 110 -12.993 7.227 5.420 1.00 0.00

ATOM 1744 ND2 ASN 110 -13.950 5.959 6.917 1.00 0.00

ATOM 1745 HD21 ASN 110 -14.859 6.143 6.517 1.00 0.00

ATOM 1746 HD22 ASN 110 -13.858 5.356 7.723 1.00 0.00

ATOM 1747 C ASN 110 -9.211 5.385 6.516 1.00 0.00

ATOM 1748 O ASN 110 -8.971 5.563 7.694 1.00 0.00

ATOM 1749 N PHE 111 -8.358 4.776 5.736 1.00 0.00

ATOM 1750 H PHE 111 -8.548 4.619 4.756 1.00 0.00

ATOM 1751 CA PHE 111 -7.064 4.289 6.290 1.00 0.00

ATOM 1752 HA PHE 111 -7.234 3.722 7.205 1.00 0.00

ATOM 1753 CB PHE 111 -6.383 3.376 5.269 1.00 0.00

ATOM 1754 HB2 PHE 111 -7.094 2.939 4.568 1.00 0.00

ATOM 1755 HB3 PHE 111 -5.598 3.891 4.716 1.00 0.00

ATOM 1756 CG PHE 111 -5.721 2.224 5.987 1.00 0.00

ATOM 1757 CD1 PHE 111 -6.476 1.104 6.357 1.00 0.00

ATOM 1758 HD1 PHE 111 -7.540 1.062 6.126 1.00 0.00

ATOM 1759 CE1 PHE 111 -5.862 0.037 7.024 1.00 0.00

ATOM	1760	HE1	PHE	111	-6.450	-0.834	7.314	1.00	0.00
ATOM	1761	CZ	PHE	111	-4.494	0.088	7.319	1.00	0.00
ATOM	1762	HZ	PHE	111	-4.016	-0.744	7.837	1.00	0.00
ATOM	1763	CE2	PHE	111	-3.740	1.208	6.948	1.00	0.00
ATOM	1764	HE2	PHE	111	-2.675	1.249	7.178	1.00	0.00
ATOM	1765	CD2	PHE	111	-4.354	2.275	6.282	1.00	0.00
ATOM	1766	HD2	PHE	111	-3.767	3.146	5.993	1.00	0.00
ATOM	1767	C	PHE	111	-6.157	5.482	6.594	1.00	0.00
ATOM	1768	O	PHE	111	-5.071	5.331	7.116	1.00	0.00
ATOM	1769	N	ARG	112	-6.594	6.670	6.273	1.00	0.00
ATOM	1770	H	ARG	112	-7.496	6.805	5.840	1.00	0.00
ATOM	1771	CA	ARG	112	-5.754	7.870	6.547	1.00	0.00
ATOM	1772	HA	ARG	112	-4.903	7.899	5.866	1.00	0.00
ATOM	1773	CB	ARG	112	-6.589	9.136	6.341	1.00	0.00
ATOM	1774	HB2	ARG	112	-7.474	9.095	6.976	1.00	0.00
ATOM	1775	HB3	ARG	112	-5.994	10.010	6.604	1.00	0.00
ATOM	1776	CG	ARG	112	-7.017	9.232	4.874	1.00	0.00
ATOM	1777	HG2	ARG	112	-6.135	9.199	4.234	1.00	0.00
ATOM	1778	HG3	ARG	112	-7.673	8.396	4.630	1.00	0.00
ATOM	1779	CD	ARG	112	-7.763	10.548	4.647	1.00	0.00

ATOM 1780 HD2 ARG 112 -7.114 11.393 4.876 1.00 0.00

ATOM 1781 HD3 ARG 112 -8.098 10.618 3.612 1.00 0.00

ATOM 1782 NE ARG 112 -8.954 10.604 5.540 1.00 0.00

ATOM 1783 HE ARG 112 -9.652 9.873 5.535 1.00 0.00

ATOM 1784 CZ ARG 112 -9.114 11.613 6.353 1.00 0.00

ATOM 1785 NH1 ARG 112 -8.665 11.541 7.576 1.00 0.00

ATOM 1786 HH11 ARG 112 -8.194 10.704 7.890 1.00 0.00

ATOM 1787 HH12 ARG 112 -8.790 12.322 8.203 1.00 0.00

ATOM 1788 NH2 ARG 112 -9.722 12.691 5.943 1.00 0.00

ATOM 1789 HH21 ARG 112 -10.068 12.742 4.996 1.00 0.00

ATOM 1790 HH22 ARG 112 -9.844 13.469 6.575 1.00 0.00

ATOM 1791 C ARG 112 -5.255 7.819 7.992 1.00 0.00

ATOM 1792 O ARG 112 -5.868 7.216 8.850 1.00 0.00

ATOM 1793 N ALA 113 -4.145 8.449 8.270 1.00 0.00

ATOM 1794 H ALA 113 -3.628 8.953 7.564 1.00 0.00

ATOM 1795 CA ALA 113 -3.610 8.434 9.660 1.00 0.00

ATOM 1796 HA ALA 113 -4.377 8.166 10.387 1.00 0.00

ATOM 1797 CB ALA 113 -2.470 7.419 9.757 1.00 0.00

ATOM 1798 HB1 ALA 113 -1.690 7.678 9.041 1.00 0.00

ATOM 1799 HB2 ALA 113 -2.056 7.431 10.765 1.00 0.00

ATOM 1800 HB3 ALA 113 -2.850 6.422 9.533 1.00 0.00

ATOM 1801 C ALA 113 -3.086 9.825 10.021 1.00 0.00

ATOM 1802 O ALA 113 -2.866 10.657 9.163 1.00 0.00

ATOM 1803 N PRO 114 -2.883 10.076 11.323 1.00 0.00

ATOM 1804 CD PRO 114 -3.126 9.122 12.418 1.00 0.00

ATOM 1805 HD2 PRO 114 -2.662 8.165 12.176 1.00 0.00

ATOM 1806 HD3 PRO 114 -4.199 8.982 12.546 1.00 0.00

ATOM 1807 CG PRO 114 -2.489 9.788 13.604 1.00 0.00

ATOM 1808 HG2 PRO 114 -1.465 9.430 13.713 1.00 0.00

ATOM 1809 HG3 PRO 114 -3.056 9.545 14.503 1.00 0.00

ATOM 1810 CB PRO 114 -2.527 11.254 13.329 1.00 0.00

ATOM 1811 HB2 PRO 114 -1.669 11.715 13.818 1.00 0.00

ATOM 1812 HB3 PRO 114 -3.440 11.752 13.653 1.00 0.00

ATOM 1813 CA PRO 114 -2.382 11.366 11.812 1.00 0.00

ATOM 1814 HA PRO 114 -2.986 12.158 11.371 1.00 0.00

ATOM 1815 C PRO 114 -0.912 11.580 11.442 1.00 0.00

ATOM 1816 O PRO 114 -0.382 12.666 11.565 1.00 0.00

ATOM 1817 N GLN 115 -0.250 10.551 10.987 1.00 0.00

ATOM 1818 H GLN 115 -0.685 9.645 10.882 1.00 0.00

ATOM 1819 CA GLN 115 1.183 10.694 10.607 1.00 0.00

ATOM	1820	HA	GLN	115	1.598	11.608	11.031	1.00	0.00
ATOM	1821	CB	GLN	115	1.975	9.501	11.147	1.00	0.00
ATOM	1822	HB2	GLN	115	1.613	8.583	10.683	1.00	0.00
ATOM	1823	HB3	GLN	115	3.032	9.631	10.915	1.00	0.00
ATOM	1824	CG	GLN	115	1.793	9.411	12.664	1.00	0.00
ATOM	1825	HG2	GLN	115	0.745	9.239	12.909	1.00	0.00
ATOM	1826	HG3	GLN	115	2.398	8.601	13.072	1.00	0.00
ATOM	1827	CD	GLN	115	2.237	10.725	13.309	1.00	0.00
ATOM	1828	OE1	GLN	115	3.332	11.194	13.066	1.00	0.00
ATOM	1829	NE2	GLN	115	1.430	11.342	14.127	1.00	0.00
ATOM	1830	HE21	GLN	115	1.718	12.210	14.556	1.00	0.00
ATOM	1831	HE22	GLN	115	0.522	10.948	14.327	1.00	0.00
ATOM	1832	C	GLN	115	1.304	10.739	9.083	1.00	0.00
ATOM	1833	O	GLN	115	0.492	10.182	8.369	1.00	0.00
ATOM	1834	N	ARG	116	2.310	11.396	8.577	1.00	0.00
ATOM	1835	H	ARG	116	2.988	11.861	9.163	1.00	0.00
ATOM	1836	CA	ARG	116	2.481	11.475	7.099	1.00	0.00
ATOM	1837	HA	ARG	116	1.655	10.972	6.595	1.00	0.00
ATOM	1838	CB	ARG	116	2.499	12.942	6.665	1.00	0.00
ATOM	1839	HB2	ARG	116	3.307	13.463	7.178	1.00	0.00

ATOM 1840 HB3 ARG 116 2.656 13.001 5.588 1.00 0.00

ATOM 1841 CG ARG 116 1.162 13.595 7.022 1.00 0.00

ATOM 1842 HG2 ARG 116 0.351 13.050 6.539 1.00 0.00

ATOM 1843 HG3 ARG 116 1.023 13.570 8.103 1.00 0.00

ATOM 1844 CD ARG 116 1.159 15.047 6.540 1.00 0.00

ATOM 1845 HD2 ARG 116 2.073 15.551 6.854 1.00 0.00

ATOM 1846 HD3 ARG 116 1.080 15.084 5.453 1.00 0.00

ATOM 1847 NE ARG 116 -0.008 15.763 7.129 1.00 0.00

ATOM 1848 HE ARG 116 0.086 16.333 7.957 1.00 0.00

ATOM 1849 CZ ARG 116 -1.183 15.663 6.572 1.00 0.00

ATOM 1850 NH1 ARG 116 -1.400 14.751 5.664 1.00 0.00

ATOM 1851 HH11 ARG 116 -0.655 14.123 5.395 1.00 0.00

ATOM 1852 HH12 ARG 116 -2.311 14.676 5.235 1.00 0.00

ATOM 1853 NH2 ARG 116 -2.144 16.473 6.925 1.00 0.00

ATOM 1854 HH21 ARG 116 -1.974 17.176 7.630 1.00 0.00

ATOM 1855 HH22 ARG 116 -3.052 16.392 6.492 1.00 0.00

ATOM 1856 C ARG 116 3.799 10.809 6.700 1.00 0.00

ATOM 1857 O ARG 116 4.744 10.777 7.462 1.00 0.00

ATOM 1858 N CYX 117 3.869 10.274 5.512 1.00 0.00

ATOM 1859 H CYX 117 3.087 10.296 4.873 1.00 0.00

```
ATOM   1860  CA  CYX   117       5.125   9.610   5.067  1.00  0.00
ATOM   1861  HA  CYX   117       5.856   9.602   5.876  1.00  0.00
ATOM   1862  CB  CYX   117       4.824   8.164   4.666  1.00  0.00
ATOM   1863  HB2 CYX   117       4.051   8.152   3.898  1.00  0.00
ATOM   1864  HB3 CYX   117       5.729   7.698   4.276  1.00  0.00
ATOM   1865  SG  CYX   117       4.194   7.259   6.101  1.00  0.00
ATOM   1866  C   CYX   117       5.701  10.361   3.865  1.00  0.00
ATOM   1867  O   CYX   117       5.004  11.079   3.177  1.00  0.00
ATOM   1868  N   ASP   118       6.971  10.200   3.606  1.00  0.00
ATOM   1869  H   ASP   118       7.558   9.605   4.173  1.00  0.00
ATOM   1870  CA  ASP   118       7.589  10.905   2.448  1.00  0.00
ATOM   1871  HA  ASP   118       7.296  11.954   2.444  1.00  0.00
ATOM   1872  CB  ASP   118       9.113  10.824   2.556  1.00  0.00
ATOM   1873  HB2 ASP   118       9.424   9.779   2.568  1.00  0.00
ATOM   1874  HB3 ASP   118       9.564  11.326   1.700  1.00  0.00
ATOM   1875  CG  ASP   118       9.570  11.506   3.847  1.00  0.00
ATOM   1876  OD1 ASP   118       8.776  12.231   4.424  1.00  0.00
ATOM   1877  OD2 ASP   118      10.706  11.290   4.238  1.00  0.00
ATOM   1878  C   ASP   118       7.133  10.243   1.146  1.00  0.00
ATOM   1879  O   ASP   118       6.920   9.049   1.088  1.00  0.00
```

ATOM 1880 N LEU 119 6.979 11.011 0.102 1.00 0.00

ATOM 1881 H LEU 119 7.154 12.004 0.143 1.00 0.00

ATOM 1882 CA LEU 119 6.534 10.425 -1.193 1.00 0.00

ATOM 1883 HA LEU 119 5.722 9.717 -1.029 1.00 0.00

ATOM 1884 CB LEU 119 6.032 11.542 -2.110 1.00 0.00

ATOM 1885 HB2 LEU 119 6.733 12.376 -2.083 1.00 0.00

ATOM 1886 HB3 LEU 119 5.951 11.168 -3.131 1.00 0.00

ATOM 1887 CG LEU 119 4.658 12.015 -1.630 1.00 0.00

ATOM 1888 HG LEU 119 4.051 11.151 -1.358 1.00 0.00

ATOM 1889 CD1 LEU 119 4.829 12.923 -0.411 1.00 0.00

ATOM 1890 HD11 LEU 119 5.436 13.786 -0.683 1.00 0.00

ATOM 1891 HD12 LEU 119 3.851 13.260 -0.069 1.00 0.00

ATOM 1892 HD13 LEU 119 5.323 12.370 0.388 1.00 0.00

ATOM 1893 CD2 LEU 119 3.963 12.790 -2.751 1.00 0.00

ATOM 1894 HD21 LEU 119 3.841 12.142 -3.619 1.00 0.00

ATOM 1895 HD22 LEU 119 2.985 13.126 -2.408 1.00 0.00

ATOM 1896 HD23 LEU 119 4.568 13.654 -3.025 1.00 0.00

ATOM 1897 C LEU 119 7.708 9.706 -1.861 1.00 0.00

ATOM 1898 O LEU 119 7.527 8.860 -2.713 1.00 0.00

ATOM 1899 N ASP 120 8.913 10.034 -1.479 1.00 0.00

ATOM 1900 H ASP 120 9.074 10.737 -0.772 1.00 0.00

ATOM 1901 CA ASP 120 10.095 9.366 -2.093 1.00 0.00

ATOM 1902 HA ASP 120 10.039 9.423 -3.180 1.00 0.00

ATOM 1903 CB ASP 120 11.375 10.066 -1.632 1.00 0.00

ATOM 1904 HB2 ASP 120 12.238 9.594 -2.102 1.00 0.00

ATOM 1905 HB3 ASP 120 11.337 11.117 -1.916 1.00 0.00

ATOM 1906 CG ASP 120 11.497 9.953 -0.111 1.00 0.00

ATOM 1907 OD1 ASP 120 10.487 9.709 0.529 1.00 0.00

ATOM 1908 OD2 ASP 120 12.599 10.112 0.388 1.00 0.00

ATOM 1909 C ASP 120 10.132 7.898 -1.664 1.00 0.00

ATOM 1910 O ASP 120 10.591 7.040 -2.391 1.00 0.00

ATOM 1911 N VAL 121 9.654 7.603 -0.486 1.00 0.00

ATOM 1912 H VAL 121 9.272 8.310 0.124 1.00 0.00

ATOM 1913 CA VAL 121 9.663 6.190 -0.011 1.00 0.00

ATOM 1914 HA VAL 121 10.688 5.837 0.109 1.00 0.00

ATOM 1915 CB VAL 121 8.955 6.104 1.343 1.00 0.00

ATOM 1916 HB VAL 121 7.994 6.614 1.284 1.00 0.00

ATOM 1917 CG1 VAL 121 8.730 4.635 1.709 1.00 0.00

ATOM 1918 HG11 VAL 121 9.691 4.123 1.769 1.00 0.00

ATOM 1919 HG12 VAL 121 8.226 4.573 2.673 1.00 0.00

ATOM 1920 HG13 VAL 121 8.113 4.160 0.946 1.00 0.00

ATOM 1921 CG2 VAL 121 9.822 6.769 2.414 1.00 0.00

ATOM 1922 HG21 VAL 121 9.983 7.815 2.154 1.00 0.00

ATOM 1923 HG22 VAL 121 9.319 6.708 3.379 1.00 0.00

ATOM 1924 HG23 VAL 121 10.783 6.257 2.473 1.00 0.00

ATOM 1925 C VAL 121 8.933 5.306 -1.025 1.00 0.00

ATOM 1926 O VAL 121 9.406 4.251 -1.397 1.00 0.00

ATOM 1927 N SER 122 7.785 5.729 -1.476 1.00 0.00

ATOM 1928 H SER 122 7.386 6.605 -1.171 1.00 0.00

ATOM 1929 CA SER 122 7.026 4.913 -2.466 1.00 0.00

ATOM 1930 HA SER 122 6.768 3.944 -2.038 1.00 0.00

ATOM 1931 CB SER 122 5.734 5.640 -2.845 1.00 0.00

ATOM 1932 HB2 SER 122 5.977 6.571 -3.357 1.00 0.00

ATOM 1933 HB3 SER 122 5.141 5.007 -3.505 1.00 0.00

ATOM 1934 OG SER 122 4.990 5.926 -1.670 1.00 0.00

ATOM 1935 HG SER 122 4.180 6.382 -1.908 1.00 0.00

ATOM 1936 C SER 122 7.880 4.705 -3.718 1.00 0.00

ATOM 1937 O SER 122 7.828 3.671 -4.353 1.00 0.00

ATOM 1938 N GLY 123 8.667 5.682 -4.080 1.00 0.00

ATOM 1939 H GLY 123 8.716 6.545 -3.558 1.00 0.00

ATOM 1940 CA GLY 123 9.523 5.539 -5.291 1.00 0.00

ATOM 1941 HA2 GLY 123 9.033 4.891 -6.018 1.00 0.00

ATOM 1942 HA3 GLY 123 9.701 6.517 -5.738 1.00 0.00

ATOM 1943 C GLY 123 10.865 4.920 -4.898 1.00 0.00

ATOM 1944 O GLY 123 11.810 4.923 -5.662 1.00 0.00

ATOM 1945 N GLY 124 10.957 4.385 -3.710 1.00 0.00

ATOM 1946 H GLY 124 10.177 4.377 -3.069 1.00 0.00

ATOM 1947 CA GLY 124 12.238 3.765 -3.270 1.00 0.00

ATOM 1948 HA2 GLY 124 13.046 4.494 -3.334 1.00 0.00

ATOM 1949 HA3 GLY 124 12.147 3.412 -2.242 1.00 0.00

ATOM 1950 C GLY 124 12.568 2.577 -4.176 1.00 0.00

ATOM 1951 O GLY 124 12.579 2.690 -5.385 1.00 0.00

ATOM 1952 N ARG 125 12.837 1.436 -3.600 1.00 0.00

ATOM 1953 H ARG 125 12.830 1.332 -2.595 1.00 0.00

ATOM 1954 CA ARG 125 13.164 0.243 -4.430 1.00 0.00

ATOM 1955 HA ARG 125 14.038 0.441 -5.051 1.00 0.00

ATOM 1956 CB ARG 125 13.474 -0.945 -3.515 1.00 0.00

ATOM 1957 HB2 ARG 125 12.649 -1.094 -2.819 1.00 0.00

ATOM 1958 HB3 ARG 125 13.607 -1.844 -4.117 1.00 0.00

ATOM 1959 CG ARG 125 14.757 -0.662 -2.732 1.00 0.00

ATOM 1960 HG2 ARG 125 15.556 -0.399 -3.425 1.00 0.00

ATOM 1961 HG3 ARG 125 14.588 0.165 -2.043 1.00 0.00

ATOM 1962 CD ARG 125 15.158 -1.910 -1.942 1.00 0.00

ATOM 1963 HD2 ARG 125 14.305 -2.295 -1.384 1.00 0.00

ATOM 1964 HD3 ARG 125 15.528 -2.682 -2.617 1.00 0.00

ATOM 1965 NE ARG 125 16.240 -1.560 -0.979 1.00 0.00

ATOM 1966 HE ARG 125 16.174 -0.748 -0.382 1.00 0.00

ATOM 1967 CZ ARG 125 17.304 -2.310 -0.893 1.00 0.00

ATOM 1968 NH1 ARG 125 17.906 -2.716 -1.978 1.00 0.00

ATOM 1969 HH11 ARG 125 17.546 -2.448 -2.883 1.00 0.00

ATOM 1970 HH12 ARG 125 18.730 -3.297 -1.908 1.00 0.00

ATOM 1971 NH2 ARG 125 17.767 -2.655 0.277 1.00 0.00

ATOM 1972 HH21 ARG 125 17.300 -2.340 1.115 1.00 0.00

ATOM 1973 HH22 ARG 125 18.591 -3.236 0.339 1.00 0.00

ATOM 1974 C ARG 125 11.969 -0.099 -5.322 1.00 0.00

ATOM 1975 O ARG 125 12.125 -0.586 -6.426 1.00 0.00

ATOM 1976 N CYX 126 10.778 0.152 -4.856 1.00 0.00

ATOM 1977 H CYX 126 10.642 0.556 -3.941 1.00 0.00

ATOM 1978 CA CYX 126 9.574 -0.158 -5.678 1.00 0.00

ATOM 1979 HA CYX 126 9.551 -1.218 -5.931 1.00 0.00

ATOM 1980 CB CYX 126 8.313 0.182 -4.882 1.00 0.00

ATOM 1981 HB2 CYX 126 8.378 1.207 -4.519 1.00 0.00

ATOM 1982 HB3 CYX 126 7.439 0.078 -5.524 1.00 0.00

ATOM 1983 SG CYX 126 8.197 -0.907 -3.442 1.00 0.00

ATOM 1984 C CYX 126 9.608 0.674 -6.962 1.00 0.00

ATOM 1985 O CYX 126 9.002 0.324 -7.956 1.00 0.00

ATOM 1986 N SER 127 10.313 1.771 -6.950 1.00 0.00

ATOM 1987 H SER 127 10.821 2.065 -6.128 1.00 0.00

ATOM 1988 CA SER 127 10.387 2.624 -8.169 1.00 0.00

ATOM 1989 HA SER 127 11.060 3.465 -8.002 1.00 0.00

ATOM 1990 CB SER 127 10.921 1.796 -9.340 1.00 0.00

ATOM 1991 HB2 SER 127 12.001 1.923 -9.413 1.00 0.00

ATOM 1992 HB3 SER 127 10.691 0.743 -9.176 1.00 0.00

ATOM 1993 OG SER 127 10.311 2.231 -10.546 1.00 0.00

ATOM 1994 HG SER 127 10.647 1.711 -11.280 1.00 0.00

ATOM 1995 C SER 127 8.989 3.146 -8.512 1.00 0.00

ATOM 1996 O SER 127 8.040 2.684 -7.901 1.00 0.00

ATOM 1997 OXT SER 127 8.893 4.001 -9.377 1.00 0.00

TER

ATOM 1998 Cl1 LIG 128 -1.688 -19.925 -10.373 1.00 0.00

ATOM 1999 Cl2 LIG 128 -1.582 -21.743 -8.056 1.00 0.00

ATOM 2000 P1 LIG 128 1.706 -18.960 -6.425 1.00 0.00

ATOM 2001 O1 LIG 128 1.427 -19.025 -4.856 1.00 0.00

ATOM 2002 O2 LIG 128 2.899 -19.988 -6.599 1.00 0.00

ATOM 2003 O3 LIG 128 0.375 -19.744 -6.996 1.00 0.00

ATOM 2004 O4 LIG 128 1.920 -17.652 -7.061 1.00 0.00

ATOM 2005 C1 LIG 128 0.393 -18.224 -4.240 1.00 0.00

ATOM 2006 H1 LIG 128 0.513 -18.358 -3.167 1.00 0.00

ATOM 2007 H2 LIG 128 -0.590 -18.581 -4.553 1.00 0.00

ATOM 2008 H3 LIG 128 0.521 -17.174 -4.503 1.00 0.00

ATOM 2009 C2 LIG 128 2.890 -21.326 -6.049 1.00 0.00

ATOM 2010 H4 LIG 128 2.023 -21.879 -6.414 1.00 0.00

ATOM 2011 H5 LIG 128 2.881 -21.280 -4.960 1.00 0.00

ATOM 2012 H6 LIG 128 3.807 -21.794 -6.400 1.00 0.00

ATOM 2013 C3 LIG 128 -0.127 -19.475 -8.243 1.00 0.00

ATOM 2014 H7 LIG 128 0.216 -18.568 -8.726 1.00 0.00

ATOM 2015 C4 LIG 128 -1.016 -20.288 -8.810 1.00 0.00

TER

ATOM 2016 Cl1 LIG 129 -30.035 10.684 8.969 1.00 0.00

ATOM 2017 Cl2 LIG 129 -29.917 13.575 9.532 1.00 0.00

ATOM 2018 P1 LIG 129 -26.028 13.573 7.063 1.00 0.00

ATOM 2019 O1 LIG 129 -26.100 15.001 6.357 1.00 0.00

ATOM 2020 O2 LIG 129 -25.054 13.848 8.283 1.00 0.00

ATOM 2021 O3 LIG 129 -27.535 13.493 7.725 1.00 0.00

ATOM 2022 O4 LIG 129 -25.691 12.392 6.256 1.00 0.00

ATOM 2023 C1 LIG 129 -26.897 15.217 5.170 1.00 0.00

ATOM 2024 H1 LIG 129 -26.657 16.222 4.829 1.00 0.00

ATOM 2025 H2 LIG 129 -27.957 15.150 5.416 1.00 0.00

ATOM 2026 H3 LIG 129 -26.635 14.489 4.402 1.00 0.00

ATOM 2027 C2 LIG 129 -25.208 14.958 9.197 1.00 0.00

ATOM 2028 H4 LIG 129 -26.195 14.932 9.661 1.00 0.00

ATOM 2029 H5 LIG 129 -25.063 15.899 8.666 1.00 0.00

ATOM 2030 H6 LIG 129 -24.436 14.827 9.951 1.00 0.00

ATOM 2031 C3 LIG 129 -28.148 12.287 7.946 1.00 0.00

ATOM 2032 H7 LIG 129 -27.730 11.423 7.447 1.00 0.00

ATOM 2033 C4 LIG 129 -29.227 12.205 8.723 1.00 0.00

TER

ATOM 2034 Cl1 LIG 130 17.497 -15.178 23.042 1.00 0.00

ATOM 2035 Cl2 LIG 130 20.159 -14.691 24.209 1.00 0.00

ATOM 2036 P1 LIG 130 17.959 -12.160 27.367 1.00 0.00

ATOM 2037 O1 LIG 130 18.839 -12.441 28.668 1.00 0.00

ATOM 2038 O2 LIG 130 18.516 -10.759 26.878 1.00 0.00

ATOM 2039 O3 LIG 130 18.603 -13.266 26.329 1.00 0.00

ATOM 2040 O4 LIG 130 16.495 -12.245 27.462 1.00 0.00

ATOM 2041 C1 LIG 130 18.663 -13.642 29.454 1.00 0.00

ATOM 2042 H1 LIG 130 19.280 -13.511 30.340 1.00 0.00

ATOM 2043 H2 LIG 130 19.001 -14.511 28.887 1.00 0.00

ATOM 2044 H3 LIG 130 17.616 -13.756 29.740 1.00 0.00

ATOM 2045 C2 LIG 130 19.924 -10.468 26.720 1.00 0.00

ATOM 2046 H4 LIG 130 20.382 -11.178 26.031 1.00 0.00

ATOM 2047 H5 LIG 130 20.422 -10.504 27.690 1.00 0.00

ATOM 2048 H6 LIG 130 19.971 -9.462 26.309 1.00 0.00

ATOM 2049 C3 LIG 130 17.858 -13.797 25.307 1.00 0.00

ATOM 2050 H7 LIG 130 16.785 -13.665 25.361 1.00 0.00

ATOM 2051 C4 LIG 130 18.443 -14.465 24.315 1.00 0.00

TER

ATOM 2052 Cl1 LIG 131 -9.807 -11.525 23.140 1.00 0.00

ATOM 2053 Cl2 LIG 131 -11.347 -9.229 24.162 1.00 0.00

ATOM 2054 P1 LIG 131 -8.073 -8.876 27.383 1.00 0.00

ATOM 2055 O1 LIG 131 -8.000 -7.295 27.588 1.00 0.00

ATOM 2056 O2 LIG 131 -8.982 -9.332 28.598 1.00 0.00

ATOM 2057 O3 LIG 131 -9.051 -8.958 26.059 1.00 0.00

ATOM 2058 O4 LIG 131 -6.826 -9.636 27.225 1.00 0.00

ATOM 2059 C1 LIG 131 -7.252 -6.451 26.683 1.00 0.00

ATOM 2060 H1 LIG 131 -7.257 -5.459 27.130 1.00 0.00

ATOM 2061 H2 LIG 131 -7.742 -6.424 25.709 1.00 0.00

ATOM 2062 H3 LIG 131 -6.228 -6.813 26.588 1.00 0.00

ATOM 2063 C2 LIG 131 -10.248 -8.714 28.929 1.00 0.00

ATOM 2064 H4 LIG 131 -10.925 -8.757 28.075 1.00 0.00

ATOM 2065 H5 LIG 131 -10.087 -7.679 29.234 1.00 0.00

ATOM 2066 H6 LIG 131 -10.651 -9.293 29.756 1.00 0.00

ATOM 2067 C3 LIG 131 -8.970 -10.009 25.183 1.00 0.00

ATOM 2068 H7 LIG 131 -8.089 -10.635 25.245 1.00 0.00

ATOM 2069 C4 LIG 131 -9.929 -10.220 24.283 1.00 0.00

TER

ATOM 2070 Cl1 LIG 132 15.954 16.326 3.519 1.00 0.00

ATOM 2071 Cl2 LIG 132 17.202 17.794 1.288 1.00 0.00

ATOM 2072 P1 LIG 132 20.175 19.392 4.422 1.00 0.00

ATOM 2073 O1 LIG 132 21.517 19.487 3.566 1.00 0.00

ATOM 2074 O2 LIG 132 19.654 20.889 4.401 1.00 0.00

```
ATOM   2075  O3  LIG   132     19.205  18.584   3.364  1.00  0.00
ATOM   2076  O4  LIG   132     20.217  18.771   5.753  1.00  0.00
ATOM   2077  C1  LIG   132     22.342  18.324   3.323  1.00  0.00
ATOM   2078  H1  LIG   132     23.245  18.694   2.843  1.00  0.00
ATOM   2079  H2  LIG   132     21.822  17.632   2.659  1.00  0.00
ATOM   2080  H3  LIG   132     22.592  17.835   4.266  1.00  0.00
ATOM   2081  C2  LIG   132     19.538  21.680   3.195  1.00  0.00
ATOM   2082  H4  LIG   132     18.894  21.176   2.473  1.00  0.00
ATOM   2083  H5  LIG   132     20.527  21.852   2.768  1.00  0.00
ATOM   2084  H6  LIG   132     19.091  22.622   3.504  1.00  0.00
ATOM   2085  C3  LIG   132     18.177  17.792   3.807  1.00  0.00
ATOM   2086  H7  LIG   132     18.185  17.516   4.854  1.00  0.00
ATOM   2087  C4  LIG   132     17.234  17.368   2.969  1.00  0.00
TER
ATOM   2088  Cl1 LIG   133     27.650 -11.363  25.200  1.00  0.00
ATOM   2089  Cl2 LIG   133     28.681 -10.228  27.717  1.00  0.00
ATOM   2090  P1  LIG   133     28.343  -6.133  25.635  1.00  0.00
ATOM   2091  O1  LIG   133     27.937  -5.189  26.856  1.00  0.00
ATOM   2092  O2  LIG   133     29.902  -5.876  25.514  1.00  0.00
ATOM   2093  O3  LIG   133     28.204  -7.613  26.345  1.00  0.00
```

```
ATOM   2094  O4  LIG   133      27.599  -6.029  24.372  1.00  0.00
ATOM   2095  C1  LIG   133      26.571  -5.106  27.323  1.00  0.00
ATOM   2096  H1  LIG   133      26.556  -4.302  28.056  1.00  0.00
ATOM   2097  H2  LIG   133      26.281  -6.046  27.794  1.00  0.00
ATOM   2098  H3  LIG   133      25.902  -4.870  26.495  1.00  0.00
ATOM   2099  C2  LIG   133      30.813  -5.928  26.637  1.00  0.00
ATOM   2100  H4  LIG   133      30.753  -6.900  27.128  1.00  0.00
ATOM   2101  H5  LIG   133      30.577  -5.130  27.343  1.00  0.00
ATOM   2102  H6  LIG   133      31.805  -5.780  26.218  1.00  0.00
ATOM   2103  C3  LIG   133      27.908  -8.729  25.607  1.00  0.00
ATOM   2104  H7  LIG   133      27.522  -8.569  24.607  1.00  0.00
ATOM   2105  C4  LIG   133      28.068  -9.947  26.119  1.00  0.00
TER
ATOM   2106  Cl1 LIG   134      15.646 -19.854  10.645  1.00  0.00
ATOM   2107  Cl2 LIG   134      18.194 -19.074   9.386  1.00  0.00
ATOM   2108  P1  LIG   134      19.847 -18.755  13.674  1.00  0.00
ATOM   2109  O1  LIG   134      21.354 -19.235  13.469  1.00  0.00
ATOM   2110  O2  LIG   134      19.994 -17.177  13.681  1.00  0.00
ATOM   2111  O3  LIG   134      19.215 -19.122  12.197  1.00  0.00
ATOM   2112  O4  LIG   134      19.065 -19.308  14.788  1.00  0.00
```

```
ATOM   2113  C1  LIG   134      21.697 -20.638  13.402  1.00  0.00
ATOM   2114  H1  LIG   134      22.784 -20.679  13.399  1.00  0.00
ATOM   2115  H2  LIG   134      21.304 -21.074  12.482  1.00  0.00
ATOM   2116  H3  LIG   134      21.305 -21.165  14.273  1.00  0.00
ATOM   2117  C2  LIG   134      20.725 -16.435  12.677  1.00  0.00
ATOM   2118  H4  LIG   134      20.327 -16.649  11.684  1.00  0.00
ATOM   2119  H5  LIG   134      21.784 -16.691  12.725  1.00  0.00
ATOM   2120  H6  LIG   134      20.578 -15.386  12.922  1.00  0.00
ATOM   2121  C3  LIG   134      17.885 -19.415  12.048  1.00  0.00
ATOM   2122  H7  LIG   134      17.328 -19.646  12.948  1.00  0.00
ATOM   2123  C4  LIG   134      17.323 -19.437  10.841  1.00  0.00
TER
ATOM   2124  Cl1 LIG   135     -30.211 -17.289  -6.315  1.00  0.00
ATOM   2125  Cl2 LIG   135     -29.125 -20.020  -6.098  1.00  0.00
ATOM   2126  P1  LIG   135     -29.638 -19.520  -1.548  1.00  0.00
ATOM   2127  O1  LIG   135     -30.027 -21.004  -1.108  1.00  0.00
ATOM   2128  O2  LIG   135     -28.091 -19.463  -1.209  1.00  0.00
ATOM   2129  O3  LIG   135     -29.715 -19.667  -3.187  1.00  0.00
ATOM   2130  O4  LIG   135     -30.427 -18.392  -1.034  1.00  0.00
ATOM   2131  C1  LIG   135     -31.378 -21.501  -1.243  1.00  0.00
```

ATOM 2132 H1 LIG 135 -31.390 -22.470 -0.748 1.00 0.00

ATOM 2133 H2 LIG 135 -31.628 -21.618 -2.298 1.00 0.00

ATOM 2134 H3 LIG 135 -32.081 -20.824 -0.755 1.00 0.00

ATOM 2135 C2 LIG 135 -27.142 -20.475 -1.619 1.00 0.00

ATOM 2136 H4 LIG 135 -27.161 -20.593 -2.703 1.00 0.00

ATOM 2137 H5 LIG 135 -27.375 -21.421 -1.129 1.00 0.00

ATOM 2138 H6 LIG 135 -26.169 -20.112 -1.298 1.00 0.00

ATOM 2139 C3 LIG 135 -30.007 -18.590 -3.982 1.00 0.00

ATOM 2140 H7 LIG 135 -30.430 -17.720 -3.496 1.00 0.00

ATOM 2141 C4 LIG 135 -29.799 -18.638 -5.297 1.00 0.00

TER

ATOM 2142 Cl1 LIG 136 19.674 6.315 -19.134 1.00 0.00

ATOM 2143 Cl2 LIG 136 18.384 8.905 -19.691 1.00 0.00

ATOM 2144 P1 LIG 136 20.448 8.687 -23.804 1.00 0.00

ATOM 2145 O1 LIG 136 19.360 9.381 -24.742 1.00 0.00

ATOM 2146 O2 LIG 136 21.471 9.869 -23.542 1.00 0.00

ATOM 2147 O3 LIG 136 19.587 8.545 -22.406 1.00 0.00

ATOM 2148 O4 LIG 136 21.060 7.423 -24.233 1.00 0.00

ATOM 2149 C1 LIG 136 18.214 8.651 -25.237 1.00 0.00

ATOM 2150 H1 LIG 136 17.725 9.312 -25.949 1.00 0.00

ATOM 2151 H2 LIG 136 17.536 8.420 -24.414 1.00 0.00

ATOM 2152 H3 LIG 136 18.536 7.737 -25.736 1.00 0.00

ATOM 2153 C2 LIG 136 21.074 11.193 -23.116 1.00 0.00

ATOM 2154 H4 LIG 136 20.506 11.138 -22.186 1.00 0.00

ATOM 2155 H5 LIG 136 20.479 11.671 -23.896 1.00 0.00

ATOM 2156 H6 LIG 136 22.000 11.741 -22.958 1.00 0.00

ATOM 2157 C3 LIG 136 19.860 7.550 -21.503 1.00 0.00

ATOM 2158 H7 LIG 136 20.472 6.726 -21.849 1.00 0.00

ATOM 2159 C4 LIG 136 19.366 7.598 -20.268 1.00 0.00

TER

ATOM 2160 Cl1 LIG 137 -30.648 13.136 -29.998 1.00 0.00

ATOM 2161 Cl2 LIG 137 -28.878 11.265 -28.565 1.00 0.00

ATOM 2162 P1 LIG 137 -29.440 14.026 -24.921 1.00 0.00

ATOM 2163 O1 LIG 137 -29.419 12.975 -23.720 1.00 0.00

ATOM 2164 O2 LIG 137 -27.958 14.588 -24.904 1.00 0.00

ATOM 2165 O3 LIG 137 -29.498 13.002 -26.210 1.00 0.00

ATOM 2166 O4 LIG 137 -30.491 15.053 -24.954 1.00 0.00

ATOM 2167 C1 LIG 137 -30.592 12.208 -23.367 1.00 0.00

ATOM 2168 H1 LIG 137 -30.341 11.686 -22.446 1.00 0.00

ATOM 2169 H2 LIG 137 -30.818 11.488 -24.155 1.00 0.00

ATOM 2170 H3 LIG 137 -31.441 12.873 -23.201 1.00 0.00

ATOM 2171 C2 LIG 137 -26.782 13.746 -24.869 1.00 0.00

ATOM 2172 H4 LIG 137 -26.784 13.058 -25.716 1.00 0.00

ATOM 2173 H5 LIG 137 -26.750 13.192 -23.930 1.00 0.00

ATOM 2174 H6 LIG 137 -25.934 14.423 -24.938 1.00 0.00

ATOM 2175 C3 LIG 137 -30.075 13.384 -27.393 1.00 0.00

ATOM 2176 H7 LIG 137 -30.706 14.265 -27.372 1.00 0.00

ATOM 2177 C4 LIG 137 -29.881 12.678 -28.505 1.00 0.00

TER

END

Table B: The number of atoms present in the system of AH-Nap

ATOM 1 N GLY 1 24.719 -9.990 -2.261 1.00 0.00

ATOM 2 H1 GLY 1 24.018 -9.275 -2.385 1.00 0.00

ATOM 3 H2 GLY 1 24.334 -10.877 -2.553 1.00 0.00

ATOM 4 H3 GLY 1 25.590 -9.664 -2.656 1.00 0.00

ATOM 5 CA GLY 1 24.979 -10.135 -0.801 1.00 0.00

ATOM 6 HA2 GLY 1 24.747 -11.151 -0.480 1.00 0.00

ATOM 7 HA3 GLY 1 26.025 -9.917 -0.585 1.00 0.00

ATOM 8 C GLY 1 24.095 -9.154 -0.027 1.00 0.00

ATOM 9 O GLY 1 24.566 -8.165 0.500 1.00 0.00

ATOM	10	N	PRO	2	22.788	-9.439	0.039	1.00	0.00
ATOM	11	CD	PRO	2	22.145	-10.615	-0.573	1.00	0.00
ATOM	12	HD2	PRO	2	22.706	-11.513	-0.312	1.00	0.00
ATOM	13	HD3	PRO	2	22.131	-10.499	-1.657	1.00	0.00
ATOM	14	CG	PRO	2	20.766	-10.602	0.022	1.00	0.00
ATOM	15	HG2	PRO	2	20.750	-11.236	0.909	1.00	0.00
ATOM	16	HG3	PRO	2	20.054	-10.984	-0.709	1.00	0.00
ATOM	17	CB	PRO	2	20.470	-9.181	0.362	1.00	0.00
ATOM	18	HB2	PRO	2	19.801	-9.167	1.222	1.00	0.00
ATOM	19	HB3	PRO	2	20.029	-8.604	-0.451	1.00	0.00
ATOM	20	CA	PRO	2	21.824	-8.587	0.748	1.00	0.00
ATOM	21	HA	PRO	2	21.963	-7.560	0.412	1.00	0.00
ATOM	22	C	PRO	2	22.013	-8.657	2.266	1.00	0.00
ATOM	23	O	PRO	2	21.568	-7.794	2.997	1.00	0.00
ATOM	24	N	MET	3	22.669	-9.678	2.745	1.00	0.00
ATOM	25	H	MET	3	23.042	-10.400	2.144	1.00	0.00
ATOM	26	CA	MET	3	22.884	-9.801	4.215	1.00	0.00
ATOM	27	HA	MET	3	21.928	-9.889	4.731	1.00	0.00
ATOM	28	CB	MET	3	23.713	-11.054	4.505	1.00	0.00
ATOM	29	HB2	MET	3	24.646	-11.012	3.942	1.00	0.00
ATOM	30	HB3	MET	3	23.935	-11.106	5.571	1.00	0.00
ATOM	31	CG	MET	3	22.922	-12.296	4.088	1.00	0.00

ATOM	32	HG2	MET	3	22.735	-12.271	3.014	1.00	0.00
ATOM	33	HG3	MET	3	23.488	-13.194	4.337	1.00	0.00
ATOM	34	SD	MET	3	21.340	-12.327	4.967	1.00	0.00
ATOM	35	CE	MET	3	22.014	-12.405	6.645	1.00	0.00
ATOM	36	HE1	MET	3	22.631	-11.526	6.831	1.00	0.00
ATOM	37	HE2	MET	3	21.196	-12.433	7.365	1.00	0.00
ATOM	38	HE3	MET	3	22.622	-13.304	6.752	1.00	0.00
ATOM	39	C	MET	3	23.628	-8.566	4.726	1.00	0.00
ATOM	40	O	MET	3	23.382	-8.088	5.816	1.00	0.00
ATOM	41	N	ARG	4	24.538	-8.045	3.948	1.00	0.00
ATOM	42	H	ARG	4	24.750	-8.438	3.042	1.00	0.00
ATOM	43	CA	ARG	4	25.295	-6.841	4.390	1.00	0.00
ATOM	44	HA	ARG	4	25.879	-7.069	5.282	1.00	0.00
ATOM	45	CB	ARG	4	26.250	-6.401	3.279	1.00	0.00
ATOM	46	HB2	ARG	4	25.687	-6.234	2.361	1.00	0.00
ATOM	47	HB3	ARG	4	26.748	-5.477	3.573	1.00	0.00
ATOM	48	CG	ARG	4	27.297	-7.491	3.043	1.00	0.00
ATOM	49	HG2	ARG	4	27.802	-7.720	3.981	1.00	0.00
ATOM	50	HG3	ARG	4	26.809	-8.390	2.666	1.00	0.00
ATOM	51	CD	ARG	4	28.322	-7.000	2.019	1.00	0.00
ATOM	52	HD2	ARG	4	28.844	-6.122	2.399	1.00	0.00
ATOM	53	HD3	ARG	4	29.045	-7.787	1.802	1.00	0.00

ATOM	54	NE	ARG	4	27.625	-6.631	0.756	1.00	0.00
ATOM	55	HE	ARG	4	27.278	-7.336	0.121	1.00	0.00
ATOM	56	CZ	ARG	4	27.451	-5.374	0.448	1.00	0.00
ATOM	57	NH1	ARG	4	28.450	-4.538	0.532	1.00	0.00
ATOM	58	HH11	ARG	4	29.356	-4.867	0.835	1.00	0.00
ATOM	59	HH12	ARG	4	28.313	-3.567	0.293	1.00	0.00
ATOM	60	NH2	ARG	4	26.280	-4.953	0.057	1.00	0.00
ATOM	61	HH21	ARG	4	25.509	-5.603	-0.007	1.00	0.00
ATOM	62	HH22	ARG	4	26.150	-3.980	-0.180	1.00	0.00
ATOM	63	C	ARG	4	24.316	-5.707	4.698	1.00	0.00
ATOM	64	O	ARG	4	24.511	-4.937	5.617	1.00	0.00
ATOM	65	N	ARG	5	23.262	-5.598	3.935	1.00	0.00
ATOM	66	H	ARG	5	23.094	-6.234	3.168	1.00	0.00
ATOM	67	CA	ARG	5	22.271	-4.514	4.185	1.00	0.00
ATOM	68	HA	ARG	5	22.743	-3.538	4.078	1.00	0.00
ATOM	69	CB	ARG	5	21.132	-4.621	3.168	1.00	0.00
ATOM	70	HB2	ARG	5	20.680	-5.611	3.231	1.00	0.00
ATOM	71	HB3	ARG	5	20.379	-3.863	3.384	1.00	0.00
ATOM	72	CG	ARG	5	21.685	-4.403	1.758	1.00	0.00
ATOM	73	HG2	ARG	5	22.197	-3.442	1.712	1.00	0.00
ATOM	74	HG3	ARG	5	22.388	-5.201	1.515	1.00	0.00
ATOM	75	CD	ARG	5	20.533	-4.415	0.752	1.00	0.00

ATOM 76 HD2 ARG 5 19.935 -5.318 0.874 1.00 0.00

ATOM 77 HD3 ARG 5 19.901 -3.539 0.892 1.00 0.00

ATOM 78 NE ARG 5 21.083 -4.391 -0.633 1.00 0.00

ATOM 79 HE ARG 5 21.610 -3.602 -0.978 1.00 0.00

ATOM 80 CZ ARG 5 20.888 -5.406 -1.430 1.00 0.00

ATOM 81 NH1 ARG 5 19.687 -5.893 -1.586 1.00 0.00

ATOM 82 HH11 ARG 5 18.910 -5.482 -1.088 1.00 0.00

ATOM 83 HH12 ARG 5 19.540 -6.679 -2.204 1.00 0.00

ATOM 84 NH2 ARG 5 21.894 -5.934 -2.071 1.00 0.00

ATOM 85 HH21 ARG 5 22.822 -5.555 -1.948 1.00 0.00

ATOM 86 HH22 ARG 5 21.740 -6.720 -2.687 1.00 0.00

ATOM 87 C ARG 5 21.707 -4.652 5.600 1.00 0.00

ATOM 88 O ARG 5 21.355 -3.678 6.235 1.00 0.00

ATOM 89 N GLU 6 21.617 -5.855 6.098 1.00 0.00

ATOM 90 H GLU 6 21.907 -6.670 5.575 1.00 0.00

ATOM 91 CA GLU 6 21.075 -6.053 7.472 1.00 0.00

ATOM 92 HA GLU 6 20.051 -5.685 7.531 1.00 0.00

ATOM 93 CB GLU 6 21.079 -7.545 7.813 1.00 0.00

ATOM 94 HB2 GLU 6 22.090 -7.940 7.708 1.00 0.00

ATOM 95 HB3 GLU 6 20.740 -7.685 8.840 1.00 0.00

ATOM 96 CG GLU 6 20.140 -8.288 6.861 1.00 0.00

ATOM 97 HG2 GLU 6 19.135 -7.875 6.942 1.00 0.00

ATOM	98	HG3	GLU	6	20.497	-8.175	5.837	1.00	0.00
ATOM	99	CD	GLU	6	20.112	-9.772	7.229	1.00	0.00
ATOM	100	OE1	GLU	6	20.910	-10.173	8.060	1.00	0.00
ATOM	101	OE2	GLU	6	19.290	-10.484	6.674	1.00	0.00
ATOM	102	C	GLU	6	21.947	-5.297	8.477	1.00	0.00
ATOM	103	O	GLU	6	21.472	-4.820	9.489	1.00	0.00
ATOM	104	N	ARG	7	23.218	-5.183	8.207	1.00	0.00
ATOM	105	H	ARG	7	23.620	-5.578	7.368	1.00	0.00
ATOM	106	CA	ARG	7	24.117	-4.457	9.147	1.00	0.00
ATOM	107	HA	ARG	7	24.219	-5.014	10.078	1.00	0.00
ATOM	108	CB	ARG	7	25.502	-4.304	8.515	1.00	0.00
ATOM	109	HB2	ARG	7	25.873	-5.283	8.211	1.00	0.00
ATOM	110	HB3	ARG	7	25.434	-3.655	7.642	1.00	0.00
ATOM	111	CG	ARG	7	26.463	-3.689	9.534	1.00	0.00
ATOM	112	HG2	ARG	7	26.075	-2.726	9.865	1.00	0.00
ATOM	113	HG3	ARG	7	26.560	-4.356	10.391	1.00	0.00
ATOM	114	CD	ARG	7	27.835	-3.490	8.886	1.00	0.00
ATOM	115	HD2	ARG	7	28.197	-4.431	8.471	1.00	0.00
ATOM	116	HD3	ARG	7	27.774	-2.744	8.094	1.00	0.00
ATOM	117	NE	ARG	7	28.805	-3.017	9.914	1.00	0.00
ATOM	118	HE	ARG	7	29.233	-3.656	10.570	1.00	0.00
ATOM	119	CZ	ARG	7	29.119	-1.752	9.981	1.00	0.00

ATOM 120 NH1 ARG 7 28.528 -0.979 10.850 1.00 0.00
ATOM 121 HH11 ARG 7 27.829 -1.363 11.469 1.00 0.00
ATOM 122 HH12 ARG 7 28.773 -0.001 10.900 1.00 0.00
ATOM 123 NH2 ARG 7 30.024 -1.261 9.179 1.00 0.00
ATOM 124 HH21 ARG 7 30.480 -1.863 8.508 1.00 0.00
ATOM 125 HH22 ARG 7 30.265 -0.282 9.233 1.00 0.00
ATOM 126 C ARG 7 23.536 -3.073 9.442 1.00 0.00
ATOM 127 O ARG 7 23.557 -2.604 10.563 1.00 0.00
ATOM 128 N GLY 8 23.015 -2.414 8.443 1.00 0.00
ATOM 129 H GLY 8 22.993 -2.797 7.509 1.00 0.00
ATOM 130 CA GLY 8 22.433 -1.060 8.666 1.00 0.00
ATOM 131 HA2 GLY 8 23.209 -0.372 9.000 1.00 0.00
ATOM 132 HA3 GLY 8 21.991 -0.688 7.742 1.00 0.00
ATOM 133 C GLY 8 21.346 -1.144 9.739 1.00 0.00
ATOM 134 O GLY 8 21.132 -0.215 10.492 1.00 0.00
ATOM 135 N ARG 9 20.655 -2.249 9.814 1.00 0.00
ATOM 136 H ARG 9 20.825 -3.025 9.190 1.00 0.00
ATOM 137 CA ARG 9 19.583 -2.389 10.840 1.00 0.00
ATOM 138 HA ARG 9 18.783 -1.674 10.651 1.00 0.00
ATOM 139 CB ARG 9 19.001 -3.802 10.779 1.00 0.00
ATOM 140 HB2 ARG 9 18.638 -4.004 9.771 1.00 0.00
ATOM 141 HB3 ARG 9 19.774 -4.526 11.037 1.00 0.00

ATOM	142	CG	ARG	9	17.842	-3.918	11.772	1.00	0.00
ATOM	143	HG2	ARG	9	18.197	-3.680	12.775	1.00	0.00
ATOM	144	HG3	ARG	9	17.052	-3.222	11.491	1.00	0.00
ATOM	145	CD	ARG	9	17.294	-5.347	11.752	1.00	0.00
ATOM	146	HD2	ARG	9	17.011	-5.629	10.738	1.00	0.00
ATOM	147	HD3	ARG	9	18.044	-6.045	12.126	1.00	0.00
ATOM	148	NE	ARG	9	16.089	-5.429	12.625	1.00	0.00
ATOM	149	HE	ARG	9	15.236	-4.941	12.395	1.00	0.00
ATOM	150	CZ	ARG	9	16.122	-6.145	13.715	1.00	0.00
ATOM	151	NH1	ARG	9	16.596	-7.360	13.685	1.00	0.00
ATOM	152	HH11	ARG	9	16.937	-7.745	12.815	1.00	0.00
ATOM	153	HH12	ARG	9	16.620	-7.911	14.531	1.00	0.00
ATOM	154	NH2	ARG	9	15.681	-5.644	14.837	1.00	0.00
ATOM	155	HH21	ARG	9	15.316	-4.703	14.857	1.00	0.00
ATOM	156	HH22	ARG	9	15.708	-6.200	15.680	1.00	0.00
ATOM	157	C	ARG	9	20.173	-2.138	12.229	1.00	0.00
ATOM	158	O	ARG	9	19.524	-1.593	13.100	1.00	0.00
ATOM	159	N	GLN	10	21.399	-2.533	12.444	1.00	0.00
ATOM	160	H	GLN	10	21.945	-2.987	11.725	1.00	0.00
ATOM	161	CA	GLN	10	22.028	-2.317	13.777	1.00	0.00
ATOM	162	HA	GLN	10	21.513	-2.903	14.539	1.00	0.00
ATOM	163	CB	GLN	10	23.491	-2.761	13.726	1.00	0.00

ATOM	164	HB2	GLN	10	23.998	-2.251	12.907	1.00	0.00
ATOM	165	HB3	GLN	10	23.981	-2.511	14.667	1.00	0.00
ATOM	166	CG	GLN	10	23.558	-4.274	13.504	1.00	0.00
ATOM	167	HG2	GLN	10	22.978	-4.794	14.267	1.00	0.00
ATOM	168	HG3	GLN	10	23.171	-4.530	12.517	1.00	0.00
ATOM	169	CD	GLN	10	25.012	-4.739	13.594	1.00	0.00
ATOM	170	OE1	GLN	10	25.673	-4.991	12.497	1.00	0.00
ATOM	171	NE2	GLN	10	25.553	-4.875	14.673	1.00	0.00
ATOM	172	HE21	GLN	10	26.514	-5.183	14.717	1.00	0.00
ATOM	173	HE22	GLN	10	25.039	-4.680	15.520	1.00	0.00
ATOM	174	C	GLN	10	21.958	-0.832	14.139	1.00	0.00
ATOM	175	O	GLN	10	21.800	-0.471	15.288	1.00	0.00
ATOM	176	N	GLY	11	22.075	0.031	13.168	1.00	0.00
ATOM	177	H	GLY	11	22.207	-0.263	12.211	1.00	0.00
ATOM	178	CA	GLY	11	22.016	1.492	13.457	1.00	0.00
ATOM	179	HA2	GLY	11	22.886	1.791	14.042	1.00	0.00
ATOM	180	HA3	GLY	11	21.994	2.055	12.524	1.00	0.00
ATOM	181	C	GLY	11	20.748	1.802	14.256	1.00	0.00
ATOM	182	O	GLY	11	20.741	2.657	15.118	1.00	0.00
ATOM	183	N	ASP	12	19.676	1.113	13.975	1.00	0.00
ATOM	184	H	ASP	12	19.674	0.401	13.258	1.00	0.00
ATOM	185	CA	ASP	12	18.411	1.370	14.720	1.00	0.00

ATOM	186	HA	ASP	12	17.601	0.767	14.308	1.00	0.00
ATOM	187	CB	ASP	12	18.599	0.996	16.191	1.00	0.00
ATOM	188	HB2	ASP	12	19.452	1.539	16.599	1.00	0.00
ATOM	189	HB3	ASP	12	17.701	1.257	16.750	1.00	0.00
ATOM	190	CG	ASP	12	18.851	-0.509	16.306	1.00	0.00
ATOM	191	OD1	ASP	12	18.613	-1.205	15.333	1.00	0.00
ATOM	192	OD2	ASP	12	19.278	-0.939	17.365	1.00	0.00
ATOM	193	C	ASP	12	18.047	2.852	14.613	1.00	0.00
ATOM	194	O	ASP	12	17.729	3.495	15.593	1.00	0.00
ATOM	195	N	SER	13	18.092	3.400	13.429	1.00	0.00
ATOM	196	H	SER	13	18.356	2.872	12.609	1.00	0.00
ATOM	197	CA	SER	13	17.748	4.840	13.262	1.00	0.00
ATOM	198	HA	SER	13	18.461	5.464	13.801	1.00	0.00
ATOM	199	CB	SER	13	17.801	5.208	11.778	1.00	0.00
ATOM	200	HB2	SER	13	17.039	4.647	11.236	1.00	0.00
ATOM	201	HB3	SER	13	17.618	6.276	11.661	1.00	0.00
ATOM	202	OG	SER	13	19.083	4.888	11.257	1.00	0.00
ATOM	203	HG	SER	13	19.117	5.118	10.326	1.00	0.00
ATOM	204	C	SER	13	16.338	5.091	13.800	1.00	0.00
ATOM	205	O	SER	13	16.065	6.112	14.400	1.00	0.00
ATOM	206	N	SER	14	15.441	4.167	13.590	1.00	0.00
ATOM	207	H	SER	14	15.663	3.315	13.093	1.00	0.00

ATOM	208	CA	SER	14	14.048	4.352	14.090	1.00	0.00
ATOM	209	HA	SER	14	14.051	4.934	15.011	1.00	0.00
ATOM	210	CB	SER	14	13.225	5.101	13.040	1.00	0.00
ATOM	211	HB2	SER	14	13.642	6.097	12.894	1.00	0.00
ATOM	212	HB3	SER	14	13.253	4.554	12.097	1.00	0.00
ATOM	213	OG	SER	14	11.880	5.211	13.484	1.00	0.00
ATOM	214	HG	SER	14	11.363	5.680	12.825	1.00	0.00
ATOM	215	C	SER	14	13.418	2.982	14.350	1.00	0.00
ATOM	216	O	SER	14	13.507	2.082	13.540	1.00	0.00
ATOM	217	N	SER	15	12.782	2.817	15.479	1.00	0.00
ATOM	218	H	SER	15	12.705	3.559	16.160	1.00	0.00
ATOM	219	CA	SER	15	12.149	1.505	15.790	1.00	0.00
ATOM	220	HA	SER	15	12.833	0.689	15.555	1.00	0.00
ATOM	221	CB	SER	15	11.809	1.444	17.281	1.00	0.00
ATOM	222	HB2	SER	15	11.472	0.439	17.537	1.00	0.00
ATOM	223	HB3	SER	15	12.695	1.689	17.867	1.00	0.00
ATOM	224	OG	SER	15	10.778	2.377	17.569	1.00	0.00
ATOM	225	HG	SER	15	10.565	2.339	18.504	1.00	0.00
ATOM	226	C	SER	15	10.869	1.344	14.967	1.00	0.00
ATOM	227	O	SER	15	10.449	0.245	14.665	1.00	0.00
ATOM	228	N	CYX	16	10.246	2.432	14.600	1.00	0.00
ATOM	229	H	CYX	16	10.588	3.349	14.847	1.00	0.00

ATOM	230	CA	CYX	16	8.996	2.337	13.794	1.00	0.00
ATOM	231	HA	CYX	16	8.235	1.781	14.342	1.00	0.00
ATOM	232	CB	CYX	16	8.464	3.742	13.507	1.00	0.00
ATOM	233	HB2	CYX	16	8.487	4.333	14.422	1.00	0.00
ATOM	234	HB3	CYX	16	9.087	4.219	12.751	1.00	0.00
ATOM	235	SG	CYX	16	6.741	3.636	12.960	1.00	0.00
ATOM	236	C	CYX	16	9.297	1.626	12.473	1.00	0.00
ATOM	237	O	CYX	16	8.427	1.040	11.860	1.00	0.00
ATOM	238	N	GLU	17	10.523	1.673	12.030	1.00	0.00
ATOM	239	H	GLU	17	11.251	2.158	12.534	1.00	0.00
ATOM	240	CA	GLU	17	10.879	1.000	10.750	1.00	0.00
ATOM	241	HA	GLU	17	10.279	1.399	9.932	1.00	0.00
ATOM	242	CB	GLU	17	12.356	1.248	10.439	1.00	0.00
ATOM	243	HB2	GLU	17	12.947	1.115	11.346	1.00	0.00
ATOM	244	HB3	GLU	17	12.692	0.541	9.680	1.00	0.00
ATOM	245	CG	GLU	17	12.534	2.677	9.919	1.00	0.00
ATOM	246	HG2	GLU	17	11.908	2.824	9.039	1.00	0.00
ATOM	247	HG3	GLU	17	12.243	3.385	10.694	1.00	0.00
ATOM	248	CD	GLU	17	14.000	2.904	9.545	1.00	0.00
ATOM	249	OE1	GLU	17	14.771	1.966	9.653	1.00	0.00
ATOM	250	OE2	GLU	17	14.326	4.014	9.157	1.00	0.00
ATOM	251	C	GLU	17	10.629	-0.504	10.879	1.00	0.00

ATOM	252	O	GLU	17	10.013	-1.115	10.028	1.00	0.00
ATOM	253	N	ARG	18	11.102	-1.106	11.936	1.00	0.00
ATOM	254	H	ARG	18	11.614	-0.605	12.648	1.00	0.00
ATOM	255	CA	ARG	18	10.891	-2.571	12.117	1.00	0.00
ATOM	256	HA	ARG	18	11.472	-3.130	11.383	1.00	0.00
ATOM	257	CB	ARG	18	11.347	-2.984	13.518	1.00	0.00
ATOM	258	HB2	ARG	18	12.402	-2.740	13.645	1.00	0.00
ATOM	259	HB3	ARG	18	10.759	-2.451	14.265	1.00	0.00
ATOM	260	CG	ARG	18	11.149	-4.491	13.694	1.00	0.00
ATOM	261	HG2	ARG	18	10.101	-4.742	13.533	1.00	0.00
ATOM	262	HG3	ARG	18	11.765	-5.026	12.971	1.00	0.00
ATOM	263	CD	ARG	18	11.558	-4.897	15.112	1.00	0.00
ATOM	264	HD2	ARG	18	11.629	-5.982	15.188	1.00	0.00
ATOM	265	HD3	ARG	18	12.519	-4.452	15.369	1.00	0.00
ATOM	266	NE	ARG	18	10.534	-4.416	16.082	1.00	0.00
ATOM	267	HE	ARG	18	9.776	-3.815	15.793	1.00	0.00
ATOM	268	CZ	ARG	18	10.606	-4.769	17.337	1.00	0.00
ATOM	269	NH1	ARG	18	11.106	-3.947	18.219	1.00	0.00
ATOM	270	HH11	ARG	18	11.437	-3.039	17.927	1.00	0.00
ATOM	271	HH12	ARG	18	11.160	-4.224	19.189	1.00	0.00
ATOM	272	NH2	ARG	18	10.179	-5.945	17.708	1.00	0.00
ATOM	273	HH21	ARG	18	9.794	-6.579	17.022	1.00	0.00

```
ATOM    274 HH22 ARG    18      10.236  -6.217  18.679  1.00  0.00
ATOM    275  C   ARG    18       9.405  -2.896  11.949  1.00  0.00
ATOM    276  O   ARG    18       9.033  -4.022  11.682  1.00  0.00
ATOM    277  N   GLN    19       8.552  -1.921  12.106  1.00  0.00
ATOM    278  H   GLN    19       8.853  -0.984  12.330  1.00  0.00
ATOM    279  CA  GLN    19       7.093  -2.178  11.958  1.00  0.00
ATOM    280  HA  GLN    19       6.821  -3.110  12.454  1.00  0.00
ATOM    281  CB  GLN    19       6.306  -1.034  12.600  1.00  0.00
ATOM    282  HB2 GLN    19       6.703  -0.080  12.254  1.00  0.00
ATOM    283  HB3 GLN    19       5.256  -1.114  12.321  1.00  0.00
ATOM    284  CG  GLN    19       6.437  -1.118  14.122  1.00  0.00
ATOM    285  HG2 GLN    19       6.178  -2.119  14.468  1.00  0.00
ATOM    286  HG3 GLN    19       7.456  -0.881  14.428  1.00  0.00
ATOM    287  CD  GLN    19       5.485  -0.114  14.774  1.00  0.00
ATOM    288  OE1 GLN    19       5.006   0.796  14.127  1.00  0.00
ATOM    289  NE2 GLN    19       5.188  -0.240  16.039  1.00  0.00
ATOM    290 HE21 GLN    19       4.560   0.419  16.475  1.00  0.00
ATOM    291 HE22 GLN    19       5.588  -0.997  16.575  1.00  0.00
ATOM    292  C   GLN    19       6.737  -2.273  10.472  1.00  0.00
ATOM    293  O   GLN    19       5.706  -2.801  10.105  1.00  0.00
ATOM    294  N   VAL    20       7.581  -1.769   9.613  1.00  0.00
ATOM    295  H   VAL    20       8.440  -1.327   9.908  1.00  0.00
```

ATOM 296 CA VAL 20 7.285 -1.835 8.154 1.00 0.00

ATOM 297 HA VAL 20 6.247 -1.562 7.967 1.00 0.00

ATOM 298 CB VAL 20 8.192 -0.858 7.401 1.00 0.00

ATOM 299 HB VAL 20 8.200 0.100 7.919 1.00 0.00

ATOM 300 CG1 VAL 20 9.615 -1.417 7.341 1.00 0.00

ATOM 301 HG11 VAL 20 9.608 -2.376 6.822 1.00 0.00

ATOM 302 HG12 VAL 20 10.258 -0.720 6.804 1.00 0.00

ATOM 303 HG13 VAL 20 9.995 -1.555 8.353 1.00 0.00

ATOM 304 CG2 VAL 20 7.662 -0.664 5.979 1.00 0.00

ATOM 305 HG21 VAL 20 6.650 -0.263 6.020 1.00 0.00

ATOM 306 HG22 VAL 20 8.307 0.031 5.442 1.00 0.00

ATOM 307 HG23 VAL 20 7.651 -1.623 5.461 1.00 0.00

ATOM 308 C VAL 20 7.534 -3.259 7.650 1.00 0.00

ATOM 309 O VAL 20 6.879 -3.728 6.739 1.00 0.00

ATOM 310 N ASP 21 8.474 -3.951 8.235 1.00 0.00

ATOM 311 H ASP 21 9.023 -3.567 8.991 1.00 0.00

ATOM 312 CA ASP 21 8.762 -5.343 7.790 1.00 0.00

ATOM 313 HA ASP 21 9.175 -5.340 6.781 1.00 0.00

ATOM 314 CB ASP 21 9.783 -5.979 8.733 1.00 0.00

ATOM 315 HB2 ASP 21 9.473 -5.822 9.766 1.00 0.00

ATOM 316 HB3 ASP 21 9.847 -7.049 8.532 1.00 0.00

ATOM 317 CG ASP 21 11.154 -5.336 8.509 1.00 0.00

ATOM	318	OD1	ASP	21	11.273	-4.552	7.581	1.00	0.00
ATOM	319	OD2	ASP	21	12.059	-5.638	9.268	1.00	0.00
ATOM	320	C	ASP	21	7.470	-6.163	7.808	1.00	0.00
ATOM	321	O	ASP	21	7.288	-7.070	7.021	1.00	0.00
ATOM	322	N	ARG	22	6.572	-5.849	8.700	1.00	0.00
ATOM	323	H	ARG	22	6.717	-5.097	9.358	1.00	0.00
ATOM	324	CA	ARG	22	5.292	-6.610	8.767	1.00	0.00
ATOM	325	HA	ARG	22	5.479	-7.677	8.639	1.00	0.00
ATOM	326	CB	ARG	22	4.637	-6.388	10.133	1.00	0.00
ATOM	327	HB2	ARG	22	5.357	-6.607	10.922	1.00	0.00
ATOM	328	HB3	ARG	22	4.312	-5.352	10.216	1.00	0.00
ATOM	329	CG	ARG	22	3.429	-7.314	10.275	1.00	0.00
ATOM	330	HG2	ARG	22	2.755	-7.165	9.432	1.00	0.00
ATOM	331	HG3	ARG	22	3.765	-8.351	10.291	1.00	0.00
ATOM	332	CD	ARG	22	2.693	-6.996	11.579	1.00	0.00
ATOM	333	HD2	ARG	22	3.391	-6.997	12.416	1.00	0.00
ATOM	334	HD3	ARG	22	2.209	-6.022	11.511	1.00	0.00
ATOM	335	NE	ARG	22	1.650	-8.032	11.826	1.00	0.00
ATOM	336	HE	ARG	22	1.525	-8.450	12.737	1.00	0.00
ATOM	337	CZ	ARG	22	0.873	-8.419	10.851	1.00	0.00
ATOM	338	NH1	ARG	22	-0.361	-7.996	10.793	1.00	0.00
ATOM	339	HH11	ARG	22	-0.711	-7.370	11.504	1.00	0.00

ATOM 340 HH12 ARG 22 -0.960 -8.298 10.037 1.00 0.00

ATOM 341 NH2 ARG 22 1.328 -9.229 9.935 1.00 0.00

ATOM 342 HH21 ARG 22 2.283 -9.556 9.983 1.00 0.00

ATOM 343 HH22 ARG 22 0.724 -9.526 9.182 1.00 0.00

ATOM 344 C ARG 22 4.354 -6.123 7.664 1.00 0.00

ATOM 345 O ARG 22 3.400 -6.786 7.308 1.00 0.00

ATOM 346 N VAL 23 4.616 -4.967 7.118 1.00 0.00

ATOM 347 H VAL 23 5.407 -4.411 7.410 1.00 0.00

ATOM 348 CA VAL 23 3.740 -4.436 6.036 1.00 0.00

ATOM 349 HA VAL 23 2.818 -4.037 6.457 1.00 0.00

ATOM 350 CB VAL 23 4.468 -3.311 5.297 1.00 0.00

ATOM 351 HB VAL 23 5.475 -3.638 5.037 1.00 0.00

ATOM 352 CG1 VAL 23 3.701 -2.960 4.020 1.00 0.00

ATOM 353 HG11 VAL 23 2.694 -2.633 4.279 1.00 0.00

ATOM 354 HG12 VAL 23 4.219 -2.159 3.493 1.00 0.00

ATOM 355 HG13 VAL 23 3.643 -3.839 3.377 1.00 0.00

ATOM 356 CG2 VAL 23 4.550 -2.078 6.199 1.00 0.00

ATOM 357 HG21 VAL 23 5.097 -2.329 7.108 1.00 0.00

ATOM 358 HG22 VAL 23 5.069 -1.277 5.673 1.00 0.00

ATOM 359 HG23 VAL 23 3.544 -1.750 6.459 1.00 0.00

ATOM 360 C VAL 23 3.406 -5.559 5.053 1.00 0.00

ATOM 361 O VAL 23 2.368 -6.184 5.138 1.00 0.00

ATOM	362	N	ASN	24	4.279	-5.819	4.121	1.00	0.00
ATOM	363	H	ASN	24	5.144	-5.304	4.046	1.00	0.00
ATOM	364	CA	ASN	24	4.013	-6.900	3.132	1.00	0.00
ATOM	365	HA	ASN	24	4.783	-6.906	2.361	1.00	0.00
ATOM	366	CB	ASN	24	4.024	-8.254	3.844	1.00	0.00
ATOM	367	HB2	ASN	24	3.255	-8.282	4.615	1.00	0.00
ATOM	368	HB3	ASN	24	3.851	-9.059	3.129	1.00	0.00
ATOM	369	CG	ASN	24	5.387	-8.472	4.505	1.00	0.00
ATOM	370	OD1	ASN	24	6.355	-7.825	4.155	1.00	0.00
ATOM	371	ND2	ASN	24	5.505	-9.362	5.452	1.00	0.00
ATOM	372	HD21	ASN	24	6.402	-9.511	5.892	1.00	0.00
ATOM	373	HD22	ASN	24	4.699	-9.898	5.740	1.00	0.00
ATOM	374	C	ASN	24	2.644	-6.672	2.486	1.00	0.00
ATOM	375	O	ASN	24	1.682	-7.346	2.794	1.00	0.00
ATOM	376	N	LEU	25	2.550	-5.727	1.592	1.00	0.00
ATOM	377	H	LEU	25	3.347	-5.165	1.330	1.00	0.00
ATOM	378	CA	LEU	25	1.244	-5.456	0.927	1.00	0.00
ATOM	379	HA	LEU	25	0.449	-6.027	1.407	1.00	0.00
ATOM	380	CB	LEU	25	0.914	-3.966	1.042	1.00	0.00
ATOM	381	HB2	LEU	25	1.806	-3.379	0.824	1.00	0.00
ATOM	382	HB3	LEU	25	0.129	-3.712	0.330	1.00	0.00
ATOM	383	CG	LEU	25	0.434	-3.657	2.462	1.00	0.00

```
ATOM    384  HG  LEU    25       1.074  -4.169   3.181  1.00  0.00
ATOM    385  CD1 LEU    25       0.497  -2.149   2.706  1.00  0.00
ATOM    386 HD11 LEU    25      -0.143  -1.638   1.987  1.00  0.00
ATOM    387 HD12 LEU    25       0.155  -1.930   3.717  1.00  0.00
ATOM    388 HD13 LEU    25       1.524  -1.804   2.587  1.00  0.00
ATOM    389  CD2 LEU    25      -1.009  -4.141   2.629  1.00  0.00
ATOM    390 HD21 LEU    25      -1.054  -5.217   2.456  1.00  0.00
ATOM    391 HD22 LEU    25      -1.351  -3.922   3.640  1.00  0.00
ATOM    392 HD23 LEU    25      -1.649  -3.631   1.910  1.00  0.00
ATOM    393  C   LEU    25       1.336  -5.845  -0.550  1.00  0.00
ATOM    394  O   LEU    25       1.308  -5.004  -1.427  1.00  0.00
ATOM    395  N   LYS    26       1.445  -7.115  -0.832  1.00  0.00
ATOM    396  H   LYS    26       1.473  -7.816  -0.105  1.00  0.00
ATOM    397  CA  LYS    26       1.541  -7.563  -2.250  1.00  0.00
ATOM    398  HA  LYS    26       2.330  -7.018  -2.768  1.00  0.00
ATOM    399  CB  LYS    26       1.873  -9.056  -2.288  1.00  0.00
ATOM    400  HB2 LYS    26       2.730  -9.255  -1.645  1.00  0.00
ATOM    401  HB3 LYS    26       1.015  -9.629  -1.937  1.00  0.00
ATOM    402  CG  LYS    26       2.207  -9.467  -3.724  1.00  0.00
ATOM    403  HG2 LYS    26       1.640 -10.360  -3.988  1.00  0.00
ATOM    404  HG3 LYS    26       1.946  -8.657  -4.404  1.00  0.00
ATOM    405  CD  LYS    26       3.704  -9.763  -3.835  1.00  0.00
```

ATOM	406	HD2	LYS	26	4.099	-9.308	-4.743	1.00	0.00
ATOM	407	HD3	LYS	26	4.221	-9.352	-2.968	1.00	0.00
ATOM	408	CE	LYS	26	3.923	-11.277	-3.888	1.00	0.00
ATOM	409	HE2	LYS	26	4.238	-11.634	-2.907	1.00	0.00
ATOM	410	HE3	LYS	26	2.993	-11.770	-4.172	1.00	0.00
ATOM	411	NZ	LYS	26	4.978	-11.592	-4.892	1.00	0.00
ATOM	412	HZ1	LYS	26	5.840	-11.137	-4.629	1.00	0.00
ATOM	413	HZ2	LYS	26	5.122	-12.591	-4.927	1.00	0.00
ATOM	414	HZ3	LYS	26	4.687	-11.263	-5.801	1.00	0.00
ATOM	415	C	LYS	26	0.214	-7.318	-2.980	1.00	0.00
ATOM	416	O	LYS	26	0.203	-6.964	-4.142	1.00	0.00
ATOM	417	N	PRO	27	-0.931	-7.515	-2.298	1.00	0.00
ATOM	418	CD	PRO	27	-1.040	-7.940	-0.888	1.00	0.00
ATOM	419	HD2	PRO	27	-0.351	-7.358	-0.276	1.00	0.00
ATOM	420	HD3	PRO	27	-0.792	-8.999	-0.805	1.00	0.00
ATOM	421	CG	PRO	27	-2.479	-7.661	-0.561	1.00	0.00
ATOM	422	HG2	PRO	27	-2.572	-6.653	-0.159	1.00	0.00
ATOM	423	HG3	PRO	27	-2.830	-8.381	0.179	1.00	0.00
ATOM	424	CB	PRO	27	-3.226	-7.807	-1.841	1.00	0.00
ATOM	425	HB2	PRO	27	-4.100	-7.158	-1.811	1.00	0.00
ATOM	426	HB3	PRO	27	-3.539	-8.828	-2.063	1.00	0.00
ATOM	427	CA	PRO	27	-2.252	-7.317	-2.911	1.00	0.00

```
ATOM    428  HA  PRO    27      -2.296  -7.905  -3.828  1.00  0.00
ATOM    429  C   PRO    27      -2.513  -5.843  -3.237  1.00  0.00
ATOM    430  O   PRO    27      -3.060  -5.516  -4.272  1.00  0.00
ATOM    431  N   CYX    28      -2.117  -4.950  -2.372  1.00  0.00
ATOM    432  H   CYX    28      -1.655  -5.208  -1.511  1.00  0.00
ATOM    433  CA  CYX    28      -2.338  -3.503  -2.650  1.00  0.00
ATOM    434  HA  CYX    28      -3.392  -3.314  -2.850  1.00  0.00
ATOM    435  CB  CYX    28      -1.915  -2.677  -1.433  1.00  0.00
ATOM    436  HB2 CYX    28      -1.254  -3.272  -0.802  1.00  0.00
ATOM    437  HB3 CYX    28      -1.390  -1.782  -1.766  1.00  0.00
ATOM    438  SG  CYX    28      -3.375  -2.273  -0.442  1.00  0.00
ATOM    439  C   CYX    28      -1.492  -3.105  -3.860  1.00  0.00
ATOM    440  O   CYX    28      -1.996  -2.620  -4.858  1.00  0.00
ATOM    441  N   GLU    29      -0.206  -3.317  -3.787  1.00  0.00
ATOM    442  H   GLU    29       0.221  -3.723  -2.966  1.00  0.00
ATOM    443  CA  GLU    29       0.669  -2.966  -4.935  1.00  0.00
ATOM    444  HA  GLU    29       0.721  -1.884  -5.052  1.00  0.00
ATOM    445  CB  GLU    29       2.080  -3.499  -4.687  1.00  0.00
ATOM    446  HB2 GLU    29       2.319  -3.417  -3.627  1.00  0.00
ATOM    447  HB3 GLU    29       2.133  -4.545  -4.991  1.00  0.00
ATOM    448  CG  GLU    29       3.084  -2.682  -5.500  1.00  0.00
ATOM    449  HG2 GLU    29       2.810  -2.714  -6.555  1.00  0.00
```

```
ATOM    450  HG3 GLU    29       3.075  -1.649  -5.154  1.00  0.00
ATOM    451  CD  GLU    29       4.486  -3.267  -5.320  1.00  0.00
ATOM    452  OE1 GLU    29       4.617  -4.217  -4.564  1.00  0.00
ATOM    453  OE2 GLU    29       5.404  -2.758  -5.940  1.00  0.00
ATOM    454  C   GLU    29       0.100  -3.604  -6.202  1.00  0.00
ATOM    455  O   GLU    29       0.192  -3.058  -7.284  1.00  0.00
ATOM    456  N   GLN    30      -0.484  -4.764  -6.077  1.00  0.00
ATOM    457  H   GLN    30      -0.558  -5.227  -5.182  1.00  0.00
ATOM    458  CA  GLN    30      -1.056  -5.442  -7.271  1.00  0.00
ATOM    459  HA  GLN    30      -0.323  -5.469  -8.078  1.00  0.00
ATOM    460  CB  GLN    30      -1.436  -6.879  -6.909  1.00  0.00
ATOM    461  HB2 GLN    30      -1.855  -6.903  -5.903  1.00  0.00
ATOM    462  HB3 GLN    30      -2.175  -7.250  -7.619  1.00  0.00
ATOM    463  CG  GLN    30      -0.188  -7.765  -6.962  1.00  0.00
ATOM    464  HG2 GLN    30       0.132  -7.905  -7.995  1.00  0.00
ATOM    465  HG3 GLN    30       0.623  -7.314  -6.390  1.00  0.00
ATOM    466  CD  GLN    30      -0.508  -9.133  -6.360  1.00  0.00
ATOM    467  OE1 GLN    30      -1.605  -9.363  -5.890  1.00  0.00
ATOM    468  NE2 GLN    30       0.411 -10.060  -6.352  1.00  0.00
ATOM    469 HE21 GLN    30       0.207 -10.967  -5.956  1.00  0.00
ATOM    470 HE22 GLN    30       1.323  -9.867  -6.742  1.00  0.00
ATOM    471  C   GLN    30      -2.300  -4.687  -7.746  1.00  0.00
```

ATOM 472 O GLN 30 -2.676 -4.764 -8.899 1.00 0.00

ATOM 473 N HID 31 -2.942 -3.953 -6.877 1.00 0.00

ATOM 474 H HID 31 -2.641 -3.879 -5.916 1.00 0.00

ATOM 475 CA HID 31 -4.152 -3.197 -7.298 1.00 0.00

ATOM 476 HA HID 31 -4.765 -3.805 -7.963 1.00 0.00

ATOM 477 CB HID 31 -4.982 -2.834 -6.062 1.00 0.00

ATOM 478 HB2 HID 31 -5.655 -3.642 -5.775 1.00 0.00

ATOM 479 HB3 HID 31 -4.357 -2.564 -5.211 1.00 0.00

ATOM 480 CG HID 31 -5.846 -1.637 -6.359 1.00 0.00

ATOM 481 ND1 HID 31 -6.917 -1.697 -7.237 1.00 0.00

ATOM 482 HD1 HID 31 -7.224 -2.509 -7.754 1.00 0.00

ATOM 483 CE1 HID 31 -7.477 -0.474 -7.278 1.00 0.00

ATOM 484 HE1 HID 31 -8.342 -0.344 -7.929 1.00 0.00

ATOM 485 NE2 HID 31 -6.842 0.389 -6.480 1.00 0.00

ATOM 486 CD2 HID 31 -5.811 -0.343 -5.898 1.00 0.00

ATOM 487 HD2 HID 31 -5.132 0.137 -5.193 1.00 0.00

ATOM 488 C HID 31 -3.699 -1.929 -8.013 1.00 0.00

ATOM 489 O HID 31 -4.432 -1.331 -8.776 1.00 0.00

ATOM 490 N ILE 32 -2.485 -1.521 -7.772 1.00 0.00

ATOM 491 H ILE 32 -1.875 -2.015 -7.136 1.00 0.00

ATOM 492 CA ILE 32 -1.966 -0.295 -8.438 1.00 0.00

ATOM 493 HA ILE 32 -2.802 0.360 -8.682 1.00 0.00

```
ATOM    494  CB  ILE    32      -0.996   0.439  -7.506  1.00  0.00
ATOM    495  HB  ILE    32      -0.033   0.561  -8.002  1.00  0.00
ATOM    496  CG2 ILE    32      -1.569   1.811  -7.164  1.00  0.00
ATOM    497 HG21 ILE    32      -2.531   1.689  -6.667  1.00  0.00
ATOM    498 HG22 ILE    32      -0.882   2.337  -6.501  1.00  0.00
ATOM    499 HG23 ILE    32      -1.703   2.387  -8.079  1.00  0.00
ATOM    500  CG1 ILE    32      -0.801  -0.364  -6.216  1.00  0.00
ATOM    501 HG12 ILE    32      -1.774  -0.607  -5.790  1.00  0.00
ATOM    502 HG13 ILE    32      -0.263  -1.286  -6.441  1.00  0.00
ATOM    503  CD1 ILE    32       0.003   0.463  -5.211  1.00  0.00
ATOM    504 HD11 ILE    32      -0.534   1.384  -4.985  1.00  0.00
ATOM    505 HD12 ILE    32       0.140  -0.111  -4.295  1.00  0.00
ATOM    506 HD13 ILE    32       0.977   0.705  -5.636  1.00  0.00
ATOM    507  C   ILE    32      -1.238  -0.689  -9.722  1.00  0.00
ATOM    508  O   ILE    32      -1.511  -0.168 -10.785  1.00  0.00
ATOM    509  N   MET    33      -0.319  -1.610  -9.636  1.00  0.00
ATOM    510  H   MET    33      -0.086  -2.050  -8.757  1.00  0.00
ATOM    511  CA  MET    33       0.418  -2.039 -10.857  1.00  0.00
ATOM    512  HA  MET    33       0.930  -1.189 -11.308  1.00  0.00
ATOM    513  CB  MET    33       1.461  -3.093 -10.479  1.00  0.00
ATOM    514  HB2 MET    33       0.957  -3.992 -10.123  1.00  0.00
ATOM    515  HB3 MET    33       2.064  -3.338 -11.353  1.00  0.00
```

```
ATOM    516  CG  MET    33       2.365  -2.544  -9.374  1.00  0.00
ATOM    517  HG2 MET    33       2.765  -1.575  -9.672  1.00  0.00
ATOM    518  HG3 MET    33       1.794  -2.433  -8.452  1.00  0.00
ATOM    519  SD  MET    33       3.736  -3.691  -9.090  1.00  0.00
ATOM    520  CE  MET    33       4.867  -2.999 -10.321  1.00  0.00
ATOM    521  HE1 MET    33       5.076  -1.957 -10.079  1.00  0.00
ATOM    522  HE2 MET    33       5.799  -3.566 -10.318  1.00  0.00
ATOM    523  HE3 MET    33       4.410  -3.058 -11.309  1.00  0.00
ATOM    524  C   MET    33      -0.572  -2.634 -11.858  1.00  0.00
ATOM    525  O   MET    33      -0.478  -2.410 -13.047  1.00  0.00
ATOM    526  N   GLN    34      -1.522  -3.390 -11.383  1.00  0.00
ATOM    527  H   GLN    34      -1.605  -3.578 -10.394  1.00  0.00
ATOM    528  CA  GLN    34      -2.520  -4.003 -12.305  1.00  0.00
ATOM    529  HA  GLN    34      -2.016  -4.601 -13.065  1.00  0.00
ATOM    530  CB  GLN    34      -3.457  -4.913 -11.508  1.00  0.00
ATOM    531  HB2 GLN    34      -3.809  -4.386 -10.622  1.00  0.00
ATOM    532  HB3 GLN    34      -4.309  -5.190 -12.128  1.00  0.00
ATOM    533  CG  GLN    34      -2.704  -6.175 -11.084  1.00  0.00
ATOM    534  HG2 GLN    34      -2.423  -6.761 -11.959  1.00  0.00
ATOM    535  HG3 GLN    34      -1.808  -5.910 -10.522  1.00  0.00
ATOM    536  CD  GLN    34      -3.605  -7.031 -10.192  1.00  0.00
ATOM    537  OE1 GLN    34      -4.660  -6.595  -9.777  1.00  0.00
```

ATOM 538 NE2 GLN 34 -3.231 -8.241 -9.878 1.00 0.00
ATOM 539 HE21 GLN 34 -3.821 -8.812 -9.290 1.00 0.00
ATOM 540 HE22 GLN 34 -2.353 -8.602 -10.225 1.00 0.00
ATOM 541 C GLN 34 -3.337 -2.904 -12.987 1.00 0.00
ATOM 542 O GLN 34 -3.572 -2.945 -14.178 1.00 0.00
ATOM 543 N ARG 35 -3.780 -1.926 -12.245 1.00 0.00
ATOM 544 H ARG 35 -3.592 -1.884 -11.254 1.00 0.00
ATOM 545 CA ARG 35 -4.589 -0.834 -12.859 1.00 0.00
ATOM 546 HA ARG 35 -5.451 -1.249 -13.380 1.00 0.00
ATOM 547 CB ARG 35 -5.087 0.107 -11.762 1.00 0.00
ATOM 548 HB2 ARG 35 -4.306 0.239 -11.013 1.00 0.00
ATOM 549 HB3 ARG 35 -5.338 1.073 -12.198 1.00 0.00
ATOM 550 CG ARG 35 -6.331 -0.494 -11.101 1.00 0.00
ATOM 551 HG2 ARG 35 -7.035 -0.812 -11.871 1.00 0.00
ATOM 552 HG3 ARG 35 -6.042 -1.354 -10.497 1.00 0.00
ATOM 553 CD ARG 35 -6.992 0.558 -10.208 1.00 0.00
ATOM 554 HD2 ARG 35 -7.402 1.364 -10.815 1.00 0.00
ATOM 555 HD3 ARG 35 -7.789 0.105 -9.619 1.00 0.00
ATOM 556 NE ARG 35 -5.977 1.129 -9.279 1.00 0.00
ATOM 557 HE ARG 35 -5.129 0.629 -9.052 1.00 0.00
ATOM 558 CZ ARG 35 -6.174 2.299 -8.736 1.00 0.00
ATOM 559 NH1 ARG 35 -6.689 3.268 -9.443 1.00 0.00

ATOM	560	HH11	ARG	35	-6.934	3.108	-10.410	1.00	0.00
ATOM	561	HH12	ARG	35	-6.840	4.172	-9.020	1.00	0.00
ATOM	562	NH2	ARG	35	-5.854	2.501	-7.488	1.00	0.00
ATOM	563	HH21	ARG	35	-5.454	1.749	-6.945	1.00	0.00
ATOM	564	HH22	ARG	35	-6.008	3.407	-7.071	1.00	0.00
ATOM	565	C	ARG	35	-3.731	-0.047	-13.853	1.00	0.00
ATOM	566	O	ARG	35	-4.089	0.115	-15.002	1.00	0.00
ATOM	567	N	ILE	36	-2.606	0.448	-13.419	1.00	0.00
ATOM	568	H	ILE	36	-2.303	0.320	-12.464	1.00	0.00
ATOM	569	CA	ILE	36	-1.732	1.230	-14.339	1.00	0.00
ATOM	570	HA	ILE	36	-2.306	2.036	-14.795	1.00	0.00
ATOM	571	CB	ILE	36	-0.564	1.821	-13.547	1.00	0.00
ATOM	572	HB	ILE	36	0.070	2.405	-14.214	1.00	0.00
ATOM	573	CG2	ILE	36	-1.108	2.725	-12.438	1.00	0.00
ATOM	574	HG21	ILE	36	-1.741	2.141	-11.771	1.00	0.00
ATOM	575	HG22	ILE	36	-0.277	3.147	-11.872	1.00	0.00
ATOM	576	HG23	ILE	36	-1.693	3.531	-12.880	1.00	0.00
ATOM	577	CG1	ILE	36	0.259	0.687	-12.930	1.00	0.00
ATOM	578	HG12	ILE	36	-0.413	-0.071	-12.528	1.00	0.00
ATOM	579	HG13	ILE	36	0.893	0.240	-13.697	1.00	0.00
ATOM	580	CD1	ILE	36	1.135	1.241	-11.804	1.00	0.00
ATOM	581	HD11	ILE	36	0.503	1.687	-11.037	1.00	0.00

ATOM	582	HD12	ILE	36	1.720	0.432	-11.366	1.00	0.00
ATOM	583	HD13	ILE	36	1.808	1.998	-12.206	1.00	0.00
ATOM	584	C	ILE	36	-1.191	0.320	-15.444	1.00	0.00
ATOM	585	O	ILE	36	-0.676	0.782	-16.442	1.00	0.00
ATOM	586	N	MET	37	-1.303	-0.970	-15.278	1.00	0.00
ATOM	587	H	MET	37	-1.727	-1.368	-14.452	1.00	0.00
ATOM	588	CA	MET	37	-0.791	-1.899	-16.327	1.00	0.00
ATOM	589	HA	MET	37	-0.140	-1.365	-17.019	1.00	0.00
ATOM	590	CB	MET	37	0.012	-3.023	-15.670	1.00	0.00
ATOM	591	HB2	MET	37	-0.555	-3.437	-14.836	1.00	0.00
ATOM	592	HB3	MET	37	0.207	-3.808	-16.402	1.00	0.00
ATOM	593	CG	MET	37	1.341	-2.465	-15.155	1.00	0.00
ATOM	594	HG2	MET	37	1.757	-1.771	-15.885	1.00	0.00
ATOM	595	HG3	MET	37	1.182	-1.946	-14.210	1.00	0.00
ATOM	596	SD	MET	37	2.506	-3.825	-14.896	1.00	0.00
ATOM	597	CE	MET	37	1.511	-4.754	-13.704	1.00	0.00
ATOM	598	HE1	MET	37	0.564	-5.037	-14.163	1.00	0.00
ATOM	599	HE2	MET	37	2.050	-5.653	-13.402	1.00	0.00
ATOM	600	HE3	MET	37	1.320	-4.134	-12.828	1.00	0.00
ATOM	601	C	MET	37	-1.969	-2.495	-17.099	1.00	0.00
ATOM	602	O	MET	37	-1.818	-2.975	-18.206	1.00	0.00
ATOM	603	N	GLY	38	-3.142	-2.470	-16.528	1.00	0.00

```
ATOM    604  H   GLY    38      -3.276  -2.074 -15.609  1.00  0.00
ATOM    605  CA  GLY    38      -4.326  -3.036 -17.233  1.00  0.00
ATOM    606  HA2 GLY    38      -4.117  -4.057 -17.555  1.00  0.00
ATOM    607  HA3 GLY    38      -5.191  -3.035 -16.571  1.00  0.00
ATOM    608  C   GLY    38      -4.638  -2.180 -18.463  1.00  0.00
ATOM    609  O   GLY    38      -5.217  -2.646 -19.425  1.00  0.00
ATOM    610  N   GLU    39      -4.259  -0.932 -18.438  1.00  0.00
ATOM    611  H   GLU    39      -3.779  -0.539 -17.641  1.00  0.00
ATOM    612  CA  GLU    39      -4.533  -0.047 -19.605  1.00  0.00
ATOM    613  HA  GLU    39      -5.601  -0.029 -19.823  1.00  0.00
ATOM    614  CB  GLU    39      -4.074   1.377 -19.285  1.00  0.00
ATOM    615  HB2 GLU    39      -4.535   1.707 -18.354  1.00  0.00
ATOM    616  HB3 GLU    39      -2.989   1.394 -19.179  1.00  0.00
ATOM    617  CG  GLU    39      -4.490   2.314 -20.420  1.00  0.00
ATOM    618  HG2 GLU    39      -4.113   1.927 -21.367  1.00  0.00
ATOM    619  HG3 GLU    39      -5.577   2.376 -20.460  1.00  0.00
ATOM    620  CD  GLU    39      -3.909   3.707 -20.171  1.00  0.00
ATOM    621  OE1 GLU    39      -3.198   3.864 -19.191  1.00  0.00
ATOM    622  OE2 GLU    39      -4.184   4.593 -20.963  1.00  0.00
ATOM    623  C   GLU    39      -3.775  -0.566 -20.828  1.00  0.00
ATOM    624  O   GLU    39      -4.159  -0.327 -21.956  1.00  0.00
ATOM    625  N   GLN    40      -2.699  -1.273 -20.615  1.00  0.00
```

ATOM	626	H	GLN	40	-2.371	-1.475	-19.681	1.00	0.00
ATOM	627	CA	GLN	40	-1.919	-1.806	-21.767	1.00	0.00
ATOM	628	HA	GLN	40	-1.645	-0.998	-22.445	1.00	0.00
ATOM	629	CB	GLN	40	-0.639	-2.469	-21.254	1.00	0.00
ATOM	630	HB2	GLN	40	-0.888	-3.179	-20.465	1.00	0.00
ATOM	631	HB3	GLN	40	-0.148	-2.996	-22.073	1.00	0.00
ATOM	632	CG	GLN	40	0.303	-1.399	-20.699	1.00	0.00
ATOM	633	HG2	GLN	40	0.560	-0.680	-21.477	1.00	0.00
ATOM	634	HG3	GLN	40	-0.167	-0.879	-19.865	1.00	0.00
ATOM	635	CD	GLN	40	1.589	-2.060	-20.200	1.00	0.00
ATOM	636	OE1	GLN	40	1.651	-3.266	-20.061	1.00	0.00
ATOM	637	NE2	GLN	40	2.627	-1.318	-19.924	1.00	0.00
ATOM	638	HE21	GLN	40	3.479	-1.750	-19.594	1.00	0.00
ATOM	639	HE22	GLN	40	2.574	-0.317	-20.042	1.00	0.00
ATOM	640	C	GLN	40	-2.763	-2.837	-22.518	1.00	0.00
ATOM	641	O	GLN	40	-2.660	-2.982	-23.719	1.00	0.00
ATOM	642	N	GLU	41	-3.598	-3.555	-21.817	1.00	0.00
ATOM	643	H	GLU	41	-3.687	-3.443	-20.817	1.00	0.00
ATOM	644	CA	GLU	41	-4.450	-4.575	-22.492	1.00	0.00
ATOM	645	HA	GLU	41	-3.828	-5.346	-22.948	1.00	0.00
ATOM	646	CB	GLU	41	-5.369	-5.234	-21.461	1.00	0.00
ATOM	647	HB2	GLU	41	-5.980	-4.472	-20.979	1.00	0.00

ATOM 648 HB3 GLU 41 -6.016 -5.956 -21.960 1.00 0.00

ATOM 649 CG GLU 41 -4.523 -5.951 -20.407 1.00 0.00

ATOM 650 HG2 GLU 41 -3.889 -6.694 -20.892 1.00 0.00

ATOM 651 HG3 GLU 41 -3.898 -5.225 -19.887 1.00 0.00

ATOM 652 CD GLU 41 -5.443 -6.645 -19.401 1.00 0.00

ATOM 653 OE1 GLU 41 -6.638 -6.412 -19.463 1.00 0.00

ATOM 654 OE2 GLU 41 -4.936 -7.398 -18.587 1.00 0.00

ATOM 655 C GLU 41 -5.295 -3.898 -23.573 1.00 0.00

ATOM 656 O GLU 41 -5.798 -2.809 -23.387 1.00 0.00

ATOM 657 N GLN 42 -5.455 -4.536 -24.700 1.00 0.00

ATOM 658 H GLN 42 -5.038 -5.442 -24.863 1.00 0.00

ATOM 659 CA GLN 42 -6.268 -3.927 -25.789 1.00 0.00

ATOM 660 HA GLN 42 -5.868 -2.950 -26.059 1.00 0.00

ATOM 661 CB GLN 42 -6.225 -4.831 -27.023 1.00 0.00

ATOM 662 HB2 GLN 42 -6.570 -5.830 -26.755 1.00 0.00

ATOM 663 HB3 GLN 42 -6.871 -4.420 -27.798 1.00 0.00

ATOM 664 CG GLN 42 -4.790 -4.911 -27.546 1.00 0.00

ATOM 665 HG2 GLN 42 -4.136 -5.353 -26.794 1.00 0.00

ATOM 666 HG3 GLN 42 -4.752 -5.510 -28.456 1.00 0.00

ATOM 667 CD GLN 42 -4.285 -3.502 -27.866 1.00 0.00

ATOM 668 OE1 GLN 42 -4.936 -2.755 -28.568 1.00 0.00

ATOM 669 NE2 GLN 42 -3.143 -3.105 -27.376 1.00 0.00

```
ATOM    670 HE21 GLN    42      -2.803  -2.177 -27.583  1.00  0.00
ATOM    671 HE22 GLN    42      -2.603  -3.728 -26.792  1.00  0.00
ATOM    672  C   GLN    42      -7.715  -3.770 -25.319  1.00  0.00
ATOM    673  O   GLN    42      -8.386  -2.814 -25.651  1.00  0.00
ATOM    674  N   TYR    43      -8.202  -4.703 -24.546  1.00  0.00
ATOM    675  H   TYR    43      -7.650  -5.501 -24.264  1.00  0.00
ATOM    676  CA  TYR    43      -9.606  -4.606 -24.055  1.00  0.00
ATOM    677  HA  TYR    43     -10.090  -3.721 -24.468  1.00  0.00
ATOM    678  CB  TYR    43     -10.390  -5.844 -24.498  1.00  0.00
ATOM    679  HB2 TYR    43      -9.928  -6.736 -24.074  1.00  0.00
ATOM    680  HB3 TYR    43     -11.420  -5.769 -24.150  1.00  0.00
ATOM    681  CG  TYR    43     -10.374  -5.937 -26.005  1.00  0.00
ATOM    682  CD1 TYR    43     -11.249  -5.150 -26.764  1.00  0.00
ATOM    683  HD1 TYR    43     -11.940  -4.470 -26.268  1.00  0.00
ATOM    684  CE1 TYR    43     -11.235  -5.238 -28.161  1.00  0.00
ATOM    685  HE1 TYR    43     -11.916  -4.626 -28.752  1.00  0.00
ATOM    686  CZ  TYR    43     -10.348  -6.113 -28.799  1.00  0.00
ATOM    687  OH  TYR    43     -10.335  -6.199 -30.177  1.00  0.00
ATOM    688  HH  TYR    43     -10.973  -5.617 -30.595  1.00  0.00
ATOM    689  CE2 TYR    43      -9.473  -6.899 -28.040  1.00  0.00
ATOM    690  HE2 TYR    43      -8.781  -7.580 -28.536  1.00  0.00
ATOM    691  CD2 TYR    43      -9.487  -6.811 -26.644  1.00  0.00
```

ATOM	692	HD2	TYR	43	-8.806	-7.424	-26.053	1.00	0.00
ATOM	693	C	TYR	43	-9.606	-4.525	-22.527	1.00	0.00
ATOM	694	O	TYR	43	-8.659	-4.917	-21.875	1.00	0.00
ATOM	695	N	ASP	44	-10.662	-4.019	-21.951	1.00	0.00
ATOM	696	H	ASP	44	-11.453	-3.692	-22.486	1.00	0.00
ATOM	697	CA	ASP	44	-10.721	-3.913	-20.466	1.00	0.00
ATOM	698	HA	ASP	44	-10.727	-4.906	-20.017	1.00	0.00
ATOM	699	CB	ASP	44	-9.493	-3.154	-19.957	1.00	0.00
ATOM	700	HB2	ASP	44	-9.618	-2.930	-18.898	1.00	0.00
ATOM	701	HB3	ASP	44	-8.602	-3.767	-20.095	1.00	0.00
ATOM	702	CG	ASP	44	-9.340	-1.848	-20.740	1.00	0.00
ATOM	703	OD1	ASP	44	-10.056	-1.675	-21.712	1.00	0.00
ATOM	704	OD2	ASP	44	-8.507	-1.045	-20.353	1.00	0.00
ATOM	705	C	ASP	44	-11.990	-3.161	-20.059	1.00	0.00
ATOM	706	O	ASP	44	-12.697	-3.558	-19.155	1.00	0.00
ATOM	707	N	SER	45	-12.285	-2.075	-20.721	1.00	0.00
ATOM	708	H	SER	45	-11.702	-1.737	-21.473	1.00	0.00
ATOM	709	CA	SER	45	-13.509	-1.300	-20.373	1.00	0.00
ATOM	710	HA	SER	45	-13.387	-0.814	-19.405	1.00	0.00
ATOM	711	CB	SER	45	-13.748	-0.224	-21.434	1.00	0.00
ATOM	712	HB2	SER	45	-14.554	0.434	-21.110	1.00	0.00
ATOM	713	HB3	SER	45	-12.837	0.358	-21.572	1.00	0.00

```
ATOM    714  OG  SER    45     -14.106  -0.841 -22.662  1.00  0.00
ATOM    715  HG  SER    45     -14.256  -0.166 -23.328  1.00  0.00
ATOM    716  C   SER    45     -14.712  -2.242 -20.321  1.00  0.00
ATOM    717  O   SER    45     -15.610  -2.075 -19.520  1.00  0.00
ATOM    718  N   TYR    46     -14.739  -3.232 -21.172  1.00  0.00
ATOM    719  H   TYR    46     -13.995  -3.378 -21.840  1.00  0.00
ATOM    720  CA  TYR    46     -15.885  -4.183 -21.173  1.00  0.00
ATOM    721  HA  TYR    46     -16.818  -3.651 -21.359  1.00  0.00
ATOM    722  CB  TYR    46     -15.685  -5.221 -22.279  1.00  0.00
ATOM    723  HB2 TYR    46     -14.719  -5.711 -22.151  1.00  0.00
ATOM    724  HB3 TYR    46     -16.479  -5.966 -22.226  1.00  0.00
ATOM    725  CG  TYR    46     -15.727  -4.536 -23.624  1.00  0.00
ATOM    726  CD1 TYR    46     -16.919  -3.962 -24.080  1.00  0.00
ATOM    727  HD1 TYR    46     -17.817  -4.011 -23.464  1.00  0.00
ATOM    728  CE1 TYR    46     -16.959  -3.326 -25.327  1.00  0.00
ATOM    729  HE1 TYR    46     -17.887  -2.880 -25.682  1.00  0.00
ATOM    730  CZ  TYR    46     -15.805  -3.263 -26.117  1.00  0.00
ATOM    731  OH  TYR    46     -15.844  -2.637 -27.346  1.00  0.00
ATOM    732  HH  TYR    46     -16.710  -2.282 -27.559  1.00  0.00
ATOM    733  CE2 TYR    46     -14.612  -3.836 -25.661  1.00  0.00
ATOM    734  HE2 TYR    46     -13.714  -3.787 -26.277  1.00  0.00
ATOM    735  CD2 TYR    46     -14.572  -4.473 -24.414  1.00  0.00
```

ATOM 736 HD2 TYR 46 -13.643 -4.919 -24.059 1.00 0.00

ATOM 737 C TYR 46 -15.962 -4.891 -19.818 1.00 0.00

ATOM 738 O TYR 46 -17.027 -5.070 -19.261 1.00 0.00

ATOM 739 N ASP 47 -14.842 -5.296 -19.284 1.00 0.00

ATOM 740 H ASP 47 -13.952 -5.151 -19.740 1.00 0.00

ATOM 741 CA ASP 47 -14.855 -5.992 -17.967 1.00 0.00

ATOM 742 HA ASP 47 -15.866 -6.316 -17.720 1.00 0.00

ATOM 743 CB ASP 47 -13.950 -7.224 -18.032 1.00 0.00

ATOM 744 HB2 ASP 47 -12.956 -6.932 -18.372 1.00 0.00

ATOM 745 HB3 ASP 47 -13.877 -7.673 -17.041 1.00 0.00

ATOM 746 CG ASP 47 -14.544 -8.241 -19.009 1.00 0.00

ATOM 747 OD1 ASP 47 -15.704 -8.092 -19.356 1.00 0.00

ATOM 748 OD2 ASP 47 -13.828 -9.150 -19.396 1.00 0.00

ATOM 749 C ASP 47 -14.343 -5.042 -16.882 1.00 0.00

ATOM 750 O ASP 47 -13.220 -4.578 -16.929 1.00 0.00

ATOM 751 N ILE 48 -15.156 -4.747 -15.905 1.00 0.00

ATOM 752 H ILE 48 -16.090 -5.129 -15.858 1.00 0.00

ATOM 753 CA ILE 48 -14.714 -3.826 -14.820 1.00 0.00

ATOM 754 HA ILE 48 -14.466 -2.854 -15.246 1.00 0.00

ATOM 755 CB ILE 48 -15.842 -3.663 -13.799 1.00 0.00

ATOM 756 HB ILE 48 -16.078 -4.633 -13.360 1.00 0.00

ATOM 757 CG2 ILE 48 -15.397 -2.700 -12.697 1.00 0.00

ATOM 758 HG21 ILE 48 -15.160 -1.731 -13.135 1.00 0.00
ATOM 759 HG22 ILE 48 -16.200 -2.584 -11.969 1.00 0.00
ATOM 760 HG23 ILE 48 -14.512 -3.100 -12.200 1.00 0.00
ATOM 761 CG1 ILE 48 -17.084 -3.102 -14.495 1.00 0.00
ATOM 762 HG12 ILE 48 -16.884 -2.085 -14.832 1.00 0.00
ATOM 763 HG13 ILE 48 -17.333 -3.726 -15.354 1.00 0.00
ATOM 764 CD1 ILE 48 -18.258 -3.093 -13.514 1.00 0.00
ATOM 765 HD11 ILE 48 -18.010 -2.469 -12.655 1.00 0.00
ATOM 766 HD12 ILE 48 -19.143 -2.694 -14.009 1.00 0.00
ATOM 767 HD13 ILE 48 -18.458 -4.111 -13.177 1.00 0.00
ATOM 768 C ILE 48 -13.481 -4.410 -14.129 1.00 0.00
ATOM 769 O ILE 48 -12.526 -3.714 -13.849 1.00 0.00
ATOM 770 N ARG 49 -13.494 -5.686 -13.850 1.00 0.00
ATOM 771 H ARG 49 -14.284 -6.272 -14.080 1.00 0.00
ATOM 772 CA ARG 49 -12.323 -6.313 -13.177 1.00 0.00
ATOM 773 HA ARG 49 -11.463 -5.644 -13.212 1.00 0.00
ATOM 774 CB ARG 49 -12.665 -6.593 -11.712 1.00 0.00
ATOM 775 HB2 ARG 49 -13.550 -7.228 -11.659 1.00 0.00
ATOM 776 HB3 ARG 49 -11.827 -7.099 -11.233 1.00 0.00
ATOM 777 CG ARG 49 -12.942 -5.271 -10.992 1.00 0.00
ATOM 778 HG2 ARG 49 -13.700 -4.711 -11.539 1.00 0.00
ATOM 779 HG3 ARG 49 -13.298 -5.475 -9.982 1.00 0.00

ATOM	780	CD	ARG	49	-11.653	-4.449	-10.922	1.00	0.00
ATOM	781	HD2	ARG	49	-10.856	-5.035	-10.462	1.00	0.00
ATOM	782	HD3	ARG	49	-11.346	-4.142	-11.922	1.00	0.00
ATOM	783	NE	ARG	49	-11.886	-3.230	-10.098	1.00	0.00
ATOM	784	HE	ARG	49	-12.797	-3.016	-9.719	1.00	0.00
ATOM	785	CZ	ARG	49	-10.901	-2.411	-9.851	1.00	0.00
ATOM	786	NH1	ARG	49	-9.671	-2.782	-10.079	1.00	0.00
ATOM	787	HH11	ARG	49	-9.485	-3.704	-10.447	1.00	0.00
ATOM	788	HH12	ARG	49	-8.911	-2.146	-9.887	1.00	0.00
ATOM	789	NH2	ARG	49	-11.146	-1.220	-9.376	1.00	0.00
ATOM	790	HH21	ARG	49	-12.099	-0.936	-9.201	1.00	0.00
ATOM	791	HH22	ARG	49	-10.381	-0.589	-9.186	1.00	0.00
ATOM	792	C	ARG	49	-11.975	-7.628	-13.879	1.00	0.00
ATOM	793	O	ARG	49	-12.842	-8.346	-14.336	1.00	0.00
ATOM	794	N	SER	50	-10.713	-7.947	-13.970	1.00	0.00
ATOM	795	H	SER	50	-9.987	-7.355	-13.594	1.00	0.00
ATOM	796	CA	SER	50	-10.312	-9.214	-14.643	1.00	0.00
ATOM	797	HA	SER	50	-10.563	-9.176	-15.703	1.00	0.00
ATOM	798	CB	SER	50	-8.800	-9.407	-14.505	1.00	0.00
ATOM	799	HB2	SER	50	-8.543	-9.527	-13.453	1.00	0.00
ATOM	800	HB3	SER	50	-8.494	-10.297	-15.057	1.00	0.00
ATOM	801	OG	SER	50	-8.129	-8.272	-15.029	1.00	0.00

ATOM	802	HG	SER	50	-7.180	-8.393	-14.943	1.00	0.00
ATOM	803	C	SER	50	-11.039	-10.392	-13.990	1.00	0.00
ATOM	804	O	SER	50	-11.481	-11.307	-14.657	1.00	0.00
ATOM	805	N	THR	51	-11.166	-10.377	-12.692	1.00	0.00
ATOM	806	H	THR	51	-10.801	-9.621	-12.132	1.00	0.00
ATOM	807	CA	THR	51	-11.864	-11.496	-11.999	1.00	0.00
ATOM	808	HA	THR	51	-12.562	-11.988	-12.676	1.00	0.00
ATOM	809	CB	THR	51	-10.835	-12.525	-11.527	1.00	0.00
ATOM	810	HB	THR	51	-10.172	-12.781	-12.354	1.00	0.00
ATOM	811	CG2	THR	51	-10.014	-11.940	-10.378	1.00	0.00
ATOM	812	HG21	THR	51	-10.676	-11.685	-9.551	1.00	0.00
ATOM	813	HG22	THR	51	-9.281	-12.675	-10.043	1.00	0.00
ATOM	814	HG23	THR	51	-9.498	-11.043	-10.720	1.00	0.00
ATOM	815	OG1	THR	51	-11.506	-13.697	-11.084	1.00	0.00
ATOM	816	HG1	THR	51	-10.860	-14.343	-10.788	1.00	0.00
ATOM	817	C	THR	51	-12.630	-10.951	-10.792	1.00	0.00
ATOM	818	O	THR	51	-12.304	-9.910	-10.257	1.00	0.00
ATOM	819	N	ARG	52	-13.646	-11.646	-10.359	1.00	0.00
ATOM	820	H	ARG	52	-13.922	-12.513	-10.798	1.00	0.00
ATOM	821	CA	ARG	52	-14.432	-11.166	-9.187	1.00	0.00
ATOM	822	HA	ARG	52	-14.846	-10.179	-9.389	1.00	0.00
ATOM	823	CB	ARG	52	-15.585	-12.134	-8.915	1.00	0.00

ATOM 824 HB2 ARG 52 -15.191 -13.143 -8.786 1.00 0.00

ATOM 825 HB3 ARG 52 -16.109 -11.831 -8.009 1.00 0.00

ATOM 826 CG ARG 52 -16.556 -12.114 -10.097 1.00 0.00

ATOM 827 HG2 ARG 52 -16.872 -11.089 -10.291 1.00 0.00

ATOM 828 HG3 ARG 52 -16.061 -12.516 -10.982 1.00 0.00

ATOM 829 CD ARG 52 -17.781 -12.971 -9.765 1.00 0.00

ATOM 830 HD2 ARG 52 -18.313 -12.555 -8.910 1.00 0.00

ATOM 831 HD3 ARG 52 -18.452 -13.019 -10.622 1.00 0.00

ATOM 832 NE ARG 52 -17.341 -14.353 -9.426 1.00 0.00

ATOM 833 HE ARG 52 -16.424 -14.538 -9.045 1.00 0.00

ATOM 834 CZ ARG 52 -18.149 -15.360 -9.620 1.00 0.00

ATOM 835 NH1 ARG 52 -18.553 -16.077 -8.607 1.00 0.00

ATOM 836 HH11 ARG 52 -18.238 -15.851 -7.674 1.00 0.00

ATOM 837 HH12 ARG 52 -19.178 -16.856 -8.761 1.00 0.00

ATOM 838 NH2 ARG 52 -18.552 -15.649 -10.826 1.00 0.00

ATOM 839 HH21 ARG 52 -18.237 -15.092 -11.607 1.00 0.00

ATOM 840 HH22 ARG 52 -19.177 -16.429 -10.973 1.00 0.00

ATOM 841 C ARG 52 -13.525 -11.097 -7.957 1.00 0.00

ATOM 842 O ARG 52 -13.688 -10.249 -7.102 1.00 0.00

ATOM 843 N SER 53 -12.571 -11.981 -7.860 1.00 0.00

ATOM 844 H SER 53 -12.431 -12.689 -8.567 1.00 0.00

ATOM 845 CA SER 53 -11.655 -11.966 -6.685 1.00 0.00

ATOM	846	HA	SER	53	-12.218	-12.119	-5.765	1.00	0.00
ATOM	847	CB	SER	53	-10.629	-13.093	-6.822	1.00	0.00
ATOM	848	HB2	SER	53	-10.069	-13.192	-5.892	1.00	0.00
ATOM	849	HB3	SER	53	-11.143	-14.030	-7.037	1.00	0.00
ATOM	850	OG	SER	53	-9.734	-12.791	-7.883	1.00	0.00
ATOM	851	HG	SER	53	-9.090	-13.498	-7.969	1.00	0.00
ATOM	852	C	SER	53	-10.931	-10.620	-6.620	1.00	0.00
ATOM	853	O	SER	53	-10.432	-10.222	-5.585	1.00	0.00
ATOM	854	N	SER	54	-10.867	-9.915	-7.716	1.00	0.00
ATOM	855	H	SER	54	-11.279	-10.240	-8.579	1.00	0.00
ATOM	856	CA	SER	54	-10.174	-8.596	-7.716	1.00	0.00
ATOM	857	HA	SER	54	-9.107	-8.730	-7.536	1.00	0.00
ATOM	858	CB	SER	54	-10.360	-7.921	-9.076	1.00	0.00
ATOM	859	HB2	SER	54	-10.041	-8.601	-9.866	1.00	0.00
ATOM	860	HB3	SER	54	-11.411	-7.669	-9.216	1.00	0.00
ATOM	861	OG	SER	54	-9.579	-6.737	-9.128	1.00	0.00
ATOM	862	HG	SER	54	-9.696	-6.314	-9.982	1.00	0.00
ATOM	863	C	SER	54	-10.768	-7.708	-6.621	1.00	0.00
ATOM	864	O	SER	54	-10.085	-6.897	-6.028	1.00	0.00
ATOM	865	N	ASP	55	-12.035	-7.853	-6.349	1.00	0.00
ATOM	866	H	ASP	55	-12.609	-8.524	-6.839	1.00	0.00
ATOM	867	CA	ASP	55	-12.671	-7.016	-5.292	1.00	0.00

ATOM	868	HA	ASP	55	-12.569	-5.959	-5.535	1.00	0.00
ATOM	869	CB	ASP	55	-14.160	-7.356	-5.200	1.00	0.00
ATOM	870	HB2	ASP	55	-14.280	-8.433	-5.081	1.00	0.00
ATOM	871	HB3	ASP	55	-14.597	-6.846	-4.342	1.00	0.00
ATOM	872	CG	ASP	55	-14.868	-6.902	-6.478	1.00	0.00
ATOM	873	OD1	ASP	55	-14.255	-6.179	-7.246	1.00	0.00
ATOM	874	OD2	ASP	55	-16.011	-7.285	-6.667	1.00	0.00
ATOM	875	C	ASP	55	-12.000	-7.295	-3.946	1.00	0.00
ATOM	876	O	ASP	55	-11.998	-6.465	-3.059	1.00	0.00
ATOM	877	N	GLN	56	-11.429	-8.458	-3.787	1.00	0.00
ATOM	878	H	GLN	56	-11.426	-9.153	-4.520	1.00	0.00
ATOM	879	CA	GLN	56	-10.758	-8.788	-2.498	1.00	0.00
ATOM	880	HA	GLN	56	-11.460	-8.692	-1.670	1.00	0.00
ATOM	881	CB	GLN	56	-10.250	-10.230	-2.543	1.00	0.00
ATOM	882	HB2	GLN	56	-9.532	-10.338	-3.357	1.00	0.00
ATOM	883	HB3	GLN	56	-9.765	-10.475	-1.598	1.00	0.00
ATOM	884	CG	GLN	56	-11.427	-11.180	-2.773	1.00	0.00
ATOM	885	HG2	GLN	56	-12.267	-10.904	-2.136	1.00	0.00
ATOM	886	HG3	GLN	56	-11.738	-11.150	-3.817	1.00	0.00
ATOM	887	CD	GLN	56	-11.003	-12.610	-2.431	1.00	0.00
ATOM	888	OE1	GLN	56	-11.786	-13.381	-1.914	1.00	0.00
ATOM	889	NE2	GLN	56	-9.787	-12.999	-2.701	1.00	0.00

```
ATOM    890 HE21 GLN    56      -9.500 -13.942  -2.478  1.00  0.00
ATOM    891 HE22 GLN    56      -9.138 -12.356  -3.132  1.00  0.00
ATOM    892  C   GLN    56      -9.578  -7.840  -2.277  1.00  0.00
ATOM    893  O   GLN    56      -9.275  -7.460  -1.164  1.00  0.00
ATOM    894  N   GLN    57      -8.910  -7.455  -3.330  1.00  0.00
ATOM    895  H   GLN    57      -9.156  -7.768  -4.258  1.00  0.00
ATOM    896  CA  GLN    57      -7.749  -6.533  -3.180  1.00  0.00
ATOM    897  HA  GLN    57      -6.903  -7.059  -2.736  1.00  0.00
ATOM    898  CB  GLN    57      -7.332  -6.010  -4.556  1.00  0.00
ATOM    899  HB2 GLN    57      -8.178  -5.513  -5.029  1.00  0.00
ATOM    900  HB3 GLN    57      -6.511  -5.302  -4.444  1.00  0.00
ATOM    901  CG  GLN    57      -6.878  -7.182  -5.428  1.00  0.00
ATOM    902  HG2 GLN    57      -5.990  -7.649  -5.002  1.00  0.00
ATOM    903  HG3 GLN    57      -7.673  -7.923  -5.510  1.00  0.00
ATOM    904  CD  GLN    57      -6.538  -6.673  -6.830  1.00  0.00
ATOM    905  OE1 GLN    57      -6.932  -5.588  -7.208  1.00  0.00
ATOM    906  NE2 GLN    57      -5.816  -7.418  -7.623  1.00  0.00
ATOM    907 HE21 GLN    57      -5.587  -7.090  -8.550  1.00  0.00
ATOM    908 HE22 GLN    57      -5.489  -8.320  -7.306  1.00  0.00
ATOM    909  C   GLN    57      -8.144  -5.357  -2.283  1.00  0.00
ATOM    910  O   GLN    57      -7.415  -4.972  -1.391  1.00  0.00
ATOM    911  N   GLN    58      -9.293  -4.782  -2.513  1.00  0.00
```

ATOM	912	H	GLN	58	-9.905	-5.097	-3.252	1.00	0.00
ATOM	913	CA	GLN	58	-9.732	-3.633	-1.674	1.00	0.00
ATOM	914	HA	GLN	58	-9.150	-2.744	-1.917	1.00	0.00
ATOM	915	CB	GLN	58	-11.210	-3.339	-1.942	1.00	0.00
ATOM	916	HB2	GLN	58	-11.811	-4.200	-1.648	1.00	0.00
ATOM	917	HB3	GLN	58	-11.518	-2.468	-1.365	1.00	0.00
ATOM	918	CG	GLN	58	-11.413	-3.061	-3.433	1.00	0.00
ATOM	919	HG2	GLN	58	-10.630	-2.401	-3.807	1.00	0.00
ATOM	920	HG3	GLN	58	-11.400	-3.994	-3.997	1.00	0.00
ATOM	921	CD	GLN	58	-12.768	-2.381	-3.643	1.00	0.00
ATOM	922	OE1	GLN	58	-12.910	-1.539	-4.507	1.00	0.00
ATOM	923	NE2	GLN	58	-13.776	-2.714	-2.885	1.00	0.00
ATOM	924	HE21	GLN	58	-14.673	-2.270	-3.018	1.00	0.00
ATOM	925	HE22	GLN	58	-13.655	-3.416	-2.168	1.00	0.00
ATOM	926	C	GLN	58	-9.544	-3.980	-0.196	1.00	0.00
ATOM	927	O	GLN	58	-9.112	-3.163	0.593	1.00	0.00
ATOM	928	N	ARG	59	-9.866	-5.186	0.186	1.00	0.00
ATOM	929	H	ARG	59	-10.226	-5.871	-0.464	1.00	0.00
ATOM	930	CA	ARG	59	-9.708	-5.582	1.613	1.00	0.00
ATOM	931	HA	ARG	59	-10.444	-5.069	2.232	1.00	0.00
ATOM	932	CB	ARG	59	-9.921	-7.091	1.750	1.00	0.00
ATOM	933	HB2	ARG	59	-9.192	-7.620	1.135	1.00	0.00

```
ATOM    934  HB3 ARG    59      -9.796  -7.384   2.793  1.00  0.00
ATOM    935  CG  ARG    59     -11.334  -7.449   1.286  1.00  0.00
ATOM    936  HG2 ARG    59     -12.063  -6.895   1.877  1.00  0.00
ATOM    937  HG3 ARG    59     -11.448  -7.190   0.233  1.00  0.00
ATOM    938  CD  ARG    59     -11.565  -8.951   1.469  1.00  0.00
ATOM    939  HD2 ARG    59     -10.776  -9.518   0.975  1.00  0.00
ATOM    940  HD3 ARG    59     -11.584  -9.205   2.529  1.00  0.00
ATOM    941  NE  ARG    59     -12.874  -9.328   0.864  1.00  0.00
ATOM    942  HE  ARG    59     -12.967  -9.506  -0.126  1.00  0.00
ATOM    943  CZ  ARG    59     -13.934  -9.436   1.619  1.00  0.00
ATOM    944  NH1 ARG    59     -14.233 -10.583   2.164  1.00  0.00
ATOM    945 HH11 ARG    59     -13.642 -11.386   2.000  1.00  0.00
ATOM    946 HH12 ARG    59     -15.053 -10.663   2.747  1.00  0.00
ATOM    947  NH2 ARG    59     -14.694  -8.395   1.828  1.00  0.00
ATOM    948 HH21 ARG    59     -14.459  -7.509   1.404  1.00  0.00
ATOM    949 HH22 ARG    59     -15.513  -8.481   2.412  1.00  0.00
ATOM    950  C   ARG    59      -8.303  -5.218   2.092  1.00  0.00
ATOM    951  O   ARG    59      -8.089  -4.925   3.251  1.00  0.00
ATOM    952  N   CYX    60      -7.343  -5.227   1.210  1.00  0.00
ATOM    953  H   CYX    60      -7.510  -5.467   0.243  1.00  0.00
ATOM    954  CA  CYX    60      -5.955  -4.875   1.620  1.00  0.00
ATOM    955  HA  CYX    60      -5.505  -5.695   2.181  1.00  0.00
```

```
ATOM    956  CB  CYX    60      -5.108  -4.614   0.371  1.00  0.00
ATOM    957  HB2 CYX    60      -4.857  -5.563  -0.104  1.00  0.00
ATOM    958  HB3 CYX    60      -5.671  -3.996  -0.328  1.00  0.00
ATOM    959  SG  CYX    60      -3.550  -3.822   0.843  1.00  0.00
ATOM    960  C   CYX    60      -5.997  -3.615   2.487  1.00  0.00
ATOM    961  O   CYX    60      -6.085  -3.684   3.698  1.00  0.00
ATOM    962  N   CYX    61      -5.935  -2.467   1.875  1.00  0.00
ATOM    963  H   CYX    61      -5.858  -2.404   0.870  1.00  0.00
ATOM    964  CA  CYX    61      -5.973  -1.202   2.662  1.00  0.00
ATOM    965  HA  CYX    61      -5.001  -1.015   3.120  1.00  0.00
ATOM    966  CB  CYX    61      -6.313  -0.031   1.739  1.00  0.00
ATOM    967  HB2 CYX    61      -6.391  -0.388   0.712  1.00  0.00
ATOM    968  HB3 CYX    61      -7.262   0.408   2.044  1.00  0.00
ATOM    969  SG  CYX    61      -4.979   1.192   1.802  1.00  0.00
ATOM    970  C   CYX    61      -7.037  -1.319   3.756  1.00  0.00
ATOM    971  O   CYX    61      -6.850  -0.868   4.868  1.00  0.00
ATOM    972  N   ASP    62      -8.151  -1.923   3.447  1.00  0.00
ATOM    973  H   ASP    62      -8.312  -2.301   2.524  1.00  0.00
ATOM    974  CA  ASP    62      -9.227  -2.069   4.469  1.00  0.00
ATOM    975  HA  ASP    62      -9.557  -1.089   4.812  1.00  0.00
ATOM    976  CB  ASP    62     -10.421  -2.799   3.853  1.00  0.00
ATOM    977  HB2 ASP    62     -10.090  -3.749   3.433  1.00  0.00
```

ATOM	978	HB3	ASP	62	-11.170	-2.984	4.622	1.00	0.00
ATOM	979	CG	ASP	62	-11.030	-1.937	2.745	1.00	0.00
ATOM	980	OD1	ASP	62	-10.676	-0.771	2.667	1.00	0.00
ATOM	981	OD2	ASP	62	-11.839	-2.455	1.993	1.00	0.00
ATOM	982	C	ASP	62	-8.696	-2.872	5.656	1.00	0.00
ATOM	983	O	ASP	62	-9.026	-2.608	6.796	1.00	0.00
ATOM	984	N	GLU	63	-7.872	-3.848	5.400	1.00	0.00
ATOM	985	H	GLU	63	-7.593	-4.075	4.456	1.00	0.00
ATOM	986	CA	GLU	63	-7.315	-4.663	6.515	1.00	0.00
ATOM	987	HA	GLU	63	-8.122	-5.095	7.107	1.00	0.00
ATOM	988	CB	GLU	63	-6.466	-5.800	5.943	1.00	0.00
ATOM	989	HB2	GLU	63	-5.862	-5.423	5.117	1.00	0.00
ATOM	990	HB3	GLU	63	-5.811	-6.192	6.721	1.00	0.00
ATOM	991	CG	GLU	63	-7.381	-6.916	5.437	1.00	0.00
ATOM	992	HG2	GLU	63	-8.058	-7.221	6.235	1.00	0.00
ATOM	993	HG3	GLU	63	-7.961	-6.554	4.588	1.00	0.00
ATOM	994	CD	GLU	63	-6.534	-8.113	5.002	1.00	0.00
ATOM	995	OE1	GLU	63	-5.322	-8.031	5.122	1.00	0.00
ATOM	996	OE2	GLU	63	-7.111	-9.092	4.556	1.00	0.00
ATOM	997	C	GLU	63	-6.447	-3.770	7.402	1.00	0.00
ATOM	998	O	GLU	63	-6.589	-3.748	8.608	1.00	0.00
ATOM	999	N	LEU	64	-5.548	-3.032	6.811	1.00	0.00

```
ATOM   1000  H   LEU    64      -5.427  -3.048   5.808  1.00  0.00
ATOM   1001  CA  LEU    64      -4.668  -2.138   7.614  1.00  0.00
ATOM   1002  HA  LEU    64      -4.193  -2.698   8.420  1.00  0.00
ATOM   1003  CB  LEU    64      -3.576  -1.556   6.713  1.00  0.00
ATOM   1004  HB2 LEU    64      -4.026  -0.890   5.978  1.00  0.00
ATOM   1005  HB3 LEU    64      -2.863  -0.998   7.321  1.00  0.00
ATOM   1006  CG  LEU    64      -2.850  -2.693   5.992  1.00  0.00
ATOM   1007  HG  LEU    64      -3.541  -3.196   5.315  1.00  0.00
ATOM   1008  CD1 LEU    64      -1.678  -2.121   5.191  1.00  0.00
ATOM   1009 HD11 LEU    64      -0.986  -1.619   5.867  1.00  0.00
ATOM   1010 HD12 LEU    64      -1.159  -2.931   4.677  1.00  0.00
ATOM   1011 HD13 LEU    64      -2.052  -1.407   4.458  1.00  0.00
ATOM   1012  CD2 LEU    64      -2.322  -3.696   7.019  1.00  0.00
ATOM   1013 HD21 LEU    64      -3.155  -4.104   7.591  1.00  0.00
ATOM   1014 HD22 LEU    64      -1.805  -4.506   6.504  1.00  0.00
ATOM   1015 HD23 LEU    64      -1.629  -3.195   7.694  1.00  0.00
ATOM   1016  C   LEU    64      -5.495  -0.995   8.211  1.00  0.00
ATOM   1017  O   LEU    64      -4.984  -0.158   8.927  1.00  0.00
ATOM   1018  N   ASN    65      -6.769  -0.951   7.925  1.00  0.00
ATOM   1019  H   ASN    65      -7.205  -1.643   7.333  1.00  0.00
ATOM   1020  CA  ASN    65      -7.619   0.141   8.482  1.00  0.00
ATOM   1021  HA  ASN    65      -6.994   0.953   8.854  1.00  0.00
```

ATOM 1022 CB ASN 65 -8.531 0.690 7.383 1.00 0.00
ATOM 1023 HB2 ASN 65 -9.217 -0.086 7.042 1.00 0.00
ATOM 1024 HB3 ASN 65 -9.101 1.541 7.755 1.00 0.00
ATOM 1025 CG ASN 65 -7.685 1.150 6.197 1.00 0.00
ATOM 1026 OD1 ASN 65 -8.201 1.137 4.998 1.00 0.00
ATOM 1027 ND2 ASN 65 -6.540 1.525 6.360 1.00 0.00
ATOM 1028 HD21 ASN 65 -5.992 1.826 5.566 1.00 0.00
ATOM 1029 HD22 ASN 65 -6.142 1.533 7.288 1.00 0.00
ATOM 1030 C ASN 65 -8.478 -0.409 9.623 1.00 0.00
ATOM 1031 O ASN 65 -9.118 0.332 10.341 1.00 0.00
ATOM 1032 N GLU 66 -8.500 -1.703 9.795 1.00 0.00
ATOM 1033 H GLU 66 -7.971 -2.329 9.204 1.00 0.00
ATOM 1034 CA GLU 66 -9.324 -2.292 10.890 1.00 0.00
ATOM 1035 HA GLU 66 -9.499 -1.554 11.672 1.00 0.00
ATOM 1036 CB GLU 66 -10.675 -2.735 10.325 1.00 0.00
ATOM 1037 HB2 GLU 66 -10.524 -3.556 9.624 1.00 0.00
ATOM 1038 HB3 GLU 66 -11.318 -3.068 11.140 1.00 0.00
ATOM 1039 CG GLU 66 -11.336 -1.561 9.601 1.00 0.00
ATOM 1040 HG2 GLU 66 -11.592 -0.787 10.324 1.00 0.00
ATOM 1041 HG3 GLU 66 -10.646 -1.154 8.862 1.00 0.00
ATOM 1042 CD GLU 66 -12.607 -2.044 8.900 1.00 0.00
ATOM 1043 OE1 GLU 66 -12.795 -3.248 8.822 1.00 0.00

```
ATOM   1044  OE2 GLU    66     -13.370  -1.204   8.453  1.00  0.00
ATOM   1045  C   GLU    66      -8.604  -3.503  11.491  1.00  0.00
ATOM   1046  O   GLU    66      -8.725  -3.787  12.665  1.00  0.00
ATOM   1047  N   MET    67      -7.860  -4.222  10.696  1.00  0.00
ATOM   1048  H   MET    67      -7.752  -3.997   9.717  1.00  0.00
ATOM   1049  CA  MET    67      -7.142  -5.415  11.229  1.00  0.00
ATOM   1050  HA  MET    67      -7.807  -6.008  11.857  1.00  0.00
ATOM   1051  CB  MET    67      -6.666  -6.285  10.064  1.00  0.00
ATOM   1052  HB2 MET    67      -6.836  -5.761   9.124  1.00  0.00
ATOM   1053  HB3 MET    67      -5.602  -6.493  10.176  1.00  0.00
ATOM   1054  CG  MET    67      -7.445  -7.602  10.061  1.00  0.00
ATOM   1055  HG2 MET    67      -7.865  -7.785  11.050  1.00  0.00
ATOM   1056  HG3 MET    67      -8.250  -7.553   9.328  1.00  0.00
ATOM   1057  SD  MET    67      -6.330  -8.960   9.628  1.00  0.00
ATOM   1058  CE  MET    67      -6.220  -8.606   7.857  1.00  0.00
ATOM   1059  HE1 MET    67      -5.812  -7.606   7.710  1.00  0.00
ATOM   1060  HE2 MET    67      -5.567  -9.338   7.380  1.00  0.00
ATOM   1061  HE3 MET    67      -7.213  -8.662   7.412  1.00  0.00
ATOM   1062  C   MET    67      -5.937  -4.963  12.053  1.00  0.00
ATOM   1063  O   MET    67      -5.955  -4.993  13.268  1.00  0.00
ATOM   1064  N   GLU    68      -4.889  -4.547  11.402  1.00  0.00
ATOM   1065  H   GLU    68      -4.868  -4.523  10.392  1.00  0.00
```

ATOM	1066	CA	GLU	68	-3.679	-4.095	12.144	1.00	0.00
ATOM	1067	HA	GLU	68	-3.473	-4.767	12.978	1.00	0.00
ATOM	1068	CB	GLU	68	-2.473	-4.101	11.203	1.00	0.00
ATOM	1069	HB2	GLU	68	-2.564	-3.283	10.489	1.00	0.00
ATOM	1070	HB3	GLU	68	-1.558	-3.977	11.783	1.00	0.00
ATOM	1071	CG	GLU	68	-2.422	-5.431	10.449	1.00	0.00
ATOM	1072	HG2	GLU	68	-2.217	-6.240	11.151	1.00	0.00
ATOM	1073	HG3	GLU	68	-3.380	-5.609	9.961	1.00	0.00
ATOM	1074	CD	GLU	68	-1.315	-5.379	9.394	1.00	0.00
ATOM	1075	OE1	GLU	68	-0.746	-4.315	9.212	1.00	0.00
ATOM	1076	OE2	GLU	68	-1.054	-6.405	8.786	1.00	0.00
ATOM	1077	C	GLU	68	-3.902	-2.679	12.680	1.00	0.00
ATOM	1078	O	GLU	68	-3.020	-2.081	13.263	1.00	0.00
ATOM	1079	N	ASN	69	-5.074	-2.138	12.489	1.00	0.00
ATOM	1080	H	ASN	69	-5.814	-2.628	12.007	1.00	0.00
ATOM	1081	CA	ASN	69	-5.347	-0.760	12.989	1.00	0.00
ATOM	1082	HA	ASN	69	-6.271	-0.377	12.557	1.00	0.00
ATOM	1083	CB	ASN	69	-5.495	-0.791	14.512	1.00	0.00
ATOM	1084	HB2	ASN	69	-4.561	-1.110	14.976	1.00	0.00
ATOM	1085	HB3	ASN	69	-5.767	0.196	14.885	1.00	0.00
ATOM	1086	CG	ASN	69	-6.595	-1.780	14.898	1.00	0.00
ATOM	1087	OD1	ASN	69	-7.530	-1.992	14.150	1.00	0.00

ATOM 1088 ND2 ASN 69 -6.524 -2.401 16.044 1.00 0.00
ATOM 1089 HD21 ASN 69 -7.247 -3.056 16.306 1.00 0.00
ATOM 1090 HD22 ASN 69 -5.746 -2.224 16.664 1.00 0.00
ATOM 1091 C ASN 69 -4.184 0.158 12.606 1.00 0.00
ATOM 1092 O ASN 69 -3.927 1.152 13.256 1.00 0.00
ATOM 1093 N THR 70 -3.481 -0.169 11.558 1.00 0.00
ATOM 1094 H THR 70 -3.692 -0.995 11.015 1.00 0.00
ATOM 1095 CA THR 70 -2.333 0.683 11.132 1.00 0.00
ATOM 1096 HA THR 70 -1.466 0.499 11.766 1.00 0.00
ATOM 1097 CB THR 70 -1.960 0.349 9.685 1.00 0.00
ATOM 1098 HB THR 70 -0.951 0.707 9.479 1.00 0.00
ATOM 1099 CG2 THR 70 -2.015 -1.166 9.477 1.00 0.00
ATOM 1100 HG21 THR 70 -3.023 -1.526 9.683 1.00 0.00
ATOM 1101 HG22 THR 70 -1.749 -1.402 8.446 1.00 0.00
ATOM 1102 HG23 THR 70 -1.311 -1.652 10.153 1.00 0.00
ATOM 1103 OG1 THR 70 -2.874 0.984 8.802 1.00 0.00
ATOM 1104 HG1 THR 70 -2.640 0.774 7.895 1.00 0.00
ATOM 1105 C THR 70 -2.728 2.158 11.229 1.00 0.00
ATOM 1106 O THR 70 -3.894 2.500 11.236 1.00 0.00
ATOM 1107 N GLN 71 -1.765 3.037 11.305 1.00 0.00
ATOM 1108 H GLN 71 -0.793 2.761 11.300 1.00 0.00
ATOM 1109 CA GLN 71 -2.088 4.489 11.402 1.00 0.00

ATOM	1110	HA	GLN	71	-2.707	4.795	10.559	1.00	0.00
ATOM	1111	CB	GLN	71	-2.858	4.755	12.696	1.00	0.00
ATOM	1112	HB2	GLN	71	-3.798	4.204	12.680	1.00	0.00
ATOM	1113	HB3	GLN	71	-2.261	4.430	13.548	1.00	0.00
ATOM	1114	CG	GLN	71	-3.147	6.254	12.817	1.00	0.00
ATOM	1115	HG2	GLN	71	-2.235	6.828	12.654	1.00	0.00
ATOM	1116	HG3	GLN	71	-3.900	6.554	12.088	1.00	0.00
ATOM	1117	CD	GLN	71	-3.675	6.561	14.221	1.00	0.00
ATOM	1118	OE1	GLN	71	-3.074	6.181	15.206	1.00	0.00
ATOM	1119	NE2	GLN	71	-4.783	7.238	14.353	1.00	0.00
ATOM	1120	HE21	GLN	71	-5.138	7.444	15.276	1.00	0.00
ATOM	1121	HE22	GLN	71	-5.280	7.552	13.532	1.00	0.00
ATOM	1122	C	GLN	71	-0.791	5.302	11.404	1.00	0.00
ATOM	1123	O	GLN	71	-0.735	6.399	10.884	1.00	0.00
ATOM	1124	N	GLY	72	0.251	4.776	11.989	1.00	0.00
ATOM	1125	H	GLY	72	0.213	3.865	12.425	1.00	0.00
ATOM	1126	CA	GLY	72	1.540	5.523	12.025	1.00	0.00
ATOM	1127	HA2	GLY	72	1.382	6.551	11.699	1.00	0.00
ATOM	1128	HA3	GLY	72	1.942	5.520	13.038	1.00	0.00
ATOM	1129	C	GLY	72	2.548	4.853	11.088	1.00	0.00
ATOM	1130	O	GLY	72	2.410	3.698	10.735	1.00	0.00
ATOM	1131	N	CYX	73	3.562	5.568	10.685	1.00	0.00

ATOM	1132	H	CYX	73	3.682	6.528	10.975	1.00	0.00
ATOM	1133	CA	CYX	73	4.578	4.974	9.772	1.00	0.00
ATOM	1134	HA	CYX	73	5.400	5.672	9.615	1.00	0.00
ATOM	1135	CB	CYX	73	5.137	3.693	10.394	1.00	0.00
ATOM	1136	HB2	CYX	73	4.532	3.412	11.256	1.00	0.00
ATOM	1137	HB3	CYX	73	5.113	2.890	9.657	1.00	0.00
ATOM	1138	SG	CYX	73	6.827	3.988	10.973	1.00	0.00
ATOM	1139	C	CYX	73	3.927	4.647	8.425	1.00	0.00
ATOM	1140	O	CYX	73	4.512	3.995	7.583	1.00	0.00
ATOM	1141	N	MET	74	2.720	5.096	8.216	1.00	0.00
ATOM	1142	H	MET	74	2.229	5.639	8.911	1.00	0.00
ATOM	1143	CA	MET	74	2.032	4.811	6.926	1.00	0.00
ATOM	1144	HA	MET	74	1.668	3.783	6.910	1.00	0.00
ATOM	1145	CB	MET	74	0.838	5.756	6.765	1.00	0.00
ATOM	1146	HB2	MET	74	1.195	6.781	6.668	1.00	0.00
ATOM	1147	HB3	MET	74	0.274	5.482	5.873	1.00	0.00
ATOM	1148	CG	MET	74	-0.066	5.644	7.995	1.00	0.00
ATOM	1149	HG2	MET	74	-0.207	4.594	8.254	1.00	0.00
ATOM	1150	HG3	MET	74	0.389	6.167	8.836	1.00	0.00
ATOM	1151	SD	MET	74	-1.675	6.389	7.631	1.00	0.00
ATOM	1152	CE	MET	74	-2.545	4.859	7.209	1.00	0.00
ATOM	1153	HE1	MET	74	-2.529	4.183	8.065	1.00	0.00

ATOM	1154	HE2	MET	74	-3.577	5.088	6.946	1.00	0.00
ATOM	1155	HE3	MET	74	-2.052	4.382	6.362	1.00	0.00
ATOM	1156	C	MET	74	3.013	5.024	5.771	1.00	0.00
ATOM	1157	O	MET	74	3.239	4.142	4.966	1.00	0.00
ATOM	1158	N	CYX	75	3.599	6.187	5.684	1.00	0.00
ATOM	1159	H	CYX	75	3.418	6.926	6.348	1.00	0.00
ATOM	1160	CA	CYX	75	4.565	6.450	4.581	1.00	0.00
ATOM	1161	HA	CYX	75	4.036	6.573	3.636	1.00	0.00
ATOM	1162	CB	CYX	75	5.341	7.735	4.878	1.00	0.00
ATOM	1163	HB2	CYX	75	4.648	8.513	5.196	1.00	0.00
ATOM	1164	HB3	CYX	75	6.065	7.547	5.671	1.00	0.00
ATOM	1165	SG	CYX	75	6.163	8.304	3.370	1.00	0.00
ATOM	1166	C	CYX	75	5.541	5.279	4.468	1.00	0.00
ATOM	1167	O	CYX	75	5.750	4.733	3.402	1.00	0.00
ATOM	1168	N	GLU	76	6.140	4.883	5.559	1.00	0.00
ATOM	1169	H	GLU	76	5.974	5.328	6.450	1.00	0.00
ATOM	1170	CA	GLU	76	7.098	3.743	5.507	1.00	0.00
ATOM	1171	HA	GLU	76	7.941	3.987	4.861	1.00	0.00
ATOM	1172	CB	GLU	76	7.628	3.453	6.913	1.00	0.00
ATOM	1173	HB2	GLU	76	6.801	3.165	7.562	1.00	0.00
ATOM	1174	HB3	GLU	76	8.353	2.640	6.868	1.00	0.00
ATOM	1175	CG	GLU	76	8.302	4.707	7.472	1.00	0.00

ATOM 1176 HG2 GLU 76 9.054 5.059 6.766 1.00 0.00
ATOM 1177 HG3 GLU 76 7.554 5.484 7.625 1.00 0.00
ATOM 1178 CD GLU 76 8.973 4.376 8.807 1.00 0.00
ATOM 1179 OE1 GLU 76 8.966 3.213 9.179 1.00 0.00
ATOM 1180 OE2 GLU 76 9.481 5.289 9.435 1.00 0.00
ATOM 1181 C GLU 76 6.377 2.507 4.966 1.00 0.00
ATOM 1182 O GLU 76 6.993 1.566 4.504 1.00 0.00
ATOM 1183 N ALA 77 5.072 2.508 5.011 1.00 0.00
ATOM 1184 H ALA 77 4.554 3.286 5.392 1.00 0.00
ATOM 1185 CA ALA 77 4.310 1.339 4.492 1.00 0.00
ATOM 1186 HA ALA 77 4.779 0.391 4.759 1.00 0.00
ATOM 1187 CB ALA 77 2.889 1.359 5.060 1.00 0.00
ATOM 1188 HB1 ALA 77 2.404 2.297 4.793 1.00 0.00
ATOM 1189 HB2 ALA 77 2.321 0.525 4.647 1.00 0.00
ATOM 1190 HB3 ALA 77 2.930 1.267 6.145 1.00 0.00
ATOM 1191 C ALA 77 4.254 1.418 2.967 1.00 0.00
ATOM 1192 O ALA 77 4.712 0.531 2.273 1.00 0.00
ATOM 1193 N LEU 78 3.704 2.475 2.437 1.00 0.00
ATOM 1194 H LEU 78 3.322 3.218 3.004 1.00 0.00
ATOM 1195 CA LEU 78 3.632 2.609 0.956 1.00 0.00
ATOM 1196 HA LEU 78 2.986 1.836 0.539 1.00 0.00
ATOM 1197 CB LEU 78 3.056 3.978 0.590 1.00 0.00

ATOM	1198	HB2	LEU	78	2.098	4.115	1.091	1.00	0.00
ATOM	1199	HB3	LEU	78	3.746	4.760	0.907	1.00	0.00
ATOM	1200	CG	LEU	78	2.856	4.058	-0.923	1.00	0.00
ATOM	1201	HG	LEU	78	3.766	3.733	-1.428	1.00	0.00
ATOM	1202	CD1	LEU	78	1.694	3.151	-1.333	1.00	0.00
ATOM	1203	HD11	LEU	78	0.784	3.475	-0.829	1.00	0.00
ATOM	1204	HD12	LEU	78	1.551	3.208	-2.412	1.00	0.00
ATOM	1205	HD13	LEU	78	1.918	2.122	-1.050	1.00	0.00
ATOM	1206	CD2	LEU	78	2.541	5.501	-1.322	1.00	0.00
ATOM	1207	HD21	LEU	78	3.369	6.147	-1.031	1.00	0.00
ATOM	1208	HD22	LEU	78	2.399	5.557	-2.401	1.00	0.00
ATOM	1209	HD23	LEU	78	1.631	5.827	-0.818	1.00	0.00
ATOM	1210	C	LEU	78	5.042	2.476	0.380	1.00	0.00
ATOM	1211	O	LEU	78	5.265	1.776	-0.587	1.00	0.00
ATOM	1212	N	GLN	79	5.996	3.141	0.973	1.00	0.00
ATOM	1213	H	GLN	79	5.816	3.724	1.778	1.00	0.00
ATOM	1214	CA	GLN	79	7.392	3.050	0.466	1.00	0.00
ATOM	1215	HA	GLN	79	7.463	3.496	-0.526	1.00	0.00
ATOM	1216	CB	GLN	79	8.332	3.805	1.410	1.00	0.00
ATOM	1217	HB2	GLN	79	8.203	3.432	2.426	1.00	0.00
ATOM	1218	HB3	GLN	79	9.364	3.651	1.095	1.00	0.00
ATOM	1219	CG	GLN	79	8.004	5.299	1.369	1.00	0.00

```
ATOM   1220  HG2 GLN    79       8.175   5.695   0.368  1.00  0.00
ATOM   1221  HG3 GLN    79       6.966   5.467   1.653  1.00  0.00
ATOM   1222  CD  GLN    79       8.908   6.046   2.352  1.00  0.00
ATOM   1223  OE1 GLN    79       9.131   7.320   2.176  1.00  0.00
ATOM   1224  NE2 GLN    79       9.418   5.464   3.289  1.00  0.00
ATOM   1225 HE21 GLN    79      10.013   5.969   3.930  1.00  0.00
ATOM   1226 HE22 GLN    79       9.245   4.477   3.422  1.00  0.00
ATOM   1227  C   GLN    79       7.807   1.582   0.400  1.00  0.00
ATOM   1228  O   GLN    79       8.371   1.128  -0.575  1.00  0.00
ATOM   1229  N   GLN    80       7.524   0.832   1.429  1.00  0.00
ATOM   1230  H   GLN    80       7.052   1.199   2.242  1.00  0.00
ATOM   1231  CA  GLN    80       7.897  -0.610   1.421  1.00  0.00
ATOM   1232  HA  GLN    80       8.982  -0.719   1.402  1.00  0.00
ATOM   1233  CB  GLN    80       7.360  -1.286   2.684  1.00  0.00
ATOM   1234  HB2 GLN    80       7.684  -0.727   3.562  1.00  0.00
ATOM   1235  HB3 GLN    80       6.271  -1.309   2.650  1.00  0.00
ATOM   1236  CG  GLN    80       7.897  -2.716   2.764  1.00  0.00
ATOM   1237  HG2 GLN    80       7.437  -3.246   3.598  1.00  0.00
ATOM   1238  HG3 GLN    80       7.692  -3.251   1.837  1.00  0.00
ATOM   1239  CD  GLN    80       9.411  -2.681   2.983  1.00  0.00
ATOM   1240  OE1 GLN    80       9.898  -1.954   3.826  1.00  0.00
ATOM   1241  NE2 GLN    80      10.181  -3.441   2.253  1.00  0.00
```

```
ATOM   1242 HE21 GLN    80      11.181  -3.424   2.392  1.00  0.00
ATOM   1243 HE22 GLN    80       9.773  -4.044   1.552  1.00  0.00
ATOM   1244  C   GLN    80       7.287  -1.277   0.187  1.00  0.00
ATOM   1245  O   GLN    80       7.952  -1.996  -0.533  1.00  0.00
ATOM   1246  N   ILE    81       6.030  -1.038  -0.066  1.00  0.00
ATOM   1247  H   ILE    81       5.472  -0.440   0.526  1.00  0.00
ATOM   1248  CA  ILE    81       5.383  -1.653  -1.258  1.00  0.00
ATOM   1249  HA  ILE    81       5.252  -2.722  -1.090  1.00  0.00
ATOM   1250  CB  ILE    81       4.018  -1.002  -1.488  1.00  0.00
ATOM   1251  HB  ILE    81       4.142   0.076  -1.584  1.00  0.00
ATOM   1252  CG2 ILE    81       3.395  -1.559  -2.769  1.00  0.00
ATOM   1253 HG21 ILE    81       3.271  -2.638  -2.674  1.00  0.00
ATOM   1254 HG22 ILE    81       2.423  -1.095  -2.932  1.00  0.00
ATOM   1255 HG23 ILE    81       4.047  -1.343  -3.615  1.00  0.00
ATOM   1256  CG1 ILE    81       3.099  -1.306  -0.303  1.00  0.00
ATOM   1257 HG12 ILE    81       3.057  -2.384  -0.144  1.00  0.00
ATOM   1258 HG13 ILE    81       3.489  -0.822   0.592  1.00  0.00
ATOM   1259  CD1 ILE    81       1.693  -0.778  -0.597  1.00  0.00
ATOM   1260 HD11 ILE    81       1.303  -1.262  -1.492  1.00  0.00
ATOM   1261 HD12 ILE    81       1.039  -0.995   0.247  1.00  0.00
ATOM   1262 HD13 ILE    81       1.735   0.299  -0.756  1.00  0.00
ATOM   1263  C   ILE    81       6.273  -1.426  -2.480  1.00  0.00
```

ATOM 1264 O ILE 81 6.765 -2.359 -3.085 1.00 0.00
ATOM 1265 N MET 82 6.490 -0.192 -2.845 1.00 0.00
ATOM 1266 H MET 82 6.086 0.587 -2.346 1.00 0.00
ATOM 1267 CA MET 82 7.354 0.095 -4.023 1.00 0.00
ATOM 1268 HA MET 82 6.824 -0.135 -4.947 1.00 0.00
ATOM 1269 CB MET 82 7.728 1.579 -4.030 1.00 0.00
ATOM 1270 HB2 MET 82 8.383 1.793 -3.185 1.00 0.00
ATOM 1271 HB3 MET 82 8.245 1.819 -4.959 1.00 0.00
ATOM 1272 CG MET 82 6.459 2.426 -3.919 1.00 0.00
ATOM 1273 HG2 MET 82 6.016 2.300 -2.931 1.00 0.00
ATOM 1274 HG3 MET 82 6.702 3.476 -4.077 1.00 0.00
ATOM 1275 SD MET 82 5.270 1.895 -5.176 1.00 0.00
ATOM 1276 CE MET 82 6.255 2.351 -6.625 1.00 0.00
ATOM 1277 HE1 MET 82 7.196 1.800 -6.614 1.00 0.00
ATOM 1278 HE2 MET 82 5.703 2.106 -7.532 1.00 0.00
ATOM 1279 HE3 MET 82 6.461 3.421 -6.603 1.00 0.00
ATOM 1280 C MET 82 8.624 -0.750 -3.937 1.00 0.00
ATOM 1281 O MET 82 8.985 -1.445 -4.864 1.00 0.00
ATOM 1282 N GLU 83 9.300 -0.701 -2.825 1.00 0.00
ATOM 1283 H GLU 83 9.004 -0.129 -2.048 1.00 0.00
ATOM 1284 CA GLU 83 10.545 -1.504 -2.676 1.00 0.00
ATOM 1285 HA GLU 83 11.224 -1.308 -3.506 1.00 0.00

ATOM 1286 CB GLU 83 11.246 -1.121 -1.370 1.00 0.00

ATOM 1287 HB2 GLU 83 10.553 -1.239 -0.537 1.00 0.00

ATOM 1288 HB3 GLU 83 12.110 -1.768 -1.217 1.00 0.00

ATOM 1289 CG GLU 83 11.707 0.336 -1.448 1.00 0.00

ATOM 1290 HG2 GLU 83 12.338 0.472 -2.327 1.00 0.00

ATOM 1291 HG3 GLU 83 10.837 0.988 -1.521 1.00 0.00

ATOM 1292 CD GLU 83 12.503 0.687 -0.190 1.00 0.00

ATOM 1293 OE1 GLU 83 12.571 -0.146 0.697 1.00 0.00

ATOM 1294 OE2 GLU 83 13.033 1.785 -0.136 1.00 0.00

ATOM 1295 C GLU 83 10.185 -2.990 -2.647 1.00 0.00

ATOM 1296 O GLU 83 11.033 -3.848 -2.789 1.00 0.00

ATOM 1297 N ASN 84 8.930 -3.297 -2.468 1.00 0.00

ATOM 1298 H ASN 84 8.222 -2.588 -2.349 1.00 0.00

ATOM 1299 CA ASN 84 8.511 -4.725 -2.434 1.00 0.00

ATOM 1300 HA ASN 84 9.249 -5.323 -1.898 1.00 0.00

ATOM 1301 CB ASN 84 7.166 -4.847 -1.714 1.00 0.00

ATOM 1302 HB2 ASN 84 7.209 -4.348 -0.746 1.00 0.00

ATOM 1303 HB3 ASN 84 6.373 -4.404 -2.316 1.00 0.00

ATOM 1304 CG ASN 84 6.841 -6.324 -1.488 1.00 0.00

ATOM 1305 OD1 ASN 84 7.462 -6.978 -0.674 1.00 0.00

ATOM 1306 ND2 ASN 84 5.886 -6.883 -2.180 1.00 0.00

ATOM 1307 HD21 ASN 84 5.666 -7.859 -2.036 1.00 0.00

ATOM 1308 HD22 ASN 84 5.371 -6.338 -2.857 1.00 0.00
ATOM 1309 C ASN 84 8.374 -5.248 -3.865 1.00 0.00
ATOM 1310 O ASN 84 8.423 -6.438 -4.107 1.00 0.00
ATOM 1311 N GLN 85 8.201 -4.370 -4.816 1.00 0.00
ATOM 1312 H GLN 85 8.156 -3.379 -4.626 1.00 0.00
ATOM 1313 CA GLN 85 8.063 -4.826 -6.228 1.00 0.00
ATOM 1314 HA GLN 85 8.869 -5.514 -6.485 1.00 0.00
ATOM 1315 CB GLN 85 6.727 -5.553 -6.400 1.00 0.00
ATOM 1316 HB2 GLN 85 6.607 -6.288 -5.604 1.00 0.00
ATOM 1317 HB3 GLN 85 5.911 -4.832 -6.354 1.00 0.00
ATOM 1318 CG GLN 85 6.704 -6.262 -7.756 1.00 0.00
ATOM 1319 HG2 GLN 85 6.810 -5.538 -8.564 1.00 0.00
ATOM 1320 HG3 GLN 85 7.512 -6.992 -7.816 1.00 0.00
ATOM 1321 CD GLN 85 5.371 -6.993 -7.926 1.00 0.00
ATOM 1322 OE1 GLN 85 4.669 -7.233 -6.964 1.00 0.00
ATOM 1323 NE2 GLN 85 4.990 -7.360 -9.119 1.00 0.00
ATOM 1324 HE21 GLN 85 4.111 -7.843 -9.239 1.00 0.00
ATOM 1325 HE22 GLN 85 5.576 -7.160 -9.917 1.00 0.00
ATOM 1326 C GLN 85 8.109 -3.619 -7.171 1.00 0.00
ATOM 1327 O GLN 85 7.291 -3.484 -8.058 1.00 0.00
ATOM 1328 N CYX 86 9.063 -2.744 -6.992 1.00 0.00
ATOM 1329 H CYX 86 9.750 -2.846 -6.258 1.00 0.00

ATOM	1330	CA	CYX	86	9.156	-1.555	-7.888	1.00	0.00
ATOM	1331	HA	CYX	86	8.174	-1.312	-8.294	1.00	0.00
ATOM	1332	CB	CYX	86	9.669	-0.351	-7.095	1.00	0.00
ATOM	1333	HB2	CYX	86	9.873	0.474	-7.778	1.00	0.00
ATOM	1334	HB3	CYX	86	8.915	-0.045	-6.371	1.00	0.00
ATOM	1335	SG	CYX	86	11.221	-0.785	-6.271	1.00	0.00
ATOM	1336	C	CYX	86	10.122	-1.857	-9.034	1.00	0.00
ATOM	1337	O	CYX	86	10.235	-1.102	-9.979	1.00	0.00
ATOM	1338	N	ASP	87	10.824	-2.956	-8.957	1.00	0.00
ATOM	1339	H	ASP	87	10.737	-3.588	-8.174	1.00	0.00
ATOM	1340	CA	ASP	87	11.783	-3.305	-10.042	1.00	0.00
ATOM	1341	HA	ASP	87	12.671	-2.675	-9.980	1.00	0.00
ATOM	1342	CB	ASP	87	12.210	-4.767	-9.893	1.00	0.00
ATOM	1343	HB2	ASP	87	11.339	-5.379	-9.657	1.00	0.00
ATOM	1344	HB3	ASP	87	12.654	-5.113	-10.827	1.00	0.00
ATOM	1345	CG	ASP	87	13.237	-4.887	-8.765	1.00	0.00
ATOM	1346	OD1	ASP	87	13.670	-3.858	-8.272	1.00	0.00
ATOM	1347	OD2	ASP	87	13.573	-6.005	-8.413	1.00	0.00
ATOM	1348	C	ASP	87	11.111	-3.107	-11.402	1.00	0.00
ATOM	1349	O	ASP	87	11.669	-2.507	-12.300	1.00	0.00
ATOM	1350	N	ARG	88	9.917	-3.609	-11.563	1.00	0.00
ATOM	1351	H	ARG	88	9.448	-4.110	-10.822	1.00	0.00

ATOM 1352 CA ARG 88 9.212 -3.451 -12.866 1.00 0.00
ATOM 1353 HA ARG 88 9.815 -3.865 -13.675 1.00 0.00
ATOM 1354 CB ARG 88 7.878 -4.200 -12.818 1.00 0.00
ATOM 1355 HB2 ARG 88 7.293 -3.847 -11.969 1.00 0.00
ATOM 1356 HB3 ARG 88 7.326 -4.019 -13.740 1.00 0.00
ATOM 1357 CG ARG 88 8.140 -5.700 -12.667 1.00 0.00
ATOM 1358 HG2 ARG 88 8.730 -6.053 -13.513 1.00 0.00
ATOM 1359 HG3 ARG 88 8.687 -5.882 -11.742 1.00 0.00
ATOM 1360 CD ARG 88 6.808 -6.450 -12.627 1.00 0.00
ATOM 1361 HD2 ARG 88 6.147 -6.005 -11.884 1.00 0.00
ATOM 1362 HD3 ARG 88 6.328 -6.418 -13.605 1.00 0.00
ATOM 1363 NE ARG 88 7.048 -7.873 -12.260 1.00 0.00
ATOM 1364 HE ARG 88 6.760 -8.244 -11.365 1.00 0.00
ATOM 1365 CZ ARG 88 7.642 -8.674 -13.104 1.00 0.00
ATOM 1366 NH1 ARG 88 6.935 -9.485 -13.841 1.00 0.00
ATOM 1367 HH11 ARG 88 5.929 -9.492 -13.757 1.00 0.00
ATOM 1368 HH12 ARG 88 7.397 -10.103 -14.493 1.00 0.00
ATOM 1369 NH2 ARG 88 8.943 -8.661 -13.210 1.00 0.00
ATOM 1370 HH21 ARG 88 9.487 -8.031 -12.638 1.00 0.00
ATOM 1371 HH22 ARG 88 9.400 -9.282 -13.863 1.00 0.00
ATOM 1372 C ARG 88 8.953 -1.966 -13.134 1.00 0.00
ATOM 1373 O ARG 88 8.597 -1.577 -14.228 1.00 0.00

ATOM 1374 N LEU 89 9.132 -1.135 -12.145 1.00 0.00

ATOM 1375 H LEU 89 9.428 -1.451 -11.232 1.00 0.00

ATOM 1376 CA LEU 89 8.897 0.323 -12.347 1.00 0.00

ATOM 1377 HA LEU 89 7.870 0.499 -12.665 1.00 0.00

ATOM 1378 CB LEU 89 9.137 1.068 -11.032 1.00 0.00

ATOM 1379 HB2 LEU 89 8.650 0.534 -10.216 1.00 0.00

ATOM 1380 HB3 LEU 89 10.208 1.127 -10.839 1.00 0.00

ATOM 1381 CG LEU 89 8.559 2.480 -11.134 1.00 0.00

ATOM 1382 HG LEU 89 8.973 2.980 -12.010 1.00 0.00

ATOM 1383 CD1 LEU 89 7.037 2.399 -11.265 1.00 0.00

ATOM 1384 HD11 LEU 89 6.623 1.899 -10.390 1.00 0.00

ATOM 1385 HD12 LEU 89 6.624 3.405 -11.338 1.00 0.00

ATOM 1386 HD13 LEU 89 6.779 1.835 -12.161 1.00 0.00

ATOM 1387 CD2 LEU 89 8.918 3.273 -9.877 1.00 0.00

ATOM 1388 HD21 LEU 89 10.002 3.331 -9.781 1.00 0.00

ATOM 1389 HD22 LEU 89 8.506 4.279 -9.951 1.00 0.00

ATOM 1390 HD23 LEU 89 8.503 2.774 -9.001 1.00 0.00

ATOM 1391 C LEU 89 9.857 0.852 -13.415 1.00 0.00

ATOM 1392 O LEU 89 9.674 1.927 -13.950 1.00 0.00

ATOM 1393 N GLN 90 10.880 0.104 -13.728 1.00 0.00

ATOM 1394 H GLN 90 11.039 -0.792 -13.288 1.00 0.00

ATOM 1395 CA GLN 90 11.850 0.565 -14.761 1.00 0.00

ATOM 1396 HA GLN 90 12.493 1.346 -14.355 1.00 0.00

ATOM 1397 CB GLN 90 12.726 -0.611 -15.198 1.00 0.00

ATOM 1398 HB2 GLN 90 12.098 -1.480 -15.395 1.00 0.00

ATOM 1399 HB3 GLN 90 13.269 -0.344 -16.105 1.00 0.00

ATOM 1400 CG GLN 90 13.724 -0.945 -14.086 1.00 0.00

ATOM 1401 HG2 GLN 90 14.296 -0.058 -13.813 1.00 0.00

ATOM 1402 HG3 GLN 90 13.202 -1.325 -13.208 1.00 0.00

ATOM 1403 CD GLN 90 14.695 -2.019 -14.580 1.00 0.00

ATOM 1404 OE1 GLN 90 14.470 -2.633 -15.604 1.00 0.00

ATOM 1405 NE2 GLN 90 15.772 -2.275 -13.889 1.00 0.00

ATOM 1406 HE21 GLN 90 16.418 -2.983 -14.209 1.00 0.00

ATOM 1407 HE22 GLN 90 15.955 -1.765 -13.037 1.00 0.00

ATOM 1408 C GLN 90 11.088 1.108 -15.970 1.00 0.00

ATOM 1409 O GLN 90 11.586 1.929 -16.714 1.00 0.00

ATOM 1410 N ASP 91 9.880 0.656 -16.174 1.00 0.00

ATOM 1411 H ASP 91 9.459 -0.026 -15.559 1.00 0.00

ATOM 1412 CA ASP 91 9.086 1.145 -17.335 1.00 0.00

ATOM 1413 HA ASP 91 9.645 1.007 -18.261 1.00 0.00

ATOM 1414 CB ASP 91 7.781 0.354 -17.430 1.00 0.00

ATOM 1415 HB2 ASP 91 7.248 0.417 -16.481 1.00 0.00

ATOM 1416 HB3 ASP 91 7.160 0.770 -18.223 1.00 0.00

ATOM 1417 CG ASP 91 8.093 -1.110 -17.742 1.00 0.00

ATOM	1418	OD1	ASP	91	9.223	-1.389	-18.109	1.00	0.00
ATOM	1419	OD2	ASP	91	7.198	-1.929	-17.610	1.00	0.00
ATOM	1420	C	ASP	91	8.769	2.630	-17.146	1.00	0.00
ATOM	1421	O	ASP	91	8.499	3.084	-16.051	1.00	0.00
ATOM	1422	N	ARG	92	8.802	3.393	-18.205	1.00	0.00
ATOM	1423	H	ARG	92	9.028	3.024	-19.118	1.00	0.00
ATOM	1424	CA	ARG	92	8.503	4.848	-18.085	1.00	0.00
ATOM	1425	HA	ARG	92	9.079	5.285	-17.269	1.00	0.00
ATOM	1426	CB	ARG	92	8.882	5.557	-19.386	1.00	0.00
ATOM	1427	HB2	ARG	92	9.958	5.478	-19.542	1.00	0.00
ATOM	1428	HB3	ARG	92	8.360	5.088	-20.220	1.00	0.00
ATOM	1429	CG	ARG	92	8.486	7.031	-19.300	1.00	0.00
ATOM	1430	HG2	ARG	92	7.443	7.109	-18.994	1.00	0.00
ATOM	1431	HG3	ARG	92	9.118	7.535	-18.569	1.00	0.00
ATOM	1432	CD	ARG	92	8.666	7.689	-20.669	1.00	0.00
ATOM	1433	HD2	ARG	92	7.954	7.275	-21.383	1.00	0.00
ATOM	1434	HD3	ARG	92	8.518	8.766	-20.592	1.00	0.00
ATOM	1435	NE	ARG	92	10.046	7.431	-21.167	1.00	0.00
ATOM	1436	HE	ARG	92	10.644	6.742	-20.732	1.00	0.00
ATOM	1437	CZ	ARG	92	10.507	8.099	-22.188	1.00	0.00
ATOM	1438	NH1	ARG	92	10.242	9.371	-22.308	1.00	0.00
ATOM	1439	HH11	ARG	92	9.680	9.834	-21.609	1.00	0.00

ATOM	1440	HH12	ARG	92	10.601	9.885	-23.100	1.00	0.00
ATOM	1441	NH2	ARG	92	11.235	7.496	-23.088	1.00	0.00
ATOM	1442	HH21	ARG	92	11.440	6.511	-22.991	1.00	0.00
ATOM	1443	HH22	ARG	92	11.591	8.016	-23.877	1.00	0.00
ATOM	1444	C	ARG	92	7.009	5.041	-17.816	1.00	0.00
ATOM	1445	O	ARG	92	6.576	6.091	-17.383	1.00	0.00
ATOM	1446	N	GLN	93	6.216	4.037	-18.070	1.00	0.00
ATOM	1447	H	GLN	93	6.568	3.161	-18.431	1.00	0.00
ATOM	1448	CA	GLN	93	4.752	4.164	-17.830	1.00	0.00
ATOM	1449	HA	GLN	93	4.395	5.135	-18.170	1.00	0.00
ATOM	1450	CB	GLN	93	4.013	3.071	-18.608	1.00	0.00
ATOM	1451	HB2	GLN	93	4.373	3.050	-19.637	1.00	0.00
ATOM	1452	HB3	GLN	93	4.197	2.103	-18.140	1.00	0.00
ATOM	1453	CG	GLN	93	2.511	3.365	-18.599	1.00	0.00
ATOM	1454	HG2	GLN	93	2.113	3.275	-17.588	1.00	0.00
ATOM	1455	HG3	GLN	93	2.321	4.369	-18.976	1.00	0.00
ATOM	1456	CD	GLN	93	1.790	2.360	-19.500	1.00	0.00
ATOM	1457	OE1	GLN	93	2.419	1.622	-20.232	1.00	0.00
ATOM	1458	NE2	GLN	93	0.487	2.299	-19.476	1.00	0.00
ATOM	1459	HE21	GLN	93	0.005	1.638	-20.068	1.00	0.00
ATOM	1460	HE22	GLN	93	-0.033	2.913	-18.866	1.00	0.00
ATOM	1461	C	GLN	93	4.461	4.012	-16.335	1.00	0.00

ATOM	1462	O	GLN	93	3.785	4.828	-15.740	1.00	0.00
ATOM	1463	N	MET	94	4.965	2.975	-15.724	1.00	0.00
ATOM	1464	H	MET	94	5.530	2.294	-16.212	1.00	0.00
ATOM	1465	CA	MET	94	4.715	2.772	-14.269	1.00	0.00
ATOM	1466	HA	MET	94	3.650	2.628	-14.085	1.00	0.00
ATOM	1467	CB	MET	94	5.465	1.529	-13.789	1.00	0.00
ATOM	1468	HB2	MET	94	6.494	1.564	-14.149	1.00	0.00
ATOM	1469	HB3	MET	94	5.463	1.502	-12.699	1.00	0.00
ATOM	1470	CG	MET	94	4.779	0.274	-14.331	1.00	0.00
ATOM	1471	HG2	MET	94	4.459	0.444	-15.359	1.00	0.00
ATOM	1472	HG3	MET	94	5.473	-0.567	-14.302	1.00	0.00
ATOM	1473	SD	MET	94	3.333	-0.112	-13.313	1.00	0.00
ATOM	1474	CE	MET	94	4.182	-0.171	-11.715	1.00	0.00
ATOM	1475	HE1	MET	94	4.646	0.794	-11.513	1.00	0.00
ATOM	1476	HE2	MET	94	3.463	-0.400	-10.929	1.00	0.00
ATOM	1477	HE3	MET	94	4.950	-0.944	-11.739	1.00	0.00
ATOM	1478	C	MET	94	5.208	3.993	-13.488	1.00	0.00
ATOM	1479	O	MET	94	4.578	4.437	-12.550	1.00	0.00
ATOM	1480	N	VAL	95	6.336	4.533	-13.864	1.00	0.00
ATOM	1481	H	VAL	95	6.867	4.164	-14.640	1.00	0.00
ATOM	1482	CA	VAL	95	6.875	5.721	-13.140	1.00	0.00
ATOM	1483	HA	VAL	95	7.372	5.409	-12.221	1.00	0.00

ATOM 1484 CB VAL 95 7.893 6.440 -14.027 1.00 0.00
ATOM 1485 HB VAL 95 7.438 6.671 -14.990 1.00 0.00
ATOM 1486 CG1 VAL 95 8.335 7.737 -13.348 1.00 0.00
ATOM 1487 HG11 VAL 95 8.791 7.506 -12.385 1.00 0.00
ATOM 1488 HG12 VAL 95 9.061 8.249 -13.980 1.00 0.00
ATOM 1489 HG13 VAL 95 7.469 8.380 -13.194 1.00 0.00
ATOM 1490 CG2 VAL 95 9.110 5.537 -14.241 1.00 0.00
ATOM 1491 HG21 VAL 95 8.796 4.611 -14.725 1.00 0.00
ATOM 1492 HG22 VAL 95 9.836 6.048 -14.873 1.00 0.00
ATOM 1493 HG23 VAL 95 9.566 5.306 -13.278 1.00 0.00
ATOM 1494 C VAL 95 5.733 6.680 -12.793 1.00 0.00
ATOM 1495 O VAL 95 5.106 6.563 -11.759 1.00 0.00
ATOM 1496 N GLN 96 5.461 7.628 -13.646 1.00 0.00
ATOM 1497 H GLN 96 5.980 7.734 -14.506 1.00 0.00
ATOM 1498 CA GLN 96 4.362 8.593 -13.360 1.00 0.00
ATOM 1499 HA GLN 96 4.601 9.187 -12.478 1.00 0.00
ATOM 1500 CB GLN 96 4.187 9.536 -14.553 1.00 0.00
ATOM 1501 HB2 GLN 96 3.903 8.959 -15.433 1.00 0.00
ATOM 1502 HB3 GLN 96 3.408 10.264 -14.329 1.00 0.00
ATOM 1503 CG GLN 96 5.504 10.265 -14.825 1.00 0.00
ATOM 1504 HG2 GLN 96 5.841 10.789 -13.930 1.00 0.00
ATOM 1505 HG3 GLN 96 6.270 9.555 -15.139 1.00 0.00

ATOM	1506	CD	GLN	96	5.298	11.289	-15.943	1.00	0.00
ATOM	1507	OE1	GLN	96	4.578	12.254	-15.775	1.00	0.00
ATOM	1508	NE2	GLN	96	5.906	11.121	-17.086	1.00	0.00
ATOM	1509	HE21	GLN	96	5.775	11.793	-17.828	1.00	0.00
ATOM	1510	HE22	GLN	96	6.505	10.319	-17.223	1.00	0.00
ATOM	1511	C	GLN	96	3.061	7.831	-13.116	1.00	0.00
ATOM	1512	O	GLN	96	2.421	7.982	-12.094	1.00	0.00
ATOM	1513	N	GLN	97	2.665	7.014	-14.048	1.00	0.00
ATOM	1514	H	GLN	97	3.192	6.889	-14.900	1.00	0.00
ATOM	1515	CA	GLN	97	1.405	6.238	-13.877	1.00	0.00
ATOM	1516	HA	GLN	97	0.540	6.883	-14.027	1.00	0.00
ATOM	1517	CB	GLN	97	1.359	5.110	-14.910	1.00	0.00
ATOM	1518	HB2	GLN	97	1.685	5.489	-15.879	1.00	0.00
ATOM	1519	HB3	GLN	97	2.020	4.301	-14.597	1.00	0.00
ATOM	1520	CG	GLN	97	-0.073	4.583	-15.024	1.00	0.00
ATOM	1521	HG2	GLN	97	-0.109	3.724	-15.695	1.00	0.00
ATOM	1522	HG3	GLN	97	-0.447	4.292	-14.042	1.00	0.00
ATOM	1523	CD	GLN	97	-0.977	5.681	-15.586	1.00	0.00
ATOM	1524	OE1	GLN	97	-0.855	6.057	-16.735	1.00	0.00
ATOM	1525	NE2	GLN	97	-1.888	6.214	-14.818	1.00	0.00
ATOM	1526	HE21	GLN	97	-2.489	6.939	-15.182	1.00	0.00
ATOM	1527	HE22	GLN	97	-1.987	5.898	-13.864	1.00	0.00

ATOM 1528 C GLN 97 1.354 5.646 -12.467 1.00 0.00
ATOM 1529 O GLN 97 0.556 6.048 -11.643 1.00 0.00
ATOM 1530 N PHE 98 2.201 4.695 -12.181 1.00 0.00
ATOM 1531 H PHE 98 2.868 4.349 -12.856 1.00 0.00
ATOM 1532 CA PHE 98 2.193 4.086 -10.820 1.00 0.00
ATOM 1533 HA PHE 98 1.271 3.525 -10.668 1.00 0.00
ATOM 1534 CB PHE 98 3.379 3.129 -10.664 1.00 0.00
ATOM 1535 HB2 PHE 98 3.426 2.399 -11.473 1.00 0.00
ATOM 1536 HB3 PHE 98 4.330 3.658 -10.598 1.00 0.00
ATOM 1537 CG PHE 98 3.231 2.342 -9.379 1.00 0.00
ATOM 1538 CD1 PHE 98 2.031 2.390 -8.654 1.00 0.00
ATOM 1539 HD1 PHE 98 1.201 2.995 -9.017 1.00 0.00
ATOM 1540 CE1 PHE 98 1.900 1.662 -7.467 1.00 0.00
ATOM 1541 HE1 PHE 98 0.966 1.700 -6.906 1.00 0.00
ATOM 1542 CZ PHE 98 2.965 0.885 -6.999 1.00 0.00
ATOM 1543 HZ PHE 98 2.862 0.318 -6.073 1.00 0.00
ATOM 1544 CE2 PHE 98 4.164 0.835 -7.720 1.00 0.00
ATOM 1545 HE2 PHE 98 4.994 0.229 -7.356 1.00 0.00
ATOM 1546 CD2 PHE 98 4.297 1.563 -8.909 1.00 0.00
ATOM 1547 HD2 PHE 98 5.231 1.524 -9.470 1.00 0.00
ATOM 1548 C PHE 98 2.292 5.195 -9.774 1.00 0.00
ATOM 1549 O PHE 98 1.662 5.143 -8.737 1.00 0.00

ATOM	1550	N	LYS	99	3.077	6.201	-10.042	1.00	0.00
ATOM	1551	H	LYS	99	3.603	6.249	-10.903	1.00	0.00
ATOM	1552	CA	LYS	99	3.214	7.318	-9.067	1.00	0.00
ATOM	1553	HA	LYS	99	3.508	6.932	-8.091	1.00	0.00
ATOM	1554	CB	LYS	99	4.289	8.292	-9.551	1.00	0.00
ATOM	1555	HB2	LYS	99	5.202	7.742	-9.779	1.00	0.00
ATOM	1556	HB3	LYS	99	3.940	8.802	-10.449	1.00	0.00
ATOM	1557	CG	LYS	99	4.574	9.323	-8.456	1.00	0.00
ATOM	1558	HG2	LYS	99	3.647	9.824	-8.177	1.00	0.00
ATOM	1559	HG3	LYS	99	4.993	8.821	-7.583	1.00	0.00
ATOM	1560	CD	LYS	99	5.574	10.356	-8.979	1.00	0.00
ATOM	1561	HD2	LYS	99	6.521	9.865	-9.203	1.00	0.00
ATOM	1562	HD3	LYS	99	5.181	10.816	-9.885	1.00	0.00
ATOM	1563	CE	LYS	99	5.797	11.433	-7.915	1.00	0.00
ATOM	1564	HE2	LYS	99	5.617	12.416	-8.349	1.00	0.00
ATOM	1565	HE3	LYS	99	5.110	11.271	-7.084	1.00	0.00
ATOM	1566	NZ	LYS	99	7.200	11.359	-7.420	1.00	0.00
ATOM	1567	HZ1	LYS	99	7.837	11.509	-8.190	1.00	0.00
ATOM	1568	HZ2	LYS	99	7.347	12.069	-6.718	1.00	0.00
ATOM	1569	HZ3	LYS	99	7.368	10.448	-7.017	1.00	0.00
ATOM	1570	C	LYS	99	1.876	8.050	-8.952	1.00	0.00
ATOM	1571	O	LYS	99	1.512	8.540	-7.902	1.00	0.00

ATOM	1572	N	ARG	100	1.139	8.121	-10.025	1.00	0.00
ATOM	1573	H	ARG	100	1.437	7.715	-10.901	1.00	0.00
ATOM	1574	CA	ARG	100	-0.179	8.813	-9.979	1.00	0.00
ATOM	1575	HA	ARG	100	-0.067	9.809	-9.551	1.00	0.00
ATOM	1576	CB	ARG	100	-0.737	8.950	-11.397	1.00	0.00
ATOM	1577	HB2	ARG	100	-0.555	8.029	-11.951	1.00	0.00
ATOM	1578	HB3	ARG	100	-1.809	9.139	-11.350	1.00	0.00
ATOM	1579	CG	ARG	100	-0.044	10.116	-12.105	1.00	0.00
ATOM	1580	HG2	ARG	100	-0.191	11.029	-11.529	1.00	0.00
ATOM	1581	HG3	ARG	100	1.023	9.907	-12.189	1.00	0.00
ATOM	1582	CD	ARG	100	-0.641	10.293	-13.503	1.00	0.00
ATOM	1583	HD2	ARG	100	-0.734	9.326	-13.999	1.00	0.00
ATOM	1584	HD3	ARG	100	-1.622	10.763	-13.438	1.00	0.00
ATOM	1585	NE	ARG	100	0.253	11.164	-14.318	1.00	0.00
ATOM	1586	HE	ARG	100	1.159	10.845	-14.631	1.00	0.00
ATOM	1587	CZ	ARG	100	-0.129	12.368	-14.643	1.00	0.00
ATOM	1588	NH1	ARG	100	0.497	13.405	-14.157	1.00	0.00
ATOM	1589	HH11	ARG	100	1.277	13.271	-13.529	1.00	0.00
ATOM	1590	HH12	ARG	100	0.199	14.335	-14.411	1.00	0.00
ATOM	1591	NH2	ARG	100	-1.137	12.536	-15.454	1.00	0.00
ATOM	1592	HH21	ARG	100	-1.619	11.731	-15.829	1.00	0.00
ATOM	1593	HH22	ARG	100	-1.430	13.469	-15.704	1.00	0.00

ATOM	1594	C	ARG	100	-1.143	7.991	-9.125	1.00	0.00
ATOM	1595	O	ARG	100	-1.744	8.490	-8.194	1.00	0.00
ATOM	1596	N	GLU	101	-1.287	6.728	-9.424	1.00	0.00
ATOM	1597	H	GLU	101	-0.788	6.302	-10.192	1.00	0.00
ATOM	1598	CA	GLU	101	-2.202	5.874	-8.618	1.00	0.00
ATOM	1599	HA	GLU	101	-3.216	6.270	-8.653	1.00	0.00
ATOM	1600	CB	GLU	101	-2.212	4.454	-9.187	1.00	0.00
ATOM	1601	HB2	GLU	101	-1.191	4.074	-9.234	1.00	0.00
ATOM	1602	HB3	GLU	101	-2.809	3.809	-8.543	1.00	0.00
ATOM	1603	CG	GLU	101	-2.815	4.469	-10.593	1.00	0.00
ATOM	1604	HG2	GLU	101	-2.144	4.997	-11.270	1.00	0.00
ATOM	1605	HG3	GLU	101	-2.951	3.445	-10.943	1.00	0.00
ATOM	1606	CD	GLU	101	-4.169	5.181	-10.559	1.00	0.00
ATOM	1607	OE1	GLU	101	-4.879	5.017	-9.581	1.00	0.00
ATOM	1608	OE2	GLU	101	-4.474	5.878	-11.514	1.00	0.00
ATOM	1609	C	GLU	101	-1.700	5.843	-7.176	1.00	0.00
ATOM	1610	O	GLU	101	-2.469	5.859	-6.235	1.00	0.00
ATOM	1611	N	LEU	102	-0.407	5.800	-6.997	1.00	0.00
ATOM	1612	H	LEU	102	0.235	5.784	-7.777	1.00	0.00
ATOM	1613	CA	LEU	102	0.156	5.771	-5.620	1.00	0.00
ATOM	1614	HA	LEU	102	-0.147	4.859	-5.106	1.00	0.00
ATOM	1615	CB	LEU	102	1.684	5.805	-5.695	1.00	0.00

ATOM 1616 HB2 LEU 102 1.997 5.747 -6.738 1.00 0.00
ATOM 1617 HB3 LEU 102 2.047 6.735 -5.259 1.00 0.00
ATOM 1618 CG LEU 102 2.266 4.618 -4.921 1.00 0.00
ATOM 1619 HG LEU 102 2.183 4.807 -3.851 1.00 0.00
ATOM 1620 CD1 LEU 102 1.495 3.344 -5.275 1.00 0.00
ATOM 1621 HD11 LEU 102 1.579 3.153 -6.345 1.00 0.00
ATOM 1622 HD12 LEU 102 1.912 2.501 -4.723 1.00 0.00
ATOM 1623 HD13 LEU 102 0.445 3.468 -5.010 1.00 0.00
ATOM 1624 CD2 LEU 102 3.739 4.441 -5.295 1.00 0.00
ATOM 1625 HD21 LEU 102 4.290 5.346 -5.041 1.00 0.00
ATOM 1626 HD22 LEU 102 4.155 3.596 -4.745 1.00 0.00
ATOM 1627 HD23 LEU 102 3.823 4.253 -6.366 1.00 0.00
ATOM 1628 C LEU 102 -0.345 6.986 -4.838 1.00 0.00
ATOM 1629 O LEU 102 -0.634 6.902 -3.663 1.00 0.00
ATOM 1630 N MET 103 -0.457 8.116 -5.483 1.00 0.00
ATOM 1631 H MET 103 -0.223 8.197 -6.462 1.00 0.00
ATOM 1632 CA MET 103 -0.946 9.329 -4.770 1.00 0.00
ATOM 1633 HA MET 103 -0.313 9.541 -3.908 1.00 0.00
ATOM 1634 CB MET 103 -0.904 10.531 -5.715 1.00 0.00
ATOM 1635 HB2 MET 103 -1.356 10.260 -6.669 1.00 0.00
ATOM 1636 HB3 MET 103 -1.456 11.360 -5.274 1.00 0.00
ATOM 1637 CG MET 103 0.551 10.950 -5.941 1.00 0.00

ATOM	1638	HG2	MET	103	1.030	11.151	-4.983	1.00	0.00
ATOM	1639	HG3	MET	103	1.089	10.153	-6.455	1.00	0.00
ATOM	1640	SD	MET	103	0.596	12.449	-6.955	1.00	0.00
ATOM	1641	CE	MET	103	0.337	13.637	-5.616	1.00	0.00
ATOM	1642	HE1	MET	103	1.143	13.545	-4.887	1.00	0.00
ATOM	1643	HE2	MET	103	0.330	14.648	-6.023	1.00	0.00
ATOM	1644	HE3	MET	103	-0.617	13.434	-5.129	1.00	0.00
ATOM	1645	C	MET	103	-2.385	9.091	-4.310	1.00	0.00
ATOM	1646	O	MET	103	-2.814	9.594	-3.291	1.00	0.00
ATOM	1647	N	SER	104	-3.132	8.321	-5.054	1.00	0.00
ATOM	1648	H	SER	104	-2.779	7.899	-5.901	1.00	0.00
ATOM	1649	CA	SER	104	-4.541	8.044	-4.662	1.00	0.00
ATOM	1650	HA	SER	104	-5.005	8.943	-4.259	1.00	0.00
ATOM	1651	CB	SER	104	-5.333	7.594	-5.891	1.00	0.00
ATOM	1652	HB2	SER	104	-6.374	7.431	-5.614	1.00	0.00
ATOM	1653	HB3	SER	104	-5.278	8.363	-6.661	1.00	0.00
ATOM	1654	OG	SER	104	-4.782	6.384	-6.391	1.00	0.00
ATOM	1655	HG	SER	104	-5.278	6.101	-7.162	1.00	0.00
ATOM	1656	C	SER	104	-4.565	6.937	-3.606	1.00	0.00
ATOM	1657	O	SER	104	-5.423	6.903	-2.747	1.00	0.00
ATOM	1658	N	LEU	105	-3.631	6.027	-3.667	1.00	0.00
ATOM	1659	H	LEU	105	-2.920	6.047	-4.384	1.00	0.00

```
ATOM   1660  CA  LEU   105      -3.599   4.919  -2.672  1.00  0.00
ATOM   1661  HA  LEU   105      -4.347   4.166  -2.921  1.00  0.00
ATOM   1662  CB  LEU   105      -2.218   4.258  -2.684  1.00  0.00
ATOM   1663  HB2 LEU   105      -1.556   4.822  -3.341  1.00  0.00
ATOM   1664  HB3 LEU   105      -1.808   4.252  -1.674  1.00  0.00
ATOM   1665  CG  LEU   105      -2.338   2.819  -3.194  1.00  0.00
ATOM   1666  HG  LEU   105      -1.400   2.292  -3.017  1.00  0.00
ATOM   1667  CD1 LEU   105      -3.472   2.103  -2.456  1.00  0.00
ATOM   1668 HD11 LEU   105      -4.410   2.628  -2.634  1.00  0.00
ATOM   1669 HD12 LEU   105      -3.554   1.079  -2.821  1.00  0.00
ATOM   1670 HD13 LEU   105      -3.259   2.090  -1.387  1.00  0.00
ATOM   1671  CD2 LEU   105      -2.634   2.833  -4.694  1.00  0.00
ATOM   1672 HD21 LEU   105      -1.824   3.338  -5.220  1.00  0.00
ATOM   1673 HD22 LEU   105      -2.719   1.809  -5.058  1.00  0.00
ATOM   1674 HD23 LEU   105      -3.570   3.361  -4.875  1.00  0.00
ATOM   1675  C   LEU   105      -3.891   5.464  -1.272  1.00  0.00
ATOM   1676  O   LEU   105      -4.734   4.949  -0.565  1.00  0.00
ATOM   1677  N   PRO   106      -3.183   6.530  -0.861  1.00  0.00
ATOM   1678  CD  PRO   106      -2.147   7.228  -1.643  1.00  0.00
ATOM   1679  HD2 PRO   106      -2.497   7.381  -2.664  1.00  0.00
ATOM   1680  HD3 PRO   106      -1.234   6.632  -1.659  1.00  0.00
ATOM   1681  CG  PRO   106      -1.976   8.518  -0.891  1.00  0.00
```

ATOM	1682	HG2	PRO	106	-2.663	9.266	-1.287	1.00	0.00
ATOM	1683	HG3	PRO	106	-0.951	8.872	-1.001	1.00	0.00
ATOM	1684	CB	PRO	106	-2.279	8.200	0.532	1.00	0.00
ATOM	1685	HB2	PRO	106	-2.665	9.102	1.006	1.00	0.00
ATOM	1686	HB3	PRO	106	-1.434	7.820	1.108	1.00	0.00
ATOM	1687	CA	PRO	106	-3.378	7.140	0.458	1.00	0.00
ATOM	1688	HA	PRO	106	-3.280	6.365	1.218	1.00	0.00
ATOM	1689	C	PRO	106	-4.757	7.789	0.568	1.00	0.00
ATOM	1690	O	PRO	106	-5.162	8.248	1.618	1.00	0.00
ATOM	1691	N	GLN	107	-5.486	7.819	-0.513	1.00	0.00
ATOM	1692	H	GLN	107	-5.149	7.437	-1.386	1.00	0.00
ATOM	1693	CA	GLN	107	-6.844	8.423	-0.485	1.00	0.00
ATOM	1694	HA	GLN	107	-6.944	9.085	0.375	1.00	0.00
ATOM	1695	CB	GLN	107	-7.070	9.239	-1.759	1.00	0.00
ATOM	1696	HB2	GLN	107	-6.287	9.991	-1.853	1.00	0.00
ATOM	1697	HB3	GLN	107	-7.042	8.577	-2.625	1.00	0.00
ATOM	1698	CG	GLN	107	-8.434	9.929	-1.688	1.00	0.00
ATOM	1699	HG2	GLN	107	-9.226	9.188	-1.578	1.00	0.00
ATOM	1700	HG3	GLN	107	-8.465	10.620	-0.846	1.00	0.00
ATOM	1701	CD	GLN	107	-8.676	10.716	-2.977	1.00	0.00
ATOM	1702	OE1	GLN	107	-7.789	10.848	-3.798	1.00	0.00
ATOM	1703	NE2	GLN	107	-9.847	11.250	-3.192	1.00	0.00

ATOM 1704 HE21 GLN 107 -10.013 11.770 -4.041 1.00 0.00
ATOM 1705 HE22 GLN 107 -10.582 11.140 -2.508 1.00 0.00
ATOM 1706 C GLN 107 -7.884 7.308 -0.399 1.00 0.00
ATOM 1707 O GLN 107 -8.787 7.347 0.412 1.00 0.00
ATOM 1708 N GLN 108 -7.766 6.313 -1.235 1.00 0.00
ATOM 1709 H GLN 108 -7.019 6.276 -1.914 1.00 0.00
ATOM 1710 CA GLN 108 -8.748 5.193 -1.204 1.00 0.00
ATOM 1711 HA GLN 108 -9.713 5.524 -1.587 1.00 0.00
ATOM 1712 CB GLN 108 -8.241 4.047 -2.082 1.00 0.00
ATOM 1713 HB2 GLN 108 -8.041 4.419 -3.087 1.00 0.00
ATOM 1714 HB3 GLN 108 -7.323 3.641 -1.656 1.00 0.00
ATOM 1715 CG GLN 108 -9.302 2.947 -2.148 1.00 0.00
ATOM 1716 HG2 GLN 108 -9.582 2.628 -1.144 1.00 0.00
ATOM 1717 HG3 GLN 108 -10.186 3.306 -2.675 1.00 0.00
ATOM 1718 CD GLN 108 -8.738 1.742 -2.904 1.00 0.00
ATOM 1719 OE1 GLN 108 -7.829 1.087 -2.436 1.00 0.00
ATOM 1720 NE2 GLN 108 -9.245 1.419 -4.063 1.00 0.00
ATOM 1721 HE21 GLN 108 -8.878 0.624 -4.568 1.00 0.00
ATOM 1722 HE22 GLN 108 -10.002 1.964 -4.449 1.00 0.00
ATOM 1723 C GLN 108 -8.915 4.699 0.233 1.00 0.00
ATOM 1724 O GLN 108 -9.959 4.208 0.615 1.00 0.00
ATOM 1725 N CYX 109 -7.894 4.825 1.035 1.00 0.00

```
ATOM   1726  H   CYX   109      -7.022   5.230   0.725  1.00  0.00
ATOM   1727  CA  CYX   109      -7.994   4.362   2.448  1.00  0.00
ATOM   1728  HA  CYX   109      -9.037   4.198   2.718  1.00  0.00
ATOM   1729  CB  CYX   109      -7.233   3.042   2.614  1.00  0.00
ATOM   1730  HB2 CYX   109      -6.785   3.002   3.607  1.00  0.00
ATOM   1731  HB3 CYX   109      -7.922   2.206   2.493  1.00  0.00
ATOM   1732  SG  CYX   109      -5.891   2.949   1.400  1.00  0.00
ATOM   1733  C   CYX   109      -7.389   5.421   3.374  1.00  0.00
ATOM   1734  O   CYX   109      -6.919   5.118   4.453  1.00  0.00
ATOM   1735  N   ASN   110      -7.401   6.658   2.961  1.00  0.00
ATOM   1736  H   ASN   110      -7.791   6.913   2.065  1.00  0.00
ATOM   1737  CA  ASN   110      -6.830   7.737   3.816  1.00  0.00
ATOM   1738  HA  ASN   110      -6.642   8.631   3.221  1.00  0.00
ATOM   1739  CB  ASN   110      -7.823   8.088   4.926  1.00  0.00
ATOM   1740  HB2 ASN   110      -7.919   7.255   5.623  1.00  0.00
ATOM   1741  HB3 ASN   110      -7.490   8.975   5.464  1.00  0.00
ATOM   1742  CG  ASN   110      -9.195   8.374   4.313  1.00  0.00
ATOM   1743  OD1 ASN   110      -9.301   9.099   3.344  1.00  0.00
ATOM   1744  ND2 ASN   110     -10.258   7.831   4.841  1.00  0.00
ATOM   1745 HD21 ASN   110     -11.167   8.015   4.441  1.00  0.00
ATOM   1746 HD22 ASN   110     -10.166   7.228   5.647  1.00  0.00
ATOM   1747  C   ASN   110      -5.519   7.257   4.440  1.00  0.00
```

ATOM	1748	O	ASN	110	-5.279	7.435	5.618	1.00	0.00
ATOM	1749	N	PHE	111	-4.666	6.648	3.660	1.00	0.00
ATOM	1750	H	PHE	111	-4.856	6.491	2.680	1.00	0.00
ATOM	1751	CA	PHE	111	-3.372	6.161	4.214	1.00	0.00
ATOM	1752	HA	PHE	111	-3.542	5.594	5.129	1.00	0.00
ATOM	1753	CB	PHE	111	-2.691	5.248	3.193	1.00	0.00
ATOM	1754	HB2	PHE	111	-3.402	4.811	2.492	1.00	0.00
ATOM	1755	HB3	PHE	111	-1.906	5.763	2.640	1.00	0.00
ATOM	1756	CG	PHE	111	-2.029	4.096	3.911	1.00	0.00
ATOM	1757	CD1	PHE	111	-2.784	2.976	4.281	1.00	0.00
ATOM	1758	HD1	PHE	111	-3.848	2.934	4.050	1.00	0.00
ATOM	1759	CE1	PHE	111	-2.170	1.909	4.948	1.00	0.00
ATOM	1760	HE1	PHE	111	-2.758	1.038	5.238	1.00	0.00
ATOM	1761	CZ	PHE	111	-0.802	1.960	5.243	1.00	0.00
ATOM	1762	HZ	PHE	111	-0.324	1.128	5.761	1.00	0.00
ATOM	1763	CE2	PHE	111	-0.048	3.080	4.872	1.00	0.00
ATOM	1764	HE2	PHE	111	1.017	3.121	5.102	1.00	0.00
ATOM	1765	CD2	PHE	111	-0.662	4.147	4.206	1.00	0.00
ATOM	1766	HD2	PHE	111	-0.075	5.018	3.917	1.00	0.00
ATOM	1767	C	PHE	111	-2.465	7.354	4.518	1.00	0.00
ATOM	1768	O	PHE	111	-1.379	7.203	5.040	1.00	0.00
ATOM	1769	N	ARG	112	-2.902	8.542	4.197	1.00	0.00

```
ATOM   1770  H   ARG   112      -3.804   8.677   3.764  1.00  0.00
ATOM   1771  CA  ARG   112      -2.062   9.742   4.471  1.00  0.00
ATOM   1772  HA  ARG   112      -1.211   9.771   3.790  1.00  0.00
ATOM   1773  CB  ARG   112      -2.897  11.008   4.265  1.00  0.00
ATOM   1774  HB2 ARG   112      -3.782  10.967   4.900  1.00  0.00
ATOM   1775  HB3 ARG   112      -2.302  11.882   4.528  1.00  0.00
ATOM   1776  CG  ARG   112      -3.325  11.104   2.798  1.00  0.00
ATOM   1777  HG2 ARG   112      -2.443  11.071   2.158  1.00  0.00
ATOM   1778  HG3 ARG   112      -3.981  10.268   2.554  1.00  0.00
ATOM   1779  CD  ARG   112      -4.071  12.420   2.571  1.00  0.00
ATOM   1780  HD2 ARG   112      -3.422  13.265   2.800  1.00  0.00
ATOM   1781  HD3 ARG   112      -4.406  12.490   1.536  1.00  0.00
ATOM   1782  NE  ARG   112      -5.262  12.476   3.464  1.00  0.00
ATOM   1783  HE  ARG   112      -5.960  11.745   3.459  1.00  0.00
ATOM   1784  CZ  ARG   112      -5.422  13.485   4.277  1.00  0.00
ATOM   1785  NH1 ARG   112      -4.973  13.413   5.500  1.00  0.00
ATOM   1786 HH11 ARG   112      -4.502  12.576   5.814  1.00  0.00
ATOM   1787 HH12 ARG   112      -5.098  14.194   6.127  1.00  0.00
ATOM   1788  NH2 ARG   112      -6.030  14.563   3.867  1.00  0.00
ATOM   1789 HH21 ARG   112      -6.376  14.614   2.920  1.00  0.00
ATOM   1790 HH22 ARG   112      -6.152  15.341   4.499  1.00  0.00
ATOM   1791  C   ARG   112      -1.563   9.691   5.916  1.00  0.00
```

ATOM	1792	O	ARG	112	-2.176	9.088	6.774	1.00	0.00
ATOM	1793	N	ALA	113	-0.453	10.321	6.194	1.00	0.00
ATOM	1794	H	ALA	113	0.064	10.825	5.488	1.00	0.00
ATOM	1795	CA	ALA	113	0.082	10.306	7.584	1.00	0.00
ATOM	1796	HA	ALA	113	-0.685	10.038	8.311	1.00	0.00
ATOM	1797	CB	ALA	113	1.222	9.291	7.681	1.00	0.00
ATOM	1798	HB1	ALA	113	2.002	9.550	6.965	1.00	0.00
ATOM	1799	HB2	ALA	113	1.636	9.303	8.689	1.00	0.00
ATOM	1800	HB3	ALA	113	0.842	8.294	7.457	1.00	0.00
ATOM	1801	C	ALA	113	0.606	11.697	7.945	1.00	0.00
ATOM	1802	O	ALA	113	0.826	12.529	7.087	1.00	0.00
ATOM	1803	N	PRO	114	0.809	11.948	9.247	1.00	0.00
ATOM	1804	CD	PRO	114	0.566	10.994	10.342	1.00	0.00
ATOM	1805	HD2	PRO	114	1.030	10.037	10.100	1.00	0.00
ATOM	1806	HD3	PRO	114	-0.507	10.854	10.470	1.00	0.00
ATOM	1807	CG	PRO	114	1.203	11.660	11.528	1.00	0.00
ATOM	1808	HG2	PRO	114	2.227	11.302	11.637	1.00	0.00
ATOM	1809	HG3	PRO	114	0.636	11.417	12.427	1.00	0.00
ATOM	1810	CB	PRO	114	1.165	13.126	11.253	1.00	0.00
ATOM	1811	HB2	PRO	114	2.023	13.587	11.742	1.00	0.00
ATOM	1812	HB3	PRO	114	0.252	13.624	11.577	1.00	0.00
ATOM	1813	CA	PRO	114	1.310	13.238	9.736	1.00	0.00

ATOM	1814	HA	PRO	114	0.706	14.030	9.295	1.00	0.00
ATOM	1815	C	PRO	114	2.780	13.452	9.366	1.00	0.00
ATOM	1816	O	PRO	114	3.310	14.538	9.489	1.00	0.00
ATOM	1817	N	GLN	115	3.442	12.423	8.911	1.00	0.00
ATOM	1818	H	GLN	115	3.007	11.517	8.806	1.00	0.00
ATOM	1819	CA	GLN	115	4.875	12.566	8.531	1.00	0.00
ATOM	1820	HA	GLN	115	5.290	13.480	8.955	1.00	0.00
ATOM	1821	CB	GLN	115	5.667	11.373	9.071	1.00	0.00
ATOM	1822	HB2	GLN	115	5.305	10.455	8.607	1.00	0.00
ATOM	1823	HB3	GLN	115	6.724	11.503	8.839	1.00	0.00
ATOM	1824	CG	GLN	115	5.485	11.283	10.588	1.00	0.00
ATOM	1825	HG2	GLN	115	4.437	11.111	10.833	1.00	0.00
ATOM	1826	HG3	GLN	115	6.090	10.473	10.996	1.00	0.00
ATOM	1827	CD	GLN	115	5.929	12.597	11.233	1.00	0.00
ATOM	1828	OE1	GLN	115	7.024	13.066	10.990	1.00	0.00
ATOM	1829	NE2	GLN	115	5.122	13.214	12.051	1.00	0.00
ATOM	1830	HE21	GLN	115	5.410	14.082	12.480	1.00	0.00
ATOM	1831	HE22	GLN	115	4.214	12.820	12.251	1.00	0.00
ATOM	1832	C	GLN	115	4.996	12.611	7.007	1.00	0.00
ATOM	1833	O	GLN	115	4.184	12.054	6.293	1.00	0.00
ATOM	1834	N	ARG	116	6.002	13.268	6.501	1.00	0.00
ATOM	1835	H	ARG	116	6.680	13.733	7.087	1.00	0.00

ATOM	1836	CA	ARG	116	6.173	13.347	5.023	1.00	0.00
ATOM	1837	HA	ARG	116	5.347	12.844	4.519	1.00	0.00
ATOM	1838	CB	ARG	116	6.191	14.814	4.589	1.00	0.00
ATOM	1839	HB2	ARG	116	6.999	15.335	5.102	1.00	0.00
ATOM	1840	HB3	ARG	116	6.348	14.873	3.512	1.00	0.00
ATOM	1841	CG	ARG	116	4.854	15.467	4.946	1.00	0.00
ATOM	1842	HG2	ARG	116	4.043	14.922	4.463	1.00	0.00
ATOM	1843	HG3	ARG	116	4.715	15.442	6.027	1.00	0.00
ATOM	1844	CD	ARG	116	4.851	16.919	4.464	1.00	0.00
ATOM	1845	HD2	ARG	116	5.765	17.423	4.778	1.00	0.00
ATOM	1846	HD3	ARG	116	4.772	16.956	3.377	1.00	0.00
ATOM	1847	NE	ARG	116	3.684	17.635	5.053	1.00	0.00
ATOM	1848	HE	ARG	116	3.778	18.205	5.881	1.00	0.00
ATOM	1849	CZ	ARG	116	2.509	17.535	4.496	1.00	0.00
ATOM	1850	NH1	ARG	116	2.292	16.623	3.588	1.00	0.00
ATOM	1851	HH11	ARG	116	3.037	15.995	3.319	1.00	0.00
ATOM	1852	HH12	ARG	116	1.381	16.548	3.159	1.00	0.00
ATOM	1853	NH2	ARG	116	1.548	18.345	4.849	1.00	0.00
ATOM	1854	HH21	ARG	116	1.718	19.048	5.554	1.00	0.00
ATOM	1855	HH22	ARG	116	0.640	18.264	4.416	1.00	0.00
ATOM	1856	C	ARG	116	7.491	12.681	4.624	1.00	0.00
ATOM	1857	O	ARG	116	8.436	12.649	5.386	1.00	0.00

ATOM	1858	N	CYX	117	7.561	12.146	3.436	1.00	0.00
ATOM	1859	H	CYX	117	6.779	12.168	2.797	1.00	0.00
ATOM	1860	CA	CYX	117	8.817	11.482	2.991	1.00	0.00
ATOM	1861	HA	CYX	117	9.548	11.474	3.800	1.00	0.00
ATOM	1862	CB	CYX	117	8.516	10.036	2.590	1.00	0.00
ATOM	1863	HB2	CYX	117	7.743	10.024	1.822	1.00	0.00
ATOM	1864	HB3	CYX	117	9.421	9.570	2.200	1.00	0.00
ATOM	1865	SG	CYX	117	7.886	9.131	4.025	1.00	0.00
ATOM	1866	C	CYX	117	9.393	12.233	1.789	1.00	0.00
ATOM	1867	O	CYX	117	8.696	12.951	1.101	1.00	0.00
ATOM	1868	N	ASP	118	10.663	12.072	1.530	1.00	0.00
ATOM	1869	H	ASP	118	11.250	11.477	2.097	1.00	0.00
ATOM	1870	CA	ASP	118	11.281	12.777	0.372	1.00	0.00
ATOM	1871	HA	ASP	118	10.988	13.826	0.368	1.00	0.00
ATOM	1872	CB	ASP	118	12.805	12.696	0.480	1.00	0.00
ATOM	1873	HB2	ASP	118	13.116	11.651	0.492	1.00	0.00
ATOM	1874	HB3	ASP	118	13.256	13.198	-0.376	1.00	0.00
ATOM	1875	CG	ASP	118	13.262	13.378	1.771	1.00	0.00
ATOM	1876	OD1	ASP	118	12.468	14.103	2.348	1.00	0.00
ATOM	1877	OD2	ASP	118	14.398	13.162	2.162	1.00	0.00
ATOM	1878	C	ASP	118	10.825	12.115	-0.930	1.00	0.00
ATOM	1879	O	ASP	118	10.612	10.921	-0.988	1.00	0.00

ATOM	1880	N	LEU	119	10.671	12.883	-1.974	1.00	0.00
ATOM	1881	H	LEU	119	10.846	13.876	-1.933	1.00	0.00
ATOM	1882	CA	LEU	119	10.226	12.297	-3.269	1.00	0.00
ATOM	1883	HA	LEU	119	9.414	11.589	-3.105	1.00	0.00
ATOM	1884	CB	LEU	119	9.724	13.414	-4.186	1.00	0.00
ATOM	1885	HB2	LEU	119	10.425	14.248	-4.159	1.00	0.00
ATOM	1886	HB3	LEU	119	9.643	13.040	-5.207	1.00	0.00
ATOM	1887	CG	LEU	119	8.350	13.887	-3.706	1.00	0.00
ATOM	1888	HG	LEU	119	7.743	13.023	-3.434	1.00	0.00
ATOM	1889	CD1	LEU	119	8.521	14.795	-2.487	1.00	0.00
ATOM	1890	HD11	LEU	119	9.128	15.658	-2.759	1.00	0.00
ATOM	1891	HD12	LEU	119	7.543	15.132	-2.145	1.00	0.00
ATOM	1892	HD13	LEU	119	9.015	14.242	-1.688	1.00	0.00
ATOM	1893	CD2	LEU	119	7.655	14.662	-4.827	1.00	0.00
ATOM	1894	HD21	LEU	119	7.533	14.014	-5.695	1.00	0.00
ATOM	1895	HD22	LEU	119	6.677	14.998	-4.484	1.00	0.00
ATOM	1896	HD23	LEU	119	8.260	15.526	-5.101	1.00	0.00
ATOM	1897	C	LEU	119	11.400	11.578	-3.937	1.00	0.00
ATOM	1898	O	LEU	119	11.219	10.732	-4.789	1.00	0.00
ATOM	1899	N	ASP	120	12.605	11.906	-3.555	1.00	0.00
ATOM	1900	H	ASP	120	12.766	12.609	-2.848	1.00	0.00
ATOM	1901	CA	ASP	120	13.787	11.238	-4.169	1.00	0.00

ATOM	1902	HA	ASP	120	13.731	11.295	-5.256	1.00	0.00
ATOM	1903	CB	ASP	120	15.067	11.938	-3.708	1.00	0.00
ATOM	1904	HB2	ASP	120	15.930	11.466	-4.178	1.00	0.00
ATOM	1905	HB3	ASP	120	15.029	12.989	-3.992	1.00	0.00
ATOM	1906	CG	ASP	120	15.189	11.825	-2.187	1.00	0.00
ATOM	1907	OD1	ASP	120	14.179	11.581	-1.547	1.00	0.00
ATOM	1908	OD2	ASP	120	16.291	11.984	-1.688	1.00	0.00
ATOM	1909	C	ASP	120	13.824	9.770	-3.740	1.00	0.00
ATOM	1910	O	ASP	120	14.283	8.912	-4.467	1.00	0.00
ATOM	1911	N	VAL	121	13.346	9.475	-2.562	1.00	0.00
ATOM	1912	H	VAL	121	12.964	10.182	-1.952	1.00	0.00
ATOM	1913	CA	VAL	121	13.355	8.062	-2.087	1.00	0.00
ATOM	1914	HA	VAL	121	14.380	7.709	-1.967	1.00	0.00
ATOM	1915	CB	VAL	121	12.647	7.976	-0.733	1.00	0.00
ATOM	1916	HB	VAL	121	11.686	8.486	-0.792	1.00	0.00
ATOM	1917	CG1	VAL	121	12.422	6.507	-0.367	1.00	0.00
ATOM	1918	HG11	VAL	121	13.383	5.995	-0.307	1.00	0.00
ATOM	1919	HG12	VAL	121	11.918	6.445	0.597	1.00	0.00
ATOM	1920	HG13	VAL	121	11.805	6.032	-1.130	1.00	0.00
ATOM	1921	CG2	VAL	121	13.514	8.641	0.338	1.00	0.00
ATOM	1922	HG21	VAL	121	13.675	9.687	0.078	1.00	0.00
ATOM	1923	HG22	VAL	121	13.011	8.580	1.303	1.00	0.00

ATOM 1924 HG23 VAL 121 14.475 8.129 0.397 1.00 0.00

ATOM 1925 C VAL 121 12.625 7.178 -3.101 1.00 0.00

ATOM 1926 O VAL 121 13.098 6.123 -3.473 1.00 0.00

ATOM 1927 N SER 122 11.477 7.601 -3.552 1.00 0.00

ATOM 1928 H SER 122 11.078 8.477 -3.247 1.00 0.00

ATOM 1929 CA SER 122 10.718 6.785 -4.542 1.00 0.00

ATOM 1930 HA SER 122 10.460 5.816 -4.114 1.00 0.00

ATOM 1931 CB SER 122 9.426 7.512 -4.921 1.00 0.00

ATOM 1932 HB2 SER 122 9.669 8.443 -5.433 1.00 0.00

ATOM 1933 HB3 SER 122 8.833 6.879 -5.581 1.00 0.00

ATOM 1934 OG SER 122 8.682 7.798 -3.746 1.00 0.00

ATOM 1935 HG SER 122 7.872 8.254 -3.984 1.00 0.00

ATOM 1936 C SER 122 11.572 6.577 -5.794 1.00 0.00

ATOM 1937 O SER 122 11.520 5.543 -6.429 1.00 0.00

ATOM 1938 N GLY 123 12.359 7.554 -6.156 1.00 0.00

ATOM 1939 H GLY 123 12.408 8.417 -5.634 1.00 0.00

ATOM 1940 CA GLY 123 13.215 7.411 -7.367 1.00 0.00

ATOM 1941 HA2 GLY 123 12.725 6.763 -8.094 1.00 0.00

ATOM 1942 HA3 GLY 123 13.393 8.389 -7.814 1.00 0.00

ATOM 1943 C GLY 123 14.557 6.792 -6.974 1.00 0.00

ATOM 1944 O GLY 123 15.502 6.795 -7.738 1.00 0.00

ATOM 1945 N GLY 124 14.649 6.257 -5.786 1.00 0.00

ATOM	1946	H	GLY	124	13.869	6.249	-5.145	1.00	0.00
ATOM	1947	CA	GLY	124	15.930	5.637	-5.346	1.00	0.00
ATOM	1948	HA2	GLY	124	16.738	6.366	-5.410	1.00	0.00
ATOM	1949	HA3	GLY	124	15.839	5.284	-4.318	1.00	0.00
ATOM	1950	C	GLY	124	16.260	4.449	-6.252	1.00	0.00
ATOM	1951	O	GLY	124	16.271	4.562	-7.461	1.00	0.00
ATOM	1952	N	ARG	125	16.529	3.308	-5.676	1.00	0.00
ATOM	1953	H	ARG	125	16.522	3.204	-4.671	1.00	0.00
ATOM	1954	CA	ARG	125	16.856	2.115	-6.506	1.00	0.00
ATOM	1955	HA	ARG	125	17.730	2.313	-7.127	1.00	0.00
ATOM	1956	CB	ARG	125	17.166	0.927	-5.591	1.00	0.00
ATOM	1957	HB2	ARG	125	16.341	0.778	-4.895	1.00	0.00
ATOM	1958	HB3	ARG	125	17.299	0.028	-6.193	1.00	0.00
ATOM	1959	CG	ARG	125	18.449	1.210	-4.808	1.00	0.00
ATOM	1960	HG2	ARG	125	19.248	1.473	-5.501	1.00	0.00
ATOM	1961	HG3	ARG	125	18.280	2.037	-4.119	1.00	0.00
ATOM	1962	CD	ARG	125	18.850	-0.038	-4.018	1.00	0.00
ATOM	1963	HD2	ARG	125	17.997	-0.423	-3.460	1.00	0.00
ATOM	1964	HD3	ARG	125	19.220	-0.810	-4.693	1.00	0.00
ATOM	1965	NE	ARG	125	19.932	0.312	-3.055	1.00	0.00
ATOM	1966	HE	ARG	125	19.866	1.124	-2.458	1.00	0.00
ATOM	1967	CZ	ARG	125	20.996	-0.438	-2.969	1.00	0.00

ATOM 1968 NH1 ARG 125 21.598 -0.844 -4.054 1.00 0.00
ATOM 1969 HH11 ARG 125 21.238 -0.576 -4.959 1.00 0.00
ATOM 1970 HH12 ARG 125 22.422 -1.425 -3.984 1.00 0.00
ATOM 1971 NH2 ARG 125 21.459 -0.783 -1.799 1.00 0.00
ATOM 1972 HH21 ARG 125 20.992 -0.468 -0.961 1.00 0.00
ATOM 1973 HH22 ARG 125 22.283 -1.364 -1.737 1.00 0.00
ATOM 1974 C ARG 125 15.661 1.773 -7.398 1.00 0.00
ATOM 1975 O ARG 125 15.817 1.286 -8.502 1.00 0.00
ATOM 1976 N CYX 126 14.470 2.024 -6.932 1.00 0.00
ATOM 1977 H CYX 126 14.334 2.428 -6.017 1.00 0.00
ATOM 1978 CA CYX 126 13.266 1.714 -7.754 1.00 0.00
ATOM 1979 HA CYX 126 13.243 0.654 -8.007 1.00 0.00
ATOM 1980 CB CYX 126 12.005 2.054 -6.958 1.00 0.00
ATOM 1981 HB2 CYX 126 12.070 3.079 -6.595 1.00 0.00
ATOM 1982 HB3 CYX 126 11.131 1.950 -7.600 1.00 0.00
ATOM 1983 SG CYX 126 11.889 0.965 -5.518 1.00 0.00
ATOM 1984 C CYX 126 13.300 2.546 -9.038 1.00 0.00
ATOM 1985 O CYX 126 12.694 2.196 -10.032 1.00 0.00
ATOM 1986 N SER 127 14.005 3.643 -9.026 1.00 0.00
ATOM 1987 H SER 127 14.513 3.937 -8.204 1.00 0.00
ATOM 1988 CA SER 127 14.079 4.496 -10.245 1.00 0.00
ATOM 1989 HA SER 127 14.752 5.337 -10.078 1.00 0.00

```
ATOM   1990  CB  SER   127      14.613   3.668 -11.416  1.00  0.00
ATOM   1991  HB2 SER   127      15.693   3.795 -11.489  1.00  0.00
ATOM   1992  HB3 SER   127      14.383   2.615 -11.252  1.00  0.00
ATOM   1993  OG  SER   127      14.003   4.103 -12.622  1.00  0.00
ATOM   1994  HG  SER   127      14.339   3.583 -13.356  1.00  0.00
ATOM   1995  C   SER   127      12.681   5.018 -10.588  1.00  0.00
ATOM   1996  O   SER   127      11.732   4.556  -9.977  1.00  0.00
ATOM   1997  OXT SER   127      12.585   5.873 -11.453  1.00  0.00
TER
ATOM   1998  S1  LIG   128     -25.710  13.916   1.899  1.00  0.00
ATOM   1999  S2  LIG   128     -18.259  14.566   3.990  1.00  0.00
ATOM   2000  O1  LIG   128     -21.981  16.533   7.094  1.00  0.00
ATOM   2001  H5  LIG   128     -21.131  16.671   7.564  1.00  0.00
ATOM   2002  O2  LIG   128     -26.488  15.167   1.261  1.00  0.00
ATOM   2003  H6  LIG   128     -26.963  15.572   2.048  1.00  0.00
ATOM   2004  O3  LIG   128     -17.683  13.629   5.162  1.00  0.00
ATOM   2005  H7  LIG   128     -17.742  14.202   5.980  1.00  0.00
ATOM   2006  O4  LIG   128     -24.895  13.348   0.845  1.00  0.00
ATOM   2007  O5  LIG   128     -26.636  13.089   2.659  1.00  0.00
ATOM   2008  O6  LIG   128     -18.307  13.756   2.790  1.00  0.00
ATOM   2009  O7  LIG   128     -17.575  15.850   4.006  1.00  0.00
ATOM   2010  O8  LIG   128     -32.758  16.967   6.620  1.00  0.00
```

ATOM	2011	O9	LIG	128	-33.288	17.432	4.566	1.00	0.00
ATOM	2012	N1	LIG	128	-24.503	16.597	6.270	1.00	0.00
ATOM	2013	H3	LIG	128	-23.865	16.851	7.008	1.00	0.00
ATOM	2014	H4	LIG	128	-25.493	16.774	6.347	1.00	0.00
ATOM	2015	N2	LIG	128	-26.330	15.835	4.581	1.00	0.00
ATOM	2016	N3	LIG	128	-19.517	15.966	6.803	1.00	0.00
ATOM	2017	N4	LIG	128	-27.256	15.846	3.719	1.00	0.00
ATOM	2018	N5	LIG	128	-18.349	15.516	6.957	1.00	0.00
ATOM	2019	N6	LIG	128	-32.496	17.073	5.427	1.00	0.00
ATOM	2020	C1	LIG	128	-22.689	15.543	5.033	1.00	0.00
ATOM	2021	C2	LIG	128	-22.301	14.831	3.863	1.00	0.00
ATOM	2022	C3	LIG	128	-24.084	15.873	5.218	1.00	0.00
ATOM	2023	C4	LIG	128	-24.599	14.716	3.100	1.00	0.00
ATOM	2024	C5	LIG	128	-23.277	14.421	2.921	1.00	0.00
ATOM	2025	H1	LIG	128	-22.973	13.845	2.057	1.00	0.00
ATOM	2026	C6	LIG	128	-21.666	15.881	5.956	1.00	0.00
ATOM	2027	C7	LIG	128	-19.965	14.864	4.536	1.00	0.00
ATOM	2028	C8	LIG	128	-20.937	14.522	3.635	1.00	0.00
ATOM	2029	H2	LIG	128	-20.655	14.015	2.721	1.00	0.00
ATOM	2030	C9	LIG	128	-25.049	15.461	4.239	1.00	0.00
ATOM	2031	C10	LIG	128	-20.309	15.539	5.746	1.00	0.00
ATOM	2032	C11	LIG	128	-28.543	16.145	4.207	1.00	0.00

```
ATOM   2033  C12 LIG   128     -17.598  16.049   8.022  1.00  0.00
ATOM   2034  C13 LIG   128     -28.904  16.031   5.561  1.00  0.00
ATOM   2035  H8  LIG   128     -28.176  15.680   6.279  1.00  0.00
ATOM   2036  C14 LIG   128     -29.497  16.533   3.257  1.00  0.00
ATOM   2037  H9  LIG   128     -29.217  16.594   2.213  1.00  0.00
ATOM   2038  C15 LIG   128     -30.195  16.336   5.959  1.00  0.00
ATOM   2039  H10 LIG   128     -30.503  16.249   6.992  1.00  0.00
ATOM   2040  C16 LIG   128     -30.790  16.846   3.651  1.00  0.00
ATOM   2041  H11 LIG   128     -31.539  17.158   2.937  1.00  0.00
ATOM   2042  C17 LIG   128     -31.119  16.748   5.000  1.00  0.00
ATOM   2043  C18 LIG   128     -17.930  17.232   8.704  1.00  0.00
ATOM   2044  H12 LIG   128     -18.795  17.804   8.396  1.00  0.00
ATOM   2045  C19 LIG   128     -16.440  15.339   8.372  1.00  0.00
ATOM   2046  H13 LIG   128     -16.180  14.443   7.822  1.00  0.00
ATOM   2047  C20 LIG   128     -17.123  17.672   9.741  1.00  0.00
ATOM   2048  H14 LIG   128     -17.370  18.590  10.263  1.00  0.00
ATOM   2049  C21 LIG   128     -15.643  15.784   9.420  1.00  0.00
ATOM   2050  H15 LIG   128     -14.755  15.228   9.694  1.00  0.00
ATOM   2051  C22 LIG   128     -15.983  16.948  10.108  1.00  0.00
ATOM   2052  H16 LIG   128     -15.356  17.302  10.918  1.00  0.00
TER
ATOM   2053  S1  LIG   129      19.780 -14.706  25.788  1.00  0.00
```

ATOM	2054	S2	LIG	129	19.368	-7.030	26.890	1.00	0.00
ATOM	2055	O1	LIG	129	23.476	-9.046	24.356	1.00	0.00
ATOM	2056	H5	LIG	129	23.594	-8.072	24.337	1.00	0.00
ATOM	2057	O2	LIG	129	20.729	-15.611	26.713	1.00	0.00
ATOM	2058	H6	LIG	129	21.569	-15.699	26.170	1.00	0.00
ATOM	2059	O3	LIG	129	18.991	-6.073	25.655	1.00	0.00
ATOM	2060	H7	LIG	129	19.877	-5.762	25.311	1.00	0.00
ATOM	2061	O4	LIG	129	18.587	-14.425	26.559	1.00	0.00
ATOM	2062	O5	LIG	129	19.692	-15.280	24.453	1.00	0.00
ATOM	2063	O6	LIG	129	18.129	-7.607	27.369	1.00	0.00
ATOM	2064	O7	LIG	129	20.273	-6.333	27.792	1.00	0.00
ATOM	2065	O8	LIG	129	26.441	-19.033	21.524	1.00	0.00
ATOM	2066	O9	LIG	129	25.989	-20.333	23.204	1.00	0.00
ATOM	2067	N1	LIG	129	23.798	-11.677	24.227	1.00	0.00
ATOM	2068	H3	LIG	129	24.195	-10.781	23.995	1.00	0.00
ATOM	2069	H4	LIG	129	24.242	-12.537	23.947	1.00	0.00
ATOM	2070	N2	LIG	129	22.832	-14.072	24.545	1.00	0.00
ATOM	2071	N3	LIG	129	22.216	-6.950	25.060	1.00	0.00
ATOM	2072	N4	LIG	129	22.673	-15.268	24.926	1.00	0.00
ATOM	2073	N5	LIG	129	21.605	-5.847	25.070	1.00	0.00
ATOM	2074	N6	LIG	129	25.891	-19.277	22.592	1.00	0.00
ATOM	2075	C1	LIG	129	21.848	-10.586	25.196	1.00	0.00

```
ATOM   2076  C2  LIG   129      20.590 -10.748  25.843  1.00  0.00
ATOM   2077  C3  LIG   129      22.579 -11.764  24.787  1.00  0.00
ATOM   2078  C4  LIG   129      20.733 -13.161  25.642  1.00  0.00
ATOM   2079  C5  LIG   129      20.053 -12.045  26.043  1.00  0.00
ATOM   2080  H1  LIG   129      19.077 -12.151  26.499  1.00  0.00
ATOM   2081  C6  LIG   129      22.303  -9.259  24.988  1.00  0.00
ATOM   2082  C7  LIG   129      20.326  -8.344  26.083  1.00  0.00
ATOM   2083  C8  LIG   129      19.865  -9.616  26.288  1.00  0.00
ATOM   2084  H2  LIG   129      18.929  -9.760  26.814  1.00  0.00
ATOM   2085  C9  LIG   129      22.019 -13.065  25.017  1.00  0.00
ATOM   2086  C10 LIG   129      21.560  -8.127  25.399  1.00  0.00
ATOM   2087  C11 LIG   129      23.494 -16.225  24.298  1.00  0.00
ATOM   2088  C12 LIG   129      22.363  -4.699  24.770  1.00  0.00
ATOM   2089  C13 LIG   129      24.139 -16.005  23.069  1.00  0.00
ATOM   2090  H8  LIG   129      23.996 -15.066  22.552  1.00  0.00
ATOM   2091  C14 LIG   129      23.616 -17.463  24.943  1.00  0.00
ATOM   2092  H9  LIG   129      23.095 -17.632  25.877  1.00  0.00
ATOM   2093  C15 LIG   129      24.923 -17.002  22.513  1.00  0.00
ATOM   2094  H10 LIG   129      25.424 -16.864  21.564  1.00  0.00
ATOM   2095  C16 LIG   129      24.406 -18.463  24.394  1.00  0.00
ATOM   2096  H11 LIG   129      24.524 -19.422  24.879  1.00  0.00
ATOM   2097  C17 LIG   129      25.053 -18.215  23.187  1.00  0.00
```

```
ATOM   2098  C18 LIG   129      23.768  -4.660  24.776  1.00  0.00
ATOM   2099  H12 LIG   129      24.328  -5.545  25.049  1.00  0.00
ATOM   2100  C19 LIG   129      21.631  -3.538  24.483  1.00  0.00
ATOM   2101  H13 LIG   129      20.549  -3.573  24.507  1.00  0.00
ATOM   2102  C20 LIG   129      24.421  -3.478  24.465  1.00  0.00
ATOM   2103  H14 LIG   129      25.504  -3.441  24.478  1.00  0.00
ATOM   2104  C21 LIG   129      22.295  -2.360  24.162  1.00  0.00
ATOM   2105  H15 LIG   129      21.727  -1.467  23.932  1.00  0.00
ATOM   2106  C22 LIG   129      23.689  -2.327  24.150  1.00  0.00
ATOM   2107  H16 LIG   129      24.208  -1.406  23.913  1.00  0.00
TER
ATOM   2108  S1  LIG   130     -29.853 -15.797  -5.583  1.00  0.00
ATOM   2109  S2  LIG   130     -22.573 -14.040  -3.527  1.00  0.00
ATOM   2110  O1  LIG   130     -23.925 -19.052  -4.166  1.00  0.00
ATOM   2111  H5  LIG   130     -22.965 -19.051  -3.965  1.00  0.00
ATOM   2112  O2  LIG   130     -30.902 -16.327  -4.490  1.00  0.00
ATOM   2113  H6  LIG   130     -30.856 -17.325  -4.588  1.00  0.00
ATOM   2114  O3  LIG   130     -21.397 -14.149  -4.617  1.00  0.00
ATOM   2115  H7  LIG   130     -20.999 -15.052  -4.453  1.00  0.00
ATOM   2116  O4  LIG   130     -29.766 -14.360  -5.418  1.00  0.00
ATOM   2117  O5  LIG   130     -30.148 -16.385  -6.881  1.00  0.00
ATOM   2118  O6  LIG   130     -23.268 -12.794  -3.782  1.00  0.00
```

ATOM	2119	O7	LIG	130	-22.051	-14.355	-2.207	1.00	0.00
ATOM	2120	O8	LIG	130	-33.050	-24.118	-6.930	1.00	0.00
ATOM	2121	O9	LIG	130	-34.674	-23.115	-5.896	1.00	0.00
ATOM	2122	N1	LIG	130	-26.466	-19.702	-4.570	1.00	0.00
ATOM	2123	H3	LIG	130	-25.532	-20.054	-4.438	1.00	0.00
ATOM	2124	H4	LIG	130	-27.240	-20.323	-4.752	1.00	0.00
ATOM	2125	N2	LIG	130	-28.901	-18.984	-5.143	1.00	0.00
ATOM	2126	N3	LIG	130	-22.050	-17.374	-3.786	1.00	0.00
ATOM	2127	N4	LIG	130	-30.153	-18.812	-5.086	1.00	0.00
ATOM	2128	N5	LIG	130	-20.988	-16.700	-3.876	1.00	0.00
ATOM	2129	N6	LIG	130	-33.520	-23.180	-6.297	1.00	0.00
ATOM	2130	C1	LIG	130	-25.646	-17.413	-4.448	1.00	0.00
ATOM	2131	C2	LIG	130	-25.969	-16.027	-4.495	1.00	0.00
ATOM	2132	C3	LIG	130	-26.697	-18.381	-4.663	1.00	0.00
ATOM	2133	C4	LIG	130	-28.286	-16.526	-5.009	1.00	0.00
ATOM	2134	C5	LIG	130	-27.293	-15.613	-4.788	1.00	0.00
ATOM	2135	H1	LIG	130	-27.516	-14.556	-4.861	1.00	0.00
ATOM	2136	C6	LIG	130	-24.293	-17.754	-4.195	1.00	0.00
ATOM	2137	C7	LIG	130	-23.671	-15.404	-4.006	1.00	0.00
ATOM	2138	C8	LIG	130	-24.971	-15.052	-4.254	1.00	0.00
ATOM	2139	H2	LIG	130	-25.242	-14.004	-4.255	1.00	0.00
ATOM	2140	C9	LIG	130	-28.032	-17.934	-4.941	1.00	0.00

ATOM 2141 C10 LIG 130 -23.288 -16.779 -3.993 1.00 0.00

ATOM 2142 C11 LIG 130 -30.940 -19.933 -5.412 1.00 0.00

ATOM 2143 C12 LIG 130 -19.783 -17.372 -3.593 1.00 0.00

ATOM 2144 C13 LIG 130 -30.460 -21.031 -6.145 1.00 0.00

ATOM 2145 H8 LIG 130 -29.439 -21.027 -6.504 1.00 0.00

ATOM 2146 C14 LIG 130 -32.281 -19.896 -5.001 1.00 0.00

ATOM 2147 H9 LIG 130 -32.649 -19.035 -4.459 1.00 0.00

ATOM 2148 C15 LIG 130 -31.302 -22.092 -6.433 1.00 0.00

ATOM 2149 H10 LIG 130 -30.962 -22.945 -7.003 1.00 0.00

ATOM 2150 C16 LIG 130 -33.127 -20.960 -5.281 1.00 0.00

ATOM 2151 H11 LIG 130 -34.160 -20.958 -4.965 1.00 0.00

ATOM 2152 C17 LIG 130 -32.623 -22.046 -5.989 1.00 0.00

ATOM 2153 C18 LIG 130 -19.710 -18.609 -2.931 1.00 0.00

ATOM 2154 H12 LIG 130 -20.616 -19.086 -2.581 1.00 0.00

ATOM 2155 C19 LIG 130 -18.608 -16.717 -3.987 1.00 0.00

ATOM 2156 H13 LIG 130 -18.675 -15.752 -4.474 1.00 0.00

ATOM 2157 C20 LIG 130 -18.472 -19.189 -2.700 1.00 0.00

ATOM 2158 H14 LIG 130 -18.411 -20.138 -2.180 1.00 0.00

ATOM 2159 C21 LIG 130 -17.372 -17.312 -3.761 1.00 0.00

ATOM 2160 H15 LIG 130 -16.467 -16.808 -4.075 1.00 0.00

ATOM 2161 C22 LIG 130 -17.301 -18.548 -3.120 1.00 0.00

ATOM 2162 H16 LIG 130 -16.337 -19.006 -2.931 1.00 0.00

```
TER
ATOM   2163  S1  LIG   131     -25.080  10.800 -25.570  1.00  0.00
ATOM   2164  S2  LIG   131     -20.512  16.725 -27.655  1.00  0.00
ATOM   2165  O1  LIG   131     -25.672  17.055 -28.446  1.00  0.00
ATOM   2166  H5  LIG   131     -25.297  17.917 -28.725  1.00  0.00
ATOM   2167  O2  LIG   131     -25.631   9.771 -26.672  1.00  0.00
ATOM   2168  H6  LIG   131     -26.546  10.126 -26.886  1.00  0.00
ATOM   2169  O3  LIG   131     -20.496  18.061 -26.763  1.00  0.00
ATOM   2170  H7  LIG   131     -21.138  18.671 -27.229  1.00  0.00
ATOM   2171  O4  LIG   131     -23.707  10.427 -25.298  1.00  0.00
ATOM   2172  O5  LIG   131     -26.046  10.938 -24.490  1.00  0.00
ATOM   2173  O6  LIG   131     -19.683  15.753 -26.971  1.00  0.00
ATOM   2174  O7  LIG   131     -20.282  17.063 -29.051  1.00  0.00
ATOM   2175  O8  LIG   131     -34.039  10.509 -26.525  1.00  0.00
ATOM   2176  O9  LIG   131     -33.410   8.499 -27.052  1.00  0.00
ATOM   2177  N1  LIG   131     -27.219  14.940 -28.028  1.00  0.00
ATOM   2178  H3  LIG   131     -27.190  15.895 -28.346  1.00  0.00
ATOM   2179  H4  LIG   131     -28.088  14.431 -27.972  1.00  0.00
ATOM   2180  N2  LIG   131     -27.534  12.552 -27.042  1.00  0.00
ATOM   2181  N3  LIG   131     -23.424  18.247 -28.470  1.00  0.00
ATOM   2182  N4  LIG   131     -27.784  11.317 -26.929  1.00  0.00
ATOM   2183  N5  LIG   131     -22.480  19.061 -28.278  1.00  0.00
```

```
ATOM   2184  N6  LIG   131     -33.184   9.674 -26.798  1.00  0.00
ATOM   2185  C1  LIG   131     -24.838  15.010 -27.523  1.00  0.00
ATOM   2186  C2  LIG   131     -23.703  14.307 -27.028  1.00  0.00
ATOM   2187  C3  LIG   131     -26.124  14.351 -27.517  1.00  0.00
ATOM   2188  C4  LIG   131     -25.066  12.378 -26.480  1.00  0.00
ATOM   2189  C5  LIG   131     -23.848  12.998 -26.503  1.00  0.00
ATOM   2190  H1  LIG   131     -22.984  12.491 -26.093  1.00  0.00
ATOM   2191  C6  LIG   131     -24.627  16.333 -27.991  1.00  0.00
ATOM   2192  C7  LIG   131     -22.240  16.187 -27.514  1.00  0.00
ATOM   2193  C8  LIG   131     -22.422  14.911 -27.052  1.00  0.00
ATOM   2194  H2  LIG   131     -21.564  14.347 -26.709  1.00  0.00
ATOM   2195  C9  LIG   131     -26.239  13.019 -26.995  1.00  0.00
ATOM   2196  C10 LIG   131     -23.354  16.949 -27.979  1.00  0.00
ATOM   2197  C11 LIG   131     -29.149  10.970 -26.888  1.00  0.00
ATOM   2198  C12 LIG   131     -22.611  20.335 -28.861  1.00  0.00
ATOM   2199  C13 LIG   131     -30.171  11.880 -26.570  1.00  0.00
ATOM   2200  H8  LIG   131     -29.916  12.902 -26.323  1.00  0.00
ATOM   2201  C14 LIG   131     -29.460   9.627 -27.143  1.00  0.00
ATOM   2202  H9  LIG   131     -28.663   8.927 -27.362  1.00  0.00
ATOM   2203  C15 LIG   131     -31.489  11.457 -26.542  1.00  0.00
ATOM   2204  H10 LIG   131     -32.294  12.133 -26.292  1.00  0.00
ATOM   2205  C16 LIG   131     -30.779   9.199 -27.123  1.00  0.00
```

```
ATOM   2206  H11 LIG   131     -31.044   8.171 -27.327  1.00  0.00
ATOM   2207  C17 LIG   131     -31.777  10.123 -26.827  1.00  0.00
ATOM   2208  C18 LIG   131     -23.536  20.646 -29.871  1.00  0.00
ATOM   2209  H12 LIG   131     -24.183  19.873 -30.264  1.00  0.00
ATOM   2210  C19 LIG   131     -21.724  21.317 -28.396  1.00  0.00
ATOM   2211  H13 LIG   131     -20.997  21.059 -27.635  1.00  0.00
ATOM   2212  C20 LIG   131     -23.586  21.935 -30.380  1.00  0.00
ATOM   2213  H14 LIG   131     -24.291  22.176 -31.166  1.00  0.00
ATOM   2214  C21 LIG   131     -21.789  22.609 -28.905  1.00  0.00
ATOM   2215  H15 LIG   131     -21.109  23.367 -28.537  1.00  0.00
ATOM   2216  C22 LIG   131     -22.720  22.921 -29.895  1.00  0.00
ATOM   2217  H16 LIG   131     -22.763  23.925 -30.301  1.00  0.00
TER
ATOM   2218  S1  LIG   132      12.811  -2.570 -24.256  1.00  0.00
ATOM   2219  S2  LIG   132      19.012   0.564 -27.724  1.00  0.00
ATOM   2220  O1  LIG   132      14.301   1.061 -29.942  1.00  0.00
ATOM   2221  H5  LIG   132      14.923   1.568 -30.506  1.00  0.00
ATOM   2222  O2  LIG   132      12.197  -3.955 -24.784  1.00  0.00
ATOM   2223  H6  LIG   132      11.449  -3.664 -25.388  1.00  0.00
ATOM   2224  O3  LIG   132      19.227   2.156 -27.669  1.00  0.00
ATOM   2225  H7  LIG   132      18.845   2.482 -28.534  1.00  0.00
ATOM   2226  O4  LIG   132      13.982  -2.903 -23.471  1.00  0.00
```

ATOM	2227	O5	LIG	132	11.753	-1.734	-23.706	1.00	0.00
ATOM	2228	O6	LIG	132	19.446	0.036	-26.447	1.00	0.00
ATOM	2229	O7	LIG	132	19.539	0.040	-28.975	1.00	0.00
ATOM	2230	O8	LIG	132	4.343	-2.186	-27.325	1.00	0.00
ATOM	2231	O9	LIG	132	4.512	-4.213	-26.565	1.00	0.00
ATOM	2232	N1	LIG	132	12.219	-0.249	-28.947	1.00	0.00
ATOM	2233	H3	LIG	132	12.541	0.357	-29.684	1.00	0.00
ATOM	2234	H4	LIG	132	11.251	-0.525	-28.880	1.00	0.00
ATOM	2235	N2	LIG	132	11.151	-1.628	-27.016	1.00	0.00
ATOM	2236	N3	LIG	132	16.753	1.740	-29.955	1.00	0.00
ATOM	2237	N4	LIG	132	10.579	-2.549	-26.364	1.00	0.00
ATOM	2238	N5	LIG	132	17.831	2.394	-29.954	1.00	0.00
ATOM	2239	N6	LIG	132	4.988	-3.134	-26.892	1.00	0.00
ATOM	2240	C1	LIG	132	14.427	-0.218	-27.923	1.00	0.00
ATOM	2241	C2	LIG	132	15.252	-0.668	-26.853	1.00	0.00
ATOM	2242	C3	LIG	132	13.031	-0.592	-27.934	1.00	0.00
ATOM	2243	C4	LIG	132	13.372	-1.777	-25.796	1.00	0.00
ATOM	2244	C5	LIG	132	14.695	-1.436	-25.799	1.00	0.00
ATOM	2245	H1	LIG	132	15.324	-1.737	-24.971	1.00	0.00
ATOM	2246	C6	LIG	132	15.040	0.584	-28.920	1.00	0.00
ATOM	2247	C7	LIG	132	17.204	0.421	-27.807	1.00	0.00
ATOM	2248	C8	LIG	132	16.631	-0.350	-26.831	1.00	0.00

ATOM	2249	H2	LIG	132	17.250	-0.733	-26.030	1.00	0.00
ATOM	2250	C9	LIG	132	12.499	-1.384	-26.862	1.00	0.00
ATOM	2251	C10	LIG	132	16.411	0.935	-28.877	1.00	0.00
ATOM	2252	C11	LIG	132	9.183	-2.637	-26.526	1.00	0.00
ATOM	2253	C12	LIG	132	18.125	3.137	-31.114	1.00	0.00
ATOM	2254	C13	LIG	132	8.390	-1.580	-27.006	1.00	0.00
ATOM	2255	H8	LIG	132	8.855	-0.635	-27.253	1.00	0.00
ATOM	2256	C14	LIG	132	8.583	-3.843	-26.138	1.00	0.00
ATOM	2257	H9	LIG	132	9.198	-4.643	-25.745	1.00	0.00
ATOM	2258	C15	LIG	132	7.020	-1.743	-27.127	1.00	0.00
ATOM	2259	H10	LIG	132	6.386	-0.945	-27.485	1.00	0.00
ATOM	2260	C16	LIG	132	7.212	-4.014	-26.263	1.00	0.00
ATOM	2261	H11	LIG	132	6.729	-4.939	-25.980	1.00	0.00
ATOM	2262	C17	LIG	132	6.450	-2.962	-26.762	1.00	0.00
ATOM	2263	C18	LIG	132	17.482	2.953	-32.349	1.00	0.00
ATOM	2264	H12	LIG	132	16.730	2.182	-32.454	1.00	0.00
ATOM	2265	C19	LIG	132	19.148	4.089	-30.990	1.00	0.00
ATOM	2266	H13	LIG	132	19.655	4.204	-30.040	1.00	0.00
ATOM	2267	C20	LIG	132	17.845	3.738	-33.432	1.00	0.00
ATOM	2268	H14	LIG	132	17.358	3.592	-34.390	1.00	0.00
ATOM	2269	C21	LIG	132	19.497	4.878	-32.079	1.00	0.00
ATOM	2270	H15	LIG	132	20.281	5.618	-31.978	1.00	0.00

```
ATOM   2271  C22 LIG   132      18.846   4.707 -33.301  1.00  0.00
ATOM   2272  H16 LIG   132      19.126   5.313 -34.154  1.00  0.00
TER
ATOM   2273  S1  LIG   133      -6.222  14.273  18.626  1.00  0.00
ATOM   2274  S2  LIG   133      -2.708  20.304  22.030  1.00  0.00
ATOM   2275  O1  LIG   133      -3.082  15.763  24.598  1.00  0.00
ATOM   2276  H5  LIG   133      -2.564  16.362  25.176  1.00  0.00
ATOM   2277  O2  LIG   133      -7.726  13.878  19.023  1.00  0.00
ATOM   2278  H6  LIG   133      -7.604  13.151  19.706  1.00  0.00
ATOM   2279  O3  LIG   133      -1.106  20.318  22.153  1.00  0.00
ATOM   2280  H7  LIG   133      -0.931  19.970  23.074  1.00  0.00
ATOM   2281  O4  LIG   133      -6.309  15.408  17.731  1.00  0.00
ATOM   2282  O5  LIG   133      -5.476  13.080  18.252  1.00  0.00
ATOM   2283  O6  LIG   133      -3.029  20.694  20.672  1.00  0.00
ATOM   2284  O7  LIG   133      -3.295  20.991  23.170  1.00  0.00
ATOM   2285  O8  LIG   133      -7.294   6.098  22.273  1.00  0.00
ATOM   2286  O9  LIG   133      -9.183   6.452  21.265  1.00  0.00
ATOM   2287  N1  LIG   133      -4.534  13.783  23.592  1.00  0.00
ATOM   2288  H3  LIG   133      -3.977  14.087  24.374  1.00  0.00
ATOM   2289  H4  LIG   133      -4.926  12.854  23.557  1.00  0.00
ATOM   2290  N2  LIG   133      -5.818  12.739  21.584  1.00  0.00
ATOM   2291  N3  LIG   133      -2.093  18.107  24.531  1.00  0.00
```

```
ATOM   2292  N4  LIG   133      -6.728  12.233  20.865  1.00  0.00
ATOM   2293  N5  LIG   133      -1.307  19.093  24.537  1.00  0.00
ATOM   2294  N6  LIG   133      -8.095   6.817  21.687  1.00  0.00
ATOM   2295  C1  LIG   133      -4.101  15.881  22.436  1.00  0.00
ATOM   2296  C2  LIG   133      -4.317  16.665  21.267  1.00  0.00
ATOM   2297  C3  LIG   133      -4.653  14.547  22.493  1.00  0.00
ATOM   2298  C4  LIG   133      -5.538  14.855  20.210  1.00  0.00
ATOM   2299  C5  LIG   133      -5.029  16.123  20.167  1.00  0.00
ATOM   2300  H1  LIG   133      -5.152  16.715  19.269  1.00  0.00
ATOM   2301  C6  LIG   133      -3.341  16.470  23.479  1.00  0.00
ATOM   2302  C7  LIG   133      -3.094  18.540  22.214  1.00  0.00
ATOM   2303  C8  LIG   133      -3.820  17.989  21.192  1.00  0.00
ATOM   2304  H2  LIG   133      -4.028  18.583  20.312  1.00  0.00
ATOM   2305  C9  LIG   133      -5.384  14.030  21.372  1.00  0.00
ATOM   2306  C10 LIG   133      -2.812  17.779  23.388  1.00  0.00
ATOM   2307  C11 LIG   133      -7.015  10.876  21.107  1.00  0.00
ATOM   2308  C12 LIG   133      -0.666  19.387  25.756  1.00  0.00
ATOM   2309  C13 LIG   133      -6.131  10.002  21.762  1.00  0.00
ATOM   2310  H8  LIG   133      -5.167  10.365  22.090  1.00  0.00
ATOM   2311  C14 LIG   133      -8.239  10.399  20.616  1.00  0.00
ATOM   2312  H9  LIG   133      -8.902  11.073  20.091  1.00  0.00
ATOM   2313  C15 LIG   133      -6.484   8.676  21.953  1.00  0.00
```

```
ATOM   2314  H10 LIG   133      -5.821   7.980  22.445  1.00  0.00
ATOM   2315  C16 LIG   133      -8.601   9.073  20.811  1.00  0.00
ATOM   2316  H11 LIG   133      -9.543   8.686  20.451  1.00  0.00
ATOM   2317  C17 LIG   133      -7.719   8.232  21.482  1.00  0.00
ATOM   2318  C18 LIG   133      -1.069  18.873  27.000  1.00  0.00
ATOM   2319  H12 LIG   133      -1.938  18.232  27.060  1.00  0.00
ATOM   2320  C19 LIG   133       0.419  20.272  25.681  1.00  0.00
ATOM   2321  H13 LIG   133       0.704  20.682  24.719  1.00  0.00
ATOM   2322  C20 LIG   133      -0.369  19.224  28.143  1.00  0.00
ATOM   2323  H14 LIG   133      -0.683  18.838  29.106  1.00  0.00
ATOM   2324  C21 LIG   133       1.121  20.609  26.832  1.00  0.00
ATOM   2325  H15 LIG   133       1.964  21.285  26.769  1.00  0.00
ATOM   2326  C22 LIG   133       0.731  20.085  28.064  1.00  0.00
ATOM   2327  H16 LIG   133       1.270  20.357  28.964  1.00  0.00
TER
ATOM   2328  S1  LIG   134     -25.482  -4.867  14.844  1.00  0.00
ATOM   2329  S2  LIG   134     -24.587  -9.072  21.311  1.00  0.00
ATOM   2330  O1  LIG   134     -26.301  -4.143  21.666  1.00  0.00
ATOM   2331  H5  LIG   134     -26.314  -4.503  22.579  1.00  0.00
ATOM   2332  O2  LIG   134     -24.386  -3.771  14.428  1.00  0.00
ATOM   2333  H6  LIG   134     -24.766  -2.913  14.787  1.00  0.00
ATOM   2334  O3  LIG   134     -25.885  -9.648  22.065  1.00  0.00
```

ATOM	2335	H7	LIG	134	-26.066	-8.975	22.782	1.00	0.00
ATOM	2336	O4	LIG	134	-24.945	-6.159	14.470	1.00	0.00
ATOM	2337	O5	LIG	134	-26.802	-4.446	14.396	1.00	0.00
ATOM	2338	O6	LIG	134	-24.334	-9.945	20.184	1.00	0.00
ATOM	2339	O7	LIG	134	-23.547	-8.774	22.283	1.00	0.00
ATOM	2340	O8	LIG	134	-28.397	3.657	14.502	1.00	0.00
ATOM	2341	O9	LIG	134	-26.809	3.608	13.024	1.00	0.00
ATOM	2342	N1	LIG	134	-26.330	-2.588	19.517	1.00	0.00
ATOM	2343	H3	LIG	134	-26.500	-2.634	20.509	1.00	0.00
ATOM	2344	H4	LIG	134	-26.512	-1.746	18.991	1.00	0.00
ATOM	2345	N2	LIG	134	-26.158	-2.330	16.933	1.00	0.00
ATOM	2346	N3	LIG	134	-25.869	-6.349	22.860	1.00	0.00
ATOM	2347	N4	LIG	134	-25.795	-1.959	15.779	1.00	0.00
ATOM	2348	N5	LIG	134	-25.988	-7.404	23.541	1.00	0.00
ATOM	2349	N6	LIG	134	-27.444	3.121	13.949	1.00	0.00
ATOM	2350	C1	LIG	134	-25.749	-4.952	19.483	1.00	0.00
ATOM	2351	C2	LIG	134	-25.339	-6.068	18.699	1.00	0.00
ATOM	2352	C3	LIG	134	-26.000	-3.691	18.823	1.00	0.00
ATOM	2353	C4	LIG	134	-25.475	-4.759	16.662	1.00	0.00
ATOM	2354	C5	LIG	134	-25.221	-5.945	17.292	1.00	0.00
ATOM	2355	H1	LIG	134	-24.948	-6.812	16.703	1.00	0.00
ATOM	2356	C6	LIG	134	-25.890	-5.158	20.879	1.00	0.00

ATOM	2357	C7	LIG	134	-25.187	-7.484	20.669	1.00	0.00
ATOM	2358	C8	LIG	134	-25.050	-7.308	19.318	1.00	0.00
ATOM	2359	H2	LIG	134	-24.702	-8.134	18.711	1.00	0.00
ATOM	2360	C9	LIG	134	-25.857	-3.591	17.399	1.00	0.00
ATOM	2361	C10	LIG	134	-25.638	-6.408	21.492	1.00	0.00
ATOM	2362	C11	LIG	134	-26.248	-0.689	15.373	1.00	0.00
ATOM	2363	C12	LIG	134	-26.161	-7.248	24.929	1.00	0.00
ATOM	2364	C13	LIG	134	-27.342	-0.032	15.961	1.00	0.00
ATOM	2365	H8	LIG	134	-27.891	-0.518	16.757	1.00	0.00
ATOM	2366	C14	LIG	134	-25.573	-0.100	14.295	1.00	0.00
ATOM	2367	H9	LIG	134	-24.747	-0.625	13.832	1.00	0.00
ATOM	2368	C15	LIG	134	-27.732	1.213	15.497	1.00	0.00
ATOM	2369	H10	LIG	134	-28.575	1.737	15.925	1.00	0.00
ATOM	2370	C16	LIG	134	-25.955	1.151	13.830	1.00	0.00
ATOM	2371	H11	LIG	134	-25.442	1.629	13.008	1.00	0.00
ATOM	2372	C17	LIG	134	-27.027	1.792	14.443	1.00	0.00
ATOM	2373	C18	LIG	134	-25.901	-6.056	25.626	1.00	0.00
ATOM	2374	H12	LIG	134	-25.517	-5.196	25.093	1.00	0.00
ATOM	2375	C19	LIG	134	-26.595	-8.386	25.625	1.00	0.00
ATOM	2376	H13	LIG	134	-26.767	-9.307	25.082	1.00	0.00
ATOM	2377	C20	LIG	134	-26.105	-6.006	26.996	1.00	0.00
ATOM	2378	H14	LIG	134	-25.896	-5.091	27.538	1.00	0.00

ATOM 2379 C21 LIG 134 -26.806 -8.322 26.997 1.00 0.00
ATOM 2380 H15 LIG 134 -27.150 -9.200 27.530 1.00 0.00
ATOM 2381 C22 LIG 134 -26.565 -7.133 27.685 1.00 0.00
ATOM 2382 H16 LIG 134 -26.717 -7.086 28.757 1.00 0.00
TER
ATOM 2383 S1 LIG 135 12.440 23.355 -0.749 1.00 0.00
ATOM 2384 S2 LIG 135 11.549 22.675 -8.434 1.00 0.00
ATOM 2385 O1 LIG 135 15.472 20.151 -6.067 1.00 0.00
ATOM 2386 H5 LIG 135 15.508 19.888 -7.012 1.00 0.00
ATOM 2387 O2 LIG 135 13.562 24.329 -0.144 1.00 0.00
ATOM 2388 H6 LIG 135 14.336 23.713 0.033 1.00 0.00
ATOM 2389 O3 LIG 135 10.951 21.313 -9.042 1.00 0.00
ATOM 2390 H7 LIG 135 11.762 20.789 -9.304 1.00 0.00
ATOM 2391 O4 LIG 135 11.329 24.194 -1.150 1.00 0.00
ATOM 2392 O5 LIG 135 12.239 22.217 0.136 1.00 0.00
ATOM 2393 O6 LIG 135 10.427 23.439 -7.930 1.00 0.00
ATOM 2394 O7 LIG 135 12.497 23.257 -9.371 1.00 0.00
ATOM 2395 O8 LIG 135 18.867 19.361 4.151 1.00 0.00
ATOM 2396 O9 LIG 135 18.726 21.343 5.024 1.00 0.00
ATOM 2397 N1 LIG 135 15.986 20.599 -3.503 1.00 0.00
ATOM 2398 H3 LIG 135 16.280 20.112 -4.334 1.00 0.00
ATOM 2399 H4 LIG 135 16.461 20.471 -2.623 1.00 0.00

ATOM	2400	N2	LIG	135	15.260	21.597	-1.212	1.00	0.00
ATOM	2401	N3	LIG	135	14.141	20.509	-8.207	1.00	0.00
ATOM	2402	N4	LIG	135	15.244	22.265	-0.137	1.00	0.00
ATOM	2403	N5	LIG	135	13.450	20.342	-9.248	1.00	0.00
ATOM	2404	N6	LIG	135	18.471	20.521	4.155	1.00	0.00
ATOM	2405	C1	LIG	135	14.085	21.541	-4.699	1.00	0.00
ATOM	2406	C2	LIG	135	12.931	22.372	-4.637	1.00	0.00
ATOM	2407	C3	LIG	135	14.854	21.324	-3.494	1.00	0.00
ATOM	2408	C4	LIG	135	13.241	22.724	-2.258	1.00	0.00
ATOM	2409	C5	LIG	135	12.527	22.941	-3.403	1.00	0.00
ATOM	2410	H1	LIG	135	11.625	23.537	-3.362	1.00	0.00
ATOM	2411	C6	LIG	135	14.404	20.968	-5.957	1.00	0.00
ATOM	2412	C7	LIG	135	12.505	22.076	-7.012	1.00	0.00
ATOM	2413	C8	LIG	135	12.176	22.634	-5.806	1.00	0.00
ATOM	2414	H2	LIG	135	11.325	23.300	-5.748	1.00	0.00
ATOM	2415	C9	LIG	135	14.431	21.925	-2.262	1.00	0.00
ATOM	2416	C10	LIG	135	13.627	21.199	-7.116	1.00	0.00
ATOM	2417	C11	LIG	135	16.059	21.773	0.901	1.00	0.00
ATOM	2418	C12	LIG	135	14.072	19.683	-10.325	1.00	0.00
ATOM	2419	C13	LIG	135	16.532	20.451	0.955	1.00	0.00
ATOM	2420	H8	LIG	135	16.254	19.753	0.178	1.00	0.00
ATOM	2421	C14	LIG	135	16.356	22.668	1.939	1.00	0.00

ATOM 2422 H9 LIG 135 15.966 23.677 1.902 1.00 0.00
ATOM 2423 C15 LIG 135 17.321 20.043 2.017 1.00 0.00
ATOM 2424 H10 LIG 135 17.692 19.030 2.089 1.00 0.00
ATOM 2425 C16 LIG 135 17.152 22.266 3.002 1.00 0.00
ATOM 2426 H11 LIG 135 17.403 22.942 3.807 1.00 0.00
ATOM 2427 C17 LIG 135 17.628 20.958 3.023 1.00 0.00
ATOM 2428 C18 LIG 135 15.459 19.488 -10.432 1.00 0.00
ATOM 2429 H12 LIG 135 16.117 19.883 -9.670 1.00 0.00
ATOM 2430 C19 LIG 135 13.220 19.233 -11.344 1.00 0.00
ATOM 2431 H13 LIG 135 12.154 19.412 -11.263 1.00 0.00
ATOM 2432 C20 LIG 135 15.973 18.824 -11.535 1.00 0.00
ATOM 2433 H14 LIG 135 17.044 18.681 -11.627 1.00 0.00
ATOM 2434 C21 LIG 135 13.744 18.559 -12.441 1.00 0.00
ATOM 2435 H15 LIG 135 13.083 18.205 -13.221 1.00 0.00
ATOM 2436 C22 LIG 135 15.120 18.351 -12.538 1.00 0.00
ATOM 2437 H16 LIG 135 15.531 17.836 -13.398 1.00 0.00
TER
ATOM 2438 S1 LIG 136 -4.733 -16.007 22.630 1.00 0.00
ATOM 2439 S2 LIG 136 -7.005 -15.755 30.051 1.00 0.00
ATOM 2440 O1 LIG 136 -4.961 -11.477 27.843 1.00 0.00
ATOM 2441 H5 LIG 136 -5.226 -11.216 28.750 1.00 0.00
ATOM 2442 O2 LIG 136 -3.178 -16.152 22.259 1.00 0.00

ATOM	2443	H6	LIG	136	-2.887	-15.204	22.097	1.00	0.00
ATOM	2444	O3	LIG	136	-8.364	-14.989	30.436	1.00	0.00
ATOM	2445	H7	LIG	136	-8.056	-14.080	30.721	1.00	0.00
ATOM	2446	O4	LIG	136	-5.199	-17.326	23.003	1.00	0.00
ATOM	2447	O5	LIG	136	-5.418	-15.231	21.606	1.00	0.00
ATOM	2448	O6	LIG	136	-7.387	-17.048	29.521	1.00	0.00
ATOM	2449	O7	LIG	136	-6.050	-15.641	31.143	1.00	0.00
ATOM	2450	O8	LIG	136	-1.147	-9.142	18.016	1.00	0.00
ATOM	2451	O9	LIG	136	0.019	-10.852	17.362	1.00	0.00
ATOM	2452	N1	LIG	136	-3.900	-11.613	25.414	1.00	0.00
ATOM	2453	H3	LIG	136	-4.073	-11.021	26.211	1.00	0.00
ATOM	2454	H4	LIG	136	-3.460	-11.255	24.580	1.00	0.00
ATOM	2455	N2	LIG	136	-3.558	-12.913	23.186	1.00	0.00
ATOM	2456	N3	LIG	136	-6.144	-12.487	29.856	1.00	0.00
ATOM	2457	N4	LIG	136	-3.022	-13.494	22.198	1.00	0.00
ATOM	2458	N5	LIG	136	-6.953	-12.728	30.794	1.00	0.00
ATOM	2459	N6	LIG	136	-0.793	-10.312	18.101	1.00	0.00
ATOM	2460	C1	LIG	136	-5.061	-13.457	26.501	1.00	0.00
ATOM	2461	C2	LIG	136	-5.497	-14.809	26.412	1.00	0.00
ATOM	2462	C3	LIG	136	-4.388	-12.864	25.367	1.00	0.00
ATOM	2463	C4	LIG	136	-4.684	-14.981	24.133	1.00	0.00
ATOM	2464	C5	LIG	136	-5.304	-15.542	25.214	1.00	0.00

ATOM	2465	H1	LIG	136	-5.677	-16.557	25.146	1.00	0.00
ATOM	2466	C6	LIG	136	-5.327	-12.769	27.713	1.00	0.00
ATOM	2467	C7	LIG	136	-6.369	-14.751	28.680	1.00	0.00
ATOM	2468	C8	LIG	136	-6.128	-15.431	27.517	1.00	0.00
ATOM	2469	H2	LIG	136	-6.416	-16.472	27.445	1.00	0.00
ATOM	2470	C9	LIG	136	-4.192	-13.635	24.173	1.00	0.00
ATOM	2471	C10	LIG	136	-5.992	-13.379	28.803	1.00	0.00
ATOM	2472	C11	LIG	136	-2.496	-12.648	21.202	1.00	0.00
ATOM	2473	C12	LIG	136	-6.999	-11.798	31.849	1.00	0.00
ATOM	2474	C13	LIG	136	-2.872	-11.302	21.050	1.00	0.00
ATOM	2475	H8	LIG	136	-3.617	-10.879	21.710	1.00	0.00
ATOM	2476	C14	LIG	136	-1.583	-13.226	20.309	1.00	0.00
ATOM	2477	H9	LIG	136	-1.318	-14.270	20.419	1.00	0.00
ATOM	2478	C15	LIG	136	-2.315	-10.539	20.038	1.00	0.00
ATOM	2479	H10	LIG	136	-2.593	-9.505	19.892	1.00	0.00
ATOM	2480	C16	LIG	136	-1.016	-12.464	19.298	1.00	0.00
ATOM	2481	H11	LIG	136	-0.302	-12.886	18.605	1.00	0.00
ATOM	2482	C17	LIG	136	-1.387	-11.128	19.180	1.00	0.00
ATOM	2483	C18	LIG	136	-6.015	-10.821	32.080	1.00	0.00
ATOM	2484	H12	LIG	136	-5.144	-10.775	31.440	1.00	0.00
ATOM	2485	C19	LIG	136	-8.096	-11.906	32.715	1.00	0.00
ATOM	2486	H13	LIG	136	-8.835	-12.679	32.543	1.00	0.00

```
ATOM   2487  C20 LIG   136      -6.154  -9.950  33.149  1.00  0.00
ATOM   2488  H14 LIG   136      -5.392  -9.202  33.336  1.00  0.00
ATOM   2489  C21 LIG   136      -8.232 -11.020  33.778  1.00  0.00
ATOM   2490  H15 LIG   136      -9.085 -11.100  34.440  1.00  0.00
ATOM   2491  C22 LIG   136      -7.264 -10.040  33.996  1.00  0.00
ATOM   2492  H16 LIG   136      -7.362  -9.357  34.831  1.00  0.00
TER
ATOM   2493  S1  LIG   137      30.663  -5.395 -12.685  1.00  0.00
ATOM   2494  S2  LIG   137      32.034  -0.452 -18.515  1.00  0.00
ATOM   2495  O1  LIG   137      31.636   1.380 -13.632  1.00  0.00
ATOM   2496  H5  LIG   137      31.862   2.150 -14.196  1.00  0.00
ATOM   2497  O2  LIG   137      29.190  -5.531 -12.063  1.00  0.00
ATOM   2498  H6  LIG   137      29.218  -4.924 -11.263  1.00  0.00
ATOM   2499  O3  LIG   137      33.507   0.165 -18.698  1.00  0.00
ATOM   2500  H7  LIG   137      33.455   1.053 -18.240  1.00  0.00
ATOM   2501  O4  LIG   137      30.667  -6.148 -13.923  1.00  0.00
ATOM   2502  O5  LIG   137      31.663  -5.641 -11.656  1.00  0.00
ATOM   2503  O6  LIG   137      32.070  -1.793 -19.062  1.00  0.00
ATOM   2504  O7  LIG   137      31.039   0.515 -18.953  1.00  0.00
ATOM   2505  O8  LIG   137      29.857  -3.145  -3.993  1.00  0.00
ATOM   2506  O9  LIG   137      28.317  -4.649  -4.269  1.00  0.00
ATOM   2507  N1  LIG   137      30.872  -0.231 -11.667  1.00  0.00
```

ATOM	2508	H3	LIG	137	31.120	0.713	-11.915	1.00	0.00
ATOM	2509	H4	LIG	137	30.658	-0.484	-10.714	1.00	0.00
ATOM	2510	N2	LIG	137	30.415	-2.638	-10.790	1.00	0.00
ATOM	2511	N3	LIG	137	32.222	1.870	-16.060	1.00	0.00
ATOM	2512	N4	LIG	137	29.846	-3.653	-10.292	1.00	0.00
ATOM	2513	N5	LIG	137	32.808	2.225	-17.119	1.00	0.00
ATOM	2514	N6	LIG	137	29.162	-3.872	-4.692	1.00	0.00
ATOM	2515	C1	LIG	137	31.292	-0.968	-13.949	1.00	0.00
ATOM	2516	C2	LIG	137	31.310	-2.057	-14.866	1.00	0.00
ATOM	2517	C3	LIG	137	30.963	-1.226	-12.566	1.00	0.00
ATOM	2518	C4	LIG	137	30.751	-3.625	-13.103	1.00	0.00
ATOM	2519	C5	LIG	137	31.046	-3.374	-14.414	1.00	0.00
ATOM	2520	H1	LIG	137	31.102	-4.197	-15.114	1.00	0.00
ATOM	2521	C6	LIG	137	31.611	0.316	-14.462	1.00	0.00
ATOM	2522	C7	LIG	137	31.892	-0.584	-16.711	1.00	0.00
ATOM	2523	C8	LIG	137	31.593	-1.833	-16.235	1.00	0.00
ATOM	2524	H2	LIG	137	31.563	-2.666	-16.926	1.00	0.00
ATOM	2525	C9	LIG	137	30.684	-2.567	-12.139	1.00	0.00
ATOM	2526	C10	LIG	137	31.932	0.533	-15.823	1.00	0.00
ATOM	2527	C11	LIG	137	29.716	-3.657	-8.890	1.00	0.00
ATOM	2528	C12	LIG	137	33.008	3.607	-17.302	1.00	0.00
ATOM	2529	C13	LIG	137	30.494	-2.854	-8.038	1.00	0.00

```
ATOM   2530  H8  LIG   137      31.250  -2.206  -8.459  1.00  0.00
ATOM   2531  C14 LIG   137      28.782  -4.552  -8.351  1.00  0.00
ATOM   2532  H9  LIG   137      28.205  -5.184  -9.014  1.00  0.00
ATOM   2533  C15 LIG   137      30.312  -2.924  -6.667  1.00  0.00
ATOM   2534  H10 LIG   137      30.902  -2.325  -5.987  1.00  0.00
ATOM   2535  C16 LIG   137      28.590  -4.621  -6.979  1.00  0.00
ATOM   2536  H11 LIG   137      27.867  -5.295  -6.542  1.00  0.00
ATOM   2537  C17 LIG   137      29.356  -3.800  -6.156  1.00  0.00
ATOM   2538  C18 LIG   137      32.343   4.602 -16.566  1.00  0.00
ATOM   2539  H12 LIG   137      31.605   4.321 -15.826  1.00  0.00
ATOM   2540  C19 LIG   137      33.915   3.967 -18.309  1.00  0.00
ATOM   2541  H13 LIG   137      34.403   3.192 -18.887  1.00  0.00
ATOM   2542  C20 LIG   137      32.613   5.937 -16.823  1.00  0.00
ATOM   2543  H14 LIG   137      32.094   6.708 -16.265  1.00  0.00
ATOM   2544  C21 LIG   137      34.187   5.308 -18.551  1.00  0.00
ATOM   2545  H15 LIG   137      34.894   5.583 -19.323  1.00  0.00
ATOM   2546  C22 LIG   137      33.539   6.295 -17.809  1.00  0.00
ATOM   2547  H16 LIG   137      33.740   7.342 -18.006  1.00  0.00
TER
END
```

Table C: The number of atoms present in the system of AH-SIs

ATOM	1	N	GLY	1	23.437	-11.685	-2.766	1.00	0.00
ATOM	2	H1	GLY	1	22.736	-10.970	-2.890	1.00	0.00
ATOM	3	H2	GLY	1	23.052	-12.572	-3.058	1.00	0.00
ATOM	4	H3	GLY	1	24.308	-11.359	-3.161	1.00	0.00
ATOM	5	CA	GLY	1	23.697	-11.830	-1.306	1.00	0.00
ATOM	6	HA2	GLY	1	23.465	-12.846	-0.985	1.00	0.00
ATOM	7	HA3	GLY	1	24.743	-11.612	-1.090	1.00	0.00
ATOM	8	C	GLY	1	22.813	-10.849	-0.532	1.00	0.00
ATOM	9	O	GLY	1	23.284	-9.860	-0.005	1.00	0.00
ATOM	10	N	PRO	2	21.506	-11.134	-0.466	1.00	0.00
ATOM	11	CD	PRO	2	20.863	-12.310	-1.078	1.00	0.00
ATOM	12	HD2	PRO	2	21.424	-13.208	-0.817	1.00	0.00
ATOM	13	HD3	PRO	2	20.849	-12.194	-2.162	1.00	0.00
ATOM	14	CG	PRO	2	19.484	-12.297	-0.483	1.00	0.00
ATOM	15	HG2	PRO	2	19.468	-12.931	0.404	1.00	0.00
ATOM	16	HG3	PRO	2	18.772	-12.679	-1.214	1.00	0.00
ATOM	17	CB	PRO	2	19.188	-10.876	-0.143	1.00	0.00
ATOM	18	HB2	PRO	2	18.519	-10.862	0.717	1.00	0.00
ATOM	19	HB3	PRO	2	18.747	-10.299	-0.956	1.00	0.00
ATOM	20	CA	PRO	2	20.542	-10.282	0.243	1.00	0.00
ATOM	21	HA	PRO	2	20.681	-9.255	-0.093	1.00	0.00
ATOM	22	C	PRO	2	20.731	-10.352	1.761	1.00	0.00

ATOM 23 O PRO 2 20.286 -9.489 2.492 1.00 0.00
ATOM 24 N MET 3 21.387 -11.373 2.240 1.00 0.00
ATOM 25 H MET 3 21.760 -12.095 1.639 1.00 0.00
ATOM 26 CA MET 3 21.602 -11.496 3.710 1.00 0.00
ATOM 27 HA MET 3 20.646 -11.584 4.226 1.00 0.00
ATOM 28 CB MET 3 22.431 -12.749 4.000 1.00 0.00
ATOM 29 HB2 MET 3 23.364 -12.707 3.437 1.00 0.00
ATOM 30 HB3 MET 3 22.653 -12.801 5.066 1.00 0.00
ATOM 31 CG MET 3 21.640 -13.991 3.583 1.00 0.00
ATOM 32 HG2 MET 3 21.453 -13.966 2.509 1.00 0.00
ATOM 33 HG3 MET 3 22.206 -14.889 3.832 1.00 0.00
ATOM 34 SD MET 3 20.058 -14.022 4.462 1.00 0.00
ATOM 35 CE MET 3 20.732 -14.100 6.140 1.00 0.00
ATOM 36 HE1 MET 3 21.349 -13.221 6.326 1.00 0.00
ATOM 37 HE2 MET 3 19.914 -14.128 6.860 1.00 0.00
ATOM 38 HE3 MET 3 21.340 -14.999 6.247 1.00 0.00
ATOM 39 C MET 3 22.346 -10.261 4.221 1.00 0.00
ATOM 40 O MET 3 22.100 -9.783 5.311 1.00 0.00
ATOM 41 N ARG 4 23.256 -9.740 3.443 1.00 0.00
ATOM 42 H ARG 4 23.468 -10.133 2.537 1.00 0.00
ATOM 43 CA ARG 4 24.013 -8.536 3.885 1.00 0.00
ATOM 44 HA ARG 4 24.597 -8.764 4.777 1.00 0.00

ATOM	45	CB	ARG	4	24.968	-8.096	2.774	1.00	0.00
ATOM	46	HB2	ARG	4	24.405	-7.929	1.856	1.00	0.00
ATOM	47	HB3	ARG	4	25.466	-7.172	3.068	1.00	0.00
ATOM	48	CG	ARG	4	26.015	-9.186	2.538	1.00	0.00
ATOM	49	HG2	ARG	4	26.520	-9.415	3.476	1.00	0.00
ATOM	50	HG3	ARG	4	25.527	-10.085	2.161	1.00	0.00
ATOM	51	CD	ARG	4	27.040	-8.695	1.514	1.00	0.00
ATOM	52	HD2	ARG	4	27.562	-7.817	1.894	1.00	0.00
ATOM	53	HD3	ARG	4	27.763	-9.482	1.297	1.00	0.00
ATOM	54	NE	ARG	4	26.343	-8.326	0.251	1.00	0.00
ATOM	55	HE	ARG	4	25.996	-9.031	-0.384	1.00	0.00
ATOM	56	CZ	ARG	4	26.169	-7.069	-0.057	1.00	0.00
ATOM	57	NH1	ARG	4	27.168	-6.233	0.027	1.00	0.00
ATOM	58	HH11	ARG	4	28.074	-6.562	0.330	1.00	0.00
ATOM	59	HH12	ARG	4	27.031	-5.262	-0.212	1.00	0.00
ATOM	60	NH2	ARG	4	24.998	-6.648	-0.448	1.00	0.00
ATOM	61	HH21	ARG	4	24.227	-7.298	-0.512	1.00	0.00
ATOM	62	HH22	ARG	4	24.868	-5.675	-0.685	1.00	0.00
ATOM	63	C	ARG	4	23.034	-7.402	4.193	1.00	0.00
ATOM	64	O	ARG	4	23.229	-6.632	5.112	1.00	0.00
ATOM	65	N	ARG	5	21.980	-7.293	3.430	1.00	0.00
ATOM	66	H	ARG	5	21.812	-7.929	2.663	1.00	0.00

ATOM	67	CA	ARG	5	20.989	-6.209	3.680	1.00	0.00
ATOM	68	HA	ARG	5	21.461	-5.233	3.573	1.00	0.00
ATOM	69	CB	ARG	5	19.850	-6.316	2.663	1.00	0.00
ATOM	70	HB2	ARG	5	19.398	-7.306	2.726	1.00	0.00
ATOM	71	HB3	ARG	5	19.097	-5.558	2.879	1.00	0.00
ATOM	72	CG	ARG	5	20.403	-6.098	1.253	1.00	0.00
ATOM	73	HG2	ARG	5	20.915	-5.137	1.207	1.00	0.00
ATOM	74	HG3	ARG	5	21.106	-6.896	1.010	1.00	0.00
ATOM	75	CD	ARG	5	19.251	-6.110	0.247	1.00	0.00
ATOM	76	HD2	ARG	5	18.653	-7.013	0.369	1.00	0.00
ATOM	77	HD3	ARG	5	18.619	-5.234	0.387	1.00	0.00
ATOM	78	NE	ARG	5	19.801	-6.086	-1.138	1.00	0.00
ATOM	79	HE	ARG	5	20.328	-5.297	-1.483	1.00	0.00
ATOM	80	CZ	ARG	5	19.606	-7.101	-1.935	1.00	0.00
ATOM	81	NH1	ARG	5	18.405	-7.588	-2.091	1.00	0.00
ATOM	82	HH11	ARG	5	17.628	-7.177	-1.593	1.00	0.00
ATOM	83	HH12	ARG	5	18.258	-8.374	-2.709	1.00	0.00
ATOM	84	NH2	ARG	5	20.612	-7.629	-2.576	1.00	0.00
ATOM	85	HH21	ARG	5	21.540	-7.250	-2.453	1.00	0.00
ATOM	86	HH22	ARG	5	20.458	-8.415	-3.192	1.00	0.00
ATOM	87	C	ARG	5	20.425	-6.347	5.095	1.00	0.00
ATOM	88	O	ARG	5	20.073	-5.373	5.730	1.00	0.00

ATOM	89	N	GLU	6	20.335	-7.550	5.593	1.00	0.00
ATOM	90	H	GLU	6	20.625	-8.365	5.070	1.00	0.00
ATOM	91	CA	GLU	6	19.793	-7.748	6.967	1.00	0.00
ATOM	92	HA	GLU	6	18.769	-7.380	7.026	1.00	0.00
ATOM	93	CB	GLU	6	19.797	-9.240	7.308	1.00	0.00
ATOM	94	HB2	GLU	6	20.808	-9.635	7.203	1.00	0.00
ATOM	95	HB3	GLU	6	19.458	-9.380	8.335	1.00	0.00
ATOM	96	CG	GLU	6	18.858	-9.983	6.356	1.00	0.00
ATOM	97	HG2	GLU	6	17.853	-9.570	6.437	1.00	0.00
ATOM	98	HG3	GLU	6	19.215	-9.870	5.332	1.00	0.00
ATOM	99	CD	GLU	6	18.830	-11.467	6.724	1.00	0.00
ATOM	100	OE1	GLU	6	19.628	-11.868	7.555	1.00	0.00
ATOM	101	OE2	GLU	6	18.008	-12.179	6.169	1.00	0.00
ATOM	102	C	GLU	6	20.665	-6.992	7.972	1.00	0.00
ATOM	103	O	GLU	6	20.190	-6.515	8.984	1.00	0.00
ATOM	104	N	ARG	7	21.936	-6.878	7.702	1.00	0.00
ATOM	105	H	ARG	7	22.338	-7.273	6.863	1.00	0.00
ATOM	106	CA	ARG	7	22.835	-6.152	8.642	1.00	0.00
ATOM	107	HA	ARG	7	22.937	-6.709	9.573	1.00	0.00
ATOM	108	CB	ARG	7	24.220	-5.999	8.010	1.00	0.00
ATOM	109	HB2	ARG	7	24.591	-6.978	7.706	1.00	0.00
ATOM	110	HB3	ARG	7	24.152	-5.350	7.137	1.00	0.00

ATOM	111	CG	ARG	7	25.181	-5.384	9.029	1.00	0.00
ATOM	112	HG2	ARG	7	24.793	-4.421	9.360	1.00	0.00
ATOM	113	HG3	ARG	7	25.278	-6.051	9.886	1.00	0.00
ATOM	114	CD	ARG	7	26.553	-5.185	8.381	1.00	0.00
ATOM	115	HD2	ARG	7	26.915	-6.126	7.966	1.00	0.00
ATOM	116	HD3	ARG	7	26.492	-4.439	7.589	1.00	0.00
ATOM	117	NE	ARG	7	27.523	-4.712	9.409	1.00	0.00
ATOM	118	HE	ARG	7	27.951	-5.351	10.065	1.00	0.00
ATOM	119	CZ	ARG	7	27.837	-3.447	9.476	1.00	0.00
ATOM	120	NH1	ARG	7	27.246	-2.674	10.345	1.00	0.00
ATOM	121	HH11	ARG	7	26.547	-3.058	10.964	1.00	0.00
ATOM	122	HH12	ARG	7	27.491	-1.696	10.395	1.00	0.00
ATOM	123	NH2	ARG	7	28.742	-2.956	8.674	1.00	0.00
ATOM	124	HH21	ARG	7	29.198	-3.558	8.003	1.00	0.00
ATOM	125	HH22	ARG	7	28.983	-1.977	8.728	1.00	0.00
ATOM	126	C	ARG	7	22.254	-4.768	8.937	1.00	0.00
ATOM	127	O	ARG	7	22.275	-4.299	10.058	1.00	0.00
ATOM	128	N	GLY	8	21.733	-4.109	7.938	1.00	0.00
ATOM	129	H	GLY	8	21.711	-4.492	7.004	1.00	0.00
ATOM	130	CA	GLY	8	21.151	-2.755	8.161	1.00	0.00
ATOM	131	HA2	GLY	8	21.927	-2.067	8.495	1.00	0.00
ATOM	132	HA3	GLY	8	20.709	-2.383	7.237	1.00	0.00

ATOM	133	C	GLY	8	20.064	-2.839	9.234	1.00	0.00
ATOM	134	O	GLY	8	19.850	-1.910	9.987	1.00	0.00
ATOM	135	N	ARG	9	19.373	-3.944	9.309	1.00	0.00
ATOM	136	H	ARG	9	19.543	-4.720	8.685	1.00	0.00
ATOM	137	CA	ARG	9	18.301	-4.084	10.335	1.00	0.00
ATOM	138	HA	ARG	9	17.501	-3.369	10.146	1.00	0.00
ATOM	139	CB	ARG	9	17.719	-5.497	10.274	1.00	0.00
ATOM	140	HB2	ARG	9	17.356	-5.699	9.266	1.00	0.00
ATOM	141	HB3	ARG	9	18.492	-6.221	10.532	1.00	0.00
ATOM	142	CG	ARG	9	16.560	-5.613	11.267	1.00	0.00
ATOM	143	HG2	ARG	9	16.915	-5.375	12.270	1.00	0.00
ATOM	144	HG3	ARG	9	15.770	-4.917	10.986	1.00	0.00
ATOM	145	CD	ARG	9	16.012	-7.042	11.247	1.00	0.00
ATOM	146	HD2	ARG	9	15.729	-7.324	10.233	1.00	0.00
ATOM	147	HD3	ARG	9	16.762	-7.740	11.621	1.00	0.00
ATOM	148	NE	ARG	9	14.807	-7.124	12.120	1.00	0.00
ATOM	149	HE	ARG	9	13.954	-6.636	11.890	1.00	0.00
ATOM	150	CZ	ARG	9	14.840	-7.840	13.210	1.00	0.00
ATOM	151	NH1	ARG	9	15.314	-9.055	13.180	1.00	0.00
ATOM	152	HH11	ARG	9	15.655	-9.440	12.310	1.00	0.00
ATOM	153	HH12	ARG	9	15.338	-9.606	14.026	1.00	0.00
ATOM	154	NH2	ARG	9	14.399	-7.339	14.332	1.00	0.00

ATOM	155	HH21	ARG	9	14.034	-6.398	14.352	1.00	0.00
ATOM	156	HH22	ARG	9	14.426	-7.895	15.175	1.00	0.00
ATOM	157	C	ARG	9	18.891	-3.833	11.724	1.00	0.00
ATOM	158	O	ARG	9	18.242	-3.288	12.595	1.00	0.00
ATOM	159	N	GLN	10	20.117	-4.228	11.939	1.00	0.00
ATOM	160	H	GLN	10	20.663	-4.682	11.220	1.00	0.00
ATOM	161	CA	GLN	10	20.746	-4.012	13.272	1.00	0.00
ATOM	162	HA	GLN	10	20.231	-4.598	14.034	1.00	0.00
ATOM	163	CB	GLN	10	22.209	-4.456	13.221	1.00	0.00
ATOM	164	HB2	GLN	10	22.716	-3.946	12.402	1.00	0.00
ATOM	165	HB3	GLN	10	22.699	-4.206	14.162	1.00	0.00
ATOM	166	CG	GLN	10	22.276	-5.969	12.999	1.00	0.00
ATOM	167	HG2	GLN	10	21.696	-6.489	13.762	1.00	0.00
ATOM	168	HG3	GLN	10	21.889	-6.225	12.012	1.00	0.00
ATOM	169	CD	GLN	10	23.730	-6.434	13.089	1.00	0.00
ATOM	170	OE1	GLN	10	24.391	-6.686	11.992	1.00	0.00
ATOM	171	NE2	GLN	10	24.271	-6.570	14.168	1.00	0.00
ATOM	172	HE21	GLN	10	25.232	-6.878	14.212	1.00	0.00
ATOM	173	HE22	GLN	10	23.757	-6.375	15.015	1.00	0.00
ATOM	174	C	GLN	10	20.676	-2.527	13.634	1.00	0.00
ATOM	175	O	GLN	10	20.518	-2.166	14.783	1.00	0.00
ATOM	176	N	GLY	11	20.793	-1.664	12.663	1.00	0.00

ATOM	177	H	GLY	11	20.925	-1.958	11.706	1.00	0.00
ATOM	178	CA	GLY	11	20.734	-0.203	12.952	1.00	0.00
ATOM	179	HA2	GLY	11	21.604	0.096	13.537	1.00	0.00
ATOM	180	HA3	GLY	11	20.712	0.360	12.019	1.00	0.00
ATOM	181	C	GLY	11	19.466	0.107	13.751	1.00	0.00
ATOM	182	O	GLY	11	19.459	0.962	14.613	1.00	0.00
ATOM	183	N	ASP	12	18.394	-0.582	13.470	1.00	0.00
ATOM	184	H	ASP	12	18.392	-1.294	12.753	1.00	0.00
ATOM	185	CA	ASP	12	17.129	-0.325	14.215	1.00	0.00
ATOM	186	HA	ASP	12	16.319	-0.928	13.803	1.00	0.00
ATOM	187	CB	ASP	12	17.317	-0.699	15.686	1.00	0.00
ATOM	188	HB2	ASP	12	18.170	-0.156	16.094	1.00	0.00
ATOM	189	HB3	ASP	12	16.419	-0.438	16.245	1.00	0.00
ATOM	190	CG	ASP	12	17.569	-2.204	15.801	1.00	0.00
ATOM	191	OD1	ASP	12	17.331	-2.900	14.828	1.00	0.00
ATOM	192	OD2	ASP	12	17.996	-2.634	16.860	1.00	0.00
ATOM	193	C	ASP	12	16.765	1.157	14.108	1.00	0.00
ATOM	194	O	ASP	12	16.447	1.800	15.088	1.00	0.00
ATOM	195	N	SER	13	16.810	1.705	12.924	1.00	0.00
ATOM	196	H	SER	13	17.074	1.177	12.104	1.00	0.00
ATOM	197	CA	SER	13	16.466	3.145	12.757	1.00	0.00
ATOM	198	HA	SER	13	17.179	3.769	13.296	1.00	0.00

ATOM	199	CB	SER	13	16.519	3.513	11.273	1.00	0.00
ATOM	200	HB2	SER	13	15.757	2.952	10.731	1.00	0.00
ATOM	201	HB3	SER	13	16.336	4.581	11.156	1.00	0.00
ATOM	202	OG	SER	13	17.801	3.193	10.752	1.00	0.00
ATOM	203	HG	SER	13	17.835	3.423	9.821	1.00	0.00
ATOM	204	C	SER	13	15.056	3.396	13.295	1.00	0.00
ATOM	205	O	SER	13	14.783	4.417	13.895	1.00	0.00
ATOM	206	N	SER	14	14.159	2.472	13.085	1.00	0.00
ATOM	207	H	SER	14	14.381	1.620	12.588	1.00	0.00
ATOM	208	CA	SER	14	12.766	2.657	13.585	1.00	0.00
ATOM	209	HA	SER	14	12.769	3.239	14.506	1.00	0.00
ATOM	210	CB	SER	14	11.943	3.406	12.535	1.00	0.00
ATOM	211	HB2	SER	14	12.360	4.402	12.389	1.00	0.00
ATOM	212	HB3	SER	14	11.971	2.859	11.592	1.00	0.00
ATOM	213	OG	SER	14	10.598	3.516	12.979	1.00	0.00
ATOM	214	HG	SER	14	10.081	3.985	12.320	1.00	0.00
ATOM	215	C	SER	14	12.136	1.287	13.845	1.00	0.00
ATOM	216	O	SER	14	12.225	0.387	13.035	1.00	0.00
ATOM	217	N	SER	15	11.500	1.122	14.974	1.00	0.00
ATOM	218	H	SER	15	11.423	1.864	15.655	1.00	0.00
ATOM	219	CA	SER	15	10.867	-0.190	15.285	1.00	0.00
ATOM	220	HA	SER	15	11.551	-1.006	15.050	1.00	0.00

ATOM	221	CB	SER	15	10.527	-0.251	16.776	1.00	0.00
ATOM	222	HB2	SER	15	10.190	-1.256	17.032	1.00	0.00
ATOM	223	HB3	SER	15	11.413	-0.006	17.362	1.00	0.00
ATOM	224	OG	SER	15	9.496	0.682	17.064	1.00	0.00
ATOM	225	HG	SER	15	9.283	0.644	17.999	1.00	0.00
ATOM	226	C	SER	15	9.587	-0.351	14.462	1.00	0.00
ATOM	227	O	SER	15	9.167	-1.450	14.160	1.00	0.00
ATOM	228	N	CYX	16	8.964	0.737	14.095	1.00	0.00
ATOM	229	H	CYX	16	9.306	1.654	14.342	1.00	0.00
ATOM	230	CA	CYX	16	7.714	0.642	13.289	1.00	0.00
ATOM	231	HA	CYX	16	6.953	0.086	13.837	1.00	0.00
ATOM	232	CB	CYX	16	7.182	2.047	13.002	1.00	0.00
ATOM	233	HB2	CYX	16	7.205	2.638	13.917	1.00	0.00
ATOM	234	HB3	CYX	16	7.805	2.524	12.246	1.00	0.00
ATOM	235	SG	CYX	16	5.459	1.941	12.455	1.00	0.00
ATOM	236	C	CYX	16	8.015	-0.069	11.968	1.00	0.00
ATOM	237	O	CYX	16	7.145	-0.655	11.355	1.00	0.00
ATOM	238	N	GLU	17	9.241	-0.022	11.525	1.00	0.00
ATOM	239	H	GLU	17	9.969	0.463	12.029	1.00	0.00
ATOM	240	CA	GLU	17	9.597	-0.695	10.245	1.00	0.00
ATOM	241	HA	GLU	17	8.997	-0.296	9.427	1.00	0.00
ATOM	242	CB	GLU	17	11.074	-0.447	9.934	1.00	0.00

ATOM	243	HB2	GLU	17	11.665	-0.580	10.841	1.00	0.00
ATOM	244	HB3	GLU	17	11.410	-1.154	9.175	1.00	0.00
ATOM	245	CG	GLU	17	11.252	0.982	9.414	1.00	0.00
ATOM	246	HG2	GLU	17	10.626	1.129	8.534	1.00	0.00
ATOM	247	HG3	GLU	17	10.961	1.690	10.189	1.00	0.00
ATOM	248	CD	GLU	17	12.718	1.209	9.040	1.00	0.00
ATOM	249	OE1	GLU	17	13.489	0.271	9.148	1.00	0.00
ATOM	250	OE2	GLU	17	13.044	2.319	8.652	1.00	0.00
ATOM	251	C	GLU	17	9.347	-2.199	10.374	1.00	0.00
ATOM	252	O	GLU	17	8.731	-2.810	9.523	1.00	0.00
ATOM	253	N	ARG	18	9.820	-2.801	11.431	1.00	0.00
ATOM	254	H	ARG	18	10.332	-2.300	12.143	1.00	0.00
ATOM	255	CA	ARG	18	9.609	-4.266	11.612	1.00	0.00
ATOM	256	HA	ARG	18	10.190	-4.825	10.878	1.00	0.00
ATOM	257	CB	ARG	18	10.065	-4.679	13.013	1.00	0.00
ATOM	258	HB2	ARG	18	11.120	-4.435	13.140	1.00	0.00
ATOM	259	HB3	ARG	18	9.477	-4.146	13.760	1.00	0.00
ATOM	260	CG	ARG	18	9.867	-6.186	13.189	1.00	0.00
ATOM	261	HG2	ARG	18	8.819	-6.437	13.028	1.00	0.00
ATOM	262	HG3	ARG	18	10.483	-6.721	12.466	1.00	0.00
ATOM	263	CD	ARG	18	10.276	-6.592	14.607	1.00	0.00
ATOM	264	HD2	ARG	18	10.347	-7.677	14.683	1.00	0.00

ATOM	265	HD3	ARG	18	11.237	-6.147	14.864	1.00	0.00
ATOM	266	NE	ARG	18	9.252	-6.111	15.577	1.00	0.00
ATOM	267	HE	ARG	18	8.494	-5.510	15.288	1.00	0.00
ATOM	268	CZ	ARG	18	9.324	-6.464	16.832	1.00	0.00
ATOM	269	NH1	ARG	18	9.824	-5.642	17.714	1.00	0.00
ATOM	270	HH11	ARG	18	10.155	-4.734	17.422	1.00	0.00
ATOM	271	HH12	ARG	18	9.878	-5.919	18.684	1.00	0.00
ATOM	272	NH2	ARG	18	8.897	-7.640	17.203	1.00	0.00
ATOM	273	HH21	ARG	18	8.512	-8.274	16.517	1.00	0.00
ATOM	274	HH22	ARG	18	8.954	-7.912	18.174	1.00	0.00
ATOM	275	C	ARG	18	8.123	-4.591	11.444	1.00	0.00
ATOM	276	O	ARG	18	7.751	-5.717	11.177	1.00	0.00
ATOM	277	N	GLN	19	7.270	-3.616	11.601	1.00	0.00
ATOM	278	H	GLN	19	7.571	-2.679	11.825	1.00	0.00
ATOM	279	CA	GLN	19	5.811	-3.873	11.453	1.00	0.00
ATOM	280	HA	GLN	19	5.539	-4.805	11.949	1.00	0.00
ATOM	281	CB	GLN	19	5.024	-2.729	12.095	1.00	0.00
ATOM	282	HB2	GLN	19	5.421	-1.775	11.749	1.00	0.00
ATOM	283	HB3	GLN	19	3.974	-2.809	11.816	1.00	0.00
ATOM	284	CG	GLN	19	5.155	-2.813	13.617	1.00	0.00
ATOM	285	HG2	GLN	19	4.896	-3.814	13.963	1.00	0.00
ATOM	286	HG3	GLN	19	6.174	-2.576	13.923	1.00	0.00

ATOM 287 CD GLN 19 4.203 -1.809 14.269 1.00 0.00

ATOM 288 OE1 GLN 19 3.724 -0.899 13.622 1.00 0.00

ATOM 289 NE2 GLN 19 3.906 -1.935 15.534 1.00 0.00

ATOM 290 HE21 GLN 19 3.278 -1.276 15.970 1.00 0.00

ATOM 291 HE22 GLN 19 4.306 -2.692 16.070 1.00 0.00

ATOM 292 C GLN 19 5.455 -3.968 9.967 1.00 0.00

ATOM 293 O GLN 19 4.424 -4.496 9.600 1.00 0.00

ATOM 294 N VAL 20 6.299 -3.464 9.108 1.00 0.00

ATOM 295 H VAL 20 7.158 -3.022 9.403 1.00 0.00

ATOM 296 CA VAL 20 6.003 -3.530 7.649 1.00 0.00

ATOM 297 HA VAL 20 4.965 -3.257 7.462 1.00 0.00

ATOM 298 CB VAL 20 6.910 -2.553 6.896 1.00 0.00

ATOM 299 HB VAL 20 6.918 -1.595 7.414 1.00 0.00

ATOM 300 CG1 VAL 20 8.333 -3.112 6.836 1.00 0.00

ATOM 301 HG11 VAL 20 8.326 -4.071 6.317 1.00 0.00

ATOM 302 HG12 VAL 20 8.976 -2.415 6.299 1.00 0.00

ATOM 303 HG13 VAL 20 8.713 -3.250 7.848 1.00 0.00

ATOM 304 CG2 VAL 20 6.380 -2.359 5.474 1.00 0.00

ATOM 305 HG21 VAL 20 5.368 -1.958 5.515 1.00 0.00

ATOM 306 HG22 VAL 20 7.025 -1.664 4.937 1.00 0.00

ATOM 307 HG23 VAL 20 6.369 -3.318 4.956 1.00 0.00

ATOM 308 C VAL 20 6.252 -4.954 7.145 1.00 0.00

ATOM	309	O	VAL	20	5.597	-5.423	6.234	1.00	0.00
ATOM	310	N	ASP	21	7.192	-5.646	7.730	1.00	0.00
ATOM	311	H	ASP	21	7.741	-5.262	8.486	1.00	0.00
ATOM	312	CA	ASP	21	7.480	-7.038	7.285	1.00	0.00
ATOM	313	HA	ASP	21	7.893	-7.035	6.276	1.00	0.00
ATOM	314	CB	ASP	21	8.501	-7.674	8.228	1.00	0.00
ATOM	315	HB2	ASP	21	8.191	-7.517	9.261	1.00	0.00
ATOM	316	HB3	ASP	21	8.565	-8.744	8.027	1.00	0.00
ATOM	317	CG	ASP	21	9.872	-7.031	8.004	1.00	0.00
ATOM	318	OD1	ASP	21	9.991	-6.247	7.076	1.00	0.00
ATOM	319	OD2	ASP	21	10.777	-7.333	8.763	1.00	0.00
ATOM	320	C	ASP	21	6.188	-7.858	7.303	1.00	0.00
ATOM	321	O	ASP	21	6.006	-8.765	6.516	1.00	0.00
ATOM	322	N	ARG	22	5.290	-7.544	8.195	1.00	0.00
ATOM	323	H	ARG	22	5.435	-6.792	8.853	1.00	0.00
ATOM	324	CA	ARG	22	4.010	-8.305	8.262	1.00	0.00
ATOM	325	HA	ARG	22	4.197	-9.372	8.134	1.00	0.00
ATOM	326	CB	ARG	22	3.355	-8.083	9.628	1.00	0.00
ATOM	327	HB2	ARG	22	4.075	-8.302	10.417	1.00	0.00
ATOM	328	HB3	ARG	22	3.030	-7.047	9.711	1.00	0.00
ATOM	329	CG	ARG	22	2.147	-9.009	9.770	1.00	0.00
ATOM	330	HG2	ARG	22	1.473	-8.860	8.927	1.00	0.00

```
ATOM    331  HG3 ARG    22       2.483 -10.046   9.786  1.00  0.00
ATOM    332  CD  ARG    22       1.411  -8.691  11.074  1.00  0.00
ATOM    333  HD2 ARG    22       2.109  -8.692  11.911  1.00  0.00
ATOM    334  HD3 ARG    22       0.927  -7.717  11.006  1.00  0.00
ATOM    335  NE  ARG    22       0.368  -9.727  11.321  1.00  0.00
ATOM    336  HE  ARG    22       0.243 -10.145  12.232  1.00  0.00
ATOM    337  CZ  ARG    22      -0.409 -10.114  10.346  1.00  0.00
ATOM    338  NH1 ARG    22      -1.643  -9.691  10.288  1.00  0.00
ATOM    339 HH11 ARG    22      -1.993  -9.065  10.999  1.00  0.00
ATOM    340 HH12 ARG    22      -2.242  -9.993   9.532  1.00  0.00
ATOM    341  NH2 ARG    22       0.046 -10.924   9.430  1.00  0.00
ATOM    342 HH21 ARG    22       1.001 -11.251   9.478  1.00  0.00
ATOM    343 HH22 ARG    22      -0.558 -11.221   8.677  1.00  0.00
ATOM    344  C   ARG    22       3.072  -7.818   7.159  1.00  0.00
ATOM    345  O   ARG    22       2.118  -8.481   6.803  1.00  0.00
ATOM    346  N   VAL    23       3.334  -6.662   6.613  1.00  0.00
ATOM    347  H   VAL    23       4.125  -6.106   6.905  1.00  0.00
ATOM    348  CA  VAL    23       2.458  -6.131   5.531  1.00  0.00
ATOM    349  HA  VAL    23       1.536  -5.732   5.952  1.00  0.00
ATOM    350  CB  VAL    23       3.186  -5.006   4.792  1.00  0.00
ATOM    351  HB  VAL    23       4.193  -5.333   4.532  1.00  0.00
ATOM    352  CG1 VAL    23       2.419  -4.655   3.515  1.00  0.00
```

```
ATOM    353 HG11 VAL    23       1.412  -4.328   3.774  1.00  0.00
ATOM    354 HG12 VAL    23       2.937  -3.854   2.988  1.00  0.00
ATOM    355 HG13 VAL    23       2.361  -5.534   2.872  1.00  0.00
ATOM    356  CG2 VAL    23       3.268  -3.773   5.694  1.00  0.00
ATOM    357 HG21 VAL    23       3.815  -4.024   6.603  1.00  0.00
ATOM    358 HG22 VAL    23       3.787  -2.972   5.168  1.00  0.00
ATOM    359 HG23 VAL    23       2.262  -3.445   5.954  1.00  0.00
ATOM    360  C   VAL    23       2.124  -7.254   4.548  1.00  0.00
ATOM    361  O   VAL    23       1.086  -7.879   4.633  1.00  0.00
ATOM    362  N   ASN    24       2.997  -7.514   3.616  1.00  0.00
ATOM    363  H   ASN    24       3.862  -6.999   3.541  1.00  0.00
ATOM    364  CA  ASN    24       2.731  -8.595   2.627  1.00  0.00
ATOM    365  HA  ASN    24       3.501  -8.601   1.856  1.00  0.00
ATOM    366  CB  ASN    24       2.742  -9.949   3.339  1.00  0.00
ATOM    367  HB2 ASN    24       1.973  -9.977   4.110  1.00  0.00
ATOM    368  HB3 ASN    24       2.569 -10.754   2.624  1.00  0.00
ATOM    369  CG  ASN    24       4.105 -10.167   4.000  1.00  0.00
ATOM    370  OD1 ASN    24       5.073  -9.520   3.650  1.00  0.00
ATOM    371  ND2 ASN    24       4.223 -11.057   4.947  1.00  0.00
ATOM    372 HD21 ASN    24       5.120 -11.206   5.387  1.00  0.00
ATOM    373 HD22 ASN    24       3.417 -11.593   5.235  1.00  0.00
ATOM    374  C   ASN    24       1.362  -8.367   1.981  1.00  0.00
```

ATOM	375	O	ASN	24	0.400	-9.041	2.289	1.00	0.00
ATOM	376	N	LEU	25	1.268	-7.422	1.087	1.00	0.00
ATOM	377	H	LEU	25	2.065	-6.860	0.825	1.00	0.00
ATOM	378	CA	LEU	25	-0.038	-7.151	0.422	1.00	0.00
ATOM	379	HA	LEU	25	-0.833	-7.722	0.902	1.00	0.00
ATOM	380	CB	LEU	25	-0.368	-5.661	0.537	1.00	0.00
ATOM	381	HB2	LEU	25	0.524	-5.074	0.319	1.00	0.00
ATOM	382	HB3	LEU	25	-1.153	-5.407	-0.175	1.00	0.00
ATOM	383	CG	LEU	25	-0.848	-5.352	1.957	1.00	0.00
ATOM	384	HG	LEU	25	-0.208	-5.864	2.676	1.00	0.00
ATOM	385	CD1	LEU	25	-0.785	-3.844	2.201	1.00	0.00
ATOM	386	HD11	LEU	25	-1.425	-3.333	1.482	1.00	0.00
ATOM	387	HD12	LEU	25	-1.127	-3.625	3.212	1.00	0.00
ATOM	388	HD13	LEU	25	0.242	-3.499	2.082	1.00	0.00
ATOM	389	CD2	LEU	25	-2.291	-5.836	2.124	1.00	0.00
ATOM	390	HD21	LEU	25	-2.336	-6.912	1.951	1.00	0.00
ATOM	391	HD22	LEU	25	-2.633	-5.617	3.135	1.00	0.00
ATOM	392	HD23	LEU	25	-2.931	-5.326	1.405	1.00	0.00
ATOM	393	C	LEU	25	0.054	-7.540	-1.055	1.00	0.00
ATOM	394	O	LEU	25	0.026	-6.699	-1.932	1.00	0.00
ATOM	395	N	LYS	26	0.163	-8.810	-1.337	1.00	0.00
ATOM	396	H	LYS	26	0.191	-9.511	-0.610	1.00	0.00

ATOM	397	CA	LYS	26	0.259	-9.258	-2.755	1.00	0.00
ATOM	398	HA	LYS	26	1.048	-8.713	-3.273	1.00	0.00
ATOM	399	CB	LYS	26	0.591	-10.751	-2.793	1.00	0.00
ATOM	400	HB2	LYS	26	1.448	-10.950	-2.150	1.00	0.00
ATOM	401	HB3	LYS	26	-0.267	-11.324	-2.442	1.00	0.00
ATOM	402	CG	LYS	26	0.925	-11.162	-4.229	1.00	0.00
ATOM	403	HG2	LYS	26	0.358	-12.055	-4.493	1.00	0.00
ATOM	404	HG3	LYS	26	0.664	-10.352	-4.909	1.00	0.00
ATOM	405	CD	LYS	26	2.422	-11.458	-4.340	1.00	0.00
ATOM	406	HD2	LYS	26	2.817	-11.003	-5.248	1.00	0.00
ATOM	407	HD3	LYS	26	2.939	-11.047	-3.473	1.00	0.00
ATOM	408	CE	LYS	26	2.641	-12.972	-4.393	1.00	0.00
ATOM	409	HE2	LYS	26	2.956	-13.329	-3.412	1.00	0.00
ATOM	410	HE3	LYS	26	1.711	-13.465	-4.677	1.00	0.00
ATOM	411	NZ	LYS	26	3.696	-13.287	-5.397	1.00	0.00
ATOM	412	HZ1	LYS	26	4.558	-12.832	-5.134	1.00	0.00
ATOM	413	HZ2	LYS	26	3.840	-14.286	-5.432	1.00	0.00
ATOM	414	HZ3	LYS	26	3.405	-12.958	-6.306	1.00	0.00
ATOM	415	C	LYS	26	-1.068	-9.013	-3.485	1.00	0.00
ATOM	416	O	LYS	26	-1.079	-8.659	-4.647	1.00	0.00
ATOM	417	N	PRO	27	-2.213	-9.210	-2.803	1.00	0.00
ATOM	418	CD	PRO	27	-2.322	-9.635	-1.393	1.00	0.00

```
ATOM    419  HD2 PRO    27      -1.633  -9.053  -0.781  1.00  0.00
ATOM    420  HD3 PRO    27      -2.074 -10.694  -1.310  1.00  0.00
ATOM    421  CG  PRO    27      -3.761  -9.356  -1.066  1.00  0.00
ATOM    422  HG2 PRO    27      -3.854  -8.348  -0.664  1.00  0.00
ATOM    423  HG3 PRO    27      -4.112 -10.076  -0.326  1.00  0.00
ATOM    424  CB  PRO    27      -4.508  -9.502  -2.346  1.00  0.00
ATOM    425  HB2 PRO    27      -5.382  -8.853  -2.316  1.00  0.00
ATOM    426  HB3 PRO    27      -4.821 -10.523  -2.568  1.00  0.00
ATOM    427  CA  PRO    27      -3.534  -9.012  -3.416  1.00  0.00
ATOM    428  HA  PRO    27      -3.578  -9.600  -4.333  1.00  0.00
ATOM    429  C   PRO    27      -3.795  -7.538  -3.742  1.00  0.00
ATOM    430  O   PRO    27      -4.342  -7.211  -4.777  1.00  0.00
ATOM    431  N   CYX    28      -3.399  -6.645  -2.877  1.00  0.00
ATOM    432  H   CYX    28      -2.937  -6.903  -2.016  1.00  0.00
ATOM    433  CA  CYX    28      -3.620  -5.198  -3.155  1.00  0.00
ATOM    434  HA  CYX    28      -4.674  -5.009  -3.355  1.00  0.00
ATOM    435  CB  CYX    28      -3.197  -4.372  -1.938  1.00  0.00
ATOM    436  HB2 CYX    28      -2.536  -4.967  -1.307  1.00  0.00
ATOM    437  HB3 CYX    28      -2.672  -3.477  -2.271  1.00  0.00
ATOM    438  SG  CYX    28      -4.657  -3.968  -0.947  1.00  0.00
ATOM    439  C   CYX    28      -2.774  -4.800  -4.365  1.00  0.00
ATOM    440  O   CYX    28      -3.278  -4.315  -5.363  1.00  0.00
```

ATOM	441	N	GLU	29	-1.488	-5.012	-4.292	1.00	0.00
ATOM	442	H	GLU	29	-1.061	-5.418	-3.471	1.00	0.00
ATOM	443	CA	GLU	29	-0.613	-4.661	-5.440	1.00	0.00
ATOM	444	HA	GLU	29	-0.561	-3.579	-5.557	1.00	0.00
ATOM	445	CB	GLU	29	0.798	-5.194	-5.192	1.00	0.00
ATOM	446	HB2	GLU	29	1.037	-5.112	-4.132	1.00	0.00
ATOM	447	HB3	GLU	29	0.851	-6.240	-5.496	1.00	0.00
ATOM	448	CG	GLU	29	1.802	-4.377	-6.005	1.00	0.00
ATOM	449	HG2	GLU	29	1.528	-4.409	-7.060	1.00	0.00
ATOM	450	HG3	GLU	29	1.793	-3.344	-5.659	1.00	0.00
ATOM	451	CD	GLU	29	3.204	-4.962	-5.825	1.00	0.00
ATOM	452	OE1	GLU	29	3.335	-5.912	-5.069	1.00	0.00
ATOM	453	OE2	GLU	29	4.122	-4.453	-6.445	1.00	0.00
ATOM	454	C	GLU	29	-1.182	-5.299	-6.707	1.00	0.00
ATOM	455	O	GLU	29	-1.090	-4.753	-7.789	1.00	0.00
ATOM	456	N	GLN	30	-1.766	-6.459	-6.582	1.00	0.00
ATOM	457	H	GLN	30	-1.840	-6.922	-5.687	1.00	0.00
ATOM	458	CA	GLN	30	-2.338	-7.137	-7.776	1.00	0.00
ATOM	459	HA	GLN	30	-1.605	-7.164	-8.583	1.00	0.00
ATOM	460	CB	GLN	30	-2.718	-8.574	-7.414	1.00	0.00
ATOM	461	HB2	GLN	30	-3.137	-8.598	-6.408	1.00	0.00
ATOM	462	HB3	GLN	30	-3.457	-8.945	-8.124	1.00	0.00

```
ATOM    463  CG  GLN    30      -1.470  -9.460  -7.467  1.00  0.00
ATOM    464  HG2 GLN    30      -1.150  -9.600  -8.500  1.00  0.00
ATOM    465  HG3 GLN    30      -0.659  -9.009  -6.895  1.00  0.00
ATOM    466  CD  GLN    30      -1.790 -10.828  -6.865  1.00  0.00
ATOM    467  OE1 GLN    30      -2.887 -11.058  -6.395  1.00  0.00
ATOM    468  NE2 GLN    30      -0.871 -11.755  -6.857  1.00  0.00
ATOM    469 HE21 GLN    30      -1.075 -12.662  -6.461  1.00  0.00
ATOM    470 HE22 GLN    30       0.041 -11.562  -7.247  1.00  0.00
ATOM    471  C   GLN    30      -3.582  -6.382  -8.251  1.00  0.00
ATOM    472  O   GLN    30      -3.958  -6.459  -9.404  1.00  0.00
ATOM    473  N   HID    31      -4.224  -5.648  -7.382  1.00  0.00
ATOM    474  H   HID    31      -3.923  -5.574  -6.421  1.00  0.00
ATOM    475  CA  HID    31      -5.434  -4.892  -7.803  1.00  0.00
ATOM    476  HA  HID    31      -6.047  -5.500  -8.468  1.00  0.00
ATOM    477  CB  HID    31      -6.264  -4.529  -6.567  1.00  0.00
ATOM    478  HB2 HID    31      -6.937  -5.337  -6.280  1.00  0.00
ATOM    479  HB3 HID    31      -5.639  -4.259  -5.716  1.00  0.00
ATOM    480  CG  HID    31      -7.128  -3.332  -6.864  1.00  0.00
ATOM    481  ND1 HID    31      -8.199  -3.392  -7.742  1.00  0.00
ATOM    482  HD1 HID    31      -8.506  -4.204  -8.259  1.00  0.00
ATOM    483  CE1 HID    31      -8.759  -2.169  -7.783  1.00  0.00
ATOM    484  HE1 HID    31      -9.624  -2.039  -8.434  1.00  0.00
```

```
ATOM    485  NE2 HID    31      -8.124  -1.306  -6.985  1.00  0.00
ATOM    486  CD2 HID    31      -7.093  -2.038  -6.403  1.00  0.00
ATOM    487  HD2 HID    31      -6.414  -1.558  -5.698  1.00  0.00
ATOM    488  C   HID    31      -4.981  -3.624  -8.518  1.00  0.00
ATOM    489  O   HID    31      -5.714  -3.026  -9.281  1.00  0.00
ATOM    490  N   ILE    32      -3.767  -3.216  -8.277  1.00  0.00
ATOM    491  H   ILE    32      -3.157  -3.710  -7.641  1.00  0.00
ATOM    492  CA  ILE    32      -3.248  -1.990  -8.943  1.00  0.00
ATOM    493  HA  ILE    32      -4.084  -1.335  -9.187  1.00  0.00
ATOM    494  CB  ILE    32      -2.278  -1.256  -8.011  1.00  0.00
ATOM    495  HB  ILE    32      -1.315  -1.134  -8.507  1.00  0.00
ATOM    496  CG2 ILE    32      -2.851   0.116  -7.669  1.00  0.00
ATOM    497 HG21 ILE    32      -3.813  -0.006  -7.172  1.00  0.00
ATOM    498 HG22 ILE    32      -2.164   0.642  -7.006  1.00  0.00
ATOM    499 HG23 ILE    32      -2.985   0.692  -8.584  1.00  0.00
ATOM    500  CG1 ILE    32      -2.083  -2.059  -6.721  1.00  0.00
ATOM    501 HG12 ILE    32      -3.056  -2.302  -6.295  1.00  0.00
ATOM    502 HG13 ILE    32      -1.545  -2.981  -6.946  1.00  0.00
ATOM    503  CD1 ILE    32      -1.279  -1.232  -5.716  1.00  0.00
ATOM    504 HD11 ILE    32      -1.816  -0.311  -5.490  1.00  0.00
ATOM    505 HD12 ILE    32      -1.142  -1.806  -4.800  1.00  0.00
ATOM    506 HD13 ILE    32      -0.305  -0.990  -6.141  1.00  0.00
```

ATOM	507	C	ILE	32	-2.520	-2.384	-10.227	1.00	0.00
ATOM	508	O	ILE	32	-2.793	-1.863	-11.290	1.00	0.00
ATOM	509	N	MET	33	-1.601	-3.305	-10.141	1.00	0.00
ATOM	510	H	MET	33	-1.368	-3.745	-9.262	1.00	0.00
ATOM	511	CA	MET	33	-0.864	-3.734	-11.362	1.00	0.00
ATOM	512	HA	MET	33	-0.352	-2.884	-11.813	1.00	0.00
ATOM	513	CB	MET	33	0.179	-4.788	-10.984	1.00	0.00
ATOM	514	HB2	MET	33	-0.325	-5.687	-10.628	1.00	0.00
ATOM	515	HB3	MET	33	0.782	-5.033	-11.858	1.00	0.00
ATOM	516	CG	MET	33	1.083	-4.239	-9.879	1.00	0.00
ATOM	517	HG2	MET	33	1.483	-3.270	-10.177	1.00	0.00
ATOM	518	HG3	MET	33	0.512	-4.128	-8.957	1.00	0.00
ATOM	519	SD	MET	33	2.454	-5.386	-9.595	1.00	0.00
ATOM	520	CE	MET	33	3.585	-4.694	-10.826	1.00	0.00
ATOM	521	HE1	MET	33	3.794	-3.652	-10.584	1.00	0.00
ATOM	522	HE2	MET	33	4.517	-5.261	-10.823	1.00	0.00
ATOM	523	HE3	MET	33	3.128	-4.753	-11.814	1.00	0.00
ATOM	524	C	MET	33	-1.854	-4.329	-12.363	1.00	0.00
ATOM	525	O	MET	33	-1.760	-4.105	-13.552	1.00	0.00
ATOM	526	N	GLN	34	-2.804	-5.085	-11.888	1.00	0.00
ATOM	527	H	GLN	34	-2.887	-5.273	-10.899	1.00	0.00
ATOM	528	CA	GLN	34	-3.802	-5.698	-12.810	1.00	0.00

ATOM 529 HA GLN 34 -3.298 -6.296 -13.570 1.00 0.00
ATOM 530 CB GLN 34 -4.739 -6.608 -12.013 1.00 0.00
ATOM 531 HB2 GLN 34 -5.091 -6.081 -11.127 1.00 0.00
ATOM 532 HB3 GLN 34 -5.591 -6.885 -12.633 1.00 0.00
ATOM 533 CG GLN 34 -3.986 -7.870 -11.589 1.00 0.00
ATOM 534 HG2 GLN 34 -3.705 -8.456 -12.464 1.00 0.00
ATOM 535 HG3 GLN 34 -3.090 -7.605 -11.027 1.00 0.00
ATOM 536 CD GLN 34 -4.887 -8.726 -10.697 1.00 0.00
ATOM 537 OE1 GLN 34 -5.942 -8.290 -10.282 1.00 0.00
ATOM 538 NE2 GLN 34 -4.513 -9.936 -10.383 1.00 0.00
ATOM 539 HE21 GLN 34 -5.103 -10.507 -9.795 1.00 0.00
ATOM 540 HE22 GLN 34 -3.635 -10.297 -10.730 1.00 0.00
ATOM 541 C GLN 34 -4.619 -4.599 -13.492 1.00 0.00
ATOM 542 O GLN 34 -4.854 -4.640 -14.683 1.00 0.00
ATOM 543 N ARG 35 -5.062 -3.621 -12.750 1.00 0.00
ATOM 544 H ARG 35 -4.874 -3.579 -11.759 1.00 0.00
ATOM 545 CA ARG 35 -5.871 -2.529 -13.364 1.00 0.00
ATOM 546 HA ARG 35 -6.733 -2.944 -13.885 1.00 0.00
ATOM 547 CB ARG 35 -6.369 -1.588 -12.267 1.00 0.00
ATOM 548 HB2 ARG 35 -5.588 -1.456 -11.518 1.00 0.00
ATOM 549 HB3 ARG 35 -6.620 -0.622 -12.703 1.00 0.00
ATOM 550 CG ARG 35 -7.613 -2.189 -11.606 1.00 0.00

```
ATOM    551  HG2 ARG    35      -8.317  -2.507 -12.376  1.00  0.00
ATOM    552  HG3 ARG    35      -7.324  -3.049 -11.002  1.00  0.00
ATOM    553  CD  ARG    35      -8.274  -1.137 -10.713  1.00  0.00
ATOM    554  HD2 ARG    35      -8.684  -0.331 -11.320  1.00  0.00
ATOM    555  HD3 ARG    35      -9.071  -1.590 -10.124  1.00  0.00
ATOM    556  NE  ARG    35      -7.259  -0.566  -9.784  1.00  0.00
ATOM    557  HE  ARG    35      -6.411  -1.066  -9.557  1.00  0.00
ATOM    558  CZ  ARG    35      -7.456   0.604  -9.241  1.00  0.00
ATOM    559  NH1 ARG    35      -7.971   1.573  -9.948  1.00  0.00
ATOM    560 HH11 ARG    35      -8.216   1.413 -10.915  1.00  0.00
ATOM    561 HH12 ARG    35      -8.122   2.477  -9.525  1.00  0.00
ATOM    562  NH2 ARG    35      -7.136   0.806  -7.993  1.00  0.00
ATOM    563 HH21 ARG    35      -6.736   0.054  -7.450  1.00  0.00
ATOM    564 HH22 ARG    35      -7.290   1.712  -7.576  1.00  0.00
ATOM    565  C   ARG    35      -5.013  -1.742 -14.358  1.00  0.00
ATOM    566  O   ARG    35      -5.371  -1.580 -15.507  1.00  0.00
ATOM    567  N   ILE    36      -3.888  -1.247 -13.924  1.00  0.00
ATOM    568  H   ILE    36      -3.585  -1.375 -12.969  1.00  0.00
ATOM    569  CA  ILE    36      -3.014  -0.465 -14.844  1.00  0.00
ATOM    570  HA  ILE    36      -3.588   0.341 -15.300  1.00  0.00
ATOM    571  CB  ILE    36      -1.846   0.126 -14.052  1.00  0.00
ATOM    572  HB  ILE    36      -1.212   0.710 -14.719  1.00  0.00
```

ATOM 573 CG2 ILE 36 -2.390 1.030 -12.943 1.00 0.00
ATOM 574 HG21 ILE 36 -3.023 0.446 -12.276 1.00 0.00
ATOM 575 HG22 ILE 36 -1.559 1.452 -12.377 1.00 0.00
ATOM 576 HG23 ILE 36 -2.975 1.836 -13.385 1.00 0.00
ATOM 577 CG1 ILE 36 -1.023 -1.008 -13.435 1.00 0.00
ATOM 578 HG12 ILE 36 -1.695 -1.766 -13.033 1.00 0.00
ATOM 579 HG13 ILE 36 -0.389 -1.455 -14.202 1.00 0.00
ATOM 580 CD1 ILE 36 -0.147 -0.454 -12.309 1.00 0.00
ATOM 581 HD11 ILE 36 -0.779 -0.008 -11.542 1.00 0.00
ATOM 582 HD12 ILE 36 0.438 -1.263 -11.871 1.00 0.00
ATOM 583 HD13 ILE 36 0.526 0.303 -12.711 1.00 0.00
ATOM 584 C ILE 36 -2.473 -1.375 -15.949 1.00 0.00
ATOM 585 O ILE 36 -1.958 -0.913 -16.947 1.00 0.00
ATOM 586 N MET 37 -2.585 -2.665 -15.783 1.00 0.00
ATOM 587 H MET 37 -3.009 -3.063 -14.957 1.00 0.00
ATOM 588 CA MET 37 -2.073 -3.594 -16.832 1.00 0.00
ATOM 589 HA MET 37 -1.422 -3.060 -17.524 1.00 0.00
ATOM 590 CB MET 37 -1.270 -4.718 -16.175 1.00 0.00
ATOM 591 HB2 MET 37 -1.837 -5.132 -15.341 1.00 0.00
ATOM 592 HB3 MET 37 -1.075 -5.503 -16.907 1.00 0.00
ATOM 593 CG MET 37 0.059 -4.160 -15.660 1.00 0.00
ATOM 594 HG2 MET 37 0.475 -3.466 -16.390 1.00 0.00

ATOM	595	HG3	MET	37	-0.100	-3.641	-14.715	1.00	0.00
ATOM	596	SD	MET	37	1.224	-5.520	-15.401	1.00	0.00
ATOM	597	CE	MET	37	0.229	-6.449	-14.209	1.00	0.00
ATOM	598	HE1	MET	37	-0.718	-6.732	-14.668	1.00	0.00
ATOM	599	HE2	MET	37	0.768	-7.348	-13.907	1.00	0.00
ATOM	600	HE3	MET	37	0.038	-5.829	-13.333	1.00	0.00
ATOM	601	C	MET	37	-3.251	-4.190	-17.604	1.00	0.00
ATOM	602	O	MET	37	-3.100	-4.670	-18.711	1.00	0.00
ATOM	603	N	GLY	38	-4.424	-4.165	-17.033	1.00	0.00
ATOM	604	H	GLY	38	-4.558	-3.769	-16.114	1.00	0.00
ATOM	605	CA	GLY	38	-5.608	-4.731	-17.738	1.00	0.00
ATOM	606	HA2	GLY	38	-5.399	-5.752	-18.060	1.00	0.00
ATOM	607	HA3	GLY	38	-6.473	-4.730	-17.076	1.00	0.00
ATOM	608	C	GLY	38	-5.920	-3.875	-18.968	1.00	0.00
ATOM	609	O	GLY	38	-6.499	-4.341	-19.930	1.00	0.00
ATOM	610	N	GLU	39	-5.541	-2.627	-18.943	1.00	0.00
ATOM	611	H	GLU	39	-5.061	-2.234	-18.146	1.00	0.00
ATOM	612	CA	GLU	39	-5.815	-1.742	-20.110	1.00	0.00
ATOM	613	HA	GLU	39	-6.883	-1.724	-20.328	1.00	0.00
ATOM	614	CB	GLU	39	-5.356	-0.318	-19.790	1.00	0.00
ATOM	615	HB2	GLU	39	-5.817	0.012	-18.859	1.00	0.00
ATOM	616	HB3	GLU	39	-4.271	-0.301	-19.684	1.00	0.00

ATOM 617 CG GLU 39 -5.772 0.619 -20.925 1.00 0.00
ATOM 618 HG2 GLU 39 -5.395 0.232 -21.872 1.00 0.00
ATOM 619 HG3 GLU 39 -6.859 0.681 -20.965 1.00 0.00
ATOM 620 CD GLU 39 -5.191 2.012 -20.676 1.00 0.00
ATOM 621 OE1 GLU 39 -4.480 2.169 -19.696 1.00 0.00
ATOM 622 OE2 GLU 39 -5.466 2.898 -21.468 1.00 0.00
ATOM 623 C GLU 39 -5.057 -2.261 -21.333 1.00 0.00
ATOM 624 O GLU 39 -5.441 -2.022 -22.461 1.00 0.00
ATOM 625 N GLN 40 -3.981 -2.968 -21.120 1.00 0.00
ATOM 626 H GLN 40 -3.653 -3.170 -20.186 1.00 0.00
ATOM 627 CA GLN 40 -3.201 -3.501 -22.272 1.00 0.00
ATOM 628 HA GLN 40 -2.927 -2.693 -22.950 1.00 0.00
ATOM 629 CB GLN 40 -1.921 -4.164 -21.759 1.00 0.00
ATOM 630 HB2 GLN 40 -2.170 -4.874 -20.970 1.00 0.00
ATOM 631 HB3 GLN 40 -1.430 -4.691 -22.578 1.00 0.00
ATOM 632 CG GLN 40 -0.979 -3.094 -21.204 1.00 0.00
ATOM 633 HG2 GLN 40 -0.722 -2.375 -21.982 1.00 0.00
ATOM 634 HG3 GLN 40 -1.449 -2.574 -20.370 1.00 0.00
ATOM 635 CD GLN 40 0.307 -3.755 -20.705 1.00 0.00
ATOM 636 OE1 GLN 40 0.369 -4.961 -20.566 1.00 0.00
ATOM 637 NE2 GLN 40 1.345 -3.013 -20.429 1.00 0.00
ATOM 638 HE21 GLN 40 2.197 -3.445 -20.099 1.00 0.00

```
ATOM    639 HE22 GLN    40       1.292  -2.012 -20.547  1.00  0.00
ATOM    640  C   GLN    40      -4.045  -4.532 -23.023  1.00  0.00
ATOM    641  O   GLN    40      -3.942  -4.677 -24.224  1.00  0.00
ATOM    642  N   GLU    41      -4.880  -5.250 -22.322  1.00  0.00
ATOM    643  H   GLU    41      -4.969  -5.138 -21.322  1.00  0.00
ATOM    644  CA  GLU    41      -5.732  -6.270 -22.997  1.00  0.00
ATOM    645  HA  GLU    41      -5.110  -7.041 -23.453  1.00  0.00
ATOM    646  CB  GLU    41      -6.651  -6.929 -21.966  1.00  0.00
ATOM    647  HB2 GLU    41      -7.262  -6.167 -21.484  1.00  0.00
ATOM    648  HB3 GLU    41      -7.298  -7.651 -22.465  1.00  0.00
ATOM    649  CG  GLU    41      -5.805  -7.646 -20.912  1.00  0.00
ATOM    650  HG2 GLU    41      -5.171  -8.389 -21.397  1.00  0.00
ATOM    651  HG3 GLU    41      -5.180  -6.920 -20.392  1.00  0.00
ATOM    652  CD  GLU    41      -6.725  -8.340 -19.906  1.00  0.00
ATOM    653  OE1 GLU    41      -7.920  -8.107 -19.968  1.00  0.00
ATOM    654  OE2 GLU    41      -6.218  -9.093 -19.092  1.00  0.00
ATOM    655  C   GLU    41      -6.577  -5.593 -24.078  1.00  0.00
ATOM    656  O   GLU    41      -7.080  -4.504 -23.892  1.00  0.00
ATOM    657  N   GLN    42      -6.737  -6.231 -25.205  1.00  0.00
ATOM    658  H   GLN    42      -6.320  -7.137 -25.368  1.00  0.00
ATOM    659  CA  GLN    42      -7.550  -5.622 -26.294  1.00  0.00
ATOM    660  HA  GLN    42      -7.150  -4.645 -26.564  1.00  0.00
```

```
ATOM    661  CB  GLN    42      -7.507  -6.526 -27.528  1.00  0.00
ATOM    662  HB2 GLN    42      -7.852  -7.525 -27.260  1.00  0.00
ATOM    663  HB3 GLN    42      -8.153  -6.115 -28.303  1.00  0.00
ATOM    664  CG  GLN    42      -6.072  -6.606 -28.051  1.00  0.00
ATOM    665  HG2 GLN    42      -5.418  -7.048 -27.299  1.00  0.00
ATOM    666  HG3 GLN    42      -6.034  -7.205 -28.961  1.00  0.00
ATOM    667  CD  GLN    42      -5.567  -5.197 -28.371  1.00  0.00
ATOM    668  OE1 GLN    42      -6.218  -4.450 -29.073  1.00  0.00
ATOM    669  NE2 GLN    42      -4.425  -4.800 -27.881  1.00  0.00
ATOM    670 HE21 GLN    42      -4.085  -3.872 -28.088  1.00  0.00
ATOM    671 HE22 GLN    42      -3.885  -5.423 -27.297  1.00  0.00
ATOM    672  C   GLN    42      -8.997  -5.465 -25.824  1.00  0.00
ATOM    673  O   GLN    42      -9.668  -4.509 -26.156  1.00  0.00
ATOM    674  N   TYR    43      -9.484  -6.398 -25.051  1.00  0.00
ATOM    675  H   TYR    43      -8.932  -7.196 -24.769  1.00  0.00
ATOM    676  CA  TYR    43     -10.888  -6.301 -24.560  1.00  0.00
ATOM    677  HA  TYR    43     -11.372  -5.416 -24.973  1.00  0.00
ATOM    678  CB  TYR    43     -11.672  -7.539 -25.003  1.00  0.00
ATOM    679  HB2 TYR    43     -11.210  -8.431 -24.579  1.00  0.00
ATOM    680  HB3 TYR    43     -12.702  -7.464 -24.655  1.00  0.00
ATOM    681  CG  TYR    43     -11.656  -7.632 -26.510  1.00  0.00
ATOM    682  CD1 TYR    43     -12.531  -6.845 -27.269  1.00  0.00
```

ATOM	683	HD1	TYR	43	-13.222	-6.165	-26.773	1.00	0.00
ATOM	684	CE1	TYR	43	-12.517	-6.933	-28.666	1.00	0.00
ATOM	685	HE1	TYR	43	-13.198	-6.321	-29.257	1.00	0.00
ATOM	686	CZ	TYR	43	-11.630	-7.808	-29.304	1.00	0.00
ATOM	687	OH	TYR	43	-11.617	-7.894	-30.682	1.00	0.00
ATOM	688	HH	TYR	43	-12.255	-7.312	-31.100	1.00	0.00
ATOM	689	CE2	TYR	43	-10.755	-8.594	-28.545	1.00	0.00
ATOM	690	HE2	TYR	43	-10.063	-9.275	-29.041	1.00	0.00
ATOM	691	CD2	TYR	43	-10.769	-8.506	-27.149	1.00	0.00
ATOM	692	HD2	TYR	43	-10.088	-9.119	-26.558	1.00	0.00
ATOM	693	C	TYR	43	-10.888	-6.220	-23.032	1.00	0.00
ATOM	694	O	TYR	43	-9.941	-6.612	-22.380	1.00	0.00
ATOM	695	N	ASP	44	-11.944	-5.714	-22.456	1.00	0.00
ATOM	696	H	ASP	44	-12.735	-5.387	-22.991	1.00	0.00
ATOM	697	CA	ASP	44	-12.003	-5.608	-20.971	1.00	0.00
ATOM	698	HA	ASP	44	-12.009	-6.601	-20.522	1.00	0.00
ATOM	699	CB	ASP	44	-10.775	-4.849	-20.462	1.00	0.00
ATOM	700	HB2	ASP	44	-10.900	-4.625	-19.403	1.00	0.00
ATOM	701	HB3	ASP	44	-9.884	-5.462	-20.600	1.00	0.00
ATOM	702	CG	ASP	44	-10.622	-3.543	-21.245	1.00	0.00
ATOM	703	OD1	ASP	44	-11.338	-3.370	-22.217	1.00	0.00
ATOM	704	OD2	ASP	44	-9.789	-2.740	-20.858	1.00	0.00

ATOM	705	C	ASP	44	-13.272	-4.856	-20.564	1.00	0.00
ATOM	706	O	ASP	44	-13.979	-5.253	-19.660	1.00	0.00
ATOM	707	N	SER	45	-13.567	-3.770	-21.226	1.00	0.00
ATOM	708	H	SER	45	-12.984	-3.432	-21.978	1.00	0.00
ATOM	709	CA	SER	45	-14.791	-2.995	-20.878	1.00	0.00
ATOM	710	HA	SER	45	-14.669	-2.509	-19.910	1.00	0.00
ATOM	711	CB	SER	45	-15.030	-1.919	-21.939	1.00	0.00
ATOM	712	HB2	SER	45	-15.836	-1.261	-21.615	1.00	0.00
ATOM	713	HB3	SER	45	-14.119	-1.337	-22.077	1.00	0.00
ATOM	714	OG	SER	45	-15.388	-2.536	-23.167	1.00	0.00
ATOM	715	HG	SER	45	-15.538	-1.861	-23.833	1.00	0.00
ATOM	716	C	SER	45	-15.994	-3.937	-20.826	1.00	0.00
ATOM	717	O	SER	45	-16.892	-3.770	-20.025	1.00	0.00
ATOM	718	N	TYR	46	-16.021	-4.927	-21.677	1.00	0.00
ATOM	719	H	TYR	46	-15.277	-5.073	-22.345	1.00	0.00
ATOM	720	CA	TYR	46	-17.167	-5.878	-21.678	1.00	0.00
ATOM	721	HA	TYR	46	-18.100	-5.346	-21.864	1.00	0.00
ATOM	722	CB	TYR	46	-16.967	-6.916	-22.784	1.00	0.00
ATOM	723	HB2	TYR	46	-16.001	-7.406	-22.656	1.00	0.00
ATOM	724	HB3	TYR	46	-17.761	-7.661	-22.731	1.00	0.00
ATOM	725	CG	TYR	46	-17.009	-6.231	-24.129	1.00	0.00
ATOM	726	CD1	TYR	46	-18.201	-5.657	-24.585	1.00	0.00

ATOM 727 HD1 TYR 46 -19.099 -5.706 -23.969 1.00 0.00
ATOM 728 CE1 TYR 46 -18.241 -5.021 -25.832 1.00 0.00
ATOM 729 HE1 TYR 46 -19.169 -4.575 -26.187 1.00 0.00
ATOM 730 CZ TYR 46 -17.087 -4.958 -26.622 1.00 0.00
ATOM 731 OH TYR 46 -17.126 -4.332 -27.851 1.00 0.00
ATOM 732 HH TYR 46 -17.992 -3.977 -28.064 1.00 0.00
ATOM 733 CE2 TYR 46 -15.894 -5.531 -26.166 1.00 0.00
ATOM 734 HE2 TYR 46 -14.996 -5.482 -26.782 1.00 0.00
ATOM 735 CD2 TYR 46 -15.854 -6.168 -24.919 1.00 0.00
ATOM 736 HD2 TYR 46 -14.925 -6.614 -24.564 1.00 0.00
ATOM 737 C TYR 46 -17.244 -6.586 -20.323 1.00 0.00
ATOM 738 O TYR 46 -18.309 -6.765 -19.766 1.00 0.00
ATOM 739 N ASP 47 -16.124 -6.991 -19.789 1.00 0.00
ATOM 740 H ASP 47 -15.234 -6.846 -20.245 1.00 0.00
ATOM 741 CA ASP 47 -16.137 -7.687 -18.472 1.00 0.00
ATOM 742 HA ASP 47 -17.148 -8.011 -18.225 1.00 0.00
ATOM 743 CB ASP 47 -15.232 -8.919 -18.537 1.00 0.00
ATOM 744 HB2 ASP 47 -14.238 -8.627 -18.877 1.00 0.00
ATOM 745 HB3 ASP 47 -15.159 -9.368 -17.546 1.00 0.00
ATOM 746 CG ASP 47 -15.826 -9.936 -19.514 1.00 0.00
ATOM 747 OD1 ASP 47 -16.986 -9.787 -19.861 1.00 0.00
ATOM 748 OD2 ASP 47 -15.110 -10.845 -19.901 1.00 0.00

ATOM	749	C	ASP	47	-15.625	-6.737	-17.387	1.00	0.00
ATOM	750	O	ASP	47	-14.502	-6.273	-17.434	1.00	0.00
ATOM	751	N	ILE	48	-16.438	-6.442	-16.410	1.00	0.00
ATOM	752	H	ILE	48	-17.372	-6.824	-16.363	1.00	0.00
ATOM	753	CA	ILE	48	-15.996	-5.521	-15.325	1.00	0.00
ATOM	754	HA	ILE	48	-15.748	-4.549	-15.751	1.00	0.00
ATOM	755	CB	ILE	48	-17.124	-5.358	-14.304	1.00	0.00
ATOM	756	HB	ILE	48	-17.360	-6.328	-13.865	1.00	0.00
ATOM	757	CG2	ILE	48	-16.679	-4.395	-13.202	1.00	0.00
ATOM	758	HG21	ILE	48	-16.442	-3.426	-13.640	1.00	0.00
ATOM	759	HG22	ILE	48	-17.482	-4.279	-12.474	1.00	0.00
ATOM	760	HG23	ILE	48	-15.794	-4.795	-12.705	1.00	0.00
ATOM	761	CG1	ILE	48	-18.366	-4.797	-15.000	1.00	0.00
ATOM	762	HG12	ILE	48	-18.166	-3.780	-15.337	1.00	0.00
ATOM	763	HG13	ILE	48	-18.615	-5.421	-15.859	1.00	0.00
ATOM	764	CD1	ILE	48	-19.540	-4.788	-14.019	1.00	0.00
ATOM	765	HD11	ILE	48	-19.292	-4.164	-13.160	1.00	0.00
ATOM	766	HD12	ILE	48	-20.425	-4.389	-14.514	1.00	0.00
ATOM	767	HD13	ILE	48	-19.740	-5.806	-13.682	1.00	0.00
ATOM	768	C	ILE	48	-14.763	-6.105	-14.634	1.00	0.00
ATOM	769	O	ILE	48	-13.808	-5.409	-14.354	1.00	0.00
ATOM	770	N	ARG	49	-14.776	-7.381	-14.355	1.00	0.00

ATOM	771	H	ARG	49	-15.566	-7.967	-14.585	1.00	0.00
ATOM	772	CA	ARG	49	-13.605	-8.008	-13.682	1.00	0.00
ATOM	773	HA	ARG	49	-12.745	-7.339	-13.717	1.00	0.00
ATOM	774	CB	ARG	49	-13.947	-8.288	-12.217	1.00	0.00
ATOM	775	HB2	ARG	49	-14.832	-8.923	-12.164	1.00	0.00
ATOM	776	HB3	ARG	49	-13.109	-8.794	-11.738	1.00	0.00
ATOM	777	CG	ARG	49	-14.224	-6.966	-11.497	1.00	0.00
ATOM	778	HG2	ARG	49	-14.982	-6.406	-12.044	1.00	0.00
ATOM	779	HG3	ARG	49	-14.580	-7.170	-10.487	1.00	0.00
ATOM	780	CD	ARG	49	-12.935	-6.144	-11.427	1.00	0.00
ATOM	781	HD2	ARG	49	-12.138	-6.730	-10.967	1.00	0.00
ATOM	782	HD3	ARG	49	-12.628	-5.837	-12.427	1.00	0.00
ATOM	783	NE	ARG	49	-13.168	-4.925	-10.603	1.00	0.00
ATOM	784	HE	ARG	49	-14.079	-4.711	-10.224	1.00	0.00
ATOM	785	CZ	ARG	49	-12.183	-4.106	-10.356	1.00	0.00
ATOM	786	NH1	ARG	49	-10.953	-4.477	-10.584	1.00	0.00
ATOM	787	HH11	ARG	49	-10.767	-5.399	-10.952	1.00	0.00
ATOM	788	HH12	ARG	49	-10.193	-3.841	-10.392	1.00	0.00
ATOM	789	NH2	ARG	49	-12.428	-2.915	-9.881	1.00	0.00
ATOM	790	HH21	ARG	49	-13.381	-2.631	-9.706	1.00	0.00
ATOM	791	HH22	ARG	49	-11.663	-2.284	-9.691	1.00	0.00
ATOM	792	C	ARG	49	-13.257	-9.323	-14.384	1.00	0.00

ATOM	793	O	ARG	49	-14.124	-10.041	-14.841	1.00	0.00
ATOM	794	N	SER	50	-11.995	-9.642	-14.475	1.00	0.00
ATOM	795	H	SER	50	-11.269	-9.050	-14.099	1.00	0.00
ATOM	796	CA	SER	50	-11.594	-10.909	-15.148	1.00	0.00
ATOM	797	HA	SER	50	-11.845	-10.871	-16.208	1.00	0.00
ATOM	798	CB	SER	50	-10.082	-11.102	-15.010	1.00	0.00
ATOM	799	HB2	SER	50	-9.825	-11.222	-13.958	1.00	0.00
ATOM	800	HB3	SER	50	-9.776	-11.992	-15.562	1.00	0.00
ATOM	801	OG	SER	50	-9.411	-9.967	-15.534	1.00	0.00
ATOM	802	HG	SER	50	-8.462	-10.088	-15.448	1.00	0.00
ATOM	803	C	SER	50	-12.321	-12.087	-14.495	1.00	0.00
ATOM	804	O	SER	50	-12.763	-13.002	-15.162	1.00	0.00
ATOM	805	N	THR	51	-12.448	-12.072	-13.197	1.00	0.00
ATOM	806	H	THR	51	-12.083	-11.316	-12.637	1.00	0.00
ATOM	807	CA	THR	51	-13.146	-13.191	-12.504	1.00	0.00
ATOM	808	HA	THR	51	-13.844	-13.683	-13.181	1.00	0.00
ATOM	809	CB	THR	51	-12.117	-14.220	-12.032	1.00	0.00
ATOM	810	HB	THR	51	-11.454	-14.476	-12.859	1.00	0.00
ATOM	811	CG2	THR	51	-11.296	-13.635	-10.883	1.00	0.00
ATOM	812	HG21	THR	51	-11.958	-13.380	-10.056	1.00	0.00
ATOM	813	HG22	THR	51	-10.563	-14.370	-10.548	1.00	0.00
ATOM	814	HG23	THR	51	-10.780	-12.738	-11.225	1.00	0.00

ATOM	815	OG1	THR	51	-12.788	-15.392	-11.589	1.00	0.00
ATOM	816	HG1	THR	51	-12.142	-16.038	-11.293	1.00	0.00
ATOM	817	C	THR	51	-13.912	-12.646	-11.297	1.00	0.00
ATOM	818	O	THR	51	-13.586	-11.605	-10.762	1.00	0.00
ATOM	819	N	ARG	52	-14.928	-13.341	-10.864	1.00	0.00
ATOM	820	H	ARG	52	-15.204	-14.208	-11.303	1.00	0.00
ATOM	821	CA	ARG	52	-15.714	-12.861	-9.692	1.00	0.00
ATOM	822	HA	ARG	52	-16.128	-11.874	-9.894	1.00	0.00
ATOM	823	CB	ARG	52	-16.867	-13.829	-9.420	1.00	0.00
ATOM	824	HB2	ARG	52	-16.473	-14.838	-9.291	1.00	0.00
ATOM	825	HB3	ARG	52	-17.391	-13.526	-8.514	1.00	0.00
ATOM	826	CG	ARG	52	-17.838	-13.809	-10.602	1.00	0.00
ATOM	827	HG2	ARG	52	-18.154	-12.784	-10.796	1.00	0.00
ATOM	828	HG3	ARG	52	-17.343	-14.211	-11.487	1.00	0.00
ATOM	829	CD	ARG	52	-19.063	-14.666	-10.270	1.00	0.00
ATOM	830	HD2	ARG	52	-19.595	-14.250	-9.415	1.00	0.00
ATOM	831	HD3	ARG	52	-19.734	-14.714	-11.127	1.00	0.00
ATOM	832	NE	ARG	52	-18.623	-16.048	-9.931	1.00	0.00
ATOM	833	HE	ARG	52	-17.706	-16.233	-9.550	1.00	0.00
ATOM	834	CZ	ARG	52	-19.431	-17.055	-10.125	1.00	0.00
ATOM	835	NH1	ARG	52	-19.835	-17.772	-9.112	1.00	0.00
ATOM	836	HH11	ARG	52	-19.520	-17.546	-8.179	1.00	0.00

ATOM	837	HH12	ARG	52	-20.460	-18.551	-9.266	1.00	0.00
ATOM	838	NH2	ARG	52	-19.834	-17.344	-11.331	1.00	0.00
ATOM	839	HH21	ARG	52	-19.519	-16.787	-12.112	1.00	0.00
ATOM	840	HH22	ARG	52	-20.459	-18.124	-11.478	1.00	0.00
ATOM	841	C	ARG	52	-14.807	-12.792	-8.462	1.00	0.00
ATOM	842	O	ARG	52	-14.970	-11.944	-7.607	1.00	0.00
ATOM	843	N	SER	53	-13.853	-13.676	-8.365	1.00	0.00
ATOM	844	H	SER	53	-13.713	-14.384	-9.072	1.00	0.00
ATOM	845	CA	SER	53	-12.937	-13.661	-7.190	1.00	0.00
ATOM	846	HA	SER	53	-13.500	-13.814	-6.270	1.00	0.00
ATOM	847	CB	SER	53	-11.911	-14.788	-7.327	1.00	0.00
ATOM	848	HB2	SER	53	-11.351	-14.887	-6.397	1.00	0.00
ATOM	849	HB3	SER	53	-12.425	-15.725	-7.542	1.00	0.00
ATOM	850	OG	SER	53	-11.016	-14.486	-8.388	1.00	0.00
ATOM	851	HG	SER	53	-10.372	-15.193	-8.474	1.00	0.00
ATOM	852	C	SER	53	-12.213	-12.315	-7.125	1.00	0.00
ATOM	853	O	SER	53	-11.714	-11.917	-6.090	1.00	0.00
ATOM	854	N	SER	54	-12.149	-11.610	-8.221	1.00	0.00
ATOM	855	H	SER	54	-12.561	-11.935	-9.084	1.00	0.00
ATOM	856	CA	SER	54	-11.456	-10.291	-8.221	1.00	0.00
ATOM	857	HA	SER	54	-10.389	-10.425	-8.041	1.00	0.00
ATOM	858	CB	SER	54	-11.642	-9.616	-9.581	1.00	0.00

```
ATOM    859  HB2 SER    54     -11.323 -10.296 -10.371  1.00  0.00
ATOM    860  HB3 SER    54     -12.693  -9.364  -9.721  1.00  0.00
ATOM    861  OG  SER    54     -10.861  -8.432  -9.633  1.00  0.00
ATOM    862  HG  SER    54     -10.978  -8.009 -10.487  1.00  0.00
ATOM    863  C   SER    54     -12.050  -9.403  -7.126  1.00  0.00
ATOM    864  O   SER    54     -11.367  -8.592  -6.533  1.00  0.00
ATOM    865  N   ASP    55     -13.317  -9.548  -6.854  1.00  0.00
ATOM    866  H   ASP    55     -13.891 -10.219  -7.344  1.00  0.00
ATOM    867  CA  ASP    55     -13.953  -8.711  -5.797  1.00  0.00
ATOM    868  HA  ASP    55     -13.851  -7.654  -6.040  1.00  0.00
ATOM    869  CB  ASP    55     -15.442  -9.051  -5.705  1.00  0.00
ATOM    870  HB2 ASP    55     -15.562 -10.128  -5.586  1.00  0.00
ATOM    871  HB3 ASP    55     -15.879  -8.541  -4.847  1.00  0.00
ATOM    872  CG  ASP    55     -16.150  -8.597  -6.983  1.00  0.00
ATOM    873  OD1 ASP    55     -15.537  -7.874  -7.751  1.00  0.00
ATOM    874  OD2 ASP    55     -17.293  -8.980  -7.172  1.00  0.00
ATOM    875  C   ASP    55     -13.282  -8.990  -4.451  1.00  0.00
ATOM    876  O   ASP    55     -13.280  -8.160  -3.564  1.00  0.00
ATOM    877  N   GLN    56     -12.711 -10.153  -4.292  1.00  0.00
ATOM    878  H   GLN    56     -12.708 -10.848  -5.025  1.00  0.00
ATOM    879  CA  GLN    56     -12.040 -10.483  -3.003  1.00  0.00
ATOM    880  HA  GLN    56     -12.742 -10.387  -2.175  1.00  0.00
```

ATOM	881	CB	GLN	56	-11.532	-11.925	-3.048	1.00	0.00
ATOM	882	HB2	GLN	56	-10.814	-12.033	-3.862	1.00	0.00
ATOM	883	HB3	GLN	56	-11.047	-12.170	-2.103	1.00	0.00
ATOM	884	CG	GLN	56	-12.709	-12.875	-3.278	1.00	0.00
ATOM	885	HG2	GLN	56	-13.549	-12.599	-2.641	1.00	0.00
ATOM	886	HG3	GLN	56	-13.020	-12.845	-4.322	1.00	0.00
ATOM	887	CD	GLN	56	-12.285	-14.305	-2.936	1.00	0.00
ATOM	888	OE1	GLN	56	-13.068	-15.076	-2.419	1.00	0.00
ATOM	889	NE2	GLN	56	-11.069	-14.694	-3.206	1.00	0.00
ATOM	890	HE21	GLN	56	-10.782	-15.637	-2.983	1.00	0.00
ATOM	891	HE22	GLN	56	-10.420	-14.051	-3.637	1.00	0.00
ATOM	892	C	GLN	56	-10.860	-9.535	-2.782	1.00	0.00
ATOM	893	O	GLN	56	-10.557	-9.155	-1.669	1.00	0.00
ATOM	894	N	GLN	57	-10.192	-9.150	-3.835	1.00	0.00
ATOM	895	H	GLN	57	-10.438	-9.463	-4.763	1.00	0.00
ATOM	896	CA	GLN	57	-9.031	-8.228	-3.685	1.00	0.00
ATOM	897	HA	GLN	57	-8.185	-8.754	-3.241	1.00	0.00
ATOM	898	CB	GLN	57	-8.614	-7.705	-5.061	1.00	0.00
ATOM	899	HB2	GLN	57	-9.460	-7.208	-5.534	1.00	0.00
ATOM	900	HB3	GLN	57	-7.793	-6.997	-4.949	1.00	0.00
ATOM	901	CG	GLN	57	-8.160	-8.877	-5.933	1.00	0.00
ATOM	902	HG2	GLN	57	-7.272	-9.344	-5.507	1.00	0.00

ATOM 903 HG3 GLN 57 -8.955 -9.618 -6.015 1.00 0.00
ATOM 904 CD GLN 57 -7.820 -8.368 -7.335 1.00 0.00
ATOM 905 OE1 GLN 57 -8.214 -7.283 -7.713 1.00 0.00
ATOM 906 NE2 GLN 57 -7.098 -9.113 -8.128 1.00 0.00
ATOM 907 HE21 GLN 57 -6.869 -8.785 -9.055 1.00 0.00
ATOM 908 HE22 GLN 57 -6.771 -10.015 -7.811 1.00 0.00
ATOM 909 C GLN 57 -9.426 -7.052 -2.788 1.00 0.00
ATOM 910 O GLN 57 -8.697 -6.667 -1.896 1.00 0.00
ATOM 911 N GLN 58 -10.575 -6.477 -3.018 1.00 0.00
ATOM 912 H GLN 58 -11.187 -6.792 -3.757 1.00 0.00
ATOM 913 CA GLN 58 -11.014 -5.328 -2.179 1.00 0.00
ATOM 914 HA GLN 58 -10.432 -4.439 -2.422 1.00 0.00
ATOM 915 CB GLN 58 -12.492 -5.034 -2.447 1.00 0.00
ATOM 916 HB2 GLN 58 -13.093 -5.895 -2.153 1.00 0.00
ATOM 917 HB3 GLN 58 -12.800 -4.163 -1.870 1.00 0.00
ATOM 918 CG GLN 58 -12.695 -4.756 -3.938 1.00 0.00
ATOM 919 HG2 GLN 58 -11.912 -4.096 -4.312 1.00 0.00
ATOM 920 HG3 GLN 58 -12.682 -5.689 -4.502 1.00 0.00
ATOM 921 CD GLN 58 -14.050 -4.076 -4.148 1.00 0.00
ATOM 922 OE1 GLN 58 -14.192 -3.234 -5.012 1.00 0.00
ATOM 923 NE2 GLN 58 -15.058 -4.409 -3.390 1.00 0.00
ATOM 924 HE21 GLN 58 -15.955 -3.965 -3.523 1.00 0.00

ATOM	925	HE22	GLN	58	-14.937	-5.111	-2.673	1.00	0.00
ATOM	926	C	GLN	58	-10.826	-5.675	-0.701	1.00	0.00
ATOM	927	O	GLN	58	-10.394	-4.858	0.088	1.00	0.00
ATOM	928	N	ARG	59	-11.148	-6.881	-0.319	1.00	0.00
ATOM	929	H	ARG	59	-11.508	-7.566	-0.969	1.00	0.00
ATOM	930	CA	ARG	59	-10.990	-7.277	1.108	1.00	0.00
ATOM	931	HA	ARG	59	-11.726	-6.764	1.727	1.00	0.00
ATOM	932	CB	ARG	59	-11.203	-8.786	1.245	1.00	0.00
ATOM	933	HB2	ARG	59	-10.474	-9.315	0.630	1.00	0.00
ATOM	934	HB3	ARG	59	-11.078	-9.079	2.288	1.00	0.00
ATOM	935	CG	ARG	59	-12.616	-9.144	0.781	1.00	0.00
ATOM	936	HG2	ARG	59	-13.345	-8.590	1.372	1.00	0.00
ATOM	937	HG3	ARG	59	-12.730	-8.885	-0.272	1.00	0.00
ATOM	938	CD	ARG	59	-12.847	-10.646	0.964	1.00	0.00
ATOM	939	HD2	ARG	59	-12.058	-11.213	0.470	1.00	0.00
ATOM	940	HD3	ARG	59	-12.866	-10.900	2.024	1.00	0.00
ATOM	941	NE	ARG	59	-14.156	-11.023	0.359	1.00	0.00
ATOM	942	HE	ARG	59	-14.249	-11.201	-0.631	1.00	0.00
ATOM	943	CZ	ARG	59	-15.216	-11.131	1.114	1.00	0.00
ATOM	944	NH1	ARG	59	-15.515	-12.278	1.659	1.00	0.00
ATOM	945	HH11	ARG	59	-14.924	-13.081	1.495	1.00	0.00
ATOM	946	HH12	ARG	59	-16.335	-12.358	2.242	1.00	0.00

ATOM	947	NH2	ARG	59	-15.976	-10.090	1.323	1.00	0.00
ATOM	948	HH21	ARG	59	-15.741	-9.204	0.899	1.00	0.00
ATOM	949	HH22	ARG	59	-16.795	-10.176	1.907	1.00	0.00
ATOM	950	C	ARG	59	-9.585	-6.913	1.587	1.00	0.00
ATOM	951	O	ARG	59	-9.371	-6.620	2.746	1.00	0.00
ATOM	952	N	CYX	60	-8.625	-6.922	0.705	1.00	0.00
ATOM	953	H	CYX	60	-8.792	-7.162	-0.262	1.00	0.00
ATOM	954	CA	CYX	60	-7.237	-6.570	1.115	1.00	0.00
ATOM	955	HA	CYX	60	-6.787	-7.390	1.676	1.00	0.00
ATOM	956	CB	CYX	60	-6.390	-6.309	-0.134	1.00	0.00
ATOM	957	HB2	CYX	60	-6.139	-7.258	-0.609	1.00	0.00
ATOM	958	HB3	CYX	60	-6.953	-5.691	-0.833	1.00	0.00
ATOM	959	SG	CYX	60	-4.832	-5.517	0.338	1.00	0.00
ATOM	960	C	CYX	60	-7.279	-5.310	1.982	1.00	0.00
ATOM	961	O	CYX	60	-7.367	-5.379	3.193	1.00	0.00
ATOM	962	N	CYX	61	-7.217	-4.162	1.370	1.00	0.00
ATOM	963	H	CYX	61	-7.140	-4.099	0.365	1.00	0.00
ATOM	964	CA	CYX	61	-7.255	-2.897	2.157	1.00	0.00
ATOM	965	HA	CYX	61	-6.283	-2.710	2.615	1.00	0.00
ATOM	966	CB	CYX	61	-7.595	-1.726	1.234	1.00	0.00
ATOM	967	HB2	CYX	61	-7.673	-2.083	0.207	1.00	0.00
ATOM	968	HB3	CYX	61	-8.544	-1.287	1.539	1.00	0.00

ATOM	969	SG	CYX	61	-6.261	-0.503	1.297	1.00	0.00
ATOM	970	C	CYX	61	-8.319	-3.014	3.251	1.00	0.00
ATOM	971	O	CYX	61	-8.132	-2.563	4.363	1.00	0.00
ATOM	972	N	ASP	62	-9.433	-3.618	2.942	1.00	0.00
ATOM	973	H	ASP	62	-9.594	-3.996	2.019	1.00	0.00
ATOM	974	CA	ASP	62	-10.509	-3.764	3.964	1.00	0.00
ATOM	975	HA	ASP	62	-10.839	-2.784	4.307	1.00	0.00
ATOM	976	CB	ASP	62	-11.703	-4.494	3.348	1.00	0.00
ATOM	977	HB2	ASP	62	-11.372	-5.444	2.928	1.00	0.00
ATOM	978	HB3	ASP	62	-12.452	-4.679	4.117	1.00	0.00
ATOM	979	CG	ASP	62	-12.312	-3.632	2.240	1.00	0.00
ATOM	980	OD1	ASP	62	-11.958	-2.466	2.162	1.00	0.00
ATOM	981	OD2	ASP	62	-13.121	-4.150	1.488	1.00	0.00
ATOM	982	C	ASP	62	-9.978	-4.567	5.151	1.00	0.00
ATOM	983	O	ASP	62	-10.308	-4.303	6.291	1.00	0.00
ATOM	984	N	GLU	63	-9.154	-5.543	4.895	1.00	0.00
ATOM	985	H	GLU	63	-8.875	-5.770	3.951	1.00	0.00
ATOM	986	CA	GLU	63	-8.597	-6.358	6.010	1.00	0.00
ATOM	987	HA	GLU	63	-9.404	-6.790	6.602	1.00	0.00
ATOM	988	CB	GLU	63	-7.748	-7.495	5.438	1.00	0.00
ATOM	989	HB2	GLU	63	-7.144	-7.118	4.612	1.00	0.00
ATOM	990	HB3	GLU	63	-7.093	-7.887	6.216	1.00	0.00

```
ATOM    991  CG  GLU    63      -8.663  -8.611   4.932  1.00  0.00
ATOM    992  HG2 GLU    63      -9.340  -8.916   5.730  1.00  0.00
ATOM    993  HG3 GLU    63      -9.243  -8.249   4.083  1.00  0.00
ATOM    994  CD  GLU    63      -7.816  -9.808   4.497  1.00  0.00
ATOM    995  OE1 GLU    63      -6.604  -9.726   4.617  1.00  0.00
ATOM    996  OE2 GLU    63      -8.393 -10.787   4.051  1.00  0.00
ATOM    997  C   GLU    63      -7.729  -5.465   6.897  1.00  0.00
ATOM    998  O   GLU    63      -7.871  -5.443   8.103  1.00  0.00
ATOM    999  N   LEU    64      -6.830  -4.727   6.306  1.00  0.00
ATOM   1000  H   LEU    64      -6.709  -4.743   5.303  1.00  0.00
ATOM   1001  CA  LEU    64      -5.950  -3.833   7.109  1.00  0.00
ATOM   1002  HA  LEU    64      -5.475  -4.393   7.915  1.00  0.00
ATOM   1003  CB  LEU    64      -4.858  -3.251   6.208  1.00  0.00
ATOM   1004  HB2 LEU    64      -5.308  -2.585   5.473  1.00  0.00
ATOM   1005  HB3 LEU    64      -4.145  -2.693   6.816  1.00  0.00
ATOM   1006  CG  LEU    64      -4.132  -4.388   5.487  1.00  0.00
ATOM   1007  HG  LEU    64      -4.823  -4.891   4.810  1.00  0.00
ATOM   1008  CD1 LEU    64      -2.960  -3.816   4.686  1.00  0.00
ATOM   1009 HD11 LEU    64      -2.268  -3.314   5.362  1.00  0.00
ATOM   1010 HD12 LEU    64      -2.441  -4.626   4.172  1.00  0.00
ATOM   1011 HD13 LEU    64      -3.334  -3.102   3.953  1.00  0.00
ATOM   1012  CD2 LEU    64      -3.604  -5.391   6.514  1.00  0.00
```

ATOM	1013	HD21	LEU	64	-4.437	-5.799	7.086	1.00	0.00
ATOM	1014	HD22	LEU	64	-3.087	-6.201	5.999	1.00	0.00
ATOM	1015	HD23	LEU	64	-2.911	-4.890	7.189	1.00	0.00
ATOM	1016	C	LEU	64	-6.777	-2.690	7.706	1.00	0.00
ATOM	1017	O	LEU	64	-6.266	-1.853	8.422	1.00	0.00
ATOM	1018	N	ASN	65	-8.051	-2.646	7.420	1.00	0.00
ATOM	1019	H	ASN	65	-8.487	-3.338	6.828	1.00	0.00
ATOM	1020	CA	ASN	65	-8.901	-1.554	7.977	1.00	0.00
ATOM	1021	HA	ASN	65	-8.276	-0.742	8.349	1.00	0.00
ATOM	1022	CB	ASN	65	-9.813	-1.005	6.878	1.00	0.00
ATOM	1023	HB2	ASN	65	-10.499	-1.781	6.537	1.00	0.00
ATOM	1024	HB3	ASN	65	-10.383	-0.154	7.250	1.00	0.00
ATOM	1025	CG	ASN	65	-8.967	-0.545	5.692	1.00	0.00
ATOM	1026	OD1	ASN	65	-9.483	-0.558	4.493	1.00	0.00
ATOM	1027	ND2	ASN	65	-7.822	-0.170	5.855	1.00	0.00
ATOM	1028	HD21	ASN	65	-7.274	0.131	5.061	1.00	0.00
ATOM	1029	HD22	ASN	65	-7.424	-0.162	6.783	1.00	0.00
ATOM	1030	C	ASN	65	-9.760	-2.104	9.118	1.00	0.00
ATOM	1031	O	ASN	65	-10.400	-1.363	9.836	1.00	0.00
ATOM	1032	N	GLU	66	-9.782	-3.398	9.290	1.00	0.00
ATOM	1033	H	GLU	66	-9.253	-4.024	8.699	1.00	0.00
ATOM	1034	CA	GLU	66	-10.606	-3.987	10.385	1.00	0.00

ATOM	1035	HA	GLU	66	-10.781	-3.249	11.167	1.00	0.00
ATOM	1036	CB	GLU	66	-11.957	-4.430	9.820	1.00	0.00
ATOM	1037	HB2	GLU	66	-11.806	-5.251	9.119	1.00	0.00
ATOM	1038	HB3	GLU	66	-12.600	-4.763	10.635	1.00	0.00
ATOM	1039	CG	GLU	66	-12.618	-3.256	9.096	1.00	0.00
ATOM	1040	HG2	GLU	66	-12.874	-2.482	9.819	1.00	0.00
ATOM	1041	HG3	GLU	66	-11.928	-2.849	8.357	1.00	0.00
ATOM	1042	CD	GLU	66	-13.889	-3.739	8.395	1.00	0.00
ATOM	1043	OE1	GLU	66	-14.077	-4.943	8.317	1.00	0.00
ATOM	1044	OE2	GLU	66	-14.652	-2.899	7.948	1.00	0.00
ATOM	1045	C	GLU	66	-9.886	-5.198	10.986	1.00	0.00
ATOM	1046	O	GLU	66	-10.007	-5.482	12.160	1.00	0.00
ATOM	1047	N	MET	67	-9.142	-5.917	10.191	1.00	0.00
ATOM	1048	H	MET	67	-9.034	-5.692	9.212	1.00	0.00
ATOM	1049	CA	MET	67	-8.424	-7.110	10.724	1.00	0.00
ATOM	1050	HA	MET	67	-9.089	-7.703	11.352	1.00	0.00
ATOM	1051	CB	MET	67	-7.948	-7.980	9.559	1.00	0.00
ATOM	1052	HB2	MET	67	-8.118	-7.456	8.619	1.00	0.00
ATOM	1053	HB3	MET	67	-6.884	-8.188	9.671	1.00	0.00
ATOM	1054	CG	MET	67	-8.727	-9.297	9.556	1.00	0.00
ATOM	1055	HG2	MET	67	-9.147	-9.480	10.545	1.00	0.00
ATOM	1056	HG3	MET	67	-9.532	-9.248	8.823	1.00	0.00

ATOM 1057 SD MET 67 -7.612 -10.655 9.123 1.00 0.00

ATOM 1058 CE MET 67 -7.502 -10.301 7.352 1.00 0.00

ATOM 1059 HE1 MET 67 -7.094 -9.301 7.205 1.00 0.00

ATOM 1060 HE2 MET 67 -6.849 -11.033 6.875 1.00 0.00

ATOM 1061 HE3 MET 67 -8.495 -10.357 6.907 1.00 0.00

ATOM 1062 C MET 67 -7.219 -6.658 11.548 1.00 0.00

ATOM 1063 O MET 67 -7.237 -6.688 12.763 1.00 0.00

ATOM 1064 N GLU 68 -6.171 -6.242 10.897 1.00 0.00

ATOM 1065 H GLU 68 -6.150 -6.218 9.887 1.00 0.00

ATOM 1066 CA GLU 68 -4.961 -5.790 11.639 1.00 0.00

ATOM 1067 HA GLU 68 -4.755 -6.462 12.473 1.00 0.00

ATOM 1068 CB GLU 68 -3.755 -5.796 10.698 1.00 0.00

ATOM 1069 HB2 GLU 68 -3.846 -4.978 9.984 1.00 0.00

ATOM 1070 HB3 GLU 68 -2.840 -5.672 11.278 1.00 0.00

ATOM 1071 CG GLU 68 -3.704 -7.126 9.944 1.00 0.00

ATOM 1072 HG2 GLU 68 -3.499 -7.935 10.646 1.00 0.00

ATOM 1073 HG3 GLU 68 -4.662 -7.304 9.456 1.00 0.00

ATOM 1074 CD GLU 68 -2.597 -7.074 8.889 1.00 0.00

ATOM 1075 OE1 GLU 68 -2.028 -6.010 8.707 1.00 0.00

ATOM 1076 OE2 GLU 68 -2.336 -8.100 8.281 1.00 0.00

ATOM 1077 C GLU 68 -5.184 -4.374 12.175 1.00 0.00

ATOM 1078 O GLU 68 -4.302 -3.776 12.758 1.00 0.00

ATOM	1079	N	ASN	69	-6.356	-3.833	11.984	1.00	0.00
ATOM	1080	H	ASN	69	-7.096	-4.323	11.502	1.00	0.00
ATOM	1081	CA	ASN	69	-6.629	-2.455	12.484	1.00	0.00
ATOM	1082	HA	ASN	69	-7.553	-2.072	12.052	1.00	0.00
ATOM	1083	CB	ASN	69	-6.777	-2.486	14.007	1.00	0.00
ATOM	1084	HB2	ASN	69	-5.843	-2.805	14.471	1.00	0.00
ATOM	1085	HB3	ASN	69	-7.049	-1.499	14.380	1.00	0.00
ATOM	1086	CG	ASN	69	-7.877	-3.475	14.393	1.00	0.00
ATOM	1087	OD1	ASN	69	-8.812	-3.687	13.645	1.00	0.00
ATOM	1088	ND2	ASN	69	-7.806	-4.096	15.539	1.00	0.00
ATOM	1089	HD21	ASN	69	-8.529	-4.751	15.801	1.00	0.00
ATOM	1090	HD22	ASN	69	-7.028	-3.919	16.159	1.00	0.00
ATOM	1091	C	ASN	69	-5.466	-1.537	12.101	1.00	0.00
ATOM	1092	O	ASN	69	-5.209	-0.543	12.751	1.00	0.00
ATOM	1093	N	THR	70	-4.763	-1.864	11.053	1.00	0.00
ATOM	1094	H	THR	70	-4.974	-2.690	10.510	1.00	0.00
ATOM	1095	CA	THR	70	-3.615	-1.012	10.627	1.00	0.00
ATOM	1096	HA	THR	70	-2.748	-1.196	11.261	1.00	0.00
ATOM	1097	CB	THR	70	-3.242	-1.346	9.180	1.00	0.00
ATOM	1098	HB	THR	70	-2.233	-0.988	8.974	1.00	0.00
ATOM	1099	CG2	THR	70	-3.297	-2.861	8.972	1.00	0.00
ATOM	1100	HG21	THR	70	-4.305	-3.221	9.178	1.00	0.00

ATOM 1101 HG22 THR 70 -3.031 -3.097 7.941 1.00 0.00
ATOM 1102 HG23 THR 70 -2.593 -3.347 9.648 1.00 0.00
ATOM 1103 OG1 THR 70 -4.156 -0.711 8.297 1.00 0.00
ATOM 1104 HG1 THR 70 -3.922 -0.921 7.390 1.00 0.00
ATOM 1105 C THR 70 -4.010 0.463 10.724 1.00 0.00
ATOM 1106 O THR 70 -5.176 0.805 10.731 1.00 0.00
ATOM 1107 N GLN 71 -3.047 1.342 10.800 1.00 0.00
ATOM 1108 H GLN 71 -2.075 1.066 10.795 1.00 0.00
ATOM 1109 CA GLN 71 -3.370 2.794 10.897 1.00 0.00
ATOM 1110 HA GLN 71 -3.989 3.100 10.054 1.00 0.00
ATOM 1111 CB GLN 71 -4.140 3.060 12.191 1.00 0.00
ATOM 1112 HB2 GLN 71 -5.080 2.509 12.175 1.00 0.00
ATOM 1113 HB3 GLN 71 -3.543 2.735 13.043 1.00 0.00
ATOM 1114 CG GLN 71 -4.429 4.559 12.312 1.00 0.00
ATOM 1115 HG2 GLN 71 -3.517 5.133 12.149 1.00 0.00
ATOM 1116 HG3 GLN 71 -5.182 4.859 11.583 1.00 0.00
ATOM 1117 CD GLN 71 -4.957 4.866 13.716 1.00 0.00
ATOM 1118 OE1 GLN 71 -4.356 4.486 14.701 1.00 0.00
ATOM 1119 NE2 GLN 71 -6.065 5.543 13.848 1.00 0.00
ATOM 1120 HE21 GLN 71 -6.420 5.749 14.771 1.00 0.00
ATOM 1121 HE22 GLN 71 -6.562 5.857 13.027 1.00 0.00
ATOM 1122 C GLN 71 -2.073 3.607 10.899 1.00 0.00

ATOM	1123	O	GLN	71	-2.017	4.704	10.379	1.00	0.00
ATOM	1124	N	GLY	72	-1.031	3.081	11.484	1.00	0.00
ATOM	1125	H	GLY	72	-1.069	2.170	11.920	1.00	0.00
ATOM	1126	CA	GLY	72	0.258	3.828	11.520	1.00	0.00
ATOM	1127	HA2	GLY	72	0.100	4.856	11.194	1.00	0.00
ATOM	1128	HA3	GLY	72	0.660	3.825	12.533	1.00	0.00
ATOM	1129	C	GLY	72	1.266	3.158	10.583	1.00	0.00
ATOM	1130	O	GLY	72	1.128	2.003	10.230	1.00	0.00
ATOM	1131	N	CYX	73	2.280	3.873	10.180	1.00	0.00
ATOM	1132	H	CYX	73	2.400	4.833	10.470	1.00	0.00
ATOM	1133	CA	CYX	73	3.296	3.279	9.267	1.00	0.00
ATOM	1134	HA	CYX	73	4.118	3.977	9.110	1.00	0.00
ATOM	1135	CB	CYX	73	3.855	1.998	9.889	1.00	0.00
ATOM	1136	HB2	CYX	73	3.250	1.717	10.751	1.00	0.00
ATOM	1137	HB3	CYX	73	3.831	1.195	9.152	1.00	0.00
ATOM	1138	SG	CYX	73	5.545	2.293	10.468	1.00	0.00
ATOM	1139	C	CYX	73	2.645	2.952	7.920	1.00	0.00
ATOM	1140	O	CYX	73	3.230	2.300	7.078	1.00	0.00
ATOM	1141	N	MET	74	1.438	3.401	7.711	1.00	0.00
ATOM	1142	H	MET	74	0.947	3.944	8.406	1.00	0.00
ATOM	1143	CA	MET	74	0.750	3.116	6.421	1.00	0.00
ATOM	1144	HA	MET	74	0.386	2.088	6.405	1.00	0.00

ATOM	1145	CB	MET	74	-0.444	4.061	6.260	1.00	0.00
ATOM	1146	HB2	MET	74	-0.087	5.086	6.163	1.00	0.00
ATOM	1147	HB3	MET	74	-1.008	3.787	5.368	1.00	0.00
ATOM	1148	CG	MET	74	-1.348	3.949	7.490	1.00	0.00
ATOM	1149	HG2	MET	74	-1.489	2.899	7.749	1.00	0.00
ATOM	1150	HG3	MET	74	-0.893	4.472	8.331	1.00	0.00
ATOM	1151	SD	MET	74	-2.957	4.694	7.126	1.00	0.00
ATOM	1152	CE	MET	74	-3.827	3.164	6.704	1.00	0.00
ATOM	1153	HE1	MET	74	-3.811	2.488	7.560	1.00	0.00
ATOM	1154	HE2	MET	74	-4.859	3.393	6.441	1.00	0.00
ATOM	1155	HE3	MET	74	-3.334	2.687	5.857	1.00	0.00
ATOM	1156	C	MET	74	1.731	3.329	5.266	1.00	0.00
ATOM	1157	O	MET	74	1.957	2.447	4.461	1.00	0.00
ATOM	1158	N	CYX	75	2.317	4.492	5.179	1.00	0.00
ATOM	1159	H	CYX	75	2.136	5.231	5.843	1.00	0.00
ATOM	1160	CA	CYX	75	3.283	4.755	4.076	1.00	0.00
ATOM	1161	HA	CYX	75	2.754	4.878	3.131	1.00	0.00
ATOM	1162	CB	CYX	75	4.059	6.040	4.373	1.00	0.00
ATOM	1163	HB2	CYX	75	3.366	6.818	4.691	1.00	0.00
ATOM	1164	HB3	CYX	75	4.783	5.852	5.166	1.00	0.00
ATOM	1165	SG	CYX	75	4.881	6.609	2.865	1.00	0.00
ATOM	1166	C	CYX	75	4.259	3.584	3.963	1.00	0.00

ATOM	1167	O	CYX	75	4.468	3.038	2.897	1.00	0.00
ATOM	1168	N	GLU	76	4.858	3.188	5.054	1.00	0.00
ATOM	1169	H	GLU	76	4.692	3.633	5.945	1.00	0.00
ATOM	1170	CA	GLU	76	5.816	2.048	5.002	1.00	0.00
ATOM	1171	HA	GLU	76	6.659	2.292	4.356	1.00	0.00
ATOM	1172	CB	GLU	76	6.346	1.758	6.408	1.00	0.00
ATOM	1173	HB2	GLU	76	5.519	1.470	7.057	1.00	0.00
ATOM	1174	HB3	GLU	76	7.071	0.945	6.363	1.00	0.00
ATOM	1175	CG	GLU	76	7.020	3.012	6.967	1.00	0.00
ATOM	1176	HG2	GLU	76	7.772	3.364	6.261	1.00	0.00
ATOM	1177	HG3	GLU	76	6.272	3.789	7.120	1.00	0.00
ATOM	1178	CD	GLU	76	7.691	2.681	8.302	1.00	0.00
ATOM	1179	OE1	GLU	76	7.684	1.518	8.674	1.00	0.00
ATOM	1180	OE2	GLU	76	8.199	3.594	8.930	1.00	0.00
ATOM	1181	C	GLU	76	5.095	0.812	4.461	1.00	0.00
ATOM	1182	O	GLU	76	5.711	-0.129	3.999	1.00	0.00
ATOM	1183	N	ALA	77	3.790	0.813	4.506	1.00	0.00
ATOM	1184	H	ALA	77	3.272	1.591	4.887	1.00	0.00
ATOM	1185	CA	ALA	77	3.028	-0.356	3.987	1.00	0.00
ATOM	1186	HA	ALA	77	3.497	-1.304	4.254	1.00	0.00
ATOM	1187	CB	ALA	77	1.607	-0.336	4.555	1.00	0.00
ATOM	1188	HB1	ALA	77	1.122	0.602	4.288	1.00	0.00

ATOM	1189	HB2	ALA	77	1.039	-1.170	4.142	1.00	0.00
ATOM	1190	HB3	ALA	77	1.648	-0.428	5.640	1.00	0.00
ATOM	1191	C	ALA	77	2.972	-0.277	2.462	1.00	0.00
ATOM	1192	O	ALA	77	3.430	-1.164	1.768	1.00	0.00
ATOM	1193	N	LEU	78	2.422	0.780	1.932	1.00	0.00
ATOM	1194	H	LEU	78	2.040	1.523	2.499	1.00	0.00
ATOM	1195	CA	LEU	78	2.350	0.914	0.451	1.00	0.00
ATOM	1196	HA	LEU	78	1.704	0.141	0.034	1.00	0.00
ATOM	1197	CB	LEU	78	1.774	2.283	0.085	1.00	0.00
ATOM	1198	HB2	LEU	78	0.816	2.420	0.586	1.00	0.00
ATOM	1199	HB3	LEU	78	2.464	3.065	0.402	1.00	0.00
ATOM	1200	CG	LEU	78	1.574	2.363	-1.428	1.00	0.00
ATOM	1201	HG	LEU	78	2.484	2.038	-1.933	1.00	0.00
ATOM	1202	CD1	LEU	78	0.412	1.456	-1.838	1.00	0.00
ATOM	1203	HD11	LEU	78	-0.498	1.780	-1.334	1.00	0.00
ATOM	1204	HD12	LEU	78	0.269	1.513	-2.917	1.00	0.00
ATOM	1205	HD13	LEU	78	0.636	0.427	-1.555	1.00	0.00
ATOM	1206	CD2	LEU	78	1.259	3.806	-1.827	1.00	0.00
ATOM	1207	HD21	LEU	78	2.087	4.452	-1.536	1.00	0.00
ATOM	1208	HD22	LEU	78	1.117	3.862	-2.906	1.00	0.00
ATOM	1209	HD23	LEU	78	0.349	4.132	-1.323	1.00	0.00
ATOM	1210	C	LEU	78	3.760	0.781	-0.125	1.00	0.00

ATOM	1211	O	LEU	78	3.983	0.081	-1.092	1.00	0.00
ATOM	1212	N	GLN	79	4.714	1.446	0.468	1.00	0.00
ATOM	1213	H	GLN	79	4.534	2.029	1.273	1.00	0.00
ATOM	1214	CA	GLN	79	6.110	1.355	-0.039	1.00	0.00
ATOM	1215	HA	GLN	79	6.181	1.801	-1.031	1.00	0.00
ATOM	1216	CB	GLN	79	7.050	2.110	0.905	1.00	0.00
ATOM	1217	HB2	GLN	79	6.921	1.737	1.921	1.00	0.00
ATOM	1218	HB3	GLN	79	8.082	1.956	0.590	1.00	0.00
ATOM	1219	CG	GLN	79	6.722	3.604	0.864	1.00	0.00
ATOM	1220	HG2	GLN	79	6.893	4.000	-0.137	1.00	0.00
ATOM	1221	HG3	GLN	79	5.684	3.772	1.148	1.00	0.00
ATOM	1222	CD	GLN	79	7.626	4.351	1.847	1.00	0.00
ATOM	1223	OE1	GLN	79	7.849	5.625	1.671	1.00	0.00
ATOM	1224	NE2	GLN	79	8.136	3.769	2.784	1.00	0.00
ATOM	1225	HE21	GLN	79	8.731	4.274	3.425	1.00	0.00
ATOM	1226	HE22	GLN	79	7.963	2.782	2.917	1.00	0.00
ATOM	1227	C	GLN	79	6.525	-0.113	-0.105	1.00	0.00
ATOM	1228	O	GLN	79	7.089	-0.567	-1.080	1.00	0.00
ATOM	1229	N	GLN	80	6.242	-0.863	0.924	1.00	0.00
ATOM	1230	H	GLN	80	5.770	-0.496	1.737	1.00	0.00
ATOM	1231	CA	GLN	80	6.615	-2.305	0.916	1.00	0.00
ATOM	1232	HA	GLN	80	7.700	-2.414	0.897	1.00	0.00

ATOM	1233	CB	GLN	80	6.078	-2.981	2.179	1.00	0.00
ATOM	1234	HB2	GLN	80	6.402	-2.422	3.057	1.00	0.00
ATOM	1235	HB3	GLN	80	4.989	-3.004	2.145	1.00	0.00
ATOM	1236	CG	GLN	80	6.615	-4.411	2.259	1.00	0.00
ATOM	1237	HG2	GLN	80	6.155	-4.941	3.093	1.00	0.00
ATOM	1238	HG3	GLN	80	6.410	-4.946	1.332	1.00	0.00
ATOM	1239	CD	GLN	80	8.129	-4.376	2.478	1.00	0.00
ATOM	1240	OE1	GLN	80	8.616	-3.649	3.321	1.00	0.00
ATOM	1241	NE2	GLN	80	8.899	-5.136	1.748	1.00	0.00
ATOM	1242	HE21	GLN	80	9.899	-5.119	1.887	1.00	0.00
ATOM	1243	HE22	GLN	80	8.491	-5.739	1.047	1.00	0.00
ATOM	1244	C	GLN	80	6.005	-2.972	-0.318	1.00	0.00
ATOM	1245	O	GLN	80	6.670	-3.691	-1.038	1.00	0.00
ATOM	1246	N	ILE	81	4.748	-2.733	-0.571	1.00	0.00
ATOM	1247	H	ILE	81	4.190	-2.135	0.021	1.00	0.00
ATOM	1248	CA	ILE	81	4.101	-3.348	-1.763	1.00	0.00
ATOM	1249	HA	ILE	81	3.970	-4.417	-1.595	1.00	0.00
ATOM	1250	CB	ILE	81	2.736	-2.697	-1.993	1.00	0.00
ATOM	1251	HB	ILE	81	2.860	-1.619	-2.089	1.00	0.00
ATOM	1252	CG2	ILE	81	2.113	-3.254	-3.274	1.00	0.00
ATOM	1253	HG21	ILE	81	1.989	-4.333	-3.179	1.00	0.00
ATOM	1254	HG22	ILE	81	1.141	-2.790	-3.437	1.00	0.00

ATOM	1255	HG23	ILE	81	2.765	-3.038	-4.120	1.00	0.00
ATOM	1256	CG1	ILE	81	1.817	-3.001	-0.808	1.00	0.00
ATOM	1257	HG12	ILE	81	1.775	-4.079	-0.649	1.00	0.00
ATOM	1258	HG13	ILE	81	2.207	-2.517	0.087	1.00	0.00
ATOM	1259	CD1	ILE	81	0.411	-2.473	-1.102	1.00	0.00
ATOM	1260	HD11	ILE	81	0.021	-2.957	-1.997	1.00	0.00
ATOM	1261	HD12	ILE	81	-0.243	-2.690	-0.258	1.00	0.00
ATOM	1262	HD13	ILE	81	0.453	-1.396	-1.261	1.00	0.00
ATOM	1263	C	ILE	81	4.991	-3.121	-2.985	1.00	0.00
ATOM	1264	O	ILE	81	5.483	-4.054	-3.590	1.00	0.00
ATOM	1265	N	MET	82	5.208	-1.887	-3.350	1.00	0.00
ATOM	1266	H	MET	82	4.804	-1.108	-2.851	1.00	0.00
ATOM	1267	CA	MET	82	6.072	-1.600	-4.528	1.00	0.00
ATOM	1268	HA	MET	82	5.542	-1.830	-5.452	1.00	0.00
ATOM	1269	CB	MET	82	6.446	-0.116	-4.535	1.00	0.00
ATOM	1270	HB2	MET	82	7.101	0.098	-3.690	1.00	0.00
ATOM	1271	HB3	MET	82	6.963	0.124	-5.464	1.00	0.00
ATOM	1272	CG	MET	82	5.177	0.731	-4.424	1.00	0.00
ATOM	1273	HG2	MET	82	4.734	0.605	-3.436	1.00	0.00
ATOM	1274	HG3	MET	82	5.420	1.781	-4.582	1.00	0.00
ATOM	1275	SD	MET	82	3.988	0.200	-5.681	1.00	0.00
ATOM	1276	CE	MET	82	4.973	0.656	-7.130	1.00	0.00

ATOM	1277	HE1	MET	82	5.914	0.105	-7.119	1.00	0.00
ATOM	1278	HE2	MET	82	4.421	0.411	-8.037	1.00	0.00
ATOM	1279	HE3	MET	82	5.179	1.726	-7.108	1.00	0.00
ATOM	1280	C	MET	82	7.342	-2.445	-4.442	1.00	0.00
ATOM	1281	O	MET	82	7.703	-3.140	-5.369	1.00	0.00
ATOM	1282	N	GLU	83	8.018	-2.396	-3.330	1.00	0.00
ATOM	1283	H	GLU	83	7.722	-1.824	-2.553	1.00	0.00
ATOM	1284	CA	GLU	83	9.263	-3.199	-3.181	1.00	0.00
ATOM	1285	HA	GLU	83	9.942	-3.003	-4.011	1.00	0.00
ATOM	1286	CB	GLU	83	9.964	-2.816	-1.875	1.00	0.00
ATOM	1287	HB2	GLU	83	9.271	-2.934	-1.042	1.00	0.00
ATOM	1288	HB3	GLU	83	10.828	-3.463	-1.722	1.00	0.00
ATOM	1289	CG	GLU	83	10.425	-1.359	-1.953	1.00	0.00
ATOM	1290	HG2	GLU	83	11.056	-1.223	-2.832	1.00	0.00
ATOM	1291	HG3	GLU	83	9.555	-0.707	-2.026	1.00	0.00
ATOM	1292	CD	GLU	83	11.221	-1.008	-0.695	1.00	0.00
ATOM	1293	OE1	GLU	83	11.289	-1.841	0.192	1.00	0.00
ATOM	1294	OE2	GLU	83	11.751	0.090	-0.641	1.00	0.00
ATOM	1295	C	GLU	83	8.903	-4.685	-3.152	1.00	0.00
ATOM	1296	O	GLU	83	9.751	-5.543	-3.294	1.00	0.00
ATOM	1297	N	ASN	84	7.648	-4.992	-2.973	1.00	0.00
ATOM	1298	H	ASN	84	6.940	-4.283	-2.854	1.00	0.00

ATOM	1299	CA	ASN	84	7.229	-6.420	-2.939	1.00	0.00
ATOM	1300	HA	ASN	84	7.967	-7.018	-2.403	1.00	0.00
ATOM	1301	CB	ASN	84	5.884	-6.542	-2.219	1.00	0.00
ATOM	1302	HB2	ASN	84	5.927	-6.043	-1.251	1.00	0.00
ATOM	1303	HB3	ASN	84	5.091	-6.099	-2.821	1.00	0.00
ATOM	1304	CG	ASN	84	5.559	-8.019	-1.993	1.00	0.00
ATOM	1305	OD1	ASN	84	6.180	-8.673	-1.179	1.00	0.00
ATOM	1306	ND2	ASN	84	4.604	-8.578	-2.685	1.00	0.00
ATOM	1307	HD21	ASN	84	4.384	-9.554	-2.541	1.00	0.00
ATOM	1308	HD22	ASN	84	4.089	-8.033	-3.362	1.00	0.00
ATOM	1309	C	ASN	84	7.092	-6.943	-4.370	1.00	0.00
ATOM	1310	O	ASN	84	7.141	-8.133	-4.612	1.00	0.00
ATOM	1311	N	GLN	85	6.919	-6.065	-5.321	1.00	0.00
ATOM	1312	H	GLN	85	6.874	-5.074	-5.131	1.00	0.00
ATOM	1313	CA	GLN	85	6.781	-6.521	-6.733	1.00	0.00
ATOM	1314	HA	GLN	85	7.587	-7.209	-6.990	1.00	0.00
ATOM	1315	CB	GLN	85	5.445	-7.248	-6.905	1.00	0.00
ATOM	1316	HB2	GLN	85	5.325	-7.983	-6.109	1.00	0.00
ATOM	1317	HB3	GLN	85	4.629	-6.527	-6.859	1.00	0.00
ATOM	1318	CG	GLN	85	5.422	-7.957	-8.261	1.00	0.00
ATOM	1319	HG2	GLN	85	5.528	-7.233	-9.069	1.00	0.00
ATOM	1320	HG3	GLN	85	6.230	-8.687	-8.321	1.00	0.00

ATOM	1321	CD	GLN	85	4.089	-8.688	-8.431	1.00	0.00
ATOM	1322	OE1	GLN	85	3.387	-8.928	-7.469	1.00	0.00
ATOM	1323	NE2	GLN	85	3.708	-9.055	-9.624	1.00	0.00
ATOM	1324	HE21	GLN	85	2.829	-9.538	-9.744	1.00	0.00
ATOM	1325	HE22	GLN	85	4.294	-8.855	-10.422	1.00	0.00
ATOM	1326	C	GLN	85	6.827	-5.314	-7.676	1.00	0.00
ATOM	1327	O	GLN	85	6.009	-5.179	-8.563	1.00	0.00
ATOM	1328	N	CYX	86	7.781	-4.439	-7.497	1.00	0.00
ATOM	1329	H	CYX	86	8.468	-4.541	-6.763	1.00	0.00
ATOM	1330	CA	CYX	86	7.874	-3.250	-8.393	1.00	0.00
ATOM	1331	HA	CYX	86	6.892	-3.007	-8.799	1.00	0.00
ATOM	1332	CB	CYX	86	8.387	-2.046	-7.600	1.00	0.00
ATOM	1333	HB2	CYX	86	8.591	-1.221	-8.283	1.00	0.00
ATOM	1334	HB3	CYX	86	7.633	-1.740	-6.876	1.00	0.00
ATOM	1335	SG	CYX	86	9.939	-2.480	-6.776	1.00	0.00
ATOM	1336	C	CYX	86	8.840	-3.552	-9.539	1.00	0.00
ATOM	1337	O	CYX	86	8.953	-2.797	-10.484	1.00	0.00
ATOM	1338	N	ASP	87	9.542	-4.651	-9.462	1.00	0.00
ATOM	1339	H	ASP	87	9.455	-5.283	-8.679	1.00	0.00
ATOM	1340	CA	ASP	87	10.501	-5.000	-10.547	1.00	0.00
ATOM	1341	HA	ASP	87	11.389	-4.370	-10.485	1.00	0.00
ATOM	1342	CB	ASP	87	10.928	-6.462	-10.398	1.00	0.00

ATOM 1343 HB2 ASP 87 10.057 -7.074 -10.162 1.00 0.00
ATOM 1344 HB3 ASP 87 11.372 -6.808 -11.332 1.00 0.00
ATOM 1345 CG ASP 87 11.955 -6.582 -9.270 1.00 0.00
ATOM 1346 OD1 ASP 87 12.388 -5.553 -8.777 1.00 0.00
ATOM 1347 OD2 ASP 87 12.291 -7.700 -8.918 1.00 0.00
ATOM 1348 C ASP 87 9.829 -4.802 -11.907 1.00 0.00
ATOM 1349 O ASP 87 10.387 -4.202 -12.805 1.00 0.00
ATOM 1350 N ARG 88 8.635 -5.304 -12.068 1.00 0.00
ATOM 1351 H ARG 88 8.166 -5.805 -11.327 1.00 0.00
ATOM 1352 CA ARG 88 7.930 -5.146 -13.371 1.00 0.00
ATOM 1353 HA ARG 88 8.533 -5.560 -14.180 1.00 0.00
ATOM 1354 CB ARG 88 6.596 -5.895 -13.323 1.00 0.00
ATOM 1355 HB2 ARG 88 6.011 -5.542 -12.474 1.00 0.00
ATOM 1356 HB3 ARG 88 6.044 -5.714 -14.245 1.00 0.00
ATOM 1357 CG ARG 88 6.858 -7.395 -13.172 1.00 0.00
ATOM 1358 HG2 ARG 88 7.448 -7.748 -14.018 1.00 0.00
ATOM 1359 HG3 ARG 88 7.405 -7.577 -12.247 1.00 0.00
ATOM 1360 CD ARG 88 5.526 -8.145 -13.132 1.00 0.00
ATOM 1361 HD2 ARG 88 4.865 -7.700 -12.389 1.00 0.00
ATOM 1362 HD3 ARG 88 5.046 -8.113 -14.110 1.00 0.00
ATOM 1363 NE ARG 88 5.766 -9.568 -12.765 1.00 0.00
ATOM 1364 HE ARG 88 5.478 -9.939 -11.870 1.00 0.00

ATOM 1365 CZ ARG 88 6.360 -10.369 -13.609 1.00 0.00
ATOM 1366 NH1 ARG 88 5.653 -11.180 -14.346 1.00 0.00
ATOM 1367 HH11 ARG 88 4.647 -11.187 -14.262 1.00 0.00
ATOM 1368 HH12 ARG 88 6.115 -11.798 -14.998 1.00 0.00
ATOM 1369 NH2 ARG 88 7.661 -10.356 -13.715 1.00 0.00
ATOM 1370 HH21 ARG 88 8.205 -9.726 -13.143 1.00 0.00
ATOM 1371 HH22 ARG 88 8.118 -10.977 -14.368 1.00 0.00
ATOM 1372 C ARG 88 7.671 -3.661 -13.639 1.00 0.00
ATOM 1373 O ARG 88 7.315 -3.272 -14.733 1.00 0.00
ATOM 1374 N LEU 89 7.850 -2.830 -12.650 1.00 0.00
ATOM 1375 H LEU 89 8.146 -3.146 -11.737 1.00 0.00
ATOM 1376 CA LEU 89 7.615 -1.372 -12.852 1.00 0.00
ATOM 1377 HA LEU 89 6.588 -1.196 -13.170 1.00 0.00
ATOM 1378 CB LEU 89 7.855 -0.627 -11.537 1.00 0.00
ATOM 1379 HB2 LEU 89 7.368 -1.161 -10.721 1.00 0.00
ATOM 1380 HB3 LEU 89 8.926 -0.568 -11.344 1.00 0.00
ATOM 1381 CG LEU 89 7.277 0.785 -11.639 1.00 0.00
ATOM 1382 HG LEU 89 7.691 1.285 -12.515 1.00 0.00
ATOM 1383 CD1 LEU 89 5.755 0.704 -11.770 1.00 0.00
ATOM 1384 HD11 LEU 89 5.341 0.204 -10.895 1.00 0.00
ATOM 1385 HD12 LEU 89 5.342 1.710 -11.843 1.00 0.00
ATOM 1386 HD13 LEU 89 5.497 0.140 -12.666 1.00 0.00

ATOM 1387 CD2 LEU 89 7.636 1.578 -10.382 1.00 0.00
ATOM 1388 HD21 LEU 89 8.720 1.636 -10.286 1.00 0.00
ATOM 1389 HD22 LEU 89 7.224 2.584 -10.456 1.00 0.00
ATOM 1390 HD23 LEU 89 7.221 1.079 -9.506 1.00 0.00
ATOM 1391 C LEU 89 8.575 -0.843 -13.920 1.00 0.00
ATOM 1392 O LEU 89 8.392 0.232 -14.455 1.00 0.00
ATOM 1393 N GLN 90 9.598 -1.591 -14.233 1.00 0.00
ATOM 1394 H GLN 90 9.757 -2.487 -13.793 1.00 0.00
ATOM 1395 CA GLN 90 10.568 -1.130 -15.266 1.00 0.00
ATOM 1396 HA GLN 90 11.211 -0.349 -14.860 1.00 0.00
ATOM 1397 CB GLN 90 11.444 -2.306 -15.703 1.00 0.00
ATOM 1398 HB2 GLN 90 10.816 -3.175 -15.900 1.00 0.00
ATOM 1399 HB3 GLN 90 11.987 -2.039 -16.610 1.00 0.00
ATOM 1400 CG GLN 90 12.442 -2.640 -14.591 1.00 0.00
ATOM 1401 HG2 GLN 90 13.014 -1.753 -14.318 1.00 0.00
ATOM 1402 HG3 GLN 90 11.920 -3.020 -13.713 1.00 0.00
ATOM 1403 CD GLN 90 13.413 -3.714 -15.085 1.00 0.00
ATOM 1404 OE1 GLN 90 13.188 -4.328 -16.109 1.00 0.00
ATOM 1405 NE2 GLN 90 14.490 -3.970 -14.394 1.00 0.00
ATOM 1406 HE21 GLN 90 15.136 -4.678 -14.714 1.00 0.00
ATOM 1407 HE22 GLN 90 14.673 -3.460 -13.542 1.00 0.00
ATOM 1408 C GLN 90 9.806 -0.587 -16.475 1.00 0.00

ATOM	1409	O	GLN	90	10.304	0.234	-17.219	1.00	0.00
ATOM	1410	N	ASP	91	8.598	-1.039	-16.679	1.00	0.00
ATOM	1411	H	ASP	91	8.177	-1.721	-16.064	1.00	0.00
ATOM	1412	CA	ASP	91	7.804	-0.550	-17.840	1.00	0.00
ATOM	1413	HA	ASP	91	8.363	-0.688	-18.766	1.00	0.00
ATOM	1414	CB	ASP	91	6.499	-1.341	-17.935	1.00	0.00
ATOM	1415	HB2	ASP	91	5.966	-1.278	-16.986	1.00	0.00
ATOM	1416	HB3	ASP	91	5.878	-0.925	-18.728	1.00	0.00
ATOM	1417	CG	ASP	91	6.811	-2.805	-18.247	1.00	0.00
ATOM	1418	OD1	ASP	91	7.941	-3.084	-18.614	1.00	0.00
ATOM	1419	OD2	ASP	91	5.916	-3.624	-18.115	1.00	0.00
ATOM	1420	C	ASP	91	7.487	0.935	-17.651	1.00	0.00
ATOM	1421	O	ASP	91	7.217	1.389	-16.556	1.00	0.00
ATOM	1422	N	ARG	92	7.520	1.698	-18.710	1.00	0.00
ATOM	1423	H	ARG	92	7.746	1.329	-19.623	1.00	0.00
ATOM	1424	CA	ARG	92	7.221	3.153	-18.590	1.00	0.00
ATOM	1425	HA	ARG	92	7.797	3.590	-17.774	1.00	0.00
ATOM	1426	CB	ARG	92	7.600	3.862	-19.891	1.00	0.00
ATOM	1427	HB2	ARG	92	8.676	3.783	-20.047	1.00	0.00
ATOM	1428	HB3	ARG	92	7.078	3.393	-20.725	1.00	0.00
ATOM	1429	CG	ARG	92	7.204	5.336	-19.805	1.00	0.00
ATOM	1430	HG2	ARG	92	6.161	5.414	-19.499	1.00	0.00

```
ATOM   1431  HG3 ARG    92       7.836   5.840 -19.074  1.00  0.00
ATOM   1432  CD  ARG    92       7.384   5.994 -21.174  1.00  0.00
ATOM   1433  HD2 ARG    92       6.672   5.580 -21.888  1.00  0.00
ATOM   1434  HD3 ARG    92       7.236   7.071 -21.097  1.00  0.00
ATOM   1435  NE  ARG    92       8.764   5.736 -21.672  1.00  0.00
ATOM   1436  HE  ARG    92       9.362   5.047 -21.237  1.00  0.00
ATOM   1437  CZ  ARG    92       9.225   6.404 -22.693  1.00  0.00
ATOM   1438  NH1 ARG    92       8.960   7.676 -22.813  1.00  0.00
ATOM   1439 HH11 ARG    92       8.398   8.139 -22.114  1.00  0.00
ATOM   1440 HH12 ARG    92       9.319   8.190 -23.605  1.00  0.00
ATOM   1441  NH2 ARG    92       9.953   5.801 -23.593  1.00  0.00
ATOM   1442 HH21 ARG    92      10.158   4.816 -23.496  1.00  0.00
ATOM   1443 HH22 ARG    92      10.309   6.321 -24.382  1.00  0.00
ATOM   1444  C   ARG    92       5.727   3.346 -18.321  1.00  0.00
ATOM   1445  O   ARG    92       5.294   4.396 -17.888  1.00  0.00
ATOM   1446  N   GLN    93       4.934   2.342 -18.575  1.00  0.00
ATOM   1447  H   GLN    93       5.286   1.466 -18.936  1.00  0.00
ATOM   1448  CA  GLN    93       3.470   2.469 -18.335  1.00  0.00
ATOM   1449  HA  GLN    93       3.113   3.440 -18.675  1.00  0.00
ATOM   1450  CB  GLN    93       2.731   1.376 -19.113  1.00  0.00
ATOM   1451  HB2 GLN    93       3.091   1.355 -20.142  1.00  0.00
ATOM   1452  HB3 GLN    93       2.915   0.408 -18.645  1.00  0.00
```

ATOM	1453	CG	GLN	93	1.229	1.670	-19.104	1.00	0.00
ATOM	1454	HG2	GLN	93	0.831	1.580	-18.093	1.00	0.00
ATOM	1455	HG3	GLN	93	1.039	2.674	-19.481	1.00	0.00
ATOM	1456	CD	GLN	93	0.508	0.665	-20.005	1.00	0.00
ATOM	1457	OE1	GLN	93	1.137	-0.073	-20.737	1.00	0.00
ATOM	1458	NE2	GLN	93	-0.795	0.604	-19.981	1.00	0.00
ATOM	1459	HE21	GLN	93	-1.277	-0.057	-20.573	1.00	0.00
ATOM	1460	HE22	GLN	93	-1.315	1.218	-19.371	1.00	0.00
ATOM	1461	C	GLN	93	3.179	2.317	-16.840	1.00	0.00
ATOM	1462	O	GLN	93	2.503	3.133	-16.245	1.00	0.00
ATOM	1463	N	MET	94	3.683	1.280	-16.229	1.00	0.00
ATOM	1464	H	MET	94	4.248	0.599	-16.717	1.00	0.00
ATOM	1465	CA	MET	94	3.433	1.077	-14.774	1.00	0.00
ATOM	1466	HA	MET	94	2.368	0.933	-14.590	1.00	0.00
ATOM	1467	CB	MET	94	4.183	-0.166	-14.294	1.00	0.00
ATOM	1468	HB2	MET	94	5.212	-0.131	-14.654	1.00	0.00
ATOM	1469	HB3	MET	94	4.181	-0.193	-13.204	1.00	0.00
ATOM	1470	CG	MET	94	3.497	-1.421	-14.836	1.00	0.00
ATOM	1471	HG2	MET	94	3.177	-1.251	-15.864	1.00	0.00
ATOM	1472	HG3	MET	94	4.191	-2.262	-14.807	1.00	0.00
ATOM	1473	SD	MET	94	2.051	-1.807	-13.818	1.00	0.00
ATOM	1474	CE	MET	94	2.900	-1.866	-12.220	1.00	0.00

ATOM 1475 HE1 MET 94 3.364 -0.901 -12.018 1.00 0.00

ATOM 1476 HE2 MET 94 2.181 -2.095 -11.434 1.00 0.00

ATOM 1477 HE3 MET 94 3.668 -2.639 -12.244 1.00 0.00

ATOM 1478 C MET 94 3.926 2.298 -13.993 1.00 0.00

ATOM 1479 O MET 94 3.296 2.742 -13.055 1.00 0.00

ATOM 1480 N VAL 95 5.054 2.838 -14.369 1.00 0.00

ATOM 1481 H VAL 95 5.585 2.469 -15.145 1.00 0.00

ATOM 1482 CA VAL 95 5.593 4.026 -13.645 1.00 0.00

ATOM 1483 HA VAL 95 6.090 3.714 -12.726 1.00 0.00

ATOM 1484 CB VAL 95 6.611 4.745 -14.532 1.00 0.00

ATOM 1485 HB VAL 95 6.156 4.976 -15.495 1.00 0.00

ATOM 1486 CG1 VAL 95 7.053 6.042 -13.853 1.00 0.00

ATOM 1487 HG11 VAL 95 7.509 5.811 -12.890 1.00 0.00

ATOM 1488 HG12 VAL 95 7.779 6.554 -14.485 1.00 0.00

ATOM 1489 HG13 VAL 95 6.187 6.685 -13.699 1.00 0.00

ATOM 1490 CG2 VAL 95 7.828 3.842 -14.746 1.00 0.00

ATOM 1491 HG21 VAL 95 7.514 2.916 -15.230 1.00 0.00

ATOM 1492 HG22 VAL 95 8.554 4.353 -15.378 1.00 0.00

ATOM 1493 HG23 VAL 95 8.284 3.611 -13.783 1.00 0.00

ATOM 1494 C VAL 95 4.451 4.985 -13.298 1.00 0.00

ATOM 1495 O VAL 95 3.824 4.868 -12.264 1.00 0.00

ATOM 1496 N GLN 96 4.179 5.933 -14.151 1.00 0.00

ATOM	1497	H	GLN	96	4.698	6.039	-15.011	1.00	0.00
ATOM	1498	CA	GLN	96	3.080	6.898	-13.865	1.00	0.00
ATOM	1499	HA	GLN	96	3.319	7.492	-12.983	1.00	0.00
ATOM	1500	CB	GLN	96	2.905	7.841	-15.058	1.00	0.00
ATOM	1501	HB2	GLN	96	2.621	7.264	-15.938	1.00	0.00
ATOM	1502	HB3	GLN	96	2.126	8.569	-14.834	1.00	0.00
ATOM	1503	CG	GLN	96	4.222	8.570	-15.330	1.00	0.00
ATOM	1504	HG2	GLN	96	4.559	9.094	-14.435	1.00	0.00
ATOM	1505	HG3	GLN	96	4.988	7.860	-15.644	1.00	0.00
ATOM	1506	CD	GLN	96	4.016	9.594	-16.448	1.00	0.00
ATOM	1507	OE1	GLN	96	3.296	10.559	-16.280	1.00	0.00
ATOM	1508	NE2	GLN	96	4.624	9.426	-17.591	1.00	0.00
ATOM	1509	HE21	GLN	96	4.493	10.098	-18.333	1.00	0.00
ATOM	1510	HE22	GLN	96	5.223	8.624	-17.728	1.00	0.00
ATOM	1511	C	GLN	96	1.779	6.136	-13.621	1.00	0.00
ATOM	1512	O	GLN	96	1.139	6.287	-12.599	1.00	0.00
ATOM	1513	N	GLN	97	1.383	5.319	-14.553	1.00	0.00
ATOM	1514	H	GLN	97	1.910	5.194	-15.405	1.00	0.00
ATOM	1515	CA	GLN	97	0.123	4.543	-14.382	1.00	0.00
ATOM	1516	HA	GLN	97	-0.742	5.188	-14.532	1.00	0.00
ATOM	1517	CB	GLN	97	0.077	3.415	-15.415	1.00	0.00
ATOM	1518	HB2	GLN	97	0.403	3.794	-16.384	1.00	0.00

ATOM	1519	HB3	GLN	97	0.738	2.606	-15.102	1.00	0.00
ATOM	1520	CG	GLN	97	-1.355	2.888	-15.529	1.00	0.00
ATOM	1521	HG2	GLN	97	-1.391	2.029	-16.200	1.00	0.00
ATOM	1522	HG3	GLN	97	-1.729	2.597	-14.547	1.00	0.00
ATOM	1523	CD	GLN	97	-2.259	3.986	-16.091	1.00	0.00
ATOM	1524	OE1	GLN	97	-2.137	4.362	-17.240	1.00	0.00
ATOM	1525	NE2	GLN	97	-3.170	4.519	-15.323	1.00	0.00
ATOM	1526	HE21	GLN	97	-3.771	5.244	-15.687	1.00	0.00
ATOM	1527	HE22	GLN	97	-3.269	4.203	-14.369	1.00	0.00
ATOM	1528	C	GLN	97	0.072	3.951	-12.972	1.00	0.00
ATOM	1529	O	GLN	97	-0.726	4.353	-12.148	1.00	0.00
ATOM	1530	N	PHE	98	0.919	3.000	-12.686	1.00	0.00
ATOM	1531	H	PHE	98	1.586	2.654	-13.361	1.00	0.00
ATOM	1532	CA	PHE	98	0.911	2.391	-11.325	1.00	0.00
ATOM	1533	HA	PHE	98	-0.011	1.830	-11.173	1.00	0.00
ATOM	1534	CB	PHE	98	2.097	1.434	-11.169	1.00	0.00
ATOM	1535	HB2	PHE	98	2.144	0.704	-11.978	1.00	0.00
ATOM	1536	HB3	PHE	98	3.048	1.963	-11.103	1.00	0.00
ATOM	1537	CG	PHE	98	1.949	0.647	-9.884	1.00	0.00
ATOM	1538	CD1	PHE	98	0.749	0.695	-9.159	1.00	0.00
ATOM	1539	HD1	PHE	98	-0.081	1.300	-9.522	1.00	0.00
ATOM	1540	CE1	PHE	98	0.618	-0.033	-7.972	1.00	0.00

ATOM 1541 HE1 PHE 98 -0.316 0.005 -7.411 1.00 0.00

ATOM 1542 CZ PHE 98 1.683 -0.810 -7.504 1.00 0.00

ATOM 1543 HZ PHE 98 1.580 -1.377 -6.578 1.00 0.00

ATOM 1544 CE2 PHE 98 2.882 -0.860 -8.225 1.00 0.00

ATOM 1545 HE2 PHE 98 3.712 -1.466 -7.861 1.00 0.00

ATOM 1546 CD2 PHE 98 3.015 -0.132 -9.414 1.00 0.00

ATOM 1547 HD2 PHE 98 3.949 -0.171 -9.975 1.00 0.00

ATOM 1548 C PHE 98 1.010 3.500 -10.279 1.00 0.00

ATOM 1549 O PHE 98 0.380 3.448 -9.242 1.00 0.00

ATOM 1550 N LYS 99 1.795 4.506 -10.547 1.00 0.00

ATOM 1551 H LYS 99 2.321 4.554 -11.408 1.00 0.00

ATOM 1552 CA LYS 99 1.932 5.623 -9.572 1.00 0.00

ATOM 1553 HA LYS 99 2.226 5.237 -8.596 1.00 0.00

ATOM 1554 CB LYS 99 3.007 6.597 -10.056 1.00 0.00

ATOM 1555 HB2 LYS 99 3.920 6.047 -10.284 1.00 0.00

ATOM 1556 HB3 LYS 99 2.658 7.107 -10.954 1.00 0.00

ATOM 1557 CG LYS 99 3.292 7.628 -8.961 1.00 0.00

ATOM 1558 HG2 LYS 99 2.365 8.129 -8.682 1.00 0.00

ATOM 1559 HG3 LYS 99 3.711 7.126 -8.088 1.00 0.00

ATOM 1560 CD LYS 99 4.292 8.661 -9.484 1.00 0.00

ATOM 1561 HD2 LYS 99 5.239 8.170 -9.708 1.00 0.00

ATOM 1562 HD3 LYS 99 3.899 9.121 -10.390 1.00 0.00

ATOM 1563 CE LYS 99 4.515 9.738 -8.420 1.00 0.00
ATOM 1564 HE2 LYS 99 4.335 10.721 -8.854 1.00 0.00
ATOM 1565 HE3 LYS 99 3.828 9.576 -7.589 1.00 0.00
ATOM 1566 NZ LYS 99 5.918 9.664 -7.925 1.00 0.00
ATOM 1567 HZ1 LYS 99 6.555 9.814 -8.695 1.00 0.00
ATOM 1568 HZ2 LYS 99 6.065 10.374 -7.223 1.00 0.00
ATOM 1569 HZ3 LYS 99 6.086 8.753 -7.522 1.00 0.00
ATOM 1570 C LYS 99 0.594 6.355 -9.457 1.00 0.00
ATOM 1571 O LYS 99 0.230 6.845 -8.407 1.00 0.00
ATOM 1572 N ARG 100 -0.143 6.426 -10.530 1.00 0.00
ATOM 1573 H ARG 100 0.155 6.020 -11.406 1.00 0.00
ATOM 1574 CA ARG 100 -1.461 7.118 -10.484 1.00 0.00
ATOM 1575 HA ARG 100 -1.349 8.114 -10.056 1.00 0.00
ATOM 1576 CB ARG 100 -2.019 7.255 -11.902 1.00 0.00
ATOM 1577 HB2 ARG 100 -1.837 6.334 -12.456 1.00 0.00
ATOM 1578 HB3 ARG 100 -3.091 7.444 -11.855 1.00 0.00
ATOM 1579 CG ARG 100 -1.326 8.421 -12.610 1.00 0.00
ATOM 1580 HG2 ARG 100 -1.473 9.334 -12.034 1.00 0.00
ATOM 1581 HG3 ARG 100 -0.259 8.212 -12.694 1.00 0.00
ATOM 1582 CD ARG 100 -1.923 8.598 -14.008 1.00 0.00
ATOM 1583 HD2 ARG 100 -2.016 7.631 -14.504 1.00 0.00
ATOM 1584 HD3 ARG 100 -2.904 9.068 -13.943 1.00 0.00

ATOM	1585	NE	ARG	100	-1.029	9.469	-14.823	1.00	0.00
ATOM	1586	HE	ARG	100	-0.123	9.150	-15.136	1.00	0.00
ATOM	1587	CZ	ARG	100	-1.411	10.673	-15.148	1.00	0.00
ATOM	1588	NH1	ARG	100	-0.785	11.710	-14.662	1.00	0.00
ATOM	1589	HH11	ARG	100	-0.005	11.576	-14.034	1.00	0.00
ATOM	1590	HH12	ARG	100	-1.083	12.640	-14.916	1.00	0.00
ATOM	1591	NH2	ARG	100	-2.419	10.841	-15.959	1.00	0.00
ATOM	1592	HH21	ARG	100	-2.901	10.036	-16.334	1.00	0.00
ATOM	1593	HH22	ARG	100	-2.712	11.774	-16.209	1.00	0.00
ATOM	1594	C	ARG	100	-2.425	6.296	-9.630	1.00	0.00
ATOM	1595	O	ARG	100	-3.026	6.795	-8.699	1.00	0.00
ATOM	1596	N	GLU	101	-2.569	5.033	-9.929	1.00	0.00
ATOM	1597	H	GLU	101	-2.070	4.607	-10.697	1.00	0.00
ATOM	1598	CA	GLU	101	-3.484	4.179	-9.123	1.00	0.00
ATOM	1599	HA	GLU	101	-4.498	4.575	-9.158	1.00	0.00
ATOM	1600	CB	GLU	101	-3.494	2.759	-9.692	1.00	0.00
ATOM	1601	HB2	GLU	101	-2.473	2.379	-9.739	1.00	0.00
ATOM	1602	HB3	GLU	101	-4.091	2.114	-9.048	1.00	0.00
ATOM	1603	CG	GLU	101	-4.097	2.774	-11.098	1.00	0.00
ATOM	1604	HG2	GLU	101	-3.426	3.302	-11.775	1.00	0.00
ATOM	1605	HG3	GLU	101	-4.233	1.750	-11.448	1.00	0.00
ATOM	1606	CD	GLU	101	-5.451	3.486	-11.064	1.00	0.00

ATOM 1607 OE1 GLU 101 -6.161 3.322 -10.086 1.00 0.00

ATOM 1608 OE2 GLU 101 -5.756 4.183 -12.019 1.00 0.00

ATOM 1609 C GLU 101 -2.982 4.148 -7.681 1.00 0.00

ATOM 1610 O GLU 101 -3.751 4.164 -6.740 1.00 0.00

ATOM 1611 N LEU 102 -1.689 4.105 -7.502 1.00 0.00

ATOM 1612 H LEU 102 -1.047 4.089 -8.282 1.00 0.00

ATOM 1613 CA LEU 102 -1.126 4.076 -6.125 1.00 0.00

ATOM 1614 HA LEU 102 -1.429 3.164 -5.611 1.00 0.00

ATOM 1615 CB LEU 102 0.402 4.110 -6.200 1.00 0.00

ATOM 1616 HB2 LEU 102 0.715 4.052 -7.243 1.00 0.00

ATOM 1617 HB3 LEU 102 0.765 5.040 -5.764 1.00 0.00

ATOM 1618 CG LEU 102 0.984 2.923 -5.426 1.00 0.00

ATOM 1619 HG LEU 102 0.901 3.112 -4.356 1.00 0.00

ATOM 1620 CD1 LEU 102 0.213 1.649 -5.780 1.00 0.00

ATOM 1621 HD11 LEU 102 0.297 1.458 -6.850 1.00 0.00

ATOM 1622 HD12 LEU 102 0.630 0.806 -5.228 1.00 0.00

ATOM 1623 HD13 LEU 102 -0.837 1.773 -5.515 1.00 0.00

ATOM 1624 CD2 LEU 102 2.457 2.746 -5.800 1.00 0.00

ATOM 1625 HD21 LEU 102 3.008 3.651 -5.546 1.00 0.00

ATOM 1626 HD22 LEU 102 2.873 1.901 -5.250 1.00 0.00

ATOM 1627 HD23 LEU 102 2.541 2.558 -6.871 1.00 0.00

ATOM 1628 C LEU 102 -1.627 5.291 -5.343 1.00 0.00

ATOM	1629	O	LEU	102	-1.916	5.207	-4.168	1.00	0.00
ATOM	1630	N	MET	103	-1.739	6.421	-5.988	1.00	0.00
ATOM	1631	H	MET	103	-1.505	6.502	-6.967	1.00	0.00
ATOM	1632	CA	MET	103	-2.228	7.634	-5.275	1.00	0.00
ATOM	1633	HA	MET	103	-1.595	7.846	-4.413	1.00	0.00
ATOM	1634	CB	MET	103	-2.186	8.836	-6.220	1.00	0.00
ATOM	1635	HB2	MET	103	-2.638	8.565	-7.174	1.00	0.00
ATOM	1636	HB3	MET	103	-2.738	9.665	-5.779	1.00	0.00
ATOM	1637	CG	MET	103	-0.731	9.255	-6.446	1.00	0.00
ATOM	1638	HG2	MET	103	-0.252	9.456	-5.488	1.00	0.00
ATOM	1639	HG3	MET	103	-0.193	8.458	-6.960	1.00	0.00
ATOM	1640	SD	MET	103	-0.686	10.754	-7.460	1.00	0.00
ATOM	1641	CE	MET	103	-0.945	11.942	-6.121	1.00	0.00
ATOM	1642	HE1	MET	103	-0.139	11.850	-5.392	1.00	0.00
ATOM	1643	HE2	MET	103	-0.952	12.953	-6.528	1.00	0.00
ATOM	1644	HE3	MET	103	-1.899	11.739	-5.634	1.00	0.00
ATOM	1645	C	MET	103	-3.667	7.396	-4.815	1.00	0.00
ATOM	1646	O	MET	103	-4.096	7.899	-3.796	1.00	0.00
ATOM	1647	N	SER	104	-4.414	6.626	-5.559	1.00	0.00
ATOM	1648	H	SER	104	-4.061	6.204	-6.406	1.00	0.00
ATOM	1649	CA	SER	104	-5.823	6.349	-5.167	1.00	0.00
ATOM	1650	HA	SER	104	-6.287	7.248	-4.764	1.00	0.00

ATOM	1651	CB	SER	104	-6.615	5.899	-6.396	1.00	0.00
ATOM	1652	HB2	SER	104	-7.656	5.736	-6.119	1.00	0.00
ATOM	1653	HB3	SER	104	-6.560	6.668	-7.166	1.00	0.00
ATOM	1654	OG	SER	104	-6.064	4.689	-6.896	1.00	0.00
ATOM	1655	HG	SER	104	-6.560	4.406	-7.667	1.00	0.00
ATOM	1656	C	SER	104	-5.847	5.242	-4.111	1.00	0.00
ATOM	1657	O	SER	104	-6.705	5.208	-3.252	1.00	0.00
ATOM	1658	N	LEU	105	-4.913	4.332	-4.172	1.00	0.00
ATOM	1659	H	LEU	105	-4.202	4.352	-4.889	1.00	0.00
ATOM	1660	CA	LEU	105	-4.881	3.224	-3.177	1.00	0.00
ATOM	1661	HA	LEU	105	-5.629	2.471	-3.426	1.00	0.00
ATOM	1662	CB	LEU	105	-3.500	2.563	-3.189	1.00	0.00
ATOM	1663	HB2	LEU	105	-2.838	3.127	-3.846	1.00	0.00
ATOM	1664	HB3	LEU	105	-3.090	2.557	-2.179	1.00	0.00
ATOM	1665	CG	LEU	105	-3.620	1.124	-3.699	1.00	0.00
ATOM	1666	HG	LEU	105	-2.682	0.597	-3.522	1.00	0.00
ATOM	1667	CD1	LEU	105	-4.754	0.408	-2.961	1.00	0.00
ATOM	1668	HD11	LEU	105	-5.692	0.933	-3.139	1.00	0.00
ATOM	1669	HD12	LEU	105	-4.836	-0.616	-3.326	1.00	0.00
ATOM	1670	HD13	LEU	105	-4.541	0.395	-1.892	1.00	0.00
ATOM	1671	CD2	LEU	105	-3.916	1.138	-5.199	1.00	0.00
ATOM	1672	HD21	LEU	105	-3.106	1.643	-5.725	1.00	0.00

ATOM	1673	HD22	LEU	105	-4.001	0.114	-5.563	1.00	0.00
ATOM	1674	HD23	LEU	105	-4.852	1.666	-5.380	1.00	0.00
ATOM	1675	C	LEU	105	-5.173	3.769	-1.777	1.00	0.00
ATOM	1676	O	LEU	105	-6.016	3.254	-1.070	1.00	0.00
ATOM	1677	N	PRO	106	-4.465	4.835	-1.366	1.00	0.00
ATOM	1678	CD	PRO	106	-3.429	5.533	-2.148	1.00	0.00
ATOM	1679	HD2	PRO	106	-3.779	5.686	-3.169	1.00	0.00
ATOM	1680	HD3	PRO	106	-2.516	4.937	-2.164	1.00	0.00
ATOM	1681	CG	PRO	106	-3.258	6.823	-1.396	1.00	0.00
ATOM	1682	HG2	PRO	106	-3.945	7.571	-1.792	1.00	0.00
ATOM	1683	HG3	PRO	106	-2.233	7.177	-1.506	1.00	0.00
ATOM	1684	CB	PRO	106	-3.561	6.505	0.027	1.00	0.00
ATOM	1685	HB2	PRO	106	-3.947	7.407	0.501	1.00	0.00
ATOM	1686	HB3	PRO	106	-2.716	6.125	0.603	1.00	0.00
ATOM	1687	CA	PRO	106	-4.660	5.445	-0.047	1.00	0.00
ATOM	1688	HA	PRO	106	-4.562	4.670	0.713	1.00	0.00
ATOM	1689	C	PRO	106	-6.039	6.094	0.063	1.00	0.00
ATOM	1690	O	PRO	106	-6.444	6.553	1.113	1.00	0.00
ATOM	1691	N	GLN	107	-6.768	6.124	-1.018	1.00	0.00
ATOM	1692	H	GLN	107	-6.431	5.742	-1.891	1.00	0.00
ATOM	1693	CA	GLN	107	-8.126	6.728	-0.990	1.00	0.00
ATOM	1694	HA	GLN	107	-8.226	7.390	-0.130	1.00	0.00

ATOM	1695	CB	GLN	107	-8.352	7.544	-2.264	1.00	0.00
ATOM	1696	HB2	GLN	107	-7.569	8.296	-2.358	1.00	0.00
ATOM	1697	HB3	GLN	107	-8.324	6.882	-3.130	1.00	0.00
ATOM	1698	CG	GLN	107	-9.716	8.234	-2.193	1.00	0.00
ATOM	1699	HG2	GLN	107	-10.508	7.493	-2.083	1.00	0.00
ATOM	1700	HG3	GLN	107	-9.747	8.925	-1.351	1.00	0.00
ATOM	1701	CD	GLN	107	-9.958	9.021	-3.482	1.00	0.00
ATOM	1702	OE1	GLN	107	-9.071	9.153	-4.303	1.00	0.00
ATOM	1703	NE2	GLN	107	-11.129	9.555	-3.697	1.00	0.00
ATOM	1704	HE21	GLN	107	-11.295	10.075	-4.546	1.00	0.00
ATOM	1705	HE22	GLN	107	-11.864	9.445	-3.013	1.00	0.00
ATOM	1706	C	GLN	107	-9.166	5.613	-0.904	1.00	0.00
ATOM	1707	O	GLN	107	-10.069	5.652	-0.093	1.00	0.00
ATOM	1708	N	GLN	108	-9.048	4.618	-1.740	1.00	0.00
ATOM	1709	H	GLN	108	-8.301	4.581	-2.419	1.00	0.00
ATOM	1710	CA	GLN	108	-10.030	3.498	-1.709	1.00	0.00
ATOM	1711	HA	GLN	108	-10.995	3.829	-2.092	1.00	0.00
ATOM	1712	CB	GLN	108	-9.523	2.352	-2.587	1.00	0.00
ATOM	1713	HB2	GLN	108	-9.323	2.724	-3.592	1.00	0.00
ATOM	1714	HB3	GLN	108	-8.605	1.946	-2.161	1.00	0.00
ATOM	1715	CG	GLN	108	-10.584	1.252	-2.653	1.00	0.00
ATOM	1716	HG2	GLN	108	-10.864	0.933	-1.649	1.00	0.00

ATOM	1717	HG3	GLN	108	-11.468	1.611	-3.180	1.00	0.00
ATOM	1718	CD	GLN	108	-10.020	0.047	-3.409	1.00	0.00
ATOM	1719	OE1	GLN	108	-9.111	-0.608	-2.941	1.00	0.00
ATOM	1720	NE2	GLN	108	-10.527	-0.276	-4.568	1.00	0.00
ATOM	1721	HE21	GLN	108	-10.160	-1.071	-5.073	1.00	0.00
ATOM	1722	HE22	GLN	108	-11.284	0.269	-4.954	1.00	0.00
ATOM	1723	C	GLN	108	-10.197	3.004	-0.272	1.00	0.00
ATOM	1724	O	GLN	108	-11.241	2.513	0.110	1.00	0.00
ATOM	1725	N	CYX	109	-9.176	3.130	0.530	1.00	0.00
ATOM	1726	H	CYX	109	-8.304	3.535	0.220	1.00	0.00
ATOM	1727	CA	CYX	109	-9.276	2.667	1.943	1.00	0.00
ATOM	1728	HA	CYX	109	-10.319	2.503	2.213	1.00	0.00
ATOM	1729	CB	CYX	109	-8.515	1.347	2.109	1.00	0.00
ATOM	1730	HB2	CYX	109	-8.067	1.307	3.102	1.00	0.00
ATOM	1731	HB3	CYX	109	-9.204	0.511	1.988	1.00	0.00
ATOM	1732	SG	CYX	109	-7.173	1.254	0.895	1.00	0.00
ATOM	1733	C	CYX	109	-8.671	3.726	2.869	1.00	0.00
ATOM	1734	O	CYX	109	-8.201	3.423	3.948	1.00	0.00
ATOM	1735	N	ASN	110	-8.683	4.963	2.456	1.00	0.00
ATOM	1736	H	ASN	110	-9.073	5.218	1.560	1.00	0.00
ATOM	1737	CA	ASN	110	-8.112	6.042	3.311	1.00	0.00
ATOM	1738	HA	ASN	110	-7.924	6.936	2.716	1.00	0.00

ATOM	1739	CB	ASN	110	-9.105	6.393	4.421	1.00	0.00
ATOM	1740	HB2	ASN	110	-9.201	5.560	5.118	1.00	0.00
ATOM	1741	HB3	ASN	110	-8.772	7.280	4.959	1.00	0.00
ATOM	1742	CG	ASN	110	-10.477	6.679	3.808	1.00	0.00
ATOM	1743	OD1	ASN	110	-10.583	7.404	2.839	1.00	0.00
ATOM	1744	ND2	ASN	110	-11.540	6.136	4.336	1.00	0.00
ATOM	1745	HD21	ASN	110	-12.449	6.320	3.936	1.00	0.00
ATOM	1746	HD22	ASN	110	-11.448	5.533	5.142	1.00	0.00
ATOM	1747	C	ASN	110	-6.801	5.562	3.935	1.00	0.00
ATOM	1748	O	ASN	110	-6.561	5.740	5.113	1.00	0.00
ATOM	1749	N	PHE	111	-5.948	4.953	3.155	1.00	0.00
ATOM	1750	H	PHE	111	-6.138	4.796	2.175	1.00	0.00
ATOM	1751	CA	PHE	111	-4.654	4.466	3.709	1.00	0.00
ATOM	1752	HA	PHE	111	-4.824	3.899	4.624	1.00	0.00
ATOM	1753	CB	PHE	111	-3.973	3.553	2.688	1.00	0.00
ATOM	1754	HB2	PHE	111	-4.684	3.116	1.987	1.00	0.00
ATOM	1755	HB3	PHE	111	-3.188	4.068	2.135	1.00	0.00
ATOM	1756	CG	PHE	111	-3.311	2.401	3.406	1.00	0.00
ATOM	1757	CD1	PHE	111	-4.066	1.281	3.776	1.00	0.00
ATOM	1758	HD1	PHE	111	-5.130	1.239	3.545	1.00	0.00
ATOM	1759	CE1	PHE	111	-3.452	0.214	4.443	1.00	0.00
ATOM	1760	HE1	PHE	111	-4.040	-0.657	4.733	1.00	0.00

ATOM 1761 CZ PHE 111 -2.084 0.265 4.738 1.00 0.00
ATOM 1762 HZ PHE 111 -1.606 -0.567 5.256 1.00 0.00
ATOM 1763 CE2 PHE 111 -1.330 1.385 4.367 1.00 0.00
ATOM 1764 HE2 PHE 111 -0.265 1.426 4.597 1.00 0.00
ATOM 1765 CD2 PHE 111 -1.944 2.452 3.701 1.00 0.00
ATOM 1766 HD2 PHE 111 -1.357 3.323 3.412 1.00 0.00
ATOM 1767 C PHE 111 -3.747 5.659 4.013 1.00 0.00
ATOM 1768 O PHE 111 -2.661 5.508 4.535 1.00 0.00
ATOM 1769 N ARG 112 -4.184 6.847 3.692 1.00 0.00
ATOM 1770 H ARG 112 -5.086 6.982 3.259 1.00 0.00
ATOM 1771 CA ARG 112 -3.344 8.047 3.966 1.00 0.00
ATOM 1772 HA ARG 112 -2.493 8.076 3.285 1.00 0.00
ATOM 1773 CB ARG 112 -4.179 9.313 3.760 1.00 0.00
ATOM 1774 HB2 ARG 112 -5.064 9.272 4.395 1.00 0.00
ATOM 1775 HB3 ARG 112 -3.584 10.187 4.023 1.00 0.00
ATOM 1776 CG ARG 112 -4.607 9.409 2.293 1.00 0.00
ATOM 1777 HG2 ARG 112 -3.725 9.376 1.653 1.00 0.00
ATOM 1778 HG3 ARG 112 -5.263 8.573 2.049 1.00 0.00
ATOM 1779 CD ARG 112 -5.353 10.725 2.066 1.00 0.00
ATOM 1780 HD2 ARG 112 -4.704 11.570 2.295 1.00 0.00
ATOM 1781 HD3 ARG 112 -5.688 10.795 1.031 1.00 0.00
ATOM 1782 NE ARG 112 -6.544 10.781 2.959 1.00 0.00

ATOM 1783 HE ARG 112 -7.242 10.050 2.954 1.00 0.00
ATOM 1784 CZ ARG 112 -6.704 11.790 3.772 1.00 0.00
ATOM 1785 NH1 ARG 112 -6.255 11.718 4.995 1.00 0.00
ATOM 1786 HH11 ARG 112 -5.784 10.881 5.309 1.00 0.00
ATOM 1787 HH12 ARG 112 -6.380 12.499 5.622 1.00 0.00
ATOM 1788 NH2 ARG 112 -7.312 12.868 3.362 1.00 0.00
ATOM 1789 HH21 ARG 112 -7.658 12.919 2.415 1.00 0.00
ATOM 1790 HH22 ARG 112 -7.434 13.646 3.994 1.00 0.00
ATOM 1791 C ARG 112 -2.845 7.996 5.411 1.00 0.00
ATOM 1792 O ARG 112 -3.458 7.393 6.269 1.00 0.00
ATOM 1793 N ALA 113 -1.735 8.626 5.689 1.00 0.00
ATOM 1794 H ALA 113 -1.218 9.130 4.983 1.00 0.00
ATOM 1795 CA ALA 113 -1.200 8.611 7.079 1.00 0.00
ATOM 1796 HA ALA 113 -1.967 8.343 7.806 1.00 0.00
ATOM 1797 CB ALA 113 -0.060 7.596 7.176 1.00 0.00
ATOM 1798 HB1 ALA 113 0.720 7.855 6.460 1.00 0.00
ATOM 1799 HB2 ALA 113 0.354 7.608 8.184 1.00 0.00
ATOM 1800 HB3 ALA 113 -0.440 6.599 6.952 1.00 0.00
ATOM 1801 C ALA 113 -0.676 10.002 7.440 1.00 0.00
ATOM 1802 O ALA 113 -0.456 10.834 6.582 1.00 0.00
ATOM 1803 N PRO 114 -0.473 10.253 8.742 1.00 0.00
ATOM 1804 CD PRO 114 -0.716 9.299 9.837 1.00 0.00

ATOM	1805	HD2	PRO	114	-0.252	8.342	9.595	1.00	0.00
ATOM	1806	HD3	PRO	114	-1.789	9.159	9.965	1.00	0.00
ATOM	1807	CG	PRO	114	-0.079	9.965	11.023	1.00	0.00
ATOM	1808	HG2	PRO	114	0.945	9.607	11.132	1.00	0.00
ATOM	1809	HG3	PRO	114	-0.646	9.722	11.922	1.00	0.00
ATOM	1810	CB	PRO	114	-0.117	11.431	10.748	1.00	0.00
ATOM	1811	HB2	PRO	114	0.741	11.892	11.237	1.00	0.00
ATOM	1812	HB3	PRO	114	-1.030	11.929	11.072	1.00	0.00
ATOM	1813	CA	PRO	114	0.028	11.543	9.231	1.00	0.00
ATOM	1814	HA	PRO	114	-0.576	12.335	8.790	1.00	0.00
ATOM	1815	C	PRO	114	1.498	11.757	8.861	1.00	0.00
ATOM	1816	O	PRO	114	2.028	12.843	8.984	1.00	0.00
ATOM	1817	N	GLN	115	2.160	10.728	8.406	1.00	0.00
ATOM	1818	H	GLN	115	1.725	9.822	8.301	1.00	0.00
ATOM	1819	CA	GLN	115	3.593	10.871	8.026	1.00	0.00
ATOM	1820	HA	GLN	115	4.008	11.785	8.450	1.00	0.00
ATOM	1821	CB	GLN	115	4.385	9.678	8.566	1.00	0.00
ATOM	1822	HB2	GLN	115	4.023	8.760	8.102	1.00	0.00
ATOM	1823	HB3	GLN	115	5.442	9.808	8.334	1.00	0.00
ATOM	1824	CG	GLN	115	4.203	9.588	10.083	1.00	0.00
ATOM	1825	HG2	GLN	115	3.155	9.416	10.328	1.00	0.00
ATOM	1826	HG3	GLN	115	4.808	8.778	10.491	1.00	0.00

ATOM	1827	CD	GLN	115	4.647	10.902	10.728	1.00	0.00
ATOM	1828	OE1	GLN	115	5.742	11.371	10.485	1.00	0.00
ATOM	1829	NE2	GLN	115	3.840	11.519	11.546	1.00	0.00
ATOM	1830	HE21	GLN	115	4.128	12.387	11.975	1.00	0.00
ATOM	1831	HE22	GLN	115	2.932	11.125	11.746	1.00	0.00
ATOM	1832	C	GLN	115	3.714	10.916	6.502	1.00	0.00
ATOM	1833	O	GLN	115	2.902	10.359	5.788	1.00	0.00
ATOM	1834	N	ARG	116	4.720	11.573	5.996	1.00	0.00
ATOM	1835	H	ARG	116	5.398	12.038	6.582	1.00	0.00
ATOM	1836	CA	ARG	116	4.891	11.652	4.518	1.00	0.00
ATOM	1837	HA	ARG	116	4.065	11.149	4.014	1.00	0.00
ATOM	1838	CB	ARG	116	4.909	13.119	4.084	1.00	0.00
ATOM	1839	HB2	ARG	116	5.717	13.640	4.597	1.00	0.00
ATOM	1840	HB3	ARG	116	5.066	13.178	3.007	1.00	0.00
ATOM	1841	CG	ARG	116	3.572	13.772	4.441	1.00	0.00
ATOM	1842	HG2	ARG	116	2.761	13.227	3.958	1.00	0.00
ATOM	1843	HG3	ARG	116	3.433	13.747	5.522	1.00	0.00
ATOM	1844	CD	ARG	116	3.569	15.224	3.959	1.00	0.00
ATOM	1845	HD2	ARG	116	4.483	15.728	4.273	1.00	0.00
ATOM	1846	HD3	ARG	116	3.490	15.261	2.872	1.00	0.00
ATOM	1847	NE	ARG	116	2.402	15.940	4.548	1.00	0.00
ATOM	1848	HE	ARG	116	2.496	16.510	5.376	1.00	0.00

ATOM 1849 CZ ARG 116 1.227 15.840 3.991 1.00 0.00

ATOM 1850 NH1 ARG 116 1.010 14.928 3.083 1.00 0.00

ATOM 1851 HH11 ARG 116 1.755 14.300 2.814 1.00 0.00

ATOM 1852 HH12 ARG 116 0.099 14.853 2.654 1.00 0.00

ATOM 1853 NH2 ARG 116 0.266 16.650 4.344 1.00 0.00

ATOM 1854 HH21 ARG 116 0.436 17.353 5.049 1.00 0.00

ATOM 1855 HH22 ARG 116 -0.642 16.569 3.911 1.00 0.00

ATOM 1856 C ARG 116 6.209 10.986 4.119 1.00 0.00

ATOM 1857 O ARG 116 7.154 10.954 4.881 1.00 0.00

ATOM 1858 N CYX 117 6.279 10.451 2.931 1.00 0.00

ATOM 1859 H CYX 117 5.497 10.473 2.292 1.00 0.00

ATOM 1860 CA CYX 117 7.535 9.787 2.486 1.00 0.00

ATOM 1861 HA CYX 117 8.266 9.779 3.295 1.00 0.00

ATOM 1862 CB CYX 117 7.234 8.341 2.085 1.00 0.00

ATOM 1863 HB2 CYX 117 6.461 8.329 1.317 1.00 0.00

ATOM 1864 HB3 CYX 117 8.139 7.875 1.695 1.00 0.00

ATOM 1865 SG CYX 117 6.604 7.436 3.520 1.00 0.00

ATOM 1866 C CYX 117 8.111 10.538 1.284 1.00 0.00

ATOM 1867 O CYX 117 7.414 11.256 0.596 1.00 0.00

ATOM 1868 N ASP 118 9.381 10.377 1.025 1.00 0.00

ATOM 1869 H ASP 118 9.968 9.782 1.592 1.00 0.00

ATOM 1870 CA ASP 118 9.999 11.082 -0.133 1.00 0.00

ATOM	1871	HA	ASP	118	9.706	12.131	-0.137	1.00	0.00
ATOM	1872	CB	ASP	118	11.523	11.001	-0.025	1.00	0.00
ATOM	1873	HB2	ASP	118	11.834	9.956	-0.013	1.00	0.00
ATOM	1874	HB3	ASP	118	11.974	11.503	-0.881	1.00	0.00
ATOM	1875	CG	ASP	118	11.980	11.683	1.266	1.00	0.00
ATOM	1876	OD1	ASP	118	11.186	12.408	1.843	1.00	0.00
ATOM	1877	OD2	ASP	118	13.116	11.467	1.657	1.00	0.00
ATOM	1878	C	ASP	118	9.543	10.420	-1.435	1.00	0.00
ATOM	1879	O	ASP	118	9.330	9.226	-1.493	1.00	0.00
ATOM	1880	N	LEU	119	9.389	11.188	-2.479	1.00	0.00
ATOM	1881	H	LEU	119	9.564	12.181	-2.438	1.00	0.00
ATOM	1882	CA	LEU	119	8.944	10.602	-3.774	1.00	0.00
ATOM	1883	HA	LEU	119	8.132	9.894	-3.610	1.00	0.00
ATOM	1884	CB	LEU	119	8.442	11.719	-4.691	1.00	0.00
ATOM	1885	HB2	LEU	119	9.143	12.553	-4.664	1.00	0.00
ATOM	1886	HB3	LEU	119	8.361	11.345	-5.712	1.00	0.00
ATOM	1887	CG	LEU	119	7.068	12.192	-4.211	1.00	0.00
ATOM	1888	HG	LEU	119	6.461	11.328	-3.939	1.00	0.00
ATOM	1889	CD1	LEU	119	7.239	13.100	-2.992	1.00	0.00
ATOM	1890	HD11	LEU	119	7.846	13.963	-3.264	1.00	0.00
ATOM	1891	HD12	LEU	119	6.261	13.437	-2.650	1.00	0.00
ATOM	1892	HD13	LEU	119	7.733	12.547	-2.193	1.00	0.00

ATOM	1893	CD2	LEU	119	6.373	12.967	-5.332	1.00	0.00
ATOM	1894	HD21	LEU	119	6.251	12.319	-6.200	1.00	0.00
ATOM	1895	HD22	LEU	119	5.395	13.303	-4.989	1.00	0.00
ATOM	1896	HD23	LEU	119	6.978	13.831	-5.606	1.00	0.00
ATOM	1897	C	LEU	119	10.118	9.883	-4.442	1.00	0.00
ATOM	1898	O	LEU	119	9.937	9.037	-5.294	1.00	0.00
ATOM	1899	N	ASP	120	11.323	10.211	-4.060	1.00	0.00
ATOM	1900	H	ASP	120	11.484	10.914	-3.353	1.00	0.00
ATOM	1901	CA	ASP	120	12.505	9.543	-4.674	1.00	0.00
ATOM	1902	HA	ASP	120	12.449	9.600	-5.761	1.00	0.00
ATOM	1903	CB	ASP	120	13.785	10.243	-4.213	1.00	0.00
ATOM	1904	HB2	ASP	120	14.648	9.771	-4.683	1.00	0.00
ATOM	1905	HB3	ASP	120	13.747	11.294	-4.497	1.00	0.00
ATOM	1906	CG	ASP	120	13.907	10.130	-2.692	1.00	0.00
ATOM	1907	OD1	ASP	120	12.897	9.886	-2.052	1.00	0.00
ATOM	1908	OD2	ASP	120	15.009	10.289	-2.193	1.00	0.00
ATOM	1909	C	ASP	120	12.542	8.075	-4.245	1.00	0.00
ATOM	1910	O	ASP	120	13.001	7.217	-4.972	1.00	0.00
ATOM	1911	N	VAL	121	12.064	7.780	-3.067	1.00	0.00
ATOM	1912	H	VAL	121	11.682	8.487	-2.457	1.00	0.00
ATOM	1913	CA	VAL	121	12.073	6.367	-2.592	1.00	0.00
ATOM	1914	HA	VAL	121	13.098	6.014	-2.472	1.00	0.00

ATOM	1915	CB	VAL	121	11.365	6.281	-1.238	1.00	0.00
ATOM	1916	HB	VAL	121	10.404	6.791	-1.297	1.00	0.00
ATOM	1917	CG1	VAL	121	11.140	4.812	-0.872	1.00	0.00
ATOM	1918	HG11	VAL	121	12.101	4.300	-0.812	1.00	0.00
ATOM	1919	HG12	VAL	121	10.636	4.750	0.092	1.00	0.00
ATOM	1920	HG13	VAL	121	10.523	4.337	-1.635	1.00	0.00
ATOM	1921	CG2	VAL	121	12.232	6.946	-0.167	1.00	0.00
ATOM	1922	HG21	VAL	121	12.393	7.992	-0.427	1.00	0.00
ATOM	1923	HG22	VAL	121	11.729	6.885	0.798	1.00	0.00
ATOM	1924	HG23	VAL	121	13.193	6.434	-0.108	1.00	0.00
ATOM	1925	C	VAL	121	11.343	5.483	-3.606	1.00	0.00
ATOM	1926	O	VAL	121	11.816	4.428	-3.978	1.00	0.00
ATOM	1927	N	SER	122	10.195	5.906	-4.057	1.00	0.00
ATOM	1928	H	SER	122	9.796	6.782	-3.752	1.00	0.00
ATOM	1929	CA	SER	122	9.436	5.090	-5.047	1.00	0.00
ATOM	1930	HA	SER	122	9.178	4.121	-4.619	1.00	0.00
ATOM	1931	CB	SER	122	8.144	5.817	-5.426	1.00	0.00
ATOM	1932	HB2	SER	122	8.387	6.748	-5.938	1.00	0.00
ATOM	1933	HB3	SER	122	7.551	5.184	-6.086	1.00	0.00
ATOM	1934	OG	SER	122	7.400	6.103	-4.251	1.00	0.00
ATOM	1935	HG	SER	122	6.590	6.559	-4.489	1.00	0.00
ATOM	1936	C	SER	122	10.290	4.882	-6.299	1.00	0.00

ATOM	1937	O	SER	122	10.238	3.848	-6.934	1.00	0.00
ATOM	1938	N	GLY	123	11.077	5.859	-6.661	1.00	0.00
ATOM	1939	H	GLY	123	11.126	6.722	-6.139	1.00	0.00
ATOM	1940	CA	GLY	123	11.933	5.716	-7.872	1.00	0.00
ATOM	1941	HA2	GLY	123	11.443	5.068	-8.599	1.00	0.00
ATOM	1942	HA3	GLY	123	12.111	6.694	-8.319	1.00	0.00
ATOM	1943	C	GLY	123	13.275	5.097	-7.479	1.00	0.00
ATOM	1944	O	GLY	123	14.220	5.100	-8.243	1.00	0.00
ATOM	1945	N	GLY	124	13.367	4.562	-6.291	1.00	0.00
ATOM	1946	H	GLY	124	12.587	4.554	-5.650	1.00	0.00
ATOM	1947	CA	GLY	124	14.648	3.942	-5.851	1.00	0.00
ATOM	1948	HA2	GLY	124	15.456	4.671	-5.915	1.00	0.00
ATOM	1949	HA3	GLY	124	14.557	3.589	-4.823	1.00	0.00
ATOM	1950	C	GLY	124	14.978	2.754	-6.757	1.00	0.00
ATOM	1951	O	GLY	124	14.989	2.867	-7.966	1.00	0.00
ATOM	1952	N	ARG	125	15.247	1.613	-6.181	1.00	0.00
ATOM	1953	H	ARG	125	15.240	1.509	-5.176	1.00	0.00
ATOM	1954	CA	ARG	125	15.574	0.420	-7.011	1.00	0.00
ATOM	1955	HA	ARG	125	16.448	0.618	-7.632	1.00	0.00
ATOM	1956	CB	ARG	125	15.884	-0.768	-6.096	1.00	0.00
ATOM	1957	HB2	ARG	125	15.059	-0.917	-5.400	1.00	0.00
ATOM	1958	HB3	ARG	125	16.017	-1.667	-6.698	1.00	0.00

ATOM	1959	CG	ARG	125	17.167	-0.485	-5.313	1.00	0.00
ATOM	1960	HG2	ARG	125	17.966	-0.222	-6.006	1.00	0.00
ATOM	1961	HG3	ARG	125	16.998	0.342	-4.624	1.00	0.00
ATOM	1962	CD	ARG	125	17.568	-1.733	-4.523	1.00	0.00
ATOM	1963	HD2	ARG	125	16.715	-2.118	-3.965	1.00	0.00
ATOM	1964	HD3	ARG	125	17.938	-2.505	-5.198	1.00	0.00
ATOM	1965	NE	ARG	125	18.650	-1.383	-3.560	1.00	0.00
ATOM	1966	HE	ARG	125	18.584	-0.571	-2.963	1.00	0.00
ATOM	1967	CZ	ARG	125	19.714	-2.133	-3.474	1.00	0.00
ATOM	1968	NH1	ARG	125	20.316	-2.539	-4.559	1.00	0.00
ATOM	1969	HH11	ARG	125	19.956	-2.271	-5.464	1.00	0.00
ATOM	1970	HH12	ARG	125	21.140	-3.120	-4.489	1.00	0.00
ATOM	1971	NH2	ARG	125	20.177	-2.478	-2.304	1.00	0.00
ATOM	1972	HH21	ARG	125	19.710	-2.163	-1.466	1.00	0.00
ATOM	1973	HH22	ARG	125	21.001	-3.059	-2.242	1.00	0.00
ATOM	1974	C	ARG	125	14.379	0.078	-7.903	1.00	0.00
ATOM	1975	O	ARG	125	14.535	-0.409	-9.007	1.00	0.00
ATOM	1976	N	CYX	126	13.188	0.329	-7.437	1.00	0.00
ATOM	1977	H	CYX	126	13.052	0.733	-6.522	1.00	0.00
ATOM	1978	CA	CYX	126	11.984	0.019	-8.259	1.00	0.00
ATOM	1979	HA	CYX	126	11.961	-1.041	-8.512	1.00	0.00
ATOM	1980	CB	CYX	126	10.723	0.359	-7.463	1.00	0.00

```
ATOM   1981  HB2 CYX   126      10.788   1.384  -7.100  1.00  0.00
ATOM   1982  HB3 CYX   126       9.849   0.255  -8.105  1.00  0.00
ATOM   1983  SG  CYX   126      10.607  -0.730  -6.023  1.00  0.00
ATOM   1984  C   CYX   126      12.018   0.851  -9.543  1.00  0.00
ATOM   1985  O   CYX   126      11.412   0.501 -10.537  1.00  0.00
ATOM   1986  N   SER   127      12.723   1.948  -9.531  1.00  0.00
ATOM   1987  H   SER   127      13.231   2.242  -8.709  1.00  0.00
ATOM   1988  CA  SER   127      12.797   2.801 -10.750  1.00  0.00
ATOM   1989  HA  SER   127      13.470   3.642 -10.583  1.00  0.00
ATOM   1990  CB  SER   127      13.331   1.973 -11.921  1.00  0.00
ATOM   1991  HB2 SER   127      14.411   2.100 -11.994  1.00  0.00
ATOM   1992  HB3 SER   127      13.101   0.920 -11.757  1.00  0.00
ATOM   1993  OG  SER   127      12.721   2.408 -13.127  1.00  0.00
ATOM   1994  HG  SER   127      13.057   1.888 -13.861  1.00  0.00
ATOM   1995  C   SER   127      11.399   3.323 -11.093  1.00  0.00
ATOM   1996  O   SER   127      10.450   2.861 -10.482  1.00  0.00
ATOM   1997  OXT SER   127      11.303   4.178 -11.958  1.00  0.00
TER
ATOM   1998  S1  LIG   128      27.707  -9.416 -10.752  1.00  0.00
ATOM   1999  O1  LIG   128      27.878 -10.790  -9.908  1.00  0.00
ATOM   2000  O2  LIG   128      29.059  -9.551 -11.624  1.00  0.00
ATOM   2001  H26 LIG   128      29.827  -9.347 -11.070  1.00  0.00
```

```
ATOM   2002  O3  LIG   128      26.609  -9.533 -11.680  1.00  0.00
ATOM   2003  O4  LIG   128      27.820  -8.289  -9.849  1.00  0.00
ATOM   2004  C1  LIG   128      25.655 -16.077  -5.048  1.00  0.00
ATOM   2005  H1  LIG   128      25.852 -16.845  -5.806  1.00  0.00
ATOM   2006  H2  LIG   128      26.592 -15.954  -4.489  1.00  0.00
ATOM   2007  C2  LIG   128      24.561 -16.575  -4.096  1.00  0.00
ATOM   2008  H3  LIG   128      23.625 -16.698  -4.654  1.00  0.00
ATOM   2009  H4  LIG   128      24.364 -15.806  -3.337  1.00  0.00
ATOM   2010  C3  LIG   128      25.305 -14.757  -5.744  1.00  0.00
ATOM   2011  H5  LIG   128      25.110 -13.988  -4.985  1.00  0.00
ATOM   2012  H6  LIG   128      24.367 -14.880  -6.301  1.00  0.00
ATOM   2013  C4  LIG   128      24.910 -17.894  -3.397  1.00  0.00
ATOM   2014  H7  LIG   128      25.847 -17.771  -2.838  1.00  0.00
ATOM   2015  H8  LIG   128      25.109 -18.663  -4.155  1.00  0.00
ATOM   2016  C5  LIG   128      26.397 -14.260  -6.699  1.00  0.00
ATOM   2017  H9  LIG   128      27.336 -14.139  -6.144  1.00  0.00
ATOM   2018  H10 LIG   128      26.590 -15.026  -7.460  1.00  0.00
ATOM   2019  C6  LIG   128      23.816 -18.394  -2.446  1.00  0.00
ATOM   2020  H11 LIG   128      22.880 -18.516  -3.005  1.00  0.00
ATOM   2021  H12 LIG   128      23.618 -17.624  -1.687  1.00  0.00
ATOM   2022  C7  LIG   128      26.045 -12.939  -7.391  1.00  0.00
ATOM   2023  H13 LIG   128      25.104 -13.060  -7.943  1.00  0.00
```

ATOM 2024 H14 LIG 128 25.857 -12.171 -6.629 1.00 0.00
ATOM 2025 C8 LIG 128 24.164 -19.713 -1.747 1.00 0.00
ATOM 2026 H15 LIG 128 24.362 -20.483 -2.505 1.00 0.00
ATOM 2027 H16 LIG 128 25.101 -19.591 -1.189 1.00 0.00
ATOM 2028 C9 LIG 128 27.143 -12.455 -8.347 1.00 0.00
ATOM 2029 H17 LIG 128 27.322 -13.206 -9.124 1.00 0.00
ATOM 2030 H18 LIG 128 28.083 -12.324 -7.803 1.00 0.00
ATOM 2031 C10 LIG 128 23.071 -20.213 -0.795 1.00 0.00
ATOM 2032 H19 LIG 128 22.135 -20.334 -1.354 1.00 0.00
ATOM 2033 H20 LIG 128 22.874 -19.444 -0.038 1.00 0.00
ATOM 2034 C11 LIG 128 26.772 -11.143 -9.012 1.00 0.00
ATOM 2035 H21 LIG 128 25.863 -11.236 -9.612 1.00 0.00
ATOM 2036 H22 LIG 128 26.648 -10.338 -8.282 1.00 0.00
ATOM 2037 C12 LIG 128 23.428 -21.532 -0.103 1.00 0.00
ATOM 2038 H23 LIG 128 24.342 -21.432 0.491 1.00 0.00
ATOM 2039 H24 LIG 128 22.630 -21.859 0.569 1.00 0.00
ATOM 2040 H25 LIG 128 23.595 -22.329 -0.834 1.00 0.00
TER
ATOM 2041 S1 LIG 129 -22.059 6.341 2.565 1.00 0.00
ATOM 2042 O1 LIG 129 -22.003 7.720 3.415 1.00 0.00
ATOM 2043 O2 LIG 129 -20.877 5.574 3.354 1.00 0.00
ATOM 2044 H26 LIG 129 -20.018 5.936 3.088 1.00 0.00

ATOM	2045	O3	LIG	129	-23.276	5.624	2.855	1.00	0.00
ATOM	2046	O4	LIG	129	-21.642	6.608	1.203	1.00	0.00
ATOM	2047	C1	LIG	129	-24.387	14.577	5.366	1.00	0.00
ATOM	2048	H1	LIG	129	-24.423	14.257	6.415	1.00	0.00
ATOM	2049	H2	LIG	129	-23.377	14.973	5.203	1.00	0.00
ATOM	2050	C2	LIG	129	-25.409	15.699	5.144	1.00	0.00
ATOM	2051	H3	LIG	129	-26.420	15.304	5.308	1.00	0.00
ATOM	2052	H4	LIG	129	-25.375	16.018	4.095	1.00	0.00
ATOM	2053	C3	LIG	129	-24.607	13.362	4.459	1.00	0.00
ATOM	2054	H5	LIG	129	-24.569	13.681	3.409	1.00	0.00
ATOM	2055	H6	LIG	129	-25.619	12.968	4.621	1.00	0.00
ATOM	2056	C4	LIG	129	-25.191	16.916	6.050	1.00	0.00
ATOM	2057	H7	LIG	129	-24.180	17.312	5.886	1.00	0.00
ATOM	2058	H8	LIG	129	-25.225	16.597	7.099	1.00	0.00
ATOM	2059	C5	LIG	129	-23.587	12.239	4.682	1.00	0.00
ATOM	2060	H9	LIG	129	-22.575	12.632	4.524	1.00	0.00
ATOM	2061	H10	LIG	129	-23.627	11.916	5.730	1.00	0.00
ATOM	2062	C6	LIG	129	-26.213	18.037	5.830	1.00	0.00
ATOM	2063	H11	LIG	129	-27.224	17.641	5.993	1.00	0.00
ATOM	2064	H12	LIG	129	-26.179	18.357	4.779	1.00	0.00
ATOM	2065	C7	LIG	129	-23.808	11.026	3.772	1.00	0.00
ATOM	2066	H13	LIG	129	-24.821	10.636	3.930	1.00	0.00

ATOM 2067 H14 LIG 129 -23.763 11.349 2.724 1.00 0.00

ATOM 2068 C8 LIG 129 -25.996 19.255 6.735 1.00 0.00

ATOM 2069 H15 LIG 129 -26.031 18.937 7.785 1.00 0.00

ATOM 2070 H16 LIG 129 -24.985 19.651 6.572 1.00 0.00

ATOM 2071 C9 LIG 129 -22.784 9.908 4.009 1.00 0.00

ATOM 2072 H17 LIG 129 -22.837 9.564 5.047 1.00 0.00

ATOM 2073 H18 LIG 129 -21.770 10.287 3.846 1.00 0.00

ATOM 2074 C10 LIG 129 -27.018 20.377 6.515 1.00 0.00

ATOM 2075 H19 LIG 129 -28.027 19.981 6.677 1.00 0.00

ATOM 2076 H20 LIG 129 -26.982 20.696 5.466 1.00 0.00

ATOM 2077 C11 LIG 129 -23.022 8.725 3.090 1.00 0.00

ATOM 2078 H21 LIG 129 -24.006 8.278 3.252 1.00 0.00

ATOM 2079 H22 LIG 129 -22.912 8.999 2.038 1.00 0.00

ATOM 2080 C12 LIG 129 -26.792 21.588 7.425 1.00 0.00

ATOM 2081 H23 LIG 129 -25.802 22.027 7.261 1.00 0.00

ATOM 2082 H24 LIG 129 -27.535 22.369 7.242 1.00 0.00

ATOM 2083 H25 LIG 129 -26.857 21.308 8.480 1.00 0.00

TER

ATOM 2084 S1 LIG 130 12.831 -11.733 22.757 1.00 0.00

ATOM 2085 O1 LIG 130 14.379 -11.262 22.862 1.00 0.00

ATOM 2086 O2 LIG 130 12.255 -10.373 22.105 1.00 0.00

ATOM 2087 H26 LIG 130 12.206 -9.681 22.782 1.00 0.00

```
ATOM   2088  O3  LIG   130      12.687 -12.759  21.755  1.00  0.00
ATOM   2089  O4  LIG   130      12.295 -11.897  24.094  1.00  0.00
ATOM   2090  C1  LIG   130      21.687 -12.279  24.302  1.00  0.00
ATOM   2091  H1  LIG   130      21.928 -11.898  23.302  1.00  0.00
ATOM   2092  H2  LIG   130      21.675 -11.405  24.966  1.00  0.00
ATOM   2093  C2  LIG   130      22.789 -13.243  24.757  1.00  0.00
ATOM   2094  H3  LIG   130      22.801 -14.117  24.093  1.00  0.00
ATOM   2095  H4  LIG   130      22.547 -13.625  25.757  1.00  0.00
ATOM   2096  C3  LIG   130      20.292 -12.914  24.278  1.00  0.00
ATOM   2097  H5  LIG   130      20.049 -13.293  25.280  1.00  0.00
ATOM   2098  H6  LIG   130      20.304 -13.790  23.617  1.00  0.00
ATOM   2099  C4  LIG   130      24.186 -12.610  24.782  1.00  0.00
ATOM   2100  H7  LIG   130      24.174 -11.736  25.447  1.00  0.00
ATOM   2101  H8  LIG   130      24.427 -12.227  23.782  1.00  0.00
ATOM   2102  C5  LIG   130      19.189 -11.952  23.821  1.00  0.00
ATOM   2103  H9  LIG   130      19.177 -11.074  24.481  1.00  0.00
ATOM   2104  H10 LIG   130      19.429 -11.575  22.819  1.00  0.00
ATOM   2105  C6  LIG   130      25.289 -13.573  25.235  1.00  0.00
ATOM   2106  H11 LIG   130      25.300 -14.448  24.572  1.00  0.00
ATOM   2107  H12 LIG   130      25.047 -13.956  26.236  1.00  0.00
ATOM   2108  C7  LIG   130      17.795 -12.588  23.801  1.00  0.00
ATOM   2109  H13 LIG   130      17.809 -13.467  23.144  1.00  0.00
```

ATOM 2110 H14 LIG 130 17.554 -12.960 24.806 1.00 0.00
ATOM 2111 C8 LIG 130 26.685 -12.942 25.261 1.00 0.00
ATOM 2112 H15 LIG 130 26.929 -12.559 24.261 1.00 0.00
ATOM 2113 H16 LIG 130 26.674 -12.067 25.924 1.00 0.00
ATOM 2114 C9 LIG 130 16.702 -11.617 23.337 1.00 0.00
ATOM 2115 H17 LIG 130 16.923 -11.257 22.327 1.00 0.00
ATOM 2116 H18 LIG 130 16.676 -10.740 23.992 1.00 0.00
ATOM 2117 C10 LIG 130 27.789 -13.904 25.714 1.00 0.00
ATOM 2118 H19 LIG 130 27.799 -14.778 25.052 1.00 0.00
ATOM 2119 H20 LIG 130 27.546 -14.285 26.714 1.00 0.00
ATOM 2120 C11 LIG 130 15.333 -12.271 23.334 1.00 0.00
ATOM 2121 H21 LIG 130 15.294 -13.124 22.653 1.00 0.00
ATOM 2122 H22 LIG 130 15.032 -12.588 24.336 1.00 0.00
ATOM 2123 C12 LIG 130 29.180 -13.263 25.736 1.00 0.00
ATOM 2124 H23 LIG 130 29.209 -12.406 26.417 1.00 0.00
ATOM 2125 H24 LIG 130 29.942 -13.974 26.065 1.00 0.00
ATOM 2126 H25 LIG 130 29.466 -12.905 24.742 1.00 0.00
TER
ATOM 2127 S1 LIG 131 -0.775 -14.715 23.850 1.00 0.00
ATOM 2128 O1 LIG 131 -2.025 -13.869 24.443 1.00 0.00
ATOM 2129 O2 LIG 131 -0.858 -15.946 24.892 1.00 0.00
ATOM 2130 H26 LIG 131 -0.516 -15.667 25.755 1.00 0.00

ATOM	2131	O3	LIG	131	-1.105	-15.254	22.555	1.00	0.00
ATOM	2132	O4	LIG	131	0.448	-13.970	24.073	1.00	0.00
ATOM	2133	C1	LIG	131	-6.969	-8.206	24.468	1.00	0.00
ATOM	2134	H1	LIG	131	-7.788	-8.933	24.534	1.00	0.00
ATOM	2135	H2	LIG	131	-6.689	-7.969	25.502	1.00	0.00
ATOM	2136	C2	LIG	131	-7.475	-6.934	23.778	1.00	0.00
ATOM	2137	H3	LIG	131	-7.756	-7.171	22.744	1.00	0.00
ATOM	2138	H4	LIG	131	-6.656	-6.207	23.711	1.00	0.00
ATOM	2139	C3	LIG	131	-5.775	-8.853	23.757	1.00	0.00
ATOM	2140	H5	LIG	131	-4.954	-8.127	23.693	1.00	0.00
ATOM	2141	H6	LIG	131	-6.055	-9.088	22.721	1.00	0.00
ATOM	2142	C4	LIG	131	-8.669	-6.284	24.488	1.00	0.00
ATOM	2143	H7	LIG	131	-8.388	-6.046	25.523	1.00	0.00
ATOM	2144	H8	LIG	131	-9.488	-7.011	24.556	1.00	0.00
ATOM	2145	C5	LIG	131	-5.270	-10.127	24.444	1.00	0.00
ATOM	2146	H9	LIG	131	-4.992	-9.894	25.480	1.00	0.00
ATOM	2147	H10	LIG	131	-6.089	-10.855	24.505	1.00	0.00
ATOM	2148	C6	LIG	131	-9.177	-5.013	23.799	1.00	0.00
ATOM	2149	H11	LIG	131	-9.456	-5.250	22.765	1.00	0.00
ATOM	2150	H12	LIG	131	-8.356	-4.285	23.731	1.00	0.00
ATOM	2151	C7	LIG	131	-4.075	-10.770	23.732	1.00	0.00
ATOM	2152	H13	LIG	131	-4.353	-11.000	22.695	1.00	0.00

```
ATOM   2153  H14 LIG   131      -3.255 -10.043  23.676  1.00  0.00
ATOM   2154  C8  LIG   131     -10.370  -4.363  24.508  1.00  0.00
ATOM   2155  H15 LIG   131     -11.191  -5.089  24.576  1.00  0.00
ATOM   2156  H16 LIG   131     -10.090  -4.126  25.543  1.00  0.00
ATOM   2157  C9  LIG   131      -3.582 -12.047  24.426  1.00  0.00
ATOM   2158  H17 LIG   131      -4.389 -12.786  24.468  1.00  0.00
ATOM   2159  H18 LIG   131      -3.296 -11.825  25.459  1.00  0.00
ATOM   2160  C10 LIG   131     -10.878  -3.091  23.820  1.00  0.00
ATOM   2161  H19 LIG   131     -11.157  -3.328  22.786  1.00  0.00
ATOM   2162  H20 LIG   131     -10.058  -2.365  23.754  1.00  0.00
ATOM   2163  C11 LIG   131      -2.396 -12.657  23.705  1.00  0.00
ATOM   2164  H21 LIG   131      -2.648 -12.943  22.681  1.00  0.00
ATOM   2165  H22 LIG   131      -1.535 -11.982  23.696  1.00  0.00
ATOM   2166  C12 LIG   131     -12.071  -2.450  24.536  1.00  0.00
ATOM   2167  H23 LIG   131     -11.812  -2.171  25.562  1.00  0.00
ATOM   2168  H24 LIG   131     -12.407  -1.546  24.021  1.00  0.00
ATOM   2169  H25 LIG   131     -12.919  -3.139  24.586  1.00  0.00
TER
ATOM   2170  S1  LIG   132      20.589  17.402 -25.063  1.00  0.00
ATOM   2171  O1  LIG   132      20.783  15.973 -25.805  1.00  0.00
ATOM   2172  O2  LIG   132      20.110  18.235 -26.362  1.00  0.00
ATOM   2173  H26 LIG   132      20.871  18.411 -26.936  1.00  0.00
```

ATOM	2174	O3	LIG	132	19.458	17.353	-24.170	1.00	0.00
ATOM	2175	O4	LIG	132	21.882	17.887	-24.624	1.00	0.00
ATOM	2176	C1	LIG	132	22.213	8.597	-26.029	1.00	0.00
ATOM	2177	H1	LIG	132	21.276	8.440	-26.578	1.00	0.00
ATOM	2178	H2	LIG	132	22.985	8.771	-26.789	1.00	0.00
ATOM	2179	C2	LIG	132	22.558	7.326	-25.243	1.00	0.00
ATOM	2180	H3	LIG	132	21.786	7.150	-24.483	1.00	0.00
ATOM	2181	H4	LIG	132	23.495	7.482	-24.693	1.00	0.00
ATOM	2182	C3	LIG	132	22.081	9.843	-25.147	1.00	0.00
ATOM	2183	H5	LIG	132	23.019	10.001	-24.599	1.00	0.00
ATOM	2184	H6	LIG	132	21.310	9.667	-24.385	1.00	0.00
ATOM	2185	C4	LIG	132	22.692	6.077	-26.123	1.00	0.00
ATOM	2186	H7	LIG	132	23.465	6.252	-26.883	1.00	0.00
ATOM	2187	H8	LIG	132	21.756	5.921	-26.674	1.00	0.00
ATOM	2188	C5	LIG	132	21.733	11.114	-25.931	1.00	0.00
ATOM	2189	H9	LIG	132	22.502	11.290	-26.695	1.00	0.00
ATOM	2190	H10	LIG	132	20.793	10.958	-26.476	1.00	0.00
ATOM	2191	C6	LIG	132	23.036	4.806	-25.338	1.00	0.00
ATOM	2192	H11	LIG	132	22.263	4.632	-24.578	1.00	0.00
ATOM	2193	H12	LIG	132	23.973	4.963	-24.787	1.00	0.00
ATOM	2194	C7	LIG	132	21.604	12.359	-25.047	1.00	0.00
ATOM	2195	H13	LIG	132	20.837	12.181	-24.282	1.00	0.00

```
ATOM   2196  H14 LIG   132      22.546  12.517 -24.506  1.00  0.00
ATOM   2197  C8  LIG   132      23.170   3.558 -26.217  1.00  0.00
ATOM   2198  H15 LIG   132      22.233   3.399 -26.768  1.00  0.00
ATOM   2199  H16 LIG   132      23.942   3.733 -26.978  1.00  0.00
ATOM   2200  C9  LIG   132      21.251  13.622 -25.843  1.00  0.00
ATOM   2201  H17 LIG   132      20.301  13.482 -26.369  1.00  0.00
ATOM   2202  H18 LIG   132      22.015  13.812 -26.603  1.00  0.00
ATOM   2203  C10 LIG   132      23.514   2.286 -25.434  1.00  0.00
ATOM   2204  H19 LIG   132      22.742   2.112 -24.674  1.00  0.00
ATOM   2205  H20 LIG   132      24.450   2.443 -24.885  1.00  0.00
ATOM   2206  C11 LIG   132      21.137  14.839 -24.945  1.00  0.00
ATOM   2207  H21 LIG   132      20.349  14.718 -24.197  1.00  0.00
ATOM   2208  H22 LIG   132      22.083  15.066 -24.446  1.00  0.00
ATOM   2209  C12 LIG   132      23.645   1.044 -26.321  1.00  0.00
ATOM   2210  H23 LIG   132      24.433   1.176 -27.070  1.00  0.00
ATOM   2211  H24 LIG   132      23.892   0.157 -25.733  1.00  0.00
ATOM   2212  H25 LIG   132      22.713   0.840 -26.856  1.00  0.00
TER
ATOM   2213  S1  LIG   133     -25.726   0.261  17.108  1.00  0.00
ATOM   2214  O1  LIG   133     -26.264  -0.834  18.176  1.00  0.00
ATOM   2215  O2  LIG   133     -25.991  -0.615  15.778  1.00  0.00
ATOM   2216  H26 LIG   133     -25.319  -1.309  15.704  1.00  0.00
```

```
ATOM   2217  O3  LIG   133     -26.629   1.383  17.045  1.00  0.00
ATOM   2218  O4  LIG   133     -24.300   0.444  17.295  1.00  0.00
ATOM   2219  C1  LIG   133     -28.135  -3.246  25.046  1.00  0.00
ATOM   2220  H1  LIG   133     -29.119  -3.546  24.664  1.00  0.00
ATOM   2221  H2  LIG   133     -27.481  -4.118  24.916  1.00  0.00
ATOM   2222  C2  LIG   133     -28.250  -2.921  26.540  1.00  0.00
ATOM   2223  H3  LIG   133     -28.905  -2.050  26.670  1.00  0.00
ATOM   2224  H4  LIG   133     -27.266  -2.621  26.922  1.00  0.00
ATOM   2225  C3  LIG   133     -27.599  -2.082  24.206  1.00  0.00
ATOM   2226  H5  LIG   133     -26.613  -1.784  24.587  1.00  0.00
ATOM   2227  H6  LIG   133     -28.253  -1.210  24.338  1.00  0.00
ATOM   2228  C4  LIG   133     -28.785  -4.084  27.382  1.00  0.00
ATOM   2229  H7  LIG   133     -28.130  -4.956  27.253  1.00  0.00
ATOM   2230  H8  LIG   133     -29.769  -4.386  27.000  1.00  0.00
ATOM   2231  C5  LIG   133     -27.486  -2.406  22.712  1.00  0.00
ATOM   2232  H9  LIG   133     -26.835  -3.279  22.578  1.00  0.00
ATOM   2233  H10 LIG   133     -28.472  -2.700  22.329  1.00  0.00
ATOM   2234  C6  LIG   133     -28.902  -3.759  28.876  1.00  0.00
ATOM   2235  H11 LIG   133     -29.556  -2.888  29.006  1.00  0.00
ATOM   2236  H12 LIG   133     -27.917  -3.458  29.259  1.00  0.00
ATOM   2237  C7  LIG   133     -26.947  -1.240  21.875  1.00  0.00
ATOM   2238  H13 LIG   133     -27.598  -0.367  22.011  1.00  0.00
```

ATOM 2239 H14 LIG 133 -25.960 -0.950 22.255 1.00 0.00
ATOM 2240 C8 LIG 133 -29.436 -4.922 29.719 1.00 0.00
ATOM 2241 H15 LIG 133 -30.421 -5.224 29.338 1.00 0.00
ATOM 2242 H16 LIG 133 -28.782 -5.794 29.589 1.00 0.00
ATOM 2243 C9 LIG 133 -26.845 -1.575 20.381 1.00 0.00
ATOM 2244 H17 LIG 133 -27.830 -1.846 19.988 1.00 0.00
ATOM 2245 H18 LIG 133 -26.191 -2.441 20.235 1.00 0.00
ATOM 2246 C10 LIG 133 -29.553 -4.598 31.213 1.00 0.00
ATOM 2247 H19 LIG 133 -30.207 -3.727 31.342 1.00 0.00
ATOM 2248 H20 LIG 133 -28.569 -4.299 31.594 1.00 0.00
ATOM 2249 C11 LIG 133 -26.302 -0.408 19.579 1.00 0.00
ATOM 2250 H21 LIG 133 -26.950 0.469 19.652 1.00 0.00
ATOM 2251 H22 LIG 133 -25.288 -0.140 19.886 1.00 0.00
ATOM 2252 C12 LIG 133 -30.089 -5.766 32.046 1.00 0.00
ATOM 2253 H23 LIG 133 -29.438 -6.642 31.963 1.00 0.00
ATOM 2254 H24 LIG 133 -30.159 -5.503 33.105 1.00 0.00
ATOM 2255 H25 LIG 133 -31.087 -6.065 31.712 1.00 0.00
TER
ATOM 2256 S1 LIG 134 14.040 16.014 -14.168 1.00 0.00
ATOM 2257 O1 LIG 134 15.383 15.880 -15.066 1.00 0.00
ATOM 2258 O2 LIG 134 13.018 16.311 -15.384 1.00 0.00
ATOM 2259 H26 LIG 134 13.138 17.220 -15.697 1.00 0.00

```
ATOM   2260  O3  LIG   134      13.669  14.730 -13.629  1.00  0.00
ATOM   2261  O4  LIG   134      14.149  17.189 -13.326  1.00  0.00
ATOM   2262  C1  LIG   134      22.678  14.442 -16.171  1.00  0.00
ATOM   2263  H1  LIG   134      22.410  13.736 -16.966  1.00  0.00
ATOM   2264  H2  LIG   134      22.795  15.419 -16.658  1.00  0.00
ATOM   2265  C2  LIG   134      24.018  14.024 -15.553  1.00  0.00
ATOM   2266  H3  LIG   134      23.902  13.047 -15.066  1.00  0.00
ATOM   2267  H4  LIG   134      24.286  14.730 -14.757  1.00  0.00
ATOM   2268  C3  LIG   134      21.534  14.518 -15.155  1.00  0.00
ATOM   2269  H5  LIG   134      21.801  15.226 -14.360  1.00  0.00
ATOM   2270  H6  LIG   134      21.419  13.541 -14.666  1.00  0.00
ATOM   2271  C4  LIG   134      25.165  13.948 -16.568  1.00  0.00
ATOM   2272  H7  LIG   134      25.282  14.926 -17.054  1.00  0.00
ATOM   2273  H8  LIG   134      24.897  13.244 -17.365  1.00  0.00
ATOM   2274  C5  LIG   134      20.193  14.933 -15.772  1.00  0.00
ATOM   2275  H9  LIG   134      20.307  15.908 -16.263  1.00  0.00
ATOM   2276  H10 LIG   134      19.923  14.224 -16.564  1.00  0.00
ATOM   2277  C6  LIG   134      26.504  13.529 -15.951  1.00  0.00
ATOM   2278  H11 LIG   134      26.387  12.553 -15.464  1.00  0.00
ATOM   2279  H12 LIG   134      26.773  14.235 -15.154  1.00  0.00
ATOM   2280  C7  LIG   134      19.052  15.011 -14.753  1.00  0.00
ATOM   2281  H13 LIG   134      18.940  14.036 -14.261  1.00  0.00
```

```
ATOM   2282  H14 LIG   134      19.320  15.725 -13.964  1.00  0.00
ATOM   2283  C8  LIG   134      27.652  13.454 -16.965  1.00  0.00
ATOM   2284  H15 LIG   134      27.385  12.747 -17.762  1.00  0.00
ATOM   2285  H16 LIG   134      27.768  14.430 -17.453  1.00  0.00
ATOM   2286  C9  LIG   134      17.715  15.422 -15.384  1.00  0.00
ATOM   2287  H17 LIG   134      17.428  14.703 -16.158  1.00  0.00
ATOM   2288  H18 LIG   134      17.815  16.397 -15.871  1.00  0.00
ATOM   2289  C10 LIG   134      28.992  13.034 -16.349  1.00  0.00
ATOM   2290  H19 LIG   134      28.874  12.059 -15.862  1.00  0.00
ATOM   2291  H20 LIG   134      29.259  13.741 -15.553  1.00  0.00
ATOM   2292  C11 LIG   134      16.606  15.498 -14.352  1.00  0.00
ATOM   2293  H21 LIG   134      16.433  14.531 -13.872  1.00  0.00
ATOM   2294  H22 LIG   134      16.811  16.254 -13.590  1.00  0.00
ATOM   2295  C12 LIG   134      30.131  12.961 -17.370  1.00  0.00
ATOM   2296  H23 LIG   134      30.294  13.932 -17.850  1.00  0.00
ATOM   2297  H24 LIG   134      31.071  12.662 -16.900  1.00  0.00
ATOM   2298  H25 LIG   134      29.909  12.236 -18.160  1.00  0.00
TER
ATOM   2299  S1  LIG   135     -27.792   4.947 -28.327  1.00  0.00
ATOM   2300  O1  LIG   135     -27.693   6.412 -27.639  1.00  0.00
ATOM   2301  O2  LIG   135     -28.180   4.127 -26.990  1.00  0.00
ATOM   2302  H26 LIG   135     -27.396   4.029 -26.428  1.00  0.00
```

ATOM	2303	O3	LIG	135	-28.941	4.878	-29.194	1.00	0.00
ATOM	2304	O4	LIG	135	-26.475	4.546	-28.778	1.00	0.00
ATOM	2305	C1	LIG	135	-26.829	13.879	-27.698	1.00	0.00
ATOM	2306	H1	LIG	135	-27.763	13.983	-27.134	1.00	0.00
ATOM	2307	H2	LIG	135	-26.030	13.791	-26.951	1.00	0.00
ATOM	2308	C2	LIG	135	-26.599	15.144	-28.534	1.00	0.00
ATOM	2309	H3	LIG	135	-27.397	15.233	-29.282	1.00	0.00
ATOM	2310	H4	LIG	135	-25.664	15.039	-29.100	1.00	0.00
ATOM	2311	C3	LIG	135	-26.883	12.595	-28.534	1.00	0.00
ATOM	2312	H5	LIG	135	-25.947	12.490	-29.097	1.00	0.00
ATOM	2313	H6	LIG	135	-27.681	12.686	-29.283	1.00	0.00
ATOM	2314	C4	LIG	135	-26.543	16.429	-27.700	1.00	0.00
ATOM	2315	H7	LIG	135	-25.743	16.341	-26.953	1.00	0.00
ATOM	2316	H8	LIG	135	-27.477	16.534	-27.134	1.00	0.00
ATOM	2317	C5	LIG	135	-27.116	11.331	-27.699	1.00	0.00
ATOM	2318	H9	LIG	135	-26.321	11.241	-26.948	1.00	0.00
ATOM	2319	H10	LIG	135	-28.054	11.434	-27.139	1.00	0.00
ATOM	2320	C6	LIG	135	-26.314	17.694	-28.535	1.00	0.00
ATOM	2321	H11	LIG	135	-27.112	17.782	-29.283	1.00	0.00
ATOM	2322	H12	LIG	135	-25.379	17.590	-29.102	1.00	0.00
ATOM	2323	C7	LIG	135	-27.167	10.049	-28.538	1.00	0.00
ATOM	2324	H13	LIG	135	-27.961	10.141	-29.290	1.00	0.00

```
ATOM   2325  H14 LIG   135     -26.227   9.944 -29.094  1.00  0.00
ATOM   2326  C8  LIG   135     -26.258  18.979 -27.703  1.00  0.00
ATOM   2327  H15 LIG   135     -27.192  19.086 -27.136  1.00  0.00
ATOM   2328  H16 LIG   135     -25.459  18.891 -26.954  1.00  0.00
ATOM   2329  C9  LIG   135     -27.407   8.791 -27.691  1.00  0.00
ATOM   2330  H17 LIG   135     -28.354   8.878 -27.149  1.00  0.00
ATOM   2331  H18 LIG   135     -26.615   8.688 -26.942  1.00  0.00
ATOM   2332  C10 LIG   135     -26.029  20.245 -28.537  1.00  0.00
ATOM   2333  H19 LIG   135     -26.826  20.333 -29.284  1.00  0.00
ATOM   2334  H20 LIG   135     -25.094  20.140 -29.101  1.00  0.00
ATOM   2335  C11 LIG   135     -27.445   7.538 -28.545  1.00  0.00
ATOM   2336  H21 LIG   135     -28.255   7.573 -29.277  1.00  0.00
ATOM   2337  H22 LIG   135     -26.495   7.366 -29.057  1.00  0.00
ATOM   2338  C12 LIG   135     -25.975  21.524 -27.695  1.00  0.00
ATOM   2339  H23 LIG   135     -25.164  21.480 -26.961  1.00  0.00
ATOM   2340  H24 LIG   135     -25.809  22.406 -28.319  1.00  0.00
ATOM   2341  H25 LIG   135     -26.909  21.676 -27.146  1.00  0.00
TER
ATOM   2342  S1  LIG   136       9.458  -8.749 -24.880  1.00  0.00
ATOM   2343  O1  LIG   136       9.818  -7.176 -25.035  1.00  0.00
ATOM   2344  O2  LIG   136       8.667  -8.634 -23.477  1.00  0.00
ATOM   2345  H26 LIG   136       9.301  -8.504 -22.755  1.00  0.00
```

ATOM 2346 O3 LIG 136 8.483 -9.138 -25.868 1.00 0.00

ATOM 2347 O4 LIG 136 10.684 -9.498 -24.688 1.00 0.00

ATOM 2348 C1 LIG 136 12.561 -1.175 -28.637 1.00 0.00

ATOM 2349 H1 LIG 136 11.607 -0.656 -28.483 1.00 0.00

ATOM 2350 H2 LIG 136 13.182 -0.937 -27.764 1.00 0.00

ATOM 2351 C2 LIG 136 13.236 -0.627 -29.900 1.00 0.00

ATOM 2352 H3 LIG 136 12.616 -0.864 -30.774 1.00 0.00

ATOM 2353 H4 LIG 136 14.191 -1.146 -30.055 1.00 0.00

ATOM 2354 C3 LIG 136 12.313 -2.687 -28.684 1.00 0.00

ATOM 2355 H5 LIG 136 13.269 -3.206 -28.837 1.00 0.00

ATOM 2356 H6 LIG 136 11.695 -2.925 -29.560 1.00 0.00

ATOM 2357 C4 LIG 136 13.486 0.885 -29.856 1.00 0.00

ATOM 2358 H7 LIG 136 14.108 1.124 -28.982 1.00 0.00

ATOM 2359 H8 LIG 136 12.532 1.405 -29.699 1.00 0.00

ATOM 2360 C5 LIG 136 11.636 -3.235 -27.423 1.00 0.00

ATOM 2361 H9 LIG 136 12.252 -2.996 -26.547 1.00 0.00

ATOM 2362 H10 LIG 136 10.679 -2.720 -27.272 1.00 0.00

ATOM 2363 C6 LIG 136 14.160 1.435 -31.118 1.00 0.00

ATOM 2364 H11 LIG 136 13.540 1.196 -31.991 1.00 0.00

ATOM 2365 H12 LIG 136 15.115 0.915 -31.274 1.00 0.00

ATOM 2366 C7 LIG 136 11.392 -4.748 -27.472 1.00 0.00

ATOM 2367 H13 LIG 136 10.778 -4.986 -28.350 1.00 0.00

```
ATOM   2368  H14 LIG   136      12.350  -5.263 -27.618  1.00  0.00
ATOM   2369  C8  LIG   136      14.410   2.946 -31.074  1.00  0.00
ATOM   2370  H15 LIG   136      13.456   3.467 -30.919  1.00  0.00
ATOM   2371  H16 LIG   136      15.031   3.185 -30.200  1.00  0.00
ATOM   2372  C9  LIG   136      10.708  -5.283 -26.207  1.00  0.00
ATOM   2373  H17 LIG   136       9.741  -4.790 -26.065  1.00  0.00
ATOM   2374  H18 LIG   136      11.318  -5.056 -25.327  1.00  0.00
ATOM   2375  C10 LIG   136      15.085   3.497 -32.336  1.00  0.00
ATOM   2376  H19 LIG   136      14.465   3.257 -33.209  1.00  0.00
ATOM   2377  H20 LIG   136      16.039   2.977 -32.490  1.00  0.00
ATOM   2378  C11 LIG   136      10.486  -6.782 -26.280  1.00  0.00
ATOM   2379  H21 LIG   136       9.839  -7.055 -27.116  1.00  0.00
ATOM   2380  H22 LIG   136      11.430  -7.329 -26.353  1.00  0.00
ATOM   2381  C12 LIG   136      15.329   5.008 -32.282  1.00  0.00
ATOM   2382  H23 LIG   136      15.974   5.274 -31.439  1.00  0.00
ATOM   2383  H24 LIG   136      15.812   5.367 -33.195  1.00  0.00
ATOM   2384  H25 LIG   136      14.390   5.556 -32.165  1.00  0.00
TER
ATOM   2385  S1  LIG   137     -17.752 -20.911 -30.591  1.00  0.00
ATOM   2386  O1  LIG   137     -17.419 -20.643 -29.027  1.00  0.00
ATOM   2387  O2  LIG   137     -16.280 -20.577 -31.166  1.00  0.00
ATOM   2388  H26 LIG   137     -16.135 -19.619 -31.156  1.00  0.00
```

```
ATOM   2389  O3  LIG   137     -17.971 -22.317 -30.822  1.00  0.00
ATOM   2390  O4  LIG   137     -18.693 -19.910 -31.053  1.00  0.00
ATOM   2391  C1  LIG   137     -19.005 -20.570 -21.679  1.00  0.00
ATOM   2392  H1  LIG   137     -18.096 -21.154 -21.486  1.00  0.00
ATOM   2393  H2  LIG   137     -18.706 -19.515 -21.627  1.00  0.00
ATOM   2394  C2  LIG   137     -20.034 -20.857 -20.580  1.00  0.00
ATOM   2395  H3  LIG   137     -20.334 -21.911 -20.631  1.00  0.00
ATOM   2396  H4  LIG   137     -20.944 -20.273 -20.773  1.00  0.00
ATOM   2397  C3  LIG   137     -19.515 -20.881 -23.090  1.00  0.00
ATOM   2398  H5  LIG   137     -20.423 -20.295 -23.285  1.00  0.00
ATOM   2399  H6  LIG   137     -19.817 -21.936 -23.141  1.00  0.00
ATOM   2400  C4  LIG   137     -19.526 -20.545 -19.167  1.00  0.00
ATOM   2401  H7  LIG   137     -19.227 -19.489 -19.115  1.00  0.00
ATOM   2402  H8  LIG   137     -18.616 -21.127 -18.973  1.00  0.00
ATOM   2403  C5  LIG   137     -18.486 -20.598 -24.190  1.00  0.00
ATOM   2404  H9  LIG   137     -18.181 -19.544 -24.139  1.00  0.00
ATOM   2405  H10 LIG   137     -17.579 -21.186 -23.999  1.00  0.00
ATOM   2406  C6  LIG   137     -20.554 -20.832 -18.067  1.00  0.00
ATOM   2407  H11 LIG   137     -20.854 -21.887 -18.119  1.00  0.00
ATOM   2408  H12 LIG   137     -21.465 -20.249 -18.261  1.00  0.00
ATOM   2409  C7  LIG   137     -19.000 -20.908 -25.600  1.00  0.00
ATOM   2410  H13 LIG   137     -19.307 -21.960 -25.650  1.00  0.00
```

ATOM 2411 H14 LIG 137 -19.904 -20.315 -25.793 1.00 0.00

ATOM 2412 C8 LIG 137 -20.048 -20.521 -16.654 1.00 0.00

ATOM 2413 H15 LIG 137 -19.138 -21.104 -16.459 1.00 0.00

ATOM 2414 H16 LIG 137 -19.747 -19.466 -16.602 1.00 0.00

ATOM 2415 C9 LIG 137 -17.959 -20.625 -26.691 1.00 0.00

ATOM 2416 H17 LIG 137 -17.061 -21.228 -26.519 1.00 0.00

ATOM 2417 H18 LIG 137 -17.653 -19.574 -26.654 1.00 0.00

ATOM 2418 C10 LIG 137 -21.075 -20.808 -15.553 1.00 0.00

ATOM 2419 H19 LIG 137 -21.374 -21.861 -15.606 1.00 0.00

ATOM 2420 H20 LIG 137 -21.983 -20.224 -15.747 1.00 0.00

ATOM 2421 C11 LIG 137 -18.497 -20.933 -28.075 1.00 0.00

ATOM 2422 H21 LIG 137 -18.770 -21.986 -28.178 1.00 0.00

ATOM 2423 H22 LIG 137 -19.357 -20.306 -28.326 1.00 0.00

ATOM 2424 C12 LIG 137 -20.558 -20.494 -14.146 1.00 0.00

ATOM 2425 H23 LIG 137 -20.282 -19.438 -14.052 1.00 0.00

ATOM 2426 H24 LIG 137 -21.314 -20.707 -13.386 1.00 0.00

ATOM 2427 H25 LIG 137 -19.671 -21.089 -13.908 1.00 0.00

TER

END

Table D: The number of atoms present in the system of AH-Sum

ATOM 1 N GLY 1 25.126 -11.130 -1.248 1.00 0.00

```
ATOM      2  H1  GLY     1      24.425 -10.415  -1.372  1.00  0.00
ATOM      3  H2  GLY     1      24.741 -12.017  -1.540  1.00  0.00
ATOM      4  H3  GLY     1      25.997 -10.804  -1.643  1.00  0.00
ATOM      5  CA  GLY     1      25.386 -11.275   0.212  1.00  0.00
ATOM      6  HA2 GLY     1      25.154 -12.291   0.533  1.00  0.00
ATOM      7  HA3 GLY     1      26.432 -11.057   0.428  1.00  0.00
ATOM      8  C   GLY     1      24.502 -10.294   0.986  1.00  0.00
ATOM      9  O   GLY     1      24.973  -9.305   1.513  1.00  0.00
ATOM     10  N   PRO     2      23.195 -10.579   1.052  1.00  0.00
ATOM     11  CD  PRO     2      22.552 -11.755   0.440  1.00  0.00
ATOM     12  HD2 PRO     2      23.113 -12.653   0.701  1.00  0.00
ATOM     13  HD3 PRO     2      22.538 -11.639  -0.644  1.00  0.00
ATOM     14  CG  PRO     2      21.173 -11.742   1.035  1.00  0.00
ATOM     15  HG2 PRO     2      21.157 -12.376   1.922  1.00  0.00
ATOM     16  HG3 PRO     2      20.461 -12.124   0.304  1.00  0.00
ATOM     17  CB  PRO     2      20.877 -10.321   1.375  1.00  0.00
ATOM     18  HB2 PRO     2      20.208 -10.307   2.235  1.00  0.00
ATOM     19  HB3 PRO     2      20.436  -9.744   0.562  1.00  0.00
ATOM     20  CA  PRO     2      22.231  -9.727   1.761  1.00  0.00
ATOM     21  HA  PRO     2      22.370  -8.700   1.425  1.00  0.00
ATOM     22  C   PRO     2      22.420  -9.797   3.279  1.00  0.00
ATOM     23  O   PRO     2      21.975  -8.934   4.010  1.00  0.00
```

ATOM	24	N	MET	3	23.076	-10.818	3.758	1.00	0.00
ATOM	25	H	MET	3	23.449	-11.540	3.157	1.00	0.00
ATOM	26	CA	MET	3	23.291	-10.941	5.228	1.00	0.00
ATOM	27	HA	MET	3	22.335	-11.029	5.744	1.00	0.00
ATOM	28	CB	MET	3	24.120	-12.194	5.518	1.00	0.00
ATOM	29	HB2	MET	3	25.053	-12.152	4.955	1.00	0.00
ATOM	30	HB3	MET	3	24.342	-12.246	6.584	1.00	0.00
ATOM	31	CG	MET	3	23.329	-13.436	5.101	1.00	0.00
ATOM	32	HG2	MET	3	23.142	-13.411	4.027	1.00	0.00
ATOM	33	HG3	MET	3	23.895	-14.334	5.350	1.00	0.00
ATOM	34	SD	MET	3	21.747	-13.467	5.980	1.00	0.00
ATOM	35	CE	MET	3	22.421	-13.545	7.658	1.00	0.00
ATOM	36	HE1	MET	3	23.038	-12.666	7.844	1.00	0.00
ATOM	37	HE2	MET	3	21.603	-13.573	8.378	1.00	0.00
ATOM	38	HE3	MET	3	23.029	-14.444	7.765	1.00	0.00
ATOM	39	C	MET	3	24.035	-9.706	5.739	1.00	0.00
ATOM	40	O	MET	3	23.789	-9.228	6.829	1.00	0.00
ATOM	41	N	ARG	4	24.945	-9.185	4.961	1.00	0.00
ATOM	42	H	ARG	4	25.157	-9.578	4.055	1.00	0.00
ATOM	43	CA	ARG	4	25.702	-7.981	5.403	1.00	0.00
ATOM	44	HA	ARG	4	26.286	-8.209	6.295	1.00	0.00
ATOM	45	CB	ARG	4	26.657	-7.541	4.292	1.00	0.00

ATOM	46	HB2	ARG	4	26.094	-7.374	3.374	1.00	0.00
ATOM	47	HB3	ARG	4	27.155	-6.617	4.586	1.00	0.00
ATOM	48	CG	ARG	4	27.704	-8.631	4.056	1.00	0.00
ATOM	49	HG2	ARG	4	28.209	-8.860	4.994	1.00	0.00
ATOM	50	HG3	ARG	4	27.216	-9.530	3.679	1.00	0.00
ATOM	51	CD	ARG	4	28.729	-8.140	3.032	1.00	0.00
ATOM	52	HD2	ARG	4	29.251	-7.262	3.412	1.00	0.00
ATOM	53	HD3	ARG	4	29.452	-8.927	2.815	1.00	0.00
ATOM	54	NE	ARG	4	28.032	-7.771	1.769	1.00	0.00
ATOM	55	HE	ARG	4	27.685	-8.476	1.134	1.00	0.00
ATOM	56	CZ	ARG	4	27.858	-6.514	1.461	1.00	0.00
ATOM	57	NH1	ARG	4	28.857	-5.678	1.545	1.00	0.00
ATOM	58	HH11	ARG	4	29.763	-6.007	1.848	1.00	0.00
ATOM	59	HH12	ARG	4	28.720	-4.707	1.306	1.00	0.00
ATOM	60	NH2	ARG	4	26.687	-6.093	1.070	1.00	0.00
ATOM	61	HH21	ARG	4	25.916	-6.743	1.006	1.00	0.00
ATOM	62	HH22	ARG	4	26.557	-5.120	0.833	1.00	0.00
ATOM	63	C	ARG	4	24.723	-6.847	5.711	1.00	0.00
ATOM	64	O	ARG	4	24.918	-6.077	6.630	1.00	0.00
ATOM	65	N	ARG	5	23.669	-6.738	4.948	1.00	0.00
ATOM	66	H	ARG	5	23.501	-7.374	4.181	1.00	0.00
ATOM	67	CA	ARG	5	22.678	-5.654	5.198	1.00	0.00

ATOM	68	HA	ARG	5	23.150	-4.678	5.091	1.00	0.00
ATOM	69	CB	ARG	5	21.539	-5.761	4.181	1.00	0.00
ATOM	70	HB2	ARG	5	21.087	-6.751	4.244	1.00	0.00
ATOM	71	HB3	ARG	5	20.786	-5.003	4.397	1.00	0.00
ATOM	72	CG	ARG	5	22.092	-5.543	2.771	1.00	0.00
ATOM	73	HG2	ARG	5	22.604	-4.582	2.725	1.00	0.00
ATOM	74	HG3	ARG	5	22.795	-6.341	2.528	1.00	0.00
ATOM	75	CD	ARG	5	20.940	-5.555	1.765	1.00	0.00
ATOM	76	HD2	ARG	5	20.342	-6.458	1.887	1.00	0.00
ATOM	77	HD3	ARG	5	20.308	-4.679	1.905	1.00	0.00
ATOM	78	NE	ARG	5	21.490	-5.531	0.380	1.00	0.00
ATOM	79	HE	ARG	5	22.017	-4.742	0.035	1.00	0.00
ATOM	80	CZ	ARG	5	21.295	-6.546	-0.417	1.00	0.00
ATOM	81	NH1	ARG	5	20.094	-7.033	-0.573	1.00	0.00
ATOM	82	HH11	ARG	5	19.317	-6.622	-0.075	1.00	0.00
ATOM	83	HH12	ARG	5	19.947	-7.819	-1.191	1.00	0.00
ATOM	84	NH2	ARG	5	22.301	-7.074	-1.058	1.00	0.00
ATOM	85	HH21	ARG	5	23.229	-6.695	-0.935	1.00	0.00
ATOM	86	HH22	ARG	5	22.147	-7.860	-1.674	1.00	0.00
ATOM	87	C	ARG	5	22.114	-5.792	6.613	1.00	0.00
ATOM	88	O	ARG	5	21.762	-4.818	7.248	1.00	0.00
ATOM	89	N	GLU	6	22.024	-6.995	7.111	1.00	0.00

```
ATOM     90  H   GLU     6      22.314  -7.810   6.588  1.00  0.00
ATOM     91  CA  GLU     6      21.482  -7.193   8.485  1.00  0.00
ATOM     92  HA  GLU     6      20.458  -6.825   8.544  1.00  0.00
ATOM     93  CB  GLU     6      21.486  -8.685   8.826  1.00  0.00
ATOM     94  HB2 GLU     6      22.497  -9.080   8.721  1.00  0.00
ATOM     95  HB3 GLU     6      21.147  -8.825   9.853  1.00  0.00
ATOM     96  CG  GLU     6      20.547  -9.428   7.874  1.00  0.00
ATOM     97  HG2 GLU     6      19.542  -9.015   7.955  1.00  0.00
ATOM     98  HG3 GLU     6      20.904  -9.315   6.850  1.00  0.00
ATOM     99  CD  GLU     6      20.519 -10.912   8.242  1.00  0.00
ATOM    100  OE1 GLU     6      21.317 -11.313   9.073  1.00  0.00
ATOM    101  OE2 GLU     6      19.697 -11.624   7.687  1.00  0.00
ATOM    102  C   GLU     6      22.354  -6.437   9.490  1.00  0.00
ATOM    103  O   GLU     6      21.879  -5.960  10.502  1.00  0.00
ATOM    104  N   ARG     7      23.625  -6.323   9.220  1.00  0.00
ATOM    105  H   ARG     7      24.027  -6.718   8.381  1.00  0.00
ATOM    106  CA  ARG     7      24.524  -5.597  10.160  1.00  0.00
ATOM    107  HA  ARG     7      24.626  -6.154  11.091  1.00  0.00
ATOM    108  CB  ARG     7      25.909  -5.444   9.528  1.00  0.00
ATOM    109  HB2 ARG     7      26.280  -6.423   9.224  1.00  0.00
ATOM    110  HB3 ARG     7      25.841  -4.795   8.655  1.00  0.00
ATOM    111  CG  ARG     7      26.870  -4.829  10.547  1.00  0.00
```

```
ATOM    112  HG2 ARG     7      26.482  -3.866  10.878  1.00  0.00
ATOM    113  HG3 ARG     7      26.967  -5.496  11.404  1.00  0.00
ATOM    114  CD  ARG     7      28.242  -4.630   9.899  1.00  0.00
ATOM    115  HD2 ARG     7      28.604  -5.571   9.484  1.00  0.00
ATOM    116  HD3 ARG     7      28.181  -3.884   9.107  1.00  0.00
ATOM    117  NE  ARG     7      29.212  -4.157  10.927  1.00  0.00
ATOM    118  HE  ARG     7      29.640  -4.796  11.583  1.00  0.00
ATOM    119  CZ  ARG     7      29.526  -2.892  10.994  1.00  0.00
ATOM    120  NH1 ARG     7      28.935  -2.119  11.863  1.00  0.00
ATOM    121 HH11 ARG     7      28.236  -2.503  12.482  1.00  0.00
ATOM    122 HH12 ARG     7      29.180  -1.141  11.913  1.00  0.00
ATOM    123  NH2 ARG     7      30.431  -2.401  10.192  1.00  0.00
ATOM    124 HH21 ARG     7      30.887  -3.003   9.521  1.00  0.00
ATOM    125 HH22 ARG     7      30.672  -1.422  10.246  1.00  0.00
ATOM    126  C   ARG     7      23.943  -4.213  10.455  1.00  0.00
ATOM    127  O   ARG     7      23.964  -3.744  11.576  1.00  0.00
ATOM    128  N   GLY     8      23.422  -3.554   9.456  1.00  0.00
ATOM    129  H   GLY     8      23.400  -3.937   8.522  1.00  0.00
ATOM    130  CA  GLY     8      22.840  -2.200   9.679  1.00  0.00
ATOM    131  HA2 GLY     8      23.616  -1.512  10.013  1.00  0.00
ATOM    132  HA3 GLY     8      22.398  -1.828   8.755  1.00  0.00
ATOM    133  C   GLY     8      21.753  -2.284  10.752  1.00  0.00
```

ATOM 134 O GLY 8 21.539 -1.355 11.505 1.00 0.00

ATOM 135 N ARG 9 21.062 -3.389 10.827 1.00 0.00

ATOM 136 H ARG 9 21.232 -4.165 10.203 1.00 0.00

ATOM 137 CA ARG 9 19.990 -3.529 11.853 1.00 0.00

ATOM 138 HA ARG 9 19.190 -2.814 11.664 1.00 0.00

ATOM 139 CB ARG 9 19.408 -4.942 11.792 1.00 0.00

ATOM 140 HB2 ARG 9 19.045 -5.144 10.784 1.00 0.00

ATOM 141 HB3 ARG 9 20.181 -5.666 12.050 1.00 0.00

ATOM 142 CG ARG 9 18.249 -5.058 12.785 1.00 0.00

ATOM 143 HG2 ARG 9 18.604 -4.820 13.788 1.00 0.00

ATOM 144 HG3 ARG 9 17.459 -4.362 12.504 1.00 0.00

ATOM 145 CD ARG 9 17.701 -6.487 12.765 1.00 0.00

ATOM 146 HD2 ARG 9 17.418 -6.769 11.751 1.00 0.00

ATOM 147 HD3 ARG 9 18.451 -7.185 13.139 1.00 0.00

ATOM 148 NE ARG 9 16.496 -6.569 13.638 1.00 0.00

ATOM 149 HE ARG 9 15.643 -6.081 13.408 1.00 0.00

ATOM 150 CZ ARG 9 16.529 -7.285 14.728 1.00 0.00

ATOM 151 NH1 ARG 9 17.003 -8.500 14.698 1.00 0.00

ATOM 152 HH11 ARG 9 17.344 -8.885 13.828 1.00 0.00

ATOM 153 HH12 ARG 9 17.027 -9.051 15.544 1.00 0.00

ATOM 154 NH2 ARG 9 16.088 -6.784 15.850 1.00 0.00

ATOM 155 HH21 ARG 9 15.723 -5.843 15.870 1.00 0.00

ATOM	156	HH22	ARG	9	16.115	-7.340	16.693	1.00	0.00
ATOM	157	C	ARG	9	20.580	-3.278	13.242	1.00	0.00
ATOM	158	O	ARG	9	19.931	-2.733	14.113	1.00	0.00
ATOM	159	N	GLN	10	21.806	-3.673	13.457	1.00	0.00
ATOM	160	H	GLN	10	22.352	-4.127	12.738	1.00	0.00
ATOM	161	CA	GLN	10	22.435	-3.457	14.790	1.00	0.00
ATOM	162	HA	GLN	10	21.920	-4.043	15.552	1.00	0.00
ATOM	163	CB	GLN	10	23.898	-3.901	14.739	1.00	0.00
ATOM	164	HB2	GLN	10	24.405	-3.391	13.920	1.00	0.00
ATOM	165	HB3	GLN	10	24.388	-3.651	15.680	1.00	0.00
ATOM	166	CG	GLN	10	23.965	-5.414	14.517	1.00	0.00
ATOM	167	HG2	GLN	10	23.385	-5.934	15.280	1.00	0.00
ATOM	168	HG3	GLN	10	23.578	-5.670	13.530	1.00	0.00
ATOM	169	CD	GLN	10	25.419	-5.879	14.607	1.00	0.00
ATOM	170	OE1	GLN	10	26.080	-6.131	13.510	1.00	0.00
ATOM	171	NE2	GLN	10	25.960	-6.015	15.686	1.00	0.00
ATOM	172	HE21	GLN	10	26.921	-6.323	15.730	1.00	0.00
ATOM	173	HE22	GLN	10	25.446	-5.820	16.533	1.00	0.00
ATOM	174	C	GLN	10	22.365	-1.972	15.152	1.00	0.00
ATOM	175	O	GLN	10	22.207	-1.611	16.301	1.00	0.00
ATOM	176	N	GLY	11	22.482	-1.109	14.181	1.00	0.00
ATOM	177	H	GLY	11	22.614	-1.403	13.224	1.00	0.00

ATOM	178	CA	GLY	11	22.423	0.352	14.470	1.00	0.00
ATOM	179	HA2	GLY	11	23.293	0.651	15.055	1.00	0.00
ATOM	180	HA3	GLY	11	22.401	0.915	13.537	1.00	0.00
ATOM	181	C	GLY	11	21.155	0.662	15.269	1.00	0.00
ATOM	182	O	GLY	11	21.148	1.517	16.131	1.00	0.00
ATOM	183	N	ASP	12	20.083	-0.027	14.988	1.00	0.00
ATOM	184	H	ASP	12	20.081	-0.739	14.271	1.00	0.00
ATOM	185	CA	ASP	12	18.818	0.230	15.733	1.00	0.00
ATOM	186	HA	ASP	12	18.008	-0.373	15.321	1.00	0.00
ATOM	187	CB	ASP	12	19.006	-0.144	17.204	1.00	0.00
ATOM	188	HB2	ASP	12	19.859	0.399	17.612	1.00	0.00
ATOM	189	HB3	ASP	12	18.108	0.117	17.763	1.00	0.00
ATOM	190	CG	ASP	12	19.258	-1.649	17.319	1.00	0.00
ATOM	191	OD1	ASP	12	19.020	-2.345	16.346	1.00	0.00
ATOM	192	OD2	ASP	12	19.685	-2.079	18.378	1.00	0.00
ATOM	193	C	ASP	12	18.454	1.712	15.626	1.00	0.00
ATOM	194	O	ASP	12	18.136	2.355	16.606	1.00	0.00
ATOM	195	N	SER	13	18.499	2.260	14.442	1.00	0.00
ATOM	196	H	SER	13	18.763	1.732	13.622	1.00	0.00
ATOM	197	CA	SER	13	18.155	3.700	14.275	1.00	0.00
ATOM	198	HA	SER	13	18.868	4.324	14.814	1.00	0.00
ATOM	199	CB	SER	13	18.208	4.068	12.791	1.00	0.00

ATOM	200	HB2	SER	13	17.446	3.507	12.249	1.00	0.00
ATOM	201	HB3	SER	13	18.025	5.136	12.674	1.00	0.00
ATOM	202	OG	SER	13	19.490	3.748	12.270	1.00	0.00
ATOM	203	HG	SER	13	19.524	3.978	11.339	1.00	0.00
ATOM	204	C	SER	13	16.745	3.951	14.813	1.00	0.00
ATOM	205	O	SER	13	16.472	4.972	15.413	1.00	0.00
ATOM	206	N	SER	14	15.848	3.027	14.603	1.00	0.00
ATOM	207	H	SER	14	16.070	2.175	14.106	1.00	0.00
ATOM	208	CA	SER	14	14.455	3.212	15.103	1.00	0.00
ATOM	209	HA	SER	14	14.458	3.794	16.024	1.00	0.00
ATOM	210	CB	SER	14	13.632	3.961	14.053	1.00	0.00
ATOM	211	HB2	SER	14	14.049	4.957	13.907	1.00	0.00
ATOM	212	HB3	SER	14	13.660	3.414	13.110	1.00	0.00
ATOM	213	OG	SER	14	12.287	4.071	14.497	1.00	0.00
ATOM	214	HG	SER	14	11.770	4.540	13.838	1.00	0.00
ATOM	215	C	SER	14	13.825	1.842	15.363	1.00	0.00
ATOM	216	O	SER	14	13.914	0.942	14.553	1.00	0.00
ATOM	217	N	SER	15	13.189	1.677	16.492	1.00	0.00
ATOM	218	H	SER	15	13.112	2.419	17.173	1.00	0.00
ATOM	219	CA	SER	15	12.556	0.365	16.803	1.00	0.00
ATOM	220	HA	SER	15	13.240	-0.451	16.568	1.00	0.00
ATOM	221	CB	SER	15	12.216	0.304	18.294	1.00	0.00

ATOM	222	HB2	SER	15	11.879	-0.701	18.550	1.00	0.00
ATOM	223	HB3	SER	15	13.102	0.549	18.880	1.00	0.00
ATOM	224	OG	SER	15	11.185	1.237	18.582	1.00	0.00
ATOM	225	HG	SER	15	10.972	1.199	19.517	1.00	0.00
ATOM	226	C	SER	15	11.276	0.204	15.980	1.00	0.00
ATOM	227	O	SER	15	10.856	-0.895	15.678	1.00	0.00
ATOM	228	N	CYX	16	10.653	1.292	15.613	1.00	0.00
ATOM	229	H	CYX	16	10.995	2.209	15.860	1.00	0.00
ATOM	230	CA	CYX	16	9.403	1.197	14.807	1.00	0.00
ATOM	231	HA	CYX	16	8.642	0.641	15.355	1.00	0.00
ATOM	232	CB	CYX	16	8.871	2.602	14.520	1.00	0.00
ATOM	233	HB2	CYX	16	8.894	3.193	15.435	1.00	0.00
ATOM	234	HB3	CYX	16	9.494	3.079	13.764	1.00	0.00
ATOM	235	SG	CYX	16	7.148	2.496	13.973	1.00	0.00
ATOM	236	C	CYX	16	9.704	0.486	13.486	1.00	0.00
ATOM	237	O	CYX	16	8.834	-0.100	12.873	1.00	0.00
ATOM	238	N	GLU	17	10.930	0.533	13.043	1.00	0.00
ATOM	239	H	GLU	17	11.658	1.018	13.547	1.00	0.00
ATOM	240	CA	GLU	17	11.286	-0.140	11.763	1.00	0.00
ATOM	241	HA	GLU	17	10.686	0.259	10.945	1.00	0.00
ATOM	242	CB	GLU	17	12.763	0.108	11.452	1.00	0.00
ATOM	243	HB2	GLU	17	13.354	-0.025	12.359	1.00	0.00

ATOM	244	HB3	GLU	17	13.099	-0.599	10.693	1.00	0.00
ATOM	245	CG	GLU	17	12.941	1.537	10.932	1.00	0.00
ATOM	246	HG2	GLU	17	12.315	1.684	10.052	1.00	0.00
ATOM	247	HG3	GLU	17	12.650	2.245	11.707	1.00	0.00
ATOM	248	CD	GLU	17	14.407	1.764	10.558	1.00	0.00
ATOM	249	OE1	GLU	17	15.178	0.826	10.666	1.00	0.00
ATOM	250	OE2	GLU	17	14.733	2.874	10.170	1.00	0.00
ATOM	251	C	GLU	17	11.036	-1.644	11.892	1.00	0.00
ATOM	252	O	GLU	17	10.420	-2.255	11.041	1.00	0.00
ATOM	253	N	ARG	18	11.509	-2.246	12.949	1.00	0.00
ATOM	254	H	ARG	18	12.021	-1.745	13.661	1.00	0.00
ATOM	255	CA	ARG	18	11.298	-3.711	13.130	1.00	0.00
ATOM	256	HA	ARG	18	11.879	-4.270	12.396	1.00	0.00
ATOM	257	CB	ARG	18	11.754	-4.124	14.531	1.00	0.00
ATOM	258	HB2	ARG	18	12.809	-3.880	14.658	1.00	0.00
ATOM	259	HB3	ARG	18	11.166	-3.591	15.278	1.00	0.00
ATOM	260	CG	ARG	18	11.556	-5.631	14.707	1.00	0.00
ATOM	261	HG2	ARG	18	10.508	-5.882	14.546	1.00	0.00
ATOM	262	HG3	ARG	18	12.172	-6.166	13.984	1.00	0.00
ATOM	263	CD	ARG	18	11.965	-6.037	16.125	1.00	0.00
ATOM	264	HD2	ARG	18	12.036	-7.122	16.201	1.00	0.00
ATOM	265	HD3	ARG	18	12.926	-5.592	16.382	1.00	0.00

ATOM	266	NE	ARG	18	10.941	-5.556	17.095	1.00	0.00
ATOM	267	HE	ARG	18	10.183	-4.955	16.806	1.00	0.00
ATOM	268	CZ	ARG	18	11.013	-5.909	18.350	1.00	0.00
ATOM	269	NH1	ARG	18	11.513	-5.087	19.232	1.00	0.00
ATOM	270	HH11	ARG	18	11.844	-4.179	18.940	1.00	0.00
ATOM	271	HH12	ARG	18	11.567	-5.364	20.202	1.00	0.00
ATOM	272	NH2	ARG	18	10.586	-7.085	18.721	1.00	0.00
ATOM	273	HH21	ARG	18	10.201	-7.719	18.035	1.00	0.00
ATOM	274	HH22	ARG	18	10.643	-7.357	19.692	1.00	0.00
ATOM	275	C	ARG	18	9.812	-4.036	12.962	1.00	0.00
ATOM	276	O	ARG	18	9.440	-5.162	12.695	1.00	0.00
ATOM	277	N	GLN	19	8.959	-3.061	13.119	1.00	0.00
ATOM	278	H	GLN	19	9.260	-2.124	13.343	1.00	0.00
ATOM	279	CA	GLN	19	7.500	-3.318	12.971	1.00	0.00
ATOM	280	HA	GLN	19	7.228	-4.250	13.467	1.00	0.00
ATOM	281	CB	GLN	19	6.713	-2.174	13.613	1.00	0.00
ATOM	282	HB2	GLN	19	7.110	-1.220	13.267	1.00	0.00
ATOM	283	HB3	GLN	19	5.663	-2.254	13.334	1.00	0.00
ATOM	284	CG	GLN	19	6.844	-2.258	15.135	1.00	0.00
ATOM	285	HG2	GLN	19	6.585	-3.259	15.481	1.00	0.00
ATOM	286	HG3	GLN	19	7.863	-2.021	15.441	1.00	0.00
ATOM	287	CD	GLN	19	5.892	-1.254	15.787	1.00	0.00

```
ATOM    288  OE1 GLN    19       5.413  -0.344  15.140  1.00  0.00
ATOM    289  NE2 GLN    19       5.595  -1.380  17.052  1.00  0.00
ATOM    290 HE21 GLN    19       4.967  -0.721  17.488  1.00  0.00
ATOM    291 HE22 GLN    19       5.995  -2.137  17.588  1.00  0.00
ATOM    292  C   GLN    19       7.144  -3.413  11.485  1.00  0.00
ATOM    293  O   GLN    19       6.113  -3.941  11.118  1.00  0.00
ATOM    294  N   VAL    20       7.988  -2.909  10.626  1.00  0.00
ATOM    295  H   VAL    20       8.847  -2.467  10.921  1.00  0.00
ATOM    296  CA  VAL    20       7.692  -2.975   9.167  1.00  0.00
ATOM    297  HA  VAL    20       6.654  -2.702   8.980  1.00  0.00
ATOM    298  CB  VAL    20       8.599  -1.998   8.414  1.00  0.00
ATOM    299  HB  VAL    20       8.607  -1.040   8.932  1.00  0.00
ATOM    300  CG1 VAL    20      10.022  -2.557   8.354  1.00  0.00
ATOM    301 HG11 VAL    20      10.015  -3.516   7.835  1.00  0.00
ATOM    302 HG12 VAL    20      10.665  -1.860   7.817  1.00  0.00
ATOM    303 HG13 VAL    20      10.402  -2.695   9.366  1.00  0.00
ATOM    304  CG2 VAL    20       8.069  -1.804   6.992  1.00  0.00
ATOM    305 HG21 VAL    20       7.057  -1.403   7.033  1.00  0.00
ATOM    306 HG22 VAL    20       8.714  -1.109   6.455  1.00  0.00
ATOM    307 HG23 VAL    20       8.058  -2.763   6.474  1.00  0.00
ATOM    308  C   VAL    20       7.941  -4.399   8.663  1.00  0.00
ATOM    309  O   VAL    20       7.286  -4.868   7.752  1.00  0.00
```

```
ATOM    310  N   ASP    21       8.881  -5.091   9.248  1.00  0.00
ATOM    311  H   ASP    21       9.430  -4.707  10.004  1.00  0.00
ATOM    312  CA  ASP    21       9.169  -6.483   8.803  1.00  0.00
ATOM    313  HA  ASP    21       9.582  -6.480   7.794  1.00  0.00
ATOM    314  CB  ASP    21      10.190  -7.119   9.746  1.00  0.00
ATOM    315  HB2 ASP    21       9.880  -6.962  10.779  1.00  0.00
ATOM    316  HB3 ASP    21      10.254  -8.189   9.545  1.00  0.00
ATOM    317  CG  ASP    21      11.561  -6.476   9.522  1.00  0.00
ATOM    318  OD1 ASP    21      11.680  -5.692   8.594  1.00  0.00
ATOM    319  OD2 ASP    21      12.466  -6.778  10.281  1.00  0.00
ATOM    320  C   ASP    21       7.877  -7.303   8.821  1.00  0.00
ATOM    321  O   ASP    21       7.695  -8.210   8.034  1.00  0.00
ATOM    322  N   ARG    22       6.979  -6.989   9.713  1.00  0.00
ATOM    323  H   ARG    22       7.124  -6.237  10.371  1.00  0.00
ATOM    324  CA  ARG    22       5.699  -7.750   9.780  1.00  0.00
ATOM    325  HA  ARG    22       5.886  -8.817   9.652  1.00  0.00
ATOM    326  CB  ARG    22       5.044  -7.528  11.146  1.00  0.00
ATOM    327  HB2 ARG    22       5.764  -7.747  11.935  1.00  0.00
ATOM    328  HB3 ARG    22       4.719  -6.492  11.229  1.00  0.00
ATOM    329  CG  ARG    22       3.836  -8.454  11.288  1.00  0.00
ATOM    330  HG2 ARG    22       3.162  -8.305  10.445  1.00  0.00
ATOM    331  HG3 ARG    22       4.172  -9.491  11.304  1.00  0.00
```

```
ATOM    332  CD  ARG    22       3.100  -8.136  12.592  1.00  0.00
ATOM    333  HD2 ARG    22       3.798  -8.137  13.429  1.00  0.00
ATOM    334  HD3 ARG    22       2.616  -7.162  12.524  1.00  0.00
ATOM    335  NE  ARG    22       2.057  -9.172  12.839  1.00  0.00
ATOM    336  HE  ARG    22       1.932  -9.590  13.750  1.00  0.00
ATOM    337  CZ  ARG    22       1.280  -9.559  11.864  1.00  0.00
ATOM    338  NH1 ARG    22       0.046  -9.136  11.806  1.00  0.00
ATOM    339 HH11 ARG    22      -0.304  -8.510  12.517  1.00  0.00
ATOM    340 HH12 ARG    22      -0.553  -9.438  11.050  1.00  0.00
ATOM    341  NH2 ARG    22       1.735 -10.369  10.948  1.00  0.00
ATOM    342 HH21 ARG    22       2.690 -10.696  10.996  1.00  0.00
ATOM    343 HH22 ARG    22       1.131 -10.666  10.195  1.00  0.00
ATOM    344  C   ARG    22       4.761  -7.263   8.677  1.00  0.00
ATOM    345  O   ARG    22       3.807  -7.926   8.321  1.00  0.00
ATOM    346  N   VAL    23       5.023  -6.107   8.131  1.00  0.00
ATOM    347  H   VAL    23       5.814  -5.551   8.423  1.00  0.00
ATOM    348  CA  VAL    23       4.147  -5.576   7.049  1.00  0.00
ATOM    349  HA  VAL    23       3.225  -5.177   7.470  1.00  0.00
ATOM    350  CB  VAL    23       4.875  -4.451   6.310  1.00  0.00
ATOM    351  HB  VAL    23       5.882  -4.778   6.050  1.00  0.00
ATOM    352  CG1 VAL    23       4.108  -4.100   5.033  1.00  0.00
ATOM    353 HG11 VAL    23       3.101  -3.773   5.292  1.00  0.00
```

```
ATOM    354 HG12 VAL    23       4.626  -3.299   4.506  1.00  0.00
ATOM    355 HG13 VAL    23       4.050  -4.979   4.390  1.00  0.00
ATOM    356  CG2 VAL    23       4.957  -3.218   7.212  1.00  0.00
ATOM    357 HG21 VAL    23       5.504  -3.469   8.121  1.00  0.00
ATOM    358 HG22 VAL    23       5.476  -2.417   6.686  1.00  0.00
ATOM    359 HG23 VAL    23       3.951  -2.890   7.472  1.00  0.00
ATOM    360  C   VAL    23       3.813  -6.699   6.066  1.00  0.00
ATOM    361  O   VAL    23       2.775  -7.324   6.151  1.00  0.00
ATOM    362  N   ASN    24       4.686  -6.959   5.134  1.00  0.00
ATOM    363  H   ASN    24       5.551  -6.444   5.059  1.00  0.00
ATOM    364  CA  ASN    24       4.420  -8.040   4.145  1.00  0.00
ATOM    365  HA  ASN    24       5.190  -8.046   3.374  1.00  0.00
ATOM    366  CB  ASN    24       4.431  -9.394   4.857  1.00  0.00
ATOM    367  HB2 ASN    24       3.662  -9.422   5.628  1.00  0.00
ATOM    368  HB3 ASN    24       4.258 -10.199   4.142  1.00  0.00
ATOM    369  CG  ASN    24       5.794  -9.612   5.518  1.00  0.00
ATOM    370  OD1 ASN    24       6.762  -8.965   5.168  1.00  0.00
ATOM    371  ND2 ASN    24       5.912 -10.502   6.465  1.00  0.00
ATOM    372 HD21 ASN    24       6.809 -10.651   6.905  1.00  0.00
ATOM    373 HD22 ASN    24       5.106 -11.038   6.753  1.00  0.00
ATOM    374  C   ASN    24       3.051  -7.812   3.499  1.00  0.00
ATOM    375  O   ASN    24       2.089  -8.486   3.807  1.00  0.00
```

ATOM	376	N	LEU	25	2.957	-6.867	2.605	1.00	0.00
ATOM	377	H	LEU	25	3.754	-6.305	2.343	1.00	0.00
ATOM	378	CA	LEU	25	1.651	-6.596	1.940	1.00	0.00
ATOM	379	HA	LEU	25	0.856	-7.167	2.420	1.00	0.00
ATOM	380	CB	LEU	25	1.321	-5.106	2.055	1.00	0.00
ATOM	381	HB2	LEU	25	2.213	-4.519	1.837	1.00	0.00
ATOM	382	HB3	LEU	25	0.536	-4.852	1.343	1.00	0.00
ATOM	383	CG	LEU	25	0.841	-4.797	3.475	1.00	0.00
ATOM	384	HG	LEU	25	1.481	-5.309	4.194	1.00	0.00
ATOM	385	CD1	LEU	25	0.904	-3.289	3.719	1.00	0.00
ATOM	386	HD11	LEU	25	0.264	-2.778	3.000	1.00	0.00
ATOM	387	HD12	LEU	25	0.562	-3.070	4.730	1.00	0.00
ATOM	388	HD13	LEU	25	1.931	-2.944	3.600	1.00	0.00
ATOM	389	CD2	LEU	25	-0.602	-5.281	3.642	1.00	0.00
ATOM	390	HD21	LEU	25	-0.647	-6.357	3.469	1.00	0.00
ATOM	391	HD22	LEU	25	-0.944	-5.062	4.653	1.00	0.00
ATOM	392	HD23	LEU	25	-1.242	-4.771	2.923	1.00	0.00
ATOM	393	C	LEU	25	1.743	-6.985	0.463	1.00	0.00
ATOM	394	O	LEU	25	1.715	-6.144	-0.414	1.00	0.00
ATOM	395	N	LYS	26	1.852	-8.255	0.181	1.00	0.00
ATOM	396	H	LYS	26	1.880	-8.956	0.908	1.00	0.00
ATOM	397	CA	LYS	26	1.948	-8.703	-1.237	1.00	0.00

ATOM 398 HA LYS 26 2.737 -8.158 -1.755 1.00 0.00
ATOM 399 CB LYS 26 2.280 -10.196 -1.275 1.00 0.00
ATOM 400 HB2 LYS 26 3.137 -10.395 -0.632 1.00 0.00
ATOM 401 HB3 LYS 26 1.422 -10.769 -0.924 1.00 0.00
ATOM 402 CG LYS 26 2.614 -10.607 -2.711 1.00 0.00
ATOM 403 HG2 LYS 26 2.047 -11.500 -2.975 1.00 0.00
ATOM 404 HG3 LYS 26 2.353 -9.797 -3.391 1.00 0.00
ATOM 405 CD LYS 26 4.111 -10.903 -2.822 1.00 0.00
ATOM 406 HD2 LYS 26 4.506 -10.448 -3.730 1.00 0.00
ATOM 407 HD3 LYS 26 4.628 -10.492 -1.955 1.00 0.00
ATOM 408 CE LYS 26 4.330 -12.417 -2.875 1.00 0.00
ATOM 409 HE2 LYS 26 4.645 -12.774 -1.894 1.00 0.00
ATOM 410 HE3 LYS 26 3.400 -12.910 -3.159 1.00 0.00
ATOM 411 NZ LYS 26 5.385 -12.732 -3.879 1.00 0.00
ATOM 412 HZ1 LYS 26 6.247 -12.277 -3.616 1.00 0.00
ATOM 413 HZ2 LYS 26 5.529 -13.731 -3.914 1.00 0.00
ATOM 414 HZ3 LYS 26 5.094 -12.403 -4.788 1.00 0.00
ATOM 415 C LYS 26 0.621 -8.458 -1.967 1.00 0.00
ATOM 416 O LYS 26 0.610 -8.104 -3.129 1.00 0.00
ATOM 417 N PRO 27 -0.524 -8.655 -1.285 1.00 0.00
ATOM 418 CD PRO 27 -0.633 -9.080 0.125 1.00 0.00
ATOM 419 HD2 PRO 27 0.056 -8.498 0.737 1.00 0.00

ATOM	420	HD3	PRO	27	-0.385	-10.139	0.208	1.00	0.00
ATOM	421	CG	PRO	27	-2.072	-8.801	0.452	1.00	0.00
ATOM	422	HG2	PRO	27	-2.165	-7.793	0.854	1.00	0.00
ATOM	423	HG3	PRO	27	-2.423	-9.521	1.192	1.00	0.00
ATOM	424	CB	PRO	27	-2.819	-8.947	-0.828	1.00	0.00
ATOM	425	HB2	PRO	27	-3.693	-8.298	-0.798	1.00	0.00
ATOM	426	HB3	PRO	27	-3.132	-9.968	-1.050	1.00	0.00
ATOM	427	CA	PRO	27	-1.845	-8.457	-1.898	1.00	0.00
ATOM	428	HA	PRO	27	-1.889	-9.045	-2.815	1.00	0.00
ATOM	429	C	PRO	27	-2.106	-6.983	-2.224	1.00	0.00
ATOM	430	O	PRO	27	-2.653	-6.656	-3.259	1.00	0.00
ATOM	431	N	CYX	28	-1.710	-6.090	-1.359	1.00	0.00
ATOM	432	H	CYX	28	-1.248	-6.348	-0.498	1.00	0.00
ATOM	433	CA	CYX	28	-1.931	-4.643	-1.637	1.00	0.00
ATOM	434	HA	CYX	28	-2.985	-4.454	-1.837	1.00	0.00
ATOM	435	CB	CYX	28	-1.508	-3.817	-0.420	1.00	0.00
ATOM	436	HB2	CYX	28	-0.847	-4.412	0.211	1.00	0.00
ATOM	437	HB3	CYX	28	-0.983	-2.922	-0.753	1.00	0.00
ATOM	438	SG	CYX	28	-2.968	-3.413	0.571	1.00	0.00
ATOM	439	C	CYX	28	-1.085	-4.245	-2.847	1.00	0.00
ATOM	440	O	CYX	28	-1.589	-3.760	-3.845	1.00	0.00
ATOM	441	N	GLU	29	0.201	-4.457	-2.774	1.00	0.00

```
ATOM    442  H   GLU    29       0.628  -4.863  -1.953  1.00  0.00
ATOM    443  CA  GLU    29       1.076  -4.106  -3.922  1.00  0.00
ATOM    444  HA  GLU    29       1.128  -3.024  -4.039  1.00  0.00
ATOM    445  CB  GLU    29       2.487  -4.639  -3.674  1.00  0.00
ATOM    446  HB2 GLU    29       2.726  -4.557  -2.614  1.00  0.00
ATOM    447  HB3 GLU    29       2.540  -5.685  -3.978  1.00  0.00
ATOM    448  CG  GLU    29       3.491  -3.822  -4.487  1.00  0.00
ATOM    449  HG2 GLU    29       3.217  -3.854  -5.542  1.00  0.00
ATOM    450  HG3 GLU    29       3.482  -2.789  -4.141  1.00  0.00
ATOM    451  CD  GLU    29       4.893  -4.407  -4.307  1.00  0.00
ATOM    452  OE1 GLU    29       5.024  -5.357  -3.551  1.00  0.00
ATOM    453  OE2 GLU    29       5.811  -3.898  -4.927  1.00  0.00
ATOM    454  C   GLU    29       0.507  -4.744  -5.189  1.00  0.00
ATOM    455  O   GLU    29       0.599  -4.198  -6.271  1.00  0.00
ATOM    456  N   GLN    30      -0.077  -5.904  -5.064  1.00  0.00
ATOM    457  H   GLN    30      -0.151  -6.367  -4.169  1.00  0.00
ATOM    458  CA  GLN    30      -0.649  -6.582  -6.258  1.00  0.00
ATOM    459  HA  GLN    30       0.084  -6.609  -7.065  1.00  0.00
ATOM    460  CB  GLN    30      -1.029  -8.019  -5.896  1.00  0.00
ATOM    461  HB2 GLN    30      -1.448  -8.043  -4.890  1.00  0.00
ATOM    462  HB3 GLN    30      -1.768  -8.390  -6.606  1.00  0.00
ATOM    463  CG  GLN    30       0.219  -8.905  -5.949  1.00  0.00
```

```
ATOM    464  HG2 GLN    30       0.539  -9.045  -6.982  1.00  0.00
ATOM    465  HG3 GLN    30       1.030  -8.454  -5.377  1.00  0.00
ATOM    466  CD  GLN    30      -0.101 -10.273  -5.347  1.00  0.00
ATOM    467  OE1 GLN    30      -1.198 -10.503  -4.877  1.00  0.00
ATOM    468  NE2 GLN    30       0.818 -11.200  -5.339  1.00  0.00
ATOM    469 HE21 GLN    30       0.614 -12.107  -4.943  1.00  0.00
ATOM    470 HE22 GLN    30       1.730 -11.007  -5.729  1.00  0.00
ATOM    471  C   GLN    30      -1.893  -5.827  -6.733  1.00  0.00
ATOM    472  O   GLN    30      -2.269  -5.904  -7.886  1.00  0.00
ATOM    473  N   HID    31      -2.535  -5.093  -5.864  1.00  0.00
ATOM    474  H   HID    31      -2.234  -5.019  -4.903  1.00  0.00
ATOM    475  CA  HID    31      -3.745  -4.337  -6.285  1.00  0.00
ATOM    476  HA  HID    31      -4.358  -4.945  -6.950  1.00  0.00
ATOM    477  CB  HID    31      -4.575  -3.974  -5.049  1.00  0.00
ATOM    478  HB2 HID    31      -5.248  -4.782  -4.762  1.00  0.00
ATOM    479  HB3 HID    31      -3.950  -3.704  -4.198  1.00  0.00
ATOM    480  CG  HID    31      -5.439  -2.777  -5.346  1.00  0.00
ATOM    481  ND1 HID    31      -6.510  -2.837  -6.224  1.00  0.00
ATOM    482  HD1 HID    31      -6.817  -3.649  -6.741  1.00  0.00
ATOM    483  CE1 HID    31      -7.070  -1.614  -6.265  1.00  0.00
ATOM    484  HE1 HID    31      -7.935  -1.484  -6.916  1.00  0.00
ATOM    485  NE2 HID    31      -6.435  -0.751  -5.467  1.00  0.00
```

```
ATOM    486  CD2 HID    31      -5.404  -1.483  -4.885  1.00  0.00
ATOM    487  HD2 HID    31      -4.725  -1.003  -4.180  1.00  0.00
ATOM    488  C   HID    31      -3.292  -3.069  -7.000  1.00  0.00
ATOM    489  O   HID    31      -4.025  -2.471  -7.763  1.00  0.00
ATOM    490  N   ILE    32      -2.078  -2.661  -6.759  1.00  0.00
ATOM    491  H   ILE    32      -1.468  -3.155  -6.123  1.00  0.00
ATOM    492  CA  ILE    32      -1.559  -1.435  -7.425  1.00  0.00
ATOM    493  HA  ILE    32      -2.395  -0.780  -7.669  1.00  0.00
ATOM    494  CB  ILE    32      -0.589  -0.701  -6.493  1.00  0.00
ATOM    495  HB  ILE    32       0.374  -0.579  -6.989  1.00  0.00
ATOM    496  CG2 ILE    32      -1.162   0.671  -6.151  1.00  0.00
ATOM    497 HG21 ILE    32      -2.124   0.549  -5.654  1.00  0.00
ATOM    498 HG22 ILE    32      -0.475   1.197  -5.488  1.00  0.00
ATOM    499 HG23 ILE    32      -1.296   1.247  -7.066  1.00  0.00
ATOM    500  CG1 ILE    32      -0.394  -1.504  -5.203  1.00  0.00
ATOM    501 HG12 ILE    32      -1.367  -1.747  -4.777  1.00  0.00
ATOM    502 HG13 ILE    32       0.144  -2.426  -5.428  1.00  0.00
ATOM    503  CD1 ILE    32       0.410  -0.677  -4.198  1.00  0.00
ATOM    504 HD11 ILE    32      -0.127   0.244  -3.972  1.00  0.00
ATOM    505 HD12 ILE    32       0.547  -1.251  -3.282  1.00  0.00
ATOM    506 HD13 ILE    32       1.384  -0.435  -4.623  1.00  0.00
ATOM    507  C   ILE    32      -0.831  -1.829  -8.709  1.00  0.00
```

ATOM	508	O	ILE	32	-1.104	-1.308	-9.772	1.00	0.00
ATOM	509	N	MET	33	0.088	-2.750	-8.623	1.00	0.00
ATOM	510	H	MET	33	0.321	-3.190	-7.744	1.00	0.00
ATOM	511	CA	MET	33	0.825	-3.179	-9.844	1.00	0.00
ATOM	512	HA	MET	33	1.337	-2.329	-10.295	1.00	0.00
ATOM	513	CB	MET	33	1.868	-4.233	-9.466	1.00	0.00
ATOM	514	HB2	MET	33	1.364	-5.132	-9.110	1.00	0.00
ATOM	515	HB3	MET	33	2.471	-4.478	-10.340	1.00	0.00
ATOM	516	CG	MET	33	2.772	-3.684	-8.361	1.00	0.00
ATOM	517	HG2	MET	33	3.172	-2.715	-8.659	1.00	0.00
ATOM	518	HG3	MET	33	2.201	-3.573	-7.439	1.00	0.00
ATOM	519	SD	MET	33	4.143	-4.831	-8.077	1.00	0.00
ATOM	520	CE	MET	33	5.274	-4.139	-9.308	1.00	0.00
ATOM	521	HE1	MET	33	5.483	-3.097	-9.066	1.00	0.00
ATOM	522	HE2	MET	33	6.206	-4.706	-9.305	1.00	0.00
ATOM	523	HE3	MET	33	4.817	-4.198	-10.296	1.00	0.00
ATOM	524	C	MET	33	-0.165	-3.774	-10.845	1.00	0.00
ATOM	525	O	MET	33	-0.071	-3.550	-12.034	1.00	0.00
ATOM	526	N	GLN	34	-1.115	-4.530	-10.370	1.00	0.00
ATOM	527	H	GLN	34	-1.198	-4.718	-9.381	1.00	0.00
ATOM	528	CA	GLN	34	-2.113	-5.143	-11.292	1.00	0.00
ATOM	529	HA	GLN	34	-1.609	-5.741	-12.052	1.00	0.00

ATOM	530	CB	GLN	34	-3.050	-6.053	-10.495	1.00	0.00
ATOM	531	HB2	GLN	34	-3.402	-5.526	-9.609	1.00	0.00
ATOM	532	HB3	GLN	34	-3.902	-6.330	-11.115	1.00	0.00
ATOM	533	CG	GLN	34	-2.297	-7.315	-10.071	1.00	0.00
ATOM	534	HG2	GLN	34	-2.016	-7.901	-10.946	1.00	0.00
ATOM	535	HG3	GLN	34	-1.401	-7.050	-9.509	1.00	0.00
ATOM	536	CD	GLN	34	-3.198	-8.171	-9.179	1.00	0.00
ATOM	537	OE1	GLN	34	-4.253	-7.735	-8.764	1.00	0.00
ATOM	538	NE2	GLN	34	-2.824	-9.381	-8.865	1.00	0.00
ATOM	539	HE21	GLN	34	-3.414	-9.952	-8.277	1.00	0.00
ATOM	540	HE22	GLN	34	-1.946	-9.742	-9.212	1.00	0.00
ATOM	541	C	GLN	34	-2.930	-4.044	-11.974	1.00	0.00
ATOM	542	O	GLN	34	-3.165	-4.085	-13.165	1.00	0.00
ATOM	543	N	ARG	35	-3.373	-3.066	-11.232	1.00	0.00
ATOM	544	H	ARG	35	-3.185	-3.024	-10.241	1.00	0.00
ATOM	545	CA	ARG	35	-4.182	-1.974	-11.846	1.00	0.00
ATOM	546	HA	ARG	35	-5.044	-2.389	-12.367	1.00	0.00
ATOM	547	CB	ARG	35	-4.680	-1.033	-10.749	1.00	0.00
ATOM	548	HB2	ARG	35	-3.899	-0.901	-10.000	1.00	0.00
ATOM	549	HB3	ARG	35	-4.931	-0.067	-11.185	1.00	0.00
ATOM	550	CG	ARG	35	-5.924	-1.634	-10.088	1.00	0.00
ATOM	551	HG2	ARG	35	-6.628	-1.952	-10.858	1.00	0.00

```
ATOM    552  HG3 ARG    35      -5.635  -2.494  -9.484  1.00  0.00
ATOM    553  CD  ARG    35      -6.585  -0.582  -9.195  1.00  0.00
ATOM    554  HD2 ARG    35      -6.995   0.224  -9.802  1.00  0.00
ATOM    555  HD3 ARG    35      -7.382  -1.035  -8.606  1.00  0.00
ATOM    556  NE  ARG    35      -5.570  -0.011  -8.266  1.00  0.00
ATOM    557  HE  ARG    35      -4.722  -0.511  -8.039  1.00  0.00
ATOM    558  CZ  ARG    35      -5.767   1.159  -7.723  1.00  0.00
ATOM    559  NH1 ARG    35      -6.282   2.128  -8.430  1.00  0.00
ATOM    560 HH11 ARG    35      -6.527   1.968  -9.397  1.00  0.00
ATOM    561 HH12 ARG    35      -6.433   3.032  -8.007  1.00  0.00
ATOM    562  NH2 ARG    35      -5.447   1.361  -6.475  1.00  0.00
ATOM    563 HH21 ARG    35      -5.047   0.609  -5.932  1.00  0.00
ATOM    564 HH22 ARG    35      -5.601   2.267  -6.058  1.00  0.00
ATOM    565  C   ARG    35      -3.324  -1.187 -12.840  1.00  0.00
ATOM    566  O   ARG    35      -3.682  -1.025 -13.989  1.00  0.00
ATOM    567  N   ILE    36      -2.199  -0.692 -12.406  1.00  0.00
ATOM    568  H   ILE    36      -1.896  -0.820 -11.451  1.00  0.00
ATOM    569  CA  ILE    36      -1.325   0.090 -13.326  1.00  0.00
ATOM    570  HA  ILE    36      -1.899   0.896 -13.782  1.00  0.00
ATOM    571  CB  ILE    36      -0.157   0.681 -12.534  1.00  0.00
ATOM    572  HB  ILE    36       0.477   1.265 -13.201  1.00  0.00
ATOM    573  CG2 ILE    36      -0.701   1.585 -11.425  1.00  0.00
```

```
ATOM    574 HG21 ILE    36      -1.334   1.001 -10.758  1.00  0.00
ATOM    575 HG22 ILE    36       0.130   2.007 -10.859  1.00  0.00
ATOM    576 HG23 ILE    36      -1.286   2.391 -11.867  1.00  0.00
ATOM    577  CG1 ILE    36       0.666  -0.453 -11.917  1.00  0.00
ATOM    578 HG12 ILE    36      -0.006  -1.211 -11.515  1.00  0.00
ATOM    579 HG13 ILE    36       1.300  -0.900 -12.684  1.00  0.00
ATOM    580  CD1 ILE    36       1.542   0.101 -10.791  1.00  0.00
ATOM    581 HD11 ILE    36       0.910   0.547 -10.024  1.00  0.00
ATOM    582 HD12 ILE    36       2.127  -0.708 -10.353  1.00  0.00
ATOM    583 HD13 ILE    36       2.215   0.858 -11.193  1.00  0.00
ATOM    584  C   ILE    36      -0.784  -0.820 -14.431  1.00  0.00
ATOM    585  O   ILE    36      -0.269  -0.358 -15.429  1.00  0.00
ATOM    586  N   MET    37      -0.896  -2.110 -14.265  1.00  0.00
ATOM    587  H   MET    37      -1.320  -2.508 -13.439  1.00  0.00
ATOM    588  CA  MET    37      -0.384  -3.039 -15.314  1.00  0.00
ATOM    589  HA  MET    37       0.267  -2.505 -16.006  1.00  0.00
ATOM    590  CB  MET    37       0.419  -4.163 -14.657  1.00  0.00
ATOM    591  HB2 MET    37      -0.148  -4.577 -13.823  1.00  0.00
ATOM    592  HB3 MET    37       0.614  -4.948 -15.389  1.00  0.00
ATOM    593  CG  MET    37       1.748  -3.605 -14.142  1.00  0.00
ATOM    594  HG2 MET    37       2.164  -2.911 -14.872  1.00  0.00
ATOM    595  HG3 MET    37       1.589  -3.086 -13.197  1.00  0.00
```

ATOM	596	SD	MET	37	2.913	-4.965	-13.883	1.00	0.00
ATOM	597	CE	MET	37	1.918	-5.894	-12.691	1.00	0.00
ATOM	598	HE1	MET	37	0.971	-6.177	-13.150	1.00	0.00
ATOM	599	HE2	MET	37	2.457	-6.793	-12.389	1.00	0.00
ATOM	600	HE3	MET	37	1.727	-5.274	-11.815	1.00	0.00
ATOM	601	C	MET	37	-1.562	-3.635	-16.086	1.00	0.00
ATOM	602	O	MET	37	-1.411	-4.115	-17.193	1.00	0.00
ATOM	603	N	GLY	38	-2.735	-3.610	-15.515	1.00	0.00
ATOM	604	H	GLY	38	-2.869	-3.214	-14.596	1.00	0.00
ATOM	605	CA	GLY	38	-3.919	-4.176	-16.220	1.00	0.00
ATOM	606	HA2	GLY	38	-3.710	-5.197	-16.542	1.00	0.00
ATOM	607	HA3	GLY	38	-4.784	-4.175	-15.558	1.00	0.00
ATOM	608	C	GLY	38	-4.231	-3.320	-17.450	1.00	0.00
ATOM	609	O	GLY	38	-4.810	-3.786	-18.412	1.00	0.00
ATOM	610	N	GLU	39	-3.852	-2.072	-17.425	1.00	0.00
ATOM	611	H	GLU	39	-3.372	-1.679	-16.628	1.00	0.00
ATOM	612	CA	GLU	39	-4.126	-1.187	-18.592	1.00	0.00
ATOM	613	HA	GLU	39	-5.194	-1.169	-18.810	1.00	0.00
ATOM	614	CB	GLU	39	-3.667	0.237	-18.272	1.00	0.00
ATOM	615	HB2	GLU	39	-4.128	0.567	-17.341	1.00	0.00
ATOM	616	HB3	GLU	39	-2.582	0.254	-18.166	1.00	0.00
ATOM	617	CG	GLU	39	-4.083	1.174	-19.407	1.00	0.00

ATOM	618	HG2	GLU	39	-3.706	0.787	-20.354	1.00	0.00
ATOM	619	HG3	GLU	39	-5.170	1.236	-19.447	1.00	0.00
ATOM	620	CD	GLU	39	-3.502	2.567	-19.158	1.00	0.00
ATOM	621	OE1	GLU	39	-2.791	2.724	-18.178	1.00	0.00
ATOM	622	OE2	GLU	39	-3.777	3.453	-19.950	1.00	0.00
ATOM	623	C	GLU	39	-3.368	-1.706	-19.815	1.00	0.00
ATOM	624	O	GLU	39	-3.752	-1.467	-20.943	1.00	0.00
ATOM	625	N	GLN	40	-2.292	-2.413	-19.602	1.00	0.00
ATOM	626	H	GLN	40	-1.964	-2.615	-18.668	1.00	0.00
ATOM	627	CA	GLN	40	-1.512	-2.946	-20.754	1.00	0.00
ATOM	628	HA	GLN	40	-1.238	-2.138	-21.432	1.00	0.00
ATOM	629	CB	GLN	40	-0.232	-3.609	-20.241	1.00	0.00
ATOM	630	HB2	GLN	40	-0.481	-4.319	-19.452	1.00	0.00
ATOM	631	HB3	GLN	40	0.259	-4.136	-21.060	1.00	0.00
ATOM	632	CG	GLN	40	0.710	-2.539	-19.686	1.00	0.00
ATOM	633	HG2	GLN	40	0.967	-1.820	-20.464	1.00	0.00
ATOM	634	HG3	GLN	40	0.240	-2.019	-18.852	1.00	0.00
ATOM	635	CD	GLN	40	1.996	-3.200	-19.187	1.00	0.00
ATOM	636	OE1	GLN	40	2.058	-4.406	-19.048	1.00	0.00
ATOM	637	NE2	GLN	40	3.034	-2.458	-18.911	1.00	0.00
ATOM	638	HE21	GLN	40	3.886	-2.890	-18.581	1.00	0.00
ATOM	639	HE22	GLN	40	2.981	-1.457	-19.029	1.00	0.00

ATOM	640	C	GLN	40	-2.356	-3.977	-21.505	1.00	0.00
ATOM	641	O	GLN	40	-2.253	-4.122	-22.706	1.00	0.00
ATOM	642	N	GLU	41	-3.191	-4.695	-20.804	1.00	0.00
ATOM	643	H	GLU	41	-3.280	-4.583	-19.804	1.00	0.00
ATOM	644	CA	GLU	41	-4.043	-5.715	-21.479	1.00	0.00
ATOM	645	HA	GLU	41	-3.421	-6.486	-21.935	1.00	0.00
ATOM	646	CB	GLU	41	-4.962	-6.374	-20.448	1.00	0.00
ATOM	647	HB2	GLU	41	-5.573	-5.612	-19.966	1.00	0.00
ATOM	648	HB3	GLU	41	-5.609	-7.096	-20.947	1.00	0.00
ATOM	649	CG	GLU	41	-4.116	-7.091	-19.394	1.00	0.00
ATOM	650	HG2	GLU	41	-3.482	-7.834	-19.879	1.00	0.00
ATOM	651	HG3	GLU	41	-3.491	-6.365	-18.874	1.00	0.00
ATOM	652	CD	GLU	41	-5.036	-7.785	-18.388	1.00	0.00
ATOM	653	OE1	GLU	41	-6.231	-7.552	-18.450	1.00	0.00
ATOM	654	OE2	GLU	41	-4.529	-8.538	-17.574	1.00	0.00
ATOM	655	C	GLU	41	-4.888	-5.038	-22.560	1.00	0.00
ATOM	656	O	GLU	41	-5.391	-3.949	-22.374	1.00	0.00
ATOM	657	N	GLN	42	-5.048	-5.676	-23.687	1.00	0.00
ATOM	658	H	GLN	42	-4.631	-6.582	-23.850	1.00	0.00
ATOM	659	CA	GLN	42	-5.861	-5.067	-24.776	1.00	0.00
ATOM	660	HA	GLN	42	-5.461	-4.090	-25.046	1.00	0.00
ATOM	661	CB	GLN	42	-5.818	-5.971	-26.010	1.00	0.00

```
ATOM    662  HB2 GLN    42      -6.163  -6.970 -25.742  1.00  0.00
ATOM    663  HB3 GLN    42      -6.464  -5.560 -26.785  1.00  0.00
ATOM    664  CG  GLN    42      -4.383  -6.051 -26.533  1.00  0.00
ATOM    665  HG2 GLN    42      -3.729  -6.493 -25.781  1.00  0.00
ATOM    666  HG3 GLN    42      -4.345  -6.650 -27.443  1.00  0.00
ATOM    667  CD  GLN    42      -3.878  -4.642 -26.853  1.00  0.00
ATOM    668  OE1 GLN    42      -4.529  -3.895 -27.555  1.00  0.00
ATOM    669  NE2 GLN    42      -2.736  -4.245 -26.363  1.00  0.00
ATOM    670 HE21 GLN    42      -2.396  -3.317 -26.570  1.00  0.00
ATOM    671 HE22 GLN    42      -2.196  -4.868 -25.779  1.00  0.00
ATOM    672  C   GLN    42      -7.308  -4.910 -24.306  1.00  0.00
ATOM    673  O   GLN    42      -7.979  -3.954 -24.638  1.00  0.00
ATOM    674  N   TYR    43      -7.795  -5.843 -23.533  1.00  0.00
ATOM    675  H   TYR    43      -7.243  -6.641 -23.251  1.00  0.00
ATOM    676  CA  TYR    43      -9.199  -5.746 -23.042  1.00  0.00
ATOM    677  HA  TYR    43      -9.683  -4.861 -23.455  1.00  0.00
ATOM    678  CB  TYR    43      -9.983  -6.984 -23.485  1.00  0.00
ATOM    679  HB2 TYR    43      -9.521  -7.876 -23.061  1.00  0.00
ATOM    680  HB3 TYR    43     -11.013  -6.909 -23.137  1.00  0.00
ATOM    681  CG  TYR    43      -9.967  -7.077 -24.992  1.00  0.00
ATOM    682  CD1 TYR    43     -10.842  -6.290 -25.751  1.00  0.00
ATOM    683  HD1 TYR    43     -11.533  -5.610 -25.255  1.00  0.00
```

```
ATOM    684  CE1 TYR    43     -10.828  -6.378 -27.148  1.00  0.00
ATOM    685  HE1 TYR    43     -11.509  -5.766 -27.739  1.00  0.00
ATOM    686  CZ  TYR    43      -9.941  -7.253 -27.786  1.00  0.00
ATOM    687  OH  TYR    43      -9.928  -7.339 -29.164  1.00  0.00
ATOM    688  HH  TYR    43     -10.566  -6.757 -29.582  1.00  0.00
ATOM    689  CE2 TYR    43      -9.066  -8.039 -27.027  1.00  0.00
ATOM    690  HE2 TYR    43      -8.374  -8.720 -27.523  1.00  0.00
ATOM    691  CD2 TYR    43      -9.080  -7.951 -25.631  1.00  0.00
ATOM    692  HD2 TYR    43      -8.399  -8.564 -25.040  1.00  0.00
ATOM    693  C   TYR    43      -9.199  -5.665 -21.514  1.00  0.00
ATOM    694  O   TYR    43      -8.252  -6.057 -20.862  1.00  0.00
ATOM    695  N   ASP    44     -10.255  -5.159 -20.938  1.00  0.00
ATOM    696  H   ASP    44     -11.046  -4.832 -21.473  1.00  0.00
ATOM    697  CA  ASP    44     -10.314  -5.053 -19.453  1.00  0.00
ATOM    698  HA  ASP    44     -10.320  -6.046 -19.004  1.00  0.00
ATOM    699  CB  ASP    44      -9.086  -4.294 -18.944  1.00  0.00
ATOM    700  HB2 ASP    44      -9.211  -4.070 -17.885  1.00  0.00
ATOM    701  HB3 ASP    44      -8.195  -4.907 -19.082  1.00  0.00
ATOM    702  CG  ASP    44      -8.933  -2.988 -19.727  1.00  0.00
ATOM    703  OD1 ASP    44      -9.649  -2.815 -20.699  1.00  0.00
ATOM    704  OD2 ASP    44      -8.100  -2.185 -19.340  1.00  0.00
ATOM    705  C   ASP    44     -11.583  -4.301 -19.046  1.00  0.00
```

```
ATOM    706  O   ASP    44     -12.290  -4.698 -18.142  1.00  0.00
ATOM    707  N   SER    45     -11.878  -3.215 -19.708  1.00  0.00
ATOM    708  H   SER    45     -11.295  -2.877 -20.460  1.00  0.00
ATOM    709  CA  SER    45     -13.102  -2.440 -19.360  1.00  0.00
ATOM    710  HA  SER    45     -12.980  -1.954 -18.392  1.00  0.00
ATOM    711  CB  SER    45     -13.341  -1.364 -20.421  1.00  0.00
ATOM    712  HB2 SER    45     -14.147  -0.706 -20.097  1.00  0.00
ATOM    713  HB3 SER    45     -12.430  -0.782 -20.559  1.00  0.00
ATOM    714  OG  SER    45     -13.699  -1.981 -21.649  1.00  0.00
ATOM    715  HG  SER    45     -13.849  -1.306 -22.315  1.00  0.00
ATOM    716  C   SER    45     -14.305  -3.382 -19.308  1.00  0.00
ATOM    717  O   SER    45     -15.203  -3.215 -18.507  1.00  0.00
ATOM    718  N   TYR    46     -14.332  -4.372 -20.159  1.00  0.00
ATOM    719  H   TYR    46     -13.588  -4.518 -20.827  1.00  0.00
ATOM    720  CA  TYR    46     -15.478  -5.323 -20.160  1.00  0.00
ATOM    721  HA  TYR    46     -16.411  -4.791 -20.346  1.00  0.00
ATOM    722  CB  TYR    46     -15.278  -6.361 -21.266  1.00  0.00
ATOM    723  HB2 TYR    46     -14.312  -6.851 -21.138  1.00  0.00
ATOM    724  HB3 TYR    46     -16.072  -7.106 -21.213  1.00  0.00
ATOM    725  CG  TYR    46     -15.320  -5.676 -22.611  1.00  0.00
ATOM    726  CD1 TYR    46     -16.512  -5.102 -23.067  1.00  0.00
ATOM    727  HD1 TYR    46     -17.410  -5.151 -22.451  1.00  0.00
```

ATOM 728 CE1 TYR 46 -16.552 -4.466 -24.314 1.00 0.00
ATOM 729 HE1 TYR 46 -17.480 -4.020 -24.669 1.00 0.00
ATOM 730 CZ TYR 46 -15.398 -4.403 -25.104 1.00 0.00
ATOM 731 OH TYR 46 -15.437 -3.777 -26.333 1.00 0.00
ATOM 732 HH TYR 46 -16.303 -3.422 -26.546 1.00 0.00
ATOM 733 CE2 TYR 46 -14.205 -4.976 -24.648 1.00 0.00
ATOM 734 HE2 TYR 46 -13.307 -4.927 -25.264 1.00 0.00
ATOM 735 CD2 TYR 46 -14.165 -5.613 -23.401 1.00 0.00
ATOM 736 HD2 TYR 46 -13.236 -6.059 -23.046 1.00 0.00
ATOM 737 C TYR 46 -15.555 -6.031 -18.805 1.00 0.00
ATOM 738 O TYR 46 -16.620 -6.210 -18.248 1.00 0.00
ATOM 739 N ASP 47 -14.435 -6.436 -18.271 1.00 0.00
ATOM 740 H ASP 47 -13.545 -6.291 -18.727 1.00 0.00
ATOM 741 CA ASP 47 -14.448 -7.132 -16.954 1.00 0.00
ATOM 742 HA ASP 47 -15.459 -7.456 -16.707 1.00 0.00
ATOM 743 CB ASP 47 -13.543 -8.364 -17.019 1.00 0.00
ATOM 744 HB2 ASP 47 -12.549 -8.072 -17.359 1.00 0.00
ATOM 745 HB3 ASP 47 -13.470 -8.813 -16.028 1.00 0.00
ATOM 746 CG ASP 47 -14.137 -9.381 -17.996 1.00 0.00
ATOM 747 OD1 ASP 47 -15.297 -9.232 -18.343 1.00 0.00
ATOM 748 OD2 ASP 47 -13.421 -10.290 -18.383 1.00 0.00
ATOM 749 C ASP 47 -13.936 -6.182 -15.869 1.00 0.00

ATOM	750	O	ASP	47	-12.813	-5.718	-15.916	1.00	0.00
ATOM	751	N	ILE	48	-14.749	-5.887	-14.892	1.00	0.00
ATOM	752	H	ILE	48	-15.683	-6.269	-14.845	1.00	0.00
ATOM	753	CA	ILE	48	-14.307	-4.966	-13.807	1.00	0.00
ATOM	754	HA	ILE	48	-14.059	-3.994	-14.233	1.00	0.00
ATOM	755	CB	ILE	48	-15.435	-4.803	-12.786	1.00	0.00
ATOM	756	HB	ILE	48	-15.671	-5.773	-12.347	1.00	0.00
ATOM	757	CG2	ILE	48	-14.990	-3.840	-11.684	1.00	0.00
ATOM	758	HG21	ILE	48	-14.753	-2.871	-12.122	1.00	0.00
ATOM	759	HG22	ILE	48	-15.793	-3.724	-10.956	1.00	0.00
ATOM	760	HG23	ILE	48	-14.105	-4.240	-11.187	1.00	0.00
ATOM	761	CG1	ILE	48	-16.677	-4.242	-13.482	1.00	0.00
ATOM	762	HG12	ILE	48	-16.477	-3.225	-13.819	1.00	0.00
ATOM	763	HG13	ILE	48	-16.926	-4.866	-14.341	1.00	0.00
ATOM	764	CD1	ILE	48	-17.851	-4.233	-12.501	1.00	0.00
ATOM	765	HD11	ILE	48	-17.603	-3.609	-11.642	1.00	0.00
ATOM	766	HD12	ILE	48	-18.736	-3.834	-12.996	1.00	0.00
ATOM	767	HD13	ILE	48	-18.051	-5.251	-12.164	1.00	0.00
ATOM	768	C	ILE	48	-13.074	-5.550	-13.116	1.00	0.00
ATOM	769	O	ILE	48	-12.119	-4.854	-12.836	1.00	0.00
ATOM	770	N	ARG	49	-13.087	-6.826	-12.837	1.00	0.00
ATOM	771	H	ARG	49	-13.877	-7.412	-13.067	1.00	0.00

ATOM 772 CA ARG 49 -11.916 -7.453 -12.164 1.00 0.00

ATOM 773 HA ARG 49 -11.056 -6.784 -12.199 1.00 0.00

ATOM 774 CB ARG 49 -12.258 -7.733 -10.699 1.00 0.00

ATOM 775 HB2 ARG 49 -13.143 -8.368 -10.646 1.00 0.00

ATOM 776 HB3 ARG 49 -11.420 -8.239 -10.220 1.00 0.00

ATOM 777 CG ARG 49 -12.535 -6.411 -9.979 1.00 0.00

ATOM 778 HG2 ARG 49 -13.293 -5.851 -10.526 1.00 0.00

ATOM 779 HG3 ARG 49 -12.891 -6.615 -8.969 1.00 0.00

ATOM 780 CD ARG 49 -11.246 -5.589 -9.909 1.00 0.00

ATOM 781 HD2 ARG 49 -10.449 -6.175 -9.449 1.00 0.00

ATOM 782 HD3 ARG 49 -10.939 -5.282 -10.909 1.00 0.00

ATOM 783 NE ARG 49 -11.479 -4.370 -9.085 1.00 0.00

ATOM 784 HE ARG 49 -12.390 -4.156 -8.706 1.00 0.00

ATOM 785 CZ ARG 49 -10.494 -3.551 -8.838 1.00 0.00

ATOM 786 NH1 ARG 49 -9.264 -3.922 -9.066 1.00 0.00

ATOM 787 HH11 ARG 49 -9.078 -4.844 -9.434 1.00 0.00

ATOM 788 HH12 ARG 49 -8.504 -3.286 -8.874 1.00 0.00

ATOM 789 NH2 ARG 49 -10.739 -2.360 -8.363 1.00 0.00

ATOM 790 HH21 ARG 49 -11.692 -2.076 -8.188 1.00 0.00

ATOM 791 HH22 ARG 49 -9.974 -1.729 -8.173 1.00 0.00

ATOM 792 C ARG 49 -11.568 -8.768 -12.866 1.00 0.00

ATOM 793 O ARG 49 -12.435 -9.486 -13.323 1.00 0.00

ATOM	794	N	SER	50	-10.306	-9.087	-12.957	1.00	0.00
ATOM	795	H	SER	50	-9.580	-8.495	-12.581	1.00	0.00
ATOM	796	CA	SER	50	-9.905	-10.354	-13.630	1.00	0.00
ATOM	797	HA	SER	50	-10.156	-10.316	-14.690	1.00	0.00
ATOM	798	CB	SER	50	-8.393	-10.547	-13.492	1.00	0.00
ATOM	799	HB2	SER	50	-8.136	-10.667	-12.440	1.00	0.00
ATOM	800	HB3	SER	50	-8.087	-11.437	-14.044	1.00	0.00
ATOM	801	OG	SER	50	-7.722	-9.412	-14.016	1.00	0.00
ATOM	802	HG	SER	50	-6.773	-9.533	-13.930	1.00	0.00
ATOM	803	C	SER	50	-10.632	-11.532	-12.977	1.00	0.00
ATOM	804	O	SER	50	-11.074	-12.447	-13.644	1.00	0.00
ATOM	805	N	THR	51	-10.759	-11.517	-11.679	1.00	0.00
ATOM	806	H	THR	51	-10.394	-10.761	-11.119	1.00	0.00
ATOM	807	CA	THR	51	-11.457	-12.636	-10.986	1.00	0.00
ATOM	808	HA	THR	51	-12.155	-13.128	-11.663	1.00	0.00
ATOM	809	CB	THR	51	-10.428	-13.665	-10.514	1.00	0.00
ATOM	810	HB	THR	51	-9.765	-13.921	-11.341	1.00	0.00
ATOM	811	CG2	THR	51	-9.607	-13.080	-9.365	1.00	0.00
ATOM	812	HG21	THR	51	-10.269	-12.825	-8.538	1.00	0.00
ATOM	813	HG22	THR	51	-8.874	-13.815	-9.030	1.00	0.00
ATOM	814	HG23	THR	51	-9.091	-12.183	-9.707	1.00	0.00
ATOM	815	OG1	THR	51	-11.099	-14.837	-10.071	1.00	0.00

```
ATOM    816  HG1 THR    51     -10.453 -15.483  -9.775  1.00  0.00
ATOM    817  C   THR    51     -12.223 -12.091  -9.779  1.00  0.00
ATOM    818  O   THR    51     -11.897 -11.050  -9.244  1.00  0.00
ATOM    819  N   ARG    52     -13.239 -12.786  -9.346  1.00  0.00
ATOM    820  H   ARG    52     -13.515 -13.653  -9.785  1.00  0.00
ATOM    821  CA  ARG    52     -14.025 -12.306  -8.174  1.00  0.00
ATOM    822  HA  ARG    52     -14.439 -11.319  -8.376  1.00  0.00
ATOM    823  CB  ARG    52     -15.178 -13.274  -7.902  1.00  0.00
ATOM    824  HB2 ARG    52     -14.784 -14.283  -7.773  1.00  0.00
ATOM    825  HB3 ARG    52     -15.702 -12.971  -6.996  1.00  0.00
ATOM    826  CG  ARG    52     -16.149 -13.254  -9.084  1.00  0.00
ATOM    827  HG2 ARG    52     -16.465 -12.229  -9.278  1.00  0.00
ATOM    828  HG3 ARG    52     -15.654 -13.656  -9.969  1.00  0.00
ATOM    829  CD  ARG    52     -17.374 -14.111  -8.752  1.00  0.00
ATOM    830  HD2 ARG    52     -17.906 -13.695  -7.897  1.00  0.00
ATOM    831  HD3 ARG    52     -18.045 -14.159  -9.609  1.00  0.00
ATOM    832  NE  ARG    52     -16.934 -15.493  -8.413  1.00  0.00
ATOM    833  HE  ARG    52     -16.017 -15.678  -8.032  1.00  0.00
ATOM    834  CZ  ARG    52     -17.742 -16.500  -8.607  1.00  0.00
ATOM    835  NH1 ARG    52     -18.146 -17.217  -7.594  1.00  0.00
ATOM    836 HH11 ARG    52     -17.831 -16.991  -6.661  1.00  0.00
ATOM    837 HH12 ARG    52     -18.771 -17.996  -7.748  1.00  0.00
```

ATOM 838 NH2 ARG 52 -18.145 -16.789 -9.813 1.00 0.00

ATOM 839 HH21 ARG 52 -17.830 -16.232 -10.594 1.00 0.00

ATOM 840 HH22 ARG 52 -18.770 -17.569 -9.960 1.00 0.00

ATOM 841 C ARG 52 -13.118 -12.237 -6.944 1.00 0.00

ATOM 842 O ARG 52 -13.281 -11.389 -6.089 1.00 0.00

ATOM 843 N SER 53 -12.164 -13.121 -6.847 1.00 0.00

ATOM 844 H SER 53 -12.024 -13.829 -7.554 1.00 0.00

ATOM 845 CA SER 53 -11.248 -13.106 -5.672 1.00 0.00

ATOM 846 HA SER 53 -11.811 -13.259 -4.752 1.00 0.00

ATOM 847 CB SER 53 -10.222 -14.233 -5.809 1.00 0.00

ATOM 848 HB2 SER 53 -9.662 -14.332 -4.879 1.00 0.00

ATOM 849 HB3 SER 53 -10.736 -15.170 -6.024 1.00 0.00

ATOM 850 OG SER 53 -9.327 -13.931 -6.870 1.00 0.00

ATOM 851 HG SER 53 -8.683 -14.638 -6.956 1.00 0.00

ATOM 852 C SER 53 -10.524 -11.760 -5.607 1.00 0.00

ATOM 853 O SER 53 -10.025 -11.362 -4.572 1.00 0.00

ATOM 854 N SER 54 -10.460 -11.055 -6.703 1.00 0.00

ATOM 855 H SER 54 -10.872 -11.380 -7.566 1.00 0.00

ATOM 856 CA SER 54 -9.767 -9.736 -6.703 1.00 0.00

ATOM 857 HA SER 54 -8.700 -9.870 -6.523 1.00 0.00

ATOM 858 CB SER 54 -9.953 -9.061 -8.063 1.00 0.00

ATOM 859 HB2 SER 54 -9.634 -9.741 -8.853 1.00 0.00

ATOM	860	HB3	SER	54	-11.004	-8.809	-8.203	1.00	0.00
ATOM	861	OG	SER	54	-9.172	-7.877	-8.115	1.00	0.00
ATOM	862	HG	SER	54	-9.289	-7.454	-8.969	1.00	0.00
ATOM	863	C	SER	54	-10.361	-8.848	-5.608	1.00	0.00
ATOM	864	O	SER	54	-9.678	-8.037	-5.015	1.00	0.00
ATOM	865	N	ASP	55	-11.628	-8.993	-5.336	1.00	0.00
ATOM	866	H	ASP	55	-12.202	-9.664	-5.826	1.00	0.00
ATOM	867	CA	ASP	55	-12.264	-8.156	-4.279	1.00	0.00
ATOM	868	HA	ASP	55	-12.162	-7.099	-4.522	1.00	0.00
ATOM	869	CB	ASP	55	-13.753	-8.496	-4.187	1.00	0.00
ATOM	870	HB2	ASP	55	-13.873	-9.573	-4.068	1.00	0.00
ATOM	871	HB3	ASP	55	-14.190	-7.986	-3.329	1.00	0.00
ATOM	872	CG	ASP	55	-14.461	-8.042	-5.465	1.00	0.00
ATOM	873	OD1	ASP	55	-13.848	-7.319	-6.233	1.00	0.00
ATOM	874	OD2	ASP	55	-15.604	-8.425	-5.654	1.00	0.00
ATOM	875	C	ASP	55	-11.593	-8.435	-2.933	1.00	0.00
ATOM	876	O	ASP	55	-11.591	-7.605	-2.046	1.00	0.00
ATOM	877	N	GLN	56	-11.022	-9.598	-2.774	1.00	0.00
ATOM	878	H	GLN	56	-11.019	-10.293	-3.507	1.00	0.00
ATOM	879	CA	GLN	56	-10.351	-9.928	-1.485	1.00	0.00
ATOM	880	HA	GLN	56	-11.053	-9.832	-0.657	1.00	0.00
ATOM	881	CB	GLN	56	-9.843	-11.370	-1.530	1.00	0.00

ATOM	882	HB2	GLN	56	-9.125	-11.478	-2.344	1.00	0.00
ATOM	883	HB3	GLN	56	-9.358	-11.615	-0.585	1.00	0.00
ATOM	884	CG	GLN	56	-11.020	-12.320	-1.760	1.00	0.00
ATOM	885	HG2	GLN	56	-11.860	-12.044	-1.123	1.00	0.00
ATOM	886	HG3	GLN	56	-11.331	-12.290	-2.804	1.00	0.00
ATOM	887	CD	GLN	56	-10.596	-13.750	-1.418	1.00	0.00
ATOM	888	OE1	GLN	56	-11.379	-14.521	-0.901	1.00	0.00
ATOM	889	NE2	GLN	56	-9.380	-14.139	-1.688	1.00	0.00
ATOM	890	HE21	GLN	56	-9.093	-15.082	-1.465	1.00	0.00
ATOM	891	HE22	GLN	56	-8.731	-13.496	-2.119	1.00	0.00
ATOM	892	C	GLN	56	-9.171	-8.980	-1.264	1.00	0.00
ATOM	893	O	GLN	56	-8.868	-8.600	-0.151	1.00	0.00
ATOM	894	N	GLN	57	-8.503	-8.595	-2.317	1.00	0.00
ATOM	895	H	GLN	57	-8.749	-8.908	-3.245	1.00	0.00
ATOM	896	CA	GLN	57	-7.342	-7.673	-2.167	1.00	0.00
ATOM	897	HA	GLN	57	-6.496	-8.199	-1.723	1.00	0.00
ATOM	898	CB	GLN	57	-6.925	-7.150	-3.543	1.00	0.00
ATOM	899	HB2	GLN	57	-7.771	-6.653	-4.016	1.00	0.00
ATOM	900	HB3	GLN	57	-6.104	-6.442	-3.431	1.00	0.00
ATOM	901	CG	GLN	57	-6.471	-8.322	-4.415	1.00	0.00
ATOM	902	HG2	GLN	57	-5.583	-8.789	-3.989	1.00	0.00
ATOM	903	HG3	GLN	57	-7.266	-9.063	-4.497	1.00	0.00

ATOM	904	CD	GLN	57	-6.131	-7.813	-5.817	1.00	0.00
ATOM	905	OE1	GLN	57	-6.525	-6.728	-6.195	1.00	0.00
ATOM	906	NE2	GLN	57	-5.409	-8.558	-6.610	1.00	0.00
ATOM	907	HE21	GLN	57	-5.180	-8.230	-7.537	1.00	0.00
ATOM	908	HE22	GLN	57	-5.082	-9.460	-6.293	1.00	0.00
ATOM	909	C	GLN	57	-7.737	-6.497	-1.270	1.00	0.00
ATOM	910	O	GLN	57	-7.008	-6.112	-0.378	1.00	0.00
ATOM	911	N	GLN	58	-8.886	-5.922	-1.500	1.00	0.00
ATOM	912	H	GLN	58	-9.498	-6.237	-2.239	1.00	0.00
ATOM	913	CA	GLN	58	-9.325	-4.773	-0.661	1.00	0.00
ATOM	914	HA	GLN	58	-8.743	-3.884	-0.904	1.00	0.00
ATOM	915	CB	GLN	58	-10.803	-4.479	-0.929	1.00	0.00
ATOM	916	HB2	GLN	58	-11.404	-5.340	-0.635	1.00	0.00
ATOM	917	HB3	GLN	58	-11.111	-3.608	-0.352	1.00	0.00
ATOM	918	CG	GLN	58	-11.006	-4.201	-2.420	1.00	0.00
ATOM	919	HG2	GLN	58	-10.223	-3.541	-2.794	1.00	0.00
ATOM	920	HG3	GLN	58	-10.993	-5.134	-2.984	1.00	0.00
ATOM	921	CD	GLN	58	-12.361	-3.521	-2.630	1.00	0.00
ATOM	922	OE1	GLN	58	-12.503	-2.679	-3.494	1.00	0.00
ATOM	923	NE2	GLN	58	-13.369	-3.854	-1.872	1.00	0.00
ATOM	924	HE21	GLN	58	-14.266	-3.410	-2.005	1.00	0.00
ATOM	925	HE22	GLN	58	-13.248	-4.556	-1.155	1.00	0.00

ATOM 926 C GLN 58 -9.137 -5.120 0.817 1.00 0.00
ATOM 927 O GLN 58 -8.705 -4.303 1.606 1.00 0.00
ATOM 928 N ARG 59 -9.459 -6.326 1.199 1.00 0.00
ATOM 929 H ARG 59 -9.819 -7.011 0.549 1.00 0.00
ATOM 930 CA ARG 59 -9.301 -6.722 2.626 1.00 0.00
ATOM 931 HA ARG 59 -10.037 -6.209 3.245 1.00 0.00
ATOM 932 CB ARG 59 -9.514 -8.231 2.763 1.00 0.00
ATOM 933 HB2 ARG 59 -8.785 -8.760 2.148 1.00 0.00
ATOM 934 HB3 ARG 59 -9.389 -8.524 3.806 1.00 0.00
ATOM 935 CG ARG 59 -10.927 -8.589 2.299 1.00 0.00
ATOM 936 HG2 ARG 59 -11.656 -8.035 2.890 1.00 0.00
ATOM 937 HG3 ARG 59 -11.041 -8.330 1.246 1.00 0.00
ATOM 938 CD ARG 59 -11.158 -10.091 2.482 1.00 0.00
ATOM 939 HD2 ARG 59 -10.369 -10.658 1.988 1.00 0.00
ATOM 940 HD3 ARG 59 -11.177 -10.345 3.542 1.00 0.00
ATOM 941 NE ARG 59 -12.467 -10.468 1.877 1.00 0.00
ATOM 942 HE ARG 59 -12.560 -10.646 0.887 1.00 0.00
ATOM 943 CZ ARG 59 -13.527 -10.576 2.632 1.00 0.00
ATOM 944 NH1 ARG 59 -13.826 -11.723 3.177 1.00 0.00
ATOM 945 HH11 ARG 59 -13.235 -12.526 3.013 1.00 0.00
ATOM 946 HH12 ARG 59 -14.646 -11.803 3.760 1.00 0.00
ATOM 947 NH2 ARG 59 -14.287 -9.535 2.841 1.00 0.00

```
ATOM    948 HH21 ARG    59     -14.052  -8.649   2.417  1.00  0.00
ATOM    949 HH22 ARG    59     -15.106  -9.621   3.425  1.00  0.00
ATOM    950  C   ARG    59      -7.896  -6.358   3.105  1.00  0.00
ATOM    951  O   ARG    59      -7.682  -6.065   4.264  1.00  0.00
ATOM    952  N   CYX    60      -6.936  -6.367   2.223  1.00  0.00
ATOM    953  H   CYX    60      -7.103  -6.607   1.256  1.00  0.00
ATOM    954  CA  CYX    60      -5.548  -6.015   2.633  1.00  0.00
ATOM    955  HA  CYX    60      -5.098  -6.835   3.194  1.00  0.00
ATOM    956  CB  CYX    60      -4.701  -5.754   1.384  1.00  0.00
ATOM    957  HB2 CYX    60      -4.450  -6.703   0.909  1.00  0.00
ATOM    958  HB3 CYX    60      -5.264  -5.136   0.685  1.00  0.00
ATOM    959  SG  CYX    60      -3.143  -4.962   1.856  1.00  0.00
ATOM    960  C   CYX    60      -5.590  -4.755   3.500  1.00  0.00
ATOM    961  O   CYX    60      -5.678  -4.824   4.711  1.00  0.00
ATOM    962  N   CYX    61      -5.528  -3.607   2.888  1.00  0.00
ATOM    963  H   CYX    61      -5.451  -3.544   1.883  1.00  0.00
ATOM    964  CA  CYX    61      -5.566  -2.342   3.675  1.00  0.00
ATOM    965  HA  CYX    61      -4.594  -2.155   4.133  1.00  0.00
ATOM    966  CB  CYX    61      -5.906  -1.171   2.752  1.00  0.00
ATOM    967  HB2 CYX    61      -5.984  -1.528   1.725  1.00  0.00
ATOM    968  HB3 CYX    61      -6.855  -0.732   3.057  1.00  0.00
ATOM    969  SG  CYX    61      -4.572   0.052   2.815  1.00  0.00
```

ATOM	970	C	CYX	61	-6.630	-2.459	4.769	1.00	0.00
ATOM	971	O	CYX	61	-6.443	-2.008	5.881	1.00	0.00
ATOM	972	N	ASP	62	-7.744	-3.063	4.460	1.00	0.00
ATOM	973	H	ASP	62	-7.905	-3.441	3.537	1.00	0.00
ATOM	974	CA	ASP	62	-8.820	-3.209	5.482	1.00	0.00
ATOM	975	HA	ASP	62	-9.150	-2.229	5.825	1.00	0.00
ATOM	976	CB	ASP	62	-10.014	-3.939	4.866	1.00	0.00
ATOM	977	HB2	ASP	62	-9.683	-4.889	4.446	1.00	0.00
ATOM	978	HB3	ASP	62	-10.763	-4.124	5.635	1.00	0.00
ATOM	979	CG	ASP	62	-10.623	-3.077	3.758	1.00	0.00
ATOM	980	OD1	ASP	62	-10.269	-1.911	3.680	1.00	0.00
ATOM	981	OD2	ASP	62	-11.432	-3.595	3.006	1.00	0.00
ATOM	982	C	ASP	62	-8.289	-4.012	6.669	1.00	0.00
ATOM	983	O	ASP	62	-8.619	-3.748	7.809	1.00	0.00
ATOM	984	N	GLU	63	-7.465	-4.988	6.413	1.00	0.00
ATOM	985	H	GLU	63	-7.186	-5.215	5.469	1.00	0.00
ATOM	986	CA	GLU	63	-6.908	-5.803	7.528	1.00	0.00
ATOM	987	HA	GLU	63	-7.715	-6.235	8.120	1.00	0.00
ATOM	988	CB	GLU	63	-6.059	-6.940	6.956	1.00	0.00
ATOM	989	HB2	GLU	63	-5.455	-6.563	6.130	1.00	0.00
ATOM	990	HB3	GLU	63	-5.404	-7.332	7.734	1.00	0.00
ATOM	991	CG	GLU	63	-6.974	-8.056	6.450	1.00	0.00

```
ATOM    992  HG2 GLU    63      -7.651  -8.361   7.248  1.00  0.00
ATOM    993  HG3 GLU    63      -7.554  -7.694   5.601  1.00  0.00
ATOM    994  CD  GLU    63      -6.127  -9.253   6.015  1.00  0.00
ATOM    995  OE1 GLU    63      -4.915  -9.171   6.135  1.00  0.00
ATOM    996  OE2 GLU    63      -6.704 -10.232   5.569  1.00  0.00
ATOM    997  C   GLU    63      -6.040  -4.910   8.415  1.00  0.00
ATOM    998  O   GLU    63      -6.182  -4.888   9.621  1.00  0.00
ATOM    999  N   LEU    64      -5.141  -4.172   7.824  1.00  0.00
ATOM   1000  H   LEU    64      -5.020  -4.188   6.821  1.00  0.00
ATOM   1001  CA  LEU    64      -4.261  -3.278   8.627  1.00  0.00
ATOM   1002  HA  LEU    64      -3.786  -3.838   9.433  1.00  0.00
ATOM   1003  CB  LEU    64      -3.169  -2.696   7.726  1.00  0.00
ATOM   1004  HB2 LEU    64      -3.619  -2.030   6.991  1.00  0.00
ATOM   1005  HB3 LEU    64      -2.456  -2.138   8.334  1.00  0.00
ATOM   1006  CG  LEU    64      -2.443  -3.833   7.005  1.00  0.00
ATOM   1007  HG  LEU    64      -3.134  -4.336   6.328  1.00  0.00
ATOM   1008  CD1 LEU    64      -1.271  -3.261   6.204  1.00  0.00
ATOM   1009 HD11 LEU    64      -0.579  -2.759   6.880  1.00  0.00
ATOM   1010 HD12 LEU    64      -0.752  -4.071   5.690  1.00  0.00
ATOM   1011 HD13 LEU    64      -1.645  -2.547   5.471  1.00  0.00
ATOM   1012  CD2 LEU    64      -1.915  -4.836   8.032  1.00  0.00
ATOM   1013 HD21 LEU    64      -2.748  -5.244   8.604  1.00  0.00
```

ATOM	1014	HD22	LEU	64	-1.398	-5.646	7.517	1.00	0.00
ATOM	1015	HD23	LEU	64	-1.222	-4.335	8.707	1.00	0.00
ATOM	1016	C	LEU	64	-5.088	-2.135	9.224	1.00	0.00
ATOM	1017	O	LEU	64	-4.577	-1.298	9.940	1.00	0.00
ATOM	1018	N	ASN	65	-6.362	-2.091	8.938	1.00	0.00
ATOM	1019	H	ASN	65	-6.798	-2.783	8.346	1.00	0.00
ATOM	1020	CA	ASN	65	-7.212	-0.999	9.495	1.00	0.00
ATOM	1021	HA	ASN	65	-6.587	-0.187	9.867	1.00	0.00
ATOM	1022	CB	ASN	65	-8.124	-0.450	8.396	1.00	0.00
ATOM	1023	HB2	ASN	65	-8.810	-1.226	8.055	1.00	0.00
ATOM	1024	HB3	ASN	65	-8.694	0.401	8.768	1.00	0.00
ATOM	1025	CG	ASN	65	-7.278	0.010	7.210	1.00	0.00
ATOM	1026	OD1	ASN	65	-7.794	-0.003	6.011	1.00	0.00
ATOM	1027	ND2	ASN	65	-6.133	0.385	7.373	1.00	0.00
ATOM	1028	HD21	ASN	65	-5.585	0.686	6.579	1.00	0.00
ATOM	1029	HD22	ASN	65	-5.735	0.393	8.301	1.00	0.00
ATOM	1030	C	ASN	65	-8.071	-1.549	10.636	1.00	0.00
ATOM	1031	O	ASN	65	-8.711	-0.808	11.354	1.00	0.00
ATOM	1032	N	GLU	66	-8.093	-2.843	10.808	1.00	0.00
ATOM	1033	H	GLU	66	-7.564	-3.469	10.217	1.00	0.00
ATOM	1034	CA	GLU	66	-8.917	-3.432	11.903	1.00	0.00
ATOM	1035	HA	GLU	66	-9.092	-2.694	12.685	1.00	0.00

ATOM	1036	CB	GLU	66	-10.268	-3.875	11.338	1.00	0.00
ATOM	1037	HB2	GLU	66	-10.117	-4.696	10.637	1.00	0.00
ATOM	1038	HB3	GLU	66	-10.911	-4.208	12.153	1.00	0.00
ATOM	1039	CG	GLU	66	-10.929	-2.701	10.614	1.00	0.00
ATOM	1040	HG2	GLU	66	-11.185	-1.927	11.337	1.00	0.00
ATOM	1041	HG3	GLU	66	-10.239	-2.294	9.875	1.00	0.00
ATOM	1042	CD	GLU	66	-12.200	-3.184	9.913	1.00	0.00
ATOM	1043	OE1	GLU	66	-12.388	-4.388	9.835	1.00	0.00
ATOM	1044	OE2	GLU	66	-12.963	-2.344	9.466	1.00	0.00
ATOM	1045	C	GLU	66	-8.197	-4.643	12.504	1.00	0.00
ATOM	1046	O	GLU	66	-8.318	-4.927	13.678	1.00	0.00
ATOM	1047	N	MET	67	-7.453	-5.362	11.709	1.00	0.00
ATOM	1048	H	MET	67	-7.345	-5.137	10.730	1.00	0.00
ATOM	1049	CA	MET	67	-6.735	-6.555	12.242	1.00	0.00
ATOM	1050	HA	MET	67	-7.400	-7.148	12.870	1.00	0.00
ATOM	1051	CB	MET	67	-6.259	-7.425	11.077	1.00	0.00
ATOM	1052	HB2	MET	67	-6.429	-6.901	10.137	1.00	0.00
ATOM	1053	HB3	MET	67	-5.195	-7.633	11.189	1.00	0.00
ATOM	1054	CG	MET	67	-7.038	-8.742	11.074	1.00	0.00
ATOM	1055	HG2	MET	67	-7.458	-8.925	12.063	1.00	0.00
ATOM	1056	HG3	MET	67	-7.843	-8.693	10.341	1.00	0.00
ATOM	1057	SD	MET	67	-5.923	-10.100	10.641	1.00	0.00

ATOM	1058	CE	MET	67	-5.813	-9.746	8.870	1.00	0.00
ATOM	1059	HE1	MET	67	-5.405	-8.746	8.723	1.00	0.00
ATOM	1060	HE2	MET	67	-5.160	-10.478	8.393	1.00	0.00
ATOM	1061	HE3	MET	67	-6.806	-9.802	8.425	1.00	0.00
ATOM	1062	C	MET	67	-5.530	-6.103	13.066	1.00	0.00
ATOM	1063	O	MET	67	-5.548	-6.133	14.281	1.00	0.00
ATOM	1064	N	GLU	68	-4.482	-5.687	12.415	1.00	0.00
ATOM	1065	H	GLU	68	-4.461	-5.663	11.405	1.00	0.00
ATOM	1066	CA	GLU	68	-3.272	-5.235	13.157	1.00	0.00
ATOM	1067	HA	GLU	68	-3.066	-5.907	13.991	1.00	0.00
ATOM	1068	CB	GLU	68	-2.066	-5.241	12.216	1.00	0.00
ATOM	1069	HB2	GLU	68	-2.157	-4.423	11.502	1.00	0.00
ATOM	1070	HB3	GLU	68	-1.151	-5.117	12.796	1.00	0.00
ATOM	1071	CG	GLU	68	-2.015	-6.571	11.462	1.00	0.00
ATOM	1072	HG2	GLU	68	-1.810	-7.380	12.164	1.00	0.00
ATOM	1073	HG3	GLU	68	-2.973	-6.749	10.974	1.00	0.00
ATOM	1074	CD	GLU	68	-0.908	-6.519	10.407	1.00	0.00
ATOM	1075	OE1	GLU	68	-0.339	-5.455	10.225	1.00	0.00
ATOM	1076	OE2	GLU	68	-0.647	-7.545	9.799	1.00	0.00
ATOM	1077	C	GLU	68	-3.495	-3.819	13.693	1.00	0.00
ATOM	1078	O	GLU	68	-2.613	-3.221	14.276	1.00	0.00
ATOM	1079	N	ASN	69	-4.667	-3.278	13.502	1.00	0.00

ATOM	1080	H	ASN	69	-5.407	-3.768	13.020	1.00	0.00
ATOM	1081	CA	ASN	69	-4.940	-1.900	14.002	1.00	0.00
ATOM	1082	HA	ASN	69	-5.864	-1.517	13.570	1.00	0.00
ATOM	1083	CB	ASN	69	-5.088	-1.931	15.525	1.00	0.00
ATOM	1084	HB2	ASN	69	-4.154	-2.250	15.989	1.00	0.00
ATOM	1085	HB3	ASN	69	-5.360	-0.944	15.898	1.00	0.00
ATOM	1086	CG	ASN	69	-6.188	-2.920	15.911	1.00	0.00
ATOM	1087	OD1	ASN	69	-7.123	-3.132	15.163	1.00	0.00
ATOM	1088	ND2	ASN	69	-6.117	-3.541	17.057	1.00	0.00
ATOM	1089	HD21	ASN	69	-6.840	-4.196	17.319	1.00	0.00
ATOM	1090	HD22	ASN	69	-5.339	-3.364	17.677	1.00	0.00
ATOM	1091	C	ASN	69	-3.777	-0.982	13.619	1.00	0.00
ATOM	1092	O	ASN	69	-3.520	0.012	14.269	1.00	0.00
ATOM	1093	N	THR	70	-3.074	-1.309	12.571	1.00	0.00
ATOM	1094	H	THR	70	-3.285	-2.135	12.028	1.00	0.00
ATOM	1095	CA	THR	70	-1.926	-0.457	12.145	1.00	0.00
ATOM	1096	HA	THR	70	-1.059	-0.641	12.779	1.00	0.00
ATOM	1097	CB	THR	70	-1.553	-0.791	10.698	1.00	0.00
ATOM	1098	HB	THR	70	-0.544	-0.433	10.492	1.00	0.00
ATOM	1099	CG2	THR	70	-1.608	-2.306	10.490	1.00	0.00
ATOM	1100	HG21	THR	70	-2.616	-2.666	10.696	1.00	0.00
ATOM	1101	HG22	THR	70	-1.342	-2.542	9.459	1.00	0.00

```
ATOM   1102 HG23 THR    70      -0.904  -2.792  11.166  1.00  0.00
ATOM   1103  OG1 THR    70      -2.467  -0.156   9.815  1.00  0.00
ATOM   1104  HG1 THR    70      -2.233  -0.366   8.908  1.00  0.00
ATOM   1105  C   THR    70      -2.321   1.018  12.242  1.00  0.00
ATOM   1106  O   THR    70      -3.487   1.360  12.249  1.00  0.00
ATOM   1107  N   GLN    71      -1.358   1.897  12.318  1.00  0.00
ATOM   1108  H   GLN    71      -0.386   1.621  12.313  1.00  0.00
ATOM   1109  CA  GLN    71      -1.681   3.349  12.415  1.00  0.00
ATOM   1110  HA  GLN    71      -2.300   3.655  11.572  1.00  0.00
ATOM   1111  CB  GLN    71      -2.451   3.615  13.709  1.00  0.00
ATOM   1112  HB2 GLN    71      -3.391   3.064  13.693  1.00  0.00
ATOM   1113  HB3 GLN    71      -1.854   3.290  14.561  1.00  0.00
ATOM   1114  CG  GLN    71      -2.740   5.114  13.830  1.00  0.00
ATOM   1115  HG2 GLN    71      -1.828   5.688  13.667  1.00  0.00
ATOM   1116  HG3 GLN    71      -3.493   5.414  13.101  1.00  0.00
ATOM   1117  CD  GLN    71      -3.268   5.421  15.234  1.00  0.00
ATOM   1118  OE1 GLN    71      -2.667   5.041  16.219  1.00  0.00
ATOM   1119  NE2 GLN    71      -4.376   6.098  15.366  1.00  0.00
ATOM   1120 HE21 GLN    71      -4.731   6.304  16.289  1.00  0.00
ATOM   1121 HE22 GLN    71      -4.873   6.412  14.545  1.00  0.00
ATOM   1122  C   GLN    71      -0.384   4.162  12.417  1.00  0.00
ATOM   1123  O   GLN    71      -0.328   5.259  11.897  1.00  0.00
```

ATOM	1124	N	GLY	72	0.658	3.636	13.002	1.00	0.00
ATOM	1125	H	GLY	72	0.620	2.725	13.438	1.00	0.00
ATOM	1126	CA	GLY	72	1.947	4.383	13.038	1.00	0.00
ATOM	1127	HA2	GLY	72	1.789	5.411	12.712	1.00	0.00
ATOM	1128	HA3	GLY	72	2.349	4.380	14.051	1.00	0.00
ATOM	1129	C	GLY	72	2.955	3.713	12.101	1.00	0.00
ATOM	1130	O	GLY	72	2.817	2.558	11.748	1.00	0.00
ATOM	1131	N	CYX	73	3.969	4.428	11.698	1.00	0.00
ATOM	1132	H	CYX	73	4.089	5.388	11.988	1.00	0.00
ATOM	1133	CA	CYX	73	4.985	3.834	10.785	1.00	0.00
ATOM	1134	HA	CYX	73	5.807	4.532	10.628	1.00	0.00
ATOM	1135	CB	CYX	73	5.544	2.553	11.407	1.00	0.00
ATOM	1136	HB2	CYX	73	4.939	2.272	12.269	1.00	0.00
ATOM	1137	HB3	CYX	73	5.520	1.750	10.670	1.00	0.00
ATOM	1138	SG	CYX	73	7.234	2.848	11.986	1.00	0.00
ATOM	1139	C	CYX	73	4.334	3.507	9.438	1.00	0.00
ATOM	1140	O	CYX	73	4.919	2.855	8.596	1.00	0.00
ATOM	1141	N	MET	74	3.127	3.956	9.229	1.00	0.00
ATOM	1142	H	MET	74	2.636	4.499	9.924	1.00	0.00
ATOM	1143	CA	MET	74	2.439	3.671	7.939	1.00	0.00
ATOM	1144	HA	MET	74	2.075	2.643	7.923	1.00	0.00
ATOM	1145	CB	MET	74	1.245	4.616	7.778	1.00	0.00

ATOM	1146	HB2	MET	74	1.602	5.641	7.681	1.00	0.00
ATOM	1147	HB3	MET	74	0.681	4.342	6.886	1.00	0.00
ATOM	1148	CG	MET	74	0.341	4.504	9.008	1.00	0.00
ATOM	1149	HG2	MET	74	0.200	3.454	9.267	1.00	0.00
ATOM	1150	HG3	MET	74	0.796	5.027	9.849	1.00	0.00
ATOM	1151	SD	MET	74	-1.268	5.249	8.644	1.00	0.00
ATOM	1152	CE	MET	74	-2.138	3.719	8.222	1.00	0.00
ATOM	1153	HE1	MET	74	-2.122	3.043	9.078	1.00	0.00
ATOM	1154	HE2	MET	74	-3.170	3.948	7.959	1.00	0.00
ATOM	1155	HE3	MET	74	-1.645	3.242	7.375	1.00	0.00
ATOM	1156	C	MET	74	3.420	3.884	6.784	1.00	0.00
ATOM	1157	O	MET	74	3.646	3.002	5.979	1.00	0.00
ATOM	1158	N	CYX	75	4.006	5.047	6.697	1.00	0.00
ATOM	1159	H	CYX	75	3.825	5.786	7.361	1.00	0.00
ATOM	1160	CA	CYX	75	4.972	5.310	5.594	1.00	0.00
ATOM	1161	HA	CYX	75	4.443	5.433	4.649	1.00	0.00
ATOM	1162	CB	CYX	75	5.748	6.595	5.891	1.00	0.00
ATOM	1163	HB2	CYX	75	5.055	7.373	6.209	1.00	0.00
ATOM	1164	HB3	CYX	75	6.472	6.407	6.684	1.00	0.00
ATOM	1165	SG	CYX	75	6.570	7.164	4.383	1.00	0.00
ATOM	1166	C	CYX	75	5.948	4.139	5.481	1.00	0.00
ATOM	1167	O	CYX	75	6.157	3.593	4.415	1.00	0.00

ATOM	1168	N	GLU	76	6.547	3.743	6.572	1.00	0.00
ATOM	1169	H	GLU	76	6.381	4.188	7.463	1.00	0.00
ATOM	1170	CA	GLU	76	7.505	2.603	6.520	1.00	0.00
ATOM	1171	HA	GLU	76	8.348	2.847	5.874	1.00	0.00
ATOM	1172	CB	GLU	76	8.035	2.313	7.926	1.00	0.00
ATOM	1173	HB2	GLU	76	7.208	2.025	8.575	1.00	0.00
ATOM	1174	HB3	GLU	76	8.760	1.500	7.881	1.00	0.00
ATOM	1175	CG	GLU	76	8.709	3.567	8.485	1.00	0.00
ATOM	1176	HG2	GLU	76	9.461	3.919	7.779	1.00	0.00
ATOM	1177	HG3	GLU	76	7.961	4.344	8.638	1.00	0.00
ATOM	1178	CD	GLU	76	9.380	3.236	9.820	1.00	0.00
ATOM	1179	OE1	GLU	76	9.373	2.073	10.192	1.00	0.00
ATOM	1180	OE2	GLU	76	9.888	4.149	10.448	1.00	0.00
ATOM	1181	C	GLU	76	6.784	1.367	5.979	1.00	0.00
ATOM	1182	O	GLU	76	7.400	0.426	5.517	1.00	0.00
ATOM	1183	N	ALA	77	5.479	1.368	6.024	1.00	0.00
ATOM	1184	H	ALA	77	4.961	2.146	6.405	1.00	0.00
ATOM	1185	CA	ALA	77	4.717	0.199	5.505	1.00	0.00
ATOM	1186	HA	ALA	77	5.186	-0.749	5.772	1.00	0.00
ATOM	1187	CB	ALA	77	3.296	0.219	6.073	1.00	0.00
ATOM	1188	HB1	ALA	77	2.811	1.157	5.806	1.00	0.00
ATOM	1189	HB2	ALA	77	2.728	-0.615	5.660	1.00	0.00

ATOM	1190	HB3	ALA	77	3.337	0.127	7.158	1.00	0.00
ATOM	1191	C	ALA	77	4.661	0.278	3.980	1.00	0.00
ATOM	1192	O	ALA	77	5.119	-0.609	3.286	1.00	0.00
ATOM	1193	N	LEU	78	4.111	1.335	3.450	1.00	0.00
ATOM	1194	H	LEU	78	3.729	2.078	4.017	1.00	0.00
ATOM	1195	CA	LEU	78	4.039	1.469	1.969	1.00	0.00
ATOM	1196	HA	LEU	78	3.393	0.696	1.552	1.00	0.00
ATOM	1197	CB	LEU	78	3.463	2.838	1.603	1.00	0.00
ATOM	1198	HB2	LEU	78	2.505	2.975	2.104	1.00	0.00
ATOM	1199	HB3	LEU	78	4.153	3.620	1.920	1.00	0.00
ATOM	1200	CG	LEU	78	3.263	2.918	0.090	1.00	0.00
ATOM	1201	HG	LEU	78	4.173	2.593	-0.415	1.00	0.00
ATOM	1202	CD1	LEU	78	2.101	2.011	-0.320	1.00	0.00
ATOM	1203	HD11	LEU	78	1.191	2.335	0.184	1.00	0.00
ATOM	1204	HD12	LEU	78	1.958	2.068	-1.399	1.00	0.00
ATOM	1205	HD13	LEU	78	2.325	0.982	-0.037	1.00	0.00
ATOM	1206	CD2	LEU	78	2.948	4.361	-0.309	1.00	0.00
ATOM	1207	HD21	LEU	78	3.776	5.007	-0.018	1.00	0.00
ATOM	1208	HD22	LEU	78	2.806	4.417	-1.388	1.00	0.00
ATOM	1209	HD23	LEU	78	2.038	4.687	0.195	1.00	0.00
ATOM	1210	C	LEU	78	5.449	1.336	1.393	1.00	0.00
ATOM	1211	O	LEU	78	5.672	0.636	0.426	1.00	0.00

ATOM	1212	N	GLN	79	6.403	2.001	1.986	1.00	0.00
ATOM	1213	H	GLN	79	6.223	2.584	2.791	1.00	0.00
ATOM	1214	CA	GLN	79	7.799	1.910	1.479	1.00	0.00
ATOM	1215	HA	GLN	79	7.870	2.356	0.487	1.00	0.00
ATOM	1216	CB	GLN	79	8.739	2.665	2.423	1.00	0.00
ATOM	1217	HB2	GLN	79	8.610	2.292	3.439	1.00	0.00
ATOM	1218	HB3	GLN	79	9.771	2.511	2.108	1.00	0.00
ATOM	1219	CG	GLN	79	8.411	4.159	2.382	1.00	0.00
ATOM	1220	HG2	GLN	79	8.582	4.555	1.381	1.00	0.00
ATOM	1221	HG3	GLN	79	7.373	4.327	2.666	1.00	0.00
ATOM	1222	CD	GLN	79	9.315	4.906	3.365	1.00	0.00
ATOM	1223	OE1	GLN	79	9.538	6.180	3.189	1.00	0.00
ATOM	1224	NE2	GLN	79	9.825	4.324	4.302	1.00	0.00
ATOM	1225	HE21	GLN	79	10.420	4.829	4.943	1.00	0.00
ATOM	1226	HE22	GLN	79	9.652	3.337	4.435	1.00	0.00
ATOM	1227	C	GLN	79	8.214	0.442	1.413	1.00	0.00
ATOM	1228	O	GLN	79	8.778	-0.012	0.438	1.00	0.00
ATOM	1229	N	GLN	80	7.931	-0.308	2.442	1.00	0.00
ATOM	1230	H	GLN	80	7.459	0.059	3.255	1.00	0.00
ATOM	1231	CA	GLN	80	8.304	-1.750	2.434	1.00	0.00
ATOM	1232	HA	GLN	80	9.389	-1.859	2.415	1.00	0.00
ATOM	1233	CB	GLN	80	7.767	-2.426	3.697	1.00	0.00

ATOM	1234	HB2	GLN	80	8.091	-1.867	4.575	1.00	0.00
ATOM	1235	HB3	GLN	80	6.678	-2.449	3.663	1.00	0.00
ATOM	1236	CG	GLN	80	8.304	-3.856	3.777	1.00	0.00
ATOM	1237	HG2	GLN	80	7.844	-4.386	4.611	1.00	0.00
ATOM	1238	HG3	GLN	80	8.099	-4.391	2.850	1.00	0.00
ATOM	1239	CD	GLN	80	9.818	-3.821	3.996	1.00	0.00
ATOM	1240	OE1	GLN	80	10.305	-3.094	4.839	1.00	0.00
ATOM	1241	NE2	GLN	80	10.588	-4.581	3.266	1.00	0.00
ATOM	1242	HE21	GLN	80	11.588	-4.564	3.405	1.00	0.00
ATOM	1243	HE22	GLN	80	10.180	-5.184	2.565	1.00	0.00
ATOM	1244	C	GLN	80	7.694	-2.417	1.200	1.00	0.00
ATOM	1245	O	GLN	80	8.359	-3.136	0.480	1.00	0.00
ATOM	1246	N	ILE	81	6.437	-2.178	0.947	1.00	0.00
ATOM	1247	H	ILE	81	5.879	-1.580	1.539	1.00	0.00
ATOM	1248	CA	ILE	81	5.790	-2.793	-0.245	1.00	0.00
ATOM	1249	HA	ILE	81	5.659	-3.862	-0.077	1.00	0.00
ATOM	1250	CB	ILE	81	4.425	-2.142	-0.475	1.00	0.00
ATOM	1251	HB	ILE	81	4.549	-1.064	-0.571	1.00	0.00
ATOM	1252	CG2	ILE	81	3.802	-2.699	-1.756	1.00	0.00
ATOM	1253	HG21	ILE	81	3.678	-3.778	-1.661	1.00	0.00
ATOM	1254	HG22	ILE	81	2.830	-2.235	-1.919	1.00	0.00
ATOM	1255	HG23	ILE	81	4.454	-2.483	-2.602	1.00	0.00

ATOM	1256	CG1	ILE	81	3.506	-2.446	0.710	1.00	0.00
ATOM	1257	HG12	ILE	81	3.464	-3.524	0.869	1.00	0.00
ATOM	1258	HG13	ILE	81	3.896	-1.962	1.605	1.00	0.00
ATOM	1259	CD1	ILE	81	2.100	-1.918	0.416	1.00	0.00
ATOM	1260	HD11	ILE	81	1.710	-2.402	-0.479	1.00	0.00
ATOM	1261	HD12	ILE	81	1.446	-2.135	1.260	1.00	0.00
ATOM	1262	HD13	ILE	81	2.142	-0.841	0.257	1.00	0.00
ATOM	1263	C	ILE	81	6.680	-2.566	-1.467	1.00	0.00
ATOM	1264	O	ILE	81	7.172	-3.499	-2.072	1.00	0.00
ATOM	1265	N	MET	82	6.897	-1.332	-1.832	1.00	0.00
ATOM	1266	H	MET	82	6.493	-0.553	-1.333	1.00	0.00
ATOM	1267	CA	MET	82	7.761	-1.045	-3.010	1.00	0.00
ATOM	1268	HA	MET	82	7.231	-1.275	-3.934	1.00	0.00
ATOM	1269	CB	MET	82	8.135	0.439	-3.017	1.00	0.00
ATOM	1270	HB2	MET	82	8.790	0.653	-2.172	1.00	0.00
ATOM	1271	HB3	MET	82	8.652	0.679	-3.946	1.00	0.00
ATOM	1272	CG	MET	82	6.866	1.286	-2.906	1.00	0.00
ATOM	1273	HG2	MET	82	6.423	1.160	-1.918	1.00	0.00
ATOM	1274	HG3	MET	82	7.109	2.336	-3.064	1.00	0.00
ATOM	1275	SD	MET	82	5.677	0.755	-4.163	1.00	0.00
ATOM	1276	CE	MET	82	6.662	1.211	-5.612	1.00	0.00
ATOM	1277	HE1	MET	82	7.603	0.660	-5.601	1.00	0.00

ATOM 1278 HE2 MET 82 6.110 0.966 -6.519 1.00 0.00
ATOM 1279 HE3 MET 82 6.868 2.281 -5.590 1.00 0.00
ATOM 1280 C MET 82 9.031 -1.890 -2.924 1.00 0.00
ATOM 1281 O MET 82 9.392 -2.585 -3.851 1.00 0.00
ATOM 1282 N GLU 83 9.707 -1.841 -1.812 1.00 0.00
ATOM 1283 H GLU 83 9.411 -1.269 -1.035 1.00 0.00
ATOM 1284 CA GLU 83 10.952 -2.644 -1.663 1.00 0.00
ATOM 1285 HA GLU 83 11.631 -2.448 -2.493 1.00 0.00
ATOM 1286 CB GLU 83 11.653 -2.261 -0.357 1.00 0.00
ATOM 1287 HB2 GLU 83 10.960 -2.379 0.476 1.00 0.00
ATOM 1288 HB3 GLU 83 12.517 -2.908 -0.204 1.00 0.00
ATOM 1289 CG GLU 83 12.114 -0.804 -0.435 1.00 0.00
ATOM 1290 HG2 GLU 83 12.745 -0.668 -1.314 1.00 0.00
ATOM 1291 HG3 GLU 83 11.244 -0.152 -0.508 1.00 0.00
ATOM 1292 CD GLU 83 12.910 -0.453 0.823 1.00 0.00
ATOM 1293 OE1 GLU 83 12.978 -1.286 1.710 1.00 0.00
ATOM 1294 OE2 GLU 83 13.440 0.645 0.877 1.00 0.00
ATOM 1295 C GLU 83 10.592 -4.130 -1.634 1.00 0.00
ATOM 1296 O GLU 83 11.440 -4.988 -1.776 1.00 0.00
ATOM 1297 N ASN 84 9.337 -4.437 -1.455 1.00 0.00
ATOM 1298 H ASN 84 8.629 -3.728 -1.336 1.00 0.00
ATOM 1299 CA ASN 84 8.918 -5.865 -1.421 1.00 0.00

ATOM	1300	HA	ASN	84	9.656	-6.463	-0.885	1.00	0.00
ATOM	1301	CB	ASN	84	7.573	-5.987	-0.701	1.00	0.00
ATOM	1302	HB2	ASN	84	7.616	-5.488	0.267	1.00	0.00
ATOM	1303	HB3	ASN	84	6.780	-5.544	-1.303	1.00	0.00
ATOM	1304	CG	ASN	84	7.248	-7.464	-0.475	1.00	0.00
ATOM	1305	OD1	ASN	84	7.869	-8.118	0.339	1.00	0.00
ATOM	1306	ND2	ASN	84	6.293	-8.023	-1.167	1.00	0.00
ATOM	1307	HD21	ASN	84	6.073	-8.999	-1.023	1.00	0.00
ATOM	1308	HD22	ASN	84	5.778	-7.478	-1.844	1.00	0.00
ATOM	1309	C	ASN	84	8.781	-6.388	-2.852	1.00	0.00
ATOM	1310	O	ASN	84	8.830	-7.578	-3.094	1.00	0.00
ATOM	1311	N	GLN	85	8.608	-5.510	-3.803	1.00	0.00
ATOM	1312	H	GLN	85	8.563	-4.519	-3.613	1.00	0.00
ATOM	1313	CA	GLN	85	8.470	-5.966	-5.215	1.00	0.00
ATOM	1314	HA	GLN	85	9.276	-6.654	-5.472	1.00	0.00
ATOM	1315	CB	GLN	85	7.134	-6.693	-5.387	1.00	0.00
ATOM	1316	HB2	GLN	85	7.014	-7.428	-4.591	1.00	0.00
ATOM	1317	HB3	GLN	85	6.318	-5.972	-5.341	1.00	0.00
ATOM	1318	CG	GLN	85	7.111	-7.402	-6.743	1.00	0.00
ATOM	1319	HG2	GLN	85	7.217	-6.678	-7.551	1.00	0.00
ATOM	1320	HG3	GLN	85	7.919	-8.132	-6.803	1.00	0.00
ATOM	1321	CD	GLN	85	5.778	-8.133	-6.913	1.00	0.00

ATOM	1322	OE1	GLN	85	5.076	-8.373	-5.951	1.00	0.00
ATOM	1323	NE2	GLN	85	5.397	-8.500	-8.106	1.00	0.00
ATOM	1324	HE21	GLN	85	4.518	-8.983	-8.226	1.00	0.00
ATOM	1325	HE22	GLN	85	5.983	-8.300	-8.904	1.00	0.00
ATOM	1326	C	GLN	85	8.516	-4.759	-6.158	1.00	0.00
ATOM	1327	O	GLN	85	7.698	-4.624	-7.045	1.00	0.00
ATOM	1328	N	CYX	86	9.470	-3.884	-5.979	1.00	0.00
ATOM	1329	H	CYX	86	10.157	-3.986	-5.245	1.00	0.00
ATOM	1330	CA	CYX	86	9.563	-2.695	-6.875	1.00	0.00
ATOM	1331	HA	CYX	86	8.581	-2.452	-7.281	1.00	0.00
ATOM	1332	CB	CYX	86	10.076	-1.491	-6.082	1.00	0.00
ATOM	1333	HB2	CYX	86	10.280	-0.666	-6.765	1.00	0.00
ATOM	1334	HB3	CYX	86	9.322	-1.185	-5.358	1.00	0.00
ATOM	1335	SG	CYX	86	11.628	-1.925	-5.258	1.00	0.00
ATOM	1336	C	CYX	86	10.529	-2.997	-8.021	1.00	0.00
ATOM	1337	O	CYX	86	10.642	-2.242	-8.966	1.00	0.00
ATOM	1338	N	ASP	87	11.231	-4.096	-7.944	1.00	0.00
ATOM	1339	H	ASP	87	11.144	-4.728	-7.161	1.00	0.00
ATOM	1340	CA	ASP	87	12.190	-4.445	-9.029	1.00	0.00
ATOM	1341	HA	ASP	87	13.078	-3.815	-8.967	1.00	0.00
ATOM	1342	CB	ASP	87	12.617	-5.907	-8.880	1.00	0.00
ATOM	1343	HB2	ASP	87	11.746	-6.519	-8.644	1.00	0.00

ATOM	1344	HB3	ASP	87	13.061	-6.253	-9.814	1.00	0.00
ATOM	1345	CG	ASP	87	13.644	-6.027	-7.752	1.00	0.00
ATOM	1346	OD1	ASP	87	14.077	-4.998	-7.259	1.00	0.00
ATOM	1347	OD2	ASP	87	13.980	-7.145	-7.400	1.00	0.00
ATOM	1348	C	ASP	87	11.518	-4.247	-10.389	1.00	0.00
ATOM	1349	O	ASP	87	12.076	-3.647	-11.287	1.00	0.00
ATOM	1350	N	ARG	88	10.324	-4.749	-10.550	1.00	0.00
ATOM	1351	H	ARG	88	9.855	-5.250	-9.809	1.00	0.00
ATOM	1352	CA	ARG	88	9.619	-4.591	-11.853	1.00	0.00
ATOM	1353	HA	ARG	88	10.222	-5.005	-12.662	1.00	0.00
ATOM	1354	CB	ARG	88	8.285	-5.340	-11.805	1.00	0.00
ATOM	1355	HB2	ARG	88	7.700	-4.987	-10.956	1.00	0.00
ATOM	1356	HB3	ARG	88	7.733	-5.159	-12.727	1.00	0.00
ATOM	1357	CG	ARG	88	8.547	-6.840	-11.654	1.00	0.00
ATOM	1358	HG2	ARG	88	9.137	-7.193	-12.500	1.00	0.00
ATOM	1359	HG3	ARG	88	9.094	-7.022	-10.729	1.00	0.00
ATOM	1360	CD	ARG	88	7.215	-7.590	-11.614	1.00	0.00
ATOM	1361	HD2	ARG	88	6.554	-7.145	-10.871	1.00	0.00
ATOM	1362	HD3	ARG	88	6.735	-7.558	-12.592	1.00	0.00
ATOM	1363	NE	ARG	88	7.455	-9.013	-11.247	1.00	0.00
ATOM	1364	HE	ARG	88	7.167	-9.384	-10.352	1.00	0.00
ATOM	1365	CZ	ARG	88	8.049	-9.814	-12.091	1.00	0.00

```
ATOM   1366  NH1 ARG    88       7.342 -10.625 -12.828  1.00  0.00
ATOM   1367 HH11 ARG    88       6.336 -10.632 -12.744  1.00  0.00
ATOM   1368 HH12 ARG    88       7.804 -11.243 -13.480  1.00  0.00
ATOM   1369  NH2 ARG    88       9.350  -9.801 -12.197  1.00  0.00
ATOM   1370 HH21 ARG    88       9.894  -9.171 -11.625  1.00  0.00
ATOM   1371 HH22 ARG    88       9.807 -10.422 -12.850  1.00  0.00
ATOM   1372  C   ARG    88       9.360  -3.106 -12.121  1.00  0.00
ATOM   1373  O   ARG    88       9.004  -2.717 -13.215  1.00  0.00
ATOM   1374  N   LEU    89       9.539  -2.275 -11.132  1.00  0.00
ATOM   1375  H   LEU    89       9.835  -2.591 -10.219  1.00  0.00
ATOM   1376  CA  LEU    89       9.304  -0.817 -11.334  1.00  0.00
ATOM   1377  HA  LEU    89       8.277  -0.641 -11.652  1.00  0.00
ATOM   1378  CB  LEU    89       9.544  -0.072 -10.019  1.00  0.00
ATOM   1379  HB2 LEU    89       9.057  -0.606  -9.203  1.00  0.00
ATOM   1380  HB3 LEU    89      10.615  -0.013  -9.826  1.00  0.00
ATOM   1381  CG  LEU    89       8.966   1.340 -10.121  1.00  0.00
ATOM   1382  HG  LEU    89       9.380   1.840 -10.997  1.00  0.00
ATOM   1383  CD1 LEU    89       7.444   1.259 -10.252  1.00  0.00
ATOM   1384 HD11 LEU    89       7.030   0.759  -9.377  1.00  0.00
ATOM   1385 HD12 LEU    89       7.031   2.265 -10.325  1.00  0.00
ATOM   1386 HD13 LEU    89       7.186   0.695 -11.148  1.00  0.00
ATOM   1387  CD2 LEU    89       9.325   2.133  -8.864  1.00  0.00
```

ATOM 1388 HD21 LEU 89 10.409 2.191 -8.768 1.00 0.00

ATOM 1389 HD22 LEU 89 8.913 3.139 -8.938 1.00 0.00

ATOM 1390 HD23 LEU 89 8.910 1.634 -7.988 1.00 0.00

ATOM 1391 C LEU 89 10.264 -0.288 -12.402 1.00 0.00

ATOM 1392 O LEU 89 10.081 0.787 -12.937 1.00 0.00

ATOM 1393 N GLN 90 11.287 -1.036 -12.715 1.00 0.00

ATOM 1394 H GLN 90 11.446 -1.932 -12.275 1.00 0.00

ATOM 1395 CA GLN 90 12.257 -0.575 -13.748 1.00 0.00

ATOM 1396 HA GLN 90 12.900 0.206 -13.342 1.00 0.00

ATOM 1397 CB GLN 90 13.133 -1.751 -14.185 1.00 0.00

ATOM 1398 HB2 GLN 90 12.505 -2.620 -14.382 1.00 0.00

ATOM 1399 HB3 GLN 90 13.676 -1.484 -15.092 1.00 0.00

ATOM 1400 CG GLN 90 14.131 -2.085 -13.073 1.00 0.00

ATOM 1401 HG2 GLN 90 14.703 -1.198 -12.800 1.00 0.00

ATOM 1402 HG3 GLN 90 13.609 -2.465 -12.195 1.00 0.00

ATOM 1403 CD GLN 90 15.102 -3.159 -13.567 1.00 0.00

ATOM 1404 OE1 GLN 90 14.877 -3.773 -14.591 1.00 0.00

ATOM 1405 NE2 GLN 90 16.179 -3.415 -12.876 1.00 0.00

ATOM 1406 HE21 GLN 90 16.825 -4.123 -13.196 1.00 0.00

ATOM 1407 HE22 GLN 90 16.362 -2.905 -12.024 1.00 0.00

ATOM 1408 C GLN 90 11.495 -0.032 -14.957 1.00 0.00

ATOM 1409 O GLN 90 11.993 0.789 -15.701 1.00 0.00

ATOM	1410	N	ASP	91	10.287	-0.484	-15.161	1.00	0.00
ATOM	1411	H	ASP	91	9.866	-1.166	-14.546	1.00	0.00
ATOM	1412	CA	ASP	91	9.493	0.005	-16.322	1.00	0.00
ATOM	1413	HA	ASP	91	10.052	-0.133	-17.248	1.00	0.00
ATOM	1414	CB	ASP	91	8.188	-0.786	-16.417	1.00	0.00
ATOM	1415	HB2	ASP	91	7.655	-0.723	-15.468	1.00	0.00
ATOM	1416	HB3	ASP	91	7.567	-0.370	-17.210	1.00	0.00
ATOM	1417	CG	ASP	91	8.500	-2.250	-16.729	1.00	0.00
ATOM	1418	OD1	ASP	91	9.630	-2.529	-17.096	1.00	0.00
ATOM	1419	OD2	ASP	91	7.605	-3.069	-16.597	1.00	0.00
ATOM	1420	C	ASP	91	9.176	1.490	-16.133	1.00	0.00
ATOM	1421	O	ASP	91	8.906	1.944	-15.038	1.00	0.00
ATOM	1422	N	ARG	92	9.209	2.253	-17.192	1.00	0.00
ATOM	1423	H	ARG	92	9.435	1.884	-18.105	1.00	0.00
ATOM	1424	CA	ARG	92	8.910	3.708	-17.072	1.00	0.00
ATOM	1425	HA	ARG	92	9.486	4.145	-16.256	1.00	0.00
ATOM	1426	CB	ARG	92	9.289	4.417	-18.373	1.00	0.00
ATOM	1427	HB2	ARG	92	10.365	4.338	-18.529	1.00	0.00
ATOM	1428	HB3	ARG	92	8.767	3.948	-19.207	1.00	0.00
ATOM	1429	CG	ARG	92	8.893	5.891	-18.287	1.00	0.00
ATOM	1430	HG2	ARG	92	7.850	5.969	-17.981	1.00	0.00
ATOM	1431	HG3	ARG	92	9.525	6.395	-17.556	1.00	0.00

```
ATOM   1432  CD  ARG    92       9.073   6.549 -19.656  1.00  0.00
ATOM   1433  HD2 ARG    92       8.361   6.135 -20.370  1.00  0.00
ATOM   1434  HD3 ARG    92       8.925   7.626 -19.579  1.00  0.00
ATOM   1435  NE  ARG    92      10.453   6.291 -20.154  1.00  0.00
ATOM   1436  HE  ARG    92      11.051   5.602 -19.719  1.00  0.00
ATOM   1437  CZ  ARG    92      10.914   6.959 -21.175  1.00  0.00
ATOM   1438  NH1 ARG    92      10.649   8.231 -21.295  1.00  0.00
ATOM   1439 HH11 ARG    92      10.087   8.694 -20.596  1.00  0.00
ATOM   1440 HH12 ARG    92      11.008   8.745 -22.087  1.00  0.00
ATOM   1441  NH2 ARG    92      11.642   6.356 -22.075  1.00  0.00
ATOM   1442 HH21 ARG    92      11.847   5.371 -21.978  1.00  0.00
ATOM   1443 HH22 ARG    92      11.998   6.876 -22.864  1.00  0.00
ATOM   1444  C   ARG    92       7.416   3.901 -16.803  1.00  0.00
ATOM   1445  O   ARG    92       6.983   4.951 -16.370  1.00  0.00
ATOM   1446  N   GLN    93       6.623   2.897 -17.057  1.00  0.00
ATOM   1447  H   GLN    93       6.975   2.021 -17.418  1.00  0.00
ATOM   1448  CA  GLN    93       5.159   3.024 -16.817  1.00  0.00
ATOM   1449  HA  GLN    93       4.802   3.995 -17.157  1.00  0.00
ATOM   1450  CB  GLN    93       4.420   1.931 -17.595  1.00  0.00
ATOM   1451  HB2 GLN    93       4.780   1.910 -18.624  1.00  0.00
ATOM   1452  HB3 GLN    93       4.604   0.963 -17.127  1.00  0.00
ATOM   1453  CG  GLN    93       2.918   2.225 -17.586  1.00  0.00
```

ATOM 1454 HG2 GLN 93 2.520 2.135 -16.575 1.00 0.00
ATOM 1455 HG3 GLN 93 2.728 3.229 -17.963 1.00 0.00
ATOM 1456 CD GLN 93 2.197 1.220 -18.487 1.00 0.00
ATOM 1457 OE1 GLN 93 2.826 0.482 -19.219 1.00 0.00
ATOM 1458 NE2 GLN 93 0.894 1.159 -18.463 1.00 0.00
ATOM 1459 HE21 GLN 93 0.412 0.498 -19.055 1.00 0.00
ATOM 1460 HE22 GLN 93 0.374 1.773 -17.853 1.00 0.00
ATOM 1461 C GLN 93 4.868 2.872 -15.322 1.00 0.00
ATOM 1462 O GLN 93 4.192 3.688 -14.727 1.00 0.00
ATOM 1463 N MET 94 5.372 1.835 -14.711 1.00 0.00
ATOM 1464 H MET 94 5.937 1.154 -15.199 1.00 0.00
ATOM 1465 CA MET 94 5.122 1.632 -13.256 1.00 0.00
ATOM 1466 HA MET 94 4.057 1.488 -13.072 1.00 0.00
ATOM 1467 CB MET 94 5.872 0.389 -12.776 1.00 0.00
ATOM 1468 HB2 MET 94 6.901 0.424 -13.136 1.00 0.00
ATOM 1469 HB3 MET 94 5.870 0.362 -11.686 1.00 0.00
ATOM 1470 CG MET 94 5.186 -0.866 -13.318 1.00 0.00
ATOM 1471 HG2 MET 94 4.866 -0.696 -14.346 1.00 0.00
ATOM 1472 HG3 MET 94 5.880 -1.707 -13.289 1.00 0.00
ATOM 1473 SD MET 94 3.740 -1.252 -12.300 1.00 0.00
ATOM 1474 CE MET 94 4.589 -1.311 -10.702 1.00 0.00
ATOM 1475 HE1 MET 94 5.053 -0.346 -10.500 1.00 0.00

ATOM 1476 HE2 MET 94 3.870 -1.540 -9.916 1.00 0.00

ATOM 1477 HE3 MET 94 5.357 -2.084 -10.726 1.00 0.00

ATOM 1478 C MET 94 5.615 2.853 -12.475 1.00 0.00

ATOM 1479 O MET 94 4.985 3.297 -11.537 1.00 0.00

ATOM 1480 N VAL 95 6.743 3.393 -12.851 1.00 0.00

ATOM 1481 H VAL 95 7.274 3.024 -13.627 1.00 0.00

ATOM 1482 CA VAL 95 7.282 4.581 -12.127 1.00 0.00

ATOM 1483 HA VAL 95 7.779 4.269 -11.208 1.00 0.00

ATOM 1484 CB VAL 95 8.300 5.300 -13.014 1.00 0.00

ATOM 1485 HB VAL 95 7.845 5.531 -13.977 1.00 0.00

ATOM 1486 CG1 VAL 95 8.742 6.597 -12.335 1.00 0.00

ATOM 1487 HG11 VAL 95 9.198 6.366 -11.372 1.00 0.00

ATOM 1488 HG12 VAL 95 9.468 7.109 -12.967 1.00 0.00

ATOM 1489 HG13 VAL 95 7.876 7.240 -12.181 1.00 0.00

ATOM 1490 CG2 VAL 95 9.517 4.397 -13.228 1.00 0.00

ATOM 1491 HG21 VAL 95 9.203 3.471 -13.712 1.00 0.00

ATOM 1492 HG22 VAL 95 10.243 4.908 -13.860 1.00 0.00

ATOM 1493 HG23 VAL 95 9.973 4.166 -12.265 1.00 0.00

ATOM 1494 C VAL 95 6.140 5.540 -11.780 1.00 0.00

ATOM 1495 O VAL 95 5.513 5.423 -10.746 1.00 0.00

ATOM 1496 N GLN 96 5.868 6.488 -12.633 1.00 0.00

ATOM 1497 H GLN 96 6.387 6.594 -13.493 1.00 0.00

ATOM	1498	CA	GLN	96	4.769	7.453	-12.347	1.00	0.00
ATOM	1499	HA	GLN	96	5.008	8.047	-11.465	1.00	0.00
ATOM	1500	CB	GLN	96	4.594	8.396	-13.540	1.00	0.00
ATOM	1501	HB2	GLN	96	4.310	7.819	-14.420	1.00	0.00
ATOM	1502	HB3	GLN	96	3.815	9.124	-13.316	1.00	0.00
ATOM	1503	CG	GLN	96	5.911	9.125	-13.812	1.00	0.00
ATOM	1504	HG2	GLN	96	6.248	9.649	-12.917	1.00	0.00
ATOM	1505	HG3	GLN	96	6.677	8.415	-14.126	1.00	0.00
ATOM	1506	CD	GLN	96	5.705	10.149	-14.930	1.00	0.00
ATOM	1507	OE1	GLN	96	4.985	11.114	-14.762	1.00	0.00
ATOM	1508	NE2	GLN	96	6.313	9.981	-16.073	1.00	0.00
ATOM	1509	HE21	GLN	96	6.182	10.653	-16.815	1.00	0.00
ATOM	1510	HE22	GLN	96	6.912	9.179	-16.210	1.00	0.00
ATOM	1511	C	GLN	96	3.468	6.691	-12.103	1.00	0.00
ATOM	1512	O	GLN	96	2.828	6.842	-11.081	1.00	0.00
ATOM	1513	N	GLN	97	3.072	5.874	-13.035	1.00	0.00
ATOM	1514	H	GLN	97	3.599	5.749	-13.887	1.00	0.00
ATOM	1515	CA	GLN	97	1.812	5.098	-12.864	1.00	0.00
ATOM	1516	HA	GLN	97	0.947	5.743	-13.014	1.00	0.00
ATOM	1517	CB	GLN	97	1.766	3.970	-13.897	1.00	0.00
ATOM	1518	HB2	GLN	97	2.092	4.349	-14.866	1.00	0.00
ATOM	1519	HB3	GLN	97	2.427	3.161	-13.584	1.00	0.00

ATOM 1520 CG GLN 97 0.334 3.443 -14.011 1.00 0.00
ATOM 1521 HG2 GLN 97 0.298 2.584 -14.682 1.00 0.00
ATOM 1522 HG3 GLN 97 -0.040 3.152 -13.029 1.00 0.00
ATOM 1523 CD GLN 97 -0.570 4.541 -14.573 1.00 0.00
ATOM 1524 OE1 GLN 97 -0.448 4.917 -15.722 1.00 0.00
ATOM 1525 NE2 GLN 97 -1.481 5.074 -13.805 1.00 0.00
ATOM 1526 HE21 GLN 97 -2.082 5.799 -14.169 1.00 0.00
ATOM 1527 HE22 GLN 97 -1.580 4.758 -12.851 1.00 0.00
ATOM 1528 C GLN 97 1.761 4.506 -11.454 1.00 0.00
ATOM 1529 O GLN 97 0.963 4.908 -10.630 1.00 0.00
ATOM 1530 N PHE 98 2.608 3.555 -11.168 1.00 0.00
ATOM 1531 H PHE 98 3.275 3.209 -11.843 1.00 0.00
ATOM 1532 CA PHE 98 2.600 2.946 -9.807 1.00 0.00
ATOM 1533 HA PHE 98 1.678 2.385 -9.655 1.00 0.00
ATOM 1534 CB PHE 98 3.786 1.989 -9.651 1.00 0.00
ATOM 1535 HB2 PHE 98 3.833 1.259 -10.460 1.00 0.00
ATOM 1536 HB3 PHE 98 4.737 2.518 -9.585 1.00 0.00
ATOM 1537 CG PHE 98 3.638 1.202 -8.366 1.00 0.00
ATOM 1538 CD1 PHE 98 2.438 1.250 -7.641 1.00 0.00
ATOM 1539 HD1 PHE 98 1.608 1.855 -8.004 1.00 0.00
ATOM 1540 CE1 PHE 98 2.307 0.522 -6.454 1.00 0.00
ATOM 1541 HE1 PHE 98 1.373 0.560 -5.893 1.00 0.00

ATOM	1542	CZ	PHE	98	3.372	-0.255	-5.986	1.00	0.00
ATOM	1543	HZ	PHE	98	3.269	-0.822	-5.060	1.00	0.00
ATOM	1544	CE2	PHE	98	4.571	-0.305	-6.707	1.00	0.00
ATOM	1545	HE2	PHE	98	5.401	-0.911	-6.343	1.00	0.00
ATOM	1546	CD2	PHE	98	4.704	0.423	-7.896	1.00	0.00
ATOM	1547	HD2	PHE	98	5.638	0.384	-8.457	1.00	0.00
ATOM	1548	C	PHE	98	2.699	4.055	-8.761	1.00	0.00
ATOM	1549	O	PHE	98	2.069	4.003	-7.724	1.00	0.00
ATOM	1550	N	LYS	99	3.484	5.061	-9.029	1.00	0.00
ATOM	1551	H	LYS	99	4.010	5.109	-9.890	1.00	0.00
ATOM	1552	CA	LYS	99	3.621	6.178	-8.054	1.00	0.00
ATOM	1553	HA	LYS	99	3.915	5.792	-7.078	1.00	0.00
ATOM	1554	CB	LYS	99	4.696	7.152	-8.538	1.00	0.00
ATOM	1555	HB2	LYS	99	5.609	6.602	-8.766	1.00	0.00
ATOM	1556	HB3	LYS	99	4.347	7.662	-9.436	1.00	0.00
ATOM	1557	CG	LYS	99	4.981	8.183	-7.443	1.00	0.00
ATOM	1558	HG2	LYS	99	4.054	8.684	-7.164	1.00	0.00
ATOM	1559	HG3	LYS	99	5.400	7.681	-6.570	1.00	0.00
ATOM	1560	CD	LYS	99	5.981	9.216	-7.966	1.00	0.00
ATOM	1561	HD2	LYS	99	6.928	8.725	-8.190	1.00	0.00
ATOM	1562	HD3	LYS	99	5.588	9.676	-8.872	1.00	0.00
ATOM	1563	CE	LYS	99	6.204	10.293	-6.902	1.00	0.00

ATOM	1564	HE2	LYS	99	6.024	11.276	-7.336	1.00	0.00
ATOM	1565	HE3	LYS	99	5.517	10.131	-6.071	1.00	0.00
ATOM	1566	NZ	LYS	99	7.607	10.219	-6.407	1.00	0.00
ATOM	1567	HZ1	LYS	99	8.244	10.369	-7.177	1.00	0.00
ATOM	1568	HZ2	LYS	99	7.754	10.929	-5.705	1.00	0.00
ATOM	1569	HZ3	LYS	99	7.775	9.308	-6.004	1.00	0.00
ATOM	1570	C	LYS	99	2.283	6.910	-7.939	1.00	0.00
ATOM	1571	O	LYS	99	1.919	7.400	-6.889	1.00	0.00
ATOM	1572	N	ARG	100	1.546	6.981	-9.012	1.00	0.00
ATOM	1573	H	ARG	100	1.844	6.575	-9.888	1.00	0.00
ATOM	1574	CA	ARG	100	0.228	7.673	-8.966	1.00	0.00
ATOM	1575	HA	ARG	100	0.340	8.669	-8.538	1.00	0.00
ATOM	1576	CB	ARG	100	-0.330	7.810	-10.384	1.00	0.00
ATOM	1577	HB2	ARG	100	-0.148	6.889	-10.938	1.00	0.00
ATOM	1578	HB3	ARG	100	-1.402	7.999	-10.337	1.00	0.00
ATOM	1579	CG	ARG	100	0.363	8.976	-11.092	1.00	0.00
ATOM	1580	HG2	ARG	100	0.216	9.889	-10.516	1.00	0.00
ATOM	1581	HG3	ARG	100	1.430	8.767	-11.176	1.00	0.00
ATOM	1582	CD	ARG	100	-0.234	9.153	-12.490	1.00	0.00
ATOM	1583	HD2	ARG	100	-0.327	8.186	-12.986	1.00	0.00
ATOM	1584	HD3	ARG	100	-1.215	9.623	-12.425	1.00	0.00
ATOM	1585	NE	ARG	100	0.660	10.024	-13.305	1.00	0.00

```
ATOM   1586  HE   ARG   100       1.566   9.705 -13.618  1.00  0.00
ATOM   1587  CZ   ARG   100       0.278  11.228 -13.630  1.00  0.00
ATOM   1588  NH1 ARG   100       0.904  12.265 -13.144  1.00  0.00
ATOM   1589 HH11 ARG   100       1.684  12.131 -12.516  1.00  0.00
ATOM   1590 HH12 ARG   100       0.606  13.195 -13.398  1.00  0.00
ATOM   1591  NH2 ARG   100      -0.730  11.396 -14.441  1.00  0.00
ATOM   1592 HH21 ARG   100      -1.212  10.591 -14.816  1.00  0.00
ATOM   1593 HH22 ARG   100      -1.023  12.329 -14.691  1.00  0.00
ATOM   1594  C    ARG   100      -0.736   6.851  -8.112  1.00  0.00
ATOM   1595  O    ARG   100      -1.337   7.350  -7.181  1.00  0.00
ATOM   1596  N    GLU   101      -0.880   5.588  -8.411  1.00  0.00
ATOM   1597  H    GLU   101      -0.381   5.162  -9.179  1.00  0.00
ATOM   1598  CA   GLU   101      -1.795   4.734  -7.605  1.00  0.00
ATOM   1599  HA   GLU   101      -2.809   5.130  -7.640  1.00  0.00
ATOM   1600  CB   GLU   101      -1.805   3.314  -8.174  1.00  0.00
ATOM   1601  HB2 GLU   101      -0.784   2.934  -8.221  1.00  0.00
ATOM   1602  HB3 GLU   101      -2.402   2.669  -7.530  1.00  0.00
ATOM   1603  CG   GLU   101      -2.408   3.329  -9.580  1.00  0.00
ATOM   1604  HG2 GLU   101      -1.737   3.857 -10.257  1.00  0.00
ATOM   1605  HG3 GLU   101      -2.544   2.305  -9.930  1.00  0.00
ATOM   1606  CD   GLU   101      -3.762   4.041  -9.546  1.00  0.00
ATOM   1607  OE1 GLU   101      -4.472   3.877  -8.568  1.00  0.00
```

ATOM 1608 OE2 GLU 101 -4.067 4.738 -10.501 1.00 0.00
ATOM 1609 C GLU 101 -1.293 4.703 -6.163 1.00 0.00
ATOM 1610 O GLU 101 -2.062 4.719 -5.222 1.00 0.00
ATOM 1611 N LEU 102 0.000 4.660 -5.984 1.00 0.00
ATOM 1612 H LEU 102 0.642 4.644 -6.764 1.00 0.00
ATOM 1613 CA LEU 102 0.563 4.631 -4.607 1.00 0.00
ATOM 1614 HA LEU 102 0.260 3.719 -4.093 1.00 0.00
ATOM 1615 CB LEU 102 2.091 4.665 -4.682 1.00 0.00
ATOM 1616 HB2 LEU 102 2.404 4.607 -5.725 1.00 0.00
ATOM 1617 HB3 LEU 102 2.454 5.595 -4.246 1.00 0.00
ATOM 1618 CG LEU 102 2.673 3.478 -3.908 1.00 0.00
ATOM 1619 HG LEU 102 2.590 3.667 -2.838 1.00 0.00
ATOM 1620 CD1 LEU 102 1.902 2.204 -4.262 1.00 0.00
ATOM 1621 HD11 LEU 102 1.986 2.013 -5.332 1.00 0.00
ATOM 1622 HD12 LEU 102 2.319 1.361 -3.710 1.00 0.00
ATOM 1623 HD13 LEU 102 0.852 2.328 -3.997 1.00 0.00
ATOM 1624 CD2 LEU 102 4.146 3.301 -4.282 1.00 0.00
ATOM 1625 HD21 LEU 102 4.697 4.206 -4.028 1.00 0.00
ATOM 1626 HD22 LEU 102 4.562 2.456 -3.732 1.00 0.00
ATOM 1627 HD23 LEU 102 4.230 3.113 -5.353 1.00 0.00
ATOM 1628 C LEU 102 0.062 5.846 -3.825 1.00 0.00
ATOM 1629 O LEU 102 -0.227 5.762 -2.650 1.00 0.00

ATOM	1630	N	MET	103	-0.050	6.976	-4.470	1.00	0.00
ATOM	1631	H	MET	103	0.184	7.057	-5.449	1.00	0.00
ATOM	1632	CA	MET	103	-0.539	8.189	-3.757	1.00	0.00
ATOM	1633	HA	MET	103	0.094	8.401	-2.895	1.00	0.00
ATOM	1634	CB	MET	103	-0.497	9.391	-4.702	1.00	0.00
ATOM	1635	HB2	MET	103	-0.949	9.120	-5.656	1.00	0.00
ATOM	1636	HB3	MET	103	-1.049	10.220	-4.261	1.00	0.00
ATOM	1637	CG	MET	103	0.958	9.810	-4.928	1.00	0.00
ATOM	1638	HG2	MET	103	1.437	10.011	-3.970	1.00	0.00
ATOM	1639	HG3	MET	103	1.496	9.013	-5.442	1.00	0.00
ATOM	1640	SD	MET	103	1.003	11.309	-5.942	1.00	0.00
ATOM	1641	CE	MET	103	0.744	12.497	-4.603	1.00	0.00
ATOM	1642	HE1	MET	103	1.550	12.405	-3.874	1.00	0.00
ATOM	1643	HE2	MET	103	0.737	13.508	-5.010	1.00	0.00
ATOM	1644	HE3	MET	103	-0.210	12.294	-4.116	1.00	0.00
ATOM	1645	C	MET	103	-1.978	7.951	-3.297	1.00	0.00
ATOM	1646	O	MET	103	-2.407	8.454	-2.278	1.00	0.00
ATOM	1647	N	SER	104	-2.725	7.181	-4.041	1.00	0.00
ATOM	1648	H	SER	104	-2.372	6.759	-4.888	1.00	0.00
ATOM	1649	CA	SER	104	-4.134	6.904	-3.649	1.00	0.00
ATOM	1650	HA	SER	104	-4.598	7.803	-3.246	1.00	0.00
ATOM	1651	CB	SER	104	-4.926	6.454	-4.878	1.00	0.00

ATOM	1652	HB2	SER	104	-5.967	6.291	-4.601	1.00	0.00
ATOM	1653	HB3	SER	104	-4.871	7.223	-5.648	1.00	0.00
ATOM	1654	OG	SER	104	-4.375	5.244	-5.378	1.00	0.00
ATOM	1655	HG	SER	104	-4.871	4.961	-6.149	1.00	0.00
ATOM	1656	C	SER	104	-4.158	5.797	-2.593	1.00	0.00
ATOM	1657	O	SER	104	-5.016	5.763	-1.734	1.00	0.00
ATOM	1658	N	LEU	105	-3.224	4.887	-2.654	1.00	0.00
ATOM	1659	H	LEU	105	-2.513	4.907	-3.371	1.00	0.00
ATOM	1660	CA	LEU	105	-3.192	3.779	-1.659	1.00	0.00
ATOM	1661	HA	LEU	105	-3.940	3.026	-1.908	1.00	0.00
ATOM	1662	CB	LEU	105	-1.811	3.118	-1.671	1.00	0.00
ATOM	1663	HB2	LEU	105	-1.149	3.682	-2.328	1.00	0.00
ATOM	1664	HB3	LEU	105	-1.401	3.112	-0.661	1.00	0.00
ATOM	1665	CG	LEU	105	-1.931	1.679	-2.181	1.00	0.00
ATOM	1666	HG	LEU	105	-0.993	1.152	-2.004	1.00	0.00
ATOM	1667	CD1	LEU	105	-3.065	0.963	-1.443	1.00	0.00
ATOM	1668	HD11	LEU	105	-4.003	1.488	-1.621	1.00	0.00
ATOM	1669	HD12	LEU	105	-3.147	-0.061	-1.808	1.00	0.00
ATOM	1670	HD13	LEU	105	-2.852	0.950	-0.374	1.00	0.00
ATOM	1671	CD2	LEU	105	-2.227	1.693	-3.681	1.00	0.00
ATOM	1672	HD21	LEU	105	-1.417	2.198	-4.207	1.00	0.00
ATOM	1673	HD22	LEU	105	-2.312	0.669	-4.045	1.00	0.00

ATOM	1674	HD23	LEU	105	-3.163	2.221	-3.862	1.00	0.00
ATOM	1675	C	LEU	105	-3.484	4.324	-0.259	1.00	0.00
ATOM	1676	O	LEU	105	-4.327	3.809	0.448	1.00	0.00
ATOM	1677	N	PRO	106	-2.776	5.390	0.152	1.00	0.00
ATOM	1678	CD	PRO	106	-1.740	6.088	-0.630	1.00	0.00
ATOM	1679	HD2	PRO	106	-2.090	6.241	-1.651	1.00	0.00
ATOM	1680	HD3	PRO	106	-0.827	5.492	-0.646	1.00	0.00
ATOM	1681	CG	PRO	106	-1.569	7.378	0.122	1.00	0.00
ATOM	1682	HG2	PRO	106	-2.256	8.126	-0.274	1.00	0.00
ATOM	1683	HG3	PRO	106	-0.544	7.732	0.012	1.00	0.00
ATOM	1684	CB	PRO	106	-1.872	7.060	1.545	1.00	0.00
ATOM	1685	HB2	PRO	106	-2.258	7.962	2.019	1.00	0.00
ATOM	1686	HB3	PRO	106	-1.027	6.680	2.121	1.00	0.00
ATOM	1687	CA	PRO	106	-2.971	6.000	1.471	1.00	0.00
ATOM	1688	HA	PRO	106	-2.873	5.225	2.231	1.00	0.00
ATOM	1689	C	PRO	106	-4.350	6.649	1.581	1.00	0.00
ATOM	1690	O	PRO	106	-4.755	7.108	2.631	1.00	0.00
ATOM	1691	N	GLN	107	-5.079	6.679	0.500	1.00	0.00
ATOM	1692	H	GLN	107	-4.742	6.297	-0.373	1.00	0.00
ATOM	1693	CA	GLN	107	-6.437	7.283	0.528	1.00	0.00
ATOM	1694	HA	GLN	107	-6.537	7.945	1.388	1.00	0.00
ATOM	1695	CB	GLN	107	-6.663	8.099	-0.746	1.00	0.00

ATOM	1696	HB2	GLN	107	-5.880	8.851	-0.840	1.00	0.00
ATOM	1697	HB3	GLN	107	-6.635	7.437	-1.612	1.00	0.00
ATOM	1698	CG	GLN	107	-8.027	8.789	-0.675	1.00	0.00
ATOM	1699	HG2	GLN	107	-8.819	8.048	-0.565	1.00	0.00
ATOM	1700	HG3	GLN	107	-8.058	9.480	0.167	1.00	0.00
ATOM	1701	CD	GLN	107	-8.269	9.576	-1.964	1.00	0.00
ATOM	1702	OE1	GLN	107	-7.382	9.708	-2.785	1.00	0.00
ATOM	1703	NE2	GLN	107	-9.440	10.110	-2.179	1.00	0.00
ATOM	1704	HE21	GLN	107	-9.606	10.630	-3.028	1.00	0.00
ATOM	1705	HE22	GLN	107	-10.175	10.000	-1.495	1.00	0.00
ATOM	1706	C	GLN	107	-7.477	6.168	0.614	1.00	0.00
ATOM	1707	O	GLN	107	-8.380	6.207	1.425	1.00	0.00
ATOM	1708	N	GLN	108	-7.359	5.173	-0.222	1.00	0.00
ATOM	1709	H	GLN	108	-6.612	5.136	-0.901	1.00	0.00
ATOM	1710	CA	GLN	108	-8.341	4.053	-0.191	1.00	0.00
ATOM	1711	HA	GLN	108	-9.306	4.384	-0.574	1.00	0.00
ATOM	1712	CB	GLN	108	-7.834	2.907	-1.069	1.00	0.00
ATOM	1713	HB2	GLN	108	-7.634	3.279	-2.074	1.00	0.00
ATOM	1714	HB3	GLN	108	-6.916	2.501	-0.643	1.00	0.00
ATOM	1715	CG	GLN	108	-8.895	1.807	-1.135	1.00	0.00
ATOM	1716	HG2	GLN	108	-9.175	1.488	-0.131	1.00	0.00
ATOM	1717	HG3	GLN	108	-9.779	2.166	-1.662	1.00	0.00

ATOM	1718	CD	GLN	108	-8.331	0.602	-1.891	1.00	0.00
ATOM	1719	OE1	GLN	108	-7.422	-0.053	-1.423	1.00	0.00
ATOM	1720	NE2	GLN	108	-8.838	0.279	-3.050	1.00	0.00
ATOM	1721	HE21	GLN	108	-8.471	-0.516	-3.555	1.00	0.00
ATOM	1722	HE22	GLN	108	-9.595	0.824	-3.436	1.00	0.00
ATOM	1723	C	GLN	108	-8.508	3.559	1.246	1.00	0.00
ATOM	1724	O	GLN	108	-9.552	3.068	1.628	1.00	0.00
ATOM	1725	N	CYX	109	-7.487	3.685	2.048	1.00	0.00
ATOM	1726	H	CYX	109	-6.615	4.090	1.738	1.00	0.00
ATOM	1727	CA	CYX	109	-7.587	3.222	3.461	1.00	0.00
ATOM	1728	HA	CYX	109	-8.630	3.058	3.731	1.00	0.00
ATOM	1729	CB	CYX	109	-6.826	1.902	3.627	1.00	0.00
ATOM	1730	HB2	CYX	109	-6.378	1.862	4.620	1.00	0.00
ATOM	1731	HB3	CYX	109	-7.515	1.066	3.506	1.00	0.00
ATOM	1732	SG	CYX	109	-5.484	1.809	2.413	1.00	0.00
ATOM	1733	C	CYX	109	-6.982	4.281	4.387	1.00	0.00
ATOM	1734	O	CYX	109	-6.512	3.978	5.466	1.00	0.00
ATOM	1735	N	ASN	110	-6.994	5.518	3.974	1.00	0.00
ATOM	1736	H	ASN	110	-7.384	5.773	3.078	1.00	0.00
ATOM	1737	CA	ASN	110	-6.423	6.597	4.829	1.00	0.00
ATOM	1738	HA	ASN	110	-6.235	7.491	4.234	1.00	0.00
ATOM	1739	CB	ASN	110	-7.416	6.948	5.939	1.00	0.00

ATOM	1740	HB2	ASN	110	-7.512	6.115	6.636	1.00	0.00
ATOM	1741	HB3	ASN	110	-7.083	7.835	6.477	1.00	0.00
ATOM	1742	CG	ASN	110	-8.788	7.234	5.326	1.00	0.00
ATOM	1743	OD1	ASN	110	-8.894	7.959	4.357	1.00	0.00
ATOM	1744	ND2	ASN	110	-9.851	6.691	5.854	1.00	0.00
ATOM	1745	HD21	ASN	110	-10.760	6.875	5.454	1.00	0.00
ATOM	1746	HD22	ASN	110	-9.759	6.088	6.660	1.00	0.00
ATOM	1747	C	ASN	110	-5.112	6.117	5.453	1.00	0.00
ATOM	1748	O	ASN	110	-4.872	6.295	6.631	1.00	0.00
ATOM	1749	N	PHE	111	-4.259	5.508	4.673	1.00	0.00
ATOM	1750	H	PHE	111	-4.449	5.351	3.693	1.00	0.00
ATOM	1751	CA	PHE	111	-2.965	5.021	5.227	1.00	0.00
ATOM	1752	HA	PHE	111	-3.135	4.454	6.142	1.00	0.00
ATOM	1753	CB	PHE	111	-2.284	4.108	4.206	1.00	0.00
ATOM	1754	HB2	PHE	111	-2.995	3.671	3.505	1.00	0.00
ATOM	1755	HB3	PHE	111	-1.499	4.623	3.653	1.00	0.00
ATOM	1756	CG	PHE	111	-1.622	2.956	4.924	1.00	0.00
ATOM	1757	CD1	PHE	111	-2.377	1.836	5.294	1.00	0.00
ATOM	1758	HD1	PHE	111	-3.441	1.794	5.063	1.00	0.00
ATOM	1759	CE1	PHE	111	-1.763	0.769	5.961	1.00	0.00
ATOM	1760	HE1	PHE	111	-2.351	-0.102	6.251	1.00	0.00
ATOM	1761	CZ	PHE	111	-0.395	0.820	6.256	1.00	0.00

ATOM	1762	HZ	PHE	111	0.083	-0.012	6.774	1.00	0.00
ATOM	1763	CE2	PHE	111	0.359	1.940	5.885	1.00	0.00
ATOM	1764	HE2	PHE	111	1.424	1.981	6.115	1.00	0.00
ATOM	1765	CD2	PHE	111	-0.255	3.007	5.219	1.00	0.00
ATOM	1766	HD2	PHE	111	0.332	3.878	4.930	1.00	0.00
ATOM	1767	C	PHE	111	-2.058	6.214	5.531	1.00	0.00
ATOM	1768	O	PHE	111	-0.972	6.063	6.053	1.00	0.00
ATOM	1769	N	ARG	112	-2.495	7.402	5.210	1.00	0.00
ATOM	1770	H	ARG	112	-3.397	7.537	4.777	1.00	0.00
ATOM	1771	CA	ARG	112	-1.655	8.602	5.484	1.00	0.00
ATOM	1772	HA	ARG	112	-0.804	8.631	4.803	1.00	0.00
ATOM	1773	CB	ARG	112	-2.490	9.868	5.278	1.00	0.00
ATOM	1774	HB2	ARG	112	-3.375	9.827	5.913	1.00	0.00
ATOM	1775	HB3	ARG	112	-1.895	10.742	5.541	1.00	0.00
ATOM	1776	CG	ARG	112	-2.918	9.964	3.811	1.00	0.00
ATOM	1777	HG2	ARG	112	-2.036	9.931	3.171	1.00	0.00
ATOM	1778	HG3	ARG	112	-3.574	9.128	3.567	1.00	0.00
ATOM	1779	CD	ARG	112	-3.664	11.280	3.584	1.00	0.00
ATOM	1780	HD2	ARG	112	-3.015	12.125	3.813	1.00	0.00
ATOM	1781	HD3	ARG	112	-3.999	11.350	2.549	1.00	0.00
ATOM	1782	NE	ARG	112	-4.855	11.336	4.477	1.00	0.00
ATOM	1783	HE	ARG	112	-5.553	10.605	4.472	1.00	0.00

ATOM	1784	CZ	ARG	112	-5.015	12.345	5.290	1.00	0.00
ATOM	1785	NH1	ARG	112	-4.566	12.273	6.513	1.00	0.00
ATOM	1786	HH11	ARG	112	-4.095	11.436	6.827	1.00	0.00
ATOM	1787	HH12	ARG	112	-4.691	13.054	7.140	1.00	0.00
ATOM	1788	NH2	ARG	112	-5.623	13.423	4.880	1.00	0.00
ATOM	1789	HH21	ARG	112	-5.969	13.474	3.933	1.00	0.00
ATOM	1790	HH22	ARG	112	-5.745	14.201	5.512	1.00	0.00
ATOM	1791	C	ARG	112	-1.156	8.551	6.929	1.00	0.00
ATOM	1792	O	ARG	112	-1.769	7.948	7.787	1.00	0.00
ATOM	1793	N	ALA	113	-0.046	9.181	7.207	1.00	0.00
ATOM	1794	H	ALA	113	0.471	9.685	6.501	1.00	0.00
ATOM	1795	CA	ALA	113	0.489	9.166	8.597	1.00	0.00
ATOM	1796	HA	ALA	113	-0.278	8.898	9.324	1.00	0.00
ATOM	1797	CB	ALA	113	1.629	8.151	8.694	1.00	0.00
ATOM	1798	HB1	ALA	113	2.409	8.410	7.978	1.00	0.00
ATOM	1799	HB2	ALA	113	2.043	8.163	9.702	1.00	0.00
ATOM	1800	HB3	ALA	113	1.249	7.154	8.470	1.00	0.00
ATOM	1801	C	ALA	113	1.013	10.557	8.958	1.00	0.00
ATOM	1802	O	ALA	113	1.233	11.389	8.100	1.00	0.00
ATOM	1803	N	PRO	114	1.216	10.808	10.260	1.00	0.00
ATOM	1804	CD	PRO	114	0.973	9.854	11.355	1.00	0.00
ATOM	1805	HD2	PRO	114	1.437	8.897	11.113	1.00	0.00

ATOM 1806 HD3 PRO 114 -0.100 9.714 11.483 1.00 0.00
ATOM 1807 CG PRO 114 1.610 10.520 12.541 1.00 0.00
ATOM 1808 HG2 PRO 114 2.634 10.162 12.650 1.00 0.00
ATOM 1809 HG3 PRO 114 1.043 10.277 13.440 1.00 0.00
ATOM 1810 CB PRO 114 1.572 11.986 12.266 1.00 0.00
ATOM 1811 HB2 PRO 114 2.430 12.447 12.755 1.00 0.00
ATOM 1812 HB3 PRO 114 0.659 12.484 12.590 1.00 0.00
ATOM 1813 CA PRO 114 1.717 12.098 10.749 1.00 0.00
ATOM 1814 HA PRO 114 1.113 12.890 10.308 1.00 0.00
ATOM 1815 C PRO 114 3.187 12.312 10.379 1.00 0.00
ATOM 1816 O PRO 114 3.717 13.398 10.502 1.00 0.00
ATOM 1817 N GLN 115 3.849 11.283 9.924 1.00 0.00
ATOM 1818 H GLN 115 3.414 10.377 9.819 1.00 0.00
ATOM 1819 CA GLN 115 5.282 11.426 9.544 1.00 0.00
ATOM 1820 HA GLN 115 5.697 12.340 9.968 1.00 0.00
ATOM 1821 CB GLN 115 6.074 10.233 10.084 1.00 0.00
ATOM 1822 HB2 GLN 115 5.712 9.315 9.620 1.00 0.00
ATOM 1823 HB3 GLN 115 7.131 10.363 9.852 1.00 0.00
ATOM 1824 CG GLN 115 5.892 10.143 11.601 1.00 0.00
ATOM 1825 HG2 GLN 115 4.844 9.971 11.846 1.00 0.00
ATOM 1826 HG3 GLN 115 6.497 9.333 12.009 1.00 0.00
ATOM 1827 CD GLN 115 6.336 11.457 12.246 1.00 0.00

ATOM	1828	OE1	GLN	115	7.431	11.926	12.003	1.00	0.00
ATOM	1829	NE2	GLN	115	5.529	12.074	13.064	1.00	0.00
ATOM	1830	HE21	GLN	115	5.817	12.942	13.493	1.00	0.00
ATOM	1831	HE22	GLN	115	4.621	11.680	13.264	1.00	0.00
ATOM	1832	C	GLN	115	5.403	11.471	8.020	1.00	0.00
ATOM	1833	O	GLN	115	4.591	10.914	7.306	1.00	0.00
ATOM	1834	N	ARG	116	6.409	12.128	7.514	1.00	0.00
ATOM	1835	H	ARG	116	7.087	12.593	8.100	1.00	0.00
ATOM	1836	CA	ARG	116	6.580	12.207	6.036	1.00	0.00
ATOM	1837	HA	ARG	116	5.754	11.704	5.532	1.00	0.00
ATOM	1838	CB	ARG	116	6.598	13.674	5.602	1.00	0.00
ATOM	1839	HB2	ARG	116	7.406	14.195	6.115	1.00	0.00
ATOM	1840	HB3	ARG	116	6.755	13.733	4.525	1.00	0.00
ATOM	1841	CG	ARG	116	5.261	14.327	5.959	1.00	0.00
ATOM	1842	HG2	ARG	116	4.450	13.782	5.476	1.00	0.00
ATOM	1843	HG3	ARG	116	5.122	14.302	7.040	1.00	0.00
ATOM	1844	CD	ARG	116	5.258	15.779	5.477	1.00	0.00
ATOM	1845	HD2	ARG	116	6.172	16.283	5.791	1.00	0.00
ATOM	1846	HD3	ARG	116	5.179	15.816	4.390	1.00	0.00
ATOM	1847	NE	ARG	116	4.091	16.495	6.066	1.00	0.00
ATOM	1848	HE	ARG	116	4.185	17.065	6.894	1.00	0.00
ATOM	1849	CZ	ARG	116	2.916	16.395	5.509	1.00	0.00

```
ATOM   1850  NH1 ARG   116       2.699  15.483   4.601  1.00  0.00
ATOM   1851 HH11 ARG   116       3.444  14.855   4.332  1.00  0.00
ATOM   1852 HH12 ARG   116       1.788  15.408   4.172  1.00  0.00
ATOM   1853  NH2 ARG   116       1.955  17.205   5.862  1.00  0.00
ATOM   1854 HH21 ARG   116       2.125  17.908   6.567  1.00  0.00
ATOM   1855 HH22 ARG   116       1.047  17.124   5.429  1.00  0.00
ATOM   1856  C   ARG   116       7.898  11.541   5.637  1.00  0.00
ATOM   1857  O   ARG   116       8.843  11.509   6.399  1.00  0.00
ATOM   1858  N   CYX   117       7.968  11.006   4.449  1.00  0.00
ATOM   1859  H   CYX   117       7.186  11.028   3.810  1.00  0.00
ATOM   1860  CA  CYX   117       9.224  10.342   4.004  1.00  0.00
ATOM   1861  HA  CYX   117       9.955  10.334   4.813  1.00  0.00
ATOM   1862  CB  CYX   117       8.923   8.896   3.603  1.00  0.00
ATOM   1863  HB2 CYX   117       8.150   8.884   2.835  1.00  0.00
ATOM   1864  HB3 CYX   117       9.828   8.430   3.213  1.00  0.00
ATOM   1865  SG  CYX   117       8.293   7.991   5.038  1.00  0.00
ATOM   1866  C   CYX   117       9.800  11.093   2.802  1.00  0.00
ATOM   1867  O   CYX   117       9.103  11.811   2.114  1.00  0.00
ATOM   1868  N   ASP   118      11.070  10.932   2.543  1.00  0.00
ATOM   1869  H   ASP   118      11.657  10.337   3.110  1.00  0.00
ATOM   1870  CA  ASP   118      11.688  11.637   1.385  1.00  0.00
ATOM   1871  HA  ASP   118      11.395  12.686   1.381  1.00  0.00
```

ATOM 1872 CB ASP 118 13.212 11.556 1.493 1.00 0.00
ATOM 1873 HB2 ASP 118 13.523 10.511 1.505 1.00 0.00
ATOM 1874 HB3 ASP 118 13.663 12.058 0.637 1.00 0.00
ATOM 1875 CG ASP 118 13.669 12.238 2.784 1.00 0.00
ATOM 1876 OD1 ASP 118 12.875 12.963 3.361 1.00 0.00
ATOM 1877 OD2 ASP 118 14.805 12.022 3.175 1.00 0.00
ATOM 1878 C ASP 118 11.232 10.975 0.083 1.00 0.00
ATOM 1879 O ASP 118 11.019 9.781 0.025 1.00 0.00
ATOM 1880 N LEU 119 11.078 11.743 -0.961 1.00 0.00
ATOM 1881 H LEU 119 11.253 12.736 -0.920 1.00 0.00
ATOM 1882 CA LEU 119 10.633 11.157 -2.256 1.00 0.00
ATOM 1883 HA LEU 119 9.821 10.449 -2.092 1.00 0.00
ATOM 1884 CB LEU 119 10.131 12.274 -3.173 1.00 0.00
ATOM 1885 HB2 LEU 119 10.832 13.108 -3.146 1.00 0.00
ATOM 1886 HB3 LEU 119 10.050 11.900 -4.194 1.00 0.00
ATOM 1887 CG LEU 119 8.757 12.747 -2.693 1.00 0.00
ATOM 1888 HG LEU 119 8.150 11.883 -2.421 1.00 0.00
ATOM 1889 CD1 LEU 119 8.928 13.655 -1.474 1.00 0.00
ATOM 1890 HD11 LEU 119 9.535 14.518 -1.746 1.00 0.00
ATOM 1891 HD12 LEU 119 7.950 13.992 -1.132 1.00 0.00
ATOM 1892 HD13 LEU 119 9.422 13.102 -0.675 1.00 0.00
ATOM 1893 CD2 LEU 119 8.062 13.522 -3.814 1.00 0.00

ATOM	1894	HD21	LEU	119	7.940	12.874	-4.682	1.00	0.00
ATOM	1895	HD22	LEU	119	7.084	13.858	-3.471	1.00	0.00
ATOM	1896	HD23	LEU	119	8.667	14.386	-4.088	1.00	0.00
ATOM	1897	C	LEU	119	11.807	10.438	-2.924	1.00	0.00
ATOM	1898	O	LEU	119	11.626	9.592	-3.776	1.00	0.00
ATOM	1899	N	ASP	120	13.012	10.766	-2.542	1.00	0.00
ATOM	1900	H	ASP	120	13.173	11.469	-1.835	1.00	0.00
ATOM	1901	CA	ASP	120	14.194	10.098	-3.156	1.00	0.00
ATOM	1902	HA	ASP	120	14.138	10.155	-4.243	1.00	0.00
ATOM	1903	CB	ASP	120	15.474	10.798	-2.695	1.00	0.00
ATOM	1904	HB2	ASP	120	16.337	10.326	-3.165	1.00	0.00
ATOM	1905	HB3	ASP	120	15.436	11.849	-2.979	1.00	0.00
ATOM	1906	CG	ASP	120	15.596	10.685	-1.174	1.00	0.00
ATOM	1907	OD1	ASP	120	14.586	10.441	-0.534	1.00	0.00
ATOM	1908	OD2	ASP	120	16.698	10.844	-0.675	1.00	0.00
ATOM	1909	C	ASP	120	14.231	8.630	-2.727	1.00	0.00
ATOM	1910	O	ASP	120	14.690	7.772	-3.454	1.00	0.00
ATOM	1911	N	VAL	121	13.753	8.335	-1.549	1.00	0.00
ATOM	1912	H	VAL	121	13.371	9.042	-0.939	1.00	0.00
ATOM	1913	CA	VAL	121	13.762	6.922	-1.074	1.00	0.00
ATOM	1914	HA	VAL	121	14.787	6.569	-0.954	1.00	0.00
ATOM	1915	CB	VAL	121	13.054	6.836	0.280	1.00	0.00

```
ATOM   1916  HB  VAL   121      12.093   7.346   0.221  1.00  0.00
ATOM   1917  CG1 VAL   121      12.829   5.367   0.646  1.00  0.00
ATOM   1918 HG11 VAL   121      13.790   4.855   0.706  1.00  0.00
ATOM   1919 HG12 VAL   121      12.325   5.305   1.610  1.00  0.00
ATOM   1920 HG13 VAL   121      12.212   4.892  -0.117  1.00  0.00
ATOM   1921  CG2 VAL   121      13.921   7.501   1.351  1.00  0.00
ATOM   1922 HG21 VAL   121      14.082   8.547   1.091  1.00  0.00
ATOM   1923 HG22 VAL   121      13.418   7.440   2.316  1.00  0.00
ATOM   1924 HG23 VAL   121      14.882   6.989   1.410  1.00  0.00
ATOM   1925  C   VAL   121      13.032   6.038  -2.088  1.00  0.00
ATOM   1926  O   VAL   121      13.505   4.983  -2.460  1.00  0.00
ATOM   1927  N   SER   122      11.884   6.461  -2.539  1.00  0.00
ATOM   1928  H   SER   122      11.485   7.337  -2.234  1.00  0.00
ATOM   1929  CA  SER   122      11.125   5.645  -3.529  1.00  0.00
ATOM   1930  HA  SER   122      10.867   4.676  -3.101  1.00  0.00
ATOM   1931  CB  SER   122       9.833   6.372  -3.908  1.00  0.00
ATOM   1932  HB2 SER   122      10.076   7.303  -4.420  1.00  0.00
ATOM   1933  HB3 SER   122       9.240   5.739  -4.568  1.00  0.00
ATOM   1934  OG  SER   122       9.089   6.658  -2.733  1.00  0.00
ATOM   1935  HG  SER   122       8.279   7.114  -2.971  1.00  0.00
ATOM   1936  C   SER   122      11.979   5.437  -4.781  1.00  0.00
ATOM   1937  O   SER   122      11.927   4.403  -5.416  1.00  0.00
```

ATOM	1938	N	GLY	123	12.766	6.414	-5.143	1.00	0.00
ATOM	1939	H	GLY	123	12.815	7.277	-4.621	1.00	0.00
ATOM	1940	CA	GLY	123	13.622	6.271	-6.354	1.00	0.00
ATOM	1941	HA2	GLY	123	13.132	5.623	-7.081	1.00	0.00
ATOM	1942	HA3	GLY	123	13.800	7.249	-6.801	1.00	0.00
ATOM	1943	C	GLY	123	14.964	5.652	-5.961	1.00	0.00
ATOM	1944	O	GLY	123	15.909	5.655	-6.725	1.00	0.00
ATOM	1945	N	GLY	124	15.056	5.117	-4.773	1.00	0.00
ATOM	1946	H	GLY	124	14.276	5.109	-4.132	1.00	0.00
ATOM	1947	CA	GLY	124	16.337	4.497	-4.333	1.00	0.00
ATOM	1948	HA2	GLY	124	17.145	5.226	-4.397	1.00	0.00
ATOM	1949	HA3	GLY	124	16.246	4.144	-3.305	1.00	0.00
ATOM	1950	C	GLY	124	16.667	3.309	-5.239	1.00	0.00
ATOM	1951	O	GLY	124	16.678	3.422	-6.448	1.00	0.00
ATOM	1952	N	ARG	125	16.936	2.168	-4.663	1.00	0.00
ATOM	1953	H	ARG	125	16.929	2.064	-3.658	1.00	0.00
ATOM	1954	CA	ARG	125	17.263	0.975	-5.493	1.00	0.00
ATOM	1955	HA	ARG	125	18.137	1.173	-6.114	1.00	0.00
ATOM	1956	CB	ARG	125	17.573	-0.213	-4.578	1.00	0.00
ATOM	1957	HB2	ARG	125	16.748	-0.362	-3.882	1.00	0.00
ATOM	1958	HB3	ARG	125	17.706	-1.112	-5.180	1.00	0.00
ATOM	1959	CG	ARG	125	18.856	0.070	-3.795	1.00	0.00

ATOM	1960	HG2	ARG	125	19.655	0.333	-4.488	1.00	0.00
ATOM	1961	HG3	ARG	125	18.687	0.897	-3.106	1.00	0.00
ATOM	1962	CD	ARG	125	19.257	-1.178	-3.005	1.00	0.00
ATOM	1963	HD2	ARG	125	18.404	-1.563	-2.447	1.00	0.00
ATOM	1964	HD3	ARG	125	19.627	-1.950	-3.680	1.00	0.00
ATOM	1965	NE	ARG	125	20.339	-0.828	-2.042	1.00	0.00
ATOM	1966	HE	ARG	125	20.273	-0.016	-1.445	1.00	0.00
ATOM	1967	CZ	ARG	125	21.403	-1.578	-1.956	1.00	0.00
ATOM	1968	NH1	ARG	125	22.005	-1.984	-3.041	1.00	0.00
ATOM	1969	HH11	ARG	125	21.645	-1.716	-3.946	1.00	0.00
ATOM	1970	HH12	ARG	125	22.829	-2.565	-2.971	1.00	0.00
ATOM	1971	NH2	ARG	125	21.866	-1.923	-0.786	1.00	0.00
ATOM	1972	HH21	ARG	125	21.399	-1.608	0.052	1.00	0.00
ATOM	1973	HH22	ARG	125	22.690	-2.504	-0.724	1.00	0.00
ATOM	1974	C	ARG	125	16.068	0.633	-6.385	1.00	0.00
ATOM	1975	O	ARG	125	16.224	0.146	-7.489	1.00	0.00
ATOM	1976	N	CYX	126	14.877	0.884	-5.919	1.00	0.00
ATOM	1977	H	CYX	126	14.741	1.288	-5.004	1.00	0.00
ATOM	1978	CA	CYX	126	13.673	0.574	-6.741	1.00	0.00
ATOM	1979	HA	CYX	126	13.650	-0.486	-6.994	1.00	0.00
ATOM	1980	CB	CYX	126	12.412	0.914	-5.945	1.00	0.00
ATOM	1981	HB2	CYX	126	12.477	1.939	-5.582	1.00	0.00

```
ATOM   1982  HB3 CYX   126      11.538   0.810  -6.587  1.00  0.00
ATOM   1983  SG  CYX   126      12.296  -0.175  -4.505  1.00  0.00
ATOM   1984  C   CYX   126      13.707   1.406  -8.025  1.00  0.00
ATOM   1985  O   CYX   126      13.101   1.056  -9.019  1.00  0.00
ATOM   1986  N   SER   127      14.412   2.503  -8.013  1.00  0.00
ATOM   1987  H   SER   127      14.920   2.797  -7.191  1.00  0.00
ATOM   1988  CA  SER   127      14.486   3.356  -9.232  1.00  0.00
ATOM   1989  HA  SER   127      15.159   4.197  -9.065  1.00  0.00
ATOM   1990  CB  SER   127      15.020   2.528 -10.403  1.00  0.00
ATOM   1991  HB2 SER   127      16.100   2.655 -10.476  1.00  0.00
ATOM   1992  HB3 SER   127      14.790   1.475 -10.239  1.00  0.00
ATOM   1993  OG  SER   127      14.410   2.963 -11.609  1.00  0.00
ATOM   1994  HG  SER   127      14.746   2.443 -12.343  1.00  0.00
ATOM   1995  C   SER   127      13.088   3.878  -9.575  1.00  0.00
ATOM   1996  O   SER   127      12.139   3.416  -8.964  1.00  0.00
ATOM   1997  OXT SER   127      12.992   4.733 -10.440  1.00  0.00
TER
ATOM   1998  S1  LIG   128       4.851 -17.007  -8.477  1.00  0.00
ATOM   1999  O1  LIG   128       7.543 -19.357  -5.587  1.00  0.00
ATOM   2000  O2  LIG   128       3.791 -15.763  -8.583  1.00  0.00
ATOM   2001  O3  LIG   128       6.346 -16.882  -9.112  1.00  0.00
ATOM   2002  N1  LIG   128       4.971 -17.164  -6.629  1.00  0.00
```

```
ATOM   2003  H1  LIG   128       4.624 -16.294  -6.233  1.00  0.00
ATOM   2004  N2  LIG   128       6.492 -18.969  -6.524  1.00  0.00
ATOM   2005  N3  LIG   128       2.139 -21.970 -10.480  1.00  0.00
ATOM   2006  H10 LIG   128       1.167 -21.957 -10.740  1.00  0.00
ATOM   2007  H11 LIG   128       2.639 -22.836 -10.579  1.00  0.00
ATOM   2008  C1  LIG   128       4.029 -18.557  -9.060  1.00  0.00
ATOM   2009  C2  LIG   128       2.680 -18.479  -9.402  1.00  0.00
ATOM   2010  H2  LIG   128       2.150 -17.538  -9.305  1.00  0.00
ATOM   2011  C3  LIG   128       4.773 -19.731  -9.175  1.00  0.00
ATOM   2012  H3  LIG   128       5.813 -19.759  -8.874  1.00  0.00
ATOM   2013  C4  LIG   128       6.008 -17.835  -6.009  1.00  0.00
ATOM   2014  C5  LIG   128       2.764 -20.838 -10.009  1.00  0.00
ATOM   2015  C6  LIG   128       2.048 -19.623  -9.878  1.00  0.00
ATOM   2016  H4  LIG   128       1.000 -19.585 -10.154  1.00  0.00
ATOM   2017  C7  LIG   128       4.133 -20.872  -9.649  1.00  0.00
ATOM   2018  H5  LIG   128       4.689 -21.799  -9.740  1.00  0.00
ATOM   2019  C8  LIG   128       6.658 -17.460  -4.783  1.00  0.00
ATOM   2020  H6  LIG   128       6.453 -16.591  -4.180  1.00  0.00
ATOM   2021  C9  LIG   128       7.590 -18.432  -4.560  1.00  0.00
ATOM   2022  C10 LIG   128       8.601 -18.664  -3.495  1.00  0.00
ATOM   2023  H7  LIG   128       8.554 -17.870  -2.746  1.00  0.00
ATOM   2024  H8  LIG   128       8.430 -19.625  -2.997  1.00  0.00
```

ATOM 2025 H9 LIG 128 9.611 -18.688 -3.917 1.00 0.00

TER

ATOM 2026 S1 LIG 129 -22.795 12.903 5.186 1.00 0.00

ATOM 2027 O1 LIG 129 -20.160 16.436 6.489 1.00 0.00

ATOM 2028 O2 LIG 129 -23.641 12.276 3.932 1.00 0.00

ATOM 2029 O3 LIG 129 -21.398 12.219 5.672 1.00 0.00

ATOM 2030 N1 LIG 129 -22.454 14.604 4.517 1.00 0.00

ATOM 2031 H1 LIG 129 -22.597 14.563 3.511 1.00 0.00

ATOM 2032 N2 LIG 129 -21.249 15.473 6.354 1.00 0.00

ATOM 2033 N3 LIG 129 -26.529 13.568 9.834 1.00 0.00

ATOM 2034 H10 LIG 129 -27.511 13.375 9.738 1.00 0.00

ATOM 2035 H11 LIG 129 -26.194 13.863 10.736 1.00 0.00

ATOM 2036 C1 LIG 129 -23.930 13.148 6.624 1.00 0.00

ATOM 2037 C2 LIG 129 -25.282 12.868 6.429 1.00 0.00

ATOM 2038 H2 LIG 129 -25.633 12.537 5.458 1.00 0.00

ATOM 2039 C3 LIG 129 -23.411 13.561 7.850 1.00 0.00

ATOM 2040 H3 LIG 129 -22.359 13.795 7.955 1.00 0.00

ATOM 2041 C4 LIG 129 -21.467 15.419 5.037 1.00 0.00

ATOM 2042 C5 LIG 129 -25.668 13.429 8.769 1.00 0.00

ATOM 2043 C6 LIG 129 -26.151 13.009 7.506 1.00 0.00

ATOM 2044 H4 LIG 129 -27.207 12.792 7.378 1.00 0.00

ATOM 2045 C7 LIG 129 -24.287 13.703 8.921 1.00 0.00

```
ATOM   2046  H5  LIG   129     -23.908  14.031   9.883  1.00  0.00
ATOM   2047  C8  LIG   129     -20.605  16.299   4.296  1.00  0.00
ATOM   2048  H6  LIG   129     -20.582  16.435   3.227  1.00  0.00
ATOM   2049  C9  LIG   129     -19.823  16.909   5.234  1.00  0.00
ATOM   2050  C10 LIG   129     -18.730  17.914   5.167  1.00  0.00
ATOM   2051  H7  LIG   129     -18.545  18.207   4.131  1.00  0.00
ATOM   2052  H8  LIG   129     -18.988  18.810   5.743  1.00  0.00
ATOM   2053  H9  LIG   129     -17.804  17.509   5.587  1.00  0.00
TER
ATOM   2054  S1  LIG   130      20.721 -12.623  25.901  1.00  0.00
ATOM   2055  O1  LIG   130      23.615  -9.507  27.644  1.00  0.00
ATOM   2056  O2  LIG   130      19.818 -13.940  26.267  1.00  0.00
ATOM   2057  O3  LIG   130      20.012 -11.188  25.598  1.00  0.00
ATOM   2058  N1  LIG   130      21.739 -12.499  27.452  1.00  0.00
ATOM   2059  H1  LIG   130      21.261 -13.043  28.166  1.00  0.00
ATOM   2060  N2  LIG   130      23.028 -10.604  26.879  1.00  0.00
ATOM   2061  N3  LIG   130      24.476 -14.085  21.458  1.00  0.00
ATOM   2062  H10 LIG   130      24.526 -15.029  21.115  1.00  0.00
ATOM   2063  H11 LIG   130      25.067 -13.396  21.023  1.00  0.00
ATOM   2064  C1  LIG   130      21.910 -13.056  24.554  1.00  0.00
ATOM   2065  C2  LIG   130      21.937 -14.381  24.123  1.00  0.00
ATOM   2066  H2  LIG   130      21.288 -15.116  24.586  1.00  0.00
```

```
ATOM   2067  C3  LIG   130      22.706 -12.060  23.990  1.00  0.00
ATOM   2068  H3  LIG   130      22.676 -11.046  24.371  1.00  0.00
ATOM   2069  C4  LIG   130      22.412 -11.345  27.805  1.00  0.00
ATOM   2070  C5  LIG   130      23.626 -13.745  22.485  1.00  0.00
ATOM   2071  C6  LIG   130      22.797 -14.724  23.085  1.00  0.00
ATOM   2072  H4  LIG   130      22.831 -15.749  22.730  1.00  0.00
ATOM   2073  C7  LIG   130      23.566 -12.411  22.956  1.00  0.00
ATOM   2074  H5  LIG   130      24.201 -11.654  22.506  1.00  0.00
ATOM   2075  C8  LIG   130      22.569 -10.818  29.132  1.00  0.00
ATOM   2076  H6  LIG   130      22.168 -11.227  30.045  1.00  0.00
ATOM   2077  C9  LIG   130      23.324  -9.690  28.983  1.00  0.00
ATOM   2078  C10 LIG   130      23.848  -8.670  29.928  1.00  0.00
ATOM   2079  H7  LIG   130      23.551  -8.910  30.952  1.00  0.00
ATOM   2080  H8  LIG   130      24.942  -8.622  29.884  1.00  0.00
ATOM   2081  H9  LIG   130      23.466  -7.675  29.677  1.00  0.00
TER
ATOM   2082  S1  LIG   131      22.268  20.755   1.311  1.00  0.00
ATOM   2083  O1  LIG   131      26.799  21.047   2.029  1.00  0.00
ATOM   2084  O2  LIG   131      20.837  21.507   1.569  1.00  0.00
ATOM   2085  O3  LIG   131      22.926  20.708  -0.179  1.00  0.00
ATOM   2086  N1  LIG   131      23.357  21.752   2.440  1.00  0.00
ATOM   2087  H1  LIG   131      22.865  22.619   2.639  1.00  0.00
```

ATOM	2088	N2	LIG	131	25.399	20.631	2.047	1.00	0.00
ATOM	2089	N3	LIG	131	21.942	15.231	3.628	1.00	0.00
ATOM	2090	H10	LIG	131	21.130	14.935	4.142	1.00	0.00
ATOM	2091	H11	LIG	131	22.688	14.567	3.506	1.00	0.00
ATOM	2092	C1	LIG	131	22.206	19.059	2.044	1.00	0.00
ATOM	2093	C2	LIG	131	21.056	18.707	2.749	1.00	0.00
ATOM	2094	H2	LIG	131	20.250	19.422	2.865	1.00	0.00
ATOM	2095	C3	LIG	131	23.271	18.182	1.845	1.00	0.00
ATOM	2096	H3	LIG	131	24.159	18.501	1.314	1.00	0.00
ATOM	2097	C4	LIG	131	24.734	21.755	2.328	1.00	0.00
ATOM	2098	C5	LIG	131	22.029	16.500	3.104	1.00	0.00
ATOM	2099	C6	LIG	131	20.969	17.423	3.279	1.00	0.00
ATOM	2100	H4	LIG	131	20.082	17.126	3.828	1.00	0.00
ATOM	2101	C7	LIG	131	23.178	16.902	2.381	1.00	0.00
ATOM	2102	H5	LIG	131	23.998	16.203	2.245	1.00	0.00
ATOM	2103	C8	LIG	131	25.606	22.883	2.515	1.00	0.00
ATOM	2104	H6	LIG	131	25.316	23.895	2.745	1.00	0.00
ATOM	2105	C9	LIG	131	26.867	22.396	2.329	1.00	0.00
ATOM	2106	C10	LIG	131	28.222	23.005	2.367	1.00	0.00
ATOM	2107	H7	LIG	131	28.154	24.070	2.602	1.00	0.00
ATOM	2108	H8	LIG	131	28.848	22.519	3.123	1.00	0.00
ATOM	2109	H9	LIG	131	28.726	22.892	1.401	1.00	0.00

```
TER
ATOM   2110  S1  LIG   132     -24.352 -18.224  -5.124  1.00  0.00
ATOM   2111  O1  LIG   132     -24.092 -21.879  -2.350  1.00  0.00
ATOM   2112  O2  LIG   132     -23.706 -16.752  -5.437  1.00  0.00
ATOM   2113  O3  LIG   132     -24.078 -19.481  -6.124  1.00  0.00
ATOM   2114  N1  LIG   132     -23.576 -18.564  -3.470  1.00  0.00
ATOM   2115  H1  LIG   132     -22.780 -17.937  -3.380  1.00  0.00
ATOM   2116  N2  LIG   132     -24.540 -20.680  -3.053  1.00  0.00
ATOM   2117  N3  LIG   132     -30.238 -17.539  -4.186  1.00  0.00
ATOM   2118  H10 LIG   132     -30.653 -16.624  -4.164  1.00  0.00
ATOM   2119  H11 LIG   132     -30.843 -18.332  -4.057  1.00  0.00
ATOM   2120  C1  LIG   132     -26.162 -18.037  -4.800  1.00  0.00
ATOM   2121  C2  LIG   132     -26.680 -16.743  -4.778  1.00  0.00
ATOM   2122  H2  LIG   132     -26.025 -15.894  -4.931  1.00  0.00
ATOM   2123  C3  LIG   132     -26.952 -19.172  -4.627  1.00  0.00
ATOM   2124  H3  LIG   132     -26.509 -20.160  -4.624  1.00  0.00
ATOM   2125  C4  LIG   132     -23.514 -19.834  -2.929  1.00  0.00
ATOM   2126  C5  LIG   132     -28.887 -17.703  -4.389  1.00  0.00
ATOM   2127  C6  LIG   132     -28.046 -16.578  -4.573  1.00  0.00
ATOM   2128  H4  LIG   132     -28.472 -15.580  -4.556  1.00  0.00
ATOM   2129  C7  LIG   132     -28.317 -18.999  -4.418  1.00  0.00
ATOM   2130  H5  LIG   132     -28.951 -19.868  -4.273  1.00  0.00
```

ATOM 2131 C8 LIG 132 -22.425 -20.390 -2.173 1.00 0.00
ATOM 2132 H6 LIG 132 -21.489 -19.912 -1.937 1.00 0.00
ATOM 2133 C9 LIG 132 -22.831 -21.647 -1.832 1.00 0.00
ATOM 2134 C10 LIG 132 -22.206 -22.759 -1.069 1.00 0.00
ATOM 2135 H7 LIG 132 -21.213 -22.469 -0.717 1.00 0.00
ATOM 2136 H8 LIG 132 -22.819 -23.031 -0.202 1.00 0.00
ATOM 2137 H9 LIG 132 -22.108 -23.653 -1.694 1.00 0.00
TER
ATOM 2138 S1 LIG 133 24.500 9.695 -22.044 1.00 0.00
ATOM 2139 O1 LIG 133 23.211 12.519 -25.433 1.00 0.00
ATOM 2140 O2 LIG 133 25.774 9.219 -21.131 1.00 0.00
ATOM 2141 O3 LIG 133 23.306 10.596 -21.398 1.00 0.00
ATOM 2142 N1 LIG 133 25.393 10.683 -23.340 1.00 0.00
ATOM 2143 H1 LIG 133 26.313 10.903 -22.967 1.00 0.00
ATOM 2144 N2 LIG 133 23.528 11.338 -24.635 1.00 0.00
ATOM 2145 N3 LIG 133 22.230 4.865 -24.783 1.00 0.00
ATOM 2146 H10 LIG 133 22.676 3.968 -24.699 1.00 0.00
ATOM 2147 H11 LIG 133 21.388 4.921 -25.331 1.00 0.00
ATOM 2148 C1 LIG 133 23.800 8.229 -22.924 1.00 0.00
ATOM 2149 C2 LIG 133 24.464 7.012 -22.773 1.00 0.00
ATOM 2150 H2 LIG 133 25.363 6.956 -22.171 1.00 0.00
ATOM 2151 C3 LIG 133 22.631 8.366 -23.671 1.00 0.00

```
ATOM   2152  H3  LIG   133      22.159   9.334 -23.788  1.00  0.00
ATOM   2153  C4  LIG   133      24.746  11.597 -24.150  1.00  0.00
ATOM   2154  C5  LIG   133      22.752   5.980 -24.167  1.00  0.00
ATOM   2155  C6  LIG   133      23.936   5.886 -23.396  1.00  0.00
ATOM   2156  H4  LIG   133      24.433   4.928 -23.289  1.00  0.00
ATOM   2157  C7  LIG   133      22.111   7.236 -24.295  1.00  0.00
ATOM   2158  H5  LIG   133      21.206   7.320 -24.888  1.00  0.00
ATOM   2159  C8  LIG   133      25.261  12.865 -24.592  1.00  0.00
ATOM   2160  H6  LIG   133      26.213  13.301 -24.339  1.00  0.00
ATOM   2161  C9  LIG   133      24.283  13.391 -25.385  1.00  0.00
ATOM   2162  C10 LIG   133      24.150  14.659 -26.151  1.00  0.00
ATOM   2163  H7  LIG   133      25.048  15.271 -26.034  1.00  0.00
ATOM   2164  H8  LIG   133      24.002  14.457 -27.218  1.00  0.00
ATOM   2165  H9  LIG   133      23.286  15.235 -25.802  1.00  0.00
TER
ATOM   2166  S1  LIG   134     -25.324  -0.420  27.128  1.00  0.00
ATOM   2167  O1  LIG   134     -25.774  -2.534  23.072  1.00  0.00
ATOM   2168  O2  LIG   134     -24.254  -0.028  28.304  1.00  0.00
ATOM   2169  O3  LIG   134     -25.918   0.724  26.130  1.00  0.00
ATOM   2170  N1  LIG   134     -24.295  -1.622  26.153  1.00  0.00
ATOM   2171  H1  LIG   134     -23.326  -1.466  26.415  1.00  0.00
ATOM   2172  N2  LIG   134     -25.874  -2.168  24.482  1.00  0.00
```

```
ATOM   2173  N3  LIG   134     -29.794  -3.556  29.612  1.00  0.00
ATOM   2174  H10 LIG   134     -29.762  -3.818  30.582  1.00  0.00
ATOM   2175  H11 LIG   134     -30.603  -3.827  29.079  1.00  0.00
ATOM   2176  C1  LIG   134     -26.698  -1.415  27.862  1.00  0.00
ATOM   2177  C2  LIG   134     -26.596  -1.752  29.211  1.00  0.00
ATOM   2178  H2  LIG   134     -25.728  -1.445  29.782  1.00  0.00
ATOM   2179  C3  LIG   134     -27.794  -1.764  27.074  1.00  0.00
ATOM   2180  H3  LIG   134     -27.824  -1.507  26.023  1.00  0.00
ATOM   2181  C4  LIG   134     -24.605  -1.978  24.854  1.00  0.00
ATOM   2182  C5  LIG   134     -28.767  -2.847  29.033  1.00  0.00
ATOM   2183  C6  LIG   134     -27.635  -2.469  29.796  1.00  0.00
ATOM   2184  H4  LIG   134     -27.580  -2.738  30.845  1.00  0.00
ATOM   2185  C7  LIG   134     -28.826  -2.484  27.665  1.00  0.00
ATOM   2186  H5  LIG   134     -29.687  -2.773  27.070  1.00  0.00
ATOM   2187  C8  LIG   134     -23.673  -2.214  23.785  1.00  0.00
ATOM   2188  H6  LIG   134     -22.600  -2.126  23.830  1.00  0.00
ATOM   2189  C9  LIG   134     -24.440  -2.562  22.711  1.00  0.00
ATOM   2190  C10 LIG   134     -24.134  -2.926  21.303  1.00  0.00
ATOM   2191  H7  LIG   134     -23.055  -2.906  21.130  1.00  0.00
ATOM   2192  H8  LIG   134     -24.507  -3.930  21.069  1.00  0.00
ATOM   2193  H9  LIG   134     -24.613  -2.228  20.608  1.00  0.00
TER
```

```
ATOM   2194  S1  LIG   135     -23.036  14.043 -26.967  1.00  0.00
ATOM   2195  O1  LIG   135     -26.148  14.996 -23.721  1.00  0.00
ATOM   2196  O2  LIG   135     -22.019  14.713 -28.062  1.00  0.00
ATOM   2197  O3  LIG   135     -22.457  13.334 -25.619  1.00  0.00
ATOM   2198  N1  LIG   135     -24.031  15.543 -26.502  1.00  0.00
ATOM   2199  H1  LIG   135     -23.496  16.357 -26.793  1.00  0.00
ATOM   2200  N2  LIG   135     -25.486  14.533 -24.938  1.00  0.00
ATOM   2201  N3  LIG   135     -26.740  10.319 -29.865  1.00  0.00
ATOM   2202  H10 LIG   135     -26.717  10.246 -30.867  1.00  0.00
ATOM   2203  H11 LIG   135     -27.394   9.739 -29.367  1.00  0.00
ATOM   2204  C1  LIG   135     -24.210  12.913 -27.839  1.00  0.00
ATOM   2205  C2  LIG   135     -24.134  12.858 -29.230  1.00  0.00
ATOM   2206  H2  LIG   135     -23.418  13.475 -29.759  1.00  0.00
ATOM   2207  C3  LIG   135     -25.098  12.135 -27.097  1.00  0.00
ATOM   2208  H3  LIG   135     -25.146  12.226 -26.019  1.00  0.00
ATOM   2209  C4  LIG   135     -24.792  15.598 -25.350  1.00  0.00
ATOM   2210  C5  LIG   135     -25.902  11.179 -29.194  1.00  0.00
ATOM   2211  C6  LIG   135     -24.982  11.987 -29.907  1.00  0.00
ATOM   2212  H4  LIG   135     -24.937  11.924 -30.989  1.00  0.00
ATOM   2213  C7  LIG   135     -25.945  11.270 -27.782  1.00  0.00
ATOM   2214  H5  LIG   135     -26.650  10.659 -27.227  1.00  0.00
ATOM   2215  C8  LIG   135     -24.968  16.740 -24.494  1.00  0.00
```

ATOM 2216 H6 LIG 135 -24.515 17.711 -24.608 1.00 0.00

ATOM 2217 C9 LIG 135 -25.817 16.322 -23.510 1.00 0.00

ATOM 2218 C10 LIG 135 -26.407 16.978 -22.313 1.00 0.00

ATOM 2219 H7 LIG 135 -26.071 18.015 -22.242 1.00 0.00

ATOM 2220 H8 LIG 135 -27.502 16.968 -22.362 1.00 0.00

ATOM 2221 H9 LIG 135 -26.114 16.451 -21.398 1.00 0.00

TER

ATOM 2222 S1 LIG 136 -4.077 6.496 -24.989 1.00 0.00

ATOM 2223 O1 LIG 136 -0.556 4.513 -27.180 1.00 0.00

ATOM 2224 O2 LIG 136 -4.546 7.158 -23.567 1.00 0.00

ATOM 2225 O3 LIG 136 -3.827 7.431 -26.299 1.00 0.00

ATOM 2226 N1 LIG 136 -2.471 5.722 -24.462 1.00 0.00

ATOM 2227 H1 LIG 136 -2.198 6.166 -23.590 1.00 0.00

ATOM 2228 N2 LIG 136 -1.839 4.772 -26.531 1.00 0.00

ATOM 2229 N3 LIG 136 -7.831 2.006 -26.308 1.00 0.00

ATOM 2230 H10 LIG 136 -8.550 1.729 -25.661 1.00 0.00

ATOM 2231 H11 LIG 136 -7.773 1.515 -27.183 1.00 0.00

ATOM 2232 C1 LIG 136 -5.200 5.088 -25.404 1.00 0.00

ATOM 2233 C2 LIG 136 -6.174 4.746 -24.467 1.00 0.00

ATOM 2234 H2 LIG 136 -6.240 5.288 -23.531 1.00 0.00

ATOM 2235 C3 LIG 136 -5.071 4.441 -26.632 1.00 0.00

ATOM 2236 H3 LIG 136 -4.284 4.718 -27.323 1.00 0.00

ATOM 2237 C4 LIG 136 -1.501 5.339 -25.369 1.00 0.00
ATOM 2238 C5 LIG 136 -6.959 3.027 -26.007 1.00 0.00
ATOM 2239 C6 LIG 136 -7.055 3.713 -24.772 1.00 0.00
ATOM 2240 H4 LIG 136 -7.824 3.433 -24.059 1.00 0.00
ATOM 2241 C7 LIG 136 -5.953 3.408 -26.928 1.00 0.00
ATOM 2242 H5 LIG 136 -5.868 2.885 -27.875 1.00 0.00
ATOM 2243 C8 LIG 136 -0.078 5.454 -25.201 1.00 0.00
ATOM 2244 H6 LIG 136 0.442 5.876 -24.357 1.00 0.00
ATOM 2245 C9 LIG 136 0.461 4.925 -26.338 1.00 0.00
ATOM 2246 C10 LIG 136 1.852 4.734 -26.825 1.00 0.00
ATOM 2247 H7 LIG 136 2.568 5.102 -26.086 1.00 0.00
ATOM 2248 H8 LIG 136 2.058 3.674 -27.013 1.00 0.00
ATOM 2249 H9 LIG 136 2.012 5.272 -27.765 1.00 0.00
TER
ATOM 2250 S1 LIG 137 -15.865 -19.990 -19.456 1.00 0.00
ATOM 2251 O1 LIG 137 -20.326 -18.905 -19.660 1.00 0.00
ATOM 2252 O2 LIG 137 -14.350 -19.368 -19.467 1.00 0.00
ATOM 2253 O3 LIG 137 -16.502 -20.604 -18.088 1.00 0.00
ATOM 2254 N1 LIG 137 -16.828 -18.486 -19.970 1.00 0.00
ATOM 2255 H1 LIG 137 -16.228 -17.680 -19.812 1.00 0.00
ATOM 2256 N2 LIG 137 -18.995 -19.428 -19.953 1.00 0.00
ATOM 2257 N3 LIG 137 -16.306 -23.960 -23.933 1.00 0.00

```
ATOM   2258  H10 LIG   137     -15.548 -24.093 -24.580  1.00  0.00
ATOM   2259  H11 LIG   137     -17.131 -24.521 -24.055  1.00  0.00
ATOM   2260  C1  LIG   137     -16.038 -21.196 -20.847  1.00  0.00
ATOM   2261  C2  LIG   137     -14.955 -21.334 -21.714  1.00  0.00
ATOM   2262  H2  LIG   137     -14.063 -20.735 -21.568  1.00  0.00
ATOM   2263  C3  LIG   137     -17.207 -21.945 -20.969  1.00  0.00
ATOM   2264  H3  LIG   137     -18.037 -21.790 -20.291  1.00  0.00
ATOM   2265  C4  LIG   137     -18.191 -18.376 -19.771  1.00  0.00
ATOM   2266  C5  LIG   137     -16.216 -23.043 -22.911  1.00  0.00
ATOM   2267  C6  LIG   137     -15.046 -22.261 -22.747  1.00  0.00
ATOM   2268  H4  LIG   137     -14.214 -22.389 -23.432  1.00  0.00
ATOM   2269  C7  LIG   137     -17.292 -22.867 -22.007  1.00  0.00
ATOM   2270  H5  LIG   137     -18.195 -23.457 -22.127  1.00  0.00
ATOM   2271  C8  LIG   137     -18.908 -17.188 -19.395  1.00  0.00
ATOM   2272  H6  LIG   137     -18.490 -16.217 -19.189  1.00  0.00
ATOM   2273  C9  LIG   137     -20.220 -17.562 -19.347  1.00  0.00
ATOM   2274  C10 LIG   137     -21.483 -16.847 -19.025  1.00  0.00
ATOM   2275  H7  LIG   137     -21.278 -15.801 -18.786  1.00  0.00
ATOM   2276  H8  LIG   137     -22.179 -16.882 -19.871  1.00  0.00
ATOM   2277  H9  LIG   137     -21.984 -17.310 -18.168  1.00  0.00
TER
END
```

Table E: The number of atoms present in the system of BE-Dic

ATOM	1	N	GLU	1	9.554	18.504	-4.758	1.00	0.00
ATOM	2	H1	GLU	1	9.864	19.119	-5.496	1.00	0.00
ATOM	3	H2	GLU	1	9.961	17.591	-4.904	1.00	0.00
ATOM	4	H3	GLU	1	9.674	18.974	-3.872	1.00	0.00
ATOM	5	CA	GLU	1	8.084	18.308	-4.904	1.00	0.00
ATOM	6	HA	GLU	1	7.855	17.251	-5.044	1.00	0.00
ATOM	7	CB	GLU	1	7.377	18.798	-3.637	1.00	0.00
ATOM	8	HB2	GLU	1	7.798	18.292	-2.768	1.00	0.00
ATOM	9	HB3	GLU	1	7.520	19.873	-3.535	1.00	0.00
ATOM	10	CG	GLU	1	5.879	18.489	-3.732	1.00	0.00
ATOM	11	HG2	GLU	1	5.482	18.933	-4.644	1.00	0.00
ATOM	12	HG3	GLU	1	5.735	17.409	-3.761	1.00	0.00
ATOM	13	CD	GLU	1	5.147	19.067	-2.525	1.00	0.00
ATOM	14	OE1	GLU	1	5.276	18.501	-1.451	1.00	0.00
ATOM	15	OE2	GLU	1	4.465	20.065	-2.692	1.00	0.00
ATOM	16	C	GLU	1	7.588	19.089	-6.119	1.00	0.00
ATOM	17	O	GLU	1	7.443	20.311	-6.066	1.00	0.00
ATOM	18	N	ALA	2	7.328	18.372	-7.210	1.00	0.00
ATOM	19	H	ALA	2	7.480	17.373	-7.212	1.00	0.00
ATOM	20	CA	ALA	2	6.844	19.002	-8.435	1.00	0.00

ATOM	21	HA	ALA	2	7.500	19.825	-8.718	1.00	0.00
ATOM	22	CB	ALA	2	6.820	17.982	-9.575	1.00	0.00
ATOM	23	HB1	ALA	2	6.160	17.156	-9.310	1.00	0.00
ATOM	24	HB2	ALA	2	6.456	18.460	-10.484	1.00	0.00
ATOM	25	HB3	ALA	2	7.828	17.601	-9.744	1.00	0.00
ATOM	26	C	ALA	2	5.443	19.570	-8.230	1.00	0.00
ATOM	27	O	ALA	2	4.650	19.027	-7.462	1.00	0.00
ATOM	28	N	GLU	3	5.148	20.666	-8.922	1.00	0.00
ATOM	29	H	GLU	3	5.841	21.093	-9.519	1.00	0.00
ATOM	30	CA	GLU	3	3.839	21.304	-8.814	1.00	0.00
ATOM	31	HA	GLU	3	3.628	21.547	-7.772	1.00	0.00
ATOM	32	CB	GLU	3	3.814	22.602	-9.636	1.00	0.00
ATOM	33	HB2	GLU	3	2.919	23.168	-9.378	1.00	0.00
ATOM	34	HB3	GLU	3	4.699	23.192	-9.395	1.00	0.00
ATOM	35	CG	GLU	3	3.804	22.285	-11.139	1.00	0.00
ATOM	36	HG2	GLU	3	2.923	21.687	-11.371	1.00	0.00
ATOM	37	HG3	GLU	3	3.765	23.220	-11.697	1.00	0.00
ATOM	38	CD	GLU	3	5.062	21.510	-11.531	1.00	0.00
ATOM	39	OE1	GLU	3	6.147	22.015	-11.293	1.00	0.00
ATOM	40	OE2	GLU	3	4.921	20.420	-12.063	1.00	0.00
ATOM	41	C	GLU	3	2.736	20.363	-9.297	1.00	0.00
ATOM	42	O	GLU	3	1.578	20.494	-8.898	1.00	0.00

ATOM	43	N	ALA	4	3.106	19.419	-10.160	1.00	0.00
ATOM	44	H	ALA	4	4.062	19.371	-10.483	1.00	0.00
ATOM	45	CA	ALA	4	2.144	18.460	-10.698	1.00	0.00
ATOM	46	HA	ALA	4	1.247	18.989	-11.018	1.00	0.00
ATOM	47	CB	ALA	4	2.741	17.735	-11.906	1.00	0.00
ATOM	48	HB1	ALA	4	3.636	17.193	-11.599	1.00	0.00
ATOM	49	HB2	ALA	4	2.010	17.032	-12.306	1.00	0.00
ATOM	50	HB3	ALA	4	3.003	18.462	-12.674	1.00	0.00
ATOM	51	C	ALA	4	1.730	17.450	-9.639	1.00	0.00
ATOM	52	O	ALA	4	0.878	16.594	-9.882	1.00	0.00
ATOM	53	N	GLN	5	2.329	17.565	-8.463	1.00	0.00
ATOM	54	H	GLN	5	3.023	18.284	-8.316	1.00	0.00
ATOM	55	CA	GLN	5	2.015	16.673	-7.357	1.00	0.00
ATOM	56	HA	GLN	5	2.382	15.666	-7.560	1.00	0.00
ATOM	57	CB	GLN	5	2.694	17.194	-6.095	1.00	0.00
ATOM	58	HB2	GLN	5	3.770	17.248	-6.262	1.00	0.00
ATOM	59	HB3	GLN	5	2.312	18.189	-5.866	1.00	0.00
ATOM	60	CG	GLN	5	2.407	16.254	-4.919	1.00	0.00
ATOM	61	HG2	GLN	5	1.335	16.078	-4.834	1.00	0.00
ATOM	62	HG3	GLN	5	2.920	15.304	-5.072	1.00	0.00
ATOM	63	CD	GLN	5	2.904	16.872	-3.616	1.00	0.00
ATOM	64	OE1	GLN	5	2.516	17.988	-3.269	1.00	0.00

ATOM	65	NE2	GLN	5	3.742	16.209	-2.868	1.00	0.00
ATOM	66	HE21	GLN	5	4.074	16.614	-2.004	1.00	0.00
ATOM	67	HE22	GLN	5	4.056	15.293	-3.156	1.00	0.00
ATOM	68	C	GLN	5	0.506	16.614	-7.132	1.00	0.00
ATOM	69	O	GLN	5	-0.044	15.561	-6.810	1.00	0.00
ATOM	70	N	GLU	6	-0.153	17.759	-7.299	1.00	0.00
ATOM	71	H	GLU	6	0.349	18.603	-7.534	1.00	0.00
ATOM	72	CA	GLU	6	-1.597	17.842	-7.109	1.00	0.00
ATOM	73	HA	GLU	6	-1.851	17.650	-6.066	1.00	0.00
ATOM	74	CB	GLU	6	-2.087	19.246	-7.483	1.00	0.00
ATOM	75	HB2	GLU	6	-3.153	19.322	-7.272	1.00	0.00
ATOM	76	HB3	GLU	6	-1.546	19.985	-6.891	1.00	0.00
ATOM	77	CG	GLU	6	-1.841	19.506	-8.974	1.00	0.00
ATOM	78	HG2	GLU	6	-0.807	19.258	-9.215	1.00	0.00
ATOM	79	HG3	GLU	6	-2.511	18.880	-9.563	1.00	0.00
ATOM	80	CD	GLU	6	-2.101	20.975	-9.297	1.00	0.00
ATOM	81	OE1	GLU	6	-3.260	21.353	-9.353	1.00	0.00
ATOM	82	OE2	GLU	6	-1.137	21.699	-9.484	1.00	0.00
ATOM	83	C	GLU	6	-2.325	16.792	-7.948	1.00	0.00
ATOM	84	O	GLU	6	-3.241	16.130	-7.461	1.00	0.00
ATOM	85	N	GLU	7	-1.903	16.631	-9.203	1.00	0.00
ATOM	86	H	GLU	7	-1.155	17.202	-9.571	1.00	0.00

ATOM	87	CA	GLU	7	-2.515	15.643	-10.083	1.00	0.00
ATOM	88	HA	GLU	7	-3.565	15.888	-10.241	1.00	0.00
ATOM	89	CB	GLU	7	-1.805	15.627	-11.443	1.00	0.00
ATOM	90	HB2	GLU	7	-2.002	16.563	-11.964	1.00	0.00
ATOM	91	HB3	GLU	7	-0.731	15.516	-11.289	1.00	0.00
ATOM	92	CG	GLU	7	-2.324	14.453	-12.286	1.00	0.00
ATOM	93	HG2	GLU	7	-3.340	14.213	-11.971	1.00	0.00
ATOM	94	HG3	GLU	7	-2.327	14.738	-13.338	1.00	0.00
ATOM	95	CD	GLU	7	-1.430	13.230	-12.093	1.00	0.00
ATOM	96	OE1	GLU	7	-0.260	13.315	-12.429	1.00	0.00
ATOM	97	OE2	GLU	7	-1.929	12.224	-11.616	1.00	0.00
ATOM	98	C	GLU	7	-2.425	14.268	-9.447	1.00	0.00
ATOM	99	O	GLU	7	-3.365	13.475	-9.508	1.00	0.00
ATOM	100	N	CYX	8	-1.272	13.995	-8.845	1.00	0.00
ATOM	101	H	CYX	8	-0.522	14.672	-8.837	1.00	0.00
ATOM	102	CA	CYX	8	-1.046	12.714	-8.209	1.00	0.00
ATOM	103	HA	CYX	8	-1.303	11.906	-8.894	1.00	0.00
ATOM	104	CB	CYX	8	0.429	12.575	-7.820	1.00	0.00
ATOM	105	HB2	CYX	8	0.680	13.325	-7.070	1.00	0.00
ATOM	106	HB3	CYX	8	0.606	11.580	-7.411	1.00	0.00
ATOM	107	SG	CYX	8	1.466	12.814	-9.283	1.00	0.00
ATOM	108	C	CYX	8	-1.916	12.565	-6.968	1.00	0.00

```
ATOM    109  O   CYX     8      -2.703  11.625  -6.866  1.00  0.00
ATOM    110  N   ARG     9      -1.767  13.499  -6.032  1.00  0.00
ATOM    111  H   ARG     9      -1.092  14.240  -6.156  1.00  0.00
ATOM    112  CA  ARG     9      -2.540  13.466  -4.794  1.00  0.00
ATOM    113  HA  ARG     9      -2.274  12.585  -4.209  1.00  0.00
ATOM    114  CB  ARG     9      -2.239  14.715  -3.962  1.00  0.00
ATOM    115  HB2 ARG     9      -1.163  14.795  -3.806  1.00  0.00
ATOM    116  HB3 ARG     9      -2.595  15.597  -4.492  1.00  0.00
ATOM    117  CG  ARG     9      -2.945  14.615  -2.605  1.00  0.00
ATOM    118  HG2 ARG     9      -4.024  14.588  -2.758  1.00  0.00
ATOM    119  HG3 ARG     9      -2.628  13.704  -2.097  1.00  0.00
ATOM    120  CD  ARG     9      -2.582  15.829  -1.748  1.00  0.00
ATOM    121  HD2 ARG     9      -2.962  15.695  -0.735  1.00  0.00
ATOM    122  HD3 ARG     9      -1.499  15.950  -1.716  1.00  0.00
ATOM    123  NE  ARG     9      -3.172  17.040  -2.313  1.00  0.00
ATOM    124  HE  ARG     9      -3.846  16.973  -3.062  1.00  0.00
ATOM    125  CZ  ARG     9      -2.839  18.249  -1.863  1.00  0.00
ATOM    126  NH1 ARG     9      -1.973  18.374  -0.894  1.00  0.00
ATOM    127 HH11 ARG     9      -1.555  17.550  -0.485  1.00  0.00
ATOM    128 HH12 ARG     9      -1.725  19.293  -0.558  1.00  0.00
ATOM    129  NH2 ARG     9      -3.382  19.311  -2.390  1.00  0.00
ATOM    130 HH21 ARG     9      -4.053  19.212  -3.138  1.00  0.00
```

ATOM 131 HH22 ARG 9 -3.128 20.226 -2.047 1.00 0.00
ATOM 132 C ARG 9 -4.035 13.389 -5.086 1.00 0.00
ATOM 133 O ARG 9 -4.758 12.621 -4.454 1.00 0.00
ATOM 134 N GLU 10 -4.493 14.187 -6.047 1.00 0.00
ATOM 135 H GLU 10 -3.870 14.817 -6.532 1.00 0.00
ATOM 136 CA GLU 10 -5.906 14.193 -6.412 1.00 0.00
ATOM 137 HA GLU 10 -6.515 14.466 -5.550 1.00 0.00
ATOM 138 CB GLU 10 -6.149 15.215 -7.527 1.00 0.00
ATOM 139 HB2 GLU 10 -5.889 16.210 -7.167 1.00 0.00
ATOM 140 HB3 GLU 10 -5.526 14.966 -8.386 1.00 0.00
ATOM 141 CG GLU 10 -7.626 15.190 -7.941 1.00 0.00
ATOM 142 HG2 GLU 10 -8.233 14.896 -7.085 1.00 0.00
ATOM 143 HG3 GLU 10 -7.920 16.186 -8.270 1.00 0.00
ATOM 144 CD GLU 10 -7.837 14.194 -9.081 1.00 0.00
ATOM 145 OE1 GLU 10 -7.187 14.342 -10.103 1.00 0.00
ATOM 146 OE2 GLU 10 -8.646 13.295 -8.914 1.00 0.00
ATOM 147 C GLU 10 -6.343 12.809 -6.882 1.00 0.00
ATOM 148 O GLU 10 -7.368 12.285 -6.442 1.00 0.00
ATOM 149 N GLN 11 -5.556 12.230 -7.785 1.00 0.00
ATOM 150 H GLN 11 -4.745 12.719 -8.135 1.00 0.00
ATOM 151 CA GLN 11 -5.856 10.910 -8.329 1.00 0.00
ATOM 152 HA GLN 11 -6.834 10.915 -8.810 1.00 0.00

ATOM 153 CB GLN 11 -4.799 10.526 -9.371 1.00 0.00
ATOM 154 HB2 GLN 11 -4.657 11.353 -10.066 1.00 0.00
ATOM 155 HB3 GLN 11 -3.856 10.308 -8.868 1.00 0.00
ATOM 156 CG GLN 11 -5.265 9.286 -10.142 1.00 0.00
ATOM 157 HG2 GLN 11 -5.341 8.435 -9.464 1.00 0.00
ATOM 158 HG3 GLN 11 -6.237 9.476 -10.596 1.00 0.00
ATOM 159 CD GLN 11 -4.268 8.941 -11.248 1.00 0.00
ATOM 160 OE1 GLN 11 -3.334 9.701 -11.510 1.00 0.00
ATOM 161 NE2 GLN 11 -4.413 7.830 -11.917 1.00 0.00
ATOM 162 HE21 GLN 11 -3.758 7.595 -12.649 1.00 0.00
ATOM 163 HE22 GLN 11 -5.179 7.209 -11.699 1.00 0.00
ATOM 164 C GLN 11 -5.915 9.850 -7.228 1.00 0.00
ATOM 165 O GLN 11 -6.690 8.898 -7.320 1.00 0.00
ATOM 166 N MET 12 -5.084 10.008 -6.196 1.00 0.00
ATOM 167 H MET 12 -4.440 10.786 -6.170 1.00 0.00
ATOM 168 CA MET 12 -5.048 9.040 -5.097 1.00 0.00
ATOM 169 HA MET 12 -4.666 8.084 -5.457 1.00 0.00
ATOM 170 CB MET 12 -4.134 9.540 -3.976 1.00 0.00
ATOM 171 HB2 MET 12 -4.421 10.553 -3.695 1.00 0.00
ATOM 172 HB3 MET 12 -4.225 8.884 -3.110 1.00 0.00
ATOM 173 CG MET 12 -2.686 9.538 -4.466 1.00 0.00
ATOM 174 HG2 MET 12 -2.401 8.528 -4.762 1.00 0.00

```
ATOM    175  HG3 MET    12      -2.587  10.208  -5.320  1.00  0.00
ATOM    176  SD  MET    12      -1.593  10.099  -3.141  1.00  0.00
ATOM    177  CE  MET    12      -0.145  10.424  -4.175  1.00  0.00
ATOM    178  HE1 MET    12      -0.394  11.176  -4.923  1.00  0.00
ATOM    179  HE2 MET    12       0.673  10.788  -3.552  1.00  0.00
ATOM    180  HE3 MET    12       0.161   9.503  -4.673  1.00  0.00
ATOM    181  C   MET    12      -6.445   8.777  -4.540  1.00  0.00
ATOM    182  O   MET    12      -6.786   7.633  -4.241  1.00  0.00
ATOM    183  N   GLN    13      -7.255   9.828  -4.409  1.00  0.00
ATOM    184  H   GLN    13      -6.947  10.760  -4.645  1.00  0.00
ATOM    185  CA  GLN    13      -8.612   9.655  -3.896  1.00  0.00
ATOM    186  HA  GLN    13      -8.572   9.249  -2.885  1.00  0.00
ATOM    187  CB  GLN    13      -9.354  10.995  -3.857  1.00  0.00
ATOM    188  HB2 GLN    13      -9.186  11.525  -4.794  1.00  0.00
ATOM    189  HB3 GLN    13     -10.421  10.811  -3.730  1.00  0.00
ATOM    190  CG  GLN    13      -8.841  11.845  -2.690  1.00  0.00
ATOM    191  HG2 GLN    13      -9.471  12.726  -2.574  1.00  0.00
ATOM    192  HG3 GLN    13      -8.859  11.262  -1.769  1.00  0.00
ATOM    193  CD  GLN    13      -7.409  12.301  -2.950  1.00  0.00
ATOM    194  OE1 GLN    13      -6.459  11.627  -2.552  1.00  0.00
ATOM    195  NE2 GLN    13      -7.197  13.415  -3.595  1.00  0.00
ATOM    196 HE21 GLN    13      -6.250  13.721  -3.770  1.00  0.00
```

ATOM	197	HE22	GLN	13	-7.980	13.965	-3.916	1.00	0.00
ATOM	198	C	GLN	13	-9.372	8.680	-4.782	1.00	0.00
ATOM	199	O	GLN	13	-10.042	7.774	-4.291	1.00	0.00
ATOM	200	N	ARG	14	-9.226	8.855	-6.093	1.00	0.00
ATOM	201	H	ARG	14	-8.669	9.623	-6.440	1.00	0.00
ATOM	202	CA	ARG	14	-9.857	7.975	-7.054	1.00	0.00
ATOM	203	HA	ARG	14	-10.940	8.011	-6.935	1.00	0.00
ATOM	204	CB	ARG	14	-9.506	8.408	-8.482	1.00	0.00
ATOM	205	HB2	ARG	14	-9.679	9.479	-8.585	1.00	0.00
ATOM	206	HB3	ARG	14	-8.455	8.190	-8.675	1.00	0.00
ATOM	207	CG	ARG	14	-10.378	7.650	-9.489	1.00	0.00
ATOM	208	HG2	ARG	14	-9.905	7.678	-10.471	1.00	0.00
ATOM	209	HG3	ARG	14	-10.485	6.614	-9.167	1.00	0.00
ATOM	210	CD	ARG	14	-11.759	8.304	-9.569	1.00	0.00
ATOM	211	HD2	ARG	14	-12.415	7.708	-10.204	1.00	0.00
ATOM	212	HD3	ARG	14	-12.191	8.380	-8.571	1.00	0.00
ATOM	213	NE	ARG	14	-11.650	9.646	-10.136	1.00	0.00
ATOM	214	HE	ARG	14	-10.855	9.889	-10.709	1.00	0.00
ATOM	215	CZ	ARG	14	-12.582	10.572	-9.916	1.00	0.00
ATOM	216	NH1	ARG	14	-13.627	10.294	-9.184	1.00	0.00
ATOM	217	HH11	ARG	14	-13.728	9.372	-8.783	1.00	0.00
ATOM	218	HH12	ARG	14	-14.329	11.001	-9.022	1.00	0.00

ATOM 219 NH2 ARG 14 -12.450 11.763 -10.434 1.00 0.00
ATOM 220 HH21 ARG 14 -11.641 11.977 -10.999 1.00 0.00
ATOM 221 HH22 ARG 14 -13.157 12.464 -10.266 1.00 0.00
ATOM 222 C ARG 14 -9.362 6.569 -6.798 1.00 0.00
ATOM 223 O ARG 14 -10.132 5.608 -6.805 1.00 0.00
ATOM 224 N GLN 15 -8.056 6.471 -6.548 1.00 0.00
ATOM 225 H GLN 15 -7.462 7.287 -6.573 1.00 0.00
ATOM 226 CA GLN 15 -7.451 5.195 -6.261 1.00 0.00
ATOM 227 HA GLN 15 -7.834 4.438 -6.945 1.00 0.00
ATOM 228 CB GLN 15 -5.928 5.282 -6.430 1.00 0.00
ATOM 229 HB2 GLN 15 -5.618 6.326 -6.383 1.00 0.00
ATOM 230 HB3 GLN 15 -5.443 4.723 -5.629 1.00 0.00
ATOM 231 CG GLN 15 -5.523 4.690 -7.784 1.00 0.00
ATOM 232 HG2 GLN 15 -4.488 4.349 -7.743 1.00 0.00
ATOM 233 HG3 GLN 15 -6.171 3.851 -8.038 1.00 0.00
ATOM 234 CD GLN 15 -5.645 5.748 -8.876 1.00 0.00
ATOM 235 OE1 GLN 15 -4.638 6.274 -9.347 1.00 0.00
ATOM 236 NE2 GLN 15 -6.828 6.097 -9.305 1.00 0.00
ATOM 237 HE21 GLN 15 -6.913 6.797 -10.028 1.00 0.00
ATOM 238 HE22 GLN 15 -7.652 5.665 -8.912 1.00 0.00
ATOM 239 C GLN 15 -7.803 4.789 -4.839 1.00 0.00
ATOM 240 O GLN 15 -6.981 4.873 -3.925 1.00 0.00

ATOM	241	N	GLN	16	-9.034	4.343	-4.674	1.00	0.00
ATOM	242	H	GLN	16	-9.672	4.321	-5.456	1.00	0.00
ATOM	243	CA	GLN	16	-9.523	3.907	-3.372	1.00	0.00
ATOM	244	HA	GLN	16	-9.112	4.539	-2.584	1.00	0.00
ATOM	245	CB	GLN	16	-11.048	4.009	-3.334	1.00	0.00
ATOM	246	HB2	GLN	16	-11.480	3.389	-4.120	1.00	0.00
ATOM	247	HB3	GLN	16	-11.417	3.674	-2.364	1.00	0.00
ATOM	248	CG	GLN	16	-11.450	5.468	-3.556	1.00	0.00
ATOM	249	HG2	GLN	16	-11.020	6.086	-2.767	1.00	0.00
ATOM	250	HG3	GLN	16	-11.081	5.806	-4.524	1.00	0.00
ATOM	251	CD	GLN	16	-12.967	5.629	-3.529	1.00	0.00
ATOM	252	OE1	GLN	16	-13.703	4.641	-3.513	1.00	0.00
ATOM	253	NE2	GLN	16	-13.477	6.830	-3.526	1.00	0.00
ATOM	254	HE21	GLN	16	-14.479	6.949	-3.508	1.00	0.00
ATOM	255	HE22	GLN	16	-12.866	7.634	-3.541	1.00	0.00
ATOM	256	C	GLN	16	-9.073	2.475	-3.111	1.00	0.00
ATOM	257	O	GLN	16	-9.843	1.641	-2.626	1.00	0.00
ATOM	258	N	MET	17	-7.818	2.200	-3.459	1.00	0.00
ATOM	259	H	MET	17	-7.243	2.913	-3.885	1.00	0.00
ATOM	260	CA	MET	17	-7.252	0.867	-3.288	1.00	0.00
ATOM	261	HA	MET	17	-7.890	0.126	-3.770	1.00	0.00
ATOM	262	CB	MET	17	-5.857	0.801	-3.924	1.00	0.00

```
ATOM    263  HB2 MET    17      -5.701  -0.198  -4.332  1.00  0.00
ATOM    264  HB3 MET    17      -5.800   1.534  -4.728  1.00  0.00
ATOM    265  CG  MET    17      -4.776   1.100  -2.884  1.00  0.00
ATOM    266  HG2 MET    17      -5.106   1.901  -2.223  1.00  0.00
ATOM    267  HG3 MET    17      -4.569   0.206  -2.295  1.00  0.00
ATOM    268  SD  MET    17      -3.267   1.614  -3.732  1.00  0.00
ATOM    269  CE  MET    17      -3.897   3.189  -4.360  1.00  0.00
ATOM    270  HE1 MET    17      -4.200   3.819  -3.524  1.00  0.00
ATOM    271  HE2 MET    17      -3.115   3.692  -4.929  1.00  0.00
ATOM    272  HE3 MET    17      -4.755   3.006  -5.006  1.00  0.00
ATOM    273  C   MET    17      -7.170   0.476  -1.821  1.00  0.00
ATOM    274  O   MET    17      -7.169  -0.706  -1.493  1.00  0.00
ATOM    275  N   LEU    18      -7.088   1.477  -0.953  1.00  0.00
ATOM    276  H   LEU    18      -7.073   2.431  -1.284  1.00  0.00
ATOM    277  CA  LEU    18      -6.977   1.242   0.484  1.00  0.00
ATOM    278  HA  LEU    18      -6.079   0.661   0.697  1.00  0.00
ATOM    279  CB  LEU    18      -6.889   2.564   1.230  1.00  0.00
ATOM    280  HB2 LEU    18      -7.810   3.125   1.074  1.00  0.00
ATOM    281  HB3 LEU    18      -6.755   2.375   2.295  1.00  0.00
ATOM    282  CG  LEU    18      -5.702   3.376   0.703  1.00  0.00
ATOM    283  HG  LEU    18      -4.846   2.716   0.560  1.00  0.00
ATOM    284  CD1 LEU    18      -6.075   4.025  -0.634  1.00  0.00
```

```
ATOM    285 HD11 LEU    18      -6.930   4.685  -0.491  1.00  0.00
ATOM    286 HD12 LEU    18      -5.229   4.602  -1.007  1.00  0.00
ATOM    287 HD13 LEU    18      -6.331   3.249  -1.356  1.00  0.00
ATOM    288  CD2 LEU    18      -5.335   4.464   1.717  1.00  0.00
ATOM    289 HD21 LEU    18      -5.064   4.000   2.666  1.00  0.00
ATOM    290 HD22 LEU    18      -4.490   5.041   1.342  1.00  0.00
ATOM    291 HD23 LEU    18      -6.188   5.125   1.867  1.00  0.00
ATOM    292  C   LEU    18      -8.163   0.440   1.002  1.00  0.00
ATOM    293  O   LEU    18      -8.056  -0.223   2.025  1.00  0.00
ATOM    294  N   SER    19      -9.292   0.490   0.299  1.00  0.00
ATOM    295  H   SER    19      -9.370   1.059  -0.532  1.00  0.00
ATOM    296  CA  SER    19     -10.459  -0.274   0.727  1.00  0.00
ATOM    297  HA  SER    19     -10.698  -0.038   1.764  1.00  0.00
ATOM    298  CB  SER    19     -11.667   0.074  -0.146  1.00  0.00
ATOM    299  HB2 SER    19     -11.494  -0.272  -1.165  1.00  0.00
ATOM    300  HB3 SER    19     -12.559  -0.409   0.254  1.00  0.00
ATOM    301  OG  SER    19     -11.852   1.484  -0.149  1.00  0.00
ATOM    302  HG  SER    19     -12.609   1.706  -0.696  1.00  0.00
ATOM    303  C   SER    19     -10.150  -1.766   0.613  1.00  0.00
ATOM    304  O   SER    19     -10.264  -2.524   1.584  1.00  0.00
ATOM    305  N   HIE    20      -9.717  -2.166  -0.581  1.00  0.00
ATOM    306  H   HIE    20      -9.642  -1.502  -1.338  1.00  0.00
```

ATOM	307	CA	HIE	20	-9.350	-3.554	-0.839	1.00	0.00
ATOM	308	HA	HIE	20	-10.126	-4.220	-0.462	1.00	0.00
ATOM	309	CB	HIE	20	-9.191	-3.791	-2.348	1.00	0.00
ATOM	310	HB2	HIE	20	-8.154	-3.755	-2.681	1.00	0.00
ATOM	311	HB3	HIE	20	-9.633	-4.743	-2.641	1.00	0.00
ATOM	312	CG	HIE	20	-9.926	-2.721	-3.110	1.00	0.00
ATOM	313	ND1	HIE	20	-9.273	-1.832	-3.948	1.00	0.00
ATOM	314	CE1	HIE	20	-10.198	-0.997	-4.454	1.00	0.00
ATOM	315	HE1	HIE	20	-9.851	-0.226	-5.142	1.00	0.00
ATOM	316	NE2	HIE	20	-11.421	-1.280	-4.000	1.00	0.00
ATOM	317	HE2	HIE	20	-12.304	-0.832	-4.198	1.00	0.00
ATOM	318	CD2	HIE	20	-11.252	-2.370	-3.152	1.00	0.00
ATOM	319	HD2	HIE	20	-12.113	-2.805	-2.645	1.00	0.00
ATOM	320	C	HIE	20	-8.039	-3.853	-0.131	1.00	0.00
ATOM	321	O	HIE	20	-7.825	-4.948	0.388	1.00	0.00
ATOM	322	N	CYX	21	-7.172	-2.847	-0.109	1.00	0.00
ATOM	323	H	CYX	21	-7.395	-1.984	-0.583	1.00	0.00
ATOM	324	CA	CYX	21	-5.882	-2.959	0.543	1.00	0.00
ATOM	325	HA	CYX	21	-5.309	-3.786	0.124	1.00	0.00
ATOM	326	CB	CYX	21	-5.101	-1.657	0.317	1.00	0.00
ATOM	327	HB2	CYX	21	-5.790	-0.866	0.024	1.00	0.00
ATOM	328	HB3	CYX	21	-4.593	-1.373	1.239	1.00	0.00

ATOM	329	SG	CYX	21	-3.905	-1.917	-1.018	1.00	0.00
ATOM	330	C	CYX	21	-6.084	-3.238	2.032	1.00	0.00
ATOM	331	O	CYX	21	-5.471	-4.147	2.594	1.00	0.00
ATOM	332	N	ARG	22	-6.984	-2.481	2.653	1.00	0.00
ATOM	333	H	ARG	22	-7.460	-1.739	2.161	1.00	0.00
ATOM	334	CA	ARG	22	-7.300	-2.690	4.059	1.00	0.00
ATOM	335	HA	ARG	22	-6.408	-2.538	4.667	1.00	0.00
ATOM	336	CB	ARG	22	-8.376	-1.699	4.512	1.00	0.00
ATOM	337	HB2	ARG	22	-8.077	-0.690	4.229	1.00	0.00
ATOM	338	HB3	ARG	22	-9.320	-1.948	4.028	1.00	0.00
ATOM	339	CG	ARG	22	-8.548	-1.772	6.032	1.00	0.00
ATOM	340	HG2	ARG	22	-8.767	-2.799	6.325	1.00	0.00
ATOM	341	HG3	ARG	22	-7.629	-1.445	6.519	1.00	0.00
ATOM	342	CD	ARG	22	-9.704	-0.862	6.459	1.00	0.00
ATOM	343	HD2	ARG	22	-10.645	-1.250	6.069	1.00	0.00
ATOM	344	HD3	ARG	22	-9.755	-0.813	7.547	1.00	0.00
ATOM	345	NE	ARG	22	-9.502	0.489	5.938	1.00	0.00
ATOM	346	HE	ARG	22	-8.880	1.126	6.416	1.00	0.00
ATOM	347	CZ	ARG	22	-10.124	0.913	4.835	1.00	0.00
ATOM	348	NH1	ARG	22	-10.953	0.127	4.200	1.00	0.00
ATOM	349	HH11	ARG	22	-11.124	-0.807	4.546	1.00	0.00
ATOM	350	HH12	ARG	22	-11.418	0.456	3.367	1.00	0.00

ATOM	351	NH2	ARG	22	-9.903	2.118	4.386	1.00	0.00
ATOM	352	HH21	ARG	22	-9.261	2.726	4.875	1.00	0.00
ATOM	353	HH22	ARG	22	-10.374	2.437	3.552	1.00	0.00
ATOM	354	C	ARG	22	-7.797	-4.116	4.230	1.00	0.00
ATOM	355	O	ARG	22	-7.367	-4.843	5.133	1.00	0.00
ATOM	356	N	MET	23	-8.688	-4.522	3.319	1.00	0.00
ATOM	357	H	MET	23	-9.024	-3.892	2.606	1.00	0.00
ATOM	358	CA	MET	23	-9.212	-5.877	3.344	1.00	0.00
ATOM	359	HA	MET	23	-9.762	-6.045	4.270	1.00	0.00
ATOM	360	CB	MET	23	-10.162	-6.102	2.161	1.00	0.00
ATOM	361	HB2	MET	23	-10.915	-5.314	2.148	1.00	0.00
ATOM	362	HB3	MET	23	-9.593	-6.076	1.232	1.00	0.00
ATOM	363	CG	MET	23	-10.850	-7.462	2.300	1.00	0.00
ATOM	364	HG2	MET	23	-10.099	-8.248	2.385	1.00	0.00
ATOM	365	HG3	MET	23	-11.481	-7.467	3.189	1.00	0.00
ATOM	366	SD	MET	23	-11.875	-7.772	0.840	1.00	0.00
ATOM	367	CE	MET	23	-10.528	-8.097	-0.326	1.00	0.00
ATOM	368	HE1	MET	23	-9.945	-8.951	0.020	1.00	0.00
ATOM	369	HE2	MET	23	-10.943	-8.316	-1.310	1.00	0.00
ATOM	370	HE3	MET	23	-9.883	-7.221	-0.391	1.00	0.00
ATOM	371	C	MET	23	-8.047	-6.857	3.275	1.00	0.00
ATOM	372	O	MET	23	-8.047	-7.885	3.949	1.00	0.00

ATOM	373	N	TYR	24	-7.045	-6.508	2.464	1.00	0.00
ATOM	374	H	TYR	24	-7.119	-5.675	1.898	1.00	0.00
ATOM	375	CA	TYR	24	-5.854	-7.338	2.321	1.00	0.00
ATOM	376	HA	TYR	24	-6.130	-8.335	1.976	1.00	0.00
ATOM	377	CB	TYR	24	-4.896	-6.707	1.289	1.00	0.00
ATOM	378	HB2	TYR	24	-4.888	-7.322	0.389	1.00	0.00
ATOM	379	HB3	TYR	24	-5.275	-5.714	1.048	1.00	0.00
ATOM	380	CG	TYR	24	-3.483	-6.590	1.833	1.00	0.00
ATOM	381	CD1	TYR	24	-2.786	-7.732	2.244	1.00	0.00
ATOM	382	HD1	TYR	24	-3.260	-8.712	2.183	1.00	0.00
ATOM	383	CE1	TYR	24	-1.479	-7.616	2.734	1.00	0.00
ATOM	384	HE1	TYR	24	-0.939	-8.505	3.062	1.00	0.00
ATOM	385	CZ	TYR	24	-0.868	-6.359	2.807	1.00	0.00
ATOM	386	OH	TYR	24	0.422	-6.245	3.283	1.00	0.00
ATOM	387	HH	TYR	24	0.805	-7.089	3.536	1.00	0.00
ATOM	388	CE2	TYR	24	-1.562	-5.219	2.394	1.00	0.00
ATOM	389	HE2	TYR	24	-1.085	-4.241	2.451	1.00	0.00
ATOM	390	CD2	TYR	24	-2.869	-5.333	1.907	1.00	0.00
ATOM	391	HD2	TYR	24	-3.410	-4.444	1.585	1.00	0.00
ATOM	392	C	TYR	24	-5.178	-7.509	3.679	1.00	0.00
ATOM	393	O	TYR	24	-4.772	-8.609	4.043	1.00	0.00
ATOM	394	N	MET	25	-5.072	-6.417	4.435	1.00	0.00

ATOM	395	H	MET	25	-5.413	-5.523	4.114	1.00	0.00
ATOM	396	CA	MET	25	-4.458	-6.489	5.758	1.00	0.00
ATOM	397	HA	MET	25	-3.409	-6.773	5.668	1.00	0.00
ATOM	398	CB	MET	25	-4.538	-5.127	6.449	1.00	0.00
ATOM	399	HB2	MET	25	-5.584	-4.853	6.583	1.00	0.00
ATOM	400	HB3	MET	25	-4.052	-5.189	7.423	1.00	0.00
ATOM	401	CG	MET	25	-3.836	-4.064	5.594	1.00	0.00
ATOM	402	HG2	MET	25	-4.252	-4.072	4.587	1.00	0.00
ATOM	403	HG3	MET	25	-3.983	-3.081	6.041	1.00	0.00
ATOM	404	SD	MET	25	-2.064	-4.419	5.507	1.00	0.00
ATOM	405	CE	MET	25	-1.656	-3.971	7.212	1.00	0.00
ATOM	406	HE1	MET	25	-2.221	-4.601	7.899	1.00	0.00
ATOM	407	HE2	MET	25	-0.589	-4.117	7.381	1.00	0.00
ATOM	408	HE3	MET	25	-1.911	-2.926	7.385	1.00	0.00
ATOM	409	C	MET	25	-5.203	-7.523	6.597	1.00	0.00
ATOM	410	O	MET	25	-4.605	-8.441	7.178	1.00	0.00
ATOM	411	N	ARG	26	-6.528	-7.389	6.625	1.00	0.00
ATOM	412	H	ARG	26	-6.982	-6.621	6.153	1.00	0.00
ATOM	413	CA	ARG	26	-7.355	-8.334	7.358	1.00	0.00
ATOM	414	HA	ARG	26	-7.079	-8.330	8.413	1.00	0.00
ATOM	415	CB	ARG	26	-8.834	-7.946	7.236	1.00	0.00
ATOM	416	HB2	ARG	26	-8.965	-6.918	7.572	1.00	0.00

ATOM	417	HB3	ARG	26	-9.143	-8.030	6.194	1.00	0.00
ATOM	418	CG	ARG	26	-9.691	-8.878	8.100	1.00	0.00
ATOM	419	HG2	ARG	26	-10.743	-8.725	7.860	1.00	0.00
ATOM	420	HG3	ARG	26	-9.418	-9.913	7.892	1.00	0.00
ATOM	421	CD	ARG	26	-9.457	-8.577	9.583	1.00	0.00
ATOM	422	HD2	ARG	26	-8.395	-8.656	9.816	1.00	0.00
ATOM	423	HD3	ARG	26	-9.806	-7.572	9.818	1.00	0.00
ATOM	424	NE	ARG	26	-10.189	-9.533	10.411	1.00	0.00
ATOM	425	HE	ARG	26	-11.098	-9.862	10.118	1.00	0.00
ATOM	426	CZ	ARG	26	-9.686	-9.991	11.558	1.00	0.00
ATOM	427	NH1	ARG	26	-8.518	-9.585	11.977	1.00	0.00
ATOM	428	HH11	ARG	26	-7.991	-8.920	11.429	1.00	0.00
ATOM	429	HH12	ARG	26	-8.146	-9.939	12.847	1.00	0.00
ATOM	430	NH2	ARG	26	-10.367	-10.850	12.265	1.00	0.00
ATOM	431	HH21	ARG	26	-11.270	-11.164	11.940	1.00	0.00
ATOM	432	HH22	ARG	26	-9.987	-11.198	13.134	1.00	0.00
ATOM	433	C	ARG	26	-7.121	-9.727	6.788	1.00	0.00
ATOM	434	O	ARG	26	-7.012	-10.714	7.523	1.00	0.00
ATOM	435	N	GLN	27	-7.004	-9.787	5.466	1.00	0.00
ATOM	436	H	GLN	27	-7.128	-8.959	4.901	1.00	0.00
ATOM	437	CA	GLN	27	-6.739	-11.040	4.796	1.00	0.00
ATOM	438	HA	GLN	27	-7.533	-11.752	5.021	1.00	0.00

ATOM 439 CB GLN 27 -6.676 -10.840 3.274 1.00 0.00

ATOM 440 HB2 GLN 27 -7.654 -10.523 2.914 1.00 0.00

ATOM 441 HB3 GLN 27 -5.937 -10.072 3.044 1.00 0.00

ATOM 442 CG GLN 27 -6.279 -12.154 2.586 1.00 0.00

ATOM 443 HG2 GLN 27 -6.395 -12.048 1.508 1.00 0.00

ATOM 444 HG3 GLN 27 -5.243 -12.404 2.817 1.00 0.00

ATOM 445 CD GLN 27 -7.172 -13.294 3.062 1.00 0.00

ATOM 446 OE1 GLN 27 -8.014 -13.786 2.311 1.00 0.00

ATOM 447 NE2 GLN 27 -7.039 -13.735 4.282 1.00 0.00

ATOM 448 HE21 GLN 27 -7.624 -14.490 4.610 1.00 0.00

ATOM 449 HE22 GLN 27 -6.351 -13.320 4.894 1.00 0.00

ATOM 450 C GLN 27 -5.432 -11.611 5.316 1.00 0.00

ATOM 451 O GLN 27 -5.298 -12.809 5.514 1.00 0.00

ATOM 452 N GLN 28 -4.467 -10.749 5.572 1.00 0.00

ATOM 453 H GLN 28 -4.576 -9.757 5.420 1.00 0.00

ATOM 454 CA GLN 28 -3.212 -11.225 6.085 1.00 0.00

ATOM 455 HA GLN 28 -2.748 -11.910 5.375 1.00 0.00

ATOM 456 CB GLN 28 -2.283 -10.056 6.306 1.00 0.00

ATOM 457 HB2 GLN 28 -2.230 -9.465 5.392 1.00 0.00

ATOM 458 HB3 GLN 28 -2.672 -9.439 7.116 1.00 0.00

ATOM 459 CG GLN 28 -0.881 -10.555 6.673 1.00 0.00

ATOM 460 HG2 GLN 28 -0.641 -11.446 6.093 1.00 0.00

ATOM	461	HG3	GLN	28	-0.145	-9.778	6.466	1.00	0.00
ATOM	462	CD	GLN	28	-0.814	-10.907	8.157	1.00	0.00
ATOM	463	OE1	GLN	28	-0.376	-12.082	8.519	1.00	0.00
ATOM	464	NE2	GLN	28	-1.170	-10.091	9.008	1.00	0.00
ATOM	465	HE21	GLN	28	-1.121	-10.334	9.988	1.00	0.00
ATOM	466	HE22	GLN	28	-1.508	-9.184	8.723	1.00	0.00
ATOM	467	C	GLN	28	-3.454	-11.942	7.402	1.00	0.00
ATOM	468	O	GLN	28	-2.847	-12.978	7.678	1.00	0.00
ATOM	469	N	MET	29	-4.359	-11.386	8.210	1.00	0.00
ATOM	470	H	MET	29	-4.823	-10.525	7.961	1.00	0.00
ATOM	471	CA	MET	29	-4.680	-11.999	9.496	1.00	0.00
ATOM	472	HA	MET	29	-3.780	-12.063	10.109	1.00	0.00
ATOM	473	CB	MET	29	-5.724	-11.164	10.245	1.00	0.00
ATOM	474	HB2	MET	29	-6.535	-10.904	9.566	1.00	0.00
ATOM	475	HB3	MET	29	-6.120	-11.743	11.079	1.00	0.00
ATOM	476	CG	MET	29	-5.075	-9.885	10.776	1.00	0.00
ATOM	477	HG2	MET	29	-4.695	-9.289	9.947	1.00	0.00
ATOM	478	HG3	MET	29	-5.806	-9.303	11.337	1.00	0.00
ATOM	479	SD	MET	29	-3.702	-10.327	11.869	1.00	0.00
ATOM	480	CE	MET	29	-4.662	-11.278	13.076	1.00	0.00
ATOM	481	HE1	MET	29	-5.144	-12.118	12.577	1.00	0.00
ATOM	482	HE2	MET	29	-3.998	-11.652	13.856	1.00	0.00

ATOM	483	HE3	MET	29	-5.421	-10.637	13.524	1.00	0.00
ATOM	484	C	MET	29	-5.211	-13.414	9.291	1.00	0.00
ATOM	485	O	MET	29	-4.666	-14.370	9.840	1.00	0.00
ATOM	486	N	GLU	30	-6.264	-13.551	8.485	1.00	0.00
ATOM	487	H	GLU	30	-6.710	-12.750	8.063	1.00	0.00
ATOM	488	CA	GLU	30	-6.822	-14.878	8.217	1.00	0.00
ATOM	489	HA	GLU	30	-7.029	-15.388	9.158	1.00	0.00
ATOM	490	CB	GLU	30	-8.128	-14.763	7.424	1.00	0.00
ATOM	491	HB2	GLU	30	-7.962	-14.145	6.542	1.00	0.00
ATOM	492	HB3	GLU	30	-8.451	-15.757	7.115	1.00	0.00
ATOM	493	CG	GLU	30	-9.210	-14.124	8.299	1.00	0.00
ATOM	494	HG2	GLU	30	-10.191	-14.475	7.980	1.00	0.00
ATOM	495	HG3	GLU	30	-9.040	-14.415	9.336	1.00	0.00
ATOM	496	CD	GLU	30	-9.151	-12.605	8.182	1.00	0.00
ATOM	497	OE1	GLU	30	-9.292	-12.110	7.075	1.00	0.00
ATOM	498	OE2	GLU	30	-8.974	-11.958	9.202	1.00	0.00
ATOM	499	C	GLU	30	-5.823	-15.730	7.435	1.00	0.00
ATOM	500	O	GLU	30	-5.640	-16.915	7.712	1.00	0.00
ATOM	501	N	GLU	31	-5.189	-15.100	6.448	1.00	0.00
ATOM	502	H	GLU	31	-5.410	-14.134	6.257	1.00	0.00
ATOM	503	CA	GLU	31	-4.207	-15.768	5.596	1.00	0.00
ATOM	504	HA	GLU	31	-4.617	-16.700	5.206	1.00	0.00

ATOM	505	CB	GLU	31	-3.847	-14.859	4.415	1.00	0.00
ATOM	506	HB2	GLU	31	-4.755	-14.398	4.028	1.00	0.00
ATOM	507	HB3	GLU	31	-3.163	-14.082	4.756	1.00	0.00
ATOM	508	CG	GLU	31	-3.176	-15.679	3.305	1.00	0.00
ATOM	509	HG2	GLU	31	-2.318	-16.205	3.723	1.00	0.00
ATOM	510	HG3	GLU	31	-3.893	-16.404	2.919	1.00	0.00
ATOM	511	CD	GLU	31	-2.710	-14.767	2.167	1.00	0.00
ATOM	512	OE1	GLU	31	-2.851	-13.559	2.293	1.00	0.00
ATOM	513	OE2	GLU	31	-2.216	-15.293	1.182	1.00	0.00
ATOM	514	C	GLU	31	-2.942	-16.132	6.381	1.00	0.00
ATOM	515	O	GLU	31	-2.036	-16.758	5.839	1.00	0.00
ATOM	516	N	SER	32	-2.881	-15.726	7.651	1.00	0.00
ATOM	517	H	SER	32	-3.641	-15.193	8.049	1.00	0.00
ATOM	518	CA	SER	32	-1.713	-16.005	8.493	1.00	0.00
ATOM	519	HA	SER	32	-0.886	-15.353	8.212	1.00	0.00
ATOM	520	CB	SER	32	-2.054	-15.756	9.962	1.00	0.00
ATOM	521	HB2	SER	32	-1.170	-15.931	10.576	1.00	0.00
ATOM	522	HB3	SER	32	-2.386	-14.726	10.090	1.00	0.00
ATOM	523	OG	SER	32	-3.092	-16.642	10.362	1.00	0.00
ATOM	524	HG	SER	32	-3.307	-16.487	11.285	1.00	0.00
ATOM	525	C	SER	32	-1.244	-17.446	8.312	1.00	0.00
ATOM	526	O	SER	32	-0.086	-17.768	8.577	1.00	0.00

```
ATOM    527  N   THR    33      -2.152 -18.304   7.860  1.00  0.00
ATOM    528  H   THR    33      -3.101 -18.000   7.698  1.00  0.00
ATOM    529  CA  THR    33      -1.824 -19.702   7.617  1.00  0.00
ATOM    530  HA  THR    33      -1.192 -20.087   8.418  1.00  0.00
ATOM    531  CB  THR    33      -3.111 -20.533   7.567  1.00  0.00
ATOM    532  HB  THR    33      -2.867 -21.564   7.308  1.00  0.00
ATOM    533  CG2 THR    33      -3.799 -20.500   8.934  1.00  0.00
ATOM    534 HG21 THR    33      -4.044 -19.470   9.194  1.00  0.00
ATOM    535 HG22 THR    33      -4.713 -21.092   8.896  1.00  0.00
ATOM    536 HG23 THR    33      -3.130 -20.915   9.688  1.00  0.00
ATOM    537  OG1 THR    33      -3.985 -19.993   6.584  1.00  0.00
ATOM    538  HG1 THR    33      -4.791 -20.513   6.553  1.00  0.00
ATOM    539  C   THR    33      -1.047 -19.876   6.300  1.00  0.00
ATOM    540  O   THR    33       0.060 -20.414   6.285  1.00  0.00
ATOM    541  N   TYR    34      -1.676 -19.455   5.193  1.00  0.00
ATOM    542  H   TYR    34      -2.598 -19.052   5.277  1.00  0.00
ATOM    543  CA  TYR    34      -1.096 -19.598   3.846  1.00  0.00
ATOM    544  HA  TYR    34      -0.309 -20.353   3.849  1.00  0.00
ATOM    545  CB  TYR    34      -2.191 -20.036   2.869  1.00  0.00
ATOM    546  HB2 TYR    34      -2.960 -19.266   2.813  1.00  0.00
ATOM    547  HB3 TYR    34      -1.754 -20.185   1.882  1.00  0.00
ATOM    548  CG  TYR    34      -2.808 -21.330   3.347  1.00  0.00
```

ATOM	549	CD1	TYR	34	-2.130	-22.541	3.160	1.00	0.00
ATOM	550	HD1	TYR	34	-1.157	-22.551	2.668	1.00	0.00
ATOM	551	CE1	TYR	34	-2.701	-23.739	3.605	1.00	0.00
ATOM	552	HE1	TYR	34	-2.173	-24.682	3.459	1.00	0.00
ATOM	553	CZ	TYR	34	-3.950	-23.727	4.238	1.00	0.00
ATOM	554	OH	TYR	34	-4.512	-24.908	4.676	1.00	0.00
ATOM	555	HH	TYR	34	-3.962	-25.675	4.496	1.00	0.00
ATOM	556	CE2	TYR	34	-4.627	-22.516	4.425	1.00	0.00
ATOM	557	HE2	TYR	34	-5.599	-22.507	4.918	1.00	0.00
ATOM	558	CD2	TYR	34	-4.057	-21.319	3.981	1.00	0.00
ATOM	559	HD2	TYR	34	-4.584	-20.377	4.128	1.00	0.00
ATOM	560	C	TYR	34	-0.431	-18.320	3.305	1.00	0.00
ATOM	561	O	TYR	34	-0.047	-18.288	2.135	1.00	0.00
ATOM	562	N	GLN	35	-0.341	-17.266	4.119	1.00	0.00
ATOM	563	H	GLN	35	-0.696	-17.322	5.063	1.00	0.00
ATOM	564	CA	GLN	35	0.239	-15.986	3.664	1.00	0.00
ATOM	565	HA	GLN	35	-0.531	-15.401	3.163	1.00	0.00
ATOM	566	CB	GLN	35	0.791	-15.167	4.837	1.00	0.00
ATOM	567	HB2	GLN	35	0.996	-14.153	4.495	1.00	0.00
ATOM	568	HB3	GLN	35	0.045	-15.139	5.632	1.00	0.00
ATOM	569	CG	GLN	35	2.086	-15.797	5.373	1.00	0.00
ATOM	570	HG2	GLN	35	2.806	-15.925	4.564	1.00	0.00

ATOM	571	HG3	GLN	35	2.516	-15.156	6.143	1.00	0.00
ATOM	572	CD	GLN	35	1.794	-17.160	5.983	1.00	0.00
ATOM	573	OE1	GLN	35	0.591	-17.418	6.400	1.00	0.00
ATOM	574	NE2	GLN	35	2.680	-18.008	6.076	1.00	0.00
ATOM	575	HE21	GLN	35	2.472	-18.909	6.483	1.00	0.00
ATOM	576	HE22	GLN	35	3.611	-17.800	5.745	1.00	0.00
ATOM	577	C	GLN	35	1.363	-16.169	2.655	1.00	0.00
ATOM	578	O	GLN	35	1.555	-15.313	1.790	1.00	0.00
ATOM	579	N	THR	36	2.111	-17.263	2.757	1.00	0.00
ATOM	580	H	THR	36	1.956	-17.956	3.475	1.00	0.00
ATOM	581	CA	THR	36	3.201	-17.473	1.822	1.00	0.00
ATOM	582	HA	THR	36	3.924	-16.661	1.901	1.00	0.00
ATOM	583	CB	THR	36	3.913	-18.794	2.132	1.00	0.00
ATOM	584	HB	THR	36	3.237	-19.625	1.930	1.00	0.00
ATOM	585	CG2	THR	36	5.161	-18.927	1.255	1.00	0.00
ATOM	586	HG21	THR	36	5.838	-18.097	1.457	1.00	0.00
ATOM	587	HG22	THR	36	5.665	-19.868	1.479	1.00	0.00
ATOM	588	HG23	THR	36	4.871	-18.912	0.205	1.00	0.00
ATOM	589	OG1	THR	36	4.291	-18.814	3.501	1.00	0.00
ATOM	590	HG1	THR	36	4.738	-19.641	3.698	1.00	0.00
ATOM	591	C	THR	36	2.639	-17.502	0.407	1.00	0.00
ATOM	592	O	THR	36	1.979	-18.459	0.003	1.00	0.00

ATOM	593	N	MET	37	2.899	-16.426	-0.327	1.00	0.00
ATOM	594	H	MET	37	3.428	-15.661	0.067	1.00	0.00
ATOM	595	CA	MET	37	2.412	-16.297	-1.697	1.00	0.00
ATOM	596	HA	MET	37	1.326	-16.381	-1.719	1.00	0.00
ATOM	597	CB	MET	37	2.809	-14.929	-2.259	1.00	0.00
ATOM	598	HB2	MET	37	3.886	-14.793	-2.154	1.00	0.00
ATOM	599	HB3	MET	37	2.538	-14.878	-3.313	1.00	0.00
ATOM	600	CG	MET	37	2.077	-13.824	-1.490	1.00	0.00
ATOM	601	HG2	MET	37	2.090	-14.048	-0.423	1.00	0.00
ATOM	602	HG3	MET	37	2.568	-12.867	-1.665	1.00	0.00
ATOM	603	SD	MET	37	0.360	-13.726	-2.058	1.00	0.00
ATOM	604	CE	MET	37	0.640	-12.597	-3.444	1.00	0.00
ATOM	605	HE1	MET	37	1.336	-13.052	-4.149	1.00	0.00
ATOM	606	HE2	MET	37	-0.306	-12.396	-3.946	1.00	0.00
ATOM	607	HE3	MET	37	1.059	-11.662	-3.072	1.00	0.00
ATOM	608	C	MET	37	2.976	-17.402	-2.590	1.00	0.00
ATOM	609	O	MET	37	4.182	-17.458	-2.831	1.00	0.00
ATOM	610	N	PRO	38	2.128	-18.269	-3.092	1.00	0.00
ATOM	611	CD	PRO	38	0.671	-18.287	-2.864	1.00	0.00
ATOM	612	HD2	PRO	38	0.234	-17.385	-3.290	1.00	0.00
ATOM	613	HD3	PRO	38	0.480	-18.312	-1.791	1.00	0.00
ATOM	614	CG	PRO	38	0.197	-19.563	-3.572	1.00	0.00

ATOM	615	HG2	PRO	38	-0.311	-19.288	-4.496	1.00	0.00
ATOM	616	HG3	PRO	38	-0.497	-20.094	-2.920	1.00	0.00
ATOM	617	CB	PRO	38	1.412	-20.389	-3.842	1.00	0.00
ATOM	618	HB2	PRO	38	1.261	-20.916	-4.784	1.00	0.00
ATOM	619	HB3	PRO	38	1.651	-21.109	-3.059	1.00	0.00
ATOM	620	CA	PRO	38	2.548	-19.381	-3.987	1.00	0.00
ATOM	621	HA	PRO	38	3.508	-19.762	-3.638	1.00	0.00
ATOM	622	C	PRO	38	2.682	-18.915	-5.434	1.00	0.00
ATOM	623	O	PRO	38	1.916	-18.066	-5.892	1.00	0.00
ATOM	624	N	ARG	39	3.652	-19.475	-6.150	1.00	0.00
ATOM	625	H	ARG	39	4.248	-20.186	-5.750	1.00	0.00
ATOM	626	CA	ARG	39	3.861	-19.101	-7.546	1.00	0.00
ATOM	627	HA	ARG	39	4.080	-18.036	-7.613	1.00	0.00
ATOM	628	CB	ARG	39	5.031	-19.877	-8.139	1.00	0.00
ATOM	629	HB2	ARG	39	4.765	-20.932	-8.207	1.00	0.00
ATOM	630	HB3	ARG	39	5.250	-19.493	-9.135	1.00	0.00
ATOM	631	CG	ARG	39	6.268	-19.720	-7.249	1.00	0.00
ATOM	632	HG2	ARG	39	6.489	-18.661	-7.118	1.00	0.00
ATOM	633	HG3	ARG	39	6.076	-20.174	-6.276	1.00	0.00
ATOM	634	CD	ARG	39	7.464	-20.413	-7.907	1.00	0.00
ATOM	635	HD2	ARG	39	7.197	-21.435	-8.177	1.00	0.00
ATOM	636	HD3	ARG	39	7.762	-19.867	-8.802	1.00	0.00

ATOM	637	NE	ARG	39	8.596	-20.454	-6.986	1.00	0.00
ATOM	638	HE	ARG	39	9.360	-19.802	-7.093	1.00	0.00
ATOM	639	CZ	ARG	39	8.652	-21.341	-5.992	1.00	0.00
ATOM	640	NH1	ARG	39	7.682	-22.198	-5.821	1.00	0.00
ATOM	641	HH11	ARG	39	6.888	-22.188	-6.444	1.00	0.00
ATOM	642	HH12	ARG	39	7.731	-22.867	-5.066	1.00	0.00
ATOM	643	NH2	ARG	39	9.680	-21.351	-5.188	1.00	0.00
ATOM	644	HH21	ARG	39	10.429	-20.687	-5.322	1.00	0.00
ATOM	645	HH22	ARG	39	9.722	-22.023	-4.435	1.00	0.00
ATOM	646	C	ARG	39	2.629	-19.442	-8.360	1.00	0.00
ATOM	647	O	ARG	39	2.227	-18.691	-9.249	1.00	0.00
ATOM	648	N	ARG	40	2.039	-20.596	-8.053	1.00	0.00
ATOM	649	H	ARG	40	2.417	-21.196	-7.333	1.00	0.00
ATOM	650	CA	ARG	40	0.862	-21.042	-8.770	1.00	0.00
ATOM	651	HA	ARG	40	1.031	-20.966	-9.844	1.00	0.00
ATOM	652	CB	ARG	40	0.551	-22.500	-8.427	1.00	0.00
ATOM	653	HB2	ARG	40	0.533	-22.623	-7.344	1.00	0.00
ATOM	654	HB3	ARG	40	-0.420	-22.772	-8.839	1.00	0.00
ATOM	655	CG	ARG	40	1.630	-23.406	-9.025	1.00	0.00
ATOM	656	HG2	ARG	40	2.612	-22.979	-8.822	1.00	0.00
ATOM	657	HG3	ARG	40	1.564	-24.394	-8.569	1.00	0.00
ATOM	658	CD	ARG	40	1.429	-23.528	-10.540	1.00	0.00

ATOM	659	HD2	ARG	40	1.443	-22.538	-10.995	1.00	0.00
ATOM	660	HD3	ARG	40	2.225	-24.136	-10.970	1.00	0.00
ATOM	661	NE	ARG	40	0.146	-24.161	-10.834	1.00	0.00
ATOM	662	HE	ARG	40	-0.647	-23.595	-11.097	1.00	0.00
ATOM	663	CZ	ARG	40	-0.011	-25.482	-10.764	1.00	0.00
ATOM	664	NH1	ARG	40	0.991	-26.247	-10.422	1.00	0.00
ATOM	665	HH11	ARG	40	1.888	-25.835	-10.210	1.00	0.00
ATOM	666	HH12	ARG	40	0.864	-27.248	-10.371	1.00	0.00
ATOM	667	NH2	ARG	40	-1.171	-26.014	-11.038	1.00	0.00
ATOM	668	HH21	ARG	40	-1.945	-25.422	-11.302	1.00	0.00
ATOM	669	HH22	ARG	40	-1.289	-27.016	-10.984	1.00	0.00
ATOM	670	C	ARG	40	-0.323	-20.164	-8.429	1.00	0.00
ATOM	671	O	ARG	40	-0.969	-20.327	-7.392	1.00	0.00
ATOM	672	N	GLY	41	-0.592	-19.240	-9.330	1.00	0.00
ATOM	673	H	GLY	41	-0.017	-19.164	-10.157	1.00	0.00
ATOM	674	CA	GLY	41	-1.708	-18.309	-9.173	1.00	0.00
ATOM	675	HA2	GLY	41	-1.645	-17.548	-9.950	1.00	0.00
ATOM	676	HA3	GLY	41	-2.643	-18.858	-9.278	1.00	0.00
ATOM	677	C	GLY	41	-1.682	-17.628	-7.807	1.00	0.00
ATOM	678	O	GLY	41	-2.338	-18.080	-6.867	1.00	0.00
ATOM	679	N	MET	42	-0.926	-16.535	-7.707	1.00	0.00
ATOM	680	H	MET	42	-0.393	-16.210	-8.501	1.00	0.00

ATOM	681	CA	MET	42	-0.823	-15.791	-6.451	1.00	0.00
ATOM	682	HA	MET	42	-0.377	-16.419	-5.679	1.00	0.00
ATOM	683	CB	MET	42	0.062	-14.556	-6.649	1.00	0.00
ATOM	684	HB2	MET	42	-0.312	-13.973	-7.490	1.00	0.00
ATOM	685	HB3	MET	42	0.040	-13.946	-5.746	1.00	0.00
ATOM	686	CG	MET	42	1.501	-14.994	-6.933	1.00	0.00
ATOM	687	HG2	MET	42	1.859	-15.633	-6.125	1.00	0.00
ATOM	688	HG3	MET	42	1.541	-15.543	-7.874	1.00	0.00
ATOM	689	SD	MET	42	2.563	-13.534	-7.051	1.00	0.00
ATOM	690	CE	MET	42	4.090	-14.397	-7.498	1.00	0.00
ATOM	691	HE1	MET	42	3.942	-14.939	-8.432	1.00	0.00
ATOM	692	HE2	MET	42	4.895	-13.672	-7.623	1.00	0.00
ATOM	693	HE3	MET	42	4.355	-15.101	-6.708	1.00	0.00
ATOM	694	C	MET	42	-2.206	-15.357	-5.966	1.00	0.00
ATOM	695	O	MET	42	-3.199	-15.503	-6.680	1.00	0.00
ATOM	696	N	GLU	43	-2.264	-14.825	-4.746	1.00	0.00
ATOM	697	H	GLU	43	-1.430	-14.738	-4.182	1.00	0.00
ATOM	698	CA	GLU	43	-3.534	-14.380	-4.178	1.00	0.00
ATOM	699	HA	GLU	43	-4.282	-15.167	-4.274	1.00	0.00
ATOM	700	CB	GLU	43	-3.357	-14.052	-2.691	1.00	0.00
ATOM	701	HB2	GLU	43	-2.760	-14.831	-2.216	1.00	0.00
ATOM	702	HB3	GLU	43	-2.849	-13.093	-2.588	1.00	0.00

ATOM	703	CG	GLU	43	-4.730	-13.978	-2.015	1.00	0.00
ATOM	704	HG2	GLU	43	-5.312	-13.172	-2.460	1.00	0.00
ATOM	705	HG3	GLU	43	-5.247	-14.926	-2.167	1.00	0.00
ATOM	706	CD	GLU	43	-4.568	-13.722	-0.518	1.00	0.00
ATOM	707	OE1	GLU	43	-4.045	-12.678	-0.168	1.00	0.00
ATOM	708	OE2	GLU	43	-4.978	-14.571	0.257	1.00	0.00
ATOM	709	C	GLU	43	-4.050	-13.146	-4.921	1.00	0.00
ATOM	710	O	GLU	43	-3.326	-12.163	-5.081	1.00	0.00
ATOM	711	N	PRO	44	-5.280	-13.187	-5.380	1.00	0.00
ATOM	712	CD	PRO	44	-6.212	-14.317	-5.230	1.00	0.00
ATOM	713	HD2	PRO	44	-6.204	-14.653	-4.193	1.00	0.00
ATOM	714	HD3	PRO	44	-5.892	-15.134	-5.878	1.00	0.00
ATOM	715	CG	PRO	44	-7.560	-13.736	-5.648	1.00	0.00
ATOM	716	HG2	PRO	44	-8.097	-13.404	-4.760	1.00	0.00
ATOM	717	HG3	PRO	44	-8.140	-14.507	-6.156	1.00	0.00
ATOM	718	CB	PRO	44	-7.268	-12.597	-6.566	1.00	0.00
ATOM	719	HB2	PRO	44	-8.030	-11.828	-6.439	1.00	0.00
ATOM	720	HB3	PRO	44	-7.230	-12.903	-7.611	1.00	0.00
ATOM	721	CA	PRO	44	-5.902	-12.053	-6.131	1.00	0.00
ATOM	722	HA	PRO	44	-5.265	-11.828	-6.986	1.00	0.00
ATOM	723	C	PRO	44	-6.064	-10.800	-5.273	1.00	0.00
ATOM	724	O	PRO	44	-6.116	-9.686	-5.798	1.00	0.00

ATOM 725 N HIE 45 -6.148 -10.987 -3.958 1.00 0.00

ATOM 726 H HIE 45 -6.120 -11.918 -3.568 1.00 0.00

ATOM 727 CA HIE 45 -6.310 -9.858 -3.045 1.00 0.00

ATOM 728 HA HIE 45 -7.283 -9.393 -3.198 1.00 0.00

ATOM 729 CB HIE 45 -6.219 -10.336 -1.593 1.00 0.00

ATOM 730 HB2 HIE 45 -5.301 -10.890 -1.396 1.00 0.00

ATOM 731 HB3 HIE 45 -6.304 -9.512 -0.885 1.00 0.00

ATOM 732 CG HIE 45 -7.359 -11.274 -1.301 1.00 0.00

ATOM 733 ND1 HIE 45 -7.153 -12.584 -0.899 1.00 0.00

ATOM 734 CE1 HIE 45 -8.361 -13.150 -0.726 1.00 0.00

ATOM 735 HE1 HIE 45 -8.383 -14.192 -0.407 1.00 0.00

ATOM 736 NE2 HIE 45 -9.351 -12.293 -0.988 1.00 0.00

ATOM 737 HE2 HIE 45 -10.351 -12.429 -0.946 1.00 0.00

ATOM 738 CD2 HIE 45 -8.721 -11.108 -1.351 1.00 0.00

ATOM 739 HD2 HIE 45 -9.315 -10.235 -1.618 1.00 0.00

ATOM 740 C HIE 45 -5.238 -8.802 -3.304 1.00 0.00

ATOM 741 O HIE 45 -5.536 -7.608 -3.375 1.00 0.00

ATOM 742 N MET 46 -3.994 -9.251 -3.455 1.00 0.00

ATOM 743 H MET 46 -3.791 -10.238 -3.387 1.00 0.00

ATOM 744 CA MET 46 -2.889 -8.334 -3.717 1.00 0.00

ATOM 745 HA MET 46 -2.860 -7.557 -2.953 1.00 0.00

ATOM 746 CB MET 46 -1.558 -9.094 -3.692 1.00 0.00

ATOM 747 HB2 MET 46 -1.531 -9.794 -4.527 1.00 0.00

ATOM 748 HB3 MET 46 -0.738 -8.382 -3.788 1.00 0.00

ATOM 749 CG MET 46 -1.412 -9.864 -2.377 1.00 0.00

ATOM 750 HG2 MET 46 -2.130 -10.684 -2.352 1.00 0.00

ATOM 751 HG3 MET 46 -0.401 -10.265 -2.299 1.00 0.00

ATOM 752 SD MET 46 -1.722 -8.759 -0.978 1.00 0.00

ATOM 753 CE MET 46 -2.328 -10.023 0.168 1.00 0.00

ATOM 754 HE1 MET 46 -1.551 -10.772 0.328 1.00 0.00

ATOM 755 HE2 MET 46 -2.585 -9.558 1.120 1.00 0.00

ATOM 756 HE3 MET 46 -3.212 -10.502 -0.253 1.00 0.00

ATOM 757 C MET 46 -3.065 -7.681 -5.087 1.00 0.00

ATOM 758 O MET 46 -2.850 -6.480 -5.251 1.00 0.00

ATOM 759 N SER 47 -3.458 -8.497 -6.063 1.00 0.00

ATOM 760 H SER 47 -3.597 -9.479 -5.868 1.00 0.00

ATOM 761 CA SER 47 -3.666 -8.026 -7.430 1.00 0.00

ATOM 762 HA SER 47 -2.738 -7.618 -7.831 1.00 0.00

ATOM 763 CB SER 47 -4.112 -9.190 -8.317 1.00 0.00

ATOM 764 HB2 SER 47 -5.104 -9.522 -8.012 1.00 0.00

ATOM 765 HB3 SER 47 -4.141 -8.866 -9.357 1.00 0.00

ATOM 766 OG SER 47 -3.191 -10.264 -8.182 1.00 0.00

ATOM 767 HG SER 47 -3.469 -10.996 -8.738 1.00 0.00

ATOM 768 C SER 47 -4.710 -6.913 -7.489 1.00 0.00

ATOM	769	O	SER	47	-4.627	-6.020	-8.332	1.00	0.00
ATOM	770	N	GLU	48	-5.701	-6.982	-6.604	1.00	0.00
ATOM	771	H	GLU	48	-5.749	-7.744	-5.942	1.00	0.00
ATOM	772	CA	GLU	48	-6.763	-5.979	-6.585	1.00	0.00
ATOM	773	HA	GLU	48	-7.327	-6.014	-7.516	1.00	0.00
ATOM	774	CB	GLU	48	-7.723	-6.255	-5.419	1.00	0.00
ATOM	775	HB2	GLU	48	-7.547	-7.262	-5.039	1.00	0.00
ATOM	776	HB3	GLU	48	-7.542	-5.531	-4.625	1.00	0.00
ATOM	777	CG	GLU	48	-9.176	-6.136	-5.898	1.00	0.00
ATOM	778	HG2	GLU	48	-9.224	-6.392	-6.956	1.00	0.00
ATOM	779	HG3	GLU	48	-9.790	-6.833	-5.328	1.00	0.00
ATOM	780	CD	GLU	48	-9.697	-4.714	-5.694	1.00	0.00
ATOM	781	OE1	GLU	48	-8.949	-3.782	-5.940	1.00	0.00
ATOM	782	OE2	GLU	48	-10.843	-4.577	-5.297	1.00	0.00
ATOM	783	C	GLU	48	-6.175	-4.575	-6.451	1.00	0.00
ATOM	784	O	GLU	48	-6.464	-3.694	-7.262	1.00	0.00
ATOM	785	N	CYX	49	-5.342	-4.377	-5.433	1.00	0.00
ATOM	786	H	CYX	49	-5.152	-5.117	-4.772	1.00	0.00
ATOM	787	CA	CYX	49	-4.713	-3.077	-5.216	1.00	0.00
ATOM	788	HA	CYX	49	-5.467	-2.291	-5.236	1.00	0.00
ATOM	789	CB	CYX	49	-4.010	-3.049	-3.848	1.00	0.00
ATOM	790	HB2	CYX	49	-3.804	-4.069	-3.523	1.00	0.00

ATOM	791	HB3	CYX	49	-3.072	-2.500	-3.932	1.00	0.00
ATOM	792	SG	CYX	49	-5.078	-2.222	-2.641	1.00	0.00
ATOM	793	C	CYX	49	-3.707	-2.769	-6.327	1.00	0.00
ATOM	794	O	CYX	49	-3.506	-1.609	-6.680	1.00	0.00
ATOM	795	N	CYX	50	-3.078	-3.817	-6.865	1.00	0.00
ATOM	796	H	CYX	50	-3.274	-4.749	-6.526	1.00	0.00
ATOM	797	CA	CYX	50	-2.079	-3.659	-7.928	1.00	0.00
ATOM	798	HA	CYX	50	-1.191	-3.163	-7.535	1.00	0.00
ATOM	799	CB	CYX	50	-1.672	-5.027	-8.474	1.00	0.00
ATOM	800	HB2	CYX	50	-1.400	-5.683	-7.647	1.00	0.00
ATOM	801	HB3	CYX	50	-2.506	-5.463	-9.023	1.00	0.00
ATOM	802	SG	CYX	50	-0.280	-4.832	-9.619	1.00	0.00
ATOM	803	C	CYX	50	-2.592	-2.806	-9.085	1.00	0.00
ATOM	804	O	CYX	50	-1.870	-1.953	-9.602	1.00	0.00
ATOM	805	N	GLU	51	-3.828	-3.055	-9.505	1.00	0.00
ATOM	806	H	GLU	51	-4.381	-3.784	-9.077	1.00	0.00
ATOM	807	CA	GLU	51	-4.405	-2.314	-10.622	1.00	0.00
ATOM	808	HA	GLU	51	-3.806	-2.465	-11.520	1.00	0.00
ATOM	809	CB	GLU	51	-5.831	-2.806	-10.895	1.00	0.00
ATOM	810	HB2	GLU	51	-5.824	-3.890	-11.013	1.00	0.00
ATOM	811	HB3	GLU	51	-6.473	-2.537	-10.056	1.00	0.00
ATOM	812	CG	GLU	51	-6.365	-2.155	-12.178	1.00	0.00

ATOM	813	HG2	GLU	51	-7.412	-2.429	-12.308	1.00	0.00
ATOM	814	HG3	GLU	51	-6.282	-1.073	-12.082	1.00	0.00
ATOM	815	CD	GLU	51	-5.560	-2.623	-13.394	1.00	0.00
ATOM	816	OE1	GLU	51	-5.069	-3.741	-13.372	1.00	0.00
ATOM	817	OE2	GLU	51	-5.450	-1.854	-14.334	1.00	0.00
ATOM	818	C	GLU	51	-4.422	-0.810	-10.349	1.00	0.00
ATOM	819	O	GLU	51	-4.204	-0.009	-11.256	1.00	0.00
ATOM	820	N	GLN	52	-4.693	-0.433	-9.102	1.00	0.00
ATOM	821	H	GLN	52	-4.898	-1.121	-8.391	1.00	0.00
ATOM	822	CA	GLN	52	-4.748	0.981	-8.734	1.00	0.00
ATOM	823	HA	GLN	52	-5.505	1.496	-9.324	1.00	0.00
ATOM	824	CB	GLN	52	-5.111	1.108	-7.257	1.00	0.00
ATOM	825	HB2	GLN	52	-4.353	0.613	-6.649	1.00	0.00
ATOM	826	HB3	GLN	52	-5.166	2.161	-6.982	1.00	0.00
ATOM	827	CG	GLN	52	-6.471	0.446	-7.015	1.00	0.00
ATOM	828	HG2	GLN	52	-6.602	-0.394	-7.697	1.00	0.00
ATOM	829	HG3	GLN	52	-6.532	0.091	-5.986	1.00	0.00
ATOM	830	CD	GLN	52	-7.597	1.448	-7.253	1.00	0.00
ATOM	831	OE1	GLN	52	-7.700	2.024	-8.335	1.00	0.00
ATOM	832	NE2	GLN	52	-8.454	1.690	-6.298	1.00	0.00
ATOM	833	HE21	GLN	52	-9.201	2.352	-6.450	1.00	0.00
ATOM	834	HE22	GLN	52	-8.365	1.213	-5.411	1.00	0.00

ATOM	835	C	GLN	52	-3.419	1.684	-9.012	1.00	0.00
ATOM	836	O	GLN	52	-3.385	2.697	-9.710	1.00	0.00
ATOM	837	N	LEU	53	-2.323	1.134	-8.481	1.00	0.00
ATOM	838	H	LEU	53	-2.380	0.278	-7.947	1.00	0.00
ATOM	839	CA	LEU	53	-1.000	1.724	-8.710	1.00	0.00
ATOM	840	HA	LEU	53	-0.971	2.729	-8.289	1.00	0.00
ATOM	841	CB	LEU	53	0.100	0.884	-8.047	1.00	0.00
ATOM	842	HB2	LEU	53	0.266	0.005	-8.670	1.00	0.00
ATOM	843	HB3	LEU	53	1.015	1.475	-8.004	1.00	0.00
ATOM	844	CG	LEU	53	-0.289	0.440	-6.630	1.00	0.00
ATOM	845	HG	LEU	53	0.608	0.445	-6.009	1.00	0.00
ATOM	846	CD1	LEU	53	-1.344	1.372	-6.013	1.00	0.00
ATOM	847	HD11	LEU	53	-2.241	1.367	-6.632	1.00	0.00
ATOM	848	HD12	LEU	53	-1.593	1.025	-5.010	1.00	0.00
ATOM	849	HD13	LEU	53	-0.946	2.385	-5.958	1.00	0.00
ATOM	850	CD2	LEU	53	-0.844	-0.981	-6.717	1.00	0.00
ATOM	851	HD21	LEU	53	-0.081	-1.646	-7.123	1.00	0.00
ATOM	852	HD22	LEU	53	-1.129	-1.322	-5.721	1.00	0.00
ATOM	853	HD23	LEU	53	-1.718	-0.991	-7.368	1.00	0.00
ATOM	854	C	LEU	53	-0.730	1.716	-10.196	1.00	0.00
ATOM	855	O	LEU	53	-0.355	2.719	-10.803	1.00	0.00
ATOM	856	N	GLU	54	-0.949	0.541	-10.750	1.00	0.00

ATOM	857	H	GLU	54	-1.214	-0.230	-10.153	1.00	0.00
ATOM	858	CA	GLU	54	-0.775	0.286	-12.158	1.00	0.00
ATOM	859	HA	GLU	54	0.268	0.390	-12.458	1.00	0.00
ATOM	860	CB	GLU	54	-1.211	-1.155	-12.403	1.00	0.00
ATOM	861	HB2	GLU	54	-0.503	-1.825	-11.914	1.00	0.00
ATOM	862	HB3	GLU	54	-2.203	-1.301	-11.975	1.00	0.00
ATOM	863	CG	GLU	54	-1.252	-1.462	-13.906	1.00	0.00
ATOM	864	HG2	GLU	54	-1.885	-0.725	-14.400	1.00	0.00
ATOM	865	HG3	GLU	54	-0.241	-1.400	-14.309	1.00	0.00
ATOM	866	CD	GLU	54	-1.814	-2.865	-14.153	1.00	0.00
ATOM	867	OE1	GLU	54	-2.131	-3.545	-13.189	1.00	0.00
ATOM	868	OE2	GLU	54	-1.918	-3.240	-15.310	1.00	0.00
ATOM	869	C	GLU	54	-1.574	1.285	-12.996	1.00	0.00
ATOM	870	O	GLU	54	-1.154	1.667	-14.089	1.00	0.00
ATOM	871	N	GLY	55	-2.713	1.720	-12.462	1.00	0.00
ATOM	872	H	GLY	55	-3.015	1.378	-11.561	1.00	0.00
ATOM	873	CA	GLY	55	-3.556	2.695	-13.152	1.00	0.00
ATOM	874	HA2	GLY	55	-3.463	2.557	-14.229	1.00	0.00
ATOM	875	HA3	GLY	55	-4.595	2.551	-12.855	1.00	0.00
ATOM	876	C	GLY	55	-3.129	4.118	-12.794	1.00	0.00
ATOM	877	O	GLY	55	-3.526	5.082	-13.449	1.00	0.00
ATOM	878	N	MET	56	-2.313	4.232	-11.748	1.00	0.00

ATOM	879	H	MET	56	-2.032	3.405	-11.240	1.00	0.00
ATOM	880	CA	MET	56	-1.818	5.529	-11.290	1.00	0.00
ATOM	881	HA	MET	56	-2.572	6.299	-11.450	1.00	0.00
ATOM	882	CB	MET	56	-1.501	5.457	-9.789	1.00	0.00
ATOM	883	HB2	MET	56	-2.417	5.239	-9.239	1.00	0.00
ATOM	884	HB3	MET	56	-0.775	4.663	-9.614	1.00	0.00
ATOM	885	CG	MET	56	-0.923	6.791	-9.309	1.00	0.00
ATOM	886	HG2	MET	56	-0.542	6.682	-8.293	1.00	0.00
ATOM	887	HG3	MET	56	-0.112	7.099	-9.969	1.00	0.00
ATOM	888	SD	MET	56	-2.217	8.054	-9.326	1.00	0.00
ATOM	889	CE	MET	56	-2.205	8.417	-7.555	1.00	0.00
ATOM	890	HE1	MET	56	-1.216	8.770	-7.262	1.00	0.00
ATOM	891	HE2	MET	56	-2.944	9.187	-7.336	1.00	0.00
ATOM	892	HE3	MET	56	-2.447	7.512	-6.996	1.00	0.00
ATOM	893	C	MET	56	-0.563	5.924	-12.061	1.00	0.00
ATOM	894	O	MET	56	0.280	5.076	-12.363	1.00	0.00
ATOM	895	N	ASP	57	-0.439	7.216	-12.374	1.00	0.00
ATOM	896	H	ASP	57	-1.162	7.877	-12.127	1.00	0.00
ATOM	897	CA	ASP	57	0.728	7.706	-13.104	1.00	0.00
ATOM	898	HA	ASP	57	0.735	7.295	-14.114	1.00	0.00
ATOM	899	CB	ASP	57	0.688	9.236	-13.197	1.00	0.00
ATOM	900	HB2	ASP	57	0.616	9.648	-12.190	1.00	0.00

ATOM	901	HB3	ASP	57	1.607	9.586	-13.667	1.00	0.00
ATOM	902	CG	ASP	57	-0.515	9.695	-14.024	1.00	0.00
ATOM	903	OD1	ASP	57	-1.080	8.878	-14.735	1.00	0.00
ATOM	904	OD2	ASP	57	-0.854	10.864	-13.934	1.00	0.00
ATOM	905	C	ASP	57	2.011	7.264	-12.404	1.00	0.00
ATOM	906	O	ASP	57	2.058	7.180	-11.177	1.00	0.00
ATOM	907	N	GLU	58	3.043	6.970	-13.190	1.00	0.00
ATOM	908	H	GLU	58	2.958	7.034	-14.194	1.00	0.00
ATOM	909	CA	GLU	58	4.314	6.519	-12.631	1.00	0.00
ATOM	910	HA	GLU	58	4.189	5.552	-12.144	1.00	0.00
ATOM	911	CB	GLU	58	5.347	6.373	-13.750	1.00	0.00
ATOM	912	HB2	GLU	58	5.073	5.539	-14.396	1.00	0.00
ATOM	913	HB3	GLU	58	5.384	7.290	-14.337	1.00	0.00
ATOM	914	CG	GLU	58	6.719	6.108	-13.133	1.00	0.00
ATOM	915	HG2	GLU	58	6.985	6.943	-12.485	1.00	0.00
ATOM	916	HG3	GLU	58	6.678	5.191	-12.544	1.00	0.00
ATOM	917	CD	GLU	58	7.770	5.961	-14.230	1.00	0.00
ATOM	918	OE1	GLU	58	7.775	4.931	-14.886	1.00	0.00
ATOM	919	OE2	GLU	58	8.557	6.879	-14.397	1.00	0.00
ATOM	920	C	GLU	58	4.849	7.486	-11.578	1.00	0.00
ATOM	921	O	GLU	58	5.091	7.096	-10.436	1.00	0.00
ATOM	922	N	SER	59	5.036	8.743	-11.966	1.00	0.00

ATOM	923	H	SER	59	4.843	9.021	-12.917	1.00	0.00
ATOM	924	CA	SER	59	5.545	9.750	-11.040	1.00	0.00
ATOM	925	HA	SER	59	6.550	9.483	-10.710	1.00	0.00
ATOM	926	CB	SER	59	5.604	11.111	-11.736	1.00	0.00
ATOM	927	HB2	SER	59	4.598	11.423	-12.015	1.00	0.00
ATOM	928	HB3	SER	59	6.039	11.848	-11.061	1.00	0.00
ATOM	929	OG	SER	59	6.408	11.006	-12.904	1.00	0.00
ATOM	930	HG	SER	59	6.447	11.858	-13.343	1.00	0.00
ATOM	931	C	SER	59	4.648	9.839	-9.812	1.00	0.00
ATOM	932	O	SER	59	5.114	10.076	-8.697	1.00	0.00
ATOM	933	N	CYX	60	3.357	9.642	-10.040	1.00	0.00
ATOM	934	H	CYX	60	3.035	9.474	-10.982	1.00	0.00
ATOM	935	CA	CYX	60	2.366	9.694	-8.967	1.00	0.00
ATOM	936	HA	CYX	60	2.562	10.547	-8.318	1.00	0.00
ATOM	937	CB	CYX	60	0.963	9.841	-9.559	1.00	0.00
ATOM	938	HB2	CYX	60	0.740	8.976	-10.183	1.00	0.00
ATOM	939	HB3	CYX	60	0.233	9.904	-8.752	1.00	0.00
ATOM	940	SG	CYX	60	0.884	11.353	-10.550	1.00	0.00
ATOM	941	C	CYX	60	2.429	8.441	-8.101	1.00	0.00
ATOM	942	O	CYX	60	2.023	8.456	-6.938	1.00	0.00
ATOM	943	N	ARG	61	2.916	7.352	-8.688	1.00	0.00
ATOM	944	H	ARG	61	3.222	7.389	-9.650	1.00	0.00

ATOM	945	CA	ARG	61	3.005	6.079	-7.983	1.00	0.00
ATOM	946	HA	ARG	61	2.020	5.766	-7.637	1.00	0.00
ATOM	947	CB	ARG	61	3.551	5.011	-8.935	1.00	0.00
ATOM	948	HB2	ARG	61	2.999	5.053	-9.873	1.00	0.00
ATOM	949	HB3	ARG	61	4.606	5.208	-9.125	1.00	0.00
ATOM	950	CG	ARG	61	3.396	3.619	-8.314	1.00	0.00
ATOM	951	HG2	ARG	61	3.867	3.601	-7.331	1.00	0.00
ATOM	952	HG3	ARG	61	2.338	3.378	-8.214	1.00	0.00
ATOM	953	CD	ARG	61	4.070	2.587	-9.220	1.00	0.00
ATOM	954	HD2	ARG	61	5.150	2.738	-9.219	1.00	0.00
ATOM	955	HD3	ARG	61	3.845	1.579	-8.870	1.00	0.00
ATOM	956	NE	ARG	61	3.579	2.728	-10.589	1.00	0.00
ATOM	957	HE	ARG	61	3.976	3.436	-11.190	1.00	0.00
ATOM	958	CZ	ARG	61	2.612	1.952	-11.074	1.00	0.00
ATOM	959	NH1	ARG	61	2.097	0.999	-10.343	1.00	0.00
ATOM	960	HH11	ARG	61	2.435	0.849	-9.403	1.00	0.00
ATOM	961	HH12	ARG	61	1.364	0.416	-10.721	1.00	0.00
ATOM	962	NH2	ARG	61	2.182	2.142	-12.290	1.00	0.00
ATOM	963	HH21	ARG	61	2.585	2.875	-12.856	1.00	0.00
ATOM	964	HH22	ARG	61	1.449	1.555	-12.660	1.00	0.00
ATOM	965	C	ARG	61	3.916	6.189	-6.763	1.00	0.00
ATOM	966	O	ARG	61	3.623	5.617	-5.712	1.00	0.00

ATOM	967	N	CYX	62	5.016	6.927	-6.903	1.00	0.00
ATOM	968	H	CYX	62	5.232	7.373	-7.783	1.00	0.00
ATOM	969	CA	CYX	62	5.950	7.097	-5.794	1.00	0.00
ATOM	970	HA	CYX	62	6.371	6.132	-5.509	1.00	0.00
ATOM	971	CB	CYX	62	7.096	8.028	-6.210	1.00	0.00
ATOM	972	HB2	CYX	62	6.770	9.065	-6.129	1.00	0.00
ATOM	973	HB3	CYX	62	7.953	7.865	-5.556	1.00	0.00
ATOM	974	SG	CYX	62	7.559	7.702	-7.930	1.00	0.00
ATOM	975	C	CYX	62	5.225	7.687	-4.586	1.00	0.00
ATOM	976	O	CYX	62	5.183	7.084	-3.513	1.00	0.00
ATOM	977	N	GLU	63	4.645	8.869	-4.784	1.00	0.00
ATOM	978	H	GLU	63	4.720	9.320	-5.684	1.00	0.00
ATOM	979	CA	GLU	63	3.904	9.551	-3.728	1.00	0.00
ATOM	980	HA	GLU	63	4.559	9.760	-2.881	1.00	0.00
ATOM	981	CB	GLU	63	3.356	10.877	-4.263	1.00	0.00
ATOM	982	HB2	GLU	63	4.164	11.434	-4.738	1.00	0.00
ATOM	983	HB3	GLU	63	2.575	10.674	-4.995	1.00	0.00
ATOM	984	CG	GLU	63	2.772	11.706	-3.109	1.00	0.00
ATOM	985	HG2	GLU	63	2.051	11.096	-2.564	1.00	0.00
ATOM	986	HG3	GLU	63	3.582	11.996	-2.439	1.00	0.00
ATOM	987	CD	GLU	63	2.078	12.961	-3.643	1.00	0.00
ATOM	988	OE1	GLU	63	1.982	13.104	-4.853	1.00	0.00

ATOM	989	OE2	GLU	63	1.649	13.764	-2.830	1.00	0.00
ATOM	990	C	GLU	63	2.742	8.692	-3.234	1.00	0.00
ATOM	991	O	GLU	63	2.412	8.695	-2.048	1.00	0.00
ATOM	992	N	GLY	64	2.109	7.981	-4.168	1.00	0.00
ATOM	993	H	GLY	64	2.423	8.015	-5.127	1.00	0.00
ATOM	994	CA	GLY	64	0.958	7.141	-3.845	1.00	0.00
ATOM	995	HA2	GLY	64	0.126	7.786	-3.565	1.00	0.00
ATOM	996	HA3	GLY	64	0.690	6.565	-4.730	1.00	0.00
ATOM	997	C	GLY	64	1.248	6.180	-2.698	1.00	0.00
ATOM	998	O	GLY	64	0.561	6.204	-1.677	1.00	0.00
ATOM	999	N	LEU	65	2.255	5.330	-2.872	1.00	0.00
ATOM	1000	H	LEU	65	2.777	5.318	-3.736	1.00	0.00
ATOM	1001	CA	LEU	65	2.605	4.360	-1.839	1.00	0.00
ATOM	1002	HA	LEU	65	1.769	3.682	-1.666	1.00	0.00
ATOM	1003	CB	LEU	65	3.820	3.536	-2.278	1.00	0.00
ATOM	1004	HB2	LEU	65	4.650	4.206	-2.501	1.00	0.00
ATOM	1005	HB3	LEU	65	4.108	2.857	-1.475	1.00	0.00
ATOM	1006	CG	LEU	65	3.467	2.725	-3.530	1.00	0.00
ATOM	1007	HG	LEU	65	3.238	3.405	-4.350	1.00	0.00
ATOM	1008	CD1	LEU	65	4.654	1.840	-3.918	1.00	0.00
ATOM	1009	HD11	LEU	65	4.884	1.159	-3.098	1.00	0.00
ATOM	1010	HD12	LEU	65	4.403	1.263	-4.808	1.00	0.00

ATOM	1011	HD13	LEU	65	5.523	2.465	-4.124	1.00	0.00
ATOM	1012	CD2	LEU	65	2.244	1.843	-3.242	1.00	0.00
ATOM	1013	HD21	LEU	65	1.398	2.472	-2.967	1.00	0.00
ATOM	1014	HD22	LEU	65	1.994	1.266	-4.132	1.00	0.00
ATOM	1015	HD23	LEU	65	2.472	1.162	-2.421	1.00	0.00
ATOM	1016	C	LEU	65	2.900	5.043	-0.508	1.00	0.00
ATOM	1017	O	LEU	65	2.425	4.603	0.537	1.00	0.00
ATOM	1018	N	ARG	66	3.682	6.120	-0.549	1.00	0.00
ATOM	1019	H	ARG	66	4.070	6.447	-1.422	1.00	0.00
ATOM	1020	CA	ARG	66	4.020	6.843	0.673	1.00	0.00
ATOM	1021	HA	ARG	66	4.565	6.190	1.356	1.00	0.00
ATOM	1022	CB	ARG	66	4.904	8.049	0.340	1.00	0.00
ATOM	1023	HB2	ARG	66	4.403	8.672	-0.401	1.00	0.00
ATOM	1024	HB3	ARG	66	5.079	8.631	1.245	1.00	0.00
ATOM	1025	CG	ARG	66	6.244	7.565	-0.222	1.00	0.00
ATOM	1026	HG2	ARG	66	6.725	6.906	0.502	1.00	0.00
ATOM	1027	HG3	ARG	66	6.073	7.020	-1.150	1.00	0.00
ATOM	1028	CD	ARG	66	7.150	8.769	-0.496	1.00	0.00
ATOM	1029	HD2	ARG	66	8.047	8.444	-1.025	1.00	0.00
ATOM	1030	HD3	ARG	66	6.618	9.502	-1.101	1.00	0.00
ATOM	1031	NE	ARG	66	7.550	9.400	0.760	1.00	0.00
ATOM	1032	HE	ARG	66	7.089	10.243	1.070	1.00	0.00

ATOM	1033	CZ	ARG	66	8.518	8.889	1.520	1.00	0.00
ATOM	1034	NH1	ARG	66	9.138	7.800	1.151	1.00	0.00
ATOM	1035	HH11	ARG	66	8.882	7.345	0.287	1.00	0.00
ATOM	1036	HH12	ARG	66	9.871	7.418	1.732	1.00	0.00
ATOM	1037	NH2	ARG	66	8.848	9.478	2.635	1.00	0.00
ATOM	1038	HH21	ARG	66	8.369	10.320	2.919	1.00	0.00
ATOM	1039	HH22	ARG	66	9.582	9.089	3.210	1.00	0.00
ATOM	1040	C	ARG	66	2.756	7.319	1.385	1.00	0.00
ATOM	1041	O	ARG	66	2.556	7.043	2.569	1.00	0.00
ATOM	1042	N	MET	67	1.907	8.032	0.649	1.00	0.00
ATOM	1043	H	MET	67	2.130	8.252	-0.311	1.00	0.00
ATOM	1044	CA	MET	67	0.658	8.545	1.205	1.00	0.00
ATOM	1045	HA	MET	67	0.862	9.170	2.075	1.00	0.00
ATOM	1046	CB	MET	67	-0.068	9.391	0.153	1.00	0.00
ATOM	1047	HB2	MET	67	0.442	10.348	0.043	1.00	0.00
ATOM	1048	HB3	MET	67	-0.062	8.865	-0.802	1.00	0.00
ATOM	1049	CG	MET	67	-1.516	9.631	0.595	1.00	0.00
ATOM	1050	HG2	MET	67	-2.072	8.695	0.551	1.00	0.00
ATOM	1051	HG3	MET	67	-1.529	10.012	1.616	1.00	0.00
ATOM	1052	SD	MET	67	-2.298	10.842	-0.503	1.00	0.00
ATOM	1053	CE	MET	67	-1.315	12.280	-0.007	1.00	0.00
ATOM	1054	HE1	MET	67	-0.261	12.087	-0.208	1.00	0.00

ATOM 1055 HE2 MET 67 -1.638 13.154 -0.572 1.00 0.00
ATOM 1056 HE3 MET 67 -1.453 12.465 1.058 1.00 0.00
ATOM 1057 C MET 67 -0.255 7.414 1.671 1.00 0.00
ATOM 1058 O MET 67 -0.793 7.452 2.778 1.00 0.00
ATOM 1059 N MET 68 -0.442 6.424 0.805 1.00 0.00
ATOM 1060 H MET 68 -0.007 6.455 -0.106 1.00 0.00
ATOM 1061 CA MET 68 -1.314 5.297 1.116 1.00 0.00
ATOM 1062 HA MET 68 -2.312 5.649 1.374 1.00 0.00
ATOM 1063 CB MET 68 -1.418 4.385 -0.110 1.00 0.00
ATOM 1064 HB2 MET 68 -2.123 4.818 -0.819 1.00 0.00
ATOM 1065 HB3 MET 68 -0.437 4.302 -0.578 1.00 0.00
ATOM 1066 CG MET 68 -1.904 2.992 0.307 1.00 0.00
ATOM 1067 HG2 MET 68 -1.113 2.473 0.850 1.00 0.00
ATOM 1068 HG3 MET 68 -2.782 3.084 0.946 1.00 0.00
ATOM 1069 SD MET 68 -2.339 2.038 -1.166 1.00 0.00
ATOM 1070 CE MET 68 -0.662 1.842 -1.814 1.00 0.00
ATOM 1071 HE1 MET 68 -0.049 1.314 -1.083 1.00 0.00
ATOM 1072 HE2 MET 68 -0.694 1.270 -2.741 1.00 0.00
ATOM 1073 HE3 MET 68 -0.230 2.823 -2.008 1.00 0.00
ATOM 1074 C MET 68 -0.811 4.509 2.322 1.00 0.00
ATOM 1075 O MET 68 -1.587 4.188 3.220 1.00 0.00
ATOM 1076 N MET 69 0.482 4.196 2.343 1.00 0.00

ATOM	1077	H	MET	69	1.094	4.457	1.583	1.00	0.00
ATOM	1078	CA	MET	69	1.047	3.440	3.456	1.00	0.00
ATOM	1079	HA	MET	69	0.650	2.425	3.459	1.00	0.00
ATOM	1080	CB	MET	69	2.572	3.369	3.320	1.00	0.00
ATOM	1081	HB2	MET	69	2.829	2.974	2.337	1.00	0.00
ATOM	1082	HB3	MET	69	2.992	4.369	3.430	1.00	0.00
ATOM	1083	CG	MET	69	3.146	2.453	4.404	1.00	0.00
ATOM	1084	HG2	MET	69	2.842	2.810	5.388	1.00	0.00
ATOM	1085	HG3	MET	69	2.781	1.436	4.259	1.00	0.00
ATOM	1086	SD	MET	69	4.953	2.456	4.302	1.00	0.00
ATOM	1087	CE	MET	69	5.258	1.225	5.593	1.00	0.00
ATOM	1088	HE1	MET	69	4.780	0.284	5.319	1.00	0.00
ATOM	1089	HE2	MET	69	6.332	1.068	5.701	1.00	0.00
ATOM	1090	HE3	MET	69	4.847	1.579	6.538	1.00	0.00
ATOM	1091	C	MET	69	0.677	4.095	4.784	1.00	0.00
ATOM	1092	O	MET	69	0.119	3.451	5.673	1.00	0.00
ATOM	1093	N	ARG	70	0.987	5.382	4.902	1.00	0.00
ATOM	1094	H	ARG	70	1.460	5.860	4.149	1.00	0.00
ATOM	1095	CA	ARG	70	0.682	6.133	6.115	1.00	0.00
ATOM	1096	HA	ARG	70	1.159	5.667	6.978	1.00	0.00
ATOM	1097	CB	ARG	70	1.211	7.564	5.977	1.00	0.00
ATOM	1098	HB2	ARG	70	0.772	8.031	5.096	1.00	0.00

ATOM	1099	HB3	ARG	70	0.943	8.138	6.864	1.00	0.00
ATOM	1100	CG	ARG	70	2.736	7.532	5.832	1.00	0.00
ATOM	1101	HG2	ARG	70	3.174	7.059	6.711	1.00	0.00
ATOM	1102	HG3	ARG	70	3.004	6.961	4.943	1.00	0.00
ATOM	1103	CD	ARG	70	3.271	8.960	5.702	1.00	0.00
ATOM	1104	HD2	ARG	70	2.703	9.503	4.947	1.00	0.00
ATOM	1105	HD3	ARG	70	3.190	9.476	6.659	1.00	0.00
ATOM	1106	NE	ARG	70	4.675	8.935	5.302	1.00	0.00
ATOM	1107	HE	ARG	70	5.251	8.137	5.531	1.00	0.00
ATOM	1108	CZ	ARG	70	5.227	9.948	4.634	1.00	0.00
ATOM	1109	NH1	ARG	70	4.518	11.003	4.330	1.00	0.00
ATOM	1110	HH11	ARG	70	3.547	11.050	4.603	1.00	0.00
ATOM	1111	HH12	ARG	70	4.945	11.765	3.824	1.00	0.00
ATOM	1112	NH2	ARG	70	6.482	9.887	4.282	1.00	0.00
ATOM	1113	HH21	ARG	70	7.031	9.072	4.518	1.00	0.00
ATOM	1114	HH22	ARG	70	6.900	10.655	3.776	1.00	0.00
ATOM	1115	C	ARG	70	-0.824	6.168	6.375	1.00	0.00
ATOM	1116	O	ARG	70	-1.270	6.016	7.512	1.00	0.00
ATOM	1117	N	MET	71	-1.598	6.377	5.309	1.00	0.00
ATOM	1118	H	MET	71	-1.178	6.519	4.402	1.00	0.00
ATOM	1119	CA	MET	71	-3.050	6.442	5.417	1.00	0.00
ATOM	1120	HA	MET	71	-3.337	7.189	6.157	1.00	0.00

ATOM	1121	CB	MET	71	-3.656	6.833	4.066	1.00	0.00
ATOM	1122	HB2	MET	71	-3.140	7.712	3.679	1.00	0.00
ATOM	1123	HB3	MET	71	-3.540	6.006	3.365	1.00	0.00
ATOM	1124	CG	MET	71	-5.145	7.150	4.241	1.00	0.00
ATOM	1125	HG2	MET	71	-5.648	6.304	4.710	1.00	0.00
ATOM	1126	HG3	MET	71	-5.261	8.034	4.868	1.00	0.00
ATOM	1127	SD	MET	71	-5.893	7.466	2.622	1.00	0.00
ATOM	1128	CE	MET	71	-4.956	8.961	2.215	1.00	0.00
ATOM	1129	HE1	MET	71	-3.891	8.728	2.194	1.00	0.00
ATOM	1130	HE2	MET	71	-5.266	9.330	1.238	1.00	0.00
ATOM	1131	HE3	MET	71	-5.145	9.725	2.969	1.00	0.00
ATOM	1132	C	MET	71	-3.621	5.106	5.872	1.00	0.00
ATOM	1133	O	MET	71	-4.569	5.062	6.656	1.00	0.00
ATOM	1134	N	MET	72	-3.048	4.020	5.365	1.00	0.00
ATOM	1135	H	MET	72	-2.286	4.096	4.706	1.00	0.00
ATOM	1136	CA	MET	72	-3.522	2.691	5.721	1.00	0.00
ATOM	1137	HA	MET	72	-4.557	2.566	5.404	1.00	0.00
ATOM	1138	CB	MET	72	-2.666	1.625	5.029	1.00	0.00
ATOM	1139	HB2	MET	72	-1.615	1.906	5.100	1.00	0.00
ATOM	1140	HB3	MET	72	-2.819	0.665	5.523	1.00	0.00
ATOM	1141	CG	MET	72	-3.065	1.510	3.555	1.00	0.00
ATOM	1142	HG2	MET	72	-3.036	2.494	3.088	1.00	0.00

ATOM	1143	HG3	MET	72	-2.375	0.842	3.038	1.00	0.00
ATOM	1144	SD	MET	72	-4.744	0.842	3.433	1.00	0.00
ATOM	1145	CE	MET	72	-4.400	-0.782	4.158	1.00	0.00
ATOM	1146	HE1	MET	72	-4.024	-0.655	5.174	1.00	0.00
ATOM	1147	HE2	MET	72	-5.316	-1.372	4.181	1.00	0.00
ATOM	1148	HE3	MET	72	-3.651	-1.298	3.557	1.00	0.00
ATOM	1149	C	MET	72	-3.475	2.483	7.229	1.00	0.00
ATOM	1150	O	MET	72	-4.490	2.167	7.853	1.00	0.00
ATOM	1151	N	GLN	73	-2.293	2.669	7.807	1.00	0.00
ATOM	1152	H	GLN	73	-1.483	2.905	7.251	1.00	0.00
ATOM	1153	CA	GLN	73	-2.120	2.508	9.245	1.00	0.00
ATOM	1154	HA	GLN	73	-2.409	1.500	9.545	1.00	0.00
ATOM	1155	CB	GLN	73	-0.654	2.730	9.626	1.00	0.00
ATOM	1156	HB2	GLN	73	-0.360	3.744	9.357	1.00	0.00
ATOM	1157	HB3	GLN	73	-0.535	2.591	10.701	1.00	0.00
ATOM	1158	CG	GLN	73	0.232	1.728	8.881	1.00	0.00
ATOM	1159	HG2	GLN	73	0.247	1.945	7.813	1.00	0.00
ATOM	1160	HG3	GLN	73	1.247	1.780	9.276	1.00	0.00
ATOM	1161	CD	GLN	73	-0.296	0.314	9.083	1.00	0.00
ATOM	1162	OE1	GLN	73	-0.260	-0.213	10.195	1.00	0.00
ATOM	1163	NE2	GLN	73	-0.803	-0.327	8.069	1.00	0.00
ATOM	1164	HE21	GLN	73	-1.155	-1.266	8.194	1.00	0.00

ATOM 1165 HE22 GLN 73 -0.841 0.117 7.163 1.00 0.00
ATOM 1166 C GLN 73 -3.005 3.490 10.006 1.00 0.00
ATOM 1167 O GLN 73 -3.600 3.146 11.023 1.00 0.00
ATOM 1168 N GLN 74 -3.077 4.717 9.503 1.00 0.00
ATOM 1169 H GLN 74 -2.542 4.965 8.683 1.00 0.00
ATOM 1170 CA GLN 74 -3.882 5.751 10.142 1.00 0.00
ATOM 1171 HA GLN 74 -3.572 5.882 11.179 1.00 0.00
ATOM 1172 CB GLN 74 -3.695 7.080 9.403 1.00 0.00
ATOM 1173 HB2 GLN 74 -2.758 7.541 9.716 1.00 0.00
ATOM 1174 HB3 GLN 74 -3.668 6.897 8.329 1.00 0.00
ATOM 1175 CG GLN 74 -4.859 8.017 9.732 1.00 0.00
ATOM 1176 HG2 GLN 74 -5.401 7.654 10.606 1.00 0.00
ATOM 1177 HG3 GLN 74 -4.483 9.021 9.930 1.00 0.00
ATOM 1178 CD GLN 74 -5.822 8.081 8.553 1.00 0.00
ATOM 1179 OE1 GLN 74 -5.852 7.095 7.702 1.00 0.00
ATOM 1180 NE2 GLN 74 -6.567 9.050 8.407 1.00 0.00
ATOM 1181 HE21 GLN 74 -7.201 9.081 7.622 1.00 0.00
ATOM 1182 HE22 GLN 74 -6.540 9.808 9.074 1.00 0.00
ATOM 1183 C GLN 74 -5.362 5.373 10.178 1.00 0.00
ATOM 1184 O GLN 74 -6.032 5.564 11.194 1.00 0.00
ATOM 1185 N LYS 75 -5.867 4.851 9.067 1.00 0.00
ATOM 1186 H LYS 75 -5.288 4.722 8.249 1.00 0.00

ATOM	1187	CA	LYS	75	-7.274	4.473	8.986	1.00	0.00
ATOM	1188	HA	LYS	75	-7.908	5.344	9.151	1.00	0.00
ATOM	1189	CB	LYS	75	-7.574	3.904	7.596	1.00	0.00
ATOM	1190	HB2	LYS	75	-7.242	4.613	6.838	1.00	0.00
ATOM	1191	HB3	LYS	75	-7.040	2.962	7.470	1.00	0.00
ATOM	1192	CG	LYS	75	-9.081	3.662	7.445	1.00	0.00
ATOM	1193	HG2	LYS	75	-9.257	3.005	6.593	1.00	0.00
ATOM	1194	HG3	LYS	75	-9.463	3.191	8.351	1.00	0.00
ATOM	1195	CD	LYS	75	-9.802	4.995	7.219	1.00	0.00
ATOM	1196	HD2	LYS	75	-9.684	5.625	8.101	1.00	0.00
ATOM	1197	HD3	LYS	75	-9.372	5.498	6.353	1.00	0.00
ATOM	1198	CE	LYS	75	-11.290	4.737	6.972	1.00	0.00
ATOM	1199	HE2	LYS	75	-11.679	4.083	7.752	1.00	0.00
ATOM	1200	HE3	LYS	75	-11.830	5.683	6.989	1.00	0.00
ATOM	1201	NZ	LYS	75	-11.469	4.086	5.643	1.00	0.00
ATOM	1202	HZ1	LYS	75	-10.968	3.208	5.628	1.00	0.00
ATOM	1203	HZ2	LYS	75	-12.451	3.916	5.481	1.00	0.00
ATOM	1204	HZ3	LYS	75	-11.108	4.692	4.920	1.00	0.00
ATOM	1205	C	LYS	75	-7.622	3.436	10.050	1.00	0.00
ATOM	1206	O	LYS	75	-8.677	3.510	10.682	1.00	0.00
ATOM	1207	N	GLU	76	-6.728	2.475	10.237	1.00	0.00
ATOM	1208	H	GLU	76	-5.886	2.442	9.679	1.00	0.00

ATOM	1209	CA	GLU	76	-6.945	1.420	11.226	1.00	0.00
ATOM	1210	HA	GLU	76	-7.259	1.854	12.175	1.00	0.00
ATOM	1211	CB	GLU	76	-8.042	0.464	10.737	1.00	0.00
ATOM	1212	HB2	GLU	76	-8.913	1.042	10.430	1.00	0.00
ATOM	1213	HB3	GLU	76	-7.669	-0.109	9.888	1.00	0.00
ATOM	1214	CG	GLU	76	-8.438	-0.497	11.868	1.00	0.00
ATOM	1215	HG2	GLU	76	-9.102	-1.262	11.465	1.00	0.00
ATOM	1216	HG3	GLU	76	-7.538	-0.969	12.263	1.00	0.00
ATOM	1217	CD	GLU	76	-9.152	0.257	12.991	1.00	0.00
ATOM	1218	OE1	GLU	76	-9.770	1.273	12.708	1.00	0.00
ATOM	1219	OE2	GLU	76	-9.070	-0.193	14.122	1.00	0.00
ATOM	1220	C	GLU	76	-5.654	0.644	11.478	1.00	0.00
ATOM	1221	O	GLU	76	-5.497	-0.483	11.004	1.00	0.00
ATOM	1222	N	MET	77	-4.729	1.253	12.213	1.00	0.00
ATOM	1223	H	MET	77	-4.884	2.188	12.561	1.00	0.00
ATOM	1224	CA	MET	77	-3.453	0.607	12.511	1.00	0.00
ATOM	1225	HA	MET	77	-2.901	0.426	11.589	1.00	0.00
ATOM	1226	CB	MET	77	-2.608	1.509	13.419	1.00	0.00
ATOM	1227	HB2	MET	77	-2.542	2.504	12.980	1.00	0.00
ATOM	1228	HB3	MET	77	-3.077	1.576	14.401	1.00	0.00
ATOM	1229	CG	MET	77	-1.201	0.922	13.566	1.00	0.00
ATOM	1230	HG2	MET	77	-1.264	-0.097	13.948	1.00	0.00

ATOM	1231	HG3	MET	77	-0.701	0.915	12.598	1.00	0.00
ATOM	1232	SD	MET	77	-0.244	1.932	14.722	1.00	0.00
ATOM	1233	CE	MET	77	1.325	1.046	14.556	1.00	0.00
ATOM	1234	HE1	MET	77	1.658	1.087	13.519	1.00	0.00
ATOM	1235	HE2	MET	77	2.075	1.510	15.197	1.00	0.00
ATOM	1236	HE3	MET	77	1.189	0.006	14.853	1.00	0.00
ATOM	1237	C	MET	77	-3.671	-0.746	13.188	1.00	0.00
ATOM	1238	O	MET	77	-3.784	-0.822	14.413	1.00	0.00
ATOM	1239	N	GLN	78	-3.715	-1.810	12.389	1.00	0.00
ATOM	1240	H	GLN	78	-3.634	-1.705	11.388	1.00	0.00
ATOM	1241	CA	GLN	78	-3.900	-3.153	12.931	1.00	0.00
ATOM	1242	HA	GLN	78	-4.886	-3.239	13.388	1.00	0.00
ATOM	1243	CB	GLN	78	-3.787	-4.194	11.810	1.00	0.00
ATOM	1244	HB2	GLN	78	-3.363	-3.725	10.922	1.00	0.00
ATOM	1245	HB3	GLN	78	-3.138	-5.008	12.136	1.00	0.00
ATOM	1246	CG	GLN	78	-5.176	-4.750	11.480	1.00	0.00
ATOM	1247	HG2	GLN	78	-5.623	-5.190	12.372	1.00	0.00
ATOM	1248	HG3	GLN	78	-5.818	-3.952	11.109	1.00	0.00
ATOM	1249	CD	GLN	78	-5.067	-5.829	10.405	1.00	0.00
ATOM	1250	OE1	GLN	78	-4.255	-6.747	10.524	1.00	0.00
ATOM	1251	NE2	GLN	78	-5.842	-5.773	9.356	1.00	0.00
ATOM	1252	HE21	GLN	78	-5.773	-6.485	8.643	1.00	0.00

ATOM 1253 HE22 GLN 78 -6.506 -5.019 9.263 1.00 0.00
ATOM 1254 C GLN 78 -2.841 -3.425 14.000 1.00 0.00
ATOM 1255 O GLN 78 -1.662 -3.135 13.789 1.00 0.00
ATOM 1256 N PRO 79 -3.221 -3.965 15.132 1.00 0.00
ATOM 1257 CD PRO 79 -4.584 -4.360 15.506 1.00 0.00
ATOM 1258 HD2 PRO 79 -4.862 -5.257 14.952 1.00 0.00
ATOM 1259 HD3 PRO 79 -5.272 -3.552 15.259 1.00 0.00
ATOM 1260 CG PRO 79 -4.490 -4.607 17.020 1.00 0.00
ATOM 1261 HG2 PRO 79 -4.603 -5.674 17.213 1.00 0.00
ATOM 1262 HG3 PRO 79 -5.290 -4.061 17.519 1.00 0.00
ATOM 1263 CB PRO 79 -3.152 -4.112 17.461 1.00 0.00
ATOM 1264 HB2 PRO 79 -2.794 -4.772 18.252 1.00 0.00
ATOM 1265 HB3 PRO 79 -3.130 -3.081 17.812 1.00 0.00
ATOM 1266 CA PRO 79 -2.273 -4.259 16.228 1.00 0.00
ATOM 1267 HA PRO 79 -1.452 -3.544 16.177 1.00 0.00
ATOM 1268 C PRO 79 -1.720 -5.672 16.123 1.00 0.00
ATOM 1269 O PRO 79 -2.440 -6.654 16.312 1.00 0.00
ATOM 1270 N ARG 80 -0.438 -5.754 15.833 1.00 0.00
ATOM 1271 H ARG 80 0.097 -4.913 15.669 1.00 0.00
ATOM 1272 CA ARG 80 0.238 -7.036 15.711 1.00 0.00
ATOM 1273 HA ARG 80 0.115 -7.607 16.632 1.00 0.00
ATOM 1274 CB ARG 80 -0.349 -7.844 14.552 1.00 0.00

ATOM 1275 HB2 ARG 80 -1.053 -7.226 13.996 1.00 0.00

ATOM 1276 HB3 ARG 80 0.454 -8.167 13.889 1.00 0.00

ATOM 1277 CG ARG 80 -1.076 -9.072 15.107 1.00 0.00

ATOM 1278 HG2 ARG 80 -0.362 -9.884 15.244 1.00 0.00

ATOM 1279 HG3 ARG 80 -1.529 -8.822 16.067 1.00 0.00

ATOM 1280 CD ARG 80 -2.165 -9.511 14.129 1.00 0.00

ATOM 1281 HD2 ARG 80 -2.008 -9.035 13.161 1.00 0.00

ATOM 1282 HD3 ARG 80 -2.140 -10.594 14.009 1.00 0.00

ATOM 1283 NE ARG 80 -3.482 -9.125 14.633 1.00 0.00

ATOM 1284 HE ARG 80 -3.950 -9.712 15.310 1.00 0.00

ATOM 1285 CZ ARG 80 -4.093 -8.013 14.224 1.00 0.00

ATOM 1286 NH1 ARG 80 -3.517 -7.221 13.360 1.00 0.00

ATOM 1287 HH11 ARG 80 -2.602 -7.453 12.999 1.00 0.00

ATOM 1288 HH12 ARG 80 -3.988 -6.381 13.057 1.00 0.00

ATOM 1289 NH2 ARG 80 -5.273 -7.714 14.691 1.00 0.00

ATOM 1290 HH21 ARG 80 -5.718 -8.326 15.360 1.00 0.00

ATOM 1291 HH22 ARG 80 -5.736 -6.872 14.382 1.00 0.00

ATOM 1292 C ARG 80 1.728 -6.817 15.485 1.00 0.00

ATOM 1293 O ARG 80 2.117 -6.069 14.587 1.00 0.00

ATOM 1294 N GLY 81 2.560 -7.460 16.297 1.00 0.00

ATOM 1295 H GLY 81 2.219 -8.070 17.027 1.00 0.00

ATOM 1296 CA GLY 81 3.999 -7.298 16.151 1.00 0.00

ATOM 1297 HA2 GLY 81 4.202 -6.302 15.759 1.00 0.00
ATOM 1298 HA3 GLY 81 4.465 -7.404 17.131 1.00 0.00
ATOM 1299 C GLY 81 4.595 -8.336 15.203 1.00 0.00
ATOM 1300 O GLY 81 4.973 -8.013 14.080 1.00 0.00
ATOM 1301 N GLU 82 4.689 -9.576 15.673 1.00 0.00
ATOM 1302 H GLU 82 4.389 -9.783 16.615 1.00 0.00
ATOM 1303 CA GLU 82 5.264 -10.660 14.875 1.00 0.00
ATOM 1304 HA GLU 82 6.271 -10.396 14.551 1.00 0.00
ATOM 1305 CB GLU 82 5.340 -11.937 15.718 1.00 0.00
ATOM 1306 HB2 GLU 82 4.356 -12.155 16.133 1.00 0.00
ATOM 1307 HB3 GLU 82 5.660 -12.767 15.088 1.00 0.00
ATOM 1308 CG GLU 82 6.347 -11.746 16.862 1.00 0.00
ATOM 1309 HG2 GLU 82 6.639 -12.726 17.240 1.00 0.00
ATOM 1310 HG3 GLU 82 7.226 -11.231 16.474 1.00 0.00
ATOM 1311 CD GLU 82 5.733 -10.923 17.998 1.00 0.00
ATOM 1312 OE1 GLU 82 4.520 -10.944 18.143 1.00 0.00
ATOM 1313 OE2 GLU 82 6.489 -10.281 18.709 1.00 0.00
ATOM 1314 C GLU 82 4.473 -10.948 13.597 1.00 0.00
ATOM 1315 O GLU 82 5.061 -11.150 12.534 1.00 0.00
ATOM 1316 N GLN 83 3.147 -11.007 13.707 1.00 0.00
ATOM 1317 H GLN 83 2.698 -10.864 14.600 1.00 0.00
ATOM 1318 CA GLN 83 2.307 -11.320 12.550 1.00 0.00

```
ATOM   1319  HA  GLN    83       2.576 -12.295  12.142  1.00  0.00
ATOM   1320  CB  GLN    83       0.835 -11.356  12.977  1.00  0.00
ATOM   1321  HB2 GLN    83       0.725 -12.022  13.833  1.00  0.00
ATOM   1322  HB3 GLN    83       0.516 -10.352  13.256  1.00  0.00
ATOM   1323  CG  GLN    83      -0.031 -11.864  11.820  1.00  0.00
ATOM   1324  HG2 GLN    83      -1.084 -11.685  12.036  1.00  0.00
ATOM   1325  HG3 GLN    83       0.243 -11.351  10.898  1.00  0.00
ATOM   1326  CD  GLN    83       0.179 -13.363  11.625  1.00  0.00
ATOM   1327  OE1 GLN    83      -0.216 -14.161  12.474  1.00  0.00
ATOM   1328  NE2 GLN    83       0.780 -13.794  10.550  1.00  0.00
ATOM   1329 HE21 GLN    83       0.921 -14.786  10.417  1.00  0.00
ATOM   1330 HE22 GLN    83       1.102 -13.135   9.856  1.00  0.00
ATOM   1331  C   GLN    83       2.485 -10.307  11.416  1.00  0.00
ATOM   1332  O   GLN    83       2.958 -10.653  10.334  1.00  0.00
ATOM   1333  N   MET    84       2.093  -9.062  11.671  1.00  0.00
ATOM   1334  H   MET    84       1.651  -8.840  12.552  1.00  0.00
ATOM   1335  CA  MET    84       2.203  -8.008  10.665  1.00  0.00
ATOM   1336  HA  MET    84       1.709  -8.320   9.745  1.00  0.00
ATOM   1337  CB  MET    84       1.538  -6.726  11.166  1.00  0.00
ATOM   1338  HB2 MET    84       1.799  -6.564  12.212  1.00  0.00
ATOM   1339  HB3 MET    84       1.881  -5.880  10.571  1.00  0.00
ATOM   1340  CG  MET    84       0.014  -6.860  11.034  1.00  0.00
```

ATOM	1341	HG2	MET	84	-0.253	-7.026	9.991	1.00	0.00
ATOM	1342	HG3	MET	84	-0.336	-7.699	11.636	1.00	0.00
ATOM	1343	SD	MET	84	-0.776	-5.339	11.616	1.00	0.00
ATOM	1344	CE	MET	84	-0.296	-4.277	10.231	1.00	0.00
ATOM	1345	HE1	MET	84	-0.701	-4.683	9.304	1.00	0.00
ATOM	1346	HE2	MET	84	-0.688	-3.273	10.390	1.00	0.00
ATOM	1347	HE3	MET	84	0.791	-4.236	10.164	1.00	0.00
ATOM	1348	C	MET	84	3.652	-7.745	10.257	1.00	0.00
ATOM	1349	O	MET	84	3.910	-7.348	9.122	1.00	0.00
ATOM	1350	N	ARG	85	4.599	-7.960	11.171	1.00	0.00
ATOM	1351	H	ARG	85	4.371	-8.266	12.106	1.00	0.00
ATOM	1352	CA	ARG	85	6.006	-7.728	10.841	1.00	0.00
ATOM	1353	HA	ARG	85	6.181	-6.663	10.692	1.00	0.00
ATOM	1354	CB	ARG	85	6.909	-8.217	11.979	1.00	0.00
ATOM	1355	HB2	ARG	85	6.678	-7.660	12.887	1.00	0.00
ATOM	1356	HB3	ARG	85	6.730	-9.279	12.150	1.00	0.00
ATOM	1357	CG	ARG	85	8.380	-8.001	11.609	1.00	0.00
ATOM	1358	HG2	ARG	85	8.635	-8.619	10.748	1.00	0.00
ATOM	1359	HG3	ARG	85	8.545	-6.952	11.365	1.00	0.00
ATOM	1360	CD	ARG	85	9.263	-8.393	12.796	1.00	0.00
ATOM	1361	HD2	ARG	85	10.302	-8.138	12.587	1.00	0.00
ATOM	1362	HD3	ARG	85	8.934	-7.869	13.694	1.00	0.00

ATOM	1363	NE	ARG	85	9.174	-9.832	13.035	1.00	0.00
ATOM	1364	HE	ARG	85	9.187	-10.472	12.254	1.00	0.00
ATOM	1365	CZ	ARG	85	9.071	-10.334	14.266	1.00	0.00
ATOM	1366	NH1	ARG	85	9.060	-9.544	15.307	1.00	0.00
ATOM	1367	HH11	ARG	85	9.130	-8.545	15.180	1.00	0.00
ATOM	1368	HH12	ARG	85	8.982	-9.937	16.235	1.00	0.00
ATOM	1369	NH2	ARG	85	8.985	-11.625	14.433	1.00	0.00
ATOM	1370	HH21	ARG	85	8.997	-12.237	13.630	1.00	0.00
ATOM	1371	HH22	ARG	85	8.907	-12.007	15.365	1.00	0.00
ATOM	1372	C	ARG	85	6.362	-8.458	9.549	1.00	0.00
ATOM	1373	O	ARG	85	6.986	-7.887	8.655	1.00	0.00
ATOM	1374	N	ARG	86	5.935	-9.714	9.451	1.00	0.00
ATOM	1375	H	ARG	86	5.442	-10.149	10.218	1.00	0.00
ATOM	1376	CA	ARG	86	6.186	-10.506	8.253	1.00	0.00
ATOM	1377	HA	ARG	86	7.258	-10.575	8.067	1.00	0.00
ATOM	1378	CB	ARG	86	5.630	-11.920	8.440	1.00	0.00
ATOM	1379	HB2	ARG	86	4.566	-11.866	8.668	1.00	0.00
ATOM	1380	HB3	ARG	86	5.777	-12.493	7.525	1.00	0.00
ATOM	1381	CG	ARG	86	6.365	-12.607	9.595	1.00	0.00
ATOM	1382	HG2	ARG	86	7.434	-12.625	9.382	1.00	0.00
ATOM	1383	HG3	ARG	86	6.188	-12.052	10.516	1.00	0.00
ATOM	1384	CD	ARG	86	5.854	-14.039	9.758	1.00	0.00

```
ATOM   1385  HD2 ARG    86       4.766 -14.041   9.812  1.00  0.00
ATOM   1386  HD3 ARG    86       6.177 -14.647   8.913  1.00  0.00
ATOM   1387  NE  ARG    86       6.384 -14.626  10.985  1.00  0.00
ATOM   1388  HE  ARG    86       7.112 -14.150  11.499  1.00  0.00
ATOM   1389  CZ  ARG    86       5.928 -15.785  11.455  1.00  0.00
ATOM   1390  NH1 ARG    86       4.991 -16.432  10.815  1.00  0.00
ATOM   1391 HH11 ARG    86       4.612 -16.051   9.960  1.00  0.00
ATOM   1392 HH12 ARG    86       4.651 -17.311  11.178  1.00  0.00
ATOM   1393  NH2 ARG    86       6.421 -16.278  12.557  1.00  0.00
ATOM   1394 HH21 ARG    86       7.148 -15.778  13.050  1.00  0.00
ATOM   1395 HH22 ARG    86       6.075 -17.158  12.913  1.00  0.00
ATOM   1396  C   ARG    86       5.530  -9.843   7.043  1.00  0.00
ATOM   1397  O   ARG    86       6.084  -9.835   5.943  1.00  0.00
ATOM   1398  N   MET    87       4.338  -9.287   7.271  1.00  0.00
ATOM   1399  H   MET    87       3.930  -9.339   8.194  1.00  0.00
ATOM   1400  CA  MET    87       3.583  -8.614   6.216  1.00  0.00
ATOM   1401  HA  MET    87       3.307  -9.329   5.441  1.00  0.00
ATOM   1402  CB  MET    87       2.303  -8.002   6.796  1.00  0.00
ATOM   1403  HB2 MET    87       1.780  -8.754   7.387  1.00  0.00
ATOM   1404  HB3 MET    87       2.564  -7.157   7.433  1.00  0.00
ATOM   1405  CG  MET    87       1.395  -7.524   5.658  1.00  0.00
ATOM   1406  HG2 MET    87       2.003  -7.112   4.853  1.00  0.00
```

ATOM 1407 HG3 MET 87 0.811 -8.363 5.279 1.00 0.00
ATOM 1408 SD MET 87 0.270 -6.243 6.271 1.00 0.00
ATOM 1409 CE MET 87 -0.765 -7.298 7.314 1.00 0.00
ATOM 1410 HE1 MET 87 -0.148 -7.771 8.079 1.00 0.00
ATOM 1411 HE2 MET 87 -1.535 -6.693 7.793 1.00 0.00
ATOM 1412 HE3 MET 87 -1.235 -8.067 6.701 1.00 0.00
ATOM 1413 C MET 87 4.408 -7.509 5.561 1.00 0.00
ATOM 1414 O MET 87 4.219 -7.206 4.385 1.00 0.00
ATOM 1415 N MET 88 5.306 -6.893 6.330 1.00 0.00
ATOM 1416 H MET 88 5.429 -7.162 7.296 1.00 0.00
ATOM 1417 CA MET 88 6.126 -5.805 5.805 1.00 0.00
ATOM 1418 HA MET 88 5.520 -4.913 5.651 1.00 0.00
ATOM 1419 CB MET 88 7.232 -5.477 6.808 1.00 0.00
ATOM 1420 HB2 MET 88 7.784 -6.386 7.049 1.00 0.00
ATOM 1421 HB3 MET 88 7.912 -4.746 6.371 1.00 0.00
ATOM 1422 CG MET 88 6.615 -4.900 8.088 1.00 0.00
ATOM 1423 HG2 MET 88 6.315 -3.866 7.918 1.00 0.00
ATOM 1424 HG3 MET 88 5.743 -5.489 8.374 1.00 0.00
ATOM 1425 SD MET 88 7.834 -4.954 9.426 1.00 0.00
ATOM 1426 CE MET 88 9.263 -4.404 8.464 1.00 0.00
ATOM 1427 HE1 MET 88 9.068 -3.412 8.057 1.00 0.00
ATOM 1428 HE2 MET 88 10.142 -4.365 9.108 1.00 0.00

ATOM	1429	HE3	MET	88	9.443	-5.103	7.647	1.00	0.00
ATOM	1430	C	MET	88	6.752	-6.192	4.470	1.00	0.00
ATOM	1431	O	MET	88	6.613	-5.472	3.481	1.00	0.00
ATOM	1432	N	ARG	89	7.423	-7.339	4.441	1.00	0.00
ATOM	1433	H	ARG	89	7.534	-7.895	5.277	1.00	0.00
ATOM	1434	CA	ARG	89	8.043	-7.812	3.210	1.00	0.00
ATOM	1435	HA	ARG	89	8.767	-7.080	2.851	1.00	0.00
ATOM	1436	CB	ARG	89	8.771	-9.136	3.464	1.00	0.00
ATOM	1437	HB2	ARG	89	8.066	-9.867	3.860	1.00	0.00
ATOM	1438	HB3	ARG	89	9.189	-9.505	2.528	1.00	0.00
ATOM	1439	CG	ARG	89	9.902	-8.917	4.477	1.00	0.00
ATOM	1440	HG2	ARG	89	9.510	-8.395	5.350	1.00	0.00
ATOM	1441	HG3	ARG	89	10.306	-9.882	4.783	1.00	0.00
ATOM	1442	CD	ARG	89	11.013	-8.078	3.837	1.00	0.00
ATOM	1443	HD2	ARG	89	11.364	-8.563	2.926	1.00	0.00
ATOM	1444	HD3	ARG	89	10.634	-7.085	3.597	1.00	0.00
ATOM	1445	NE	ARG	89	12.138	-7.939	4.756	1.00	0.00
ATOM	1446	HE	ARG	89	12.948	-8.535	4.658	1.00	0.00
ATOM	1447	CZ	ARG	89	12.129	-7.035	5.734	1.00	0.00
ATOM	1448	NH1	ARG	89	11.092	-6.258	5.901	1.00	0.00
ATOM	1449	HH11	ARG	89	10.296	-6.345	5.286	1.00	0.00
ATOM	1450	HH12	ARG	89	11.092	-5.575	6.645	1.00	0.00

ATOM 1451 NH2 ARG 89 13.157 -6.927 6.528 1.00 0.00
ATOM 1452 HH21 ARG 89 13.957 -7.530 6.399 1.00 0.00
ATOM 1453 HH22 ARG 89 13.149 -6.241 7.270 1.00 0.00
ATOM 1454 C ARG 89 6.982 -7.999 2.128 1.00 0.00
ATOM 1455 O ARG 89 7.174 -7.605 0.977 1.00 0.00
ATOM 1456 N LEU 90 5.856 -8.594 2.519 1.00 0.00
ATOM 1457 H LEU 90 5.758 -8.910 3.473 1.00 0.00
ATOM 1458 CA LEU 90 4.752 -8.827 1.596 1.00 0.00
ATOM 1459 HA LEU 90 5.086 -9.428 0.750 1.00 0.00
ATOM 1460 CB LEU 90 3.633 -9.583 2.322 1.00 0.00
ATOM 1461 HB2 LEU 90 4.050 -10.460 2.818 1.00 0.00
ATOM 1462 HB3 LEU 90 3.188 -8.923 3.067 1.00 0.00
ATOM 1463 CG LEU 90 2.557 -10.024 1.321 1.00 0.00
ATOM 1464 HG LEU 90 3.015 -10.198 0.348 1.00 0.00
ATOM 1465 CD1 LEU 90 1.901 -11.317 1.814 1.00 0.00
ATOM 1466 HD11 LEU 90 1.443 -11.144 2.788 1.00 0.00
ATOM 1467 HD12 LEU 90 1.137 -11.631 1.104 1.00 0.00
ATOM 1468 HD13 LEU 90 2.657 -12.098 1.903 1.00 0.00
ATOM 1469 CD2 LEU 90 1.487 -8.932 1.202 1.00 0.00
ATOM 1470 HD21 LEU 90 1.948 -8.008 0.855 1.00 0.00
ATOM 1471 HD22 LEU 90 0.724 -9.247 0.491 1.00 0.00
ATOM 1472 HD23 LEU 90 1.028 -8.765 2.177 1.00 0.00

ATOM	1473	C	LEU	90	4.217	-7.504	1.058	1.00	0.00
ATOM	1474	O	LEU	90	3.892	-7.388	-0.124	1.00	0.00
ATOM	1475	N	ALA	91	4.122	-6.513	1.941	1.00	0.00
ATOM	1476	H	ALA	91	4.383	-6.663	2.906	1.00	0.00
ATOM	1477	CA	ALA	91	3.617	-5.201	1.557	1.00	0.00
ATOM	1478	HA	ALA	91	2.577	-5.276	1.240	1.00	0.00
ATOM	1479	CB	ALA	91	3.698	-4.241	2.747	1.00	0.00
ATOM	1480	HB1	ALA	91	4.735	-4.149	3.071	1.00	0.00
ATOM	1481	HB2	ALA	91	3.323	-3.262	2.451	1.00	0.00
ATOM	1482	HB3	ALA	91	3.095	-4.628	3.569	1.00	0.00
ATOM	1483	C	ALA	91	4.419	-4.642	0.390	1.00	0.00
ATOM	1484	O	ALA	91	3.852	-4.239	-0.619	1.00	0.00
ATOM	1485	N	GLU	92	5.741	-4.630	0.538	1.00	0.00
ATOM	1486	H	GLU	92	6.154	-4.951	1.402	1.00	0.00
ATOM	1487	CA	GLU	92	6.629	-4.130	-0.511	1.00	0.00
ATOM	1488	HA	GLU	92	6.394	-3.091	-0.740	1.00	0.00
ATOM	1489	CB	GLU	92	8.083	-4.209	-0.037	1.00	0.00
ATOM	1490	HB2	GLU	92	8.176	-3.707	0.926	1.00	0.00
ATOM	1491	HB3	GLU	92	8.372	-5.255	0.070	1.00	0.00
ATOM	1492	CG	GLU	92	9.002	-3.527	-1.060	1.00	0.00
ATOM	1493	HG2	GLU	92	8.850	-3.990	-2.035	1.00	0.00
ATOM	1494	HG3	GLU	92	8.743	-2.470	-1.115	1.00	0.00

ATOM	1495	CD	GLU	92	10.469	-3.671	-0.651	1.00	0.00
ATOM	1496	OE1	GLU	92	10.732	-4.307	0.360	1.00	0.00
ATOM	1497	OE2	GLU	92	11.311	-3.143	-1.357	1.00	0.00
ATOM	1498	C	GLU	92	6.461	-4.932	-1.801	1.00	0.00
ATOM	1499	O	GLU	92	6.654	-4.405	-2.896	1.00	0.00
ATOM	1500	N	ASN	93	6.117	-6.210	-1.662	1.00	0.00
ATOM	1501	H	ASN	93	5.992	-6.609	-0.742	1.00	0.00
ATOM	1502	CA	ASN	93	5.945	-7.081	-2.825	1.00	0.00
ATOM	1503	HA	ASN	93	6.807	-6.995	-3.487	1.00	0.00
ATOM	1504	CB	ASN	93	5.818	-8.540	-2.373	1.00	0.00
ATOM	1505	HB2	ASN	93	4.996	-8.638	-1.664	1.00	0.00
ATOM	1506	HB3	ASN	93	5.633	-9.182	-3.234	1.00	0.00
ATOM	1507	CG	ASN	93	7.107	-8.998	-1.691	1.00	0.00
ATOM	1508	OD1	ASN	93	8.122	-8.182	-1.603	1.00	0.00
ATOM	1509	ND2	ASN	93	7.189	-10.135	-1.226	1.00	0.00
ATOM	1510	HD21	ASN	93	8.043	-10.433	-0.776	1.00	0.00
ATOM	1511	HD22	ASN	93	6.402	-10.764	-1.296	1.00	0.00
ATOM	1512	C	ASN	93	4.710	-6.691	-3.638	1.00	0.00
ATOM	1513	O	ASN	93	4.711	-6.804	-4.864	1.00	0.00
ATOM	1514	N	ILE	94	3.658	-6.239	-2.955	1.00	0.00
ATOM	1515	H	ILE	94	3.680	-6.197	-1.946	1.00	0.00
ATOM	1516	CA	ILE	94	2.426	-5.847	-3.639	1.00	0.00

ATOM	1517	HA	ILE	94	1.977	-6.726	-4.102	1.00	0.00
ATOM	1518	CB	ILE	94	1.438	-5.237	-2.632	1.00	0.00
ATOM	1519	HB	ILE	94	1.986	-4.609	-1.929	1.00	0.00
ATOM	1520	CG2	ILE	94	0.390	-4.390	-3.365	1.00	0.00
ATOM	1521	HG21	ILE	94	-0.158	-5.018	-4.067	1.00	0.00
ATOM	1522	HG22	ILE	94	-0.304	-3.964	-2.641	1.00	0.00
ATOM	1523	HG23	ILE	94	0.887	-3.587	-3.908	1.00	0.00
ATOM	1524	CG1	ILE	94	0.734	-6.368	-1.870	1.00	0.00
ATOM	1525	HG12	ILE	94	-0.054	-6.789	-2.494	1.00	0.00
ATOM	1526	HG13	ILE	94	1.459	-7.146	-1.626	1.00	0.00
ATOM	1527	CD1	ILE	94	0.122	-5.818	-0.578	1.00	0.00
ATOM	1528	HD11	ILE	94	-0.602	-5.041	-0.820	1.00	0.00
ATOM	1529	HD12	ILE	94	-0.377	-6.624	-0.040	1.00	0.00
ATOM	1530	HD13	ILE	94	0.910	-5.398	0.048	1.00	0.00
ATOM	1531	C	ILE	94	2.722	-4.856	-4.773	1.00	0.00
ATOM	1532	O	ILE	94	2.369	-5.106	-5.925	1.00	0.00
ATOM	1533	N	PRO	95	3.362	-3.751	-4.477	1.00	0.00
ATOM	1534	CD	PRO	95	3.818	-3.355	-3.136	1.00	0.00
ATOM	1535	HD2	PRO	95	4.389	-4.174	-2.698	1.00	0.00
ATOM	1536	HD3	PRO	95	2.949	-3.145	-2.512	1.00	0.00
ATOM	1537	CG	PRO	95	4.670	-2.114	-3.384	1.00	0.00
ATOM	1538	HG2	PRO	95	5.720	-2.403	-3.445	1.00	0.00

ATOM 1539 HG3 PRO 95 4.535 -1.412 -2.561 1.00 0.00

ATOM 1540 CB PRO 95 4.191 -1.529 -4.666 1.00 0.00

ATOM 1541 HB2 PRO 95 5.021 -1.035 -5.170 1.00 0.00

ATOM 1542 HB3 PRO 95 3.376 -0.820 -4.527 1.00 0.00

ATOM 1543 CA PRO 95 3.711 -2.720 -5.499 1.00 0.00

ATOM 1544 HA PRO 95 2.808 -2.492 -6.065 1.00 0.00

ATOM 1545 C PRO 95 4.809 -3.184 -6.458 1.00 0.00

ATOM 1546 O PRO 95 4.904 -2.689 -7.577 1.00 0.00

ATOM 1547 N SER 96 5.628 -4.144 -6.018 1.00 0.00

ATOM 1548 H SER 96 5.522 -4.518 -5.086 1.00 0.00

ATOM 1549 CA SER 96 6.703 -4.670 -6.858 1.00 0.00

ATOM 1550 HA SER 96 7.364 -3.858 -7.161 1.00 0.00

ATOM 1551 CB SER 96 7.525 -5.710 -6.094 1.00 0.00

ATOM 1552 HB2 SER 96 6.884 -6.545 -5.810 1.00 0.00

ATOM 1553 HB3 SER 96 8.334 -6.073 -6.728 1.00 0.00

ATOM 1554 OG SER 96 8.070 -5.114 -4.926 1.00 0.00

ATOM 1555 HG SER 96 8.586 -5.766 -4.445 1.00 0.00

ATOM 1556 C SER 96 6.090 -5.300 -8.081 1.00 0.00

ATOM 1557 O SER 96 6.566 -5.150 -9.207 1.00 0.00

ATOM 1558 N ARG 97 5.009 -5.999 -7.812 1.00 0.00

ATOM 1559 H ARG 97 4.708 -6.078 -6.851 1.00 0.00

ATOM 1560 CA ARG 97 4.247 -6.682 -8.824 1.00 0.00

ATOM	1561	HA	ARG	97	4.839	-7.463	-9.302	1.00	0.00
ATOM	1562	CB	ARG	97	3.048	-7.329	-8.143	1.00	0.00
ATOM	1563	HB2	ARG	97	2.589	-6.613	-7.462	1.00	0.00
ATOM	1564	HB3	ARG	97	2.322	-7.628	-8.898	1.00	0.00
ATOM	1565	CG	ARG	97	3.503	-8.563	-7.355	1.00	0.00
ATOM	1566	HG2	ARG	97	4.042	-9.238	-8.020	1.00	0.00
ATOM	1567	HG3	ARG	97	4.162	-8.251	-6.544	1.00	0.00
ATOM	1568	CD	ARG	97	2.286	-9.286	-6.773	1.00	0.00
ATOM	1569	HD2	ARG	97	1.647	-9.643	-7.581	1.00	0.00
ATOM	1570	HD3	ARG	97	2.613	-10.132	-6.168	1.00	0.00
ATOM	1571	NE	ARG	97	1.511	-8.379	-5.929	1.00	0.00
ATOM	1572	HE	ARG	97	1.778	-8.239	-4.965	1.00	0.00
ATOM	1573	CZ	ARG	97	0.450	-7.723	-6.397	1.00	0.00
ATOM	1574	NH1	ARG	97	0.064	-7.884	-7.635	1.00	0.00
ATOM	1575	HH11	ARG	97	0.574	-8.510	-8.242	1.00	0.00
ATOM	1576	HH12	ARG	97	-0.741	-7.382	-7.979	1.00	0.00
ATOM	1577	NH2	ARG	97	-0.207	-6.914	-5.614	1.00	0.00
ATOM	1578	HH21	ARG	97	0.092	-6.789	-4.657	1.00	0.00
ATOM	1579	HH22	ARG	97	-1.011	-6.416	-5.967	1.00	0.00
ATOM	1580	C	ARG	97	3.782	-5.725	-9.921	1.00	0.00
ATOM	1581	O	ARG	97	3.726	-6.099	-11.093	1.00	0.00
ATOM	1582	N	CYX	98	3.459	-4.490	-9.538	1.00	0.00

ATOM	1583	H	CYX	98	3.523	-4.222	-8.566	1.00	0.00
ATOM	1584	CA	CYX	98	3.012	-3.495	-10.500	1.00	0.00
ATOM	1585	HA	CYX	98	2.697	-3.993	-11.417	1.00	0.00
ATOM	1586	CB	CYX	98	1.836	-2.688	-9.953	1.00	0.00
ATOM	1587	HB2	CYX	98	2.202	-1.751	-9.533	1.00	0.00
ATOM	1588	HB3	CYX	98	1.135	-2.475	-10.759	1.00	0.00
ATOM	1589	SG	CYX	98	1.007	-3.622	-8.640	1.00	0.00
ATOM	1590	C	CYX	98	4.132	-2.536	-10.814	1.00	0.00
ATOM	1591	O	CYX	98	4.015	-1.720	-11.728	1.00	0.00
ATOM	1592	N	ASN	99	5.205	-2.602	-10.032	1.00	0.00
ATOM	1593	H	ASN	99	5.254	-3.233	-9.244	1.00	0.00
ATOM	1594	CA	ASN	99	6.300	-1.685	-10.243	1.00	0.00
ATOM	1595	HA	ASN	99	5.919	-0.671	-10.123	1.00	0.00
ATOM	1596	CB	ASN	99	7.457	-1.880	-9.294	1.00	0.00
ATOM	1597	HB2	ASN	99	7.592	-2.937	-9.065	1.00	0.00
ATOM	1598	HB3	ASN	99	8.372	-1.485	-9.736	1.00	0.00
ATOM	1599	CG	ASN	99	7.181	-1.132	-7.995	1.00	0.00
ATOM	1600	OD1	ASN	99	7.477	-1.629	-6.909	1.00	0.00
ATOM	1601	ND2	ASN	99	6.631	0.052	-8.050	1.00	0.00
ATOM	1602	HD21	ASN	99	6.443	0.558	-7.196	1.00	0.00
ATOM	1603	HD22	ASN	99	6.397	0.455	-8.945	1.00	0.00
ATOM	1604	C	ASN	99	6.796	-1.746	-11.654	1.00	0.00

ATOM 1605 O ASN 99 6.624 -2.732 -12.374 1.00 0.00
ATOM 1606 N LEU 100 7.325 -0.621 -12.049 1.00 0.00
ATOM 1607 H LEU 100 7.427 0.138 -11.390 1.00 0.00
ATOM 1608 CA LEU 100 7.772 -0.416 -13.401 1.00 0.00
ATOM 1609 HA LEU 100 8.100 -1.346 -13.865 1.00 0.00
ATOM 1610 CB LEU 100 6.562 0.119 -14.176 1.00 0.00
ATOM 1611 HB2 LEU 100 6.878 0.989 -14.751 1.00 0.00
ATOM 1612 HB3 LEU 100 6.163 -0.638 -14.851 1.00 0.00
ATOM 1613 CG LEU 100 5.462 0.540 -13.163 1.00 0.00
ATOM 1614 HG LEU 100 5.463 -0.153 -12.321 1.00 0.00
ATOM 1615 CD1 LEU 100 5.737 1.956 -12.657 1.00 0.00
ATOM 1616 HD11 LEU 100 5.736 2.649 -13.498 1.00 0.00
ATOM 1617 HD12 LEU 100 4.963 2.245 -11.947 1.00 0.00
ATOM 1618 HD13 LEU 100 6.709 1.984 -12.164 1.00 0.00
ATOM 1619 CD2 LEU 100 4.091 0.473 -13.819 1.00 0.00
ATOM 1620 HD21 LEU 100 3.897 -0.547 -14.153 1.00 0.00
ATOM 1621 HD22 LEU 100 3.329 0.770 -13.099 1.00 0.00
ATOM 1622 HD23 LEU 100 4.064 1.147 -14.675 1.00 0.00
ATOM 1623 C LEU 100 8.921 0.587 -13.431 1.00 0.00
ATOM 1624 O LEU 100 8.895 1.581 -12.708 1.00 0.00
ATOM 1625 N SER 101 9.925 0.306 -14.265 1.00 0.00
ATOM 1626 H SER 101 9.891 -0.538 -14.819 1.00 0.00

ATOM	1627	CA	SER	101	11.101	1.172	-14.390	1.00	0.00
ATOM	1628	HA	SER	101	11.838	0.919	-13.627	1.00	0.00
ATOM	1629	CB	SER	101	11.738	0.980	-15.768	1.00	0.00
ATOM	1630	HB2	SER	101	12.025	-0.064	-15.895	1.00	0.00
ATOM	1631	HB3	SER	101	11.023	1.257	-16.542	1.00	0.00
ATOM	1632	OG	SER	101	12.892	1.804	-15.872	1.00	0.00
ATOM	1633	HG	SER	101	13.294	1.685	-16.736	1.00	0.00
ATOM	1634	C	SER	101	10.724	2.641	-14.202	1.00	0.00
ATOM	1635	O	SER	101	10.275	3.305	-15.137	1.00	0.00
ATOM	1636	N	PRO	102	10.895	3.150	-13.007	1.00	0.00
ATOM	1637	CD	PRO	102	11.409	2.444	-11.837	1.00	0.00
ATOM	1638	HD2	PRO	102	12.484	2.602	-11.743	1.00	0.00
ATOM	1639	HD3	PRO	102	11.206	1.376	-11.922	1.00	0.00
ATOM	1640	CG	PRO	102	10.622	3.096	-10.712	1.00	0.00
ATOM	1641	HG2	PRO	102	11.205	3.048	-9.792	1.00	0.00
ATOM	1642	HG3	PRO	102	9.682	2.561	-10.574	1.00	0.00
ATOM	1643	CB	PRO	102	10.374	4.518	-11.132	1.00	0.00
ATOM	1644	HB2	PRO	102	11.117	5.150	-10.645	1.00	0.00
ATOM	1645	HB3	PRO	102	9.375	4.870	-10.877	1.00	0.00
ATOM	1646	CA	PRO	102	10.572	4.558	-12.661	1.00	0.00
ATOM	1647	HA	PRO	102	9.661	4.840	-13.188	1.00	0.00
ATOM	1648	C	PRO	102	11.699	5.510	-13.049	1.00	0.00

ATOM	1649	O	PRO	102	12.817	5.398	-12.542	1.00	0.00
ATOM	1650	N	MET	103	11.403	6.442	-13.951	1.00	0.00
ATOM	1651	H	MET	103	10.480	6.493	-14.358	1.00	0.00
ATOM	1652	CA	MET	103	12.408	7.399	-14.395	1.00	0.00
ATOM	1653	HA	MET	103	13.313	6.875	-14.703	1.00	0.00
ATOM	1654	CB	MET	103	11.875	8.200	-15.588	1.00	0.00
ATOM	1655	HB2	MET	103	10.949	8.698	-15.303	1.00	0.00
ATOM	1656	HB3	MET	103	12.614	8.946	-15.880	1.00	0.00
ATOM	1657	CG	MET	103	11.606	7.258	-16.767	1.00	0.00
ATOM	1658	HG2	MET	103	10.819	6.553	-16.499	1.00	0.00
ATOM	1659	HG3	MET	103	11.293	7.838	-17.634	1.00	0.00
ATOM	1660	SD	MET	103	13.113	6.341	-17.177	1.00	0.00
ATOM	1661	CE	MET	103	14.129	7.758	-17.664	1.00	0.00
ATOM	1662	HE1	MET	103	14.222	8.445	-16.823	1.00	0.00
ATOM	1663	HE2	MET	103	15.119	7.411	-17.960	1.00	0.00
ATOM	1664	HE3	MET	103	13.659	8.271	-18.502	1.00	0.00
ATOM	1665	C	MET	103	12.776	8.352	-13.263	1.00	0.00
ATOM	1666	O	MET	103	13.942	8.444	-12.874	1.00	0.00
ATOM	1667	N	ARG	104	11.775	9.052	-12.732	1.00	0.00
ATOM	1668	H	ARG	104	10.836	8.959	-13.091	1.00	0.00
ATOM	1669	CA	ARG	104	12.007	9.987	-11.635	1.00	0.00
ATOM	1670	HA	ARG	104	12.828	9.636	-11.010	1.00	0.00

ATOM 1671 CB ARG 104 12.374 11.367 -12.197 1.00 0.00
ATOM 1672 HB2 ARG 104 12.074 11.419 -13.243 1.00 0.00
ATOM 1673 HB3 ARG 104 11.850 12.136 -11.630 1.00 0.00
ATOM 1674 CG ARG 104 13.888 11.593 -12.087 1.00 0.00
ATOM 1675 HG2 ARG 104 14.410 10.777 -12.588 1.00 0.00
ATOM 1676 HG3 ARG 104 14.146 12.537 -12.566 1.00 0.00
ATOM 1677 CD ARG 104 14.307 11.638 -10.612 1.00 0.00
ATOM 1678 HD2 ARG 104 14.482 10.625 -10.249 1.00 0.00
ATOM 1679 HD3 ARG 104 15.221 12.223 -10.507 1.00 0.00
ATOM 1680 NE ARG 104 13.261 12.253 -9.798 1.00 0.00
ATOM 1681 HE ARG 104 12.659 11.673 -9.231 1.00 0.00
ATOM 1682 CZ ARG 104 13.073 13.571 -9.781 1.00 0.00
ATOM 1683 NH1 ARG 104 13.836 14.353 -10.497 1.00 0.00
ATOM 1684 HH11 ARG 104 14.571 13.955 -11.064 1.00 0.00
ATOM 1685 HH12 ARG 104 13.688 15.352 -10.479 1.00 0.00
ATOM 1686 NH2 ARG 104 12.126 14.082 -9.046 1.00 0.00
ATOM 1687 HH21 ARG 104 11.538 13.475 -8.492 1.00 0.00
ATOM 1688 HH22 ARG 104 11.985 15.082 -9.034 1.00 0.00
ATOM 1689 C ARG 104 10.769 10.116 -10.749 1.00 0.00
ATOM 1690 O ARG 104 9.644 10.173 -11.245 1.00 0.00
ATOM 1691 N CYX 105 10.994 10.193 -9.438 1.00 0.00
ATOM 1692 H CYX 105 11.936 10.125 -9.079 1.00 0.00

ATOM 1693 CA CYX 105 9.900 10.353 -8.484 1.00 0.00
ATOM 1694 HA CYX 105 8.947 10.181 -8.983 1.00 0.00
ATOM 1695 CB CYX 105 10.039 9.349 -7.337 1.00 0.00
ATOM 1696 HB2 CYX 105 11.075 9.325 -6.998 1.00 0.00
ATOM 1697 HB3 CYX 105 9.394 9.648 -6.511 1.00 0.00
ATOM 1698 SG CYX 105 9.580 7.695 -7.917 1.00 0.00
ATOM 1699 C CYX 105 9.906 11.777 -7.926 1.00 0.00
ATOM 1700 O CYX 105 10.815 12.149 -7.184 1.00 0.00
ATOM 1701 N PRO 106 8.923 12.577 -8.268 1.00 0.00
ATOM 1702 CD PRO 106 7.805 12.230 -9.157 1.00 0.00
ATOM 1703 HD2 PRO 106 7.113 11.577 -8.624 1.00 0.00
ATOM 1704 HD3 PRO 106 8.192 11.710 -10.033 1.00 0.00
ATOM 1705 CG PRO 106 7.184 13.583 -9.508 1.00 0.00
ATOM 1706 HG2 PRO 106 6.105 13.464 -9.604 1.00 0.00
ATOM 1707 HG3 PRO 106 7.597 13.928 -10.456 1.00 0.00
ATOM 1708 CB PRO 106 7.533 14.523 -8.401 1.00 0.00
ATOM 1709 HB2 PRO 106 6.739 14.526 -7.654 1.00 0.00
ATOM 1710 HB3 PRO 106 7.688 15.537 -8.770 1.00 0.00
ATOM 1711 CA PRO 106 8.826 13.990 -7.783 1.00 0.00
ATOM 1712 HA PRO 106 9.698 14.526 -8.157 1.00 0.00
ATOM 1713 C PRO 106 8.773 14.104 -6.265 1.00 0.00
ATOM 1714 O PRO 106 8.596 15.194 -5.724 1.00 0.00

ATOM	1715	N	MET	107	8.927	12.981	-5.587	1.00	0.00
ATOM	1716	H	MET	107	9.085	12.108	-6.070	1.00	0.00
ATOM	1717	CA	MET	107	8.895	12.968	-4.136	1.00	0.00
ATOM	1718	HA	MET	107	8.068	13.574	-3.766	1.00	0.00
ATOM	1719	CB	MET	107	8.703	11.550	-3.661	1.00	0.00
ATOM	1720	HB2	MET	107	9.309	10.884	-4.275	1.00	0.00
ATOM	1721	HB3	MET	107	9.024	11.476	-2.622	1.00	0.00
ATOM	1722	CG	MET	107	7.228	11.145	-3.770	1.00	0.00
ATOM	1723	HG2	MET	107	7.149	10.211	-4.327	1.00	0.00
ATOM	1724	HG3	MET	107	6.816	11.007	-2.770	1.00	0.00
ATOM	1725	SD	MET	107	6.287	12.434	-4.631	1.00	0.00
ATOM	1726	CE	MET	107	6.264	11.660	-6.265	1.00	0.00
ATOM	1727	HE1	MET	107	5.769	10.691	-6.202	1.00	0.00
ATOM	1728	HE2	MET	107	5.724	12.299	-6.963	1.00	0.00
ATOM	1729	HE3	MET	107	7.287	11.522	-6.616	1.00	0.00
ATOM	1730	C	MET	107	10.202	13.490	-3.585	1.00	0.00
ATOM	1731	O	MET	107	11.151	12.737	-3.358	1.00	0.00
ATOM	1732	N	GLY	108	10.236	14.786	-3.378	1.00	0.00
ATOM	1733	H	GLY	108	9.428	15.356	-3.583	1.00	0.00
ATOM	1734	CA	GLY	108	11.419	15.435	-2.855	1.00	0.00
ATOM	1735	HA2	GLY	108	11.155	16.431	-2.501	1.00	0.00
ATOM	1736	HA3	GLY	108	11.814	14.848	-2.025	1.00	0.00

ATOM	1737	C	GLY	108	12.493	15.559	-3.930	1.00	0.00
ATOM	1738	O	GLY	108	13.685	15.615	-3.626	1.00	0.00
ATOM	1739	N	GLY	109	12.056	15.608	-5.187	1.00	0.00
ATOM	1740	H	GLY	109	11.067	15.557	-5.384	1.00	0.00
ATOM	1741	CA	GLY	109	12.981	15.735	-6.310	1.00	0.00
ATOM	1742	HA2	GLY	109	12.426	15.626	-7.241	1.00	0.00
ATOM	1743	HA3	GLY	109	13.447	16.720	-6.277	1.00	0.00
ATOM	1744	C	GLY	109	14.072	14.668	-6.255	1.00	0.00
ATOM	1745	O	GLY	109	15.243	14.980	-6.034	1.00	0.00
ATOM	1746	N	SER	110	13.681	13.410	-6.459	1.00	0.00
ATOM	1747	H	SER	110	12.707	13.195	-6.615	1.00	0.00
ATOM	1748	CA	SER	110	14.639	12.305	-6.432	1.00	0.00
ATOM	1749	HA	SER	110	15.069	12.208	-5.435	1.00	0.00
ATOM	1750	CB	SER	110	13.939	10.995	-6.791	1.00	0.00
ATOM	1751	HB2	SER	110	13.575	11.045	-7.817	1.00	0.00
ATOM	1752	HB3	SER	110	14.642	10.167	-6.695	1.00	0.00
ATOM	1753	OG	SER	110	12.843	10.790	-5.908	1.00	0.00
ATOM	1754	HG	SER	110	12.402	9.967	-6.131	1.00	0.00
ATOM	1755	C	SER	110	15.782	12.566	-7.411	1.00	0.00
ATOM	1756	O	SER	110	15.625	13.317	-8.374	1.00	0.00
ATOM	1757	N	ILE	111	16.933	11.949	-7.156	1.00	0.00
ATOM	1758	H	ILE	111	17.031	11.360	-6.341	1.00	0.00

ATOM	1759	CA	ILE	111	18.096	12.133	-8.021	1.00	0.00
ATOM	1760	HA	ILE	111	18.006	13.082	-8.549	1.00	0.00
ATOM	1761	CB	ILE	111	19.378	12.137	-7.180	1.00	0.00
ATOM	1762	HB	ILE	111	19.474	11.183	-6.660	1.00	0.00
ATOM	1763	CG2	ILE	111	20.592	12.345	-8.093	1.00	0.00
ATOM	1764	HG21	ILE	111	20.498	13.298	-8.612	1.00	0.00
ATOM	1765	HG22	ILE	111	21.502	12.347	-7.493	1.00	0.00
ATOM	1766	HG23	ILE	111	20.641	11.537	-8.823	1.00	0.00
ATOM	1767	CG1	ILE	111	19.309	13.275	-6.153	1.00	0.00
ATOM	1768	HG12	ILE	111	19.338	14.233	-6.672	1.00	0.00
ATOM	1769	HG13	ILE	111	18.380	13.196	-5.588	1.00	0.00
ATOM	1770	CD1	ILE	111	20.499	13.178	-5.194	1.00	0.00
ATOM	1771	HD11	ILE	111	21.429	13.256	-5.758	1.00	0.00
ATOM	1772	HD12	ILE	111	20.447	13.987	-4.466	1.00	0.00
ATOM	1773	HD13	ILE	111	20.471	12.220	-4.674	1.00	0.00
ATOM	1774	C	ILE	111	18.165	11.028	-9.073	1.00	0.00
ATOM	1775	O	ILE	111	17.773	11.228	-10.224	1.00	0.00
ATOM	1776	N	ALA	112	18.640	9.855	-8.659	1.00	0.00
ATOM	1777	H	ALA	112	18.941	9.743	-7.701	1.00	0.00
ATOM	1778	CA	ALA	112	18.731	8.711	-9.547	1.00	0.00
ATOM	1779	HA	ALA	112	19.220	8.957	-10.489	1.00	0.00
ATOM	1780	CB	ALA	112	19.508	7.583	-8.875	1.00	0.00

ATOM 1781 HB1 ALA 112 19.025 7.319 -7.934 1.00 0.00
ATOM 1782 HB2 ALA 112 19.526 6.712 -9.530 1.00 0.00
ATOM 1783 HB3 ALA 112 20.529 7.910 -8.679 1.00 0.00
ATOM 1784 C ALA 112 17.340 8.235 -9.856 1.00 0.00
ATOM 1785 O ALA 112 16.364 8.913 -9.530 1.00 0.00
ATOM 1786 N GLY 113 17.226 7.051 -10.429 1.00 0.00
ATOM 1787 H GLY 113 18.015 6.482 -10.702 1.00 0.00
ATOM 1788 CA GLY 113 15.907 6.547 -10.675 1.00 0.00
ATOM 1789 HA2 GLY 113 15.253 7.332 -11.052 1.00 0.00
ATOM 1790 HA3 GLY 113 15.931 5.721 -11.386 1.00 0.00
ATOM 1791 C GLY 113 15.362 6.044 -9.354 1.00 0.00
ATOM 1792 O GLY 113 15.651 4.925 -8.928 1.00 0.00
ATOM 1793 N PHE 114 14.602 6.911 -8.702 1.00 0.00
ATOM 1794 H PHE 114 14.427 7.820 -9.105 1.00 0.00
ATOM 1795 CA PHE 114 14.025 6.611 -7.396 1.00 0.00
ATOM 1796 HA PHE 114 14.772 6.749 -6.614 1.00 0.00
ATOM 1797 CB PHE 114 12.850 7.552 -7.121 1.00 0.00
ATOM 1798 HB2 PHE 114 13.138 8.601 -7.191 1.00 0.00
ATOM 1799 HB3 PHE 114 12.009 7.361 -7.787 1.00 0.00
ATOM 1800 CG PHE 114 12.344 7.336 -5.712 1.00 0.00
ATOM 1801 CD1 PHE 114 12.952 8.004 -4.641 1.00 0.00
ATOM 1802 HD1 PHE 114 13.790 8.677 -4.822 1.00 0.00

```
ATOM   1803  CE1 PHE   114      12.483   7.806  -3.337  1.00  0.00
ATOM   1804  HE1 PHE   114      12.956   8.326  -2.504  1.00  0.00
ATOM   1805  CZ  PHE   114      11.409   6.941  -3.102  1.00  0.00
ATOM   1806  HZ  PHE   114      11.046   6.787  -2.086  1.00  0.00
ATOM   1807  CE2 PHE   114      10.801   6.273  -4.172  1.00  0.00
ATOM   1808  HE2 PHE   114       9.965   5.599  -3.989  1.00  0.00
ATOM   1809  CD2 PHE   114      11.268   6.471  -5.477  1.00  0.00
ATOM   1810  HD2 PHE   114      10.794   5.952  -6.310  1.00  0.00
ATOM   1811  C   PHE   114      13.545   5.160  -7.323  1.00  0.00
ATOM   1812  O   PHE   114      13.946   4.471  -6.400  1.00  0.00
ATOM   1813  OXT PHE   114      12.783   4.762  -8.190  1.00  0.00
TER
ATOM   1814  Cl1 LIG   115      19.563 -14.861  -5.275  1.00  0.00
ATOM   1815  Cl2 LIG   115      20.572 -17.559  -5.900  1.00  0.00
ATOM   1816  P1  LIG   115      18.426 -18.906  -2.054  1.00  0.00
ATOM   1817  O1  LIG   115      17.946 -20.373  -2.456  1.00  0.00
ATOM   1818  O2  LIG   115      19.741 -19.198  -1.220  1.00  0.00
ATOM   1819  O3  LIG   115      18.955 -18.356  -3.514  1.00  0.00
ATOM   1820  O4  LIG   115      17.473 -17.994  -1.407  1.00  0.00
ATOM   1821  C1  LIG   115      16.751 -20.588  -3.242  1.00  0.00
ATOM   1822  H1  LIG   115      16.588 -21.663  -3.249  1.00  0.00
ATOM   1823  H2  LIG   115      16.905 -20.227  -4.260  1.00  0.00
```

```
ATOM   1824  H3  LIG   115      15.901 -20.083  -2.782  1.00  0.00
ATOM   1825  C2  LIG   115      20.803 -20.069  -1.676  1.00  0.00
ATOM   1826  H4  LIG   115      21.183 -19.729  -2.640  1.00  0.00
ATOM   1827  H5  LIG   115      20.435 -21.093  -1.753  1.00  0.00
ATOM   1828  H6  LIG   115      21.582 -20.002  -0.921  1.00  0.00
ATOM   1829  C3  LIG   115      18.941 -17.019  -3.815  1.00  0.00
ATOM   1830  H7  LIG   115      18.346 -16.377  -3.178  1.00  0.00
ATOM   1831  C4  LIG   115      19.614 -16.550  -4.864  1.00  0.00
TER
ATOM   1832  Cl1 LIG   116     -18.105  16.421   2.805  1.00  0.00
ATOM   1833  Cl2 LIG   116     -17.645  17.084   5.640  1.00  0.00
ATOM   1834  P1  LIG   116     -19.445  21.204   4.639  1.00  0.00
ATOM   1835  O1  LIG   116     -20.209  21.554   5.995  1.00  0.00
ATOM   1836  O2  LIG   116     -18.095  22.020   4.791  1.00  0.00
ATOM   1837  O3  LIG   116     -19.035  19.635   4.926  1.00  0.00
ATOM   1838  O4  LIG   116     -20.140  21.392   3.357  1.00  0.00
ATOM   1839  C1  LIG   116     -21.518  21.012   6.286  1.00  0.00
ATOM   1840  H1  LIG   116     -21.854  21.515   7.190  1.00  0.00
ATOM   1841  H2  LIG   116     -21.445  19.938   6.461  1.00  0.00
ATOM   1842  H3  LIG   116     -22.204  21.221   5.464  1.00  0.00
ATOM   1843  C2  LIG   116     -17.262  21.977   5.973  1.00  0.00
ATOM   1844  H4  LIG   116     -16.965  20.950   6.190  1.00  0.00
```

```
ATOM   1845  H5  LIG   116     -17.799  22.404   6.821  1.00  0.00
ATOM   1846  H6  LIG   116     -16.388  22.579   5.738  1.00  0.00
ATOM   1847  C3  LIG   116     -18.870  18.745   3.896  1.00  0.00
ATOM   1848  H7  LIG   116     -19.260  19.034   2.929  1.00  0.00
ATOM   1849  C4  LIG   116     -18.276  17.571   4.100  1.00  0.00
TER
ATOM   1850  Cl1 LIG   117      -6.782 -10.137 -19.452  1.00  0.00
ATOM   1851  Cl2 LIG   117      -4.261  -9.069 -18.359  1.00  0.00
ATOM   1852  P1  LIG   117      -6.020 -10.039 -14.213  1.00  0.00
ATOM   1853  O1  LIG   117      -4.712 -10.465 -13.406  1.00  0.00
ATOM   1854  O2  LIG   117      -6.222  -8.534 -13.759  1.00  0.00
ATOM   1855  O3  LIG   117      -5.410  -9.950 -15.741  1.00  0.00
ATOM   1856  O4  LIG   117      -7.222 -10.879 -14.123  1.00  0.00
ATOM   1857  C1  LIG   117      -4.153 -11.794 -13.521  1.00  0.00
ATOM   1858  H1  LIG   117      -3.366 -11.851 -12.773  1.00  0.00
ATOM   1859  H2  LIG   117      -3.733 -11.936 -14.517  1.00  0.00
ATOM   1860  H3  LIG   117      -4.918 -12.544 -13.317  1.00  0.00
ATOM   1861  C2  LIG   117      -5.166  -7.545 -13.770  1.00  0.00
ATOM   1862  H4  LIG   117      -4.743  -7.454 -14.771  1.00  0.00
ATOM   1863  H5  LIG   117      -4.391  -7.822 -13.054  1.00  0.00
ATOM   1864  H6  LIG   117      -5.636  -6.610 -13.475  1.00  0.00
ATOM   1865  C3  LIG   117      -6.206 -10.164 -16.836  1.00  0.00
```

```
ATOM   1866  H7  LIG   117      -7.164 -10.637 -16.663  1.00  0.00
ATOM   1867  C4  LIG   117      -5.792  -9.825 -18.055  1.00  0.00
TER
ATOM   1868  Cl1 LIG   118      14.088  23.776  -0.253  1.00  0.00
ATOM   1869  Cl2 LIG   118      16.618  23.709   1.258  1.00  0.00
ATOM   1870  P1  LIG   118      16.041  28.246   1.805  1.00  0.00
ATOM   1871  O1  LIG   118      16.649  28.469   3.263  1.00  0.00
ATOM   1872  O2  LIG   118      17.242  28.683   0.868  1.00  0.00
ATOM   1873  O3  LIG   118      16.022  26.600   1.740  1.00  0.00
ATOM   1874  O4  LIG   118      14.744  28.850   1.470  1.00  0.00
ATOM   1875  C1  LIG   118      15.877  28.190   4.453  1.00  0.00
ATOM   1876  H1  LIG   118      16.472  28.556   5.287  1.00  0.00
ATOM   1877  H2  LIG   118      15.718  27.115   4.551  1.00  0.00
ATOM   1878  H3  LIG   118      14.922  28.716   4.416  1.00  0.00
ATOM   1879  C2  LIG   118      18.599  28.206   1.029  1.00  0.00
ATOM   1880  H4  LIG   118      18.624  27.116   0.983  1.00  0.00
ATOM   1881  H5  LIG   118      19.005  28.556   1.978  1.00  0.00
ATOM   1882  H6  LIG   118      19.159  28.629   0.198  1.00  0.00
ATOM   1883  C3  LIG   118      15.110  25.932   0.964  1.00  0.00
ATOM   1884  H7  LIG   118      14.260  26.498   0.606  1.00  0.00
ATOM   1885  C4  LIG   118      15.266  24.637   0.694  1.00  0.00
TER
```

```
ATOM   1886  Cl1 LIG   119      21.170 -11.366  21.605  1.00  0.00
ATOM   1887  Cl2 LIG   119      19.775  -8.861  20.923  1.00  0.00
ATOM   1888  P1  LIG   119      23.970  -7.075  20.272  1.00  0.00
ATOM   1889  O1  LIG   119      23.701  -6.066  19.066  1.00  0.00
ATOM   1890  O2  LIG   119      23.940  -6.112  21.530  1.00  0.00
ATOM   1891  O3  LIG   119      22.533  -7.879  20.312  1.00  0.00
ATOM   1892  O4  LIG   119      25.131  -7.973  20.208  1.00  0.00
ATOM   1893  C1  LIG   119      23.644  -6.525  17.696  1.00  0.00
ATOM   1894  H1  LIG   119      23.590  -5.628  17.083  1.00  0.00
ATOM   1895  H2  LIG   119      22.753  -7.136  17.545  1.00  0.00
ATOM   1896  H3  LIG   119      24.543  -7.092  17.450  1.00  0.00
ATOM   1897  C2  LIG   119      22.888  -5.150  21.779  1.00  0.00
ATOM   1898  H4  LIG   119      21.920  -5.651  21.820  1.00  0.00
ATOM   1899  H5  LIG   119      22.889  -4.388  20.999  1.00  0.00
ATOM   1900  H6  LIG   119      23.120  -4.705  22.744  1.00  0.00
ATOM   1901  C3  LIG   119      22.454  -9.168  20.772  1.00  0.00
ATOM   1902  H7  LIG   119      23.384  -9.715  20.864  1.00  0.00
ATOM   1903  C4  LIG   119      21.277  -9.716  21.064  1.00  0.00
TER
ATOM   1904  Cl1 LIG   120     -24.886  18.693 -23.580  1.00  0.00
ATOM   1905  Cl2 LIG   120     -23.261  16.772 -22.045  1.00  0.00
ATOM   1906  P1  LIG   120     -20.400  20.298 -21.271  1.00  0.00
```

```
ATOM   1907  O1  LIG   120     -19.887  19.972 -19.796  1.00  0.00
ATOM   1908  O2  LIG   120     -19.213  19.753 -22.169  1.00  0.00
ATOM   1909  O3  LIG   120     -21.602  19.181 -21.420  1.00  0.00
ATOM   1910  O4  LIG   120     -20.840  21.663 -21.590  1.00  0.00
ATOM   1911  C1  LIG   120     -20.675  20.308 -18.631  1.00  0.00
ATOM   1912  H1  LIG   120     -20.038  20.106 -17.772  1.00  0.00
ATOM   1913  H2  LIG   120     -21.567  19.680 -18.591  1.00  0.00
ATOM   1914  H3  LIG   120     -20.950  21.363 -18.653  1.00  0.00
ATOM   1915  C2  LIG   120     -18.632  18.437 -22.017  1.00  0.00
ATOM   1916  H4  LIG   120     -19.401  17.670 -22.118  1.00  0.00
ATOM   1917  H5  LIG   120     -18.142  18.358 -21.046  1.00  0.00
ATOM   1918  H6  LIG   120     -17.902  18.344 -22.817  1.00  0.00
ATOM   1919  C3  LIG   120     -22.677  19.403 -22.241  1.00  0.00
ATOM   1920  H7  LIG   120     -22.837  20.419 -22.580  1.00  0.00
ATOM   1921  C4  LIG   120     -23.495  18.406 -22.576  1.00  0.00
TER
ATOM   1922  Cl1 LIG   121      -5.603   2.492 -20.115  1.00  0.00
ATOM   1923  Cl2 LIG   121      -6.333   4.451 -22.193  1.00  0.00
ATOM   1924  P1  LIG   121      -3.933   7.500 -19.712  1.00  0.00
ATOM   1925  O1  LIG   121      -3.309   8.420 -20.856  1.00  0.00
ATOM   1926  O2  LIG   121      -5.182   8.352 -19.237  1.00  0.00
ATOM   1927  O3  LIG   121      -4.564   6.281 -20.622  1.00  0.00
```

ATOM 1928 O4 LIG 121 -3.065 7.019 -18.628 1.00 0.00

ATOM 1929 C1 LIG 121 -2.124 8.022 -21.582 1.00 0.00

ATOM 1930 H1 LIG 121 -1.845 8.879 -22.191 1.00 0.00

ATOM 1931 H2 LIG 121 -2.348 7.167 -22.222 1.00 0.00

ATOM 1932 H3 LIG 121 -1.319 7.779 -20.887 1.00 0.00

ATOM 1933 C2 LIG 121 -6.156 8.919 -20.144 1.00 0.00

ATOM 1934 H4 LIG 121 -6.602 8.136 -20.759 1.00 0.00

ATOM 1935 H5 LIG 121 -5.683 9.674 -20.773 1.00 0.00

ATOM 1936 H6 LIG 121 -6.914 9.376 -19.512 1.00 0.00

ATOM 1937 C3 LIG 121 -4.705 5.016 -20.112 1.00 0.00

ATOM 1938 H7 LIG 121 -4.163 4.788 -19.203 1.00 0.00

ATOM 1939 C4 LIG 121 -5.458 4.111 -20.735 1.00 0.00

TER

ATOM 1940 Cl1 LIG 122 -16.032 -26.748 -21.053 1.00 0.00

ATOM 1941 Cl2 LIG 122 -16.149 -28.538 -18.714 1.00 0.00

ATOM 1942 P1 LIG 122 -14.699 -24.914 -16.268 1.00 0.00

ATOM 1943 O1 LIG 122 -15.463 -25.139 -14.886 1.00 0.00

ATOM 1944 O2 LIG 122 -13.256 -25.489 -15.954 1.00 0.00

ATOM 1945 O3 LIG 122 -15.427 -26.062 -17.199 1.00 0.00

ATOM 1946 O4 LIG 122 -14.713 -23.580 -16.884 1.00 0.00

ATOM 1947 C1 LIG 122 -16.844 -24.749 -14.714 1.00 0.00

ATOM 1948 H1 LIG 122 -17.064 -24.885 -13.657 1.00 0.00

ATOM 1949 H2 LIG 122 -17.490 -25.391 -15.316 1.00 0.00

ATOM 1950 H3 LIG 122 -16.979 -23.703 -14.992 1.00 0.00

ATOM 1951 C2 LIG 122 -13.023 -26.781 -15.347 1.00 0.00

ATOM 1952 H4 LIG 122 -13.485 -27.568 -15.943 1.00 0.00

ATOM 1953 H5 LIG 122 -13.419 -26.791 -14.331 1.00 0.00

ATOM 1954 H6 LIG 122 -11.943 -26.908 -15.332 1.00 0.00

ATOM 1955 C3 LIG 122 -15.529 -25.913 -18.558 1.00 0.00

ATOM 1956 H7 LIG 122 -15.352 -24.923 -18.959 1.00 0.00

ATOM 1957 C4 LIG 122 -15.858 -26.944 -19.333 1.00 0.00

TER

ATOM 1958 Cl1 LIG 123 -6.139 19.241 23.395 1.00 0.00

ATOM 1959 Cl2 LIG 123 -7.658 19.156 20.871 1.00 0.00

ATOM 1960 P1 LIG 123 -3.626 19.948 18.789 1.00 0.00

ATOM 1961 O1 LIG 123 -3.793 19.109 17.443 1.00 0.00

ATOM 1962 O2 LIG 123 -3.968 21.421 18.313 1.00 0.00

ATOM 1963 O3 LIG 123 -4.956 19.444 19.621 1.00 0.00

ATOM 1964 O4 LIG 123 -2.382 19.821 19.561 1.00 0.00

ATOM 1965 C1 LIG 123 -3.571 17.680 17.412 1.00 0.00

ATOM 1966 H1 LIG 123 -3.617 17.395 16.363 1.00 0.00

ATOM 1967 H2 LIG 123 -4.355 17.169 17.973 1.00 0.00

ATOM 1968 H3 LIG 123 -2.589 17.442 17.822 1.00 0.00

ATOM 1969 C2 LIG 123 -5.143 21.761 17.540 1.00 0.00

```
ATOM   1970  H4  LIG   123      -6.046  21.447  18.067  1.00  0.00
ATOM   1971  H5  LIG   123      -5.091  21.287  16.559  1.00  0.00
ATOM   1972  H6  LIG   123      -5.125  22.843  17.439  1.00  0.00
ATOM   1973  C3  LIG   123      -4.973  19.435  20.991  1.00  0.00
ATOM   1974  H7  LIG   123      -4.019  19.522  21.496  1.00  0.00
ATOM   1975  C4  LIG   123      -6.118  19.299  21.657  1.00  0.00
TER
ATOM   1976  Cl1 LIG   124     -17.741  -3.026  -4.047  1.00  0.00
ATOM   1977  Cl2 LIG   124     -18.163  -4.636  -1.615  1.00  0.00
ATOM   1978  P1  LIG   124     -18.473  -0.614   0.609  1.00  0.00
ATOM   1979  O1  LIG   124     -19.559  -0.953   1.728  1.00  0.00
ATOM   1980  O2  LIG   124     -17.111  -0.757   1.407  1.00  0.00
ATOM   1981  O3  LIG   124     -18.572  -1.970  -0.321  1.00  0.00
ATOM   1982  O4  LIG   124     -18.609   0.627  -0.167  1.00  0.00
ATOM   1983  C1  LIG   124     -20.975  -0.942   1.433  1.00  0.00
ATOM   1984  H1  LIG   124     -21.478  -1.068   2.389  1.00  0.00
ATOM   1985  H2  LIG   124     -21.223  -1.769   0.767  1.00  0.00
ATOM   1986  H3  LIG   124     -21.259   0.010   0.983  1.00  0.00
ATOM   1987  C2  LIG   124     -16.781  -1.898   2.232  1.00  0.00
ATOM   1988  H4  LIG   124     -16.829  -2.816   1.644  1.00  0.00
ATOM   1989  H5  LIG   124     -17.465  -1.954   3.079  1.00  0.00
ATOM   1990  H6  LIG   124     -15.765  -1.727   2.577  1.00  0.00
```

```
ATOM   1991  C3  LIG   124    -18.270  -1.937  -1.658  1.00  0.00
ATOM   1992  H7  LIG   124    -18.216  -0.962  -2.126  1.00  0.00
ATOM   1993  C4  LIG   124    -18.080  -3.064  -2.341  1.00  0.00
TER
END
```

Table F: The number of atoms present in the system of BE-Nap

```
ATOM      1  N   GLU     1       7.051  19.438  -6.219  1.00  0.00
ATOM      2  H1  GLU     1       7.361  20.053  -6.957  1.00  0.00
ATOM      3  H2  GLU     1       7.458  18.525  -6.365  1.00  0.00
ATOM      4  H3  GLU     1       7.171  19.908  -5.333  1.00  0.00
ATOM      5  CA  GLU     1       5.581  19.242  -6.365  1.00  0.00
ATOM      6  HA  GLU     1       5.352  18.185  -6.505  1.00  0.00
ATOM      7  CB  GLU     1       4.874  19.732  -5.098  1.00  0.00
ATOM      8  HB2 GLU     1       5.295  19.226  -4.229  1.00  0.00
ATOM      9  HB3 GLU     1       5.017  20.807  -4.996  1.00  0.00
ATOM     10  CG  GLU     1       3.376  19.423  -5.193  1.00  0.00
ATOM     11  HG2 GLU     1       2.979  19.867  -6.105  1.00  0.00
ATOM     12  HG3 GLU     1       3.232  18.343  -5.222  1.00  0.00
ATOM     13  CD  GLU     1       2.644  20.001  -3.986  1.00  0.00
ATOM     14  OE1 GLU     1       2.773  19.435  -2.912  1.00  0.00
ATOM     15  OE2 GLU     1       1.962  20.999  -4.153  1.00  0.00
```

```
ATOM     16  C   GLU     1       5.085  20.023  -7.580  1.00  0.00
ATOM     17  O   GLU     1       4.940  21.245  -7.527  1.00  0.00
ATOM     18  N   ALA     2       4.825  19.306  -8.671  1.00  0.00
ATOM     19  H   ALA     2       4.977  18.307  -8.673  1.00  0.00
ATOM     20  CA  ALA     2       4.341  19.936  -9.896  1.00  0.00
ATOM     21  HA  ALA     2       4.997  20.759 -10.179  1.00  0.00
ATOM     22  CB  ALA     2       4.317  18.916 -11.036  1.00  0.00
ATOM     23  HB1 ALA     2       3.657  18.090 -10.771  1.00  0.00
ATOM     24  HB2 ALA     2       3.953  19.394 -11.945  1.00  0.00
ATOM     25  HB3 ALA     2       5.325  18.535 -11.205  1.00  0.00
ATOM     26  C   ALA     2       2.940  20.504  -9.691  1.00  0.00
ATOM     27  O   ALA     2       2.147  19.961  -8.923  1.00  0.00
ATOM     28  N   GLU     3       2.645  21.600 -10.383  1.00  0.00
ATOM     29  H   GLU     3       3.338  22.027 -10.980  1.00  0.00
ATOM     30  CA  GLU     3       1.336  22.238 -10.275  1.00  0.00
ATOM     31  HA  GLU     3       1.125  22.481  -9.233  1.00  0.00
ATOM     32  CB  GLU     3       1.311  23.536 -11.097  1.00  0.00
ATOM     33  HB2 GLU     3       0.416  24.102 -10.839  1.00  0.00
ATOM     34  HB3 GLU     3       2.196  24.126 -10.856  1.00  0.00
ATOM     35  CG  GLU     3       1.301  23.219 -12.600  1.00  0.00
ATOM     36  HG2 GLU     3       0.420  22.621 -12.832  1.00  0.00
ATOM     37  HG3 GLU     3       1.262  24.154 -13.158  1.00  0.00
```

```
ATOM     38  CD  GLU     3       2.559  22.444 -12.992  1.00  0.00
ATOM     39  OE1 GLU     3       3.644  22.949 -12.754  1.00  0.00
ATOM     40  OE2 GLU     3       2.418  21.354 -13.524  1.00  0.00
ATOM     41  C   GLU     3       0.233  21.297 -10.758  1.00  0.00
ATOM     42  O   GLU     3      -0.925  21.428 -10.359  1.00  0.00
ATOM     43  N   ALA     4       0.603  20.353 -11.621  1.00  0.00
ATOM     44  H   ALA     4       1.559  20.305 -11.944  1.00  0.00
ATOM     45  CA  ALA     4      -0.359  19.394 -12.159  1.00  0.00
ATOM     46  HA  ALA     4      -1.256  19.923 -12.479  1.00  0.00
ATOM     47  CB  ALA     4       0.238  18.669 -13.367  1.00  0.00
ATOM     48  HB1 ALA     4       1.133  18.127 -13.060  1.00  0.00
ATOM     49  HB2 ALA     4      -0.493  17.966 -13.767  1.00  0.00
ATOM     50  HB3 ALA     4       0.500  19.396 -14.135  1.00  0.00
ATOM     51  C   ALA     4      -0.773  18.384 -11.100  1.00  0.00
ATOM     52  O   ALA     4      -1.625  17.528 -11.343  1.00  0.00
ATOM     53  N   GLN     5      -0.174  18.499  -9.924  1.00  0.00
ATOM     54  H   GLN     5       0.520  19.218  -9.777  1.00  0.00
ATOM     55  CA  GLN     5      -0.488  17.607  -8.818  1.00  0.00
ATOM     56  HA  GLN     5      -0.121  16.600  -9.021  1.00  0.00
ATOM     57  CB  GLN     5       0.191  18.128  -7.556  1.00  0.00
ATOM     58  HB2 GLN     5       1.267  18.182  -7.723  1.00  0.00
ATOM     59  HB3 GLN     5      -0.191  19.123  -7.327  1.00  0.00
```

ATOM 60 CG GLN 5 -0.096 17.188 -6.380 1.00 0.00

ATOM 61 HG2 GLN 5 -1.168 17.012 -6.295 1.00 0.00

ATOM 62 HG3 GLN 5 0.417 16.238 -6.533 1.00 0.00

ATOM 63 CD GLN 5 0.401 17.806 -5.077 1.00 0.00

ATOM 64 OE1 GLN 5 0.013 18.922 -4.730 1.00 0.00

ATOM 65 NE2 GLN 5 1.239 17.143 -4.329 1.00 0.00

ATOM 66 HE21 GLN 5 1.571 17.548 -3.465 1.00 0.00

ATOM 67 HE22 GLN 5 1.553 16.227 -4.617 1.00 0.00

ATOM 68 C GLN 5 -1.997 17.548 -8.593 1.00 0.00

ATOM 69 O GLN 5 -2.547 16.495 -8.271 1.00 0.00

ATOM 70 N GLU 6 -2.656 18.693 -8.760 1.00 0.00

ATOM 71 H GLU 6 -2.154 19.537 -8.995 1.00 0.00

ATOM 72 CA GLU 6 -4.100 18.776 -8.570 1.00 0.00

ATOM 73 HA GLU 6 -4.354 18.584 -7.527 1.00 0.00

ATOM 74 CB GLU 6 -4.590 20.180 -8.944 1.00 0.00

ATOM 75 HB2 GLU 6 -5.656 20.256 -8.733 1.00 0.00

ATOM 76 HB3 GLU 6 -4.049 20.919 -8.352 1.00 0.00

ATOM 77 CG GLU 6 -4.344 20.440 -10.435 1.00 0.00

ATOM 78 HG2 GLU 6 -3.310 20.192 -10.676 1.00 0.00

ATOM 79 HG3 GLU 6 -5.014 19.814 -11.024 1.00 0.00

ATOM 80 CD GLU 6 -4.604 21.909 -10.758 1.00 0.00

ATOM 81 OE1 GLU 6 -5.763 22.287 -10.814 1.00 0.00

```
ATOM     82  OE2 GLU     6      -3.640  22.633 -10.945  1.00  0.00
ATOM     83  C   GLU     6      -4.828  17.726  -9.409  1.00  0.00
ATOM     84  O   GLU     6      -5.744  17.064  -8.922  1.00  0.00
ATOM     85  N   GLU     7      -4.406  17.565 -10.664  1.00  0.00
ATOM     86  H   GLU     7      -3.658  18.136 -11.032  1.00  0.00
ATOM     87  CA  GLU     7      -5.018  16.577 -11.544  1.00  0.00
ATOM     88  HA  GLU     7      -6.068  16.822 -11.702  1.00  0.00
ATOM     89  CB  GLU     7      -4.308  16.561 -12.904  1.00  0.00
ATOM     90  HB2 GLU     7      -4.505  17.497 -13.425  1.00  0.00
ATOM     91  HB3 GLU     7      -3.234  16.450 -12.750  1.00  0.00
ATOM     92  CG  GLU     7      -4.827  15.387 -13.747  1.00  0.00
ATOM     93  HG2 GLU     7      -5.843  15.147 -13.432  1.00  0.00
ATOM     94  HG3 GLU     7      -4.830  15.672 -14.799  1.00  0.00
ATOM     95  CD  GLU     7      -3.933  14.164 -13.554  1.00  0.00
ATOM     96  OE1 GLU     7      -2.763  14.249 -13.890  1.00  0.00
ATOM     97  OE2 GLU     7      -4.432  13.158 -13.077  1.00  0.00
ATOM     98  C   GLU     7      -4.928  15.202 -10.908  1.00  0.00
ATOM     99  O   GLU     7      -5.868  14.409 -10.969  1.00  0.00
ATOM    100  N   CYX     8      -3.775  14.929 -10.306  1.00  0.00
ATOM    101  H   CYX     8      -3.025  15.606 -10.298  1.00  0.00
ATOM    102  CA  CYX     8      -3.549  13.648  -9.670  1.00  0.00
ATOM    103  HA  CYX     8      -3.806  12.840 -10.355  1.00  0.00
```

ATOM	104	CB	CYX	8	-2.074	13.509	-9.281	1.00	0.00
ATOM	105	HB2	CYX	8	-1.823	14.259	-8.531	1.00	0.00
ATOM	106	HB3	CYX	8	-1.897	12.514	-8.872	1.00	0.00
ATOM	107	SG	CYX	8	-1.037	13.748	-10.744	1.00	0.00
ATOM	108	C	CYX	8	-4.419	13.499	-8.429	1.00	0.00
ATOM	109	O	CYX	8	-5.206	12.559	-8.327	1.00	0.00
ATOM	110	N	ARG	9	-4.270	14.433	-7.493	1.00	0.00
ATOM	111	H	ARG	9	-3.595	15.174	-7.617	1.00	0.00
ATOM	112	CA	ARG	9	-5.043	14.400	-6.255	1.00	0.00
ATOM	113	HA	ARG	9	-4.777	13.519	-5.670	1.00	0.00
ATOM	114	CB	ARG	9	-4.742	15.649	-5.423	1.00	0.00
ATOM	115	HB2	ARG	9	-3.666	15.729	-5.267	1.00	0.00
ATOM	116	HB3	ARG	9	-5.098	16.531	-5.953	1.00	0.00
ATOM	117	CG	ARG	9	-5.448	15.549	-4.066	1.00	0.00
ATOM	118	HG2	ARG	9	-6.527	15.522	-4.219	1.00	0.00
ATOM	119	HG3	ARG	9	-5.131	14.638	-3.558	1.00	0.00
ATOM	120	CD	ARG	9	-5.085	16.763	-3.209	1.00	0.00
ATOM	121	HD2	ARG	9	-5.465	16.629	-2.196	1.00	0.00
ATOM	122	HD3	ARG	9	-4.002	16.884	-3.177	1.00	0.00
ATOM	123	NE	ARG	9	-5.675	17.974	-3.774	1.00	0.00
ATOM	124	HE	ARG	9	-6.349	17.907	-4.523	1.00	0.00
ATOM	125	CZ	ARG	9	-5.342	19.183	-3.324	1.00	0.00

```
ATOM    126  NH1 ARG     9      -4.476  19.308  -2.355  1.00  0.00
ATOM    127 HH11 ARG     9      -4.058  18.484  -1.946  1.00  0.00
ATOM    128 HH12 ARG     9      -4.228  20.227  -2.019  1.00  0.00
ATOM    129  NH2 ARG     9      -5.885  20.245  -3.851  1.00  0.00
ATOM    130 HH21 ARG     9      -6.556  20.146  -4.599  1.00  0.00
ATOM    131 HH22 ARG     9      -5.631  21.160  -3.508  1.00  0.00
ATOM    132  C   ARG     9      -6.538  14.323  -6.547  1.00  0.00
ATOM    133  O   ARG     9      -7.261  13.555  -5.915  1.00  0.00
ATOM    134  N   GLU    10      -6.996  15.121  -7.508  1.00  0.00
ATOM    135  H   GLU    10      -6.373  15.751  -7.993  1.00  0.00
ATOM    136  CA  GLU    10      -8.409  15.127  -7.873  1.00  0.00
ATOM    137  HA  GLU    10      -9.018  15.400  -7.011  1.00  0.00
ATOM    138  CB  GLU    10      -8.652  16.149  -8.988  1.00  0.00
ATOM    139  HB2 GLU    10      -8.392  17.144  -8.628  1.00  0.00
ATOM    140  HB3 GLU    10      -8.029  15.900  -9.847  1.00  0.00
ATOM    141  CG  GLU    10     -10.129  16.124  -9.402  1.00  0.00
ATOM    142  HG2 GLU    10     -10.736  15.830  -8.546  1.00  0.00
ATOM    143  HG3 GLU    10     -10.423  17.120  -9.731  1.00  0.00
ATOM    144  CD  GLU    10     -10.340  15.128 -10.542  1.00  0.00
ATOM    145  OE1 GLU    10      -9.690  15.276 -11.564  1.00  0.00
ATOM    146  OE2 GLU    10     -11.149  14.229 -10.375  1.00  0.00
ATOM    147  C   GLU    10      -8.846  13.743  -8.343  1.00  0.00
```

ATOM	148	O	GLU	10	-9.871	13.219	-7.903	1.00	0.00
ATOM	149	N	GLN	11	-8.059	13.164	-9.246	1.00	0.00
ATOM	150	H	GLN	11	-7.248	13.653	-9.596	1.00	0.00
ATOM	151	CA	GLN	11	-8.359	11.844	-9.790	1.00	0.00
ATOM	152	HA	GLN	11	-9.337	11.849	-10.271	1.00	0.00
ATOM	153	CB	GLN	11	-7.302	11.460	-10.832	1.00	0.00
ATOM	154	HB2	GLN	11	-7.160	12.287	-11.527	1.00	0.00
ATOM	155	HB3	GLN	11	-6.359	11.242	-10.329	1.00	0.00
ATOM	156	CG	GLN	11	-7.768	10.220	-11.603	1.00	0.00
ATOM	157	HG2	GLN	11	-7.844	9.369	-10.925	1.00	0.00
ATOM	158	HG3	GLN	11	-8.740	10.410	-12.057	1.00	0.00
ATOM	159	CD	GLN	11	-6.771	9.875	-12.709	1.00	0.00
ATOM	160	OE1	GLN	11	-5.837	10.635	-12.971	1.00	0.00
ATOM	161	NE2	GLN	11	-6.916	8.764	-13.378	1.00	0.00
ATOM	162	HE21	GLN	11	-6.261	8.529	-14.110	1.00	0.00
ATOM	163	HE22	GLN	11	-7.682	8.143	-13.160	1.00	0.00
ATOM	164	C	GLN	11	-8.418	10.784	-8.689	1.00	0.00
ATOM	165	O	GLN	11	-9.193	9.832	-8.781	1.00	0.00
ATOM	166	N	MET	12	-7.587	10.942	-7.657	1.00	0.00
ATOM	167	H	MET	12	-6.943	11.720	-7.631	1.00	0.00
ATOM	168	CA	MET	12	-7.551	9.974	-6.558	1.00	0.00
ATOM	169	HA	MET	12	-7.169	9.018	-6.918	1.00	0.00

ATOM	170	CB	MET	12	-6.637	10.474	-5.437	1.00	0.00
ATOM	171	HB2	MET	12	-6.924	11.487	-5.156	1.00	0.00
ATOM	172	HB3	MET	12	-6.728	9.818	-4.571	1.00	0.00
ATOM	173	CG	MET	12	-5.189	10.472	-5.927	1.00	0.00
ATOM	174	HG2	MET	12	-4.904	9.462	-6.223	1.00	0.00
ATOM	175	HG3	MET	12	-5.090	11.142	-6.781	1.00	0.00
ATOM	176	SD	MET	12	-4.096	11.033	-4.602	1.00	0.00
ATOM	177	CE	MET	12	-2.648	11.358	-5.636	1.00	0.00
ATOM	178	HE1	MET	12	-2.897	12.110	-6.384	1.00	0.00
ATOM	179	HE2	MET	12	-1.830	11.722	-5.013	1.00	0.00
ATOM	180	HE3	MET	12	-2.342	10.437	-6.134	1.00	0.00
ATOM	181	C	MET	12	-8.948	9.711	-6.001	1.00	0.00
ATOM	182	O	MET	12	-9.289	8.567	-5.702	1.00	0.00
ATOM	183	N	GLN	13	-9.758	10.762	-5.870	1.00	0.00
ATOM	184	H	GLN	13	-9.450	11.694	-6.106	1.00	0.00
ATOM	185	CA	GLN	13	-11.115	10.589	-5.357	1.00	0.00
ATOM	186	HA	GLN	13	-11.075	10.183	-4.346	1.00	0.00
ATOM	187	CB	GLN	13	-11.857	11.929	-5.318	1.00	0.00
ATOM	188	HB2	GLN	13	-11.689	12.459	-6.255	1.00	0.00
ATOM	189	HB3	GLN	13	-12.924	11.745	-5.191	1.00	0.00
ATOM	190	CG	GLN	13	-11.344	12.779	-4.151	1.00	0.00
ATOM	191	HG2	GLN	13	-11.974	13.660	-4.035	1.00	0.00

ATOM	192	HG3	GLN	13	-11.362	12.196	-3.230	1.00	0.00
ATOM	193	CD	GLN	13	-9.912	13.235	-4.411	1.00	0.00
ATOM	194	OE1	GLN	13	-8.962	12.561	-4.013	1.00	0.00
ATOM	195	NE2	GLN	13	-9.700	14.349	-5.056	1.00	0.00
ATOM	196	HE21	GLN	13	-8.753	14.655	-5.231	1.00	0.00
ATOM	197	HE22	GLN	13	-10.483	14.899	-5.377	1.00	0.00
ATOM	198	C	GLN	13	-11.875	9.614	-6.243	1.00	0.00
ATOM	199	O	GLN	13	-12.545	8.708	-5.752	1.00	0.00
ATOM	200	N	ARG	14	-11.729	9.789	-7.554	1.00	0.00
ATOM	201	H	ARG	14	-11.172	10.557	-7.901	1.00	0.00
ATOM	202	CA	ARG	14	-12.360	8.909	-8.515	1.00	0.00
ATOM	203	HA	ARG	14	-13.443	8.945	-8.396	1.00	0.00
ATOM	204	CB	ARG	14	-12.009	9.342	-9.943	1.00	0.00
ATOM	205	HB2	ARG	14	-12.182	10.413	-10.046	1.00	0.00
ATOM	206	HB3	ARG	14	-10.958	9.124	-10.136	1.00	0.00
ATOM	207	CG	ARG	14	-12.881	8.584	-10.950	1.00	0.00
ATOM	208	HG2	ARG	14	-12.408	8.612	-11.932	1.00	0.00
ATOM	209	HG3	ARG	14	-12.988	7.548	-10.628	1.00	0.00
ATOM	210	CD	ARG	14	-14.262	9.238	-11.030	1.00	0.00
ATOM	211	HD2	ARG	14	-14.918	8.642	-11.665	1.00	0.00
ATOM	212	HD3	ARG	14	-14.694	9.314	-10.032	1.00	0.00
ATOM	213	NE	ARG	14	-14.153	10.580	-11.597	1.00	0.00

```
ATOM    214  HE  ARG    14     -13.358  10.823 -12.170  1.00  0.00
ATOM    215  CZ  ARG    14     -15.085  11.506 -11.377  1.00  0.00
ATOM    216  NH1 ARG    14     -16.130  11.228 -10.645  1.00  0.00
ATOM    217 HH11 ARG    14     -16.231  10.306 -10.244  1.00  0.00
ATOM    218 HH12 ARG    14     -16.832  11.935 -10.483  1.00  0.00
ATOM    219  NH2 ARG    14     -14.953  12.697 -11.895  1.00  0.00
ATOM    220 HH21 ARG    14     -14.144  12.911 -12.460  1.00  0.00
ATOM    221 HH22 ARG    14     -15.660  13.398 -11.727  1.00  0.00
ATOM    222  C   ARG    14     -11.865   7.503  -8.259  1.00  0.00
ATOM    223  O   ARG    14     -12.635   6.542  -8.266  1.00  0.00
ATOM    224  N   GLN    15     -10.559   7.405  -8.009  1.00  0.00
ATOM    225  H   GLN    15      -9.965   8.221  -8.034  1.00  0.00
ATOM    226  CA  GLN    15      -9.954   6.129  -7.722  1.00  0.00
ATOM    227  HA  GLN    15     -10.337   5.372  -8.406  1.00  0.00
ATOM    228  CB  GLN    15      -8.431   6.216  -7.891  1.00  0.00
ATOM    229  HB2 GLN    15      -8.121   7.260  -7.844  1.00  0.00
ATOM    230  HB3 GLN    15      -7.946   5.657  -7.090  1.00  0.00
ATOM    231  CG  GLN    15      -8.026   5.624  -9.245  1.00  0.00
ATOM    232  HG2 GLN    15      -6.991   5.283  -9.204  1.00  0.00
ATOM    233  HG3 GLN    15      -8.674   4.785  -9.499  1.00  0.00
ATOM    234  CD  GLN    15      -8.148   6.682 -10.337  1.00  0.00
ATOM    235  OE1 GLN    15      -7.141   7.208 -10.808  1.00  0.00
```

ATOM 236 NE2 GLN 15 -9.331 7.031 -10.766 1.00 0.00
ATOM 237 HE21 GLN 15 -9.416 7.731 -11.489 1.00 0.00
ATOM 238 HE22 GLN 15 -10.155 6.599 -10.373 1.00 0.00
ATOM 239 C GLN 15 -10.306 5.723 -6.300 1.00 0.00
ATOM 240 O GLN 15 -9.484 5.807 -5.386 1.00 0.00
ATOM 241 N GLN 16 -11.537 5.277 -6.135 1.00 0.00
ATOM 242 H GLN 16 -12.175 5.255 -6.917 1.00 0.00
ATOM 243 CA GLN 16 -12.026 4.841 -4.833 1.00 0.00
ATOM 244 HA GLN 16 -11.615 5.473 -4.045 1.00 0.00
ATOM 245 CB GLN 16 -13.551 4.943 -4.795 1.00 0.00
ATOM 246 HB2 GLN 16 -13.983 4.323 -5.581 1.00 0.00
ATOM 247 HB3 GLN 16 -13.920 4.608 -3.825 1.00 0.00
ATOM 248 CG GLN 16 -13.953 6.402 -5.017 1.00 0.00
ATOM 249 HG2 GLN 16 -13.523 7.020 -4.228 1.00 0.00
ATOM 250 HG3 GLN 16 -13.584 6.740 -5.985 1.00 0.00
ATOM 251 CD GLN 16 -15.470 6.563 -4.990 1.00 0.00
ATOM 252 OE1 GLN 16 -16.206 5.575 -4.974 1.00 0.00
ATOM 253 NE2 GLN 16 -15.980 7.764 -4.987 1.00 0.00
ATOM 254 HE21 GLN 16 -16.982 7.883 -4.969 1.00 0.00
ATOM 255 HE22 GLN 16 -15.369 8.568 -5.002 1.00 0.00
ATOM 256 C GLN 16 -11.576 3.409 -4.572 1.00 0.00
ATOM 257 O GLN 16 -12.346 2.575 -4.087 1.00 0.00

ATOM	258	N	MET	17	-10.321	3.134	-4.920	1.00	0.00
ATOM	259	H	MET	17	-9.746	3.847	-5.346	1.00	0.00
ATOM	260	CA	MET	17	-9.755	1.801	-4.749	1.00	0.00
ATOM	261	HA	MET	17	-10.393	1.060	-5.231	1.00	0.00
ATOM	262	CB	MET	17	-8.360	1.735	-5.385	1.00	0.00
ATOM	263	HB2	MET	17	-8.204	0.736	-5.793	1.00	0.00
ATOM	264	HB3	MET	17	-8.303	2.468	-6.189	1.00	0.00
ATOM	265	CG	MET	17	-7.279	2.034	-4.345	1.00	0.00
ATOM	266	HG2	MET	17	-7.609	2.835	-3.684	1.00	0.00
ATOM	267	HG3	MET	17	-7.072	1.140	-3.756	1.00	0.00
ATOM	268	SD	MET	17	-5.770	2.548	-5.193	1.00	0.00
ATOM	269	CE	MET	17	-6.400	4.123	-5.821	1.00	0.00
ATOM	270	HE1	MET	17	-6.703	4.753	-4.985	1.00	0.00
ATOM	271	HE2	MET	17	-5.618	4.626	-6.390	1.00	0.00
ATOM	272	HE3	MET	17	-7.258	3.940	-6.467	1.00	0.00
ATOM	273	C	MET	17	-9.673	1.410	-3.282	1.00	0.00
ATOM	274	O	MET	17	-9.672	0.228	-2.954	1.00	0.00
ATOM	275	N	LEU	18	-9.591	2.411	-2.414	1.00	0.00
ATOM	276	H	LEU	18	-9.576	3.365	-2.745	1.00	0.00
ATOM	277	CA	LEU	18	-9.480	2.176	-0.977	1.00	0.00
ATOM	278	HA	LEU	18	-8.582	1.595	-0.764	1.00	0.00
ATOM	279	CB	LEU	18	-9.392	3.498	-0.231	1.00	0.00

ATOM	280	HB2	LEU	18	-10.313	4.059	-0.387	1.00	0.00
ATOM	281	HB3	LEU	18	-9.258	3.309	0.834	1.00	0.00
ATOM	282	CG	LEU	18	-8.205	4.310	-0.758	1.00	0.00
ATOM	283	HG	LEU	18	-7.349	3.650	-0.901	1.00	0.00
ATOM	284	CD1	LEU	18	-8.578	4.959	-2.095	1.00	0.00
ATOM	285	HD11	LEU	18	-9.433	5.619	-1.952	1.00	0.00
ATOM	286	HD12	LEU	18	-7.732	5.536	-2.468	1.00	0.00
ATOM	287	HD13	LEU	18	-8.834	4.183	-2.817	1.00	0.00
ATOM	288	CD2	LEU	18	-7.838	5.398	0.256	1.00	0.00
ATOM	289	HD21	LEU	18	-7.567	4.934	1.205	1.00	0.00
ATOM	290	HD22	LEU	18	-6.993	5.975	-0.119	1.00	0.00
ATOM	291	HD23	LEU	18	-8.691	6.059	0.406	1.00	0.00
ATOM	292	C	LEU	18	-10.666	1.374	-0.459	1.00	0.00
ATOM	293	O	LEU	18	-10.559	0.711	0.564	1.00	0.00
ATOM	294	N	SER	19	-11.795	1.424	-1.162	1.00	0.00
ATOM	295	H	SER	19	-11.873	1.993	-1.993	1.00	0.00
ATOM	296	CA	SER	19	-12.962	0.660	-0.734	1.00	0.00
ATOM	297	HA	SER	19	-13.201	0.896	0.303	1.00	0.00
ATOM	298	CB	SER	19	-14.170	1.008	-1.607	1.00	0.00
ATOM	299	HB2	SER	19	-13.997	0.662	-2.626	1.00	0.00
ATOM	300	HB3	SER	19	-15.062	0.525	-1.207	1.00	0.00
ATOM	301	OG	SER	19	-14.355	2.418	-1.610	1.00	0.00

```
ATOM    302  HG  SER    19     -15.112   2.640  -2.157  1.00  0.00
ATOM    303  C   SER    19     -12.653  -0.832  -0.848  1.00  0.00
ATOM    304  O   SER    19     -12.767  -1.590   0.123  1.00  0.00
ATOM    305  N   HIE    20     -12.220  -1.232  -2.042  1.00  0.00
ATOM    306  H   HIE    20     -12.145  -0.568  -2.799  1.00  0.00
ATOM    307  CA  HIE    20     -11.853  -2.620  -2.300  1.00  0.00
ATOM    308  HA  HIE    20     -12.629  -3.286  -1.923  1.00  0.00
ATOM    309  CB  HIE    20     -11.694  -2.857  -3.809  1.00  0.00
ATOM    310  HB2 HIE    20     -10.657  -2.821  -4.142  1.00  0.00
ATOM    311  HB3 HIE    20     -12.136  -3.809  -4.102  1.00  0.00
ATOM    312  CG  HIE    20     -12.429  -1.787  -4.571  1.00  0.00
ATOM    313  ND1 HIE    20     -11.776  -0.898  -5.409  1.00  0.00
ATOM    314  CE1 HIE    20     -12.701  -0.063  -5.915  1.00  0.00
ATOM    315  HE1 HIE    20     -12.354   0.708  -6.603  1.00  0.00
ATOM    316  NE2 HIE    20     -13.924  -0.346  -5.461  1.00  0.00
ATOM    317  HE2 HIE    20     -14.807   0.102  -5.659  1.00  0.00
ATOM    318  CD2 HIE    20     -13.755  -1.436  -4.613  1.00  0.00
ATOM    319  HD2 HIE    20     -14.616  -1.871  -4.106  1.00  0.00
ATOM    320  C   HIE    20     -10.542  -2.919  -1.592  1.00  0.00
ATOM    321  O   HIE    20     -10.328  -4.014  -1.073  1.00  0.00
ATOM    322  N   CYX    21      -9.675  -1.913  -1.570  1.00  0.00
ATOM    323  H   CYX    21      -9.898  -1.050  -2.044  1.00  0.00
```

ATOM	324	CA	CYX	21	-8.385	-2.025	-0.918	1.00	0.00
ATOM	325	HA	CYX	21	-7.812	-2.852	-1.337	1.00	0.00
ATOM	326	CB	CYX	21	-7.604	-0.723	-1.144	1.00	0.00
ATOM	327	HB2	CYX	21	-8.293	0.068	-1.437	1.00	0.00
ATOM	328	HB3	CYX	21	-7.096	-0.439	-0.222	1.00	0.00
ATOM	329	SG	CYX	21	-6.408	-0.983	-2.479	1.00	0.00
ATOM	330	C	CYX	21	-8.587	-2.304	0.571	1.00	0.00
ATOM	331	O	CYX	21	-7.974	-3.213	1.133	1.00	0.00
ATOM	332	N	ARG	22	-9.487	-1.547	1.192	1.00	0.00
ATOM	333	H	ARG	22	-9.963	-0.805	0.700	1.00	0.00
ATOM	334	CA	ARG	22	-9.803	-1.756	2.598	1.00	0.00
ATOM	335	HA	ARG	22	-8.911	-1.604	3.206	1.00	0.00
ATOM	336	CB	ARG	22	-10.879	-0.765	3.051	1.00	0.00
ATOM	337	HB2	ARG	22	-10.580	0.244	2.768	1.00	0.00
ATOM	338	HB3	ARG	22	-11.823	-1.014	2.567	1.00	0.00
ATOM	339	CG	ARG	22	-11.051	-0.838	4.571	1.00	0.00
ATOM	340	HG2	ARG	22	-11.270	-1.865	4.864	1.00	0.00
ATOM	341	HG3	ARG	22	-10.132	-0.511	5.058	1.00	0.00
ATOM	342	CD	ARG	22	-12.207	0.072	4.998	1.00	0.00
ATOM	343	HD2	ARG	22	-13.148	-0.316	4.608	1.00	0.00
ATOM	344	HD3	ARG	22	-12.258	0.121	6.086	1.00	0.00
ATOM	345	NE	ARG	22	-12.005	1.423	4.477	1.00	0.00

ATOM	346	HE	ARG	22	-11.383	2.060	4.955	1.00	0.00
ATOM	347	CZ	ARG	22	-12.627	1.847	3.374	1.00	0.00
ATOM	348	NH1	ARG	22	-13.456	1.061	2.739	1.00	0.00
ATOM	349	HH11	ARG	22	-13.627	0.127	3.085	1.00	0.00
ATOM	350	HH12	ARG	22	-13.921	1.390	1.906	1.00	0.00
ATOM	351	NH2	ARG	22	-12.406	3.052	2.925	1.00	0.00
ATOM	352	HH21	ARG	22	-11.764	3.660	3.414	1.00	0.00
ATOM	353	HH22	ARG	22	-12.877	3.371	2.091	1.00	0.00
ATOM	354	C	ARG	22	-10.300	-3.182	2.769	1.00	0.00
ATOM	355	O	ARG	22	-9.870	-3.909	3.672	1.00	0.00
ATOM	356	N	MET	23	-11.191	-3.588	1.858	1.00	0.00
ATOM	357	H	MET	23	-11.527	-2.958	1.145	1.00	0.00
ATOM	358	CA	MET	23	-11.715	-4.943	1.883	1.00	0.00
ATOM	359	HA	MET	23	-12.265	-5.111	2.809	1.00	0.00
ATOM	360	CB	MET	23	-12.665	-5.168	0.700	1.00	0.00
ATOM	361	HB2	MET	23	-13.418	-4.380	0.687	1.00	0.00
ATOM	362	HB3	MET	23	-12.096	-5.142	-0.229	1.00	0.00
ATOM	363	CG	MET	23	-13.353	-6.528	0.839	1.00	0.00
ATOM	364	HG2	MET	23	-12.602	-7.314	0.924	1.00	0.00
ATOM	365	HG3	MET	23	-13.984	-6.533	1.728	1.00	0.00
ATOM	366	SD	MET	23	-14.378	-6.838	-0.621	1.00	0.00
ATOM	367	CE	MET	23	-13.031	-7.163	-1.787	1.00	0.00

ATOM	368	HE1	MET	23	-12.448	-8.017	-1.441	1.00	0.00
ATOM	369	HE2	MET	23	-13.446	-7.382	-2.771	1.00	0.00
ATOM	370	HE3	MET	23	-12.386	-6.287	-1.852	1.00	0.00
ATOM	371	C	MET	23	-10.550	-5.923	1.814	1.00	0.00
ATOM	372	O	MET	23	-10.550	-6.951	2.488	1.00	0.00
ATOM	373	N	TYR	24	-9.548	-5.574	1.003	1.00	0.00
ATOM	374	H	TYR	24	-9.622	-4.741	0.437	1.00	0.00
ATOM	375	CA	TYR	24	-8.357	-6.404	0.860	1.00	0.00
ATOM	376	HA	TYR	24	-8.633	-7.401	0.515	1.00	0.00
ATOM	377	CB	TYR	24	-7.399	-5.773	-0.172	1.00	0.00
ATOM	378	HB2	TYR	24	-7.391	-6.388	-1.072	1.00	0.00
ATOM	379	HB3	TYR	24	-7.778	-4.780	-0.413	1.00	0.00
ATOM	380	CG	TYR	24	-5.986	-5.656	0.372	1.00	0.00
ATOM	381	CD1	TYR	24	-5.289	-6.798	0.783	1.00	0.00
ATOM	382	HD1	TYR	24	-5.763	-7.778	0.722	1.00	0.00
ATOM	383	CE1	TYR	24	-3.982	-6.682	1.273	1.00	0.00
ATOM	384	HE1	TYR	24	-3.442	-7.571	1.601	1.00	0.00
ATOM	385	CZ	TYR	24	-3.371	-5.425	1.346	1.00	0.00
ATOM	386	OH	TYR	24	-2.081	-5.311	1.822	1.00	0.00
ATOM	387	HH	TYR	24	-1.698	-6.155	2.075	1.00	0.00
ATOM	388	CE2	TYR	24	-4.065	-4.285	0.933	1.00	0.00
ATOM	389	HE2	TYR	24	-3.588	-3.307	0.990	1.00	0.00

```
ATOM    390  CD2 TYR    24      -5.372  -4.399   0.446  1.00  0.00
ATOM    391  HD2 TYR    24      -5.913  -3.510   0.124  1.00  0.00
ATOM    392  C   TYR    24      -7.681  -6.575   2.218  1.00  0.00
ATOM    393  O   TYR    24      -7.275  -7.675   2.582  1.00  0.00
ATOM    394  N   MET    25      -7.575  -5.483   2.974  1.00  0.00
ATOM    395  H   MET    25      -7.916  -4.589   2.653  1.00  0.00
ATOM    396  CA  MET    25      -6.961  -5.555   4.297  1.00  0.00
ATOM    397  HA  MET    25      -5.912  -5.839   4.207  1.00  0.00
ATOM    398  CB  MET    25      -7.041  -4.193   4.988  1.00  0.00
ATOM    399  HB2 MET    25      -8.087  -3.919   5.122  1.00  0.00
ATOM    400  HB3 MET    25      -6.555  -4.255   5.962  1.00  0.00
ATOM    401  CG  MET    25      -6.339  -3.130   4.133  1.00  0.00
ATOM    402  HG2 MET    25      -6.755  -3.138   3.126  1.00  0.00
ATOM    403  HG3 MET    25      -6.486  -2.147   4.580  1.00  0.00
ATOM    404  SD  MET    25      -4.567  -3.485   4.046  1.00  0.00
ATOM    405  CE  MET    25      -4.159  -3.037   5.751  1.00  0.00
ATOM    406  HE1 MET    25      -4.724  -3.667   6.438  1.00  0.00
ATOM    407  HE2 MET    25      -3.092  -3.183   5.920  1.00  0.00
ATOM    408  HE3 MET    25      -4.414  -1.992   5.924  1.00  0.00
ATOM    409  C   MET    25      -7.706  -6.589   5.136  1.00  0.00
ATOM    410  O   MET    25      -7.108  -7.507   5.717  1.00  0.00
ATOM    411  N   ARG    26      -9.031  -6.455   5.164  1.00  0.00
```

ATOM	412	H	ARG	26	-9.485	-5.687	4.692	1.00	0.00
ATOM	413	CA	ARG	26	-9.858	-7.400	5.897	1.00	0.00
ATOM	414	HA	ARG	26	-9.582	-7.396	6.952	1.00	0.00
ATOM	415	CB	ARG	26	-11.337	-7.012	5.775	1.00	0.00
ATOM	416	HB2	ARG	26	-11.468	-5.984	6.111	1.00	0.00
ATOM	417	HB3	ARG	26	-11.646	-7.096	4.733	1.00	0.00
ATOM	418	CG	ARG	26	-12.194	-7.944	6.639	1.00	0.00
ATOM	419	HG2	ARG	26	-13.246	-7.791	6.399	1.00	0.00
ATOM	420	HG3	ARG	26	-11.921	-8.979	6.431	1.00	0.00
ATOM	421	CD	ARG	26	-11.960	-7.643	8.122	1.00	0.00
ATOM	422	HD2	ARG	26	-10.898	-7.722	8.355	1.00	0.00
ATOM	423	HD3	ARG	26	-12.309	-6.638	8.357	1.00	0.00
ATOM	424	NE	ARG	26	-12.692	-8.599	8.950	1.00	0.00
ATOM	425	HE	ARG	26	-13.601	-8.928	8.657	1.00	0.00
ATOM	426	CZ	ARG	26	-12.189	-9.057	10.097	1.00	0.00
ATOM	427	NH1	ARG	26	-11.021	-8.651	10.516	1.00	0.00
ATOM	428	HH11	ARG	26	-10.494	-7.986	9.968	1.00	0.00
ATOM	429	HH12	ARG	26	-10.649	-9.005	11.386	1.00	0.00
ATOM	430	NH2	ARG	26	-12.870	-9.916	10.804	1.00	0.00
ATOM	431	HH21	ARG	26	-13.773	-10.230	10.479	1.00	0.00
ATOM	432	HH22	ARG	26	-12.490	-10.264	11.673	1.00	0.00
ATOM	433	C	ARG	26	-9.624	-8.793	5.327	1.00	0.00

ATOM	434	O	ARG	26	-9.515	-9.780	6.062	1.00	0.00
ATOM	435	N	GLN	27	-9.507	-8.853	4.005	1.00	0.00
ATOM	436	H	GLN	27	-9.631	-8.025	3.440	1.00	0.00
ATOM	437	CA	GLN	27	-9.242	-10.106	3.335	1.00	0.00
ATOM	438	HA	GLN	27	-10.036	-10.818	3.560	1.00	0.00
ATOM	439	CB	GLN	27	-9.179	-9.906	1.813	1.00	0.00
ATOM	440	HB2	GLN	27	-10.157	-9.589	1.453	1.00	0.00
ATOM	441	HB3	GLN	27	-8.440	-9.138	1.583	1.00	0.00
ATOM	442	CG	GLN	27	-8.782	-11.220	1.125	1.00	0.00
ATOM	443	HG2	GLN	27	-8.898	-11.114	0.047	1.00	0.00
ATOM	444	HG3	GLN	27	-7.746	-11.470	1.356	1.00	0.00
ATOM	445	CD	GLN	27	-9.675	-12.360	1.601	1.00	0.00
ATOM	446	OE1	GLN	27	-10.517	-12.852	0.850	1.00	0.00
ATOM	447	NE2	GLN	27	-9.542	-12.801	2.821	1.00	0.00
ATOM	448	HE21	GLN	27	-10.127	-13.556	3.149	1.00	0.00
ATOM	449	HE22	GLN	27	-8.854	-12.386	3.433	1.00	0.00
ATOM	450	C	GLN	27	-7.935	-10.677	3.855	1.00	0.00
ATOM	451	O	GLN	27	-7.801	-11.875	4.053	1.00	0.00
ATOM	452	N	GLN	28	-6.970	-9.815	4.111	1.00	0.00
ATOM	453	H	GLN	28	-7.079	-8.823	3.959	1.00	0.00
ATOM	454	CA	GLN	28	-5.715	-10.291	4.624	1.00	0.00
ATOM	455	HA	GLN	28	-5.251	-10.976	3.914	1.00	0.00

ATOM 456 CB GLN 28 -4.786 -9.122 4.845 1.00 0.00
ATOM 457 HB2 GLN 28 -4.733 -8.531 3.931 1.00 0.00
ATOM 458 HB3 GLN 28 -5.175 -8.505 5.655 1.00 0.00
ATOM 459 CG GLN 28 -3.384 -9.621 5.212 1.00 0.00
ATOM 460 HG2 GLN 28 -3.144 -10.512 4.632 1.00 0.00
ATOM 461 HG3 GLN 28 -2.648 -8.844 5.005 1.00 0.00
ATOM 462 CD GLN 28 -3.317 -9.973 6.696 1.00 0.00
ATOM 463 OE1 GLN 28 -2.879 -11.148 7.058 1.00 0.00
ATOM 464 NE2 GLN 28 -3.673 -9.157 7.547 1.00 0.00
ATOM 465 HE21 GLN 28 -3.624 -9.400 8.527 1.00 0.00
ATOM 466 HE22 GLN 28 -4.011 -8.250 7.262 1.00 0.00
ATOM 467 C GLN 28 -5.957 -11.008 5.941 1.00 0.00
ATOM 468 O GLN 28 -5.350 -12.044 6.217 1.00 0.00
ATOM 469 N MET 29 -6.862 -10.452 6.749 1.00 0.00
ATOM 470 H MET 29 -7.326 -9.591 6.500 1.00 0.00
ATOM 471 CA MET 29 -7.183 -11.065 8.035 1.00 0.00
ATOM 472 HA MET 29 -6.283 -11.129 8.648 1.00 0.00
ATOM 473 CB MET 29 -8.227 -10.230 8.784 1.00 0.00
ATOM 474 HB2 MET 29 -9.038 -9.970 8.105 1.00 0.00
ATOM 475 HB3 MET 29 -8.623 -10.809 9.618 1.00 0.00
ATOM 476 CG MET 29 -7.578 -8.951 9.315 1.00 0.00
ATOM 477 HG2 MET 29 -7.198 -8.355 8.486 1.00 0.00

ATOM	478	HG3	MET	29	-8.309	-8.369	9.876	1.00	0.00
ATOM	479	SD	MET	29	-6.205	-9.393	10.408	1.00	0.00
ATOM	480	CE	MET	29	-7.165	-10.344	11.615	1.00	0.00
ATOM	481	HE1	MET	29	-7.647	-11.184	11.116	1.00	0.00
ATOM	482	HE2	MET	29	-6.501	-10.718	12.395	1.00	0.00
ATOM	483	HE3	MET	29	-7.924	-9.703	12.063	1.00	0.00
ATOM	484	C	MET	29	-7.714	-12.480	7.830	1.00	0.00
ATOM	485	O	MET	29	-7.169	-13.436	8.379	1.00	0.00
ATOM	486	N	GLU	30	-8.767	-12.617	7.024	1.00	0.00
ATOM	487	H	GLU	30	-9.213	-11.816	6.602	1.00	0.00
ATOM	488	CA	GLU	30	-9.325	-13.944	6.756	1.00	0.00
ATOM	489	HA	GLU	30	-9.532	-14.454	7.697	1.00	0.00
ATOM	490	CB	GLU	30	-10.631	-13.829	5.963	1.00	0.00
ATOM	491	HB2	GLU	30	-10.465	-13.211	5.081	1.00	0.00
ATOM	492	HB3	GLU	30	-10.954	-14.823	5.654	1.00	0.00
ATOM	493	CG	GLU	30	-11.713	-13.190	6.838	1.00	0.00
ATOM	494	HG2	GLU	30	-12.694	-13.541	6.519	1.00	0.00
ATOM	495	HG3	GLU	30	-11.543	-13.481	7.875	1.00	0.00
ATOM	496	CD	GLU	30	-11.654	-11.671	6.721	1.00	0.00
ATOM	497	OE1	GLU	30	-11.795	-11.176	5.614	1.00	0.00
ATOM	498	OE2	GLU	30	-11.477	-11.024	7.741	1.00	0.00
ATOM	499	C	GLU	30	-8.326	-14.796	5.974	1.00	0.00

ATOM	500	O	GLU	30	-8.143	-15.981	6.251	1.00	0.00
ATOM	501	N	GLU	31	-7.692	-14.166	4.987	1.00	0.00
ATOM	502	H	GLU	31	-7.913	-13.200	4.796	1.00	0.00
ATOM	503	CA	GLU	31	-6.710	-14.834	4.135	1.00	0.00
ATOM	504	HA	GLU	31	-7.120	-15.766	3.745	1.00	0.00
ATOM	505	CB	GLU	31	-6.350	-13.925	2.954	1.00	0.00
ATOM	506	HB2	GLU	31	-7.258	-13.464	2.567	1.00	0.00
ATOM	507	HB3	GLU	31	-5.666	-13.148	3.295	1.00	0.00
ATOM	508	CG	GLU	31	-5.679	-14.745	1.844	1.00	0.00
ATOM	509	HG2	GLU	31	-4.821	-15.271	2.262	1.00	0.00
ATOM	510	HG3	GLU	31	-6.396	-15.470	1.458	1.00	0.00
ATOM	511	CD	GLU	31	-5.213	-13.833	0.706	1.00	0.00
ATOM	512	OE1	GLU	31	-5.354	-12.625	0.832	1.00	0.00
ATOM	513	OE2	GLU	31	-4.719	-14.359	-0.279	1.00	0.00
ATOM	514	C	GLU	31	-5.445	-15.198	4.920	1.00	0.00
ATOM	515	O	GLU	31	-4.539	-15.824	4.378	1.00	0.00
ATOM	516	N	SER	32	-5.384	-14.792	6.190	1.00	0.00
ATOM	517	H	SER	32	-6.144	-14.259	6.588	1.00	0.00
ATOM	518	CA	SER	32	-4.216	-15.071	7.032	1.00	0.00
ATOM	519	HA	SER	32	-3.389	-14.419	6.751	1.00	0.00
ATOM	520	CB	SER	32	-4.557	-14.822	8.501	1.00	0.00
ATOM	521	HB2	SER	32	-3.673	-14.997	9.115	1.00	0.00

ATOM 522 HB3 SER 32 -4.889 -13.792 8.629 1.00 0.00
ATOM 523 OG SER 32 -5.595 -15.708 8.901 1.00 0.00
ATOM 524 HG SER 32 -5.810 -15.553 9.824 1.00 0.00
ATOM 525 C SER 32 -3.747 -16.512 6.851 1.00 0.00
ATOM 526 O SER 32 -2.589 -16.834 7.116 1.00 0.00
ATOM 527 N THR 33 -4.655 -17.370 6.399 1.00 0.00
ATOM 528 H THR 33 -5.604 -17.066 6.237 1.00 0.00
ATOM 529 CA THR 33 -4.327 -18.768 6.156 1.00 0.00
ATOM 530 HA THR 33 -3.695 -19.153 6.957 1.00 0.00
ATOM 531 CB THR 33 -5.614 -19.599 6.106 1.00 0.00
ATOM 532 HB THR 33 -5.370 -20.630 5.847 1.00 0.00
ATOM 533 CG2 THR 33 -6.302 -19.566 7.473 1.00 0.00
ATOM 534 HG21 THR 33 -6.547 -18.536 7.733 1.00 0.00
ATOM 535 HG22 THR 33 -7.216 -20.158 7.435 1.00 0.00
ATOM 536 HG23 THR 33 -5.633 -19.981 8.227 1.00 0.00
ATOM 537 OG1 THR 33 -6.488 -19.059 5.123 1.00 0.00
ATOM 538 HG1 THR 33 -7.294 -19.579 5.092 1.00 0.00
ATOM 539 C THR 33 -3.550 -18.942 4.839 1.00 0.00
ATOM 540 O THR 33 -2.443 -19.480 4.824 1.00 0.00
ATOM 541 N TYR 34 -4.179 -18.521 3.732 1.00 0.00
ATOM 542 H TYR 34 -5.101 -18.118 3.816 1.00 0.00
ATOM 543 CA TYR 34 -3.599 -18.664 2.385 1.00 0.00

ATOM	544	HA	TYR	34	-2.812	-19.419	2.388	1.00	0.00
ATOM	545	CB	TYR	34	-4.694	-19.102	1.408	1.00	0.00
ATOM	546	HB2	TYR	34	-5.463	-18.332	1.352	1.00	0.00
ATOM	547	HB3	TYR	34	-4.257	-19.251	0.421	1.00	0.00
ATOM	548	CG	TYR	34	-5.311	-20.396	1.886	1.00	0.00
ATOM	549	CD1	TYR	34	-4.633	-21.607	1.699	1.00	0.00
ATOM	550	HD1	TYR	34	-3.660	-21.617	1.207	1.00	0.00
ATOM	551	CE1	TYR	34	-5.204	-22.805	2.144	1.00	0.00
ATOM	552	HE1	TYR	34	-4.676	-23.748	1.998	1.00	0.00
ATOM	553	CZ	TYR	34	-6.453	-22.793	2.777	1.00	0.00
ATOM	554	OH	TYR	34	-7.015	-23.974	3.215	1.00	0.00
ATOM	555	HH	TYR	34	-6.465	-24.741	3.035	1.00	0.00
ATOM	556	CE2	TYR	34	-7.130	-21.582	2.964	1.00	0.00
ATOM	557	HE2	TYR	34	-8.102	-21.573	3.457	1.00	0.00
ATOM	558	CD2	TYR	34	-6.560	-20.385	2.520	1.00	0.00
ATOM	559	HD2	TYR	34	-7.087	-19.443	2.667	1.00	0.00
ATOM	560	C	TYR	34	-2.934	-17.386	1.844	1.00	0.00
ATOM	561	O	TYR	34	-2.550	-17.354	0.674	1.00	0.00
ATOM	562	N	GLN	35	-2.844	-16.332	2.658	1.00	0.00
ATOM	563	H	GLN	35	-3.199	-16.388	3.602	1.00	0.00
ATOM	564	CA	GLN	35	-2.264	-15.052	2.203	1.00	0.00
ATOM	565	HA	GLN	35	-3.034	-14.467	1.702	1.00	0.00

```
ATOM    566  CB  GLN    35      -1.712 -14.233   3.376  1.00  0.00
ATOM    567  HB2 GLN    35      -1.507 -13.219   3.034  1.00  0.00
ATOM    568  HB3 GLN    35      -2.458 -14.205   4.171  1.00  0.00
ATOM    569  CG  GLN    35      -0.417 -14.863   3.912  1.00  0.00
ATOM    570  HG2 GLN    35       0.303 -14.991   3.103  1.00  0.00
ATOM    571  HG3 GLN    35       0.013 -14.222   4.682  1.00  0.00
ATOM    572  CD  GLN    35      -0.709 -16.226   4.522  1.00  0.00
ATOM    573  OE1 GLN    35      -1.912 -16.484   4.939  1.00  0.00
ATOM    574  NE2 GLN    35       0.177 -17.074   4.615  1.00  0.00
ATOM    575 HE21 GLN    35      -0.031 -17.975   5.022  1.00  0.00
ATOM    576 HE22 GLN    35       1.108 -16.866   4.284  1.00  0.00
ATOM    577  C   GLN    35      -1.140 -15.235   1.194  1.00  0.00
ATOM    578  O   GLN    35      -0.948 -14.379   0.329  1.00  0.00
ATOM    579  N   THR    36      -0.392 -16.329   1.296  1.00  0.00
ATOM    580  H   THR    36      -0.547 -17.022   2.014  1.00  0.00
ATOM    581  CA  THR    36       0.698 -16.539   0.361  1.00  0.00
ATOM    582  HA  THR    36       1.421 -15.727   0.440  1.00  0.00
ATOM    583  CB  THR    36       1.410 -17.860   0.671  1.00  0.00
ATOM    584  HB  THR    36       0.734 -18.691   0.469  1.00  0.00
ATOM    585  CG2 THR    36       2.658 -17.993  -0.206  1.00  0.00
ATOM    586 HG21 THR    36       3.335 -17.163  -0.004  1.00  0.00
ATOM    587 HG22 THR    36       3.162 -18.934   0.018  1.00  0.00
```

ATOM 588 HG23 THR 36 2.368 -17.978 -1.256 1.00 0.00

ATOM 589 OG1 THR 36 1.788 -17.880 2.040 1.00 0.00

ATOM 590 HG1 THR 36 2.235 -18.707 2.237 1.00 0.00

ATOM 591 C THR 36 0.136 -16.568 -1.054 1.00 0.00

ATOM 592 O THR 36 -0.524 -17.525 -1.458 1.00 0.00

ATOM 593 N MET 37 0.396 -15.492 -1.788 1.00 0.00

ATOM 594 H MET 37 0.925 -14.727 -1.394 1.00 0.00

ATOM 595 CA MET 37 -0.091 -15.363 -3.158 1.00 0.00

ATOM 596 HA MET 37 -1.177 -15.447 -3.180 1.00 0.00

ATOM 597 CB MET 37 0.306 -13.995 -3.720 1.00 0.00

ATOM 598 HB2 MET 37 1.383 -13.859 -3.615 1.00 0.00

ATOM 599 HB3 MET 37 0.035 -13.944 -4.774 1.00 0.00

ATOM 600 CG MET 37 -0.426 -12.890 -2.951 1.00 0.00

ATOM 601 HG2 MET 37 -0.413 -13.114 -1.884 1.00 0.00

ATOM 602 HG3 MET 37 0.065 -11.933 -3.126 1.00 0.00

ATOM 603 SD MET 37 -2.143 -12.792 -3.519 1.00 0.00

ATOM 604 CE MET 37 -1.863 -11.663 -4.905 1.00 0.00

ATOM 605 HE1 MET 37 -1.167 -12.118 -5.610 1.00 0.00

ATOM 606 HE2 MET 37 -2.809 -11.462 -5.407 1.00 0.00

ATOM 607 HE3 MET 37 -1.444 -10.728 -4.533 1.00 0.00

ATOM 608 C MET 37 0.473 -16.468 -4.051 1.00 0.00

ATOM 609 O MET 37 1.679 -16.524 -4.292 1.00 0.00

ATOM	610	N	PRO	38	-0.375	-17.335	-4.553	1.00	0.00
ATOM	611	CD	PRO	38	-1.832	-17.353	-4.325	1.00	0.00
ATOM	612	HD2	PRO	38	-2.269	-16.451	-4.751	1.00	0.00
ATOM	613	HD3	PRO	38	-2.023	-17.378	-3.252	1.00	0.00
ATOM	614	CG	PRO	38	-2.306	-18.629	-5.033	1.00	0.00
ATOM	615	HG2	PRO	38	-2.814	-18.354	-5.957	1.00	0.00
ATOM	616	HG3	PRO	38	-3.000	-19.160	-4.381	1.00	0.00
ATOM	617	CB	PRO	38	-1.091	-19.455	-5.303	1.00	0.00
ATOM	618	HB2	PRO	38	-1.242	-19.982	-6.245	1.00	0.00
ATOM	619	HB3	PRO	38	-0.852	-20.175	-4.520	1.00	0.00
ATOM	620	CA	PRO	38	0.045	-18.447	-5.448	1.00	0.00
ATOM	621	HA	PRO	38	1.005	-18.828	-5.099	1.00	0.00
ATOM	622	C	PRO	38	0.179	-17.981	-6.895	1.00	0.00
ATOM	623	O	PRO	38	-0.587	-17.132	-7.353	1.00	0.00
ATOM	624	N	ARG	39	1.149	-18.541	-7.611	1.00	0.00
ATOM	625	H	ARG	39	1.745	-19.252	-7.211	1.00	0.00
ATOM	626	CA	ARG	39	1.358	-18.167	-9.007	1.00	0.00
ATOM	627	HA	ARG	39	1.577	-17.102	-9.074	1.00	0.00
ATOM	628	CB	ARG	39	2.528	-18.943	-9.600	1.00	0.00
ATOM	629	HB2	ARG	39	2.262	-19.998	-9.668	1.00	0.00
ATOM	630	HB3	ARG	39	2.747	-18.559	-10.596	1.00	0.00
ATOM	631	CG	ARG	39	3.765	-18.786	-8.710	1.00	0.00

```
ATOM    632  HG2 ARG    39       3.986 -17.727  -8.579  1.00  0.00
ATOM    633  HG3 ARG    39       3.573 -19.240  -7.737  1.00  0.00
ATOM    634  CD  ARG    39       4.961 -19.479  -9.368  1.00  0.00
ATOM    635  HD2 ARG    39       4.694 -20.501  -9.638  1.00  0.00
ATOM    636  HD3 ARG    39       5.259 -18.933 -10.263  1.00  0.00
ATOM    637  NE  ARG    39       6.093 -19.520  -8.447  1.00  0.00
ATOM    638  HE  ARG    39       6.857 -18.868  -8.554  1.00  0.00
ATOM    639  CZ  ARG    39       6.149 -20.407  -7.453  1.00  0.00
ATOM    640  NH1 ARG    39       5.179 -21.264  -7.282  1.00  0.00
ATOM    641 HH11 ARG    39       4.385 -21.254  -7.905  1.00  0.00
ATOM    642 HH12 ARG    39       5.228 -21.933  -6.527  1.00  0.00
ATOM    643  NH2 ARG    39       7.177 -20.417  -6.649  1.00  0.00
ATOM    644 HH21 ARG    39       7.926 -19.753  -6.783  1.00  0.00
ATOM    645 HH22 ARG    39       7.219 -21.089  -5.896  1.00  0.00
ATOM    646  C   ARG    39       0.126 -18.508  -9.821  1.00  0.00
ATOM    647  O   ARG    39      -0.276 -17.757 -10.710  1.00  0.00
ATOM    648  N   ARG    40      -0.464 -19.662  -9.514  1.00  0.00
ATOM    649  H   ARG    40      -0.086 -20.262  -8.794  1.00  0.00
ATOM    650  CA  ARG    40      -1.641 -20.108 -10.231  1.00  0.00
ATOM    651  HA  ARG    40      -1.472 -20.032 -11.305  1.00  0.00
ATOM    652  CB  ARG    40      -1.952 -21.566  -9.888  1.00  0.00
ATOM    653  HB2 ARG    40      -1.970 -21.689  -8.805  1.00  0.00
```

```
ATOM    654  HB3 ARG    40      -2.923 -21.838 -10.300  1.00  0.00
ATOM    655  CG  ARG    40      -0.873 -22.472 -10.486  1.00  0.00
ATOM    656  HG2 ARG    40       0.109 -22.045 -10.283  1.00  0.00
ATOM    657  HG3 ARG    40      -0.939 -23.460 -10.030  1.00  0.00
ATOM    658  CD  ARG    40      -1.074 -22.594 -12.001  1.00  0.00
ATOM    659  HD2 ARG    40      -1.060 -21.604 -12.456  1.00  0.00
ATOM    660  HD3 ARG    40      -0.278 -23.202 -12.431  1.00  0.00
ATOM    661  NE  ARG    40      -2.357 -23.227 -12.295  1.00  0.00
ATOM    662  HE  ARG    40      -3.150 -22.661 -12.558  1.00  0.00
ATOM    663  CZ  ARG    40      -2.514 -24.548 -12.225  1.00  0.00
ATOM    664  NH1 ARG    40      -1.512 -25.313 -11.883  1.00  0.00
ATOM    665 HH11 ARG    40      -0.615 -24.901 -11.671  1.00  0.00
ATOM    666 HH12 ARG    40      -1.639 -26.314 -11.832  1.00  0.00
ATOM    667  NH2 ARG    40      -3.674 -25.080 -12.499  1.00  0.00
ATOM    668 HH21 ARG    40      -4.448 -24.488 -12.763  1.00  0.00
ATOM    669 HH22 ARG    40      -3.792 -26.082 -12.445  1.00  0.00
ATOM    670  C   ARG    40      -2.826 -19.230  -9.890  1.00  0.00
ATOM    671  O   ARG    40      -3.472 -19.393  -8.853  1.00  0.00
ATOM    672  N   GLY    41      -3.095 -18.306 -10.791  1.00  0.00
ATOM    673  H   GLY    41      -2.520 -18.230 -11.618  1.00  0.00
ATOM    674  CA  GLY    41      -4.211 -17.375 -10.634  1.00  0.00
ATOM    675  HA2 GLY    41      -4.148 -16.614 -11.411  1.00  0.00
```

ATOM 676 HA3 GLY 41 -5.146 -17.924 -10.739 1.00 0.00
ATOM 677 C GLY 41 -4.185 -16.694 -9.268 1.00 0.00
ATOM 678 O GLY 41 -4.841 -17.146 -8.328 1.00 0.00
ATOM 679 N MET 42 -3.429 -15.601 -9.168 1.00 0.00
ATOM 680 H MET 42 -2.896 -15.276 -9.962 1.00 0.00
ATOM 681 CA MET 42 -3.326 -14.857 -7.912 1.00 0.00
ATOM 682 HA MET 42 -2.880 -15.485 -7.140 1.00 0.00
ATOM 683 CB MET 42 -2.441 -13.622 -8.110 1.00 0.00
ATOM 684 HB2 MET 42 -2.815 -13.039 -8.951 1.00 0.00
ATOM 685 HB3 MET 42 -2.463 -13.012 -7.207 1.00 0.00
ATOM 686 CG MET 42 -1.002 -14.060 -8.394 1.00 0.00
ATOM 687 HG2 MET 42 -0.644 -14.699 -7.586 1.00 0.00
ATOM 688 HG3 MET 42 -0.962 -14.609 -9.335 1.00 0.00
ATOM 689 SD MET 42 0.060 -12.600 -8.512 1.00 0.00
ATOM 690 CE MET 42 1.587 -13.463 -8.959 1.00 0.00
ATOM 691 HE1 MET 42 1.439 -14.005 -9.893 1.00 0.00
ATOM 692 HE2 MET 42 2.392 -12.738 -9.084 1.00 0.00
ATOM 693 HE3 MET 42 1.852 -14.167 -8.169 1.00 0.00
ATOM 694 C MET 42 -4.709 -14.423 -7.427 1.00 0.00
ATOM 695 O MET 42 -5.702 -14.569 -8.141 1.00 0.00
ATOM 696 N GLU 43 -4.767 -13.891 -6.207 1.00 0.00
ATOM 697 H GLU 43 -3.933 -13.804 -5.643 1.00 0.00

ATOM 698 CA GLU 43 -6.037 -13.446 -5.639 1.00 0.00

ATOM 699 HA GLU 43 -6.785 -14.233 -5.735 1.00 0.00

ATOM 700 CB GLU 43 -5.860 -13.118 -4.152 1.00 0.00

ATOM 701 HB2 GLU 43 -5.263 -13.897 -3.677 1.00 0.00

ATOM 702 HB3 GLU 43 -5.352 -12.159 -4.049 1.00 0.00

ATOM 703 CG GLU 43 -7.233 -13.044 -3.476 1.00 0.00

ATOM 704 HG2 GLU 43 -7.815 -12.238 -3.921 1.00 0.00

ATOM 705 HG3 GLU 43 -7.750 -13.992 -3.628 1.00 0.00

ATOM 706 CD GLU 43 -7.071 -12.788 -1.979 1.00 0.00

ATOM 707 OE1 GLU 43 -6.548 -11.744 -1.629 1.00 0.00

ATOM 708 OE2 GLU 43 -7.481 -13.637 -1.204 1.00 0.00

ATOM 709 C GLU 43 -6.553 -12.212 -6.382 1.00 0.00

ATOM 710 O GLU 43 -5.829 -11.229 -6.542 1.00 0.00

ATOM 711 N PRO 44 -7.783 -12.253 -6.841 1.00 0.00

ATOM 712 CD PRO 44 -8.715 -13.383 -6.691 1.00 0.00

ATOM 713 HD2 PRO 44 -8.707 -13.719 -5.654 1.00 0.00

ATOM 714 HD3 PRO 44 -8.395 -14.200 -7.339 1.00 0.00

ATOM 715 CG PRO 44 -10.063 -12.802 -7.109 1.00 0.00

ATOM 716 HG2 PRO 44 -10.600 -12.470 -6.221 1.00 0.00

ATOM 717 HG3 PRO 44 -10.643 -13.573 -7.617 1.00 0.00

ATOM 718 CB PRO 44 -9.771 -11.663 -8.027 1.00 0.00

ATOM 719 HB2 PRO 44 -10.533 -10.894 -7.900 1.00 0.00

ATOM 720 HB3 PRO 44 -9.733 -11.969 -9.072 1.00 0.00
ATOM 721 CA PRO 44 -8.405 -11.119 -7.592 1.00 0.00
ATOM 722 HA PRO 44 -7.768 -10.894 -8.447 1.00 0.00
ATOM 723 C PRO 44 -8.567 -9.866 -6.734 1.00 0.00
ATOM 724 O PRO 44 -8.619 -8.752 -7.259 1.00 0.00
ATOM 725 N HIE 45 -8.651 -10.053 -5.419 1.00 0.00
ATOM 726 H HIE 45 -8.623 -10.984 -5.029 1.00 0.00
ATOM 727 CA HIE 45 -8.813 -8.924 -4.506 1.00 0.00
ATOM 728 HA HIE 45 -9.786 -8.459 -4.659 1.00 0.00
ATOM 729 CB HIE 45 -8.722 -9.402 -3.054 1.00 0.00
ATOM 730 HB2 HIE 45 -7.804 -9.956 -2.857 1.00 0.00
ATOM 731 HB3 HIE 45 -8.807 -8.578 -2.346 1.00 0.00
ATOM 732 CG HIE 45 -9.862 -10.340 -2.762 1.00 0.00
ATOM 733 ND1 HIE 45 -9.656 -11.650 -2.360 1.00 0.00
ATOM 734 CE1 HIE 45 -10.864 -12.216 -2.187 1.00 0.00
ATOM 735 HE1 HIE 45 -10.886 -13.258 -1.868 1.00 0.00
ATOM 736 NE2 HIE 45 -11.854 -11.359 -2.449 1.00 0.00
ATOM 737 HE2 HIE 45 -12.854 -11.495 -2.407 1.00 0.00
ATOM 738 CD2 HIE 45 -11.224 -10.174 -2.812 1.00 0.00
ATOM 739 HD2 HIE 45 -11.818 -9.301 -3.079 1.00 0.00
ATOM 740 C HIE 45 -7.741 -7.868 -4.765 1.00 0.00
ATOM 741 O HIE 45 -8.039 -6.674 -4.836 1.00 0.00

ATOM	742	N	MET	46	-6.497	-8.317	-4.916	1.00	0.00
ATOM	743	H	MET	46	-6.294	-9.304	-4.848	1.00	0.00
ATOM	744	CA	MET	46	-5.392	-7.400	-5.178	1.00	0.00
ATOM	745	HA	MET	46	-5.363	-6.623	-4.414	1.00	0.00
ATOM	746	CB	MET	46	-4.061	-8.160	-5.153	1.00	0.00
ATOM	747	HB2	MET	46	-4.034	-8.860	-5.988	1.00	0.00
ATOM	748	HB3	MET	46	-3.241	-7.448	-5.249	1.00	0.00
ATOM	749	CG	MET	46	-3.915	-8.930	-3.838	1.00	0.00
ATOM	750	HG2	MET	46	-4.633	-9.750	-3.813	1.00	0.00
ATOM	751	HG3	MET	46	-2.904	-9.331	-3.760	1.00	0.00
ATOM	752	SD	MET	46	-4.225	-7.825	-2.439	1.00	0.00
ATOM	753	CE	MET	46	-4.831	-9.089	-1.293	1.00	0.00
ATOM	754	HE1	MET	46	-4.054	-9.838	-1.133	1.00	0.00
ATOM	755	HE2	MET	46	-5.088	-8.624	-0.341	1.00	0.00
ATOM	756	HE3	MET	46	-5.715	-9.568	-1.714	1.00	0.00
ATOM	757	C	MET	46	-5.568	-6.747	-6.548	1.00	0.00
ATOM	758	O	MET	46	-5.353	-5.546	-6.712	1.00	0.00
ATOM	759	N	SER	47	-5.961	-7.563	-7.524	1.00	0.00
ATOM	760	H	SER	47	-6.100	-8.545	-7.329	1.00	0.00
ATOM	761	CA	SER	47	-6.169	-7.092	-8.891	1.00	0.00
ATOM	762	HA	SER	47	-5.241	-6.684	-9.292	1.00	0.00
ATOM	763	CB	SER	47	-6.615	-8.256	-9.778	1.00	0.00

ATOM 764 HB2 SER 47 -7.607 -8.588 -9.473 1.00 0.00
ATOM 765 HB3 SER 47 -6.644 -7.932 -10.818 1.00 0.00
ATOM 766 OG SER 47 -5.694 -9.330 -9.643 1.00 0.00
ATOM 767 HG SER 47 -5.972 -10.062 -10.199 1.00 0.00
ATOM 768 C SER 47 -7.213 -5.979 -8.950 1.00 0.00
ATOM 769 O SER 47 -7.130 -5.086 -9.793 1.00 0.00
ATOM 770 N GLU 48 -8.204 -6.048 -8.065 1.00 0.00
ATOM 771 H GLU 48 -8.252 -6.810 -7.403 1.00 0.00
ATOM 772 CA GLU 48 -9.266 -5.045 -8.046 1.00 0.00
ATOM 773 HA GLU 48 -9.830 -5.080 -8.977 1.00 0.00
ATOM 774 CB GLU 48 -10.226 -5.321 -6.880 1.00 0.00
ATOM 775 HB2 GLU 48 -10.050 -6.328 -6.500 1.00 0.00
ATOM 776 HB3 GLU 48 -10.045 -4.597 -6.086 1.00 0.00
ATOM 777 CG GLU 48 -11.679 -5.202 -7.359 1.00 0.00
ATOM 778 HG2 GLU 48 -11.727 -5.458 -8.417 1.00 0.00
ATOM 779 HG3 GLU 48 -12.293 -5.899 -6.789 1.00 0.00
ATOM 780 CD GLU 48 -12.200 -3.780 -7.155 1.00 0.00
ATOM 781 OE1 GLU 48 -11.452 -2.848 -7.401 1.00 0.00
ATOM 782 OE2 GLU 48 -13.346 -3.643 -6.758 1.00 0.00
ATOM 783 C GLU 48 -8.678 -3.641 -7.912 1.00 0.00
ATOM 784 O GLU 48 -8.967 -2.760 -8.723 1.00 0.00
ATOM 785 N CYX 49 -7.845 -3.443 -6.894 1.00 0.00

ATOM	786	H	CYX	49	-7.655	-4.183	-6.233	1.00	0.00
ATOM	787	CA	CYX	49	-7.216	-2.143	-6.677	1.00	0.00
ATOM	788	HA	CYX	49	-7.970	-1.357	-6.697	1.00	0.00
ATOM	789	CB	CYX	49	-6.513	-2.115	-5.309	1.00	0.00
ATOM	790	HB2	CYX	49	-6.307	-3.135	-4.984	1.00	0.00
ATOM	791	HB3	CYX	49	-5.575	-1.566	-5.393	1.00	0.00
ATOM	792	SG	CYX	49	-7.581	-1.288	-4.102	1.00	0.00
ATOM	793	C	CYX	49	-6.210	-1.835	-7.788	1.00	0.00
ATOM	794	O	CYX	49	-6.009	-0.675	-8.141	1.00	0.00
ATOM	795	N	CYX	50	-5.581	-2.883	-8.326	1.00	0.00
ATOM	796	H	CYX	50	-5.777	-3.815	-7.987	1.00	0.00
ATOM	797	CA	CYX	50	-4.582	-2.725	-9.389	1.00	0.00
ATOM	798	HA	CYX	50	-3.694	-2.229	-8.996	1.00	0.00
ATOM	799	CB	CYX	50	-4.175	-4.093	-9.935	1.00	0.00
ATOM	800	HB2	CYX	50	-3.903	-4.749	-9.108	1.00	0.00
ATOM	801	HB3	CYX	50	-5.009	-4.529	-10.484	1.00	0.00
ATOM	802	SG	CYX	50	-2.783	-3.898	-11.080	1.00	0.00
ATOM	803	C	CYX	50	-5.095	-1.872	-10.546	1.00	0.00
ATOM	804	O	CYX	50	-4.373	-1.019	-11.063	1.00	0.00
ATOM	805	N	GLU	51	-6.331	-2.121	-10.966	1.00	0.00
ATOM	806	H	GLU	51	-6.884	-2.850	-10.538	1.00	0.00
ATOM	807	CA	GLU	51	-6.908	-1.380	-12.083	1.00	0.00

```
ATOM    808  HA  GLU    51      -6.309  -1.531 -12.981  1.00  0.00
ATOM    809  CB  GLU    51      -8.334  -1.872 -12.356  1.00  0.00
ATOM    810  HB2 GLU    51      -8.327  -2.956 -12.474  1.00  0.00
ATOM    811  HB3 GLU    51      -8.976  -1.603 -11.517  1.00  0.00
ATOM    812  CG  GLU    51      -8.868  -1.221 -13.639  1.00  0.00
ATOM    813  HG2 GLU    51      -9.915  -1.495 -13.769  1.00  0.00
ATOM    814  HG3 GLU    51      -8.785  -0.139 -13.543  1.00  0.00
ATOM    815  CD  GLU    51      -8.063  -1.689 -14.855  1.00  0.00
ATOM    816  OE1 GLU    51      -7.572  -2.807 -14.833  1.00  0.00
ATOM    817  OE2 GLU    51      -7.953  -0.920 -15.795  1.00  0.00
ATOM    818  C   GLU    51      -6.925   0.124 -11.810  1.00  0.00
ATOM    819  O   GLU    51      -6.707   0.925 -12.717  1.00  0.00
ATOM    820  N   GLN    52      -7.196   0.501 -10.563  1.00  0.00
ATOM    821  H   GLN    52      -7.401  -0.187  -9.852  1.00  0.00
ATOM    822  CA  GLN    52      -7.251   1.915 -10.195  1.00  0.00
ATOM    823  HA  GLN    52      -8.008   2.430 -10.785  1.00  0.00
ATOM    824  CB  GLN    52      -7.614   2.042  -8.718  1.00  0.00
ATOM    825  HB2 GLN    52      -6.856   1.547  -8.110  1.00  0.00
ATOM    826  HB3 GLN    52      -7.669   3.095  -8.443  1.00  0.00
ATOM    827  CG  GLN    52      -8.974   1.380  -8.476  1.00  0.00
ATOM    828  HG2 GLN    52      -9.105   0.540  -9.158  1.00  0.00
ATOM    829  HG3 GLN    52      -9.035   1.025  -7.447  1.00  0.00
```

```
ATOM    830  CD  GLN    52     -10.100   2.382  -8.714  1.00  0.00
ATOM    831  OE1 GLN    52     -10.203   2.958  -9.796  1.00  0.00
ATOM    832  NE2 GLN    52     -10.957   2.624  -7.759  1.00  0.00
ATOM    833 HE21 GLN    52     -11.704   3.286  -7.911  1.00  0.00
ATOM    834 HE22 GLN    52     -10.868   2.147  -6.872  1.00  0.00
ATOM    835  C   GLN    52      -5.922   2.618 -10.473  1.00  0.00
ATOM    836  O   GLN    52      -5.888   3.631 -11.171  1.00  0.00
ATOM    837  N   LEU    53      -4.826   2.068  -9.942  1.00  0.00
ATOM    838  H   LEU    53      -4.883   1.212  -9.408  1.00  0.00
ATOM    839  CA  LEU    53      -3.503   2.658 -10.171  1.00  0.00
ATOM    840  HA  LEU    53      -3.474   3.663  -9.750  1.00  0.00
ATOM    841  CB  LEU    53      -2.403   1.818  -9.508  1.00  0.00
ATOM    842  HB2 LEU    53      -2.237   0.939 -10.131  1.00  0.00
ATOM    843  HB3 LEU    53      -1.488   2.409  -9.465  1.00  0.00
ATOM    844  CG  LEU    53      -2.792   1.374  -8.091  1.00  0.00
ATOM    845  HG  LEU    53      -1.895   1.379  -7.470  1.00  0.00
ATOM    846  CD1 LEU    53      -3.847   2.306  -7.474  1.00  0.00
ATOM    847 HD11 LEU    53      -4.744   2.301  -8.093  1.00  0.00
ATOM    848 HD12 LEU    53      -4.096   1.959  -6.471  1.00  0.00
ATOM    849 HD13 LEU    53      -3.449   3.319  -7.419  1.00  0.00
ATOM    850  CD2 LEU    53      -3.347  -0.047  -8.178  1.00  0.00
ATOM    851 HD21 LEU    53      -2.584  -0.712  -8.584  1.00  0.00
```

ATOM	852	HD22	LEU	53	-3.632	-0.388	-7.182	1.00	0.00
ATOM	853	HD23	LEU	53	-4.221	-0.057	-8.829	1.00	0.00
ATOM	854	C	LEU	53	-3.233	2.650	-11.657	1.00	0.00
ATOM	855	O	LEU	53	-2.858	3.653	-12.264	1.00	0.00
ATOM	856	N	GLU	54	-3.452	1.475	-12.211	1.00	0.00
ATOM	857	H	GLU	54	-3.717	0.704	-11.614	1.00	0.00
ATOM	858	CA	GLU	54	-3.278	1.220	-13.619	1.00	0.00
ATOM	859	HA	GLU	54	-2.235	1.324	-13.919	1.00	0.00
ATOM	860	CB	GLU	54	-3.714	-0.221	-13.864	1.00	0.00
ATOM	861	HB2	GLU	54	-3.006	-0.891	-13.375	1.00	0.00
ATOM	862	HB3	GLU	54	-4.706	-0.367	-13.436	1.00	0.00
ATOM	863	CG	GLU	54	-3.755	-0.528	-15.367	1.00	0.00
ATOM	864	HG2	GLU	54	-4.388	0.209	-15.861	1.00	0.00
ATOM	865	HG3	GLU	54	-2.744	-0.466	-15.770	1.00	0.00
ATOM	866	CD	GLU	54	-4.317	-1.931	-15.614	1.00	0.00
ATOM	867	OE1	GLU	54	-4.634	-2.611	-14.650	1.00	0.00
ATOM	868	OE2	GLU	54	-4.421	-2.306	-16.771	1.00	0.00
ATOM	869	C	GLU	54	-4.077	2.219	-14.457	1.00	0.00
ATOM	870	O	GLU	54	-3.657	2.601	-15.550	1.00	0.00
ATOM	871	N	GLY	55	-5.216	2.654	-13.923	1.00	0.00
ATOM	872	H	GLY	55	-5.518	2.312	-13.022	1.00	0.00
ATOM	873	CA	GLY	55	-6.059	3.629	-14.613	1.00	0.00

ATOM 874 HA2 GLY 55 -5.966 3.491 -15.690 1.00 0.00
ATOM 875 HA3 GLY 55 -7.098 3.485 -14.316 1.00 0.00
ATOM 876 C GLY 55 -5.632 5.052 -14.255 1.00 0.00
ATOM 877 O GLY 55 -6.029 6.016 -14.910 1.00 0.00
ATOM 878 N MET 56 -4.816 5.166 -13.209 1.00 0.00
ATOM 879 H MET 56 -4.535 4.339 -12.701 1.00 0.00
ATOM 880 CA MET 56 -4.321 6.463 -12.751 1.00 0.00
ATOM 881 HA MET 56 -5.075 7.233 -12.911 1.00 0.00
ATOM 882 CB MET 56 -4.004 6.391 -11.250 1.00 0.00
ATOM 883 HB2 MET 56 -4.920 6.173 -10.700 1.00 0.00
ATOM 884 HB3 MET 56 -3.278 5.597 -11.075 1.00 0.00
ATOM 885 CG MET 56 -3.426 7.725 -10.770 1.00 0.00
ATOM 886 HG2 MET 56 -3.045 7.616 -9.754 1.00 0.00
ATOM 887 HG3 MET 56 -2.615 8.033 -11.430 1.00 0.00
ATOM 888 SD MET 56 -4.720 8.988 -10.787 1.00 0.00
ATOM 889 CE MET 56 -4.708 9.351 -9.016 1.00 0.00
ATOM 890 HE1 MET 56 -3.719 9.704 -8.723 1.00 0.00
ATOM 891 HE2 MET 56 -5.447 10.121 -8.797 1.00 0.00
ATOM 892 HE3 MET 56 -4.950 8.446 -8.457 1.00 0.00
ATOM 893 C MET 56 -3.066 6.858 -13.522 1.00 0.00
ATOM 894 O MET 56 -2.223 6.010 -13.824 1.00 0.00
ATOM 895 N ASP 57 -2.942 8.150 -13.835 1.00 0.00

ATOM 896 H ASP 57 -3.665 8.811 -13.588 1.00 0.00

ATOM 897 CA ASP 57 -1.775 8.640 -14.565 1.00 0.00

ATOM 898 HA ASP 57 -1.768 8.229 -15.575 1.00 0.00

ATOM 899 CB ASP 57 -1.815 10.170 -14.658 1.00 0.00

ATOM 900 HB2 ASP 57 -1.887 10.582 -13.651 1.00 0.00

ATOM 901 HB3 ASP 57 -0.896 10.520 -15.128 1.00 0.00

ATOM 902 CG ASP 57 -3.018 10.629 -15.485 1.00 0.00

ATOM 903 OD1 ASP 57 -3.583 9.812 -16.196 1.00 0.00

ATOM 904 OD2 ASP 57 -3.357 11.798 -15.395 1.00 0.00

ATOM 905 C ASP 57 -0.492 8.198 -13.865 1.00 0.00

ATOM 906 O ASP 57 -0.445 8.114 -12.638 1.00 0.00

ATOM 907 N GLU 58 0.540 7.904 -14.651 1.00 0.00

ATOM 908 H GLU 58 0.455 7.968 -15.655 1.00 0.00

ATOM 909 CA GLU 58 1.811 7.453 -14.092 1.00 0.00

ATOM 910 HA GLU 58 1.686 6.486 -13.605 1.00 0.00

ATOM 911 CB GLU 58 2.844 7.307 -15.211 1.00 0.00

ATOM 912 HB2 GLU 58 2.570 6.473 -15.857 1.00 0.00

ATOM 913 HB3 GLU 58 2.881 8.224 -15.798 1.00 0.00

ATOM 914 CG GLU 58 4.216 7.042 -14.594 1.00 0.00

ATOM 915 HG2 GLU 58 4.482 7.877 -13.946 1.00 0.00

ATOM 916 HG3 GLU 58 4.175 6.125 -14.005 1.00 0.00

ATOM 917 CD GLU 58 5.267 6.895 -15.691 1.00 0.00

ATOM	918	OE1	GLU	58	5.272	5.865	-16.347	1.00	0.00
ATOM	919	OE2	GLU	58	6.054	7.813	-15.858	1.00	0.00
ATOM	920	C	GLU	58	2.346	8.420	-13.039	1.00	0.00
ATOM	921	O	GLU	58	2.588	8.030	-11.897	1.00	0.00
ATOM	922	N	SER	59	2.533	9.677	-13.427	1.00	0.00
ATOM	923	H	SER	59	2.340	9.955	-14.378	1.00	0.00
ATOM	924	CA	SER	59	3.042	10.684	-12.501	1.00	0.00
ATOM	925	HA	SER	59	4.047	10.417	-12.171	1.00	0.00
ATOM	926	CB	SER	59	3.101	12.045	-13.197	1.00	0.00
ATOM	927	HB2	SER	59	2.095	12.357	-13.476	1.00	0.00
ATOM	928	HB3	SER	59	3.536	12.782	-12.522	1.00	0.00
ATOM	929	OG	SER	59	3.905	11.940	-14.365	1.00	0.00
ATOM	930	HG	SER	59	3.944	12.792	-14.804	1.00	0.00
ATOM	931	C	SER	59	2.145	10.773	-11.273	1.00	0.00
ATOM	932	O	SER	59	2.611	11.010	-10.158	1.00	0.00
ATOM	933	N	CYX	60	0.854	10.576	-11.501	1.00	0.00
ATOM	934	H	CYX	60	0.532	10.408	-12.443	1.00	0.00
ATOM	935	CA	CYX	60	-0.137	10.628	-10.428	1.00	0.00
ATOM	936	HA	CYX	60	0.059	11.481	-9.779	1.00	0.00
ATOM	937	CB	CYX	60	-1.540	10.775	-11.020	1.00	0.00
ATOM	938	HB2	CYX	60	-1.763	9.910	-11.644	1.00	0.00
ATOM	939	HB3	CYX	60	-2.270	10.838	-10.213	1.00	0.00

ATOM	940	SG	CYX	60	-1.619	12.287	-12.011	1.00	0.00
ATOM	941	C	CYX	60	-0.074	9.375	-9.562	1.00	0.00
ATOM	942	O	CYX	60	-0.480	9.390	-8.399	1.00	0.00
ATOM	943	N	ARG	61	0.413	8.286	-10.149	1.00	0.00
ATOM	944	H	ARG	61	0.719	8.323	-11.111	1.00	0.00
ATOM	945	CA	ARG	61	0.502	7.013	-9.444	1.00	0.00
ATOM	946	HA	ARG	61	-0.483	6.700	-9.098	1.00	0.00
ATOM	947	CB	ARG	61	1.048	5.945	-10.396	1.00	0.00
ATOM	948	HB2	ARG	61	0.496	5.987	-11.334	1.00	0.00
ATOM	949	HB3	ARG	61	2.103	6.142	-10.586	1.00	0.00
ATOM	950	CG	ARG	61	0.893	4.553	-9.775	1.00	0.00
ATOM	951	HG2	ARG	61	1.364	4.535	-8.792	1.00	0.00
ATOM	952	HG3	ARG	61	-0.165	4.312	-9.675	1.00	0.00
ATOM	953	CD	ARG	61	1.567	3.521	-10.681	1.00	0.00
ATOM	954	HD2	ARG	61	2.647	3.672	-10.680	1.00	0.00
ATOM	955	HD3	ARG	61	1.342	2.513	-10.331	1.00	0.00
ATOM	956	NE	ARG	61	1.076	3.662	-12.050	1.00	0.00
ATOM	957	HE	ARG	61	1.473	4.370	-12.651	1.00	0.00
ATOM	958	CZ	ARG	61	0.109	2.886	-12.535	1.00	0.00
ATOM	959	NH1	ARG	61	-0.406	1.933	-11.804	1.00	0.00
ATOM	960	HH11	ARG	61	-0.068	1.783	-10.864	1.00	0.00
ATOM	961	HH12	ARG	61	-1.139	1.350	-12.182	1.00	0.00

ATOM	962	NH2	ARG	61	-0.321	3.076	-13.751	1.00	0.00
ATOM	963	HH21	ARG	61	0.082	3.809	-14.317	1.00	0.00
ATOM	964	HH22	ARG	61	-1.054	2.489	-14.121	1.00	0.00
ATOM	965	C	ARG	61	1.413	7.123	-8.224	1.00	0.00
ATOM	966	O	ARG	61	1.120	6.551	-7.173	1.00	0.00
ATOM	967	N	CYX	62	2.513	7.861	-8.364	1.00	0.00
ATOM	968	H	CYX	62	2.729	8.307	-9.244	1.00	0.00
ATOM	969	CA	CYX	62	3.447	8.031	-7.255	1.00	0.00
ATOM	970	HA	CYX	62	3.868	7.066	-6.970	1.00	0.00
ATOM	971	CB	CYX	62	4.593	8.962	-7.671	1.00	0.00
ATOM	972	HB2	CYX	62	4.267	9.999	-7.590	1.00	0.00
ATOM	973	HB3	CYX	62	5.450	8.799	-7.017	1.00	0.00
ATOM	974	SG	CYX	62	5.056	8.636	-9.391	1.00	0.00
ATOM	975	C	CYX	62	2.722	8.621	-6.047	1.00	0.00
ATOM	976	O	CYX	62	2.680	8.018	-4.974	1.00	0.00
ATOM	977	N	GLU	63	2.142	9.803	-6.245	1.00	0.00
ATOM	978	H	GLU	63	2.217	10.254	-7.145	1.00	0.00
ATOM	979	CA	GLU	63	1.401	10.485	-5.189	1.00	0.00
ATOM	980	HA	GLU	63	2.056	10.694	-4.342	1.00	0.00
ATOM	981	CB	GLU	63	0.853	11.811	-5.724	1.00	0.00
ATOM	982	HB2	GLU	63	1.661	12.368	-6.199	1.00	0.00
ATOM	983	HB3	GLU	63	0.072	11.608	-6.456	1.00	0.00

ATOM	984	CG	GLU	63	0.269	12.640	-4.570	1.00	0.00
ATOM	985	HG2	GLU	63	-0.452	12.030	-4.025	1.00	0.00
ATOM	986	HG3	GLU	63	1.079	12.930	-3.900	1.00	0.00
ATOM	987	CD	GLU	63	-0.425	13.895	-5.104	1.00	0.00
ATOM	988	OE1	GLU	63	-0.521	14.038	-6.314	1.00	0.00
ATOM	989	OE2	GLU	63	-0.854	14.698	-4.291	1.00	0.00
ATOM	990	C	GLU	63	0.239	9.626	-4.695	1.00	0.00
ATOM	991	O	GLU	63	-0.091	9.629	-3.509	1.00	0.00
ATOM	992	N	GLY	64	-0.394	8.915	-5.629	1.00	0.00
ATOM	993	H	GLY	64	-0.080	8.949	-6.588	1.00	0.00
ATOM	994	CA	GLY	64	-1.545	8.075	-5.306	1.00	0.00
ATOM	995	HA2	GLY	64	-2.377	8.720	-5.026	1.00	0.00
ATOM	996	HA3	GLY	64	-1.813	7.499	-6.191	1.00	0.00
ATOM	997	C	GLY	64	-1.255	7.114	-4.159	1.00	0.00
ATOM	998	O	GLY	64	-1.942	7.138	-3.138	1.00	0.00
ATOM	999	N	LEU	65	-0.248	6.264	-4.333	1.00	0.00
ATOM	1000	H	LEU	65	0.274	6.252	-5.197	1.00	0.00
ATOM	1001	CA	LEU	65	0.102	5.294	-3.300	1.00	0.00
ATOM	1002	HA	LEU	65	-0.734	4.616	-3.127	1.00	0.00
ATOM	1003	CB	LEU	65	1.317	4.470	-3.739	1.00	0.00
ATOM	1004	HB2	LEU	65	2.147	5.140	-3.962	1.00	0.00
ATOM	1005	HB3	LEU	65	1.605	3.791	-2.936	1.00	0.00

ATOM 1006 CG LEU 65 0.964 3.659 -4.991 1.00 0.00

ATOM 1007 HG LEU 65 0.735 4.339 -5.811 1.00 0.00

ATOM 1008 CD1 LEU 65 2.151 2.774 -5.379 1.00 0.00

ATOM 1009 HD11 LEU 65 2.381 2.093 -4.559 1.00 0.00

ATOM 1010 HD12 LEU 65 1.900 2.197 -6.269 1.00 0.00

ATOM 1011 HD13 LEU 65 3.020 3.399 -5.585 1.00 0.00

ATOM 1012 CD2 LEU 65 -0.259 2.777 -4.703 1.00 0.00

ATOM 1013 HD21 LEU 65 -1.105 3.406 -4.428 1.00 0.00

ATOM 1014 HD22 LEU 65 -0.509 2.200 -5.593 1.00 0.00

ATOM 1015 HD23 LEU 65 -0.031 2.096 -3.882 1.00 0.00

ATOM 1016 C LEU 65 0.397 5.977 -1.969 1.00 0.00

ATOM 1017 O LEU 65 -0.078 5.537 -0.924 1.00 0.00

ATOM 1018 N ARG 66 1.179 7.054 -2.010 1.00 0.00

ATOM 1019 H ARG 66 1.567 7.381 -2.883 1.00 0.00

ATOM 1020 CA ARG 66 1.517 7.777 -0.788 1.00 0.00

ATOM 1021 HA ARG 66 2.062 7.124 -0.105 1.00 0.00

ATOM 1022 CB ARG 66 2.401 8.983 -1.121 1.00 0.00

ATOM 1023 HB2 ARG 66 1.900 9.606 -1.862 1.00 0.00

ATOM 1024 HB3 ARG 66 2.576 9.565 -0.216 1.00 0.00

ATOM 1025 CG ARG 66 3.741 8.499 -1.683 1.00 0.00

ATOM 1026 HG2 ARG 66 4.222 7.840 -0.959 1.00 0.00

ATOM 1027 HG3 ARG 66 3.570 7.954 -2.611 1.00 0.00

ATOM	1028	CD	ARG	66	4.647	9.703	-1.957	1.00	0.00
ATOM	1029	HD2	ARG	66	5.544	9.378	-2.486	1.00	0.00
ATOM	1030	HD3	ARG	66	4.115	10.436	-2.562	1.00	0.00
ATOM	1031	NE	ARG	66	5.047	10.334	-0.701	1.00	0.00
ATOM	1032	HE	ARG	66	4.586	11.177	-0.391	1.00	0.00
ATOM	1033	CZ	ARG	66	6.015	9.823	0.059	1.00	0.00
ATOM	1034	NH1	ARG	66	6.635	8.734	-0.310	1.00	0.00
ATOM	1035	HH11	ARG	66	6.379	8.279	-1.174	1.00	0.00
ATOM	1036	HH12	ARG	66	7.368	8.352	0.271	1.00	0.00
ATOM	1037	NH2	ARG	66	6.345	10.412	1.174	1.00	0.00
ATOM	1038	HH21	ARG	66	5.866	11.254	1.458	1.00	0.00
ATOM	1039	HH22	ARG	66	7.079	10.023	1.749	1.00	0.00
ATOM	1040	C	ARG	66	0.253	8.253	-0.076	1.00	0.00
ATOM	1041	O	ARG	66	0.053	7.977	1.108	1.00	0.00
ATOM	1042	N	MET	67	-0.596	8.966	-0.812	1.00	0.00
ATOM	1043	H	MET	67	-0.373	9.186	-1.772	1.00	0.00
ATOM	1044	CA	MET	67	-1.845	9.479	-0.256	1.00	0.00
ATOM	1045	HA	MET	67	-1.641	10.104	0.614	1.00	0.00
ATOM	1046	CB	MET	67	-2.571	10.325	-1.308	1.00	0.00
ATOM	1047	HB2	MET	67	-2.061	11.282	-1.418	1.00	0.00
ATOM	1048	HB3	MET	67	-2.565	9.799	-2.263	1.00	0.00
ATOM	1049	CG	MET	67	-4.019	10.565	-0.866	1.00	0.00

ATOM	1050	HG2	MET	67	-4.575	9.629	-0.910	1.00	0.00
ATOM	1051	HG3	MET	67	-4.032	10.946	0.155	1.00	0.00
ATOM	1052	SD	MET	67	-4.801	11.776	-1.964	1.00	0.00
ATOM	1053	CE	MET	67	-3.818	13.214	-1.468	1.00	0.00
ATOM	1054	HE1	MET	67	-2.764	13.021	-1.669	1.00	0.00
ATOM	1055	HE2	MET	67	-4.141	14.088	-2.033	1.00	0.00
ATOM	1056	HE3	MET	67	-3.956	13.399	-0.403	1.00	0.00
ATOM	1057	C	MET	67	-2.758	8.348	0.210	1.00	0.00
ATOM	1058	O	MET	67	-3.296	8.386	1.317	1.00	0.00
ATOM	1059	N	MET	68	-2.945	7.358	-0.656	1.00	0.00
ATOM	1060	H	MET	68	-2.510	7.389	-1.567	1.00	0.00
ATOM	1061	CA	MET	68	-3.817	6.231	-0.345	1.00	0.00
ATOM	1062	HA	MET	68	-4.815	6.583	-0.087	1.00	0.00
ATOM	1063	CB	MET	68	-3.921	5.319	-1.571	1.00	0.00
ATOM	1064	HB2	MET	68	-4.626	5.752	-2.280	1.00	0.00
ATOM	1065	HB3	MET	68	-2.940	5.236	-2.039	1.00	0.00
ATOM	1066	CG	MET	68	-4.407	3.926	-1.154	1.00	0.00
ATOM	1067	HG2	MET	68	-3.616	3.407	-0.611	1.00	0.00
ATOM	1068	HG3	MET	68	-5.285	4.018	-0.515	1.00	0.00
ATOM	1069	SD	MET	68	-4.842	2.972	-2.627	1.00	0.00
ATOM	1070	CE	MET	68	-3.165	2.776	-3.275	1.00	0.00
ATOM	1071	HE1	MET	68	-2.552	2.248	-2.544	1.00	0.00

ATOM 1072 HE2 MET 68 -3.197 2.204 -4.202 1.00 0.00
ATOM 1073 HE3 MET 68 -2.733 3.757 -3.469 1.00 0.00
ATOM 1074 C MET 68 -3.314 5.443 0.861 1.00 0.00
ATOM 1075 O MET 68 -4.090 5.122 1.759 1.00 0.00
ATOM 1076 N MET 69 -2.021 5.130 0.882 1.00 0.00
ATOM 1077 H MET 69 -1.409 5.391 0.122 1.00 0.00
ATOM 1078 CA MET 69 -1.456 4.374 1.995 1.00 0.00
ATOM 1079 HA MET 69 -1.853 3.359 1.998 1.00 0.00
ATOM 1080 CB MET 69 0.069 4.303 1.859 1.00 0.00
ATOM 1081 HB2 MET 69 0.326 3.908 0.876 1.00 0.00
ATOM 1082 HB3 MET 69 0.489 5.303 1.969 1.00 0.00
ATOM 1083 CG MET 69 0.643 3.387 2.943 1.00 0.00
ATOM 1084 HG2 MET 69 0.339 3.744 3.927 1.00 0.00
ATOM 1085 HG3 MET 69 0.278 2.370 2.798 1.00 0.00
ATOM 1086 SD MET 69 2.450 3.390 2.841 1.00 0.00
ATOM 1087 CE MET 69 2.755 2.159 4.132 1.00 0.00
ATOM 1088 HE1 MET 69 2.277 1.218 3.858 1.00 0.00
ATOM 1089 HE2 MET 69 3.829 2.002 4.240 1.00 0.00
ATOM 1090 HE3 MET 69 2.344 2.513 5.077 1.00 0.00
ATOM 1091 C MET 69 -1.826 5.029 3.323 1.00 0.00
ATOM 1092 O MET 69 -2.384 4.385 4.212 1.00 0.00
ATOM 1093 N ARG 70 -1.516 6.316 3.441 1.00 0.00

```
ATOM   1094  H   ARG    70      -1.043   6.794   2.688  1.00  0.00
ATOM   1095  CA  ARG    70      -1.821   7.067   4.654  1.00  0.00
ATOM   1096  HA  ARG    70      -1.344   6.601   5.517  1.00  0.00
ATOM   1097  CB  ARG    70      -1.292   8.498   4.516  1.00  0.00
ATOM   1098  HB2 ARG    70      -1.731   8.965   3.635  1.00  0.00
ATOM   1099  HB3 ARG    70      -1.560   9.072   5.403  1.00  0.00
ATOM   1100  CG  ARG    70       0.233   8.466   4.371  1.00  0.00
ATOM   1101  HG2 ARG    70       0.671   7.993   5.250  1.00  0.00
ATOM   1102  HG3 ARG    70       0.501   7.895   3.482  1.00  0.00
ATOM   1103  CD  ARG    70       0.768   9.894   4.241  1.00  0.00
ATOM   1104  HD2 ARG    70       0.200  10.437   3.486  1.00  0.00
ATOM   1105  HD3 ARG    70       0.687  10.410   5.198  1.00  0.00
ATOM   1106  NE  ARG    70       2.172   9.869   3.841  1.00  0.00
ATOM   1107  HE  ARG    70       2.748   9.071   4.070  1.00  0.00
ATOM   1108  CZ  ARG    70       2.724  10.882   3.173  1.00  0.00
ATOM   1109  NH1 ARG    70       2.015  11.937   2.869  1.00  0.00
ATOM   1110 HH11 ARG    70       1.044  11.984   3.142  1.00  0.00
ATOM   1111 HH12 ARG    70       2.442  12.699   2.363  1.00  0.00
ATOM   1112  NH2 ARG    70       3.979  10.821   2.821  1.00  0.00
ATOM   1113 HH21 ARG    70       4.528  10.006   3.057  1.00  0.00
ATOM   1114 HH22 ARG    70       4.397  11.589   2.315  1.00  0.00
ATOM   1115  C   ARG    70      -3.327   7.102   4.914  1.00  0.00
```

```
ATOM   1116  O   ARG    70      -3.773   6.950   6.051  1.00  0.00
ATOM   1117  N   MET    71      -4.101   7.311   3.848  1.00  0.00
ATOM   1118  H   MET    71      -3.681   7.453   2.941  1.00  0.00
ATOM   1119  CA  MET    71      -5.553   7.376   3.956  1.00  0.00
ATOM   1120  HA  MET    71      -5.840   8.123   4.696  1.00  0.00
ATOM   1121  CB  MET    71      -6.159   7.767   2.605  1.00  0.00
ATOM   1122  HB2 MET    71      -5.643   8.646   2.218  1.00  0.00
ATOM   1123  HB3 MET    71      -6.043   6.940   1.904  1.00  0.00
ATOM   1124  CG  MET    71      -7.648   8.084   2.780  1.00  0.00
ATOM   1125  HG2 MET    71      -8.151   7.238   3.249  1.00  0.00
ATOM   1126  HG3 MET    71      -7.764   8.968   3.407  1.00  0.00
ATOM   1127  SD  MET    71      -8.396   8.400   1.161  1.00  0.00
ATOM   1128  CE  MET    71      -7.459   9.895   0.754  1.00  0.00
ATOM   1129  HE1 MET    71      -6.394   9.662   0.733  1.00  0.00
ATOM   1130  HE2 MET    71      -7.769  10.264  -0.223  1.00  0.00
ATOM   1131  HE3 MET    71      -7.648  10.659   1.508  1.00  0.00
ATOM   1132  C   MET    71      -6.124   6.040   4.411  1.00  0.00
ATOM   1133  O   MET    71      -7.072   5.996   5.195  1.00  0.00
ATOM   1134  N   MET    72      -5.551   4.954   3.904  1.00  0.00
ATOM   1135  H   MET    72      -4.789   5.030   3.245  1.00  0.00
ATOM   1136  CA  MET    72      -6.025   3.625   4.260  1.00  0.00
ATOM   1137  HA  MET    72      -7.060   3.500   3.943  1.00  0.00
```

ATOM	1138	CB	MET	72	-5.169	2.559	3.568	1.00	0.00
ATOM	1139	HB2	MET	72	-4.118	2.840	3.639	1.00	0.00
ATOM	1140	HB3	MET	72	-5.322	1.599	4.062	1.00	0.00
ATOM	1141	CG	MET	72	-5.568	2.444	2.094	1.00	0.00
ATOM	1142	HG2	MET	72	-5.539	3.428	1.627	1.00	0.00
ATOM	1143	HG3	MET	72	-4.878	1.776	1.577	1.00	0.00
ATOM	1144	SD	MET	72	-7.247	1.776	1.972	1.00	0.00
ATOM	1145	CE	MET	72	-6.903	0.152	2.697	1.00	0.00
ATOM	1146	HE1	MET	72	-6.527	0.279	3.713	1.00	0.00
ATOM	1147	HE2	MET	72	-7.819	-0.438	2.720	1.00	0.00
ATOM	1148	HE3	MET	72	-6.154	-0.364	2.096	1.00	0.00
ATOM	1149	C	MET	72	-5.978	3.417	5.768	1.00	0.00
ATOM	1150	O	MET	72	-6.993	3.101	6.392	1.00	0.00
ATOM	1151	N	GLN	73	-4.796	3.603	6.346	1.00	0.00
ATOM	1152	H	GLN	73	-3.986	3.839	5.790	1.00	0.00
ATOM	1153	CA	GLN	73	-4.623	3.442	7.784	1.00	0.00
ATOM	1154	HA	GLN	73	-4.912	2.434	8.084	1.00	0.00
ATOM	1155	CB	GLN	73	-3.157	3.664	8.165	1.00	0.00
ATOM	1156	HB2	GLN	73	-2.863	4.678	7.896	1.00	0.00
ATOM	1157	HB3	GLN	73	-3.038	3.525	9.240	1.00	0.00
ATOM	1158	CG	GLN	73	-2.271	2.662	7.420	1.00	0.00
ATOM	1159	HG2	GLN	73	-2.256	2.879	6.352	1.00	0.00

```
ATOM   1160  HG3 GLN    73      -1.256   2.714   7.815  1.00  0.00
ATOM   1161  CD  GLN    73      -2.799   1.248   7.622  1.00  0.00
ATOM   1162  OE1 GLN    73      -2.763   0.721   8.734  1.00  0.00
ATOM   1163  NE2 GLN    73      -3.306   0.607   6.608  1.00  0.00
ATOM   1164 HE21 GLN    73      -3.658  -0.332   6.733  1.00  0.00
ATOM   1165 HE22 GLN    73      -3.344   1.051   5.702  1.00  0.00
ATOM   1166  C   GLN    73      -5.508   4.424   8.545  1.00  0.00
ATOM   1167  O   GLN    73      -6.103   4.080   9.562  1.00  0.00
ATOM   1168  N   GLN    74      -5.580   5.651   8.042  1.00  0.00
ATOM   1169  H   GLN    74      -5.045   5.899   7.222  1.00  0.00
ATOM   1170  CA  GLN    74      -6.385   6.685   8.681  1.00  0.00
ATOM   1171  HA  GLN    74      -6.075   6.816   9.718  1.00  0.00
ATOM   1172  CB  GLN    74      -6.198   8.014   7.942  1.00  0.00
ATOM   1173  HB2 GLN    74      -5.261   8.475   8.255  1.00  0.00
ATOM   1174  HB3 GLN    74      -6.171   7.831   6.868  1.00  0.00
ATOM   1175  CG  GLN    74      -7.362   8.951   8.271  1.00  0.00
ATOM   1176  HG2 GLN    74      -7.904   8.588   9.145  1.00  0.00
ATOM   1177  HG3 GLN    74      -6.986   9.955   8.469  1.00  0.00
ATOM   1178  CD  GLN    74      -8.325   9.015   7.092  1.00  0.00
ATOM   1179  OE1 GLN    74      -8.355   8.029   6.241  1.00  0.00
ATOM   1180  NE2 GLN    74      -9.070   9.984   6.946  1.00  0.00
ATOM   1181 HE21 GLN    74      -9.704  10.015   6.161  1.00  0.00
```

ATOM	1182	HE22	GLN	74	-9.043	10.742	7.613	1.00	0.00
ATOM	1183	C	GLN	74	-7.865	6.307	8.717	1.00	0.00
ATOM	1184	O	GLN	74	-8.535	6.498	9.733	1.00	0.00
ATOM	1185	N	LYS	75	-8.370	5.785	7.606	1.00	0.00
ATOM	1186	H	LYS	75	-7.791	5.656	6.788	1.00	0.00
ATOM	1187	CA	LYS	75	-9.777	5.407	7.525	1.00	0.00
ATOM	1188	HA	LYS	75	-10.411	6.278	7.690	1.00	0.00
ATOM	1189	CB	LYS	75	-10.077	4.838	6.135	1.00	0.00
ATOM	1190	HB2	LYS	75	-9.745	5.547	5.377	1.00	0.00
ATOM	1191	HB3	LYS	75	-9.543	3.896	6.009	1.00	0.00
ATOM	1192	CG	LYS	75	-11.584	4.596	5.984	1.00	0.00
ATOM	1193	HG2	LYS	75	-11.760	3.939	5.132	1.00	0.00
ATOM	1194	HG3	LYS	75	-11.966	4.125	6.890	1.00	0.00
ATOM	1195	CD	LYS	75	-12.305	5.929	5.758	1.00	0.00
ATOM	1196	HD2	LYS	75	-12.187	6.559	6.640	1.00	0.00
ATOM	1197	HD3	LYS	75	-11.875	6.432	4.892	1.00	0.00
ATOM	1198	CE	LYS	75	-13.793	5.671	5.511	1.00	0.00
ATOM	1199	HE2	LYS	75	-14.182	5.017	6.291	1.00	0.00
ATOM	1200	HE3	LYS	75	-14.333	6.617	5.528	1.00	0.00
ATOM	1201	NZ	LYS	75	-13.972	5.020	4.182	1.00	0.00
ATOM	1202	HZ1	LYS	75	-13.471	4.142	4.167	1.00	0.00
ATOM	1203	HZ2	LYS	75	-14.954	4.850	4.020	1.00	0.00

ATOM	1204	HZ3	LYS	75	-13.611	5.626	3.459	1.00	0.00
ATOM	1205	C	LYS	75	-10.125	4.370	8.589	1.00	0.00
ATOM	1206	O	LYS	75	-11.180	4.444	9.221	1.00	0.00
ATOM	1207	N	GLU	76	-9.231	3.409	8.776	1.00	0.00
ATOM	1208	H	GLU	76	-8.389	3.376	8.218	1.00	0.00
ATOM	1209	CA	GLU	76	-9.448	2.354	9.765	1.00	0.00
ATOM	1210	HA	GLU	76	-9.762	2.788	10.714	1.00	0.00
ATOM	1211	CB	GLU	76	-10.545	1.398	9.276	1.00	0.00
ATOM	1212	HB2	GLU	76	-11.416	1.976	8.969	1.00	0.00
ATOM	1213	HB3	GLU	76	-10.172	0.825	8.427	1.00	0.00
ATOM	1214	CG	GLU	76	-10.941	0.437	10.407	1.00	0.00
ATOM	1215	HG2	GLU	76	-11.605	-0.328	10.004	1.00	0.00
ATOM	1216	HG3	GLU	76	-10.041	-0.035	10.802	1.00	0.00
ATOM	1217	CD	GLU	76	-11.655	1.191	11.530	1.00	0.00
ATOM	1218	OE1	GLU	76	-12.273	2.207	11.247	1.00	0.00
ATOM	1219	OE2	GLU	76	-11.573	0.741	12.661	1.00	0.00
ATOM	1220	C	GLU	76	-8.157	1.578	10.017	1.00	0.00
ATOM	1221	O	GLU	76	-8.000	0.451	9.543	1.00	0.00
ATOM	1222	N	MET	77	-7.232	2.187	10.752	1.00	0.00
ATOM	1223	H	MET	77	-7.387	3.122	11.100	1.00	0.00
ATOM	1224	CA	MET	77	-5.956	1.541	11.050	1.00	0.00
ATOM	1225	HA	MET	77	-5.404	1.360	10.128	1.00	0.00

ATOM	1226	CB	MET	77	-5.111	2.443	11.958	1.00	0.00
ATOM	1227	HB2	MET	77	-5.045	3.438	11.519	1.00	0.00
ATOM	1228	HB3	MET	77	-5.580	2.510	12.940	1.00	0.00
ATOM	1229	CG	MET	77	-3.704	1.856	12.105	1.00	0.00
ATOM	1230	HG2	MET	77	-3.767	0.837	12.487	1.00	0.00
ATOM	1231	HG3	MET	77	-3.204	1.849	11.137	1.00	0.00
ATOM	1232	SD	MET	77	-2.747	2.866	13.261	1.00	0.00
ATOM	1233	CE	MET	77	-1.178	1.980	13.095	1.00	0.00
ATOM	1234	HE1	MET	77	-0.845	2.021	12.058	1.00	0.00
ATOM	1235	HE2	MET	77	-0.428	2.444	13.736	1.00	0.00
ATOM	1236	HE3	MET	77	-1.314	0.940	13.392	1.00	0.00
ATOM	1237	C	MET	77	-6.174	0.188	11.727	1.00	0.00
ATOM	1238	O	MET	77	-6.287	0.112	12.952	1.00	0.00
ATOM	1239	N	GLN	78	-6.218	-0.876	10.928	1.00	0.00
ATOM	1240	H	GLN	78	-6.137	-0.771	9.927	1.00	0.00
ATOM	1241	CA	GLN	78	-6.403	-2.219	11.470	1.00	0.00
ATOM	1242	HA	GLN	78	-7.389	-2.305	11.927	1.00	0.00
ATOM	1243	CB	GLN	78	-6.290	-3.260	10.349	1.00	0.00
ATOM	1244	HB2	GLN	78	-5.866	-2.791	9.461	1.00	0.00
ATOM	1245	HB3	GLN	78	-5.641	-4.074	10.675	1.00	0.00
ATOM	1246	CG	GLN	78	-7.679	-3.816	10.019	1.00	0.00
ATOM	1247	HG2	GLN	78	-8.126	-4.256	10.911	1.00	0.00

```
ATOM   1248  HG3 GLN    78      -8.321  -3.018   9.648  1.00  0.00
ATOM   1249  CD  GLN    78      -7.570  -4.895   8.944  1.00  0.00
ATOM   1250  OE1 GLN    78      -6.758  -5.813   9.063  1.00  0.00
ATOM   1251  NE2 GLN    78      -8.345  -4.839   7.895  1.00  0.00
ATOM   1252 HE21 GLN    78      -8.276  -5.551   7.182  1.00  0.00
ATOM   1253 HE22 GLN    78      -9.009  -4.085   7.802  1.00  0.00
ATOM   1254  C   GLN    78      -5.344  -2.491  12.539  1.00  0.00
ATOM   1255  O   GLN    78      -4.165  -2.201  12.328  1.00  0.00
ATOM   1256  N   PRO    79      -5.724  -3.031  13.671  1.00  0.00
ATOM   1257  CD  PRO    79      -7.087  -3.426  14.045  1.00  0.00
ATOM   1258  HD2 PRO    79      -7.365  -4.323  13.491  1.00  0.00
ATOM   1259  HD3 PRO    79      -7.775  -2.618  13.798  1.00  0.00
ATOM   1260  CG  PRO    79      -6.993  -3.673  15.559  1.00  0.00
ATOM   1261  HG2 PRO    79      -7.106  -4.740  15.752  1.00  0.00
ATOM   1262  HG3 PRO    79      -7.793  -3.127  16.058  1.00  0.00
ATOM   1263  CB  PRO    79      -5.655  -3.178  16.000  1.00  0.00
ATOM   1264  HB2 PRO    79      -5.297  -3.838  16.791  1.00  0.00
ATOM   1265  HB3 PRO    79      -5.633  -2.147  16.351  1.00  0.00
ATOM   1266  CA  PRO    79      -4.776  -3.325  14.767  1.00  0.00
ATOM   1267  HA  PRO    79      -3.955  -2.610  14.716  1.00  0.00
ATOM   1268  C   PRO    79      -4.223  -4.738  14.662  1.00  0.00
ATOM   1269  O   PRO    79      -4.943  -5.720  14.851  1.00  0.00
```

```
ATOM   1270  N   ARG    80      -2.941  -4.820  14.372  1.00  0.00
ATOM   1271  H   ARG    80      -2.406  -3.979  14.208  1.00  0.00
ATOM   1272  CA  ARG    80      -2.265  -6.102  14.250  1.00  0.00
ATOM   1273  HA  ARG    80      -2.388  -6.673  15.171  1.00  0.00
ATOM   1274  CB  ARG    80      -2.852  -6.910  13.091  1.00  0.00
ATOM   1275  HB2 ARG    80      -3.556  -6.292  12.535  1.00  0.00
ATOM   1276  HB3 ARG    80      -2.049  -7.233  12.428  1.00  0.00
ATOM   1277  CG  ARG    80      -3.579  -8.138  13.646  1.00  0.00
ATOM   1278  HG2 ARG    80      -2.865  -8.950  13.783  1.00  0.00
ATOM   1279  HG3 ARG    80      -4.032  -7.888  14.606  1.00  0.00
ATOM   1280  CD  ARG    80      -4.668  -8.577  12.668  1.00  0.00
ATOM   1281  HD2 ARG    80      -4.511  -8.101  11.700  1.00  0.00
ATOM   1282  HD3 ARG    80      -4.643  -9.660  12.548  1.00  0.00
ATOM   1283  NE  ARG    80      -5.985  -8.191  13.172  1.00  0.00
ATOM   1284  HE  ARG    80      -6.453  -8.778  13.849  1.00  0.00
ATOM   1285  CZ  ARG    80      -6.596  -7.079  12.763  1.00  0.00
ATOM   1286  NH1 ARG    80      -6.020  -6.287  11.899  1.00  0.00
ATOM   1287 HH11 ARG    80      -5.105  -6.519  11.538  1.00  0.00
ATOM   1288 HH12 ARG    80      -6.491  -5.447  11.596  1.00  0.00
ATOM   1289  NH2 ARG    80      -7.776  -6.780  13.230  1.00  0.00
ATOM   1290 HH21 ARG    80      -8.221  -7.392  13.899  1.00  0.00
ATOM   1291 HH22 ARG    80      -8.239  -5.938  12.921  1.00  0.00
```

```
ATOM   1292  C   ARG    80      -0.775  -5.883  14.024  1.00  0.00
ATOM   1293  O   ARG    80      -0.386  -5.135  13.126  1.00  0.00
ATOM   1294  N   GLY    81       0.057  -6.526  14.836  1.00  0.00
ATOM   1295  H   GLY    81      -0.284  -7.136  15.566  1.00  0.00
ATOM   1296  CA  GLY    81       1.496  -6.364  14.690  1.00  0.00
ATOM   1297  HA2 GLY    81       1.699  -5.368  14.298  1.00  0.00
ATOM   1298  HA3 GLY    81       1.962  -6.470  15.670  1.00  0.00
ATOM   1299  C   GLY    81       2.092  -7.402  13.742  1.00  0.00
ATOM   1300  O   GLY    81       2.470  -7.079  12.619  1.00  0.00
ATOM   1301  N   GLU    82       2.186  -8.642  14.212  1.00  0.00
ATOM   1302  H   GLU    82       1.886  -8.849  15.154  1.00  0.00
ATOM   1303  CA  GLU    82       2.761  -9.726  13.414  1.00  0.00
ATOM   1304  HA  GLU    82       3.768  -9.462  13.090  1.00  0.00
ATOM   1305  CB  GLU    82       2.837 -11.003  14.257  1.00  0.00
ATOM   1306  HB2 GLU    82       1.853 -11.221  14.672  1.00  0.00
ATOM   1307  HB3 GLU    82       3.157 -11.833  13.627  1.00  0.00
ATOM   1308  CG  GLU    82       3.844 -10.812  15.401  1.00  0.00
ATOM   1309  HG2 GLU    82       4.136 -11.792  15.779  1.00  0.00
ATOM   1310  HG3 GLU    82       4.723 -10.297  15.013  1.00  0.00
ATOM   1311  CD  GLU    82       3.230  -9.989  16.537  1.00  0.00
ATOM   1312  OE1 GLU    82       2.017 -10.010  16.682  1.00  0.00
ATOM   1313  OE2 GLU    82       3.986  -9.347  17.248  1.00  0.00
```

ATOM	1314	C	GLU	82	1.970	-10.014	12.136	1.00	0.00
ATOM	1315	O	GLU	82	2.558	-10.216	11.073	1.00	0.00
ATOM	1316	N	GLN	83	0.644	-10.073	12.246	1.00	0.00
ATOM	1317	H	GLN	83	0.195	-9.930	13.139	1.00	0.00
ATOM	1318	CA	GLN	83	-0.196	-10.386	11.089	1.00	0.00
ATOM	1319	HA	GLN	83	0.073	-11.361	10.681	1.00	0.00
ATOM	1320	CB	GLN	83	-1.668	-10.422	11.516	1.00	0.00
ATOM	1321	HB2	GLN	83	-1.778	-11.088	12.372	1.00	0.00
ATOM	1322	HB3	GLN	83	-1.987	-9.418	11.795	1.00	0.00
ATOM	1323	CG	GLN	83	-2.534	-10.930	10.359	1.00	0.00
ATOM	1324	HG2	GLN	83	-3.587	-10.751	10.575	1.00	0.00
ATOM	1325	HG3	GLN	83	-2.260	-10.417	9.437	1.00	0.00
ATOM	1326	CD	GLN	83	-2.324	-12.429	10.164	1.00	0.00
ATOM	1327	OE1	GLN	83	-2.719	-13.227	11.013	1.00	0.00
ATOM	1328	NE2	GLN	83	-1.723	-12.860	9.089	1.00	0.00
ATOM	1329	HE21	GLN	83	-1.582	-13.852	8.956	1.00	0.00
ATOM	1330	HE22	GLN	83	-1.401	-12.201	8.395	1.00	0.00
ATOM	1331	C	GLN	83	-0.018	-9.373	9.955	1.00	0.00
ATOM	1332	O	GLN	83	0.455	-9.719	8.873	1.00	0.00
ATOM	1333	N	MET	84	-0.410	-8.128	10.210	1.00	0.00
ATOM	1334	H	MET	84	-0.852	-7.906	11.091	1.00	0.00
ATOM	1335	CA	MET	84	-0.300	-7.074	9.204	1.00	0.00

ATOM 1336 HA MET 84 -0.794 -7.386 8.284 1.00 0.00

ATOM 1337 CB MET 84 -0.965 -5.792 9.705 1.00 0.00

ATOM 1338 HB2 MET 84 -0.704 -5.630 10.751 1.00 0.00

ATOM 1339 HB3 MET 84 -0.622 -4.946 9.110 1.00 0.00

ATOM 1340 CG MET 84 -2.489 -5.926 9.573 1.00 0.00

ATOM 1341 HG2 MET 84 -2.756 -6.092 8.530 1.00 0.00

ATOM 1342 HG3 MET 84 -2.839 -6.765 10.175 1.00 0.00

ATOM 1343 SD MET 84 -3.279 -4.405 10.155 1.00 0.00

ATOM 1344 CE MET 84 -2.799 -3.343 8.770 1.00 0.00

ATOM 1345 HE1 MET 84 -3.204 -3.749 7.843 1.00 0.00

ATOM 1346 HE2 MET 84 -3.191 -2.339 8.929 1.00 0.00

ATOM 1347 HE3 MET 84 -1.712 -3.302 8.703 1.00 0.00

ATOM 1348 C MET 84 1.149 -6.811 8.796 1.00 0.00

ATOM 1349 O MET 84 1.407 -6.414 7.661 1.00 0.00

ATOM 1350 N ARG 85 2.096 -7.026 9.710 1.00 0.00

ATOM 1351 H ARG 85 1.868 -7.332 10.645 1.00 0.00

ATOM 1352 CA ARG 85 3.503 -6.794 9.380 1.00 0.00

ATOM 1353 HA ARG 85 3.678 -5.729 9.231 1.00 0.00

ATOM 1354 CB ARG 85 4.406 -7.283 10.518 1.00 0.00

ATOM 1355 HB2 ARG 85 4.175 -6.726 11.426 1.00 0.00

ATOM 1356 HB3 ARG 85 4.227 -8.345 10.689 1.00 0.00

ATOM 1357 CG ARG 85 5.877 -7.067 10.148 1.00 0.00

ATOM 1358 HG2 ARG 85 6.132 -7.685 9.287 1.00 0.00
ATOM 1359 HG3 ARG 85 6.042 -6.018 9.904 1.00 0.00
ATOM 1360 CD ARG 85 6.760 -7.459 11.335 1.00 0.00
ATOM 1361 HD2 ARG 85 7.799 -7.204 11.126 1.00 0.00
ATOM 1362 HD3 ARG 85 6.431 -6.935 12.233 1.00 0.00
ATOM 1363 NE ARG 85 6.671 -8.898 11.574 1.00 0.00
ATOM 1364 HE ARG 85 6.684 -9.538 10.793 1.00 0.00
ATOM 1365 CZ ARG 85 6.568 -9.400 12.805 1.00 0.00
ATOM 1366 NH1 ARG 85 6.557 -8.610 13.846 1.00 0.00
ATOM 1367 HH11 ARG 85 6.627 -7.611 13.719 1.00 0.00
ATOM 1368 HH12 ARG 85 6.479 -9.003 14.774 1.00 0.00
ATOM 1369 NH2 ARG 85 6.482 -10.691 12.972 1.00 0.00
ATOM 1370 HH21 ARG 85 6.494 -11.303 12.169 1.00 0.00
ATOM 1371 HH22 ARG 85 6.404 -11.073 13.904 1.00 0.00
ATOM 1372 C ARG 85 3.859 -7.524 8.088 1.00 0.00
ATOM 1373 O ARG 85 4.483 -6.953 7.194 1.00 0.00
ATOM 1374 N ARG 86 3.432 -8.780 7.990 1.00 0.00
ATOM 1375 H ARG 86 2.939 -9.215 8.757 1.00 0.00
ATOM 1376 CA ARG 86 3.683 -9.572 6.792 1.00 0.00
ATOM 1377 HA ARG 86 4.755 -9.641 6.606 1.00 0.00
ATOM 1378 CB ARG 86 3.127 -10.986 6.979 1.00 0.00
ATOM 1379 HB2 ARG 86 2.063 -10.932 7.207 1.00 0.00

ATOM	1380	HB3	ARG	86	3.274	-11.559	6.064	1.00	0.00
ATOM	1381	CG	ARG	86	3.862	-11.673	8.134	1.00	0.00
ATOM	1382	HG2	ARG	86	4.931	-11.691	7.921	1.00	0.00
ATOM	1383	HG3	ARG	86	3.685	-11.118	9.055	1.00	0.00
ATOM	1384	CD	ARG	86	3.351	-13.105	8.297	1.00	0.00
ATOM	1385	HD2	ARG	86	2.263	-13.107	8.351	1.00	0.00
ATOM	1386	HD3	ARG	86	3.674	-13.713	7.452	1.00	0.00
ATOM	1387	NE	ARG	86	3.881	-13.692	9.524	1.00	0.00
ATOM	1388	HE	ARG	86	4.609	-13.216	10.038	1.00	0.00
ATOM	1389	CZ	ARG	86	3.425	-14.851	9.994	1.00	0.00
ATOM	1390	NH1	ARG	86	2.488	-15.498	9.354	1.00	0.00
ATOM	1391	HH11	ARG	86	2.109	-15.117	8.499	1.00	0.00
ATOM	1392	HH12	ARG	86	2.148	-16.377	9.717	1.00	0.00
ATOM	1393	NH2	ARG	86	3.918	-15.344	11.096	1.00	0.00
ATOM	1394	HH21	ARG	86	4.645	-14.844	11.589	1.00	0.00
ATOM	1395	HH22	ARG	86	3.572	-16.224	11.452	1.00	0.00
ATOM	1396	C	ARG	86	3.027	-8.909	5.582	1.00	0.00
ATOM	1397	O	ARG	86	3.581	-8.901	4.482	1.00	0.00
ATOM	1398	N	MET	87	1.835	-8.353	5.810	1.00	0.00
ATOM	1399	H	MET	87	1.427	-8.405	6.733	1.00	0.00
ATOM	1400	CA	MET	87	1.080	-7.680	4.755	1.00	0.00
ATOM	1401	HA	MET	87	0.804	-8.395	3.980	1.00	0.00

ATOM	1402	CB	MET	87	-0.200	-7.068	5.335	1.00	0.00
ATOM	1403	HB2	MET	87	-0.723	-7.820	5.926	1.00	0.00
ATOM	1404	HB3	MET	87	0.061	-6.223	5.972	1.00	0.00
ATOM	1405	CG	MET	87	-1.108	-6.590	4.197	1.00	0.00
ATOM	1406	HG2	MET	87	-0.500	-6.178	3.392	1.00	0.00
ATOM	1407	HG3	MET	87	-1.692	-7.429	3.818	1.00	0.00
ATOM	1408	SD	MET	87	-2.233	-5.309	4.810	1.00	0.00
ATOM	1409	CE	MET	87	-3.268	-6.364	5.853	1.00	0.00
ATOM	1410	HE1	MET	87	-2.651	-6.837	6.618	1.00	0.00
ATOM	1411	HE2	MET	87	-4.038	-5.759	6.332	1.00	0.00
ATOM	1412	HE3	MET	87	-3.738	-7.133	5.240	1.00	0.00
ATOM	1413	C	MET	87	1.905	-6.575	4.100	1.00	0.00
ATOM	1414	O	MET	87	1.716	-6.272	2.924	1.00	0.00
ATOM	1415	N	MET	88	2.803	-5.959	4.869	1.00	0.00
ATOM	1416	H	MET	88	2.926	-6.228	5.835	1.00	0.00
ATOM	1417	CA	MET	88	3.623	-4.871	4.344	1.00	0.00
ATOM	1418	HA	MET	88	3.017	-3.979	4.190	1.00	0.00
ATOM	1419	CB	MET	88	4.729	-4.543	5.347	1.00	0.00
ATOM	1420	HB2	MET	88	5.281	-5.452	5.588	1.00	0.00
ATOM	1421	HB3	MET	88	5.409	-3.812	4.910	1.00	0.00
ATOM	1422	CG	MET	88	4.112	-3.966	6.627	1.00	0.00
ATOM	1423	HG2	MET	88	3.812	-2.932	6.457	1.00	0.00

ATOM 1424 HG3 MET 88 3.240 -4.555 6.913 1.00 0.00
ATOM 1425 SD MET 88 5.331 -4.020 7.965 1.00 0.00
ATOM 1426 CE MET 88 6.760 -3.470 7.003 1.00 0.00
ATOM 1427 HE1 MET 88 6.565 -2.478 6.596 1.00 0.00
ATOM 1428 HE2 MET 88 7.639 -3.431 7.647 1.00 0.00
ATOM 1429 HE3 MET 88 6.940 -4.169 6.186 1.00 0.00
ATOM 1430 C MET 88 4.249 -5.258 3.009 1.00 0.00
ATOM 1431 O MET 88 4.110 -4.538 2.020 1.00 0.00
ATOM 1432 N ARG 89 4.920 -6.405 2.980 1.00 0.00
ATOM 1433 H ARG 89 5.031 -6.961 3.816 1.00 0.00
ATOM 1434 CA ARG 89 5.540 -6.878 1.749 1.00 0.00
ATOM 1435 HA ARG 89 6.264 -6.146 1.390 1.00 0.00
ATOM 1436 CB ARG 89 6.268 -8.202 2.003 1.00 0.00
ATOM 1437 HB2 ARG 89 5.563 -8.933 2.399 1.00 0.00
ATOM 1438 HB3 ARG 89 6.686 -8.571 1.067 1.00 0.00
ATOM 1439 CG ARG 89 7.399 -7.983 3.016 1.00 0.00
ATOM 1440 HG2 ARG 89 7.007 -7.461 3.889 1.00 0.00
ATOM 1441 HG3 ARG 89 7.803 -8.948 3.322 1.00 0.00
ATOM 1442 CD ARG 89 8.510 -7.144 2.376 1.00 0.00
ATOM 1443 HD2 ARG 89 8.861 -7.629 1.465 1.00 0.00
ATOM 1444 HD3 ARG 89 8.131 -6.151 2.136 1.00 0.00
ATOM 1445 NE ARG 89 9.635 -7.005 3.295 1.00 0.00

ATOM 1446 HE ARG 89 10.445 -7.601 3.197 1.00 0.00
ATOM 1447 CZ ARG 89 9.626 -6.101 4.273 1.00 0.00
ATOM 1448 NH1 ARG 89 8.589 -5.324 4.440 1.00 0.00
ATOM 1449 HH11 ARG 89 7.793 -5.411 3.825 1.00 0.00
ATOM 1450 HH12 ARG 89 8.589 -4.641 5.184 1.00 0.00
ATOM 1451 NH2 ARG 89 10.654 -5.993 5.067 1.00 0.00
ATOM 1452 HH21 ARG 89 11.454 -6.596 4.938 1.00 0.00
ATOM 1453 HH22 ARG 89 10.646 -5.307 5.809 1.00 0.00
ATOM 1454 C ARG 89 4.479 -7.065 0.667 1.00 0.00
ATOM 1455 O ARG 89 4.671 -6.671 -0.484 1.00 0.00
ATOM 1456 N LEU 90 3.353 -7.660 1.058 1.00 0.00
ATOM 1457 H LEU 90 3.255 -7.976 2.012 1.00 0.00
ATOM 1458 CA LEU 90 2.249 -7.893 0.135 1.00 0.00
ATOM 1459 HA LEU 90 2.583 -8.494 -0.711 1.00 0.00
ATOM 1460 CB LEU 90 1.130 -8.649 0.861 1.00 0.00
ATOM 1461 HB2 LEU 90 1.547 -9.526 1.357 1.00 0.00
ATOM 1462 HB3 LEU 90 0.685 -7.989 1.606 1.00 0.00
ATOM 1463 CG LEU 90 0.054 -9.090 -0.140 1.00 0.00
ATOM 1464 HG LEU 90 0.512 -9.264 -1.113 1.00 0.00
ATOM 1465 CD1 LEU 90 -0.602 -10.383 0.353 1.00 0.00
ATOM 1466 HD11 LEU 90 -1.060 -10.210 1.327 1.00 0.00
ATOM 1467 HD12 LEU 90 -1.366 -10.697 -0.357 1.00 0.00

ATOM	1468	HD13	LEU	90	0.154	-11.164	0.442	1.00	0.00
ATOM	1469	CD2	LEU	90	-1.016	-7.998	-0.259	1.00	0.00
ATOM	1470	HD21	LEU	90	-0.555	-7.074	-0.606	1.00	0.00
ATOM	1471	HD22	LEU	90	-1.779	-8.313	-0.970	1.00	0.00
ATOM	1472	HD23	LEU	90	-1.475	-7.831	0.716	1.00	0.00
ATOM	1473	C	LEU	90	1.714	-6.570	-0.403	1.00	0.00
ATOM	1474	O	LEU	90	1.389	-6.454	-1.585	1.00	0.00
ATOM	1475	N	ALA	91	1.619	-5.579	0.480	1.00	0.00
ATOM	1476	H	ALA	91	1.880	-5.729	1.445	1.00	0.00
ATOM	1477	CA	ALA	91	1.114	-4.267	0.096	1.00	0.00
ATOM	1478	HA	ALA	91	0.074	-4.342	-0.221	1.00	0.00
ATOM	1479	CB	ALA	91	1.195	-3.307	1.286	1.00	0.00
ATOM	1480	HB1	ALA	91	2.232	-3.215	1.610	1.00	0.00
ATOM	1481	HB2	ALA	91	0.820	-2.328	0.990	1.00	0.00
ATOM	1482	HB3	ALA	91	0.592	-3.694	2.108	1.00	0.00
ATOM	1483	C	ALA	91	1.916	-3.708	-1.071	1.00	0.00
ATOM	1484	O	ALA	91	1.349	-3.305	-2.080	1.00	0.00
ATOM	1485	N	GLU	92	3.238	-3.696	-0.923	1.00	0.00
ATOM	1486	H	GLU	92	3.651	-4.017	-0.059	1.00	0.00
ATOM	1487	CA	GLU	92	4.126	-3.196	-1.972	1.00	0.00
ATOM	1488	HA	GLU	92	3.891	-2.157	-2.201	1.00	0.00
ATOM	1489	CB	GLU	92	5.580	-3.275	-1.498	1.00	0.00

ATOM	1490	HB2	GLU	92	5.673	-2.773	-0.535	1.00	0.00
ATOM	1491	HB3	GLU	92	5.869	-4.321	-1.391	1.00	0.00
ATOM	1492	CG	GLU	92	6.499	-2.593	-2.521	1.00	0.00
ATOM	1493	HG2	GLU	92	6.347	-3.056	-3.496	1.00	0.00
ATOM	1494	HG3	GLU	92	6.240	-1.536	-2.576	1.00	0.00
ATOM	1495	CD	GLU	92	7.966	-2.737	-2.112	1.00	0.00
ATOM	1496	OE1	GLU	92	8.229	-3.373	-1.101	1.00	0.00
ATOM	1497	OE2	GLU	92	8.808	-2.209	-2.818	1.00	0.00
ATOM	1498	C	GLU	92	3.958	-3.998	-3.262	1.00	0.00
ATOM	1499	O	GLU	92	4.151	-3.471	-4.357	1.00	0.00
ATOM	1500	N	ASN	93	3.614	-5.276	-3.123	1.00	0.00
ATOM	1501	H	ASN	93	3.489	-5.675	-2.203	1.00	0.00
ATOM	1502	CA	ASN	93	3.442	-6.147	-4.286	1.00	0.00
ATOM	1503	HA	ASN	93	4.304	-6.061	-4.948	1.00	0.00
ATOM	1504	CB	ASN	93	3.315	-7.606	-3.834	1.00	0.00
ATOM	1505	HB2	ASN	93	2.493	-7.704	-3.125	1.00	0.00
ATOM	1506	HB3	ASN	93	3.130	-8.248	-4.695	1.00	0.00
ATOM	1507	CG	ASN	93	4.604	-8.064	-3.152	1.00	0.00
ATOM	1508	OD1	ASN	93	5.619	-7.248	-3.064	1.00	0.00
ATOM	1509	ND2	ASN	93	4.686	-9.201	-2.687	1.00	0.00
ATOM	1510	HD21	ASN	93	5.540	-9.499	-2.237	1.00	0.00
ATOM	1511	HD22	ASN	93	3.899	-9.830	-2.757	1.00	0.00

ATOM	1512	C	ASN	93	2.207	-5.757	-5.099	1.00	0.00
ATOM	1513	O	ASN	93	2.208	-5.870	-6.325	1.00	0.00
ATOM	1514	N	ILE	94	1.155	-5.305	-4.416	1.00	0.00
ATOM	1515	H	ILE	94	1.177	-5.263	-3.407	1.00	0.00
ATOM	1516	CA	ILE	94	-0.077	-4.913	-5.100	1.00	0.00
ATOM	1517	HA	ILE	94	-0.526	-5.792	-5.563	1.00	0.00
ATOM	1518	CB	ILE	94	-1.065	-4.303	-4.093	1.00	0.00
ATOM	1519	HB	ILE	94	-0.517	-3.675	-3.390	1.00	0.00
ATOM	1520	CG2	ILE	94	-2.113	-3.456	-4.826	1.00	0.00
ATOM	1521	HG21	ILE	94	-2.661	-4.084	-5.528	1.00	0.00
ATOM	1522	HG22	ILE	94	-2.807	-3.030	-4.102	1.00	0.00
ATOM	1523	HG23	ILE	94	-1.616	-2.653	-5.369	1.00	0.00
ATOM	1524	CG1	ILE	94	-1.769	-5.434	-3.331	1.00	0.00
ATOM	1525	HG12	ILE	94	-2.557	-5.855	-3.955	1.00	0.00
ATOM	1526	HG13	ILE	94	-1.044	-6.212	-3.087	1.00	0.00
ATOM	1527	CD1	ILE	94	-2.381	-4.884	-2.039	1.00	0.00
ATOM	1528	HD11	ILE	94	-3.105	-4.107	-2.281	1.00	0.00
ATOM	1529	HD12	ILE	94	-2.880	-5.690	-1.501	1.00	0.00
ATOM	1530	HD13	ILE	94	-1.593	-4.464	-1.413	1.00	0.00
ATOM	1531	C	ILE	94	0.219	-3.922	-6.234	1.00	0.00
ATOM	1532	O	ILE	94	-0.134	-4.172	-7.386	1.00	0.00
ATOM	1533	N	PRO	95	0.859	-2.817	-5.938	1.00	0.00

ATOM	1534	CD	PRO	95	1.315	-2.421	-4.597	1.00	0.00
ATOM	1535	HD2	PRO	95	1.886	-3.240	-4.159	1.00	0.00
ATOM	1536	HD3	PRO	95	0.446	-2.211	-3.973	1.00	0.00
ATOM	1537	CG	PRO	95	2.167	-1.180	-4.845	1.00	0.00
ATOM	1538	HG2	PRO	95	3.217	-1.469	-4.906	1.00	0.00
ATOM	1539	HG3	PRO	95	2.032	-0.478	-4.022	1.00	0.00
ATOM	1540	CB	PRO	95	1.688	-0.595	-6.127	1.00	0.00
ATOM	1541	HB2	PRO	95	2.518	-0.101	-6.631	1.00	0.00
ATOM	1542	HB3	PRO	95	0.873	0.114	-5.988	1.00	0.00
ATOM	1543	CA	PRO	95	1.208	-1.786	-6.960	1.00	0.00
ATOM	1544	HA	PRO	95	0.305	-1.558	-7.526	1.00	0.00
ATOM	1545	C	PRO	95	2.306	-2.250	-7.919	1.00	0.00
ATOM	1546	O	PRO	95	2.401	-1.755	-9.038	1.00	0.00
ATOM	1547	N	SER	96	3.125	-3.210	-7.479	1.00	0.00
ATOM	1548	H	SER	96	3.019	-3.584	-6.547	1.00	0.00
ATOM	1549	CA	SER	96	4.200	-3.736	-8.319	1.00	0.00
ATOM	1550	HA	SER	96	4.861	-2.924	-8.622	1.00	0.00
ATOM	1551	CB	SER	96	5.022	-4.776	-7.555	1.00	0.00
ATOM	1552	HB2	SER	96	4.381	-5.611	-7.271	1.00	0.00
ATOM	1553	HB3	SER	96	5.831	-5.139	-8.189	1.00	0.00
ATOM	1554	OG	SER	96	5.567	-4.180	-6.387	1.00	0.00
ATOM	1555	HG	SER	96	6.083	-4.832	-5.906	1.00	0.00

ATOM	1556	C	SER	96	3.587	-4.366	-9.542	1.00	0.00
ATOM	1557	O	SER	96	4.063	-4.216	-10.668	1.00	0.00
ATOM	1558	N	ARG	97	2.506	-5.065	-9.273	1.00	0.00
ATOM	1559	H	ARG	97	2.205	-5.144	-8.312	1.00	0.00
ATOM	1560	CA	ARG	97	1.744	-5.748	-10.285	1.00	0.00
ATOM	1561	HA	ARG	97	2.336	-6.529	-10.763	1.00	0.00
ATOM	1562	CB	ARG	97	0.545	-6.395	-9.604	1.00	0.00
ATOM	1563	HB2	ARG	97	0.086	-5.679	-8.923	1.00	0.00
ATOM	1564	HB3	ARG	97	-0.181	-6.694	-10.359	1.00	0.00
ATOM	1565	CG	ARG	97	1.000	-7.629	-8.816	1.00	0.00
ATOM	1566	HG2	ARG	97	1.539	-8.304	-9.481	1.00	0.00
ATOM	1567	HG3	ARG	97	1.659	-7.317	-8.005	1.00	0.00
ATOM	1568	CD	ARG	97	-0.217	-8.352	-8.234	1.00	0.00
ATOM	1569	HD2	ARG	97	-0.856	-8.709	-9.042	1.00	0.00
ATOM	1570	HD3	ARG	97	0.110	-9.198	-7.629	1.00	0.00
ATOM	1571	NE	ARG	97	-0.992	-7.445	-7.390	1.00	0.00
ATOM	1572	HE	ARG	97	-0.725	-7.305	-6.426	1.00	0.00
ATOM	1573	CZ	ARG	97	-2.053	-6.789	-7.858	1.00	0.00
ATOM	1574	NH1	ARG	97	-2.439	-6.950	-9.096	1.00	0.00
ATOM	1575	HH11	ARG	97	-1.929	-7.576	-9.703	1.00	0.00
ATOM	1576	HH12	ARG	97	-3.244	-6.448	-9.440	1.00	0.00
ATOM	1577	NH2	ARG	97	-2.710	-5.980	-7.075	1.00	0.00

```
ATOM   1578 HH21 ARG    97      -2.411  -5.855  -6.118  1.00  0.00
ATOM   1579 HH22 ARG    97      -3.514  -5.482  -7.428  1.00  0.00
ATOM   1580  C   ARG    97       1.279  -4.791 -11.382  1.00  0.00
ATOM   1581  O   ARG    97       1.223  -5.165 -12.554  1.00  0.00
ATOM   1582  N   CYX    98       0.956  -3.556 -10.999  1.00  0.00
ATOM   1583  H   CYX    98       1.020  -3.288 -10.027  1.00  0.00
ATOM   1584  CA  CYX    98       0.509  -2.561 -11.961  1.00  0.00
ATOM   1585  HA  CYX    98       0.194  -3.059 -12.878  1.00  0.00
ATOM   1586  CB  CYX    98      -0.667  -1.754 -11.414  1.00  0.00
ATOM   1587  HB2 CYX    98      -0.301  -0.817 -10.994  1.00  0.00
ATOM   1588  HB3 CYX    98      -1.368  -1.541 -12.220  1.00  0.00
ATOM   1589  SG  CYX    98      -1.496  -2.688 -10.101  1.00  0.00
ATOM   1590  C   CYX    98       1.629  -1.602 -12.275  1.00  0.00
ATOM   1591  O   CYX    98       1.512  -0.786 -13.189  1.00  0.00
ATOM   1592  N   ASN    99       2.702  -1.668 -11.493  1.00  0.00
ATOM   1593  H   ASN    99       2.751  -2.299 -10.705  1.00  0.00
ATOM   1594  CA  ASN    99       3.797  -0.751 -11.704  1.00  0.00
ATOM   1595  HA  ASN    99       3.416   0.263 -11.584  1.00  0.00
ATOM   1596  CB  ASN    99       4.954  -0.946 -10.755  1.00  0.00
ATOM   1597  HB2 ASN    99       5.089  -2.003 -10.526  1.00  0.00
ATOM   1598  HB3 ASN    99       5.869  -0.551 -11.197  1.00  0.00
ATOM   1599  CG  ASN    99       4.678  -0.198  -9.456  1.00  0.00
```

ATOM	1600	OD1	ASN	99	4.974	-0.695	-8.370	1.00	0.00
ATOM	1601	ND2	ASN	99	4.128	0.986	-9.511	1.00	0.00
ATOM	1602	HD21	ASN	99	3.940	1.492	-8.657	1.00	0.00
ATOM	1603	HD22	ASN	99	3.894	1.389	-10.406	1.00	0.00
ATOM	1604	C	ASN	99	4.293	-0.812	-13.115	1.00	0.00
ATOM	1605	O	ASN	99	4.121	-1.798	-13.835	1.00	0.00
ATOM	1606	N	LEU	100	4.822	0.313	-13.510	1.00	0.00
ATOM	1607	H	LEU	100	4.924	1.072	-12.851	1.00	0.00
ATOM	1608	CA	LEU	100	5.269	0.518	-14.862	1.00	0.00
ATOM	1609	HA	LEU	100	5.597	-0.412	-15.326	1.00	0.00
ATOM	1610	CB	LEU	100	4.059	1.053	-15.637	1.00	0.00
ATOM	1611	HB2	LEU	100	4.375	1.923	-16.212	1.00	0.00
ATOM	1612	HB3	LEU	100	3.660	0.296	-16.312	1.00	0.00
ATOM	1613	CG	LEU	100	2.959	1.474	-14.624	1.00	0.00
ATOM	1614	HG	LEU	100	2.960	0.781	-13.782	1.00	0.00
ATOM	1615	CD1	LEU	100	3.234	2.890	-14.118	1.00	0.00
ATOM	1616	HD11	LEU	100	3.233	3.583	-14.959	1.00	0.00
ATOM	1617	HD12	LEU	100	2.460	3.179	-13.408	1.00	0.00
ATOM	1618	HD13	LEU	100	4.206	2.918	-13.625	1.00	0.00
ATOM	1619	CD2	LEU	100	1.588	1.407	-15.280	1.00	0.00
ATOM	1620	HD21	LEU	100	1.394	0.387	-15.614	1.00	0.00
ATOM	1621	HD22	LEU	100	0.826	1.704	-14.560	1.00	0.00

ATOM	1622	HD23	LEU	100	1.561	2.081	-16.136	1.00	0.00
ATOM	1623	C	LEU	100	6.418	1.521	-14.892	1.00	0.00
ATOM	1624	O	LEU	100	6.392	2.515	-14.169	1.00	0.00
ATOM	1625	N	SER	101	7.422	1.240	-15.726	1.00	0.00
ATOM	1626	H	SER	101	7.388	0.396	-16.280	1.00	0.00
ATOM	1627	CA	SER	101	8.598	2.106	-15.851	1.00	0.00
ATOM	1628	HA	SER	101	9.335	1.853	-15.088	1.00	0.00
ATOM	1629	CB	SER	101	9.235	1.914	-17.229	1.00	0.00
ATOM	1630	HB2	SER	101	9.522	0.870	-17.356	1.00	0.00
ATOM	1631	HB3	SER	101	8.520	2.191	-18.003	1.00	0.00
ATOM	1632	OG	SER	101	10.389	2.738	-17.333	1.00	0.00
ATOM	1633	HG	SER	101	10.791	2.619	-18.197	1.00	0.00
ATOM	1634	C	SER	101	8.221	3.575	-15.663	1.00	0.00
ATOM	1635	O	SER	101	7.772	4.239	-16.598	1.00	0.00
ATOM	1636	N	PRO	102	8.392	4.084	-14.468	1.00	0.00
ATOM	1637	CD	PRO	102	8.906	3.378	-13.298	1.00	0.00
ATOM	1638	HD2	PRO	102	9.981	3.536	-13.204	1.00	0.00
ATOM	1639	HD3	PRO	102	8.703	2.310	-13.383	1.00	0.00
ATOM	1640	CG	PRO	102	8.119	4.030	-12.173	1.00	0.00
ATOM	1641	HG2	PRO	102	8.702	3.982	-11.253	1.00	0.00
ATOM	1642	HG3	PRO	102	7.179	3.495	-12.035	1.00	0.00
ATOM	1643	CB	PRO	102	7.871	5.452	-12.593	1.00	0.00

ATOM	1644	HB2	PRO	102	8.614	6.084	-12.106	1.00	0.00
ATOM	1645	HB3	PRO	102	6.872	5.804	-12.338	1.00	0.00
ATOM	1646	CA	PRO	102	8.069	5.492	-14.122	1.00	0.00
ATOM	1647	HA	PRO	102	7.158	5.774	-14.649	1.00	0.00
ATOM	1648	C	PRO	102	9.196	6.444	-14.510	1.00	0.00
ATOM	1649	O	PRO	102	10.314	6.332	-14.003	1.00	0.00
ATOM	1650	N	MET	103	8.900	7.376	-15.412	1.00	0.00
ATOM	1651	H	MET	103	7.977	7.427	-15.819	1.00	0.00
ATOM	1652	CA	MET	103	9.905	8.333	-15.856	1.00	0.00
ATOM	1653	HA	MET	103	10.810	7.809	-16.164	1.00	0.00
ATOM	1654	CB	MET	103	9.372	9.134	-17.049	1.00	0.00
ATOM	1655	HB2	MET	103	8.446	9.632	-16.764	1.00	0.00
ATOM	1656	HB3	MET	103	10.111	9.880	-17.341	1.00	0.00
ATOM	1657	CG	MET	103	9.103	8.192	-18.228	1.00	0.00
ATOM	1658	HG2	MET	103	8.316	7.487	-17.960	1.00	0.00
ATOM	1659	HG3	MET	103	8.790	8.772	-19.095	1.00	0.00
ATOM	1660	SD	MET	103	10.610	7.275	-18.638	1.00	0.00
ATOM	1661	CE	MET	103	11.626	8.692	-19.125	1.00	0.00
ATOM	1662	HE1	MET	103	11.719	9.379	-18.284	1.00	0.00
ATOM	1663	HE2	MET	103	12.616	8.345	-19.421	1.00	0.00
ATOM	1664	HE3	MET	103	11.156	9.205	-19.963	1.00	0.00
ATOM	1665	C	MET	103	10.273	9.286	-14.724	1.00	0.00

ATOM 1666 O MET 103 11.439 9.378 -14.335 1.00 0.00

ATOM 1667 N ARG 104 9.272 9.986 -14.193 1.00 0.00

ATOM 1668 H ARG 104 8.333 9.893 -14.552 1.00 0.00

ATOM 1669 CA ARG 104 9.504 10.921 -13.096 1.00 0.00

ATOM 1670 HA ARG 104 10.325 10.570 -12.471 1.00 0.00

ATOM 1671 CB ARG 104 9.871 12.301 -13.658 1.00 0.00

ATOM 1672 HB2 ARG 104 9.571 12.353 -14.704 1.00 0.00

ATOM 1673 HB3 ARG 104 9.347 13.070 -13.091 1.00 0.00

ATOM 1674 CG ARG 104 11.385 12.527 -13.548 1.00 0.00

ATOM 1675 HG2 ARG 104 11.907 11.711 -14.049 1.00 0.00

ATOM 1676 HG3 ARG 104 11.643 13.471 -14.027 1.00 0.00

ATOM 1677 CD ARG 104 11.804 12.572 -12.073 1.00 0.00

ATOM 1678 HD2 ARG 104 11.979 11.559 -11.710 1.00 0.00

ATOM 1679 HD3 ARG 104 12.718 13.157 -11.968 1.00 0.00

ATOM 1680 NE ARG 104 10.758 13.187 -11.259 1.00 0.00

ATOM 1681 HE ARG 104 10.156 12.607 -10.692 1.00 0.00

ATOM 1682 CZ ARG 104 10.570 14.505 -11.242 1.00 0.00

ATOM 1683 NH1 ARG 104 11.333 15.287 -11.958 1.00 0.00

ATOM 1684 HH11 ARG 104 12.068 14.889 -12.525 1.00 0.00

ATOM 1685 HH12 ARG 104 11.185 16.286 -11.940 1.00 0.00

ATOM 1686 NH2 ARG 104 9.623 15.016 -10.507 1.00 0.00

ATOM 1687 HH21 ARG 104 9.035 14.409 -9.953 1.00 0.00

ATOM	1688	HH22	ARG	104	9.482	16.016	-10.495	1.00	0.00
ATOM	1689	C	ARG	104	8.266	11.050	-12.210	1.00	0.00
ATOM	1690	O	ARG	104	7.141	11.107	-12.706	1.00	0.00
ATOM	1691	N	CYX	105	8.491	11.127	-10.899	1.00	0.00
ATOM	1692	H	CYX	105	9.433	11.059	-10.540	1.00	0.00
ATOM	1693	CA	CYX	105	7.397	11.287	-9.945	1.00	0.00
ATOM	1694	HA	CYX	105	6.444	11.115	-10.444	1.00	0.00
ATOM	1695	CB	CYX	105	7.536	10.283	-8.798	1.00	0.00
ATOM	1696	HB2	CYX	105	8.572	10.259	-8.459	1.00	0.00
ATOM	1697	HB3	CYX	105	6.891	10.582	-7.972	1.00	0.00
ATOM	1698	SG	CYX	105	7.077	8.629	-9.378	1.00	0.00
ATOM	1699	C	CYX	105	7.403	12.711	-9.387	1.00	0.00
ATOM	1700	O	CYX	105	8.312	13.083	-8.645	1.00	0.00
ATOM	1701	N	PRO	106	6.420	13.511	-9.729	1.00	0.00
ATOM	1702	CD	PRO	106	5.302	13.164	-10.618	1.00	0.00
ATOM	1703	HD2	PRO	106	4.610	12.511	-10.085	1.00	0.00
ATOM	1704	HD3	PRO	106	5.689	12.644	-11.494	1.00	0.00
ATOM	1705	CG	PRO	106	4.681	14.517	-10.969	1.00	0.00
ATOM	1706	HG2	PRO	106	3.602	14.398	-11.065	1.00	0.00
ATOM	1707	HG3	PRO	106	5.094	14.862	-11.917	1.00	0.00
ATOM	1708	CB	PRO	106	5.030	15.457	-9.862	1.00	0.00
ATOM	1709	HB2	PRO	106	4.236	15.460	-9.115	1.00	0.00

ATOM 1710 HB3 PRO 106 5.185 16.471 -10.231 1.00 0.00

ATOM 1711 CA PRO 106 6.323 14.924 -9.244 1.00 0.00

ATOM 1712 HA PRO 106 7.195 15.460 -9.618 1.00 0.00

ATOM 1713 C PRO 106 6.270 15.038 -7.726 1.00 0.00

ATOM 1714 O PRO 106 6.093 16.128 -7.185 1.00 0.00

ATOM 1715 N MET 107 6.424 13.915 -7.048 1.00 0.00

ATOM 1716 H MET 107 6.582 13.042 -7.531 1.00 0.00

ATOM 1717 CA MET 107 6.392 13.902 -5.597 1.00 0.00

ATOM 1718 HA MET 107 5.565 14.508 -5.227 1.00 0.00

ATOM 1719 CB MET 107 6.200 12.484 -5.122 1.00 0.00

ATOM 1720 HB2 MET 107 6.806 11.818 -5.736 1.00 0.00

ATOM 1721 HB3 MET 107 6.521 12.410 -4.083 1.00 0.00

ATOM 1722 CG MET 107 4.725 12.079 -5.231 1.00 0.00

ATOM 1723 HG2 MET 107 4.646 11.145 -5.788 1.00 0.00

ATOM 1724 HG3 MET 107 4.313 11.941 -4.231 1.00 0.00

ATOM 1725 SD MET 107 3.784 13.368 -6.092 1.00 0.00

ATOM 1726 CE MET 107 3.761 12.594 -7.726 1.00 0.00

ATOM 1727 HE1 MET 107 3.266 11.625 -7.663 1.00 0.00

ATOM 1728 HE2 MET 107 3.221 13.233 -8.424 1.00 0.00

ATOM 1729 HE3 MET 107 4.784 12.456 -8.077 1.00 0.00

ATOM 1730 C MET 107 7.699 14.424 -5.046 1.00 0.00

ATOM 1731 O MET 107 8.648 13.671 -4.819 1.00 0.00

ATOM	1732	N	GLY	108	7.733	15.720	-4.839	1.00	0.00
ATOM	1733	H	GLY	108	6.925	16.290	-5.044	1.00	0.00
ATOM	1734	CA	GLY	108	8.916	16.369	-4.316	1.00	0.00
ATOM	1735	HA2	GLY	108	8.652	17.365	-3.962	1.00	0.00
ATOM	1736	HA3	GLY	108	9.311	15.782	-3.486	1.00	0.00
ATOM	1737	C	GLY	108	9.990	16.493	-5.391	1.00	0.00
ATOM	1738	O	GLY	108	11.182	16.549	-5.087	1.00	0.00
ATOM	1739	N	GLY	109	9.553	16.542	-6.648	1.00	0.00
ATOM	1740	H	GLY	109	8.564	16.491	-6.845	1.00	0.00
ATOM	1741	CA	GLY	109	10.478	16.669	-7.771	1.00	0.00
ATOM	1742	HA2	GLY	109	9.923	16.560	-8.702	1.00	0.00
ATOM	1743	HA3	GLY	109	10.944	17.654	-7.738	1.00	0.00
ATOM	1744	C	GLY	109	11.569	15.602	-7.716	1.00	0.00
ATOM	1745	O	GLY	109	12.740	15.914	-7.495	1.00	0.00
ATOM	1746	N	SER	110	11.178	14.344	-7.920	1.00	0.00
ATOM	1747	H	SER	110	10.204	14.129	-8.076	1.00	0.00
ATOM	1748	CA	SER	110	12.136	13.239	-7.893	1.00	0.00
ATOM	1749	HA	SER	110	12.566	13.142	-6.896	1.00	0.00
ATOM	1750	CB	SER	110	11.436	11.929	-8.252	1.00	0.00
ATOM	1751	HB2	SER	110	11.072	11.979	-9.278	1.00	0.00
ATOM	1752	HB3	SER	110	12.139	11.101	-8.156	1.00	0.00
ATOM	1753	OG	SER	110	10.340	11.724	-7.369	1.00	0.00

ATOM	1754	HG	SER	110	9.899	10.901	-7.592	1.00	0.00
ATOM	1755	C	SER	110	13.279	13.500	-8.872	1.00	0.00
ATOM	1756	O	SER	110	13.122	14.251	-9.835	1.00	0.00
ATOM	1757	N	ILE	111	14.430	12.883	-8.617	1.00	0.00
ATOM	1758	H	ILE	111	14.528	12.294	-7.802	1.00	0.00
ATOM	1759	CA	ILE	111	15.593	13.067	-9.482	1.00	0.00
ATOM	1760	HA	ILE	111	15.503	14.016	-10.010	1.00	0.00
ATOM	1761	CB	ILE	111	16.875	13.071	-8.641	1.00	0.00
ATOM	1762	HB	ILE	111	16.971	12.117	-8.121	1.00	0.00
ATOM	1763	CG2	ILE	111	18.089	13.279	-9.554	1.00	0.00
ATOM	1764	HG21	ILE	111	17.995	14.232	-10.073	1.00	0.00
ATOM	1765	HG22	ILE	111	18.999	13.281	-8.954	1.00	0.00
ATOM	1766	HG23	ILE	111	18.138	12.471	-10.284	1.00	0.00
ATOM	1767	CG1	ILE	111	16.806	14.209	-7.614	1.00	0.00
ATOM	1768	HG12	ILE	111	16.835	15.167	-8.133	1.00	0.00
ATOM	1769	HG13	ILE	111	15.877	14.130	-7.049	1.00	0.00
ATOM	1770	CD1	ILE	111	17.996	14.112	-6.655	1.00	0.00
ATOM	1771	HD11	ILE	111	18.926	14.190	-7.219	1.00	0.00
ATOM	1772	HD12	ILE	111	17.944	14.921	-5.927	1.00	0.00
ATOM	1773	HD13	ILE	111	17.968	13.154	-6.135	1.00	0.00
ATOM	1774	C	ILE	111	15.662	11.962	-10.534	1.00	0.00
ATOM	1775	O	ILE	111	15.270	12.162	-11.685	1.00	0.00

ATOM	1776	N	ALA	112	16.137	10.789	-10.120	1.00	0.00
ATOM	1777	H	ALA	112	16.438	10.677	-9.162	1.00	0.00
ATOM	1778	CA	ALA	112	16.228	9.645	-11.008	1.00	0.00
ATOM	1779	HA	ALA	112	16.717	9.891	-11.950	1.00	0.00
ATOM	1780	CB	ALA	112	17.005	8.517	-10.336	1.00	0.00
ATOM	1781	HB1	ALA	112	16.522	8.253	-9.395	1.00	0.00
ATOM	1782	HB2	ALA	112	17.023	7.646	-10.991	1.00	0.00
ATOM	1783	HB3	ALA	112	18.026	8.844	-10.140	1.00	0.00
ATOM	1784	C	ALA	112	14.837	9.169	-11.317	1.00	0.00
ATOM	1785	O	ALA	112	13.861	9.847	-10.991	1.00	0.00
ATOM	1786	N	GLY	113	14.723	7.985	-11.890	1.00	0.00
ATOM	1787	H	GLY	113	15.512	7.416	-12.163	1.00	0.00
ATOM	1788	CA	GLY	113	13.404	7.481	-12.136	1.00	0.00
ATOM	1789	HA2	GLY	113	12.750	8.266	-12.513	1.00	0.00
ATOM	1790	HA3	GLY	113	13.428	6.655	-12.847	1.00	0.00
ATOM	1791	C	GLY	113	12.859	6.978	-10.815	1.00	0.00
ATOM	1792	O	GLY	113	13.148	5.859	-10.389	1.00	0.00
ATOM	1793	N	PHE	114	12.099	7.845	-10.163	1.00	0.00
ATOM	1794	H	PHE	114	11.924	8.754	-10.566	1.00	0.00
ATOM	1795	CA	PHE	114	11.522	7.545	-8.857	1.00	0.00
ATOM	1796	HA	PHE	114	12.269	7.683	-8.075	1.00	0.00
ATOM	1797	CB	PHE	114	10.347	8.486	-8.582	1.00	0.00

```
ATOM   1798  HB2 PHE   114      10.635   9.535  -8.652  1.00  0.00
ATOM   1799  HB3 PHE   114       9.506   8.295  -9.248  1.00  0.00
ATOM   1800  CG  PHE   114       9.841   8.270  -7.173  1.00  0.00
ATOM   1801  CD1 PHE   114      10.449   8.938  -6.102  1.00  0.00
ATOM   1802  HD1 PHE   114      11.287   9.611  -6.283  1.00  0.00
ATOM   1803  CE1 PHE   114       9.980   8.740  -4.798  1.00  0.00
ATOM   1804  HE1 PHE   114      10.453   9.260  -3.965  1.00  0.00
ATOM   1805  CZ  PHE   114       8.906   7.875  -4.563  1.00  0.00
ATOM   1806  HZ  PHE   114       8.543   7.721  -3.547  1.00  0.00
ATOM   1807  CE2 PHE   114       8.298   7.207  -5.633  1.00  0.00
ATOM   1808  HE2 PHE   114       7.462   6.533  -5.450  1.00  0.00
ATOM   1809  CD2 PHE   114       8.765   7.405  -6.938  1.00  0.00
ATOM   1810  HD2 PHE   114       8.291   6.886  -7.771  1.00  0.00
ATOM   1811  C   PHE   114      11.042   6.094  -8.784  1.00  0.00
ATOM   1812  O   PHE   114      11.443   5.405  -7.861  1.00  0.00
ATOM   1813  OXT PHE   114      10.280   5.696  -9.651  1.00  0.00
TER
ATOM   1814  S1  LIG   115      20.186 -17.648  -7.229  1.00  0.00
ATOM   1815  S2  LIG   115      17.500 -22.374  -1.683  1.00  0.00
ATOM   1816  O1  LIG   115      14.611 -18.284  -3.197  1.00  0.00
ATOM   1817  H5  LIG   115      14.071 -18.772  -2.539  1.00  0.00
ATOM   1818  O2  LIG   115      20.828 -16.228  -6.847  1.00  0.00
```

ATOM	1819	H6	LIG	115	20.063	-15.587	-6.964	1.00	0.00
ATOM	1820	O3	LIG	115	16.328	-23.416	-2.033	1.00	0.00
ATOM	1821	H7	LIG	115	15.493	-22.948	-1.741	1.00	0.00
ATOM	1822	O4	LIG	115	21.197	-18.650	-6.962	1.00	0.00
ATOM	1823	O5	LIG	115	19.536	-17.567	-8.529	1.00	0.00
ATOM	1824	O6	LIG	115	18.733	-22.944	-2.184	1.00	0.00
ATOM	1825	O7	LIG	115	17.376	-21.942	-0.299	1.00	0.00
ATOM	1826	O8	LIG	115	15.898	-10.411	-10.471	1.00	0.00
ATOM	1827	O9	LIG	115	17.965	-9.758	-10.344	1.00	0.00
ATOM	1828	N1	LIG	115	15.624	-16.486	-4.865	1.00	0.00
ATOM	1829	H3	LIG	115	14.830	-16.736	-4.298	1.00	0.00
ATOM	1830	H4	LIG	115	15.616	-15.663	-5.447	1.00	0.00
ATOM	1831	N2	LIG	115	17.480	-15.784	-6.548	1.00	0.00
ATOM	1832	N3	LIG	115	14.734	-20.426	-1.828	1.00	0.00
ATOM	1833	N4	LIG	115	18.410	-15.145	-7.122	1.00	0.00
ATOM	1834	N5	LIG	115	14.510	-21.563	-1.331	1.00	0.00
ATOM	1835	N6	LIG	115	17.061	-10.549	-10.110	1.00	0.00
ATOM	1836	C1	LIG	115	16.761	-18.539	-4.217	1.00	0.00
ATOM	1837	C2	LIG	115	17.927	-19.348	-4.344	1.00	0.00
ATOM	1838	C3	LIG	115	16.671	-17.320	-4.987	1.00	0.00
ATOM	1839	C4	LIG	115	18.870	-17.831	-5.984	1.00	0.00
ATOM	1840	C5	LIG	115	18.958	-18.975	-5.241	1.00	0.00

```
ATOM   1841  H1  LIG   115      19.820 -19.619  -5.357  1.00  0.00
ATOM   1842  C6  LIG   115      15.748 -18.996  -3.335  1.00  0.00
ATOM   1843  C7  LIG   115      17.075 -20.950  -2.726  1.00  0.00
ATOM   1844  C8  LIG   115      18.062 -20.530  -3.577  1.00  0.00
ATOM   1845  H2  LIG   115      18.972 -21.111  -3.656  1.00  0.00
ATOM   1846  C9  LIG   115      17.738 -16.958  -5.876  1.00  0.00
ATOM   1847  C10 LIG   115      15.868 -20.198  -2.598  1.00  0.00
ATOM   1848  C11 LIG   115      18.011 -14.016  -7.863  1.00  0.00
ATOM   1849  C12 LIG   115      13.369 -21.687  -0.517  1.00  0.00
ATOM   1850  C13 LIG   115      16.695 -13.811  -8.313  1.00  0.00
ATOM   1851  H8  LIG   115      15.938 -14.555  -8.106  1.00  0.00
ATOM   1852  C14 LIG   115      19.015 -13.092  -8.184  1.00  0.00
ATOM   1853  H9  LIG   115      20.031 -13.272  -7.855  1.00  0.00
ATOM   1854  C15 LIG   115      16.385 -12.677  -9.046  1.00  0.00
ATOM   1855  H10 LIG   115      15.385 -12.498  -9.413  1.00  0.00
ATOM   1856  C16 LIG   115      18.708 -11.952  -8.912  1.00  0.00
ATOM   1857  H11 LIG   115      19.464 -11.221  -9.162  1.00  0.00
ATOM   1858  C17 LIG   115      17.395 -11.758  -9.328  1.00  0.00
ATOM   1859  C18 LIG   115      12.665 -20.597   0.021  1.00  0.00
ATOM   1860  H12 LIG   115      13.013 -19.589  -0.162  1.00  0.00
ATOM   1861  C19 LIG   115      12.961 -22.997  -0.226  1.00  0.00
ATOM   1862  H13 LIG   115      13.527 -23.830  -0.625  1.00  0.00
```

```
ATOM   1863  C20 LIG   115      11.550 -20.826   0.813  1.00  0.00
ATOM   1864  H14 LIG   115      11.011 -19.987   1.238  1.00  0.00
ATOM   1865  C21 LIG   115      11.836 -23.216   0.560  1.00  0.00
ATOM   1866  H15 LIG   115      11.519 -24.229   0.775  1.00  0.00
ATOM   1867  C22 LIG   115      11.128 -22.133   1.079  1.00  0.00
ATOM   1868  H16 LIG   115      10.258 -22.303   1.702  1.00  0.00
TER
ATOM   1869  S1  LIG   116     -18.229  18.521   5.449  1.00  0.00
ATOM   1870  S2  LIG   116     -20.882  25.742   6.508  1.00  0.00
ATOM   1871  O1  LIG   116     -23.239  22.622   3.034  1.00  0.00
ATOM   1872  H5  LIG   116     -23.757  23.454   3.050  1.00  0.00
ATOM   1873  O2  LIG   116     -17.318  18.168   4.176  1.00  0.00
ATOM   1874  H6  LIG   116     -17.976  17.798   3.513  1.00  0.00
ATOM   1875  O3  LIG   116     -22.245  25.971   7.327  1.00  0.00
ATOM   1876  H7  LIG   116     -22.945  26.046   6.616  1.00  0.00
ATOM   1877  O4  LIG   116     -17.369  19.180   6.410  1.00  0.00
ATOM   1878  O5  LIG   116     -19.027  17.363   5.827  1.00  0.00
ATOM   1879  O6  LIG   116     -19.838  25.513   7.485  1.00  0.00
ATOM   1880  O7  LIG   116     -20.746  26.768   5.485  1.00  0.00
ATOM   1881  O8  LIG   116     -21.670  12.466  -0.276  1.00  0.00
ATOM   1882  O9  LIG   116     -19.526  12.183  -0.471  1.00  0.00
ATOM   1883  N1  LIG   116     -22.178  20.265   2.430  1.00  0.00
```

ATOM	1884	H3	LIG	116	-22.920	20.917	2.228	1.00	0.00
ATOM	1885	H4	LIG	116	-22.130	19.367	1.974	1.00	0.00
ATOM	1886	N2	LIG	116	-20.476	18.370	2.961	1.00	0.00
ATOM	1887	N3	LIG	116	-23.285	24.825	4.306	1.00	0.00
ATOM	1888	N4	LIG	116	-19.540	17.525	2.857	1.00	0.00
ATOM	1889	N5	LIG	116	-23.623	25.805	5.025	1.00	0.00
ATOM	1890	N6	LIG	116	-20.507	12.799	-0.077	1.00	0.00
ATOM	1891	C1	LIG	116	-21.317	21.746	4.160	1.00	0.00
ATOM	1892	C2	LIG	116	-20.327	21.961	5.161	1.00	0.00
ATOM	1893	C3	LIG	116	-21.308	20.501	3.427	1.00	0.00
ATOM	1894	C4	LIG	116	-19.378	19.763	4.776	1.00	0.00
ATOM	1895	C5	LIG	116	-19.379	20.949	5.454	1.00	0.00
ATOM	1896	H1	LIG	116	-18.658	21.110	6.245	1.00	0.00
ATOM	1897	C6	LIG	116	-22.262	22.783	3.951	1.00	0.00
ATOM	1898	C7	LIG	116	-21.204	24.175	5.651	1.00	0.00
ATOM	1899	C8	LIG	116	-20.285	23.185	5.873	1.00	0.00
ATOM	1900	H2	LIG	116	-19.502	23.346	6.602	1.00	0.00
ATOM	1901	C9	LIG	116	-20.326	19.501	3.734	1.00	0.00
ATOM	1902	C10	LIG	116	-22.244	23.990	4.689	1.00	0.00
ATOM	1903	C11	LIG	116	-19.846	16.361	2.125	1.00	0.00
ATOM	1904	C12	LIG	116	-24.649	26.632	4.529	1.00	0.00
ATOM	1905	C13	LIG	116	-21.158	15.924	1.876	1.00	0.00

ATOM 1906 H8 LIG 116 -21.992 16.487 2.272 1.00 0.00

ATOM 1907 C14 LIG 116 -18.756 15.602 1.675 1.00 0.00

ATOM 1908 H9 LIG 116 -17.748 15.933 1.894 1.00 0.00

ATOM 1909 C15 LIG 116 -21.374 14.761 1.155 1.00 0.00

ATOM 1910 H10 LIG 116 -22.372 14.397 0.956 1.00 0.00

ATOM 1911 C16 LIG 116 -18.966 14.440 0.947 1.00 0.00

ATOM 1912 H11 LIG 116 -18.142 13.845 0.581 1.00 0.00

ATOM 1913 C17 LIG 116 -20.275 14.039 0.693 1.00 0.00

ATOM 1914 C18 LIG 116 -25.086 26.629 3.194 1.00 0.00

ATOM 1915 H12 LIG 116 -24.605 25.981 2.473 1.00 0.00

ATOM 1916 C19 LIG 116 -25.223 27.517 5.453 1.00 0.00

ATOM 1917 H13 LIG 116 -24.860 27.529 6.474 1.00 0.00

ATOM 1918 C20 LIG 116 -26.106 27.483 2.806 1.00 0.00

ATOM 1919 H14 LIG 116 -26.439 27.491 1.775 1.00 0.00

ATOM 1920 C21 LIG 116 -26.252 28.363 5.057 1.00 0.00

ATOM 1921 H15 LIG 116 -26.700 29.039 5.774 1.00 0.00

ATOM 1922 C22 LIG 116 -26.697 28.346 3.735 1.00 0.00

ATOM 1923 H16 LIG 116 -27.491 29.014 3.422 1.00 0.00

TER

ATOM 1924 S1 LIG 117 16.222 -4.253 18.092 1.00 0.00

ATOM 1925 S2 LIG 117 23.654 -2.819 16.356 1.00 0.00

ATOM 1926 O1 LIG 117 22.415 -7.199 18.932 1.00 0.00

ATOM	1927	H5	LIG	117	23.382	-7.242	18.769	1.00	0.00
ATOM	1928	O2	LIG	117	15.669	-3.995	19.576	1.00	0.00
ATOM	1929	H6	LIG	117	15.725	-4.900	20.007	1.00	0.00
ATOM	1930	O3	LIG	117	24.360	-3.638	15.167	1.00	0.00
ATOM	1931	H7	LIG	117	24.835	-4.383	15.636	1.00	0.00
ATOM	1932	O4	LIG	117	16.291	-2.962	17.439	1.00	0.00
ATOM	1933	O5	LIG	117	15.507	-5.365	17.480	1.00	0.00
ATOM	1934	O6	LIG	117	22.853	-1.784	15.736	1.00	0.00
ATOM	1935	O7	LIG	117	24.634	-2.499	17.382	1.00	0.00
ATOM	1936	O8	LIG	117	13.175	-11.527	22.461	1.00	0.00
ATOM	1937	O9	LIG	117	11.988	-9.908	23.287	1.00	0.00
ATOM	1938	N1	LIG	117	19.937	-7.573	19.803	1.00	0.00
ATOM	1939	H3	LIG	117	20.872	-7.947	19.780	1.00	0.00
ATOM	1940	H4	LIG	117	19.181	-8.076	20.244	1.00	0.00
ATOM	1941	N2	LIG	117	17.427	-6.885	19.789	1.00	0.00
ATOM	1942	N3	LIG	117	24.214	-5.863	17.726	1.00	0.00
ATOM	1943	N4	LIG	117	16.273	-6.522	20.161	1.00	0.00
ATOM	1944	N5	LIG	117	25.137	-5.496	16.948	1.00	0.00
ATOM	1945	N6	LIG	117	12.920	-10.339	22.622	1.00	0.00
ATOM	1946	C1	LIG	117	20.629	-5.688	18.426	1.00	0.00
ATOM	1947	C2	LIG	117	20.242	-4.485	17.770	1.00	0.00
ATOM	1948	C3	LIG	117	19.622	-6.462	19.115	1.00	0.00

ATOM	1949	C4	LIG	117	17.925	-4.820	18.401	1.00	0.00
ATOM	1950	C5	LIG	117	18.883	-4.082	17.765	1.00	0.00
ATOM	1951	H1	LIG	117	18.597	-3.187	17.228	1.00	0.00
ATOM	1952	C6	LIG	117	21.998	-6.055	18.352	1.00	0.00
ATOM	1953	C7	LIG	117	22.527	-4.055	17.062	1.00	0.00
ATOM	1954	C8	LIG	117	21.210	-3.685	17.116	1.00	0.00
ATOM	1955	H2	LIG	117	20.904	-2.754	16.657	1.00	0.00
ATOM	1956	C9	LIG	117	18.257	-6.021	19.108	1.00	0.00
ATOM	1957	C10	LIG	117	22.958	-5.275	17.665	1.00	0.00
ATOM	1958	C11	LIG	117	15.479	-7.521	20.756	1.00	0.00
ATOM	1959	C12	LIG	117	26.394	-6.105	17.121	1.00	0.00
ATOM	1960	C13	LIG	117	15.716	-8.897	20.596	1.00	0.00
ATOM	1961	H8	LIG	117	16.535	-9.228	19.972	1.00	0.00
ATOM	1962	C14	LIG	117	14.378	-7.080	21.504	1.00	0.00
ATOM	1963	H9	LIG	117	14.189	-6.019	21.601	1.00	0.00
ATOM	1964	C15	LIG	117	14.881	-9.817	21.207	1.00	0.00
ATOM	1965	H10	LIG	117	15.034	-10.881	21.094	1.00	0.00
ATOM	1966	C16	LIG	117	13.543	-7.998	22.124	1.00	0.00
ATOM	1967	H11	LIG	117	12.695	-7.682	22.715	1.00	0.00
ATOM	1968	C17	LIG	117	13.810	-9.355	21.970	1.00	0.00
ATOM	1969	C18	LIG	117	26.764	-6.834	18.264	1.00	0.00
ATOM	1970	H12	LIG	117	26.071	-6.927	19.090	1.00	0.00

```
ATOM   1971  C19 LIG   117      27.312  -5.925  16.077  1.00  0.00
ATOM   1972  H13 LIG   117      27.025  -5.339  15.212  1.00  0.00
ATOM   1973  C20 LIG   117      28.029  -7.397  18.336  1.00  0.00
ATOM   1974  H14 LIG   117      28.322  -7.951  19.220  1.00  0.00
ATOM   1975  C21 LIG   117      28.574  -6.502  16.154  1.00  0.00
ATOM   1976  H15 LIG   117      29.278  -6.368  15.342  1.00  0.00
ATOM   1977  C22 LIG   117      28.934  -7.240  17.281  1.00  0.00
ATOM   1978  H16 LIG   117      29.923  -7.680  17.348  1.00  0.00
TER
ATOM   1979  S1  LIG   118     -23.141  15.892 -20.995  1.00  0.00
ATOM   1980  S2  LIG   118     -19.790  22.757 -19.596  1.00  0.00
ATOM   1981  O1  LIG   118     -23.512  22.411 -23.255  1.00  0.00
ATOM   1982  H5  LIG   118     -23.268  23.359 -23.202  1.00  0.00
ATOM   1983  O2  LIG   118     -22.711  15.022 -22.273  1.00  0.00
ATOM   1984  H6  LIG   118     -23.401  15.262 -22.962  1.00  0.00
ATOM   1985  O3  LIG   118     -20.602  23.880 -18.782  1.00  0.00
ATOM   1986  H7  LIG   118     -20.996  24.459 -19.497  1.00  0.00
ATOM   1987  O4  LIG   118     -22.116  15.696 -19.990  1.00  0.00
ATOM   1988  O5  LIG   118     -24.545  15.663 -20.686  1.00  0.00
ATOM   1989  O6  LIG   118     -19.282  21.815 -18.620  1.00  0.00
ATOM   1990  O7  LIG   118     -18.907  23.391 -20.562  1.00  0.00
ATOM   1991  O8  LIG   118     -29.630  14.373 -27.066  1.00  0.00
```

ATOM	1992	O9	LIG	118	-28.349	12.629	-27.249	1.00	0.00
ATOM	1993	N1	LIG	118	-24.460	20.037	-23.967	1.00	0.00
ATOM	1994	H3	LIG	118	-24.490	21.028	-24.144	1.00	0.00
ATOM	1995	H4	LIG	118	-25.055	19.395	-24.468	1.00	0.00
ATOM	1996	N2	LIG	118	-24.683	17.484	-23.514	1.00	0.00
ATOM	1997	N3	LIG	118	-22.008	23.926	-21.871	1.00	0.00
ATOM	1998	N4	LIG	118	-24.646	16.226	-23.650	1.00	0.00
ATOM	1999	N5	LIG	118	-21.565	24.827	-21.107	1.00	0.00
ATOM	2000	N6	LIG	118	-28.597	13.753	-26.836	1.00	0.00
ATOM	2001	C1	LIG	118	-22.874	20.385	-22.152	1.00	0.00
ATOM	2002	C2	LIG	118	-22.083	19.788	-21.129	1.00	0.00
ATOM	2003	C3	LIG	118	-23.736	19.541	-22.948	1.00	0.00
ATOM	2004	C4	LIG	118	-23.002	17.598	-21.616	1.00	0.00
ATOM	2005	C5	LIG	118	-22.175	18.397	-20.877	1.00	0.00
ATOM	2006	H1	LIG	118	-21.599	17.963	-20.070	1.00	0.00
ATOM	2007	C6	LIG	118	-22.765	21.789	-22.319	1.00	0.00
ATOM	2008	C7	LIG	118	-21.107	21.935	-20.537	1.00	0.00
ATOM	2009	C8	LIG	118	-21.201	20.581	-20.355	1.00	0.00
ATOM	2010	H2	LIG	118	-20.578	20.105	-19.609	1.00	0.00
ATOM	2011	C9	LIG	118	-23.797	18.133	-22.682	1.00	0.00
ATOM	2012	C10	LIG	118	-21.913	22.586	-21.519	1.00	0.00
ATOM	2013	C11	LIG	118	-25.665	15.666	-24.445	1.00	0.00

```
ATOM   2014  C12 LIG   118     -21.650  26.153 -21.571  1.00  0.00
ATOM   2015  C13 LIG   118     -26.873  16.324 -24.732  1.00  0.00
ATOM   2016  H8  LIG   118     -27.058  17.305 -24.317  1.00  0.00
ATOM   2017  C14 LIG   118     -25.443  14.366 -24.920  1.00  0.00
ATOM   2018  H9  LIG   118     -24.520  13.857 -24.672  1.00  0.00
ATOM   2019  C15 LIG   118     -27.830  15.699 -25.515  1.00  0.00
ATOM   2020  H10 LIG   118     -28.772  16.177 -25.744  1.00  0.00
ATOM   2021  C16 LIG   118     -26.396  13.738 -25.710  1.00  0.00
ATOM   2022  H11 LIG   118     -26.242  12.741 -26.096  1.00  0.00
ATOM   2023  C17 LIG   118     -27.575  14.418 -26.000  1.00  0.00
ATOM   2024  C18 LIG   118     -21.892  26.503 -22.910  1.00  0.00
ATOM   2025  H12 LIG   118     -21.996  25.728 -23.658  1.00  0.00
ATOM   2026  C19 LIG   118     -21.447  27.154 -20.610  1.00  0.00
ATOM   2027  H13 LIG   118     -21.235  26.872 -19.586  1.00  0.00
ATOM   2028  C20 LIG   118     -21.958  27.842 -23.266  1.00  0.00
ATOM   2029  H14 LIG   118     -22.134  28.116 -24.299  1.00  0.00
ATOM   2030  C21 LIG   118     -21.526  28.493 -20.975  1.00  0.00
ATOM   2031  H15 LIG   118     -21.377  29.263 -20.228  1.00  0.00
ATOM   2032  C22 LIG   118     -21.783  28.839 -22.301  1.00  0.00
ATOM   2033  H16 LIG   118     -21.832  29.883 -22.588  1.00  0.00
TER
ATOM   2034  S1  LIG   119     -11.262  21.248  15.337  1.00  0.00
```

ATOM	2035	S2	LIG	119	-3.905	23.609	14.558	1.00	0.00
ATOM	2036	O1	LIG	119	-5.140	20.266	18.386	1.00	0.00
ATOM	2037	H5	LIG	119	-4.168	20.388	18.433	1.00	0.00
ATOM	2038	O2	LIG	119	-12.205	21.883	16.470	1.00	0.00
ATOM	2039	H6	LIG	119	-12.135	21.227	17.227	1.00	0.00
ATOM	2040	O3	LIG	119	-2.814	22.604	13.941	1.00	0.00
ATOM	2041	H7	LIG	119	-2.375	22.200	14.745	1.00	0.00
ATOM	2042	O4	LIG	119	-11.209	22.204	14.251	1.00	0.00
ATOM	2043	O5	LIG	119	-11.637	19.862	15.097	1.00	0.00
ATOM	2044	O6	LIG	119	-4.658	24.148	13.444	1.00	0.00
ATOM	2045	O7	LIG	119	-3.272	24.484	15.533	1.00	0.00
ATOM	2046	O8	LIG	119	-14.283	15.507	21.597	1.00	0.00
ATOM	2047	O9	LIG	119	-15.854	17.001	21.483	1.00	0.00
ATOM	2048	N1	LIG	119	-7.682	19.677	18.868	1.00	0.00
ATOM	2049	H3	LIG	119	-6.729	19.541	19.165	1.00	0.00
ATOM	2050	H4	LIG	119	-8.448	19.207	19.326	1.00	0.00
ATOM	2051	N2	LIG	119	-10.176	19.731	18.126	1.00	0.00
ATOM	2052	N3	LIG	119	-3.296	21.457	17.099	1.00	0.00
ATOM	2053	N4	LIG	119	-11.425	19.933	18.107	1.00	0.00
ATOM	2054	N5	LIG	119	-2.266	21.723	16.421	1.00	0.00
ATOM	2055	N6	LIG	119	-14.733	16.585	21.226	1.00	0.00
ATOM	2056	C1	LIG	119	-6.930	21.056	17.008	1.00	0.00

```
ATOM   2057  C2  LIG   119      -7.300  21.829  15.870  1.00  0.00
ATOM   2058  C3  LIG   119      -7.962  20.371  17.752  1.00  0.00
ATOM   2059  C4  LIG   119      -9.633  21.227  16.149  1.00  0.00
ATOM   2060  C5  LIG   119      -8.655  21.887  15.459  1.00  0.00
ATOM   2061  H1  LIG   119      -8.917  22.445  14.569  1.00  0.00
ATOM   2062  C6  LIG   119      -5.550  21.005  17.335  1.00  0.00
ATOM   2063  C7  LIG   119      -4.992  22.483  15.478  1.00  0.00
ATOM   2064  C8  LIG   119      -6.318  22.541  15.140  1.00  0.00
ATOM   2065  H2  LIG   119      -6.622  23.155  14.303  1.00  0.00
ATOM   2066  C9  LIG   119      -9.328  20.461  17.322  1.00  0.00
ATOM   2067  C10 LIG   119      -4.565  21.686  16.582  1.00  0.00
ATOM   2068  C11 LIG   119     -12.199  19.058  18.894  1.00  0.00
ATOM   2069  C12 LIG   119      -1.023  21.514  17.047  1.00  0.00
ATOM   2070  C13 LIG   119     -11.741  17.806  19.338  1.00  0.00
ATOM   2071  H8  LIG   119     -10.752  17.469  19.057  1.00  0.00
ATOM   2072  C14 LIG   119     -13.503  19.475  19.193  1.00  0.00
ATOM   2073  H9  LIG   119     -13.856  20.430  18.825  1.00  0.00
ATOM   2074  C15 LIG   119     -12.568  16.999  20.101  1.00  0.00
ATOM   2075  H10 LIG   119     -12.246  16.027  20.449  1.00  0.00
ATOM   2076  C16 LIG   119     -14.334  18.673  19.963  1.00  0.00
ATOM   2077  H11 LIG   119     -15.340  18.979  20.215  1.00  0.00
ATOM   2078  C17 LIG   119     -13.851  17.447  20.411  1.00  0.00
```

```
ATOM   2079  C18 LIG   119      -0.858  21.341  18.431  1.00  0.00
ATOM   2080  H12 LIG   119      -1.718  21.393  19.085  1.00  0.00
ATOM   2081  C19 LIG   119       0.097  21.515  16.202  1.00  0.00
ATOM   2082  H13 LIG   119      -0.039  21.676  15.140  1.00  0.00
ATOM   2083  C20 LIG   119       0.412  21.140  18.949  1.00  0.00
ATOM   2084  H14 LIG   119       0.544  21.016  20.018  1.00  0.00
ATOM   2085  C21 LIG   119       1.365  21.303  16.730  1.00  0.00
ATOM   2086  H15 LIG   119       2.227  21.295  16.074  1.00  0.00
ATOM   2087  C22 LIG   119       1.526  21.113  18.102  1.00  0.00
ATOM   2088  H16 LIG   119       2.515  20.960  18.516  1.00  0.00
TER
ATOM   2089  S1  LIG   120       8.140 -24.573 -19.691  1.00  0.00
ATOM   2090  S2  LIG   120       8.784 -18.132 -15.400  1.00  0.00
ATOM   2091  O1  LIG   120      11.657 -18.625 -19.743  1.00  0.00
ATOM   2092  H5  LIG   120      11.847 -17.730 -19.389  1.00  0.00
ATOM   2093  O2  LIG   120       9.253 -25.725 -19.601  1.00  0.00
ATOM   2094  H6  LIG   120       9.862 -25.523 -20.375  1.00  0.00
ATOM   2095  O3  LIG   120       8.113 -16.767 -15.919  1.00  0.00
ATOM   2096  H7  LIG   120       8.864 -16.285 -16.371  1.00  0.00
ATOM   2097  O4  LIG   120       7.290 -24.720 -18.528  1.00  0.00
ATOM   2098  O5  LIG   120       7.574 -24.531 -21.032  1.00  0.00
ATOM   2099  O6  LIG   120       7.720 -18.933 -14.830  1.00  0.00
```

```
ATOM   2100  O7  LIG   120       9.989 -17.831 -14.644  1.00  0.00
ATOM   2101  O8  LIG   120      12.656 -26.263 -27.308  1.00  0.00
ATOM   2102  O9  LIG   120      12.660 -28.177 -26.285  1.00  0.00
ATOM   2103  N1  LIG   120      11.687 -20.927 -21.063  1.00  0.00
ATOM   2104  H3  LIG   120      12.066 -19.995 -21.014  1.00  0.00
ATOM   2105  H4  LIG   120      11.940 -21.559 -21.807  1.00  0.00
ATOM   2106  N2  LIG   120      10.669 -23.285 -21.482  1.00  0.00
ATOM   2107  N3  LIG   120      10.882 -17.109 -17.852  1.00  0.00
ATOM   2108  N4  LIG   120      10.537 -24.537 -21.610  1.00  0.00
ATOM   2109  N5  LIG   120      10.407 -16.153 -17.180  1.00  0.00
ATOM   2110  N6  LIG   120      12.463 -26.970 -26.327  1.00  0.00
ATOM   2111  C1  LIG   120      10.260 -20.474 -19.143  1.00  0.00
ATOM   2112  C2  LIG   120       9.269 -20.972 -18.250  1.00  0.00
ATOM   2113  C3  LIG   120      10.712 -21.319 -20.224  1.00  0.00
ATOM   2114  C4  LIG   120       9.126 -23.057 -19.480  1.00  0.00
ATOM   2115  C5  LIG   120       8.714 -22.261 -18.448  1.00  0.00
ATOM   2116  H1  LIG   120       7.933 -22.611 -17.786  1.00  0.00
ATOM   2117  C6  LIG   120      10.738 -19.158 -18.912  1.00  0.00
ATOM   2118  C7  LIG   120       9.310 -18.918 -16.950  1.00  0.00
ATOM   2119  C8  LIG   120       8.829 -20.183 -17.159  1.00  0.00
ATOM   2120  H2  LIG   120       8.105 -20.593 -16.467  1.00  0.00
ATOM   2121  C9  LIG   120      10.144 -22.626 -20.392  1.00  0.00
```

```
ATOM   2122  C10 LIG   120      10.270 -18.356 -17.844  1.00  0.00
ATOM   2123  C11 LIG   120      11.024 -25.084 -22.813  1.00  0.00
ATOM   2124  C12 LIG   120      11.125 -14.943 -17.201  1.00  0.00
ATOM   2125  C13 LIG   120      11.254 -24.325 -23.972  1.00  0.00
ATOM   2126  H8  LIG   120      11.035 -23.266 -23.968  1.00  0.00
ATOM   2127  C14 LIG   120      11.239 -26.470 -22.827  1.00  0.00
ATOM   2128  H9  LIG   120      11.035 -27.051 -21.937  1.00  0.00
ATOM   2129  C15 LIG   120      11.725 -24.941 -25.120  1.00  0.00
ATOM   2130  H10 LIG   120      11.902 -24.383 -26.029  1.00  0.00
ATOM   2131  C16 LIG   120      11.718 -27.091 -23.972  1.00  0.00
ATOM   2132  H11 LIG   120      11.901 -28.156 -24.003  1.00  0.00
ATOM   2133  C17 LIG   120      11.959 -26.315 -25.102  1.00  0.00
ATOM   2134  C18 LIG   120      12.453 -14.824 -17.643  1.00  0.00
ATOM   2135  H12 LIG   120      12.992 -15.705 -17.968  1.00  0.00
ATOM   2136  C19 LIG   120      10.445 -13.817 -16.715  1.00  0.00
ATOM   2137  H13 LIG   120       9.429 -13.925 -16.353  1.00  0.00
ATOM   2138  C20 LIG   120      13.072 -13.584 -17.624  1.00  0.00
ATOM   2139  H14 LIG   120      14.100 -13.490 -17.955  1.00  0.00
ATOM   2140  C21 LIG   120      11.070 -12.576 -16.710  1.00  0.00
ATOM   2141  H15 LIG   120      10.539 -11.707 -16.344  1.00  0.00
ATOM   2142  C22 LIG   120      12.383 -12.456 -17.166  1.00  0.00
ATOM   2143  H16 LIG   120      12.876 -11.491 -17.151  1.00  0.00
```

TER

ATOM 2144 S1 LIG 121 -9.771 -25.003 14.510 1.00 0.00

ATOM 2145 S2 LIG 121 -4.513 -22.885 19.817 1.00 0.00

ATOM 2146 O1 LIG 121 -8.561 -25.867 21.257 1.00 0.00

ATOM 2147 H5 LIG 121 -8.000 -25.765 22.055 1.00 0.00

ATOM 2148 O2 LIG 121 -11.188 -24.253 14.424 1.00 0.00

ATOM 2149 H6 LIG 121 -11.798 -24.846 14.958 1.00 0.00

ATOM 2150 O3 LIG 121 -3.445 -23.963 20.345 1.00 0.00

ATOM 2151 H7 LIG 121 -3.848 -24.308 21.193 1.00 0.00

ATOM 2152 O4 LIG 121 -8.802 -24.142 13.864 1.00 0.00

ATOM 2153 O5 LIG 121 -9.919 -26.395 14.110 1.00 0.00

ATOM 2154 O6 LIG 121 -4.042 -22.423 18.527 1.00 0.00

ATOM 2155 O7 LIG 121 -4.832 -21.947 20.883 1.00 0.00

ATOM 2156 O8 LIG 121 -17.019 -30.130 16.075 1.00 0.00

ATOM 2157 O9 LIG 121 -17.759 -28.615 14.708 1.00 0.00

ATOM 2158 N1 LIG 121 -10.528 -26.355 19.544 1.00 0.00

ATOM 2159 H3 LIG 121 -10.198 -26.489 20.486 1.00 0.00

ATOM 2160 H4 LIG 121 -11.392 -26.768 19.227 1.00 0.00

ATOM 2161 N2 LIG 121 -11.445 -26.306 17.109 1.00 0.00

ATOM 2162 N3 LIG 121 -6.330 -24.831 21.908 1.00 0.00

ATOM 2163 N4 LIG 121 -12.170 -26.077 16.097 1.00 0.00

ATOM 2164 N5 LIG 121 -5.149 -24.646 22.309 1.00 0.00

ATOM	2165	N6	LIG	121	-16.910	-29.077	15.458	1.00	0.00
ATOM	2166	C1	LIG	121	-8.489	-25.154	18.974	1.00	0.00
ATOM	2167	C2	LIG	121	-7.750	-24.471	17.966	1.00	0.00
ATOM	2168	C3	LIG	121	-9.758	-25.751	18.624	1.00	0.00
ATOM	2169	C4	LIG	121	-9.430	-24.994	16.298	1.00	0.00
ATOM	2170	C5	LIG	121	-8.239	-24.416	16.637	1.00	0.00
ATOM	2171	H1	LIG	121	-7.648	-23.928	15.873	1.00	0.00
ATOM	2172	C6	LIG	121	-7.918	-25.209	20.271	1.00	0.00
ATOM	2173	C7	LIG	121	-5.990	-23.905	19.545	1.00	0.00
ATOM	2174	C8	LIG	121	-6.520	-23.845	18.284	1.00	0.00
ATOM	2175	H2	LIG	121	-5.991	-23.296	17.515	1.00	0.00
ATOM	2176	C9	LIG	121	-10.237	-25.665	17.274	1.00	0.00
ATOM	2177	C10	LIG	121	-6.670	-24.617	20.578	1.00	0.00
ATOM	2178	C11	LIG	121	-13.331	-26.866	15.985	1.00	0.00
ATOM	2179	C12	LIG	121	-4.909	-24.831	23.684	1.00	0.00
ATOM	2180	C13	LIG	121	-13.507	-28.087	16.659	1.00	0.00
ATOM	2181	H8	LIG	121	-12.713	-28.468	17.287	1.00	0.00
ATOM	2182	C14	LIG	121	-14.324	-26.395	15.115	1.00	0.00
ATOM	2183	H9	LIG	121	-14.169	-25.466	14.581	1.00	0.00
ATOM	2184	C15	LIG	121	-14.676	-28.808	16.487	1.00	0.00
ATOM	2185	H10	LIG	121	-14.835	-29.754	16.985	1.00	0.00
ATOM	2186	C16	LIG	121	-15.501	-27.111	14.945	1.00	0.00

```
ATOM   2187  H11 LIG   121     -16.285 -26.762  14.287  1.00  0.00
ATOM   2188  C17 LIG   121     -15.662 -28.306  15.639  1.00  0.00
ATOM   2189  C18 LIG   121      -5.920 -24.892  24.658  1.00  0.00
ATOM   2190  H12 LIG   121      -6.954 -24.766  24.367  1.00  0.00
ATOM   2191  C19 LIG   121      -3.562 -24.926  24.064  1.00  0.00
ATOM   2192  H13 LIG   121      -2.790 -24.851  23.308  1.00  0.00
ATOM   2193  C20 LIG   121      -5.577 -25.078  25.988  1.00  0.00
ATOM   2194  H14 LIG   121      -6.354 -25.115  26.743  1.00  0.00
ATOM   2195  C21 LIG   121      -3.230 -25.123  25.399  1.00  0.00
ATOM   2196  H15 LIG   121      -2.190 -25.205  25.688  1.00  0.00
ATOM   2197  C22 LIG   121      -4.235 -25.201  26.363  1.00  0.00
ATOM   2198  H16 LIG   121      -3.976 -25.342  27.406  1.00  0.00
TER
ATOM   2199  S1  LIG   122     -16.117 -18.440 -16.385  1.00  0.00
ATOM   2200  S2  LIG   122      -8.784 -17.707 -13.936  1.00  0.00
ATOM   2201  O1  LIG   122     -11.120 -13.669 -16.301  1.00  0.00
ATOM   2202  H5  LIG   122     -10.234 -13.350 -16.028  1.00  0.00
ATOM   2203  O2  LIG   122     -16.435 -18.697 -17.937  1.00  0.00
ATOM   2204  H6  LIG   122     -16.630 -17.777 -18.290  1.00  0.00
ATOM   2205  O3  LIG   122      -8.475 -16.842 -12.617  1.00  0.00
ATOM   2206  H7  LIG   122      -8.221 -15.942 -12.973  1.00  0.00
ATOM   2207  O4  LIG   122     -15.700 -19.710 -15.827  1.00  0.00
```

```
ATOM   2208  O5  LIG   122     -17.194 -17.673 -15.776  1.00  0.00
ATOM   2209  O6  LIG   122      -9.267 -18.997 -13.488  1.00  0.00
ATOM   2210  O7  LIG   122      -7.671 -17.601 -14.866  1.00  0.00
ATOM   2211  O8  LIG   122     -20.924 -12.028 -20.514  1.00  0.00
ATOM   2212  O9  LIG   122     -21.467 -13.835 -21.587  1.00  0.00
ATOM   2213  N1  LIG   122     -13.508 -13.981 -17.415  1.00  0.00
ATOM   2214  H3  LIG   122     -12.744 -13.344 -17.259  1.00  0.00
ATOM   2215  H4  LIG   122     -14.344 -13.690 -17.899  1.00  0.00
ATOM   2216  N2  LIG   122     -15.665 -15.402 -17.734  1.00  0.00
ATOM   2217  N3  LIG   122      -9.099 -14.510 -15.003  1.00  0.00
ATOM   2218  N4  LIG   122     -16.610 -16.059 -18.260  1.00  0.00
ATOM   2219  N5  LIG   122      -8.177 -14.658 -14.156  1.00  0.00
ATOM   2220  N6  LIG   122     -20.777 -13.213 -20.791  1.00  0.00
ATOM   2221  C1  LIG   122     -12.375 -15.699 -16.113  1.00  0.00
ATOM   2222  C2  LIG   122     -12.417 -17.025 -15.597  1.00  0.00
ATOM   2223  C3  LIG   122     -13.513 -15.202 -16.852  1.00  0.00
ATOM   2224  C4  LIG   122     -14.660 -17.351 -16.457  1.00  0.00
ATOM   2225  C5  LIG   122     -13.574 -17.823 -15.774  1.00  0.00
ATOM   2226  H1  LIG   122     -13.608 -18.815 -15.342  1.00  0.00
ATOM   2227  C6  LIG   122     -11.203 -14.941 -15.858  1.00  0.00
ATOM   2228  C7  LIG   122     -10.180 -16.808 -14.669  1.00  0.00
ATOM   2229  C8  LIG   122     -11.304 -17.555 -14.899  1.00  0.00
```

```
ATOM   2230  H2  LIG   122     -11.338 -18.578 -14.548  1.00  0.00
ATOM   2231  C9  LIG   122     -14.666 -16.038 -17.031  1.00  0.00
ATOM   2232  C10 LIG   122     -10.107 -15.458 -15.128  1.00  0.00
ATOM   2233  C11 LIG   122     -17.626 -15.294 -18.864  1.00  0.00
ATOM   2234  C12 LIG   122      -7.166 -13.678 -14.140  1.00  0.00
ATOM   2235  C13 LIG   122     -17.849 -13.937 -18.576  1.00  0.00
ATOM   2236  H8  LIG   122     -17.231 -13.439 -17.842  1.00  0.00
ATOM   2237  C14 LIG   122     -18.465 -15.968 -19.763  1.00  0.00
ATOM   2238  H9  LIG   122     -18.302 -17.020 -19.959  1.00  0.00
ATOM   2239  C15 LIG   122     -18.878 -13.257 -19.207  1.00  0.00
ATOM   2240  H10 LIG   122     -19.078 -12.215 -18.998  1.00  0.00
ATOM   2241  C16 LIG   122     -19.493 -15.290 -20.403  1.00  0.00
ATOM   2242  H11 LIG   122     -20.145 -15.786 -21.107  1.00  0.00
ATOM   2243  C17 LIG   122     -19.681 -13.941 -20.119  1.00  0.00
ATOM   2244  C18 LIG   122      -6.950 -12.754 -15.177  1.00  0.00
ATOM   2245  H12 LIG   122      -7.565 -12.791 -16.066  1.00  0.00
ATOM   2246  C19 LIG   122      -6.328 -13.679 -13.016  1.00  0.00
ATOM   2247  H13 LIG   122      -6.487 -14.413 -12.235  1.00  0.00
ATOM   2248  C20 LIG   122      -5.925 -11.827 -15.064  1.00  0.00
ATOM   2249  H14 LIG   122      -5.749 -11.119 -15.865  1.00  0.00
ATOM   2250  C21 LIG   122      -5.310 -12.739 -12.907  1.00  0.00
ATOM   2251  H15 LIG   122      -4.671 -12.736 -12.033  1.00  0.00
```

```
ATOM   2252  C22 LIG   122      -5.108 -11.810 -13.928  1.00  0.00
ATOM   2253  H16 LIG   122      -4.307 -11.085 -13.850  1.00  0.00
TER
ATOM   2254  S1  LIG   123     -21.049   8.985  10.753  1.00  0.00
ATOM   2255  S2  LIG   123     -27.312  13.422   9.570  1.00  0.00
ATOM   2256  O1  LIG   123     -24.147  14.465  13.601  1.00  0.00
ATOM   2257  H5  LIG   123     -24.832  15.167  13.603  1.00  0.00
ATOM   2258  O2  LIG   123     -21.058   7.853  11.889  1.00  0.00
ATOM   2259  H6  LIG   123     -20.625   8.306  12.674  1.00  0.00
ATOM   2260  O3  LIG   123     -27.131  14.898   8.959  1.00  0.00
ATOM   2261  H7  LIG   123     -27.124  15.493   9.764  1.00  0.00
ATOM   2262  O4  LIG   123     -21.775   8.447   9.620  1.00  0.00
ATOM   2263  O5  LIG   123     -19.705   9.522  10.595  1.00  0.00
ATOM   2264  O6  LIG   123     -27.218  12.494   8.461  1.00  0.00
ATOM   2265  O7  LIG   123     -28.450  13.402  10.476  1.00  0.00
ATOM   2266  O8  LIG   123     -15.052  10.058  17.397  1.00  0.00
ATOM   2267  O9  LIG   123     -15.288   7.903  17.274  1.00  0.00
ATOM   2268  N1  LIG   123     -22.177  12.795  14.211  1.00  0.00
ATOM   2269  H3  LIG   123     -22.661  13.638  14.476  1.00  0.00
ATOM   2270  H4  LIG   123     -21.370  12.470  14.721  1.00  0.00
ATOM   2271  N2  LIG   123     -20.671  10.773  13.567  1.00  0.00
ATOM   2272  N3  LIG   123     -26.125  15.213  12.186  1.00  0.00
```

```
ATOM   2273  N4  LIG   123     -20.078   9.655  13.587  1.00  0.00
ATOM   2274  N5  LIG   123     -26.915  15.872  11.456  1.00  0.00
ATOM   2275  N6  LIG   123     -15.615   9.048  16.992  1.00  0.00
ATOM   2276  C1  LIG   123     -23.611  12.558  12.258  1.00  0.00
ATOM   2277  C2  LIG   123     -23.932  11.794  11.100  1.00  0.00
ATOM   2278  C3  LIG   123     -22.491  12.151  13.074  1.00  0.00
ATOM   2279  C4  LIG   123     -22.065  10.299  11.500  1.00  0.00
ATOM   2280  C5  LIG   123     -23.136  10.677  10.740  1.00  0.00
ATOM   2281  H1  LIG   123     -23.367  10.130   9.835  1.00  0.00
ATOM   2282  C6  LIG   123     -24.422  13.689  12.533  1.00  0.00
ATOM   2283  C7  LIG   123     -25.819  13.238  10.585  1.00  0.00
ATOM   2284  C8  LIG   123     -25.045  12.145  10.298  1.00  0.00
ATOM   2285  H2  LIG   123     -25.298  11.530   9.444  1.00  0.00
ATOM   2286  C9  LIG   123     -21.713  11.006  12.695  1.00  0.00
ATOM   2287  C10 LIG   123     -25.511  14.062  11.709  1.00  0.00
ATOM   2288  C11 LIG   123     -18.965   9.569  14.445  1.00  0.00
ATOM   2289  C12 LIG   123     -27.537  16.991  12.041  1.00  0.00
ATOM   2290  C13 LIG   123     -18.272  10.691  14.931  1.00  0.00
ATOM   2291  H8  LIG   123     -18.582  11.682  14.628  1.00  0.00
ATOM   2292  C14 LIG   123     -18.529   8.278  14.775  1.00  0.00
ATOM   2293  H9  LIG   123     -19.054   7.419  14.376  1.00  0.00
ATOM   2294  C15 LIG   123     -17.179  10.520  15.764  1.00  0.00
```

```
ATOM   2295  H10 LIG   123     -16.621  11.365  16.145  1.00  0.00
ATOM   2296  C16 LIG   123     -17.439   8.101  15.615  1.00  0.00
ATOM   2297  H11 LIG   123     -17.092   7.116  15.892  1.00  0.00
ATOM   2298  C17 LIG   123     -16.782   9.228  16.102  1.00  0.00
ATOM   2299  C18 LIG   123     -27.584  17.230  13.425  1.00  0.00
ATOM   2300  H12 LIG   123     -27.148  16.515  14.109  1.00  0.00
ATOM   2301  C19 LIG   123     -28.159  17.881  11.154  1.00  0.00
ATOM   2302  H13 LIG   123     -28.139  17.673  10.091  1.00  0.00
ATOM   2303  C20 LIG   123     -28.222  18.365  13.901  1.00  0.00
ATOM   2304  H14 LIG   123     -28.270  18.548  14.968  1.00  0.00
ATOM   2305  C21 LIG   123     -28.788  19.021  11.640  1.00  0.00
ATOM   2306  H15 LIG   123     -29.259  19.711  10.952  1.00  0.00
ATOM   2307  C22 LIG   123     -28.819  19.267  13.013  1.00  0.00
ATOM   2308  H16 LIG   123     -29.319  20.149  13.394  1.00  0.00
TER
ATOM   2309  S1  LIG   124     -15.611 -25.205   7.386  1.00  0.00
ATOM   2310  S2  LIG   124     -11.234 -25.150   0.971  1.00  0.00
ATOM   2311  O1  LIG   124     -13.234 -20.659   2.756  1.00  0.00
ATOM   2312  H5  LIG   124     -12.679 -20.423   1.983  1.00  0.00
ATOM   2313  O2  LIG   124     -17.210 -25.124   7.280  1.00  0.00
ATOM   2314  H6  LIG   124     -17.403 -24.146   7.410  1.00  0.00
ATOM   2315  O3  LIG   124      -9.730 -24.588   1.046  1.00  0.00
```

ATOM	2316	H7	LIG	124	-9.812	-23.638	0.741	1.00	0.00
ATOM	2317	O4	LIG	124	-15.240	-26.572	7.082	1.00	0.00
ATOM	2318	O5	LIG	124	-15.160	-24.560	8.611	1.00	0.00
ATOM	2319	O6	LIG	124	-11.206	-26.496	1.508	1.00	0.00
ATOM	2320	O7	LIG	124	-11.797	-24.874	-0.341	1.00	0.00
ATOM	2321	O8	LIG	124	-19.427	-17.973	11.182	1.00	0.00
ATOM	2322	O9	LIG	124	-20.956	-19.503	11.363	1.00	0.00
ATOM	2323	N1	LIG	124	-14.973	-20.683	4.760	1.00	0.00
ATOM	2324	H3	LIG	124	-14.494	-20.108	4.086	1.00	0.00
ATOM	2325	H4	LIG	124	-15.587	-20.280	5.451	1.00	0.00
ATOM	2326	N2	LIG	124	-16.133	-21.960	6.708	1.00	0.00
ATOM	2327	N3	LIG	124	-11.657	-21.795	1.113	1.00	0.00
ATOM	2328	N4	LIG	124	-17.012	-22.474	7.460	1.00	0.00
ATOM	2329	N5	LIG	124	-10.649	-22.136	0.437	1.00	0.00
ATOM	2330	N6	LIG	124	-19.896	-19.076	10.926	1.00	0.00
ATOM	2331	C1	LIG	124	-13.807	-22.657	3.943	1.00	0.00
ATOM	2332	C2	LIG	124	-13.607	-24.061	4.070	1.00	0.00
ATOM	2333	C3	LIG	124	-14.697	-21.994	4.868	1.00	0.00
ATOM	2334	C4	LIG	124	-15.072	-24.154	6.000	1.00	0.00
ATOM	2335	C5	LIG	124	-14.243	-24.781	5.112	1.00	0.00
ATOM	2336	H1	LIG	124	-14.050	-25.840	5.222	1.00	0.00
ATOM	2337	C6	LIG	124	-13.103	-21.994	2.906	1.00	0.00

```
ATOM   2338  C7  LIG   124     -12.103 -24.089   2.160  1.00  0.00
ATOM   2339  C8  LIG   124     -12.768 -24.748   3.159  1.00  0.00
ATOM   2340  H2  LIG   124     -12.659 -25.822   3.246  1.00  0.00
ATOM   2341  C9  LIG   124     -15.341 -22.750   5.903  1.00  0.00
ATOM   2342  C10 LIG   124     -12.235 -22.674   2.019  1.00  0.00
ATOM   2343  C11 LIG   124     -17.689 -21.578   8.311  1.00  0.00
ATOM   2344  C12 LIG   124     -10.166 -21.202  -0.499  1.00  0.00
ATOM   2345  C13 LIG   124     -17.194 -20.308   8.650  1.00  0.00
ATOM   2346  H8  LIG   124     -16.236 -19.988   8.261  1.00  0.00
ATOM   2347  C14 LIG   124     -18.895 -22.032   8.863  1.00  0.00
ATOM   2348  H9  LIG   124     -19.256 -23.022   8.616  1.00  0.00
ATOM   2349  C15 LIG   124     -17.915 -19.490   9.503  1.00  0.00
ATOM   2350  H10 LIG   124     -17.555 -18.511   9.787  1.00  0.00
ATOM   2351  C16 LIG   124     -19.626 -21.214   9.713  1.00  0.00
ATOM   2352  H11 LIG   124     -20.564 -21.538  10.141  1.00  0.00
ATOM   2353  C17 LIG   124     -19.127 -19.951  10.016  1.00  0.00
ATOM   2354  C18 LIG   124     -10.894 -20.085  -0.944  1.00  0.00
ATOM   2355  H12 LIG   124     -11.901 -19.922  -0.582  1.00  0.00
ATOM   2356  C19 LIG   124      -8.887 -21.455  -1.016  1.00  0.00
ATOM   2357  H13 LIG   124      -8.345 -22.334  -0.685  1.00  0.00
ATOM   2358  C20 LIG   124     -10.327 -19.224  -1.870  1.00  0.00
ATOM   2359  H14 LIG   124     -10.890 -18.367  -2.222  1.00  0.00
```

ATOM 2360 C21 LIG 124 -8.324 -20.579 -1.936 1.00 0.00

ATOM 2361 H15 LIG 124 -7.332 -20.773 -2.325 1.00 0.00

ATOM 2362 C22 LIG 124 -9.040 -19.462 -2.363 1.00 0.00

ATOM 2363 H16 LIG 124 -8.607 -18.785 -3.090 1.00 0.00

TER

END

Table G: The number of atoms present in the system of BE-SIs

ATOM 1 N GLU 1 4.907 18.557 -2.106 1.00 0.00

ATOM 2 H1 GLU 1 5.217 19.172 -2.844 1.00 0.00

ATOM 3 H2 GLU 1 5.314 17.644 -2.252 1.00 0.00

ATOM 4 H3 GLU 1 5.027 19.027 -1.220 1.00 0.00

ATOM 5 CA GLU 1 3.437 18.361 -2.252 1.00 0.00

ATOM 6 HA GLU 1 3.208 17.304 -2.392 1.00 0.00

ATOM 7 CB GLU 1 2.730 18.851 -0.985 1.00 0.00

ATOM 8 HB2 GLU 1 3.151 18.345 -0.116 1.00 0.00

ATOM 9 HB3 GLU 1 2.873 19.926 -0.883 1.00 0.00

ATOM 10 CG GLU 1 1.232 18.542 -1.080 1.00 0.00

ATOM 11 HG2 GLU 1 0.835 18.986 -1.992 1.00 0.00

ATOM 12 HG3 GLU 1 1.088 17.462 -1.109 1.00 0.00

ATOM 13 CD GLU 1 0.500 19.120 0.127 1.00 0.00

ATOM 14 OE1 GLU 1 0.629 18.554 1.201 1.00 0.00

ATOM	15	OE2	GLU	1	-0.182	20.118	-0.040	1.00	0.00
ATOM	16	C	GLU	1	2.941	19.142	-3.467	1.00	0.00
ATOM	17	O	GLU	1	2.796	20.364	-3.414	1.00	0.00
ATOM	18	N	ALA	2	2.681	18.425	-4.558	1.00	0.00
ATOM	19	H	ALA	2	2.833	17.426	-4.560	1.00	0.00
ATOM	20	CA	ALA	2	2.197	19.055	-5.783	1.00	0.00
ATOM	21	HA	ALA	2	2.853	19.878	-6.066	1.00	0.00
ATOM	22	CB	ALA	2	2.173	18.035	-6.923	1.00	0.00
ATOM	23	HB1	ALA	2	1.513	17.209	-6.658	1.00	0.00
ATOM	24	HB2	ALA	2	1.809	18.513	-7.832	1.00	0.00
ATOM	25	HB3	ALA	2	3.181	17.654	-7.092	1.00	0.00
ATOM	26	C	ALA	2	0.796	19.623	-5.578	1.00	0.00
ATOM	27	O	ALA	2	0.003	19.080	-4.810	1.00	0.00
ATOM	28	N	GLU	3	0.501	20.719	-6.270	1.00	0.00
ATOM	29	H	GLU	3	1.194	21.146	-6.867	1.00	0.00
ATOM	30	CA	GLU	3	-0.808	21.357	-6.162	1.00	0.00
ATOM	31	HA	GLU	3	-1.019	21.600	-5.120	1.00	0.00
ATOM	32	CB	GLU	3	-0.833	22.655	-6.984	1.00	0.00
ATOM	33	HB2	GLU	3	-1.728	23.221	-6.726	1.00	0.00
ATOM	34	HB3	GLU	3	0.052	23.245	-6.743	1.00	0.00
ATOM	35	CG	GLU	3	-0.843	22.338	-8.487	1.00	0.00
ATOM	36	HG2	GLU	3	-1.724	21.740	-8.719	1.00	0.00

```
ATOM     37  HG3 GLU     3      -0.882  23.273  -9.045  1.00  0.00
ATOM     38  CD  GLU     3       0.415  21.563  -8.879  1.00  0.00
ATOM     39  OE1 GLU     3       1.500  22.068  -8.641  1.00  0.00
ATOM     40  OE2 GLU     3       0.274  20.473  -9.411  1.00  0.00
ATOM     41  C   GLU     3      -1.911  20.416  -6.645  1.00  0.00
ATOM     42  O   GLU     3      -3.069  20.547  -6.246  1.00  0.00
ATOM     43  N   ALA     4      -1.541  19.472  -7.508  1.00  0.00
ATOM     44  H   ALA     4      -0.585  19.424  -7.831  1.00  0.00
ATOM     45  CA  ALA     4      -2.503  18.513  -8.046  1.00  0.00
ATOM     46  HA  ALA     4      -3.400  19.042  -8.366  1.00  0.00
ATOM     47  CB  ALA     4      -1.906  17.788  -9.254  1.00  0.00
ATOM     48  HB1 ALA     4      -1.011  17.246  -8.947  1.00  0.00
ATOM     49  HB2 ALA     4      -2.637  17.085  -9.654  1.00  0.00
ATOM     50  HB3 ALA     4      -1.644  18.515 -10.022  1.00  0.00
ATOM     51  C   ALA     4      -2.917  17.503  -6.987  1.00  0.00
ATOM     52  O   ALA     4      -3.769  16.647  -7.230  1.00  0.00
ATOM     53  N   GLN     5      -2.318  17.618  -5.811  1.00  0.00
ATOM     54  H   GLN     5      -1.624  18.337  -5.664  1.00  0.00
ATOM     55  CA  GLN     5      -2.632  16.726  -4.705  1.00  0.00
ATOM     56  HA  GLN     5      -2.265  15.719  -4.908  1.00  0.00
ATOM     57  CB  GLN     5      -1.953  17.247  -3.443  1.00  0.00
ATOM     58  HB2 GLN     5      -0.877  17.301  -3.610  1.00  0.00
```

```
ATOM     59  HB3 GLN     5      -2.335  18.242  -3.214  1.00  0.00
ATOM     60  CG  GLN     5      -2.240  16.307  -2.267  1.00  0.00
ATOM     61  HG2 GLN     5      -3.312  16.131  -2.182  1.00  0.00
ATOM     62  HG3 GLN     5      -1.727  15.357  -2.420  1.00  0.00
ATOM     63  CD  GLN     5      -1.743  16.925  -0.964  1.00  0.00
ATOM     64  OE1 GLN     5      -2.131  18.041  -0.617  1.00  0.00
ATOM     65  NE2 GLN     5      -0.905  16.262  -0.216  1.00  0.00
ATOM     66 HE21 GLN     5      -0.573  16.667   0.648  1.00  0.00
ATOM     67 HE22 GLN     5      -0.591  15.346  -0.504  1.00  0.00
ATOM     68  C   GLN     5      -4.141  16.667  -4.480  1.00  0.00
ATOM     69  O   GLN     5      -4.691  15.614  -4.158  1.00  0.00
ATOM     70  N   GLU     6      -4.800  17.812  -4.647  1.00  0.00
ATOM     71  H   GLU     6      -4.298  18.656  -4.882  1.00  0.00
ATOM     72  CA  GLU     6      -6.244  17.895  -4.457  1.00  0.00
ATOM     73  HA  GLU     6      -6.498  17.703  -3.414  1.00  0.00
ATOM     74  CB  GLU     6      -6.734  19.299  -4.831  1.00  0.00
ATOM     75  HB2 GLU     6      -7.800  19.375  -4.620  1.00  0.00
ATOM     76  HB3 GLU     6      -6.193  20.038  -4.239  1.00  0.00
ATOM     77  CG  GLU     6      -6.488  19.559  -6.322  1.00  0.00
ATOM     78  HG2 GLU     6      -5.454  19.311  -6.563  1.00  0.00
ATOM     79  HG3 GLU     6      -7.158  18.933  -6.911  1.00  0.00
ATOM     80  CD  GLU     6      -6.748  21.028  -6.645  1.00  0.00
```

```
ATOM     81  OE1 GLU     6      -7.907  21.406  -6.701  1.00  0.00
ATOM     82  OE2 GLU     6      -5.784  21.752  -6.832  1.00  0.00
ATOM     83  C   GLU     6      -6.972  16.845  -5.296  1.00  0.00
ATOM     84  O   GLU     6      -7.888  16.183  -4.809  1.00  0.00
ATOM     85  N   GLU     7      -6.550  16.684  -6.551  1.00  0.00
ATOM     86  H   GLU     7      -5.802  17.255  -6.919  1.00  0.00
ATOM     87  CA  GLU     7      -7.162  15.696  -7.431  1.00  0.00
ATOM     88  HA  GLU     7      -8.212  15.941  -7.589  1.00  0.00
ATOM     89  CB  GLU     7      -6.452  15.680  -8.791  1.00  0.00
ATOM     90  HB2 GLU     7      -6.649  16.616  -9.312  1.00  0.00
ATOM     91  HB3 GLU     7      -5.378  15.569  -8.637  1.00  0.00
ATOM     92  CG  GLU     7      -6.971  14.506  -9.634  1.00  0.00
ATOM     93  HG2 GLU     7      -7.987  14.266  -9.319  1.00  0.00
ATOM     94  HG3 GLU     7      -6.974  14.791 -10.686  1.00  0.00
ATOM     95  CD  GLU     7      -6.077  13.283  -9.441  1.00  0.00
ATOM     96  OE1 GLU     7      -4.907  13.368  -9.777  1.00  0.00
ATOM     97  OE2 GLU     7      -6.576  12.277  -8.964  1.00  0.00
ATOM     98  C   GLU     7      -7.072  14.321  -6.795  1.00  0.00
ATOM     99  O   GLU     7      -8.012  13.528  -6.856  1.00  0.00
ATOM    100  N   CYX     8      -5.919  14.048  -6.193  1.00  0.00
ATOM    101  H   CYX     8      -5.169  14.725  -6.185  1.00  0.00
ATOM    102  CA  CYX     8      -5.693  12.767  -5.557  1.00  0.00
```

ATOM	103	HA	CYX	8	-5.950	11.959	-6.242	1.00	0.00
ATOM	104	CB	CYX	8	-4.218	12.628	-5.168	1.00	0.00
ATOM	105	HB2	CYX	8	-3.967	13.378	-4.418	1.00	0.00
ATOM	106	HB3	CYX	8	-4.041	11.633	-4.759	1.00	0.00
ATOM	107	SG	CYX	8	-3.181	12.867	-6.631	1.00	0.00
ATOM	108	C	CYX	8	-6.563	12.618	-4.316	1.00	0.00
ATOM	109	O	CYX	8	-7.350	11.678	-4.214	1.00	0.00
ATOM	110	N	ARG	9	-6.414	13.552	-3.380	1.00	0.00
ATOM	111	H	ARG	9	-5.739	14.293	-3.504	1.00	0.00
ATOM	112	CA	ARG	9	-7.187	13.519	-2.142	1.00	0.00
ATOM	113	HA	ARG	9	-6.921	12.638	-1.557	1.00	0.00
ATOM	114	CB	ARG	9	-6.886	14.768	-1.310	1.00	0.00
ATOM	115	HB2	ARG	9	-5.810	14.848	-1.154	1.00	0.00
ATOM	116	HB3	ARG	9	-7.242	15.650	-1.840	1.00	0.00
ATOM	117	CG	ARG	9	-7.592	14.668	0.047	1.00	0.00
ATOM	118	HG2	ARG	9	-8.671	14.641	-0.106	1.00	0.00
ATOM	119	HG3	ARG	9	-7.275	13.757	0.555	1.00	0.00
ATOM	120	CD	ARG	9	-7.229	15.882	0.904	1.00	0.00
ATOM	121	HD2	ARG	9	-7.609	15.748	1.917	1.00	0.00
ATOM	122	HD3	ARG	9	-6.146	16.003	0.936	1.00	0.00
ATOM	123	NE	ARG	9	-7.819	17.093	0.339	1.00	0.00
ATOM	124	HE	ARG	9	-8.493	17.026	-0.410	1.00	0.00

ATOM	125	CZ	ARG	9	-7.486	18.302	0.789	1.00	0.00
ATOM	126	NH1	ARG	9	-6.620	18.427	1.758	1.00	0.00
ATOM	127	HH11	ARG	9	-6.202	17.603	2.167	1.00	0.00
ATOM	128	HH12	ARG	9	-6.372	19.346	2.094	1.00	0.00
ATOM	129	NH2	ARG	9	-8.029	19.364	0.262	1.00	0.00
ATOM	130	HH21	ARG	9	-8.700	19.265	-0.486	1.00	0.00
ATOM	131	HH22	ARG	9	-7.775	20.279	0.605	1.00	0.00
ATOM	132	C	ARG	9	-8.682	13.442	-2.434	1.00	0.00
ATOM	133	O	ARG	9	-9.405	12.674	-1.802	1.00	0.00
ATOM	134	N	GLU	10	-9.140	14.240	-3.395	1.00	0.00
ATOM	135	H	GLU	10	-8.517	14.870	-3.880	1.00	0.00
ATOM	136	CA	GLU	10	-10.553	14.246	-3.760	1.00	0.00
ATOM	137	HA	GLU	10	-11.162	14.519	-2.898	1.00	0.00
ATOM	138	CB	GLU	10	-10.796	15.268	-4.875	1.00	0.00
ATOM	139	HB2	GLU	10	-10.536	16.263	-4.515	1.00	0.00
ATOM	140	HB3	GLU	10	-10.173	15.019	-5.734	1.00	0.00
ATOM	141	CG	GLU	10	-12.273	15.243	-5.289	1.00	0.00
ATOM	142	HG2	GLU	10	-12.880	14.949	-4.433	1.00	0.00
ATOM	143	HG3	GLU	10	-12.567	16.239	-5.618	1.00	0.00
ATOM	144	CD	GLU	10	-12.484	14.247	-6.429	1.00	0.00
ATOM	145	OE1	GLU	10	-11.834	14.395	-7.451	1.00	0.00
ATOM	146	OE2	GLU	10	-13.293	13.348	-6.262	1.00	0.00

ATOM	147	C	GLU	10	-10.990	12.862	-4.230	1.00	0.00
ATOM	148	O	GLU	10	-12.015	12.338	-3.790	1.00	0.00
ATOM	149	N	GLN	11	-10.203	12.283	-5.133	1.00	0.00
ATOM	150	H	GLN	11	-9.392	12.772	-5.483	1.00	0.00
ATOM	151	CA	GLN	11	-10.503	10.963	-5.677	1.00	0.00
ATOM	152	HA	GLN	11	-11.481	10.968	-6.158	1.00	0.00
ATOM	153	CB	GLN	11	-9.446	10.579	-6.719	1.00	0.00
ATOM	154	HB2	GLN	11	-9.304	11.406	-7.414	1.00	0.00
ATOM	155	HB3	GLN	11	-8.503	10.361	-6.216	1.00	0.00
ATOM	156	CG	GLN	11	-9.912	9.339	-7.490	1.00	0.00
ATOM	157	HG2	GLN	11	-9.988	8.488	-6.812	1.00	0.00
ATOM	158	HG3	GLN	11	-10.884	9.529	-7.944	1.00	0.00
ATOM	159	CD	GLN	11	-8.915	8.994	-8.596	1.00	0.00
ATOM	160	OE1	GLN	11	-7.981	9.754	-8.858	1.00	0.00
ATOM	161	NE2	GLN	11	-9.060	7.883	-9.265	1.00	0.00
ATOM	162	HE21	GLN	11	-8.405	7.648	-9.997	1.00	0.00
ATOM	163	HE22	GLN	11	-9.826	7.262	-9.047	1.00	0.00
ATOM	164	C	GLN	11	-10.562	9.903	-4.576	1.00	0.00
ATOM	165	O	GLN	11	-11.337	8.951	-4.668	1.00	0.00
ATOM	166	N	MET	12	-9.731	10.061	-3.544	1.00	0.00
ATOM	167	H	MET	12	-9.087	10.839	-3.518	1.00	0.00
ATOM	168	CA	MET	12	-9.695	9.093	-2.445	1.00	0.00

ATOM 169 HA MET 12 -9.313 8.137 -2.805 1.00 0.00
ATOM 170 CB MET 12 -8.781 9.593 -1.324 1.00 0.00
ATOM 171 HB2 MET 12 -9.068 10.606 -1.043 1.00 0.00
ATOM 172 HB3 MET 12 -8.872 8.937 -0.458 1.00 0.00
ATOM 173 CG MET 12 -7.333 9.591 -1.814 1.00 0.00
ATOM 174 HG2 MET 12 -7.048 8.581 -2.110 1.00 0.00
ATOM 175 HG3 MET 12 -7.234 10.261 -2.668 1.00 0.00
ATOM 176 SD MET 12 -6.240 10.152 -0.489 1.00 0.00
ATOM 177 CE MET 12 -4.792 10.477 -1.523 1.00 0.00
ATOM 178 HE1 MET 12 -5.041 11.229 -2.271 1.00 0.00
ATOM 179 HE2 MET 12 -3.974 10.841 -0.900 1.00 0.00
ATOM 180 HE3 MET 12 -4.486 9.556 -2.021 1.00 0.00
ATOM 181 C MET 12 -11.092 8.830 -1.888 1.00 0.00
ATOM 182 O MET 12 -11.433 7.686 -1.589 1.00 0.00
ATOM 183 N GLN 13 -11.902 9.881 -1.757 1.00 0.00
ATOM 184 H GLN 13 -11.594 10.813 -1.993 1.00 0.00
ATOM 185 CA GLN 13 -13.259 9.708 -1.244 1.00 0.00
ATOM 186 HA GLN 13 -13.219 9.302 -0.233 1.00 0.00
ATOM 187 CB GLN 13 -14.001 11.048 -1.205 1.00 0.00
ATOM 188 HB2 GLN 13 -13.833 11.578 -2.142 1.00 0.00
ATOM 189 HB3 GLN 13 -15.068 10.864 -1.078 1.00 0.00
ATOM 190 CG GLN 13 -13.488 11.898 -0.038 1.00 0.00

ATOM	191	HG2	GLN	13	-14.118	12.779	0.078	1.00	0.00
ATOM	192	HG3	GLN	13	-13.506	11.315	0.883	1.00	0.00
ATOM	193	CD	GLN	13	-12.056	12.354	-0.298	1.00	0.00
ATOM	194	OE1	GLN	13	-11.106	11.680	0.100	1.00	0.00
ATOM	195	NE2	GLN	13	-11.844	13.468	-0.943	1.00	0.00
ATOM	196	HE21	GLN	13	-10.897	13.774	-1.118	1.00	0.00
ATOM	197	HE22	GLN	13	-12.627	14.018	-1.264	1.00	0.00
ATOM	198	C	GLN	13	-14.019	8.733	-2.130	1.00	0.00
ATOM	199	O	GLN	13	-14.689	7.827	-1.639	1.00	0.00
ATOM	200	N	ARG	14	-13.873	8.908	-3.441	1.00	0.00
ATOM	201	H	ARG	14	-13.316	9.676	-3.788	1.00	0.00
ATOM	202	CA	ARG	14	-14.504	8.028	-4.402	1.00	0.00
ATOM	203	HA	ARG	14	-15.587	8.064	-4.283	1.00	0.00
ATOM	204	CB	ARG	14	-14.153	8.461	-5.830	1.00	0.00
ATOM	205	HB2	ARG	14	-14.326	9.532	-5.933	1.00	0.00
ATOM	206	HB3	ARG	14	-13.102	8.243	-6.023	1.00	0.00
ATOM	207	CG	ARG	14	-15.025	7.703	-6.837	1.00	0.00
ATOM	208	HG2	ARG	14	-14.552	7.731	-7.819	1.00	0.00
ATOM	209	HG3	ARG	14	-15.132	6.667	-6.515	1.00	0.00
ATOM	210	CD	ARG	14	-16.406	8.357	-6.917	1.00	0.00
ATOM	211	HD2	ARG	14	-17.062	7.761	-7.552	1.00	0.00
ATOM	212	HD3	ARG	14	-16.838	8.433	-5.919	1.00	0.00

ATOM	213	NE	ARG	14	-16.297	9.699	-7.484	1.00	0.00
ATOM	214	HE	ARG	14	-15.502	9.942	-8.057	1.00	0.00
ATOM	215	CZ	ARG	14	-17.229	10.625	-7.264	1.00	0.00
ATOM	216	NH1	ARG	14	-18.274	10.347	-6.532	1.00	0.00
ATOM	217	HH11	ARG	14	-18.375	9.425	-6.131	1.00	0.00
ATOM	218	HH12	ARG	14	-18.976	11.054	-6.370	1.00	0.00
ATOM	219	NH2	ARG	14	-17.097	11.816	-7.782	1.00	0.00
ATOM	220	HH21	ARG	14	-16.288	12.030	-8.347	1.00	0.00
ATOM	221	HH22	ARG	14	-17.804	12.517	-7.614	1.00	0.00
ATOM	222	C	ARG	14	-14.009	6.622	-4.146	1.00	0.00
ATOM	223	O	ARG	14	-14.779	5.661	-4.153	1.00	0.00
ATOM	224	N	GLN	15	-12.703	6.524	-3.896	1.00	0.00
ATOM	225	H	GLN	15	-12.109	7.340	-3.921	1.00	0.00
ATOM	226	CA	GLN	15	-12.098	5.248	-3.609	1.00	0.00
ATOM	227	HA	GLN	15	-12.481	4.491	-4.293	1.00	0.00
ATOM	228	CB	GLN	15	-10.575	5.335	-3.778	1.00	0.00
ATOM	229	HB2	GLN	15	-10.265	6.379	-3.731	1.00	0.00
ATOM	230	HB3	GLN	15	-10.090	4.776	-2.977	1.00	0.00
ATOM	231	CG	GLN	15	-10.170	4.743	-5.132	1.00	0.00
ATOM	232	HG2	GLN	15	-9.135	4.402	-5.091	1.00	0.00
ATOM	233	HG3	GLN	15	-10.818	3.904	-5.386	1.00	0.00
ATOM	234	CD	GLN	15	-10.292	5.801	-6.224	1.00	0.00

ATOM	235	OE1	GLN	15	-9.285	6.327	-6.695	1.00	0.00
ATOM	236	NE2	GLN	15	-11.475	6.150	-6.653	1.00	0.00
ATOM	237	HE21	GLN	15	-11.560	6.850	-7.376	1.00	0.00
ATOM	238	HE22	GLN	15	-12.299	5.718	-6.260	1.00	0.00
ATOM	239	C	GLN	15	-12.450	4.842	-2.187	1.00	0.00
ATOM	240	O	GLN	15	-11.628	4.926	-1.273	1.00	0.00
ATOM	241	N	GLN	16	-13.681	4.396	-2.022	1.00	0.00
ATOM	242	H	GLN	16	-14.319	4.374	-2.804	1.00	0.00
ATOM	243	CA	GLN	16	-14.170	3.960	-0.720	1.00	0.00
ATOM	244	HA	GLN	16	-13.759	4.592	0.068	1.00	0.00
ATOM	245	CB	GLN	16	-15.695	4.062	-0.682	1.00	0.00
ATOM	246	HB2	GLN	16	-16.127	3.442	-1.468	1.00	0.00
ATOM	247	HB3	GLN	16	-16.064	3.727	0.288	1.00	0.00
ATOM	248	CG	GLN	16	-16.097	5.521	-0.904	1.00	0.00
ATOM	249	HG2	GLN	16	-15.667	6.139	-0.115	1.00	0.00
ATOM	250	HG3	GLN	16	-15.728	5.859	-1.872	1.00	0.00
ATOM	251	CD	GLN	16	-17.614	5.682	-0.877	1.00	0.00
ATOM	252	OE1	GLN	16	-18.350	4.694	-0.861	1.00	0.00
ATOM	253	NE2	GLN	16	-18.124	6.883	-0.874	1.00	0.00
ATOM	254	HE21	GLN	16	-19.126	7.002	-0.856	1.00	0.00
ATOM	255	HE22	GLN	16	-17.513	7.687	-0.889	1.00	0.00
ATOM	256	C	GLN	16	-13.720	2.528	-0.459	1.00	0.00

ATOM	257	O	GLN	16	-14.490	1.694	0.026	1.00	0.00
ATOM	258	N	MET	17	-12.465	2.253	-0.807	1.00	0.00
ATOM	259	H	MET	17	-11.890	2.966	-1.233	1.00	0.00
ATOM	260	CA	MET	17	-11.899	0.920	-0.636	1.00	0.00
ATOM	261	HA	MET	17	-12.537	0.179	-1.118	1.00	0.00
ATOM	262	CB	MET	17	-10.504	0.854	-1.272	1.00	0.00
ATOM	263	HB2	MET	17	-10.348	-0.145	-1.680	1.00	0.00
ATOM	264	HB3	MET	17	-10.447	1.587	-2.076	1.00	0.00
ATOM	265	CG	MET	17	-9.423	1.153	-0.232	1.00	0.00
ATOM	266	HG2	MET	17	-9.753	1.954	0.429	1.00	0.00
ATOM	267	HG3	MET	17	-9.216	0.259	0.357	1.00	0.00
ATOM	268	SD	MET	17	-7.914	1.667	-1.080	1.00	0.00
ATOM	269	CE	MET	17	-8.544	3.242	-1.708	1.00	0.00
ATOM	270	HE1	MET	17	-8.847	3.872	-0.872	1.00	0.00
ATOM	271	HE2	MET	17	-7.762	3.745	-2.277	1.00	0.00
ATOM	272	HE3	MET	17	-9.402	3.059	-2.354	1.00	0.00
ATOM	273	C	MET	17	-11.817	0.529	0.831	1.00	0.00
ATOM	274	O	MET	17	-11.816	-0.653	1.159	1.00	0.00
ATOM	275	N	LEU	18	-11.735	1.530	1.699	1.00	0.00
ATOM	276	H	LEU	18	-11.720	2.484	1.368	1.00	0.00
ATOM	277	CA	LEU	18	-11.624	1.295	3.136	1.00	0.00
ATOM	278	HA	LEU	18	-10.726	0.714	3.349	1.00	0.00

ATOM	279	CB	LEU	18	-11.536	2.617	3.882	1.00	0.00
ATOM	280	HB2	LEU	18	-12.457	3.178	3.726	1.00	0.00
ATOM	281	HB3	LEU	18	-11.402	2.428	4.947	1.00	0.00
ATOM	282	CG	LEU	18	-10.349	3.429	3.355	1.00	0.00
ATOM	283	HG	LEU	18	-9.493	2.769	3.212	1.00	0.00
ATOM	284	CD1	LEU	18	-10.722	4.078	2.018	1.00	0.00
ATOM	285	HD11	LEU	18	-11.577	4.738	2.161	1.00	0.00
ATOM	286	HD12	LEU	18	-9.876	4.655	1.645	1.00	0.00
ATOM	287	HD13	LEU	18	-10.978	3.302	1.296	1.00	0.00
ATOM	288	CD2	LEU	18	-9.982	4.517	4.369	1.00	0.00
ATOM	289	HD21	LEU	18	-9.711	4.053	5.318	1.00	0.00
ATOM	290	HD22	LEU	18	-9.137	5.094	3.994	1.00	0.00
ATOM	291	HD23	LEU	18	-10.835	5.178	4.519	1.00	0.00
ATOM	292	C	LEU	18	-12.810	0.493	3.654	1.00	0.00
ATOM	293	O	LEU	18	-12.703	-0.170	4.677	1.00	0.00
ATOM	294	N	SER	19	-13.939	0.543	2.951	1.00	0.00
ATOM	295	H	SER	19	-14.017	1.112	2.120	1.00	0.00
ATOM	296	CA	SER	19	-15.106	-0.221	3.379	1.00	0.00
ATOM	297	HA	SER	19	-15.345	0.015	4.416	1.00	0.00
ATOM	298	CB	SER	19	-16.314	0.127	2.506	1.00	0.00
ATOM	299	HB2	SER	19	-16.141	-0.219	1.487	1.00	0.00
ATOM	300	HB3	SER	19	-17.206	-0.356	2.906	1.00	0.00

ATOM	301	OG	SER	19	-16.499	1.537	2.503	1.00	0.00
ATOM	302	HG	SER	19	-17.256	1.759	1.956	1.00	0.00
ATOM	303	C	SER	19	-14.797	-1.713	3.265	1.00	0.00
ATOM	304	O	SER	19	-14.911	-2.471	4.236	1.00	0.00
ATOM	305	N	HIE	20	-14.364	-2.113	2.071	1.00	0.00
ATOM	306	H	HIE	20	-14.289	-1.449	1.314	1.00	0.00
ATOM	307	CA	HIE	20	-13.997	-3.501	1.813	1.00	0.00
ATOM	308	HA	HIE	20	-14.773	-4.167	2.190	1.00	0.00
ATOM	309	CB	HIE	20	-13.838	-3.738	0.304	1.00	0.00
ATOM	310	HB2	HIE	20	-12.801	-3.702	-0.029	1.00	0.00
ATOM	311	HB3	HIE	20	-14.280	-4.690	0.011	1.00	0.00
ATOM	312	CG	HIE	20	-14.573	-2.668	-0.458	1.00	0.00
ATOM	313	ND1	HIE	20	-13.920	-1.779	-1.296	1.00	0.00
ATOM	314	CE1	HIE	20	-14.845	-0.944	-1.802	1.00	0.00
ATOM	315	HE1	HIE	20	-14.498	-0.173	-2.490	1.00	0.00
ATOM	316	NE2	HIE	20	-16.068	-1.227	-1.348	1.00	0.00
ATOM	317	HE2	HIE	20	-16.951	-0.779	-1.546	1.00	0.00
ATOM	318	CD2	HIE	20	-15.899	-2.317	-0.500	1.00	0.00
ATOM	319	HD2	HIE	20	-16.760	-2.752	0.007	1.00	0.00
ATOM	320	C	HIE	20	-12.686	-3.800	2.521	1.00	0.00
ATOM	321	O	HIE	20	-12.472	-4.895	3.040	1.00	0.00
ATOM	322	N	CYX	21	-11.819	-2.794	2.543	1.00	0.00

ATOM	323	H	CYX	21	-12.042	-1.931	2.069	1.00	0.00
ATOM	324	CA	CYX	21	-10.529	-2.906	3.195	1.00	0.00
ATOM	325	HA	CYX	21	-9.956	-3.733	2.776	1.00	0.00
ATOM	326	CB	CYX	21	-9.748	-1.604	2.969	1.00	0.00
ATOM	327	HB2	CYX	21	-10.437	-0.813	2.676	1.00	0.00
ATOM	328	HB3	CYX	21	-9.240	-1.320	3.891	1.00	0.00
ATOM	329	SG	CYX	21	-8.552	-1.864	1.634	1.00	0.00
ATOM	330	C	CYX	21	-10.731	-3.185	4.684	1.00	0.00
ATOM	331	O	CYX	21	-10.118	-4.094	5.246	1.00	0.00
ATOM	332	N	ARG	22	-11.631	-2.428	5.305	1.00	0.00
ATOM	333	H	ARG	22	-12.107	-1.686	4.813	1.00	0.00
ATOM	334	CA	ARG	22	-11.947	-2.637	6.711	1.00	0.00
ATOM	335	HA	ARG	22	-11.055	-2.485	7.319	1.00	0.00
ATOM	336	CB	ARG	22	-13.023	-1.646	7.164	1.00	0.00
ATOM	337	HB2	ARG	22	-12.724	-0.637	6.881	1.00	0.00
ATOM	338	HB3	ARG	22	-13.967	-1.895	6.680	1.00	0.00
ATOM	339	CG	ARG	22	-13.195	-1.719	8.684	1.00	0.00
ATOM	340	HG2	ARG	22	-13.414	-2.746	8.977	1.00	0.00
ATOM	341	HG3	ARG	22	-12.276	-1.392	9.171	1.00	0.00
ATOM	342	CD	ARG	22	-14.351	-0.809	9.111	1.00	0.00
ATOM	343	HD2	ARG	22	-15.292	-1.197	8.721	1.00	0.00
ATOM	344	HD3	ARG	22	-14.402	-0.760	10.199	1.00	0.00

ATOM	345	NE	ARG	22	-14.149	0.542	8.590	1.00	0.00
ATOM	346	HE	ARG	22	-13.527	1.179	9.068	1.00	0.00
ATOM	347	CZ	ARG	22	-14.771	0.966	7.487	1.00	0.00
ATOM	348	NH1	ARG	22	-15.600	0.180	6.852	1.00	0.00
ATOM	349	HH11	ARG	22	-15.771	-0.754	7.198	1.00	0.00
ATOM	350	HH12	ARG	22	-16.065	0.509	6.019	1.00	0.00
ATOM	351	NH2	ARG	22	-14.550	2.171	7.038	1.00	0.00
ATOM	352	HH21	ARG	22	-13.908	2.779	7.527	1.00	0.00
ATOM	353	HH22	ARG	22	-15.021	2.490	6.204	1.00	0.00
ATOM	354	C	ARG	22	-12.444	-4.063	6.882	1.00	0.00
ATOM	355	O	ARG	22	-12.014	-4.790	7.785	1.00	0.00
ATOM	356	N	MET	23	-13.335	-4.469	5.971	1.00	0.00
ATOM	357	H	MET	23	-13.671	-3.839	5.258	1.00	0.00
ATOM	358	CA	MET	23	-13.859	-5.824	5.996	1.00	0.00
ATOM	359	HA	MET	23	-14.409	-5.992	6.922	1.00	0.00
ATOM	360	CB	MET	23	-14.809	-6.049	4.813	1.00	0.00
ATOM	361	HB2	MET	23	-15.562	-5.261	4.800	1.00	0.00
ATOM	362	HB3	MET	23	-14.240	-6.023	3.884	1.00	0.00
ATOM	363	CG	MET	23	-15.497	-7.409	4.952	1.00	0.00
ATOM	364	HG2	MET	23	-14.746	-8.195	5.037	1.00	0.00
ATOM	365	HG3	MET	23	-16.128	-7.414	5.841	1.00	0.00
ATOM	366	SD	MET	23	-16.522	-7.719	3.492	1.00	0.00

ATOM	367	CE	MET	23	-15.175	-8.044	2.326	1.00	0.00
ATOM	368	HE1	MET	23	-14.592	-8.898	2.672	1.00	0.00
ATOM	369	HE2	MET	23	-15.590	-8.263	1.342	1.00	0.00
ATOM	370	HE3	MET	23	-14.530	-7.168	2.261	1.00	0.00
ATOM	371	C	MET	23	-12.694	-6.804	5.927	1.00	0.00
ATOM	372	O	MET	23	-12.694	-7.832	6.601	1.00	0.00
ATOM	373	N	TYR	24	-11.692	-6.455	5.116	1.00	0.00
ATOM	374	H	TYR	24	-11.766	-5.622	4.550	1.00	0.00
ATOM	375	CA	TYR	24	-10.501	-7.285	4.973	1.00	0.00
ATOM	376	HA	TYR	24	-10.777	-8.282	4.628	1.00	0.00
ATOM	377	CB	TYR	24	-9.543	-6.654	3.941	1.00	0.00
ATOM	378	HB2	TYR	24	-9.535	-7.269	3.041	1.00	0.00
ATOM	379	HB3	TYR	24	-9.922	-5.661	3.700	1.00	0.00
ATOM	380	CG	TYR	24	-8.130	-6.537	4.485	1.00	0.00
ATOM	381	CD1	TYR	24	-7.433	-7.679	4.896	1.00	0.00
ATOM	382	HD1	TYR	24	-7.907	-8.659	4.835	1.00	0.00
ATOM	383	CE1	TYR	24	-6.126	-7.563	5.386	1.00	0.00
ATOM	384	HE1	TYR	24	-5.586	-8.452	5.714	1.00	0.00
ATOM	385	CZ	TYR	24	-5.515	-6.306	5.459	1.00	0.00
ATOM	386	OH	TYR	24	-4.225	-6.192	5.935	1.00	0.00
ATOM	387	HH	TYR	24	-3.842	-7.036	6.188	1.00	0.00
ATOM	388	CE2	TYR	24	-6.209	-5.166	5.046	1.00	0.00

ATOM 389 HE2 TYR 24 -5.732 -4.188 5.103 1.00 0.00
ATOM 390 CD2 TYR 24 -7.516 -5.280 4.559 1.00 0.00
ATOM 391 HD2 TYR 24 -8.057 -4.391 4.237 1.00 0.00
ATOM 392 C TYR 24 -9.825 -7.456 6.331 1.00 0.00
ATOM 393 O TYR 24 -9.419 -8.556 6.695 1.00 0.00
ATOM 394 N MET 25 -9.719 -6.364 7.087 1.00 0.00
ATOM 395 H MET 25 -10.060 -5.470 6.766 1.00 0.00
ATOM 396 CA MET 25 -9.105 -6.436 8.410 1.00 0.00
ATOM 397 HA MET 25 -8.056 -6.720 8.320 1.00 0.00
ATOM 398 CB MET 25 -9.185 -5.074 9.101 1.00 0.00
ATOM 399 HB2 MET 25 -10.231 -4.800 9.235 1.00 0.00
ATOM 400 HB3 MET 25 -8.699 -5.136 10.075 1.00 0.00
ATOM 401 CG MET 25 -8.483 -4.011 8.246 1.00 0.00
ATOM 402 HG2 MET 25 -8.899 -4.019 7.239 1.00 0.00
ATOM 403 HG3 MET 25 -8.630 -3.028 8.693 1.00 0.00
ATOM 404 SD MET 25 -6.711 -4.366 8.159 1.00 0.00
ATOM 405 CE MET 25 -6.303 -3.918 9.864 1.00 0.00
ATOM 406 HE1 MET 25 -6.868 -4.548 10.551 1.00 0.00
ATOM 407 HE2 MET 25 -5.236 -4.064 10.033 1.00 0.00
ATOM 408 HE3 MET 25 -6.558 -2.873 10.037 1.00 0.00
ATOM 409 C MET 25 -9.850 -7.470 9.249 1.00 0.00
ATOM 410 O MET 25 -9.252 -8.388 9.830 1.00 0.00

```
ATOM    411  N   ARG    26     -11.175  -7.336   9.277  1.00  0.00
ATOM    412  H   ARG    26     -11.629  -6.568   8.805  1.00  0.00
ATOM    413  CA  ARG    26     -12.002  -8.281  10.010  1.00  0.00
ATOM    414  HA  ARG    26     -11.726  -8.277  11.065  1.00  0.00
ATOM    415  CB  ARG    26     -13.481  -7.893   9.888  1.00  0.00
ATOM    416  HB2 ARG    26     -13.612  -6.865  10.224  1.00  0.00
ATOM    417  HB3 ARG    26     -13.790  -7.977   8.846  1.00  0.00
ATOM    418  CG  ARG    26     -14.338  -8.825  10.752  1.00  0.00
ATOM    419  HG2 ARG    26     -15.390  -8.672  10.512  1.00  0.00
ATOM    420  HG3 ARG    26     -14.065  -9.860  10.544  1.00  0.00
ATOM    421  CD  ARG    26     -14.104  -8.524  12.235  1.00  0.00
ATOM    422  HD2 ARG    26     -13.042  -8.603  12.468  1.00  0.00
ATOM    423  HD3 ARG    26     -14.453  -7.519  12.470  1.00  0.00
ATOM    424  NE  ARG    26     -14.836  -9.480  13.063  1.00  0.00
ATOM    425  HE  ARG    26     -15.745  -9.809  12.770  1.00  0.00
ATOM    426  CZ  ARG    26     -14.333  -9.938  14.210  1.00  0.00
ATOM    427  NH1 ARG    26     -13.165  -9.532  14.629  1.00  0.00
ATOM    428 HH11 ARG    26     -12.638  -8.867  14.081  1.00  0.00
ATOM    429 HH12 ARG    26     -12.793  -9.886  15.499  1.00  0.00
ATOM    430  NH2 ARG    26     -15.014 -10.797  14.917  1.00  0.00
ATOM    431 HH21 ARG    26     -15.917 -11.111  14.592  1.00  0.00
ATOM    432 HH22 ARG    26     -14.634 -11.145  15.786  1.00  0.00
```

ATOM 433 C ARG 26 -11.768 -9.674 9.440 1.00 0.00
ATOM 434 O ARG 26 -11.659 -10.661 10.175 1.00 0.00
ATOM 435 N GLN 27 -11.651 -9.734 8.118 1.00 0.00
ATOM 436 H GLN 27 -11.775 -8.906 7.553 1.00 0.00
ATOM 437 CA GLN 27 -11.386 -10.987 7.448 1.00 0.00
ATOM 438 HA GLN 27 -12.180 -11.699 7.673 1.00 0.00
ATOM 439 CB GLN 27 -11.323 -10.787 5.926 1.00 0.00
ATOM 440 HB2 GLN 27 -12.301 -10.470 5.566 1.00 0.00
ATOM 441 HB3 GLN 27 -10.584 -10.019 5.696 1.00 0.00
ATOM 442 CG GLN 27 -10.926 -12.101 5.238 1.00 0.00
ATOM 443 HG2 GLN 27 -11.042 -11.995 4.160 1.00 0.00
ATOM 444 HG3 GLN 27 -9.890 -12.351 5.469 1.00 0.00
ATOM 445 CD GLN 27 -11.819 -13.241 5.714 1.00 0.00
ATOM 446 OE1 GLN 27 -12.661 -13.733 4.963 1.00 0.00
ATOM 447 NE2 GLN 27 -11.686 -13.682 6.934 1.00 0.00
ATOM 448 HE21 GLN 27 -12.271 -14.437 7.262 1.00 0.00
ATOM 449 HE22 GLN 27 -10.998 -13.267 7.546 1.00 0.00
ATOM 450 C GLN 27 -10.079 -11.558 7.968 1.00 0.00
ATOM 451 O GLN 27 -9.945 -12.756 8.166 1.00 0.00
ATOM 452 N GLN 28 -9.114 -10.696 8.224 1.00 0.00
ATOM 453 H GLN 28 -9.223 -9.704 8.072 1.00 0.00
ATOM 454 CA GLN 28 -7.859 -11.172 8.737 1.00 0.00

ATOM 455 HA GLN 28 -7.395 -11.857 8.027 1.00 0.00

ATOM 456 CB GLN 28 -6.930 -10.003 8.958 1.00 0.00

ATOM 457 HB2 GLN 28 -6.877 -9.412 8.044 1.00 0.00

ATOM 458 HB3 GLN 28 -7.319 -9.386 9.768 1.00 0.00

ATOM 459 CG GLN 28 -5.528 -10.502 9.325 1.00 0.00

ATOM 460 HG2 GLN 28 -5.288 -11.393 8.745 1.00 0.00

ATOM 461 HG3 GLN 28 -4.792 -9.725 9.118 1.00 0.00

ATOM 462 CD GLN 28 -5.461 -10.854 10.809 1.00 0.00

ATOM 463 OE1 GLN 28 -5.023 -12.029 11.171 1.00 0.00

ATOM 464 NE2 GLN 28 -5.817 -10.038 11.660 1.00 0.00

ATOM 465 HE21 GLN 28 -5.768 -10.281 12.640 1.00 0.00

ATOM 466 HE22 GLN 28 -6.155 -9.131 11.375 1.00 0.00

ATOM 467 C GLN 28 -8.101 -11.889 10.054 1.00 0.00

ATOM 468 O GLN 28 -7.494 -12.925 10.330 1.00 0.00

ATOM 469 N MET 29 -9.006 -11.333 10.862 1.00 0.00

ATOM 470 H MET 29 -9.470 -10.472 10.613 1.00 0.00

ATOM 471 CA MET 29 -9.327 -11.946 12.148 1.00 0.00

ATOM 472 HA MET 29 -8.427 -12.010 12.761 1.00 0.00

ATOM 473 CB MET 29 -10.371 -11.111 12.897 1.00 0.00

ATOM 474 HB2 MET 29 -11.182 -10.851 12.218 1.00 0.00

ATOM 475 HB3 MET 29 -10.767 -11.690 13.731 1.00 0.00

ATOM 476 CG MET 29 -9.722 -9.832 13.428 1.00 0.00

ATOM 477 HG2 MET 29 -9.342 -9.236 12.599 1.00 0.00
ATOM 478 HG3 MET 29 -10.453 -9.250 13.989 1.00 0.00
ATOM 479 SD MET 29 -8.349 -10.274 14.521 1.00 0.00
ATOM 480 CE MET 29 -9.309 -11.225 15.728 1.00 0.00
ATOM 481 HE1 MET 29 -9.791 -12.065 15.229 1.00 0.00
ATOM 482 HE2 MET 29 -8.645 -11.599 16.508 1.00 0.00
ATOM 483 HE3 MET 29 -10.068 -10.584 16.176 1.00 0.00
ATOM 484 C MET 29 -9.858 -13.361 11.943 1.00 0.00
ATOM 485 O MET 29 -9.313 -14.317 12.492 1.00 0.00
ATOM 486 N GLU 30 -10.911 -13.498 11.137 1.00 0.00
ATOM 487 H GLU 30 -11.357 -12.697 10.715 1.00 0.00
ATOM 488 CA GLU 30 -11.469 -14.825 10.869 1.00 0.00
ATOM 489 HA GLU 30 -11.676 -15.335 11.810 1.00 0.00
ATOM 490 CB GLU 30 -12.775 -14.710 10.076 1.00 0.00
ATOM 491 HB2 GLU 30 -12.609 -14.092 9.194 1.00 0.00
ATOM 492 HB3 GLU 30 -13.098 -15.704 9.767 1.00 0.00
ATOM 493 CG GLU 30 -13.857 -14.071 10.951 1.00 0.00
ATOM 494 HG2 GLU 30 -14.838 -14.422 10.632 1.00 0.00
ATOM 495 HG3 GLU 30 -13.687 -14.362 11.988 1.00 0.00
ATOM 496 CD GLU 30 -13.798 -12.552 10.834 1.00 0.00
ATOM 497 OE1 GLU 30 -13.939 -12.057 9.727 1.00 0.00
ATOM 498 OE2 GLU 30 -13.621 -11.905 11.854 1.00 0.00

ATOM	499	C	GLU	30	-10.470	-15.677	10.087	1.00	0.00
ATOM	500	O	GLU	30	-10.287	-16.862	10.364	1.00	0.00
ATOM	501	N	GLU	31	-9.836	-15.047	9.100	1.00	0.00
ATOM	502	H	GLU	31	-10.057	-14.081	8.909	1.00	0.00
ATOM	503	CA	GLU	31	-8.854	-15.715	8.248	1.00	0.00
ATOM	504	HA	GLU	31	-9.264	-16.647	7.858	1.00	0.00
ATOM	505	CB	GLU	31	-8.494	-14.806	7.067	1.00	0.00
ATOM	506	HB2	GLU	31	-9.402	-14.345	6.680	1.00	0.00
ATOM	507	HB3	GLU	31	-7.810	-14.029	7.408	1.00	0.00
ATOM	508	CG	GLU	31	-7.823	-15.626	5.957	1.00	0.00
ATOM	509	HG2	GLU	31	-6.965	-16.152	6.375	1.00	0.00
ATOM	510	HG3	GLU	31	-8.540	-16.351	5.571	1.00	0.00
ATOM	511	CD	GLU	31	-7.357	-14.714	4.819	1.00	0.00
ATOM	512	OE1	GLU	31	-7.498	-13.506	4.945	1.00	0.00
ATOM	513	OE2	GLU	31	-6.863	-15.240	3.834	1.00	0.00
ATOM	514	C	GLU	31	-7.589	-16.079	9.033	1.00	0.00
ATOM	515	O	GLU	31	-6.683	-16.705	8.491	1.00	0.00
ATOM	516	N	SER	32	-7.528	-15.673	10.303	1.00	0.00
ATOM	517	H	SER	32	-8.288	-15.140	10.701	1.00	0.00
ATOM	518	CA	SER	32	-6.360	-15.952	11.145	1.00	0.00
ATOM	519	HA	SER	32	-5.533	-15.300	10.864	1.00	0.00
ATOM	520	CB	SER	32	-6.701	-15.703	12.614	1.00	0.00

```
ATOM    521  HB2 SER    32      -5.817 -15.878  13.228  1.00  0.00
ATOM    522  HB3 SER    32      -7.033 -14.673  12.742  1.00  0.00
ATOM    523  OG  SER    32      -7.739 -16.589  13.014  1.00  0.00
ATOM    524  HG  SER    32      -7.954 -16.434  13.937  1.00  0.00
ATOM    525  C   SER    32      -5.891 -17.393  10.964  1.00  0.00
ATOM    526  O   SER    32      -4.733 -17.715  11.229  1.00  0.00
ATOM    527  N   THR    33      -6.799 -18.251  10.512  1.00  0.00
ATOM    528  H   THR    33      -7.748 -17.947  10.350  1.00  0.00
ATOM    529  CA  THR    33      -6.471 -19.649  10.269  1.00  0.00
ATOM    530  HA  THR    33      -5.839 -20.034  11.070  1.00  0.00
ATOM    531  CB  THR    33      -7.758 -20.480  10.219  1.00  0.00
ATOM    532  HB  THR    33      -7.514 -21.511   9.960  1.00  0.00
ATOM    533  CG2 THR    33      -8.446 -20.447  11.586  1.00  0.00
ATOM    534 HG21 THR    33      -8.691 -19.417  11.846  1.00  0.00
ATOM    535 HG22 THR    33      -9.360 -21.039  11.548  1.00  0.00
ATOM    536 HG23 THR    33      -7.777 -20.862  12.340  1.00  0.00
ATOM    537  OG1 THR    33      -8.632 -19.940   9.236  1.00  0.00
ATOM    538  HG1 THR    33      -9.438 -20.460   9.205  1.00  0.00
ATOM    539  C   THR    33      -5.694 -19.823   8.952  1.00  0.00
ATOM    540  O   THR    33      -4.587 -20.361   8.937  1.00  0.00
ATOM    541  N   TYR    34      -6.323 -19.402   7.845  1.00  0.00
ATOM    542  H   TYR    34      -7.245 -18.999   7.929  1.00  0.00
```

ATOM	543	CA	TYR	34	-5.743	-19.545	6.498	1.00	0.00
ATOM	544	HA	TYR	34	-4.956	-20.300	6.501	1.00	0.00
ATOM	545	CB	TYR	34	-6.838	-19.983	5.521	1.00	0.00
ATOM	546	HB2	TYR	34	-7.607	-19.213	5.465	1.00	0.00
ATOM	547	HB3	TYR	34	-6.401	-20.132	4.534	1.00	0.00
ATOM	548	CG	TYR	34	-7.455	-21.277	5.999	1.00	0.00
ATOM	549	CD1	TYR	34	-6.777	-22.488	5.812	1.00	0.00
ATOM	550	HD1	TYR	34	-5.804	-22.498	5.320	1.00	0.00
ATOM	551	CE1	TYR	34	-7.348	-23.686	6.257	1.00	0.00
ATOM	552	HE1	TYR	34	-6.820	-24.629	6.111	1.00	0.00
ATOM	553	CZ	TYR	34	-8.597	-23.674	6.890	1.00	0.00
ATOM	554	OH	TYR	34	-9.159	-24.855	7.328	1.00	0.00
ATOM	555	HH	TYR	34	-8.609	-25.622	7.148	1.00	0.00
ATOM	556	CE2	TYR	34	-9.274	-22.463	7.077	1.00	0.00
ATOM	557	HE2	TYR	34	-10.246	-22.454	7.570	1.00	0.00
ATOM	558	CD2	TYR	34	-8.704	-21.266	6.633	1.00	0.00
ATOM	559	HD2	TYR	34	-9.231	-20.324	6.780	1.00	0.00
ATOM	560	C	TYR	34	-5.078	-18.267	5.957	1.00	0.00
ATOM	561	O	TYR	34	-4.694	-18.235	4.787	1.00	0.00
ATOM	562	N	GLN	35	-4.988	-17.213	6.771	1.00	0.00
ATOM	563	H	GLN	35	-5.343	-17.269	7.715	1.00	0.00
ATOM	564	CA	GLN	35	-4.408	-15.933	6.316	1.00	0.00

ATOM	565	HA	GLN	35	-5.178	-15.348	5.815	1.00	0.00
ATOM	566	CB	GLN	35	-3.856	-15.114	7.489	1.00	0.00
ATOM	567	HB2	GLN	35	-3.651	-14.100	7.147	1.00	0.00
ATOM	568	HB3	GLN	35	-4.602	-15.086	8.284	1.00	0.00
ATOM	569	CG	GLN	35	-2.561	-15.744	8.025	1.00	0.00
ATOM	570	HG2	GLN	35	-1.841	-15.872	7.216	1.00	0.00
ATOM	571	HG3	GLN	35	-2.131	-15.103	8.795	1.00	0.00
ATOM	572	CD	GLN	35	-2.853	-17.107	8.635	1.00	0.00
ATOM	573	OE1	GLN	35	-4.056	-17.365	9.052	1.00	0.00
ATOM	574	NE2	GLN	35	-1.967	-17.955	8.728	1.00	0.00
ATOM	575	HE21	GLN	35	-2.175	-18.856	9.135	1.00	0.00
ATOM	576	HE22	GLN	35	-1.036	-17.747	8.397	1.00	0.00
ATOM	577	C	GLN	35	-3.284	-16.116	5.307	1.00	0.00
ATOM	578	O	GLN	35	-3.092	-15.260	4.442	1.00	0.00
ATOM	579	N	THR	36	-2.536	-17.210	5.409	1.00	0.00
ATOM	580	H	THR	36	-2.691	-17.903	6.127	1.00	0.00
ATOM	581	CA	THR	36	-1.446	-17.420	4.474	1.00	0.00
ATOM	582	HA	THR	36	-0.723	-16.608	4.553	1.00	0.00
ATOM	583	CB	THR	36	-0.734	-18.741	4.784	1.00	0.00
ATOM	584	HB	THR	36	-1.410	-19.572	4.582	1.00	0.00
ATOM	585	CG2	THR	36	0.514	-18.874	3.907	1.00	0.00
ATOM	586	HG21	THR	36	1.191	-18.044	4.109	1.00	0.00

ATOM 587 HG22 THR 36 1.018 -19.815 4.131 1.00 0.00
ATOM 588 HG23 THR 36 0.224 -18.859 2.857 1.00 0.00
ATOM 589 OG1 THR 36 -0.356 -18.761 6.153 1.00 0.00
ATOM 590 HG1 THR 36 0.091 -19.588 6.350 1.00 0.00
ATOM 591 C THR 36 -2.008 -17.449 3.059 1.00 0.00
ATOM 592 O THR 36 -2.668 -18.406 2.655 1.00 0.00
ATOM 593 N MET 37 -1.748 -16.373 2.325 1.00 0.00
ATOM 594 H MET 37 -1.219 -15.608 2.719 1.00 0.00
ATOM 595 CA MET 37 -2.235 -16.244 0.955 1.00 0.00
ATOM 596 HA MET 37 -3.321 -16.328 0.933 1.00 0.00
ATOM 597 CB MET 37 -1.838 -14.876 0.393 1.00 0.00
ATOM 598 HB2 MET 37 -0.761 -14.740 0.498 1.00 0.00
ATOM 599 HB3 MET 37 -2.109 -14.825 -0.661 1.00 0.00
ATOM 600 CG MET 37 -2.570 -13.771 1.162 1.00 0.00
ATOM 601 HG2 MET 37 -2.557 -13.995 2.229 1.00 0.00
ATOM 602 HG3 MET 37 -2.079 -12.814 0.987 1.00 0.00
ATOM 603 SD MET 37 -4.287 -13.673 0.594 1.00 0.00
ATOM 604 CE MET 37 -4.007 -12.544 -0.792 1.00 0.00
ATOM 605 HE1 MET 37 -3.311 -12.999 -1.497 1.00 0.00
ATOM 606 HE2 MET 37 -4.953 -12.343 -1.294 1.00 0.00
ATOM 607 HE3 MET 37 -3.588 -11.609 -0.420 1.00 0.00
ATOM 608 C MET 37 -1.671 -17.349 0.062 1.00 0.00

ATOM	609	O	MET	37	-0.465	-17.405	-0.179	1.00	0.00
ATOM	610	N	PRO	38	-2.519	-18.216	-0.440	1.00	0.00
ATOM	611	CD	PRO	38	-3.976	-18.234	-0.212	1.00	0.00
ATOM	612	HD2	PRO	38	-4.413	-17.332	-0.638	1.00	0.00
ATOM	613	HD3	PRO	38	-4.167	-18.259	0.861	1.00	0.00
ATOM	614	CG	PRO	38	-4.450	-19.510	-0.920	1.00	0.00
ATOM	615	HG2	PRO	38	-4.958	-19.235	-1.844	1.00	0.00
ATOM	616	HG3	PRO	38	-5.144	-20.041	-0.268	1.00	0.00
ATOM	617	CB	PRO	38	-3.235	-20.336	-1.190	1.00	0.00
ATOM	618	HB2	PRO	38	-3.386	-20.863	-2.132	1.00	0.00
ATOM	619	HB3	PRO	38	-2.996	-21.056	-0.407	1.00	0.00
ATOM	620	CA	PRO	38	-2.099	-19.328	-1.335	1.00	0.00
ATOM	621	HA	PRO	38	-1.139	-19.709	-0.986	1.00	0.00
ATOM	622	C	PRO	38	-1.965	-18.862	-2.782	1.00	0.00
ATOM	623	O	PRO	38	-2.731	-18.013	-3.240	1.00	0.00
ATOM	624	N	ARG	39	-0.995	-19.422	-3.498	1.00	0.00
ATOM	625	H	ARG	39	-0.399	-20.133	-3.098	1.00	0.00
ATOM	626	CA	ARG	39	-0.786	-19.048	-4.894	1.00	0.00
ATOM	627	HA	ARG	39	-0.567	-17.983	-4.961	1.00	0.00
ATOM	628	CB	ARG	39	0.384	-19.824	-5.487	1.00	0.00
ATOM	629	HB2	ARG	39	0.118	-20.879	-5.555	1.00	0.00
ATOM	630	HB3	ARG	39	0.603	-19.440	-6.483	1.00	0.00

ATOM 631 CG ARG 39 1.621 -19.667 -4.597 1.00 0.00
ATOM 632 HG2 ARG 39 1.842 -18.608 -4.466 1.00 0.00
ATOM 633 HG3 ARG 39 1.429 -20.121 -3.624 1.00 0.00
ATOM 634 CD ARG 39 2.817 -20.360 -5.255 1.00 0.00
ATOM 635 HD2 ARG 39 2.550 -21.382 -5.525 1.00 0.00
ATOM 636 HD3 ARG 39 3.115 -19.814 -6.150 1.00 0.00
ATOM 637 NE ARG 39 3.949 -20.401 -4.334 1.00 0.00
ATOM 638 HE ARG 39 4.713 -19.749 -4.441 1.00 0.00
ATOM 639 CZ ARG 39 4.005 -21.288 -3.340 1.00 0.00
ATOM 640 NH1 ARG 39 3.035 -22.145 -3.169 1.00 0.00
ATOM 641 HH11 ARG 39 2.241 -22.135 -3.792 1.00 0.00
ATOM 642 HH12 ARG 39 3.084 -22.814 -2.414 1.00 0.00
ATOM 643 NH2 ARG 39 5.033 -21.298 -2.536 1.00 0.00
ATOM 644 HH21 ARG 39 5.782 -20.634 -2.670 1.00 0.00
ATOM 645 HH22 ARG 39 5.075 -21.970 -1.783 1.00 0.00
ATOM 646 C ARG 39 -2.018 -19.389 -5.708 1.00 0.00
ATOM 647 O ARG 39 -2.420 -18.638 -6.597 1.00 0.00
ATOM 648 N ARG 40 -2.608 -20.543 -5.401 1.00 0.00
ATOM 649 H ARG 40 -2.230 -21.143 -4.681 1.00 0.00
ATOM 650 CA ARG 40 -3.785 -20.989 -6.118 1.00 0.00
ATOM 651 HA ARG 40 -3.616 -20.913 -7.192 1.00 0.00
ATOM 652 CB ARG 40 -4.096 -22.447 -5.775 1.00 0.00

ATOM	653	HB2	ARG	40	-4.114	-22.570	-4.692	1.00	0.00
ATOM	654	HB3	ARG	40	-5.067	-22.719	-6.187	1.00	0.00
ATOM	655	CG	ARG	40	-3.017	-23.353	-6.373	1.00	0.00
ATOM	656	HG2	ARG	40	-2.035	-22.926	-6.170	1.00	0.00
ATOM	657	HG3	ARG	40	-3.083	-24.341	-5.917	1.00	0.00
ATOM	658	CD	ARG	40	-3.218	-23.475	-7.888	1.00	0.00
ATOM	659	HD2	ARG	40	-3.204	-22.485	-8.343	1.00	0.00
ATOM	660	HD3	ARG	40	-2.422	-24.083	-8.318	1.00	0.00
ATOM	661	NE	ARG	40	-4.501	-24.108	-8.182	1.00	0.00
ATOM	662	HE	ARG	40	-5.294	-23.542	-8.445	1.00	0.00
ATOM	663	CZ	ARG	40	-4.658	-25.429	-8.112	1.00	0.00
ATOM	664	NH1	ARG	40	-3.656	-26.194	-7.770	1.00	0.00
ATOM	665	HH11	ARG	40	-2.759	-25.782	-7.558	1.00	0.00
ATOM	666	HH12	ARG	40	-3.783	-27.195	-7.719	1.00	0.00
ATOM	667	NH2	ARG	40	-5.818	-25.961	-8.386	1.00	0.00
ATOM	668	HH21	ARG	40	-6.592	-25.369	-8.650	1.00	0.00
ATOM	669	HH22	ARG	40	-5.936	-26.963	-8.332	1.00	0.00
ATOM	670	C	ARG	40	-4.970	-20.111	-5.777	1.00	0.00
ATOM	671	O	ARG	40	-5.616	-20.274	-4.740	1.00	0.00
ATOM	672	N	GLY	41	-5.239	-19.187	-6.678	1.00	0.00
ATOM	673	H	GLY	41	-4.664	-19.111	-7.505	1.00	0.00
ATOM	674	CA	GLY	41	-6.355	-18.256	-6.521	1.00	0.00

ATOM	675	HA2	GLY	41	-6.292	-17.495	-7.298	1.00	0.00
ATOM	676	HA3	GLY	41	-7.290	-18.805	-6.626	1.00	0.00
ATOM	677	C	GLY	41	-6.329	-17.575	-5.155	1.00	0.00
ATOM	678	O	GLY	41	-6.985	-18.027	-4.215	1.00	0.00
ATOM	679	N	MET	42	-5.573	-16.482	-5.055	1.00	0.00
ATOM	680	H	MET	42	-5.040	-16.157	-5.849	1.00	0.00
ATOM	681	CA	MET	42	-5.470	-15.738	-3.799	1.00	0.00
ATOM	682	HA	MET	42	-5.024	-16.366	-3.027	1.00	0.00
ATOM	683	CB	MET	42	-4.585	-14.503	-3.997	1.00	0.00
ATOM	684	HB2	MET	42	-4.959	-13.920	-4.838	1.00	0.00
ATOM	685	HB3	MET	42	-4.607	-13.893	-3.094	1.00	0.00
ATOM	686	CG	MET	42	-3.146	-14.941	-4.281	1.00	0.00
ATOM	687	HG2	MET	42	-2.788	-15.580	-3.473	1.00	0.00
ATOM	688	HG3	MET	42	-3.106	-15.490	-5.222	1.00	0.00
ATOM	689	SD	MET	42	-2.084	-13.481	-4.399	1.00	0.00
ATOM	690	CE	MET	42	-0.557	-14.344	-4.846	1.00	0.00
ATOM	691	HE1	MET	42	-0.705	-14.886	-5.780	1.00	0.00
ATOM	692	HE2	MET	42	0.248	-13.619	-4.971	1.00	0.00
ATOM	693	HE3	MET	42	-0.292	-15.048	-4.056	1.00	0.00
ATOM	694	C	MET	42	-6.853	-15.304	-3.314	1.00	0.00
ATOM	695	O	MET	42	-7.846	-15.450	-4.028	1.00	0.00
ATOM	696	N	GLU	43	-6.911	-14.772	-2.094	1.00	0.00

ATOM	697	H	GLU	43	-6.077	-14.685	-1.530	1.00	0.00
ATOM	698	CA	GLU	43	-8.181	-14.327	-1.526	1.00	0.00
ATOM	699	HA	GLU	43	-8.929	-15.114	-1.622	1.00	0.00
ATOM	700	CB	GLU	43	-8.004	-13.999	-0.039	1.00	0.00
ATOM	701	HB2	GLU	43	-7.407	-14.778	0.436	1.00	0.00
ATOM	702	HB3	GLU	43	-7.496	-13.040	0.064	1.00	0.00
ATOM	703	CG	GLU	43	-9.377	-13.925	0.637	1.00	0.00
ATOM	704	HG2	GLU	43	-9.959	-13.119	0.192	1.00	0.00
ATOM	705	HG3	GLU	43	-9.894	-14.873	0.485	1.00	0.00
ATOM	706	CD	GLU	43	-9.215	-13.669	2.134	1.00	0.00
ATOM	707	OE1	GLU	43	-8.692	-12.625	2.484	1.00	0.00
ATOM	708	OE2	GLU	43	-9.625	-14.518	2.909	1.00	0.00
ATOM	709	C	GLU	43	-8.697	-13.093	-2.269	1.00	0.00
ATOM	710	O	GLU	43	-7.973	-12.110	-2.429	1.00	0.00
ATOM	711	N	PRO	44	-9.927	-13.134	-2.728	1.00	0.00
ATOM	712	CD	PRO	44	-10.859	-14.264	-2.578	1.00	0.00
ATOM	713	HD2	PRO	44	-10.851	-14.600	-1.541	1.00	0.00
ATOM	714	HD3	PRO	44	-10.539	-15.081	-3.226	1.00	0.00
ATOM	715	CG	PRO	44	-12.207	-13.683	-2.996	1.00	0.00
ATOM	716	HG2	PRO	44	-12.744	-13.351	-2.108	1.00	0.00
ATOM	717	HG3	PRO	44	-12.787	-14.454	-3.504	1.00	0.00
ATOM	718	CB	PRO	44	-11.915	-12.544	-3.914	1.00	0.00

```
ATOM    719  HB2 PRO    44     -12.677 -11.775  -3.787  1.00  0.00
ATOM    720  HB3 PRO    44     -11.877 -12.850  -4.959  1.00  0.00
ATOM    721  CA  PRO    44     -10.549 -12.000  -3.479  1.00  0.00
ATOM    722  HA  PRO    44      -9.912 -11.775  -4.334  1.00  0.00
ATOM    723  C   PRO    44     -10.711 -10.747  -2.621  1.00  0.00
ATOM    724  O   PRO    44     -10.763  -9.633  -3.146  1.00  0.00
ATOM    725  N   HIE    45     -10.795 -10.934  -1.306  1.00  0.00
ATOM    726  H   HIE    45     -10.767 -11.865  -0.916  1.00  0.00
ATOM    727  CA  HIE    45     -10.957  -9.805  -0.393  1.00  0.00
ATOM    728  HA  HIE    45     -11.930  -9.340  -0.546  1.00  0.00
ATOM    729  CB  HIE    45     -10.866 -10.283   1.059  1.00  0.00
ATOM    730  HB2 HIE    45      -9.948 -10.837   1.256  1.00  0.00
ATOM    731  HB3 HIE    45     -10.951  -9.459   1.767  1.00  0.00
ATOM    732  CG  HIE    45     -12.006 -11.221   1.351  1.00  0.00
ATOM    733  ND1 HIE    45     -11.800 -12.531   1.753  1.00  0.00
ATOM    734  CE1 HIE    45     -13.008 -13.097   1.926  1.00  0.00
ATOM    735  HE1 HIE    45     -13.030 -14.139   2.245  1.00  0.00
ATOM    736  NE2 HIE    45     -13.998 -12.240   1.664  1.00  0.00
ATOM    737  HE2 HIE    45     -14.998 -12.376   1.706  1.00  0.00
ATOM    738  CD2 HIE    45     -13.368 -11.055   1.301  1.00  0.00
ATOM    739  HD2 HIE    45     -13.962 -10.182   1.034  1.00  0.00
ATOM    740  C   HIE    45      -9.885  -8.749  -0.652  1.00  0.00
```

```
ATOM    741  O   HIE    45     -10.183  -7.555  -0.723  1.00  0.00
ATOM    742  N   MET    46      -8.641  -9.198  -0.803  1.00  0.00
ATOM    743  H   MET    46      -8.438 -10.185  -0.735  1.00  0.00
ATOM    744  CA  MET    46      -7.536  -8.281  -1.065  1.00  0.00
ATOM    745  HA  MET    46      -7.507  -7.504  -0.301  1.00  0.00
ATOM    746  CB  MET    46      -6.205  -9.041  -1.040  1.00  0.00
ATOM    747  HB2 MET    46      -6.178  -9.741  -1.875  1.00  0.00
ATOM    748  HB3 MET    46      -5.385  -8.329  -1.136  1.00  0.00
ATOM    749  CG  MET    46      -6.059  -9.811   0.275  1.00  0.00
ATOM    750  HG2 MET    46      -6.777 -10.631   0.300  1.00  0.00
ATOM    751  HG3 MET    46      -5.048 -10.212   0.353  1.00  0.00
ATOM    752  SD  MET    46      -6.369  -8.706   1.674  1.00  0.00
ATOM    753  CE  MET    46      -6.975  -9.970   2.820  1.00  0.00
ATOM    754  HE1 MET    46      -6.198 -10.719   2.980  1.00  0.00
ATOM    755  HE2 MET    46      -7.232  -9.505   3.772  1.00  0.00
ATOM    756  HE3 MET    46      -7.859 -10.449   2.399  1.00  0.00
ATOM    757  C   MET    46      -7.712  -7.628  -2.435  1.00  0.00
ATOM    758  O   MET    46      -7.497  -6.427  -2.599  1.00  0.00
ATOM    759  N   SER    47      -8.105  -8.444  -3.411  1.00  0.00
ATOM    760  H   SER    47      -8.244  -9.426  -3.216  1.00  0.00
ATOM    761  CA  SER    47      -8.313  -7.973  -4.778  1.00  0.00
ATOM    762  HA  SER    47      -7.385  -7.565  -5.179  1.00  0.00
```

ATOM	763	CB	SER	47	-8.759	-9.137	-5.665	1.00	0.00
ATOM	764	HB2	SER	47	-9.751	-9.469	-5.360	1.00	0.00
ATOM	765	HB3	SER	47	-8.788	-8.813	-6.705	1.00	0.00
ATOM	766	OG	SER	47	-7.838	-10.211	-5.530	1.00	0.00
ATOM	767	HG	SER	47	-8.116	-10.943	-6.086	1.00	0.00
ATOM	768	C	SER	47	-9.357	-6.860	-4.837	1.00	0.00
ATOM	769	O	SER	47	-9.274	-5.967	-5.680	1.00	0.00
ATOM	770	N	GLU	48	-10.348	-6.929	-3.952	1.00	0.00
ATOM	771	H	GLU	48	-10.396	-7.691	-3.290	1.00	0.00
ATOM	772	CA	GLU	48	-11.410	-5.926	-3.933	1.00	0.00
ATOM	773	HA	GLU	48	-11.974	-5.961	-4.864	1.00	0.00
ATOM	774	CB	GLU	48	-12.370	-6.202	-2.767	1.00	0.00
ATOM	775	HB2	GLU	48	-12.194	-7.209	-2.387	1.00	0.00
ATOM	776	HB3	GLU	48	-12.189	-5.478	-1.973	1.00	0.00
ATOM	777	CG	GLU	48	-13.823	-6.083	-3.246	1.00	0.00
ATOM	778	HG2	GLU	48	-13.871	-6.339	-4.304	1.00	0.00
ATOM	779	HG3	GLU	48	-14.437	-6.780	-2.676	1.00	0.00
ATOM	780	CD	GLU	48	-14.344	-4.661	-3.042	1.00	0.00
ATOM	781	OE1	GLU	48	-13.596	-3.729	-3.288	1.00	0.00
ATOM	782	OE2	GLU	48	-15.490	-4.524	-2.645	1.00	0.00
ATOM	783	C	GLU	48	-10.822	-4.522	-3.799	1.00	0.00
ATOM	784	O	GLU	48	-11.111	-3.641	-4.610	1.00	0.00

ATOM	785	N	CYX	49	-9.989	-4.324	-2.781	1.00	0.00
ATOM	786	H	CYX	49	-9.799	-5.064	-2.120	1.00	0.00
ATOM	787	CA	CYX	49	-9.360	-3.024	-2.564	1.00	0.00
ATOM	788	HA	CYX	49	-10.114	-2.238	-2.584	1.00	0.00
ATOM	789	CB	CYX	49	-8.657	-2.996	-1.196	1.00	0.00
ATOM	790	HB2	CYX	49	-8.451	-4.016	-0.871	1.00	0.00
ATOM	791	HB3	CYX	49	-7.719	-2.447	-1.280	1.00	0.00
ATOM	792	SG	CYX	49	-9.725	-2.169	0.011	1.00	0.00
ATOM	793	C	CYX	49	-8.354	-2.716	-3.675	1.00	0.00
ATOM	794	O	CYX	49	-8.153	-1.556	-4.028	1.00	0.00
ATOM	795	N	CYX	50	-7.725	-3.764	-4.213	1.00	0.00
ATOM	796	H	CYX	50	-7.921	-4.696	-3.874	1.00	0.00
ATOM	797	CA	CYX	50	-6.726	-3.606	-5.276	1.00	0.00
ATOM	798	HA	CYX	50	-5.838	-3.110	-4.883	1.00	0.00
ATOM	799	CB	CYX	50	-6.319	-4.974	-5.822	1.00	0.00
ATOM	800	HB2	CYX	50	-6.047	-5.630	-4.995	1.00	0.00
ATOM	801	HB3	CYX	50	-7.153	-5.410	-6.371	1.00	0.00
ATOM	802	SG	CYX	50	-4.927	-4.779	-6.967	1.00	0.00
ATOM	803	C	CYX	50	-7.239	-2.753	-6.433	1.00	0.00
ATOM	804	O	CYX	50	-6.517	-1.900	-6.950	1.00	0.00
ATOM	805	N	GLU	51	-8.475	-3.002	-6.853	1.00	0.00
ATOM	806	H	GLU	51	-9.028	-3.731	-6.425	1.00	0.00

```
ATOM    807  CA  GLU    51      -9.052  -2.261  -7.970  1.00  0.00
ATOM    808  HA  GLU    51      -8.453  -2.412  -8.868  1.00  0.00
ATOM    809  CB  GLU    51     -10.478  -2.753  -8.243  1.00  0.00
ATOM    810  HB2 GLU    51     -10.471  -3.837  -8.361  1.00  0.00
ATOM    811  HB3 GLU    51     -11.120  -2.484  -7.404  1.00  0.00
ATOM    812  CG  GLU    51     -11.012  -2.102  -9.526  1.00  0.00
ATOM    813  HG2 GLU    51     -12.059  -2.376  -9.656  1.00  0.00
ATOM    814  HG3 GLU    51     -10.929  -1.020  -9.430  1.00  0.00
ATOM    815  CD  GLU    51     -10.207  -2.570 -10.742  1.00  0.00
ATOM    816  OE1 GLU    51      -9.716  -3.688 -10.720  1.00  0.00
ATOM    817  OE2 GLU    51     -10.097  -1.801 -11.682  1.00  0.00
ATOM    818  C   GLU    51      -9.069  -0.757  -7.697  1.00  0.00
ATOM    819  O   GLU    51      -8.851   0.044  -8.604  1.00  0.00
ATOM    820  N   GLN    52      -9.340  -0.380  -6.450  1.00  0.00
ATOM    821  H   GLN    52      -9.545  -1.068  -5.739  1.00  0.00
ATOM    822  CA  GLN    52      -9.395   1.034  -6.082  1.00  0.00
ATOM    823  HA  GLN    52     -10.152   1.549  -6.672  1.00  0.00
ATOM    824  CB  GLN    52      -9.758   1.161  -4.605  1.00  0.00
ATOM    825  HB2 GLN    52      -9.000   0.666  -3.997  1.00  0.00
ATOM    826  HB3 GLN    52      -9.813   2.214  -4.330  1.00  0.00
ATOM    827  CG  GLN    52     -11.118   0.499  -4.363  1.00  0.00
ATOM    828  HG2 GLN    52     -11.249  -0.341  -5.045  1.00  0.00
```

ATOM	829	HG3	GLN	52	-11.179	0.144	-3.334	1.00	0.00
ATOM	830	CD	GLN	52	-12.244	1.501	-4.601	1.00	0.00
ATOM	831	OE1	GLN	52	-12.347	2.077	-5.683	1.00	0.00
ATOM	832	NE2	GLN	52	-13.101	1.743	-3.646	1.00	0.00
ATOM	833	HE21	GLN	52	-13.848	2.405	-3.798	1.00	0.00
ATOM	834	HE22	GLN	52	-13.012	1.266	-2.759	1.00	0.00
ATOM	835	C	GLN	52	-8.066	1.737	-6.360	1.00	0.00
ATOM	836	O	GLN	52	-8.032	2.750	-7.058	1.00	0.00
ATOM	837	N	LEU	53	-6.970	1.187	-5.829	1.00	0.00
ATOM	838	H	LEU	53	-7.027	0.331	-5.295	1.00	0.00
ATOM	839	CA	LEU	53	-5.647	1.777	-6.058	1.00	0.00
ATOM	840	HA	LEU	53	-5.618	2.782	-5.637	1.00	0.00
ATOM	841	CB	LEU	53	-4.547	0.937	-5.395	1.00	0.00
ATOM	842	HB2	LEU	53	-4.381	0.058	-6.018	1.00	0.00
ATOM	843	HB3	LEU	53	-3.632	1.528	-5.352	1.00	0.00
ATOM	844	CG	LEU	53	-4.936	0.493	-3.978	1.00	0.00
ATOM	845	HG	LEU	53	-4.039	0.498	-3.357	1.00	0.00
ATOM	846	CD1	LEU	53	-5.991	1.425	-3.361	1.00	0.00
ATOM	847	HD11	LEU	53	-6.888	1.420	-3.980	1.00	0.00
ATOM	848	HD12	LEU	53	-6.240	1.078	-2.358	1.00	0.00
ATOM	849	HD13	LEU	53	-5.593	2.438	-3.306	1.00	0.00
ATOM	850	CD2	LEU	53	-5.491	-0.928	-4.065	1.00	0.00

```
ATOM    851 HD21 LEU    53      -4.728  -1.593  -4.471  1.00  0.00
ATOM    852 HD22 LEU    53      -5.776  -1.269  -3.069  1.00  0.00
ATOM    853 HD23 LEU    53      -6.365  -0.938  -4.716  1.00  0.00
ATOM    854  C   LEU    53      -5.377   1.769  -7.544  1.00  0.00
ATOM    855  O   LEU    53      -5.002   2.772  -8.151  1.00  0.00
ATOM    856  N   GLU    54      -5.596   0.594  -8.098  1.00  0.00
ATOM    857  H   GLU    54      -5.861  -0.177  -7.501  1.00  0.00
ATOM    858  CA  GLU    54      -5.422   0.339  -9.506  1.00  0.00
ATOM    859  HA  GLU    54      -4.379   0.443  -9.806  1.00  0.00
ATOM    860  CB  GLU    54      -5.858  -1.102  -9.751  1.00  0.00
ATOM    861  HB2 GLU    54      -5.150  -1.772  -9.262  1.00  0.00
ATOM    862  HB3 GLU    54      -6.850  -1.248  -9.323  1.00  0.00
ATOM    863  CG  GLU    54      -5.899  -1.409 -11.254  1.00  0.00
ATOM    864  HG2 GLU    54      -6.532  -0.672 -11.748  1.00  0.00
ATOM    865  HG3 GLU    54      -4.888  -1.347 -11.657  1.00  0.00
ATOM    866  CD  GLU    54      -6.461  -2.812 -11.501  1.00  0.00
ATOM    867  OE1 GLU    54      -6.778  -3.492 -10.537  1.00  0.00
ATOM    868  OE2 GLU    54      -6.565  -3.187 -12.658  1.00  0.00
ATOM    869  C   GLU    54      -6.221   1.338 -10.344  1.00  0.00
ATOM    870  O   GLU    54      -5.801   1.720 -11.437  1.00  0.00
ATOM    871  N   GLY    55      -7.360   1.773  -9.810  1.00  0.00
ATOM    872  H   GLY    55      -7.662   1.431  -8.909  1.00  0.00
```

ATOM	873	CA	GLY	55	-8.203	2.748	-10.500	1.00	0.00
ATOM	874	HA2	GLY	55	-8.110	2.610	-11.577	1.00	0.00
ATOM	875	HA3	GLY	55	-9.242	2.604	-10.203	1.00	0.00
ATOM	876	C	GLY	55	-7.776	4.171	-10.142	1.00	0.00
ATOM	877	O	GLY	55	-8.173	5.135	-10.797	1.00	0.00
ATOM	878	N	MET	56	-6.960	4.285	-9.096	1.00	0.00
ATOM	879	H	MET	56	-6.679	3.458	-8.588	1.00	0.00
ATOM	880	CA	MET	56	-6.465	5.582	-8.638	1.00	0.00
ATOM	881	HA	MET	56	-7.219	6.352	-8.798	1.00	0.00
ATOM	882	CB	MET	56	-6.148	5.510	-7.137	1.00	0.00
ATOM	883	HB2	MET	56	-7.064	5.292	-6.587	1.00	0.00
ATOM	884	HB3	MET	56	-5.422	4.716	-6.962	1.00	0.00
ATOM	885	CG	MET	56	-5.570	6.844	-6.657	1.00	0.00
ATOM	886	HG2	MET	56	-5.189	6.735	-5.641	1.00	0.00
ATOM	887	HG3	MET	56	-4.759	7.152	-7.317	1.00	0.00
ATOM	888	SD	MET	56	-6.864	8.107	-6.674	1.00	0.00
ATOM	889	CE	MET	56	-6.852	8.470	-4.903	1.00	0.00
ATOM	890	HE1	MET	56	-5.863	8.823	-4.610	1.00	0.00
ATOM	891	HE2	MET	56	-7.591	9.240	-4.684	1.00	0.00
ATOM	892	HE3	MET	56	-7.094	7.565	-4.344	1.00	0.00
ATOM	893	C	MET	56	-5.210	5.977	-9.409	1.00	0.00
ATOM	894	O	MET	56	-4.367	5.129	-9.711	1.00	0.00

ATOM	895	N	ASP	57	-5.086	7.269	-9.722	1.00	0.00
ATOM	896	H	ASP	57	-5.809	7.930	-9.475	1.00	0.00
ATOM	897	CA	ASP	57	-3.919	7.759	-10.452	1.00	0.00
ATOM	898	HA	ASP	57	-3.912	7.348	-11.462	1.00	0.00
ATOM	899	CB	ASP	57	-3.959	9.289	-10.545	1.00	0.00
ATOM	900	HB2	ASP	57	-4.031	9.701	-9.538	1.00	0.00
ATOM	901	HB3	ASP	57	-3.040	9.639	-11.015	1.00	0.00
ATOM	902	CG	ASP	57	-5.162	9.748	-11.372	1.00	0.00
ATOM	903	OD1	ASP	57	-5.727	8.931	-12.083	1.00	0.00
ATOM	904	OD2	ASP	57	-5.501	10.917	-11.282	1.00	0.00
ATOM	905	C	ASP	57	-2.636	7.317	-9.752	1.00	0.00
ATOM	906	O	ASP	57	-2.589	7.233	-8.525	1.00	0.00
ATOM	907	N	GLU	58	-1.604	7.023	-10.538	1.00	0.00
ATOM	908	H	GLU	58	-1.689	7.087	-11.542	1.00	0.00
ATOM	909	CA	GLU	58	-0.333	6.572	-9.979	1.00	0.00
ATOM	910	HA	GLU	58	-0.458	5.605	-9.492	1.00	0.00
ATOM	911	CB	GLU	58	0.700	6.426	-11.098	1.00	0.00
ATOM	912	HB2	GLU	58	0.426	5.592	-11.744	1.00	0.00
ATOM	913	HB3	GLU	58	0.737	7.343	-11.685	1.00	0.00
ATOM	914	CG	GLU	58	2.072	6.161	-10.481	1.00	0.00
ATOM	915	HG2	GLU	58	2.338	6.996	-9.833	1.00	0.00
ATOM	916	HG3	GLU	58	2.031	5.244	-9.892	1.00	0.00

```
ATOM    917  CD  GLU    58       3.123   6.014 -11.578  1.00  0.00
ATOM    918  OE1 GLU    58       3.128   4.984 -12.234  1.00  0.00
ATOM    919  OE2 GLU    58       3.910   6.932 -11.745  1.00  0.00
ATOM    920  C   GLU    58       0.202   7.539  -8.926  1.00  0.00
ATOM    921  O   GLU    58       0.444   7.149  -7.784  1.00  0.00
ATOM    922  N   SER    59       0.389   8.796  -9.314  1.00  0.00
ATOM    923  H   SER    59       0.196   9.074 -10.265  1.00  0.00
ATOM    924  CA  SER    59       0.898   9.803  -8.388  1.00  0.00
ATOM    925  HA  SER    59       1.903   9.536  -8.058  1.00  0.00
ATOM    926  CB  SER    59       0.957  11.164  -9.084  1.00  0.00
ATOM    927  HB2 SER    59      -0.049  11.476  -9.363  1.00  0.00
ATOM    928  HB3 SER    59       1.392  11.901  -8.409  1.00  0.00
ATOM    929  OG  SER    59       1.761  11.059 -10.252  1.00  0.00
ATOM    930  HG  SER    59       1.800  11.911 -10.691  1.00  0.00
ATOM    931  C   SER    59       0.001   9.892  -7.160  1.00  0.00
ATOM    932  O   SER    59       0.467  10.129  -6.045  1.00  0.00
ATOM    933  N   CYX    60      -1.290   9.695  -7.388  1.00  0.00
ATOM    934  H   CYX    60      -1.612   9.527  -8.330  1.00  0.00
ATOM    935  CA  CYX    60      -2.281   9.747  -6.315  1.00  0.00
ATOM    936  HA  CYX    60      -2.085  10.600  -5.666  1.00  0.00
ATOM    937  CB  CYX    60      -3.684   9.894  -6.907  1.00  0.00
ATOM    938  HB2 CYX    60      -3.907   9.029  -7.531  1.00  0.00
```

ATOM	939	HB3	CYX	60	-4.414	9.957	-6.100	1.00	0.00
ATOM	940	SG	CYX	60	-3.763	11.406	-7.898	1.00	0.00
ATOM	941	C	CYX	60	-2.218	8.494	-5.449	1.00	0.00
ATOM	942	O	CYX	60	-2.624	8.509	-4.286	1.00	0.00
ATOM	943	N	ARG	61	-1.731	7.405	-6.036	1.00	0.00
ATOM	944	H	ARG	61	-1.425	7.442	-6.998	1.00	0.00
ATOM	945	CA	ARG	61	-1.642	6.132	-5.331	1.00	0.00
ATOM	946	HA	ARG	61	-2.627	5.819	-4.985	1.00	0.00
ATOM	947	CB	ARG	61	-1.096	5.064	-6.283	1.00	0.00
ATOM	948	HB2	ARG	61	-1.648	5.106	-7.221	1.00	0.00
ATOM	949	HB3	ARG	61	-0.041	5.261	-6.473	1.00	0.00
ATOM	950	CG	ARG	61	-1.251	3.672	-5.662	1.00	0.00
ATOM	951	HG2	ARG	61	-0.780	3.654	-4.679	1.00	0.00
ATOM	952	HG3	ARG	61	-2.309	3.431	-5.562	1.00	0.00
ATOM	953	CD	ARG	61	-0.577	2.640	-6.568	1.00	0.00
ATOM	954	HD2	ARG	61	0.503	2.791	-6.567	1.00	0.00
ATOM	955	HD3	ARG	61	-0.802	1.632	-6.218	1.00	0.00
ATOM	956	NE	ARG	61	-1.068	2.781	-7.937	1.00	0.00
ATOM	957	HE	ARG	61	-0.671	3.489	-8.538	1.00	0.00
ATOM	958	CZ	ARG	61	-2.035	2.005	-8.422	1.00	0.00
ATOM	959	NH1	ARG	61	-2.550	1.052	-7.691	1.00	0.00
ATOM	960	HH11	ARG	61	-2.212	0.902	-6.751	1.00	0.00

```
ATOM    961 HH12 ARG    61      -3.283   0.469  -8.069  1.00  0.00
ATOM    962  NH2 ARG    61      -2.465   2.195  -9.638  1.00  0.00
ATOM    963 HH21 ARG    61      -2.062   2.928 -10.204  1.00  0.00
ATOM    964 HH22 ARG    61      -3.198   1.608 -10.008  1.00  0.00
ATOM    965  C   ARG    61      -0.731   6.242  -4.111  1.00  0.00
ATOM    966  O   ARG    61      -1.024   5.670  -3.060  1.00  0.00
ATOM    967  N   CYX    62       0.369   6.980  -4.251  1.00  0.00
ATOM    968  H   CYX    62       0.585   7.426  -5.131  1.00  0.00
ATOM    969  CA  CYX    62       1.303   7.150  -3.142  1.00  0.00
ATOM    970  HA  CYX    62       1.724   6.185  -2.857  1.00  0.00
ATOM    971  CB  CYX    62       2.449   8.081  -3.558  1.00  0.00
ATOM    972  HB2 CYX    62       2.123   9.118  -3.477  1.00  0.00
ATOM    973  HB3 CYX    62       3.306   7.918  -2.904  1.00  0.00
ATOM    974  SG  CYX    62       2.912   7.755  -5.278  1.00  0.00
ATOM    975  C   CYX    62       0.578   7.740  -1.934  1.00  0.00
ATOM    976  O   CYX    62       0.536   7.137  -0.861  1.00  0.00
ATOM    977  N   GLU    63      -0.002   8.922  -2.132  1.00  0.00
ATOM    978  H   GLU    63       0.073   9.373  -3.032  1.00  0.00
ATOM    979  CA  GLU    63      -0.743   9.604  -1.076  1.00  0.00
ATOM    980  HA  GLU    63      -0.088   9.813  -0.229  1.00  0.00
ATOM    981  CB  GLU    63      -1.291  10.930  -1.611  1.00  0.00
ATOM    982  HB2 GLU    63      -0.483  11.487  -2.086  1.00  0.00
```

ATOM 983 HB3 GLU 63 -2.072 10.727 -2.343 1.00 0.00
ATOM 984 CG GLU 63 -1.875 11.759 -0.457 1.00 0.00
ATOM 985 HG2 GLU 63 -2.596 11.149 0.088 1.00 0.00
ATOM 986 HG3 GLU 63 -1.065 12.049 0.213 1.00 0.00
ATOM 987 CD GLU 63 -2.569 13.014 -0.991 1.00 0.00
ATOM 988 OE1 GLU 63 -2.665 13.157 -2.201 1.00 0.00
ATOM 989 OE2 GLU 63 -2.998 13.817 -0.178 1.00 0.00
ATOM 990 C GLU 63 -1.905 8.745 -0.582 1.00 0.00
ATOM 991 O GLU 63 -2.235 8.748 0.604 1.00 0.00
ATOM 992 N GLY 64 -2.538 8.034 -1.516 1.00 0.00
ATOM 993 H GLY 64 -2.224 8.068 -2.475 1.00 0.00
ATOM 994 CA GLY 64 -3.689 7.194 -1.193 1.00 0.00
ATOM 995 HA2 GLY 64 -4.521 7.839 -0.913 1.00 0.00
ATOM 996 HA3 GLY 64 -3.957 6.618 -2.078 1.00 0.00
ATOM 997 C GLY 64 -3.399 6.233 -0.046 1.00 0.00
ATOM 998 O GLY 64 -4.086 6.257 0.975 1.00 0.00
ATOM 999 N LEU 65 -2.392 5.383 -0.220 1.00 0.00
ATOM 1000 H LEU 65 -1.870 5.371 -1.084 1.00 0.00
ATOM 1001 CA LEU 65 -2.042 4.413 0.813 1.00 0.00
ATOM 1002 HA LEU 65 -2.878 3.735 0.986 1.00 0.00
ATOM 1003 CB LEU 65 -0.827 3.589 0.374 1.00 0.00
ATOM 1004 HB2 LEU 65 0.003 4.259 0.151 1.00 0.00

ATOM 1005 HB3 LEU 65 -0.539 2.910 1.177 1.00 0.00

ATOM 1006 CG LEU 65 -1.180 2.778 -0.878 1.00 0.00

ATOM 1007 HG LEU 65 -1.409 3.458 -1.698 1.00 0.00

ATOM 1008 CD1 LEU 65 0.007 1.893 -1.266 1.00 0.00

ATOM 1009 HD11 LEU 65 0.237 1.212 -0.446 1.00 0.00

ATOM 1010 HD12 LEU 65 -0.244 1.316 -2.156 1.00 0.00

ATOM 1011 HD13 LEU 65 0.876 2.518 -1.472 1.00 0.00

ATOM 1012 CD2 LEU 65 -2.403 1.896 -0.590 1.00 0.00

ATOM 1013 HD21 LEU 65 -3.249 2.525 -0.315 1.00 0.00

ATOM 1014 HD22 LEU 65 -2.653 1.319 -1.480 1.00 0.00

ATOM 1015 HD23 LEU 65 -2.175 1.215 0.231 1.00 0.00

ATOM 1016 C LEU 65 -1.747 5.096 2.144 1.00 0.00

ATOM 1017 O LEU 65 -2.222 4.656 3.189 1.00 0.00

ATOM 1018 N ARG 66 -0.965 6.173 2.103 1.00 0.00

ATOM 1019 H ARG 66 -0.577 6.500 1.230 1.00 0.00

ATOM 1020 CA ARG 66 -0.627 6.896 3.325 1.00 0.00

ATOM 1021 HA ARG 66 -0.082 6.243 4.008 1.00 0.00

ATOM 1022 CB ARG 66 0.257 8.102 2.992 1.00 0.00

ATOM 1023 HB2 ARG 66 -0.244 8.725 2.251 1.00 0.00

ATOM 1024 HB3 ARG 66 0.432 8.684 3.897 1.00 0.00

ATOM 1025 CG ARG 66 1.597 7.618 2.430 1.00 0.00

ATOM 1026 HG2 ARG 66 2.078 6.959 3.154 1.00 0.00

ATOM	1027	HG3	ARG	66	1.426	7.073	1.502	1.00	0.00
ATOM	1028	CD	ARG	66	2.503	8.822	2.156	1.00	0.00
ATOM	1029	HD2	ARG	66	3.400	8.497	1.627	1.00	0.00
ATOM	1030	HD3	ARG	66	1.971	9.555	1.551	1.00	0.00
ATOM	1031	NE	ARG	66	2.903	9.453	3.412	1.00	0.00
ATOM	1032	HE	ARG	66	2.442	10.296	3.722	1.00	0.00
ATOM	1033	CZ	ARG	66	3.871	8.942	4.172	1.00	0.00
ATOM	1034	NH1	ARG	66	4.491	7.853	3.803	1.00	0.00
ATOM	1035	HH11	ARG	66	4.235	7.398	2.939	1.00	0.00
ATOM	1036	HH12	ARG	66	5.224	7.471	4.384	1.00	0.00
ATOM	1037	NH2	ARG	66	4.201	9.531	5.287	1.00	0.00
ATOM	1038	HH21	ARG	66	3.722	10.373	5.571	1.00	0.00
ATOM	1039	HH22	ARG	66	4.935	9.142	5.862	1.00	0.00
ATOM	1040	C	ARG	66	-1.891	7.372	4.037	1.00	0.00
ATOM	1041	O	ARG	66	-2.091	7.096	5.221	1.00	0.00
ATOM	1042	N	MET	67	-2.740	8.085	3.301	1.00	0.00
ATOM	1043	H	MET	67	-2.517	8.305	2.341	1.00	0.00
ATOM	1044	CA	MET	67	-3.989	8.598	3.857	1.00	0.00
ATOM	1045	HA	MET	67	-3.785	9.223	4.727	1.00	0.00
ATOM	1046	CB	MET	67	-4.715	9.444	2.805	1.00	0.00
ATOM	1047	HB2	MET	67	-4.205	10.401	2.695	1.00	0.00
ATOM	1048	HB3	MET	67	-4.709	8.918	1.850	1.00	0.00

ATOM	1049	CG	MET	67	-6.163	9.684	3.247	1.00	0.00
ATOM	1050	HG2	MET	67	-6.719	8.748	3.203	1.00	0.00
ATOM	1051	HG3	MET	67	-6.176	10.065	4.268	1.00	0.00
ATOM	1052	SD	MET	67	-6.945	10.895	2.149	1.00	0.00
ATOM	1053	CE	MET	67	-5.962	12.333	2.645	1.00	0.00
ATOM	1054	HE1	MET	67	-4.908	12.140	2.444	1.00	0.00
ATOM	1055	HE2	MET	67	-6.285	13.207	2.080	1.00	0.00
ATOM	1056	HE3	MET	67	-6.100	12.518	3.710	1.00	0.00
ATOM	1057	C	MET	67	-4.902	7.467	4.323	1.00	0.00
ATOM	1058	O	MET	67	-5.440	7.505	5.430	1.00	0.00
ATOM	1059	N	MET	68	-5.089	6.477	3.457	1.00	0.00
ATOM	1060	H	MET	68	-4.654	6.508	2.546	1.00	0.00
ATOM	1061	CA	MET	68	-5.961	5.350	3.768	1.00	0.00
ATOM	1062	HA	MET	68	-6.959	5.702	4.026	1.00	0.00
ATOM	1063	CB	MET	68	-6.065	4.438	2.542	1.00	0.00
ATOM	1064	HB2	MET	68	-6.770	4.871	1.833	1.00	0.00
ATOM	1065	HB3	MET	68	-5.084	4.355	2.074	1.00	0.00
ATOM	1066	CG	MET	68	-6.551	3.045	2.959	1.00	0.00
ATOM	1067	HG2	MET	68	-5.760	2.526	3.502	1.00	0.00
ATOM	1068	HG3	MET	68	-7.429	3.137	3.598	1.00	0.00
ATOM	1069	SD	MET	68	-6.986	2.091	1.486	1.00	0.00
ATOM	1070	CE	MET	68	-5.309	1.895	0.838	1.00	0.00

ATOM	1071	HE1	MET	68	-4.696	1.367	1.569	1.00	0.00
ATOM	1072	HE2	MET	68	-5.341	1.323	-0.089	1.00	0.00
ATOM	1073	HE3	MET	68	-4.877	2.876	0.644	1.00	0.00
ATOM	1074	C	MET	68	-5.458	4.562	4.974	1.00	0.00
ATOM	1075	O	MET	68	-6.234	4.241	5.872	1.00	0.00
ATOM	1076	N	MET	69	-4.165	4.249	4.995	1.00	0.00
ATOM	1077	H	MET	69	-3.553	4.510	4.235	1.00	0.00
ATOM	1078	CA	MET	69	-3.600	3.493	6.108	1.00	0.00
ATOM	1079	HA	MET	69	-3.997	2.478	6.111	1.00	0.00
ATOM	1080	CB	MET	69	-2.075	3.422	5.972	1.00	0.00
ATOM	1081	HB2	MET	69	-1.818	3.027	4.989	1.00	0.00
ATOM	1082	HB3	MET	69	-1.655	4.422	6.082	1.00	0.00
ATOM	1083	CG	MET	69	-1.501	2.506	7.056	1.00	0.00
ATOM	1084	HG2	MET	69	-1.805	2.863	8.040	1.00	0.00
ATOM	1085	HG3	MET	69	-1.866	1.489	6.911	1.00	0.00
ATOM	1086	SD	MET	69	0.306	2.509	6.954	1.00	0.00
ATOM	1087	CE	MET	69	0.611	1.278	8.245	1.00	0.00
ATOM	1088	HE1	MET	69	0.133	0.337	7.971	1.00	0.00
ATOM	1089	HE2	MET	69	1.685	1.121	8.353	1.00	0.00
ATOM	1090	HE3	MET	69	0.200	1.632	9.190	1.00	0.00
ATOM	1091	C	MET	69	-3.970	4.148	7.436	1.00	0.00
ATOM	1092	O	MET	69	-4.528	3.504	8.325	1.00	0.00

ATOM	1093	N	ARG	70	-3.660	5.435	7.554	1.00	0.00
ATOM	1094	H	ARG	70	-3.187	5.913	6.801	1.00	0.00
ATOM	1095	CA	ARG	70	-3.965	6.186	8.767	1.00	0.00
ATOM	1096	HA	ARG	70	-3.488	5.720	9.630	1.00	0.00
ATOM	1097	CB	ARG	70	-3.436	7.617	8.629	1.00	0.00
ATOM	1098	HB2	ARG	70	-3.875	8.084	7.748	1.00	0.00
ATOM	1099	HB3	ARG	70	-3.704	8.191	9.516	1.00	0.00
ATOM	1100	CG	ARG	70	-1.911	7.585	8.484	1.00	0.00
ATOM	1101	HG2	ARG	70	-1.473	7.112	9.363	1.00	0.00
ATOM	1102	HG3	ARG	70	-1.643	7.014	7.595	1.00	0.00
ATOM	1103	CD	ARG	70	-1.376	9.013	8.354	1.00	0.00
ATOM	1104	HD2	ARG	70	-1.944	9.556	7.599	1.00	0.00
ATOM	1105	HD3	ARG	70	-1.457	9.529	9.311	1.00	0.00
ATOM	1106	NE	ARG	70	0.028	8.988	7.954	1.00	0.00
ATOM	1107	HE	ARG	70	0.604	8.190	8.183	1.00	0.00
ATOM	1108	CZ	ARG	70	0.580	10.001	7.286	1.00	0.00
ATOM	1109	NH1	ARG	70	-0.129	11.056	6.982	1.00	0.00
ATOM	1110	HH11	ARG	70	-1.100	11.103	7.255	1.00	0.00
ATOM	1111	HH12	ARG	70	0.298	11.818	6.476	1.00	0.00
ATOM	1112	NH2	ARG	70	1.835	9.940	6.934	1.00	0.00
ATOM	1113	HH21	ARG	70	2.384	9.125	7.170	1.00	0.00
ATOM	1114	HH22	ARG	70	2.253	10.708	6.428	1.00	0.00

```
ATOM   1115  C   ARG    70      -5.471   6.221   9.027  1.00  0.00
ATOM   1116  O   ARG    70      -5.917   6.069  10.164  1.00  0.00
ATOM   1117  N   MET    71      -6.245   6.430   7.961  1.00  0.00
ATOM   1118  H   MET    71      -5.825   6.572   7.054  1.00  0.00
ATOM   1119  CA  MET    71      -7.697   6.495   8.069  1.00  0.00
ATOM   1120  HA  MET    71      -7.984   7.242   8.809  1.00  0.00
ATOM   1121  CB  MET    71      -8.303   6.886   6.718  1.00  0.00
ATOM   1122  HB2 MET    71      -7.787   7.765   6.331  1.00  0.00
ATOM   1123  HB3 MET    71      -8.187   6.059   6.017  1.00  0.00
ATOM   1124  CG  MET    71      -9.792   7.203   6.893  1.00  0.00
ATOM   1125  HG2 MET    71     -10.295   6.357   7.362  1.00  0.00
ATOM   1126  HG3 MET    71      -9.908   8.087   7.520  1.00  0.00
ATOM   1127  SD  MET    71     -10.540   7.519   5.274  1.00  0.00
ATOM   1128  CE  MET    71      -9.603   9.014   4.867  1.00  0.00
ATOM   1129  HE1 MET    71      -8.538   8.781   4.846  1.00  0.00
ATOM   1130  HE2 MET    71      -9.913   9.383   3.890  1.00  0.00
ATOM   1131  HE3 MET    71      -9.792   9.778   5.621  1.00  0.00
ATOM   1132  C   MET    71      -8.268   5.159   8.524  1.00  0.00
ATOM   1133  O   MET    71      -9.216   5.115   9.308  1.00  0.00
ATOM   1134  N   MET    72      -7.695   4.073   8.017  1.00  0.00
ATOM   1135  H   MET    72      -6.933   4.149   7.358  1.00  0.00
ATOM   1136  CA  MET    72      -8.169   2.744   8.373  1.00  0.00
```

ATOM	1137	HA	MET	72	-9.204	2.619	8.056	1.00	0.00
ATOM	1138	CB	MET	72	-7.313	1.678	7.681	1.00	0.00
ATOM	1139	HB2	MET	72	-6.262	1.959	7.752	1.00	0.00
ATOM	1140	HB3	MET	72	-7.466	0.718	8.175	1.00	0.00
ATOM	1141	CG	MET	72	-7.712	1.563	6.207	1.00	0.00
ATOM	1142	HG2	MET	72	-7.683	2.547	5.740	1.00	0.00
ATOM	1143	HG3	MET	72	-7.022	0.895	5.690	1.00	0.00
ATOM	1144	SD	MET	72	-9.391	0.895	6.085	1.00	0.00
ATOM	1145	CE	MET	72	-9.047	-0.729	6.810	1.00	0.00
ATOM	1146	HE1	MET	72	-8.671	-0.602	7.826	1.00	0.00
ATOM	1147	HE2	MET	72	-9.963	-1.319	6.833	1.00	0.00
ATOM	1148	HE3	MET	72	-8.298	-1.245	6.209	1.00	0.00
ATOM	1149	C	MET	72	-8.122	2.536	9.881	1.00	0.00
ATOM	1150	O	MET	72	-9.137	2.220	10.505	1.00	0.00
ATOM	1151	N	GLN	73	-6.940	2.722	10.459	1.00	0.00
ATOM	1152	H	GLN	73	-6.130	2.958	9.903	1.00	0.00
ATOM	1153	CA	GLN	73	-6.767	2.561	11.897	1.00	0.00
ATOM	1154	HA	GLN	73	-7.056	1.553	12.197	1.00	0.00
ATOM	1155	CB	GLN	73	-5.301	2.783	12.278	1.00	0.00
ATOM	1156	HB2	GLN	73	-5.007	3.797	12.009	1.00	0.00
ATOM	1157	HB3	GLN	73	-5.182	2.644	13.353	1.00	0.00
ATOM	1158	CG	GLN	73	-4.415	1.781	11.533	1.00	0.00

ATOM	1159	HG2	GLN	73	-4.400	1.998	10.465	1.00	0.00
ATOM	1160	HG3	GLN	73	-3.400	1.833	11.928	1.00	0.00
ATOM	1161	CD	GLN	73	-4.943	0.367	11.735	1.00	0.00
ATOM	1162	OE1	GLN	73	-4.907	-0.160	12.847	1.00	0.00
ATOM	1163	NE2	GLN	73	-5.450	-0.274	10.721	1.00	0.00
ATOM	1164	HE21	GLN	73	-5.802	-1.213	10.846	1.00	0.00
ATOM	1165	HE22	GLN	73	-5.488	0.170	9.815	1.00	0.00
ATOM	1166	C	GLN	73	-7.652	3.543	12.658	1.00	0.00
ATOM	1167	O	GLN	73	-8.247	3.199	13.675	1.00	0.00
ATOM	1168	N	GLN	74	-7.724	4.770	12.155	1.00	0.00
ATOM	1169	H	GLN	74	-7.189	5.018	11.335	1.00	0.00
ATOM	1170	CA	GLN	74	-8.529	5.804	12.794	1.00	0.00
ATOM	1171	HA	GLN	74	-8.219	5.935	13.831	1.00	0.00
ATOM	1172	CB	GLN	74	-8.342	7.133	12.055	1.00	0.00
ATOM	1173	HB2	GLN	74	-7.405	7.594	12.368	1.00	0.00
ATOM	1174	HB3	GLN	74	-8.315	6.950	10.981	1.00	0.00
ATOM	1175	CG	GLN	74	-9.506	8.070	12.384	1.00	0.00
ATOM	1176	HG2	GLN	74	-10.048	7.707	13.258	1.00	0.00
ATOM	1177	HG3	GLN	74	-9.130	9.074	12.582	1.00	0.00
ATOM	1178	CD	GLN	74	-10.469	8.134	11.205	1.00	0.00
ATOM	1179	OE1	GLN	74	-10.499	7.148	10.354	1.00	0.00
ATOM	1180	NE2	GLN	74	-11.214	9.103	11.059	1.00	0.00

```
ATOM   1181 HE21 GLN    74     -11.848   9.134  10.274  1.00  0.00
ATOM   1182 HE22 GLN    74     -11.187   9.861  11.726  1.00  0.00
ATOM   1183  C   GLN    74     -10.009   5.426  12.830  1.00  0.00
ATOM   1184  O   GLN    74     -10.679   5.617  13.846  1.00  0.00
ATOM   1185  N   LYS    75     -10.514   4.904  11.719  1.00  0.00
ATOM   1186  H   LYS    75      -9.935   4.775  10.901  1.00  0.00
ATOM   1187  CA  LYS    75     -11.921   4.526  11.638  1.00  0.00
ATOM   1188  HA  LYS    75     -12.555   5.397  11.803  1.00  0.00
ATOM   1189  CB  LYS    75     -12.221   3.957  10.248  1.00  0.00
ATOM   1190  HB2 LYS    75     -11.889   4.666   9.490  1.00  0.00
ATOM   1191  HB3 LYS    75     -11.687   3.015  10.122  1.00  0.00
ATOM   1192  CG  LYS    75     -13.728   3.715  10.097  1.00  0.00
ATOM   1193  HG2 LYS    75     -13.904   3.058   9.245  1.00  0.00
ATOM   1194  HG3 LYS    75     -14.110   3.244  11.003  1.00  0.00
ATOM   1195  CD  LYS    75     -14.449   5.048   9.871  1.00  0.00
ATOM   1196  HD2 LYS    75     -14.331   5.678  10.753  1.00  0.00
ATOM   1197  HD3 LYS    75     -14.019   5.551   9.005  1.00  0.00
ATOM   1198  CE  LYS    75     -15.937   4.790   9.624  1.00  0.00
ATOM   1199  HE2 LYS    75     -16.326   4.136  10.404  1.00  0.00
ATOM   1200  HE3 LYS    75     -16.477   5.736   9.641  1.00  0.00
ATOM   1201  NZ  LYS    75     -16.116   4.139   8.295  1.00  0.00
ATOM   1202  HZ1 LYS    75     -15.615   3.261   8.280  1.00  0.00
```

ATOM 1203 HZ2 LYS 75 -17.098 3.969 8.133 1.00 0.00

ATOM 1204 HZ3 LYS 75 -15.755 4.745 7.572 1.00 0.00

ATOM 1205 C LYS 75 -12.269 3.489 12.702 1.00 0.00

ATOM 1206 O LYS 75 -13.324 3.563 13.334 1.00 0.00

ATOM 1207 N GLU 76 -11.375 2.528 12.889 1.00 0.00

ATOM 1208 H GLU 76 -10.533 2.495 12.331 1.00 0.00

ATOM 1209 CA GLU 76 -11.592 1.473 13.878 1.00 0.00

ATOM 1210 HA GLU 76 -11.906 1.907 14.827 1.00 0.00

ATOM 1211 CB GLU 76 -12.689 0.517 13.389 1.00 0.00

ATOM 1212 HB2 GLU 76 -13.560 1.095 13.082 1.00 0.00

ATOM 1213 HB3 GLU 76 -12.316 -0.056 12.540 1.00 0.00

ATOM 1214 CG GLU 76 -13.085 -0.444 14.520 1.00 0.00

ATOM 1215 HG2 GLU 76 -13.749 -1.209 14.117 1.00 0.00

ATOM 1216 HG3 GLU 76 -12.185 -0.916 14.915 1.00 0.00

ATOM 1217 CD GLU 76 -13.799 0.310 15.643 1.00 0.00

ATOM 1218 OE1 GLU 76 -14.417 1.326 15.360 1.00 0.00

ATOM 1219 OE2 GLU 76 -13.717 -0.140 16.774 1.00 0.00

ATOM 1220 C GLU 76 -10.301 0.697 14.130 1.00 0.00

ATOM 1221 O GLU 76 -10.144 -0.430 13.656 1.00 0.00

ATOM 1222 N MET 77 -9.376 1.306 14.865 1.00 0.00

ATOM 1223 H MET 77 -9.531 2.241 15.213 1.00 0.00

ATOM 1224 CA MET 77 -8.100 0.660 15.163 1.00 0.00

ATOM	1225	HA	MET	77	-7.548	0.479	14.241	1.00	0.00
ATOM	1226	CB	MET	77	-7.255	1.562	16.071	1.00	0.00
ATOM	1227	HB2	MET	77	-7.189	2.557	15.632	1.00	0.00
ATOM	1228	HB3	MET	77	-7.724	1.629	17.053	1.00	0.00
ATOM	1229	CG	MET	77	-5.848	0.975	16.218	1.00	0.00
ATOM	1230	HG2	MET	77	-5.911	-0.044	16.600	1.00	0.00
ATOM	1231	HG3	MET	77	-5.348	0.968	15.250	1.00	0.00
ATOM	1232	SD	MET	77	-4.891	1.985	17.374	1.00	0.00
ATOM	1233	CE	MET	77	-3.322	1.099	17.208	1.00	0.00
ATOM	1234	HE1	MET	77	-2.989	1.140	16.171	1.00	0.00
ATOM	1235	HE2	MET	77	-2.572	1.563	17.849	1.00	0.00
ATOM	1236	HE3	MET	77	-3.458	0.059	17.505	1.00	0.00
ATOM	1237	C	MET	77	-8.318	-0.693	15.840	1.00	0.00
ATOM	1238	O	MET	77	-8.431	-0.769	17.065	1.00	0.00
ATOM	1239	N	GLN	78	-8.362	-1.757	15.041	1.00	0.00
ATOM	1240	H	GLN	78	-8.281	-1.652	14.040	1.00	0.00
ATOM	1241	CA	GLN	78	-8.547	-3.100	15.583	1.00	0.00
ATOM	1242	HA	GLN	78	-9.533	-3.186	16.040	1.00	0.00
ATOM	1243	CB	GLN	78	-8.434	-4.141	14.462	1.00	0.00
ATOM	1244	HB2	GLN	78	-8.010	-3.672	13.574	1.00	0.00
ATOM	1245	HB3	GLN	78	-7.785	-4.955	14.788	1.00	0.00
ATOM	1246	CG	GLN	78	-9.823	-4.697	14.132	1.00	0.00

ATOM 1247 HG2 GLN 78 -10.270 -5.137 15.024 1.00 0.00
ATOM 1248 HG3 GLN 78 -10.465 -3.899 13.761 1.00 0.00
ATOM 1249 CD GLN 78 -9.714 -5.776 13.057 1.00 0.00
ATOM 1250 OE1 GLN 78 -8.902 -6.694 13.176 1.00 0.00
ATOM 1251 NE2 GLN 78 -10.489 -5.720 12.008 1.00 0.00
ATOM 1252 HE21 GLN 78 -10.420 -6.432 11.295 1.00 0.00
ATOM 1253 HE22 GLN 78 -11.153 -4.966 11.915 1.00 0.00
ATOM 1254 C GLN 78 -7.488 -3.372 16.652 1.00 0.00
ATOM 1255 O GLN 78 -6.309 -3.082 16.441 1.00 0.00
ATOM 1256 N PRO 79 -7.868 -3.912 17.784 1.00 0.00
ATOM 1257 CD PRO 79 -9.231 -4.307 18.158 1.00 0.00
ATOM 1258 HD2 PRO 79 -9.509 -5.204 17.604 1.00 0.00
ATOM 1259 HD3 PRO 79 -9.919 -3.499 17.911 1.00 0.00
ATOM 1260 CG PRO 79 -9.137 -4.554 19.672 1.00 0.00
ATOM 1261 HG2 PRO 79 -9.250 -5.621 19.865 1.00 0.00
ATOM 1262 HG3 PRO 79 -9.937 -4.008 20.171 1.00 0.00
ATOM 1263 CB PRO 79 -7.799 -4.059 20.113 1.00 0.00
ATOM 1264 HB2 PRO 79 -7.441 -4.719 20.904 1.00 0.00
ATOM 1265 HB3 PRO 79 -7.777 -3.028 20.464 1.00 0.00
ATOM 1266 CA PRO 79 -6.920 -4.206 18.880 1.00 0.00
ATOM 1267 HA PRO 79 -6.099 -3.491 18.829 1.00 0.00
ATOM 1268 C PRO 79 -6.367 -5.619 18.775 1.00 0.00

```
ATOM   1269  O   PRO    79      -7.087  -6.601  18.964  1.00  0.00
ATOM   1270  N   ARG    80      -5.085  -5.701  18.485  1.00  0.00
ATOM   1271  H   ARG    80      -4.550  -4.860  18.321  1.00  0.00
ATOM   1272  CA  ARG    80      -4.409  -6.983  18.363  1.00  0.00
ATOM   1273  HA  ARG    80      -4.532  -7.554  19.284  1.00  0.00
ATOM   1274  CB  ARG    80      -4.996  -7.791  17.204  1.00  0.00
ATOM   1275  HB2 ARG    80      -5.700  -7.173  16.648  1.00  0.00
ATOM   1276  HB3 ARG    80      -4.193  -8.114  16.541  1.00  0.00
ATOM   1277  CG  ARG    80      -5.723  -9.019  17.759  1.00  0.00
ATOM   1278  HG2 ARG    80      -5.009  -9.831  17.896  1.00  0.00
ATOM   1279  HG3 ARG    80      -6.176  -8.769  18.719  1.00  0.00
ATOM   1280  CD  ARG    80      -6.812  -9.458  16.781  1.00  0.00
ATOM   1281  HD2 ARG    80      -6.655  -8.982  15.813  1.00  0.00
ATOM   1282  HD3 ARG    80      -6.787 -10.541  16.661  1.00  0.00
ATOM   1283  NE  ARG    80      -8.129  -9.072  17.285  1.00  0.00
ATOM   1284  HE  ARG    80      -8.597  -9.659  17.962  1.00  0.00
ATOM   1285  CZ  ARG    80      -8.740  -7.960  16.876  1.00  0.00
ATOM   1286  NH1 ARG    80      -8.164  -7.168  16.012  1.00  0.00
ATOM   1287 HH11 ARG    80      -7.249  -7.400  15.651  1.00  0.00
ATOM   1288 HH12 ARG    80      -8.635  -6.328  15.709  1.00  0.00
ATOM   1289  NH2 ARG    80      -9.920  -7.661  17.343  1.00  0.00
ATOM   1290 HH21 ARG    80     -10.365  -8.273  18.012  1.00  0.00
```

ATOM 1291 HH22 ARG 80 -10.383 -6.819 17.034 1.00 0.00
ATOM 1292 C ARG 80 -2.919 -6.764 18.137 1.00 0.00
ATOM 1293 O ARG 80 -2.530 -6.016 17.239 1.00 0.00
ATOM 1294 N GLY 81 -2.087 -7.407 18.949 1.00 0.00
ATOM 1295 H GLY 81 -2.428 -8.017 19.679 1.00 0.00
ATOM 1296 CA GLY 81 -0.648 -7.245 18.803 1.00 0.00
ATOM 1297 HA2 GLY 81 -0.445 -6.249 18.411 1.00 0.00
ATOM 1298 HA3 GLY 81 -0.182 -7.351 19.783 1.00 0.00
ATOM 1299 C GLY 81 -0.052 -8.283 17.855 1.00 0.00
ATOM 1300 O GLY 81 0.326 -7.960 16.732 1.00 0.00
ATOM 1301 N GLU 82 0.042 -9.523 18.325 1.00 0.00
ATOM 1302 H GLU 82 -0.258 -9.730 19.267 1.00 0.00
ATOM 1303 CA GLU 82 0.617 -10.607 17.527 1.00 0.00
ATOM 1304 HA GLU 82 1.624 -10.343 17.203 1.00 0.00
ATOM 1305 CB GLU 82 0.693 -11.884 18.370 1.00 0.00
ATOM 1306 HB2 GLU 82 -0.291 -12.102 18.785 1.00 0.00
ATOM 1307 HB3 GLU 82 1.013 -12.714 17.740 1.00 0.00
ATOM 1308 CG GLU 82 1.700 -11.693 19.514 1.00 0.00
ATOM 1309 HG2 GLU 82 1.992 -12.673 19.892 1.00 0.00
ATOM 1310 HG3 GLU 82 2.579 -11.178 19.126 1.00 0.00
ATOM 1311 CD GLU 82 1.086 -10.870 20.650 1.00 0.00
ATOM 1312 OE1 GLU 82 -0.127 -10.891 20.795 1.00 0.00

```
ATOM   1313  OE2 GLU    82       1.842 -10.228  21.361  1.00  0.00
ATOM   1314  C   GLU    82      -0.174 -10.895  16.249  1.00  0.00
ATOM   1315  O   GLU    82       0.414 -11.097  15.186  1.00  0.00
ATOM   1316  N   GLN    83      -1.500 -10.954  16.359  1.00  0.00
ATOM   1317  H   GLN    83      -1.949 -10.811  17.252  1.00  0.00
ATOM   1318  CA  GLN    83      -2.340 -11.267  15.202  1.00  0.00
ATOM   1319  HA  GLN    83      -2.071 -12.242  14.794  1.00  0.00
ATOM   1320  CB  GLN    83      -3.812 -11.303  15.629  1.00  0.00
ATOM   1321  HB2 GLN    83      -3.922 -11.969  16.485  1.00  0.00
ATOM   1322  HB3 GLN    83      -4.131 -10.299  15.908  1.00  0.00
ATOM   1323  CG  GLN    83      -4.678 -11.811  14.472  1.00  0.00
ATOM   1324  HG2 GLN    83      -5.731 -11.632  14.688  1.00  0.00
ATOM   1325  HG3 GLN    83      -4.404 -11.298  13.550  1.00  0.00
ATOM   1326  CD  GLN    83      -4.468 -13.310  14.277  1.00  0.00
ATOM   1327  OE1 GLN    83      -4.863 -14.108  15.126  1.00  0.00
ATOM   1328  NE2 GLN    83      -3.867 -13.741  13.202  1.00  0.00
ATOM   1329 HE21 GLN    83      -3.726 -14.733  13.069  1.00  0.00
ATOM   1330 HE22 GLN    83      -3.545 -13.082  12.508  1.00  0.00
ATOM   1331  C   GLN    83      -2.162 -10.254  14.068  1.00  0.00
ATOM   1332  O   GLN    83      -1.689 -10.600  12.986  1.00  0.00
ATOM   1333  N   MET    84      -2.554  -9.009  14.323  1.00  0.00
ATOM   1334  H   MET    84      -2.996  -8.787  15.204  1.00  0.00
```

ATOM	1335	CA	MET	84	-2.444	-7.955	13.317	1.00	0.00
ATOM	1336	HA	MET	84	-2.938	-8.267	12.397	1.00	0.00
ATOM	1337	CB	MET	84	-3.109	-6.673	13.818	1.00	0.00
ATOM	1338	HB2	MET	84	-2.848	-6.511	14.864	1.00	0.00
ATOM	1339	HB3	MET	84	-2.766	-5.827	13.223	1.00	0.00
ATOM	1340	CG	MET	84	-4.633	-6.807	13.686	1.00	0.00
ATOM	1341	HG2	MET	84	-4.900	-6.973	12.643	1.00	0.00
ATOM	1342	HG3	MET	84	-4.983	-7.646	14.288	1.00	0.00
ATOM	1343	SD	MET	84	-5.423	-5.286	14.268	1.00	0.00
ATOM	1344	CE	MET	84	-4.943	-4.224	12.883	1.00	0.00
ATOM	1345	HE1	MET	84	-5.348	-4.630	11.956	1.00	0.00
ATOM	1346	HE2	MET	84	-5.335	-3.220	13.042	1.00	0.00
ATOM	1347	HE3	MET	84	-3.856	-4.183	12.816	1.00	0.00
ATOM	1348	C	MET	84	-0.995	-7.692	12.909	1.00	0.00
ATOM	1349	O	MET	84	-0.737	-7.295	11.774	1.00	0.00
ATOM	1350	N	ARG	85	-0.048	-7.907	13.823	1.00	0.00
ATOM	1351	H	ARG	85	-0.276	-8.213	14.758	1.00	0.00
ATOM	1352	CA	ARG	85	1.359	-7.675	13.493	1.00	0.00
ATOM	1353	HA	ARG	85	1.534	-6.610	13.344	1.00	0.00
ATOM	1354	CB	ARG	85	2.262	-8.164	14.631	1.00	0.00
ATOM	1355	HB2	ARG	85	2.031	-7.607	15.539	1.00	0.00
ATOM	1356	HB3	ARG	85	2.083	-9.226	14.802	1.00	0.00

```
ATOM   1357  CG  ARG    85       3.733  -7.948  14.261  1.00  0.00
ATOM   1358  HG2 ARG    85       3.988  -8.566  13.400  1.00  0.00
ATOM   1359  HG3 ARG    85       3.898  -6.899  14.017  1.00  0.00
ATOM   1360  CD  ARG    85       4.616  -8.340  15.448  1.00  0.00
ATOM   1361  HD2 ARG    85       5.655  -8.085  15.239  1.00  0.00
ATOM   1362  HD3 ARG    85       4.287  -7.816  16.346  1.00  0.00
ATOM   1363  NE  ARG    85       4.527  -9.779  15.687  1.00  0.00
ATOM   1364  HE  ARG    85       4.540 -10.419  14.906  1.00  0.00
ATOM   1365  CZ  ARG    85       4.424 -10.281  16.918  1.00  0.00
ATOM   1366  NH1 ARG    85       4.413  -9.491  17.959  1.00  0.00
ATOM   1367 HH11 ARG    85       4.483  -8.492  17.832  1.00  0.00
ATOM   1368 HH12 ARG    85       4.335  -9.884  18.887  1.00  0.00
ATOM   1369  NH2 ARG    85       4.338 -11.572  17.085  1.00  0.00
ATOM   1370 HH21 ARG    85       4.350 -12.184  16.282  1.00  0.00
ATOM   1371 HH22 ARG    85       4.260 -11.954  18.017  1.00  0.00
ATOM   1372  C   ARG    85       1.715  -8.405  12.201  1.00  0.00
ATOM   1373  O   ARG    85       2.339  -7.834  11.307  1.00  0.00
ATOM   1374  N   ARG    86       1.288  -9.661  12.103  1.00  0.00
ATOM   1375  H   ARG    86       0.795 -10.096  12.870  1.00  0.00
ATOM   1376  CA  ARG    86       1.539 -10.453  10.905  1.00  0.00
ATOM   1377  HA  ARG    86       2.611 -10.522  10.719  1.00  0.00
ATOM   1378  CB  ARG    86       0.983 -11.867  11.092  1.00  0.00
```

ATOM	1379	HB2	ARG	86	-0.081	-11.813	11.320	1.00	0.00
ATOM	1380	HB3	ARG	86	1.130	-12.440	10.177	1.00	0.00
ATOM	1381	CG	ARG	86	1.718	-12.554	12.247	1.00	0.00
ATOM	1382	HG2	ARG	86	2.787	-12.572	12.034	1.00	0.00
ATOM	1383	HG3	ARG	86	1.541	-11.999	13.168	1.00	0.00
ATOM	1384	CD	ARG	86	1.207	-13.986	12.410	1.00	0.00
ATOM	1385	HD2	ARG	86	0.119	-13.988	12.464	1.00	0.00
ATOM	1386	HD3	ARG	86	1.530	-14.594	11.565	1.00	0.00
ATOM	1387	NE	ARG	86	1.737	-14.573	13.637	1.00	0.00
ATOM	1388	HE	ARG	86	2.465	-14.097	14.151	1.00	0.00
ATOM	1389	CZ	ARG	86	1.281	-15.732	14.107	1.00	0.00
ATOM	1390	NH1	ARG	86	0.344	-16.379	13.467	1.00	0.00
ATOM	1391	HH11	ARG	86	-0.035	-15.998	12.612	1.00	0.00
ATOM	1392	HH12	ARG	86	0.004	-17.258	13.830	1.00	0.00
ATOM	1393	NH2	ARG	86	1.774	-16.225	15.209	1.00	0.00
ATOM	1394	HH21	ARG	86	2.501	-15.725	15.702	1.00	0.00
ATOM	1395	HH22	ARG	86	1.428	-17.105	15.565	1.00	0.00
ATOM	1396	C	ARG	86	0.883	-9.790	9.695	1.00	0.00
ATOM	1397	O	ARG	86	1.437	-9.782	8.595	1.00	0.00
ATOM	1398	N	MET	87	-0.309	-9.234	9.923	1.00	0.00
ATOM	1399	H	MET	87	-0.717	-9.286	10.846	1.00	0.00
ATOM	1400	CA	MET	87	-1.064	-8.561	8.868	1.00	0.00

ATOM 1401 HA MET 87 -1.340 -9.276 8.093 1.00 0.00

ATOM 1402 CB MET 87 -2.344 -7.949 9.448 1.00 0.00

ATOM 1403 HB2 MET 87 -2.867 -8.701 10.039 1.00 0.00

ATOM 1404 HB3 MET 87 -2.083 -7.104 10.085 1.00 0.00

ATOM 1405 CG MET 87 -3.252 -7.471 8.310 1.00 0.00

ATOM 1406 HG2 MET 87 -2.644 -7.059 7.505 1.00 0.00

ATOM 1407 HG3 MET 87 -3.836 -8.310 7.931 1.00 0.00

ATOM 1408 SD MET 87 -4.377 -6.190 8.923 1.00 0.00

ATOM 1409 CE MET 87 -5.412 -7.245 9.966 1.00 0.00

ATOM 1410 HE1 MET 87 -4.795 -7.718 10.731 1.00 0.00

ATOM 1411 HE2 MET 87 -6.182 -6.640 10.445 1.00 0.00

ATOM 1412 HE3 MET 87 -5.882 -8.014 9.353 1.00 0.00

ATOM 1413 C MET 87 -0.239 -7.456 8.213 1.00 0.00

ATOM 1414 O MET 87 -0.428 -7.153 7.037 1.00 0.00

ATOM 1415 N MET 88 0.659 -6.840 8.982 1.00 0.00

ATOM 1416 H MET 88 0.782 -7.109 9.948 1.00 0.00

ATOM 1417 CA MET 88 1.479 -5.752 8.457 1.00 0.00

ATOM 1418 HA MET 88 0.873 -4.860 8.303 1.00 0.00

ATOM 1419 CB MET 88 2.585 -5.424 9.460 1.00 0.00

ATOM 1420 HB2 MET 88 3.137 -6.333 9.701 1.00 0.00

ATOM 1421 HB3 MET 88 3.265 -4.693 9.023 1.00 0.00

ATOM 1422 CG MET 88 1.968 -4.847 10.740 1.00 0.00

ATOM	1423	HG2	MET	88	1.668	-3.813	10.570	1.00	0.00
ATOM	1424	HG3	MET	88	1.096	-5.436	11.026	1.00	0.00
ATOM	1425	SD	MET	88	3.187	-4.901	12.078	1.00	0.00
ATOM	1426	CE	MET	88	4.616	-4.351	11.116	1.00	0.00
ATOM	1427	HE1	MET	88	4.421	-3.359	10.709	1.00	0.00
ATOM	1428	HE2	MET	88	5.495	-4.312	11.760	1.00	0.00
ATOM	1429	HE3	MET	88	4.796	-5.050	10.299	1.00	0.00
ATOM	1430	C	MET	88	2.105	-6.139	7.122	1.00	0.00
ATOM	1431	O	MET	88	1.966	-5.419	6.133	1.00	0.00
ATOM	1432	N	ARG	89	2.776	-7.286	7.093	1.00	0.00
ATOM	1433	H	ARG	89	2.887	-7.842	7.929	1.00	0.00
ATOM	1434	CA	ARG	89	3.396	-7.759	5.862	1.00	0.00
ATOM	1435	HA	ARG	89	4.120	-7.027	5.503	1.00	0.00
ATOM	1436	CB	ARG	89	4.124	-9.083	6.116	1.00	0.00
ATOM	1437	HB2	ARG	89	3.419	-9.814	6.512	1.00	0.00
ATOM	1438	HB3	ARG	89	4.542	-9.452	5.180	1.00	0.00
ATOM	1439	CG	ARG	89	5.255	-8.864	7.129	1.00	0.00
ATOM	1440	HG2	ARG	89	4.863	-8.342	8.002	1.00	0.00
ATOM	1441	HG3	ARG	89	5.659	-9.829	7.435	1.00	0.00
ATOM	1442	CD	ARG	89	6.366	-8.025	6.489	1.00	0.00
ATOM	1443	HD2	ARG	89	6.717	-8.510	5.578	1.00	0.00
ATOM	1444	HD3	ARG	89	5.987	-7.032	6.249	1.00	0.00

ATOM 1445 NE ARG 89 7.491 -7.886 7.408 1.00 0.00
ATOM 1446 HE ARG 89 8.301 -8.482 7.310 1.00 0.00
ATOM 1447 CZ ARG 89 7.482 -6.982 8.386 1.00 0.00
ATOM 1448 NH1 ARG 89 6.445 -6.205 8.553 1.00 0.00
ATOM 1449 HH11 ARG 89 5.649 -6.292 7.938 1.00 0.00
ATOM 1450 HH12 ARG 89 6.445 -5.522 9.297 1.00 0.00
ATOM 1451 NH2 ARG 89 8.510 -6.874 9.180 1.00 0.00
ATOM 1452 HH21 ARG 89 9.310 -7.477 9.051 1.00 0.00
ATOM 1453 HH22 ARG 89 8.502 -6.188 9.922 1.00 0.00
ATOM 1454 C ARG 89 2.335 -7.946 4.780 1.00 0.00
ATOM 1455 O ARG 89 2.527 -7.552 3.629 1.00 0.00
ATOM 1456 N LEU 90 1.209 -8.541 5.171 1.00 0.00
ATOM 1457 H LEU 90 1.111 -8.857 6.125 1.00 0.00
ATOM 1458 CA LEU 90 0.105 -8.774 4.248 1.00 0.00
ATOM 1459 HA LEU 90 0.439 -9.375 3.402 1.00 0.00
ATOM 1460 CB LEU 90 -1.014 -9.530 4.974 1.00 0.00
ATOM 1461 HB2 LEU 90 -0.597 -10.407 5.470 1.00 0.00
ATOM 1462 HB3 LEU 90 -1.459 -8.870 5.719 1.00 0.00
ATOM 1463 CG LEU 90 -2.090 -9.971 3.973 1.00 0.00
ATOM 1464 HG LEU 90 -1.632 -10.145 3.000 1.00 0.00
ATOM 1465 CD1 LEU 90 -2.746 -11.264 4.466 1.00 0.00
ATOM 1466 HD11 LEU 90 -3.204 -11.091 5.440 1.00 0.00

ATOM	1467	HD12	LEU	90	-3.510	-11.578	3.756	1.00	0.00
ATOM	1468	HD13	LEU	90	-1.990	-12.045	4.555	1.00	0.00
ATOM	1469	CD2	LEU	90	-3.160	-8.879	3.854	1.00	0.00
ATOM	1470	HD21	LEU	90	-2.699	-7.955	3.507	1.00	0.00
ATOM	1471	HD22	LEU	90	-3.923	-9.194	3.143	1.00	0.00
ATOM	1472	HD23	LEU	90	-3.619	-8.712	4.829	1.00	0.00
ATOM	1473	C	LEU	90	-0.430	-7.451	3.710	1.00	0.00
ATOM	1474	O	LEU	90	-0.755	-7.335	2.528	1.00	0.00
ATOM	1475	N	ALA	91	-0.525	-6.460	4.593	1.00	0.00
ATOM	1476	H	ALA	91	-0.264	-6.610	5.558	1.00	0.00
ATOM	1477	CA	ALA	91	-1.030	-5.148	4.209	1.00	0.00
ATOM	1478	HA	ALA	91	-2.070	-5.223	3.892	1.00	0.00
ATOM	1479	CB	ALA	91	-0.949	-4.188	5.399	1.00	0.00
ATOM	1480	HB1	ALA	91	0.088	-4.096	5.723	1.00	0.00
ATOM	1481	HB2	ALA	91	-1.324	-3.209	5.103	1.00	0.00
ATOM	1482	HB3	ALA	91	-1.552	-4.575	6.221	1.00	0.00
ATOM	1483	C	ALA	91	-0.228	-4.589	3.042	1.00	0.00
ATOM	1484	O	ALA	91	-0.795	-4.186	2.033	1.00	0.00
ATOM	1485	N	GLU	92	1.094	-4.577	3.190	1.00	0.00
ATOM	1486	H	GLU	92	1.507	-4.898	4.054	1.00	0.00
ATOM	1487	CA	GLU	92	1.982	-4.077	2.141	1.00	0.00
ATOM	1488	HA	GLU	92	1.747	-3.038	1.912	1.00	0.00

ATOM	1489	CB	GLU	92	3.436	-4.156	2.615	1.00	0.00
ATOM	1490	HB2	GLU	92	3.529	-3.654	3.578	1.00	0.00
ATOM	1491	HB3	GLU	92	3.725	-5.202	2.722	1.00	0.00
ATOM	1492	CG	GLU	92	4.355	-3.474	1.592	1.00	0.00
ATOM	1493	HG2	GLU	92	4.203	-3.937	0.617	1.00	0.00
ATOM	1494	HG3	GLU	92	4.096	-2.417	1.537	1.00	0.00
ATOM	1495	CD	GLU	92	5.822	-3.618	2.001	1.00	0.00
ATOM	1496	OE1	GLU	92	6.085	-4.254	3.012	1.00	0.00
ATOM	1497	OE2	GLU	92	6.664	-3.090	1.295	1.00	0.00
ATOM	1498	C	GLU	92	1.814	-4.879	0.851	1.00	0.00
ATOM	1499	O	GLU	92	2.007	-4.352	-0.244	1.00	0.00
ATOM	1500	N	ASN	93	1.470	-6.157	0.990	1.00	0.00
ATOM	1501	H	ASN	93	1.345	-6.556	1.910	1.00	0.00
ATOM	1502	CA	ASN	93	1.298	-7.028	-0.173	1.00	0.00
ATOM	1503	HA	ASN	93	2.160	-6.942	-0.835	1.00	0.00
ATOM	1504	CB	ASN	93	1.171	-8.487	0.279	1.00	0.00
ATOM	1505	HB2	ASN	93	0.349	-8.585	0.988	1.00	0.00
ATOM	1506	HB3	ASN	93	0.986	-9.129	-0.582	1.00	0.00
ATOM	1507	CG	ASN	93	2.460	-8.945	0.961	1.00	0.00
ATOM	1508	OD1	ASN	93	3.475	-8.129	1.049	1.00	0.00
ATOM	1509	ND2	ASN	93	2.542	-10.082	1.426	1.00	0.00
ATOM	1510	HD21	ASN	93	3.396	-10.380	1.876	1.00	0.00

```
ATOM   1511 HD22 ASN    93       1.755 -10.711   1.356  1.00  0.00
ATOM   1512  C   ASN    93       0.063  -6.638  -0.986  1.00  0.00
ATOM   1513  O   ASN    93       0.064  -6.751  -2.212  1.00  0.00
ATOM   1514  N   ILE    94      -0.989  -6.186  -0.303  1.00  0.00
ATOM   1515  H   ILE    94      -0.967  -6.144   0.706  1.00  0.00
ATOM   1516  CA  ILE    94      -2.221  -5.794  -0.987  1.00  0.00
ATOM   1517  HA  ILE    94      -2.670  -6.673  -1.450  1.00  0.00
ATOM   1518  CB  ILE    94      -3.209  -5.184   0.020  1.00  0.00
ATOM   1519  HB  ILE    94      -2.661  -4.556   0.723  1.00  0.00
ATOM   1520  CG2 ILE    94      -4.257  -4.337  -0.713  1.00  0.00
ATOM   1521 HG21 ILE    94      -4.805  -4.965  -1.415  1.00  0.00
ATOM   1522 HG22 ILE    94      -4.951  -3.911   0.011  1.00  0.00
ATOM   1523 HG23 ILE    94      -3.760  -3.534  -1.256  1.00  0.00
ATOM   1524  CG1 ILE    94      -3.913  -6.315   0.782  1.00  0.00
ATOM   1525 HG12 ILE    94      -4.701  -6.736   0.158  1.00  0.00
ATOM   1526 HG13 ILE    94      -3.188  -7.093   1.026  1.00  0.00
ATOM   1527  CD1 ILE    94      -4.525  -5.765   2.074  1.00  0.00
ATOM   1528 HD11 ILE    94      -5.249  -4.988   1.832  1.00  0.00
ATOM   1529 HD12 ILE    94      -5.024  -6.571   2.612  1.00  0.00
ATOM   1530 HD13 ILE    94      -3.737  -5.345   2.700  1.00  0.00
ATOM   1531  C   ILE    94      -1.925  -4.803  -2.121  1.00  0.00
ATOM   1532  O   ILE    94      -2.278  -5.053  -3.273  1.00  0.00
```

ATOM 1533 N PRO 95 -1.285 -3.698 -1.825 1.00 0.00
ATOM 1534 CD PRO 95 -0.829 -3.302 -0.484 1.00 0.00
ATOM 1535 HD2 PRO 95 -0.258 -4.121 -0.046 1.00 0.00
ATOM 1536 HD3 PRO 95 -1.698 -3.092 0.140 1.00 0.00
ATOM 1537 CG PRO 95 0.023 -2.061 -0.732 1.00 0.00
ATOM 1538 HG2 PRO 95 1.073 -2.350 -0.793 1.00 0.00
ATOM 1539 HG3 PRO 95 -0.112 -1.359 0.091 1.00 0.00
ATOM 1540 CB PRO 95 -0.456 -1.476 -2.014 1.00 0.00
ATOM 1541 HB2 PRO 95 0.374 -0.982 -2.518 1.00 0.00
ATOM 1542 HB3 PRO 95 -1.271 -0.767 -1.875 1.00 0.00
ATOM 1543 CA PRO 95 -0.936 -2.667 -2.847 1.00 0.00
ATOM 1544 HA PRO 95 -1.839 -2.439 -3.413 1.00 0.00
ATOM 1545 C PRO 95 0.162 -3.131 -3.806 1.00 0.00
ATOM 1546 O PRO 95 0.257 -2.636 -4.925 1.00 0.00
ATOM 1547 N SER 96 0.981 -4.091 -3.366 1.00 0.00
ATOM 1548 H SER 96 0.875 -4.465 -2.434 1.00 0.00
ATOM 1549 CA SER 96 2.056 -4.617 -4.206 1.00 0.00
ATOM 1550 HA SER 96 2.717 -3.805 -4.509 1.00 0.00
ATOM 1551 CB SER 96 2.878 -5.657 -3.442 1.00 0.00
ATOM 1552 HB2 SER 96 2.237 -6.492 -3.158 1.00 0.00
ATOM 1553 HB3 SER 96 3.687 -6.020 -4.076 1.00 0.00
ATOM 1554 OG SER 96 3.423 -5.061 -2.274 1.00 0.00

ATOM	1555	HG	SER	96	3.939	-5.713	-1.793	1.00	0.00
ATOM	1556	C	SER	96	1.443	-5.247	-5.429	1.00	0.00
ATOM	1557	O	SER	96	1.919	-5.097	-6.555	1.00	0.00
ATOM	1558	N	ARG	97	0.362	-5.946	-5.160	1.00	0.00
ATOM	1559	H	ARG	97	0.061	-6.025	-4.199	1.00	0.00
ATOM	1560	CA	ARG	97	-0.400	-6.629	-6.172	1.00	0.00
ATOM	1561	HA	ARG	97	0.192	-7.410	-6.650	1.00	0.00
ATOM	1562	CB	ARG	97	-1.599	-7.276	-5.491	1.00	0.00
ATOM	1563	HB2	ARG	97	-2.058	-6.560	-4.810	1.00	0.00
ATOM	1564	HB3	ARG	97	-2.325	-7.575	-6.246	1.00	0.00
ATOM	1565	CG	ARG	97	-1.144	-8.510	-4.703	1.00	0.00
ATOM	1566	HG2	ARG	97	-0.605	-9.185	-5.368	1.00	0.00
ATOM	1567	HG3	ARG	97	-0.485	-8.198	-3.892	1.00	0.00
ATOM	1568	CD	ARG	97	-2.361	-9.233	-4.121	1.00	0.00
ATOM	1569	HD2	ARG	97	-3.000	-9.590	-4.929	1.00	0.00
ATOM	1570	HD3	ARG	97	-2.034	-10.079	-3.516	1.00	0.00
ATOM	1571	NE	ARG	97	-3.136	-8.326	-3.277	1.00	0.00
ATOM	1572	HE	ARG	97	-2.869	-8.186	-2.313	1.00	0.00
ATOM	1573	CZ	ARG	97	-4.197	-7.670	-3.745	1.00	0.00
ATOM	1574	NH1	ARG	97	-4.583	-7.831	-4.983	1.00	0.00
ATOM	1575	HH11	ARG	97	-4.073	-8.457	-5.590	1.00	0.00
ATOM	1576	HH12	ARG	97	-5.388	-7.329	-5.327	1.00	0.00

ATOM 1577 NH2 ARG 97 -4.854 -6.861 -2.962 1.00 0.00
ATOM 1578 HH21 ARG 97 -4.555 -6.736 -2.005 1.00 0.00
ATOM 1579 HH22 ARG 97 -5.658 -6.363 -3.315 1.00 0.00
ATOM 1580 C ARG 97 -0.865 -5.672 -7.269 1.00 0.00
ATOM 1581 O ARG 97 -0.921 -6.046 -8.441 1.00 0.00
ATOM 1582 N CYX 98 -1.188 -4.437 -6.886 1.00 0.00
ATOM 1583 H CYX 98 -1.124 -4.169 -5.914 1.00 0.00
ATOM 1584 CA CYX 98 -1.635 -3.442 -7.848 1.00 0.00
ATOM 1585 HA CYX 98 -1.950 -3.940 -8.765 1.00 0.00
ATOM 1586 CB CYX 98 -2.811 -2.635 -7.301 1.00 0.00
ATOM 1587 HB2 CYX 98 -2.445 -1.698 -6.881 1.00 0.00
ATOM 1588 HB3 CYX 98 -3.512 -2.422 -8.107 1.00 0.00
ATOM 1589 SG CYX 98 -3.640 -3.569 -5.988 1.00 0.00
ATOM 1590 C CYX 98 -0.515 -2.483 -8.162 1.00 0.00
ATOM 1591 O CYX 98 -0.632 -1.667 -9.076 1.00 0.00
ATOM 1592 N ASN 99 0.558 -2.549 -7.380 1.00 0.00
ATOM 1593 H ASN 99 0.607 -3.180 -6.592 1.00 0.00
ATOM 1594 CA ASN 99 1.653 -1.632 -7.591 1.00 0.00
ATOM 1595 HA ASN 99 1.272 -0.618 -7.471 1.00 0.00
ATOM 1596 CB ASN 99 2.810 -1.827 -6.642 1.00 0.00
ATOM 1597 HB2 ASN 99 2.945 -2.884 -6.413 1.00 0.00
ATOM 1598 HB3 ASN 99 3.725 -1.432 -7.084 1.00 0.00

```
ATOM   1599  CG  ASN    99       2.534  -1.079  -5.343  1.00  0.00
ATOM   1600  OD1 ASN    99       2.830  -1.576  -4.257  1.00  0.00
ATOM   1601  ND2 ASN    99       1.984   0.105  -5.398  1.00  0.00
ATOM   1602 HD21 ASN    99       1.796   0.611  -4.544  1.00  0.00
ATOM   1603 HD22 ASN    99       1.750   0.508  -6.293  1.00  0.00
ATOM   1604  C   ASN    99       2.149  -1.693  -9.002  1.00  0.00
ATOM   1605  O   ASN    99       1.977  -2.679  -9.722  1.00  0.00
ATOM   1606  N   LEU   100       2.678  -0.568  -9.397  1.00  0.00
ATOM   1607  H   LEU   100       2.780   0.191  -8.738  1.00  0.00
ATOM   1608  CA  LEU   100       3.125  -0.363 -10.749  1.00  0.00
ATOM   1609  HA  LEU   100       3.453  -1.293 -11.213  1.00  0.00
ATOM   1610  CB  LEU   100       1.915   0.172 -11.524  1.00  0.00
ATOM   1611  HB2 LEU   100       2.231   1.042 -12.099  1.00  0.00
ATOM   1612  HB3 LEU   100       1.516  -0.585 -12.199  1.00  0.00
ATOM   1613  CG  LEU   100       0.815   0.593 -10.511  1.00  0.00
ATOM   1614  HG  LEU   100       0.816  -0.100  -9.669  1.00  0.00
ATOM   1615  CD1 LEU   100       1.090   2.009 -10.005  1.00  0.00
ATOM   1616 HD11 LEU   100       1.089   2.702 -10.846  1.00  0.00
ATOM   1617 HD12 LEU   100       0.316   2.298  -9.295  1.00  0.00
ATOM   1618 HD13 LEU   100       2.062   2.037  -9.512  1.00  0.00
ATOM   1619  CD2 LEU   100      -0.556   0.526 -11.167  1.00  0.00
ATOM   1620 HD21 LEU   100      -0.750  -0.494 -11.501  1.00  0.00
```

```
ATOM   1621 HD22 LEU   100      -1.318   0.823 -10.447  1.00  0.00
ATOM   1622 HD23 LEU   100      -0.583   1.200 -12.023  1.00  0.00
ATOM   1623  C   LEU   100       4.274   0.640 -10.779  1.00  0.00
ATOM   1624  O   LEU   100       4.248   1.634 -10.056  1.00  0.00
ATOM   1625  N   SER   101       5.278   0.359 -11.613  1.00  0.00
ATOM   1626  H   SER   101       5.244  -0.485 -12.167  1.00  0.00
ATOM   1627  CA  SER   101       6.454   1.225 -11.738  1.00  0.00
ATOM   1628  HA  SER   101       7.191   0.972 -10.975  1.00  0.00
ATOM   1629  CB  SER   101       7.091   1.033 -13.116  1.00  0.00
ATOM   1630  HB2 SER   101       7.378  -0.011 -13.243  1.00  0.00
ATOM   1631  HB3 SER   101       6.376   1.310 -13.890  1.00  0.00
ATOM   1632  OG  SER   101       8.245   1.857 -13.220  1.00  0.00
ATOM   1633  HG  SER   101       8.647   1.738 -14.084  1.00  0.00
ATOM   1634  C   SER   101       6.077   2.694 -11.550  1.00  0.00
ATOM   1635  O   SER   101       5.628   3.358 -12.485  1.00  0.00
ATOM   1636  N   PRO   102       6.248   3.203 -10.355  1.00  0.00
ATOM   1637  CD  PRO   102       6.762   2.497  -9.185  1.00  0.00
ATOM   1638  HD2 PRO   102       7.837   2.655  -9.091  1.00  0.00
ATOM   1639  HD3 PRO   102       6.559   1.429  -9.270  1.00  0.00
ATOM   1640  CG  PRO   102       5.975   3.149  -8.060  1.00  0.00
ATOM   1641  HG2 PRO   102       6.558   3.101  -7.140  1.00  0.00
ATOM   1642  HG3 PRO   102       5.035   2.614  -7.922  1.00  0.00
```

```
ATOM   1643  CB  PRO   102       5.727   4.571   -8.480  1.00  0.00
ATOM   1644  HB2 PRO   102       6.470   5.203   -7.993  1.00  0.00
ATOM   1645  HB3 PRO   102       4.728   4.923   -8.225  1.00  0.00
ATOM   1646  CA  PRO   102       5.925   4.611 -10.009  1.00  0.00
ATOM   1647  HA  PRO   102       5.014   4.893 -10.536  1.00  0.00
ATOM   1648  C   PRO   102       7.052   5.563 -10.397  1.00  0.00
ATOM   1649  O   PRO   102       8.170   5.451   -9.890  1.00  0.00
ATOM   1650  N   MET   103       6.756   6.495 -11.299  1.00  0.00
ATOM   1651  H   MET   103       5.833   6.546 -11.706  1.00  0.00
ATOM   1652  CA  MET   103       7.761   7.452 -11.743  1.00  0.00
ATOM   1653  HA  MET   103       8.666   6.928 -12.051  1.00  0.00
ATOM   1654  CB  MET   103       7.228   8.253 -12.936  1.00  0.00
ATOM   1655  HB2 MET   103       6.302   8.751 -12.651  1.00  0.00
ATOM   1656  HB3 MET   103       7.967   8.999 -13.228  1.00  0.00
ATOM   1657  CG  MET   103       6.959   7.311 -14.115  1.00  0.00
ATOM   1658  HG2 MET   103       6.172   6.606 -13.847  1.00  0.00
ATOM   1659  HG3 MET   103       6.646   7.891 -14.982  1.00  0.00
ATOM   1660  SD  MET   103       8.466   6.394 -14.525  1.00  0.00
ATOM   1661  CE  MET   103       9.482   7.811 -15.012  1.00  0.00
ATOM   1662  HE1 MET   103       9.575   8.498 -14.171  1.00  0.00
ATOM   1663  HE2 MET   103      10.472   7.464 -15.308  1.00  0.00
ATOM   1664  HE3 MET   103       9.012   8.324 -15.850  1.00  0.00
```

ATOM	1665	C	MET	103	8.129	8.405	-10.611	1.00	0.00
ATOM	1666	O	MET	103	9.295	8.497	-10.222	1.00	0.00
ATOM	1667	N	ARG	104	7.128	9.105	-10.080	1.00	0.00
ATOM	1668	H	ARG	104	6.189	9.012	-10.439	1.00	0.00
ATOM	1669	CA	ARG	104	7.360	10.040	-8.983	1.00	0.00
ATOM	1670	HA	ARG	104	8.181	9.689	-8.358	1.00	0.00
ATOM	1671	CB	ARG	104	7.727	11.420	-9.545	1.00	0.00
ATOM	1672	HB2	ARG	104	7.427	11.472	-10.591	1.00	0.00
ATOM	1673	HB3	ARG	104	7.203	12.189	-8.978	1.00	0.00
ATOM	1674	CG	ARG	104	9.241	11.646	-9.435	1.00	0.00
ATOM	1675	HG2	ARG	104	9.763	10.830	-9.936	1.00	0.00
ATOM	1676	HG3	ARG	104	9.499	12.590	-9.914	1.00	0.00
ATOM	1677	CD	ARG	104	9.660	11.691	-7.960	1.00	0.00
ATOM	1678	HD2	ARG	104	9.835	10.678	-7.597	1.00	0.00
ATOM	1679	HD3	ARG	104	10.574	12.276	-7.855	1.00	0.00
ATOM	1680	NE	ARG	104	8.614	12.306	-7.146	1.00	0.00
ATOM	1681	HE	ARG	104	8.012	11.726	-6.579	1.00	0.00
ATOM	1682	CZ	ARG	104	8.426	13.624	-7.129	1.00	0.00
ATOM	1683	NH1	ARG	104	9.189	14.406	-7.845	1.00	0.00
ATOM	1684	HH11	ARG	104	9.924	14.008	-8.412	1.00	0.00
ATOM	1685	HH12	ARG	104	9.041	15.405	-7.827	1.00	0.00
ATOM	1686	NH2	ARG	104	7.479	14.135	-6.394	1.00	0.00

ATOM	1687	HH21	ARG	104	6.891	13.528	-5.840	1.00	0.00
ATOM	1688	HH22	ARG	104	7.338	15.135	-6.382	1.00	0.00
ATOM	1689	C	ARG	104	6.122	10.169	-8.097	1.00	0.00
ATOM	1690	O	ARG	104	4.997	10.226	-8.593	1.00	0.00
ATOM	1691	N	CYX	105	6.347	10.246	-6.786	1.00	0.00
ATOM	1692	H	CYX	105	7.289	10.178	-6.427	1.00	0.00
ATOM	1693	CA	CYX	105	5.253	10.406	-5.832	1.00	0.00
ATOM	1694	HA	CYX	105	4.300	10.234	-6.331	1.00	0.00
ATOM	1695	CB	CYX	105	5.392	9.402	-4.685	1.00	0.00
ATOM	1696	HB2	CYX	105	6.428	9.378	-4.346	1.00	0.00
ATOM	1697	HB3	CYX	105	4.747	9.701	-3.859	1.00	0.00
ATOM	1698	SG	CYX	105	4.933	7.748	-5.265	1.00	0.00
ATOM	1699	C	CYX	105	5.259	11.830	-5.274	1.00	0.00
ATOM	1700	O	CYX	105	6.168	12.202	-4.532	1.00	0.00
ATOM	1701	N	PRO	106	4.276	12.630	-5.616	1.00	0.00
ATOM	1702	CD	PRO	106	3.158	12.283	-6.505	1.00	0.00
ATOM	1703	HD2	PRO	106	2.466	11.630	-5.972	1.00	0.00
ATOM	1704	HD3	PRO	106	3.545	11.763	-7.381	1.00	0.00
ATOM	1705	CG	PRO	106	2.537	13.636	-6.856	1.00	0.00
ATOM	1706	HG2	PRO	106	1.458	13.517	-6.952	1.00	0.00
ATOM	1707	HG3	PRO	106	2.950	13.981	-7.804	1.00	0.00
ATOM	1708	CB	PRO	106	2.886	14.576	-5.749	1.00	0.00

ATOM	1709	HB2	PRO	106	2.092	14.579	-5.002	1.00	0.00
ATOM	1710	HB3	PRO	106	3.041	15.590	-6.118	1.00	0.00
ATOM	1711	CA	PRO	106	4.179	14.043	-5.131	1.00	0.00
ATOM	1712	HA	PRO	106	5.051	14.579	-5.505	1.00	0.00
ATOM	1713	C	PRO	106	4.126	14.157	-3.613	1.00	0.00
ATOM	1714	O	PRO	106	3.949	15.247	-3.072	1.00	0.00
ATOM	1715	N	MET	107	4.280	13.034	-2.935	1.00	0.00
ATOM	1716	H	MET	107	4.438	12.161	-3.418	1.00	0.00
ATOM	1717	CA	MET	107	4.248	13.021	-1.484	1.00	0.00
ATOM	1718	HA	MET	107	3.421	13.627	-1.114	1.00	0.00
ATOM	1719	CB	MET	107	4.056	11.603	-1.009	1.00	0.00
ATOM	1720	HB2	MET	107	4.662	10.937	-1.623	1.00	0.00
ATOM	1721	HB3	MET	107	4.377	11.529	0.030	1.00	0.00
ATOM	1722	CG	MET	107	2.581	11.198	-1.118	1.00	0.00
ATOM	1723	HG2	MET	107	2.502	10.264	-1.675	1.00	0.00
ATOM	1724	HG3	MET	107	2.169	11.060	-0.118	1.00	0.00
ATOM	1725	SD	MET	107	1.640	12.487	-1.979	1.00	0.00
ATOM	1726	CE	MET	107	1.617	11.713	-3.613	1.00	0.00
ATOM	1727	HE1	MET	107	1.122	10.744	-3.550	1.00	0.00
ATOM	1728	HE2	MET	107	1.077	12.352	-4.311	1.00	0.00
ATOM	1729	HE3	MET	107	2.640	11.575	-3.964	1.00	0.00
ATOM	1730	C	MET	107	5.555	13.543	-0.933	1.00	0.00

ATOM	1731	O	MET	107	6.504	12.790	-0.706	1.00	0.00
ATOM	1732	N	GLY	108	5.589	14.839	-0.726	1.00	0.00
ATOM	1733	H	GLY	108	4.781	15.409	-0.931	1.00	0.00
ATOM	1734	CA	GLY	108	6.772	15.488	-0.203	1.00	0.00
ATOM	1735	HA2	GLY	108	6.508	16.484	0.151	1.00	0.00
ATOM	1736	HA3	GLY	108	7.167	14.901	0.627	1.00	0.00
ATOM	1737	C	GLY	108	7.846	15.612	-1.278	1.00	0.00
ATOM	1738	O	GLY	108	9.038	15.668	-0.974	1.00	0.00
ATOM	1739	N	GLY	109	7.409	15.661	-2.535	1.00	0.00
ATOM	1740	H	GLY	109	6.420	15.610	-2.732	1.00	0.00
ATOM	1741	CA	GLY	109	8.334	15.788	-3.658	1.00	0.00
ATOM	1742	HA2	GLY	109	7.779	15.679	-4.589	1.00	0.00
ATOM	1743	HA3	GLY	109	8.800	16.773	-3.625	1.00	0.00
ATOM	1744	C	GLY	109	9.425	14.721	-3.603	1.00	0.00
ATOM	1745	O	GLY	109	10.596	15.033	-3.382	1.00	0.00
ATOM	1746	N	SER	110	9.034	13.463	-3.807	1.00	0.00
ATOM	1747	H	SER	110	8.060	13.248	-3.963	1.00	0.00
ATOM	1748	CA	SER	110	9.992	12.358	-3.780	1.00	0.00
ATOM	1749	HA	SER	110	10.422	12.261	-2.783	1.00	0.00
ATOM	1750	CB	SER	110	9.292	11.048	-4.139	1.00	0.00
ATOM	1751	HB2	SER	110	8.928	11.098	-5.165	1.00	0.00
ATOM	1752	HB3	SER	110	9.995	10.220	-4.043	1.00	0.00

```
ATOM   1753  OG  SER   110       8.196  10.843  -3.256  1.00  0.00
ATOM   1754  HG  SER   110       7.755  10.020  -3.479  1.00  0.00
ATOM   1755  C   SER   110      11.135  12.619  -4.759  1.00  0.00
ATOM   1756  O   SER   110      10.978  13.370  -5.722  1.00  0.00
ATOM   1757  N   ILE   111      12.286  12.002  -4.504  1.00  0.00
ATOM   1758  H   ILE   111      12.384  11.413  -3.689  1.00  0.00
ATOM   1759  CA  ILE   111      13.449  12.186  -5.369  1.00  0.00
ATOM   1760  HA  ILE   111      13.359  13.135  -5.897  1.00  0.00
ATOM   1761  CB  ILE   111      14.731  12.190  -4.528  1.00  0.00
ATOM   1762  HB  ILE   111      14.827  11.236  -4.008  1.00  0.00
ATOM   1763  CG2 ILE   111      15.945  12.398  -5.441  1.00  0.00
ATOM   1764 HG21 ILE   111      15.851  13.351  -5.960  1.00  0.00
ATOM   1765 HG22 ILE   111      16.855  12.400  -4.841  1.00  0.00
ATOM   1766 HG23 ILE   111      15.994  11.590  -6.171  1.00  0.00
ATOM   1767  CG1 ILE   111      14.662  13.328  -3.501  1.00  0.00
ATOM   1768 HG12 ILE   111      14.691  14.286  -4.020  1.00  0.00
ATOM   1769 HG13 ILE   111      13.733  13.249  -2.936  1.00  0.00
ATOM   1770  CD1 ILE   111      15.852  13.231  -2.542  1.00  0.00
ATOM   1771 HD11 ILE   111      16.782  13.309  -3.106  1.00  0.00
ATOM   1772 HD12 ILE   111      15.800  14.040  -1.814  1.00  0.00
ATOM   1773 HD13 ILE   111      15.824  12.273  -2.022  1.00  0.00
ATOM   1774  C   ILE   111      13.518  11.081  -6.421  1.00  0.00
```

ATOM	1775	O	ILE	111	13.126	11.281	-7.572	1.00	0.00
ATOM	1776	N	ALA	112	13.993	9.908	-6.007	1.00	0.00
ATOM	1777	H	ALA	112	14.294	9.796	-5.049	1.00	0.00
ATOM	1778	CA	ALA	112	14.084	8.764	-6.895	1.00	0.00
ATOM	1779	HA	ALA	112	14.573	9.010	-7.837	1.00	0.00
ATOM	1780	CB	ALA	112	14.861	7.636	-6.223	1.00	0.00
ATOM	1781	HB1	ALA	112	14.378	7.372	-5.282	1.00	0.00
ATOM	1782	HB2	ALA	112	14.879	6.765	-6.878	1.00	0.00
ATOM	1783	HB3	ALA	112	15.882	7.963	-6.027	1.00	0.00
ATOM	1784	C	ALA	112	12.693	8.288	-7.204	1.00	0.00
ATOM	1785	O	ALA	112	11.717	8.966	-6.878	1.00	0.00
ATOM	1786	N	GLY	113	12.579	7.104	-7.777	1.00	0.00
ATOM	1787	H	GLY	113	13.368	6.535	-8.050	1.00	0.00
ATOM	1788	CA	GLY	113	11.260	6.600	-8.023	1.00	0.00
ATOM	1789	HA2	GLY	113	10.606	7.385	-8.400	1.00	0.00
ATOM	1790	HA3	GLY	113	11.284	5.774	-8.734	1.00	0.00
ATOM	1791	C	GLY	113	10.715	6.097	-6.702	1.00	0.00
ATOM	1792	O	GLY	113	11.004	4.978	-6.276	1.00	0.00
ATOM	1793	N	PHE	114	9.955	6.964	-6.050	1.00	0.00
ATOM	1794	H	PHE	114	9.780	7.873	-6.453	1.00	0.00
ATOM	1795	CA	PHE	114	9.378	6.664	-4.744	1.00	0.00
ATOM	1796	HA	PHE	114	10.125	6.802	-3.962	1.00	0.00

```
ATOM   1797  CB  PHE   114       8.203   7.605  -4.469  1.00  0.00
ATOM   1798  HB2 PHE   114       8.491   8.654  -4.539  1.00  0.00
ATOM   1799  HB3 PHE   114       7.362   7.414  -5.135  1.00  0.00
ATOM   1800  CG  PHE   114       7.697   7.389  -3.060  1.00  0.00
ATOM   1801  CD1 PHE   114       8.305   8.057  -1.989  1.00  0.00
ATOM   1802  HD1 PHE   114       9.143   8.730  -2.170  1.00  0.00
ATOM   1803  CE1 PHE   114       7.836   7.859  -0.685  1.00  0.00
ATOM   1804  HE1 PHE   114       8.309   8.379   0.148  1.00  0.00
ATOM   1805  CZ  PHE   114       6.762   6.994  -0.450  1.00  0.00
ATOM   1806  HZ  PHE   114       6.399   6.840   0.566  1.00  0.00
ATOM   1807  CE2 PHE   114       6.154   6.326  -1.520  1.00  0.00
ATOM   1808  HE2 PHE   114       5.318   5.652  -1.337  1.00  0.00
ATOM   1809  CD2 PHE   114       6.621   6.524  -2.825  1.00  0.00
ATOM   1810  HD2 PHE   114       6.147   6.005  -3.658  1.00  0.00
ATOM   1811  C   PHE   114       8.898   5.213  -4.671  1.00  0.00
ATOM   1812  O   PHE   114       9.299   4.524  -3.748  1.00  0.00
ATOM   1813  OXT PHE   114       8.136   4.815  -5.538  1.00  0.00
TER
ATOM   1814  S1  LIG   115      16.459 -12.786  -5.790  1.00  0.00
ATOM   1815  O1  LIG   115      16.631 -14.160  -4.946  1.00  0.00
ATOM   1816  O2  LIG   115      17.812 -12.920  -6.663  1.00  0.00
ATOM   1817  H26 LIG   115      18.580 -12.717  -6.108  1.00  0.00
```

ATOM	1818	O3	LIG	115	15.362	-12.903	-6.718	1.00	0.00
ATOM	1819	O4	LIG	115	16.573	-11.658	-4.887	1.00	0.00
ATOM	1820	C1	LIG	115	14.408	-19.446	-0.086	1.00	0.00
ATOM	1821	H1	LIG	115	14.605	-20.214	-0.844	1.00	0.00
ATOM	1822	H2	LIG	115	15.345	-19.324	0.473	1.00	0.00
ATOM	1823	C2	LIG	115	13.314	-19.944	0.866	1.00	0.00
ATOM	1824	H3	LIG	115	12.378	-20.068	0.308	1.00	0.00
ATOM	1825	H4	LIG	115	13.117	-19.176	1.625	1.00	0.00
ATOM	1826	C3	LIG	115	14.058	-18.127	-0.782	1.00	0.00
ATOM	1827	H5	LIG	115	13.863	-17.357	-0.023	1.00	0.00
ATOM	1828	H6	LIG	115	13.120	-18.249	-1.339	1.00	0.00
ATOM	1829	C4	LIG	115	13.663	-21.264	1.565	1.00	0.00
ATOM	1830	H7	LIG	115	14.599	-21.141	2.124	1.00	0.00
ATOM	1831	H8	LIG	115	13.862	-22.033	0.807	1.00	0.00
ATOM	1832	C5	LIG	115	15.150	-17.630	-1.737	1.00	0.00
ATOM	1833	H9	LIG	115	16.089	-17.509	-1.182	1.00	0.00
ATOM	1834	H10	LIG	115	15.343	-18.396	-2.498	1.00	0.00
ATOM	1835	C6	LIG	115	12.569	-21.763	2.516	1.00	0.00
ATOM	1836	H11	LIG	115	11.633	-21.886	1.957	1.00	0.00
ATOM	1837	H12	LIG	115	12.371	-20.994	3.275	1.00	0.00
ATOM	1838	C7	LIG	115	14.798	-16.308	-2.429	1.00	0.00
ATOM	1839	H13	LIG	115	13.857	-16.429	-2.982	1.00	0.00

```
ATOM   1840  H14 LIG   115      14.610 -15.541  -1.667  1.00  0.00
ATOM   1841  C8  LIG   115      12.917 -23.082   3.215  1.00  0.00
ATOM   1842  H15 LIG   115      13.115 -23.853   2.457  1.00  0.00
ATOM   1843  H16 LIG   115      13.854 -22.960   3.773  1.00  0.00
ATOM   1844  C9  LIG   115      15.896 -15.824  -3.386  1.00  0.00
ATOM   1845  H17 LIG   115      16.075 -16.575  -4.162  1.00  0.00
ATOM   1846  H18 LIG   115      16.836 -15.694  -2.841  1.00  0.00
ATOM   1847  C10 LIG   115      11.824 -23.583   4.166  1.00  0.00
ATOM   1848  H19 LIG   115      10.888 -23.704   3.608  1.00  0.00
ATOM   1849  H20 LIG   115      11.627 -22.814   4.924  1.00  0.00
ATOM   1850  C11 LIG   115      15.525 -14.513  -4.050  1.00  0.00
ATOM   1851  H21 LIG   115      14.616 -14.606  -4.650  1.00  0.00
ATOM   1852  H22 LIG   115      15.401 -13.708  -3.321  1.00  0.00
ATOM   1853  C12 LIG   115      12.181 -24.902   4.859  1.00  0.00
ATOM   1854  H23 LIG   115      13.095 -24.801   5.453  1.00  0.00
ATOM   1855  H24 LIG   115      11.383 -25.229   5.530  1.00  0.00
ATOM   1856  H25 LIG   115      12.348 -25.699   4.128  1.00  0.00
TER
ATOM   1857  S1  LIG   116     -21.339  12.666   5.442  1.00  0.00
ATOM   1858  O1  LIG   116     -21.283  14.045   6.293  1.00  0.00
ATOM   1859  O2  LIG   116     -20.157  11.899   6.232  1.00  0.00
ATOM   1860  H26 LIG   116     -19.298  12.261   5.966  1.00  0.00
```

ATOM	1861	O3	LIG	116	-22.555	11.949	5.733	1.00	0.00
ATOM	1862	O4	LIG	116	-20.921	12.933	4.081	1.00	0.00
ATOM	1863	C1	LIG	116	-23.667	20.902	8.244	1.00	0.00
ATOM	1864	H1	LIG	116	-23.702	20.583	9.293	1.00	0.00
ATOM	1865	H2	LIG	116	-22.656	21.298	8.081	1.00	0.00
ATOM	1866	C2	LIG	116	-24.689	22.024	8.022	1.00	0.00
ATOM	1867	H3	LIG	116	-25.700	21.629	8.186	1.00	0.00
ATOM	1868	H4	LIG	116	-24.654	22.343	6.973	1.00	0.00
ATOM	1869	C3	LIG	116	-23.886	19.687	7.337	1.00	0.00
ATOM	1870	H5	LIG	116	-23.849	20.007	6.287	1.00	0.00
ATOM	1871	H6	LIG	116	-24.898	19.293	7.499	1.00	0.00
ATOM	1872	C4	LIG	116	-24.471	23.241	8.928	1.00	0.00
ATOM	1873	H7	LIG	116	-23.460	23.637	8.764	1.00	0.00
ATOM	1874	H8	LIG	116	-24.504	22.922	9.977	1.00	0.00
ATOM	1875	C5	LIG	116	-22.867	18.564	7.560	1.00	0.00
ATOM	1876	H9	LIG	116	-21.854	18.957	7.401	1.00	0.00
ATOM	1877	H10	LIG	116	-22.906	18.241	8.608	1.00	0.00
ATOM	1878	C6	LIG	116	-25.493	24.362	8.707	1.00	0.00
ATOM	1879	H11	LIG	116	-26.503	23.967	8.870	1.00	0.00
ATOM	1880	H12	LIG	116	-25.459	24.682	7.657	1.00	0.00
ATOM	1881	C7	LIG	116	-23.087	17.351	6.650	1.00	0.00
ATOM	1882	H13	LIG	116	-24.101	16.961	6.808	1.00	0.00

```
ATOM   1883  H14 LIG   116     -23.042  17.674   5.602  1.00  0.00
ATOM   1884  C8  LIG   116     -25.276  25.580   9.612  1.00  0.00
ATOM   1885  H15 LIG   116     -25.310  25.262  10.663  1.00  0.00
ATOM   1886  H16 LIG   116     -24.264  25.976   9.450  1.00  0.00
ATOM   1887  C9  LIG   116     -22.064  16.233   6.887  1.00  0.00
ATOM   1888  H17 LIG   116     -22.117  15.889   7.925  1.00  0.00
ATOM   1889  H18 LIG   116     -21.050  16.612   6.724  1.00  0.00
ATOM   1890  C10 LIG   116     -26.297  26.702   9.393  1.00  0.00
ATOM   1891  H19 LIG   116     -27.307  26.306   9.555  1.00  0.00
ATOM   1892  H20 LIG   116     -26.261  27.021   8.344  1.00  0.00
ATOM   1893  C11 LIG   116     -22.301  15.050   5.968  1.00  0.00
ATOM   1894  H21 LIG   116     -23.285  14.603   6.129  1.00  0.00
ATOM   1895  H22 LIG   116     -22.192  15.324   4.915  1.00  0.00
ATOM   1896  C12 LIG   116     -26.072  27.913  10.302  1.00  0.00
ATOM   1897  H23 LIG   116     -25.082  28.352  10.138  1.00  0.00
ATOM   1898  H24 LIG   116     -26.814  28.694  10.120  1.00  0.00
ATOM   1899  H25 LIG   116     -26.137  27.633  11.358  1.00  0.00
TER
ATOM   1900  S1  LIG   117      10.662  -9.386  25.795  1.00  0.00
ATOM   1901  O1  LIG   117      11.743  -8.464  25.014  1.00  0.00
ATOM   1902  O2  LIG   117       9.349  -8.538  25.390  1.00  0.00
ATOM   1903  H26 LIG   117       9.318  -7.717  25.905  1.00  0.00
```

ATOM	1904	O3	LIG	117	10.536	-10.667	25.147	1.00	0.00
ATOM	1905	O4	LIG	117	10.890	-9.277	27.222	1.00	0.00
ATOM	1906	C1	LIG	117	18.614	-6.733	22.505	1.00	0.00
ATOM	1907	H1	LIG	117	18.209	-6.630	21.491	1.00	0.00
ATOM	1908	H2	LIG	117	18.532	-5.742	22.968	1.00	0.00
ATOM	1909	C2	LIG	117	20.094	-7.128	22.421	1.00	0.00
ATOM	1910	H3	LIG	117	20.177	-8.120	21.958	1.00	0.00
ATOM	1911	H4	LIG	117	20.499	-7.232	23.436	1.00	0.00
ATOM	1912	C3	LIG	117	17.759	-7.732	23.293	1.00	0.00
ATOM	1913	H5	LIG	117	18.163	-7.833	24.308	1.00	0.00
ATOM	1914	H6	LIG	117	17.845	-8.724	22.831	1.00	0.00
ATOM	1915	C4	LIG	117	20.952	-6.130	21.634	1.00	0.00
ATOM	1916	H7	LIG	117	20.869	-5.138	22.098	1.00	0.00
ATOM	1917	H8	LIG	117	20.546	-6.025	20.620	1.00	0.00
ATOM	1918	C5	LIG	117	16.280	-7.339	23.375	1.00	0.00
ATOM	1919	H9	LIG	117	16.193	-6.346	23.834	1.00	0.00
ATOM	1920	H10	LIG	117	15.873	-7.241	22.360	1.00	0.00
ATOM	1921	C6	LIG	117	22.431	-6.525	21.549	1.00	0.00
ATOM	1922	H11	LIG	117	22.513	-7.517	21.086	1.00	0.00
ATOM	1923	H12	LIG	117	22.837	-6.630	22.564	1.00	0.00
ATOM	1924	C7	LIG	117	15.428	-8.338	24.165	1.00	0.00
ATOM	1925	H13	LIG	117	15.517	-9.331	23.706	1.00	0.00

ATOM 1926 H14 LIG 117 15.831 -8.432 25.181 1.00 0.00

ATOM 1927 C8 LIG 117 23.289 -5.528 20.763 1.00 0.00

ATOM 1928 H15 LIG 117 22.885 -5.423 19.747 1.00 0.00

ATOM 1929 H16 LIG 117 23.206 -4.535 21.225 1.00 0.00

ATOM 1930 C9 LIG 117 13.948 -7.937 24.235 1.00 0.00

ATOM 1931 H17 LIG 117 13.531 -7.862 23.225 1.00 0.00

ATOM 1932 H18 LIG 117 13.849 -6.950 24.698 1.00 0.00

ATOM 1933 C10 LIG 117 24.768 -5.921 20.677 1.00 0.00

ATOM 1934 H19 LIG 117 24.850 -6.913 20.215 1.00 0.00

ATOM 1935 H20 LIG 117 25.173 -6.025 21.691 1.00 0.00

ATOM 1936 C11 LIG 117 13.131 -8.938 25.029 1.00 0.00

ATOM 1937 H21 LIG 117 13.157 -9.932 24.575 1.00 0.00

ATOM 1938 H22 LIG 117 13.463 -9.001 26.068 1.00 0.00

ATOM 1939 C12 LIG 117 25.617 -4.919 19.889 1.00 0.00

ATOM 1940 H23 LIG 117 25.581 -3.925 20.346 1.00 0.00

ATOM 1941 H24 LIG 117 26.665 -5.228 19.848 1.00 0.00

ATOM 1942 H25 LIG 117 25.259 -4.821 18.859 1.00 0.00

TER

ATOM 1943 S1 LIG 118 9.754 27.226 -7.170 1.00 0.00

ATOM 1944 O1 LIG 118 10.381 26.308 -5.989 1.00 0.00

ATOM 1945 O2 LIG 118 10.654 26.638 -8.375 1.00 0.00

ATOM 1946 H26 LIG 118 11.556 26.987 -8.311 1.00 0.00

ATOM	1947	O3	LIG	118	8.396	26.830	-7.444	1.00	0.00
ATOM	1948	O4	LIG	118	10.108	28.611	-6.931	1.00	0.00
ATOM	1949	C1	LIG	118	10.803	23.668	1.037	1.00	0.00
ATOM	1950	H1	LIG	118	10.755	22.644	0.646	1.00	0.00
ATOM	1951	H2	LIG	118	11.868	23.932	1.070	1.00	0.00
ATOM	1952	C2	LIG	118	10.233	23.693	2.460	1.00	0.00
ATOM	1953	H3	LIG	118	9.168	23.429	2.428	1.00	0.00
ATOM	1954	H4	LIG	118	10.279	24.718	2.851	1.00	0.00
ATOM	1955	C3	LIG	118	10.078	24.612	0.071	1.00	0.00
ATOM	1956	H5	LIG	118	10.127	25.638	0.461	1.00	0.00
ATOM	1957	H6	LIG	118	9.012	24.349	0.040	1.00	0.00
ATOM	1958	C4	LIG	118	10.957	22.751	3.428	1.00	0.00
ATOM	1959	H7	LIG	118	12.022	23.016	3.462	1.00	0.00
ATOM	1960	H8	LIG	118	10.912	21.726	3.037	1.00	0.00
ATOM	1961	C5	LIG	118	10.645	24.585	-1.353	1.00	0.00
ATOM	1962	H9	LIG	118	11.711	24.845	-1.323	1.00	0.00
ATOM	1963	H10	LIG	118	10.593	23.562	-1.745	1.00	0.00
ATOM	1964	C6	LIG	118	10.387	22.775	4.851	1.00	0.00
ATOM	1965	H11	LIG	118	9.322	22.511	4.818	1.00	0.00
ATOM	1966	H12	LIG	118	10.433	23.800	5.242	1.00	0.00
ATOM	1967	C7	LIG	118	9.920	25.532	-2.315	1.00	0.00
ATOM	1968	H13	LIG	118	8.854	25.273	-2.342	1.00	0.00

```
ATOM   1969  H14 LIG   118       9.977  26.556  -1.925  1.00  0.00
ATOM   1970  C8  LIG   118      11.110  21.833   5.820  1.00  0.00
ATOM   1971  H15 LIG   118      11.065  20.807   5.430  1.00  0.00
ATOM   1972  H16 LIG   118      12.176  22.096   5.852  1.00  0.00
ATOM   1973  C9  LIG   118      10.493  25.492  -3.738  1.00  0.00
ATOM   1974  H17 LIG   118      10.421  24.477  -4.144  1.00  0.00
ATOM   1975  H18 LIG   118      11.556  25.757  -3.721  1.00  0.00
ATOM   1976  C10 LIG   118      10.542  21.856   7.243  1.00  0.00
ATOM   1977  H19 LIG   118       9.477  21.593   7.209  1.00  0.00
ATOM   1978  H20 LIG   118      10.589  22.880   7.633  1.00  0.00
ATOM   1979  C11 LIG   118       9.762  26.443  -4.666  1.00  0.00
ATOM   1980  H21 LIG   118       8.704  26.187  -4.756  1.00  0.00
ATOM   1981  H22 LIG   118       9.862  27.483  -4.343  1.00  0.00
ATOM   1982  C12 LIG   118      11.271  20.910   8.202  1.00  0.00
ATOM   1983  H23 LIG   118      12.331  21.170   8.283  1.00  0.00
ATOM   1984  H24 LIG   118      10.842  20.952   9.207  1.00  0.00
ATOM   1985  H25 LIG   118      11.211  19.874   7.858  1.00  0.00
TER
ATOM   1986  S1  LIG   119     -24.976  19.284 -10.074  1.00  0.00
ATOM   1987  O1  LIG   119     -25.293  19.923 -11.530  1.00  0.00
ATOM   1988  O2  LIG   119     -25.517  20.524  -9.193  1.00  0.00
ATOM   1989  H26 LIG   119     -24.881  21.255  -9.231  1.00  0.00
```

```
ATOM   1990  O3  LIG   119     -25.862  18.178  -9.810  1.00  0.00
ATOM   1991  O4  LIG   119     -23.541  19.162  -9.914  1.00  0.00
ATOM   1992  C1  LIG   119     -25.734  19.796 -19.034  1.00  0.00
ATOM   1993  H1  LIG   119     -26.778  20.126 -18.970  1.00  0.00
ATOM   1994  H2  LIG   119     -25.128  20.710 -19.083  1.00  0.00
ATOM   1995  C2  LIG   119     -25.536  18.991 -20.323  1.00  0.00
ATOM   1996  H3  LIG   119     -26.142  18.077 -20.275  1.00  0.00
ATOM   1997  H4  LIG   119     -24.491  18.659 -20.388  1.00  0.00
ATOM   1998  C3  LIG   119     -25.371  19.017 -17.765  1.00  0.00
ATOM   1999  H5  LIG   119     -24.325  18.688 -17.828  1.00  0.00
ATOM   2000  H6  LIG   119     -25.975  18.101 -17.718  1.00  0.00
ATOM   2001  C4  LIG   119     -25.897  19.768 -21.594  1.00  0.00
ATOM   2002  H7  LIG   119     -25.291  20.683 -21.644  1.00  0.00
ATOM   2003  H8  LIG   119     -26.941  20.101 -21.530  1.00  0.00
ATOM   2004  C5  LIG   119     -25.571  19.820 -16.475  1.00  0.00
ATOM   2005  H9  LIG   119     -24.970  20.737 -16.522  1.00  0.00
ATOM   2006  H10 LIG   119     -26.617  20.146 -16.409  1.00  0.00
ATOM   2007  C6  LIG   119     -25.701  18.963 -22.884  1.00  0.00
ATOM   2008  H11 LIG   119     -26.306  18.049 -22.835  1.00  0.00
ATOM   2009  H12 LIG   119     -24.656  18.631 -22.948  1.00  0.00
ATOM   2010  C7  LIG   119     -25.205  19.039 -15.208  1.00  0.00
ATOM   2011  H13 LIG   119     -25.805  18.122 -15.163  1.00  0.00
```

```
ATOM   2012  H14 LIG   119     -24.157  18.719 -15.272  1.00  0.00
ATOM   2013  C8  LIG   119     -26.062  19.740 -24.155  1.00  0.00
ATOM   2014  H15 LIG   119     -27.107  20.072 -24.093  1.00  0.00
ATOM   2015  H16 LIG   119     -25.457  20.655 -24.204  1.00  0.00
ATOM   2016  C9  LIG   119     -25.415  19.853 -13.924  1.00  0.00
ATOM   2017  H17 LIG   119     -26.463  20.159 -13.840  1.00  0.00
ATOM   2018  H18 LIG   119     -24.814  20.767 -13.956  1.00  0.00
ATOM   2019  C10 LIG   119     -25.866  18.936 -25.446  1.00  0.00
ATOM   2020  H19 LIG   119     -26.470  18.022 -25.396  1.00  0.00
ATOM   2021  H20 LIG   119     -24.822  18.606 -25.509  1.00  0.00
ATOM   2022  C11 LIG   119     -25.038  19.059 -12.688  1.00  0.00
ATOM   2023  H21 LIG   119     -25.648  18.159 -12.583  1.00  0.00
ATOM   2024  H22 LIG   119     -23.979  18.785 -12.688  1.00  0.00
ATOM   2025  C12 LIG   119     -26.230  19.721 -26.710  1.00  0.00
ATOM   2026  H23 LIG   119     -25.618  20.624 -26.804  1.00  0.00
ATOM   2027  H24 LIG   119     -26.078  19.121 -27.611  1.00  0.00
ATOM   2028  H25 LIG   119     -27.278  20.034 -26.692  1.00  0.00
TER
ATOM   2029  S1  LIG   120      -3.728  10.132 -23.690  1.00  0.00
ATOM   2030  O1  LIG   120      -4.159   9.413 -22.302  1.00  0.00
ATOM   2031  O2  LIG   120      -2.136   9.908 -23.543  1.00  0.00
ATOM   2032  H26 LIG   120      -1.779  10.522 -22.883  1.00  0.00
```

```
ATOM   2033  O3  LIG   120      -4.149   9.337 -24.816  1.00  0.00
ATOM   2034  O4  LIG   120      -4.031  11.547 -23.604  1.00  0.00
ATOM   2035  C1  LIG   120      -9.098   6.760 -17.294  1.00  0.00
ATOM   2036  H1  LIG   120      -8.585   5.791 -17.314  1.00  0.00
ATOM   2037  H2  LIG   120      -8.610   7.347 -16.505  1.00  0.00
ATOM   2038  C2  LIG   120     -10.571   6.546 -16.925  1.00  0.00
ATOM   2039  H3  LIG   120     -11.059   5.959 -17.713  1.00  0.00
ATOM   2040  H4  LIG   120     -11.085   7.516 -16.906  1.00  0.00
ATOM   2041  C3  LIG   120      -8.902   7.464 -18.642  1.00  0.00
ATOM   2042  H5  LIG   120      -9.414   8.435 -18.621  1.00  0.00
ATOM   2043  H6  LIG   120      -9.393   6.879 -19.430  1.00  0.00
ATOM   2044  C4  LIG   120     -10.770   5.843 -15.577  1.00  0.00
ATOM   2045  H7  LIG   120     -10.282   6.430 -14.787  1.00  0.00
ATOM   2046  H8  LIG   120     -10.255   4.874 -15.595  1.00  0.00
ATOM   2047  C5  LIG   120      -7.430   7.676 -19.013  1.00  0.00
ATOM   2048  H9  LIG   120      -6.937   8.259 -18.224  1.00  0.00
ATOM   2049  H10 LIG   120      -6.918   6.706 -19.038  1.00  0.00
ATOM   2050  C6  LIG   120     -12.242   5.627 -15.208  1.00  0.00
ATOM   2051  H11 LIG   120     -12.730   5.041 -15.997  1.00  0.00
ATOM   2052  H12 LIG   120     -12.756   6.597 -15.189  1.00  0.00
ATOM   2053  C7  LIG   120      -7.237   8.383 -20.359  1.00  0.00
ATOM   2054  H13 LIG   120      -7.732   7.800 -21.147  1.00  0.00
```

```
ATOM   2055  H14 LIG   120      -7.745   9.355 -20.332  1.00  0.00
ATOM   2056  C8  LIG   120     -12.441   4.925 -13.860  1.00  0.00
ATOM   2057  H15 LIG   120     -11.928   3.954 -13.877  1.00  0.00
ATOM   2058  H16 LIG   120     -11.953   5.511 -13.071  1.00  0.00
ATOM   2059  C9  LIG   120      -5.760   8.584 -20.722  1.00  0.00
ATOM   2060  H17 LIG   120      -5.250   7.616 -20.772  1.00  0.00
ATOM   2061  H18 LIG   120      -5.260   9.171 -19.945  1.00  0.00
ATOM   2062  C10 LIG   120     -13.913   4.708 -13.489  1.00  0.00
ATOM   2063  H19 LIG   120     -14.401   4.123 -14.279  1.00  0.00
ATOM   2064  H20 LIG   120     -14.426   5.678 -13.470  1.00  0.00
ATOM   2065  C11 LIG   120      -5.599   9.291 -22.054  1.00  0.00
ATOM   2066  H21 LIG   120      -6.039   8.717 -22.873  1.00  0.00
ATOM   2067  H22 LIG   120      -6.032  10.295 -22.036  1.00  0.00
ATOM   2068  C12 LIG   120     -14.101   4.005 -12.142  1.00  0.00
ATOM   2069  H23 LIG   120     -13.653   4.584 -11.328  1.00  0.00
ATOM   2070  H24 LIG   120     -15.160   3.868 -11.907  1.00  0.00
ATOM   2071  H25 LIG   120     -13.631   3.017 -12.142  1.00  0.00
TER
ATOM   2072  S1  LIG   121     -25.682 -25.403  -7.810  1.00  0.00
ATOM   2073  O1  LIG   121     -24.792 -24.861  -9.052  1.00  0.00
ATOM   2074  O2  LIG   121     -26.660 -24.123  -7.694  1.00  0.00
ATOM   2075  H26 LIG   121     -26.183 -23.384  -7.288  1.00  0.00
```

```
ATOM   2076  O3  LIG   121     -26.506 -26.509  -8.231  1.00  0.00
ATOM   2077  O4  LIG   121     -24.846 -25.486  -6.629  1.00  0.00
ATOM   2078  C1  LIG   121     -19.515 -25.454 -14.372  1.00  0.00
ATOM   2079  H1  LIG   121     -20.250 -25.142 -15.125  1.00  0.00
ATOM   2080  H2  LIG   121     -19.044 -24.533 -14.006  1.00  0.00
ATOM   2081  C2  LIG   121     -18.450 -26.333 -15.040  1.00  0.00
ATOM   2082  H3  LIG   121     -18.919 -27.254 -15.407  1.00  0.00
ATOM   2083  H4  LIG   121     -17.714 -26.646 -14.287  1.00  0.00
ATOM   2084  C3  LIG   121     -20.240 -26.147 -13.214  1.00  0.00
ATOM   2085  H5  LIG   121     -19.504 -26.457 -12.460  1.00  0.00
ATOM   2086  H6  LIG   121     -20.708 -27.070 -13.580  1.00  0.00
ATOM   2087  C4  LIG   121     -17.722 -25.642 -16.199  1.00  0.00
ATOM   2088  H7  LIG   121     -17.251 -24.720 -15.832  1.00  0.00
ATOM   2089  H8  LIG   121     -18.457 -25.327 -16.951  1.00  0.00
ATOM   2090  C5  LIG   121     -21.307 -25.271 -12.548  1.00  0.00
ATOM   2091  H9  LIG   121     -20.841 -24.346 -12.183  1.00  0.00
ATOM   2092  H10 LIG   121     -22.044 -24.963 -13.299  1.00  0.00
ATOM   2093  C6  LIG   121     -16.658 -26.519 -16.867  1.00  0.00
ATOM   2094  H11 LIG   121     -17.128 -27.441 -17.234  1.00  0.00
ATOM   2095  H12 LIG   121     -15.922 -26.833 -16.115  1.00  0.00
ATOM   2096  C7  LIG   121     -22.028 -25.965 -11.388  1.00  0.00
ATOM   2097  H13 LIG   121     -22.492 -26.890 -11.753  1.00  0.00
```

```
ATOM   2098  H14 LIG   121     -21.290 -26.268 -10.634  1.00  0.00
ATOM   2099  C8  LIG   121     -15.930 -25.829 -18.026  1.00  0.00
ATOM   2100  H15 LIG   121     -16.664 -25.515 -18.780  1.00  0.00
ATOM   2101  H16 LIG   121     -15.460 -24.907 -17.660  1.00  0.00
ATOM   2102  C9  LIG   121     -23.097 -25.080 -10.733  1.00  0.00
ATOM   2103  H17 LIG   121     -23.850 -24.790 -11.474  1.00  0.00
ATOM   2104  H18 LIG   121     -22.642 -24.157 -10.359  1.00  0.00
ATOM   2105  C10 LIG   121     -14.865 -26.706 -18.695  1.00  0.00
ATOM   2106  H19 LIG   121     -15.335 -27.627 -19.060  1.00  0.00
ATOM   2107  H20 LIG   121     -14.131 -27.018 -17.942  1.00  0.00
ATOM   2108  C11 LIG   121     -23.789 -25.789  -9.585  1.00  0.00
ATOM   2109  H21 LIG   121     -24.303 -26.693  -9.919  1.00  0.00
ATOM   2110  H22 LIG   121     -23.090 -26.040  -8.783  1.00  0.00
ATOM   2111  C12 LIG   121     -14.145 -26.007 -19.852  1.00  0.00
ATOM   2112  H23 LIG   121     -13.636 -25.100 -19.511  1.00  0.00
ATOM   2113  H24 LIG   121     -13.392 -26.658 -20.306  1.00  0.00
ATOM   2114  H25 LIG   121     -14.848 -25.715 -20.638  1.00  0.00
TER
ATOM   2115  S1  LIG   122      -5.962 -13.658 -22.200  1.00  0.00
ATOM   2116  O1  LIG   122      -6.680 -13.237 -20.809  1.00  0.00
ATOM   2117  O2  LIG   122      -5.223 -14.984 -21.648  1.00  0.00
ATOM   2118  H26 LIG   122      -4.464 -14.731 -21.102  1.00  0.00
```

```
ATOM   2119  O3  LIG   122      -6.945 -14.106 -23.155  1.00  0.00
ATOM   2120  O4  LIG   122      -4.972 -12.657 -22.543  1.00  0.00
ATOM   2121  C1  LIG   122     -11.253  -9.075 -16.533  1.00  0.00
ATOM   2122  H1  LIG   122     -11.753  -9.990 -16.193  1.00  0.00
ATOM   2123  H2  LIG   122     -10.430  -8.899 -15.828  1.00  0.00
ATOM   2124  C2  LIG   122     -12.238  -7.903 -16.461  1.00  0.00
ATOM   2125  H3  LIG   122     -13.061  -8.079 -17.165  1.00  0.00
ATOM   2126  H4  LIG   122     -11.737  -6.987 -16.803  1.00  0.00
ATOM   2127  C3  LIG   122     -10.677  -9.305 -17.935  1.00  0.00
ATOM   2128  H5  LIG   122     -10.174  -8.390 -18.275  1.00  0.00
ATOM   2129  H6  LIG   122     -11.501  -9.479 -18.640  1.00  0.00
ATOM   2130  C4  LIG   122     -12.814  -7.669 -15.060  1.00  0.00
ATOM   2131  H7  LIG   122     -11.991  -7.492 -14.355  1.00  0.00
ATOM   2132  H8  LIG   122     -13.314  -8.585 -14.718  1.00  0.00
ATOM   2133  C5  LIG   122      -9.694 -10.479 -18.008  1.00  0.00
ATOM   2134  H9  LIG   122      -8.872 -10.308 -17.302  1.00  0.00
ATOM   2135  H10 LIG   122     -10.197 -11.395 -17.672  1.00  0.00
ATOM   2136  C6  LIG   122     -13.800  -6.498 -14.988  1.00  0.00
ATOM   2137  H11 LIG   122     -14.623  -6.675 -15.692  1.00  0.00
ATOM   2138  H12 LIG   122     -13.300  -5.582 -15.330  1.00  0.00
ATOM   2139  C7  LIG   122      -9.119 -10.705 -19.410  1.00  0.00
ATOM   2140  H13 LIG   122      -9.942 -10.874 -20.116  1.00  0.00
```

ATOM 2141 H14 LIG 122 -8.611 -9.791 -19.743 1.00 0.00

ATOM 2142 C8 LIG 122 -14.377 -6.264 -13.587 1.00 0.00

ATOM 2143 H15 LIG 122 -14.878 -7.179 -13.244 1.00 0.00

ATOM 2144 H16 LIG 122 -13.554 -6.088 -12.882 1.00 0.00

ATOM 2145 C9 LIG 122 -8.141 -11.886 -19.471 1.00 0.00

ATOM 2146 H17 LIG 122 -8.645 -12.808 -19.161 1.00 0.00

ATOM 2147 H18 LIG 122 -7.312 -11.723 -18.775 1.00 0.00

ATOM 2148 C10 LIG 122 -15.363 -5.093 -13.513 1.00 0.00

ATOM 2149 H19 LIG 122 -16.184 -5.269 -14.218 1.00 0.00

ATOM 2150 H20 LIG 122 -14.862 -4.179 -13.855 1.00 0.00

ATOM 2151 C11 LIG 122 -7.582 -12.082 -20.867 1.00 0.00

ATOM 2152 H21 LIG 122 -8.370 -12.300 -21.592 1.00 0.00

ATOM 2153 H22 LIG 122 -7.008 -11.213 -21.200 1.00 0.00

ATOM 2154 C12 LIG 122 -15.934 -4.869 -12.110 1.00 0.00

ATOM 2155 H23 LIG 122 -15.138 -4.656 -11.389 1.00 0.00

ATOM 2156 H24 LIG 122 -16.631 -4.026 -12.092 1.00 0.00

ATOM 2157 H25 LIG 122 -16.472 -5.753 -11.756 1.00 0.00

TER

ATOM 2158 S1 LIG 123 3.262 -15.010 -17.416 1.00 0.00

ATOM 2159 O1 LIG 123 4.610 -15.903 -17.530 1.00 0.00

ATOM 2160 O2 LIG 123 3.616 -13.967 -18.597 1.00 0.00

ATOM 2161 H26 LIG 123 4.292 -13.344 -18.292 1.00 0.00

ATOM	2162	O3	LIG	123	2.121	-15.766	-17.867	1.00	0.00
ATOM	2163	O4	LIG	123	3.258	-14.319	-16.142	1.00	0.00
ATOM	2164	C1	LIG	123	9.051	-21.716	-15.797	1.00	0.00
ATOM	2165	H1	LIG	123	9.131	-21.962	-16.863	1.00	0.00
ATOM	2166	H2	LIG	123	9.873	-21.021	-15.583	1.00	0.00
ATOM	2167	C2	LIG	123	9.240	-22.991	-14.967	1.00	0.00
ATOM	2168	H3	LIG	123	8.418	-23.687	-15.181	1.00	0.00
ATOM	2169	H4	LIG	123	9.160	-22.746	-13.901	1.00	0.00
ATOM	2170	C3	LIG	123	7.714	-21.012	-15.539	1.00	0.00
ATOM	2171	H5	LIG	123	7.636	-20.763	-14.472	1.00	0.00
ATOM	2172	H6	LIG	123	6.892	-21.708	-15.750	1.00	0.00
ATOM	2173	C4	LIG	123	10.577	-23.697	-15.223	1.00	0.00
ATOM	2174	H7	LIG	123	11.400	-23.002	-15.008	1.00	0.00
ATOM	2175	H8	LIG	123	10.658	-23.943	-16.289	1.00	0.00
ATOM	2176	C5	LIG	123	7.523	-19.738	-16.370	1.00	0.00
ATOM	2177	H9	LIG	123	8.346	-19.042	-16.161	1.00	0.00
ATOM	2178	H10	LIG	123	7.598	-19.986	-17.436	1.00	0.00
ATOM	2179	C6	LIG	123	10.766	-24.973	-14.395	1.00	0.00
ATOM	2180	H11	LIG	123	9.944	-25.668	-14.608	1.00	0.00
ATOM	2181	H12	LIG	123	10.685	-24.728	-13.327	1.00	0.00
ATOM	2182	C7	LIG	123	6.187	-19.035	-16.107	1.00	0.00
ATOM	2183	H13	LIG	123	5.365	-19.732	-16.314	1.00	0.00

ATOM 2184 H14 LIG 123 6.115 -18.782 -15.042 1.00 0.00

ATOM 2185 C8 LIG 123 12.102 -25.680 -14.649 1.00 0.00

ATOM 2186 H15 LIG 123 12.185 -25.927 -15.716 1.00 0.00

ATOM 2187 H16 LIG 123 12.926 -24.985 -14.436 1.00 0.00

ATOM 2188 C9 LIG 123 6.005 -17.764 -16.949 1.00 0.00

ATOM 2189 H17 LIG 123 6.054 -18.010 -18.015 1.00 0.00

ATOM 2190 H18 LIG 123 6.818 -17.059 -16.744 1.00 0.00

ATOM 2191 C10 LIG 123 12.293 -26.956 -13.822 1.00 0.00

ATOM 2192 H19 LIG 123 11.471 -27.649 -14.035 1.00 0.00

ATOM 2193 H20 LIG 123 12.213 -26.709 -12.756 1.00 0.00

ATOM 2194 C11 LIG 123 4.680 -17.086 -16.664 1.00 0.00

ATOM 2195 H21 LIG 123 3.833 -17.734 -16.903 1.00 0.00

ATOM 2196 H22 LIG 123 4.607 -16.757 -15.624 1.00 0.00

ATOM 2197 C12 LIG 123 13.631 -27.654 -14.085 1.00 0.00

ATOM 2198 H23 LIG 123 14.473 -26.996 -13.848 1.00 0.00

ATOM 2199 H24 LIG 123 13.736 -28.558 -13.478 1.00 0.00

ATOM 2200 H25 LIG 123 13.725 -27.946 -15.135 1.00 0.00

TER

ATOM 2201 S1 LIG 124 -17.692 10.307 -26.344 1.00 0.00

ATOM 2202 O1 LIG 124 -17.618 10.143 -24.733 1.00 0.00

ATOM 2203 O2 LIG 124 -16.258 9.644 -26.676 1.00 0.00

ATOM 2204 H26 LIG 124 -15.550 10.265 -26.447 1.00 0.00

```
ATOM   2205  O3  LIG   124     -18.688   9.420 -26.891  1.00  0.00
ATOM   2206  O4  LIG   124     -17.666  11.717 -26.678  1.00  0.00
ATOM   2207  C1  LIG   124     -20.364  10.556 -17.747  1.00  0.00
ATOM   2208  H1  LIG   124     -20.095   9.504 -17.588  1.00  0.00
ATOM   2209  H2  LIG   124     -19.490  11.144 -17.440  1.00  0.00
ATOM   2210  C2  LIG   124     -21.554  10.915 -16.848  1.00  0.00
ATOM   2211  H3  LIG   124     -22.429  10.327 -17.155  1.00  0.00
ATOM   2212  H4  LIG   124     -21.824  11.967 -17.008  1.00  0.00
ATOM   2213  C3  LIG   124     -20.632  10.789 -19.238  1.00  0.00
ATOM   2214  H5  LIG   124     -20.900  11.842 -19.398  1.00  0.00
ATOM   2215  H6  LIG   124     -21.509  10.203 -19.544  1.00  0.00
ATOM   2216  C4  LIG   124     -21.288  10.683 -15.356  1.00  0.00
ATOM   2217  H7  LIG   124     -20.413  11.272 -15.049  1.00  0.00
ATOM   2218  H8  LIG   124     -21.016   9.632 -15.196  1.00  0.00
ATOM   2219  C5  LIG   124     -19.445  10.428 -20.137  1.00  0.00
ATOM   2220  H9  LIG   124     -18.567  11.011 -19.831  1.00  0.00
ATOM   2221  H10 LIG   124     -19.179   9.375 -19.981  1.00  0.00
ATOM   2222  C6  LIG   124     -22.477  11.041 -14.457  1.00  0.00
ATOM   2223  H11 LIG   124     -23.351  10.453 -14.764  1.00  0.00
ATOM   2224  H12 LIG   124     -22.748  12.093 -14.617  1.00  0.00
ATOM   2225  C7  LIG   124     -19.715  10.665 -21.627  1.00  0.00
ATOM   2226  H13 LIG   124     -20.594  10.082 -21.932  1.00  0.00
```

```
ATOM   2227  H14 LIG   124     -19.976  11.719 -21.783  1.00  0.00
ATOM   2228  C8  LIG   124     -22.212  10.810 -12.966  1.00  0.00
ATOM   2229  H15 LIG   124     -21.941   9.758 -12.804  1.00  0.00
ATOM   2230  H16 LIG   124     -21.337  11.398 -12.658  1.00  0.00
ATOM   2231  C9  LIG   124     -18.521  10.294 -22.517  1.00  0.00
ATOM   2232  H17 LIG   124     -18.267   9.237 -22.384  1.00  0.00
ATOM   2233  H18 LIG   124     -17.641  10.876 -22.225  1.00  0.00
ATOM   2234  C10 LIG   124     -23.400  11.167 -12.065  1.00  0.00
ATOM   2235  H19 LIG   124     -24.274  10.580 -12.373  1.00  0.00
ATOM   2236  H20 LIG   124     -23.670  12.219 -12.226  1.00  0.00
ATOM   2237  C11 LIG   124     -18.813  10.545 -23.984  1.00  0.00
ATOM   2238  H21 LIG   124     -19.655   9.946 -24.337  1.00  0.00
ATOM   2239  H22 LIG   124     -19.007  11.602 -24.184  1.00  0.00
ATOM   2240  C12 LIG   124     -23.125  10.932 -10.577  1.00  0.00
ATOM   2241  H23 LIG   124     -22.277  11.532 -10.233  1.00  0.00
ATOM   2242  H24 LIG   124     -23.991  11.198  -9.964  1.00  0.00
ATOM   2243  H25 LIG   124     -22.888   9.882 -10.380  1.00  0.00
TER
EMD
```

Table H: The number of atoms present in the system of BE-Sum

```
ATOM      1  N   GLU     1       9.101  17.697  -4.770  1.00  0.00
```

```
ATOM      2  H1  GLU     1       9.411  18.312  -5.508  1.00  0.00
ATOM      3  H2  GLU     1       9.508  16.784  -4.916  1.00  0.00
ATOM      4  H3  GLU     1       9.221  18.167  -3.884  1.00  0.00
ATOM      5  CA  GLU     1       7.631  17.501  -4.916  1.00  0.00
ATOM      6  HA  GLU     1       7.402  16.444  -5.056  1.00  0.00
ATOM      7  CB  GLU     1       6.924  17.991  -3.649  1.00  0.00
ATOM      8  HB2 GLU     1       7.345  17.485  -2.780  1.00  0.00
ATOM      9  HB3 GLU     1       7.067  19.066  -3.547  1.00  0.00
ATOM     10  CG  GLU     1       5.426  17.682  -3.744  1.00  0.00
ATOM     11  HG2 GLU     1       5.029  18.126  -4.656  1.00  0.00
ATOM     12  HG3 GLU     1       5.282  16.602  -3.773  1.00  0.00
ATOM     13  CD  GLU     1       4.694  18.260  -2.537  1.00  0.00
ATOM     14  OE1 GLU     1       4.823  17.694  -1.463  1.00  0.00
ATOM     15  OE2 GLU     1       4.012  19.258  -2.704  1.00  0.00
ATOM     16  C   GLU     1       7.135  18.282  -6.131  1.00  0.00
ATOM     17  O   GLU     1       6.990  19.504  -6.078  1.00  0.00
ATOM     18  N   ALA     2       6.875  17.565  -7.222  1.00  0.00
ATOM     19  H   ALA     2       7.027  16.566  -7.224  1.00  0.00
ATOM     20  CA  ALA     2       6.391  18.195  -8.447  1.00  0.00
ATOM     21  HA  ALA     2       7.047  19.018  -8.730  1.00  0.00
ATOM     22  CB  ALA     2       6.367  17.175  -9.587  1.00  0.00
ATOM     23  HB1 ALA     2       5.707  16.349  -9.322  1.00  0.00
```

ATOM 24 HB2 ALA 2 6.003 17.653 -10.496 1.00 0.00
ATOM 25 HB3 ALA 2 7.375 16.794 -9.756 1.00 0.00
ATOM 26 C ALA 2 4.990 18.763 -8.242 1.00 0.00
ATOM 27 O ALA 2 4.197 18.220 -7.474 1.00 0.00
ATOM 28 N GLU 3 4.695 19.859 -8.934 1.00 0.00
ATOM 29 H GLU 3 5.388 20.286 -9.531 1.00 0.00
ATOM 30 CA GLU 3 3.386 20.497 -8.826 1.00 0.00
ATOM 31 HA GLU 3 3.175 20.740 -7.784 1.00 0.00
ATOM 32 CB GLU 3 3.361 21.795 -9.648 1.00 0.00
ATOM 33 HB2 GLU 3 2.466 22.361 -9.390 1.00 0.00
ATOM 34 HB3 GLU 3 4.246 22.385 -9.407 1.00 0.00
ATOM 35 CG GLU 3 3.351 21.478 -11.151 1.00 0.00
ATOM 36 HG2 GLU 3 2.470 20.880 -11.383 1.00 0.00
ATOM 37 HG3 GLU 3 3.312 22.413 -11.709 1.00 0.00
ATOM 38 CD GLU 3 4.609 20.703 -11.543 1.00 0.00
ATOM 39 OE1 GLU 3 5.694 21.208 -11.305 1.00 0.00
ATOM 40 OE2 GLU 3 4.468 19.613 -12.075 1.00 0.00
ATOM 41 C GLU 3 2.283 19.556 -9.309 1.00 0.00
ATOM 42 O GLU 3 1.125 19.687 -8.910 1.00 0.00
ATOM 43 N ALA 4 2.653 18.612 -10.172 1.00 0.00
ATOM 44 H ALA 4 3.609 18.564 -10.495 1.00 0.00
ATOM 45 CA ALA 4 1.691 17.653 -10.710 1.00 0.00

ATOM	46	HA	ALA	4	0.794	18.182	-11.030	1.00	0.00
ATOM	47	CB	ALA	4	2.288	16.928	-11.918	1.00	0.00
ATOM	48	HB1	ALA	4	3.183	16.386	-11.611	1.00	0.00
ATOM	49	HB2	ALA	4	1.557	16.225	-12.318	1.00	0.00
ATOM	50	HB3	ALA	4	2.550	17.655	-12.686	1.00	0.00
ATOM	51	C	ALA	4	1.277	16.643	-9.651	1.00	0.00
ATOM	52	O	ALA	4	0.425	15.787	-9.894	1.00	0.00
ATOM	53	N	GLN	5	1.876	16.758	-8.475	1.00	0.00
ATOM	54	H	GLN	5	2.570	17.477	-8.328	1.00	0.00
ATOM	55	CA	GLN	5	1.562	15.866	-7.369	1.00	0.00
ATOM	56	HA	GLN	5	1.929	14.859	-7.572	1.00	0.00
ATOM	57	CB	GLN	5	2.241	16.387	-6.107	1.00	0.00
ATOM	58	HB2	GLN	5	3.317	16.441	-6.274	1.00	0.00
ATOM	59	HB3	GLN	5	1.859	17.382	-5.878	1.00	0.00
ATOM	60	CG	GLN	5	1.954	15.447	-4.931	1.00	0.00
ATOM	61	HG2	GLN	5	0.882	15.271	-4.846	1.00	0.00
ATOM	62	HG3	GLN	5	2.467	14.497	-5.084	1.00	0.00
ATOM	63	CD	GLN	5	2.451	16.065	-3.628	1.00	0.00
ATOM	64	OE1	GLN	5	2.063	17.181	-3.281	1.00	0.00
ATOM	65	NE2	GLN	5	3.289	15.402	-2.880	1.00	0.00
ATOM	66	HE21	GLN	5	3.621	15.807	-2.016	1.00	0.00
ATOM	67	HE22	GLN	5	3.603	14.486	-3.168	1.00	0.00

ATOM	68	C	GLN	5	0.053	15.807	-7.144	1.00	0.00
ATOM	69	O	GLN	5	-0.497	14.754	-6.822	1.00	0.00
ATOM	70	N	GLU	6	-0.606	16.952	-7.311	1.00	0.00
ATOM	71	H	GLU	6	-0.104	17.796	-7.546	1.00	0.00
ATOM	72	CA	GLU	6	-2.050	17.035	-7.121	1.00	0.00
ATOM	73	HA	GLU	6	-2.304	16.843	-6.078	1.00	0.00
ATOM	74	CB	GLU	6	-2.540	18.439	-7.495	1.00	0.00
ATOM	75	HB2	GLU	6	-3.606	18.515	-7.284	1.00	0.00
ATOM	76	HB3	GLU	6	-1.999	19.178	-6.903	1.00	0.00
ATOM	77	CG	GLU	6	-2.294	18.699	-8.986	1.00	0.00
ATOM	78	HG2	GLU	6	-1.260	18.451	-9.227	1.00	0.00
ATOM	79	HG3	GLU	6	-2.964	18.073	-9.575	1.00	0.00
ATOM	80	CD	GLU	6	-2.554	20.168	-9.309	1.00	0.00
ATOM	81	OE1	GLU	6	-3.713	20.546	-9.365	1.00	0.00
ATOM	82	OE2	GLU	6	-1.590	20.892	-9.496	1.00	0.00
ATOM	83	C	GLU	6	-2.778	15.985	-7.960	1.00	0.00
ATOM	84	O	GLU	6	-3.694	15.323	-7.473	1.00	0.00
ATOM	85	N	GLU	7	-2.356	15.824	-9.215	1.00	0.00
ATOM	86	H	GLU	7	-1.608	16.395	-9.583	1.00	0.00
ATOM	87	CA	GLU	7	-2.968	14.836	-10.095	1.00	0.00
ATOM	88	HA	GLU	7	-4.018	15.081	-10.253	1.00	0.00
ATOM	89	CB	GLU	7	-2.258	14.820	-11.455	1.00	0.00

ATOM	90	HB2	GLU	7	-2.455	15.756	-11.976	1.00	0.00
ATOM	91	HB3	GLU	7	-1.184	14.709	-11.301	1.00	0.00
ATOM	92	CG	GLU	7	-2.777	13.646	-12.298	1.00	0.00
ATOM	93	HG2	GLU	7	-3.793	13.406	-11.983	1.00	0.00
ATOM	94	HG3	GLU	7	-2.780	13.931	-13.350	1.00	0.00
ATOM	95	CD	GLU	7	-1.883	12.423	-12.105	1.00	0.00
ATOM	96	OE1	GLU	7	-0.713	12.508	-12.441	1.00	0.00
ATOM	97	OE2	GLU	7	-2.382	11.417	-11.628	1.00	0.00
ATOM	98	C	GLU	7	-2.878	13.461	-9.459	1.00	0.00
ATOM	99	O	GLU	7	-3.818	12.668	-9.520	1.00	0.00
ATOM	100	N	CYX	8	-1.725	13.188	-8.857	1.00	0.00
ATOM	101	H	CYX	8	-0.975	13.865	-8.849	1.00	0.00
ATOM	102	CA	CYX	8	-1.499	11.907	-8.221	1.00	0.00
ATOM	103	HA	CYX	8	-1.756	11.099	-8.906	1.00	0.00
ATOM	104	CB	CYX	8	-0.024	11.768	-7.832	1.00	0.00
ATOM	105	HB2	CYX	8	0.227	12.518	-7.082	1.00	0.00
ATOM	106	HB3	CYX	8	0.153	10.773	-7.423	1.00	0.00
ATOM	107	SG	CYX	8	1.013	12.007	-9.295	1.00	0.00
ATOM	108	C	CYX	8	-2.369	11.758	-6.980	1.00	0.00
ATOM	109	O	CYX	8	-3.156	10.818	-6.878	1.00	0.00
ATOM	110	N	ARG	9	-2.220	12.692	-6.044	1.00	0.00
ATOM	111	H	ARG	9	-1.545	13.433	-6.168	1.00	0.00

```
ATOM    112  CA   ARG     9      -2.993  12.659  -4.806  1.00  0.00
ATOM    113  HA   ARG     9      -2.727  11.778  -4.221  1.00  0.00
ATOM    114  CB   ARG     9      -2.692  13.908  -3.974  1.00  0.00
ATOM    115  HB2 ARG     9      -1.616  13.988  -3.818  1.00  0.00
ATOM    116  HB3 ARG     9      -3.048  14.790  -4.504  1.00  0.00
ATOM    117  CG   ARG     9      -3.398  13.808  -2.617  1.00  0.00
ATOM    118  HG2 ARG     9      -4.477  13.781  -2.770  1.00  0.00
ATOM    119  HG3 ARG     9      -3.081  12.897  -2.109  1.00  0.00
ATOM    120  CD   ARG     9      -3.035  15.022  -1.760  1.00  0.00
ATOM    121  HD2 ARG     9      -3.415  14.888  -0.747  1.00  0.00
ATOM    122  HD3 ARG     9      -1.952  15.143  -1.728  1.00  0.00
ATOM    123  NE   ARG     9      -3.625  16.233  -2.325  1.00  0.00
ATOM    124  HE   ARG     9      -4.299  16.166  -3.074  1.00  0.00
ATOM    125  CZ   ARG     9      -3.292  17.442  -1.875  1.00  0.00
ATOM    126  NH1 ARG     9      -2.426  17.567  -0.906  1.00  0.00
ATOM    127 HH11 ARG     9      -2.008  16.743  -0.497  1.00  0.00
ATOM    128 HH12 ARG     9      -2.178  18.486  -0.570  1.00  0.00
ATOM    129  NH2 ARG     9      -3.835  18.504  -2.402  1.00  0.00
ATOM    130 HH21 ARG     9      -4.506  18.405  -3.150  1.00  0.00
ATOM    131 HH22 ARG     9      -3.581  19.419  -2.059  1.00  0.00
ATOM    132  C    ARG     9      -4.488  12.582  -5.098  1.00  0.00
ATOM    133  O    ARG     9      -5.211  11.814  -4.466  1.00  0.00
```

ATOM	134	N	GLU	10	-4.946	13.380	-6.059	1.00	0.00
ATOM	135	H	GLU	10	-4.323	14.010	-6.544	1.00	0.00
ATOM	136	CA	GLU	10	-6.359	13.386	-6.424	1.00	0.00
ATOM	137	HA	GLU	10	-6.968	13.659	-5.562	1.00	0.00
ATOM	138	CB	GLU	10	-6.602	14.408	-7.539	1.00	0.00
ATOM	139	HB2	GLU	10	-6.342	15.403	-7.179	1.00	0.00
ATOM	140	HB3	GLU	10	-5.979	14.159	-8.398	1.00	0.00
ATOM	141	CG	GLU	10	-8.079	14.383	-7.953	1.00	0.00
ATOM	142	HG2	GLU	10	-8.686	14.089	-7.097	1.00	0.00
ATOM	143	HG3	GLU	10	-8.373	15.379	-8.282	1.00	0.00
ATOM	144	CD	GLU	10	-8.290	13.387	-9.093	1.00	0.00
ATOM	145	OE1	GLU	10	-7.640	13.535	-10.115	1.00	0.00
ATOM	146	OE2	GLU	10	-9.099	12.488	-8.926	1.00	0.00
ATOM	147	C	GLU	10	-6.796	12.002	-6.894	1.00	0.00
ATOM	148	O	GLU	10	-7.821	11.478	-6.454	1.00	0.00
ATOM	149	N	GLN	11	-6.009	11.423	-7.797	1.00	0.00
ATOM	150	H	GLN	11	-5.198	11.912	-8.147	1.00	0.00
ATOM	151	CA	GLN	11	-6.309	10.103	-8.341	1.00	0.00
ATOM	152	HA	GLN	11	-7.287	10.108	-8.822	1.00	0.00
ATOM	153	CB	GLN	11	-5.252	9.719	-9.383	1.00	0.00
ATOM	154	HB2	GLN	11	-5.110	10.546	-10.078	1.00	0.00
ATOM	155	HB3	GLN	11	-4.309	9.501	-8.880	1.00	0.00

ATOM	156	CG	GLN	11	-5.718	8.479	-10.154	1.00	0.00
ATOM	157	HG2	GLN	11	-5.794	7.628	-9.476	1.00	0.00
ATOM	158	HG3	GLN	11	-6.690	8.669	-10.608	1.00	0.00
ATOM	159	CD	GLN	11	-4.721	8.134	-11.260	1.00	0.00
ATOM	160	OE1	GLN	11	-3.787	8.894	-11.522	1.00	0.00
ATOM	161	NE2	GLN	11	-4.866	7.023	-11.929	1.00	0.00
ATOM	162	HE21	GLN	11	-4.211	6.788	-12.661	1.00	0.00
ATOM	163	HE22	GLN	11	-5.632	6.402	-11.711	1.00	0.00
ATOM	164	C	GLN	11	-6.368	9.043	-7.240	1.00	0.00
ATOM	165	O	GLN	11	-7.143	8.091	-7.332	1.00	0.00
ATOM	166	N	MET	12	-5.537	9.201	-6.208	1.00	0.00
ATOM	167	H	MET	12	-4.893	9.979	-6.182	1.00	0.00
ATOM	168	CA	MET	12	-5.501	8.233	-5.109	1.00	0.00
ATOM	169	HA	MET	12	-5.119	7.277	-5.469	1.00	0.00
ATOM	170	CB	MET	12	-4.587	8.733	-3.988	1.00	0.00
ATOM	171	HB2	MET	12	-4.874	9.746	-3.707	1.00	0.00
ATOM	172	HB3	MET	12	-4.678	8.077	-3.122	1.00	0.00
ATOM	173	CG	MET	12	-3.139	8.731	-4.478	1.00	0.00
ATOM	174	HG2	MET	12	-2.854	7.721	-4.774	1.00	0.00
ATOM	175	HG3	MET	12	-3.040	9.401	-5.332	1.00	0.00
ATOM	176	SD	MET	12	-2.046	9.292	-3.153	1.00	0.00
ATOM	177	CE	MET	12	-0.598	9.617	-4.187	1.00	0.00

ATOM 178 HE1 MET 12 -0.847 10.369 -4.935 1.00 0.00
ATOM 179 HE2 MET 12 0.220 9.981 -3.564 1.00 0.00
ATOM 180 HE3 MET 12 -0.292 8.696 -4.685 1.00 0.00
ATOM 181 C MET 12 -6.898 7.970 -4.552 1.00 0.00
ATOM 182 O MET 12 -7.239 6.826 -4.253 1.00 0.00
ATOM 183 N GLN 13 -7.708 9.021 -4.421 1.00 0.00
ATOM 184 H GLN 13 -7.400 9.953 -4.657 1.00 0.00
ATOM 185 CA GLN 13 -9.065 8.848 -3.908 1.00 0.00
ATOM 186 HA GLN 13 -9.025 8.442 -2.897 1.00 0.00
ATOM 187 CB GLN 13 -9.807 10.188 -3.869 1.00 0.00
ATOM 188 HB2 GLN 13 -9.639 10.718 -4.806 1.00 0.00
ATOM 189 HB3 GLN 13 -10.874 10.004 -3.742 1.00 0.00
ATOM 190 CG GLN 13 -9.294 11.038 -2.702 1.00 0.00
ATOM 191 HG2 GLN 13 -9.924 11.919 -2.586 1.00 0.00
ATOM 192 HG3 GLN 13 -9.312 10.455 -1.781 1.00 0.00
ATOM 193 CD GLN 13 -7.862 11.494 -2.962 1.00 0.00
ATOM 194 OE1 GLN 13 -6.912 10.820 -2.564 1.00 0.00
ATOM 195 NE2 GLN 13 -7.650 12.608 -3.607 1.00 0.00
ATOM 196 HE21 GLN 13 -6.703 12.914 -3.782 1.00 0.00
ATOM 197 HE22 GLN 13 -8.433 13.158 -3.928 1.00 0.00
ATOM 198 C GLN 13 -9.825 7.873 -4.794 1.00 0.00
ATOM 199 O GLN 13 -10.495 6.967 -4.303 1.00 0.00

ATOM	200	N	ARG	14	-9.679	8.048	-6.105	1.00	0.00
ATOM	201	H	ARG	14	-9.122	8.816	-6.452	1.00	0.00
ATOM	202	CA	ARG	14	-10.310	7.168	-7.066	1.00	0.00
ATOM	203	HA	ARG	14	-11.393	7.204	-6.947	1.00	0.00
ATOM	204	CB	ARG	14	-9.959	7.601	-8.494	1.00	0.00
ATOM	205	HB2	ARG	14	-10.132	8.672	-8.597	1.00	0.00
ATOM	206	HB3	ARG	14	-8.908	7.383	-8.687	1.00	0.00
ATOM	207	CG	ARG	14	-10.831	6.843	-9.501	1.00	0.00
ATOM	208	HG2	ARG	14	-10.358	6.871	-10.483	1.00	0.00
ATOM	209	HG3	ARG	14	-10.938	5.807	-9.179	1.00	0.00
ATOM	210	CD	ARG	14	-12.212	7.497	-9.581	1.00	0.00
ATOM	211	HD2	ARG	14	-12.868	6.901	-10.216	1.00	0.00
ATOM	212	HD3	ARG	14	-12.644	7.573	-8.583	1.00	0.00
ATOM	213	NE	ARG	14	-12.103	8.839	-10.148	1.00	0.00
ATOM	214	HE	ARG	14	-11.308	9.082	-10.721	1.00	0.00
ATOM	215	CZ	ARG	14	-13.035	9.765	-9.928	1.00	0.00
ATOM	216	NH1	ARG	14	-14.080	9.487	-9.196	1.00	0.00
ATOM	217	HH11	ARG	14	-14.181	8.565	-8.795	1.00	0.00
ATOM	218	HH12	ARG	14	-14.782	10.194	-9.034	1.00	0.00
ATOM	219	NH2	ARG	14	-12.903	10.956	-10.446	1.00	0.00
ATOM	220	HH21	ARG	14	-12.094	11.170	-11.011	1.00	0.00
ATOM	221	HH22	ARG	14	-13.610	11.657	-10.278	1.00	0.00

ATOM	222	C	ARG	14	-9.815	5.762	-6.810	1.00	0.00
ATOM	223	O	ARG	14	-10.585	4.801	-6.817	1.00	0.00
ATOM	224	N	GLN	15	-8.509	5.664	-6.560	1.00	0.00
ATOM	225	H	GLN	15	-7.915	6.480	-6.585	1.00	0.00
ATOM	226	CA	GLN	15	-7.904	4.388	-6.273	1.00	0.00
ATOM	227	HA	GLN	15	-8.287	3.631	-6.957	1.00	0.00
ATOM	228	CB	GLN	15	-6.381	4.475	-6.442	1.00	0.00
ATOM	229	HB2	GLN	15	-6.071	5.519	-6.395	1.00	0.00
ATOM	230	HB3	GLN	15	-5.896	3.916	-5.641	1.00	0.00
ATOM	231	CG	GLN	15	-5.976	3.883	-7.796	1.00	0.00
ATOM	232	HG2	GLN	15	-4.941	3.542	-7.755	1.00	0.00
ATOM	233	HG3	GLN	15	-6.624	3.044	-8.050	1.00	0.00
ATOM	234	CD	GLN	15	-6.098	4.941	-8.888	1.00	0.00
ATOM	235	OE1	GLN	15	-5.091	5.467	-9.359	1.00	0.00
ATOM	236	NE2	GLN	15	-7.281	5.290	-9.317	1.00	0.00
ATOM	237	HE21	GLN	15	-7.366	5.990	-10.040	1.00	0.00
ATOM	238	HE22	GLN	15	-8.105	4.858	-8.924	1.00	0.00
ATOM	239	C	GLN	15	-8.256	3.982	-4.851	1.00	0.00
ATOM	240	O	GLN	15	-7.434	4.066	-3.937	1.00	0.00
ATOM	241	N	GLN	16	-9.487	3.536	-4.686	1.00	0.00
ATOM	242	H	GLN	16	-10.125	3.514	-5.468	1.00	0.00
ATOM	243	CA	GLN	16	-9.976	3.100	-3.384	1.00	0.00

ATOM 244 HA GLN 16 -9.565 3.732 -2.596 1.00 0.00
ATOM 245 CB GLN 16 -11.501 3.202 -3.346 1.00 0.00
ATOM 246 HB2 GLN 16 -11.933 2.582 -4.132 1.00 0.00
ATOM 247 HB3 GLN 16 -11.870 2.867 -2.376 1.00 0.00
ATOM 248 CG GLN 16 -11.903 4.661 -3.568 1.00 0.00
ATOM 249 HG2 GLN 16 -11.473 5.279 -2.779 1.00 0.00
ATOM 250 HG3 GLN 16 -11.534 4.999 -4.536 1.00 0.00
ATOM 251 CD GLN 16 -13.420 4.822 -3.541 1.00 0.00
ATOM 252 OE1 GLN 16 -14.156 3.834 -3.525 1.00 0.00
ATOM 253 NE2 GLN 16 -13.930 6.023 -3.538 1.00 0.00
ATOM 254 HE21 GLN 16 -14.932 6.142 -3.520 1.00 0.00
ATOM 255 HE22 GLN 16 -13.319 6.827 -3.553 1.00 0.00
ATOM 256 C GLN 16 -9.526 1.668 -3.123 1.00 0.00
ATOM 257 O GLN 16 -10.296 0.834 -2.638 1.00 0.00
ATOM 258 N MET 17 -8.271 1.393 -3.471 1.00 0.00
ATOM 259 H MET 17 -7.696 2.106 -3.897 1.00 0.00
ATOM 260 CA MET 17 -7.705 0.060 -3.300 1.00 0.00
ATOM 261 HA MET 17 -8.343 -0.681 -3.782 1.00 0.00
ATOM 262 CB MET 17 -6.310 -0.006 -3.936 1.00 0.00
ATOM 263 HB2 MET 17 -6.154 -1.005 -4.344 1.00 0.00
ATOM 264 HB3 MET 17 -6.253 0.727 -4.740 1.00 0.00
ATOM 265 CG MET 17 -5.229 0.293 -2.896 1.00 0.00

ATOM	266	HG2	MET	17	-5.559	1.094	-2.235	1.00	0.00
ATOM	267	HG3	MET	17	-5.022	-0.601	-2.307	1.00	0.00
ATOM	268	SD	MET	17	-3.720	0.807	-3.744	1.00	0.00
ATOM	269	CE	MET	17	-4.350	2.382	-4.372	1.00	0.00
ATOM	270	HE1	MET	17	-4.653	3.012	-3.536	1.00	0.00
ATOM	271	HE2	MET	17	-3.568	2.885	-4.941	1.00	0.00
ATOM	272	HE3	MET	17	-5.208	2.199	-5.018	1.00	0.00
ATOM	273	C	MET	17	-7.623	-0.331	-1.833	1.00	0.00
ATOM	274	O	MET	17	-7.622	-1.513	-1.505	1.00	0.00
ATOM	275	N	LEU	18	-7.541	0.670	-0.965	1.00	0.00
ATOM	276	H	LEU	18	-7.526	1.624	-1.296	1.00	0.00
ATOM	277	CA	LEU	18	-7.430	0.435	0.472	1.00	0.00
ATOM	278	HA	LEU	18	-6.532	-0.146	0.685	1.00	0.00
ATOM	279	CB	LEU	18	-7.342	1.757	1.218	1.00	0.00
ATOM	280	HB2	LEU	18	-8.263	2.318	1.062	1.00	0.00
ATOM	281	HB3	LEU	18	-7.208	1.568	2.283	1.00	0.00
ATOM	282	CG	LEU	18	-6.155	2.569	0.691	1.00	0.00
ATOM	283	HG	LEU	18	-5.299	1.909	0.548	1.00	0.00
ATOM	284	CD1	LEU	18	-6.528	3.218	-0.646	1.00	0.00
ATOM	285	HD11	LEU	18	-7.383	3.878	-0.503	1.00	0.00
ATOM	286	HD12	LEU	18	-5.682	3.795	-1.019	1.00	0.00
ATOM	287	HD13	LEU	18	-6.784	2.442	-1.368	1.00	0.00

```
ATOM    288  CD2 LEU    18      -5.788   3.657   1.705  1.00  0.00
ATOM    289 HD21 LEU    18      -5.517   3.193   2.654  1.00  0.00
ATOM    290 HD22 LEU    18      -4.943   4.234   1.330  1.00  0.00
ATOM    291 HD23 LEU    18      -6.641   4.318   1.855  1.00  0.00
ATOM    292  C   LEU    18      -8.616  -0.367   0.990  1.00  0.00
ATOM    293  O   LEU    18      -8.509  -1.030   2.013  1.00  0.00
ATOM    294  N   SER    19      -9.745  -0.317   0.287  1.00  0.00
ATOM    295  H   SER    19      -9.823   0.252  -0.544  1.00  0.00
ATOM    296  CA  SER    19     -10.912  -1.081   0.715  1.00  0.00
ATOM    297  HA  SER    19     -11.151  -0.845   1.752  1.00  0.00
ATOM    298  CB  SER    19     -12.120  -0.733  -0.158  1.00  0.00
ATOM    299  HB2 SER    19     -11.947  -1.079  -1.177  1.00  0.00
ATOM    300  HB3 SER    19     -13.012  -1.216   0.242  1.00  0.00
ATOM    301  OG  SER    19     -12.305   0.677  -0.161  1.00  0.00
ATOM    302  HG  SER    19     -13.062   0.899  -0.708  1.00  0.00
ATOM    303  C   SER    19     -10.603  -2.573   0.601  1.00  0.00
ATOM    304  O   SER    19     -10.717  -3.331   1.572  1.00  0.00
ATOM    305  N   HIE    20     -10.170  -2.973  -0.593  1.00  0.00
ATOM    306  H   HIE    20     -10.095  -2.309  -1.350  1.00  0.00
ATOM    307  CA  HIE    20      -9.803  -4.361  -0.851  1.00  0.00
ATOM    308  HA  HIE    20     -10.579  -5.027  -0.474  1.00  0.00
ATOM    309  CB  HIE    20      -9.644  -4.598  -2.360  1.00  0.00
```

ATOM 310 HB2 HIE 20 -8.607 -4.562 -2.693 1.00 0.00
ATOM 311 HB3 HIE 20 -10.086 -5.550 -2.653 1.00 0.00
ATOM 312 CG HIE 20 -10.379 -3.528 -3.122 1.00 0.00
ATOM 313 ND1 HIE 20 -9.726 -2.639 -3.960 1.00 0.00
ATOM 314 CE1 HIE 20 -10.651 -1.804 -4.466 1.00 0.00
ATOM 315 HE1 HIE 20 -10.304 -1.033 -5.154 1.00 0.00
ATOM 316 NE2 HIE 20 -11.874 -2.087 -4.012 1.00 0.00
ATOM 317 HE2 HIE 20 -12.757 -1.639 -4.210 1.00 0.00
ATOM 318 CD2 HIE 20 -11.705 -3.177 -3.164 1.00 0.00
ATOM 319 HD2 HIE 20 -12.566 -3.612 -2.657 1.00 0.00
ATOM 320 C HIE 20 -8.492 -4.660 -0.143 1.00 0.00
ATOM 321 O HIE 20 -8.278 -5.755 0.376 1.00 0.00
ATOM 322 N CYX 21 -7.625 -3.654 -0.121 1.00 0.00
ATOM 323 H CYX 21 -7.848 -2.791 -0.595 1.00 0.00
ATOM 324 CA CYX 21 -6.335 -3.766 0.531 1.00 0.00
ATOM 325 HA CYX 21 -5.762 -4.593 0.112 1.00 0.00
ATOM 326 CB CYX 21 -5.554 -2.464 0.305 1.00 0.00
ATOM 327 HB2 CYX 21 -6.243 -1.673 0.012 1.00 0.00
ATOM 328 HB3 CYX 21 -5.046 -2.180 1.227 1.00 0.00
ATOM 329 SG CYX 21 -4.358 -2.724 -1.030 1.00 0.00
ATOM 330 C CYX 21 -6.537 -4.045 2.020 1.00 0.00
ATOM 331 O CYX 21 -5.924 -4.954 2.582 1.00 0.00

```
ATOM    332  N   ARG    22      -7.437  -3.288   2.641  1.00  0.00
ATOM    333  H   ARG    22      -7.913  -2.546   2.149  1.00  0.00
ATOM    334  CA  ARG    22      -7.753  -3.497   4.047  1.00  0.00
ATOM    335  HA  ARG    22      -6.861  -3.345   4.655  1.00  0.00
ATOM    336  CB  ARG    22      -8.829  -2.506   4.500  1.00  0.00
ATOM    337  HB2 ARG    22      -8.530  -1.497   4.217  1.00  0.00
ATOM    338  HB3 ARG    22      -9.773  -2.755   4.016  1.00  0.00
ATOM    339  CG  ARG    22      -9.001  -2.579   6.020  1.00  0.00
ATOM    340  HG2 ARG    22      -9.220  -3.606   6.313  1.00  0.00
ATOM    341  HG3 ARG    22      -8.082  -2.252   6.507  1.00  0.00
ATOM    342  CD  ARG    22     -10.157  -1.669   6.447  1.00  0.00
ATOM    343  HD2 ARG    22     -11.098  -2.057   6.057  1.00  0.00
ATOM    344  HD3 ARG    22     -10.208  -1.620   7.535  1.00  0.00
ATOM    345  NE  ARG    22      -9.955  -0.318   5.926  1.00  0.00
ATOM    346  HE  ARG    22      -9.333   0.319   6.404  1.00  0.00
ATOM    347  CZ  ARG    22     -10.577   0.106   4.823  1.00  0.00
ATOM    348  NH1 ARG    22     -11.406  -0.680   4.188  1.00  0.00
ATOM    349 HH11 ARG    22     -11.577  -1.614   4.534  1.00  0.00
ATOM    350 HH12 ARG    22     -11.871  -0.351   3.355  1.00  0.00
ATOM    351  NH2 ARG    22     -10.356   1.311   4.374  1.00  0.00
ATOM    352 HH21 ARG    22      -9.714   1.919   4.863  1.00  0.00
ATOM    353 HH22 ARG    22     -10.827   1.630   3.540  1.00  0.00
```

ATOM	354	C	ARG	22	-8.250	-4.923	4.218	1.00	0.00
ATOM	355	O	ARG	22	-7.820	-5.650	5.121	1.00	0.00
ATOM	356	N	MET	23	-9.141	-5.329	3.307	1.00	0.00
ATOM	357	H	MET	23	-9.477	-4.699	2.594	1.00	0.00
ATOM	358	CA	MET	23	-9.665	-6.684	3.332	1.00	0.00
ATOM	359	HA	MET	23	-10.215	-6.852	4.258	1.00	0.00
ATOM	360	CB	MET	23	-10.615	-6.909	2.149	1.00	0.00
ATOM	361	HB2	MET	23	-11.368	-6.121	2.136	1.00	0.00
ATOM	362	HB3	MET	23	-10.046	-6.883	1.220	1.00	0.00
ATOM	363	CG	MET	23	-11.303	-8.269	2.288	1.00	0.00
ATOM	364	HG2	MET	23	-10.552	-9.055	2.373	1.00	0.00
ATOM	365	HG3	MET	23	-11.934	-8.274	3.177	1.00	0.00
ATOM	366	SD	MET	23	-12.328	-8.579	0.828	1.00	0.00
ATOM	367	CE	MET	23	-10.981	-8.904	-0.338	1.00	0.00
ATOM	368	HE1	MET	23	-10.398	-9.758	0.008	1.00	0.00
ATOM	369	HE2	MET	23	-11.396	-9.123	-1.322	1.00	0.00
ATOM	370	HE3	MET	23	-10.336	-8.028	-0.403	1.00	0.00
ATOM	371	C	MET	23	-8.500	-7.664	3.263	1.00	0.00
ATOM	372	O	MET	23	-8.500	-8.692	3.937	1.00	0.00
ATOM	373	N	TYR	24	-7.498	-7.315	2.452	1.00	0.00
ATOM	374	H	TYR	24	-7.572	-6.482	1.886	1.00	0.00
ATOM	375	CA	TYR	24	-6.307	-8.145	2.309	1.00	0.00

ATOM	376	HA	TYR	24	-6.583	-9.142	1.964	1.00	0.00
ATOM	377	CB	TYR	24	-5.349	-7.514	1.277	1.00	0.00
ATOM	378	HB2	TYR	24	-5.341	-8.129	0.377	1.00	0.00
ATOM	379	HB3	TYR	24	-5.728	-6.521	1.036	1.00	0.00
ATOM	380	CG	TYR	24	-3.936	-7.397	1.821	1.00	0.00
ATOM	381	CD1	TYR	24	-3.239	-8.539	2.232	1.00	0.00
ATOM	382	HD1	TYR	24	-3.713	-9.519	2.171	1.00	0.00
ATOM	383	CE1	TYR	24	-1.932	-8.423	2.722	1.00	0.00
ATOM	384	HE1	TYR	24	-1.392	-9.312	3.050	1.00	0.00
ATOM	385	CZ	TYR	24	-1.321	-7.166	2.795	1.00	0.00
ATOM	386	OH	TYR	24	-0.031	-7.052	3.271	1.00	0.00
ATOM	387	HH	TYR	24	0.352	-7.896	3.524	1.00	0.00
ATOM	388	CE2	TYR	24	-2.015	-6.026	2.382	1.00	0.00
ATOM	389	HE2	TYR	24	-1.538	-5.048	2.439	1.00	0.00
ATOM	390	CD2	TYR	24	-3.322	-6.140	1.895	1.00	0.00
ATOM	391	HD2	TYR	24	-3.863	-5.251	1.573	1.00	0.00
ATOM	392	C	TYR	24	-5.631	-8.316	3.667	1.00	0.00
ATOM	393	O	TYR	24	-5.225	-9.416	4.031	1.00	0.00
ATOM	394	N	MET	25	-5.525	-7.224	4.423	1.00	0.00
ATOM	395	H	MET	25	-5.866	-6.330	4.102	1.00	0.00
ATOM	396	CA	MET	25	-4.911	-7.296	5.746	1.00	0.00
ATOM	397	HA	MET	25	-3.862	-7.580	5.656	1.00	0.00

ATOM	398	CB	MET	25	-4.991	-5.934	6.437	1.00	0.00
ATOM	399	HB2	MET	25	-6.037	-5.660	6.571	1.00	0.00
ATOM	400	HB3	MET	25	-4.505	-5.996	7.411	1.00	0.00
ATOM	401	CG	MET	25	-4.289	-4.871	5.582	1.00	0.00
ATOM	402	HG2	MET	25	-4.705	-4.879	4.575	1.00	0.00
ATOM	403	HG3	MET	25	-4.436	-3.888	6.029	1.00	0.00
ATOM	404	SD	MET	25	-2.517	-5.226	5.495	1.00	0.00
ATOM	405	CE	MET	25	-2.109	-4.778	7.200	1.00	0.00
ATOM	406	HE1	MET	25	-2.674	-5.408	7.887	1.00	0.00
ATOM	407	HE2	MET	25	-1.042	-4.924	7.369	1.00	0.00
ATOM	408	HE3	MET	25	-2.364	-3.733	7.373	1.00	0.00
ATOM	409	C	MET	25	-5.656	-8.330	6.585	1.00	0.00
ATOM	410	O	MET	25	-5.058	-9.248	7.166	1.00	0.00
ATOM	411	N	ARG	26	-6.981	-8.196	6.613	1.00	0.00
ATOM	412	H	ARG	26	-7.435	-7.428	6.141	1.00	0.00
ATOM	413	CA	ARG	26	-7.808	-9.141	7.346	1.00	0.00
ATOM	414	HA	ARG	26	-7.532	-9.137	8.401	1.00	0.00
ATOM	415	CB	ARG	26	-9.287	-8.753	7.224	1.00	0.00
ATOM	416	HB2	ARG	26	-9.418	-7.725	7.560	1.00	0.00
ATOM	417	HB3	ARG	26	-9.596	-8.837	6.182	1.00	0.00
ATOM	418	CG	ARG	26	-10.144	-9.685	8.088	1.00	0.00
ATOM	419	HG2	ARG	26	-11.196	-9.532	7.848	1.00	0.00

ATOM 420 HG3 ARG 26 -9.871 -10.720 7.880 1.00 0.00

ATOM 421 CD ARG 26 -9.910 -9.384 9.571 1.00 0.00

ATOM 422 HD2 ARG 26 -8.848 -9.463 9.804 1.00 0.00

ATOM 423 HD3 ARG 26 -10.259 -8.379 9.806 1.00 0.00

ATOM 424 NE ARG 26 -10.642 -10.340 10.399 1.00 0.00

ATOM 425 HE ARG 26 -11.551 -10.669 10.106 1.00 0.00

ATOM 426 CZ ARG 26 -10.139 -10.798 11.546 1.00 0.00

ATOM 427 NH1 ARG 26 -8.971 -10.392 11.965 1.00 0.00

ATOM 428 HH11 ARG 26 -8.444 -9.727 11.417 1.00 0.00

ATOM 429 HH12 ARG 26 -8.599 -10.746 12.835 1.00 0.00

ATOM 430 NH2 ARG 26 -10.820 -11.657 12.253 1.00 0.00

ATOM 431 HH21 ARG 26 -11.723 -11.971 11.928 1.00 0.00

ATOM 432 HH22 ARG 26 -10.440 -12.005 13.122 1.00 0.00

ATOM 433 C ARG 26 -7.574 -10.534 6.776 1.00 0.00

ATOM 434 O ARG 26 -7.465 -11.521 7.511 1.00 0.00

ATOM 435 N GLN 27 -7.457 -10.594 5.454 1.00 0.00

ATOM 436 H GLN 27 -7.581 -9.766 4.889 1.00 0.00

ATOM 437 CA GLN 27 -7.192 -11.847 4.784 1.00 0.00

ATOM 438 HA GLN 27 -7.986 -12.559 5.009 1.00 0.00

ATOM 439 CB GLN 27 -7.129 -11.647 3.262 1.00 0.00

ATOM 440 HB2 GLN 27 -8.107 -11.330 2.902 1.00 0.00

ATOM 441 HB3 GLN 27 -6.390 -10.879 3.032 1.00 0.00

ATOM 442 CG GLN 27 -6.732 -12.961 2.574 1.00 0.00
ATOM 443 HG2 GLN 27 -6.848 -12.855 1.496 1.00 0.00
ATOM 444 HG3 GLN 27 -5.696 -13.211 2.805 1.00 0.00
ATOM 445 CD GLN 27 -7.625 -14.101 3.050 1.00 0.00
ATOM 446 OE1 GLN 27 -8.467 -14.593 2.299 1.00 0.00
ATOM 447 NE2 GLN 27 -7.492 -14.542 4.270 1.00 0.00
ATOM 448 HE21 GLN 27 -8.077 -15.297 4.598 1.00 0.00
ATOM 449 HE22 GLN 27 -6.804 -14.127 4.882 1.00 0.00
ATOM 450 C GLN 27 -5.885 -12.418 5.304 1.00 0.00
ATOM 451 O GLN 27 -5.751 -13.616 5.502 1.00 0.00
ATOM 452 N GLN 28 -4.920 -11.556 5.560 1.00 0.00
ATOM 453 H GLN 28 -5.029 -10.564 5.408 1.00 0.00
ATOM 454 CA GLN 28 -3.665 -12.032 6.073 1.00 0.00
ATOM 455 HA GLN 28 -3.201 -12.717 5.363 1.00 0.00
ATOM 456 CB GLN 28 -2.736 -10.863 6.294 1.00 0.00
ATOM 457 HB2 GLN 28 -2.683 -10.272 5.380 1.00 0.00
ATOM 458 HB3 GLN 28 -3.125 -10.246 7.104 1.00 0.00
ATOM 459 CG GLN 28 -1.334 -11.362 6.661 1.00 0.00
ATOM 460 HG2 GLN 28 -1.094 -12.253 6.081 1.00 0.00
ATOM 461 HG3 GLN 28 -0.598 -10.585 6.454 1.00 0.00
ATOM 462 CD GLN 28 -1.267 -11.714 8.145 1.00 0.00
ATOM 463 OE1 GLN 28 -0.829 -12.889 8.507 1.00 0.00

```
ATOM    464  NE2 GLN    28      -1.623 -10.898   8.996  1.00  0.00
ATOM    465 HE21 GLN    28      -1.574 -11.141   9.976  1.00  0.00
ATOM    466 HE22 GLN    28      -1.961  -9.991   8.711  1.00  0.00
ATOM    467  C   GLN    28      -3.907 -12.749   7.390  1.00  0.00
ATOM    468  O   GLN    28      -3.300 -13.785   7.666  1.00  0.00
ATOM    469  N   MET    29      -4.812 -12.193   8.198  1.00  0.00
ATOM    470  H   MET    29      -5.276 -11.332   7.949  1.00  0.00
ATOM    471  CA  MET    29      -5.133 -12.806   9.484  1.00  0.00
ATOM    472  HA  MET    29      -4.233 -12.870  10.097  1.00  0.00
ATOM    473  CB  MET    29      -6.177 -11.971  10.233  1.00  0.00
ATOM    474  HB2 MET    29      -6.988 -11.711   9.554  1.00  0.00
ATOM    475  HB3 MET    29      -6.573 -12.550  11.067  1.00  0.00
ATOM    476  CG  MET    29      -5.528 -10.692  10.764  1.00  0.00
ATOM    477  HG2 MET    29      -5.148 -10.096   9.935  1.00  0.00
ATOM    478  HG3 MET    29      -6.259 -10.110  11.325  1.00  0.00
ATOM    479  SD  MET    29      -4.155 -11.134  11.857  1.00  0.00
ATOM    480  CE  MET    29      -5.115 -12.085  13.064  1.00  0.00
ATOM    481  HE1 MET    29      -5.597 -12.925  12.565  1.00  0.00
ATOM    482  HE2 MET    29      -4.451 -12.459  13.844  1.00  0.00
ATOM    483  HE3 MET    29      -5.874 -11.444  13.512  1.00  0.00
ATOM    484  C   MET    29      -5.664 -14.221   9.279  1.00  0.00
ATOM    485  O   MET    29      -5.119 -15.177   9.828  1.00  0.00
```

ATOM	486	N	GLU	30	-6.717	-14.358	8.473	1.00	0.00
ATOM	487	H	GLU	30	-7.163	-13.557	8.051	1.00	0.00
ATOM	488	CA	GLU	30	-7.275	-15.685	8.205	1.00	0.00
ATOM	489	HA	GLU	30	-7.482	-16.195	9.146	1.00	0.00
ATOM	490	CB	GLU	30	-8.581	-15.570	7.412	1.00	0.00
ATOM	491	HB2	GLU	30	-8.415	-14.952	6.530	1.00	0.00
ATOM	492	HB3	GLU	30	-8.904	-16.564	7.103	1.00	0.00
ATOM	493	CG	GLU	30	-9.663	-14.931	8.287	1.00	0.00
ATOM	494	HG2	GLU	30	-10.644	-15.282	7.968	1.00	0.00
ATOM	495	HG3	GLU	30	-9.493	-15.222	9.324	1.00	0.00
ATOM	496	CD	GLU	30	-9.604	-13.412	8.170	1.00	0.00
ATOM	497	OE1	GLU	30	-9.745	-12.917	7.063	1.00	0.00
ATOM	498	OE2	GLU	30	-9.427	-12.765	9.190	1.00	0.00
ATOM	499	C	GLU	30	-6.276	-16.537	7.423	1.00	0.00
ATOM	500	O	GLU	30	-6.093	-17.722	7.700	1.00	0.00
ATOM	501	N	GLU	31	-5.642	-15.907	6.436	1.00	0.00
ATOM	502	H	GLU	31	-5.863	-14.941	6.245	1.00	0.00
ATOM	503	CA	GLU	31	-4.660	-16.575	5.584	1.00	0.00
ATOM	504	HA	GLU	31	-5.070	-17.507	5.194	1.00	0.00
ATOM	505	CB	GLU	31	-4.300	-15.666	4.403	1.00	0.00
ATOM	506	HB2	GLU	31	-5.208	-15.205	4.016	1.00	0.00
ATOM	507	HB3	GLU	31	-3.616	-14.889	4.744	1.00	0.00

ATOM 508 CG GLU 31 -3.629 -16.486 3.293 1.00 0.00
ATOM 509 HG2 GLU 31 -2.771 -17.012 3.711 1.00 0.00
ATOM 510 HG3 GLU 31 -4.346 -17.211 2.907 1.00 0.00
ATOM 511 CD GLU 31 -3.163 -15.574 2.155 1.00 0.00
ATOM 512 OE1 GLU 31 -3.304 -14.366 2.281 1.00 0.00
ATOM 513 OE2 GLU 31 -2.669 -16.100 1.170 1.00 0.00
ATOM 514 C GLU 31 -3.395 -16.939 6.369 1.00 0.00
ATOM 515 O GLU 31 -2.489 -17.565 5.827 1.00 0.00
ATOM 516 N SER 32 -3.334 -16.533 7.639 1.00 0.00
ATOM 517 H SER 32 -4.094 -16.000 8.037 1.00 0.00
ATOM 518 CA SER 32 -2.166 -16.812 8.481 1.00 0.00
ATOM 519 HA SER 32 -1.339 -16.160 8.200 1.00 0.00
ATOM 520 CB SER 32 -2.507 -16.563 9.950 1.00 0.00
ATOM 521 HB2 SER 32 -1.623 -16.738 10.564 1.00 0.00
ATOM 522 HB3 SER 32 -2.839 -15.533 10.078 1.00 0.00
ATOM 523 OG SER 32 -3.545 -17.449 10.350 1.00 0.00
ATOM 524 HG SER 32 -3.760 -17.294 11.273 1.00 0.00
ATOM 525 C SER 32 -1.697 -18.253 8.300 1.00 0.00
ATOM 526 O SER 32 -0.539 -18.575 8.565 1.00 0.00
ATOM 527 N THR 33 -2.605 -19.111 7.848 1.00 0.00
ATOM 528 H THR 33 -3.554 -18.807 7.686 1.00 0.00
ATOM 529 CA THR 33 -2.277 -20.509 7.605 1.00 0.00

```
ATOM    530  HA  THR    33      -1.645 -20.894   8.406  1.00  0.00
ATOM    531  CB  THR    33      -3.564 -21.340   7.555  1.00  0.00
ATOM    532  HB  THR    33      -3.320 -22.371   7.296  1.00  0.00
ATOM    533  CG2 THR    33      -4.252 -21.307   8.922  1.00  0.00
ATOM    534 HG21 THR    33      -4.497 -20.277   9.182  1.00  0.00
ATOM    535 HG22 THR    33      -5.166 -21.899   8.884  1.00  0.00
ATOM    536 HG23 THR    33      -3.583 -21.722   9.676  1.00  0.00
ATOM    537  OG1 THR    33      -4.438 -20.800   6.572  1.00  0.00
ATOM    538  HG1 THR    33      -5.244 -21.320   6.541  1.00  0.00
ATOM    539  C   THR    33      -1.500 -20.683   6.288  1.00  0.00
ATOM    540  O   THR    33      -0.393 -21.221   6.273  1.00  0.00
ATOM    541  N   TYR    34      -2.129 -20.262   5.181  1.00  0.00
ATOM    542  H   TYR    34      -3.051 -19.859   5.265  1.00  0.00
ATOM    543  CA  TYR    34      -1.549 -20.405   3.834  1.00  0.00
ATOM    544  HA  TYR    34      -0.762 -21.160   3.837  1.00  0.00
ATOM    545  CB  TYR    34      -2.644 -20.843   2.857  1.00  0.00
ATOM    546  HB2 TYR    34      -3.413 -20.073   2.801  1.00  0.00
ATOM    547  HB3 TYR    34      -2.207 -20.992   1.870  1.00  0.00
ATOM    548  CG  TYR    34      -3.261 -22.137   3.335  1.00  0.00
ATOM    549  CD1 TYR    34      -2.583 -23.348   3.148  1.00  0.00
ATOM    550  HD1 TYR    34      -1.610 -23.358   2.656  1.00  0.00
ATOM    551  CE1 TYR    34      -3.154 -24.546   3.593  1.00  0.00
```

ATOM	552	HE1	TYR	34	-2.626	-25.489	3.447	1.00	0.00
ATOM	553	CZ	TYR	34	-4.403	-24.534	4.226	1.00	0.00
ATOM	554	OH	TYR	34	-4.965	-25.715	4.664	1.00	0.00
ATOM	555	HH	TYR	34	-4.415	-26.482	4.484	1.00	0.00
ATOM	556	CE2	TYR	34	-5.080	-23.323	4.413	1.00	0.00
ATOM	557	HE2	TYR	34	-6.052	-23.314	4.906	1.00	0.00
ATOM	558	CD2	TYR	34	-4.510	-22.126	3.969	1.00	0.00
ATOM	559	HD2	TYR	34	-5.037	-21.184	4.116	1.00	0.00
ATOM	560	C	TYR	34	-0.884	-19.127	3.293	1.00	0.00
ATOM	561	O	TYR	34	-0.500	-19.095	2.123	1.00	0.00
ATOM	562	N	GLN	35	-0.794	-18.073	4.107	1.00	0.00
ATOM	563	H	GLN	35	-1.149	-18.129	5.051	1.00	0.00
ATOM	564	CA	GLN	35	-0.214	-16.793	3.652	1.00	0.00
ATOM	565	HA	GLN	35	-0.984	-16.208	3.151	1.00	0.00
ATOM	566	CB	GLN	35	0.338	-15.974	4.825	1.00	0.00
ATOM	567	HB2	GLN	35	0.543	-14.960	4.483	1.00	0.00
ATOM	568	HB3	GLN	35	-0.408	-15.946	5.620	1.00	0.00
ATOM	569	CG	GLN	35	1.633	-16.604	5.361	1.00	0.00
ATOM	570	HG2	GLN	35	2.353	-16.732	4.552	1.00	0.00
ATOM	571	HG3	GLN	35	2.063	-15.963	6.131	1.00	0.00
ATOM	572	CD	GLN	35	1.341	-17.967	5.971	1.00	0.00
ATOM	573	OE1	GLN	35	0.138	-18.225	6.388	1.00	0.00

ATOM 574 NE2 GLN 35 2.227 -18.815 6.064 1.00 0.00
ATOM 575 HE21 GLN 35 2.019 -19.716 6.471 1.00 0.00
ATOM 576 HE22 GLN 35 3.158 -18.607 5.733 1.00 0.00
ATOM 577 C GLN 35 0.910 -16.976 2.643 1.00 0.00
ATOM 578 O GLN 35 1.102 -16.120 1.778 1.00 0.00
ATOM 579 N THR 36 1.658 -18.070 2.745 1.00 0.00
ATOM 580 H THR 36 1.503 -18.763 3.463 1.00 0.00
ATOM 581 CA THR 36 2.748 -18.280 1.810 1.00 0.00
ATOM 582 HA THR 36 3.471 -17.468 1.889 1.00 0.00
ATOM 583 CB THR 36 3.460 -19.601 2.120 1.00 0.00
ATOM 584 HB THR 36 2.784 -20.432 1.918 1.00 0.00
ATOM 585 CG2 THR 36 4.708 -19.734 1.243 1.00 0.00
ATOM 586 HG21 THR 36 5.385 -18.904 1.445 1.00 0.00
ATOM 587 HG22 THR 36 5.212 -20.675 1.467 1.00 0.00
ATOM 588 HG23 THR 36 4.418 -19.719 0.193 1.00 0.00
ATOM 589 OG1 THR 36 3.838 -19.621 3.489 1.00 0.00
ATOM 590 HG1 THR 36 4.285 -20.448 3.686 1.00 0.00
ATOM 591 C THR 36 2.186 -18.309 0.395 1.00 0.00
ATOM 592 O THR 36 1.526 -19.266 -0.009 1.00 0.00
ATOM 593 N MET 37 2.446 -17.233 -0.339 1.00 0.00
ATOM 594 H MET 37 2.975 -16.468 0.055 1.00 0.00
ATOM 595 CA MET 37 1.959 -17.104 -1.709 1.00 0.00

ATOM	596	HA	MET	37	0.873	-17.188	-1.731	1.00	0.00
ATOM	597	CB	MET	37	2.356	-15.736	-2.271	1.00	0.00
ATOM	598	HB2	MET	37	3.433	-15.600	-2.166	1.00	0.00
ATOM	599	HB3	MET	37	2.085	-15.685	-3.325	1.00	0.00
ATOM	600	CG	MET	37	1.624	-14.631	-1.502	1.00	0.00
ATOM	601	HG2	MET	37	1.637	-14.855	-0.435	1.00	0.00
ATOM	602	HG3	MET	37	2.115	-13.674	-1.677	1.00	0.00
ATOM	603	SD	MET	37	-0.093	-14.533	-2.070	1.00	0.00
ATOM	604	CE	MET	37	0.187	-13.404	-3.456	1.00	0.00
ATOM	605	HE1	MET	37	0.883	-13.859	-4.161	1.00	0.00
ATOM	606	HE2	MET	37	-0.759	-13.203	-3.958	1.00	0.00
ATOM	607	HE3	MET	37	0.606	-12.469	-3.084	1.00	0.00
ATOM	608	C	MET	37	2.523	-18.209	-2.602	1.00	0.00
ATOM	609	O	MET	37	3.729	-18.265	-2.843	1.00	0.00
ATOM	610	N	PRO	38	1.675	-19.076	-3.104	1.00	0.00
ATOM	611	CD	PRO	38	0.218	-19.094	-2.876	1.00	0.00
ATOM	612	HD2	PRO	38	-0.219	-18.192	-3.302	1.00	0.00
ATOM	613	HD3	PRO	38	0.027	-19.119	-1.803	1.00	0.00
ATOM	614	CG	PRO	38	-0.256	-20.370	-3.584	1.00	0.00
ATOM	615	HG2	PRO	38	-0.764	-20.095	-4.508	1.00	0.00
ATOM	616	HG3	PRO	38	-0.950	-20.901	-2.932	1.00	0.00
ATOM	617	CB	PRO	38	0.959	-21.196	-3.854	1.00	0.00

ATOM	618	HB2	PRO	38	0.808	-21.723	-4.796	1.00	0.00
ATOM	619	HB3	PRO	38	1.198	-21.916	-3.071	1.00	0.00
ATOM	620	CA	PRO	38	2.095	-20.188	-3.999	1.00	0.00
ATOM	621	HA	PRO	38	3.055	-20.569	-3.650	1.00	0.00
ATOM	622	C	PRO	38	2.229	-19.722	-5.446	1.00	0.00
ATOM	623	O	PRO	38	1.463	-18.873	-5.904	1.00	0.00
ATOM	624	N	ARG	39	3.199	-20.282	-6.162	1.00	0.00
ATOM	625	H	ARG	39	3.795	-20.993	-5.762	1.00	0.00
ATOM	626	CA	ARG	39	3.408	-19.908	-7.558	1.00	0.00
ATOM	627	HA	ARG	39	3.627	-18.843	-7.625	1.00	0.00
ATOM	628	CB	ARG	39	4.578	-20.684	-8.151	1.00	0.00
ATOM	629	HB2	ARG	39	4.312	-21.739	-8.219	1.00	0.00
ATOM	630	HB3	ARG	39	4.797	-20.300	-9.147	1.00	0.00
ATOM	631	CG	ARG	39	5.815	-20.527	-7.261	1.00	0.00
ATOM	632	HG2	ARG	39	6.036	-19.468	-7.130	1.00	0.00
ATOM	633	HG3	ARG	39	5.623	-20.981	-6.288	1.00	0.00
ATOM	634	CD	ARG	39	7.011	-21.220	-7.919	1.00	0.00
ATOM	635	HD2	ARG	39	6.744	-22.242	-8.189	1.00	0.00
ATOM	636	HD3	ARG	39	7.309	-20.674	-8.814	1.00	0.00
ATOM	637	NE	ARG	39	8.143	-21.261	-6.998	1.00	0.00
ATOM	638	HE	ARG	39	8.907	-20.609	-7.105	1.00	0.00
ATOM	639	CZ	ARG	39	8.199	-22.148	-6.004	1.00	0.00

ATOM 640 NH1 ARG 39 7.229 -23.005 -5.833 1.00 0.00
ATOM 641 HH11 ARG 39 6.435 -22.995 -6.456 1.00 0.00
ATOM 642 HH12 ARG 39 7.278 -23.674 -5.078 1.00 0.00
ATOM 643 NH2 ARG 39 9.227 -22.158 -5.200 1.00 0.00
ATOM 644 HH21 ARG 39 9.976 -21.494 -5.334 1.00 0.00
ATOM 645 HH22 ARG 39 9.269 -22.830 -4.447 1.00 0.00
ATOM 646 C ARG 39 2.176 -20.249 -8.372 1.00 0.00
ATOM 647 O ARG 39 1.774 -19.498 -9.261 1.00 0.00
ATOM 648 N ARG 40 1.586 -21.403 -8.065 1.00 0.00
ATOM 649 H ARG 40 1.964 -22.003 -7.345 1.00 0.00
ATOM 650 CA ARG 40 0.409 -21.849 -8.782 1.00 0.00
ATOM 651 HA ARG 40 0.578 -21.773 -9.856 1.00 0.00
ATOM 652 CB ARG 40 0.098 -23.307 -8.439 1.00 0.00
ATOM 653 HB2 ARG 40 0.080 -23.430 -7.356 1.00 0.00
ATOM 654 HB3 ARG 40 -0.873 -23.579 -8.851 1.00 0.00
ATOM 655 CG ARG 40 1.177 -24.213 -9.037 1.00 0.00
ATOM 656 HG2 ARG 40 2.159 -23.786 -8.834 1.00 0.00
ATOM 657 HG3 ARG 40 1.111 -25.201 -8.581 1.00 0.00
ATOM 658 CD ARG 40 0.976 -24.335 -10.552 1.00 0.00
ATOM 659 HD2 ARG 40 0.990 -23.345 -11.007 1.00 0.00
ATOM 660 HD3 ARG 40 1.772 -24.943 -10.982 1.00 0.00
ATOM 661 NE ARG 40 -0.307 -24.968 -10.846 1.00 0.00

ATOM 662 HE ARG 40 -1.100 -24.402 -11.109 1.00 0.00
ATOM 663 CZ ARG 40 -0.464 -26.289 -10.776 1.00 0.00
ATOM 664 NH1 ARG 40 0.538 -27.054 -10.434 1.00 0.00
ATOM 665 HH11 ARG 40 1.435 -26.642 -10.222 1.00 0.00
ATOM 666 HH12 ARG 40 0.411 -28.055 -10.383 1.00 0.00
ATOM 667 NH2 ARG 40 -1.624 -26.821 -11.050 1.00 0.00
ATOM 668 HH21 ARG 40 -2.398 -26.229 -11.314 1.00 0.00
ATOM 669 HH22 ARG 40 -1.742 -27.823 -10.996 1.00 0.00
ATOM 670 C ARG 40 -0.776 -20.971 -8.441 1.00 0.00
ATOM 671 O ARG 40 -1.422 -21.134 -7.404 1.00 0.00
ATOM 672 N GLY 41 -1.045 -20.047 -9.342 1.00 0.00
ATOM 673 H GLY 41 -0.470 -19.971 -10.169 1.00 0.00
ATOM 674 CA GLY 41 -2.161 -19.116 -9.185 1.00 0.00
ATOM 675 HA2 GLY 41 -2.098 -18.355 -9.962 1.00 0.00
ATOM 676 HA3 GLY 41 -3.096 -19.665 -9.290 1.00 0.00
ATOM 677 C GLY 41 -2.135 -18.435 -7.819 1.00 0.00
ATOM 678 O GLY 41 -2.791 -18.887 -6.879 1.00 0.00
ATOM 679 N MET 42 -1.379 -17.342 -7.719 1.00 0.00
ATOM 680 H MET 42 -0.846 -17.017 -8.513 1.00 0.00
ATOM 681 CA MET 42 -1.276 -16.598 -6.463 1.00 0.00
ATOM 682 HA MET 42 -0.830 -17.226 -5.691 1.00 0.00
ATOM 683 CB MET 42 -0.391 -15.363 -6.661 1.00 0.00

ATOM 684 HB2 MET 42 -0.765 -14.780 -7.502 1.00 0.00
ATOM 685 HB3 MET 42 -0.413 -14.753 -5.758 1.00 0.00
ATOM 686 CG MET 42 1.048 -15.801 -6.945 1.00 0.00
ATOM 687 HG2 MET 42 1.406 -16.440 -6.137 1.00 0.00
ATOM 688 HG3 MET 42 1.088 -16.350 -7.886 1.00 0.00
ATOM 689 SD MET 42 2.110 -14.341 -7.063 1.00 0.00
ATOM 690 CE MET 42 3.637 -15.204 -7.510 1.00 0.00
ATOM 691 HE1 MET 42 3.489 -15.746 -8.444 1.00 0.00
ATOM 692 HE2 MET 42 4.442 -14.479 -7.635 1.00 0.00
ATOM 693 HE3 MET 42 3.902 -15.908 -6.720 1.00 0.00
ATOM 694 C MET 42 -2.659 -16.164 -5.978 1.00 0.00
ATOM 695 O MET 42 -3.652 -16.310 -6.692 1.00 0.00
ATOM 696 N GLU 43 -2.717 -15.632 -4.758 1.00 0.00
ATOM 697 H GLU 43 -1.883 -15.545 -4.194 1.00 0.00
ATOM 698 CA GLU 43 -3.987 -15.187 -4.190 1.00 0.00
ATOM 699 HA GLU 43 -4.735 -15.974 -4.286 1.00 0.00
ATOM 700 CB GLU 43 -3.810 -14.859 -2.703 1.00 0.00
ATOM 701 HB2 GLU 43 -3.213 -15.638 -2.228 1.00 0.00
ATOM 702 HB3 GLU 43 -3.302 -13.900 -2.600 1.00 0.00
ATOM 703 CG GLU 43 -5.183 -14.785 -2.027 1.00 0.00
ATOM 704 HG2 GLU 43 -5.765 -13.979 -2.472 1.00 0.00
ATOM 705 HG3 GLU 43 -5.700 -15.733 -2.179 1.00 0.00

ATOM 706 CD GLU 43 -5.021 -14.529 -0.530 1.00 0.00
ATOM 707 OE1 GLU 43 -4.498 -13.485 -0.180 1.00 0.00
ATOM 708 OE2 GLU 43 -5.431 -15.378 0.245 1.00 0.00
ATOM 709 C GLU 43 -4.503 -13.953 -4.933 1.00 0.00
ATOM 710 O GLU 43 -3.779 -12.970 -5.093 1.00 0.00
ATOM 711 N PRO 44 -5.733 -13.994 -5.392 1.00 0.00
ATOM 712 CD PRO 44 -6.665 -15.124 -5.242 1.00 0.00
ATOM 713 HD2 PRO 44 -6.657 -15.460 -4.205 1.00 0.00
ATOM 714 HD3 PRO 44 -6.345 -15.941 -5.890 1.00 0.00
ATOM 715 CG PRO 44 -8.013 -14.543 -5.660 1.00 0.00
ATOM 716 HG2 PRO 44 -8.550 -14.211 -4.772 1.00 0.00
ATOM 717 HG3 PRO 44 -8.593 -15.314 -6.168 1.00 0.00
ATOM 718 CB PRO 44 -7.721 -13.404 -6.578 1.00 0.00
ATOM 719 HB2 PRO 44 -8.483 -12.635 -6.451 1.00 0.00
ATOM 720 HB3 PRO 44 -7.683 -13.710 -7.623 1.00 0.00
ATOM 721 CA PRO 44 -6.355 -12.860 -6.143 1.00 0.00
ATOM 722 HA PRO 44 -5.718 -12.635 -6.998 1.00 0.00
ATOM 723 C PRO 44 -6.517 -11.607 -5.285 1.00 0.00
ATOM 724 O PRO 44 -6.569 -10.493 -5.810 1.00 0.00
ATOM 725 N HIE 45 -6.601 -11.794 -3.970 1.00 0.00
ATOM 726 H HIE 45 -6.573 -12.725 -3.580 1.00 0.00
ATOM 727 CA HIE 45 -6.763 -10.665 -3.057 1.00 0.00

ATOM 728 HA HIE 45 -7.736 -10.200 -3.210 1.00 0.00
ATOM 729 CB HIE 45 -6.672 -11.143 -1.605 1.00 0.00
ATOM 730 HB2 HIE 45 -5.754 -11.697 -1.408 1.00 0.00
ATOM 731 HB3 HIE 45 -6.757 -10.319 -0.897 1.00 0.00
ATOM 732 CG HIE 45 -7.812 -12.081 -1.313 1.00 0.00
ATOM 733 ND1 HIE 45 -7.606 -13.391 -0.911 1.00 0.00
ATOM 734 CE1 HIE 45 -8.814 -13.957 -0.738 1.00 0.00
ATOM 735 HE1 HIE 45 -8.836 -14.999 -0.419 1.00 0.00
ATOM 736 NE2 HIE 45 -9.804 -13.100 -1.000 1.00 0.00
ATOM 737 HE2 HIE 45 -10.804 -13.236 -0.958 1.00 0.00
ATOM 738 CD2 HIE 45 -9.174 -11.915 -1.363 1.00 0.00
ATOM 739 HD2 HIE 45 -9.768 -11.042 -1.630 1.00 0.00
ATOM 740 C HIE 45 -5.691 -9.609 -3.316 1.00 0.00
ATOM 741 O HIE 45 -5.989 -8.415 -3.387 1.00 0.00
ATOM 742 N MET 46 -4.447 -10.058 -3.467 1.00 0.00
ATOM 743 H MET 46 -4.244 -11.045 -3.399 1.00 0.00
ATOM 744 CA MET 46 -3.342 -9.141 -3.729 1.00 0.00
ATOM 745 HA MET 46 -3.313 -8.364 -2.965 1.00 0.00
ATOM 746 CB MET 46 -2.011 -9.901 -3.704 1.00 0.00
ATOM 747 HB2 MET 46 -1.984 -10.601 -4.539 1.00 0.00
ATOM 748 HB3 MET 46 -1.191 -9.189 -3.800 1.00 0.00
ATOM 749 CG MET 46 -1.865 -10.671 -2.389 1.00 0.00

ATOM	750	HG2	MET	46	-2.583	-11.491	-2.364	1.00	0.00
ATOM	751	HG3	MET	46	-0.854	-11.072	-2.311	1.00	0.00
ATOM	752	SD	MET	46	-2.175	-9.566	-0.990	1.00	0.00
ATOM	753	CE	MET	46	-2.781	-10.830	0.156	1.00	0.00
ATOM	754	HE1	MET	46	-2.004	-11.579	0.316	1.00	0.00
ATOM	755	HE2	MET	46	-3.038	-10.365	1.108	1.00	0.00
ATOM	756	HE3	MET	46	-3.665	-11.309	-0.265	1.00	0.00
ATOM	757	C	MET	46	-3.518	-8.488	-5.099	1.00	0.00
ATOM	758	O	MET	46	-3.303	-7.287	-5.263	1.00	0.00
ATOM	759	N	SER	47	-3.911	-9.304	-6.075	1.00	0.00
ATOM	760	H	SER	47	-4.050	-10.286	-5.880	1.00	0.00
ATOM	761	CA	SER	47	-4.119	-8.833	-7.442	1.00	0.00
ATOM	762	HA	SER	47	-3.191	-8.425	-7.843	1.00	0.00
ATOM	763	CB	SER	47	-4.565	-9.997	-8.329	1.00	0.00
ATOM	764	HB2	SER	47	-5.557	-10.329	-8.024	1.00	0.00
ATOM	765	HB3	SER	47	-4.594	-9.673	-9.369	1.00	0.00
ATOM	766	OG	SER	47	-3.644	-11.071	-8.194	1.00	0.00
ATOM	767	HG	SER	47	-3.922	-11.803	-8.750	1.00	0.00
ATOM	768	C	SER	47	-5.163	-7.720	-7.501	1.00	0.00
ATOM	769	O	SER	47	-5.080	-6.827	-8.344	1.00	0.00
ATOM	770	N	GLU	48	-6.154	-7.789	-6.616	1.00	0.00
ATOM	771	H	GLU	48	-6.202	-8.551	-5.954	1.00	0.00

ATOM 772 CA GLU 48 -7.216 -6.786 -6.597 1.00 0.00

ATOM 773 HA GLU 48 -7.780 -6.821 -7.528 1.00 0.00

ATOM 774 CB GLU 48 -8.176 -7.062 -5.431 1.00 0.00

ATOM 775 HB2 GLU 48 -8.000 -8.069 -5.051 1.00 0.00

ATOM 776 HB3 GLU 48 -7.995 -6.338 -4.637 1.00 0.00

ATOM 777 CG GLU 48 -9.629 -6.943 -5.910 1.00 0.00

ATOM 778 HG2 GLU 48 -9.677 -7.199 -6.968 1.00 0.00

ATOM 779 HG3 GLU 48 -10.243 -7.640 -5.340 1.00 0.00

ATOM 780 CD GLU 48 -10.150 -5.521 -5.706 1.00 0.00

ATOM 781 OE1 GLU 48 -9.402 -4.589 -5.952 1.00 0.00

ATOM 782 OE2 GLU 48 -11.296 -5.384 -5.309 1.00 0.00

ATOM 783 C GLU 48 -6.628 -5.382 -6.463 1.00 0.00

ATOM 784 O GLU 48 -6.917 -4.501 -7.274 1.00 0.00

ATOM 785 N CYX 49 -5.795 -5.184 -5.445 1.00 0.00

ATOM 786 H CYX 49 -5.605 -5.924 -4.784 1.00 0.00

ATOM 787 CA CYX 49 -5.166 -3.884 -5.228 1.00 0.00

ATOM 788 HA CYX 49 -5.920 -3.098 -5.248 1.00 0.00

ATOM 789 CB CYX 49 -4.463 -3.856 -3.860 1.00 0.00

ATOM 790 HB2 CYX 49 -4.257 -4.876 -3.535 1.00 0.00

ATOM 791 HB3 CYX 49 -3.525 -3.307 -3.944 1.00 0.00

ATOM 792 SG CYX 49 -5.531 -3.029 -2.653 1.00 0.00

ATOM 793 C CYX 49 -4.160 -3.576 -6.339 1.00 0.00

ATOM	794	O	CYX	49	-3.959	-2.416	-6.692	1.00	0.00
ATOM	795	N	CYX	50	-3.531	-4.624	-6.877	1.00	0.00
ATOM	796	H	CYX	50	-3.727	-5.556	-6.538	1.00	0.00
ATOM	797	CA	CYX	50	-2.532	-4.466	-7.940	1.00	0.00
ATOM	798	HA	CYX	50	-1.644	-3.970	-7.547	1.00	0.00
ATOM	799	CB	CYX	50	-2.125	-5.834	-8.486	1.00	0.00
ATOM	800	HB2	CYX	50	-1.853	-6.490	-7.659	1.00	0.00
ATOM	801	HB3	CYX	50	-2.959	-6.270	-9.035	1.00	0.00
ATOM	802	SG	CYX	50	-0.733	-5.639	-9.631	1.00	0.00
ATOM	803	C	CYX	50	-3.045	-3.613	-9.097	1.00	0.00
ATOM	804	O	CYX	50	-2.323	-2.760	-9.614	1.00	0.00
ATOM	805	N	GLU	51	-4.281	-3.862	-9.517	1.00	0.00
ATOM	806	H	GLU	51	-4.834	-4.591	-9.089	1.00	0.00
ATOM	807	CA	GLU	51	-4.858	-3.121	-10.634	1.00	0.00
ATOM	808	HA	GLU	51	-4.259	-3.272	-11.532	1.00	0.00
ATOM	809	CB	GLU	51	-6.284	-3.613	-10.907	1.00	0.00
ATOM	810	HB2	GLU	51	-6.277	-4.697	-11.025	1.00	0.00
ATOM	811	HB3	GLU	51	-6.926	-3.344	-10.068	1.00	0.00
ATOM	812	CG	GLU	51	-6.818	-2.962	-12.190	1.00	0.00
ATOM	813	HG2	GLU	51	-7.865	-3.236	-12.320	1.00	0.00
ATOM	814	HG3	GLU	51	-6.735	-1.880	-12.094	1.00	0.00
ATOM	815	CD	GLU	51	-6.013	-3.430	-13.406	1.00	0.00

ATOM	816	OE1	GLU	51	-5.522	-4.548	-13.384	1.00	0.00
ATOM	817	OE2	GLU	51	-5.903	-2.661	-14.346	1.00	0.00
ATOM	818	C	GLU	51	-4.875	-1.617	-10.361	1.00	0.00
ATOM	819	O	GLU	51	-4.657	-0.816	-11.268	1.00	0.00
ATOM	820	N	GLN	52	-5.146	-1.240	-9.114	1.00	0.00
ATOM	821	H	GLN	52	-5.351	-1.928	-8.403	1.00	0.00
ATOM	822	CA	GLN	52	-5.201	0.174	-8.746	1.00	0.00
ATOM	823	HA	GLN	52	-5.958	0.689	-9.336	1.00	0.00
ATOM	824	CB	GLN	52	-5.564	0.301	-7.269	1.00	0.00
ATOM	825	HB2	GLN	52	-4.806	-0.194	-6.661	1.00	0.00
ATOM	826	HB3	GLN	52	-5.619	1.354	-6.994	1.00	0.00
ATOM	827	CG	GLN	52	-6.924	-0.361	-7.027	1.00	0.00
ATOM	828	HG2	GLN	52	-7.055	-1.201	-7.709	1.00	0.00
ATOM	829	HG3	GLN	52	-6.985	-0.716	-5.998	1.00	0.00
ATOM	830	CD	GLN	52	-8.050	0.641	-7.265	1.00	0.00
ATOM	831	OE1	GLN	52	-8.153	1.217	-8.347	1.00	0.00
ATOM	832	NE2	GLN	52	-8.907	0.883	-6.310	1.00	0.00
ATOM	833	HE21	GLN	52	-9.654	1.545	-6.462	1.00	0.00
ATOM	834	HE22	GLN	52	-8.818	0.406	-5.423	1.00	0.00
ATOM	835	C	GLN	52	-3.872	0.877	-9.024	1.00	0.00
ATOM	836	O	GLN	52	-3.838	1.890	-9.722	1.00	0.00
ATOM	837	N	LEU	53	-2.776	0.327	-8.493	1.00	0.00

```
ATOM    838  H   LEU    53      -2.833  -0.529  -7.959  1.00  0.00
ATOM    839  CA  LEU    53      -1.453   0.917  -8.722  1.00  0.00
ATOM    840  HA  LEU    53      -1.424   1.922  -8.301  1.00  0.00
ATOM    841  CB  LEU    53      -0.353   0.077  -8.059  1.00  0.00
ATOM    842  HB2 LEU    53      -0.187  -0.802  -8.682  1.00  0.00
ATOM    843  HB3 LEU    53       0.562   0.668  -8.016  1.00  0.00
ATOM    844  CG  LEU    53      -0.742  -0.367  -6.642  1.00  0.00
ATOM    845  HG  LEU    53       0.155  -0.362  -6.021  1.00  0.00
ATOM    846  CD1 LEU    53      -1.797   0.565  -6.025  1.00  0.00
ATOM    847 HD11 LEU    53      -2.694   0.560  -6.644  1.00  0.00
ATOM    848 HD12 LEU    53      -2.046   0.218  -5.022  1.00  0.00
ATOM    849 HD13 LEU    53      -1.399   1.578  -5.970  1.00  0.00
ATOM    850  CD2 LEU    53      -1.297  -1.788  -6.729  1.00  0.00
ATOM    851 HD21 LEU    53      -0.534  -2.453  -7.135  1.00  0.00
ATOM    852 HD22 LEU    53      -1.582  -2.129  -5.733  1.00  0.00
ATOM    853 HD23 LEU    53      -2.171  -1.798  -7.380  1.00  0.00
ATOM    854  C   LEU    53      -1.183   0.909 -10.208  1.00  0.00
ATOM    855  O   LEU    53      -0.808   1.912 -10.815  1.00  0.00
ATOM    856  N   GLU    54      -1.402  -0.266 -10.762  1.00  0.00
ATOM    857  H   GLU    54      -1.667  -1.037 -10.165  1.00  0.00
ATOM    858  CA  GLU    54      -1.228  -0.521 -12.170  1.00  0.00
ATOM    859  HA  GLU    54      -0.185  -0.417 -12.470  1.00  0.00
```

ATOM	860	CB	GLU	54	-1.664	-1.962	-12.415	1.00	0.00
ATOM	861	HB2	GLU	54	-0.956	-2.632	-11.926	1.00	0.00
ATOM	862	HB3	GLU	54	-2.656	-2.108	-11.987	1.00	0.00
ATOM	863	CG	GLU	54	-1.705	-2.269	-13.918	1.00	0.00
ATOM	864	HG2	GLU	54	-2.338	-1.532	-14.412	1.00	0.00
ATOM	865	HG3	GLU	54	-0.694	-2.207	-14.321	1.00	0.00
ATOM	866	CD	GLU	54	-2.267	-3.672	-14.165	1.00	0.00
ATOM	867	OE1	GLU	54	-2.584	-4.352	-13.201	1.00	0.00
ATOM	868	OE2	GLU	54	-2.371	-4.047	-15.322	1.00	0.00
ATOM	869	C	GLU	54	-2.027	0.478	-13.008	1.00	0.00
ATOM	870	O	GLU	54	-1.607	0.860	-14.101	1.00	0.00
ATOM	871	N	GLY	55	-3.166	0.913	-12.474	1.00	0.00
ATOM	872	H	GLY	55	-3.468	0.571	-11.573	1.00	0.00
ATOM	873	CA	GLY	55	-4.009	1.888	-13.164	1.00	0.00
ATOM	874	HA2	GLY	55	-3.916	1.750	-14.241	1.00	0.00
ATOM	875	HA3	GLY	55	-5.048	1.744	-12.867	1.00	0.00
ATOM	876	C	GLY	55	-3.582	3.311	-12.806	1.00	0.00
ATOM	877	O	GLY	55	-3.979	4.275	-13.461	1.00	0.00
ATOM	878	N	MET	56	-2.766	3.425	-11.760	1.00	0.00
ATOM	879	H	MET	56	-2.485	2.598	-11.252	1.00	0.00
ATOM	880	CA	MET	56	-2.271	4.722	-11.302	1.00	0.00
ATOM	881	HA	MET	56	-3.025	5.492	-11.462	1.00	0.00

ATOM	882	CB	MET	56	-1.954	4.650	-9.801	1.00	0.00
ATOM	883	HB2	MET	56	-2.870	4.432	-9.251	1.00	0.00
ATOM	884	HB3	MET	56	-1.228	3.856	-9.626	1.00	0.00
ATOM	885	CG	MET	56	-1.376	5.984	-9.321	1.00	0.00
ATOM	886	HG2	MET	56	-0.995	5.875	-8.305	1.00	0.00
ATOM	887	HG3	MET	56	-0.565	6.292	-9.981	1.00	0.00
ATOM	888	SD	MET	56	-2.670	7.247	-9.338	1.00	0.00
ATOM	889	CE	MET	56	-2.658	7.610	-7.567	1.00	0.00
ATOM	890	HE1	MET	56	-1.669	7.963	-7.274	1.00	0.00
ATOM	891	HE2	MET	56	-3.397	8.380	-7.348	1.00	0.00
ATOM	892	HE3	MET	56	-2.900	6.705	-7.008	1.00	0.00
ATOM	893	C	MET	56	-1.016	5.117	-12.073	1.00	0.00
ATOM	894	O	MET	56	-0.173	4.269	-12.375	1.00	0.00
ATOM	895	N	ASP	57	-0.892	6.409	-12.386	1.00	0.00
ATOM	896	H	ASP	57	-1.615	7.070	-12.139	1.00	0.00
ATOM	897	CA	ASP	57	0.275	6.899	-13.116	1.00	0.00
ATOM	898	HA	ASP	57	0.282	6.488	-14.126	1.00	0.00
ATOM	899	CB	ASP	57	0.235	8.429	-13.209	1.00	0.00
ATOM	900	HB2	ASP	57	0.163	8.841	-12.202	1.00	0.00
ATOM	901	HB3	ASP	57	1.154	8.779	-13.679	1.00	0.00
ATOM	902	CG	ASP	57	-0.968	8.888	-14.036	1.00	0.00
ATOM	903	OD1	ASP	57	-1.533	8.071	-14.747	1.00	0.00

ATOM	904	OD2	ASP	57	-1.307	10.057	-13.946	1.00	0.00
ATOM	905	C	ASP	57	1.558	6.457	-12.416	1.00	0.00
ATOM	906	O	ASP	57	1.605	6.373	-11.189	1.00	0.00
ATOM	907	N	GLU	58	2.590	6.163	-13.202	1.00	0.00
ATOM	908	H	GLU	58	2.505	6.227	-14.206	1.00	0.00
ATOM	909	CA	GLU	58	3.861	5.712	-12.643	1.00	0.00
ATOM	910	HA	GLU	58	3.736	4.745	-12.156	1.00	0.00
ATOM	911	CB	GLU	58	4.894	5.566	-13.762	1.00	0.00
ATOM	912	HB2	GLU	58	4.620	4.732	-14.408	1.00	0.00
ATOM	913	HB3	GLU	58	4.931	6.483	-14.349	1.00	0.00
ATOM	914	CG	GLU	58	6.266	5.301	-13.145	1.00	0.00
ATOM	915	HG2	GLU	58	6.532	6.136	-12.497	1.00	0.00
ATOM	916	HG3	GLU	58	6.225	4.384	-12.556	1.00	0.00
ATOM	917	CD	GLU	58	7.317	5.154	-14.242	1.00	0.00
ATOM	918	OE1	GLU	58	7.322	4.124	-14.898	1.00	0.00
ATOM	919	OE2	GLU	58	8.104	6.072	-14.409	1.00	0.00
ATOM	920	C	GLU	58	4.396	6.679	-11.590	1.00	0.00
ATOM	921	O	GLU	58	4.638	6.289	-10.448	1.00	0.00
ATOM	922	N	SER	59	4.583	7.936	-11.978	1.00	0.00
ATOM	923	H	SER	59	4.390	8.214	-12.929	1.00	0.00
ATOM	924	CA	SER	59	5.092	8.943	-11.052	1.00	0.00
ATOM	925	HA	SER	59	6.097	8.676	-10.722	1.00	0.00

ATOM	926	CB	SER	59	5.151	10.304	-11.748	1.00	0.00
ATOM	927	HB2	SER	59	4.145	10.616	-12.027	1.00	0.00
ATOM	928	HB3	SER	59	5.586	11.041	-11.073	1.00	0.00
ATOM	929	OG	SER	59	5.955	10.199	-12.916	1.00	0.00
ATOM	930	HG	SER	59	5.994	11.051	-13.355	1.00	0.00
ATOM	931	C	SER	59	4.195	9.032	-9.824	1.00	0.00
ATOM	932	O	SER	59	4.661	9.269	-8.709	1.00	0.00
ATOM	933	N	CYX	60	2.904	8.835	-10.052	1.00	0.00
ATOM	934	H	CYX	60	2.582	8.667	-10.994	1.00	0.00
ATOM	935	CA	CYX	60	1.913	8.887	-8.979	1.00	0.00
ATOM	936	HA	CYX	60	2.109	9.740	-8.330	1.00	0.00
ATOM	937	CB	CYX	60	0.510	9.034	-9.571	1.00	0.00
ATOM	938	HB2	CYX	60	0.287	8.169	-10.195	1.00	0.00
ATOM	939	HB3	CYX	60	-0.220	9.097	-8.764	1.00	0.00
ATOM	940	SG	CYX	60	0.431	10.546	-10.562	1.00	0.00
ATOM	941	C	CYX	60	1.976	7.634	-8.113	1.00	0.00
ATOM	942	O	CYX	60	1.570	7.649	-6.950	1.00	0.00
ATOM	943	N	ARG	61	2.463	6.545	-8.700	1.00	0.00
ATOM	944	H	ARG	61	2.769	6.582	-9.662	1.00	0.00
ATOM	945	CA	ARG	61	2.552	5.272	-7.995	1.00	0.00
ATOM	946	HA	ARG	61	1.567	4.959	-7.649	1.00	0.00
ATOM	947	CB	ARG	61	3.098	4.204	-8.947	1.00	0.00

ATOM	948	HB2	ARG	61	2.546	4.246	-9.885	1.00	0.00
ATOM	949	HB3	ARG	61	4.153	4.401	-9.137	1.00	0.00
ATOM	950	CG	ARG	61	2.943	2.812	-8.326	1.00	0.00
ATOM	951	HG2	ARG	61	3.414	2.794	-7.343	1.00	0.00
ATOM	952	HG3	ARG	61	1.885	2.571	-8.226	1.00	0.00
ATOM	953	CD	ARG	61	3.617	1.780	-9.232	1.00	0.00
ATOM	954	HD2	ARG	61	4.697	1.931	-9.231	1.00	0.00
ATOM	955	HD3	ARG	61	3.392	0.772	-8.882	1.00	0.00
ATOM	956	NE	ARG	61	3.126	1.921	-10.601	1.00	0.00
ATOM	957	HE	ARG	61	3.523	2.629	-11.202	1.00	0.00
ATOM	958	CZ	ARG	61	2.159	1.145	-11.086	1.00	0.00
ATOM	959	NH1	ARG	61	1.644	0.192	-10.355	1.00	0.00
ATOM	960	HH11	ARG	61	1.982	0.042	-9.415	1.00	0.00
ATOM	961	HH12	ARG	61	0.911	-0.391	-10.733	1.00	0.00
ATOM	962	NH2	ARG	61	1.729	1.335	-12.302	1.00	0.00
ATOM	963	HH21	ARG	61	2.132	2.068	-12.868	1.00	0.00
ATOM	964	HH22	ARG	61	0.996	0.748	-12.672	1.00	0.00
ATOM	965	C	ARG	61	3.463	5.382	-6.775	1.00	0.00
ATOM	966	O	ARG	61	3.170	4.810	-5.724	1.00	0.00
ATOM	967	N	CYX	62	4.563	6.120	-6.915	1.00	0.00
ATOM	968	H	CYX	62	4.779	6.566	-7.795	1.00	0.00
ATOM	969	CA	CYX	62	5.497	6.290	-5.806	1.00	0.00

ATOM	970	HA	CYX	62	5.918	5.325	-5.521	1.00	0.00
ATOM	971	CB	CYX	62	6.643	7.221	-6.222	1.00	0.00
ATOM	972	HB2	CYX	62	6.317	8.258	-6.141	1.00	0.00
ATOM	973	HB3	CYX	62	7.500	7.058	-5.568	1.00	0.00
ATOM	974	SG	CYX	62	7.106	6.895	-7.942	1.00	0.00
ATOM	975	C	CYX	62	4.772	6.880	-4.598	1.00	0.00
ATOM	976	O	CYX	62	4.730	6.277	-3.525	1.00	0.00
ATOM	977	N	GLU	63	4.192	8.062	-4.796	1.00	0.00
ATOM	978	H	GLU	63	4.267	8.513	-5.696	1.00	0.00
ATOM	979	CA	GLU	63	3.451	8.744	-3.740	1.00	0.00
ATOM	980	HA	GLU	63	4.106	8.953	-2.893	1.00	0.00
ATOM	981	CB	GLU	63	2.903	10.070	-4.275	1.00	0.00
ATOM	982	HB2	GLU	63	3.711	10.627	-4.750	1.00	0.00
ATOM	983	HB3	GLU	63	2.122	9.867	-5.007	1.00	0.00
ATOM	984	CG	GLU	63	2.319	10.899	-3.121	1.00	0.00
ATOM	985	HG2	GLU	63	1.598	10.289	-2.576	1.00	0.00
ATOM	986	HG3	GLU	63	3.129	11.189	-2.451	1.00	0.00
ATOM	987	CD	GLU	63	1.625	12.154	-3.655	1.00	0.00
ATOM	988	OE1	GLU	63	1.529	12.297	-4.865	1.00	0.00
ATOM	989	OE2	GLU	63	1.196	12.957	-2.842	1.00	0.00
ATOM	990	C	GLU	63	2.289	7.885	-3.246	1.00	0.00
ATOM	991	O	GLU	63	1.959	7.888	-2.060	1.00	0.00

ATOM	992	N	GLY	64	1.656	7.174	-4.180	1.00	0.00
ATOM	993	H	GLY	64	1.970	7.208	-5.139	1.00	0.00
ATOM	994	CA	GLY	64	0.505	6.334	-3.857	1.00	0.00
ATOM	995	HA2	GLY	64	-0.327	6.979	-3.577	1.00	0.00
ATOM	996	HA3	GLY	64	0.237	5.758	-4.742	1.00	0.00
ATOM	997	C	GLY	64	0.795	5.373	-2.710	1.00	0.00
ATOM	998	O	GLY	64	0.108	5.397	-1.689	1.00	0.00
ATOM	999	N	LEU	65	1.802	4.523	-2.884	1.00	0.00
ATOM	1000	H	LEU	65	2.324	4.511	-3.748	1.00	0.00
ATOM	1001	CA	LEU	65	2.152	3.553	-1.851	1.00	0.00
ATOM	1002	HA	LEU	65	1.316	2.875	-1.678	1.00	0.00
ATOM	1003	CB	LEU	65	3.367	2.729	-2.290	1.00	0.00
ATOM	1004	HB2	LEU	65	4.197	3.399	-2.513	1.00	0.00
ATOM	1005	HB3	LEU	65	3.655	2.050	-1.487	1.00	0.00
ATOM	1006	CG	LEU	65	3.014	1.918	-3.542	1.00	0.00
ATOM	1007	HG	LEU	65	2.785	2.598	-4.362	1.00	0.00
ATOM	1008	CD1	LEU	65	4.201	1.033	-3.930	1.00	0.00
ATOM	1009	HD11	LEU	65	4.431	0.352	-3.110	1.00	0.00
ATOM	1010	HD12	LEU	65	3.950	0.456	-4.820	1.00	0.00
ATOM	1011	HD13	LEU	65	5.070	1.658	-4.136	1.00	0.00
ATOM	1012	CD2	LEU	65	1.791	1.036	-3.254	1.00	0.00
ATOM	1013	HD21	LEU	65	0.945	1.665	-2.979	1.00	0.00

ATOM 1014 HD22 LEU 65 1.541 0.459 -4.144 1.00 0.00

ATOM 1015 HD23 LEU 65 2.019 0.355 -2.433 1.00 0.00

ATOM 1016 C LEU 65 2.447 4.236 -0.520 1.00 0.00

ATOM 1017 O LEU 65 1.972 3.796 0.525 1.00 0.00

ATOM 1018 N ARG 66 3.229 5.313 -0.561 1.00 0.00

ATOM 1019 H ARG 66 3.617 5.640 -1.434 1.00 0.00

ATOM 1020 CA ARG 66 3.567 6.036 0.661 1.00 0.00

ATOM 1021 HA ARG 66 4.112 5.383 1.344 1.00 0.00

ATOM 1022 CB ARG 66 4.451 7.242 0.328 1.00 0.00

ATOM 1023 HB2 ARG 66 3.950 7.865 -0.413 1.00 0.00

ATOM 1024 HB3 ARG 66 4.626 7.824 1.233 1.00 0.00

ATOM 1025 CG ARG 66 5.791 6.758 -0.234 1.00 0.00

ATOM 1026 HG2 ARG 66 6.272 6.099 0.490 1.00 0.00

ATOM 1027 HG3 ARG 66 5.620 6.213 -1.162 1.00 0.00

ATOM 1028 CD ARG 66 6.697 7.962 -0.508 1.00 0.00

ATOM 1029 HD2 ARG 66 7.594 7.637 -1.037 1.00 0.00

ATOM 1030 HD3 ARG 66 6.165 8.695 -1.113 1.00 0.00

ATOM 1031 NE ARG 66 7.097 8.593 0.748 1.00 0.00

ATOM 1032 HE ARG 66 6.636 9.436 1.058 1.00 0.00

ATOM 1033 CZ ARG 66 8.065 8.082 1.508 1.00 0.00

ATOM 1034 NH1 ARG 66 8.685 6.993 1.139 1.00 0.00

ATOM 1035 HH11 ARG 66 8.429 6.538 0.275 1.00 0.00

ATOM	1036	HH12	ARG	66	9.418	6.611	1.720	1.00	0.00
ATOM	1037	NH2	ARG	66	8.395	8.671	2.623	1.00	0.00
ATOM	1038	HH21	ARG	66	7.916	9.513	2.907	1.00	0.00
ATOM	1039	HH22	ARG	66	9.129	8.282	3.198	1.00	0.00
ATOM	1040	C	ARG	66	2.303	6.512	1.373	1.00	0.00
ATOM	1041	O	ARG	66	2.103	6.236	2.557	1.00	0.00
ATOM	1042	N	MET	67	1.454	7.225	0.637	1.00	0.00
ATOM	1043	H	MET	67	1.677	7.445	-0.323	1.00	0.00
ATOM	1044	CA	MET	67	0.205	7.738	1.193	1.00	0.00
ATOM	1045	HA	MET	67	0.409	8.363	2.063	1.00	0.00
ATOM	1046	CB	MET	67	-0.521	8.584	0.141	1.00	0.00
ATOM	1047	HB2	MET	67	-0.011	9.541	0.031	1.00	0.00
ATOM	1048	HB3	MET	67	-0.515	8.058	-0.814	1.00	0.00
ATOM	1049	CG	MET	67	-1.969	8.824	0.583	1.00	0.00
ATOM	1050	HG2	MET	67	-2.525	7.888	0.539	1.00	0.00
ATOM	1051	HG3	MET	67	-1.982	9.205	1.604	1.00	0.00
ATOM	1052	SD	MET	67	-2.751	10.035	-0.515	1.00	0.00
ATOM	1053	CE	MET	67	-1.768	11.473	-0.019	1.00	0.00
ATOM	1054	HE1	MET	67	-0.714	11.280	-0.220	1.00	0.00
ATOM	1055	HE2	MET	67	-2.091	12.347	-0.584	1.00	0.00
ATOM	1056	HE3	MET	67	-1.906	11.658	1.046	1.00	0.00
ATOM	1057	C	MET	67	-0.708	6.607	1.659	1.00	0.00

```
ATOM   1058  O   MET    67      -1.246   6.645   2.766  1.00  0.00
ATOM   1059  N   MET    68      -0.895   5.617   0.793  1.00  0.00
ATOM   1060  H   MET    68      -0.460   5.648  -0.118  1.00  0.00
ATOM   1061  CA  MET    68      -1.767   4.490   1.104  1.00  0.00
ATOM   1062  HA  MET    68      -2.765   4.842   1.362  1.00  0.00
ATOM   1063  CB  MET    68      -1.871   3.578  -0.122  1.00  0.00
ATOM   1064  HB2 MET    68      -2.576   4.011  -0.831  1.00  0.00
ATOM   1065  HB3 MET    68      -0.890   3.495  -0.590  1.00  0.00
ATOM   1066  CG  MET    68      -2.357   2.185   0.295  1.00  0.00
ATOM   1067  HG2 MET    68      -1.566   1.666   0.838  1.00  0.00
ATOM   1068  HG3 MET    68      -3.235   2.277   0.934  1.00  0.00
ATOM   1069  SD  MET    68      -2.792   1.231  -1.178  1.00  0.00
ATOM   1070  CE  MET    68      -1.115   1.035  -1.826  1.00  0.00
ATOM   1071  HE1 MET    68      -0.502   0.507  -1.095  1.00  0.00
ATOM   1072  HE2 MET    68      -1.147   0.463  -2.753  1.00  0.00
ATOM   1073  HE3 MET    68      -0.683   2.016  -2.020  1.00  0.00
ATOM   1074  C   MET    68      -1.264   3.702   2.310  1.00  0.00
ATOM   1075  O   MET    68      -2.040   3.381   3.208  1.00  0.00
ATOM   1076  N   MET    69       0.029   3.389   2.331  1.00  0.00
ATOM   1077  H   MET    69       0.641   3.650   1.571  1.00  0.00
ATOM   1078  CA  MET    69       0.594   2.633   3.444  1.00  0.00
ATOM   1079  HA  MET    69       0.197   1.618   3.447  1.00  0.00
```

ATOM	1080	CB	MET	69	2.119	2.562	3.308	1.00	0.00
ATOM	1081	HB2	MET	69	2.376	2.167	2.325	1.00	0.00
ATOM	1082	HB3	MET	69	2.539	3.562	3.418	1.00	0.00
ATOM	1083	CG	MET	69	2.693	1.646	4.392	1.00	0.00
ATOM	1084	HG2	MET	69	2.389	2.003	5.376	1.00	0.00
ATOM	1085	HG3	MET	69	2.328	0.629	4.247	1.00	0.00
ATOM	1086	SD	MET	69	4.500	1.649	4.290	1.00	0.00
ATOM	1087	CE	MET	69	4.805	0.418	5.581	1.00	0.00
ATOM	1088	HE1	MET	69	4.327	-0.523	5.307	1.00	0.00
ATOM	1089	HE2	MET	69	5.879	0.261	5.689	1.00	0.00
ATOM	1090	HE3	MET	69	4.394	0.772	6.526	1.00	0.00
ATOM	1091	C	MET	69	0.224	3.288	4.772	1.00	0.00
ATOM	1092	O	MET	69	-0.334	2.644	5.661	1.00	0.00
ATOM	1093	N	ARG	70	0.534	4.575	4.890	1.00	0.00
ATOM	1094	H	ARG	70	1.007	5.053	4.137	1.00	0.00
ATOM	1095	CA	ARG	70	0.229	5.326	6.103	1.00	0.00
ATOM	1096	HA	ARG	70	0.706	4.860	6.966	1.00	0.00
ATOM	1097	CB	ARG	70	0.758	6.757	5.965	1.00	0.00
ATOM	1098	HB2	ARG	70	0.319	7.224	5.084	1.00	0.00
ATOM	1099	HB3	ARG	70	0.490	7.331	6.852	1.00	0.00
ATOM	1100	CG	ARG	70	2.283	6.725	5.820	1.00	0.00
ATOM	1101	HG2	ARG	70	2.721	6.252	6.699	1.00	0.00

ATOM 1102 HG3 ARG 70 2.551 6.154 4.931 1.00 0.00
ATOM 1103 CD ARG 70 2.818 8.153 5.690 1.00 0.00
ATOM 1104 HD2 ARG 70 2.250 8.696 4.935 1.00 0.00
ATOM 1105 HD3 ARG 70 2.737 8.669 6.647 1.00 0.00
ATOM 1106 NE ARG 70 4.222 8.128 5.290 1.00 0.00
ATOM 1107 HE ARG 70 4.798 7.330 5.519 1.00 0.00
ATOM 1108 CZ ARG 70 4.774 9.141 4.622 1.00 0.00
ATOM 1109 NH1 ARG 70 4.065 10.196 4.318 1.00 0.00
ATOM 1110 HH11 ARG 70 3.094 10.243 4.591 1.00 0.00
ATOM 1111 HH12 ARG 70 4.492 10.958 3.812 1.00 0.00
ATOM 1112 NH2 ARG 70 6.029 9.080 4.270 1.00 0.00
ATOM 1113 HH21 ARG 70 6.578 8.265 4.506 1.00 0.00
ATOM 1114 HH22 ARG 70 6.447 9.848 3.764 1.00 0.00
ATOM 1115 C ARG 70 -1.277 5.361 6.363 1.00 0.00
ATOM 1116 O ARG 70 -1.723 5.209 7.500 1.00 0.00
ATOM 1117 N MET 71 -2.051 5.570 5.297 1.00 0.00
ATOM 1118 H MET 71 -1.631 5.712 4.390 1.00 0.00
ATOM 1119 CA MET 71 -3.503 5.635 5.405 1.00 0.00
ATOM 1120 HA MET 71 -3.790 6.382 6.145 1.00 0.00
ATOM 1121 CB MET 71 -4.109 6.026 4.054 1.00 0.00
ATOM 1122 HB2 MET 71 -3.593 6.905 3.667 1.00 0.00
ATOM 1123 HB3 MET 71 -3.993 5.199 3.353 1.00 0.00

ATOM	1124	CG	MET	71	-5.598	6.343	4.229	1.00	0.00
ATOM	1125	HG2	MET	71	-6.101	5.497	4.698	1.00	0.00
ATOM	1126	HG3	MET	71	-5.714	7.227	4.856	1.00	0.00
ATOM	1127	SD	MET	71	-6.346	6.659	2.610	1.00	0.00
ATOM	1128	CE	MET	71	-5.409	8.154	2.203	1.00	0.00
ATOM	1129	HE1	MET	71	-4.344	7.921	2.182	1.00	0.00
ATOM	1130	HE2	MET	71	-5.719	8.523	1.226	1.00	0.00
ATOM	1131	HE3	MET	71	-5.598	8.918	2.957	1.00	0.00
ATOM	1132	C	MET	71	-4.074	4.299	5.860	1.00	0.00
ATOM	1133	O	MET	71	-5.022	4.255	6.644	1.00	0.00
ATOM	1134	N	MET	72	-3.501	3.213	5.353	1.00	0.00
ATOM	1135	H	MET	72	-2.739	3.289	4.694	1.00	0.00
ATOM	1136	CA	MET	72	-3.975	1.884	5.709	1.00	0.00
ATOM	1137	HA	MET	72	-5.010	1.759	5.392	1.00	0.00
ATOM	1138	CB	MET	72	-3.119	0.818	5.017	1.00	0.00
ATOM	1139	HB2	MET	72	-2.068	1.099	5.088	1.00	0.00
ATOM	1140	HB3	MET	72	-3.272	-0.142	5.511	1.00	0.00
ATOM	1141	CG	MET	72	-3.518	0.703	3.543	1.00	0.00
ATOM	1142	HG2	MET	72	-3.489	1.687	3.076	1.00	0.00
ATOM	1143	HG3	MET	72	-2.828	0.035	3.026	1.00	0.00
ATOM	1144	SD	MET	72	-5.197	0.035	3.421	1.00	0.00
ATOM	1145	CE	MET	72	-4.853	-1.589	4.146	1.00	0.00

ATOM	1146	HE1	MET	72	-4.477	-1.462	5.162	1.00	0.00
ATOM	1147	HE2	MET	72	-5.769	-2.179	4.169	1.00	0.00
ATOM	1148	HE3	MET	72	-4.104	-2.105	3.545	1.00	0.00
ATOM	1149	C	MET	72	-3.928	1.676	7.217	1.00	0.00
ATOM	1150	O	MET	72	-4.943	1.360	7.841	1.00	0.00
ATOM	1151	N	GLN	73	-2.746	1.862	7.795	1.00	0.00
ATOM	1152	H	GLN	73	-1.936	2.098	7.239	1.00	0.00
ATOM	1153	CA	GLN	73	-2.573	1.701	9.233	1.00	0.00
ATOM	1154	HA	GLN	73	-2.862	0.693	9.533	1.00	0.00
ATOM	1155	CB	GLN	73	-1.107	1.923	9.614	1.00	0.00
ATOM	1156	HB2	GLN	73	-0.813	2.937	9.345	1.00	0.00
ATOM	1157	HB3	GLN	73	-0.988	1.784	10.689	1.00	0.00
ATOM	1158	CG	GLN	73	-0.221	0.921	8.869	1.00	0.00
ATOM	1159	HG2	GLN	73	-0.206	1.138	7.801	1.00	0.00
ATOM	1160	HG3	GLN	73	0.794	0.973	9.264	1.00	0.00
ATOM	1161	CD	GLN	73	-0.749	-0.493	9.071	1.00	0.00
ATOM	1162	OE1	GLN	73	-0.713	-1.020	10.183	1.00	0.00
ATOM	1163	NE2	GLN	73	-1.256	-1.134	8.057	1.00	0.00
ATOM	1164	HE21	GLN	73	-1.608	-2.073	8.182	1.00	0.00
ATOM	1165	HE22	GLN	73	-1.294	-0.690	7.151	1.00	0.00
ATOM	1166	C	GLN	73	-3.458	2.683	9.994	1.00	0.00
ATOM	1167	O	GLN	73	-4.053	2.339	11.011	1.00	0.00

ATOM 1168 N GLN 74 -3.530 3.910 9.491 1.00 0.00
ATOM 1169 H GLN 74 -2.995 4.158 8.671 1.00 0.00
ATOM 1170 CA GLN 74 -4.335 4.944 10.130 1.00 0.00
ATOM 1171 HA GLN 74 -4.025 5.075 11.167 1.00 0.00
ATOM 1172 CB GLN 74 -4.148 6.273 9.391 1.00 0.00
ATOM 1173 HB2 GLN 74 -3.211 6.734 9.704 1.00 0.00
ATOM 1174 HB3 GLN 74 -4.121 6.090 8.317 1.00 0.00
ATOM 1175 CG GLN 74 -5.312 7.210 9.720 1.00 0.00
ATOM 1176 HG2 GLN 74 -5.854 6.847 10.594 1.00 0.00
ATOM 1177 HG3 GLN 74 -4.936 8.214 9.918 1.00 0.00
ATOM 1178 CD GLN 74 -6.275 7.274 8.541 1.00 0.00
ATOM 1179 OE1 GLN 74 -6.305 6.288 7.690 1.00 0.00
ATOM 1180 NE2 GLN 74 -7.020 8.243 8.395 1.00 0.00
ATOM 1181 HE21 GLN 74 -7.654 8.274 7.610 1.00 0.00
ATOM 1182 HE22 GLN 74 -6.993 9.001 9.062 1.00 0.00
ATOM 1183 C GLN 74 -5.815 4.566 10.166 1.00 0.00
ATOM 1184 O GLN 74 -6.485 4.757 11.182 1.00 0.00
ATOM 1185 N LYS 75 -6.320 4.044 9.055 1.00 0.00
ATOM 1186 H LYS 75 -5.741 3.915 8.237 1.00 0.00
ATOM 1187 CA LYS 75 -7.727 3.666 8.974 1.00 0.00
ATOM 1188 HA LYS 75 -8.361 4.537 9.139 1.00 0.00
ATOM 1189 CB LYS 75 -8.027 3.097 7.584 1.00 0.00

ATOM	1190	HB2	LYS	75	-7.695	3.806	6.826	1.00	0.00
ATOM	1191	HB3	LYS	75	-7.493	2.155	7.458	1.00	0.00
ATOM	1192	CG	LYS	75	-9.534	2.855	7.433	1.00	0.00
ATOM	1193	HG2	LYS	75	-9.710	2.198	6.581	1.00	0.00
ATOM	1194	HG3	LYS	75	-9.916	2.384	8.339	1.00	0.00
ATOM	1195	CD	LYS	75	-10.255	4.188	7.207	1.00	0.00
ATOM	1196	HD2	LYS	75	-10.137	4.818	8.089	1.00	0.00
ATOM	1197	HD3	LYS	75	-9.825	4.691	6.341	1.00	0.00
ATOM	1198	CE	LYS	75	-11.743	3.930	6.960	1.00	0.00
ATOM	1199	HE2	LYS	75	-12.132	3.276	7.740	1.00	0.00
ATOM	1200	HE3	LYS	75	-12.283	4.876	6.977	1.00	0.00
ATOM	1201	NZ	LYS	75	-11.922	3.279	5.631	1.00	0.00
ATOM	1202	HZ1	LYS	75	-11.421	2.401	5.616	1.00	0.00
ATOM	1203	HZ2	LYS	75	-12.904	3.109	5.469	1.00	0.00
ATOM	1204	HZ3	LYS	75	-11.561	3.885	4.908	1.00	0.00
ATOM	1205	C	LYS	75	-8.075	2.629	10.038	1.00	0.00
ATOM	1206	O	LYS	75	-9.130	2.703	10.670	1.00	0.00
ATOM	1207	N	GLU	76	-7.181	1.668	10.225	1.00	0.00
ATOM	1208	H	GLU	76	-6.339	1.635	9.667	1.00	0.00
ATOM	1209	CA	GLU	76	-7.398	0.613	11.214	1.00	0.00
ATOM	1210	HA	GLU	76	-7.712	1.047	12.163	1.00	0.00
ATOM	1211	CB	GLU	76	-8.495	-0.343	10.725	1.00	0.00

ATOM	1212	HB2	GLU	76	-9.366	0.235	10.418	1.00	0.00
ATOM	1213	HB3	GLU	76	-8.122	-0.916	9.876	1.00	0.00
ATOM	1214	CG	GLU	76	-8.891	-1.304	11.856	1.00	0.00
ATOM	1215	HG2	GLU	76	-9.555	-2.069	11.453	1.00	0.00
ATOM	1216	HG3	GLU	76	-7.991	-1.776	12.251	1.00	0.00
ATOM	1217	CD	GLU	76	-9.605	-0.550	12.979	1.00	0.00
ATOM	1218	OE1	GLU	76	-10.223	0.466	12.696	1.00	0.00
ATOM	1219	OE2	GLU	76	-9.523	-1.000	14.110	1.00	0.00
ATOM	1220	C	GLU	76	-6.107	-0.163	11.466	1.00	0.00
ATOM	1221	O	GLU	76	-5.950	-1.290	10.992	1.00	0.00
ATOM	1222	N	MET	77	-5.182	0.446	12.201	1.00	0.00
ATOM	1223	H	MET	77	-5.337	1.381	12.549	1.00	0.00
ATOM	1224	CA	MET	77	-3.906	-0.200	12.499	1.00	0.00
ATOM	1225	HA	MET	77	-3.354	-0.381	11.577	1.00	0.00
ATOM	1226	CB	MET	77	-3.061	0.702	13.407	1.00	0.00
ATOM	1227	HB2	MET	77	-2.995	1.697	12.968	1.00	0.00
ATOM	1228	HB3	MET	77	-3.530	0.769	14.389	1.00	0.00
ATOM	1229	CG	MET	77	-1.654	0.115	13.554	1.00	0.00
ATOM	1230	HG2	MET	77	-1.717	-0.904	13.936	1.00	0.00
ATOM	1231	HG3	MET	77	-1.154	0.108	12.586	1.00	0.00
ATOM	1232	SD	MET	77	-0.697	1.125	14.710	1.00	0.00
ATOM	1233	CE	MET	77	0.872	0.239	14.544	1.00	0.00

ATOM	1234	HE1	MET	77	1.205	0.280	13.507	1.00	0.00
ATOM	1235	HE2	MET	77	1.622	0.703	15.185	1.00	0.00
ATOM	1236	HE3	MET	77	0.736	-0.801	14.841	1.00	0.00
ATOM	1237	C	MET	77	-4.124	-1.553	13.176	1.00	0.00
ATOM	1238	O	MET	77	-4.237	-1.629	14.401	1.00	0.00
ATOM	1239	N	GLN	78	-4.168	-2.617	12.377	1.00	0.00
ATOM	1240	H	GLN	78	-4.087	-2.512	11.376	1.00	0.00
ATOM	1241	CA	GLN	78	-4.353	-3.960	12.919	1.00	0.00
ATOM	1242	HA	GLN	78	-5.339	-4.046	13.376	1.00	0.00
ATOM	1243	CB	GLN	78	-4.240	-5.001	11.798	1.00	0.00
ATOM	1244	HB2	GLN	78	-3.816	-4.532	10.910	1.00	0.00
ATOM	1245	HB3	GLN	78	-3.591	-5.815	12.124	1.00	0.00
ATOM	1246	CG	GLN	78	-5.629	-5.557	11.468	1.00	0.00
ATOM	1247	HG2	GLN	78	-6.076	-5.997	12.360	1.00	0.00
ATOM	1248	HG3	GLN	78	-6.271	-4.759	11.097	1.00	0.00
ATOM	1249	CD	GLN	78	-5.520	-6.636	10.393	1.00	0.00
ATOM	1250	OE1	GLN	78	-4.708	-7.554	10.512	1.00	0.00
ATOM	1251	NE2	GLN	78	-6.295	-6.580	9.344	1.00	0.00
ATOM	1252	HE21	GLN	78	-6.226	-7.292	8.631	1.00	0.00
ATOM	1253	HE22	GLN	78	-6.959	-5.826	9.251	1.00	0.00
ATOM	1254	C	GLN	78	-3.294	-4.232	13.988	1.00	0.00
ATOM	1255	O	GLN	78	-2.115	-3.942	13.777	1.00	0.00

ATOM	1256	N	PRO	79	-3.674	-4.772	15.120	1.00	0.00
ATOM	1257	CD	PRO	79	-5.037	-5.167	15.494	1.00	0.00
ATOM	1258	HD2	PRO	79	-5.315	-6.064	14.940	1.00	0.00
ATOM	1259	HD3	PRO	79	-5.725	-4.359	15.247	1.00	0.00
ATOM	1260	CG	PRO	79	-4.943	-5.414	17.008	1.00	0.00
ATOM	1261	HG2	PRO	79	-5.056	-6.481	17.201	1.00	0.00
ATOM	1262	HG3	PRO	79	-5.743	-4.868	17.507	1.00	0.00
ATOM	1263	CB	PRO	79	-3.605	-4.919	17.449	1.00	0.00
ATOM	1264	HB2	PRO	79	-3.247	-5.579	18.240	1.00	0.00
ATOM	1265	HB3	PRO	79	-3.583	-3.888	17.800	1.00	0.00
ATOM	1266	CA	PRO	79	-2.726	-5.066	16.216	1.00	0.00
ATOM	1267	HA	PRO	79	-1.905	-4.351	16.165	1.00	0.00
ATOM	1268	C	PRO	79	-2.173	-6.479	16.111	1.00	0.00
ATOM	1269	O	PRO	79	-2.893	-7.461	16.300	1.00	0.00
ATOM	1270	N	ARG	80	-0.891	-6.561	15.821	1.00	0.00
ATOM	1271	H	ARG	80	-0.356	-5.720	15.657	1.00	0.00
ATOM	1272	CA	ARG	80	-0.215	-7.843	15.699	1.00	0.00
ATOM	1273	HA	ARG	80	-0.338	-8.414	16.620	1.00	0.00
ATOM	1274	CB	ARG	80	-0.802	-8.651	14.540	1.00	0.00
ATOM	1275	HB2	ARG	80	-1.506	-8.033	13.984	1.00	0.00
ATOM	1276	HB3	ARG	80	0.001	-8.974	13.877	1.00	0.00
ATOM	1277	CG	ARG	80	-1.529	-9.879	15.095	1.00	0.00

ATOM	1278	HG2	ARG	80	-0.815	-10.691	15.232	1.00	0.00
ATOM	1279	HG3	ARG	80	-1.982	-9.629	16.055	1.00	0.00
ATOM	1280	CD	ARG	80	-2.618	-10.318	14.117	1.00	0.00
ATOM	1281	HD2	ARG	80	-2.461	-9.842	13.149	1.00	0.00
ATOM	1282	HD3	ARG	80	-2.593	-11.401	13.997	1.00	0.00
ATOM	1283	NE	ARG	80	-3.935	-9.932	14.621	1.00	0.00
ATOM	1284	HE	ARG	80	-4.403	-10.519	15.298	1.00	0.00
ATOM	1285	CZ	ARG	80	-4.546	-8.820	14.212	1.00	0.00
ATOM	1286	NH1	ARG	80	-3.970	-8.028	13.348	1.00	0.00
ATOM	1287	HH11	ARG	80	-3.055	-8.260	12.987	1.00	0.00
ATOM	1288	HH12	ARG	80	-4.441	-7.188	13.045	1.00	0.00
ATOM	1289	NH2	ARG	80	-5.726	-8.521	14.679	1.00	0.00
ATOM	1290	HH21	ARG	80	-6.171	-9.133	15.348	1.00	0.00
ATOM	1291	HH22	ARG	80	-6.189	-7.679	14.370	1.00	0.00
ATOM	1292	C	ARG	80	1.275	-7.624	15.473	1.00	0.00
ATOM	1293	O	ARG	80	1.664	-6.876	14.575	1.00	0.00
ATOM	1294	N	GLY	81	2.107	-8.267	16.285	1.00	0.00
ATOM	1295	H	GLY	81	1.766	-8.877	17.015	1.00	0.00
ATOM	1296	CA	GLY	81	3.546	-8.105	16.139	1.00	0.00
ATOM	1297	HA2	GLY	81	3.749	-7.109	15.747	1.00	0.00
ATOM	1298	HA3	GLY	81	4.012	-8.211	17.119	1.00	0.00
ATOM	1299	C	GLY	81	4.142	-9.143	15.191	1.00	0.00

ATOM 1300 O GLY 81 4.520 -8.820 14.068 1.00 0.00
ATOM 1301 N GLU 82 4.236 -10.383 15.661 1.00 0.00
ATOM 1302 H GLU 82 3.936 -10.590 16.603 1.00 0.00
ATOM 1303 CA GLU 82 4.811 -11.467 14.863 1.00 0.00
ATOM 1304 HA GLU 82 5.818 -11.203 14.539 1.00 0.00
ATOM 1305 CB GLU 82 4.887 -12.744 15.706 1.00 0.00
ATOM 1306 HB2 GLU 82 3.903 -12.962 16.121 1.00 0.00
ATOM 1307 HB3 GLU 82 5.207 -13.574 15.076 1.00 0.00
ATOM 1308 CG GLU 82 5.894 -12.553 16.850 1.00 0.00
ATOM 1309 HG2 GLU 82 6.186 -13.533 17.228 1.00 0.00
ATOM 1310 HG3 GLU 82 6.773 -12.038 16.462 1.00 0.00
ATOM 1311 CD GLU 82 5.280 -11.730 17.986 1.00 0.00
ATOM 1312 OE1 GLU 82 4.067 -11.751 18.131 1.00 0.00
ATOM 1313 OE2 GLU 82 6.036 -11.088 18.697 1.00 0.00
ATOM 1314 C GLU 82 4.020 -11.755 13.585 1.00 0.00
ATOM 1315 O GLU 82 4.608 -11.957 12.522 1.00 0.00
ATOM 1316 N GLN 83 2.694 -11.814 13.695 1.00 0.00
ATOM 1317 H GLN 83 2.245 -11.671 14.588 1.00 0.00
ATOM 1318 CA GLN 83 1.854 -12.127 12.538 1.00 0.00
ATOM 1319 HA GLN 83 2.123 -13.102 12.130 1.00 0.00
ATOM 1320 CB GLN 83 0.382 -12.163 12.965 1.00 0.00
ATOM 1321 HB2 GLN 83 0.272 -12.829 13.821 1.00 0.00

ATOM 1322 HB3 GLN 83 0.063 -11.159 13.244 1.00 0.00
ATOM 1323 CG GLN 83 -0.484 -12.671 11.808 1.00 0.00
ATOM 1324 HG2 GLN 83 -1.537 -12.492 12.024 1.00 0.00
ATOM 1325 HG3 GLN 83 -0.210 -12.158 10.886 1.00 0.00
ATOM 1326 CD GLN 83 -0.274 -14.170 11.613 1.00 0.00
ATOM 1327 OE1 GLN 83 -0.669 -14.968 12.462 1.00 0.00
ATOM 1328 NE2 GLN 83 0.327 -14.601 10.538 1.00 0.00
ATOM 1329 HE21 GLN 83 0.468 -15.593 10.405 1.00 0.00
ATOM 1330 HE22 GLN 83 0.649 -13.942 9.844 1.00 0.00
ATOM 1331 C GLN 83 2.032 -11.114 11.404 1.00 0.00
ATOM 1332 O GLN 83 2.505 -11.460 10.322 1.00 0.00
ATOM 1333 N MET 84 1.640 -9.869 11.659 1.00 0.00
ATOM 1334 H MET 84 1.198 -9.647 12.540 1.00 0.00
ATOM 1335 CA MET 84 1.750 -8.815 10.653 1.00 0.00
ATOM 1336 HA MET 84 1.256 -9.127 9.733 1.00 0.00
ATOM 1337 CB MET 84 1.085 -7.533 11.154 1.00 0.00
ATOM 1338 HB2 MET 84 1.346 -7.371 12.200 1.00 0.00
ATOM 1339 HB3 MET 84 1.428 -6.687 10.559 1.00 0.00
ATOM 1340 CG MET 84 -0.439 -7.667 11.022 1.00 0.00
ATOM 1341 HG2 MET 84 -0.706 -7.833 9.979 1.00 0.00
ATOM 1342 HG3 MET 84 -0.789 -8.506 11.624 1.00 0.00
ATOM 1343 SD MET 84 -1.229 -6.146 11.604 1.00 0.00

ATOM	1344	CE	MET	84	-0.749	-5.084	10.219	1.00	0.00
ATOM	1345	HE1	MET	84	-1.154	-5.490	9.292	1.00	0.00
ATOM	1346	HE2	MET	84	-1.141	-4.080	10.378	1.00	0.00
ATOM	1347	HE3	MET	84	0.338	-5.043	10.152	1.00	0.00
ATOM	1348	C	MET	84	3.199	-8.552	10.245	1.00	0.00
ATOM	1349	O	MET	84	3.457	-8.155	9.110	1.00	0.00
ATOM	1350	N	ARG	85	4.146	-8.767	11.159	1.00	0.00
ATOM	1351	H	ARG	85	3.918	-9.073	12.094	1.00	0.00
ATOM	1352	CA	ARG	85	5.553	-8.535	10.829	1.00	0.00
ATOM	1353	HA	ARG	85	5.728	-7.470	10.680	1.00	0.00
ATOM	1354	CB	ARG	85	6.456	-9.024	11.967	1.00	0.00
ATOM	1355	HB2	ARG	85	6.225	-8.467	12.875	1.00	0.00
ATOM	1356	HB3	ARG	85	6.277	-10.086	12.138	1.00	0.00
ATOM	1357	CG	ARG	85	7.927	-8.808	11.597	1.00	0.00
ATOM	1358	HG2	ARG	85	8.182	-9.426	10.736	1.00	0.00
ATOM	1359	HG3	ARG	85	8.092	-7.759	11.353	1.00	0.00
ATOM	1360	CD	ARG	85	8.810	-9.200	12.784	1.00	0.00
ATOM	1361	HD2	ARG	85	9.849	-8.945	12.575	1.00	0.00
ATOM	1362	HD3	ARG	85	8.481	-8.676	13.682	1.00	0.00
ATOM	1363	NE	ARG	85	8.721	-10.639	13.023	1.00	0.00
ATOM	1364	HE	ARG	85	8.734	-11.279	12.242	1.00	0.00
ATOM	1365	CZ	ARG	85	8.618	-11.141	14.254	1.00	0.00

ATOM 1366 NH1 ARG 85 8.607 -10.351 15.295 1.00 0.00

ATOM 1367 HH11 ARG 85 8.677 -9.352 15.168 1.00 0.00

ATOM 1368 HH12 ARG 85 8.529 -10.744 16.223 1.00 0.00

ATOM 1369 NH2 ARG 85 8.532 -12.432 14.421 1.00 0.00

ATOM 1370 HH21 ARG 85 8.544 -13.044 13.618 1.00 0.00

ATOM 1371 HH22 ARG 85 8.454 -12.814 15.353 1.00 0.00

ATOM 1372 C ARG 85 5.909 -9.265 9.537 1.00 0.00

ATOM 1373 O ARG 85 6.533 -8.694 8.643 1.00 0.00

ATOM 1374 N ARG 86 5.482 -10.521 9.439 1.00 0.00

ATOM 1375 H ARG 86 4.989 -10.956 10.206 1.00 0.00

ATOM 1376 CA ARG 86 5.733 -11.313 8.241 1.00 0.00

ATOM 1377 HA ARG 86 6.805 -11.382 8.055 1.00 0.00

ATOM 1378 CB ARG 86 5.177 -12.727 8.428 1.00 0.00

ATOM 1379 HB2 ARG 86 4.113 -12.673 8.656 1.00 0.00

ATOM 1380 HB3 ARG 86 5.324 -13.300 7.513 1.00 0.00

ATOM 1381 CG ARG 86 5.912 -13.414 9.583 1.00 0.00

ATOM 1382 HG2 ARG 86 6.981 -13.432 9.370 1.00 0.00

ATOM 1383 HG3 ARG 86 5.735 -12.859 10.504 1.00 0.00

ATOM 1384 CD ARG 86 5.401 -14.846 9.746 1.00 0.00

ATOM 1385 HD2 ARG 86 4.313 -14.848 9.800 1.00 0.00

ATOM 1386 HD3 ARG 86 5.724 -15.454 8.901 1.00 0.00

ATOM 1387 NE ARG 86 5.931 -15.433 10.973 1.00 0.00

ATOM 1388 HE ARG 86 6.659 -14.957 11.487 1.00 0.00
ATOM 1389 CZ ARG 86 5.475 -16.592 11.443 1.00 0.00
ATOM 1390 NH1 ARG 86 4.538 -17.239 10.803 1.00 0.00
ATOM 1391 HH11 ARG 86 4.159 -16.858 9.948 1.00 0.00
ATOM 1392 HH12 ARG 86 4.198 -18.118 11.166 1.00 0.00
ATOM 1393 NH2 ARG 86 5.968 -17.085 12.545 1.00 0.00
ATOM 1394 HH21 ARG 86 6.695 -16.585 13.038 1.00 0.00
ATOM 1395 HH22 ARG 86 5.622 -17.965 12.901 1.00 0.00
ATOM 1396 C ARG 86 5.077 -10.650 7.031 1.00 0.00
ATOM 1397 O ARG 86 5.631 -10.642 5.931 1.00 0.00
ATOM 1398 N MET 87 3.885 -10.094 7.259 1.00 0.00
ATOM 1399 H MET 87 3.477 -10.146 8.182 1.00 0.00
ATOM 1400 CA MET 87 3.130 -9.421 6.204 1.00 0.00
ATOM 1401 HA MET 87 2.854 -10.136 5.429 1.00 0.00
ATOM 1402 CB MET 87 1.850 -8.809 6.784 1.00 0.00
ATOM 1403 HB2 MET 87 1.327 -9.561 7.375 1.00 0.00
ATOM 1404 HB3 MET 87 2.111 -7.964 7.421 1.00 0.00
ATOM 1405 CG MET 87 0.942 -8.331 5.646 1.00 0.00
ATOM 1406 HG2 MET 87 1.550 -7.919 4.841 1.00 0.00
ATOM 1407 HG3 MET 87 0.358 -9.170 5.267 1.00 0.00
ATOM 1408 SD MET 87 -0.183 -7.050 6.259 1.00 0.00
ATOM 1409 CE MET 87 -1.218 -8.105 7.302 1.00 0.00

ATOM 1410 HE1 MET 87 -0.601 -8.578 8.067 1.00 0.00

ATOM 1411 HE2 MET 87 -1.988 -7.500 7.781 1.00 0.00

ATOM 1412 HE3 MET 87 -1.688 -8.874 6.689 1.00 0.00

ATOM 1413 C MET 87 3.955 -8.316 5.549 1.00 0.00

ATOM 1414 O MET 87 3.766 -8.013 4.373 1.00 0.00

ATOM 1415 N MET 88 4.853 -7.700 6.318 1.00 0.00

ATOM 1416 H MET 88 4.976 -7.969 7.284 1.00 0.00

ATOM 1417 CA MET 88 5.673 -6.612 5.793 1.00 0.00

ATOM 1418 HA MET 88 5.067 -5.720 5.639 1.00 0.00

ATOM 1419 CB MET 88 6.779 -6.284 6.796 1.00 0.00

ATOM 1420 HB2 MET 88 7.331 -7.193 7.037 1.00 0.00

ATOM 1421 HB3 MET 88 7.459 -5.553 6.359 1.00 0.00

ATOM 1422 CG MET 88 6.162 -5.707 8.076 1.00 0.00

ATOM 1423 HG2 MET 88 5.862 -4.673 7.906 1.00 0.00

ATOM 1424 HG3 MET 88 5.290 -6.296 8.362 1.00 0.00

ATOM 1425 SD MET 88 7.381 -5.761 9.414 1.00 0.00

ATOM 1426 CE MET 88 8.810 -5.211 8.452 1.00 0.00

ATOM 1427 HE1 MET 88 8.615 -4.219 8.045 1.00 0.00

ATOM 1428 HE2 MET 88 9.689 -5.172 9.096 1.00 0.00

ATOM 1429 HE3 MET 88 8.990 -5.910 7.635 1.00 0.00

ATOM 1430 C MET 88 6.299 -6.999 4.458 1.00 0.00

ATOM 1431 O MET 88 6.160 -6.279 3.469 1.00 0.00

```
ATOM   1432  N   ARG    89       6.970  -8.146   4.429  1.00  0.00
ATOM   1433  H   ARG    89       7.081  -8.702   5.265  1.00  0.00
ATOM   1434  CA  ARG    89       7.590  -8.619   3.198  1.00  0.00
ATOM   1435  HA  ARG    89       8.314  -7.887   2.839  1.00  0.00
ATOM   1436  CB  ARG    89       8.318  -9.943   3.452  1.00  0.00
ATOM   1437  HB2 ARG    89       7.613 -10.674   3.848  1.00  0.00
ATOM   1438  HB3 ARG    89       8.736 -10.312   2.516  1.00  0.00
ATOM   1439  CG  ARG    89       9.449  -9.724   4.465  1.00  0.00
ATOM   1440  HG2 ARG    89       9.057  -9.202   5.338  1.00  0.00
ATOM   1441  HG3 ARG    89       9.853 -10.689   4.771  1.00  0.00
ATOM   1442  CD  ARG    89      10.560  -8.885   3.825  1.00  0.00
ATOM   1443  HD2 ARG    89      10.911  -9.370   2.914  1.00  0.00
ATOM   1444  HD3 ARG    89      10.181  -7.892   3.585  1.00  0.00
ATOM   1445  NE  ARG    89      11.685  -8.746   4.744  1.00  0.00
ATOM   1446  HE  ARG    89      12.495  -9.342   4.646  1.00  0.00
ATOM   1447  CZ  ARG    89      11.676  -7.842   5.722  1.00  0.00
ATOM   1448  NH1 ARG    89      10.639  -7.065   5.889  1.00  0.00
ATOM   1449 HH11 ARG    89       9.843  -7.152   5.274  1.00  0.00
ATOM   1450 HH12 ARG    89      10.639  -6.382   6.633  1.00  0.00
ATOM   1451  NH2 ARG    89      12.704  -7.734   6.516  1.00  0.00
ATOM   1452 HH21 ARG    89      13.504  -8.337   6.387  1.00  0.00
ATOM   1453 HH22 ARG    89      12.696  -7.048   7.258  1.00  0.00
```

```
ATOM   1454  C   ARG    89       6.529  -8.806   2.116  1.00  0.00
ATOM   1455  O   ARG    89       6.721  -8.412   0.965  1.00  0.00
ATOM   1456  N   LEU    90       5.403  -9.401   2.507  1.00  0.00
ATOM   1457  H   LEU    90       5.305  -9.717   3.461  1.00  0.00
ATOM   1458  CA  LEU    90       4.299  -9.634   1.584  1.00  0.00
ATOM   1459  HA  LEU    90       4.633 -10.235   0.738  1.00  0.00
ATOM   1460  CB  LEU    90       3.180 -10.390   2.310  1.00  0.00
ATOM   1461  HB2 LEU    90       3.597 -11.267   2.806  1.00  0.00
ATOM   1462  HB3 LEU    90       2.735  -9.730   3.055  1.00  0.00
ATOM   1463  CG  LEU    90       2.104 -10.831   1.309  1.00  0.00
ATOM   1464  HG  LEU    90       2.562 -11.005   0.336  1.00  0.00
ATOM   1465  CD1 LEU    90       1.448 -12.124   1.802  1.00  0.00
ATOM   1466 HD11 LEU    90       0.990 -11.951   2.776  1.00  0.00
ATOM   1467 HD12 LEU    90       0.684 -12.438   1.092  1.00  0.00
ATOM   1468 HD13 LEU    90       2.204 -12.905   1.891  1.00  0.00
ATOM   1469  CD2 LEU    90       1.034  -9.739   1.190  1.00  0.00
ATOM   1470 HD21 LEU    90       1.495  -8.815   0.843  1.00  0.00
ATOM   1471 HD22 LEU    90       0.271 -10.054   0.479  1.00  0.00
ATOM   1472 HD23 LEU    90       0.575  -9.572   2.165  1.00  0.00
ATOM   1473  C   LEU    90       3.764  -8.311   1.046  1.00  0.00
ATOM   1474  O   LEU    90       3.439  -8.195  -0.136  1.00  0.00
ATOM   1475  N   ALA    91       3.669  -7.320   1.929  1.00  0.00
```

ATOM	1476	H	ALA	91	3.930	-7.470	2.894	1.00	0.00
ATOM	1477	CA	ALA	91	3.164	-6.008	1.545	1.00	0.00
ATOM	1478	HA	ALA	91	2.124	-6.083	1.228	1.00	0.00
ATOM	1479	CB	ALA	91	3.245	-5.048	2.735	1.00	0.00
ATOM	1480	HB1	ALA	91	4.282	-4.956	3.059	1.00	0.00
ATOM	1481	HB2	ALA	91	2.870	-4.069	2.439	1.00	0.00
ATOM	1482	HB3	ALA	91	2.642	-5.435	3.557	1.00	0.00
ATOM	1483	C	ALA	91	3.966	-5.449	0.378	1.00	0.00
ATOM	1484	O	ALA	91	3.399	-5.046	-0.631	1.00	0.00
ATOM	1485	N	GLU	92	5.288	-5.437	0.526	1.00	0.00
ATOM	1486	H	GLU	92	5.701	-5.758	1.390	1.00	0.00
ATOM	1487	CA	GLU	92	6.176	-4.937	-0.523	1.00	0.00
ATOM	1488	HA	GLU	92	5.941	-3.898	-0.752	1.00	0.00
ATOM	1489	CB	GLU	92	7.630	-5.016	-0.049	1.00	0.00
ATOM	1490	HB2	GLU	92	7.723	-4.514	0.914	1.00	0.00
ATOM	1491	HB3	GLU	92	7.919	-6.062	0.058	1.00	0.00
ATOM	1492	CG	GLU	92	8.549	-4.334	-1.072	1.00	0.00
ATOM	1493	HG2	GLU	92	8.397	-4.797	-2.047	1.00	0.00
ATOM	1494	HG3	GLU	92	8.290	-3.277	-1.127	1.00	0.00
ATOM	1495	CD	GLU	92	10.016	-4.478	-0.663	1.00	0.00
ATOM	1496	OE1	GLU	92	10.279	-5.114	0.348	1.00	0.00
ATOM	1497	OE2	GLU	92	10.858	-3.950	-1.369	1.00	0.00

ATOM	1498	C	GLU	92	6.008	-5.739	-1.813	1.00	0.00
ATOM	1499	O	GLU	92	6.201	-5.212	-2.908	1.00	0.00
ATOM	1500	N	ASN	93	5.664	-7.017	-1.674	1.00	0.00
ATOM	1501	H	ASN	93	5.539	-7.416	-0.754	1.00	0.00
ATOM	1502	CA	ASN	93	5.492	-7.888	-2.837	1.00	0.00
ATOM	1503	HA	ASN	93	6.354	-7.802	-3.499	1.00	0.00
ATOM	1504	CB	ASN	93	5.365	-9.347	-2.385	1.00	0.00
ATOM	1505	HB2	ASN	93	4.543	-9.445	-1.676	1.00	0.00
ATOM	1506	HB3	ASN	93	5.180	-9.989	-3.246	1.00	0.00
ATOM	1507	CG	ASN	93	6.654	-9.805	-1.703	1.00	0.00
ATOM	1508	OD1	ASN	93	7.669	-8.989	-1.615	1.00	0.00
ATOM	1509	ND2	ASN	93	6.736	-10.942	-1.238	1.00	0.00
ATOM	1510	HD21	ASN	93	7.590	-11.240	-0.788	1.00	0.00
ATOM	1511	HD22	ASN	93	5.949	-11.571	-1.308	1.00	0.00
ATOM	1512	C	ASN	93	4.257	-7.498	-3.650	1.00	0.00
ATOM	1513	O	ASN	93	4.258	-7.611	-4.876	1.00	0.00
ATOM	1514	N	ILE	94	3.205	-7.046	-2.967	1.00	0.00
ATOM	1515	H	ILE	94	3.227	-7.004	-1.958	1.00	0.00
ATOM	1516	CA	ILE	94	1.973	-6.654	-3.651	1.00	0.00
ATOM	1517	HA	ILE	94	1.524	-7.533	-4.114	1.00	0.00
ATOM	1518	CB	ILE	94	0.985	-6.044	-2.644	1.00	0.00
ATOM	1519	HB	ILE	94	1.533	-5.416	-1.941	1.00	0.00

ATOM 1520 CG2 ILE 94 -0.063 -5.197 -3.377 1.00 0.00
ATOM 1521 HG21 ILE 94 -0.611 -5.825 -4.079 1.00 0.00
ATOM 1522 HG22 ILE 94 -0.757 -4.771 -2.653 1.00 0.00
ATOM 1523 HG23 ILE 94 0.434 -4.394 -3.920 1.00 0.00
ATOM 1524 CG1 ILE 94 0.281 -7.175 -1.882 1.00 0.00
ATOM 1525 HG12 ILE 94 -0.507 -7.596 -2.506 1.00 0.00
ATOM 1526 HG13 ILE 94 1.006 -7.953 -1.638 1.00 0.00
ATOM 1527 CD1 ILE 94 -0.331 -6.625 -0.590 1.00 0.00
ATOM 1528 HD11 ILE 94 -1.055 -5.848 -0.832 1.00 0.00
ATOM 1529 HD12 ILE 94 -0.830 -7.431 -0.052 1.00 0.00
ATOM 1530 HD13 ILE 94 0.457 -6.205 0.036 1.00 0.00
ATOM 1531 C ILE 94 2.269 -5.663 -4.785 1.00 0.00
ATOM 1532 O ILE 94 1.916 -5.913 -5.937 1.00 0.00
ATOM 1533 N PRO 95 2.909 -4.558 -4.489 1.00 0.00
ATOM 1534 CD PRO 95 3.365 -4.162 -3.148 1.00 0.00
ATOM 1535 HD2 PRO 95 3.936 -4.981 -2.710 1.00 0.00
ATOM 1536 HD3 PRO 95 2.496 -3.952 -2.524 1.00 0.00
ATOM 1537 CG PRO 95 4.217 -2.921 -3.396 1.00 0.00
ATOM 1538 HG2 PRO 95 5.267 -3.210 -3.457 1.00 0.00
ATOM 1539 HG3 PRO 95 4.082 -2.219 -2.573 1.00 0.00
ATOM 1540 CB PRO 95 3.738 -2.336 -4.678 1.00 0.00
ATOM 1541 HB2 PRO 95 4.568 -1.842 -5.182 1.00 0.00

```
ATOM   1542  HB3 PRO    95       2.923  -1.627  -4.539  1.00  0.00
ATOM   1543  CA  PRO    95       3.258  -3.527  -5.511  1.00  0.00
ATOM   1544  HA  PRO    95       2.355  -3.299  -6.077  1.00  0.00
ATOM   1545  C   PRO    95       4.356  -3.991  -6.470  1.00  0.00
ATOM   1546  O   PRO    95       4.451  -3.496  -7.589  1.00  0.00
ATOM   1547  N   SER    96       5.175  -4.951  -6.030  1.00  0.00
ATOM   1548  H   SER    96       5.069  -5.325  -5.098  1.00  0.00
ATOM   1549  CA  SER    96       6.250  -5.477  -6.870  1.00  0.00
ATOM   1550  HA  SER    96       6.911  -4.665  -7.173  1.00  0.00
ATOM   1551  CB  SER    96       7.072  -6.517  -6.106  1.00  0.00
ATOM   1552  HB2 SER    96       6.431  -7.352  -5.822  1.00  0.00
ATOM   1553  HB3 SER    96       7.881  -6.880  -6.740  1.00  0.00
ATOM   1554  OG  SER    96       7.617  -5.921  -4.938  1.00  0.00
ATOM   1555  HG  SER    96       8.133  -6.573  -4.457  1.00  0.00
ATOM   1556  C   SER    96       5.637  -6.107  -8.093  1.00  0.00
ATOM   1557  O   SER    96       6.113  -5.957  -9.219  1.00  0.00
ATOM   1558  N   ARG    97       4.556  -6.806  -7.824  1.00  0.00
ATOM   1559  H   ARG    97       4.255  -6.885  -6.863  1.00  0.00
ATOM   1560  CA  ARG    97       3.794  -7.489  -8.836  1.00  0.00
ATOM   1561  HA  ARG    97       4.386  -8.270  -9.314  1.00  0.00
ATOM   1562  CB  ARG    97       2.595  -8.136  -8.155  1.00  0.00
ATOM   1563  HB2 ARG    97       2.136  -7.420  -7.474  1.00  0.00
```

ATOM	1564	HB3	ARG	97	1.869	-8.435	-8.910	1.00	0.00
ATOM	1565	CG	ARG	97	3.050	-9.370	-7.367	1.00	0.00
ATOM	1566	HG2	ARG	97	3.589	-10.045	-8.032	1.00	0.00
ATOM	1567	HG3	ARG	97	3.709	-9.058	-6.556	1.00	0.00
ATOM	1568	CD	ARG	97	1.833	-10.093	-6.785	1.00	0.00
ATOM	1569	HD2	ARG	97	1.194	-10.450	-7.593	1.00	0.00
ATOM	1570	HD3	ARG	97	2.160	-10.939	-6.180	1.00	0.00
ATOM	1571	NE	ARG	97	1.058	-9.186	-5.941	1.00	0.00
ATOM	1572	HE	ARG	97	1.325	-9.046	-4.977	1.00	0.00
ATOM	1573	CZ	ARG	97	-0.003	-8.530	-6.409	1.00	0.00
ATOM	1574	NH1	ARG	97	-0.389	-8.691	-7.647	1.00	0.00
ATOM	1575	HH11	ARG	97	0.121	-9.317	-8.254	1.00	0.00
ATOM	1576	HH12	ARG	97	-1.194	-8.189	-7.991	1.00	0.00
ATOM	1577	NH2	ARG	97	-0.660	-7.721	-5.626	1.00	0.00
ATOM	1578	HH21	ARG	97	-0.361	-7.596	-4.669	1.00	0.00
ATOM	1579	HH22	ARG	97	-1.464	-7.223	-5.979	1.00	0.00
ATOM	1580	C	ARG	97	3.329	-6.532	-9.933	1.00	0.00
ATOM	1581	O	ARG	97	3.273	-6.906	-11.105	1.00	0.00
ATOM	1582	N	CYX	98	3.006	-5.297	-9.550	1.00	0.00
ATOM	1583	H	CYX	98	3.070	-5.029	-8.578	1.00	0.00
ATOM	1584	CA	CYX	98	2.559	-4.302	-10.512	1.00	0.00
ATOM	1585	HA	CYX	98	2.244	-4.800	-11.429	1.00	0.00

```
ATOM   1586  CB  CYX    98       1.383  -3.495  -9.965  1.00  0.00
ATOM   1587  HB2 CYX    98       1.749  -2.558  -9.545  1.00  0.00
ATOM   1588  HB3 CYX    98       0.682  -3.282 -10.771  1.00  0.00
ATOM   1589  SG  CYX    98       0.554  -4.429  -8.652  1.00  0.00
ATOM   1590  C   CYX    98       3.679  -3.343 -10.826  1.00  0.00
ATOM   1591  O   CYX    98       3.562  -2.527 -11.740  1.00  0.00
ATOM   1592  N   ASN    99       4.752  -3.409 -10.044  1.00  0.00
ATOM   1593  H   ASN    99       4.801  -4.040  -9.256  1.00  0.00
ATOM   1594  CA  ASN    99       5.847  -2.492 -10.255  1.00  0.00
ATOM   1595  HA  ASN    99       5.466  -1.478 -10.135  1.00  0.00
ATOM   1596  CB  ASN    99       7.004  -2.687  -9.306  1.00  0.00
ATOM   1597  HB2 ASN    99       7.139  -3.744  -9.077  1.00  0.00
ATOM   1598  HB3 ASN    99       7.919  -2.292  -9.748  1.00  0.00
ATOM   1599  CG  ASN    99       6.728  -1.939  -8.007  1.00  0.00
ATOM   1600  OD1 ASN    99       7.024  -2.436  -6.921  1.00  0.00
ATOM   1601  ND2 ASN    99       6.178  -0.755  -8.062  1.00  0.00
ATOM   1602 HD21 ASN    99       5.990  -0.249  -7.208  1.00  0.00
ATOM   1603 HD22 ASN    99       5.944  -0.352  -8.957  1.00  0.00
ATOM   1604  C   ASN    99       6.343  -2.553 -11.666  1.00  0.00
ATOM   1605  O   ASN    99       6.171  -3.539 -12.386  1.00  0.00
ATOM   1606  N   LEU   100       6.872  -1.428 -12.061  1.00  0.00
ATOM   1607  H   LEU   100       6.974  -0.669 -11.402  1.00  0.00
```

ATOM	1608	CA	LEU	100	7.319	-1.223	-13.413	1.00	0.00
ATOM	1609	HA	LEU	100	7.647	-2.153	-13.877	1.00	0.00
ATOM	1610	CB	LEU	100	6.109	-0.688	-14.188	1.00	0.00
ATOM	1611	HB2	LEU	100	6.425	0.182	-14.763	1.00	0.00
ATOM	1612	HB3	LEU	100	5.710	-1.445	-14.863	1.00	0.00
ATOM	1613	CG	LEU	100	5.009	-0.267	-13.175	1.00	0.00
ATOM	1614	HG	LEU	100	5.010	-0.960	-12.333	1.00	0.00
ATOM	1615	CD1	LEU	100	5.284	1.149	-12.669	1.00	0.00
ATOM	1616	HD11	LEU	100	5.283	1.842	-13.510	1.00	0.00
ATOM	1617	HD12	LEU	100	4.510	1.438	-11.959	1.00	0.00
ATOM	1618	HD13	LEU	100	6.256	1.177	-12.176	1.00	0.00
ATOM	1619	CD2	LEU	100	3.638	-0.334	-13.831	1.00	0.00
ATOM	1620	HD21	LEU	100	3.444	-1.354	-14.165	1.00	0.00
ATOM	1621	HD22	LEU	100	2.876	-0.037	-13.111	1.00	0.00
ATOM	1622	HD23	LEU	100	3.611	0.340	-14.687	1.00	0.00
ATOM	1623	C	LEU	100	8.468	-0.220	-13.443	1.00	0.00
ATOM	1624	O	LEU	100	8.442	0.774	-12.720	1.00	0.00
ATOM	1625	N	SER	101	9.472	-0.501	-14.277	1.00	0.00
ATOM	1626	H	SER	101	9.438	-1.345	-14.831	1.00	0.00
ATOM	1627	CA	SER	101	10.648	0.365	-14.402	1.00	0.00
ATOM	1628	HA	SER	101	11.385	0.112	-13.639	1.00	0.00
ATOM	1629	CB	SER	101	11.285	0.173	-15.780	1.00	0.00

```
ATOM   1630  HB2 SER   101      11.572  -0.871 -15.907  1.00  0.00
ATOM   1631  HB3 SER   101      10.570   0.450 -16.554  1.00  0.00
ATOM   1632  OG  SER   101      12.439   0.997 -15.884  1.00  0.00
ATOM   1633  HG  SER   101      12.841   0.878 -16.748  1.00  0.00
ATOM   1634  C   SER   101      10.271   1.834 -14.214  1.00  0.00
ATOM   1635  O   SER   101       9.822   2.498 -15.149  1.00  0.00
ATOM   1636  N   PRO   102      10.442   2.343 -13.019  1.00  0.00
ATOM   1637  CD  PRO   102      10.956   1.637 -11.849  1.00  0.00
ATOM   1638  HD2 PRO   102      12.031   1.795 -11.755  1.00  0.00
ATOM   1639  HD3 PRO   102      10.753   0.569 -11.934  1.00  0.00
ATOM   1640  CG  PRO   102      10.169   2.289 -10.724  1.00  0.00
ATOM   1641  HG2 PRO   102      10.752   2.241  -9.804  1.00  0.00
ATOM   1642  HG3 PRO   102       9.229   1.754 -10.586  1.00  0.00
ATOM   1643  CB  PRO   102       9.921   3.711 -11.144  1.00  0.00
ATOM   1644  HB2 PRO   102      10.664   4.343 -10.657  1.00  0.00
ATOM   1645  HB3 PRO   102       8.922   4.063 -10.889  1.00  0.00
ATOM   1646  CA  PRO   102      10.119   3.751 -12.673  1.00  0.00
ATOM   1647  HA  PRO   102       9.208   4.033 -13.200  1.00  0.00
ATOM   1648  C   PRO   102      11.246   4.703 -13.061  1.00  0.00
ATOM   1649  O   PRO   102      12.364   4.591 -12.554  1.00  0.00
ATOM   1650  N   MET   103      10.950   5.635 -13.963  1.00  0.00
ATOM   1651  H   MET   103      10.027   5.686 -14.370  1.00  0.00
```

ATOM 1652 CA MET 103 11.955 6.592 -14.407 1.00 0.00

ATOM 1653 HA MET 103 12.860 6.068 -14.715 1.00 0.00

ATOM 1654 CB MET 103 11.422 7.393 -15.600 1.00 0.00

ATOM 1655 HB2 MET 103 10.496 7.891 -15.315 1.00 0.00

ATOM 1656 HB3 MET 103 12.161 8.139 -15.892 1.00 0.00

ATOM 1657 CG MET 103 11.153 6.451 -16.779 1.00 0.00

ATOM 1658 HG2 MET 103 10.366 5.746 -16.511 1.00 0.00

ATOM 1659 HG3 MET 103 10.840 7.031 -17.646 1.00 0.00

ATOM 1660 SD MET 103 12.660 5.534 -17.189 1.00 0.00

ATOM 1661 CE MET 103 13.676 6.951 -17.676 1.00 0.00

ATOM 1662 HE1 MET 103 13.769 7.638 -16.835 1.00 0.00

ATOM 1663 HE2 MET 103 14.666 6.604 -17.972 1.00 0.00

ATOM 1664 HE3 MET 103 13.206 7.464 -18.514 1.00 0.00

ATOM 1665 C MET 103 12.323 7.545 -13.275 1.00 0.00

ATOM 1666 O MET 103 13.489 7.637 -12.886 1.00 0.00

ATOM 1667 N ARG 104 11.322 8.245 -12.744 1.00 0.00

ATOM 1668 H ARG 104 10.383 8.152 -13.103 1.00 0.00

ATOM 1669 CA ARG 104 11.554 9.180 -11.647 1.00 0.00

ATOM 1670 HA ARG 104 12.375 8.829 -11.022 1.00 0.00

ATOM 1671 CB ARG 104 11.921 10.560 -12.209 1.00 0.00

ATOM 1672 HB2 ARG 104 11.621 10.612 -13.255 1.00 0.00

ATOM 1673 HB3 ARG 104 11.397 11.329 -11.642 1.00 0.00

ATOM	1674	CG	ARG	104	13.435	10.786	-12.099	1.00	0.00
ATOM	1675	HG2	ARG	104	13.957	9.970	-12.600	1.00	0.00
ATOM	1676	HG3	ARG	104	13.693	11.730	-12.578	1.00	0.00
ATOM	1677	CD	ARG	104	13.854	10.831	-10.624	1.00	0.00
ATOM	1678	HD2	ARG	104	14.029	9.818	-10.261	1.00	0.00
ATOM	1679	HD3	ARG	104	14.768	11.416	-10.519	1.00	0.00
ATOM	1680	NE	ARG	104	12.808	11.446	-9.810	1.00	0.00
ATOM	1681	HE	ARG	104	12.206	10.866	-9.243	1.00	0.00
ATOM	1682	CZ	ARG	104	12.620	12.764	-9.793	1.00	0.00
ATOM	1683	NH1	ARG	104	13.383	13.546	-10.509	1.00	0.00
ATOM	1684	HH11	ARG	104	14.118	13.148	-11.076	1.00	0.00
ATOM	1685	HH12	ARG	104	13.235	14.545	-10.491	1.00	0.00
ATOM	1686	NH2	ARG	104	11.673	13.275	-9.058	1.00	0.00
ATOM	1687	HH21	ARG	104	11.085	12.668	-8.504	1.00	0.00
ATOM	1688	HH22	ARG	104	11.532	14.275	-9.046	1.00	0.00
ATOM	1689	C	ARG	104	10.316	9.309	-10.761	1.00	0.00
ATOM	1690	O	ARG	104	9.191	9.366	-11.257	1.00	0.00
ATOM	1691	N	CYX	105	10.541	9.386	-9.450	1.00	0.00
ATOM	1692	H	CYX	105	11.483	9.318	-9.091	1.00	0.00
ATOM	1693	CA	CYX	105	9.447	9.546	-8.496	1.00	0.00
ATOM	1694	HA	CYX	105	8.494	9.374	-8.995	1.00	0.00
ATOM	1695	CB	CYX	105	9.586	8.542	-7.349	1.00	0.00

ATOM	1696	HB2	CYX	105	10.622	8.518	-7.010	1.00	0.00
ATOM	1697	HB3	CYX	105	8.941	8.841	-6.523	1.00	0.00
ATOM	1698	SG	CYX	105	9.127	6.888	-7.929	1.00	0.00
ATOM	1699	C	CYX	105	9.453	10.970	-7.938	1.00	0.00
ATOM	1700	O	CYX	105	10.362	11.342	-7.196	1.00	0.00
ATOM	1701	N	PRO	106	8.470	11.770	-8.280	1.00	0.00
ATOM	1702	CD	PRO	106	7.352	11.423	-9.169	1.00	0.00
ATOM	1703	HD2	PRO	106	6.660	10.770	-8.636	1.00	0.00
ATOM	1704	HD3	PRO	106	7.739	10.903	-10.045	1.00	0.00
ATOM	1705	CG	PRO	106	6.731	12.776	-9.520	1.00	0.00
ATOM	1706	HG2	PRO	106	5.652	12.657	-9.616	1.00	0.00
ATOM	1707	HG3	PRO	106	7.144	13.121	-10.468	1.00	0.00
ATOM	1708	CB	PRO	106	7.080	13.716	-8.413	1.00	0.00
ATOM	1709	HB2	PRO	106	6.286	13.719	-7.666	1.00	0.00
ATOM	1710	HB3	PRO	106	7.235	14.730	-8.782	1.00	0.00
ATOM	1711	CA	PRO	106	8.373	13.183	-7.795	1.00	0.00
ATOM	1712	HA	PRO	106	9.245	13.719	-8.169	1.00	0.00
ATOM	1713	C	PRO	106	8.320	13.297	-6.277	1.00	0.00
ATOM	1714	O	PRO	106	8.143	14.387	-5.736	1.00	0.00
ATOM	1715	N	MET	107	8.474	12.174	-5.599	1.00	0.00
ATOM	1716	H	MET	107	8.632	11.301	-6.082	1.00	0.00
ATOM	1717	CA	MET	107	8.442	12.161	-4.148	1.00	0.00

ATOM	1718	HA	MET	107	7.615	12.767	-3.778	1.00	0.00
ATOM	1719	CB	MET	107	8.250	10.743	-3.673	1.00	0.00
ATOM	1720	HB2	MET	107	8.856	10.077	-4.287	1.00	0.00
ATOM	1721	HB3	MET	107	8.571	10.669	-2.634	1.00	0.00
ATOM	1722	CG	MET	107	6.775	10.338	-3.782	1.00	0.00
ATOM	1723	HG2	MET	107	6.696	9.404	-4.339	1.00	0.00
ATOM	1724	HG3	MET	107	6.363	10.200	-2.782	1.00	0.00
ATOM	1725	SD	MET	107	5.834	11.627	-4.643	1.00	0.00
ATOM	1726	CE	MET	107	5.811	10.853	-6.277	1.00	0.00
ATOM	1727	HE1	MET	107	5.316	9.884	-6.214	1.00	0.00
ATOM	1728	HE2	MET	107	5.271	11.492	-6.975	1.00	0.00
ATOM	1729	HE3	MET	107	6.834	10.715	-6.628	1.00	0.00
ATOM	1730	C	MET	107	9.749	12.683	-3.597	1.00	0.00
ATOM	1731	O	MET	107	10.698	11.930	-3.370	1.00	0.00
ATOM	1732	N	GLY	108	9.783	13.979	-3.390	1.00	0.00
ATOM	1733	H	GLY	108	8.975	14.549	-3.595	1.00	0.00
ATOM	1734	CA	GLY	108	10.966	14.628	-2.867	1.00	0.00
ATOM	1735	HA2	GLY	108	10.702	15.624	-2.513	1.00	0.00
ATOM	1736	HA3	GLY	108	11.361	14.041	-2.037	1.00	0.00
ATOM	1737	C	GLY	108	12.040	14.752	-3.942	1.00	0.00
ATOM	1738	O	GLY	108	13.232	14.808	-3.638	1.00	0.00
ATOM	1739	N	GLY	109	11.603	14.801	-5.199	1.00	0.00

ATOM	1740	H	GLY	109	10.614	14.750	-5.396	1.00	0.00
ATOM	1741	CA	GLY	109	12.528	14.928	-6.322	1.00	0.00
ATOM	1742	HA2	GLY	109	11.973	14.819	-7.253	1.00	0.00
ATOM	1743	HA3	GLY	109	12.994	15.913	-6.289	1.00	0.00
ATOM	1744	C	GLY	109	13.619	13.861	-6.267	1.00	0.00
ATOM	1745	O	GLY	109	14.790	14.173	-6.046	1.00	0.00
ATOM	1746	N	SER	110	13.228	12.603	-6.471	1.00	0.00
ATOM	1747	H	SER	110	12.254	12.388	-6.627	1.00	0.00
ATOM	1748	CA	SER	110	14.186	11.498	-6.444	1.00	0.00
ATOM	1749	HA	SER	110	14.616	11.401	-5.447	1.00	0.00
ATOM	1750	CB	SER	110	13.486	10.188	-6.803	1.00	0.00
ATOM	1751	HB2	SER	110	13.122	10.238	-7.829	1.00	0.00
ATOM	1752	HB3	SER	110	14.189	9.360	-6.707	1.00	0.00
ATOM	1753	OG	SER	110	12.390	9.983	-5.920	1.00	0.00
ATOM	1754	HG	SER	110	11.949	9.160	-6.143	1.00	0.00
ATOM	1755	C	SER	110	15.329	11.759	-7.423	1.00	0.00
ATOM	1756	O	SER	110	15.172	12.510	-8.386	1.00	0.00
ATOM	1757	N	ILE	111	16.480	11.142	-7.168	1.00	0.00
ATOM	1758	H	ILE	111	16.578	10.553	-6.353	1.00	0.00
ATOM	1759	CA	ILE	111	17.643	11.326	-8.033	1.00	0.00
ATOM	1760	HA	ILE	111	17.553	12.275	-8.561	1.00	0.00
ATOM	1761	CB	ILE	111	18.925	11.330	-7.192	1.00	0.00

ATOM	1762	HB	ILE	111	19.021	10.376	-6.672	1.00	0.00
ATOM	1763	CG2	ILE	111	20.139	11.538	-8.105	1.00	0.00
ATOM	1764	HG21	ILE	111	20.045	12.491	-8.624	1.00	0.00
ATOM	1765	HG22	ILE	111	21.049	11.540	-7.505	1.00	0.00
ATOM	1766	HG23	ILE	111	20.188	10.730	-8.835	1.00	0.00
ATOM	1767	CG1	ILE	111	18.856	12.468	-6.165	1.00	0.00
ATOM	1768	HG12	ILE	111	18.885	13.426	-6.684	1.00	0.00
ATOM	1769	HG13	ILE	111	17.927	12.389	-5.600	1.00	0.00
ATOM	1770	CD1	ILE	111	20.046	12.371	-5.206	1.00	0.00
ATOM	1771	HD11	ILE	111	20.976	12.449	-5.770	1.00	0.00
ATOM	1772	HD12	ILE	111	19.994	13.180	-4.478	1.00	0.00
ATOM	1773	HD13	ILE	111	20.018	11.413	-4.686	1.00	0.00
ATOM	1774	C	ILE	111	17.712	10.221	-9.085	1.00	0.00
ATOM	1775	O	ILE	111	17.320	10.421	-10.236	1.00	0.00
ATOM	1776	N	ALA	112	18.187	9.048	-8.671	1.00	0.00
ATOM	1777	H	ALA	112	18.488	8.936	-7.713	1.00	0.00
ATOM	1778	CA	ALA	112	18.278	7.904	-9.559	1.00	0.00
ATOM	1779	HA	ALA	112	18.767	8.150	-10.501	1.00	0.00
ATOM	1780	CB	ALA	112	19.055	6.776	-8.887	1.00	0.00
ATOM	1781	HB1	ALA	112	18.572	6.512	-7.946	1.00	0.00
ATOM	1782	HB2	ALA	112	19.073	5.905	-9.542	1.00	0.00
ATOM	1783	HB3	ALA	112	20.076	7.103	-8.691	1.00	0.00

ATOM	1784	C	ALA	112	16.887	7.428	-9.868	1.00	0.00
ATOM	1785	O	ALA	112	15.911	8.106	-9.542	1.00	0.00
ATOM	1786	N	GLY	113	16.773	6.244	-10.441	1.00	0.00
ATOM	1787	H	GLY	113	17.562	5.675	-10.714	1.00	0.00
ATOM	1788	CA	GLY	113	15.454	5.740	-10.687	1.00	0.00
ATOM	1789	HA2	GLY	113	14.800	6.525	-11.064	1.00	0.00
ATOM	1790	HA3	GLY	113	15.478	4.914	-11.398	1.00	0.00
ATOM	1791	C	GLY	113	14.909	5.237	-9.366	1.00	0.00
ATOM	1792	O	GLY	113	15.198	4.118	-8.940	1.00	0.00
ATOM	1793	N	PHE	114	14.149	6.104	-8.714	1.00	0.00
ATOM	1794	H	PHE	114	13.974	7.013	-9.117	1.00	0.00
ATOM	1795	CA	PHE	114	13.572	5.804	-7.408	1.00	0.00
ATOM	1796	HA	PHE	114	14.319	5.942	-6.626	1.00	0.00
ATOM	1797	CB	PHE	114	12.397	6.745	-7.133	1.00	0.00
ATOM	1798	HB2	PHE	114	12.685	7.794	-7.203	1.00	0.00
ATOM	1799	HB3	PHE	114	11.556	6.554	-7.799	1.00	0.00
ATOM	1800	CG	PHE	114	11.891	6.529	-5.724	1.00	0.00
ATOM	1801	CD1	PHE	114	12.499	7.197	-4.653	1.00	0.00
ATOM	1802	HD1	PHE	114	13.337	7.870	-4.834	1.00	0.00
ATOM	1803	CE1	PHE	114	12.030	6.999	-3.349	1.00	0.00
ATOM	1804	HE1	PHE	114	12.503	7.519	-2.516	1.00	0.00
ATOM	1805	CZ	PHE	114	10.956	6.134	-3.114	1.00	0.00

```
ATOM   1806  HZ  PHE   114      10.593   5.980  -2.098  1.00  0.00
ATOM   1807  CE2 PHE   114      10.348   5.466  -4.184  1.00  0.00
ATOM   1808  HE2 PHE   114       9.512   4.792  -4.001  1.00  0.00
ATOM   1809  CD2 PHE   114      10.815   5.664  -5.489  1.00  0.00
ATOM   1810  HD2 PHE   114      10.341   5.145  -6.322  1.00  0.00
ATOM   1811  C   PHE   114      13.092   4.353  -7.335  1.00  0.00
ATOM   1812  O   PHE   114      13.493   3.664  -6.412  1.00  0.00
ATOM   1813  OXT PHE   114      12.330   3.955  -8.202  1.00  0.00
TER
ATOM   1814  S1  LIG   115      16.890 -18.302  -2.528  1.00  0.00
ATOM   1815  O1  LIG   115      18.914 -22.094  -0.901  1.00  0.00
ATOM   1816  O2  LIG   115      15.495 -17.709  -3.149  1.00  0.00
ATOM   1817  O3  LIG   115      17.365 -17.901  -1.022  1.00  0.00
ATOM   1818  N1  LIG   115      16.519 -20.122  -2.600  1.00  0.00
ATOM   1819  H1  LIG   115      15.512 -20.217  -2.695  1.00  0.00
ATOM   1820  N2  LIG   115      18.552 -20.934  -1.711  1.00  0.00
ATOM   1821  N3  LIG   115      21.298 -17.259  -6.461  1.00  0.00
ATOM   1822  H10 LIG   115      21.101 -16.855  -7.360  1.00  0.00
ATOM   1823  H11 LIG   115      22.257 -17.470  -6.241  1.00  0.00
ATOM   1824  C1  LIG   115      18.261 -18.021  -3.736  1.00  0.00
ATOM   1825  C2  LIG   115      17.926 -17.461  -4.968  1.00  0.00
ATOM   1826  H2  LIG   115      16.893 -17.224  -5.193  1.00  0.00
```

```
ATOM   1827  C3  LIG   115      19.571 -18.335  -3.377  1.00  0.00
ATOM   1828  H3  LIG   115      19.788 -18.794  -2.420  1.00  0.00
ATOM   1829  C4  LIG   115      17.230 -21.052  -1.865  1.00  0.00
ATOM   1830  C5  LIG   115      20.290 -17.512  -5.559  1.00  0.00
ATOM   1831  C6  LIG   115      18.945 -17.206  -5.880  1.00  0.00
ATOM   1832  H4  LIG   115      18.709 -16.767  -6.843  1.00  0.00
ATOM   1833  C7  LIG   115      20.584 -18.081  -4.295  1.00  0.00
ATOM   1834  H5  LIG   115      21.611 -18.324  -4.042  1.00  0.00
ATOM   1835  C8  LIG   115      16.699 -22.224  -1.225  1.00  0.00
ATOM   1836  H6  LIG   115      15.669 -22.539  -1.202  1.00  0.00
ATOM   1837  C9  LIG   115      17.775 -22.838  -0.652  1.00  0.00
ATOM   1838  C10 LIG   115      17.945 -24.073   0.158  1.00  0.00
ATOM   1839  H7  LIG   115      16.983 -24.571   0.299  1.00  0.00
ATOM   1840  H8  LIG   115      18.632 -24.771  -0.334  1.00  0.00
ATOM   1841  H9  LIG   115      18.364 -23.837   1.142  1.00  0.00
TER
ATOM   1842  S1  LIG   116     -20.788  19.378   3.383  1.00  0.00
ATOM   1843  O1  LIG   116     -19.212  22.496   6.368  1.00  0.00
ATOM   1844  O2  LIG   116     -22.137  18.613   2.857  1.00  0.00
ATOM   1845  O3  LIG   116     -20.054  20.500   2.457  1.00  0.00
ATOM   1846  N1  LIG   116     -21.458  20.179   4.920  1.00  0.00
ATOM   1847  H1  LIG   116     -22.471  20.184   4.836  1.00  0.00
```

ATOM	1848	N2	LIG	116	-19.485	21.255	5.649	1.00	0.00
ATOM	1849	N3	LIG	116	-16.839	15.225	5.158	1.00	0.00
ATOM	1850	H10	LIG	116	-17.086	14.250	5.169	1.00	0.00
ATOM	1851	H11	LIG	116	-15.907	15.476	5.442	1.00	0.00
ATOM	1852	C1	LIG	116	-19.564	18.120	3.965	1.00	0.00
ATOM	1853	C2	LIG	116	-19.966	16.785	3.963	1.00	0.00
ATOM	1854	H2	LIG	116	-20.967	16.520	3.643	1.00	0.00
ATOM	1855	C3	LIG	116	-18.287	18.527	4.349	1.00	0.00
ATOM	1856	H3	LIG	116	-18.022	19.576	4.364	1.00	0.00
ATOM	1857	C4	LIG	116	-20.817	21.232	5.545	1.00	0.00
ATOM	1858	C5	LIG	116	-17.743	16.184	4.763	1.00	0.00
ATOM	1859	C6	LIG	116	-19.051	15.816	4.362	1.00	0.00
ATOM	1860	H4	LIG	116	-19.340	14.770	4.364	1.00	0.00
ATOM	1861	C7	LIG	116	-17.379	17.552	4.751	1.00	0.00
ATOM	1862	H5	LIG	116	-16.382	17.845	5.062	1.00	0.00
ATOM	1863	C8	LIG	116	-21.441	22.367	6.167	1.00	0.00
ATOM	1864	H6	LIG	116	-22.497	22.580	6.216	1.00	0.00
ATOM	1865	C9	LIG	116	-20.414	23.113	6.666	1.00	0.00
ATOM	1866	C10	LIG	116	-20.340	24.397	7.411	1.00	0.00
ATOM	1867	H7	LIG	116	-21.343	24.797	7.581	1.00	0.00
ATOM	1868	H8	LIG	116	-19.850	24.259	8.381	1.00	0.00
ATOM	1869	H9	LIG	116	-19.759	25.139	6.852	1.00	0.00

```
TER
ATOM   1870  S1  LIG   117      14.622  25.305   0.646  1.00  0.00
ATOM   1871  O1  LIG   117      18.776  27.268   0.770  1.00  0.00
ATOM   1872  O2  LIG   117      13.133  25.403  -0.027  1.00  0.00
ATOM   1873  O3  LIG   117      15.652  24.118   0.215  1.00  0.00
ATOM   1874  N1  LIG   117      15.314  26.949   0.122  1.00  0.00
ATOM   1875  H1  LIG   117      14.738  27.283  -0.646  1.00  0.00
ATOM   1876  N2  LIG   117      17.448  26.771   1.119  1.00  0.00
ATOM   1877  N3  LIG   117      14.013  25.487   6.611  1.00  0.00
ATOM   1878  H10 LIG   117      13.113  25.637   7.033  1.00  0.00
ATOM   1879  H11 LIG   117      14.802  25.358   7.222  1.00  0.00
ATOM   1880  C1  LIG   117      14.466  25.391   2.485  1.00  0.00
ATOM   1881  C2  LIG   117      13.192  25.604   3.012  1.00  0.00
ATOM   1882  H2  LIG   117      12.345  25.729   2.348  1.00  0.00
ATOM   1883  C3  LIG   117      15.594  25.207   3.283  1.00  0.00
ATOM   1884  H3  LIG   117      16.571  25.070   2.836  1.00  0.00
ATOM   1885  C4  LIG   117      16.669  27.219   0.130  1.00  0.00
ATOM   1886  C5  LIG   117      14.162  25.456   5.243  1.00  0.00
ATOM   1887  C6  LIG   117      13.043  25.635   4.395  1.00  0.00
ATOM   1888  H4  LIG   117      12.060  25.796   4.827  1.00  0.00
ATOM   1889  C7  LIG   117      15.437  25.243   4.664  1.00  0.00
ATOM   1890  H5  LIG   117      16.302  25.110   5.306  1.00  0.00
```

```
ATOM   1891  C8  LIG   117      17.394  27.995  -0.839  1.00  0.00
ATOM   1892  H6  LIG   117      16.996  28.460  -1.726  1.00  0.00
ATOM   1893  C9  LIG   117      18.685  28.003  -0.398  1.00  0.00
ATOM   1894  C10 LIG   117      19.945  28.603  -0.911  1.00  0.00
ATOM   1895  H7  LIG   117      19.756  29.150  -1.838  1.00  0.00
ATOM   1896  H8  LIG   117      20.375  29.295  -0.179  1.00  0.00
ATOM   1897  H9  LIG   117      20.692  27.827  -1.109  1.00  0.00
TER
ATOM   1898  S1  LIG   118      22.218 -10.003  21.044  1.00  0.00
ATOM   1899  O1  LIG   118      22.069  -5.554  22.191  1.00  0.00
ATOM   1900  O2  LIG   118      23.062 -11.392  21.239  1.00  0.00
ATOM   1901  O3  LIG   118      20.763  -9.808  21.751  1.00  0.00
ATOM   1902  N1  LIG   118      23.429  -8.792  21.767  1.00  0.00
ATOM   1903  H1  LIG   118      24.084  -9.327  22.330  1.00  0.00
ATOM   1904  N2  LIG   118      22.180  -6.811  21.454  1.00  0.00
ATOM   1905  N3  LIG   118      21.863  -8.822  15.172  1.00  0.00
ATOM   1906  H10 LIG   118      22.344  -9.396  14.501  1.00  0.00
ATOM   1907  H11 LIG   118      21.314  -8.054  14.825  1.00  0.00
ATOM   1908  C1  LIG   118      22.120  -9.599  19.242  1.00  0.00
ATOM   1909  C2  LIG   118      22.811 -10.428  18.360  1.00  0.00
ATOM   1910  H2  LIG   118      23.390 -11.262  18.741  1.00  0.00
ATOM   1911  C3  LIG   118      21.345  -8.520  18.819  1.00  0.00
```

```
ATOM   1912  H3  LIG   118      20.845  -7.882  19.537  1.00  0.00
ATOM   1913  C4  LIG   118      23.057  -7.523  22.169  1.00  0.00
ATOM   1914  C5  LIG   118      21.949  -9.080  16.522  1.00  0.00
ATOM   1915  C6  LIG   118      22.723 -10.167  16.997  1.00  0.00
ATOM   1916  H4  LIG   118      23.249 -10.802  16.291  1.00  0.00
ATOM   1917  C7  LIG   118      21.265  -8.263  17.454  1.00  0.00
ATOM   1918  H5  LIG   118      20.674  -7.424  17.100  1.00  0.00
ATOM   1919  C8  LIG   118      23.545  -6.811  23.318  1.00  0.00
ATOM   1920  H6  LIG   118      24.252  -7.168  24.049  1.00  0.00
ATOM   1921  C9  LIG   118      22.914  -5.602  23.285  1.00  0.00
ATOM   1922  C10 LIG   118      22.946  -4.399  24.158  1.00  0.00
ATOM   1923  H7  LIG   118      23.637  -4.552  24.991  1.00  0.00
ATOM   1924  H8  LIG   118      23.268  -3.516  23.596  1.00  0.00
ATOM   1925  H9  LIG   118      21.951  -4.187  24.564  1.00  0.00
TER
ATOM   1926  S1  LIG   119     -23.009  18.754 -22.550  1.00  0.00
ATOM   1927  O1  LIG   119     -18.734  17.090 -22.274  1.00  0.00
ATOM   1928  O2  LIG   119     -23.933  19.919 -23.235  1.00  0.00
ATOM   1929  O3  LIG   119     -23.140  17.202 -23.029  1.00  0.00
ATOM   1930  N1  LIG   119     -21.320  19.398 -22.980  1.00  0.00
ATOM   1931  H1  LIG   119     -21.439  20.066 -23.737  1.00  0.00
ATOM   1932  N2  LIG   119     -20.024  17.701 -21.967  1.00  0.00
```

```
ATOM   1933  N3  LIG   119     -23.570  19.161 -16.591  1.00  0.00
ATOM   1934  H10 LIG   119     -24.102  19.905 -16.173  1.00  0.00
ATOM   1935  H11 LIG   119     -23.149  18.484 -15.978  1.00  0.00
ATOM   1936  C1  LIG   119     -23.140  18.877 -20.711  1.00  0.00
ATOM   1937  C2  LIG   119     -23.886  19.932 -20.189  1.00  0.00
ATOM   1938  H2  LIG   119     -24.349  20.651 -20.856  1.00  0.00
ATOM   1939  C3  LIG   119     -22.532  17.911 -19.910  1.00  0.00
ATOM   1940  H3  LIG   119     -21.938  17.120 -20.351  1.00  0.00
ATOM   1941  C4  LIG   119     -20.192  18.600 -22.942  1.00  0.00
ATOM   1942  C5  LIG   119     -23.428  19.068 -17.957  1.00  0.00
ATOM   1943  C6  LIG   119     -24.030  20.026 -18.809  1.00  0.00
ATOM   1944  H4  LIG   119     -24.610  20.836 -18.381  1.00  0.00
ATOM   1945  C7  LIG   119     -22.677  18.013 -18.530  1.00  0.00
ATOM   1946  H5  LIG   119     -22.207  17.277 -17.886  1.00  0.00
ATOM   1947  C8  LIG   119     -19.087  18.635 -23.860  1.00  0.00
ATOM   1948  H6  LIG   119     -18.984  19.267 -24.727  1.00  0.00
ATOM   1949  C9  LIG   119     -18.211  17.693 -23.404  1.00  0.00
ATOM   1950  C10 LIG   119     -16.886  17.206 -23.870  1.00  0.00
ATOM   1951  H7  LIG   119     -16.578  17.744 -24.770  1.00  0.00
ATOM   1952  H8  LIG   119     -16.123  17.351 -23.098  1.00  0.00
ATOM   1953  H9  LIG   119     -16.924  16.136 -24.099  1.00  0.00
TER
```

```
ATOM   1954  S1  LIG   120      -5.268   4.240 -19.785  1.00  0.00
ATOM   1955  O1  LIG   120      -7.237   8.361 -20.293  1.00  0.00
ATOM   1956  O2  LIG   120      -4.676   3.183 -18.683  1.00  0.00
ATOM   1957  O3  LIG   120      -6.668   3.935 -20.560  1.00  0.00
ATOM   1958  N1  LIG   120      -5.467   5.741 -18.708  1.00  0.00
ATOM   1959  H1  LIG   120      -5.460   5.419 -17.744  1.00  0.00
ATOM   1960  N2  LIG   120      -6.308   7.236 -20.333  1.00  0.00
ATOM   1961  N3  LIG   120      -0.921   5.484 -23.729  1.00  0.00
ATOM   1962  H10 LIG   120       0.007   5.120 -23.604  1.00  0.00
ATOM   1963  H11 LIG   120      -1.098   6.052 -24.540  1.00  0.00
ATOM   1964  C1  LIG   120      -3.937   4.665 -20.996  1.00  0.00
ATOM   1965  C2  LIG   120      -2.669   4.133 -20.768  1.00  0.00
ATOM   1966  H2  LIG   120      -2.489   3.512 -19.899  1.00  0.00
ATOM   1967  C3  LIG   120      -4.239   5.458 -22.102  1.00  0.00
ATOM   1968  H3  LIG   120      -5.231   5.875 -22.230  1.00  0.00
ATOM   1969  C4  LIG   120      -6.267   6.809 -19.068  1.00  0.00
ATOM   1970  C5  LIG   120      -1.920   5.212 -22.822  1.00  0.00
ATOM   1971  C6  LIG   120      -1.660   4.408 -21.685  1.00  0.00
ATOM   1972  H4  LIG   120      -0.666   4.002 -21.531  1.00  0.00
ATOM   1973  C7  LIG   120      -3.224   5.732 -23.012  1.00  0.00
ATOM   1974  H5  LIG   120      -3.433   6.354 -23.877  1.00  0.00
ATOM   1975  C8  LIG   120      -7.095   7.592 -18.192  1.00  0.00
```

```
ATOM   1976  H6  LIG   120      -7.236   7.449 -17.133  1.00  0.00
ATOM   1977  C9  LIG   120      -7.666   8.540 -18.990  1.00  0.00
ATOM   1978  C10 LIG   120      -8.620   9.654 -18.746  1.00  0.00
ATOM   1979  H7  LIG   120      -8.896   9.694 -17.690  1.00  0.00
ATOM   1980  H8  LIG   120      -8.178  10.616 -19.029  1.00  0.00
ATOM   1981  H9  LIG   120      -9.530   9.523 -19.341  1.00  0.00
TER
ATOM   1982  S1  LIG   121     -15.675 -26.578 -18.687  1.00  0.00
ATOM   1983  O1  LIG   121     -13.076 -28.135 -15.231  1.00  0.00
ATOM   1984  O2  LIG   121     -15.747 -25.476 -19.896  1.00  0.00
ATOM   1985  O3  LIG   121     -15.456 -28.159 -19.016  1.00  0.00
ATOM   1986  N1  LIG   121     -14.206 -25.927 -17.753  1.00  0.00
ATOM   1987  H1  LIG   121     -13.702 -25.309 -18.384  1.00  0.00
ATOM   1988  N2  LIG   121     -14.147 -27.532 -16.020  1.00  0.00
ATOM   1989  N3  LIG   121     -20.465 -25.820 -15.156  1.00  0.00
ATOM   1990  H10 LIG   121     -21.107 -25.063 -15.314  1.00  0.00
ATOM   1991  H11 LIG   121     -20.660 -26.450 -14.396  1.00  0.00
ATOM   1992  C1  LIG   121     -17.126 -26.346 -17.565  1.00  0.00
ATOM   1993  C2  LIG   121     -17.978 -25.276 -17.832  1.00  0.00
ATOM   1994  H2  LIG   121     -17.769 -24.609 -18.660  1.00  0.00
ATOM   1995  C3  LIG   121     -17.345 -27.249 -16.526  1.00  0.00
ATOM   1996  H3  LIG   121     -16.645 -28.052 -16.334  1.00  0.00
```

```
ATOM   1997  C4  LIG   121     -13.506 -26.698 -16.844  1.00  0.00
ATOM   1998  C5  LIG   121     -19.358 -25.993 -15.955  1.00  0.00
ATOM   1999  C6  LIG   121     -19.097 -25.101 -17.024  1.00  0.00
ATOM   2000  H4  LIG   121     -19.777 -24.278 -17.216  1.00  0.00
ATOM   2001  C7  LIG   121     -18.464 -27.066 -15.720  1.00  0.00
ATOM   2002  H5  LIG   121     -18.652 -27.751 -14.900  1.00  0.00
ATOM   2003  C8  LIG   121     -12.083 -26.697 -16.637  1.00  0.00
ATOM   2004  H6  LIG   121     -11.351 -26.116 -17.173  1.00  0.00
ATOM   2005  C9  LIG   121     -11.868 -27.591 -15.629  1.00  0.00
ATOM   2006  C10 LIG   121     -10.651 -28.078 -14.927  1.00  0.00
ATOM   2007  H7  LIG   121      -9.759 -27.590 -15.328  1.00  0.00
ATOM   2008  H8  LIG   121     -10.710 -27.871 -13.853  1.00  0.00
ATOM   2009  H9  LIG   121     -10.540 -29.161 -15.048  1.00  0.00
TER
ATOM   2010  S1  LIG   122      -8.609 -10.453 -18.697  1.00  0.00
ATOM   2011  O1  LIG   122      -6.408  -6.925 -20.655  1.00  0.00
ATOM   2012  O2  LIG   122      -9.043 -11.108 -17.261  1.00  0.00
ATOM   2013  O3  LIG   122      -9.721  -9.758 -19.664  1.00  0.00
ATOM   2014  N1  LIG   122      -7.407  -9.164 -18.106  1.00  0.00
ATOM   2015  H1  LIG   122      -7.586  -9.022 -17.116  1.00  0.00
ATOM   2016  N2  LIG   122      -6.800  -8.256 -20.201  1.00  0.00
ATOM   2017  N3  LIG   122      -5.437 -14.529 -21.749  1.00  0.00
```

```
ATOM   2018  H10 LIG   122      -5.194 -15.411 -21.332  1.00  0.00
ATOM   2019  H11 LIG   122      -5.141 -14.361 -22.696  1.00  0.00
ATOM   2020  C1  LIG   122      -7.602 -11.679 -19.646  1.00  0.00
ATOM   2021  C2  LIG   122      -7.293 -12.882 -19.014  1.00  0.00
ATOM   2022  H2  LIG   122      -7.627 -13.063 -17.998  1.00  0.00
ATOM   2023  C3  LIG   122      -7.218 -11.390 -20.955  1.00  0.00
ATOM   2024  H3  LIG   122      -7.454 -10.433 -21.403  1.00  0.00
ATOM   2025  C4  LIG   122      -7.021  -8.096 -18.893  1.00  0.00
ATOM   2026  C5  LIG   122      -6.154 -13.585 -21.051  1.00  0.00
ATOM   2027  C6  LIG   122      -6.568 -13.837 -19.720  1.00  0.00
ATOM   2028  H4  LIG   122      -6.320 -14.782 -19.250  1.00  0.00
ATOM   2029  C7  LIG   122      -6.489 -12.348 -21.653  1.00  0.00
ATOM   2030  H5  LIG   122      -6.172 -12.143 -22.671  1.00  0.00
ATOM   2031  C8  LIG   122      -6.781  -6.749 -18.451  1.00  0.00
ATOM   2032  H6  LIG   122      -6.891  -6.366 -17.450  1.00  0.00
ATOM   2033  C9  LIG   122      -6.399  -6.067 -19.570  1.00  0.00
ATOM   2034  C10 LIG   122      -6.013  -4.656 -19.834  1.00  0.00
ATOM   2035  H7  LIG   122      -6.050  -4.072 -18.911  1.00  0.00
ATOM   2036  H8  LIG   122      -4.998  -4.599 -20.244  1.00  0.00
ATOM   2037  H9  LIG   122      -6.689  -4.199 -20.565  1.00  0.00
TER
ATOM   2038  S1  LIG   123      -5.631  17.028  17.908  1.00  0.00
```

ATOM	2039	O1	LIG	123	-2.085	18.932	20.127	1.00	0.00
ATOM	2040	O2	LIG	123	-6.174	16.563	16.435	1.00	0.00
ATOM	2041	O3	LIG	123	-5.200	15.921	19.023	1.00	0.00
ATOM	2042	N1	LIG	123	-4.139	18.011	17.398	1.00	0.00
ATOM	2043	H1	LIG	123	-3.912	17.736	16.446	1.00	0.00
ATOM	2044	N2	LIG	123	-3.398	18.670	19.543	1.00	0.00
ATOM	2045	N3	LIG	123	-9.571	20.911	20.230	1.00	0.00
ATOM	2046	H10	LIG	123	-10.362	21.225	19.694	1.00	0.00
ATOM	2047	H11	LIG	123	-9.472	21.260	21.168	1.00	0.00
ATOM	2048	C1	LIG	123	-6.813	18.250	18.634	1.00	0.00
ATOM	2049	C2	LIG	123	-7.888	18.652	17.842	1.00	0.00
ATOM	2050	H2	LIG	123	-7.996	18.262	16.836	1.00	0.00
ATOM	2051	C3	LIG	123	-6.624	18.701	19.939	1.00	0.00
ATOM	2052	H3	LIG	123	-5.761	18.387	20.514	1.00	0.00
ATOM	2053	C4	LIG	123	-3.123	18.326	18.281	1.00	0.00
ATOM	2054	C5	LIG	123	-8.657	20.029	19.699	1.00	0.00
ATOM	2055	C6	LIG	123	-8.812	19.543	18.378	1.00	0.00
ATOM	2056	H4	LIG	123	-9.658	19.866	17.782	1.00	0.00
ATOM	2057	C7	LIG	123	-7.550	19.595	20.467	1.00	0.00
ATOM	2058	H5	LIG	123	-7.419	19.966	21.479	1.00	0.00
ATOM	2059	C8	LIG	123	-1.716	18.363	17.990	1.00	0.00
ATOM	2060	H6	LIG	123	-1.242	18.126	17.052	1.00	0.00

```
ATOM   2061  C9  LIG   123      -1.119  18.748  19.155  1.00  0.00
ATOM   2062  C10 LIG   123       0.292  18.979  19.562  1.00  0.00
ATOM   2063  H7  LIG   123       0.966  18.796  18.721  1.00  0.00
ATOM   2064  H8  LIG   123       0.436  20.009  19.907  1.00  0.00
ATOM   2065  H9  LIG   123       0.573  18.313  20.385  1.00  0.00
TER
ATOM   2066  S1  LIG   124     -20.923  -3.296   0.955  1.00  0.00
ATOM   2067  O1  LIG   124     -19.123   0.571  -0.757  1.00  0.00
ATOM   2068  O2  LIG   124     -21.621  -3.956   2.281  1.00  0.00
ATOM   2069  O3  LIG   124     -21.803  -2.988  -0.381  1.00  0.00
ATOM   2070  N1  LIG   124     -20.315  -1.697   1.682  1.00  0.00
ATOM   2071  H1  LIG   124     -20.828  -1.549   2.547  1.00  0.00
ATOM   2072  N2  LIG   124     -19.310  -0.787  -0.253  1.00  0.00
ATOM   2073  N3  LIG   124     -16.071  -6.539  -0.436  1.00  0.00
ATOM   2074  H10 LIG   124     -15.771  -7.296   0.154  1.00  0.00
ATOM   2075  H11 LIG   124     -15.540  -6.354  -1.269  1.00  0.00
ATOM   2076  C1  LIG   124     -19.406  -4.259   0.522  1.00  0.00
ATOM   2077  C2  LIG   124     -19.036  -5.294   1.381  1.00  0.00
ATOM   2078  H2  LIG   124     -19.621  -5.496   2.270  1.00  0.00
ATOM   2079  C3  LIG   124     -18.706  -3.957  -0.645  1.00  0.00
ATOM   2080  H3  LIG   124     -19.009  -3.128  -1.273  1.00  0.00
ATOM   2081  C4  LIG   124     -19.966  -0.613   0.898  1.00  0.00
```

ATOM	2082	C5	LIG	124	-17.176	-5.784	-0.117	1.00	0.00
ATOM	2083	C6	LIG	124	-17.919	-6.057	1.058	1.00	0.00
ATOM	2084	H4	LIG	124	-17.615	-6.870	1.709	1.00	0.00
ATOM	2085	C7	LIG	124	-17.588	-4.722	-0.959	1.00	0.00
ATOM	2086	H5	LIG	124	-17.023	-4.501	-1.859	1.00	0.00
ATOM	2087	C8	LIG	124	-20.207	0.772	1.195	1.00	0.00
ATOM	2088	H6	LIG	124	-20.717	1.170	2.057	1.00	0.00
ATOM	2089	C9	LIG	124	-19.664	1.463	0.151	1.00	0.00
ATOM	2090	C10	LIG	124	-19.562	2.907	-0.186	1.00	0.00
ATOM	2091	H7	LIG	124	-20.029	3.514	0.594	1.00	0.00
ATOM	2092	H8	LIG	124	-18.515	3.212	-0.287	1.00	0.00
ATOM	2093	H9	LIG	124	-20.060	3.119	-1.139	1.00	0.00

TER

END

Table I: The number of atoms present in the system of BN-Dic

ATOM	1	N	GLN	1	-7.077	-12.405	1.432	1.00	0.00
ATOM	2	H1	GLN	1	-6.146	-12.736	1.641	1.00	0.00
ATOM	3	H2	GLN	1	-7.308	-11.659	2.072	1.00	0.00
ATOM	4	H3	GLN	1	-7.154	-12.258	0.436	1.00	0.00
ATOM	5	CA	GLN	1	-8.022	-13.483	1.738	1.00	0.00
ATOM	6	HA	GLN	1	-8.724	-13.145	2.500	1.00	0.00

```
ATOM      7  CB  GLN     1      -8.815 -13.899   0.491  1.00  0.00
ATOM      8  HB2 GLN     1      -8.518 -13.265  -0.344  1.00  0.00
ATOM      9  HB3 GLN     1      -8.585 -14.939   0.257  1.00  0.00
ATOM     10  CG  GLN     1     -10.315 -13.752   0.732  1.00  0.00
ATOM     11  HG2 GLN     1     -10.930 -14.559   0.333  1.00  0.00
ATOM     12  HG3 GLN     1     -10.391 -13.741   1.819  1.00  0.00
ATOM     13  CD  GLN     1     -10.840 -12.431   0.195  1.00  0.00
ATOM     14  OE1 GLN     1     -11.198 -11.530   0.935  1.00  0.00
ATOM     15  NE2 GLN     1     -10.736 -12.271  -1.111  1.00  0.00
ATOM     16 HE21 GLN     1     -11.064 -11.418  -1.540  1.00  0.00
ATOM     17 HE22 GLN     1     -10.329 -13.003  -1.676  1.00  0.00
ATOM     18  C   GLN     1      -7.290 -14.686   2.322  1.00  0.00
ATOM     19  O   GLN     1      -7.642 -15.166   3.390  1.00  0.00
ATOM     20  N   PRO     2      -6.232 -15.137   1.626  1.00  0.00
ATOM     21  CD  PRO     2      -5.736 -14.613   0.355  1.00  0.00
ATOM     22  HD2 PRO     2      -5.331 -13.619   0.545  1.00  0.00
ATOM     23  HD3 PRO     2      -6.586 -14.535  -0.322  1.00  0.00
ATOM     24  CG  PRO     2      -4.682 -15.586  -0.148  1.00  0.00
ATOM     25  HG2 PRO     2      -3.717 -15.081  -0.189  1.00  0.00
ATOM     26  HG3 PRO     2      -4.956 -15.927  -1.147  1.00  0.00
ATOM     27  CB  PRO     2      -4.659 -16.752   0.839  1.00  0.00
ATOM     28  HB2 PRO     2      -3.639 -17.013   1.122  1.00  0.00
```

ATOM	29	HB3	PRO	2	-5.153	-17.627	0.417	1.00	0.00
ATOM	30	CA	PRO	2	-5.428	-16.259	2.063	1.00	0.00
ATOM	31	HA	PRO	2	-6.098	-17.065	2.362	1.00	0.00
ATOM	32	C	PRO	2	-4.538	-15.893	3.219	1.00	0.00
ATOM	33	O	PRO	2	-3.549	-15.212	3.007	1.00	0.00
ATOM	34	N	GLN	3	-4.884	-16.356	4.424	1.00	0.00
ATOM	35	H	GLN	3	-5.707	-16.934	4.519	1.00	0.00
ATOM	36	CA	GLN	3	-4.116	-16.054	5.613	1.00	0.00
ATOM	37	HA	GLN	3	-4.042	-14.974	5.736	1.00	0.00
ATOM	38	CB	GLN	3	-4.782	-16.642	6.856	1.00	0.00
ATOM	39	HB2	GLN	3	-5.833	-16.831	6.637	1.00	0.00
ATOM	40	HB3	GLN	3	-4.290	-17.582	7.108	1.00	0.00
ATOM	41	CG	GLN	3	-4.673	-15.673	8.041	1.00	0.00
ATOM	42	HG2	GLN	3	-4.227	-16.217	8.874	1.00	0.00
ATOM	43	HG3	GLN	3	-4.043	-14.821	7.785	1.00	0.00
ATOM	44	CD	GLN	3	-6.030	-15.160	8.470	1.00	0.00
ATOM	45	OE1	GLN	3	-6.650	-15.674	9.380	1.00	0.00
ATOM	46	NE2	GLN	3	-6.496	-14.148	7.760	1.00	0.00
ATOM	47	HE21	GLN	3	-7.396	-13.750	7.985	1.00	0.00
ATOM	48	HE22	GLN	3	-5.950	-13.778	6.995	1.00	0.00
ATOM	49	C	GLN	3	-2.734	-16.631	5.489	1.00	0.00
ATOM	50	O	GLN	3	-1.793	-16.054	5.961	1.00	0.00

```
ATOM     51  N   LYS     4      -2.568 -17.745   4.784  1.00  0.00
ATOM     52  H   LYS     4      -3.384 -18.227   4.435  1.00  0.00
ATOM     53  CA  LYS     4      -1.264 -18.352   4.568  1.00  0.00
ATOM     54  HA  LYS     4      -0.913 -18.831   5.482  1.00  0.00
ATOM     55  CB  LYS     4      -1.371 -19.414   3.470  1.00  0.00
ATOM     56  HB2 LYS     4      -0.539 -20.110   3.581  1.00  0.00
ATOM     57  HB3 LYS     4      -2.311 -19.950   3.599  1.00  0.00
ATOM     58  CG  LYS     4      -1.329 -18.785   2.068  1.00  0.00
ATOM     59  HG2 LYS     4      -1.538 -17.719   2.156  1.00  0.00
ATOM     60  HG3 LYS     4      -0.332 -18.927   1.650  1.00  0.00
ATOM     61  CD  LYS     4      -2.363 -19.432   1.148  1.00  0.00
ATOM     62  HD2 LYS     4      -3.326 -19.454   1.659  1.00  0.00
ATOM     63  HD3 LYS     4      -2.448 -18.837   0.239  1.00  0.00
ATOM     64  CE  LYS     4      -1.947 -20.857   0.786  1.00  0.00
ATOM     65  HE2 LYS     4      -1.746 -20.910  -0.284  1.00  0.00
ATOM     66  HE3 LYS     4      -1.044 -21.117   1.338  1.00  0.00
ATOM     67  NZ  LYS     4      -3.023 -21.804   1.131  1.00  0.00
ATOM     68  HZ1 LYS     4      -3.860 -21.564   0.619  1.00  0.00
ATOM     69  HZ2 LYS     4      -2.737 -22.742   0.887  1.00  0.00
ATOM     70  HZ3 LYS     4      -3.209 -21.756   2.122  1.00  0.00
ATOM     71  C   LYS     4      -0.210 -17.280   4.239  1.00  0.00
ATOM     72  O   LYS     4       0.933 -17.444   4.603  1.00  0.00
```

ATOM	73	N	CYX	5	-0.654	-16.181	3.603	1.00	0.00
ATOM	74	H	CYX	5	-1.614	-16.148	3.291	1.00	0.00
ATOM	75	CA	CYX	5	0.185	-15.064	3.327	1.00	0.00
ATOM	76	HA	CYX	5	1.202	-15.260	3.666	1.00	0.00
ATOM	77	CB	CYX	5	0.212	-14.819	1.841	1.00	0.00
ATOM	78	HB2	CYX	5	-0.379	-15.583	1.335	1.00	0.00
ATOM	79	HB3	CYX	5	-0.206	-13.836	1.627	1.00	0.00
ATOM	80	SG	CYX	5	1.878	-14.897	1.115	1.00	0.00
ATOM	81	C	CYX	5	-0.327	-13.858	4.045	1.00	0.00
ATOM	82	O	CYX	5	0.429	-13.101	4.571	1.00	0.00
ATOM	83	N	GLN	6	-1.637	-13.667	4.168	1.00	0.00
ATOM	84	H	GLN	6	-2.251	-14.305	3.681	1.00	0.00
ATOM	85	CA	GLN	6	-2.227	-12.562	4.887	1.00	0.00
ATOM	86	HA	GLN	6	-1.816	-11.622	4.519	1.00	0.00
ATOM	87	CB	GLN	6	-3.744	-12.542	4.692	1.00	0.00
ATOM	88	HB2	GLN	6	-4.062	-13.528	4.354	1.00	0.00
ATOM	89	HB3	GLN	6	-4.213	-12.318	5.650	1.00	0.00
ATOM	90	CG	GLN	6	-4.160	-11.495	3.666	1.00	0.00
ATOM	91	HG2	GLN	6	-3.246	-10.990	3.353	1.00	0.00
ATOM	92	HG3	GLN	6	-4.634	-11.957	2.800	1.00	0.00
ATOM	93	CD	GLN	6	-5.106	-10.467	4.244	1.00	0.00
ATOM	94	OE1	GLN	6	-5.126	-10.168	5.423	1.00	0.00

ATOM 95 NE2 GLN 6 -5.968 -9.949 3.380 1.00 0.00

ATOM 96 HE21 GLN 6 -6.633 -9.255 3.689 1.00 0.00

ATOM 97 HE22 GLN 6 -5.955 -10.251 2.416 1.00 0.00

ATOM 98 C GLN 6 -1.875 -12.601 6.364 1.00 0.00

ATOM 99 O GLN 6 -2.120 -11.654 7.092 1.00 0.00

ATOM 100 N ARG 7 -1.309 -13.709 6.824 1.00 0.00

ATOM 101 H ARG 7 -1.185 -14.459 6.158 1.00 0.00

ATOM 102 CA ARG 7 -0.898 -13.929 8.178 1.00 0.00

ATOM 103 HA ARG 7 -1.303 -13.138 8.809 1.00 0.00

ATOM 104 CB ARG 7 -1.393 -15.279 8.707 1.00 0.00

ATOM 105 HB2 ARG 7 -2.198 -15.638 8.065 1.00 0.00

ATOM 106 HB3 ARG 7 -0.568 -15.992 8.687 1.00 0.00

ATOM 107 CG ARG 7 -1.908 -15.137 10.136 1.00 0.00

ATOM 108 HG2 ARG 7 -1.180 -14.570 10.715 1.00 0.00

ATOM 109 HG3 ARG 7 -2.855 -14.597 10.114 1.00 0.00

ATOM 110 CD ARG 7 -2.118 -16.510 10.784 1.00 0.00

ATOM 111 HD2 ARG 7 -3.112 -16.886 10.539 1.00 0.00

ATOM 112 HD3 ARG 7 -1.366 -17.210 10.418 1.00 0.00

ATOM 113 NE ARG 7 -1.998 -16.411 12.253 1.00 0.00

ATOM 114 HE ARG 7 -2.860 -16.319 12.771 1.00 0.00

ATOM 115 CZ ARG 7 -0.856 -16.429 12.924 1.00 0.00

ATOM 116 NH1 ARG 7 0.286 -16.560 12.290 1.00 0.00

```
ATOM    117 HH11 ARG     7       0.294 -16.648  11.284  1.00  0.00
ATOM    118 HH12 ARG     7       1.151 -16.573  12.811  1.00  0.00
ATOM    119  NH2 ARG     7      -0.854 -16.322  14.231  1.00  0.00
ATOM    120 HH21 ARG     7      -1.728 -16.226  14.728  1.00  0.00
ATOM    121 HH22 ARG     7       0.021 -16.337  14.734  1.00  0.00
ATOM    122  C   ARG     7       0.603 -13.875   8.279  1.00  0.00
ATOM    123  O   ARG     7       1.079 -13.882   9.387  1.00  0.00
ATOM    124  N   GLU     8       1.299 -13.863   7.155  1.00  0.00
ATOM    125  H   GLU     8       0.801 -13.956   6.281  1.00  0.00
ATOM    126  CA  GLU     8       2.744 -13.786   7.116  1.00  0.00
ATOM    127  HA  GLU     8       3.146 -13.828   8.128  1.00  0.00
ATOM    128  CB  GLU     8       3.313 -14.958   6.316  1.00  0.00
ATOM    129  HB2 GLU     8       2.490 -15.502   5.851  1.00  0.00
ATOM    130  HB3 GLU     8       3.975 -14.570   5.542  1.00  0.00
ATOM    131  CG  GLU     8       4.098 -15.905   7.231  1.00  0.00
ATOM    132  HG2 GLU     8       3.754 -15.710   8.246  1.00  0.00
ATOM    133  HG3 GLU     8       3.906 -16.948   6.977  1.00  0.00
ATOM    134  CD  GLU     8       5.602 -15.626   7.139  1.00  0.00
ATOM    135  OE1 GLU     8       6.161 -15.890   6.055  1.00  0.00
ATOM    136  OE2 GLU     8       6.158 -15.210   8.174  1.00  0.00
ATOM    137  C   GLU     8       3.220 -12.465   6.529  1.00  0.00
ATOM    138  O   GLU     8       4.288 -11.980   6.845  1.00  0.00
```

ATOM 139 N PHE 9 2.361 -11.809 5.761 1.00 0.00

ATOM 140 H PHE 9 1.447 -12.219 5.629 1.00 0.00

ATOM 141 CA PHE 9 2.646 -10.567 5.116 1.00 0.00

ATOM 142 HA PHE 9 3.684 -10.534 4.786 1.00 0.00

ATOM 143 CB PHE 9 1.742 -10.431 3.895 1.00 0.00

ATOM 144 HB2 PHE 9 2.217 -10.927 3.048 1.00 0.00

ATOM 145 HB3 PHE 9 0.771 -10.889 4.086 1.00 0.00

ATOM 146 CG PHE 9 1.471 -9.004 3.466 1.00 0.00

ATOM 147 CD1 PHE 9 0.648 -8.165 4.236 1.00 0.00

ATOM 148 HD1 PHE 9 0.176 -8.567 5.132 1.00 0.00

ATOM 149 CE1 PHE 9 0.431 -6.839 3.870 1.00 0.00

ATOM 150 HE1 PHE 9 -0.168 -6.186 4.504 1.00 0.00

ATOM 151 CZ PHE 9 0.982 -6.349 2.689 1.00 0.00

ATOM 152 HZ PHE 9 0.808 -5.318 2.380 1.00 0.00

ATOM 153 CE2 PHE 9 1.764 -7.192 1.897 1.00 0.00

ATOM 154 HE2 PHE 9 2.176 -6.808 0.964 1.00 0.00

ATOM 155 CD2 PHE 9 2.021 -8.510 2.284 1.00 0.00

ATOM 156 HD2 PHE 9 2.649 -9.148 1.662 1.00 0.00

ATOM 157 C PHE 9 2.378 -9.454 6.086 1.00 0.00

ATOM 158 O PHE 9 3.052 -8.444 6.106 1.00 0.00

ATOM 159 N GLN 10 1.305 -9.581 6.883 1.00 0.00

ATOM 160 H GLN 10 0.716 -10.397 6.788 1.00 0.00

ATOM	161	CA	GLN	10	0.917	-8.563	7.830	1.00	0.00
ATOM	162	HA	GLN	10	1.371	-7.612	7.551	1.00	0.00
ATOM	163	CB	GLN	10	-0.606	-8.391	7.853	1.00	0.00
ATOM	164	HB2	GLN	10	-0.902	-7.793	6.992	1.00	0.00
ATOM	165	HB3	GLN	10	-1.069	-9.376	7.787	1.00	0.00
ATOM	166	CG	GLN	10	-1.065	-7.695	9.142	1.00	0.00
ATOM	167	HG2	GLN	10	-0.917	-8.354	9.997	1.00	0.00
ATOM	168	HG3	GLN	10	-0.464	-6.795	9.269	1.00	0.00
ATOM	169	CD	GLN	10	-2.516	-7.300	9.084	1.00	0.00
ATOM	170	OE1	GLN	10	-2.885	-6.303	8.484	1.00	0.00
ATOM	171	NE2	GLN	10	-3.355	-8.115	9.697	1.00	0.00
ATOM	172	HE21	GLN	10	-4.344	-7.910	9.697	1.00	0.00
ATOM	173	HE22	GLN	10	-3.004	-8.940	10.163	1.00	0.00
ATOM	174	C	GLN	10	1.460	-8.934	9.204	1.00	0.00
ATOM	175	O	GLN	10	1.443	-8.126	10.131	1.00	0.00
ATOM	176	N	GLN	11	1.929	-10.166	9.362	1.00	0.00
ATOM	177	H	GLN	11	1.885	-10.818	8.591	1.00	0.00
ATOM	178	CA	GLN	11	2.459	-10.629	10.618	1.00	0.00
ATOM	179	HA	GLN	11	1.654	-10.729	11.346	1.00	0.00
ATOM	180	CB	GLN	11	3.128	-11.987	10.442	1.00	0.00
ATOM	181	HB2	GLN	11	2.800	-12.424	9.498	1.00	0.00
ATOM	182	HB3	GLN	11	4.209	-11.849	10.422	1.00	0.00

ATOM	183	CG	GLN	11	2.755	-12.924	11.594	1.00	0.00
ATOM	184	HG2	GLN	11	1.936	-12.453	12.137	1.00	0.00
ATOM	185	HG3	GLN	11	2.430	-13.895	11.219	1.00	0.00
ATOM	186	CD	GLN	11	3.911	-13.136	12.550	1.00	0.00
ATOM	187	OE1	GLN	11	5.065	-13.175	12.182	1.00	0.00
ATOM	188	NE2	GLN	11	3.571	-13.328	13.814	1.00	0.00
ATOM	189	HE21	GLN	11	4.288	-13.477	14.510	1.00	0.00
ATOM	190	HE22	GLN	11	2.596	-13.326	14.077	1.00	0.00
ATOM	191	C	GLN	11	3.447	-9.628	11.186	1.00	0.00
ATOM	192	O	GLN	11	3.248	-9.113	12.282	1.00	0.00
ATOM	193	N	GLU	12	4.437	-9.259	10.372	1.00	0.00
ATOM	194	H	GLU	12	4.507	-9.672	9.453	1.00	0.00
ATOM	195	CA	GLU	12	5.481	-8.344	10.797	1.00	0.00
ATOM	196	HA	GLU	12	5.034	-7.372	11.006	1.00	0.00
ATOM	197	CB	GLU	12	6.207	-8.832	12.060	1.00	0.00
ATOM	198	HB2	GLU	12	7.231	-8.459	12.034	1.00	0.00
ATOM	199	HB3	GLU	12	5.694	-8.424	12.931	1.00	0.00
ATOM	200	CG	GLU	12	6.223	-10.361	12.148	1.00	0.00
ATOM	201	HG2	GLU	12	5.324	-10.727	12.644	1.00	0.00
ATOM	202	HG3	GLU	12	6.242	-10.722	11.119	1.00	0.00
ATOM	203	CD	GLU	12	7.449	-10.869	12.884	1.00	0.00
ATOM	204	OE1	GLU	12	8.499	-11.008	12.216	1.00	0.00

ATOM	205	OE2	GLU	12	7.338	-11.060	14.119	1.00	0.00
ATOM	206	C	GLU	12	6.467	-8.104	9.662	1.00	0.00
ATOM	207	O	GLU	12	7.652	-7.868	9.868	1.00	0.00
ATOM	208	N	GLN	13	5.958	-8.121	8.431	1.00	0.00
ATOM	209	H	GLN	13	4.981	-8.342	8.299	1.00	0.00
ATOM	210	CA	GLN	13	6.775	-7.908	7.261	1.00	0.00
ATOM	211	HA	GLN	13	7.519	-7.139	7.467	1.00	0.00
ATOM	212	CB	GLN	13	7.501	-9.197	6.866	1.00	0.00
ATOM	213	HB2	GLN	13	6.922	-10.051	7.219	1.00	0.00
ATOM	214	HB3	GLN	13	7.583	-9.238	5.780	1.00	0.00
ATOM	215	CG	GLN	13	8.897	-9.239	7.483	1.00	0.00
ATOM	216	HG2	GLN	13	9.381	-8.263	7.474	1.00	0.00
ATOM	217	HG3	GLN	13	8.767	-9.570	8.513	1.00	0.00
ATOM	218	CD	GLN	13	9.793	-10.231	6.765	1.00	0.00
ATOM	219	OE1	GLN	13	9.716	-10.456	5.566	1.00	0.00
ATOM	220	NE2	GLN	13	10.730	-10.796	7.509	1.00	0.00
ATOM	221	HE21	GLN	13	11.362	-11.467	7.096	1.00	0.00
ATOM	222	HE22	GLN	13	10.808	-10.554	8.486	1.00	0.00
ATOM	223	C	GLN	13	5.933	-7.385	6.129	1.00	0.00
ATOM	224	O	GLN	13	5.952	-7.921	5.060	1.00	0.00
ATOM	225	N	HIE	14	5.149	-6.339	6.383	1.00	0.00
ATOM	226	H	HIE	14	5.129	-5.974	7.324	1.00	0.00

```
ATOM    227  CA  HIE    14       4.254  -5.760   5.403  1.00  0.00
ATOM    228  HA  HIE    14       3.646  -6.546   4.955  1.00  0.00
ATOM    229  CB  HIE    14       3.324  -4.731   6.052  1.00  0.00
ATOM    230  HB2 HIE    14       3.786  -3.744   6.057  1.00  0.00
ATOM    231  HB3 HIE    14       2.403  -4.698   5.469  1.00  0.00
ATOM    232  CG  HIE    14       2.925  -5.025   7.468  1.00  0.00
ATOM    233  ND1 HIE    14       2.373  -4.097   8.316  1.00  0.00
ATOM    234  CE1 HIE    14       2.149  -4.682   9.500  1.00  0.00
ATOM    235  HE1 HIE    14       1.685  -4.131  10.318  1.00  0.00
ATOM    236  NE2 HIE    14       2.582  -5.943   9.428  1.00  0.00
ATOM    237  HE2 HIE    14       2.567  -6.615  10.182  1.00  0.00
ATOM    238  CD2 HIE    14       3.053  -6.189   8.167  1.00  0.00
ATOM    239  HD2 HIE    14       3.430  -7.179   7.909  1.00  0.00
ATOM    240  C   HIE    14       5.100  -5.178   4.280  1.00  0.00
ATOM    241  O   HIE    14       6.210  -5.589   3.982  1.00  0.00
ATOM    242  N   LEU    15       4.602  -4.101   3.675  1.00  0.00
ATOM    243  H   LEU    15       3.673  -3.782   3.908  1.00  0.00
ATOM    244  CA  LEU    15       5.305  -3.436   2.602  1.00  0.00
ATOM    245  HA  LEU    15       6.305  -3.855   2.495  1.00  0.00
ATOM    246  CB  LEU    15       4.550  -3.627   1.280  1.00  0.00
ATOM    247  HB2 LEU    15       3.722  -2.924   1.367  1.00  0.00
ATOM    248  HB3 LEU    15       5.187  -3.335   0.445  1.00  0.00
```

ATOM	249	CG	LEU	15	4.000	-5.035	1.034	1.00	0.00
ATOM	250	HG	LEU	15	3.144	-5.198	1.689	1.00	0.00
ATOM	251	CD1	LEU	15	3.567	-5.202	-0.418	1.00	0.00
ATOM	252	HD11	LEU	15	4.422	-5.040	-1.074	1.00	0.00
ATOM	253	HD12	LEU	15	3.180	-6.210	-0.569	1.00	0.00
ATOM	254	HD13	LEU	15	2.788	-4.476	-0.652	1.00	0.00
ATOM	255	CD2	LEU	15	4.985	-6.099	1.426	1.00	0.00
ATOM	256	HD21	LEU	15	5.219	-6.006	2.486	1.00	0.00
ATOM	257	HD22	LEU	15	4.554	-7.082	1.235	1.00	0.00
ATOM	258	HD23	LEU	15	5.898	-5.983	0.841	1.00	0.00
ATOM	259	C	LEU	15	5.532	-1.986	2.958	1.00	0.00
ATOM	260	O	LEU	15	4.939	-1.488	3.921	1.00	0.00
ATOM	261	N	ARG	16	6.344	-1.296	2.156	1.00	0.00
ATOM	262	H	ARG	16	6.759	-1.754	1.357	1.00	0.00
ATOM	263	CA	ARG	16	6.653	0.096	2.384	1.00	0.00
ATOM	264	HA	ARG	16	5.794	0.589	2.840	1.00	0.00
ATOM	265	CB	ARG	16	7.854	0.248	3.315	1.00	0.00
ATOM	266	HB2	ARG	16	8.558	-0.558	3.109	1.00	0.00
ATOM	267	HB3	ARG	16	8.331	1.207	3.114	1.00	0.00
ATOM	268	CG	ARG	16	7.426	0.189	4.782	1.00	0.00
ATOM	269	HG2	ARG	16	6.355	-0.008	4.825	1.00	0.00
ATOM	270	HG3	ARG	16	7.964	-0.624	5.270	1.00	0.00

ATOM	271	CD	ARG	16	7.732	1.506	5.498	1.00	0.00
ATOM	272	HD2	ARG	16	7.700	2.326	4.781	1.00	0.00
ATOM	273	HD3	ARG	16	6.991	1.677	6.278	1.00	0.00
ATOM	274	NE	ARG	16	9.071	1.471	6.117	1.00	0.00
ATOM	275	HE	ARG	16	9.708	0.773	5.761	1.00	0.00
ATOM	276	CZ	ARG	16	9.489	2.275	7.077	1.00	0.00
ATOM	277	NH1	ARG	16	8.694	3.203	7.559	1.00	0.00
ATOM	278	HH11	ARG	16	7.758	3.301	7.193	1.00	0.00
ATOM	279	HH12	ARG	16	9.023	3.813	8.293	1.00	0.00
ATOM	280	NH2	ARG	16	10.699	2.151	7.565	1.00	0.00
ATOM	281	HH21	ARG	16	11.315	1.436	7.204	1.00	0.00
ATOM	282	HH22	ARG	16	11.010	2.770	8.299	1.00	0.00
ATOM	283	C	ARG	16	6.975	0.750	1.089	1.00	0.00
ATOM	284	O	ARG	16	6.157	1.485	0.606	1.00	0.00
ATOM	285	N	ALA	17	8.111	0.463	0.491	1.00	0.00
ATOM	286	H	ALA	17	8.771	-0.126	0.978	1.00	0.00
ATOM	287	CA	ALA	17	8.498	1.014	-0.781	1.00	0.00
ATOM	288	HA	ALA	17	8.655	2.086	-0.666	1.00	0.00
ATOM	289	CB	ALA	17	9.814	0.373	-1.246	1.00	0.00
ATOM	290	HB1	ALA	17	9.671	-0.700	-1.375	1.00	0.00
ATOM	291	HB2	ALA	17	10.118	0.814	-2.195	1.00	0.00
ATOM	292	HB3	ALA	17	10.588	0.548	-0.499	1.00	0.00

ATOM	293	C	ALA	17	7.374	0.840	-1.821	1.00	0.00
ATOM	294	O	ALA	17	7.276	1.583	-2.788	1.00	0.00
ATOM	295	N	CYX	18	6.435	-0.076	-1.563	1.00	0.00
ATOM	296	H	CYX	18	6.536	-0.634	-0.727	1.00	0.00
ATOM	297	CA	CYX	18	5.338	-0.350	-2.429	1.00	0.00
ATOM	298	HA	CYX	18	5.625	-0.133	-3.458	1.00	0.00
ATOM	299	CB	CYX	18	4.917	-1.811	-2.340	1.00	0.00
ATOM	300	HB2	CYX	18	5.727	-2.397	-1.905	1.00	0.00
ATOM	301	HB3	CYX	18	4.030	-1.897	-1.713	1.00	0.00
ATOM	302	SG	CYX	18	4.555	-2.606	-3.923	1.00	0.00
ATOM	303	C	CYX	18	4.224	0.584	-2.050	1.00	0.00
ATOM	304	O	CYX	18	3.668	1.282	-2.880	1.00	0.00
ATOM	305	N	GLN	19	3.895	0.630	-0.762	1.00	0.00
ATOM	306	H	GLN	19	4.328	-0.019	-0.121	1.00	0.00
ATOM	307	CA	GLN	19	2.880	1.510	-0.257	1.00	0.00
ATOM	308	HA	GLN	19	2.162	1.747	-1.042	1.00	0.00
ATOM	309	CB	GLN	19	2.145	0.837	0.884	1.00	0.00
ATOM	310	HB2	GLN	19	1.323	1.489	1.178	1.00	0.00
ATOM	311	HB3	GLN	19	1.744	-0.105	0.509	1.00	0.00
ATOM	312	CG	GLN	19	3.034	0.565	2.091	1.00	0.00
ATOM	313	HG2	GLN	19	2.854	-0.451	2.444	1.00	0.00
ATOM	314	HG3	GLN	19	4.080	0.669	1.803	1.00	0.00

ATOM	315	CD	GLN	19	2.744	1.534	3.228	1.00	0.00
ATOM	316	OE1	GLN	19	3.630	2.166	3.803	1.00	0.00
ATOM	317	NE2	GLN	19	1.469	1.655	3.570	1.00	0.00
ATOM	318	HE21	GLN	19	1.207	2.281	4.317	1.00	0.00
ATOM	319	HE22	GLN	19	0.765	1.120	3.081	1.00	0.00
ATOM	320	C	GLN	19	3.463	2.837	0.159	1.00	0.00
ATOM	321	O	GLN	19	2.967	3.451	1.074	1.00	0.00
ATOM	322	N	GLN	20	4.546	3.278	-0.458	1.00	0.00
ATOM	323	H	GLN	20	4.925	2.710	-1.203	1.00	0.00
ATOM	324	CA	GLN	20	5.236	4.495	-0.107	1.00	0.00
ATOM	325	HA	GLN	20	4.590	5.089	0.539	1.00	0.00
ATOM	326	CB	GLN	20	6.555	4.236	0.625	1.00	0.00
ATOM	327	HB2	GLN	20	7.113	3.476	0.078	1.00	0.00
ATOM	328	HB3	GLN	20	7.129	5.162	0.647	1.00	0.00
ATOM	329	CG	GLN	20	6.307	3.754	2.062	1.00	0.00
ATOM	330	HG2	GLN	20	5.345	3.244	2.123	1.00	0.00
ATOM	331	HG3	GLN	20	7.105	3.055	2.315	1.00	0.00
ATOM	332	CD	GLN	20	6.319	4.878	3.059	1.00	0.00
ATOM	333	OE1	GLN	20	6.879	5.940	2.856	1.00	0.00
ATOM	334	NE2	GLN	20	5.650	4.667	4.181	1.00	0.00
ATOM	335	HE21	GLN	20	5.621	5.385	4.890	1.00	0.00
ATOM	336	HE22	GLN	20	5.172	3.788	4.322	1.00	0.00

ATOM	337	C	GLN	20	5.500	5.283	-1.364	1.00	0.00
ATOM	338	O	GLN	20	5.317	6.491	-1.388	1.00	0.00
ATOM	339	N	TRP	21	5.876	4.596	-2.443	1.00	0.00
ATOM	340	H	TRP	21	6.042	3.602	-2.373	1.00	0.00
ATOM	341	CA	TRP	21	6.121	5.245	-3.713	1.00	0.00
ATOM	342	HA	TRP	21	6.884	6.014	-3.595	1.00	0.00
ATOM	343	CB	TRP	21	6.608	4.229	-4.745	1.00	0.00
ATOM	344	HB2	TRP	21	7.110	4.742	-5.565	1.00	0.00
ATOM	345	HB3	TRP	21	7.311	3.556	-4.254	1.00	0.00
ATOM	346	CG	TRP	21	5.553	3.370	-5.361	1.00	0.00
ATOM	347	CD1	TRP	21	5.208	2.101	-5.053	1.00	0.00
ATOM	348	HD1	TRP	21	5.795	1.645	-4.256	1.00	0.00
ATOM	349	NE1	TRP	21	4.162	1.644	-5.833	1.00	0.00
ATOM	350	HE1	TRP	21	3.723	0.737	-5.756	1.00	0.00
ATOM	351	CE2	TRP	21	3.794	2.635	-6.708	1.00	0.00
ATOM	352	CZ2	TRP	21	2.807	2.710	-7.689	1.00	0.00
ATOM	353	HZ2	TRP	21	2.185	1.829	-7.851	1.00	0.00
ATOM	354	CH2	TRP	21	2.674	3.900	-8.402	1.00	0.00
ATOM	355	HH2	TRP	21	1.931	3.985	-9.195	1.00	0.00
ATOM	356	CZ3	TRP	21	3.490	5.004	-8.113	1.00	0.00
ATOM	357	HZ3	TRP	21	3.344	5.945	-8.643	1.00	0.00
ATOM	358	CE3	TRP	21	4.504	4.942	-7.152	1.00	0.00

ATOM 359 HE3 TRP 21 5.135 5.817 -7.000 1.00 0.00
ATOM 360 CD2 TRP 21 4.658 3.748 -6.419 1.00 0.00
ATOM 361 C TRP 21 4.846 5.939 -4.160 1.00 0.00
ATOM 362 O TRP 21 4.863 7.009 -4.757 1.00 0.00
ATOM 363 N ILE 22 3.695 5.339 -3.833 1.00 0.00
ATOM 364 H ILE 22 3.721 4.455 -3.345 1.00 0.00
ATOM 365 CA ILE 22 2.406 5.879 -4.204 1.00 0.00
ATOM 366 HA ILE 22 2.393 6.208 -5.243 1.00 0.00
ATOM 367 CB ILE 22 1.311 4.827 -3.988 1.00 0.00
ATOM 368 HB ILE 22 1.257 4.579 -2.928 1.00 0.00
ATOM 369 CG2 ILE 22 -0.043 5.371 -4.454 1.00 0.00
ATOM 370 HG21 ILE 22 0.010 5.618 -5.514 1.00 0.00
ATOM 371 HG22 ILE 22 -0.813 4.615 -4.296 1.00 0.00
ATOM 372 HG23 ILE 22 -0.291 6.266 -3.884 1.00 0.00
ATOM 373 CG1 ILE 22 1.657 3.526 -4.719 1.00 0.00
ATOM 374 HG12 ILE 22 1.392 3.638 -5.771 1.00 0.00
ATOM 375 HG13 ILE 22 2.730 3.354 -4.631 1.00 0.00
ATOM 376 CD1 ILE 22 0.902 2.339 -4.129 1.00 0.00
ATOM 377 HD11 ILE 22 -0.171 2.510 -4.217 1.00 0.00
ATOM 378 HD12 ILE 22 1.170 1.431 -4.671 1.00 0.00
ATOM 379 HD13 ILE 22 1.166 2.225 -3.078 1.00 0.00
ATOM 380 C ILE 22 2.159 7.130 -3.385 1.00 0.00

ATOM	381	O	ILE	22	1.521	8.068	-3.839	1.00	0.00
ATOM	382	N	ARG	23	2.681	7.168	-2.153	1.00	0.00
ATOM	383	H	ARG	23	3.140	6.344	-1.790	1.00	0.00
ATOM	384	CA	ARG	23	2.581	8.329	-1.299	1.00	0.00
ATOM	385	HA	ARG	23	1.543	8.659	-1.255	1.00	0.00
ATOM	386	CB	ARG	23	3.051	8.014	0.114	1.00	0.00
ATOM	387	HB2	ARG	23	3.828	7.251	0.065	1.00	0.00
ATOM	388	HB3	ARG	23	3.460	8.920	0.559	1.00	0.00
ATOM	389	CG	ARG	23	1.894	7.506	0.968	1.00	0.00
ATOM	390	HG2	ARG	23	2.046	7.831	1.997	1.00	0.00
ATOM	391	HG3	ARG	23	0.964	7.928	0.588	1.00	0.00
ATOM	392	CD	ARG	23	1.817	5.985	0.923	1.00	0.00
ATOM	393	HD2	ARG	23	0.895	5.670	0.434	1.00	0.00
ATOM	394	HD3	ARG	23	2.671	5.582	0.379	1.00	0.00
ATOM	395	NE	ARG	23	1.833	5.440	2.308	1.00	0.00
ATOM	396	HE	ARG	23	1.049	4.850	2.549	1.00	0.00
ATOM	397	CZ	ARG	23	2.791	5.594	3.186	1.00	0.00
ATOM	398	NH1	ARG	23	3.818	6.347	2.884	1.00	0.00
ATOM	399	HH11	ARG	23	3.861	6.799	1.982	1.00	0.00
ATOM	400	HH12	ARG	23	4.563	6.472	3.554	1.00	0.00
ATOM	401	NH2	ARG	23	2.690	5.074	4.383	1.00	0.00
ATOM	402	HH21	ARG	23	1.865	4.548	4.634	1.00	0.00

ATOM	403	HH22	ARG	23	3.437	5.201	5.050	1.00	0.00
ATOM	404	C	ARG	23	3.396	9.499	-1.845	1.00	0.00
ATOM	405	O	ARG	23	3.182	10.646	-1.452	1.00	0.00
ATOM	406	N	GLN	24	4.331	9.208	-2.743	1.00	0.00
ATOM	407	H	GLN	24	4.518	8.242	-2.972	1.00	0.00
ATOM	408	CA	GLN	24	5.137	10.225	-3.365	1.00	0.00
ATOM	409	HA	GLN	24	5.354	11.018	-2.649	1.00	0.00
ATOM	410	CB	GLN	24	6.457	9.628	-3.846	1.00	0.00
ATOM	411	HB2	GLN	24	6.331	8.554	-3.983	1.00	0.00
ATOM	412	HB3	GLN	24	6.727	10.086	-4.798	1.00	0.00
ATOM	413	CG	GLN	24	7.566	9.884	-2.825	1.00	0.00
ATOM	414	HG2	GLN	24	8.283	10.589	-3.245	1.00	0.00
ATOM	415	HG3	GLN	24	7.121	10.311	-1.927	1.00	0.00
ATOM	416	CD	GLN	24	8.300	8.615	-2.445	1.00	0.00
ATOM	417	OE1	GLN	24	8.325	7.646	-3.176	1.00	0.00
ATOM	418	NE2	GLN	24	8.916	8.617	-1.284	1.00	0.00
ATOM	419	HE21	GLN	24	9.421	7.796	-0.982	1.00	0.00
ATOM	420	HE22	GLN	24	8.882	9.439	-0.699	1.00	0.00
ATOM	421	C	GLN	24	4.401	10.883	-4.519	1.00	0.00
ATOM	422	O	GLN	24	4.830	11.912	-5.028	1.00	0.00
ATOM	423	N	GLN	25	3.289	10.265	-4.945	1.00	0.00
ATOM	424	H	GLN	25	3.030	9.371	-4.553	1.00	0.00

ATOM	425	CA	GLN	25	2.479	10.806	-6.009	1.00	0.00
ATOM	426	HA	GLN	25	3.116	11.134	-6.830	1.00	0.00
ATOM	427	CB	GLN	25	1.512	9.741	-6.535	1.00	0.00
ATOM	428	HB2	GLN	25	1.605	8.843	-5.925	1.00	0.00
ATOM	429	HB3	GLN	25	0.493	10.121	-6.468	1.00	0.00
ATOM	430	CG	GLN	25	1.834	9.403	-7.990	1.00	0.00
ATOM	431	HG2	GLN	25	2.915	9.268	-8.012	1.00	0.00
ATOM	432	HG3	GLN	25	1.348	8.488	-8.330	1.00	0.00
ATOM	433	CD	GLN	25	1.466	10.530	-8.939	1.00	0.00
ATOM	434	OE1	GLN	25	0.974	11.572	-8.586	1.00	0.00
ATOM	435	NE2	GLN	25	1.616	10.284	-10.223	1.00	0.00
ATOM	436	HE21	GLN	25	1.387	10.997	-10.901	1.00	0.00
ATOM	437	HE22	GLN	25	1.960	9.383	-10.525	1.00	0.00
ATOM	438	C	GLN	25	1.727	12.048	-5.535	1.00	0.00
ATOM	439	O	GLN	25	1.114	12.779	-6.301	1.00	0.00
ATOM	440	N	LEU	26	1.738	12.298	-4.229	1.00	0.00
ATOM	441	H	LEU	26	2.161	11.622	-3.609	1.00	0.00
ATOM	442	CA	LEU	26	1.063	13.426	-3.649	1.00	0.00
ATOM	443	HA	LEU	26	0.545	13.985	-4.428	1.00	0.00
ATOM	444	CB	LEU	26	0.032	12.964	-2.608	1.00	0.00
ATOM	445	HB2	LEU	26	0.547	13.064	-1.653	1.00	0.00
ATOM	446	HB3	LEU	26	-0.818	13.645	-2.636	1.00	0.00

ATOM 447 CG LEU 26 -0.464 11.520 -2.772 1.00 0.00
ATOM 448 HG LEU 26 -0.349 11.220 -3.814 1.00 0.00
ATOM 449 CD1 LEU 26 0.336 10.582 -1.885 1.00 0.00
ATOM 450 HD11 LEU 26 0.221 10.880 -0.843 1.00 0.00
ATOM 451 HD12 LEU 26 -0.028 9.562 -2.013 1.00 0.00
ATOM 452 HD13 LEU 26 1.389 10.629 -2.162 1.00 0.00
ATOM 453 CD2 LEU 26 -1.954 11.419 -2.450 1.00 0.00
ATOM 454 HD21 LEU 26 -2.517 12.062 -3.126 1.00 0.00
ATOM 455 HD22 LEU 26 -2.284 10.387 -2.573 1.00 0.00
ATOM 456 HD23 LEU 26 -2.126 11.735 -1.421 1.00 0.00
ATOM 457 C LEU 26 2.096 14.396 -3.102 1.00 0.00
ATOM 458 O LEU 26 3.290 14.272 -3.345 1.00 0.00
ATOM 459 N ALA 27 1.623 15.361 -2.316 1.00 0.00
ATOM 460 H ALA 27 0.625 15.460 -2.193 1.00 0.00
ATOM 461 CA ALA 27 2.494 16.337 -1.689 1.00 0.00
ATOM 462 HA ALA 27 3.390 16.488 -2.291 1.00 0.00
ATOM 463 CB ALA 27 1.747 17.661 -1.587 1.00 0.00
ATOM 464 HB1 ALA 27 0.852 17.528 -0.980 1.00 0.00
ATOM 465 HB2 ALA 27 2.392 18.408 -1.124 1.00 0.00
ATOM 466 HB3 ALA 27 1.462 17.996 -2.585 1.00 0.00
ATOM 467 C ALA 27 2.970 15.823 -0.333 1.00 0.00
ATOM 468 O ALA 27 3.409 16.590 0.519 1.00 0.00

ATOM	469	N	GLY	28	2.826	14.510	-0.100	1.00	0.00
ATOM	470	H	GLY	28	2.399	13.933	-0.811	1.00	0.00
ATOM	471	CA	GLY	28	3.255	13.880	1.127	1.00	0.00
ATOM	472	HA2	GLY	28	3.047	14.551	1.960	1.00	0.00
ATOM	473	HA3	GLY	28	2.699	12.952	1.261	1.00	0.00
ATOM	474	C	GLY	28	4.743	13.567	1.103	1.00	0.00
ATOM	475	O	GLY	28	5.221	12.803	1.936	1.00	0.00
ATOM	476	N	SER	29	5.463	14.112	0.122	1.00	0.00
ATOM	477	H	SER	29	4.989	14.655	-0.585	1.00	0.00
ATOM	478	CA	SER	29	6.885	13.912	-0.009	1.00	0.00
ATOM	479	HA	SER	29	7.337	13.776	0.973	1.00	0.00
ATOM	480	CB	SER	29	7.158	12.666	-0.848	1.00	0.00
ATOM	481	HB2	SER	29	6.908	11.799	-0.236	1.00	0.00
ATOM	482	HB3	SER	29	6.495	12.703	-1.712	1.00	0.00
ATOM	483	OG	SER	29	8.498	12.562	-1.287	1.00	0.00
ATOM	484	HG	SER	29	8.603	11.761	-1.807	1.00	0.00
ATOM	485	C	SER	29	7.557	15.119	-0.620	1.00	0.00
ATOM	486	O	SER	29	6.911	15.849	-1.343	1.00	0.00
ATOM	487	N	PRO	30	8.816	15.363	-0.254	1.00	0.00
ATOM	488	CD	PRO	30	9.599	14.552	0.662	1.00	0.00
ATOM	489	HD2	PRO	30	9.769	13.583	0.192	1.00	0.00
ATOM	490	HD3	PRO	30	9.016	14.416	1.573	1.00	0.00

ATOM	491	CG	PRO	30	10.894	15.318	0.909	1.00	0.00
ATOM	492	HG2	PRO	30	11.733	14.726	0.542	1.00	0.00
ATOM	493	HG3	PRO	30	11.009	15.486	1.980	1.00	0.00
ATOM	494	CB	PRO	30	10.775	16.646	0.157	1.00	0.00
ATOM	495	HB2	PRO	30	11.677	16.864	-0.415	1.00	0.00
ATOM	496	HB3	PRO	30	10.574	17.467	0.845	1.00	0.00
ATOM	497	CA	PRO	30	9.593	16.459	-0.789	1.00	0.00
ATOM	498	HA	PRO	30	8.982	17.360	-0.740	1.00	0.00
ATOM	499	C	PRO	30	10.021	16.219	-2.216	1.00	0.00
ATOM	500	O	PRO	30	9.695	15.188	-2.793	1.00	0.00
ATOM	501	N	PHE	31	10.790	17.163	-2.770	1.00	0.00
ATOM	502	H	PHE	31	11.061	17.970	-2.227	1.00	0.00
ATOM	503	CA	PHE	31	11.261	17.065	-4.133	1.00	0.00
ATOM	504	HA	PHE	31	11.516	16.036	-4.387	1.00	0.00
ATOM	505	CB	PHE	31	10.131	17.531	-5.056	1.00	0.00
ATOM	506	HB2	PHE	31	10.063	18.617	-5.114	1.00	0.00
ATOM	507	HB3	PHE	31	10.357	17.129	-6.044	1.00	0.00
ATOM	508	CG	PHE	31	8.748	17.029	-4.690	1.00	0.00
ATOM	509	CD1	PHE	31	8.341	15.740	-5.069	1.00	0.00
ATOM	510	HD1	PHE	31	9.003	15.122	-5.677	1.00	0.00
ATOM	511	CE1	PHE	31	7.090	15.245	-4.670	1.00	0.00
ATOM	512	HE1	PHE	31	6.798	14.227	-4.929	1.00	0.00

ATOM	513	CZ	PHE	31	6.217	16.059	-3.939	1.00	0.00
ATOM	514	HZ	PHE	31	5.225	15.696	-3.668	1.00	0.00
ATOM	515	CE2	PHE	31	6.623	17.348	-3.554	1.00	0.00
ATOM	516	HE2	PHE	31	5.942	17.981	-2.985	1.00	0.00
ATOM	517	CD2	PHE	31	7.897	17.821	-3.899	1.00	0.00
ATOM	518	HD2	PHE	31	8.226	18.801	-3.554	1.00	0.00
ATOM	519	C	PHE	31	12.479	17.945	-4.353	1.00	0.00
ATOM	520	O	PHE	31	12.613	18.989	-3.711	1.00	0.00
ATOM	521	N	SER	32	13.359	17.518	-5.256	1.00	0.00
ATOM	522	H	SER	32	13.219	16.628	-5.714	1.00	0.00
ATOM	523	CA	SER	32	14.557	18.273	-5.572	1.00	0.00
ATOM	524	HA	SER	32	14.458	19.294	-5.205	1.00	0.00
ATOM	525	CB	SER	32	15.786	17.630	-4.911	1.00	0.00
ATOM	526	HB2	SER	32	15.473	16.699	-4.438	1.00	0.00
ATOM	527	HB3	SER	32	16.519	17.413	-5.688	1.00	0.00
ATOM	528	OG	SER	32	16.366	18.480	-3.941	1.00	0.00
ATOM	529	HG	SER	32	17.128	18.046	-3.551	1.00	0.00
ATOM	530	C	SER	32	14.738	18.353	-7.080	1.00	0.00
ATOM	531	O	SER	32	14.379	19.350	-7.706	1.00	0.00
ATOM	532	N	GLU	33	15.291	17.285	-7.664	1.00	0.00
ATOM	533	H	GLU	33	15.619	16.519	-7.094	1.00	0.00
ATOM	534	CA	GLU	33	15.524	17.227	-9.086	1.00	0.00

ATOM	535	HA	GLU	33	15.719	18.229	-9.468	1.00	0.00
ATOM	536	CB	GLU	33	16.734	16.338	-9.400	1.00	0.00
ATOM	537	HB2	GLU	33	17.005	15.791	-8.497	1.00	0.00
ATOM	538	HB3	GLU	33	16.451	15.632	-10.181	1.00	0.00
ATOM	539	CG	GLU	33	17.926	17.165	-9.870	1.00	0.00
ATOM	540	HG2	GLU	33	18.045	17.025	-10.945	1.00	0.00
ATOM	541	HG3	GLU	33	17.732	18.216	-9.660	1.00	0.00
ATOM	542	CD	GLU	33	19.204	16.731	-9.156	1.00	0.00
ATOM	543	OE1	GLU	33	19.585	15.551	-9.333	1.00	0.00
ATOM	544	OE2	GLU	33	19.775	17.584	-8.444	1.00	0.00
ATOM	545	C	GLU	33	14.268	16.756	-9.809	1.00	0.00
ATOM	546	O	GLU	33	13.185	16.662	-9.224	1.00	0.00
ATOM	547	N	ASN	34	14.420	16.431	-11.098	1.00	0.00
ATOM	548	H	ASN	34	15.318	16.556	-11.542	1.00	0.00
ATOM	549	CA	ASN	34	13.337	15.912	-11.886	1.00	0.00
ATOM	550	HA	ASN	34	12.387	16.305	-11.524	1.00	0.00
ATOM	551	CB	ASN	34	13.513	16.327	-13.345	1.00	0.00
ATOM	552	HB2	ASN	34	14.580	16.353	-13.568	1.00	0.00
ATOM	553	HB3	ASN	34	13.022	15.610	-14.003	1.00	0.00
ATOM	554	CG	ASN	34	12.931	17.699	-13.605	1.00	0.00
ATOM	555	OD1	ASN	34	11.863	18.054	-13.135	1.00	0.00
ATOM	556	ND2	ASN	34	13.647	18.475	-14.395	1.00	0.00

ATOM 557 HD21 ASN 34 13.321 19.405 -14.614 1.00 0.00
ATOM 558 HD22 ASN 34 14.518 18.135 -14.778 1.00 0.00
ATOM 559 C ASN 34 13.294 14.402 -11.805 1.00 0.00
ATOM 560 O ASN 34 14.340 13.756 -11.748 1.00 0.00
ATOM 561 N GLN 35 12.094 13.838 -11.897 1.00 0.00
ATOM 562 H GLN 35 11.276 14.429 -11.939 1.00 0.00
ATOM 563 CA GLN 35 11.904 12.410 -11.879 1.00 0.00
ATOM 564 HA GLN 35 12.406 11.964 -12.738 1.00 0.00
ATOM 565 CB GLN 35 12.482 11.798 -10.603 1.00 0.00
ATOM 566 HB2 GLN 35 13.262 12.459 -10.227 1.00 0.00
ATOM 567 HB3 GLN 35 11.683 11.723 -9.865 1.00 0.00
ATOM 568 CG GLN 35 13.069 10.414 -10.859 1.00 0.00
ATOM 569 HG2 GLN 35 13.304 9.931 -9.910 1.00 0.00
ATOM 570 HG3 GLN 35 12.331 9.818 -11.396 1.00 0.00
ATOM 571 CD GLN 35 14.337 10.486 -11.695 1.00 0.00
ATOM 572 OE1 GLN 35 15.379 10.946 -11.272 1.00 0.00
ATOM 573 NE2 GLN 35 14.231 9.995 -12.919 1.00 0.00
ATOM 574 HE21 GLN 35 15.031 10.007 -13.536 1.00 0.00
ATOM 575 HE22 GLN 35 13.351 9.610 -13.231 1.00 0.00
ATOM 576 C GLN 35 10.437 12.090 -12.012 1.00 0.00
ATOM 577 O GLN 35 9.593 12.943 -11.733 1.00 0.00
ATOM 578 N TRP 36 10.141 10.833 -12.391 1.00 0.00

ATOM	579	H	TRP	36	10.883	10.192	-12.634	1.00	0.00
ATOM	580	CA	TRP	36	8.766	10.378	-12.525	1.00	0.00
ATOM	581	HA	TRP	36	8.310	10.836	-13.403	1.00	0.00
ATOM	582	CB	TRP	36	8.715	8.855	-12.686	1.00	0.00
ATOM	583	HB2	TRP	36	8.999	8.570	-13.699	1.00	0.00
ATOM	584	HB3	TRP	36	9.406	8.403	-11.975	1.00	0.00
ATOM	585	CG	TRP	36	7.368	8.239	-12.427	1.00	0.00
ATOM	586	CD1	TRP	36	6.686	8.159	-11.251	1.00	0.00
ATOM	587	HD1	TRP	36	7.193	8.588	-10.387	1.00	0.00
ATOM	588	NE1	TRP	36	5.462	7.532	-11.412	1.00	0.00
ATOM	589	HE1	TRP	36	4.795	7.347	-10.677	1.00	0.00
ATOM	590	CE2	TRP	36	5.311	7.182	-12.733	1.00	0.00
ATOM	591	CZ2	TRP	36	4.283	6.527	-13.412	1.00	0.00
ATOM	592	HZ2	TRP	36	3.403	6.224	-12.845	1.00	0.00
ATOM	593	CH2	TRP	36	4.447	6.299	-14.787	1.00	0.00
ATOM	594	HH2	TRP	36	3.668	5.791	-15.356	1.00	0.00
ATOM	595	CZ3	TRP	36	5.613	6.720	-15.452	1.00	0.00
ATOM	596	HZ3	TRP	36	5.729	6.537	-16.521	1.00	0.00
ATOM	597	CE3	TRP	36	6.652	7.378	-14.781	1.00	0.00
ATOM	598	HE3	TRP	36	7.536	7.671	-15.347	1.00	0.00
ATOM	599	CD2	TRP	36	6.511	7.618	-13.400	1.00	0.00
ATOM	600	C	TRP	36	7.963	10.840	-11.301	1.00	0.00

ATOM	601	O	TRP	36	6.768	11.089	-11.393	1.00	0.00
ATOM	602	N	GLY	37	8.641	10.938	-10.145	1.00	0.00
ATOM	603	H	GLY	37	9.605	10.638	-10.110	1.00	0.00
ATOM	604	CA	GLY	37	8.045	11.456	-8.945	1.00	0.00
ATOM	605	HA2	GLY	37	7.601	12.428	-9.157	1.00	0.00
ATOM	606	HA3	GLY	37	7.269	10.769	-8.606	1.00	0.00
ATOM	607	C	GLY	37	9.078	11.614	-7.847	1.00	0.00
ATOM	608	O	GLY	37	9.620	12.699	-7.651	1.00	0.00
ATOM	609	N	PRO	38	9.377	10.505	-7.153	1.00	0.00
ATOM	610	CD	PRO	38	8.791	9.184	-7.347	1.00	0.00
ATOM	611	HD2	PRO	38	9.133	8.804	-8.310	1.00	0.00
ATOM	612	HD3	PRO	38	7.707	9.298	-7.366	1.00	0.00
ATOM	613	CG	PRO	38	9.266	8.326	-6.176	1.00	0.00
ATOM	614	HG2	PRO	38	9.876	7.507	-6.557	1.00	0.00
ATOM	615	HG3	PRO	38	8.399	7.920	-5.654	1.00	0.00
ATOM	616	CB	PRO	38	10.073	9.244	-5.261	1.00	0.00
ATOM	617	HB2	PRO	38	11.014	8.776	-4.971	1.00	0.00
ATOM	618	HB3	PRO	38	9.506	9.503	-4.367	1.00	0.00
ATOM	619	CA	PRO	38	10.349	10.493	-6.081	1.00	0.00
ATOM	620	HA	PRO	38	10.158	11.346	-5.430	1.00	0.00
ATOM	621	C	PRO	38	11.757	10.575	-6.616	1.00	0.00
ATOM	622	O	PRO	38	12.470	9.587	-6.664	1.00	0.00

ATOM	623	N	GLN	39	12.182	11.764	-7.033	1.00	0.00
ATOM	624	H	GLN	39	11.553	12.553	-7.017	1.00	0.00
ATOM	625	CA	GLN	39	13.509	11.957	-7.570	1.00	0.00
ATOM	626	HA	GLN	39	13.666	11.296	-8.423	1.00	0.00
ATOM	627	CB	GLN	39	13.683	13.408	-8.036	1.00	0.00
ATOM	628	HB2	GLN	39	12.862	13.673	-8.703	1.00	0.00
ATOM	629	HB3	GLN	39	13.675	14.068	-7.169	1.00	0.00
ATOM	630	CG	GLN	39	15.014	13.559	-8.780	1.00	0.00
ATOM	631	HG2	GLN	39	15.145	12.729	-9.475	1.00	0.00
ATOM	632	HG3	GLN	39	14.976	14.496	-9.336	1.00	0.00
ATOM	633	CD	GLN	39	16.205	13.599	-7.843	1.00	0.00
ATOM	634	OE1	GLN	39	16.095	13.982	-6.681	1.00	0.00
ATOM	635	NE2	GLN	39	17.343	13.173	-8.346	1.00	0.00
ATOM	636	HE21	GLN	39	18.176	13.173	-7.775	1.00	0.00
ATOM	637	HE22	GLN	39	17.379	12.847	-9.302	1.00	0.00
ATOM	638	C	GLN	39	14.557	11.591	-6.532	1.00	0.00
ATOM	639	O	GLN	39	15.619	11.074	-6.873	1.00	0.00
ATOM	640	N	GLN	40	14.236	11.839	-5.258	1.00	0.00
ATOM	641	H	GLN	40	13.368	12.305	-5.036	1.00	0.00
ATOM	642	CA	GLN	40	15.133	11.516	-4.179	1.00	0.00
ATOM	643	HA	GLN	40	16.168	11.613	-4.508	1.00	0.00
ATOM	644	CB	GLN	40	14.899	12.474	-3.012	1.00	0.00

ATOM	645	HB2	GLN	40	14.679	13.467	-3.404	1.00	0.00
ATOM	646	HB3	GLN	40	14.052	12.120	-2.425	1.00	0.00
ATOM	647	CG	GLN	40	16.146	12.541	-2.121	1.00	0.00
ATOM	648	HG2	GLN	40	16.973	12.123	-2.696	1.00	0.00
ATOM	649	HG3	GLN	40	16.377	13.571	-1.849	1.00	0.00
ATOM	650	CD	GLN	40	15.980	11.725	-0.843	1.00	0.00
ATOM	651	OE1	GLN	40	14.904	11.279	-0.464	1.00	0.00
ATOM	652	NE2	GLN	40	17.078	11.564	-0.125	1.00	0.00
ATOM	653	HE21	GLN	40	17.043	11.034	0.734	1.00	0.00
ATOM	654	HE22	GLN	40	17.947	11.971	-0.439	1.00	0.00
ATOM	655	C	GLN	40	14.960	10.064	-3.740	1.00	0.00
ATOM	656	O	GLN	40	15.683	9.571	-2.884	1.00	0.00
ATOM	657	N	GLY	41	13.979	9.371	-4.319	1.00	0.00
ATOM	658	H	GLY	41	13.388	9.844	-4.987	1.00	0.00
ATOM	659	CA	GLY	41	13.718	7.979	-4.040	1.00	0.00
ATOM	660	HA2	GLY	41	14.650	7.493	-3.752	1.00	0.00
ATOM	661	HA3	GLY	41	13.003	7.908	-3.221	1.00	0.00
ATOM	662	C	GLY	41	13.149	7.273	-5.253	1.00	0.00
ATOM	663	O	GLY	41	12.025	6.814	-5.248	1.00	0.00
ATOM	664	N	PRO	42	13.902	7.203	-6.350	1.00	0.00
ATOM	665	CD	PRO	42	15.243	7.732	-6.488	1.00	0.00
ATOM	666	HD2	PRO	42	15.901	7.155	-5.838	1.00	0.00

ATOM	667	HD3	PRO	42	15.230	8.771	-6.161	1.00	0.00
ATOM	668	CG	PRO	42	15.606	7.591	-7.963	1.00	0.00
ATOM	669	HG2	PRO	42	16.456	6.914	-8.057	1.00	0.00
ATOM	670	HG3	PRO	42	15.877	8.570	-8.357	1.00	0.00
ATOM	671	CB	PRO	42	14.369	7.037	-8.677	1.00	0.00
ATOM	672	HB2	PRO	42	14.638	6.207	-9.330	1.00	0.00
ATOM	673	HB3	PRO	42	13.864	7.808	-9.259	1.00	0.00
ATOM	674	CA	PRO	42	13.452	6.543	-7.562	1.00	0.00
ATOM	675	HA	PRO	42	12.439	6.881	-7.780	1.00	0.00
ATOM	676	C	PRO	42	13.461	5.037	-7.421	1.00	0.00
ATOM	677	O	PRO	42	12.622	4.346	-8.012	1.00	0.00
ATOM	678	N	SER	43	14.396	4.539	-6.619	1.00	0.00
ATOM	679	H	SER	43	15.058	5.170	-6.192	1.00	0.00
ATOM	680	CA	SER	43	14.544	3.124	-6.375	1.00	0.00
ATOM	681	HA	SER	43	14.441	2.581	-7.315	1.00	0.00
ATOM	682	CB	SER	43	15.916	2.805	-5.774	1.00	0.00
ATOM	683	HB2	SER	43	15.814	2.752	-4.690	1.00	0.00
ATOM	684	HB3	SER	43	16.243	1.837	-6.155	1.00	0.00
ATOM	685	OG	SER	43	16.877	3.796	-6.112	1.00	0.00
ATOM	686	HG	SER	43	17.723	3.567	-5.721	1.00	0.00
ATOM	687	C	SER	43	13.428	2.644	-5.454	1.00	0.00
ATOM	688	O	SER	43	13.637	2.333	-4.288	1.00	0.00

ATOM	689	N	LEU	44	12.201	2.599	-5.971	1.00	0.00
ATOM	690	H	LEU	44	12.058	2.907	-6.922	1.00	0.00
ATOM	691	CA	LEU	44	11.056	2.176	-5.213	1.00	0.00
ATOM	692	HA	LEU	44	11.380	1.712	-4.281	1.00	0.00
ATOM	693	CB	LEU	44	10.166	3.373	-4.880	1.00	0.00
ATOM	694	HB2	LEU	44	10.384	4.119	-5.644	1.00	0.00
ATOM	695	HB3	LEU	44	9.122	3.069	-4.957	1.00	0.00
ATOM	696	CG	LEU	44	10.424	3.975	-3.488	1.00	0.00
ATOM	697	HG	LEU	44	11.415	3.670	-3.153	1.00	0.00
ATOM	698	CD1	LEU	44	10.348	5.483	-3.528	1.00	0.00
ATOM	699	HD11	LEU	44	9.357	5.788	-3.863	1.00	0.00
ATOM	700	HD12	LEU	44	10.534	5.883	-2.532	1.00	0.00
ATOM	701	HD13	LEU	44	11.098	5.867	-4.219	1.00	0.00
ATOM	702	CD2	LEU	44	9.437	3.447	-2.469	1.00	0.00
ATOM	703	HD21	LEU	44	9.530	2.363	-2.400	1.00	0.00
ATOM	704	HD22	LEU	44	9.646	3.891	-1.496	1.00	0.00
ATOM	705	HD23	LEU	44	8.424	3.705	-2.776	1.00	0.00
ATOM	706	C	LEU	44	10.328	1.149	-6.019	1.00	0.00
ATOM	707	O	LEU	44	9.914	0.181	-5.453	1.00	0.00
ATOM	708	N	ARG	45	10.164	1.321	-7.326	1.00	0.00
ATOM	709	H	ARG	45	10.533	2.162	-7.745	1.00	0.00
ATOM	710	CA	ARG	45	9.490	0.370	-8.179	1.00	0.00

ATOM	711	HA	ARG	45	8.477	0.201	-7.813	1.00	0.00
ATOM	712	CB	ARG	45	9.410	0.891	-9.610	1.00	0.00
ATOM	713	HB2	ARG	45	10.412	1.172	-9.935	1.00	0.00
ATOM	714	HB3	ARG	45	9.032	0.093	-10.250	1.00	0.00
ATOM	715	CG	ARG	45	8.487	2.100	-9.706	1.00	0.00
ATOM	716	HG2	ARG	45	8.996	2.958	-9.267	1.00	0.00
ATOM	717	HG3	ARG	45	8.286	2.297	-10.759	1.00	0.00
ATOM	718	CD	ARG	45	7.159	1.859	-8.967	1.00	0.00
ATOM	719	HD2	ARG	45	6.927	0.794	-8.968	1.00	0.00
ATOM	720	HD3	ARG	45	7.242	2.211	-7.939	1.00	0.00
ATOM	721	NE	ARG	45	6.060	2.581	-9.625	1.00	0.00
ATOM	722	HE	ARG	45	5.321	2.018	-10.022	1.00	0.00
ATOM	723	CZ	ARG	45	5.958	3.895	-9.697	1.00	0.00
ATOM	724	NH1	ARG	45	6.863	4.669	-9.135	1.00	0.00
ATOM	725	HH11	ARG	45	7.643	4.255	-8.644	1.00	0.00
ATOM	726	HH12	ARG	45	6.773	5.673	-9.198	1.00	0.00
ATOM	727	NH2	ARG	45	4.970	4.442	-10.366	1.00	0.00
ATOM	728	HH21	ARG	45	4.289	3.854	-10.825	1.00	0.00
ATOM	729	HH22	ARG	45	4.896	5.448	-10.419	1.00	0.00
ATOM	730	C	ARG	45	10.223	-0.929	-8.198	1.00	0.00
ATOM	731	O	ARG	45	9.746	-1.879	-7.651	1.00	0.00
ATOM	732	N	GLU	46	11.424	-0.966	-8.738	1.00	0.00

ATOM	733	H	GLU	46	11.802	-0.126	-9.151	1.00	0.00
ATOM	734	CA	GLU	46	12.219	-2.182	-8.799	1.00	0.00
ATOM	735	HA	GLU	46	11.705	-2.932	-9.400	1.00	0.00
ATOM	736	CB	GLU	46	13.583	-1.893	-9.437	1.00	0.00
ATOM	737	HB2	GLU	46	14.049	-1.061	-8.910	1.00	0.00
ATOM	738	HB3	GLU	46	14.211	-2.779	-9.342	1.00	0.00
ATOM	739	CG	GLU	46	13.425	-1.535	-10.920	1.00	0.00
ATOM	740	HG2	GLU	46	13.187	-2.452	-11.460	1.00	0.00
ATOM	741	HG3	GLU	46	12.611	-0.821	-11.043	1.00	0.00
ATOM	742	CD	GLU	46	14.716	-0.937	-11.476	1.00	0.00
ATOM	743	OE1	GLU	46	15.218	0.023	-10.846	1.00	0.00
ATOM	744	OE2	GLU	46	15.168	-1.428	-12.530	1.00	0.00
ATOM	745	C	GLU	46	12.412	-2.769	-7.401	1.00	0.00
ATOM	746	O	GLU	46	12.691	-3.956	-7.260	1.00	0.00
ATOM	747	N	GLN	47	12.227	-1.933	-6.367	1.00	0.00
ATOM	748	H	GLN	47	12.048	-0.957	-6.552	1.00	0.00
ATOM	749	CA	GLN	47	12.326	-2.357	-5.007	1.00	0.00
ATOM	750	HA	GLN	47	13.092	-3.127	-4.913	1.00	0.00
ATOM	751	CB	GLN	47	12.704	-1.183	-4.114	1.00	0.00
ATOM	752	HB2	GLN	47	12.657	-0.263	-4.696	1.00	0.00
ATOM	753	HB3	GLN	47	11.999	-1.125	-3.285	1.00	0.00
ATOM	754	CG	GLN	47	14.124	-1.368	-3.565	1.00	0.00

ATOM	755	HG2	GLN	47	14.202	-2.350	-3.097	1.00	0.00
ATOM	756	HG3	GLN	47	14.854	-1.292	-4.371	1.00	0.00
ATOM	757	CD	GLN	47	14.439	-0.306	-2.522	1.00	0.00
ATOM	758	OE1	GLN	47	15.398	0.443	-2.623	1.00	0.00
ATOM	759	NE2	GLN	47	13.613	-0.255	-1.488	1.00	0.00
ATOM	760	HE21	GLN	47	13.764	0.426	-0.758	1.00	0.00
ATOM	761	HE22	GLN	47	12.836	-0.898	-1.434	1.00	0.00
ATOM	762	C	GLN	47	11.038	-2.970	-4.551	1.00	0.00
ATOM	763	O	GLN	47	11.088	-4.068	-4.085	1.00	0.00
ATOM	764	N	CYX	48	9.903	-2.314	-4.688	1.00	0.00
ATOM	765	H	CYX	48	9.992	-1.386	-5.076	1.00	0.00
ATOM	766	CA	CYX	48	8.603	-2.768	-4.311	1.00	0.00
ATOM	767	HA	CYX	48	8.643	-3.129	-3.283	1.00	0.00
ATOM	768	CB	CYX	48	7.551	-1.673	-4.401	1.00	0.00
ATOM	769	HB2	CYX	48	7.248	-1.377	-3.397	1.00	0.00
ATOM	770	HB3	CYX	48	7.966	-0.812	-4.924	1.00	0.00
ATOM	771	SG	CYX	48	6.016	-2.103	-5.258	1.00	0.00
ATOM	772	C	CYX	48	8.227	-3.913	-5.193	1.00	0.00
ATOM	773	O	CYX	48	7.769	-4.918	-4.725	1.00	0.00
ATOM	774	N	CYX	49	8.456	-3.826	-6.495	1.00	0.00
ATOM	775	H	CYX	49	8.816	-2.944	-6.829	1.00	0.00
ATOM	776	CA	CYX	49	8.186	-4.861	-7.458	1.00	0.00

ATOM 777 HA CYX 49 7.108 -5.000 -7.536 1.00 0.00
ATOM 778 CB CYX 49 8.738 -4.517 -8.848 1.00 0.00
ATOM 779 HB2 CYX 49 9.819 -4.390 -8.789 1.00 0.00
ATOM 780 HB3 CYX 49 8.506 -5.325 -9.542 1.00 0.00
ATOM 781 SG CYX 49 8.117 -2.986 -9.601 1.00 0.00
ATOM 782 C CYX 49 8.792 -6.161 -6.939 1.00 0.00
ATOM 783 O CYX 49 8.342 -7.235 -7.309 1.00 0.00
ATOM 784 N ASN 50 9.824 -6.065 -6.088 1.00 0.00
ATOM 785 H ASN 50 10.196 -5.149 -5.883 1.00 0.00
ATOM 786 CA ASN 50 10.412 -7.194 -5.443 1.00 0.00
ATOM 787 HA ASN 50 10.147 -8.113 -5.966 1.00 0.00
ATOM 788 CB ASN 50 11.928 -7.045 -5.459 1.00 0.00
ATOM 789 HB2 ASN 50 12.210 -6.028 -5.732 1.00 0.00
ATOM 790 HB3 ASN 50 12.312 -7.274 -4.465 1.00 0.00
ATOM 791 CG ASN 50 12.552 -8.001 -6.449 1.00 0.00
ATOM 792 OD1 ASN 50 13.120 -9.025 -6.101 1.00 0.00
ATOM 793 ND2 ASN 50 12.420 -7.665 -7.719 1.00 0.00
ATOM 794 HD21 ASN 50 12.812 -8.257 -8.438 1.00 0.00
ATOM 795 HD22 ASN 50 11.928 -6.819 -7.966 1.00 0.00
ATOM 796 C ASN 50 9.971 -7.296 -4.014 1.00 0.00
ATOM 797 O ASN 50 9.733 -8.375 -3.562 1.00 0.00
ATOM 798 N GLU 51 9.858 -6.207 -3.285 1.00 0.00

ATOM	799	H	GLU	51	10.154	-5.351	-3.730	1.00	0.00
ATOM	800	CA	GLU	51	9.442	-6.152	-1.912	1.00	0.00
ATOM	801	HA	GLU	51	10.024	-6.869	-1.333	1.00	0.00
ATOM	802	CB	GLU	51	9.646	-4.756	-1.312	1.00	0.00
ATOM	803	HB2	GLU	51	10.219	-4.856	-0.390	1.00	0.00
ATOM	804	HB3	GLU	51	10.207	-4.152	-2.025	1.00	0.00
ATOM	805	CG	GLU	51	8.311	-4.079	-1.010	1.00	0.00
ATOM	806	HG2	GLU	51	7.768	-4.091	-1.956	1.00	0.00
ATOM	807	HG3	GLU	51	7.730	-4.604	-0.252	1.00	0.00
ATOM	808	CD	GLU	51	8.528	-2.675	-0.582	1.00	0.00
ATOM	809	OE1	GLU	51	8.793	-2.375	0.599	1.00	0.00
ATOM	810	OE2	GLU	51	8.334	-1.835	-1.453	1.00	0.00
ATOM	811	C	GLU	51	7.989	-6.610	-1.793	1.00	0.00
ATOM	812	O	GLU	51	7.492	-6.837	-0.700	1.00	0.00
ATOM	813	N	LEU	52	7.295	-6.735	-2.918	1.00	0.00
ATOM	814	H	LEU	52	7.730	-6.421	-3.774	1.00	0.00
ATOM	815	CA	LEU	52	5.927	-7.147	-2.964	1.00	0.00
ATOM	816	HA	LEU	52	5.503	-7.155	-1.960	1.00	0.00
ATOM	817	CB	LEU	52	5.119	-6.169	-3.830	1.00	0.00
ATOM	818	HB2	LEU	52	4.239	-5.999	-3.210	1.00	0.00
ATOM	819	HB3	LEU	52	5.665	-5.233	-3.949	1.00	0.00
ATOM	820	CG	LEU	52	4.683	-6.684	-5.206	1.00	0.00

ATOM 821 HG LEU 52 4.316 -5.835 -5.783 1.00 0.00
ATOM 822 CD1 LEU 52 5.812 -7.351 -5.955 1.00 0.00
ATOM 823 HD11 LEU 52 6.179 -8.201 -5.379 1.00 0.00
ATOM 824 HD12 LEU 52 5.451 -7.698 -6.923 1.00 0.00
ATOM 825 HD13 LEU 52 6.622 -6.637 -6.104 1.00 0.00
ATOM 826 CD2 LEU 52 3.483 -7.597 -5.082 1.00 0.00
ATOM 827 HD21 LEU 52 2.654 -7.050 -4.633 1.00 0.00
ATOM 828 HD22 LEU 52 3.191 -7.951 -6.071 1.00 0.00
ATOM 829 HD23 LEU 52 3.738 -8.450 -4.453 1.00 0.00
ATOM 830 C LEU 52 5.876 -8.603 -3.396 1.00 0.00
ATOM 831 O LEU 52 4.882 -9.263 -3.150 1.00 0.00
ATOM 832 N TYR 53 6.935 -9.084 -4.058 1.00 0.00
ATOM 833 H TYR 53 7.752 -8.497 -4.139 1.00 0.00
ATOM 834 CA TYR 53 6.985 -10.397 -4.623 1.00 0.00
ATOM 835 HA TYR 53 5.980 -10.790 -4.776 1.00 0.00
ATOM 836 CB TYR 53 7.690 -10.318 -5.977 1.00 0.00
ATOM 837 HB2 TYR 53 7.215 -9.512 -6.536 1.00 0.00
ATOM 838 HB3 TYR 53 8.731 -10.061 -5.783 1.00 0.00
ATOM 839 CG TYR 53 7.628 -11.597 -6.787 1.00 0.00
ATOM 840 CD1 TYR 53 6.389 -12.140 -7.158 1.00 0.00
ATOM 841 HD1 TYR 53 5.476 -11.629 -6.853 1.00 0.00
ATOM 842 CE1 TYR 53 6.313 -13.322 -7.909 1.00 0.00

ATOM	843	HE1	TYR	53	5.349	-13.732	-8.211	1.00	0.00
ATOM	844	CZ	TYR	53	7.503	-13.973	-8.284	1.00	0.00
ATOM	845	OH	TYR	53	7.446	-15.129	-8.994	1.00	0.00
ATOM	846	HH	TYR	53	6.547	-15.409	-9.184	1.00	0.00
ATOM	847	CE2	TYR	53	8.755	-13.440	-7.916	1.00	0.00
ATOM	848	HE2	TYR	53	9.676	-13.946	-8.207	1.00	0.00
ATOM	849	CD2	TYR	53	8.812	-12.248	-7.168	1.00	0.00
ATOM	850	HD2	TYR	53	9.777	-11.829	-6.884	1.00	0.00
ATOM	851	C	TYR	53	7.783	-11.277	-3.714	1.00	0.00
ATOM	852	O	TYR	53	7.570	-12.469	-3.676	1.00	0.00
ATOM	853	N	GLN	54	8.739	-10.714	-2.987	1.00	0.00
ATOM	854	H	GLN	54	8.889	-9.724	-3.121	1.00	0.00
ATOM	855	CA	GLN	54	9.593	-11.414	-2.069	1.00	0.00
ATOM	856	HA	GLN	54	10.219	-12.116	-2.620	1.00	0.00
ATOM	857	CB	GLN	54	10.501	-10.444	-1.303	1.00	0.00
ATOM	858	HB2	GLN	54	11.058	-11.012	-0.558	1.00	0.00
ATOM	859	HB3	GLN	54	11.197	-9.994	-2.011	1.00	0.00
ATOM	860	CG	GLN	54	9.696	-9.344	-0.609	1.00	0.00
ATOM	861	HG2	GLN	54	9.216	-8.695	-1.342	1.00	0.00
ATOM	862	HG3	GLN	54	8.933	-9.832	-0.002	1.00	0.00
ATOM	863	CD	GLN	54	10.563	-8.495	0.295	1.00	0.00
ATOM	864	OE1	GLN	54	11.729	-8.226	0.021	1.00	0.00

```
ATOM    865  NE2 GLN    54       9.978  -8.025   1.374  1.00  0.00
ATOM    866 HE21 GLN    54      10.502  -7.451   2.019  1.00  0.00
ATOM    867 HE22 GLN    54       9.007  -8.240   1.553  1.00  0.00
ATOM    868  C   GLN    54       8.743 -12.219  -1.098  1.00  0.00
ATOM    869  O   GLN    54       9.190 -13.245  -0.601  1.00  0.00
ATOM    870  N   GLU    55       7.508 -11.761  -0.863  1.00  0.00
ATOM    871  H   GLU    55       7.237 -10.865  -1.243  1.00  0.00
ATOM    872  CA  GLU    55       6.580 -12.447  -0.010  1.00  0.00
ATOM    873  HA  GLU    55       7.118 -12.963   0.785  1.00  0.00
ATOM    874  CB  GLU    55       5.616 -11.459   0.619  1.00  0.00
ATOM    875  HB2 GLU    55       4.918 -11.124  -0.149  1.00  0.00
ATOM    876  HB3 GLU    55       5.067 -11.971   1.409  1.00  0.00
ATOM    877  CG  GLU    55       6.343 -10.257   1.203  1.00  0.00
ATOM    878  HG2 GLU    55       7.274 -10.652   1.608  1.00  0.00
ATOM    879  HG3 GLU    55       6.567  -9.508   0.444  1.00  0.00
ATOM    880  CD  GLU    55       5.539  -9.624   2.314  1.00  0.00
ATOM    881  OE1 GLU    55       4.599 -10.247   2.841  1.00  0.00
ATOM    882  OE2 GLU    55       5.853  -8.459   2.560  1.00  0.00
ATOM    883  C   GLU    55       5.853 -13.542  -0.759  1.00  0.00
ATOM    884  O   GLU    55       4.817 -14.002  -0.321  1.00  0.00
ATOM    885  N   ASP    56       6.386 -13.990  -1.885  1.00  0.00
ATOM    886  H   ASP    56       7.275 -13.593  -2.153  1.00  0.00
```

ATOM 887 CA ASP 56 5.799 -14.980 -2.740 1.00 0.00
ATOM 888 HA ASP 56 6.550 -15.399 -3.410 1.00 0.00
ATOM 889 CB ASP 56 5.224 -16.112 -1.881 1.00 0.00
ATOM 890 HB2 ASP 56 5.914 -16.144 -1.038 1.00 0.00
ATOM 891 HB3 ASP 56 4.226 -15.857 -1.525 1.00 0.00
ATOM 892 CG ASP 56 5.187 -17.464 -2.552 1.00 0.00
ATOM 893 OD1 ASP 56 5.621 -17.551 -3.721 1.00 0.00
ATOM 894 OD2 ASP 56 4.632 -18.395 -1.934 1.00 0.00
ATOM 895 C ASP 56 4.685 -14.361 -3.572 1.00 0.00
ATOM 896 O ASP 56 4.459 -13.144 -3.599 1.00 0.00
ATOM 897 N GLN 57 3.921 -15.220 -4.258 1.00 0.00
ATOM 898 H GLN 57 4.151 -16.204 -4.250 1.00 0.00
ATOM 899 CA GLN 57 2.805 -14.786 -5.060 1.00 0.00
ATOM 900 HA GLN 57 2.975 -13.771 -5.419 1.00 0.00
ATOM 901 CB GLN 57 2.638 -15.710 -6.267 1.00 0.00
ATOM 902 HB2 GLN 57 3.558 -16.276 -6.412 1.00 0.00
ATOM 903 HB3 GLN 57 1.815 -16.399 -6.077 1.00 0.00
ATOM 904 CG GLN 57 2.339 -14.895 -7.524 1.00 0.00
ATOM 905 HG2 GLN 57 2.948 -13.991 -7.507 1.00 0.00
ATOM 906 HG3 GLN 57 2.570 -15.472 -8.420 1.00 0.00
ATOM 907 CD GLN 57 0.885 -14.496 -7.570 1.00 0.00
ATOM 908 OE1 GLN 57 -0.006 -15.294 -7.714 1.00 0.00

ATOM	909	NE2	GLN	57	0.617	-13.223	-7.470	1.00	0.00
ATOM	910	HE21	GLN	57	-0.343	-12.909	-7.496	1.00	0.00
ATOM	911	HE22	GLN	57	1.370	-12.558	-7.367	1.00	0.00
ATOM	912	C	GLN	57	1.529	-14.745	-4.246	1.00	0.00
ATOM	913	O	GLN	57	0.598	-14.003	-4.539	1.00	0.00
ATOM	914	N	VAL	58	1.489	-15.521	-3.171	1.00	0.00
ATOM	915	H	VAL	58	2.267	-16.141	-2.997	1.00	0.00
ATOM	916	CA	VAL	58	0.345	-15.588	-2.282	1.00	0.00
ATOM	917	HA	VAL	58	-0.593	-15.578	-2.837	1.00	0.00
ATOM	918	CB	VAL	58	0.397	-16.863	-1.431	1.00	0.00
ATOM	919	HB	VAL	58	0.736	-16.609	-0.427	1.00	0.00
ATOM	920	CG1	VAL	58	-0.992	-17.504	-1.354	1.00	0.00
ATOM	921	HG11	VAL	58	-1.331	-17.759	-2.358	1.00	0.00
ATOM	922	HG12	VAL	58	-0.942	-18.409	-0.747	1.00	0.00
ATOM	923	HG13	VAL	58	-1.692	-16.802	-0.902	1.00	0.00
ATOM	924	CG2	VAL	58	1.404	-17.872	-1.993	1.00	0.00
ATOM	925	HG21	VAL	58	2.398	-17.426	-2.006	1.00	0.00
ATOM	926	HG22	VAL	58	1.415	-18.764	-1.366	1.00	0.00
ATOM	927	HG23	VAL	58	1.116	-18.147	-3.008	1.00	0.00
ATOM	928	C	VAL	58	0.300	-14.328	-1.439	1.00	0.00
ATOM	929	O	VAL	58	-0.643	-14.153	-0.670	1.00	0.00
ATOM	930	N	CYX	59	1.285	-13.442	-1.615	1.00	0.00

```
ATOM    931  H   CYX    59       2.024 -13.664  -2.266  1.00  0.00
ATOM    932  CA  CYX    59       1.360 -12.205  -0.899  1.00  0.00
ATOM    933  HA  CYX    59       0.530 -12.138  -0.196  1.00  0.00
ATOM    934  CB  CYX    59       2.650 -12.109  -0.130  1.00  0.00
ATOM    935  HB2 CYX    59       3.470 -12.473  -0.749  1.00  0.00
ATOM    936  HB3 CYX    59       2.835 -11.070   0.143  1.00  0.00
ATOM    937  SG  CYX    59       2.726 -13.083   1.417  1.00  0.00
ATOM    938  C   CYX    59       1.249 -11.055  -1.869  1.00  0.00
ATOM    939  O   CYX    59       0.664 -10.018  -1.541  1.00  0.00
ATOM    940  N   VAL    60       1.692 -11.266  -3.117  1.00  0.00
ATOM    941  H   VAL    60       2.160 -12.135  -3.331  1.00  0.00
ATOM    942  CA  VAL    60       1.590 -10.269  -4.158  1.00  0.00
ATOM    943  HA  VAL    60       2.206  -9.388  -3.982  1.00  0.00
ATOM    944  CB  VAL    60       1.981 -10.876  -5.500  1.00  0.00
ATOM    945  HB  VAL    60       1.628 -11.907  -5.535  1.00  0.00
ATOM    946  CG1 VAL    60       1.368 -10.090  -6.654  1.00  0.00
ATOM    947 HG11 VAL    60       1.721  -9.060  -6.621  1.00  0.00
ATOM    948 HG12 VAL    60       1.662 -10.544  -7.600  1.00  0.00
ATOM    949 HG13 VAL    60       0.281 -10.105  -6.567  1.00  0.00
ATOM    950  CG2 VAL    60       3.485 -10.973  -5.636  1.00  0.00
ATOM    951 HG21 VAL    60       3.882 -11.603  -4.840  1.00  0.00
ATOM    952 HG22 VAL    60       3.735 -11.410  -6.603  1.00  0.00
```

ATOM 953 HG23 VAL 60 3.922 -9.977 -5.564 1.00 0.00

ATOM 954 C VAL 60 0.190 -9.700 -4.188 1.00 0.00

ATOM 955 O VAL 60 -0.008 -8.575 -4.613 1.00 0.00

ATOM 956 N CYX 61 -0.802 -10.475 -3.757 1.00 0.00

ATOM 957 H CYX 61 -0.583 -11.439 -3.548 1.00 0.00

ATOM 958 CA CYX 61 -2.149 -10.053 -3.646 1.00 0.00

ATOM 959 HA CYX 61 -2.252 -9.327 -2.840 1.00 0.00

ATOM 960 CB CYX 61 -2.586 -9.399 -4.949 1.00 0.00

ATOM 961 HB2 CYX 61 -1.821 -9.556 -5.709 1.00 0.00

ATOM 962 HB3 CYX 61 -3.524 -9.843 -5.282 1.00 0.00

ATOM 963 SG CYX 61 -2.858 -7.609 -4.893 1.00 0.00

ATOM 964 C CYX 61 -3.012 -11.251 -3.270 1.00 0.00

ATOM 965 O CYX 61 -2.560 -12.374 -3.370 1.00 0.00

ATOM 966 N PRO 62 -4.261 -10.995 -2.847 1.00 0.00

ATOM 967 CD PRO 62 -5.270 -11.948 -2.430 1.00 0.00

ATOM 968 HD2 PRO 62 -4.916 -12.446 -1.527 1.00 0.00

ATOM 969 HD3 PRO 62 -5.394 -12.684 -3.225 1.00 0.00

ATOM 970 CG PRO 62 -6.544 -11.126 -2.189 1.00 0.00

ATOM 971 HG2 PRO 62 -6.828 -11.220 -1.141 1.00 0.00

ATOM 972 HG3 PRO 62 -7.341 -11.523 -2.818 1.00 0.00

ATOM 973 CB PRO 62 -6.231 -9.674 -2.555 1.00 0.00

ATOM 974 HB2 PRO 62 -6.577 -8.964 -1.805 1.00 0.00

```
ATOM    975  HB3 PRO    62      -6.667  -9.422  -3.522  1.00  0.00
ATOM    976  CA  PRO    62      -4.720  -9.634  -2.643  1.00  0.00
ATOM    977  HA  PRO    62      -4.485  -9.061  -3.540  1.00  0.00
ATOM    978  C   PRO    62      -4.080  -8.990  -1.459  1.00  0.00
ATOM    979  O   PRO    62      -4.367  -7.871  -1.165  1.00  0.00
ATOM    980  N   THR    63      -3.248  -9.702  -0.744  1.00  0.00
ATOM    981  H   THR    63      -3.155 -10.670  -1.018  1.00  0.00
ATOM    982  CA  THR    63      -2.513  -9.245   0.409  1.00  0.00
ATOM    983  HA  THR    63      -3.216  -9.338   1.237  1.00  0.00
ATOM    984  CB  THR    63      -1.333 -10.177   0.665  1.00  0.00
ATOM    985  HB  THR    63      -0.424  -9.669   0.343  1.00  0.00
ATOM    986  CG2 THR    63      -1.209 -10.555   2.117  1.00  0.00
ATOM    987 HG21 THR    63      -2.117 -11.064   2.439  1.00  0.00
ATOM    988 HG22 THR    63      -0.355 -11.219   2.248  1.00  0.00
ATOM    989 HG23 THR    63      -1.065  -9.656   2.716  1.00  0.00
ATOM    990  OG1 THR    63      -1.648 -11.400   0.012  1.00  0.00
ATOM    991  HG1 THR    63      -0.932 -12.027   0.143  1.00  0.00
ATOM    992  C   THR    63      -2.043  -7.837   0.236  1.00  0.00
ATOM    993  O   THR    63      -2.358  -7.060   1.113  1.00  0.00
ATOM    994  N   LEU    64      -1.442  -7.488  -0.901  1.00  0.00
ATOM    995  H   LEU    64      -1.256  -8.213  -1.580  1.00  0.00
ATOM    996  CA  LEU    64      -1.002  -6.139  -1.197  1.00  0.00
```

ATOM 997 HA LEU 64 -0.134 -5.885 -0.589 1.00 0.00
ATOM 998 CB LEU 64 -0.613 -6.034 -2.670 1.00 0.00
ATOM 999 HB2 LEU 64 -0.432 -7.063 -2.980 1.00 0.00
ATOM 1000 HB3 LEU 64 -1.448 -5.626 -3.239 1.00 0.00
ATOM 1001 CG LEU 64 0.643 -5.193 -2.931 1.00 0.00
ATOM 1002 HG LEU 64 1.285 -5.233 -2.051 1.00 0.00
ATOM 1003 CD1 LEU 64 1.397 -5.723 -4.129 1.00 0.00
ATOM 1004 HD11 LEU 64 0.756 -5.683 -5.010 1.00 0.00
ATOM 1005 HD12 LEU 64 2.285 -5.114 -4.299 1.00 0.00
ATOM 1006 HD13 LEU 64 1.695 -6.755 -3.944 1.00 0.00
ATOM 1007 CD2 LEU 64 0.289 -3.722 -3.129 1.00 0.00
ATOM 1008 HD21 LEU 64 -0.204 -3.343 -2.234 1.00 0.00
ATOM 1009 HD22 LEU 64 1.199 -3.151 -3.312 1.00 0.00
ATOM 1010 HD23 LEU 64 -0.381 -3.621 -3.983 1.00 0.00
ATOM 1011 C LEU 64 -2.077 -5.130 -0.869 1.00 0.00
ATOM 1012 O LEU 64 -1.763 -4.112 -0.264 1.00 0.00
ATOM 1013 N LYS 65 -3.348 -5.460 -1.159 1.00 0.00
ATOM 1014 H LYS 65 -3.491 -6.281 -1.731 1.00 0.00
ATOM 1015 CA LYS 65 -4.498 -4.667 -0.803 1.00 0.00
ATOM 1016 HA LYS 65 -4.554 -3.798 -1.459 1.00 0.00
ATOM 1017 CB LYS 65 -5.799 -5.470 -0.942 1.00 0.00
ATOM 1018 HB2 LYS 65 -5.913 -5.776 -1.982 1.00 0.00

```
ATOM   1019  HB3 LYS    65      -5.734  -6.355  -0.309  1.00  0.00
ATOM   1020  CG  LYS    65      -7.002  -4.634  -0.524  1.00  0.00
ATOM   1021  HG2 LYS    65      -6.739  -3.580  -0.611  1.00  0.00
ATOM   1022  HG3 LYS    65      -7.831  -4.858  -1.196  1.00  0.00
ATOM   1023  CD  LYS    65      -7.419  -4.939   0.921  1.00  0.00
ATOM   1024  HD2 LYS    65      -6.604  -5.459   1.424  1.00  0.00
ATOM   1025  HD3 LYS    65      -7.625  -4.002   1.437  1.00  0.00
ATOM   1026  CE  LYS    65      -8.671  -5.817   0.940  1.00  0.00
ATOM   1027  HE2 LYS    65      -9.233  -5.656   0.020  1.00  0.00
ATOM   1028  HE3 LYS    65      -8.372  -6.863   1.003  1.00  0.00
ATOM   1029  NZ  LYS    65      -9.518  -5.477   2.100  1.00  0.00
ATOM   1030  HZ1 LYS    65      -9.796  -4.508   2.041  1.00  0.00
ATOM   1031  HZ2 LYS    65     -10.340  -6.064   2.100  1.00  0.00
ATOM   1032  HZ3 LYS    65      -8.997  -5.627   2.952  1.00  0.00
ATOM   1033  C   LYS    65      -4.317  -4.096   0.585  1.00  0.00
ATOM   1034  O   LYS    65      -4.765  -3.018   0.824  1.00  0.00
ATOM   1035  N   GLN    66      -3.685  -4.792   1.509  1.00  0.00
ATOM   1036  H   GLN    66      -3.497  -5.754   1.265  1.00  0.00
ATOM   1037  CA  GLN    66      -3.331  -4.347   2.806  1.00  0.00
ATOM   1038  HA  GLN    66      -4.218  -3.971   3.316  1.00  0.00
ATOM   1039  CB  GLN    66      -2.735  -5.487   3.638  1.00  0.00
ATOM   1040  HB2 GLN    66      -3.442  -6.316   3.670  1.00  0.00
```

ATOM	1041	HB3	GLN	66	-1.805	-5.821	3.177	1.00	0.00
ATOM	1042	CG	GLN	66	-2.451	-4.999	5.071	1.00	0.00
ATOM	1043	HG2	GLN	66	-2.173	-5.808	5.747	1.00	0.00
ATOM	1044	HG3	GLN	66	-1.623	-4.295	4.988	1.00	0.00
ATOM	1045	CD	GLN	66	-3.656	-4.268	5.655	1.00	0.00
ATOM	1046	OE1	GLN	66	-3.543	-3.235	6.291	1.00	0.00
ATOM	1047	NE2	GLN	66	-4.844	-4.782	5.349	1.00	0.00
ATOM	1048	HE21	GLN	66	-5.685	-4.348	5.702	1.00	0.00
ATOM	1049	HE22	GLN	66	-4.900	-5.604	4.765	1.00	0.00
ATOM	1050	C	GLN	66	-2.385	-3.173	2.788	1.00	0.00
ATOM	1051	O	GLN	66	-2.776	-2.070	3.149	1.00	0.00
ATOM	1052	N	ALA	67	-1.130	-3.396	2.404	1.00	0.00
ATOM	1053	H	ALA	67	-0.831	-4.343	2.217	1.00	0.00
ATOM	1054	CA	ALA	67	-0.154	-2.337	2.302	1.00	0.00
ATOM	1055	HA	ALA	67	0.065	-1.926	3.287	1.00	0.00
ATOM	1056	CB	ALA	67	1.134	-2.897	1.711	1.00	0.00
ATOM	1057	HB1	ALA	67	0.933	-3.304	0.720	1.00	0.00
ATOM	1058	HB2	ALA	67	1.875	-2.101	1.633	1.00	0.00
ATOM	1059	HB3	ALA	67	1.517	-3.687	2.357	1.00	0.00
ATOM	1060	C	ALA	67	-0.707	-1.193	1.459	1.00	0.00
ATOM	1061	O	ALA	67	-0.329	-0.051	1.655	1.00	0.00
ATOM	1062	N	ALA	68	-1.684	-1.468	0.602	1.00	0.00

```
ATOM   1063  H   ALA    68      -1.959  -2.433   0.487  1.00  0.00
ATOM   1064  CA  ALA    68      -2.328  -0.477  -0.207  1.00  0.00
ATOM   1065  HA  ALA    68      -1.638   0.335  -0.438  1.00  0.00
ATOM   1066  CB  ALA    68      -2.766  -1.130  -1.512  1.00  0.00
ATOM   1067  HB1 ALA    68      -3.464  -1.939  -1.298  1.00  0.00
ATOM   1068  HB2 ALA    68      -3.254  -0.388  -2.145  1.00  0.00
ATOM   1069  HB3 ALA    68      -1.894  -1.531  -2.029  1.00  0.00
ATOM   1070  C   ALA    68      -3.507   0.141   0.531  1.00  0.00
ATOM   1071  O   ALA    68      -3.879   1.292   0.330  1.00  0.00
ATOM   1072  N   LYS    69      -4.119  -0.600   1.443  1.00  0.00
ATOM   1073  H   LYS    69      -3.820  -1.561   1.526  1.00  0.00
ATOM   1074  CA  LYS    69      -5.223  -0.159   2.246  1.00  0.00
ATOM   1075  HA  LYS    69      -5.925   0.399   1.626  1.00  0.00
ATOM   1076  CB  LYS    69      -5.961  -1.352   2.870  1.00  0.00
ATOM   1077  HB2 LYS    69      -6.319  -2.001   2.071  1.00  0.00
ATOM   1078  HB3 LYS    69      -5.265  -1.905   3.502  1.00  0.00
ATOM   1079  CG  LYS    69      -7.148  -0.878   3.712  1.00  0.00
ATOM   1080  HG2 LYS    69      -7.775  -1.739   3.946  1.00  0.00
ATOM   1081  HG3 LYS    69      -6.768  -0.444   4.637  1.00  0.00
ATOM   1082  CD  LYS    69      -7.979   0.171   2.957  1.00  0.00
ATOM   1083  HD2 LYS    69      -7.436   1.116   2.963  1.00  0.00
ATOM   1084  HD3 LYS    69      -8.111  -0.165   1.928  1.00  0.00
```

ATOM	1085	CE	LYS	69	-9.341	0.365	3.606	1.00	0.00
ATOM	1086	HE2	LYS	69	-10.069	-0.263	3.092	1.00	0.00
ATOM	1087	HE3	LYS	69	-9.279	0.066	4.652	1.00	0.00
ATOM	1088	NZ	LYS	69	-9.763	1.776	3.522	1.00	0.00
ATOM	1089	HZ1	LYS	69	-9.821	2.052	2.552	1.00	0.00
ATOM	1090	HZ2	LYS	69	-10.668	1.883	3.958	1.00	0.00
ATOM	1091	HZ3	LYS	69	-9.089	2.358	3.998	1.00	0.00
ATOM	1092	C	LYS	69	-4.712	0.812	3.281	1.00	0.00
ATOM	1093	O	LYS	69	-5.398	1.762	3.599	1.00	0.00
ATOM	1094	N	SER	70	-3.491	0.626	3.766	1.00	0.00
ATOM	1095	H	SER	70	-2.972	-0.189	3.472	1.00	0.00
ATOM	1096	CA	SER	70	-2.885	1.512	4.736	1.00	0.00
ATOM	1097	HA	SER	70	-3.653	2.128	5.204	1.00	0.00
ATOM	1098	CB	SER	70	-2.176	0.711	5.820	1.00	0.00
ATOM	1099	HB2	SER	70	-2.892	0.019	6.264	1.00	0.00
ATOM	1100	HB3	SER	70	-1.364	0.147	5.360	1.00	0.00
ATOM	1101	OG	SER	70	-1.650	1.560	6.829	1.00	0.00
ATOM	1102	HG	SER	70	-1.210	1.029	7.497	1.00	0.00
ATOM	1103	C	SER	70	-1.914	2.449	4.044	1.00	0.00
ATOM	1104	O	SER	70	-1.110	3.137	4.675	1.00	0.00
ATOM	1105	N	VAL	71	-1.974	2.502	2.708	1.00	0.00
ATOM	1106	H	VAL	71	-2.590	1.872	2.214	1.00	0.00

ATOM	1107	CA	VAL	71	-1.118	3.374	1.932	1.00	0.00
ATOM	1108	HA	VAL	71	-0.061	3.276	2.179	1.00	0.00
ATOM	1109	CB	VAL	71	-1.299	3.090	0.441	1.00	0.00
ATOM	1110	HB	VAL	71	-1.767	2.111	0.328	1.00	0.00
ATOM	1111	CG1	VAL	71	-2.162	4.138	-0.216	1.00	0.00
ATOM	1112	HG11	VAL	71	-1.695	5.116	-0.104	1.00	0.00
ATOM	1113	HG12	VAL	71	-2.271	3.907	-1.276	1.00	0.00
ATOM	1114	HG13	VAL	71	-3.144	4.148	0.256	1.00	0.00
ATOM	1115	CG2	VAL	71	0.032	2.972	-0.273	1.00	0.00
ATOM	1116	HG21	VAL	71	0.608	2.156	0.164	1.00	0.00
ATOM	1117	HG22	VAL	71	-0.139	2.770	-1.331	1.00	0.00
ATOM	1118	HG23	VAL	71	0.586	3.904	-0.167	1.00	0.00
ATOM	1119	C	VAL	71	-1.427	4.817	2.304	1.00	0.00
ATOM	1120	O	VAL	71	-0.717	5.741	1.966	1.00	0.00
ATOM	1121	N	ARG	72	-2.553	5.071	2.964	1.00	0.00
ATOM	1122	H	ARG	72	-3.186	4.306	3.151	1.00	0.00
ATOM	1123	CA	ARG	72	-2.932	6.389	3.394	1.00	0.00
ATOM	1124	HA	ARG	72	-2.054	6.958	3.699	1.00	0.00
ATOM	1125	CB	ARG	72	-3.608	7.118	2.230	1.00	0.00
ATOM	1126	HB2	ARG	72	-3.589	6.475	1.349	1.00	0.00
ATOM	1127	HB3	ARG	72	-4.642	7.334	2.499	1.00	0.00
ATOM	1128	CG	ARG	72	-2.878	8.424	1.923	1.00	0.00

```
ATOM   1129  HG2 ARG    72      -1.946   8.438   2.487  1.00  0.00
ATOM   1130  HG3 ARG    72      -2.657   8.452   0.856  1.00  0.00
ATOM   1131  CD  ARG    72      -3.717   9.638   2.299  1.00  0.00
ATOM   1132  HD2 ARG    72      -4.450   9.818   1.512  1.00  0.00
ATOM   1133  HD3 ARG    72      -4.234   9.432   3.236  1.00  0.00
ATOM   1134  NE  ARG    72      -2.902  10.845   2.467  1.00  0.00
ATOM   1135  HE  ARG    72      -2.949  11.532   1.729  1.00  0.00
ATOM   1136  CZ  ARG    72      -2.109  11.082   3.504  1.00  0.00
ATOM   1137  NH1 ARG    72      -2.050  10.225   4.499  1.00  0.00
ATOM   1138 HH11 ARG    72      -2.610   9.385   4.472  1.00  0.00
ATOM   1139 HH12 ARG    72      -1.445  10.410   5.286  1.00  0.00
ATOM   1140  NH2 ARG    72      -1.394  12.177   3.545  1.00  0.00
ATOM   1141 HH21 ARG    72      -1.448  12.840   2.785  1.00  0.00
ATOM   1142 HH22 ARG    72      -0.792  12.353   4.336  1.00  0.00
ATOM   1143  C   ARG    72      -3.857   6.296   4.579  1.00  0.00
ATOM   1144  O   ARG    72      -3.452   6.638   5.682  1.00  0.00
ATOM   1145  N   VAL    73      -5.122   5.929   4.313  1.00  0.00
ATOM   1146  H   VAL    73      -5.396   5.775   3.353  1.00  0.00
ATOM   1147  CA  VAL    73      -6.134   5.828   5.338  1.00  0.00
ATOM   1148  HA  VAL    73      -6.418   6.797   5.748  1.00  0.00
ATOM   1149  CB  VAL    73      -7.382   5.156   4.765  1.00  0.00
ATOM   1150  HB  VAL    73      -7.552   5.551   3.764  1.00  0.00
```

ATOM 1151 CG1 VAL 73 -7.234 3.643 4.694 1.00 0.00

ATOM 1152 HG11 VAL 73 -7.064 3.246 5.695 1.00 0.00

ATOM 1153 HG12 VAL 73 -8.144 3.207 4.281 1.00 0.00

ATOM 1154 HG13 VAL 73 -6.388 3.389 4.055 1.00 0.00

ATOM 1155 CG2 VAL 73 -8.621 5.521 5.586 1.00 0.00

ATOM 1156 HG21 VAL 73 -8.764 6.601 5.568 1.00 0.00

ATOM 1157 HG22 VAL 73 -9.497 5.031 5.160 1.00 0.00

ATOM 1158 HG23 VAL 73 -8.486 5.190 6.616 1.00 0.00

ATOM 1159 C VAL 73 -5.553 5.081 6.537 1.00 0.00

ATOM 1160 O VAL 73 -4.587 4.318 6.414 1.00 0.00

ATOM 1161 N GLN 74 -6.181 5.270 7.683 1.00 0.00

ATOM 1162 H GLN 74 -6.956 5.915 7.738 1.00 0.00

ATOM 1163 CA GLN 74 -5.737 4.625 8.901 1.00 0.00

ATOM 1164 HA GLN 74 -5.753 3.542 8.772 1.00 0.00

ATOM 1165 CB GLN 74 -4.307 5.056 9.247 1.00 0.00

ATOM 1166 HB2 GLN 74 -3.823 5.440 8.349 1.00 0.00

ATOM 1167 HB3 GLN 74 -4.344 5.840 10.003 1.00 0.00

ATOM 1168 CG GLN 74 -3.510 3.863 9.786 1.00 0.00

ATOM 1169 HG2 GLN 74 -4.204 3.176 10.271 1.00 0.00

ATOM 1170 HG3 GLN 74 -3.003 3.349 8.969 1.00 0.00

ATOM 1171 CD GLN 74 -2.468 4.302 10.805 1.00 0.00

ATOM 1172 OE1 GLN 74 -2.125 5.469 10.934 1.00 0.00

ATOM	1173	NE2	GLN	74	-1.937	3.335	11.531	1.00	0.00
ATOM	1174	HE21	GLN	74	-1.239	3.555	12.227	1.00	0.00
ATOM	1175	HE22	GLN	74	-2.232	2.379	11.387	1.00	0.00
ATOM	1176	C	GLN	74	-6.695	4.927	10.049	1.00	0.00
ATOM	1177	O	GLN	74	-6.299	5.103	11.195	1.00	0.00
ATOM	1178	N	GLY	75	-7.983	5.055	9.717	1.00	0.00
ATOM	1179	H	GLY	75	-8.264	4.950	8.752	1.00	0.00
ATOM	1180	CA	GLY	75	-9.004	5.342	10.702	1.00	0.00
ATOM	1181	HA2	GLY	75	-9.119	4.482	11.362	1.00	0.00
ATOM	1182	HA3	GLY	75	-8.705	6.211	11.287	1.00	0.00
ATOM	1183	C	GLY	75	-10.327	5.630	10.036	1.00	0.00
ATOM	1184	O	GLY	75	-11.315	4.954	10.306	1.00	0.00
ATOM	1185	N	GLN	76	-10.327	6.590	9.098	1.00	0.00
ATOM	1186	H	GLN	76	-9.475	7.096	8.905	1.00	0.00
ATOM	1187	CA	GLN	76	-11.529	6.973	8.387	1.00	0.00
ATOM	1188	HA	GLN	76	-11.841	6.154	7.739	1.00	0.00
ATOM	1189	CB	GLN	76	-12.674	7.296	9.355	1.00	0.00
ATOM	1190	HB2	GLN	76	-12.413	6.931	10.348	1.00	0.00
ATOM	1191	HB3	GLN	76	-12.816	8.376	9.390	1.00	0.00
ATOM	1192	CG	GLN	76	-13.970	6.627	8.890	1.00	0.00
ATOM	1193	HG2	GLN	76	-14.024	6.654	7.802	1.00	0.00
ATOM	1194	HG3	GLN	76	-13.997	5.591	9.228	1.00	0.00

ATOM	1195	CD	GLN	76	-15.178	7.353	9.454	1.00	0.00
ATOM	1196	OE1	GLN	76	-15.753	8.220	8.817	1.00	0.00
ATOM	1197	NE2	GLN	76	-15.550	6.988	10.664	1.00	0.00
ATOM	1198	HE21	GLN	76	-16.347	7.431	11.099	1.00	0.00
ATOM	1199	HE22	GLN	76	-15.037	6.267	11.151	1.00	0.00
ATOM	1200	C	GLN	76	-11.254	8.161	7.485	1.00	0.00
ATOM	1201	O	GLN	76	-11.075	9.288	7.934	1.00	0.00
ATOM	1202	N	HID	77	-11.137	7.893	6.186	1.00	0.00
ATOM	1203	H	HID	77	-11.121	6.930	5.882	1.00	0.00
ATOM	1204	CA	HID	77	-10.903	8.932	5.196	1.00	0.00
ATOM	1205	HA	HID	77	-11.072	9.916	5.633	1.00	0.00
ATOM	1206	CB	HID	77	-9.454	8.855	4.710	1.00	0.00
ATOM	1207	HB2	HID	77	-8.769	9.020	5.541	1.00	0.00
ATOM	1208	HB3	HID	77	-9.251	7.882	4.261	1.00	0.00
ATOM	1209	CG	HID	77	-9.123	9.888	3.667	1.00	0.00
ATOM	1210	ND1	HID	77	-8.503	11.090	3.932	1.00	0.00
ATOM	1211	HD1	HID	77	-8.209	11.412	4.843	1.00	0.00
ATOM	1212	CE1	HID	77	-8.358	11.758	2.771	1.00	0.00
ATOM	1213	HE1	HID	77	-7.877	12.736	2.756	1.00	0.00
ATOM	1214	NE2	HID	77	-8.882	11.018	1.779	1.00	0.00
ATOM	1215	CD2	HID	77	-9.358	9.835	2.315	1.00	0.00
ATOM	1216	HD2	HID	77	-9.813	9.086	1.666	1.00	0.00

ATOM 1217 C HID 77 -11.935 8.834 4.079 1.00 0.00

ATOM 1218 O HID 77 -11.824 9.502 3.062 1.00 0.00

ATOM 1219 N GLY 78 -12.944 7.982 4.264 1.00 0.00

ATOM 1220 H GLY 78 -13.000 7.459 5.126 1.00 0.00

ATOM 1221 CA GLY 78 -13.968 7.776 3.272 1.00 0.00

ATOM 1222 HA2 GLY 78 -14.633 6.979 3.607 1.00 0.00

ATOM 1223 HA3 GLY 78 -14.538 8.697 3.153 1.00 0.00

ATOM 1224 C GLY 78 -13.365 7.385 1.921 1.00 0.00

ATOM 1225 O GLY 78 -12.142 7.209 1.766 1.00 0.00

ATOM 1226 N PRO 79 -14.242 7.183 0.937 1.00 0.00

ATOM 1227 CD PRO 79 -15.689 7.311 1.038 1.00 0.00

ATOM 1228 HD2 PRO 79 -15.925 8.362 1.200 1.00 0.00

ATOM 1229 HD3 PRO 79 -16.021 6.728 1.897 1.00 0.00

ATOM 1230 CG PRO 79 -16.253 6.778 -0.283 1.00 0.00

ATOM 1231 HG2 PRO 79 -16.767 7.587 -0.801 1.00 0.00

ATOM 1232 HG3 PRO 79 -16.961 5.977 -0.069 1.00 0.00

ATOM 1233 CB PRO 79 -15.066 6.255 -1.101 1.00 0.00

ATOM 1234 HB2 PRO 79 -15.099 6.597 -2.136 1.00 0.00

ATOM 1235 HB3 PRO 79 -15.034 5.165 -1.080 1.00 0.00

ATOM 1236 CA PRO 79 -13.833 6.826 -0.404 1.00 0.00

ATOM 1237 HA PRO 79 -13.109 6.014 -0.333 1.00 0.00

ATOM 1238 C PRO 79 -13.210 7.994 -1.144 1.00 0.00

```
ATOM   1239  O   PRO    79     -13.912   8.844  -1.671  1.00  0.00
ATOM   1240  N   PHE    80     -11.872   8.033  -1.169  1.00  0.00
ATOM   1241  H   PHE    80     -11.334   7.298  -0.731  1.00  0.00
ATOM   1242  CA  PHE    80     -11.159   9.111  -1.818  1.00  0.00
ATOM   1243  HA  PHE    80     -11.718   9.500  -2.669  1.00  0.00
ATOM   1244  CB  PHE    80     -10.991  10.231  -0.791  1.00  0.00
ATOM   1245  HB2 PHE    80     -11.835  10.238  -0.101  1.00  0.00
ATOM   1246  HB3 PHE    80     -10.067  10.082  -0.233  1.00  0.00
ATOM   1247  CG  PHE    80     -10.914  11.608  -1.392  1.00  0.00
ATOM   1248  CD1 PHE    80     -12.084  12.249  -1.821  1.00  0.00
ATOM   1249  HD1 PHE    80     -13.045  11.745  -1.720  1.00  0.00
ATOM   1250  CE1 PHE    80     -12.021  13.536  -2.380  1.00  0.00
ATOM   1251  HE1 PHE    80     -12.937  14.026  -2.711  1.00  0.00
ATOM   1252  CZ  PHE    80     -10.788  14.190  -2.514  1.00  0.00
ATOM   1253  HZ  PHE    80     -10.733  15.187  -2.950  1.00  0.00
ATOM   1254  CE2 PHE    80      -9.618  13.548  -2.081  1.00  0.00
ATOM   1255  HE2 PHE    80      -8.657  14.052  -2.180  1.00  0.00
ATOM   1256  CD2 PHE    80      -9.682  12.262  -1.522  1.00  0.00
ATOM   1257  HD2 PHE    80      -8.770  11.767  -1.187  1.00  0.00
ATOM   1258  C   PHE    80      -9.796   8.645  -2.296  1.00  0.00
ATOM   1259  O   PHE    80      -9.500   8.611  -3.479  1.00  0.00
ATOM   1260  N   GLN    81      -8.948   8.248  -1.339  1.00  0.00
```

ATOM	1261	H	GLN	81	-9.254	8.249	-0.377	1.00	0.00
ATOM	1262	CA	GLN	81	-7.597	7.832	-1.627	1.00	0.00
ATOM	1263	HA	GLN	81	-7.139	8.549	-2.308	1.00	0.00
ATOM	1264	CB	GLN	81	-6.751	7.751	-0.363	1.00	0.00
ATOM	1265	HB2	GLN	81	-7.105	6.915	0.241	1.00	0.00
ATOM	1266	HB3	GLN	81	-5.713	7.578	-0.648	1.00	0.00
ATOM	1267	CG	GLN	81	-6.848	9.038	0.445	1.00	0.00
ATOM	1268	HG2	GLN	81	-5.994	9.665	0.189	1.00	0.00
ATOM	1269	HG3	GLN	81	-7.771	9.569	0.213	1.00	0.00
ATOM	1270	CD	GLN	81	-6.813	8.756	1.934	1.00	0.00
ATOM	1271	OE1	GLN	81	-7.137	7.693	2.431	1.00	0.00
ATOM	1272	NE2	GLN	81	-6.431	9.763	2.688	1.00	0.00
ATOM	1273	HE21	GLN	81	-6.383	9.649	3.690	1.00	0.00
ATOM	1274	HE22	GLN	81	-6.187	10.645	2.261	1.00	0.00
ATOM	1275	C	GLN	81	-7.666	6.492	-2.282	1.00	0.00
ATOM	1276	O	GLN	81	-7.214	6.407	-3.392	1.00	0.00
ATOM	1277	N	SER	82	-8.280	5.490	-1.641	1.00	0.00
ATOM	1278	H	SER	82	-8.679	5.677	-0.733	1.00	0.00
ATOM	1279	CA	SER	82	-8.398	4.147	-2.181	1.00	0.00
ATOM	1280	HA	SER	82	-7.467	3.603	-2.021	1.00	0.00
ATOM	1281	CB	SER	82	-9.530	3.389	-1.495	1.00	0.00
ATOM	1282	HB2	SER	82	-9.267	3.276	-0.444	1.00	0.00

```
ATOM   1283  HB3 SER    82     -10.434   3.991  -1.580  1.00  0.00
ATOM   1284  OG  SER    82      -9.757   2.121  -2.060  1.00  0.00
ATOM   1285  HG  SER    82     -10.476   1.687  -1.595  1.00  0.00
ATOM   1286  C   SER    82      -8.683   4.193  -3.665  1.00  0.00
ATOM   1287  O   SER    82      -7.980   3.569  -4.416  1.00  0.00
ATOM   1288  N   THR    83      -9.620   5.039  -4.081  1.00  0.00
ATOM   1289  H   THR    83     -10.187   5.514  -3.393  1.00  0.00
ATOM   1290  CA  THR    83      -9.942   5.222  -5.479  1.00  0.00
ATOM   1291  HA  THR    83     -10.521   4.355  -5.798  1.00  0.00
ATOM   1292  CB  THR    83     -10.809   6.471  -5.632  1.00  0.00
ATOM   1293  HB  THR    83     -10.634   7.098  -4.758  1.00  0.00
ATOM   1294  CG2 THR    83     -10.512   7.269  -6.898  1.00  0.00
ATOM   1295 HG21 THR    83     -10.687   6.642  -7.773  1.00  0.00
ATOM   1296 HG22 THR    83     -11.165   8.141  -6.940  1.00  0.00
ATOM   1297 HG23 THR    83      -9.472   7.594  -6.887  1.00  0.00
ATOM   1298  OG1 THR    83     -12.124   6.015  -5.756  1.00  0.00
ATOM   1299  HG1 THR    83     -12.715   6.765  -5.856  1.00  0.00
ATOM   1300  C   THR    83      -8.687   5.259  -6.341  1.00  0.00
ATOM   1301  O   THR    83      -8.362   4.298  -7.040  1.00  0.00
ATOM   1302  N   ARG    84      -7.930   6.359  -6.247  1.00  0.00
ATOM   1303  H   ARG    84      -8.210   7.101  -5.623  1.00  0.00
ATOM   1304  CA  ARG    84      -6.733   6.533  -7.016  1.00  0.00
```

ATOM 1305 HA ARG 84 -6.930 6.287 -8.060 1.00 0.00
ATOM 1306 CB ARG 84 -6.241 7.979 -6.939 1.00 0.00
ATOM 1307 HB2 ARG 84 -6.910 8.544 -6.290 1.00 0.00
ATOM 1308 HB3 ARG 84 -5.235 7.987 -6.519 1.00 0.00
ATOM 1309 CG ARG 84 -6.218 8.620 -8.332 1.00 0.00
ATOM 1310 HG2 ARG 84 -7.247 8.804 -8.640 1.00 0.00
ATOM 1311 HG3 ARG 84 -5.684 9.568 -8.263 1.00 0.00
ATOM 1312 CD ARG 84 -5.530 7.721 -9.364 1.00 0.00
ATOM 1313 HD2 ARG 84 -5.061 6.878 -8.856 1.00 0.00
ATOM 1314 HD3 ARG 84 -6.270 7.350 -10.074 1.00 0.00
ATOM 1315 NE ARG 84 -4.495 8.461 -10.105 1.00 0.00
ATOM 1316 HE ARG 84 -4.163 9.309 -9.668 1.00 0.00
ATOM 1317 CZ ARG 84 -3.993 8.118 -11.274 1.00 0.00
ATOM 1318 NH1 ARG 84 -4.454 7.065 -11.908 1.00 0.00
ATOM 1319 HH11 ARG 84 -5.196 6.516 -11.497 1.00 0.00
ATOM 1320 HH12 ARG 84 -4.065 6.808 -12.804 1.00 0.00
ATOM 1321 NH2 ARG 84 -3.016 8.817 -11.804 1.00 0.00
ATOM 1322 HH21 ARG 84 -2.649 9.619 -11.313 1.00 0.00
ATOM 1323 HH22 ARG 84 -2.636 8.548 -12.701 1.00 0.00
ATOM 1324 C ARG 84 -5.662 5.618 -6.497 1.00 0.00
ATOM 1325 O ARG 84 -4.943 5.023 -7.280 1.00 0.00
ATOM 1326 N ILE 85 -5.497 5.549 -5.184 1.00 0.00

ATOM	1327	H	ILE	85	-6.032	6.196	-4.623	1.00	0.00
ATOM	1328	CA	ILE	85	-4.549	4.695	-4.522	1.00	0.00
ATOM	1329	HA	ILE	85	-3.517	5.007	-4.682	1.00	0.00
ATOM	1330	CB	ILE	85	-4.831	4.612	-3.016	1.00	0.00
ATOM	1331	HB	ILE	85	-5.909	4.654	-2.859	1.00	0.00
ATOM	1332	CG2	ILE	85	-4.283	3.318	-2.428	1.00	0.00
ATOM	1333	HG21	ILE	85	-3.205	3.275	-2.584	1.00	0.00
ATOM	1334	HG22	ILE	85	-4.496	3.284	-1.360	1.00	0.00
ATOM	1335	HG23	ILE	85	-4.755	2.466	-2.919	1.00	0.00
ATOM	1336	CG1	ILE	85	-4.289	5.854	-2.287	1.00	0.00
ATOM	1337	HG12	ILE	85	-4.830	6.729	-2.646	1.00	0.00
ATOM	1338	HG13	ILE	85	-4.465	5.732	-1.218	1.00	0.00
ATOM	1339	CD1	ILE	85	-2.794	6.041	-2.539	1.00	0.00
ATOM	1340	HD11	ILE	85	-2.618	6.163	-3.608	1.00	0.00
ATOM	1341	HD12	ILE	85	-2.444	6.927	-2.010	1.00	0.00
ATOM	1342	HD13	ILE	85	-2.252	5.166	-2.180	1.00	0.00
ATOM	1343	C	ILE	85	-4.543	3.330	-5.181	1.00	0.00
ATOM	1344	O	ILE	85	-3.489	2.748	-5.332	1.00	0.00
ATOM	1345	N	TYR	86	-5.689	2.873	-5.660	1.00	0.00
ATOM	1346	H	TYR	86	-6.504	3.459	-5.550	1.00	0.00
ATOM	1347	CA	TYR	86	-5.837	1.629	-6.346	1.00	0.00
ATOM	1348	HA	TYR	86	-5.151	0.875	-5.960	1.00	0.00

ATOM	1349	CB	TYR	86	-7.263	1.156	-6.107	1.00	0.00
ATOM	1350	HB2	TYR	86	-7.883	2.051	-6.067	1.00	0.00
ATOM	1351	HB3	TYR	86	-7.555	0.549	-6.965	1.00	0.00
ATOM	1352	CG	TYR	86	-7.451	0.355	-4.835	1.00	0.00
ATOM	1353	CD1	TYR	86	-7.139	0.914	-3.584	1.00	0.00
ATOM	1354	HD1	TYR	86	-6.762	1.935	-3.538	1.00	0.00
ATOM	1355	CE1	TYR	86	-7.307	0.174	-2.404	1.00	0.00
ATOM	1356	HE1	TYR	86	-7.028	0.591	-1.437	1.00	0.00
ATOM	1357	CZ	TYR	86	-7.792	-1.139	-2.488	1.00	0.00
ATOM	1358	OH	TYR	86	-8.004	-1.832	-1.344	1.00	0.00
ATOM	1359	HH	TYR	86	-7.790	-1.329	-0.555	1.00	0.00
ATOM	1360	CE2	TYR	86	-8.107	-1.718	-3.729	1.00	0.00
ATOM	1361	HE2	TYR	86	-8.485	-2.739	-3.788	1.00	0.00
ATOM	1362	CD2	TYR	86	-7.929	-0.965	-4.905	1.00	0.00
ATOM	1363	HD2	TYR	86	-8.162	-1.406	-5.875	1.00	0.00
ATOM	1364	C	TYR	86	-5.630	1.796	-7.827	1.00	0.00
ATOM	1365	O	TYR	86	-4.963	0.994	-8.461	1.00	0.00
ATOM	1366	N	GLN	87	-6.135	2.879	-8.410	1.00	0.00
ATOM	1367	H	GLN	87	-6.695	3.507	-7.851	1.00	0.00
ATOM	1368	CA	GLN	87	-5.967	3.176	-9.808	1.00	0.00
ATOM	1369	HA	GLN	87	-6.297	2.327	-10.407	1.00	0.00
ATOM	1370	CB	GLN	87	-6.791	4.398	-10.199	1.00	0.00

ATOM 1371 HB2 GLN 87 -7.512 4.605 -9.409 1.00 0.00
ATOM 1372 HB3 GLN 87 -6.123 5.252 -10.314 1.00 0.00
ATOM 1373 CG GLN 87 -7.533 4.151 -11.514 1.00 0.00
ATOM 1374 HG2 GLN 87 -6.915 4.212 -12.410 1.00 0.00
ATOM 1375 HG3 GLN 87 -7.908 3.134 -11.401 1.00 0.00
ATOM 1376 CD GLN 87 -8.719 5.092 -11.675 1.00 0.00
ATOM 1377 OE1 GLN 87 -8.769 6.158 -11.072 1.00 0.00
ATOM 1378 NE2 GLN 87 -9.585 4.758 -12.597 1.00 0.00
ATOM 1379 HE21 GLN 87 -10.396 5.338 -12.758 1.00 0.00
ATOM 1380 HE22 GLN 87 -9.438 3.921 -13.144 1.00 0.00
ATOM 1381 C GLN 87 -4.504 3.405 -10.137 1.00 0.00
ATOM 1382 O GLN 87 -4.129 3.503 -11.299 1.00 0.00
ATOM 1383 N ILE 88 -3.639 3.514 -9.143 1.00 0.00
ATOM 1384 H ILE 88 -4.012 3.520 -8.204 1.00 0.00
ATOM 1385 CA ILE 88 -2.226 3.696 -9.315 1.00 0.00
ATOM 1386 HA ILE 88 -1.950 3.696 -10.370 1.00 0.00
ATOM 1387 CB ILE 88 -1.800 5.019 -8.676 1.00 0.00
ATOM 1388 HB ILE 88 -2.143 5.037 -7.642 1.00 0.00
ATOM 1389 CG2 ILE 88 -0.281 5.171 -8.711 1.00 0.00
ATOM 1390 HG21 ILE 88 0.063 5.153 -9.745 1.00 0.00
ATOM 1391 HG22 ILE 88 -0.000 6.118 -8.252 1.00 0.00
ATOM 1392 HG23 ILE 88 0.180 4.350 -8.161 1.00 0.00

ATOM 1393 CG1 ILE 88 -2.466 6.198 -9.390 1.00 0.00
ATOM 1394 HG12 ILE 88 -1.923 6.413 -10.310 1.00 0.00
ATOM 1395 HG13 ILE 88 -3.497 5.936 -9.630 1.00 0.00
ATOM 1396 CD1 ILE 88 -2.451 7.438 -8.485 1.00 0.00
ATOM 1397 HD11 ILE 88 -1.421 7.700 -8.245 1.00 0.00
ATOM 1398 HD12 ILE 88 -2.927 8.271 -9.002 1.00 0.00
ATOM 1399 HD13 ILE 88 -2.995 7.223 -7.565 1.00 0.00
ATOM 1400 C ILE 88 -1.501 2.504 -8.725 1.00 0.00
ATOM 1401 O ILE 88 -0.318 2.348 -8.938 1.00 0.00
ATOM 1402 N ALA 89 -2.201 1.643 -8.007 1.00 0.00
ATOM 1403 H ALA 89 -3.166 1.860 -7.800 1.00 0.00
ATOM 1404 CA ALA 89 -1.634 0.445 -7.441 1.00 0.00
ATOM 1405 HA ALA 89 -0.545 0.492 -7.468 1.00 0.00
ATOM 1406 CB ALA 89 -2.079 0.324 -5.993 1.00 0.00
ATOM 1407 HB1 ALA 89 -3.167 0.266 -5.950 1.00 0.00
ATOM 1408 HB2 ALA 89 -1.651 -0.578 -5.555 1.00 0.00
ATOM 1409 HB3 ALA 89 -1.740 1.196 -5.434 1.00 0.00
ATOM 1410 C ALA 89 -2.034 -0.763 -8.271 1.00 0.00
ATOM 1411 O ALA 89 -1.698 -1.897 -7.942 1.00 0.00
ATOM 1412 N LYS 90 -2.749 -0.523 -9.376 1.00 0.00
ATOM 1413 H LYS 90 -3.054 0.422 -9.561 1.00 0.00
ATOM 1414 CA LYS 90 -3.200 -1.576 -10.262 1.00 0.00

ATOM	1415	HA	LYS	90	-3.153	-2.537	-9.750	1.00	0.00
ATOM	1416	CB	LYS	90	-4.650	-1.322	-10.696	1.00	0.00
ATOM	1417	HB2	LYS	90	-5.167	-2.282	-10.712	1.00	0.00
ATOM	1418	HB3	LYS	90	-5.112	-0.673	-9.952	1.00	0.00
ATOM	1419	CG	LYS	90	-4.748	-0.672	-12.066	1.00	0.00
ATOM	1420	HG2	LYS	90	-4.165	-1.263	-12.773	1.00	0.00
ATOM	1421	HG3	LYS	90	-5.794	-0.668	-12.374	1.00	0.00
ATOM	1422	CD	LYS	90	-4.224	0.746	-12.041	1.00	0.00
ATOM	1423	HD2	LYS	90	-4.909	1.364	-11.460	1.00	0.00
ATOM	1424	HD3	LYS	90	-3.242	0.749	-11.568	1.00	0.00
ATOM	1425	CE	LYS	90	-4.109	1.307	-13.457	1.00	0.00
ATOM	1426	HE2	LYS	90	-3.684	0.538	-14.103	1.00	0.00
ATOM	1427	HE3	LYS	90	-5.107	1.566	-13.811	1.00	0.00
ATOM	1428	NZ	LYS	90	-3.251	2.504	-13.488	1.00	0.00
ATOM	1429	HZ1	LYS	90	-2.326	2.264	-13.161	1.00	0.00
ATOM	1430	HZ2	LYS	90	-3.193	2.852	-14.434	1.00	0.00
ATOM	1431	HZ3	LYS	90	-3.645	3.216	-12.891	1.00	0.00
ATOM	1432	C	LYS	90	-2.243	-1.696	-11.419	1.00	0.00
ATOM	1433	O	LYS	90	-2.261	-2.670	-12.153	1.00	0.00
ATOM	1434	N	ASN	91	-1.439	-0.647	-11.637	1.00	0.00
ATOM	1435	H	ASN	91	-1.536	0.168	-11.049	1.00	0.00
ATOM	1436	CA	ASN	91	-0.457	-0.618	-12.684	1.00	0.00

```
ATOM   1437  HA  ASN    91      -0.778  -1.255 -13.509  1.00  0.00
ATOM   1438  CB  ASN    91      -0.274   0.807 -13.209  1.00  0.00
ATOM   1439  HB2 ASN    91       0.604   0.795 -13.854  1.00  0.00
ATOM   1440  HB3 ASN    91      -1.143   1.125 -13.785  1.00  0.00
ATOM   1441  CG  ASN    91      -0.043   1.798 -12.100  1.00  0.00
ATOM   1442  OD1 ASN    91       0.599   1.497 -11.123  1.00  0.00
ATOM   1443  ND2 ASN    91      -0.628   2.971 -12.252  1.00  0.00
ATOM   1444 HD21 ASN    91      -0.515   3.683 -11.545  1.00  0.00
ATOM   1445 HD22 ASN    91      -1.186   3.150 -13.075  1.00  0.00
ATOM   1446  C   ASN    91       0.881  -1.141 -12.171  1.00  0.00
ATOM   1447  O   ASN    91       1.877  -1.106 -12.885  1.00  0.00
ATOM   1448  N   LEU    92       0.916  -1.544 -10.905  1.00  0.00
ATOM   1449  H   LEU    92       0.097  -1.415 -10.328  1.00  0.00
ATOM   1450  CA  LEU    92       2.098  -2.094 -10.297  1.00  0.00
ATOM   1451  HA  LEU    92       2.970  -1.499 -10.567  1.00  0.00
ATOM   1452  CB  LEU    92       1.950  -2.083  -8.769  1.00  0.00
ATOM   1453  HB2 LEU    92       0.873  -2.066  -8.604  1.00  0.00
ATOM   1454  HB3 LEU    92       2.368  -2.996  -8.344  1.00  0.00
ATOM   1455  CG  LEU    92       2.590  -0.865  -8.093  1.00  0.00
ATOM   1456  HG  LEU    92       2.408   0.019  -8.704  1.00  0.00
ATOM   1457  CD1 LEU    92       1.989  -0.655  -6.701  1.00  0.00
ATOM   1458 HD11 LEU    92       2.171  -1.539  -6.089  1.00  0.00
```

```
ATOM   1459 HD12 LEU    92       2.452   0.213  -6.232  1.00  0.00
ATOM   1460 HD13 LEU    92       0.915  -0.490  -6.790  1.00  0.00
ATOM   1461  CD2 LEU    92       4.106  -1.009  -8.024  1.00  0.00
ATOM   1462 HD21 LEU    92       4.509  -1.100  -9.033  1.00  0.00
ATOM   1463 HD22 LEU    92       4.533  -0.131  -7.540  1.00  0.00
ATOM   1464 HD23 LEU    92       4.361  -1.900  -7.450  1.00  0.00
ATOM   1465  C   LEU    92       2.354  -3.493 -10.827  1.00  0.00
ATOM   1466  O   LEU    92       3.436  -3.752 -11.312  1.00  0.00
ATOM   1467  N   PRO    93       1.338  -4.370 -10.816  1.00  0.00
ATOM   1468  CD  PRO    93      -0.006  -4.119 -10.323  1.00  0.00
ATOM   1469  HD2 PRO    93      -0.467  -3.389 -10.988  1.00  0.00
ATOM   1470  HD3 PRO    93       0.086  -3.693  -9.324  1.00  0.00
ATOM   1471  CG  PRO    93      -0.737  -5.444 -10.320  1.00  0.00
ATOM   1472  HG2 PRO    93      -1.577  -5.393 -11.013  1.00  0.00
ATOM   1473  HG3 PRO    93      -1.108  -5.648  -9.316  1.00  0.00
ATOM   1474  CB  PRO    93       0.281  -6.502 -10.752  1.00  0.00
ATOM   1475  HB2 PRO    93      -0.130  -7.177 -11.503  1.00  0.00
ATOM   1476  HB3 PRO    93       0.619  -7.079  -9.892  1.00  0.00
ATOM   1477  CA  PRO    93       1.450  -5.716 -11.345  1.00  0.00
ATOM   1478  HA  PRO    93       2.367  -6.156 -10.952  1.00  0.00
ATOM   1479  C   PRO    93       1.485  -5.746 -12.863  1.00  0.00
ATOM   1480  O   PRO    93       1.608  -6.813 -13.460  1.00  0.00
```

```
ATOM   1481  N   ASN    94       1.378  -4.563 -13.473  1.00  0.00
ATOM   1482  H   ASN    94       1.275  -3.724 -12.920  1.00  0.00
ATOM   1483  CA  ASN    94       1.409  -4.437 -14.900  1.00  0.00
ATOM   1484  HA  ASN    94       0.972  -5.322 -15.363  1.00  0.00
ATOM   1485  CB  ASN    94       0.604  -3.211 -15.334  1.00  0.00
ATOM   1486  HB2 ASN    94       0.834  -2.376 -14.673  1.00  0.00
ATOM   1487  HB3 ASN    94       0.884  -2.953 -16.355  1.00  0.00
ATOM   1488  CG  ASN    94      -0.885  -3.466 -15.291  1.00  0.00
ATOM   1489  OD1 ASN    94      -1.361  -4.586 -15.224  1.00  0.00
ATOM   1490  ND2 ASN    94      -1.642  -2.384 -15.323  1.00  0.00
ATOM   1491 HD21 ASN    94      -2.648  -2.474 -15.298  1.00  0.00
ATOM   1492 HD22 ASN    94      -1.212  -1.472 -15.373  1.00  0.00
ATOM   1493  C   ASN    94       2.838  -4.274 -15.376  1.00  0.00
ATOM   1494  O   ASN    94       3.225  -4.817 -16.403  1.00  0.00
ATOM   1495  N   VAL    95       3.620  -3.501 -14.620  1.00  0.00
ATOM   1496  H   VAL    95       3.224  -3.022 -13.824  1.00  0.00
ATOM   1497  CA  VAL    95       5.013  -3.261 -14.943  1.00  0.00
ATOM   1498  HA  VAL    95       5.274  -3.626 -15.937  1.00  0.00
ATOM   1499  CB  VAL    95       5.302  -1.760 -14.881  1.00  0.00
ATOM   1500  HB  VAL    95       5.167  -1.420 -13.854  1.00  0.00
ATOM   1501  CG1 VAL    95       6.729  -1.458 -15.334  1.00  0.00
ATOM   1502 HG11 VAL    95       6.864  -1.798 -16.361  1.00  0.00
```

ATOM 1503 HG12 VAL 95 6.908 -0.385 -15.280 1.00 0.00

ATOM 1504 HG13 VAL 95 7.434 -1.977 -14.685 1.00 0.00

ATOM 1505 CG2 VAL 95 4.300 -0.982 -15.746 1.00 0.00

ATOM 1506 HG21 VAL 95 3.288 -1.163 -15.382 1.00 0.00

ATOM 1507 HG22 VAL 95 4.521 0.083 -15.690 1.00 0.00

ATOM 1508 HG23 VAL 95 4.378 -1.315 -16.781 1.00 0.00

ATOM 1509 C VAL 95 5.910 -4.052 -14.003 1.00 0.00

ATOM 1510 O VAL 95 7.117 -4.118 -14.185 1.00 0.00

ATOM 1511 N CYX 96 5.305 -4.711 -13.019 1.00 0.00

ATOM 1512 H CYX 96 4.308 -4.599 -12.907 1.00 0.00

ATOM 1513 CA CYX 96 6.014 -5.503 -12.054 1.00 0.00

ATOM 1514 HA CYX 96 7.053 -5.599 -12.369 1.00 0.00

ATOM 1515 CB CYX 96 5.982 -4.878 -10.659 1.00 0.00

ATOM 1516 HB2 CYX 96 4.979 -4.970 -10.242 1.00 0.00

ATOM 1517 HB3 CYX 96 6.692 -5.394 -10.013 1.00 0.00

ATOM 1518 SG CYX 96 6.403 -3.143 -10.624 1.00 0.00

ATOM 1519 C CYX 96 5.434 -6.879 -12.074 1.00 0.00

ATOM 1520 O CYX 96 5.308 -7.413 -10.998 1.00 0.00

ATOM 1521 N ASN 97 5.057 -7.402 -13.252 1.00 0.00

ATOM 1522 H ASN 97 5.186 -6.836 -14.078 1.00 0.00

ATOM 1523 CA ASN 97 4.480 -8.720 -13.407 1.00 0.00

ATOM 1524 HA ASN 97 3.393 -8.645 -13.432 1.00 0.00

```
ATOM   1525  CB  ASN    97       4.954  -9.368 -14.708  1.00  0.00
ATOM   1526  HB2 ASN    97       5.936  -8.987 -14.988  1.00  0.00
ATOM   1527  HB3 ASN    97       5.016 -10.444 -14.543  1.00  0.00
ATOM   1528  CG  ASN    97       3.998  -9.110 -15.840  1.00  0.00
ATOM   1529  OD1 ASN    97       3.267  -9.980 -16.283  1.00  0.00
ATOM   1530  ND2 ASN    97       3.988  -7.874 -16.289  1.00  0.00
ATOM   1531 HD21 ASN    97       3.372  -7.621 -17.049  1.00  0.00
ATOM   1532 HD22 ASN    97       4.596  -7.184 -15.872  1.00  0.00
ATOM   1533  C   ASN    97       4.876  -9.639 -12.253  1.00  0.00
ATOM   1534  O   ASN    97       5.979 -10.186 -12.247  1.00  0.00
ATOM   1535  N   MET    98       4.007  -9.718 -11.248  1.00  0.00
ATOM   1536  H   MET    98       3.132  -9.216 -11.300  1.00  0.00
ATOM   1537  CA  MET    98       4.307 -10.448 -10.037  1.00  0.00
ATOM   1538  HA  MET    98       5.155 -11.109 -10.219  1.00  0.00
ATOM   1539  CB  MET    98       4.656  -9.513  -8.881  1.00  0.00
ATOM   1540  HB2 MET    98       4.995 -10.113  -8.036  1.00  0.00
ATOM   1541  HB3 MET    98       5.458  -8.847  -9.199  1.00  0.00
ATOM   1542  CG  MET    98       3.445  -8.686  -8.463  1.00  0.00
ATOM   1543  HG2 MET    98       2.581  -9.035  -9.028  1.00  0.00
ATOM   1544  HG3 MET    98       3.271  -8.847  -7.399  1.00  0.00
ATOM   1545  SD  MET    98       3.678  -6.941  -8.763  1.00  0.00
ATOM   1546  CE  MET    98       2.345  -6.246  -7.829  1.00  0.00
```

```
ATOM   1547  HE1 MET    98       2.453  -6.523  -6.780  1.00  0.00
ATOM   1548  HE2 MET    98       2.363  -5.160  -7.922  1.00  0.00
ATOM   1549  HE3 MET    98       1.397  -6.627  -8.209  1.00  0.00
ATOM   1550  C   MET    98       3.132 -11.329  -9.694  1.00  0.00
ATOM   1551  O   MET    98       3.061 -11.897  -8.614  1.00  0.00
ATOM   1552  N   LYS    99       2.148 -11.409 -10.598  1.00  0.00
ATOM   1553  H   LYS    99       2.195 -10.800 -11.403  1.00  0.00
ATOM   1554  CA  LYS    99       0.959 -12.193 -10.406  1.00  0.00
ATOM   1555  HA  LYS    99       1.159 -13.019  -9.723  1.00  0.00
ATOM   1556  CB  LYS    99      -0.149 -11.316  -9.804  1.00  0.00
ATOM   1557  HB2 LYS    99      -0.079 -11.367  -8.718  1.00  0.00
ATOM   1558  HB3 LYS    99       0.008 -10.288 -10.130  1.00  0.00
ATOM   1559  CG  LYS    99      -1.541 -11.787 -10.249  1.00  0.00
ATOM   1560  HG2 LYS    99      -1.476 -12.821 -10.589  1.00  0.00
ATOM   1561  HG3 LYS    99      -2.225 -11.726  -9.403  1.00  0.00
ATOM   1562  CD  LYS    99      -2.064 -10.900 -11.398  1.00  0.00
ATOM   1563  HD2 LYS    99      -2.781 -10.188 -10.990  1.00  0.00
ATOM   1564  HD3 LYS    99      -1.223 -10.360 -11.832  1.00  0.00
ATOM   1565  CE  LYS    99      -2.747 -11.749 -12.489  1.00  0.00
ATOM   1566  HE2 LYS    99      -2.584 -12.803 -12.262  1.00  0.00
ATOM   1567  HE3 LYS    99      -3.816 -11.536 -12.477  1.00  0.00
ATOM   1568  NZ  LYS    99      -2.199 -11.443 -13.828  1.00  0.00
```

```
ATOM   1569  HZ1 LYS    99      -1.209 -11.641 -13.840  1.00  0.00
ATOM   1570  HZ2 LYS    99      -2.664 -12.013 -14.520  1.00  0.00
ATOM   1571  HZ3 LYS    99      -2.350 -10.467 -14.039  1.00  0.00
ATOM   1572  C   LYS    99       0.623 -12.884 -11.708  1.00  0.00
ATOM   1573  O   LYS    99      -0.066 -12.351 -12.554  1.00  0.00
ATOM   1574  N   GLN   100       1.128 -14.076 -11.917  1.00  0.00
ATOM   1575  H   GLN   100       1.771 -14.481 -11.252  1.00  0.00
ATOM   1576  CA  GLN   100       0.832 -14.817 -13.121  1.00  0.00
ATOM   1577  HA  GLN   100       0.900 -14.164 -13.991  1.00  0.00
ATOM   1578  CB  GLN   100       1.840 -15.960 -13.286  1.00  0.00
ATOM   1579  HB2 GLN   100       2.749 -15.717 -12.736  1.00  0.00
ATOM   1580  HB3 GLN   100       1.408 -16.878 -12.886  1.00  0.00
ATOM   1581  CG  GLN   100       2.178 -16.158 -14.768  1.00  0.00
ATOM   1582  HG2 GLN   100       2.311 -15.166 -15.199  1.00  0.00
ATOM   1583  HG3 GLN   100       3.100 -16.729 -14.879  1.00  0.00
ATOM   1584  CD  GLN   100       1.068 -16.874 -15.528  1.00  0.00
ATOM   1585  OE1 GLN   100       0.008 -17.183 -15.022  1.00  0.00
ATOM   1586  NE2 GLN   100       1.332 -17.192 -16.773  1.00  0.00
ATOM   1587 HE21 GLN   100       0.635 -17.668 -17.328  1.00  0.00
ATOM   1588 HE22 GLN   100       2.231 -16.960 -17.171  1.00  0.00
ATOM   1589  C   GLN   100      -0.592 -15.348 -13.083  1.00  0.00
ATOM   1590  O   GLN   100      -1.353 -15.137 -14.020  1.00  0.00
```

ATOM	1591	N	ILE	101	-0.943	-15.991	-11.969	1.00	0.00
ATOM	1592	H	ILE	101	-0.245	-16.180	-11.264	1.00	0.00
ATOM	1593	CA	ILE	101	-2.260	-16.541	-11.789	1.00	0.00
ATOM	1594	HA	ILE	101	-2.569	-17.154	-12.636	1.00	0.00
ATOM	1595	CB	ILE	101	-2.338	-17.375	-10.497	1.00	0.00
ATOM	1596	HB	ILE	101	-1.912	-18.360	-10.687	1.00	0.00
ATOM	1597	CG2	ILE	101	-1.569	-16.698	-9.373	1.00	0.00
ATOM	1598	HG21	ILE	101	-1.995	-15.713	-9.183	1.00	0.00
ATOM	1599	HG22	ILE	101	-1.637	-17.304	-8.470	1.00	0.00
ATOM	1600	HG23	ILE	101	-0.523	-16.592	-9.661	1.00	0.00
ATOM	1601	CG1	ILE	101	-3.793	-17.650	-10.083	1.00	0.00
ATOM	1602	HG12	ILE	101	-3.812	-18.510	-9.413	1.00	0.00
ATOM	1603	HG13	ILE	101	-4.182	-16.776	-9.562	1.00	0.00
ATOM	1604	CD1	ILE	101	-4.662	-17.941	-11.313	1.00	0.00
ATOM	1605	HD11	ILE	101	-4.273	-18.816	-11.834	1.00	0.00
ATOM	1606	HD12	ILE	101	-5.687	-18.133	-10.997	1.00	0.00
ATOM	1607	HD13	ILE	101	-4.644	-17.082	-11.983	1.00	0.00
ATOM	1608	C	ILE	101	-3.291	-15.410	-11.852	1.00	0.00
ATOM	1609	O	ILE	101	-3.815	-15.076	-12.909	1.00	0.00
ATOM	1610	N	GLY	102	-3.590	-14.795	-10.703	1.00	0.00
ATOM	1611	H	GLY	102	-3.097	-15.061	-9.862	1.00	0.00
ATOM	1612	CA	GLY	102	-4.588	-13.766	-10.611	1.00	0.00

```
ATOM   1613  HA2 GLY   102      -4.121 -12.804 -10.822  1.00  0.00
ATOM   1614  HA3 GLY   102      -5.364 -13.963 -11.351  1.00  0.00
ATOM   1615  C   GLY   102      -5.216 -13.716  -9.245  1.00  0.00
ATOM   1616  O   GLY   102      -6.314 -14.182  -9.010  1.00  0.00
ATOM   1617  N   THR   103      -4.503 -13.124  -8.307  1.00  0.00
ATOM   1618  H   THR   103      -3.587 -12.777  -8.552  1.00  0.00
ATOM   1619  CA  THR   103      -4.936 -13.013  -6.919  1.00  0.00
ATOM   1620  HA  THR   103      -5.494 -13.925  -6.705  1.00  0.00
ATOM   1621  CB  THR   103      -3.696 -12.976  -6.022  1.00  0.00
ATOM   1622  HB  THR   103      -3.712 -12.047  -5.453  1.00  0.00
ATOM   1623  CG2 THR   103      -3.659 -14.161  -5.060  1.00  0.00
ATOM   1624 HG21 THR   103      -3.643 -15.091  -5.629  1.00  0.00
ATOM   1625 HG22 THR   103      -2.764 -14.099  -4.441  1.00  0.00
ATOM   1626 HG23 THR   103      -4.543 -14.141  -4.423  1.00  0.00
ATOM   1627  OG1 THR   103      -2.544 -13.153  -6.846  1.00  0.00
ATOM   1628  HG1 THR   103      -1.754 -13.133  -6.301  1.00  0.00
ATOM   1629  C   THR   103      -5.815 -11.789  -6.753  1.00  0.00
ATOM   1630  O   THR   103      -6.933 -11.886  -6.249  1.00  0.00
ATOM   1631  N   CYX   104      -5.327 -10.630  -7.216  1.00  0.00
ATOM   1632  H   CYX   104      -4.368 -10.593  -7.531  1.00  0.00
ATOM   1633  CA  CYX   104      -6.089  -9.397  -7.187  1.00  0.00
ATOM   1634  HA  CYX   104      -7.128  -9.609  -6.935  1.00  0.00
```

ATOM 1635 CB CYX 104 -5.515 -8.442 -6.136 1.00 0.00
ATOM 1636 HB2 CYX 104 -6.238 -7.654 -5.927 1.00 0.00
ATOM 1637 HB3 CYX 104 -5.305 -8.994 -5.220 1.00 0.00
ATOM 1638 SG CYX 104 -3.940 -7.595 -6.552 1.00 0.00
ATOM 1639 C CYX 104 -6.125 -8.795 -8.584 1.00 0.00
ATOM 1640 O CYX 104 -5.514 -7.768 -8.853 1.00 0.00
ATOM 1641 N PRO 105 -6.791 -9.472 -9.521 1.00 0.00
ATOM 1642 CD PRO 105 -7.524 -10.712 -9.324 1.00 0.00
ATOM 1643 HD2 PRO 105 -8.375 -10.497 -8.677 1.00 0.00
ATOM 1644 HD3 PRO 105 -6.859 -11.418 -8.826 1.00 0.00
ATOM 1645 CG PRO 105 -7.948 -11.191 -10.710 1.00 0.00
ATOM 1646 HG2 PRO 105 -9.037 -11.207 -10.764 1.00 0.00
ATOM 1647 HG3 PRO 105 -7.563 -12.198 -10.873 1.00 0.00
ATOM 1648 CB PRO 105 -7.352 -10.208 -11.720 1.00 0.00
ATOM 1649 HB2 PRO 105 -8.077 -9.898 -12.472 1.00 0.00
ATOM 1650 HB3 PRO 105 -6.486 -10.650 -12.212 1.00 0.00
ATOM 1651 CA PRO 105 -6.920 -9.003 -10.887 1.00 0.00
ATOM 1652 HA PRO 105 -5.928 -8.723 -11.242 1.00 0.00
ATOM 1653 C PRO 105 -7.857 -7.810 -11.006 1.00 0.00
ATOM 1654 O PRO 105 -8.805 -7.813 -11.781 1.00 0.00
ATOM 1655 N PHE 106 -7.608 -6.785 -10.201 1.00 0.00
ATOM 1656 H PHE 106 -6.808 -6.834 -9.587 1.00 0.00

ATOM	1657	CA	PHE	106	-8.414	-5.598	-10.175	1.00	0.00
ATOM	1658	HA	PHE	106	-9.450	-5.821	-10.431	1.00	0.00
ATOM	1659	CB	PHE	106	-8.372	-5.039	-8.757	1.00	0.00
ATOM	1660	HB2	PHE	106	-9.304	-4.536	-8.500	1.00	0.00
ATOM	1661	HB3	PHE	106	-8.204	-5.866	-8.067	1.00	0.00
ATOM	1662	CG	PHE	106	-7.257	-4.038	-8.515	1.00	0.00
ATOM	1663	CD1	PHE	106	-5.933	-4.471	-8.352	1.00	0.00
ATOM	1664	HD1	PHE	106	-5.711	-5.538	-8.397	1.00	0.00
ATOM	1665	CE1	PHE	106	-4.904	-3.552	-8.134	1.00	0.00
ATOM	1666	HE1	PHE	106	-3.872	-3.890	-8.035	1.00	0.00
ATOM	1667	CZ	PHE	106	-5.205	-2.194	-8.044	1.00	0.00
ATOM	1668	HZ	PHE	106	-4.418	-1.479	-7.806	1.00	0.00
ATOM	1669	CE2	PHE	106	-6.514	-1.748	-8.258	1.00	0.00
ATOM	1670	HE2	PHE	106	-6.733	-0.681	-8.241	1.00	0.00
ATOM	1671	CD2	PHE	106	-7.538	-2.666	-8.493	1.00	0.00
ATOM	1672	HD2	PHE	106	-8.557	-2.316	-8.660	1.00	0.00
ATOM	1673	C	PHE	106	-7.878	-4.565	-11.119	1.00	0.00
ATOM	1674	O	PHE	106	-6.681	-4.598	-11.395	1.00	0.00
ATOM	1675	N	ILE	107	-8.739	-3.640	-11.546	1.00	0.00
ATOM	1676	H	ILE	107	-9.699	-3.691	-11.237	1.00	0.00
ATOM	1677	CA	ILE	107	-8.375	-2.601	-12.492	1.00	0.00
ATOM	1678	HA	ILE	107	-7.767	-1.823	-12.031	1.00	0.00

```
ATOM   1679  CB  ILE   107      -7.630  -3.178 -13.716  1.00  0.00
ATOM   1680  HB  ILE   107      -7.944  -2.637 -14.609  1.00  0.00
ATOM   1681  CG2 ILE   107      -6.110  -3.036 -13.539  1.00  0.00
ATOM   1682 HG21 ILE   107      -5.796  -3.578 -12.647  1.00  0.00
ATOM   1683 HG22 ILE   107      -5.602  -3.448 -14.411  1.00  0.00
ATOM   1684 HG23 ILE   107      -5.853  -1.982 -13.434  1.00  0.00
ATOM   1685  CG1 ILE   107      -8.030  -4.645 -13.991  1.00  0.00
ATOM   1686 HG12 ILE   107      -7.319  -5.299 -13.485  1.00  0.00
ATOM   1687 HG13 ILE   107      -9.028  -4.813 -13.587  1.00  0.00
ATOM   1688  CD1 ILE   107      -8.028  -4.949 -15.469  1.00  0.00
ATOM   1689 HD11 ILE   107      -7.030  -4.782 -15.874  1.00  0.00
ATOM   1690 HD12 ILE   107      -8.314  -5.989 -15.627  1.00  0.00
ATOM   1691 HD13 ILE   107      -8.739  -4.297 -15.976  1.00  0.00
ATOM   1692  C   ILE   107      -9.607  -1.840 -12.930  1.00  0.00
ATOM   1693  O   ILE   107      -9.524  -0.627 -13.065  1.00  0.00
ATOM   1694  N   ALA   108     -10.722  -2.549 -13.142  1.00  0.00
ATOM   1695  H   ALA   108     -10.676  -3.557 -13.091  1.00  0.00
ATOM   1696  CA  ALA   108     -11.977  -1.939 -13.517  1.00  0.00
ATOM   1697  HA  ALA   108     -11.816  -1.220 -14.320  1.00  0.00
ATOM   1698  CB  ALA   108     -12.932  -3.022 -14.016  1.00  0.00
ATOM   1699  HB1 ALA   108     -13.111  -3.745 -13.220  1.00  0.00
ATOM   1700  HB2 ALA   108     -13.877  -2.566 -14.311  1.00  0.00
```

ATOM 1701 HB3 ALA 108 -12.491 -3.529 -14.874 1.00 0.00
ATOM 1702 C ALA 108 -12.560 -1.157 -12.341 1.00 0.00
ATOM 1703 O ALA 108 -13.599 -1.516 -11.800 1.00 0.00
ATOM 1704 N ILE 109 -11.871 -0.103 -11.933 1.00 0.00
ATOM 1705 H ILE 109 -10.985 0.065 -12.387 1.00 0.00
ATOM 1706 CA ILE 109 -12.233 0.728 -10.808 1.00 0.00
ATOM 1707 HA ILE 109 -13.280 0.607 -10.528 1.00 0.00
ATOM 1708 CB ILE 109 -11.340 0.444 -9.588 1.00 0.00
ATOM 1709 HB ILE 109 -11.206 1.367 -9.025 1.00 0.00
ATOM 1710 CG2 ILE 109 -11.984 -0.618 -8.683 1.00 0.00
ATOM 1711 HG21 ILE 109 -12.118 -1.542 -9.245 1.00 0.00
ATOM 1712 HG22 ILE 109 -11.338 -0.806 -7.826 1.00 0.00
ATOM 1713 HG23 ILE 109 -12.953 -0.261 -8.336 1.00 0.00
ATOM 1714 CG1 ILE 109 -9.915 0.034 -9.987 1.00 0.00
ATOM 1715 HG12 ILE 109 -9.382 -0.300 -9.097 1.00 0.00
ATOM 1716 HG13 ILE 109 -9.972 -0.785 -10.704 1.00 0.00
ATOM 1717 CD1 ILE 109 -9.171 1.206 -10.614 1.00 0.00
ATOM 1718 HD11 ILE 109 -9.113 2.025 -9.898 1.00 0.00
ATOM 1719 HD12 ILE 109 -8.164 0.892 -10.889 1.00 0.00
ATOM 1720 HD13 ILE 109 -9.703 1.540 -11.505 1.00 0.00
ATOM 1721 C ILE 109 -12.237 2.203 -11.239 1.00 0.00
ATOM 1722 O ILE 109 -13.264 2.863 -11.053 1.00 0.00

ATOM 1723 OXT ILE 109 -11.224 2.698 -11.767 1.00 0.00

TER

ATOM 1724 Cl1 LIG 110 20.727 -12.783 -6.849 1.00 0.00

ATOM 1725 Cl2 LIG 110 21.737 -15.481 -7.475 1.00 0.00

ATOM 1726 P1 LIG 110 19.590 -16.829 -3.628 1.00 0.00

ATOM 1727 O1 LIG 110 19.110 -18.296 -4.030 1.00 0.00

ATOM 1728 O2 LIG 110 20.906 -17.121 -2.794 1.00 0.00

ATOM 1729 O3 LIG 110 20.119 -16.279 -5.088 1.00 0.00

ATOM 1730 O4 LIG 110 18.637 -15.916 -2.981 1.00 0.00

ATOM 1731 C1 LIG 110 17.916 -18.510 -4.816 1.00 0.00

ATOM 1732 H1 LIG 110 17.753 -19.586 -4.823 1.00 0.00

ATOM 1733 H2 LIG 110 18.070 -18.149 -5.834 1.00 0.00

ATOM 1734 H3 LIG 110 17.065 -18.006 -4.356 1.00 0.00

ATOM 1735 C2 LIG 110 21.967 -17.992 -3.250 1.00 0.00

ATOM 1736 H4 LIG 110 22.348 -17.651 -4.214 1.00 0.00

ATOM 1737 H5 LIG 110 21.600 -19.015 -3.327 1.00 0.00

ATOM 1738 H6 LIG 110 22.747 -17.925 -2.495 1.00 0.00

ATOM 1739 C3 LIG 110 20.106 -14.941 -5.390 1.00 0.00

ATOM 1740 H7 LIG 110 19.510 -14.300 -4.752 1.00 0.00

ATOM 1741 C4 LIG 110 20.779 -14.473 -6.438 1.00 0.00

TER

ATOM 1742 Cl1 LIG 111 18.772 -13.594 20.693 1.00 0.00

```
ATOM   1743  Cl2 LIG   111      15.946 -13.070  20.038  1.00  0.00
ATOM   1744  P1  LIG   111      17.215 -13.520  15.633  1.00  0.00
ATOM   1745  O1  LIG   111      15.918 -14.198  14.997  1.00  0.00
ATOM   1746  O2  LIG   111      17.023 -11.995  15.246  1.00  0.00
ATOM   1747  O3  LIG   111      16.840 -13.608  17.235  1.00  0.00
ATOM   1748  O4  LIG   111      18.539 -14.075  15.321  1.00  0.00
ATOM   1749  C1  LIG   111      15.677 -15.619  15.111  1.00  0.00
ATOM   1750  H1  LIG   111      14.814 -15.826  14.482  1.00  0.00
ATOM   1751  H2  LIG   111      15.453 -15.877  16.148  1.00  0.00
ATOM   1752  H3  LIG   111      16.542 -16.179  14.755  1.00  0.00
ATOM   1753  C2  LIG   111      15.796 -11.260  15.467  1.00  0.00
ATOM   1754  H4  LIG   111      15.522 -11.291  16.522  1.00  0.00
ATOM   1755  H5  LIG   111      14.997 -11.679  14.854  1.00  0.00
ATOM   1756  H6  LIG   111      16.008 -10.238  15.164  1.00  0.00
ATOM   1757  C3  LIG   111      17.822 -13.674  18.190  1.00  0.00
ATOM   1758  H7  LIG   111      18.820 -13.922  17.853  1.00  0.00
ATOM   1759  C4  LIG   111      17.537 -13.466  19.474  1.00  0.00
TER
ATOM   1760  Cl1 LIG   112      -4.189  -4.328  17.766  1.00  0.00
ATOM   1761  Cl2 LIG   112      -2.350  -6.630  17.850  1.00  0.00
ATOM   1762  P1  LIG   112      -5.854  -9.084  16.142  1.00  0.00
ATOM   1763  O1  LIG   112      -5.086 -10.123  15.205  1.00  0.00
```

```
ATOM   1764  O2  LIG   112      -6.115  -9.939  17.451  1.00  0.00
ATOM   1765  O3  LIG   112      -4.618  -8.067  16.531  1.00  0.00
ATOM   1766  O4  LIG   112      -7.032  -8.382  15.615  1.00  0.00
ATOM   1767  C1  LIG   112      -4.645  -9.760  13.877  1.00  0.00
ATOM   1768  H1  LIG   112      -4.285 -10.679  13.421  1.00  0.00
ATOM   1769  H2  LIG   112      -3.835  -9.031  13.940  1.00  0.00
ATOM   1770  H3  LIG   112      -5.478  -9.357  13.300  1.00  0.00
ATOM   1771  C2  LIG   112      -5.091 -10.706  18.125  1.00  0.00
ATOM   1772  H4  LIG   112      -4.257 -10.060  18.405  1.00  0.00
ATOM   1773  H5  LIG   112      -4.745 -11.513  17.478  1.00  0.00
ATOM   1774  H6  LIG   112      -5.566 -11.112  19.015  1.00  0.00
ATOM   1775  C3  LIG   112      -4.852  -6.752  16.839  1.00  0.00
ATOM   1776  H7  LIG   112      -5.822  -6.350  16.572  1.00  0.00
ATOM   1777  C4  LIG   112      -3.910  -6.009  17.415  1.00  0.00
TER
ATOM   1778  Cl1 LIG   113      21.716  23.789  -1.963  1.00  0.00
ATOM   1779  Cl2 LIG   113      20.936  23.210   0.820  1.00  0.00
ATOM   1780  P1  LIG   113      16.602  23.170  -0.741  1.00  0.00
ATOM   1781  O1  LIG   113      15.944  22.045   0.179  1.00  0.00
ATOM   1782  O2  LIG   113      16.150  24.505  -0.016  1.00  0.00
ATOM   1783  O3  LIG   113      18.191  22.984  -0.347  1.00  0.00
ATOM   1784  O4  LIG   113      16.361  23.140  -2.190  1.00  0.00
```

```
ATOM   1785  C1  LIG   113      16.103  20.639  -0.118  1.00  0.00
ATOM   1786  H1  LIG   113      15.450  20.112   0.574  1.00  0.00
ATOM   1787  H2  LIG   113      17.140  20.339   0.045  1.00  0.00
ATOM   1788  H3  LIG   113      15.802  20.434  -1.146  1.00  0.00
ATOM   1789  C2  LIG   113      16.296  24.735   1.404  1.00  0.00
ATOM   1790  H4  LIG   113      17.341  24.627   1.700  1.00  0.00
ATOM   1791  H5  LIG   113      15.670  24.036   1.960  1.00  0.00
ATOM   1792  H6  LIG   113      15.963  25.757   1.572  1.00  0.00
ATOM   1793  C3  LIG   113      19.184  23.307  -1.235  1.00  0.00
ATOM   1794  H7  LIG   113      18.895  23.442  -2.269  1.00  0.00
ATOM   1795  C4  LIG   113      20.448  23.422  -0.831  1.00  0.00
TER
ATOM   1796  Cl1 LIG   114      19.549  13.893 -15.675  1.00  0.00
ATOM   1797  Cl2 LIG   114      19.922  13.966 -18.598  1.00  0.00
ATOM   1798  P1  LIG   114      16.879  10.512 -18.752  1.00  0.00
ATOM   1799  O1  LIG   114      16.113  10.824 -20.116  1.00  0.00
ATOM   1800  O2  LIG   114      17.913   9.395 -19.194  1.00  0.00
ATOM   1801  O3  LIG   114      17.772  11.887 -18.591  1.00  0.00
ATOM   1802  O4  LIG   114      16.097  10.190 -17.550  1.00  0.00
ATOM   1803  C1  LIG   114      15.055  11.808 -20.180  1.00  0.00
ATOM   1804  H1  LIG   114      14.625  11.719 -21.175  1.00  0.00
ATOM   1805  H2  LIG   114      15.468  12.808 -20.040  1.00  0.00
```

```
ATOM   1806  H3  LIG   114      14.298  11.598 -19.424  1.00  0.00
ATOM   1807  C2  LIG   114      18.776   9.514 -20.349  1.00  0.00
ATOM   1808  H4  LIG   114      19.390  10.413 -20.272  1.00  0.00
ATOM   1809  H5  LIG   114      18.176   9.540 -21.259  1.00  0.00
ATOM   1810  H6  LIG   114      19.406   8.628 -20.337  1.00  0.00
ATOM   1811  C3  LIG   114      18.155  12.343 -17.357  1.00  0.00
ATOM   1812  H7  LIG   114      17.645  11.925 -16.498  1.00  0.00
ATOM   1813  C4  LIG   114      19.094  13.279 -17.238  1.00  0.00
TER
ATOM   1814  Cl1 LIG   115     -16.545  18.034   8.585  1.00  0.00
ATOM   1815  Cl2 LIG   115     -18.625  19.760   9.760  1.00  0.00
ATOM   1816  P1  LIG   115     -21.594  16.441   8.583  1.00  0.00
ATOM   1817  O1  LIG   115     -22.645  16.516   9.780  1.00  0.00
ATOM   1818  O2  LIG   115     -22.323  17.263   7.441  1.00  0.00
ATOM   1819  O3  LIG   115     -20.420  17.445   9.155  1.00  0.00
ATOM   1820  O4  LIG   115     -21.076  15.130   8.167  1.00  0.00
ATOM   1821  C1  LIG   115     -22.385  15.883  11.054  1.00  0.00
ATOM   1822  H1  LIG   115     -23.307  15.972  11.624  1.00  0.00
ATOM   1823  H2  LIG   115     -21.576  16.402  11.570  1.00  0.00
ATOM   1824  H3  LIG   115     -22.134  14.832  10.909  1.00  0.00
ATOM   1825  C2  LIG   115     -22.904  18.573   7.643  1.00  0.00
ATOM   1826  H4  LIG   115     -22.150  19.268   8.016  1.00  0.00
```

ATOM	1827	H5	LIG	115	-23.737	18.505	8.344	1.00	0.00
ATOM	1828	H6	LIG	115	-23.257	18.891	6.665	1.00	0.00
ATOM	1829	C3	LIG	115	-19.110	17.289	8.783	1.00	0.00
ATOM	1830	H7	LIG	115	-18.839	16.349	8.318	1.00	0.00
ATOM	1831	C4	LIG	115	-18.217	18.249	9.015	1.00	0.00
TER									
ATOM	1832	Cl1	LIG	116	-17.227	-3.718	17.615	1.00	0.00
ATOM	1833	Cl2	LIG	116	-18.189	-1.641	15.758	1.00	0.00
ATOM	1834	P1	LIG	116	-22.418	-2.762	17.197	1.00	0.00
ATOM	1835	O1	LIG	116	-23.153	-1.347	17.154	1.00	0.00
ATOM	1836	O2	LIG	116	-22.888	-3.428	15.837	1.00	0.00
ATOM	1837	O3	LIG	116	-20.860	-2.296	16.932	1.00	0.00
ATOM	1838	O4	LIG	116	-22.570	-3.612	18.386	1.00	0.00
ATOM	1839	C1	LIG	116	-23.004	-0.387	18.225	1.00	0.00
ATOM	1840	H1	LIG	116	-23.708	0.413	18.008	1.00	0.00
ATOM	1841	H2	LIG	116	-21.985	0.005	18.234	1.00	0.00
ATOM	1842	H3	LIG	116	-23.245	-0.849	19.183	1.00	0.00
ATOM	1843	C2	LIG	116	-22.825	-2.761	14.555	1.00	0.00
ATOM	1844	H4	LIG	116	-21.803	-2.444	14.343	1.00	0.00
ATOM	1845	H5	LIG	116	-23.498	-1.902	14.550	1.00	0.00
ATOM	1846	H6	LIG	116	-23.148	-3.499	13.824	1.00	0.00
ATOM	1847	C3	LIG	116	-19.809	-3.031	17.413	1.00	0.00

```
ATOM   1848  H7  LIG   116     -20.031  -3.769  18.174  1.00  0.00
ATOM   1849  C4  LIG   116     -18.570  -2.817  16.973  1.00  0.00
TER
ATOM   1850  Cl1 LIG   117     -17.659 -19.072 -17.285  1.00  0.00
ATOM   1851  Cl2 LIG   117     -17.437 -20.884 -14.971  1.00  0.00
ATOM   1852  P1  LIG   117     -14.337 -23.190 -17.480  1.00  0.00
ATOM   1853  O1  LIG   117     -13.320 -23.682 -16.353  1.00  0.00
ATOM   1854  O2  LIG   117     -15.232 -24.477 -17.712  1.00  0.00
ATOM   1855  O3  LIG   117     -15.279 -22.163 -16.601  1.00  0.00
ATOM   1856  O4  LIG   117     -13.806 -22.577 -18.705  1.00  0.00
ATOM   1857  C1  LIG   117     -12.297 -22.807 -15.826  1.00  0.00
ATOM   1858  H1  LIG   117     -11.672 -23.429 -15.188  1.00  0.00
ATOM   1859  H2  LIG   117     -12.755 -22.011 -15.237  1.00  0.00
ATOM   1860  H3  LIG   117     -11.702 -22.388 -16.639  1.00  0.00
ATOM   1861  C2  LIG   117     -15.847 -25.230 -16.640  1.00  0.00
ATOM   1862  H4  LIG   117     -16.486 -24.581 -16.041  1.00  0.00
ATOM   1863  H5  LIG   117     -15.077 -25.685 -16.017  1.00  0.00
ATOM   1864  H6  LIG   117     -16.444 -25.998 -17.126  1.00  0.00
ATOM   1865  C3  LIG   117     -15.915 -21.103 -17.192  1.00  0.00
ATOM   1866  H7  LIG   117     -15.578 -20.817 -18.181  1.00  0.00
ATOM   1867  C4  LIG   117     -16.882 -20.447 -16.555  1.00  0.00
TER
```

```
ATOM   1868  Cl1 LIG   118      20.074  -3.003  -1.149  1.00  0.00
ATOM   1869  Cl2 LIG   118      22.024  -4.270  -2.959  1.00  0.00
ATOM   1870  P1  LIG   118      20.480  -1.338  -6.158  1.00  0.00
ATOM   1871  O1  LIG   118      21.810  -1.044  -6.988  1.00  0.00
ATOM   1872  O2  LIG   118      19.758  -2.429  -7.053  1.00  0.00
ATOM   1873  O3  LIG   118      21.104  -2.172  -4.882  1.00  0.00
ATOM   1874  O4  LIG   118      19.628  -0.212  -5.749  1.00  0.00
ATOM   1875  C1  LIG   118      22.790  -0.085  -6.528  1.00  0.00
ATOM   1876  H1  LIG   118      23.510   0.017  -7.337  1.00  0.00
ATOM   1877  H2  LIG   118      23.285  -0.459  -5.631  1.00  0.00
ATOM   1878  H3  LIG   118      22.313   0.876  -6.329  1.00  0.00
ATOM   1879  C2  LIG   118      20.417  -3.612  -7.562  1.00  0.00
ATOM   1880  H4  LIG   118      20.837  -4.194  -6.740  1.00  0.00
ATOM   1881  H5  LIG   118      21.201  -3.326  -8.265  1.00  0.00
ATOM   1882  H6  LIG   118      19.644  -4.183  -8.070  1.00  0.00
ATOM   1883  C3  LIG   118      20.486  -2.162  -3.658  1.00  0.00
ATOM   1884  H7  LIG   118      19.735  -1.400  -3.492  1.00  0.00
ATOM   1885  C4  LIG   118      20.824  -3.042  -2.718  1.00  0.00
TER
ATOM   1886  Cl1 LIG   119      -4.692  15.153  10.549  1.00  0.00
ATOM   1887  Cl2 LIG   119      -5.886  17.384  12.061  1.00  0.00
ATOM   1888  P1  LIG   119      -3.555  15.756  15.685  1.00  0.00
```

ATOM 1889 O1 LIG 119 -3.184 17.103 16.454 1.00 0.00

ATOM 1890 O2 LIG 119 -4.824 15.248 16.488 1.00 0.00

ATOM 1891 O3 LIG 119 -4.153 16.372 14.279 1.00 0.00

ATOM 1892 O4 LIG 119 -2.512 14.743 15.472 1.00 0.00

ATOM 1893 C1 LIG 119 -2.059 17.918 16.050 1.00 0.00

ATOM 1894 H1 LIG 119 -1.955 18.683 16.816 1.00 0.00

ATOM 1895 H2 LIG 119 -2.264 18.381 15.084 1.00 0.00

ATOM 1896 H3 LIG 119 -1.153 17.312 16.000 1.00 0.00

ATOM 1897 C2 LIG 119 -5.959 16.086 16.806 1.00 0.00

ATOM 1898 H4 LIG 119 -6.387 16.506 15.894 1.00 0.00

ATOM 1899 H5 LIG 119 -5.654 16.883 17.484 1.00 0.00

ATOM 1900 H6 LIG 119 -6.680 15.432 17.290 1.00 0.00

ATOM 1901 C3 LIG 119 -4.088 15.661 13.109 1.00 0.00

ATOM 1902 H7 LIG 119 -3.417 14.811 13.086 1.00 0.00

ATOM 1903 C4 LIG 119 -4.806 16.027 12.049 1.00 0.00

TER

END

Table J: The number of atoms present in the system of BN-Nap

ATOM 1 N GLN 1 -10.822 -8.568 3.550 1.00 0.00

ATOM 2 H1 GLN 1 -9.891 -8.899 3.759 1.00 0.00

ATOM 3 H2 GLN 1 -11.053 -7.822 4.190 1.00 0.00

```
ATOM      4  H3  GLN     1     -10.899  -8.421   2.554  1.00  0.00
ATOM      5  CA  GLN     1     -11.767  -9.646   3.856  1.00  0.00
ATOM      6  HA  GLN     1     -12.469  -9.308   4.618  1.00  0.00
ATOM      7  CB  GLN     1     -12.560 -10.062   2.609  1.00  0.00
ATOM      8  HB2 GLN     1     -12.263  -9.428   1.774  1.00  0.00
ATOM      9  HB3 GLN     1     -12.330 -11.102   2.375  1.00  0.00
ATOM     10  CG  GLN     1     -14.060  -9.915   2.850  1.00  0.00
ATOM     11  HG2 GLN     1     -14.675 -10.722   2.451  1.00  0.00
ATOM     12  HG3 GLN     1     -14.136  -9.904   3.937  1.00  0.00
ATOM     13  CD  GLN     1     -14.585  -8.594   2.313  1.00  0.00
ATOM     14  OE1 GLN     1     -14.943  -7.693   3.053  1.00  0.00
ATOM     15  NE2 GLN     1     -14.481  -8.434   1.007  1.00  0.00
ATOM     16 HE21 GLN     1     -14.809  -7.581   0.578  1.00  0.00
ATOM     17 HE22 GLN     1     -14.074  -9.166   0.442  1.00  0.00
ATOM     18  C   GLN     1     -11.035 -10.849   4.440  1.00  0.00
ATOM     19  O   GLN     1     -11.387 -11.329   5.508  1.00  0.00
ATOM     20  N   PRO     2      -9.977 -11.300   3.744  1.00  0.00
ATOM     21  CD  PRO     2      -9.481 -10.776   2.473  1.00  0.00
ATOM     22  HD2 PRO     2      -9.076  -9.782   2.663  1.00  0.00
ATOM     23  HD3 PRO     2     -10.331 -10.698   1.796  1.00  0.00
ATOM     24  CG  PRO     2      -8.427 -11.749   1.970  1.00  0.00
ATOM     25  HG2 PRO     2      -7.462 -11.244   1.929  1.00  0.00
```

```
ATOM     26  HG3 PRO     2      -8.701 -12.090   0.971  1.00  0.00
ATOM     27  CB  PRO     2      -8.404 -12.915   2.957  1.00  0.00
ATOM     28  HB2 PRO     2      -7.384 -13.176   3.240  1.00  0.00
ATOM     29  HB3 PRO     2      -8.898 -13.790   2.535  1.00  0.00
ATOM     30  CA  PRO     2      -9.173 -12.422   4.181  1.00  0.00
ATOM     31  HA  PRO     2      -9.843 -13.228   4.480  1.00  0.00
ATOM     32  C   PRO     2      -8.283 -12.056   5.337  1.00  0.00
ATOM     33  O   PRO     2      -7.294 -11.375   5.125  1.00  0.00
ATOM     34  N   GLN     3      -8.629 -12.519   6.542  1.00  0.00
ATOM     35  H   GLN     3      -9.452 -13.097   6.637  1.00  0.00
ATOM     36  CA  GLN     3      -7.861 -12.217   7.731  1.00  0.00
ATOM     37  HA  GLN     3      -7.787 -11.137   7.854  1.00  0.00
ATOM     38  CB  GLN     3      -8.527 -12.805   8.974  1.00  0.00
ATOM     39  HB2 GLN     3      -9.578 -12.994   8.755  1.00  0.00
ATOM     40  HB3 GLN     3      -8.035 -13.745   9.226  1.00  0.00
ATOM     41  CG  GLN     3      -8.418 -11.836  10.159  1.00  0.00
ATOM     42  HG2 GLN     3      -7.972 -12.380  10.992  1.00  0.00
ATOM     43  HG3 GLN     3      -7.788 -10.984   9.903  1.00  0.00
ATOM     44  CD  GLN     3      -9.775 -11.323  10.588  1.00  0.00
ATOM     45  OE1 GLN     3     -10.395 -11.837  11.498  1.00  0.00
ATOM     46  NE2 GLN     3     -10.241 -10.311   9.878  1.00  0.00
ATOM     47 HE21 GLN     3     -11.141  -9.913  10.103  1.00  0.00
```

```
ATOM     48 HE22 GLN     3      -9.695  -9.941   9.113  1.00  0.00
ATOM     49  C   GLN     3      -6.479 -12.794   7.607  1.00  0.00
ATOM     50  O   GLN     3      -5.538 -12.217   8.079  1.00  0.00
ATOM     51  N   LYS     4      -6.313 -13.908   6.902  1.00  0.00
ATOM     52  H   LYS     4      -7.129 -14.390   6.553  1.00  0.00
ATOM     53  CA  LYS     4      -5.009 -14.515   6.686  1.00  0.00
ATOM     54  HA  LYS     4      -4.658 -14.994   7.600  1.00  0.00
ATOM     55  CB  LYS     4      -5.116 -15.577   5.588  1.00  0.00
ATOM     56  HB2 LYS     4      -4.284 -16.273   5.699  1.00  0.00
ATOM     57  HB3 LYS     4      -6.056 -16.113   5.717  1.00  0.00
ATOM     58  CG  LYS     4      -5.074 -14.948   4.186  1.00  0.00
ATOM     59  HG2 LYS     4      -5.283 -13.882   4.274  1.00  0.00
ATOM     60  HG3 LYS     4      -4.077 -15.090   3.768  1.00  0.00
ATOM     61  CD  LYS     4      -6.108 -15.595   3.266  1.00  0.00
ATOM     62  HD2 LYS     4      -7.071 -15.617   3.777  1.00  0.00
ATOM     63  HD3 LYS     4      -6.193 -15.000   2.357  1.00  0.00
ATOM     64  CE  LYS     4      -5.692 -17.020   2.904  1.00  0.00
ATOM     65  HE2 LYS     4      -5.491 -17.073   1.834  1.00  0.00
ATOM     66  HE3 LYS     4      -4.789 -17.280   3.456  1.00  0.00
ATOM     67  NZ  LYS     4      -6.768 -17.967   3.249  1.00  0.00
ATOM     68  HZ1 LYS     4      -7.605 -17.727   2.737  1.00  0.00
ATOM     69  HZ2 LYS     4      -6.482 -18.905   3.005  1.00  0.00
```

```
ATOM     70  HZ3 LYS     4      -6.954 -17.919   4.240  1.00  0.00
ATOM     71  C   LYS     4      -3.955 -13.443   6.357  1.00  0.00
ATOM     72  O   LYS     4      -2.812 -13.607   6.721  1.00  0.00
ATOM     73  N   CYX     5      -4.399 -12.344   5.721  1.00  0.00
ATOM     74  H   CYX     5      -5.359 -12.311   5.409  1.00  0.00
ATOM     75  CA  CYX     5      -3.560 -11.227   5.445  1.00  0.00
ATOM     76  HA  CYX     5      -2.543 -11.423   5.784  1.00  0.00
ATOM     77  CB  CYX     5      -3.533 -10.982   3.959  1.00  0.00
ATOM     78  HB2 CYX     5      -4.124 -11.746   3.453  1.00  0.00
ATOM     79  HB3 CYX     5      -3.951  -9.999   3.745  1.00  0.00
ATOM     80  SG  CYX     5      -1.867 -11.060   3.233  1.00  0.00
ATOM     81  C   CYX     5      -4.072 -10.021   6.163  1.00  0.00
ATOM     82  O   CYX     5      -3.316  -9.264   6.689  1.00  0.00
ATOM     83  N   GLN     6      -5.382  -9.830   6.286  1.00  0.00
ATOM     84  H   GLN     6      -5.996 -10.468   5.799  1.00  0.00
ATOM     85  CA  GLN     6      -5.972  -8.725   7.005  1.00  0.00
ATOM     86  HA  GLN     6      -5.561  -7.785   6.637  1.00  0.00
ATOM     87  CB  GLN     6      -7.489  -8.705   6.810  1.00  0.00
ATOM     88  HB2 GLN     6      -7.807  -9.691   6.472  1.00  0.00
ATOM     89  HB3 GLN     6      -7.958  -8.481   7.768  1.00  0.00
ATOM     90  CG  GLN     6      -7.905  -7.658   5.784  1.00  0.00
ATOM     91  HG2 GLN     6      -6.991  -7.153   5.471  1.00  0.00
```

ATOM 92 HG3 GLN 6 -8.379 -8.120 4.918 1.00 0.00
ATOM 93 CD GLN 6 -8.851 -6.630 6.362 1.00 0.00
ATOM 94 OE1 GLN 6 -8.871 -6.331 7.541 1.00 0.00
ATOM 95 NE2 GLN 6 -9.713 -6.112 5.498 1.00 0.00
ATOM 96 HE21 GLN 6 -10.378 -5.418 5.807 1.00 0.00
ATOM 97 HE22 GLN 6 -9.700 -6.414 4.534 1.00 0.00
ATOM 98 C GLN 6 -5.620 -8.764 8.482 1.00 0.00
ATOM 99 O GLN 6 -5.865 -7.817 9.210 1.00 0.00
ATOM 100 N ARG 7 -5.054 -9.872 8.942 1.00 0.00
ATOM 101 H ARG 7 -4.930 -10.622 8.276 1.00 0.00
ATOM 102 CA ARG 7 -4.643 -10.092 10.296 1.00 0.00
ATOM 103 HA ARG 7 -5.048 -9.301 10.927 1.00 0.00
ATOM 104 CB ARG 7 -5.138 -11.442 10.825 1.00 0.00
ATOM 105 HB2 ARG 7 -5.943 -11.801 10.183 1.00 0.00
ATOM 106 HB3 ARG 7 -4.313 -12.155 10.805 1.00 0.00
ATOM 107 CG ARG 7 -5.653 -11.300 12.254 1.00 0.00
ATOM 108 HG2 ARG 7 -4.925 -10.733 12.833 1.00 0.00
ATOM 109 HG3 ARG 7 -6.600 -10.760 12.232 1.00 0.00
ATOM 110 CD ARG 7 -5.863 -12.673 12.902 1.00 0.00
ATOM 111 HD2 ARG 7 -6.857 -13.049 12.657 1.00 0.00
ATOM 112 HD3 ARG 7 -5.111 -13.373 12.536 1.00 0.00
ATOM 113 NE ARG 7 -5.743 -12.574 14.371 1.00 0.00

ATOM	114	HE	ARG	7	-6.605	-12.482	14.889	1.00	0.00
ATOM	115	CZ	ARG	7	-4.601	-12.592	15.042	1.00	0.00
ATOM	116	NH1	ARG	7	-3.459	-12.723	14.408	1.00	0.00
ATOM	117	HH11	ARG	7	-3.451	-12.811	13.402	1.00	0.00
ATOM	118	HH12	ARG	7	-2.594	-12.736	14.929	1.00	0.00
ATOM	119	NH2	ARG	7	-4.599	-12.485	16.349	1.00	0.00
ATOM	120	HH21	ARG	7	-5.473	-12.389	16.846	1.00	0.00
ATOM	121	HH22	ARG	7	-3.724	-12.500	16.852	1.00	0.00
ATOM	122	C	ARG	7	-3.142	-10.038	10.397	1.00	0.00
ATOM	123	O	ARG	7	-2.666	-10.045	11.505	1.00	0.00
ATOM	124	N	GLU	8	-2.446	-10.026	9.273	1.00	0.00
ATOM	125	H	GLU	8	-2.944	-10.119	8.399	1.00	0.00
ATOM	126	CA	GLU	8	-1.001	-9.949	9.234	1.00	0.00
ATOM	127	HA	GLU	8	-0.599	-9.991	10.246	1.00	0.00
ATOM	128	CB	GLU	8	-0.432	-11.121	8.434	1.00	0.00
ATOM	129	HB2	GLU	8	-1.255	-11.665	7.969	1.00	0.00
ATOM	130	HB3	GLU	8	0.230	-10.733	7.660	1.00	0.00
ATOM	131	CG	GLU	8	0.353	-12.068	9.349	1.00	0.00
ATOM	132	HG2	GLU	8	0.009	-11.873	10.364	1.00	0.00
ATOM	133	HG3	GLU	8	0.161	-13.111	9.095	1.00	0.00
ATOM	134	CD	GLU	8	1.857	-11.789	9.257	1.00	0.00
ATOM	135	OE1	GLU	8	2.416	-12.053	8.173	1.00	0.00

ATOM	136	OE2	GLU	8	2.413	-11.373	10.292	1.00	0.00
ATOM	137	C	GLU	8	-0.525	-8.628	8.647	1.00	0.00
ATOM	138	O	GLU	8	0.543	-8.143	8.963	1.00	0.00
ATOM	139	N	PHE	9	-1.384	-7.972	7.879	1.00	0.00
ATOM	140	H	PHE	9	-2.298	-8.382	7.747	1.00	0.00
ATOM	141	CA	PHE	9	-1.099	-6.730	7.234	1.00	0.00
ATOM	142	HA	PHE	9	-0.061	-6.697	6.904	1.00	0.00
ATOM	143	CB	PHE	9	-2.003	-6.594	6.013	1.00	0.00
ATOM	144	HB2	PHE	9	-1.528	-7.090	5.166	1.00	0.00
ATOM	145	HB3	PHE	9	-2.974	-7.052	6.204	1.00	0.00
ATOM	146	CG	PHE	9	-2.274	-5.167	5.584	1.00	0.00
ATOM	147	CD1	PHE	9	-3.097	-4.328	6.354	1.00	0.00
ATOM	148	HD1	PHE	9	-3.569	-4.730	7.250	1.00	0.00
ATOM	149	CE1	PHE	9	-3.314	-3.002	5.988	1.00	0.00
ATOM	150	HE1	PHE	9	-3.913	-2.349	6.622	1.00	0.00
ATOM	151	CZ	PHE	9	-2.763	-2.512	4.807	1.00	0.00
ATOM	152	HZ	PHE	9	-2.937	-1.481	4.498	1.00	0.00
ATOM	153	CE2	PHE	9	-1.981	-3.355	4.015	1.00	0.00
ATOM	154	HE2	PHE	9	-1.569	-2.971	3.082	1.00	0.00
ATOM	155	CD2	PHE	9	-1.724	-4.673	4.402	1.00	0.00
ATOM	156	HD2	PHE	9	-1.096	-5.311	3.780	1.00	0.00
ATOM	157	C	PHE	9	-1.367	-5.617	8.204	1.00	0.00

ATOM	158	O	PHE	9	-0.693	-4.607	8.224	1.00	0.00
ATOM	159	N	GLN	10	-2.440	-5.744	9.001	1.00	0.00
ATOM	160	H	GLN	10	-3.029	-6.560	8.906	1.00	0.00
ATOM	161	CA	GLN	10	-2.828	-4.726	9.948	1.00	0.00
ATOM	162	HA	GLN	10	-2.374	-3.775	9.669	1.00	0.00
ATOM	163	CB	GLN	10	-4.351	-4.554	9.971	1.00	0.00
ATOM	164	HB2	GLN	10	-4.647	-3.956	9.110	1.00	0.00
ATOM	165	HB3	GLN	10	-4.814	-5.539	9.905	1.00	0.00
ATOM	166	CG	GLN	10	-4.810	-3.858	11.260	1.00	0.00
ATOM	167	HG2	GLN	10	-4.662	-4.517	12.115	1.00	0.00
ATOM	168	HG3	GLN	10	-4.209	-2.958	11.387	1.00	0.00
ATOM	169	CD	GLN	10	-6.261	-3.463	11.202	1.00	0.00
ATOM	170	OE1	GLN	10	-6.630	-2.466	10.602	1.00	0.00
ATOM	171	NE2	GLN	10	-7.100	-4.278	11.815	1.00	0.00
ATOM	172	HE21	GLN	10	-8.089	-4.073	11.815	1.00	0.00
ATOM	173	HE22	GLN	10	-6.749	-5.103	12.281	1.00	0.00
ATOM	174	C	GLN	10	-2.285	-5.097	11.322	1.00	0.00
ATOM	175	O	GLN	10	-2.302	-4.289	12.249	1.00	0.00
ATOM	176	N	GLN	11	-1.816	-6.329	11.480	1.00	0.00
ATOM	177	H	GLN	11	-1.860	-6.981	10.709	1.00	0.00
ATOM	178	CA	GLN	11	-1.286	-6.792	12.736	1.00	0.00
ATOM	179	HA	GLN	11	-2.091	-6.892	13.464	1.00	0.00

```
ATOM    180  CB  GLN    11      -0.617  -8.150  12.560  1.00  0.00
ATOM    181  HB2 GLN    11      -0.945  -8.587  11.616  1.00  0.00
ATOM    182  HB3 GLN    11       0.464  -8.012  12.540  1.00  0.00
ATOM    183  CG  GLN    11      -0.990  -9.087  13.712  1.00  0.00
ATOM    184  HG2 GLN    11      -1.809  -8.616  14.255  1.00  0.00
ATOM    185  HG3 GLN    11      -1.315 -10.058  13.337  1.00  0.00
ATOM    186  CD  GLN    11       0.166  -9.299  14.668  1.00  0.00
ATOM    187  OE1 GLN    11       1.320  -9.338  14.300  1.00  0.00
ATOM    188  NE2 GLN    11      -0.174  -9.491  15.932  1.00  0.00
ATOM    189 HE21 GLN    11       0.543  -9.640  16.628  1.00  0.00
ATOM    190 HE22 GLN    11      -1.149  -9.489  16.195  1.00  0.00
ATOM    191  C   GLN    11      -0.298  -5.791  13.304  1.00  0.00
ATOM    192  O   GLN    11      -0.497  -5.276  14.400  1.00  0.00
ATOM    193  N   GLU    12       0.692  -5.422  12.490  1.00  0.00
ATOM    194  H   GLU    12       0.762  -5.835  11.571  1.00  0.00
ATOM    195  CA  GLU    12       1.736  -4.507  12.915  1.00  0.00
ATOM    196  HA  GLU    12       1.289  -3.535  13.124  1.00  0.00
ATOM    197  CB  GLU    12       2.462  -4.995  14.178  1.00  0.00
ATOM    198  HB2 GLU    12       3.486  -4.622  14.152  1.00  0.00
ATOM    199  HB3 GLU    12       1.949  -4.587  15.049  1.00  0.00
ATOM    200  CG  GLU    12       2.478  -6.524  14.266  1.00  0.00
ATOM    201  HG2 GLU    12       1.579  -6.890  14.762  1.00  0.00
```

```
ATOM    202  HG3 GLU    12       2.497  -6.885  13.237  1.00  0.00
ATOM    203  CD  GLU    12       3.704  -7.032  15.002  1.00  0.00
ATOM    204  OE1 GLU    12       4.754  -7.171  14.334  1.00  0.00
ATOM    205  OE2 GLU    12       3.593  -7.223  16.237  1.00  0.00
ATOM    206  C   GLU    12       2.722  -4.267  11.780  1.00  0.00
ATOM    207  O   GLU    12       3.907  -4.031  11.986  1.00  0.00
ATOM    208  N   GLN    13       2.213  -4.284  10.549  1.00  0.00
ATOM    209  H   GLN    13       1.236  -4.505  10.417  1.00  0.00
ATOM    210  CA  GLN    13       3.030  -4.071   9.379  1.00  0.00
ATOM    211  HA  GLN    13       3.774  -3.302   9.585  1.00  0.00
ATOM    212  CB  GLN    13       3.756  -5.360   8.984  1.00  0.00
ATOM    213  HB2 GLN    13       3.177  -6.214   9.337  1.00  0.00
ATOM    214  HB3 GLN    13       3.838  -5.401   7.898  1.00  0.00
ATOM    215  CG  GLN    13       5.152  -5.402   9.601  1.00  0.00
ATOM    216  HG2 GLN    13       5.636  -4.426   9.592  1.00  0.00
ATOM    217  HG3 GLN    13       5.022  -5.733  10.631  1.00  0.00
ATOM    218  CD  GLN    13       6.048  -6.394   8.883  1.00  0.00
ATOM    219  OE1 GLN    13       5.971  -6.619   7.684  1.00  0.00
ATOM    220  NE2 GLN    13       6.985  -6.959   9.627  1.00  0.00
ATOM    221 HE21 GLN    13       7.617  -7.630   9.214  1.00  0.00
ATOM    222 HE22 GLN    13       7.063  -6.717  10.604  1.00  0.00
ATOM    223  C   GLN    13       2.188  -3.548   8.247  1.00  0.00
```

ATOM	224	O	GLN	13	2.207	-4.084	7.178	1.00	0.00
ATOM	225	N	HIE	14	1.404	-2.502	8.501	1.00	0.00
ATOM	226	H	HIE	14	1.384	-2.137	9.442	1.00	0.00
ATOM	227	CA	HIE	14	0.509	-1.923	7.521	1.00	0.00
ATOM	228	HA	HIE	14	-0.099	-2.709	7.073	1.00	0.00
ATOM	229	CB	HIE	14	-0.421	-0.894	8.170	1.00	0.00
ATOM	230	HB2	HIE	14	0.041	0.093	8.175	1.00	0.00
ATOM	231	HB3	HIE	14	-1.342	-0.861	7.587	1.00	0.00
ATOM	232	CG	HIE	14	-0.820	-1.188	9.586	1.00	0.00
ATOM	233	ND1	HIE	14	-1.372	-0.260	10.434	1.00	0.00
ATOM	234	CE1	HIE	14	-1.596	-0.845	11.618	1.00	0.00
ATOM	235	HE1	HIE	14	-2.060	-0.294	12.436	1.00	0.00
ATOM	236	NE2	HIE	14	-1.163	-2.106	11.546	1.00	0.00
ATOM	237	HE2	HIE	14	-1.178	-2.778	12.300	1.00	0.00
ATOM	238	CD2	HIE	14	-0.692	-2.352	10.285	1.00	0.00
ATOM	239	HD2	HIE	14	-0.315	-3.342	10.027	1.00	0.00
ATOM	240	C	HIE	14	1.355	-1.341	6.398	1.00	0.00
ATOM	241	O	HIE	14	2.465	-1.752	6.100	1.00	0.00
ATOM	242	N	LEU	15	0.857	-0.264	5.793	1.00	0.00
ATOM	243	H	LEU	15	-0.072	0.055	6.026	1.00	0.00
ATOM	244	CA	LEU	15	1.560	0.401	4.720	1.00	0.00
ATOM	245	HA	LEU	15	2.560	-0.018	4.613	1.00	0.00

ATOM	246	CB	LEU	15	0.805	0.210	3.398	1.00	0.00
ATOM	247	HB2	LEU	15	-0.023	0.913	3.485	1.00	0.00
ATOM	248	HB3	LEU	15	1.442	0.502	2.563	1.00	0.00
ATOM	249	CG	LEU	15	0.255	-1.198	3.152	1.00	0.00
ATOM	250	HG	LEU	15	-0.601	-1.361	3.807	1.00	0.00
ATOM	251	CD1	LEU	15	-0.178	-1.365	1.700	1.00	0.00
ATOM	252	HD11	LEU	15	0.677	-1.203	1.044	1.00	0.00
ATOM	253	HD12	LEU	15	-0.565	-2.373	1.549	1.00	0.00
ATOM	254	HD13	LEU	15	-0.957	-0.639	1.466	1.00	0.00
ATOM	255	CD2	LEU	15	1.240	-2.262	3.544	1.00	0.00
ATOM	256	HD21	LEU	15	1.474	-2.169	4.604	1.00	0.00
ATOM	257	HD22	LEU	15	0.809	-3.245	3.353	1.00	0.00
ATOM	258	HD23	LEU	15	2.153	-2.146	2.959	1.00	0.00
ATOM	259	C	LEU	15	1.787	1.851	5.076	1.00	0.00
ATOM	260	O	LEU	15	1.194	2.349	6.039	1.00	0.00
ATOM	261	N	ARG	16	2.599	2.541	4.274	1.00	0.00
ATOM	262	H	ARG	16	3.014	2.083	3.475	1.00	0.00
ATOM	263	CA	ARG	16	2.908	3.933	4.502	1.00	0.00
ATOM	264	HA	ARG	16	2.049	4.426	4.958	1.00	0.00
ATOM	265	CB	ARG	16	4.109	4.085	5.433	1.00	0.00
ATOM	266	HB2	ARG	16	4.813	3.279	5.227	1.00	0.00
ATOM	267	HB3	ARG	16	4.586	5.044	5.232	1.00	0.00

```
ATOM    268  CG   ARG    16       3.681   4.026   6.900  1.00  0.00
ATOM    269  HG2 ARG    16       2.610   3.829   6.943  1.00  0.00
ATOM    270  HG3 ARG    16       4.219   3.213   7.388  1.00  0.00
ATOM    271  CD   ARG    16       3.987   5.343   7.616  1.00  0.00
ATOM    272  HD2 ARG    16       3.955   6.163   6.899  1.00  0.00
ATOM    273  HD3 ARG    16       3.246   5.514   8.396  1.00  0.00
ATOM    274  NE   ARG    16       5.326   5.308   8.235  1.00  0.00
ATOM    275  HE   ARG    16       5.963   4.610   7.879  1.00  0.00
ATOM    276  CZ   ARG    16       5.744   6.112   9.195  1.00  0.00
ATOM    277  NH1 ARG    16       4.949   7.040   9.677  1.00  0.00
ATOM    278 HH11 ARG    16       4.013   7.138   9.311  1.00  0.00
ATOM    279 HH12 ARG    16       5.278   7.650  10.411  1.00  0.00
ATOM    280  NH2 ARG    16       6.954   5.988   9.683  1.00  0.00
ATOM    281 HH21 ARG    16       7.570   5.273   9.322  1.00  0.00
ATOM    282 HH22 ARG    16       7.265   6.607  10.417  1.00  0.00
ATOM    283  C    ARG    16       3.230   4.587   3.207  1.00  0.00
ATOM    284  O    ARG    16       2.412   5.322   2.724  1.00  0.00
ATOM    285  N    ALA    17       4.366   4.300   2.609  1.00  0.00
ATOM    286  H    ALA    17       5.026   3.711   3.096  1.00  0.00
ATOM    287  CA   ALA    17       4.753   4.851   1.337  1.00  0.00
ATOM    288  HA   ALA    17       4.910   5.923   1.452  1.00  0.00
ATOM    289  CB   ALA    17       6.069   4.210   0.872  1.00  0.00
```

ATOM	290	HB1	ALA	17	5.926	3.137	0.743	1.00	0.00
ATOM	291	HB2	ALA	17	6.373	4.651	-0.077	1.00	0.00
ATOM	292	HB3	ALA	17	6.843	4.385	1.619	1.00	0.00
ATOM	293	C	ALA	17	3.629	4.677	0.297	1.00	0.00
ATOM	294	O	ALA	17	3.531	5.420	-0.670	1.00	0.00
ATOM	295	N	CYX	18	2.690	3.761	0.555	1.00	0.00
ATOM	296	H	CYX	18	2.791	3.203	1.391	1.00	0.00
ATOM	297	CA	CYX	18	1.593	3.487	-0.311	1.00	0.00
ATOM	298	HA	CYX	18	1.880	3.704	-1.340	1.00	0.00
ATOM	299	CB	CYX	18	1.172	2.026	-0.222	1.00	0.00
ATOM	300	HB2	CYX	18	1.982	1.440	0.213	1.00	0.00
ATOM	301	HB3	CYX	18	0.285	1.940	0.405	1.00	0.00
ATOM	302	SG	CYX	18	0.810	1.231	-1.805	1.00	0.00
ATOM	303	C	CYX	18	0.479	4.421	0.068	1.00	0.00
ATOM	304	O	CYX	18	-0.077	5.119	-0.762	1.00	0.00
ATOM	305	N	GLN	19	0.150	4.467	1.356	1.00	0.00
ATOM	306	H	GLN	19	0.583	3.818	1.997	1.00	0.00
ATOM	307	CA	GLN	19	-0.865	5.347	1.861	1.00	0.00
ATOM	308	HA	GLN	19	-1.583	5.584	1.076	1.00	0.00
ATOM	309	CB	GLN	19	-1.600	4.674	3.002	1.00	0.00
ATOM	310	HB2	GLN	19	-2.422	5.326	3.296	1.00	0.00
ATOM	311	HB3	GLN	19	-2.001	3.732	2.627	1.00	0.00

ATOM	312	CG	GLN	19	-0.711	4.402	4.209	1.00	0.00
ATOM	313	HG2	GLN	19	-0.891	3.386	4.562	1.00	0.00
ATOM	314	HG3	GLN	19	0.335	4.506	3.921	1.00	0.00
ATOM	315	CD	GLN	19	-1.001	5.371	5.346	1.00	0.00
ATOM	316	OE1	GLN	19	-0.115	6.003	5.921	1.00	0.00
ATOM	317	NE2	GLN	19	-2.276	5.492	5.688	1.00	0.00
ATOM	318	HE21	GLN	19	-2.538	6.118	6.435	1.00	0.00
ATOM	319	HE22	GLN	19	-2.980	4.957	5.199	1.00	0.00
ATOM	320	C	GLN	19	-0.282	6.674	2.277	1.00	0.00
ATOM	321	O	GLN	19	-0.778	7.288	3.192	1.00	0.00
ATOM	322	N	GLN	20	0.801	7.115	1.660	1.00	0.00
ATOM	323	H	GLN	20	1.180	6.547	0.915	1.00	0.00
ATOM	324	CA	GLN	20	1.491	8.332	2.011	1.00	0.00
ATOM	325	HA	GLN	20	0.845	8.926	2.657	1.00	0.00
ATOM	326	CB	GLN	20	2.810	8.073	2.743	1.00	0.00
ATOM	327	HB2	GLN	20	3.368	7.313	2.196	1.00	0.00
ATOM	328	HB3	GLN	20	3.384	8.999	2.765	1.00	0.00
ATOM	329	CG	GLN	20	2.562	7.591	4.180	1.00	0.00
ATOM	330	HG2	GLN	20	1.600	7.081	4.241	1.00	0.00
ATOM	331	HG3	GLN	20	3.360	6.892	4.433	1.00	0.00
ATOM	332	CD	GLN	20	2.574	8.715	5.177	1.00	0.00
ATOM	333	OE1	GLN	20	3.134	9.777	4.974	1.00	0.00

ATOM	334	NE2	GLN	20	1.905	8.504	6.299	1.00	0.00
ATOM	335	HE21	GLN	20	1.876	9.222	7.008	1.00	0.00
ATOM	336	HE22	GLN	20	1.427	7.625	6.440	1.00	0.00
ATOM	337	C	GLN	20	1.755	9.120	0.754	1.00	0.00
ATOM	338	O	GLN	20	1.572	10.328	0.730	1.00	0.00
ATOM	339	N	TRP	21	2.131	8.433	-0.325	1.00	0.00
ATOM	340	H	TRP	21	2.297	7.439	-0.255	1.00	0.00
ATOM	341	CA	TRP	21	2.376	9.082	-1.595	1.00	0.00
ATOM	342	HA	TRP	21	3.139	9.851	-1.477	1.00	0.00
ATOM	343	CB	TRP	21	2.863	8.066	-2.627	1.00	0.00
ATOM	344	HB2	TRP	21	3.365	8.579	-3.447	1.00	0.00
ATOM	345	HB3	TRP	21	3.566	7.393	-2.136	1.00	0.00
ATOM	346	CG	TRP	21	1.808	7.207	-3.243	1.00	0.00
ATOM	347	CD1	TRP	21	1.463	5.938	-2.935	1.00	0.00
ATOM	348	HD1	TRP	21	2.050	5.482	-2.138	1.00	0.00
ATOM	349	NE1	TRP	21	0.417	5.481	-3.715	1.00	0.00
ATOM	350	HE1	TRP	21	-0.022	4.574	-3.638	1.00	0.00
ATOM	351	CE2	TRP	21	0.049	6.472	-4.590	1.00	0.00
ATOM	352	CZ2	TRP	21	-0.938	6.547	-5.571	1.00	0.00
ATOM	353	HZ2	TRP	21	-1.560	5.666	-5.733	1.00	0.00
ATOM	354	CH2	TRP	21	-1.071	7.737	-6.284	1.00	0.00
ATOM	355	HH2	TRP	21	-1.814	7.822	-7.077	1.00	0.00

ATOM	356	CZ3	TRP	21	-0.255	8.841	-5.995	1.00	0.00
ATOM	357	HZ3	TRP	21	-0.401	9.782	-6.525	1.00	0.00
ATOM	358	CE3	TRP	21	0.759	8.779	-5.034	1.00	0.00
ATOM	359	HE3	TRP	21	1.390	9.654	-4.882	1.00	0.00
ATOM	360	CD2	TRP	21	0.913	7.585	-4.301	1.00	0.00
ATOM	361	C	TRP	21	1.101	9.776	-2.042	1.00	0.00
ATOM	362	O	TRP	21	1.118	10.846	-2.639	1.00	0.00
ATOM	363	N	ILE	22	-0.050	9.176	-1.715	1.00	0.00
ATOM	364	H	ILE	22	-0.024	8.292	-1.227	1.00	0.00
ATOM	365	CA	ILE	22	-1.339	9.716	-2.086	1.00	0.00
ATOM	366	HA	ILE	22	-1.352	10.045	-3.125	1.00	0.00
ATOM	367	CB	ILE	22	-2.434	8.664	-1.870	1.00	0.00
ATOM	368	HB	ILE	22	-2.488	8.416	-0.810	1.00	0.00
ATOM	369	CG2	ILE	22	-3.788	9.208	-2.336	1.00	0.00
ATOM	370	HG21	ILE	22	-3.735	9.455	-3.396	1.00	0.00
ATOM	371	HG22	ILE	22	-4.558	8.452	-2.178	1.00	0.00
ATOM	372	HG23	ILE	22	-4.036	10.103	-1.766	1.00	0.00
ATOM	373	CG1	ILE	22	-2.088	7.363	-2.601	1.00	0.00
ATOM	374	HG12	ILE	22	-2.353	7.475	-3.653	1.00	0.00
ATOM	375	HG13	ILE	22	-1.015	7.191	-2.513	1.00	0.00
ATOM	376	CD1	ILE	22	-2.843	6.176	-2.011	1.00	0.00
ATOM	377	HD11	ILE	22	-3.916	6.347	-2.099	1.00	0.00

ATOM	378	HD12	ILE	22	-2.575	5.268	-2.553	1.00	0.00
ATOM	379	HD13	ILE	22	-2.579	6.062	-0.960	1.00	0.00
ATOM	380	C	ILE	22	-1.586	10.967	-1.267	1.00	0.00
ATOM	381	O	ILE	22	-2.224	11.905	-1.721	1.00	0.00
ATOM	382	N	ARG	23	-1.064	11.005	-0.035	1.00	0.00
ATOM	383	H	ARG	23	-0.605	10.181	0.328	1.00	0.00
ATOM	384	CA	ARG	23	-1.164	12.166	0.819	1.00	0.00
ATOM	385	HA	ARG	23	-2.202	12.496	0.863	1.00	0.00
ATOM	386	CB	ARG	23	-0.694	11.851	2.232	1.00	0.00
ATOM	387	HB2	ARG	23	0.083	11.088	2.183	1.00	0.00
ATOM	388	HB3	ARG	23	-0.285	12.757	2.677	1.00	0.00
ATOM	389	CG	ARG	23	-1.851	11.343	3.086	1.00	0.00
ATOM	390	HG2	ARG	23	-1.699	11.668	4.115	1.00	0.00
ATOM	391	HG3	ARG	23	-2.781	11.765	2.706	1.00	0.00
ATOM	392	CD	ARG	23	-1.928	9.822	3.041	1.00	0.00
ATOM	393	HD2	ARG	23	-2.850	9.507	2.552	1.00	0.00
ATOM	394	HD3	ARG	23	-1.074	9.419	2.497	1.00	0.00
ATOM	395	NE	ARG	23	-1.912	9.277	4.426	1.00	0.00
ATOM	396	HE	ARG	23	-2.696	8.687	4.667	1.00	0.00
ATOM	397	CZ	ARG	23	-0.954	9.431	5.304	1.00	0.00
ATOM	398	NH1	ARG	23	0.073	10.184	5.002	1.00	0.00
ATOM	399	HH11	ARG	23	0.116	10.636	4.100	1.00	0.00

```
ATOM    400 HH12 ARG    23       0.818  10.309   5.672  1.00  0.00
ATOM    401  NH2 ARG    23      -1.055   8.911   6.501  1.00  0.00
ATOM    402 HH21 ARG    23      -1.880   8.385   6.752  1.00  0.00
ATOM    403 HH22 ARG    23      -0.308   9.038   7.168  1.00  0.00
ATOM    404  C   ARG    23      -0.349  13.336   0.273  1.00  0.00
ATOM    405  O   ARG    23      -0.563  14.483   0.666  1.00  0.00
ATOM    406  N   GLN    24       0.586  13.045  -0.625  1.00  0.00
ATOM    407  H   GLN    24       0.773  12.079  -0.854  1.00  0.00
ATOM    408  CA  GLN    24       1.392  14.062  -1.247  1.00  0.00
ATOM    409  HA  GLN    24       1.609  14.855  -0.531  1.00  0.00
ATOM    410  CB  GLN    24       2.712  13.465  -1.728  1.00  0.00
ATOM    411  HB2 GLN    24       2.586  12.391  -1.865  1.00  0.00
ATOM    412  HB3 GLN    24       2.982  13.923  -2.680  1.00  0.00
ATOM    413  CG  GLN    24       3.821  13.721  -0.707  1.00  0.00
ATOM    414  HG2 GLN    24       4.538  14.426  -1.127  1.00  0.00
ATOM    415  HG3 GLN    24       3.376  14.148   0.191  1.00  0.00
ATOM    416  CD  GLN    24       4.555  12.452  -0.327  1.00  0.00
ATOM    417  OE1 GLN    24       4.580  11.483  -1.058  1.00  0.00
ATOM    418  NE2 GLN    24       5.171  12.454   0.834  1.00  0.00
ATOM    419 HE21 GLN    24       5.676  11.633   1.136  1.00  0.00
ATOM    420 HE22 GLN    24       5.137  13.276   1.419  1.00  0.00
ATOM    421  C   GLN    24       0.656  14.720  -2.401  1.00  0.00
```

ATOM	422	O	GLN	24	1.085	15.749	-2.910	1.00	0.00
ATOM	423	N	GLN	25	-0.456	14.102	-2.827	1.00	0.00
ATOM	424	H	GLN	25	-0.715	13.208	-2.435	1.00	0.00
ATOM	425	CA	GLN	25	-1.266	14.643	-3.891	1.00	0.00
ATOM	426	HA	GLN	25	-0.629	14.971	-4.712	1.00	0.00
ATOM	427	CB	GLN	25	-2.233	13.578	-4.417	1.00	0.00
ATOM	428	HB2	GLN	25	-2.140	12.680	-3.807	1.00	0.00
ATOM	429	HB3	GLN	25	-3.252	13.958	-4.350	1.00	0.00
ATOM	430	CG	GLN	25	-1.911	13.240	-5.872	1.00	0.00
ATOM	431	HG2	GLN	25	-0.830	13.105	-5.894	1.00	0.00
ATOM	432	HG3	GLN	25	-2.397	12.325	-6.212	1.00	0.00
ATOM	433	CD	GLN	25	-2.279	14.367	-6.821	1.00	0.00
ATOM	434	OE1	GLN	25	-2.771	15.409	-6.468	1.00	0.00
ATOM	435	NE2	GLN	25	-2.129	14.121	-8.105	1.00	0.00
ATOM	436	HE21	GLN	25	-2.358	14.834	-8.783	1.00	0.00
ATOM	437	HE22	GLN	25	-1.785	13.220	-8.407	1.00	0.00
ATOM	438	C	GLN	25	-2.018	15.885	-3.417	1.00	0.00
ATOM	439	O	GLN	25	-2.631	16.616	-4.183	1.00	0.00
ATOM	440	N	LEU	26	-2.007	16.135	-2.111	1.00	0.00
ATOM	441	H	LEU	26	-1.584	15.459	-1.491	1.00	0.00
ATOM	442	CA	LEU	26	-2.682	17.263	-1.531	1.00	0.00
ATOM	443	HA	LEU	26	-3.200	17.822	-2.310	1.00	0.00

ATOM	444	CB	LEU	26	-3.713	16.801	-0.490	1.00	0.00
ATOM	445	HB2	LEU	26	-3.198	16.901	0.465	1.00	0.00
ATOM	446	HB3	LEU	26	-4.563	17.482	-0.518	1.00	0.00
ATOM	447	CG	LEU	26	-4.209	15.357	-0.654	1.00	0.00
ATOM	448	HG	LEU	26	-4.094	15.057	-1.696	1.00	0.00
ATOM	449	CD1	LEU	26	-3.409	14.419	0.233	1.00	0.00
ATOM	450	HD11	LEU	26	-3.524	14.717	1.275	1.00	0.00
ATOM	451	HD12	LEU	26	-3.773	13.399	0.105	1.00	0.00
ATOM	452	HD13	LEU	26	-2.356	14.466	-0.044	1.00	0.00
ATOM	453	CD2	LEU	26	-5.699	15.256	-0.332	1.00	0.00
ATOM	454	HD21	LEU	26	-6.262	15.899	-1.008	1.00	0.00
ATOM	455	HD22	LEU	26	-6.029	14.224	-0.455	1.00	0.00
ATOM	456	HD23	LEU	26	-5.871	15.572	0.697	1.00	0.00
ATOM	457	C	LEU	26	-1.649	18.233	-0.984	1.00	0.00
ATOM	458	O	LEU	26	-0.455	18.109	-1.227	1.00	0.00
ATOM	459	N	ALA	27	-2.122	19.198	-0.198	1.00	0.00
ATOM	460	H	ALA	27	-3.120	19.297	-0.075	1.00	0.00
ATOM	461	CA	ALA	27	-1.251	20.174	0.429	1.00	0.00
ATOM	462	HA	ALA	27	-0.355	20.325	-0.173	1.00	0.00
ATOM	463	CB	ALA	27	-1.998	21.498	0.531	1.00	0.00
ATOM	464	HB1	ALA	27	-2.893	21.365	1.138	1.00	0.00
ATOM	465	HB2	ALA	27	-1.353	22.245	0.994	1.00	0.00

ATOM	466	HB3	ALA	27	-2.283	21.833	-0.467	1.00	0.00
ATOM	467	C	ALA	27	-0.775	19.660	1.785	1.00	0.00
ATOM	468	O	ALA	27	-0.336	20.427	2.637	1.00	0.00
ATOM	469	N	GLY	28	-0.919	18.347	2.018	1.00	0.00
ATOM	470	H	GLY	28	-1.346	17.770	1.307	1.00	0.00
ATOM	471	CA	GLY	28	-0.490	17.717	3.245	1.00	0.00
ATOM	472	HA2	GLY	28	-0.698	18.388	4.078	1.00	0.00
ATOM	473	HA3	GLY	28	-1.046	16.789	3.379	1.00	0.00
ATOM	474	C	GLY	28	0.998	17.404	3.221	1.00	0.00
ATOM	475	O	GLY	28	1.476	16.640	4.054	1.00	0.00
ATOM	476	N	SER	29	1.718	17.949	2.240	1.00	0.00
ATOM	477	H	SER	29	1.244	18.492	1.533	1.00	0.00
ATOM	478	CA	SER	29	3.140	17.749	2.109	1.00	0.00
ATOM	479	HA	SER	29	3.592	17.613	3.091	1.00	0.00
ATOM	480	CB	SER	29	3.413	16.503	1.270	1.00	0.00
ATOM	481	HB2	SER	29	3.163	15.636	1.882	1.00	0.00
ATOM	482	HB3	SER	29	2.750	16.540	0.406	1.00	0.00
ATOM	483	OG	SER	29	4.753	16.399	0.831	1.00	0.00
ATOM	484	HG	SER	29	4.858	15.598	0.311	1.00	0.00
ATOM	485	C	SER	29	3.812	18.956	1.498	1.00	0.00
ATOM	486	O	SER	29	3.166	19.686	0.775	1.00	0.00
ATOM	487	N	PRO	30	5.071	19.200	1.864	1.00	0.00

ATOM	488	CD	PRO	30	5.854	18.389	2.780	1.00	0.00
ATOM	489	HD2	PRO	30	6.024	17.420	2.310	1.00	0.00
ATOM	490	HD3	PRO	30	5.271	18.253	3.691	1.00	0.00
ATOM	491	CG	PRO	30	7.149	19.155	3.027	1.00	0.00
ATOM	492	HG2	PRO	30	7.988	18.563	2.660	1.00	0.00
ATOM	493	HG3	PRO	30	7.264	19.323	4.098	1.00	0.00
ATOM	494	CB	PRO	30	7.030	20.483	2.275	1.00	0.00
ATOM	495	HB2	PRO	30	7.932	20.701	1.703	1.00	0.00
ATOM	496	HB3	PRO	30	6.829	21.304	2.963	1.00	0.00
ATOM	497	CA	PRO	30	5.848	20.296	1.329	1.00	0.00
ATOM	498	HA	PRO	30	5.237	21.197	1.378	1.00	0.00
ATOM	499	C	PRO	30	6.276	20.056	-0.098	1.00	0.00
ATOM	500	O	PRO	30	5.950	19.025	-0.675	1.00	0.00
ATOM	501	N	PHE	31	7.045	21.000	-0.652	1.00	0.00
ATOM	502	H	PHE	31	7.316	21.807	-0.109	1.00	0.00
ATOM	503	CA	PHE	31	7.516	20.902	-2.015	1.00	0.00
ATOM	504	HA	PHE	31	7.771	19.873	-2.269	1.00	0.00
ATOM	505	CB	PHE	31	6.386	21.368	-2.938	1.00	0.00
ATOM	506	HB2	PHE	31	6.318	22.454	-2.996	1.00	0.00
ATOM	507	HB3	PHE	31	6.612	20.966	-3.926	1.00	0.00
ATOM	508	CG	PHE	31	5.003	20.866	-2.572	1.00	0.00
ATOM	509	CD1	PHE	31	4.596	19.577	-2.951	1.00	0.00

ATOM	510	HD1	PHE	31	5.258	18.959	-3.559	1.00	0.00
ATOM	511	CE1	PHE	31	3.345	19.082	-2.552	1.00	0.00
ATOM	512	HE1	PHE	31	3.053	18.064	-2.811	1.00	0.00
ATOM	513	CZ	PHE	31	2.472	19.896	-1.821	1.00	0.00
ATOM	514	HZ	PHE	31	1.480	19.533	-1.550	1.00	0.00
ATOM	515	CE2	PHE	31	2.878	21.185	-1.436	1.00	0.00
ATOM	516	HE2	PHE	31	2.197	21.818	-0.867	1.00	0.00
ATOM	517	CD2	PHE	31	4.152	21.658	-1.781	1.00	0.00
ATOM	518	HD2	PHE	31	4.481	22.638	-1.436	1.00	0.00
ATOM	519	C	PHE	31	8.734	21.782	-2.235	1.00	0.00
ATOM	520	O	PHE	31	8.868	22.826	-1.593	1.00	0.00
ATOM	521	N	SER	32	9.614	21.355	-3.138	1.00	0.00
ATOM	522	H	SER	32	9.474	20.465	-3.596	1.00	0.00
ATOM	523	CA	SER	32	10.812	22.110	-3.454	1.00	0.00
ATOM	524	HA	SER	32	10.713	23.131	-3.087	1.00	0.00
ATOM	525	CB	SER	32	12.041	21.467	-2.793	1.00	0.00
ATOM	526	HB2	SER	32	11.728	20.536	-2.320	1.00	0.00
ATOM	527	HB3	SER	32	12.774	21.250	-3.570	1.00	0.00
ATOM	528	OG	SER	32	12.621	22.317	-1.823	1.00	0.00
ATOM	529	HG	SER	32	13.383	21.883	-1.433	1.00	0.00
ATOM	530	C	SER	32	10.993	22.190	-4.962	1.00	0.00
ATOM	531	O	SER	32	10.634	23.187	-5.588	1.00	0.00

ATOM	532	N	GLU	33	11.546	21.122	-5.546	1.00	0.00
ATOM	533	H	GLU	33	11.874	20.356	-4.976	1.00	0.00
ATOM	534	CA	GLU	33	11.779	21.064	-6.968	1.00	0.00
ATOM	535	HA	GLU	33	11.974	22.066	-7.350	1.00	0.00
ATOM	536	CB	GLU	33	12.989	20.175	-7.282	1.00	0.00
ATOM	537	HB2	GLU	33	13.260	19.628	-6.379	1.00	0.00
ATOM	538	HB3	GLU	33	12.706	19.469	-8.063	1.00	0.00
ATOM	539	CG	GLU	33	14.181	21.002	-7.752	1.00	0.00
ATOM	540	HG2	GLU	33	14.300	20.862	-8.827	1.00	0.00
ATOM	541	HG3	GLU	33	13.987	22.053	-7.542	1.00	0.00
ATOM	542	CD	GLU	33	15.459	20.568	-7.038	1.00	0.00
ATOM	543	OE1	GLU	33	15.840	19.388	-7.215	1.00	0.00
ATOM	544	OE2	GLU	33	16.030	21.421	-6.326	1.00	0.00
ATOM	545	C	GLU	33	10.523	20.593	-7.691	1.00	0.00
ATOM	546	O	GLU	33	9.440	20.499	-7.106	1.00	0.00
ATOM	547	N	ASN	34	10.675	20.268	-8.980	1.00	0.00
ATOM	548	H	ASN	34	11.573	20.393	-9.424	1.00	0.00
ATOM	549	CA	ASN	34	9.592	19.749	-9.768	1.00	0.00
ATOM	550	HA	ASN	34	8.642	20.142	-9.406	1.00	0.00
ATOM	551	CB	ASN	34	9.768	20.164	-11.227	1.00	0.00
ATOM	552	HB2	ASN	34	10.835	20.190	-11.450	1.00	0.00
ATOM	553	HB3	ASN	34	9.277	19.447	-11.885	1.00	0.00

ATOM	554	CG	ASN	34	9.186	21.536	-11.487	1.00	0.00
ATOM	555	OD1	ASN	34	8.118	21.891	-11.017	1.00	0.00
ATOM	556	ND2	ASN	34	9.902	22.312	-12.277	1.00	0.00
ATOM	557	HD21	ASN	34	9.576	23.242	-12.496	1.00	0.00
ATOM	558	HD22	ASN	34	10.773	21.972	-12.660	1.00	0.00
ATOM	559	C	ASN	34	9.549	18.239	-9.687	1.00	0.00
ATOM	560	O	ASN	34	10.595	17.593	-9.630	1.00	0.00
ATOM	561	N	GLN	35	8.349	17.675	-9.779	1.00	0.00
ATOM	562	H	GLN	35	7.531	18.266	-9.821	1.00	0.00
ATOM	563	CA	GLN	35	8.159	16.247	-9.761	1.00	0.00
ATOM	564	HA	GLN	35	8.661	15.801	-10.620	1.00	0.00
ATOM	565	CB	GLN	35	8.737	15.635	-8.485	1.00	0.00
ATOM	566	HB2	GLN	35	9.517	16.296	-8.109	1.00	0.00
ATOM	567	HB3	GLN	35	7.938	15.560	-7.747	1.00	0.00
ATOM	568	CG	GLN	35	9.324	14.251	-8.741	1.00	0.00
ATOM	569	HG2	GLN	35	9.559	13.768	-7.792	1.00	0.00
ATOM	570	HG3	GLN	35	8.586	13.655	-9.278	1.00	0.00
ATOM	571	CD	GLN	35	10.592	14.323	-9.577	1.00	0.00
ATOM	572	OE1	GLN	35	11.634	14.783	-9.154	1.00	0.00
ATOM	573	NE2	GLN	35	10.486	13.832	-10.801	1.00	0.00
ATOM	574	HE21	GLN	35	11.286	13.844	-11.418	1.00	0.00
ATOM	575	HE22	GLN	35	9.606	13.447	-11.113	1.00	0.00

ATOM	576	C	GLN	35	6.692	15.927	-9.894	1.00	0.00
ATOM	577	O	GLN	35	5.848	16.780	-9.615	1.00	0.00
ATOM	578	N	TRP	36	6.396	14.670	-10.273	1.00	0.00
ATOM	579	H	TRP	36	7.138	14.029	-10.516	1.00	0.00
ATOM	580	CA	TRP	36	5.021	14.215	-10.407	1.00	0.00
ATOM	581	HA	TRP	36	4.565	14.673	-11.285	1.00	0.00
ATOM	582	CB	TRP	36	4.970	12.692	-10.568	1.00	0.00
ATOM	583	HB2	TRP	36	5.254	12.407	-11.581	1.00	0.00
ATOM	584	HB3	TRP	36	5.661	12.240	-9.857	1.00	0.00
ATOM	585	CG	TRP	36	3.623	12.076	-10.309	1.00	0.00
ATOM	586	CD1	TRP	36	2.941	11.996	-9.133	1.00	0.00
ATOM	587	HD1	TRP	36	3.448	12.425	-8.269	1.00	0.00
ATOM	588	NE1	TRP	36	1.717	11.369	-9.294	1.00	0.00
ATOM	589	HE1	TRP	36	1.050	11.184	-8.559	1.00	0.00
ATOM	590	CE2	TRP	36	1.566	11.019	-10.615	1.00	0.00
ATOM	591	CZ2	TRP	36	0.538	10.364	-11.294	1.00	0.00
ATOM	592	HZ2	TRP	36	-0.342	10.061	-10.727	1.00	0.00
ATOM	593	CH2	TRP	36	0.702	10.136	-12.669	1.00	0.00
ATOM	594	HH2	TRP	36	-0.077	9.628	-13.238	1.00	0.00
ATOM	595	CZ3	TRP	36	1.868	10.557	-13.334	1.00	0.00
ATOM	596	HZ3	TRP	36	1.984	10.374	-14.403	1.00	0.00
ATOM	597	CE3	TRP	36	2.907	11.215	-12.663	1.00	0.00

ATOM	598	HE3	TRP	36	3.791	11.508	-13.229	1.00	0.00
ATOM	599	CD2	TRP	36	2.766	11.455	-11.282	1.00	0.00
ATOM	600	C	TRP	36	4.218	14.677	-9.183	1.00	0.00
ATOM	601	O	TRP	36	3.023	14.926	-9.275	1.00	0.00
ATOM	602	N	GLY	37	4.896	14.775	-8.027	1.00	0.00
ATOM	603	H	GLY	37	5.860	14.475	-7.992	1.00	0.00
ATOM	604	CA	GLY	37	4.300	15.293	-6.827	1.00	0.00
ATOM	605	HA2	GLY	37	3.856	16.265	-7.039	1.00	0.00
ATOM	606	HA3	GLY	37	3.524	14.606	-6.488	1.00	0.00
ATOM	607	C	GLY	37	5.333	15.451	-5.729	1.00	0.00
ATOM	608	O	GLY	37	5.875	16.536	-5.533	1.00	0.00
ATOM	609	N	PRO	38	5.632	14.342	-5.035	1.00	0.00
ATOM	610	CD	PRO	38	5.046	13.021	-5.229	1.00	0.00
ATOM	611	HD2	PRO	38	5.388	12.641	-6.192	1.00	0.00
ATOM	612	HD3	PRO	38	3.962	13.135	-5.248	1.00	0.00
ATOM	613	CG	PRO	38	5.521	12.163	-4.058	1.00	0.00
ATOM	614	HG2	PRO	38	6.131	11.344	-4.439	1.00	0.00
ATOM	615	HG3	PRO	38	4.654	11.757	-3.536	1.00	0.00
ATOM	616	CB	PRO	38	6.328	13.081	-3.143	1.00	0.00
ATOM	617	HB2	PRO	38	7.269	12.613	-2.853	1.00	0.00
ATOM	618	HB3	PRO	38	5.761	13.340	-2.249	1.00	0.00
ATOM	619	CA	PRO	38	6.604	14.330	-3.963	1.00	0.00

ATOM 620 HA PRO 38 6.413 15.183 -3.312 1.00 0.00
ATOM 621 C PRO 38 8.012 14.412 -4.498 1.00 0.00
ATOM 622 O PRO 38 8.725 13.424 -4.546 1.00 0.00
ATOM 623 N GLN 39 8.437 15.601 -4.915 1.00 0.00
ATOM 624 H GLN 39 7.808 16.390 -4.899 1.00 0.00
ATOM 625 CA GLN 39 9.764 15.794 -5.452 1.00 0.00
ATOM 626 HA GLN 39 9.921 15.133 -6.305 1.00 0.00
ATOM 627 CB GLN 39 9.938 17.245 -5.918 1.00 0.00
ATOM 628 HB2 GLN 39 9.117 17.510 -6.585 1.00 0.00
ATOM 629 HB3 GLN 39 9.930 17.905 -5.051 1.00 0.00
ATOM 630 CG GLN 39 11.269 17.396 -6.662 1.00 0.00
ATOM 631 HG2 GLN 39 11.400 16.566 -7.357 1.00 0.00
ATOM 632 HG3 GLN 39 11.231 18.333 -7.218 1.00 0.00
ATOM 633 CD GLN 39 12.460 17.436 -5.725 1.00 0.00
ATOM 634 OE1 GLN 39 12.350 17.819 -4.563 1.00 0.00
ATOM 635 NE2 GLN 39 13.598 17.010 -6.228 1.00 0.00
ATOM 636 HE21 GLN 39 14.431 17.010 -5.657 1.00 0.00
ATOM 637 HE22 GLN 39 13.634 16.684 -7.184 1.00 0.00
ATOM 638 C GLN 39 10.812 15.428 -4.414 1.00 0.00
ATOM 639 O GLN 39 11.874 14.911 -4.755 1.00 0.00
ATOM 640 N GLN 40 10.491 15.676 -3.140 1.00 0.00
ATOM 641 H GLN 40 9.623 16.142 -2.918 1.00 0.00

ATOM	642	CA	GLN	40	11.388	15.353	-2.061	1.00	0.00
ATOM	643	HA	GLN	40	12.423	15.450	-2.390	1.00	0.00
ATOM	644	CB	GLN	40	11.154	16.311	-0.894	1.00	0.00
ATOM	645	HB2	GLN	40	10.934	17.304	-1.286	1.00	0.00
ATOM	646	HB3	GLN	40	10.307	15.957	-0.307	1.00	0.00
ATOM	647	CG	GLN	40	12.401	16.378	-0.003	1.00	0.00
ATOM	648	HG2	GLN	40	13.228	15.960	-0.578	1.00	0.00
ATOM	649	HG3	GLN	40	12.632	17.408	0.269	1.00	0.00
ATOM	650	CD	GLN	40	12.235	15.562	1.275	1.00	0.00
ATOM	651	OE1	GLN	40	11.159	15.116	1.654	1.00	0.00
ATOM	652	NE2	GLN	40	13.333	15.401	1.993	1.00	0.00
ATOM	653	HE21	GLN	40	13.298	14.871	2.852	1.00	0.00
ATOM	654	HE22	GLN	40	14.202	15.808	1.679	1.00	0.00
ATOM	655	C	GLN	40	11.215	13.901	-1.622	1.00	0.00
ATOM	656	O	GLN	40	11.938	13.408	-0.766	1.00	0.00
ATOM	657	N	GLY	41	10.234	13.208	-2.201	1.00	0.00
ATOM	658	H	GLY	41	9.643	13.681	-2.869	1.00	0.00
ATOM	659	CA	GLY	41	9.973	11.816	-1.922	1.00	0.00
ATOM	660	HA2	GLY	41	10.905	11.330	-1.634	1.00	0.00
ATOM	661	HA3	GLY	41	9.258	11.745	-1.103	1.00	0.00
ATOM	662	C	GLY	41	9.404	11.110	-3.135	1.00	0.00
ATOM	663	O	GLY	41	8.280	10.651	-3.130	1.00	0.00

ATOM	664	N	PRO	42	10.157	11.040	-4.232	1.00	0.00
ATOM	665	CD	PRO	42	11.498	11.569	-4.370	1.00	0.00
ATOM	666	HD2	PRO	42	12.156	10.992	-3.720	1.00	0.00
ATOM	667	HD3	PRO	42	11.485	12.608	-4.043	1.00	0.00
ATOM	668	CG	PRO	42	11.861	11.428	-5.845	1.00	0.00
ATOM	669	HG2	PRO	42	12.711	10.751	-5.939	1.00	0.00
ATOM	670	HG3	PRO	42	12.132	12.407	-6.239	1.00	0.00
ATOM	671	CB	PRO	42	10.624	10.874	-6.559	1.00	0.00
ATOM	672	HB2	PRO	42	10.893	10.044	-7.212	1.00	0.00
ATOM	673	HB3	PRO	42	10.119	11.645	-7.141	1.00	0.00
ATOM	674	CA	PRO	42	9.707	10.380	-5.444	1.00	0.00
ATOM	675	HA	PRO	42	8.694	10.718	-5.662	1.00	0.00
ATOM	676	C	PRO	42	9.716	8.874	-5.303	1.00	0.00
ATOM	677	O	PRO	42	8.877	8.183	-5.894	1.00	0.00
ATOM	678	N	SER	43	10.651	8.376	-4.501	1.00	0.00
ATOM	679	H	SER	43	11.313	9.007	-4.074	1.00	0.00
ATOM	680	CA	SER	43	10.799	6.961	-4.257	1.00	0.00
ATOM	681	HA	SER	43	10.696	6.418	-5.197	1.00	0.00
ATOM	682	CB	SER	43	12.171	6.642	-3.656	1.00	0.00
ATOM	683	HB2	SER	43	12.069	6.589	-2.572	1.00	0.00
ATOM	684	HB3	SER	43	12.498	5.674	-4.037	1.00	0.00
ATOM	685	OG	SER	43	13.132	7.633	-3.994	1.00	0.00

ATOM	686	HG	SER	43	13.978	7.404	-3.603	1.00	0.00
ATOM	687	C	SER	43	9.683	6.481	-3.336	1.00	0.00
ATOM	688	O	SER	43	9.892	6.170	-2.170	1.00	0.00
ATOM	689	N	LEU	44	8.456	6.436	-3.853	1.00	0.00
ATOM	690	H	LEU	44	8.313	6.744	-4.804	1.00	0.00
ATOM	691	CA	LEU	44	7.311	6.013	-3.095	1.00	0.00
ATOM	692	HA	LEU	44	7.635	5.549	-2.163	1.00	0.00
ATOM	693	CB	LEU	44	6.421	7.210	-2.762	1.00	0.00
ATOM	694	HB2	LEU	44	6.639	7.956	-3.526	1.00	0.00
ATOM	695	HB3	LEU	44	5.377	6.906	-2.839	1.00	0.00
ATOM	696	CG	LEU	44	6.679	7.812	-1.370	1.00	0.00
ATOM	697	HG	LEU	44	7.670	7.507	-1.035	1.00	0.00
ATOM	698	CD1	LEU	44	6.603	9.320	-1.410	1.00	0.00
ATOM	699	HD11	LEU	44	5.612	9.625	-1.745	1.00	0.00
ATOM	700	HD12	LEU	44	6.789	9.720	-0.414	1.00	0.00
ATOM	701	HD13	LEU	44	7.353	9.704	-2.101	1.00	0.00
ATOM	702	CD2	LEU	44	5.692	7.284	-0.351	1.00	0.00
ATOM	703	HD21	LEU	44	5.785	6.200	-0.282	1.00	0.00
ATOM	704	HD22	LEU	44	5.901	7.728	0.622	1.00	0.00
ATOM	705	HD23	LEU	44	4.679	7.542	-0.658	1.00	0.00
ATOM	706	C	LEU	44	6.583	4.986	-3.901	1.00	0.00
ATOM	707	O	LEU	44	6.169	4.018	-3.335	1.00	0.00

ATOM	708	N	ARG	45	6.419	5.158	-5.208	1.00	0.00
ATOM	709	H	ARG	45	6.788	5.999	-5.627	1.00	0.00
ATOM	710	CA	ARG	45	5.745	4.207	-6.061	1.00	0.00
ATOM	711	HA	ARG	45	4.732	4.038	-5.695	1.00	0.00
ATOM	712	CB	ARG	45	5.665	4.728	-7.492	1.00	0.00
ATOM	713	HB2	ARG	45	6.667	5.009	-7.817	1.00	0.00
ATOM	714	HB3	ARG	45	5.287	3.930	-8.132	1.00	0.00
ATOM	715	CG	ARG	45	4.742	5.937	-7.588	1.00	0.00
ATOM	716	HG2	ARG	45	5.251	6.795	-7.149	1.00	0.00
ATOM	717	HG3	ARG	45	4.541	6.134	-8.641	1.00	0.00
ATOM	718	CD	ARG	45	3.414	5.696	-6.849	1.00	0.00
ATOM	719	HD2	ARG	45	3.182	4.631	-6.850	1.00	0.00
ATOM	720	HD3	ARG	45	3.497	6.048	-5.821	1.00	0.00
ATOM	721	NE	ARG	45	2.315	6.418	-7.507	1.00	0.00
ATOM	722	HE	ARG	45	1.576	5.855	-7.904	1.00	0.00
ATOM	723	CZ	ARG	45	2.213	7.732	-7.579	1.00	0.00
ATOM	724	NH1	ARG	45	3.118	8.506	-7.017	1.00	0.00
ATOM	725	HH11	ARG	45	3.898	8.092	-6.526	1.00	0.00
ATOM	726	HH12	ARG	45	3.028	9.510	-7.080	1.00	0.00
ATOM	727	NH2	ARG	45	1.225	8.279	-8.248	1.00	0.00
ATOM	728	HH21	ARG	45	0.544	7.691	-8.707	1.00	0.00
ATOM	729	HH22	ARG	45	1.151	9.285	-8.301	1.00	0.00

ATOM	730	C	ARG	45	6.478	2.908	-6.080	1.00	0.00
ATOM	731	O	ARG	45	6.001	1.958	-5.533	1.00	0.00
ATOM	732	N	GLU	46	7.679	2.871	-6.620	1.00	0.00
ATOM	733	H	GLU	46	8.057	3.711	-7.033	1.00	0.00
ATOM	734	CA	GLU	46	8.474	1.655	-6.681	1.00	0.00
ATOM	735	HA	GLU	46	7.960	0.905	-7.282	1.00	0.00
ATOM	736	CB	GLU	46	9.838	1.944	-7.319	1.00	0.00
ATOM	737	HB2	GLU	46	10.304	2.776	-6.792	1.00	0.00
ATOM	738	HB3	GLU	46	10.466	1.058	-7.224	1.00	0.00
ATOM	739	CG	GLU	46	9.680	2.302	-8.802	1.00	0.00
ATOM	740	HG2	GLU	46	9.442	1.385	-9.342	1.00	0.00
ATOM	741	HG3	GLU	46	8.866	3.016	-8.925	1.00	0.00
ATOM	742	CD	GLU	46	10.971	2.900	-9.358	1.00	0.00
ATOM	743	OE1	GLU	46	11.473	3.860	-8.728	1.00	0.00
ATOM	744	OE2	GLU	46	11.423	2.409	-10.412	1.00	0.00
ATOM	745	C	GLU	46	8.667	1.068	-5.283	1.00	0.00
ATOM	746	O	GLU	46	8.946	-0.119	-5.142	1.00	0.00
ATOM	747	N	GLN	47	8.482	1.904	-4.249	1.00	0.00
ATOM	748	H	GLN	47	8.303	2.880	-4.434	1.00	0.00
ATOM	749	CA	GLN	47	8.581	1.480	-2.889	1.00	0.00
ATOM	750	HA	GLN	47	9.347	0.710	-2.795	1.00	0.00
ATOM	751	CB	GLN	47	8.959	2.654	-1.996	1.00	0.00

ATOM	752	HB2	GLN	47	8.912	3.574	-2.578	1.00	0.00
ATOM	753	HB3	GLN	47	8.254	2.712	-1.167	1.00	0.00
ATOM	754	CG	GLN	47	10.379	2.469	-1.447	1.00	0.00
ATOM	755	HG2	GLN	47	10.457	1.487	-0.979	1.00	0.00
ATOM	756	HG3	GLN	47	11.109	2.545	-2.253	1.00	0.00
ATOM	757	CD	GLN	47	10.694	3.531	-0.404	1.00	0.00
ATOM	758	OE1	GLN	47	11.653	4.280	-0.505	1.00	0.00
ATOM	759	NE2	GLN	47	9.868	3.582	0.630	1.00	0.00
ATOM	760	HE21	GLN	47	10.019	4.263	1.360	1.00	0.00
ATOM	761	HE22	GLN	47	9.091	2.939	0.684	1.00	0.00
ATOM	762	C	GLN	47	7.293	0.867	-2.433	1.00	0.00
ATOM	763	O	GLN	47	7.343	-0.231	-1.967	1.00	0.00
ATOM	764	N	CYX	48	6.158	1.523	-2.570	1.00	0.00
ATOM	765	H	CYX	48	6.247	2.451	-2.958	1.00	0.00
ATOM	766	CA	CYX	48	4.858	1.069	-2.193	1.00	0.00
ATOM	767	HA	CYX	48	4.898	0.708	-1.165	1.00	0.00
ATOM	768	CB	CYX	48	3.806	2.164	-2.283	1.00	0.00
ATOM	769	HB2	CYX	48	3.503	2.460	-1.279	1.00	0.00
ATOM	770	HB3	CYX	48	4.221	3.025	-2.806	1.00	0.00
ATOM	771	SG	CYX	48	2.271	1.734	-3.140	1.00	0.00
ATOM	772	C	CYX	48	4.482	-0.076	-3.075	1.00	0.00
ATOM	773	O	CYX	48	4.024	-1.081	-2.607	1.00	0.00

ATOM 774 N CYX 49 4.711 0.011 -4.377 1.00 0.00
ATOM 775 H CYX 49 5.071 0.893 -4.711 1.00 0.00
ATOM 776 CA CYX 49 4.441 -1.024 -5.340 1.00 0.00
ATOM 777 HA CYX 49 3.363 -1.163 -5.418 1.00 0.00
ATOM 778 CB CYX 49 4.993 -0.680 -6.730 1.00 0.00
ATOM 779 HB2 CYX 49 6.074 -0.553 -6.671 1.00 0.00
ATOM 780 HB3 CYX 49 4.761 -1.488 -7.424 1.00 0.00
ATOM 781 SG CYX 49 4.372 0.851 -7.483 1.00 0.00
ATOM 782 C CYX 49 5.047 -2.324 -4.821 1.00 0.00
ATOM 783 O CYX 49 4.597 -3.398 -5.191 1.00 0.00
ATOM 784 N ASN 50 6.079 -2.228 -3.970 1.00 0.00
ATOM 785 H ASN 50 6.451 -1.312 -3.765 1.00 0.00
ATOM 786 CA ASN 50 6.667 -3.357 -3.325 1.00 0.00
ATOM 787 HA ASN 50 6.402 -4.276 -3.848 1.00 0.00
ATOM 788 CB ASN 50 8.183 -3.208 -3.341 1.00 0.00
ATOM 789 HB2 ASN 50 8.465 -2.191 -3.614 1.00 0.00
ATOM 790 HB3 ASN 50 8.567 -3.437 -2.347 1.00 0.00
ATOM 791 CG ASN 50 8.807 -4.164 -4.331 1.00 0.00
ATOM 792 OD1 ASN 50 9.375 -5.188 -3.983 1.00 0.00
ATOM 793 ND2 ASN 50 8.675 -3.828 -5.601 1.00 0.00
ATOM 794 HD21 ASN 50 9.067 -4.420 -6.320 1.00 0.00
ATOM 795 HD22 ASN 50 8.183 -2.982 -5.848 1.00 0.00

ATOM	796	C	ASN	50	6.226	-3.459	-1.896	1.00	0.00
ATOM	797	O	ASN	50	5.988	-4.538	-1.444	1.00	0.00
ATOM	798	N	GLU	51	6.113	-2.370	-1.167	1.00	0.00
ATOM	799	H	GLU	51	6.409	-1.514	-1.612	1.00	0.00
ATOM	800	CA	GLU	51	5.697	-2.315	0.206	1.00	0.00
ATOM	801	HA	GLU	51	6.279	-3.032	0.785	1.00	0.00
ATOM	802	CB	GLU	51	5.901	-0.919	0.806	1.00	0.00
ATOM	803	HB2	GLU	51	6.474	-1.019	1.728	1.00	0.00
ATOM	804	HB3	GLU	51	6.462	-0.315	0.093	1.00	0.00
ATOM	805	CG	GLU	51	4.566	-0.242	1.108	1.00	0.00
ATOM	806	HG2	GLU	51	4.023	-0.254	0.162	1.00	0.00
ATOM	807	HG3	GLU	51	3.985	-0.767	1.866	1.00	0.00
ATOM	808	CD	GLU	51	4.783	1.162	1.536	1.00	0.00
ATOM	809	OE1	GLU	51	5.048	1.462	2.717	1.00	0.00
ATOM	810	OE2	GLU	51	4.589	2.002	0.665	1.00	0.00
ATOM	811	C	GLU	51	4.244	-2.773	0.325	1.00	0.00
ATOM	812	O	GLU	51	3.747	-3.000	1.418	1.00	0.00
ATOM	813	N	LEU	52	3.550	-2.898	-0.800	1.00	0.00
ATOM	814	H	LEU	52	3.985	-2.584	-1.656	1.00	0.00
ATOM	815	CA	LEU	52	2.182	-3.310	-0.846	1.00	0.00
ATOM	816	HA	LEU	52	1.758	-3.318	0.158	1.00	0.00
ATOM	817	CB	LEU	52	1.374	-2.332	-1.712	1.00	0.00

ATOM	818	HB2	LEU	52	0.494	-2.162	-1.092	1.00	0.00
ATOM	819	HB3	LEU	52	1.920	-1.396	-1.831	1.00	0.00
ATOM	820	CG	LEU	52	0.938	-2.847	-3.088	1.00	0.00
ATOM	821	HG	LEU	52	0.571	-1.998	-3.665	1.00	0.00
ATOM	822	CD1	LEU	52	2.067	-3.514	-3.837	1.00	0.00
ATOM	823	HD11	LEU	52	2.434	-4.364	-3.261	1.00	0.00
ATOM	824	HD12	LEU	52	1.706	-3.861	-4.805	1.00	0.00
ATOM	825	HD13	LEU	52	2.877	-2.800	-3.986	1.00	0.00
ATOM	826	CD2	LEU	52	-0.262	-3.760	-2.964	1.00	0.00
ATOM	827	HD21	LEU	52	-1.091	-3.213	-2.515	1.00	0.00
ATOM	828	HD22	LEU	52	-0.554	-4.114	-3.953	1.00	0.00
ATOM	829	HD23	LEU	52	-0.007	-4.613	-2.335	1.00	0.00
ATOM	830	C	LEU	52	2.131	-4.766	-1.278	1.00	0.00
ATOM	831	O	LEU	52	1.137	-5.426	-1.032	1.00	0.00
ATOM	832	N	TYR	53	3.190	-5.247	-1.940	1.00	0.00
ATOM	833	H	TYR	53	4.007	-4.660	-2.021	1.00	0.00
ATOM	834	CA	TYR	53	3.240	-6.560	-2.505	1.00	0.00
ATOM	835	HA	TYR	53	2.235	-6.953	-2.658	1.00	0.00
ATOM	836	CB	TYR	53	3.945	-6.481	-3.859	1.00	0.00
ATOM	837	HB2	TYR	53	3.470	-5.675	-4.418	1.00	0.00
ATOM	838	HB3	TYR	53	4.986	-6.224	-3.665	1.00	0.00
ATOM	839	CG	TYR	53	3.883	-7.760	-4.669	1.00	0.00

ATOM	840	CD1	TYR	53	2.644	-8.303	-5.040	1.00	0.00
ATOM	841	HD1	TYR	53	1.731	-7.792	-4.735	1.00	0.00
ATOM	842	CE1	TYR	53	2.568	-9.485	-5.791	1.00	0.00
ATOM	843	HE1	TYR	53	1.604	-9.895	-6.093	1.00	0.00
ATOM	844	CZ	TYR	53	3.758	-10.136	-6.166	1.00	0.00
ATOM	845	OH	TYR	53	3.701	-11.292	-6.876	1.00	0.00
ATOM	846	HH	TYR	53	2.802	-11.572	-7.066	1.00	0.00
ATOM	847	CE2	TYR	53	5.010	-9.603	-5.798	1.00	0.00
ATOM	848	HE2	TYR	53	5.931	-10.109	-6.089	1.00	0.00
ATOM	849	CD2	TYR	53	5.067	-8.411	-5.050	1.00	0.00
ATOM	850	HD2	TYR	53	6.032	-7.992	-4.766	1.00	0.00
ATOM	851	C	TYR	53	4.038	-7.440	-1.596	1.00	0.00
ATOM	852	O	TYR	53	3.825	-8.632	-1.558	1.00	0.00
ATOM	853	N	GLN	54	4.994	-6.877	-0.869	1.00	0.00
ATOM	854	H	GLN	54	5.144	-5.887	-1.003	1.00	0.00
ATOM	855	CA	GLN	54	5.848	-7.577	0.049	1.00	0.00
ATOM	856	HA	GLN	54	6.474	-8.279	-0.502	1.00	0.00
ATOM	857	CB	GLN	54	6.756	-6.607	0.815	1.00	0.00
ATOM	858	HB2	GLN	54	7.313	-7.175	1.560	1.00	0.00
ATOM	859	HB3	GLN	54	7.452	-6.157	0.107	1.00	0.00
ATOM	860	CG	GLN	54	5.951	-5.507	1.509	1.00	0.00
ATOM	861	HG2	GLN	54	5.471	-4.858	0.776	1.00	0.00

ATOM	862	HG3	GLN	54	5.188	-5.995	2.116	1.00	0.00
ATOM	863	CD	GLN	54	6.818	-4.658	2.413	1.00	0.00
ATOM	864	OE1	GLN	54	7.984	-4.389	2.139	1.00	0.00
ATOM	865	NE2	GLN	54	6.233	-4.188	3.492	1.00	0.00
ATOM	866	HE21	GLN	54	6.757	-3.614	4.137	1.00	0.00
ATOM	867	HE22	GLN	54	5.262	-4.403	3.671	1.00	0.00
ATOM	868	C	GLN	54	4.998	-8.382	1.020	1.00	0.00
ATOM	869	O	GLN	54	5.445	-9.408	1.517	1.00	0.00
ATOM	870	N	GLU	55	3.763	-7.924	1.255	1.00	0.00
ATOM	871	H	GLU	55	3.492	-7.028	0.875	1.00	0.00
ATOM	872	CA	GLU	55	2.835	-8.610	2.108	1.00	0.00
ATOM	873	HA	GLU	55	3.373	-9.126	2.903	1.00	0.00
ATOM	874	CB	GLU	55	1.871	-7.622	2.737	1.00	0.00
ATOM	875	HB2	GLU	55	1.173	-7.287	1.969	1.00	0.00
ATOM	876	HB3	GLU	55	1.322	-8.134	3.527	1.00	0.00
ATOM	877	CG	GLU	55	2.598	-6.420	3.321	1.00	0.00
ATOM	878	HG2	GLU	55	3.529	-6.815	3.726	1.00	0.00
ATOM	879	HG3	GLU	55	2.822	-5.671	2.562	1.00	0.00
ATOM	880	CD	GLU	55	1.794	-5.787	4.432	1.00	0.00
ATOM	881	OE1	GLU	55	0.854	-6.410	4.959	1.00	0.00
ATOM	882	OE2	GLU	55	2.108	-4.622	4.678	1.00	0.00
ATOM	883	C	GLU	55	2.108	-9.705	1.359	1.00	0.00

ATOM	884	O	GLU	55	1.072	-10.165	1.797	1.00	0.00
ATOM	885	N	ASP	56	2.641	-10.153	0.233	1.00	0.00
ATOM	886	H	ASP	56	3.530	-9.756	-0.035	1.00	0.00
ATOM	887	CA	ASP	56	2.054	-11.143	-0.622	1.00	0.00
ATOM	888	HA	ASP	56	2.805	-11.562	-1.292	1.00	0.00
ATOM	889	CB	ASP	56	1.479	-12.275	0.237	1.00	0.00
ATOM	890	HB2	ASP	56	2.169	-12.307	1.080	1.00	0.00
ATOM	891	HB3	ASP	56	0.481	-12.020	0.593	1.00	0.00
ATOM	892	CG	ASP	56	1.442	-13.627	-0.434	1.00	0.00
ATOM	893	OD1	ASP	56	1.876	-13.714	-1.603	1.00	0.00
ATOM	894	OD2	ASP	56	0.887	-14.558	0.184	1.00	0.00
ATOM	895	C	ASP	56	0.940	-10.524	-1.454	1.00	0.00
ATOM	896	O	ASP	56	0.714	-9.307	-1.481	1.00	0.00
ATOM	897	N	GLN	57	0.176	-11.383	-2.140	1.00	0.00
ATOM	898	H	GLN	57	0.406	-12.367	-2.132	1.00	0.00
ATOM	899	CA	GLN	57	-0.940	-10.949	-2.942	1.00	0.00
ATOM	900	HA	GLN	57	-0.770	-9.934	-3.301	1.00	0.00
ATOM	901	CB	GLN	57	-1.107	-11.873	-4.149	1.00	0.00
ATOM	902	HB2	GLN	57	-0.187	-12.439	-4.294	1.00	0.00
ATOM	903	HB3	GLN	57	-1.930	-12.562	-3.959	1.00	0.00
ATOM	904	CG	GLN	57	-1.406	-11.058	-5.406	1.00	0.00
ATOM	905	HG2	GLN	57	-0.797	-10.154	-5.389	1.00	0.00

```
ATOM    906  HG3 GLN    57      -1.175 -11.635  -6.302  1.00  0.00
ATOM    907  CD  GLN    57      -2.860 -10.659  -5.452  1.00  0.00
ATOM    908  OE1 GLN    57      -3.751 -11.457  -5.596  1.00  0.00
ATOM    909  NE2 GLN    57      -3.128  -9.386  -5.352  1.00  0.00
ATOM    910 HE21 GLN    57      -4.088  -9.072  -5.378  1.00  0.00
ATOM    911 HE22 GLN    57      -2.375  -8.721  -5.249  1.00  0.00
ATOM    912  C   GLN    57      -2.216 -10.908  -2.128  1.00  0.00
ATOM    913  O   GLN    57      -3.147 -10.166  -2.421  1.00  0.00
ATOM    914  N   VAL    58      -2.256 -11.684  -1.053  1.00  0.00
ATOM    915  H   VAL    58      -1.478 -12.304  -0.879  1.00  0.00
ATOM    916  CA  VAL    58      -3.400 -11.751  -0.164  1.00  0.00
ATOM    917  HA  VAL    58      -4.338 -11.741  -0.719  1.00  0.00
ATOM    918  CB  VAL    58      -3.348 -13.026   0.687  1.00  0.00
ATOM    919  HB  VAL    58      -3.009 -12.772   1.691  1.00  0.00
ATOM    920  CG1 VAL    58      -4.737 -13.667   0.764  1.00  0.00
ATOM    921 HG11 VAL    58      -5.076 -13.922  -0.240  1.00  0.00
ATOM    922 HG12 VAL    58      -4.687 -14.572   1.371  1.00  0.00
ATOM    923 HG13 VAL    58      -5.437 -12.965   1.216  1.00  0.00
ATOM    924  CG2 VAL    58      -2.341 -14.035   0.125  1.00  0.00
ATOM    925 HG21 VAL    58      -1.347 -13.589   0.112  1.00  0.00
ATOM    926 HG22 VAL    58      -2.330 -14.927   0.752  1.00  0.00
ATOM    927 HG23 VAL    58      -2.629 -14.310  -0.890  1.00  0.00
```

ATOM 928 C VAL 58 -3.445 -10.491 0.679 1.00 0.00
ATOM 929 O VAL 58 -4.388 -10.316 1.448 1.00 0.00
ATOM 930 N CYX 59 -2.460 -9.605 0.503 1.00 0.00
ATOM 931 H CYX 59 -1.721 -9.827 -0.148 1.00 0.00
ATOM 932 CA CYX 59 -2.385 -8.368 1.219 1.00 0.00
ATOM 933 HA CYX 59 -3.215 -8.301 1.922 1.00 0.00
ATOM 934 CB CYX 59 -1.095 -8.272 1.988 1.00 0.00
ATOM 935 HB2 CYX 59 -0.275 -8.636 1.369 1.00 0.00
ATOM 936 HB3 CYX 59 -0.910 -7.233 2.261 1.00 0.00
ATOM 937 SG CYX 59 -1.019 -9.246 3.535 1.00 0.00
ATOM 938 C CYX 59 -2.496 -7.218 0.249 1.00 0.00
ATOM 939 O CYX 59 -3.081 -6.181 0.577 1.00 0.00
ATOM 940 N VAL 60 -2.053 -7.429 -0.999 1.00 0.00
ATOM 941 H VAL 60 -1.585 -8.298 -1.213 1.00 0.00
ATOM 942 CA VAL 60 -2.155 -6.432 -2.040 1.00 0.00
ATOM 943 HA VAL 60 -1.539 -5.551 -1.864 1.00 0.00
ATOM 944 CB VAL 60 -1.764 -7.039 -3.382 1.00 0.00
ATOM 945 HB VAL 60 -2.117 -8.070 -3.417 1.00 0.00
ATOM 946 CG1 VAL 60 -2.377 -6.253 -4.536 1.00 0.00
ATOM 947 HG11 VAL 60 -2.024 -5.223 -4.503 1.00 0.00
ATOM 948 HG12 VAL 60 -2.083 -6.707 -5.482 1.00 0.00
ATOM 949 HG13 VAL 60 -3.464 -6.268 -4.449 1.00 0.00

ATOM 950 CG2 VAL 60 -0.260 -7.136 -3.518 1.00 0.00
ATOM 951 HG21 VAL 60 0.137 -7.766 -2.722 1.00 0.00
ATOM 952 HG22 VAL 60 -0.010 -7.573 -4.485 1.00 0.00
ATOM 953 HG23 VAL 60 0.177 -6.140 -3.446 1.00 0.00
ATOM 954 C VAL 60 -3.555 -5.863 -2.070 1.00 0.00
ATOM 955 O VAL 60 -3.753 -4.738 -2.495 1.00 0.00
ATOM 956 N CYX 61 -4.547 -6.638 -1.639 1.00 0.00
ATOM 957 H CYX 61 -4.328 -7.602 -1.430 1.00 0.00
ATOM 958 CA CYX 61 -5.894 -6.216 -1.528 1.00 0.00
ATOM 959 HA CYX 61 -5.997 -5.490 -0.722 1.00 0.00
ATOM 960 CB CYX 61 -6.331 -5.562 -2.831 1.00 0.00
ATOM 961 HB2 CYX 61 -5.566 -5.719 -3.591 1.00 0.00
ATOM 962 HB3 CYX 61 -7.269 -6.006 -3.164 1.00 0.00
ATOM 963 SG CYX 61 -6.603 -3.772 -2.775 1.00 0.00
ATOM 964 C CYX 61 -6.757 -7.414 -1.152 1.00 0.00
ATOM 965 O CYX 61 -6.305 -8.537 -1.252 1.00 0.00
ATOM 966 N PRO 62 -8.006 -7.158 -0.729 1.00 0.00
ATOM 967 CD PRO 62 -9.015 -8.111 -0.312 1.00 0.00
ATOM 968 HD2 PRO 62 -8.661 -8.609 0.591 1.00 0.00
ATOM 969 HD3 PRO 62 -9.139 -8.847 -1.107 1.00 0.00
ATOM 970 CG PRO 62 -10.289 -7.289 -0.071 1.00 0.00
ATOM 971 HG2 PRO 62 -10.573 -7.383 0.977 1.00 0.00

```
ATOM    972  HG3 PRO    62     -11.086  -7.686  -0.700  1.00  0.00
ATOM    973  CB  PRO    62      -9.976  -5.837  -0.437  1.00  0.00
ATOM    974  HB2 PRO    62     -10.322  -5.127   0.313  1.00  0.00
ATOM    975  HB3 PRO    62     -10.412  -5.585  -1.404  1.00  0.00
ATOM    976  CA  PRO    62      -8.465  -5.797  -0.525  1.00  0.00
ATOM    977  HA  PRO    62      -8.230  -5.224  -1.422  1.00  0.00
ATOM    978  C   PRO    62      -7.825  -5.153   0.659  1.00  0.00
ATOM    979  O   PRO    62      -8.112  -4.034   0.953  1.00  0.00
ATOM    980  N   THR    63      -6.993  -5.865   1.374  1.00  0.00
ATOM    981  H   THR    63      -6.900  -6.833   1.100  1.00  0.00
ATOM    982  CA  THR    63      -6.258  -5.408   2.527  1.00  0.00
ATOM    983  HA  THR    63      -6.961  -5.501   3.355  1.00  0.00
ATOM    984  CB  THR    63      -5.078  -6.340   2.783  1.00  0.00
ATOM    985  HB  THR    63      -4.169  -5.832   2.461  1.00  0.00
ATOM    986  CG2 THR    63      -4.954  -6.718   4.235  1.00  0.00
ATOM    987 HG21 THR    63      -5.862  -7.227   4.557  1.00  0.00
ATOM    988 HG22 THR    63      -4.100  -7.382   4.366  1.00  0.00
ATOM    989 HG23 THR    63      -4.810  -5.819   4.834  1.00  0.00
ATOM    990  OG1 THR    63      -5.393  -7.563   2.130  1.00  0.00
ATOM    991  HG1 THR    63      -4.677  -8.190   2.261  1.00  0.00
ATOM    992  C   THR    63      -5.788  -4.000   2.354  1.00  0.00
ATOM    993  O   THR    63      -6.103  -3.223   3.231  1.00  0.00
```

ATOM 994 N LEU 64 -5.187 -3.651 1.217 1.00 0.00

ATOM 995 H LEU 64 -5.001 -4.376 0.538 1.00 0.00

ATOM 996 CA LEU 64 -4.747 -2.302 0.921 1.00 0.00

ATOM 997 HA LEU 64 -3.879 -2.048 1.529 1.00 0.00

ATOM 998 CB LEU 64 -4.358 -2.197 -0.552 1.00 0.00

ATOM 999 HB2 LEU 64 -4.177 -3.226 -0.862 1.00 0.00

ATOM 1000 HB3 LEU 64 -5.193 -1.789 -1.121 1.00 0.00

ATOM 1001 CG LEU 64 -3.102 -1.356 -0.813 1.00 0.00

ATOM 1002 HG LEU 64 -2.460 -1.396 0.067 1.00 0.00

ATOM 1003 CD1 LEU 64 -2.348 -1.886 -2.011 1.00 0.00

ATOM 1004 HD11 LEU 64 -2.989 -1.846 -2.892 1.00 0.00

ATOM 1005 HD12 LEU 64 -1.460 -1.277 -2.181 1.00 0.00

ATOM 1006 HD13 LEU 64 -2.050 -2.918 -1.826 1.00 0.00

ATOM 1007 CD2 LEU 64 -3.456 0.115 -1.011 1.00 0.00

ATOM 1008 HD21 LEU 64 -3.949 0.494 -0.116 1.00 0.00

ATOM 1009 HD22 LEU 64 -2.546 0.686 -1.194 1.00 0.00

ATOM 1010 HD23 LEU 64 -4.126 0.216 -1.865 1.00 0.00

ATOM 1011 C LEU 64 -5.822 -1.293 1.249 1.00 0.00

ATOM 1012 O LEU 64 -5.508 -0.275 1.854 1.00 0.00

ATOM 1013 N LYS 65 -7.093 -1.623 0.959 1.00 0.00

ATOM 1014 H LYS 65 -7.236 -2.444 0.387 1.00 0.00

ATOM 1015 CA LYS 65 -8.243 -0.830 1.315 1.00 0.00

ATOM	1016	HA	LYS	65	-8.299	0.039	0.659	1.00	0.00
ATOM	1017	CB	LYS	65	-9.544	-1.633	1.176	1.00	0.00
ATOM	1018	HB2	LYS	65	-9.658	-1.939	0.136	1.00	0.00
ATOM	1019	HB3	LYS	65	-9.479	-2.518	1.809	1.00	0.00
ATOM	1020	CG	LYS	65	-10.747	-0.797	1.594	1.00	0.00
ATOM	1021	HG2	LYS	65	-10.484	0.257	1.507	1.00	0.00
ATOM	1022	HG3	LYS	65	-11.576	-1.021	0.922	1.00	0.00
ATOM	1023	CD	LYS	65	-11.164	-1.102	3.039	1.00	0.00
ATOM	1024	HD2	LYS	65	-10.349	-1.622	3.542	1.00	0.00
ATOM	1025	HD3	LYS	65	-11.370	-0.165	3.555	1.00	0.00
ATOM	1026	CE	LYS	65	-12.416	-1.980	3.058	1.00	0.00
ATOM	1027	HE2	LYS	65	-12.978	-1.819	2.138	1.00	0.00
ATOM	1028	HE3	LYS	65	-12.117	-3.026	3.121	1.00	0.00
ATOM	1029	NZ	LYS	65	-13.263	-1.640	4.218	1.00	0.00
ATOM	1030	HZ1	LYS	65	-13.541	-0.671	4.159	1.00	0.00
ATOM	1031	HZ2	LYS	65	-14.085	-2.227	4.218	1.00	0.00
ATOM	1032	HZ3	LYS	65	-12.742	-1.790	5.070	1.00	0.00
ATOM	1033	C	LYS	65	-8.062	-0.259	2.703	1.00	0.00
ATOM	1034	O	LYS	65	-8.510	0.819	2.942	1.00	0.00
ATOM	1035	N	GLN	66	-7.430	-0.955	3.627	1.00	0.00
ATOM	1036	H	GLN	66	-7.242	-1.917	3.383	1.00	0.00
ATOM	1037	CA	GLN	66	-7.076	-0.510	4.924	1.00	0.00

ATOM	1038	HA	GLN	66	-7.963	-0.134	5.434	1.00	0.00
ATOM	1039	CB	GLN	66	-6.480	-1.650	5.756	1.00	0.00
ATOM	1040	HB2	GLN	66	-7.187	-2.479	5.788	1.00	0.00
ATOM	1041	HB3	GLN	66	-5.550	-1.984	5.295	1.00	0.00
ATOM	1042	CG	GLN	66	-6.196	-1.162	7.189	1.00	0.00
ATOM	1043	HG2	GLN	66	-5.918	-1.971	7.865	1.00	0.00
ATOM	1044	HG3	GLN	66	-5.368	-0.458	7.106	1.00	0.00
ATOM	1045	CD	GLN	66	-7.401	-0.431	7.773	1.00	0.00
ATOM	1046	OE1	GLN	66	-7.288	0.602	8.409	1.00	0.00
ATOM	1047	NE2	GLN	66	-8.589	-0.945	7.467	1.00	0.00
ATOM	1048	HE21	GLN	66	-9.430	-0.511	7.820	1.00	0.00
ATOM	1049	HE22	GLN	66	-8.645	-1.767	6.883	1.00	0.00
ATOM	1050	C	GLN	66	-6.130	0.664	4.906	1.00	0.00
ATOM	1051	O	GLN	66	-6.521	1.767	5.267	1.00	0.00
ATOM	1052	N	ALA	67	-4.875	0.441	4.522	1.00	0.00
ATOM	1053	H	ALA	67	-4.576	-0.506	4.335	1.00	0.00
ATOM	1054	CA	ALA	67	-3.899	1.500	4.420	1.00	0.00
ATOM	1055	HA	ALA	67	-3.680	1.911	5.405	1.00	0.00
ATOM	1056	CB	ALA	67	-2.611	0.940	3.829	1.00	0.00
ATOM	1057	HB1	ALA	67	-2.812	0.533	2.838	1.00	0.00
ATOM	1058	HB2	ALA	67	-1.870	1.736	3.751	1.00	0.00
ATOM	1059	HB3	ALA	67	-2.228	0.150	4.475	1.00	0.00

```
ATOM   1060  C   ALA    67      -4.452   2.644   3.577  1.00  0.00
ATOM   1061  O   ALA    67      -4.074   3.786   3.773  1.00  0.00
ATOM   1062  N   ALA    68      -5.429   2.369   2.720  1.00  0.00
ATOM   1063  H   ALA    68      -5.704   1.404   2.605  1.00  0.00
ATOM   1064  CA  ALA    68      -6.073   3.360   1.911  1.00  0.00
ATOM   1065  HA  ALA    68      -5.383   4.172   1.680  1.00  0.00
ATOM   1066  CB  ALA    68      -6.511   2.707   0.606  1.00  0.00
ATOM   1067  HB1 ALA    68      -7.209   1.898   0.820  1.00  0.00
ATOM   1068  HB2 ALA    68      -6.999   3.449  -0.027  1.00  0.00
ATOM   1069  HB3 ALA    68      -5.639   2.306   0.089  1.00  0.00
ATOM   1070  C   ALA    68      -7.252   3.978   2.649  1.00  0.00
ATOM   1071  O   ALA    68      -7.624   5.129   2.448  1.00  0.00
ATOM   1072  N   LYS    69      -7.864   3.237   3.561  1.00  0.00
ATOM   1073  H   LYS    69      -7.565   2.276   3.644  1.00  0.00
ATOM   1074  CA  LYS    69      -8.968   3.678   4.364  1.00  0.00
ATOM   1075  HA  LYS    69      -9.670   4.236   3.744  1.00  0.00
ATOM   1076  CB  LYS    69      -9.706   2.485   4.988  1.00  0.00
ATOM   1077  HB2 LYS    69     -10.064   1.836   4.189  1.00  0.00
ATOM   1078  HB3 LYS    69      -9.010   1.932   5.620  1.00  0.00
ATOM   1079  CG  LYS    69     -10.893   2.959   5.830  1.00  0.00
ATOM   1080  HG2 LYS    69     -11.520   2.098   6.064  1.00  0.00
ATOM   1081  HG3 LYS    69     -10.513   3.393   6.755  1.00  0.00
```

ATOM	1082	CD	LYS	69	-11.724	4.008	5.075	1.00	0.00
ATOM	1083	HD2	LYS	69	-11.181	4.953	5.081	1.00	0.00
ATOM	1084	HD3	LYS	69	-11.856	3.672	4.046	1.00	0.00
ATOM	1085	CE	LYS	69	-13.086	4.202	5.724	1.00	0.00
ATOM	1086	HE2	LYS	69	-13.814	3.574	5.210	1.00	0.00
ATOM	1087	HE3	LYS	69	-13.024	3.903	6.770	1.00	0.00
ATOM	1088	NZ	LYS	69	-13.508	5.613	5.640	1.00	0.00
ATOM	1089	HZ1	LYS	69	-13.566	5.889	4.670	1.00	0.00
ATOM	1090	HZ2	LYS	69	-14.413	5.720	6.076	1.00	0.00
ATOM	1091	HZ3	LYS	69	-12.834	6.195	6.116	1.00	0.00
ATOM	1092	C	LYS	69	-8.457	4.649	5.399	1.00	0.00
ATOM	1093	O	LYS	69	-9.143	5.599	5.717	1.00	0.00
ATOM	1094	N	SER	70	-7.236	4.463	5.884	1.00	0.00
ATOM	1095	H	SER	70	-6.717	3.648	5.590	1.00	0.00
ATOM	1096	CA	SER	70	-6.630	5.349	6.854	1.00	0.00
ATOM	1097	HA	SER	70	-7.398	5.965	7.322	1.00	0.00
ATOM	1098	CB	SER	70	-5.921	4.548	7.938	1.00	0.00
ATOM	1099	HB2	SER	70	-6.637	3.856	8.382	1.00	0.00
ATOM	1100	HB3	SER	70	-5.109	3.984	7.478	1.00	0.00
ATOM	1101	OG	SER	70	-5.395	5.397	8.947	1.00	0.00
ATOM	1102	HG	SER	70	-4.955	4.866	9.615	1.00	0.00
ATOM	1103	C	SER	70	-5.659	6.286	6.162	1.00	0.00

ATOM 1104 O SER 70 -4.855 6.974 6.793 1.00 0.00

ATOM 1105 N VAL 71 -5.719 6.339 4.826 1.00 0.00

ATOM 1106 H VAL 71 -6.335 5.709 4.332 1.00 0.00

ATOM 1107 CA VAL 71 -4.863 7.211 4.050 1.00 0.00

ATOM 1108 HA VAL 71 -3.806 7.113 4.297 1.00 0.00

ATOM 1109 CB VAL 71 -5.044 6.927 2.559 1.00 0.00

ATOM 1110 HB VAL 71 -5.512 5.948 2.446 1.00 0.00

ATOM 1111 CG1 VAL 71 -5.907 7.975 1.902 1.00 0.00

ATOM 1112 HG11 VAL 71 -5.440 8.953 2.014 1.00 0.00

ATOM 1113 HG12 VAL 71 -6.016 7.744 0.842 1.00 0.00

ATOM 1114 HG13 VAL 71 -6.889 7.985 2.374 1.00 0.00

ATOM 1115 CG2 VAL 71 -3.713 6.809 1.845 1.00 0.00

ATOM 1116 HG21 VAL 71 -3.137 5.993 2.282 1.00 0.00

ATOM 1117 HG22 VAL 71 -3.884 6.607 0.787 1.00 0.00

ATOM 1118 HG23 VAL 71 -3.159 7.741 1.951 1.00 0.00

ATOM 1119 C VAL 71 -5.172 8.654 4.422 1.00 0.00

ATOM 1120 O VAL 71 -4.462 9.578 4.084 1.00 0.00

ATOM 1121 N ARG 72 -6.298 8.908 5.082 1.00 0.00

ATOM 1122 H ARG 72 -6.931 8.143 5.269 1.00 0.00

ATOM 1123 CA ARG 72 -6.677 10.226 5.512 1.00 0.00

ATOM 1124 HA ARG 72 -5.799 10.795 5.817 1.00 0.00

ATOM 1125 CB ARG 72 -7.353 10.955 4.348 1.00 0.00

ATOM 1126 HB2 ARG 72 -7.334 10.312 3.467 1.00 0.00
ATOM 1127 HB3 ARG 72 -8.387 11.171 4.617 1.00 0.00
ATOM 1128 CG ARG 72 -6.623 12.261 4.041 1.00 0.00
ATOM 1129 HG2 ARG 72 -5.691 12.275 4.605 1.00 0.00
ATOM 1130 HG3 ARG 72 -6.402 12.289 2.974 1.00 0.00
ATOM 1131 CD ARG 72 -7.462 13.475 4.417 1.00 0.00
ATOM 1132 HD2 ARG 72 -8.195 13.655 3.630 1.00 0.00
ATOM 1133 HD3 ARG 72 -7.979 13.269 5.354 1.00 0.00
ATOM 1134 NE ARG 72 -6.647 14.682 4.585 1.00 0.00
ATOM 1135 HE ARG 72 -6.694 15.369 3.847 1.00 0.00
ATOM 1136 CZ ARG 72 -5.854 14.919 5.622 1.00 0.00
ATOM 1137 NH1 ARG 72 -5.795 14.062 6.617 1.00 0.00
ATOM 1138 HH11 ARG 72 -6.355 13.222 6.590 1.00 0.00
ATOM 1139 HH12 ARG 72 -5.190 14.247 7.404 1.00 0.00
ATOM 1140 NH2 ARG 72 -5.139 16.014 5.663 1.00 0.00
ATOM 1141 HH21 ARG 72 -5.193 16.677 4.903 1.00 0.00
ATOM 1142 HH22 ARG 72 -4.537 16.190 6.454 1.00 0.00
ATOM 1143 C ARG 72 -7.602 10.133 6.697 1.00 0.00
ATOM 1144 O ARG 72 -7.197 10.475 7.800 1.00 0.00
ATOM 1145 N VAL 73 -8.867 9.766 6.431 1.00 0.00
ATOM 1146 H VAL 73 -9.141 9.612 5.471 1.00 0.00
ATOM 1147 CA VAL 73 -9.879 9.665 7.456 1.00 0.00

```
ATOM   1148  HA  VAL    73     -10.163  10.634   7.866  1.00  0.00
ATOM   1149  CB  VAL    73     -11.127   8.993   6.883  1.00  0.00
ATOM   1150  HB  VAL    73     -11.297   9.388   5.882  1.00  0.00
ATOM   1151  CG1 VAL    73     -10.979   7.480   6.812  1.00  0.00
ATOM   1152 HG11 VAL    73     -10.809   7.083   7.813  1.00  0.00
ATOM   1153 HG12 VAL    73     -11.889   7.044   6.399  1.00  0.00
ATOM   1154 HG13 VAL    73     -10.133   7.226   6.173  1.00  0.00
ATOM   1155  CG2 VAL    73     -12.366   9.358   7.704  1.00  0.00
ATOM   1156 HG21 VAL    73     -12.509  10.438   7.686  1.00  0.00
ATOM   1157 HG22 VAL    73     -13.242   8.868   7.278  1.00  0.00
ATOM   1158 HG23 VAL    73     -12.231   9.027   8.734  1.00  0.00
ATOM   1159  C   VAL    73      -9.298   8.918   8.655  1.00  0.00
ATOM   1160  O   VAL    73      -8.332   8.155   8.532  1.00  0.00
ATOM   1161  N   GLN    74      -9.926   9.107   9.801  1.00  0.00
ATOM   1162  H   GLN    74     -10.701   9.752   9.856  1.00  0.00
ATOM   1163  CA  GLN    74      -9.482   8.462  11.019  1.00  0.00
ATOM   1164  HA  GLN    74      -9.498   7.379  10.890  1.00  0.00
ATOM   1165  CB  GLN    74      -8.052   8.893  11.365  1.00  0.00
ATOM   1166  HB2 GLN    74      -7.568   9.277  10.467  1.00  0.00
ATOM   1167  HB3 GLN    74      -8.089   9.677  12.121  1.00  0.00
ATOM   1168  CG  GLN    74      -7.255   7.700  11.904  1.00  0.00
ATOM   1169  HG2 GLN    74      -7.949   7.013  12.389  1.00  0.00
```

ATOM 1170 HG3 GLN 74 -6.748 7.186 11.087 1.00 0.00

ATOM 1171 CD GLN 74 -6.213 8.139 12.923 1.00 0.00

ATOM 1172 OE1 GLN 74 -5.870 9.306 13.052 1.00 0.00

ATOM 1173 NE2 GLN 74 -5.682 7.172 13.649 1.00 0.00

ATOM 1174 HE21 GLN 74 -4.984 7.392 14.345 1.00 0.00

ATOM 1175 HE22 GLN 74 -5.977 6.216 13.505 1.00 0.00

ATOM 1176 C GLN 74 -10.440 8.764 12.167 1.00 0.00

ATOM 1177 O GLN 74 -10.044 8.940 13.313 1.00 0.00

ATOM 1178 N GLY 75 -11.728 8.892 11.835 1.00 0.00

ATOM 1179 H GLY 75 -12.009 8.787 10.870 1.00 0.00

ATOM 1180 CA GLY 75 -12.749 9.179 12.820 1.00 0.00

ATOM 1181 HA2 GLY 75 -12.864 8.319 13.480 1.00 0.00

ATOM 1182 HA3 GLY 75 -12.450 10.048 13.405 1.00 0.00

ATOM 1183 C GLY 75 -14.072 9.467 12.154 1.00 0.00

ATOM 1184 O GLY 75 -15.060 8.791 12.424 1.00 0.00

ATOM 1185 N GLN 76 -14.072 10.427 11.216 1.00 0.00

ATOM 1186 H GLN 76 -13.220 10.933 11.023 1.00 0.00

ATOM 1187 CA GLN 76 -15.274 10.810 10.505 1.00 0.00

ATOM 1188 HA GLN 76 -15.586 9.991 9.857 1.00 0.00

ATOM 1189 CB GLN 76 -16.419 11.133 11.473 1.00 0.00

ATOM 1190 HB2 GLN 76 -16.158 10.768 12.466 1.00 0.00

ATOM 1191 HB3 GLN 76 -16.561 12.213 11.508 1.00 0.00

ATOM 1192 CG GLN 76 -17.715 10.464 11.008 1.00 0.00

ATOM 1193 HG2 GLN 76 -17.769 10.491 9.920 1.00 0.00

ATOM 1194 HG3 GLN 76 -17.742 9.428 11.346 1.00 0.00

ATOM 1195 CD GLN 76 -18.923 11.190 11.572 1.00 0.00

ATOM 1196 OE1 GLN 76 -19.498 12.057 10.935 1.00 0.00

ATOM 1197 NE2 GLN 76 -19.295 10.825 12.782 1.00 0.00

ATOM 1198 HE21 GLN 76 -20.092 11.268 13.217 1.00 0.00

ATOM 1199 HE22 GLN 76 -18.782 10.104 13.269 1.00 0.00

ATOM 1200 C GLN 76 -14.999 11.998 9.603 1.00 0.00

ATOM 1201 O GLN 76 -14.820 13.125 10.052 1.00 0.00

ATOM 1202 N HID 77 -14.882 11.730 8.304 1.00 0.00

ATOM 1203 H HID 77 -14.866 10.767 8.000 1.00 0.00

ATOM 1204 CA HID 77 -14.648 12.769 7.314 1.00 0.00

ATOM 1205 HA HID 77 -14.817 13.753 7.751 1.00 0.00

ATOM 1206 CB HID 77 -13.199 12.692 6.828 1.00 0.00

ATOM 1207 HB2 HID 77 -12.514 12.857 7.659 1.00 0.00

ATOM 1208 HB3 HID 77 -12.996 11.719 6.379 1.00 0.00

ATOM 1209 CG HID 77 -12.868 13.725 5.785 1.00 0.00

ATOM 1210 ND1 HID 77 -12.248 14.927 6.050 1.00 0.00

ATOM 1211 HD1 HID 77 -11.954 15.249 6.961 1.00 0.00

ATOM 1212 CE1 HID 77 -12.103 15.595 4.889 1.00 0.00

ATOM 1213 HE1 HID 77 -11.622 16.573 4.874 1.00 0.00

ATOM 1214 NE2 HID 77 -12.627 14.855 3.897 1.00 0.00
ATOM 1215 CD2 HID 77 -13.103 13.672 4.433 1.00 0.00
ATOM 1216 HD2 HID 77 -13.558 12.923 3.784 1.00 0.00
ATOM 1217 C HID 77 -15.680 12.671 6.197 1.00 0.00
ATOM 1218 O HID 77 -15.569 13.339 5.180 1.00 0.00
ATOM 1219 N GLY 78 -16.689 11.819 6.382 1.00 0.00
ATOM 1220 H GLY 78 -16.745 11.296 7.244 1.00 0.00
ATOM 1221 CA GLY 78 -17.713 11.613 5.390 1.00 0.00
ATOM 1222 HA2 GLY 78 -18.378 10.816 5.725 1.00 0.00
ATOM 1223 HA3 GLY 78 -18.283 12.534 5.271 1.00 0.00
ATOM 1224 C GLY 78 -17.110 11.222 4.039 1.00 0.00
ATOM 1225 O GLY 78 -15.887 11.046 3.884 1.00 0.00
ATOM 1226 N PRO 79 -17.987 11.020 3.055 1.00 0.00
ATOM 1227 CD PRO 79 -19.434 11.148 3.156 1.00 0.00
ATOM 1228 HD2 PRO 79 -19.670 12.199 3.318 1.00 0.00
ATOM 1229 HD3 PRO 79 -19.766 10.565 4.015 1.00 0.00
ATOM 1230 CG PRO 79 -19.998 10.615 1.835 1.00 0.00
ATOM 1231 HG2 PRO 79 -20.512 11.424 1.317 1.00 0.00
ATOM 1232 HG3 PRO 79 -20.706 9.814 2.049 1.00 0.00
ATOM 1233 CB PRO 79 -18.811 10.092 1.017 1.00 0.00
ATOM 1234 HB2 PRO 79 -18.844 10.434 -0.018 1.00 0.00
ATOM 1235 HB3 PRO 79 -18.779 9.002 1.038 1.00 0.00

ATOM	1236	CA	PRO	79	-17.578	10.663	1.714	1.00	0.00
ATOM	1237	HA	PRO	79	-16.854	9.851	1.785	1.00	0.00
ATOM	1238	C	PRO	79	-16.955	11.831	0.974	1.00	0.00
ATOM	1239	O	PRO	79	-17.657	12.681	0.447	1.00	0.00
ATOM	1240	N	PHE	80	-15.617	11.870	0.949	1.00	0.00
ATOM	1241	H	PHE	80	-15.079	11.135	1.387	1.00	0.00
ATOM	1242	CA	PHE	80	-14.904	12.948	0.300	1.00	0.00
ATOM	1243	HA	PHE	80	-15.463	13.337	-0.551	1.00	0.00
ATOM	1244	CB	PHE	80	-14.736	14.068	1.327	1.00	0.00
ATOM	1245	HB2	PHE	80	-15.580	14.075	2.017	1.00	0.00
ATOM	1246	HB3	PHE	80	-13.812	13.919	1.885	1.00	0.00
ATOM	1247	CG	PHE	80	-14.659	15.445	0.726	1.00	0.00
ATOM	1248	CD1	PHE	80	-15.829	16.086	0.297	1.00	0.00
ATOM	1249	HD1	PHE	80	-16.790	15.582	0.398	1.00	0.00
ATOM	1250	CE1	PHE	80	-15.766	17.373	-0.262	1.00	0.00
ATOM	1251	HE1	PHE	80	-16.682	17.863	-0.593	1.00	0.00
ATOM	1252	CZ	PHE	80	-14.533	18.027	-0.396	1.00	0.00
ATOM	1253	HZ	PHE	80	-14.478	19.024	-0.832	1.00	0.00
ATOM	1254	CE2	PHE	80	-13.363	17.385	0.037	1.00	0.00
ATOM	1255	HE2	PHE	80	-12.402	17.889	-0.062	1.00	0.00
ATOM	1256	CD2	PHE	80	-13.427	16.099	0.596	1.00	0.00
ATOM	1257	HD2	PHE	80	-12.515	15.604	0.931	1.00	0.00

ATOM 1258 C PHE 80 -13.541 12.482 -0.178 1.00 0.00
ATOM 1259 O PHE 80 -13.245 12.448 -1.361 1.00 0.00
ATOM 1260 N GLN 81 -12.693 12.085 0.779 1.00 0.00
ATOM 1261 H GLN 81 -12.999 12.086 1.741 1.00 0.00
ATOM 1262 CA GLN 81 -11.342 11.669 0.491 1.00 0.00
ATOM 1263 HA GLN 81 -10.884 12.386 -0.190 1.00 0.00
ATOM 1264 CB GLN 81 -10.496 11.588 1.755 1.00 0.00
ATOM 1265 HB2 GLN 81 -10.850 10.752 2.359 1.00 0.00
ATOM 1266 HB3 GLN 81 -9.458 11.415 1.470 1.00 0.00
ATOM 1267 CG GLN 81 -10.593 12.875 2.563 1.00 0.00
ATOM 1268 HG2 GLN 81 -9.739 13.502 2.307 1.00 0.00
ATOM 1269 HG3 GLN 81 -11.516 13.406 2.331 1.00 0.00
ATOM 1270 CD GLN 81 -10.558 12.593 4.052 1.00 0.00
ATOM 1271 OE1 GLN 81 -10.882 11.530 4.549 1.00 0.00
ATOM 1272 NE2 GLN 81 -10.176 13.600 4.806 1.00 0.00
ATOM 1273 HE21 GLN 81 -10.128 13.486 5.808 1.00 0.00
ATOM 1274 HE22 GLN 81 -9.932 14.482 4.379 1.00 0.00
ATOM 1275 C GLN 81 -11.411 10.329 -0.164 1.00 0.00
ATOM 1276 O GLN 81 -10.959 10.244 -1.274 1.00 0.00
ATOM 1277 N SER 82 -12.025 9.327 0.477 1.00 0.00
ATOM 1278 H SER 82 -12.424 9.514 1.385 1.00 0.00
ATOM 1279 CA SER 82 -12.143 7.984 -0.063 1.00 0.00

ATOM	1280	HA	SER	82	-11.212	7.440	0.097	1.00	0.00
ATOM	1281	CB	SER	82	-13.275	7.226	0.623	1.00	0.00
ATOM	1282	HB2	SER	82	-13.012	7.113	1.674	1.00	0.00
ATOM	1283	HB3	SER	82	-14.179	7.828	0.538	1.00	0.00
ATOM	1284	OG	SER	82	-13.502	5.958	0.058	1.00	0.00
ATOM	1285	HG	SER	82	-14.221	5.524	0.523	1.00	0.00
ATOM	1286	C	SER	82	-12.428	8.030	-1.547	1.00	0.00
ATOM	1287	O	SER	82	-11.725	7.406	-2.298	1.00	0.00
ATOM	1288	N	THR	83	-13.365	8.876	-1.963	1.00	0.00
ATOM	1289	H	THR	83	-13.932	9.351	-1.275	1.00	0.00
ATOM	1290	CA	THR	83	-13.687	9.059	-3.361	1.00	0.00
ATOM	1291	HA	THR	83	-14.266	8.192	-3.680	1.00	0.00
ATOM	1292	CB	THR	83	-14.554	10.308	-3.514	1.00	0.00
ATOM	1293	HB	THR	83	-14.379	10.935	-2.640	1.00	0.00
ATOM	1294	CG2	THR	83	-14.257	11.106	-4.780	1.00	0.00
ATOM	1295	HG21	THR	83	-14.432	10.479	-5.655	1.00	0.00
ATOM	1296	HG22	THR	83	-14.910	11.978	-4.822	1.00	0.00
ATOM	1297	HG23	THR	83	-13.217	11.431	-4.769	1.00	0.00
ATOM	1298	OG1	THR	83	-15.869	9.852	-3.638	1.00	0.00
ATOM	1299	HG1	THR	83	-16.460	10.602	-3.738	1.00	0.00
ATOM	1300	C	THR	83	-12.432	9.096	-4.223	1.00	0.00
ATOM	1301	O	THR	83	-12.107	8.135	-4.922	1.00	0.00

```
ATOM   1302  N    ARG    84     -11.675  10.196  -4.129  1.00  0.00
ATOM   1303  H    ARG    84     -11.955  10.938  -3.505  1.00  0.00
ATOM   1304  CA   ARG    84     -10.478  10.370  -4.898  1.00  0.00
ATOM   1305  HA   ARG    84     -10.675  10.124  -5.942  1.00  0.00
ATOM   1306  CB   ARG    84      -9.986  11.816  -4.821  1.00  0.00
ATOM   1307  HB2 ARG    84     -10.655  12.381  -4.172  1.00  0.00
ATOM   1308  HB3 ARG    84      -8.980  11.824  -4.401  1.00  0.00
ATOM   1309  CG   ARG    84      -9.963  12.457  -6.214  1.00  0.00
ATOM   1310  HG2 ARG    84     -10.992  12.641  -6.522  1.00  0.00
ATOM   1311  HG3 ARG    84      -9.429  13.405  -6.145  1.00  0.00
ATOM   1312  CD   ARG    84      -9.275  11.558  -7.246  1.00  0.00
ATOM   1313  HD2 ARG    84      -8.806  10.715  -6.738  1.00  0.00
ATOM   1314  HD3 ARG    84     -10.015  11.187  -7.956  1.00  0.00
ATOM   1315  NE   ARG    84      -8.240  12.298  -7.987  1.00  0.00
ATOM   1316  HE   ARG    84      -7.908  13.146  -7.550  1.00  0.00
ATOM   1317  CZ   ARG    84      -7.738  11.955  -9.156  1.00  0.00
ATOM   1318  NH1 ARG    84      -8.199  10.902  -9.790  1.00  0.00
ATOM   1319 HH11 ARG    84      -8.941  10.353  -9.379  1.00  0.00
ATOM   1320 HH12 ARG    84      -7.810  10.645 -10.686  1.00  0.00
ATOM   1321  NH2 ARG    84      -6.761  12.654  -9.686  1.00  0.00
ATOM   1322 HH21 ARG    84      -6.394  13.456  -9.195  1.00  0.00
ATOM   1323 HH22 ARG    84      -6.381  12.385 -10.583  1.00  0.00
```

ATOM	1324	C	ARG	84	-9.407	9.455	-4.379	1.00	0.00
ATOM	1325	O	ARG	84	-8.688	8.860	-5.162	1.00	0.00
ATOM	1326	N	ILE	85	-9.242	9.386	-3.066	1.00	0.00
ATOM	1327	H	ILE	85	-9.777	10.033	-2.505	1.00	0.00
ATOM	1328	CA	ILE	85	-8.294	8.532	-2.404	1.00	0.00
ATOM	1329	HA	ILE	85	-7.262	8.844	-2.564	1.00	0.00
ATOM	1330	CB	ILE	85	-8.576	8.449	-0.898	1.00	0.00
ATOM	1331	HB	ILE	85	-9.654	8.491	-0.741	1.00	0.00
ATOM	1332	CG2	ILE	85	-8.028	7.155	-0.310	1.00	0.00
ATOM	1333	HG21	ILE	85	-6.950	7.112	-0.466	1.00	0.00
ATOM	1334	HG22	ILE	85	-8.241	7.121	0.758	1.00	0.00
ATOM	1335	HG23	ILE	85	-8.500	6.303	-0.801	1.00	0.00
ATOM	1336	CG1	ILE	85	-8.034	9.691	-0.169	1.00	0.00
ATOM	1337	HG12	ILE	85	-8.575	10.566	-0.528	1.00	0.00
ATOM	1338	HG13	ILE	85	-8.210	9.569	0.900	1.00	0.00
ATOM	1339	CD1	ILE	85	-6.539	9.878	-0.421	1.00	0.00
ATOM	1340	HD11	ILE	85	-6.363	10.000	-1.490	1.00	0.00
ATOM	1341	HD12	ILE	85	-6.189	10.764	0.108	1.00	0.00
ATOM	1342	HD13	ILE	85	-5.997	9.003	-0.062	1.00	0.00
ATOM	1343	C	ILE	85	-8.288	7.167	-3.063	1.00	0.00
ATOM	1344	O	ILE	85	-7.234	6.585	-3.214	1.00	0.00
ATOM	1345	N	TYR	86	-9.434	6.710	-3.542	1.00	0.00

ATOM 1346 H TYR 86 -10.249 7.296 -3.432 1.00 0.00

ATOM 1347 CA TYR 86 -9.582 5.466 -4.228 1.00 0.00

ATOM 1348 HA TYR 86 -8.896 4.712 -3.842 1.00 0.00

ATOM 1349 CB TYR 86 -11.008 4.993 -3.989 1.00 0.00

ATOM 1350 HB2 TYR 86 -11.628 5.888 -3.949 1.00 0.00

ATOM 1351 HB3 TYR 86 -11.300 4.386 -4.847 1.00 0.00

ATOM 1352 CG TYR 86 -11.196 4.192 -2.717 1.00 0.00

ATOM 1353 CD1 TYR 86 -10.884 4.751 -1.466 1.00 0.00

ATOM 1354 HD1 TYR 86 -10.507 5.772 -1.420 1.00 0.00

ATOM 1355 CE1 TYR 86 -11.052 4.011 -0.286 1.00 0.00

ATOM 1356 HE1 TYR 86 -10.773 4.428 0.681 1.00 0.00

ATOM 1357 CZ TYR 86 -11.537 2.698 -0.370 1.00 0.00

ATOM 1358 OH TYR 86 -11.749 2.005 0.774 1.00 0.00

ATOM 1359 HH TYR 86 -11.535 2.508 1.563 1.00 0.00

ATOM 1360 CE2 TYR 86 -11.852 2.119 -1.611 1.00 0.00

ATOM 1361 HE2 TYR 86 -12.230 1.098 -1.670 1.00 0.00

ATOM 1362 CD2 TYR 86 -11.674 2.872 -2.787 1.00 0.00

ATOM 1363 HD2 TYR 86 -11.907 2.431 -3.757 1.00 0.00

ATOM 1364 C TYR 86 -9.375 5.633 -5.709 1.00 0.00

ATOM 1365 O TYR 86 -8.708 4.831 -6.343 1.00 0.00

ATOM 1366 N GLN 87 -9.880 6.716 -6.292 1.00 0.00

ATOM 1367 H GLN 87 -10.440 7.344 -5.733 1.00 0.00

ATOM 1368 CA GLN 87 -9.712 7.013 -7.690 1.00 0.00
ATOM 1369 HA GLN 87 -10.042 6.164 -8.289 1.00 0.00
ATOM 1370 CB GLN 87 -10.536 8.235 -8.081 1.00 0.00
ATOM 1371 HB2 GLN 87 -11.257 8.442 -7.291 1.00 0.00
ATOM 1372 HB3 GLN 87 -9.868 9.089 -8.196 1.00 0.00
ATOM 1373 CG GLN 87 -11.278 7.988 -9.396 1.00 0.00
ATOM 1374 HG2 GLN 87 -10.660 8.049 -10.292 1.00 0.00
ATOM 1375 HG3 GLN 87 -11.653 6.971 -9.283 1.00 0.00
ATOM 1376 CD GLN 87 -12.464 8.929 -9.557 1.00 0.00
ATOM 1377 OE1 GLN 87 -12.514 9.995 -8.954 1.00 0.00
ATOM 1378 NE2 GLN 87 -13.330 8.595 -10.479 1.00 0.00
ATOM 1379 HE21 GLN 87 -14.141 9.175 -10.640 1.00 0.00
ATOM 1380 HE22 GLN 87 -13.183 7.758 -11.026 1.00 0.00
ATOM 1381 C GLN 87 -8.249 7.242 -8.019 1.00 0.00
ATOM 1382 O GLN 87 -7.874 7.340 -9.181 1.00 0.00
ATOM 1383 N ILE 88 -7.384 7.351 -7.025 1.00 0.00
ATOM 1384 H ILE 88 -7.757 7.357 -6.086 1.00 0.00
ATOM 1385 CA ILE 88 -5.971 7.533 -7.197 1.00 0.00
ATOM 1386 HA ILE 88 -5.695 7.533 -8.252 1.00 0.00
ATOM 1387 CB ILE 88 -5.545 8.856 -6.558 1.00 0.00
ATOM 1388 HB ILE 88 -5.888 8.874 -5.524 1.00 0.00
ATOM 1389 CG2 ILE 88 -4.026 9.008 -6.593 1.00 0.00

ATOM 1390 HG21 ILE 88 -3.682 8.990 -7.627 1.00 0.00

ATOM 1391 HG22 ILE 88 -3.745 9.955 -6.134 1.00 0.00

ATOM 1392 HG23 ILE 88 -3.565 8.187 -6.043 1.00 0.00

ATOM 1393 CG1 ILE 88 -6.211 10.035 -7.272 1.00 0.00

ATOM 1394 HG12 ILE 88 -5.668 10.250 -8.192 1.00 0.00

ATOM 1395 HG13 ILE 88 -7.242 9.773 -7.512 1.00 0.00

ATOM 1396 CD1 ILE 88 -6.196 11.275 -6.367 1.00 0.00

ATOM 1397 HD11 ILE 88 -5.166 11.537 -6.127 1.00 0.00

ATOM 1398 HD12 ILE 88 -6.672 12.108 -6.884 1.00 0.00

ATOM 1399 HD13 ILE 88 -6.740 11.060 -5.447 1.00 0.00

ATOM 1400 C ILE 88 -5.246 6.341 -6.607 1.00 0.00

ATOM 1401 O ILE 88 -4.063 6.185 -6.820 1.00 0.00

ATOM 1402 N ALA 89 -5.946 5.480 -5.889 1.00 0.00

ATOM 1403 H ALA 89 -6.911 5.697 -5.682 1.00 0.00

ATOM 1404 CA ALA 89 -5.379 4.282 -5.323 1.00 0.00

ATOM 1405 HA ALA 89 -4.290 4.329 -5.350 1.00 0.00

ATOM 1406 CB ALA 89 -5.824 4.161 -3.875 1.00 0.00

ATOM 1407 HB1 ALA 89 -6.912 4.103 -3.832 1.00 0.00

ATOM 1408 HB2 ALA 89 -5.396 3.259 -3.437 1.00 0.00

ATOM 1409 HB3 ALA 89 -5.485 5.033 -3.316 1.00 0.00

ATOM 1410 C ALA 89 -5.779 3.074 -6.153 1.00 0.00

ATOM 1411 O ALA 89 -5.443 1.940 -5.824 1.00 0.00

ATOM	1412	N	LYS	90	-6.494	3.314	-7.258	1.00	0.00
ATOM	1413	H	LYS	90	-6.799	4.259	-7.443	1.00	0.00
ATOM	1414	CA	LYS	90	-6.945	2.261	-8.144	1.00	0.00
ATOM	1415	HA	LYS	90	-6.898	1.300	-7.632	1.00	0.00
ATOM	1416	CB	LYS	90	-8.395	2.515	-8.578	1.00	0.00
ATOM	1417	HB2	LYS	90	-8.912	1.555	-8.594	1.00	0.00
ATOM	1418	HB3	LYS	90	-8.857	3.164	-7.834	1.00	0.00
ATOM	1419	CG	LYS	90	-8.493	3.165	-9.948	1.00	0.00
ATOM	1420	HG2	LYS	90	-7.910	2.574	-10.655	1.00	0.00
ATOM	1421	HG3	LYS	90	-9.539	3.169	-10.256	1.00	0.00
ATOM	1422	CD	LYS	90	-7.969	4.583	-9.923	1.00	0.00
ATOM	1423	HD2	LYS	90	-8.654	5.201	-9.342	1.00	0.00
ATOM	1424	HD3	LYS	90	-6.987	4.586	-9.450	1.00	0.00
ATOM	1425	CE	LYS	90	-7.854	5.144	-11.339	1.00	0.00
ATOM	1426	HE2	LYS	90	-7.429	4.375	-11.985	1.00	0.00
ATOM	1427	HE3	LYS	90	-8.852	5.403	-11.693	1.00	0.00
ATOM	1428	NZ	LYS	90	-6.996	6.341	-11.370	1.00	0.00
ATOM	1429	HZ1	LYS	90	-6.071	6.101	-11.043	1.00	0.00
ATOM	1430	HZ2	LYS	90	-6.938	6.689	-12.316	1.00	0.00
ATOM	1431	HZ3	LYS	90	-7.390	7.053	-10.773	1.00	0.00
ATOM	1432	C	LYS	90	-5.988	2.141	-9.301	1.00	0.00
ATOM	1433	O	LYS	90	-6.006	1.167	-10.035	1.00	0.00

ATOM 1434 N ASN 91 -5.184 3.190 -9.519 1.00 0.00
ATOM 1435 H ASN 91 -5.281 4.005 -8.931 1.00 0.00
ATOM 1436 CA ASN 91 -4.202 3.219 -10.566 1.00 0.00
ATOM 1437 HA ASN 91 -4.523 2.582 -11.391 1.00 0.00
ATOM 1438 CB ASN 91 -4.019 4.644 -11.091 1.00 0.00
ATOM 1439 HB2 ASN 91 -3.141 4.632 -11.736 1.00 0.00
ATOM 1440 HB3 ASN 91 -4.888 4.962 -11.667 1.00 0.00
ATOM 1441 CG ASN 91 -3.788 5.635 -9.982 1.00 0.00
ATOM 1442 OD1 ASN 91 -3.146 5.334 -9.005 1.00 0.00
ATOM 1443 ND2 ASN 91 -4.373 6.808 -10.134 1.00 0.00
ATOM 1444 HD21 ASN 91 -4.260 7.520 -9.427 1.00 0.00
ATOM 1445 HD22 ASN 91 -4.931 6.987 -10.957 1.00 0.00
ATOM 1446 C ASN 91 -2.864 2.696 -10.053 1.00 0.00
ATOM 1447 O ASN 91 -1.868 2.731 -10.767 1.00 0.00
ATOM 1448 N LEU 92 -2.829 2.293 -8.787 1.00 0.00
ATOM 1449 H LEU 92 -3.648 2.422 -8.210 1.00 0.00
ATOM 1450 CA LEU 92 -1.647 1.743 -8.179 1.00 0.00
ATOM 1451 HA LEU 92 -0.775 2.338 -8.449 1.00 0.00
ATOM 1452 CB LEU 92 -1.795 1.754 -6.651 1.00 0.00
ATOM 1453 HB2 LEU 92 -2.872 1.771 -6.486 1.00 0.00
ATOM 1454 HB3 LEU 92 -1.377 0.841 -6.226 1.00 0.00
ATOM 1455 CG LEU 92 -1.155 2.972 -5.975 1.00 0.00

ATOM 1456 HG LEU 92 -1.337 3.856 -6.586 1.00 0.00

ATOM 1457 CD1 LEU 92 -1.756 3.182 -4.583 1.00 0.00

ATOM 1458 HD11 LEU 92 -1.574 2.298 -3.971 1.00 0.00

ATOM 1459 HD12 LEU 92 -1.293 4.050 -4.114 1.00 0.00

ATOM 1460 HD13 LEU 92 -2.830 3.347 -4.672 1.00 0.00

ATOM 1461 CD2 LEU 92 0.361 2.828 -5.906 1.00 0.00

ATOM 1462 HD21 LEU 92 0.764 2.737 -6.915 1.00 0.00

ATOM 1463 HD22 LEU 92 0.788 3.706 -5.422 1.00 0.00

ATOM 1464 HD23 LEU 92 0.616 1.937 -5.332 1.00 0.00

ATOM 1465 C LEU 92 -1.391 0.344 -8.709 1.00 0.00

ATOM 1466 O LEU 92 -0.309 0.085 -9.194 1.00 0.00

ATOM 1467 N PRO 93 -2.407 -0.533 -8.698 1.00 0.00

ATOM 1468 CD PRO 93 -3.751 -0.282 -8.205 1.00 0.00

ATOM 1469 HD2 PRO 93 -4.212 0.448 -8.870 1.00 0.00

ATOM 1470 HD3 PRO 93 -3.659 0.144 -7.206 1.00 0.00

ATOM 1471 CG PRO 93 -4.482 -1.607 -8.202 1.00 0.00

ATOM 1472 HG2 PRO 93 -5.322 -1.556 -8.895 1.00 0.00

ATOM 1473 HG3 PRO 93 -4.853 -1.811 -7.198 1.00 0.00

ATOM 1474 CB PRO 93 -3.464 -2.665 -8.634 1.00 0.00

ATOM 1475 HB2 PRO 93 -3.875 -3.340 -9.385 1.00 0.00

ATOM 1476 HB3 PRO 93 -3.126 -3.242 -7.774 1.00 0.00

ATOM 1477 CA PRO 93 -2.295 -1.879 -9.227 1.00 0.00

ATOM	1478	HA	PRO	93	-1.378	-2.319	-8.834	1.00	0.00
ATOM	1479	C	PRO	93	-2.260	-1.909	-10.745	1.00	0.00
ATOM	1480	O	PRO	93	-2.137	-2.976	-11.342	1.00	0.00
ATOM	1481	N	ASN	94	-2.367	-0.726	-11.355	1.00	0.00
ATOM	1482	H	ASN	94	-2.470	0.113	-10.802	1.00	0.00
ATOM	1483	CA	ASN	94	-2.336	-0.600	-12.782	1.00	0.00
ATOM	1484	HA	ASN	94	-2.773	-1.485	-13.245	1.00	0.00
ATOM	1485	CB	ASN	94	-3.141	0.626	-13.216	1.00	0.00
ATOM	1486	HB2	ASN	94	-2.911	1.461	-12.555	1.00	0.00
ATOM	1487	HB3	ASN	94	-2.861	0.884	-14.237	1.00	0.00
ATOM	1488	CG	ASN	94	-4.630	0.371	-13.173	1.00	0.00
ATOM	1489	OD1	ASN	94	-5.106	-0.749	-13.106	1.00	0.00
ATOM	1490	ND2	ASN	94	-5.387	1.453	-13.205	1.00	0.00
ATOM	1491	HD21	ASN	94	-6.393	1.363	-13.180	1.00	0.00
ATOM	1492	HD22	ASN	94	-4.957	2.365	-13.255	1.00	0.00
ATOM	1493	C	ASN	94	-0.907	-0.437	-13.258	1.00	0.00
ATOM	1494	O	ASN	94	-0.520	-0.980	-14.285	1.00	0.00
ATOM	1495	N	VAL	95	-0.125	0.336	-12.502	1.00	0.00
ATOM	1496	H	VAL	95	-0.521	0.815	-11.706	1.00	0.00
ATOM	1497	CA	VAL	95	1.268	0.576	-12.825	1.00	0.00
ATOM	1498	HA	VAL	95	1.529	0.211	-13.819	1.00	0.00
ATOM	1499	CB	VAL	95	1.557	2.077	-12.763	1.00	0.00

ATOM	1500	HB	VAL	95	1.422	2.417	-11.736	1.00	0.00
ATOM	1501	CG1	VAL	95	2.984	2.379	-13.216	1.00	0.00
ATOM	1502	HG11	VAL	95	3.119	2.039	-14.243	1.00	0.00
ATOM	1503	HG12	VAL	95	3.163	3.452	-13.162	1.00	0.00
ATOM	1504	HG13	VAL	95	3.689	1.860	-12.567	1.00	0.00
ATOM	1505	CG2	VAL	95	0.555	2.855	-13.628	1.00	0.00
ATOM	1506	HG21	VAL	95	-0.457	2.674	-13.264	1.00	0.00
ATOM	1507	HG22	VAL	95	0.776	3.920	-13.572	1.00	0.00
ATOM	1508	HG23	VAL	95	0.633	2.522	-14.663	1.00	0.00
ATOM	1509	C	VAL	95	2.165	-0.215	-11.885	1.00	0.00
ATOM	1510	O	VAL	95	3.372	-0.281	-12.067	1.00	0.00
ATOM	1511	N	CYX	96	1.560	-0.874	-10.901	1.00	0.00
ATOM	1512	H	CYX	96	0.563	-0.762	-10.789	1.00	0.00
ATOM	1513	CA	CYX	96	2.269	-1.666	-9.936	1.00	0.00
ATOM	1514	HA	CYX	96	3.308	-1.762	-10.251	1.00	0.00
ATOM	1515	CB	CYX	96	2.237	-1.041	-8.541	1.00	0.00
ATOM	1516	HB2	CYX	96	1.234	-1.133	-8.124	1.00	0.00
ATOM	1517	HB3	CYX	96	2.947	-1.557	-7.895	1.00	0.00
ATOM	1518	SG	CYX	96	2.658	0.694	-8.506	1.00	0.00
ATOM	1519	C	CYX	96	1.689	-3.042	-9.956	1.00	0.00
ATOM	1520	O	CYX	96	1.563	-3.576	-8.880	1.00	0.00
ATOM	1521	N	ASN	97	1.312	-3.565	-11.134	1.00	0.00

ATOM 1522 H ASN 97 1.441 -2.999 -11.960 1.00 0.00

ATOM 1523 CA ASN 97 0.735 -4.883 -11.289 1.00 0.00

ATOM 1524 HA ASN 97 -0.352 -4.808 -11.314 1.00 0.00

ATOM 1525 CB ASN 97 1.209 -5.531 -12.590 1.00 0.00

ATOM 1526 HB2 ASN 97 2.191 -5.150 -12.870 1.00 0.00

ATOM 1527 HB3 ASN 97 1.271 -6.607 -12.425 1.00 0.00

ATOM 1528 CG ASN 97 0.253 -5.273 -13.722 1.00 0.00

ATOM 1529 OD1 ASN 97 -0.478 -6.143 -14.165 1.00 0.00

ATOM 1530 ND2 ASN 97 0.243 -4.037 -14.171 1.00 0.00

ATOM 1531 HD21 ASN 97 -0.373 -3.784 -14.931 1.00 0.00

ATOM 1532 HD22 ASN 97 0.851 -3.347 -13.754 1.00 0.00

ATOM 1533 C ASN 97 1.131 -5.802 -10.135 1.00 0.00

ATOM 1534 O ASN 97 2.234 -6.349 -10.129 1.00 0.00

ATOM 1535 N MET 98 0.262 -5.881 -9.130 1.00 0.00

ATOM 1536 H MET 98 -0.613 -5.379 -9.182 1.00 0.00

ATOM 1537 CA MET 98 0.562 -6.611 -7.919 1.00 0.00

ATOM 1538 HA MET 98 1.410 -7.272 -8.101 1.00 0.00

ATOM 1539 CB MET 98 0.911 -5.676 -6.763 1.00 0.00

ATOM 1540 HB2 MET 98 1.250 -6.276 -5.918 1.00 0.00

ATOM 1541 HB3 MET 98 1.713 -5.010 -7.081 1.00 0.00

ATOM 1542 CG MET 98 -0.300 -4.849 -6.345 1.00 0.00

ATOM 1543 HG2 MET 98 -1.164 -5.198 -6.910 1.00 0.00

ATOM	1544	HG3	MET	98	-0.474	-5.010	-5.281	1.00	0.00
ATOM	1545	SD	MET	98	-0.067	-3.104	-6.645	1.00	0.00
ATOM	1546	CE	MET	98	-1.400	-2.409	-5.711	1.00	0.00
ATOM	1547	HE1	MET	98	-1.292	-2.686	-4.662	1.00	0.00
ATOM	1548	HE2	MET	98	-1.382	-1.323	-5.804	1.00	0.00
ATOM	1549	HE3	MET	98	-2.348	-2.790	-6.091	1.00	0.00
ATOM	1550	C	MET	98	-0.613	-7.492	-7.576	1.00	0.00
ATOM	1551	O	MET	98	-0.684	-8.060	-6.496	1.00	0.00
ATOM	1552	N	LYS	99	-1.597	-7.572	-8.480	1.00	0.00
ATOM	1553	H	LYS	99	-1.550	-6.963	-9.285	1.00	0.00
ATOM	1554	CA	LYS	99	-2.786	-8.356	-8.288	1.00	0.00
ATOM	1555	HA	LYS	99	-2.586	-9.182	-7.605	1.00	0.00
ATOM	1556	CB	LYS	99	-3.894	-7.479	-7.686	1.00	0.00
ATOM	1557	HB2	LYS	99	-3.824	-7.530	-6.600	1.00	0.00
ATOM	1558	HB3	LYS	99	-3.737	-6.451	-8.012	1.00	0.00
ATOM	1559	CG	LYS	99	-5.286	-7.950	-8.131	1.00	0.00
ATOM	1560	HG2	LYS	99	-5.221	-8.984	-8.471	1.00	0.00
ATOM	1561	HG3	LYS	99	-5.970	-7.889	-7.285	1.00	0.00
ATOM	1562	CD	LYS	99	-5.809	-7.063	-9.280	1.00	0.00
ATOM	1563	HD2	LYS	99	-6.526	-6.351	-8.872	1.00	0.00
ATOM	1564	HD3	LYS	99	-4.968	-6.523	-9.714	1.00	0.00
ATOM	1565	CE	LYS	99	-6.492	-7.912	-10.371	1.00	0.00

ATOM	1566	HE2	LYS	99	-6.329	-8.966	-10.144	1.00	0.00
ATOM	1567	HE3	LYS	99	-7.561	-7.699	-10.359	1.00	0.00
ATOM	1568	NZ	LYS	99	-5.944	-7.606	-11.710	1.00	0.00
ATOM	1569	HZ1	LYS	99	-4.954	-7.804	-11.722	1.00	0.00
ATOM	1570	HZ2	LYS	99	-6.409	-8.176	-12.402	1.00	0.00
ATOM	1571	HZ3	LYS	99	-6.095	-6.630	-11.921	1.00	0.00
ATOM	1572	C	LYS	99	-3.122	-9.047	-9.590	1.00	0.00
ATOM	1573	O	LYS	99	-3.811	-8.514	-10.436	1.00	0.00
ATOM	1574	N	GLN	100	-2.617	-10.239	-9.799	1.00	0.00
ATOM	1575	H	GLN	100	-1.974	-10.644	-9.134	1.00	0.00
ATOM	1576	CA	GLN	100	-2.913	-10.980	-11.003	1.00	0.00
ATOM	1577	HA	GLN	100	-2.845	-10.327	-11.873	1.00	0.00
ATOM	1578	CB	GLN	100	-1.905	-12.123	-11.168	1.00	0.00
ATOM	1579	HB2	GLN	100	-0.996	-11.880	-10.618	1.00	0.00
ATOM	1580	HB3	GLN	100	-2.337	-13.041	-10.768	1.00	0.00
ATOM	1581	CG	GLN	100	-1.567	-12.321	-12.650	1.00	0.00
ATOM	1582	HG2	GLN	100	-1.434	-11.329	-13.081	1.00	0.00
ATOM	1583	HG3	GLN	100	-0.645	-12.892	-12.761	1.00	0.00
ATOM	1584	CD	GLN	100	-2.677	-13.037	-13.410	1.00	0.00
ATOM	1585	OE1	GLN	100	-3.737	-13.346	-12.904	1.00	0.00
ATOM	1586	NE2	GLN	100	-2.413	-13.355	-14.655	1.00	0.00
ATOM	1587	HE21	GLN	100	-3.110	-13.831	-15.210	1.00	0.00

```
ATOM   1588 HE22 GLN   100      -1.514 -13.123 -15.053  1.00  0.00
ATOM   1589  C   GLN   100      -4.337 -11.511 -10.965  1.00  0.00
ATOM   1590  O   GLN   100      -5.098 -11.300 -11.902  1.00  0.00
ATOM   1591  N   ILE   101      -4.688 -12.154  -9.851  1.00  0.00
ATOM   1592  H   ILE   101      -3.990 -12.343  -9.146  1.00  0.00
ATOM   1593  CA  ILE   101      -6.005 -12.704  -9.671  1.00  0.00
ATOM   1594  HA  ILE   101      -6.314 -13.317 -10.518  1.00  0.00
ATOM   1595  CB  ILE   101      -6.083 -13.538  -8.379  1.00  0.00
ATOM   1596  HB  ILE   101      -5.657 -14.523  -8.569  1.00  0.00
ATOM   1597  CG2 ILE   101      -5.314 -12.861  -7.255  1.00  0.00
ATOM   1598 HG21 ILE   101      -5.740 -11.876  -7.065  1.00  0.00
ATOM   1599 HG22 ILE   101      -5.382 -13.467  -6.352  1.00  0.00
ATOM   1600 HG23 ILE   101      -4.268 -12.755  -7.543  1.00  0.00
ATOM   1601  CG1 ILE   101      -7.538 -13.813  -7.965  1.00  0.00
ATOM   1602 HG12 ILE   101      -7.557 -14.673  -7.295  1.00  0.00
ATOM   1603 HG13 ILE   101      -7.927 -12.939  -7.444  1.00  0.00
ATOM   1604  CD1 ILE   101      -8.407 -14.104  -9.195  1.00  0.00
ATOM   1605 HD11 ILE   101      -8.018 -14.979  -9.716  1.00  0.00
ATOM   1606 HD12 ILE   101      -9.432 -14.296  -8.879  1.00  0.00
ATOM   1607 HD13 ILE   101      -8.389 -13.245  -9.865  1.00  0.00
ATOM   1608  C   ILE   101      -7.036 -11.573  -9.734  1.00  0.00
ATOM   1609  O   ILE   101      -7.560 -11.239 -10.791  1.00  0.00
```

ATOM	1610	N	GLY	102	-7.335	-10.958	-8.585	1.00	0.00
ATOM	1611	H	GLY	102	-6.842	-11.224	-7.744	1.00	0.00
ATOM	1612	CA	GLY	102	-8.333	-9.929	-8.493	1.00	0.00
ATOM	1613	HA2	GLY	102	-7.866	-8.967	-8.704	1.00	0.00
ATOM	1614	HA3	GLY	102	-9.109	-10.126	-9.233	1.00	0.00
ATOM	1615	C	GLY	102	-8.961	-9.879	-7.127	1.00	0.00
ATOM	1616	O	GLY	102	-10.059	-10.345	-6.892	1.00	0.00
ATOM	1617	N	THR	103	-8.248	-9.287	-6.189	1.00	0.00
ATOM	1618	H	THR	103	-7.332	-8.940	-6.434	1.00	0.00
ATOM	1619	CA	THR	103	-8.681	-9.176	-4.801	1.00	0.00
ATOM	1620	HA	THR	103	-9.239	-10.088	-4.587	1.00	0.00
ATOM	1621	CB	THR	103	-7.441	-9.139	-3.904	1.00	0.00
ATOM	1622	HB	THR	103	-7.457	-8.210	-3.335	1.00	0.00
ATOM	1623	CG2	THR	103	-7.404	-10.324	-2.942	1.00	0.00
ATOM	1624	HG21	THR	103	-7.388	-11.254	-3.511	1.00	0.00
ATOM	1625	HG22	THR	103	-6.509	-10.262	-2.323	1.00	0.00
ATOM	1626	HG23	THR	103	-8.288	-10.304	-2.305	1.00	0.00
ATOM	1627	OG1	THR	103	-6.289	-9.316	-4.728	1.00	0.00
ATOM	1628	HG1	THR	103	-5.499	-9.296	-4.183	1.00	0.00
ATOM	1629	C	THR	103	-9.560	-7.952	-4.635	1.00	0.00
ATOM	1630	O	THR	103	-10.678	-8.049	-4.131	1.00	0.00
ATOM	1631	N	CYX	104	-9.072	-6.793	-5.098	1.00	0.00

```
ATOM   1632  H   CYX   104      -8.113  -6.756  -5.413  1.00  0.00
ATOM   1633  CA  CYX   104      -9.834  -5.560  -5.069  1.00  0.00
ATOM   1634  HA  CYX   104     -10.873  -5.772  -4.817  1.00  0.00
ATOM   1635  CB  CYX   104      -9.260  -4.605  -4.018  1.00  0.00
ATOM   1636  HB2 CYX   104      -9.983  -3.817  -3.809  1.00  0.00
ATOM   1637  HB3 CYX   104      -9.050  -5.157  -3.102  1.00  0.00
ATOM   1638  SG  CYX   104      -7.685  -3.758  -4.434  1.00  0.00
ATOM   1639  C   CYX   104      -9.870  -4.958  -6.466  1.00  0.00
ATOM   1640  O   CYX   104      -9.259  -3.931  -6.735  1.00  0.00
ATOM   1641  N   PRO   105     -10.536  -5.635  -7.403  1.00  0.00
ATOM   1642  CD  PRO   105     -11.269  -6.875  -7.206  1.00  0.00
ATOM   1643  HD2 PRO   105     -12.120  -6.660  -6.559  1.00  0.00
ATOM   1644  HD3 PRO   105     -10.604  -7.581  -6.708  1.00  0.00
ATOM   1645  CG  PRO   105     -11.693  -7.354  -8.592  1.00  0.00
ATOM   1646  HG2 PRO   105     -12.782  -7.370  -8.646  1.00  0.00
ATOM   1647  HG3 PRO   105     -11.308  -8.361  -8.755  1.00  0.00
ATOM   1648  CB  PRO   105     -11.097  -6.371  -9.602  1.00  0.00
ATOM   1649  HB2 PRO   105     -11.822  -6.061 -10.354  1.00  0.00
ATOM   1650  HB3 PRO   105     -10.231  -6.813 -10.094  1.00  0.00
ATOM   1651  CA  PRO   105     -10.665  -5.166  -8.769  1.00  0.00
ATOM   1652  HA  PRO   105      -9.673  -4.886  -9.124  1.00  0.00
ATOM   1653  C   PRO   105     -11.602  -3.973  -8.888  1.00  0.00
```

ATOM 1654 O PRO 105 -12.550 -3.976 -9.663 1.00 0.00

ATOM 1655 N PHE 106 -11.353 -2.948 -8.083 1.00 0.00

ATOM 1656 H PHE 106 -10.553 -2.997 -7.469 1.00 0.00

ATOM 1657 CA PHE 106 -12.159 -1.761 -8.057 1.00 0.00

ATOM 1658 HA PHE 106 -13.195 -1.984 -8.313 1.00 0.00

ATOM 1659 CB PHE 106 -12.117 -1.202 -6.639 1.00 0.00

ATOM 1660 HB2 PHE 106 -13.049 -0.699 -6.382 1.00 0.00

ATOM 1661 HB3 PHE 106 -11.949 -2.029 -5.949 1.00 0.00

ATOM 1662 CG PHE 106 -11.002 -0.201 -6.397 1.00 0.00

ATOM 1663 CD1 PHE 106 -9.678 -0.634 -6.234 1.00 0.00

ATOM 1664 HD1 PHE 106 -9.456 -1.701 -6.279 1.00 0.00

ATOM 1665 CE1 PHE 106 -8.649 0.285 -6.016 1.00 0.00

ATOM 1666 HE1 PHE 106 -7.617 -0.053 -5.917 1.00 0.00

ATOM 1667 CZ PHE 106 -8.950 1.643 -5.926 1.00 0.00

ATOM 1668 HZ PHE 106 -8.163 2.358 -5.688 1.00 0.00

ATOM 1669 CE2 PHE 106 -10.259 2.089 -6.140 1.00 0.00

ATOM 1670 HE2 PHE 106 -10.478 3.156 -6.123 1.00 0.00

ATOM 1671 CD2 PHE 106 -11.283 1.171 -6.375 1.00 0.00

ATOM 1672 HD2 PHE 106 -12.302 1.521 -6.542 1.00 0.00

ATOM 1673 C PHE 106 -11.623 -0.728 -9.001 1.00 0.00

ATOM 1674 O PHE 106 -10.426 -0.761 -9.277 1.00 0.00

ATOM 1675 N ILE 107 -12.484 0.197 -9.428 1.00 0.00

ATOM	1676	H	ILE	107	-13.444	0.146	-9.119	1.00	0.00
ATOM	1677	CA	ILE	107	-12.120	1.236	-10.374	1.00	0.00
ATOM	1678	HA	ILE	107	-11.512	2.014	-9.913	1.00	0.00
ATOM	1679	CB	ILE	107	-11.375	0.659	-11.598	1.00	0.00
ATOM	1680	HB	ILE	107	-11.689	1.200	-12.491	1.00	0.00
ATOM	1681	CG2	ILE	107	-9.855	0.801	-11.421	1.00	0.00
ATOM	1682	HG21	ILE	107	-9.541	0.259	-10.529	1.00	0.00
ATOM	1683	HG22	ILE	107	-9.347	0.389	-12.293	1.00	0.00
ATOM	1684	HG23	ILE	107	-9.598	1.855	-11.316	1.00	0.00
ATOM	1685	CG1	ILE	107	-11.775	-0.808	-11.873	1.00	0.00
ATOM	1686	HG12	ILE	107	-11.064	-1.462	-11.367	1.00	0.00
ATOM	1687	HG13	ILE	107	-12.773	-0.976	-11.469	1.00	0.00
ATOM	1688	CD1	ILE	107	-11.773	-1.112	-13.351	1.00	0.00
ATOM	1689	HD11	ILE	107	-10.775	-0.945	-13.756	1.00	0.00
ATOM	1690	HD12	ILE	107	-12.059	-2.152	-13.509	1.00	0.00
ATOM	1691	HD13	ILE	107	-12.484	-0.460	-13.858	1.00	0.00
ATOM	1692	C	ILE	107	-13.352	1.997	-10.812	1.00	0.00
ATOM	1693	O	ILE	107	-13.269	3.210	-10.947	1.00	0.00
ATOM	1694	N	ALA	108	-14.467	1.288	-11.024	1.00	0.00
ATOM	1695	H	ALA	108	-14.421	0.280	-10.973	1.00	0.00
ATOM	1696	CA	ALA	108	-15.722	1.898	-11.399	1.00	0.00
ATOM	1697	HA	ALA	108	-15.561	2.617	-12.202	1.00	0.00

ATOM 1698 CB ALA 108 -16.677 0.815 -11.898 1.00 0.00

ATOM 1699 HB1 ALA 108 -16.856 0.092 -11.102 1.00 0.00

ATOM 1700 HB2 ALA 108 -17.622 1.271 -12.193 1.00 0.00

ATOM 1701 HB3 ALA 108 -16.236 0.308 -12.756 1.00 0.00

ATOM 1702 C ALA 108 -16.305 2.680 -10.223 1.00 0.00

ATOM 1703 O ALA 108 -17.344 2.321 -9.682 1.00 0.00

ATOM 1704 N ILE 109 -15.616 3.734 -9.815 1.00 0.00

ATOM 1705 H ILE 109 -14.730 3.902 -10.269 1.00 0.00

ATOM 1706 CA ILE 109 -15.978 4.565 -8.690 1.00 0.00

ATOM 1707 HA ILE 109 -17.025 4.444 -8.410 1.00 0.00

ATOM 1708 CB ILE 109 -15.085 4.281 -7.470 1.00 0.00

ATOM 1709 HB ILE 109 -14.951 5.204 -6.907 1.00 0.00

ATOM 1710 CG2 ILE 109 -15.729 3.219 -6.565 1.00 0.00

ATOM 1711 HG21 ILE 109 -15.863 2.295 -7.127 1.00 0.00

ATOM 1712 HG22 ILE 109 -15.083 3.031 -5.708 1.00 0.00

ATOM 1713 HG23 ILE 109 -16.698 3.576 -6.218 1.00 0.00

ATOM 1714 CG1 ILE 109 -13.660 3.871 -7.869 1.00 0.00

ATOM 1715 HG12 ILE 109 -13.127 3.537 -6.979 1.00 0.00

ATOM 1716 HG13 ILE 109 -13.717 3.052 -8.586 1.00 0.00

ATOM 1717 CD1 ILE 109 -12.916 5.043 -8.496 1.00 0.00

ATOM 1718 HD11 ILE 109 -12.858 5.862 -7.780 1.00 0.00

ATOM 1719 HD12 ILE 109 -11.909 4.729 -8.771 1.00 0.00

ATOM 1720 HD13 ILE 109 -13.448 5.377 -9.387 1.00 0.00

ATOM 1721 C ILE 109 -15.982 6.040 -9.121 1.00 0.00

ATOM 1722 O ILE 109 -17.009 6.700 -8.935 1.00 0.00

ATOM 1723 OXT ILE 109 -14.969 6.535 -9.649 1.00 0.00

TER

ATOM 1724 S1 LIG 110 20.108 -12.667 -5.224 1.00 0.00

ATOM 1725 S2 LIG 110 17.422 -17.393 0.322 1.00 0.00

ATOM 1726 O1 LIG 110 14.533 -13.304 -1.192 1.00 0.00

ATOM 1727 H5 LIG 110 13.994 -13.791 -0.534 1.00 0.00

ATOM 1728 O2 LIG 110 20.750 -11.247 -4.842 1.00 0.00

ATOM 1729 H6 LIG 110 19.986 -10.606 -4.959 1.00 0.00

ATOM 1730 O3 LIG 110 16.250 -18.435 -0.028 1.00 0.00

ATOM 1731 H7 LIG 110 15.415 -17.967 0.264 1.00 0.00

ATOM 1732 O4 LIG 110 21.120 -13.669 -4.958 1.00 0.00

ATOM 1733 O5 LIG 110 19.458 -12.586 -6.524 1.00 0.00

ATOM 1734 O6 LIG 110 18.656 -17.964 -0.179 1.00 0.00

ATOM 1735 O7 LIG 110 17.298 -16.961 1.706 1.00 0.00

ATOM 1736 O8 LIG 110 15.821 -5.430 -8.466 1.00 0.00

ATOM 1737 O9 LIG 110 17.887 -4.777 -8.339 1.00 0.00

ATOM 1738 N1 LIG 110 15.547 -11.505 -2.860 1.00 0.00

ATOM 1739 H3 LIG 110 14.752 -11.755 -2.294 1.00 0.00

ATOM 1740 H4 LIG 110 15.539 -10.682 -3.442 1.00 0.00

```
ATOM   1741  N2  LIG   110      17.403 -10.803  -4.543  1.00  0.00
ATOM   1742  N3  LIG   110      14.657 -15.445   0.177  1.00  0.00
ATOM   1743  N4  LIG   110      18.332 -10.165  -5.117  1.00  0.00
ATOM   1744  N5  LIG   110      14.433 -16.583   0.673  1.00  0.00
ATOM   1745  N6  LIG   110      16.984  -5.568  -8.105  1.00  0.00
ATOM   1746  C1  LIG   110      16.684 -13.559  -2.212  1.00  0.00
ATOM   1747  C2  LIG   110      17.849 -14.367  -2.339  1.00  0.00
ATOM   1748  C3  LIG   110      16.594 -12.339  -2.982  1.00  0.00
ATOM   1749  C4  LIG   110      18.792 -12.851  -3.979  1.00  0.00
ATOM   1750  C5  LIG   110      18.881 -13.994  -3.236  1.00  0.00
ATOM   1751  H1  LIG   110      19.743 -14.639  -3.352  1.00  0.00
ATOM   1752  C6  LIG   110      15.671 -14.016  -1.330  1.00  0.00
ATOM   1753  C7  LIG   110      16.997 -15.969  -0.721  1.00  0.00
ATOM   1754  C8  LIG   110      17.985 -15.550  -1.572  1.00  0.00
ATOM   1755  H2  LIG   110      18.894 -16.131  -1.651  1.00  0.00
ATOM   1756  C9  LIG   110      17.661 -11.978  -3.871  1.00  0.00
ATOM   1757  C10 LIG   110      15.790 -15.218  -0.593  1.00  0.00
ATOM   1758  C11 LIG   110      17.933  -9.036  -5.858  1.00  0.00
ATOM   1759  C12 LIG   110      13.291 -16.706   1.488  1.00  0.00
ATOM   1760  C13 LIG   110      16.618  -8.830  -6.308  1.00  0.00
ATOM   1761  H8  LIG   110      15.861  -9.574  -6.101  1.00  0.00
ATOM   1762  C14 LIG   110      18.937  -8.112  -6.179  1.00  0.00
```

```
ATOM   1763  H9  LIG   110      19.953  -8.291  -5.850  1.00  0.00
ATOM   1764  C15 LIG   110      16.308  -7.697  -7.041  1.00  0.00
ATOM   1765  H10 LIG   110      15.307  -7.517  -7.408  1.00  0.00
ATOM   1766  C16 LIG   110      18.631  -6.971  -6.907  1.00  0.00
ATOM   1767  H11 LIG   110      19.386  -6.240  -7.157  1.00  0.00
ATOM   1768  C17 LIG   110      17.317  -6.778  -7.324  1.00  0.00
ATOM   1769  C18 LIG   110      12.587 -15.616   2.026  1.00  0.00
ATOM   1770  H12 LIG   110      12.936 -14.608   1.843  1.00  0.00
ATOM   1771  C19 LIG   110      12.884 -18.016   1.779  1.00  0.00
ATOM   1772  H13 LIG   110      13.449 -18.850   1.380  1.00  0.00
ATOM   1773  C20 LIG   110      11.473 -15.845   2.818  1.00  0.00
ATOM   1774  H14 LIG   110      10.933 -15.007   3.243  1.00  0.00
ATOM   1775  C21 LIG   110      11.759 -18.235   2.565  1.00  0.00
ATOM   1776  H15 LIG   110      11.441 -19.248   2.779  1.00  0.00
ATOM   1777  C22 LIG   110      11.050 -17.152   3.084  1.00  0.00
ATOM   1778  H16 LIG   110      10.180 -17.322   3.707  1.00  0.00
TER
ATOM   1779  S1  LIG   111      10.851  -7.304  22.174  1.00  0.00
ATOM   1780  S2  LIG   111      10.239  -7.724  14.443  1.00  0.00
ATOM   1781  O1  LIG   111      13.704 -10.763  16.916  1.00  0.00
ATOM   1782  H5  LIG   111      13.751 -11.011  15.968  1.00  0.00
ATOM   1783  O2  LIG   111      12.056  -6.490  22.851  1.00  0.00
```

ATOM	1784	H6	LIG	111	12.738	-7.201	23.046	1.00	0.00
ATOM	1785	O3	LIG	111	9.505	-8.989	13.777	1.00	0.00
ATOM	1786	H7	LIG	111	10.256	-9.605	13.537	1.00	0.00
ATOM	1787	O4	LIG	111	9.872	-6.326	21.746	1.00	0.00
ATOM	1788	O5	LIG	111	10.469	-8.425	23.021	1.00	0.00
ATOM	1789	O6	LIG	111	9.198	-6.835	14.916	1.00	0.00
ATOM	1790	O7	LIG	111	11.294	-7.247	13.562	1.00	0.00
ATOM	1791	O8	LIG	111	16.498	-12.163	27.250	1.00	0.00
ATOM	1792	O9	LIG	111	16.564	-10.195	28.165	1.00	0.00
ATOM	1793	N1	LIG	111	14.150	-10.430	19.511	1.00	0.00
ATOM	1794	H3	LIG	111	14.420	-10.935	18.682	1.00	0.00
ATOM	1795	H4	LIG	111	14.565	-10.634	20.407	1.00	0.00
ATOM	1796	N2	LIG	111	13.449	-9.393	21.792	1.00	0.00
ATOM	1797	N3	LIG	111	12.528	-10.201	14.730	1.00	0.00
ATOM	1798	N4	LIG	111	13.467	-8.748	22.881	1.00	0.00
ATOM	1799	N5	LIG	111	11.870	-10.261	13.656	1.00	0.00
ATOM	1800	N6	LIG	111	16.250	-10.963	27.266	1.00	0.00
ATOM	1801	C1	LIG	111	12.439	-9.236	18.257	1.00	0.00
ATOM	1802	C2	LIG	111	11.395	-8.268	18.288	1.00	0.00
ATOM	1803	C3	LIG	111	13.118	-9.570	19.488	1.00	0.00
ATOM	1804	C4	LIG	111	11.637	-8.003	20.687	1.00	0.00
ATOM	1805	C5	LIG	111	11.009	-7.677	19.517	1.00	0.00

ATOM	1806	H1	LIG	111	10.187	-6.973	19.533	1.00	0.00
ATOM	1807	C6	LIG	111	12.742	-9.821	17.000	1.00	0.00
ATOM	1808	C7	LIG	111	11.047	-8.464	15.891	1.00	0.00
ATOM	1809	C8	LIG	111	10.733	-7.892	17.094	1.00	0.00
ATOM	1810	H2	LIG	111	9.970	-7.126	17.131	1.00	0.00
ATOM	1811	C9	LIG	111	12.717	-8.944	20.715	1.00	0.00
ATOM	1812	C10	LIG	111	12.054	-9.473	15.814	1.00	0.00
ATOM	1813	C11	LIG	111	14.165	-9.357	23.941	1.00	0.00
ATOM	1814	C12	LIG	111	12.455	-10.973	12.592	1.00	0.00
ATOM	1815	C13	LIG	111	14.467	-10.729	23.984	1.00	0.00
ATOM	1816	H8	LIG	111	14.140	-11.372	23.178	1.00	0.00
ATOM	1817	C14	LIG	111	14.523	-8.526	25.013	1.00	0.00
ATOM	1818	H9	LIG	111	14.263	-7.476	24.983	1.00	0.00
ATOM	1819	C15	LIG	111	15.150	-11.253	25.069	1.00	0.00
ATOM	1820	H10	LIG	111	15.388	-12.305	25.132	1.00	0.00
ATOM	1821	C16	LIG	111	15.213	-9.045	26.099	1.00	0.00
ATOM	1822	H11	LIG	111	15.508	-8.421	26.931	1.00	0.00
ATOM	1823	C17	LIG	111	15.521	-10.402	26.109	1.00	0.00
ATOM	1824	C18	LIG	111	13.811	-11.337	12.540	1.00	0.00
ATOM	1825	H12	LIG	111	14.477	-11.043	13.340	1.00	0.00
ATOM	1826	C19	LIG	111	11.602	-11.293	11.526	1.00	0.00
ATOM	1827	H13	LIG	111	10.564	-10.984	11.564	1.00	0.00

ATOM 1828 C20 LIG 111 14.289 -12.040 11.445 1.00 0.00

ATOM 1829 H14 LIG 111 15.337 -12.314 11.397 1.00 0.00

ATOM 1830 C21 LIG 111 12.088 -12.007 10.437 1.00 0.00

ATOM 1831 H15 LIG 111 11.425 -12.261 9.620 1.00 0.00

ATOM 1832 C22 LIG 111 13.430 -12.384 10.395 1.00 0.00

ATOM 1833 H16 LIG 111 13.814 -12.929 9.541 1.00 0.00

TER

ATOM 1834 S1 LIG 112 -22.156 16.776 -21.134 1.00 0.00

ATOM 1835 S2 LIG 112 -24.732 17.758 -13.874 1.00 0.00

ATOM 1836 O1 LIG 112 -25.685 21.444 -17.461 1.00 0.00

ATOM 1837 H5 LIG 112 -26.137 21.737 -16.641 1.00 0.00

ATOM 1838 O2 LIG 112 -20.839 17.539 -21.640 1.00 0.00

ATOM 1839 H6 LIG 112 -21.204 18.352 -22.104 1.00 0.00

ATOM 1840 O3 LIG 112 -26.296 17.567 -13.558 1.00 0.00

ATOM 1841 H7 LIG 112 -26.664 18.497 -13.588 1.00 0.00

ATOM 1842 O4 LIG 112 -21.709 15.649 -20.341 1.00 0.00

ATOM 1843 O5 LIG 112 -23.082 16.591 -22.243 1.00 0.00

ATOM 1844 O6 LIG 112 -24.155 16.432 -13.957 1.00 0.00

ATOM 1845 O7 LIG 112 -24.173 18.770 -12.991 1.00 0.00

ATOM 1846 O8 LIG 112 -23.353 22.562 -27.942 1.00 0.00

ATOM 1847 O9 LIG 112 -21.312 21.874 -28.218 1.00 0.00

ATOM 1848 N1 LIG 112 -24.565 21.284 -19.861 1.00 0.00

ATOM	1849	H3	LIG	112	-25.146	21.839	-19.253	1.00	0.00
ATOM	1850	H4	LIG	112	-24.379	21.567	-20.811	1.00	0.00
ATOM	1851	N2	LIG	112	-23.274	19.898	-21.645	1.00	0.00
ATOM	1852	N3	LIG	112	-26.133	20.582	-15.109	1.00	0.00
ATOM	1853	N4	LIG	112	-22.403	19.522	-22.483	1.00	0.00
ATOM	1854	N5	LIG	112	-26.685	20.181	-14.048	1.00	0.00
ATOM	1855	N6	LIG	112	-22.345	21.972	-27.570	1.00	0.00
ATOM	1856	C1	LIG	112	-24.381	19.560	-18.152	1.00	0.00
ATOM	1857	C2	LIG	112	-23.847	18.290	-17.792	1.00	0.00
ATOM	1858	C3	LIG	112	-24.139	20.067	-19.483	1.00	0.00
ATOM	1859	C4	LIG	112	-22.908	17.993	-20.008	1.00	0.00
ATOM	1860	C5	LIG	112	-23.125	17.526	-18.742	1.00	0.00
ATOM	1861	H1	LIG	112	-22.759	16.544	-18.468	1.00	0.00
ATOM	1862	C6	LIG	112	-25.132	20.249	-17.166	1.00	0.00
ATOM	1863	C7	LIG	112	-24.759	18.465	-15.546	1.00	0.00
ATOM	1864	C8	LIG	112	-24.036	17.780	-16.485	1.00	0.00
ATOM	1865	H2	LIG	112	-23.589	16.831	-16.217	1.00	0.00
ATOM	1866	C9	LIG	112	-23.390	19.279	-20.420	1.00	0.00
ATOM	1867	C10	LIG	112	-25.353	19.722	-15.871	1.00	0.00
ATOM	1868	C11	LIG	112	-22.447	20.160	-23.738	1.00	0.00
ATOM	1869	C12	LIG	112	-27.408	21.137	-13.308	1.00	0.00
ATOM	1870	C13	LIG	112	-23.578	20.840	-24.223	1.00	0.00

ATOM 1871 H8 LIG 112 -24.478 20.873 -23.624 1.00 0.00

ATOM 1872 C14 LIG 112 -21.299 20.052 -24.535 1.00 0.00

ATOM 1873 H9 LIG 112 -20.442 19.504 -24.164 1.00 0.00

ATOM 1874 C15 LIG 112 -23.544 21.433 -25.474 1.00 0.00

ATOM 1875 H10 LIG 112 -24.401 21.955 -25.875 1.00 0.00

ATOM 1876 C16 LIG 112 -21.257 20.650 -25.786 1.00 0.00

ATOM 1877 H11 LIG 112 -20.378 20.589 -26.413 1.00 0.00

ATOM 1878 C17 LIG 112 -22.380 21.338 -26.236 1.00 0.00

ATOM 1879 C18 LIG 112 -27.295 22.525 -13.494 1.00 0.00

ATOM 1880 H12 LIG 112 -26.599 22.914 -14.225 1.00 0.00

ATOM 1881 C19 LIG 112 -28.259 20.629 -12.316 1.00 0.00

ATOM 1882 H13 LIG 112 -28.318 19.558 -12.164 1.00 0.00

ATOM 1883 C20 LIG 112 -28.053 23.383 -12.713 1.00 0.00

ATOM 1884 H14 LIG 112 -27.962 24.455 -12.847 1.00 0.00

ATOM 1885 C21 LIG 112 -29.022 21.498 -11.545 1.00 0.00

ATOM 1886 H15 LIG 112 -29.686 21.102 -10.786 1.00 0.00

ATOM 1887 C22 LIG 112 -28.923 22.874 -11.743 1.00 0.00

ATOM 1888 H16 LIG 112 -29.509 23.553 -11.134 1.00 0.00

TER

ATOM 1889 S1 LIG 113 -4.325 -24.043 -3.550 1.00 0.00

ATOM 1890 S2 LIG 113 -2.239 -21.438 -10.562 1.00 0.00

ATOM 1891 O1 LIG 113 0.648 -20.176 -6.387 1.00 0.00

```
ATOM   1892  H5  LIG   113       1.111 -19.797  -7.164  1.00  0.00
ATOM   1893  O2  LIG   113      -5.078 -23.075  -2.513  1.00  0.00
ATOM   1894  H6  LIG   113      -4.334 -22.732  -1.932  1.00  0.00
ATOM   1895  O3  LIG   113      -0.984 -22.106 -11.311  1.00  0.00
ATOM   1896  H7  LIG   113      -0.220 -21.498 -11.093  1.00  0.00
ATOM   1897  O4  LIG   113      -5.318 -24.484  -4.508  1.00  0.00
ATOM   1898  O5  LIG   113      -3.503 -25.003  -2.827  1.00  0.00
ATOM   1899  O6  LIG   113      -3.363 -22.333 -10.745  1.00  0.00
ATOM   1900  O7  LIG   113      -2.331 -20.031 -10.917  1.00  0.00
ATOM   1901  O8  LIG   113       0.048 -22.400   4.160  1.00  0.00
ATOM   1902  O9  LIG   113      -2.042 -22.252   4.725  1.00  0.00
ATOM   1903  N1  LIG   113      -0.199 -20.793  -3.949  1.00  0.00
ATOM   1904  H3  LIG   113       0.518 -20.331  -4.485  1.00  0.00
ATOM   1905  H4  LIG   113      -0.146 -20.855  -2.944  1.00  0.00
ATOM   1906  N2  LIG   113      -1.829 -22.113  -2.409  1.00  0.00
ATOM   1907  N3  LIG   113       0.429 -20.153  -8.922  1.00  0.00
ATOM   1908  N4  LIG   113      -2.696 -22.398  -1.533  1.00  0.00
ATOM   1909  N5  LIG   113       0.632 -20.292 -10.159  1.00  0.00
ATOM   1910  N6  LIG   113      -1.146 -22.335   3.896  1.00  0.00
ATOM   1911  C1  LIG   113      -1.323 -21.477  -5.998  1.00  0.00
ATOM   1912  C2  LIG   113      -2.422 -22.170  -6.581  1.00  0.00
ATOM   1913  C3  LIG   113      -1.180 -21.479  -4.560  1.00  0.00
```

```
ATOM   1914  C4  LIG   113      -3.191 -22.900  -4.400  1.00  0.00
ATOM   1915  C5  LIG   113      -3.332 -22.881  -5.759  1.00  0.00
ATOM   1916  H1  LIG   113      -4.139 -23.437  -6.218  1.00  0.00
ATOM   1917  C6  LIG   113      -0.425 -20.825  -6.883  1.00  0.00
ATOM   1918  C7  LIG   113      -1.735 -21.517  -8.820  1.00  0.00
ATOM   1919  C8  LIG   113      -2.611 -22.158  -7.984  1.00  0.00
ATOM   1920  H2  LIG   113      -3.474 -22.657  -8.406  1.00  0.00
ATOM   1921  C9  LIG   113      -2.126 -22.193  -3.752  1.00  0.00
ATOM   1922  C10 LIG   113      -0.593 -20.846  -8.288  1.00  0.00
ATOM   1923  C11 LIG   113      -2.247 -22.379  -0.198  1.00  0.00
ATOM   1924  C12 LIG   113       1.651 -19.506 -10.729  1.00  0.00
ATOM   1925  C13 LIG   113      -0.892 -22.463   0.165  1.00  0.00
ATOM   1926  H8  LIG   113      -0.139 -22.566  -0.605  1.00  0.00
ATOM   1927  C14 LIG   113      -3.236 -22.314   0.793  1.00  0.00
ATOM   1928  H9  LIG   113      -4.279 -22.275   0.505  1.00  0.00
ATOM   1929  C15 LIG   113      -0.532 -22.447   1.502  1.00  0.00
ATOM   1930  H10 LIG   113       0.501 -22.519   1.811  1.00  0.00
ATOM   1931  C16 LIG   113      -2.881 -22.291   2.135  1.00  0.00
ATOM   1932  H11 LIG   113      -3.626 -22.230   2.915  1.00  0.00
ATOM   1933  C17 LIG   113      -1.532 -22.353   2.470  1.00  0.00
ATOM   1934  C18 LIG   113       2.223 -18.386 -10.103  1.00  0.00
ATOM   1935  H12 LIG   113       1.859 -18.067  -9.135  1.00  0.00
```

ATOM	1936	C19	LIG	113	2.072	-19.883	-12.012	1.00	0.00
ATOM	1937	H13	LIG	113	1.606	-20.733	-12.496	1.00	0.00
ATOM	1938	C20	LIG	113	3.224	-17.678	-10.750	1.00	0.00
ATOM	1939	H14	LIG	113	3.660	-16.807	-10.275	1.00	0.00
ATOM	1940	C21	LIG	113	3.084	-19.173	-12.648	1.00	0.00
ATOM	1941	H15	LIG	113	3.413	-19.473	-13.635	1.00	0.00
ATOM	1942	C22	LIG	113	3.663	-18.072	-12.019	1.00	0.00
ATOM	1943	H16	LIG	113	4.443	-17.510	-12.519	1.00	0.00
TER									
ATOM	1944	S1	LIG	114	21.974	2.179	-23.522	1.00	0.00
ATOM	1945	S2	LIG	114	14.565	1.764	-21.233	1.00	0.00
ATOM	1946	O1	LIG	114	17.738	5.300	-19.044	1.00	0.00
ATOM	1947	H5	LIG	114	16.849	5.453	-18.657	1.00	0.00
ATOM	1948	O2	LIG	114	23.139	1.533	-22.627	1.00	0.00
ATOM	1949	H6	LIG	114	23.480	2.312	-22.091	1.00	0.00
ATOM	1950	O3	LIG	114	13.526	2.894	-21.711	1.00	0.00
ATOM	1951	H7	LIG	114	13.504	3.540	-20.947	1.00	0.00
ATOM	1952	O4	LIG	114	21.342	1.088	-24.236	1.00	0.00
ATOM	1953	O5	LIG	114	22.479	3.346	-24.230	1.00	0.00
ATOM	1954	O6	LIG	114	14.731	0.849	-22.343	1.00	0.00
ATOM	1955	O7	LIG	114	14.195	1.285	-19.910	1.00	0.00
ATOM	1956	O8	LIG	114	28.122	7.952	-20.339	1.00	0.00

ATOM	1957	O9	LIG	114	29.233	6.098	-20.548	1.00	0.00
ATOM	1958	N1	LIG	114	20.336	5.267	-19.582	1.00	0.00
ATOM	1959	H3	LIG	114	19.607	5.705	-19.042	1.00	0.00
ATOM	1960	H4	LIG	114	21.290	5.592	-19.537	1.00	0.00
ATOM	1961	N2	LIG	114	22.313	4.415	-21.043	1.00	0.00
ATOM	1962	N3	LIG	114	15.360	4.429	-19.303	1.00	0.00
ATOM	1963	N4	LIG	114	23.403	3.887	-21.408	1.00	0.00
ATOM	1964	N5	LIG	114	14.122	4.328	-19.516	1.00	0.00
ATOM	1965	N6	LIG	114	28.194	6.744	-20.533	1.00	0.00
ATOM	1966	C1	LIG	114	18.701	3.822	-20.662	1.00	0.00
ATOM	1967	C2	LIG	114	18.468	2.784	-21.608	1.00	0.00
ATOM	1968	C3	LIG	114	20.044	4.331	-20.501	1.00	0.00
ATOM	1969	C4	LIG	114	20.802	2.782	-22.265	1.00	0.00
ATOM	1970	C5	LIG	114	19.534	2.292	-22.402	1.00	0.00
ATOM	1971	H1	LIG	114	19.335	1.530	-23.145	1.00	0.00
ATOM	1972	C6	LIG	114	17.580	4.299	-19.934	1.00	0.00
ATOM	1973	C7	LIG	114	16.104	2.708	-21.047	1.00	0.00
ATOM	1974	C8	LIG	114	17.171	2.238	-21.765	1.00	0.00
ATOM	1975	H2	LIG	114	17.021	1.422	-22.460	1.00	0.00
ATOM	1976	C9	LIG	114	21.107	3.803	-21.306	1.00	0.00
ATOM	1977	C10	LIG	114	16.277	3.779	-20.119	1.00	0.00
ATOM	1978	C11	LIG	114	24.562	4.653	-21.176	1.00	0.00

ATOM 1979 C12 LIG 114 13.265 4.972 -18.603 1.00 0.00

ATOM 1980 C13 LIG 114 24.543 6.041 -20.959 1.00 0.00

ATOM 1981 H8 LIG 114 23.601 6.572 -20.986 1.00 0.00

ATOM 1982 C14 LIG 114 25.783 3.965 -21.213 1.00 0.00

ATOM 1983 H9 LIG 114 25.790 2.900 -21.405 1.00 0.00

ATOM 1984 C15 LIG 114 25.729 6.724 -20.748 1.00 0.00

ATOM 1985 H10 LIG 114 25.745 7.793 -20.588 1.00 0.00

ATOM 1986 C16 LIG 114 26.974 4.643 -20.995 1.00 0.00

ATOM 1987 H11 LIG 114 27.926 4.132 -21.007 1.00 0.00

ATOM 1988 C17 LIG 114 26.929 6.014 -20.760 1.00 0.00

ATOM 1989 C18 LIG 114 13.667 5.435 -17.339 1.00 0.00

ATOM 1990 H12 LIG 114 14.685 5.276 -17.008 1.00 0.00

ATOM 1991 C19 LIG 114 11.931 5.115 -19.010 1.00 0.00

ATOM 1992 H13 LIG 114 11.626 4.732 -19.976 1.00 0.00

ATOM 1993 C20 LIG 114 12.744 6.058 -16.513 1.00 0.00

ATOM 1994 H14 LIG 114 13.049 6.407 -15.533 1.00 0.00

ATOM 1995 C21 LIG 114 11.016 5.751 -18.179 1.00 0.00

ATOM 1996 H15 LIG 114 9.988 5.868 -18.501 1.00 0.00

ATOM 1997 C22 LIG 114 11.420 6.226 -16.932 1.00 0.00

ATOM 1998 H16 LIG 114 10.704 6.710 -16.278 1.00 0.00

TER

ATOM 1999 S1 LIG 115 -11.665 -23.382 16.837 1.00 0.00

ATOM	2000	S2	LIG	115	-11.302	-24.023	9.106	1.00	0.00
ATOM	2001	O1	LIG	115	-12.972	-19.549	11.239	1.00	0.00
ATOM	2002	H5	LIG	115	-12.952	-19.399	10.270	1.00	0.00
ATOM	2003	O2	LIG	115	-13.071	-23.515	17.599	1.00	0.00
ATOM	2004	H6	LIG	115	-13.364	-22.562	17.717	1.00	0.00
ATOM	2005	O3	LIG	115	-10.123	-23.279	8.307	1.00	0.00
ATOM	2006	H7	LIG	115	-10.540	-22.422	8.003	1.00	0.00
ATOM	2007	O4	LIG	115	-11.232	-24.726	16.517	1.00	0.00
ATOM	2008	O5	LIG	115	-10.792	-22.466	17.558	1.00	0.00
ATOM	2009	O6	LIG	115	-10.731	-25.228	9.672	1.00	0.00
ATOM	2010	O7	LIG	115	-12.500	-24.071	8.283	1.00	0.00
ATOM	2011	O8	LIG	115	-14.379	-16.073	21.363	1.00	0.00
ATOM	2012	O9	LIG	115	-15.252	-17.714	22.485	1.00	0.00
ATOM	2013	N1	LIG	115	-13.395	-19.399	13.854	1.00	0.00
ATOM	2014	H3	LIG	115	-13.456	-18.910	12.976	1.00	0.00
ATOM	2015	H4	LIG	115	-13.636	-18.949	14.724	1.00	0.00
ATOM	2016	N2	LIG	115	-13.107	-20.413	16.234	1.00	0.00
ATOM	2017	N3	LIG	115	-12.266	-20.778	9.126	1.00	0.00
ATOM	2018	N4	LIG	115	-13.352	-20.876	17.386	1.00	0.00
ATOM	2019	N5	LIG	115	-11.700	-21.117	8.051	1.00	0.00
ATOM	2020	N6	LIG	115	-14.678	-17.252	21.508	1.00	0.00
ATOM	2021	C1	LIG	115	-12.437	-21.338	12.736	1.00	0.00

ATOM	2022	C2	LIG	115	-11.920	-22.658	12.871	1.00	0.00
ATOM	2023	C3	LIG	115	-12.843	-20.623	13.924	1.00	0.00
ATOM	2024	C4	LIG	115	-12.137	-22.557	15.284	1.00	0.00
ATOM	2025	C5	LIG	115	-11.771	-23.237	14.156	1.00	0.00
ATOM	2026	H1	LIG	115	-11.339	-24.225	14.247	1.00	0.00
ATOM	2027	C6	LIG	115	-12.515	-20.804	11.423	1.00	0.00
ATOM	2028	C7	LIG	115	-11.636	-22.867	10.466	1.00	0.00
ATOM	2029	C8	LIG	115	-11.546	-23.400	11.724	1.00	0.00
ATOM	2030	H2	LIG	115	-11.192	-24.416	11.843	1.00	0.00
ATOM	2031	C9	LIG	115	-12.697	-21.240	15.211	1.00	0.00
ATOM	2032	C10	LIG	115	-12.106	-21.532	10.281	1.00	0.00
ATOM	2033	C11	LIG	115	-13.663	-19.923	18.375	1.00	0.00
ATOM	2034	C12	LIG	115	-11.966	-20.328	6.916	1.00	0.00
ATOM	2035	C13	LIG	115	-13.335	-18.561	18.269	1.00	0.00
ATOM	2036	H8	LIG	115	-12.799	-18.207	17.398	1.00	0.00
ATOM	2037	C14	LIG	115	-14.296	-20.405	19.530	1.00	0.00
ATOM	2038	H9	LIG	115	-14.522	-21.460	19.613	1.00	0.00
ATOM	2039	C15	LIG	115	-13.668	-17.688	19.292	1.00	0.00
ATOM	2040	H10	LIG	115	-13.420	-16.637	19.241	1.00	0.00
ATOM	2041	C16	LIG	115	-14.638	-19.534	20.554	1.00	0.00
ATOM	2042	H11	LIG	115	-15.136	-19.881	21.449	1.00	0.00
ATOM	2043	C17	LIG	115	-14.323	-18.185	20.418	1.00	0.00

```
ATOM   2044  C18 LIG   115     -13.028 -19.412   6.826  1.00  0.00
ATOM   2045  H12 LIG   115     -13.717 -19.306   7.654  1.00  0.00
ATOM   2046  C19 LIG   115     -11.111 -20.519   5.821  1.00  0.00
ATOM   2047  H13 LIG   115     -10.311 -21.246   5.893  1.00  0.00
ATOM   2048  C20 LIG   115     -13.204 -18.681   5.662  1.00  0.00
ATOM   2049  H14 LIG   115     -14.028 -17.981   5.585  1.00  0.00
ATOM   2050  C21 LIG   115     -11.289 -19.773   4.662  1.00  0.00
ATOM   2051  H15 LIG   115     -10.621 -19.916   3.822  1.00  0.00
ATOM   2052  C22 LIG   115     -12.333 -18.852   4.581  1.00  0.00
ATOM   2053  H16 LIG   115     -12.480 -18.279   3.673  1.00  0.00
TER
ATOM   2054  S1  LIG   116      13.801  25.766   3.292  1.00  0.00
ATOM   2055  S2  LIG   116       8.984  25.620   9.381  1.00  0.00
ATOM   2056  O1  LIG   116      14.146  25.347  10.180  1.00  0.00
ATOM   2057  H5  LIG   116      13.732  25.369  11.069  1.00  0.00
ATOM   2058  O2  LIG   116      14.467  24.411   2.749  1.00  0.00
ATOM   2059  H6  LIG   116      15.368  24.399   3.194  1.00  0.00
ATOM   2060  O3  LIG   116       8.842  26.926  10.307  1.00  0.00
ATOM   2061  H7  LIG   116       9.467  26.751  11.069  1.00  0.00
ATOM   2062  O4  LIG   116      12.442  25.794   2.791  1.00  0.00
ATOM   2063  O5  LIG   116      14.703  26.887   3.072  1.00  0.00
ATOM   2064  O6  LIG   116       8.184  25.856   8.197  1.00  0.00
```

ATOM	2065	O7	LIG	116	8.801	24.423	10.187	1.00	0.00
ATOM	2066	O8	LIG	116	22.797	25.377	3.722	1.00	0.00
ATOM	2067	O9	LIG	116	22.320	24.129	2.011	1.00	0.00
ATOM	2068	N1	LIG	116	15.798	25.091	8.119	1.00	0.00
ATOM	2069	H3	LIG	116	15.725	25.132	9.123	1.00	0.00
ATOM	2070	H4	LIG	116	16.693	25.020	7.660	1.00	0.00
ATOM	2071	N2	LIG	116	16.211	25.182	5.552	1.00	0.00
ATOM	2072	N3	LIG	116	11.832	25.598	11.210	1.00	0.00
ATOM	2073	N4	LIG	116	16.531	24.865	4.369	1.00	0.00
ATOM	2074	N5	LIG	116	10.831	26.002	11.862	1.00	0.00
ATOM	2075	N6	LIG	116	22.010	24.768	3.007	1.00	0.00
ATOM	2076	C1	LIG	116	13.395	25.425	7.908	1.00	0.00
ATOM	2077	C2	LIG	116	12.283	25.561	7.030	1.00	0.00
ATOM	2078	C3	LIG	116	14.718	25.283	7.343	1.00	0.00
ATOM	2079	C4	LIG	116	13.734	25.478	5.088	1.00	0.00
ATOM	2080	C5	LIG	116	12.483	25.595	5.627	1.00	0.00
ATOM	2081	H1	LIG	116	11.633	25.739	4.972	1.00	0.00
ATOM	2082	C6	LIG	116	13.127	25.445	9.301	1.00	0.00
ATOM	2083	C7	LIG	116	10.733	25.677	8.899	1.00	0.00
ATOM	2084	C8	LIG	116	10.970	25.666	7.550	1.00	0.00
ATOM	2085	H2	LIG	116	10.133	25.728	6.867	1.00	0.00
ATOM	2086	C9	LIG	116	14.889	25.304	5.919	1.00	0.00

ATOM 2087 C10 LIG 116 11.819 25.589 9.821 1.00 0.00
ATOM 2088 C11 LIG 116 17.911 24.872 4.086 1.00 0.00
ATOM 2089 C12 LIG 116 10.912 25.920 13.265 1.00 0.00
ATOM 2090 C13 LIG 116 18.858 25.561 4.862 1.00 0.00
ATOM 2091 H8 LIG 116 18.530 26.138 5.716 1.00 0.00
ATOM 2092 C14 LIG 116 18.316 24.178 2.937 1.00 0.00
ATOM 2093 H9 LIG 116 17.576 23.671 2.331 1.00 0.00
ATOM 2094 C15 LIG 116 20.197 25.526 4.510 1.00 0.00
ATOM 2095 H10 LIG 116 20.946 26.054 5.084 1.00 0.00
ATOM 2096 C16 LIG 116 19.657 24.134 2.584 1.00 0.00
ATOM 2097 H11 LIG 116 19.994 23.596 1.709 1.00 0.00
ATOM 2098 C17 LIG 116 20.580 24.805 3.380 1.00 0.00
ATOM 2099 C18 LIG 116 11.864 25.151 13.955 1.00 0.00
ATOM 2100 H12 LIG 116 12.576 24.554 13.400 1.00 0.00
ATOM 2101 C19 LIG 116 9.945 26.643 13.978 1.00 0.00
ATOM 2102 H13 LIG 116 9.199 27.212 13.436 1.00 0.00
ATOM 2103 C20 LIG 116 11.859 25.139 15.341 1.00 0.00
ATOM 2104 H14 LIG 116 12.586 24.539 15.876 1.00 0.00
ATOM 2105 C21 LIG 116 9.955 26.632 15.368 1.00 0.00
ATOM 2106 H15 LIG 116 9.212 27.199 15.916 1.00 0.00
ATOM 2107 C22 LIG 116 10.912 25.883 16.052 1.00 0.00
ATOM 2108 H16 LIG 116 10.912 25.863 17.135 1.00 0.00

TER

ATOM 2109 S1 LIG 117 -8.153 -25.813 -17.240 1.00 0.00

ATOM 2110 S2 LIG 117 -2.588 -24.625 -22.524 1.00 0.00

ATOM 2111 O1 LIG 117 -4.896 -20.517 -20.254 1.00 0.00

ATOM 2112 H5 LIG 117 -4.197 -20.150 -20.837 1.00 0.00

ATOM 2113 O2 LIG 117 -9.699 -25.642 -17.635 1.00 0.00

ATOM 2114 H6 LIG 117 -9.913 -24.705 -17.342 1.00 0.00

ATOM 2115 O3 LIG 117 -1.128 -24.156 -22.042 1.00 0.00

ATOM 2116 H7 LIG 117 -1.146 -23.161 -22.150 1.00 0.00

ATOM 2117 O4 LIG 117 -7.731 -27.102 -17.748 1.00 0.00

ATOM 2118 O5 LIG 117 -7.954 -25.456 -15.843 1.00 0.00

ATOM 2119 O6 LIG 117 -2.670 -26.052 -22.289 1.00 0.00

ATOM 2120 O7 LIG 117 -2.879 -24.058 -23.832 1.00 0.00

ATOM 2121 O8 LIG 117 -12.636 -19.363 -12.817 1.00 0.00

ATOM 2122 O9 LIG 117 -14.173 -20.829 -13.264 1.00 0.00

ATOM 2123 N1 LIG 117 -6.999 -20.878 -18.677 1.00 0.00

ATOM 2124 H3 LIG 117 -6.394 -20.198 -19.108 1.00 0.00

ATOM 2125 H4 LIG 117 -7.738 -20.600 -18.049 1.00 0.00

ATOM 2126 N2 LIG 117 -8.525 -22.479 -17.306 1.00 0.00

ATOM 2127 N3 LIG 117 -3.026 -21.356 -21.762 1.00 0.00

ATOM 2128 N4 LIG 117 -9.537 -23.098 -16.865 1.00 0.00

ATOM 2129 N5 LIG 117 -1.903 -21.593 -22.286 1.00 0.00

ATOM 2130 N6 LIG 117 -13.047 -20.367 -13.386 1.00 0.00

ATOM 2131 C1 LIG 117 -5.697 -22.690 -19.650 1.00 0.00

ATOM 2132 C2 LIG 117 -5.528 -24.097 -19.786 1.00 0.00

ATOM 2133 C3 LIG 117 -6.752 -22.193 -18.797 1.00 0.00

ATOM 2134 C4 LIG 117 -7.348 -24.523 -18.242 1.00 0.00

ATOM 2135 C5 LIG 117 -6.360 -24.988 -19.063 1.00 0.00

ATOM 2136 H1 LIG 117 -6.194 -26.055 -19.145 1.00 0.00

ATOM 2137 C6 LIG 117 -4.799 -21.857 -20.367 1.00 0.00

ATOM 2138 C7 LIG 117 -3.674 -23.795 -21.330 1.00 0.00

ATOM 2139 C8 LIG 117 -4.526 -24.617 -20.642 1.00 0.00

ATOM 2140 H2 LIG 117 -4.438 -25.689 -20.764 1.00 0.00

ATOM 2141 C9 LIG 117 -7.590 -23.119 -18.090 1.00 0.00

ATOM 2142 C10 LIG 117 -3.774 -22.378 -21.192 1.00 0.00

ATOM 2143 C11 LIG 117 -10.368 -22.369 -15.991 1.00 0.00

ATOM 2144 C12 LIG 117 -1.243 -20.508 -22.892 1.00 0.00

ATOM 2145 C13 LIG 117 -9.948 -21.218 -15.302 1.00 0.00

ATOM 2146 H8 LIG 117 -8.932 -20.867 -15.421 1.00 0.00

ATOM 2147 C14 LIG 117 -11.660 -22.874 -15.792 1.00 0.00

ATOM 2148 H9 LIG 117 -11.967 -23.775 -16.308 1.00 0.00

ATOM 2149 C15 LIG 117 -10.824 -20.564 -14.453 1.00 0.00

ATOM 2150 H10 LIG 117 -10.525 -19.682 -13.903 1.00 0.00

ATOM 2151 C16 LIG 117 -12.544 -22.218 -14.947 1.00 0.00

```
ATOM   2152  H11 LIG   117     -13.549 -22.582 -14.787  1.00  0.00
ATOM   2153  C17 LIG   117     -12.114 -21.068 -14.293  1.00  0.00
ATOM   2154  C18 LIG   117      -1.866 -19.293 -23.223  1.00  0.00
ATOM   2155  H12 LIG   117      -2.924 -19.164 -23.038  1.00  0.00
ATOM   2156  C19 LIG   117       0.113 -20.705 -23.192  1.00  0.00
ATOM   2157  H13 LIG   117       0.577 -21.655 -22.956  1.00  0.00
ATOM   2158  C20 LIG   117      -1.125 -18.284 -23.818  1.00  0.00
ATOM   2159  H14 LIG   117      -1.605 -17.349 -24.085  1.00  0.00
ATOM   2160  C21 LIG   117       0.850 -19.683 -23.779  1.00  0.00
ATOM   2161  H15 LIG   117       1.899 -19.835 -24.001  1.00  0.00
ATOM   2162  C22 LIG   117       0.234 -18.471 -24.091  1.00  0.00
ATOM   2163  H16 LIG   117       0.805 -17.678 -24.561  1.00  0.00
TER
ATOM   2164  S1  LIG   118      13.302  17.171   9.087  1.00  0.00
ATOM   2165  S2  LIG   118       7.484  13.243  12.409  1.00  0.00
ATOM   2166  O1  LIG   118      12.052  10.841  11.561  1.00  0.00
ATOM   2167  H5  LIG   118      11.486  10.193  12.031  1.00  0.00
ATOM   2168  O2  LIG   118      13.642  16.961   7.533  1.00  0.00
ATOM   2169  H6  LIG   118      14.385  16.285   7.546  1.00  0.00
ATOM   2170  O3  LIG   118       7.538  12.934  13.985  1.00  0.00
ATOM   2171  H7  LIG   118       7.911  12.007  14.037  1.00  0.00
ATOM   2172  O4  LIG   118      12.147  18.043   9.150  1.00  0.00
```

ATOM	2173	O5	LIG	118	14.518	17.483	9.825	1.00	0.00
ATOM	2174	O6	LIG	118	7.056	14.619	12.265	1.00	0.00
ATOM	2175	O7	LIG	118	6.795	12.163	11.720	1.00	0.00
ATOM	2176	O8	LIG	118	21.491	13.863	7.278	1.00	0.00
ATOM	2177	O9	LIG	118	21.041	15.076	5.535	1.00	0.00
ATOM	2178	N1	LIG	118	13.952	12.054	10.161	1.00	0.00
ATOM	2179	H3	LIG	118	13.685	11.206	10.634	1.00	0.00
ATOM	2180	H4	LIG	118	14.863	12.157	9.740	1.00	0.00
ATOM	2181	N2	LIG	118	14.904	14.223	9.082	1.00	0.00
ATOM	2182	N3	LIG	118	9.750	10.739	12.641	1.00	0.00
ATOM	2183	N4	LIG	118	15.360	15.054	8.243	1.00	0.00
ATOM	2184	N5	LIG	118	8.798	10.618	13.458	1.00	0.00
ATOM	2185	N6	LIG	118	20.729	14.521	6.580	1.00	0.00
ATOM	2186	C1	LIG	118	11.853	13.104	10.808	1.00	0.00
ATOM	2187	C2	LIG	118	11.039	14.272	10.760	1.00	0.00
ATOM	2188	C3	LIG	118	13.164	13.142	10.201	1.00	0.00
ATOM	2189	C4	LIG	118	12.777	15.505	9.602	1.00	0.00
ATOM	2190	C5	LIG	118	11.531	15.459	10.162	1.00	0.00
ATOM	2191	H1	LIG	118	10.918	16.351	10.169	1.00	0.00
ATOM	2192	C6	LIG	118	11.315	11.966	11.462	1.00	0.00
ATOM	2193	C7	LIG	118	9.234	13.146	11.936	1.00	0.00
ATOM	2194	C8	LIG	118	9.736	14.258	11.314	1.00	0.00

ATOM	2195	H2	LIG	118	9.117	15.142	11.234	1.00	0.00
ATOM	2196	C9	LIG	118	13.629	14.353	9.587	1.00	0.00
ATOM	2197	C10	LIG	118	10.027	11.965	12.049	1.00	0.00
ATOM	2198	C11	LIG	118	16.708	14.878	7.877	1.00	0.00
ATOM	2199	C12	LIG	118	8.553	9.327	13.963	1.00	0.00
ATOM	2200	C13	LIG	118	17.630	14.145	8.644	1.00	0.00
ATOM	2201	H8	LIG	118	17.312	13.698	9.576	1.00	0.00
ATOM	2202	C14	LIG	118	17.125	15.519	6.702	1.00	0.00
ATOM	2203	H9	LIG	118	16.414	16.103	6.131	1.00	0.00
ATOM	2204	C15	LIG	118	18.943	14.027	8.219	1.00	0.00
ATOM	2205	H10	LIG	118	19.675	13.477	8.793	1.00	0.00
ATOM	2206	C16	LIG	118	18.438	15.398	6.268	1.00	0.00
ATOM	2207	H11	LIG	118	18.779	15.874	5.360	1.00	0.00
ATOM	2208	C17	LIG	118	19.327	14.649	7.032	1.00	0.00
ATOM	2209	C18	LIG	118	9.072	8.150	13.397	1.00	0.00
ATOM	2210	H12	LIG	118	9.678	8.206	12.503	1.00	0.00
ATOM	2211	C19	LIG	118	7.712	9.255	15.083	1.00	0.00
ATOM	2212	H13	LIG	118	7.297	10.167	15.495	1.00	0.00
ATOM	2213	C20	LIG	118	8.772	6.925	13.972	1.00	0.00
ATOM	2214	H14	LIG	118	9.162	6.014	13.533	1.00	0.00
ATOM	2215	C21	LIG	118	7.426	8.022	15.658	1.00	0.00
ATOM	2216	H15	LIG	118	6.784	7.971	16.528	1.00	0.00

```
ATOM   2217  C22 LIG   118       7.956   6.857  15.106  1.00  0.00
ATOM   2218  H16 LIG   118       7.723   5.894  15.547  1.00  0.00
TER
ATOM   2219  S1  LIG   119      21.802 -19.705 -10.797  1.00  0.00
ATOM   2220  S2  LIG   119      23.612 -22.021 -17.984  1.00  0.00
ATOM   2221  O1  LIG   119      18.598 -21.220 -16.728  1.00  0.00
ATOM   2222  H5  LIG   119      18.608 -21.450 -17.682  1.00  0.00
ATOM   2223  O2  LIG   119      21.230 -20.753  -9.724  1.00  0.00
ATOM   2224  H6  LIG   119      20.234 -20.632  -9.787  1.00  0.00
ATOM   2225  O3  LIG   119      23.547 -20.966 -19.194  1.00  0.00
ATOM   2226  H7  LIG   119      22.646 -21.119 -19.601  1.00  0.00
ATOM   2227  O4  LIG   119      23.235 -19.907 -10.857  1.00  0.00
ATOM   2228  O5  LIG   119      21.241 -18.384 -10.550  1.00  0.00
ATOM   2229  O6  LIG   119      24.853 -21.776 -17.279  1.00  0.00
ATOM   2230  O7  LIG   119      23.272 -23.349 -18.472  1.00  0.00
ATOM   2231  O8  LIG   119      13.472 -18.059  -7.768  1.00  0.00
ATOM   2232  O9  LIG   119      14.425 -19.068  -6.099  1.00  0.00
ATOM   2233  N1  LIG   119      17.922 -20.724 -14.211  1.00  0.00
ATOM   2234  H3  LIG   119      17.580 -20.876 -15.146  1.00  0.00
ATOM   2235  H4  LIG   119      17.295 -20.504 -13.452  1.00  0.00
ATOM   2236  N2  LIG   119      18.619 -20.095 -11.784  1.00  0.00
ATOM   2237  N3  LIG   119      20.293 -21.697 -18.566  1.00  0.00
```

```
ATOM   2238  N4  LIG   119      18.771 -20.118 -10.528  1.00  0.00
ATOM   2239  N5  LIG   119      20.984 -21.656 -19.620  1.00  0.00
ATOM   2240  N6  LIG   119      14.386 -18.701  -7.265  1.00  0.00
ATOM   2241  C1  LIG   119      20.219 -20.926 -14.992  1.00  0.00
ATOM   2242  C2  LIG   119      21.601 -20.903 -14.649  1.00  0.00
ATOM   2243  C3  LIG   119      19.241 -20.657 -13.963  1.00  0.00
ATOM   2244  C4  LIG   119      21.082 -20.308 -12.357  1.00  0.00
ATOM   2245  C5  LIG   119      22.003 -20.581 -13.329  1.00  0.00
ATOM   2246  H1  LIG   119      23.058 -20.527 -13.092  1.00  0.00
ATOM   2247  C6  LIG   119      19.891 -21.211 -16.342  1.00  0.00
ATOM   2248  C7  LIG   119      22.244 -21.477 -16.922  1.00  0.00
ATOM   2249  C8  LIG   119      22.584 -21.198 -15.625  1.00  0.00
ATOM   2250  H2  LIG   119      23.627 -21.214 -15.338  1.00  0.00
ATOM   2251  C9  LIG   119      19.675 -20.349 -12.630  1.00  0.00
ATOM   2252  C10 LIG   119      20.875 -21.468 -17.326  1.00  0.00
ATOM   2253  C11 LIG   119      17.647 -19.742  -9.768  1.00  0.00
ATOM   2254  C12 LIG   119      20.323 -21.959 -20.825  1.00  0.00
ATOM   2255  C13 LIG   119      16.575 -18.997 -10.287  1.00  0.00
ATOM   2256  H8  LIG   119      16.602 -18.669 -11.317  1.00  0.00
ATOM   2257  C14 LIG   119      17.654 -20.112  -8.415  1.00  0.00
ATOM   2258  H9  LIG   119      18.497 -20.665  -8.018  1.00  0.00
ATOM   2259  C15 LIG   119      15.509 -18.658  -9.470  1.00  0.00
```

```
ATOM   2260  H10 LIG   119      14.675 -18.078  -9.839  1.00  0.00
ATOM   2261  C16 LIG   119      16.586 -19.782  -7.594  1.00  0.00
ATOM   2262  H11 LIG   119      16.565 -20.066  -6.551  1.00  0.00
ATOM   2263  C17 LIG   119      15.525 -19.062  -8.136  1.00  0.00
ATOM   2264  C18 LIG   119      19.071 -22.593 -20.898  1.00  0.00
ATOM   2265  H12 LIG   119      18.573 -22.903 -19.989  1.00  0.00
ATOM   2266  C19 LIG   119      21.004 -21.616 -22.002  1.00  0.00
ATOM   2267  H13 LIG   119      21.981 -21.152 -21.934  1.00  0.00
ATOM   2268  C20 LIG   119      18.504 -22.847 -22.137  1.00  0.00
ATOM   2269  H14 LIG   119      17.543 -23.345 -22.197  1.00  0.00
ATOM   2270  C21 LIG   119      20.422 -21.866 -23.239  1.00  0.00
ATOM   2271  H15 LIG   119      20.947 -21.591 -24.146  1.00  0.00
ATOM   2272  C22 LIG   119      19.172 -22.478 -23.310  1.00  0.00
ATOM   2273  H16 LIG   119      18.723 -22.685 -24.274  1.00  0.00
TER
END
```

Table K: The number of atoms present in the system of BN-Sls

```
ATOM      1  N   GLN     1      -8.592 -10.154   3.231  1.00  0.00
ATOM      2  H1  GLN     1      -7.661 -10.485   3.440  1.00  0.00
ATOM      3  H2  GLN     1      -8.823  -9.408   3.871  1.00  0.00
ATOM      4  H3  GLN     1      -8.669 -10.007   2.235  1.00  0.00
```

ATOM	5	CA	GLN	1	-9.537	-11.232	3.537	1.00	0.00
ATOM	6	HA	GLN	1	-10.239	-10.894	4.299	1.00	0.00
ATOM	7	CB	GLN	1	-10.330	-11.648	2.290	1.00	0.00
ATOM	8	HB2	GLN	1	-10.033	-11.014	1.455	1.00	0.00
ATOM	9	HB3	GLN	1	-10.100	-12.688	2.056	1.00	0.00
ATOM	10	CG	GLN	1	-11.830	-11.501	2.531	1.00	0.00
ATOM	11	HG2	GLN	1	-12.445	-12.308	2.132	1.00	0.00
ATOM	12	HG3	GLN	1	-11.906	-11.490	3.618	1.00	0.00
ATOM	13	CD	GLN	1	-12.355	-10.180	1.994	1.00	0.00
ATOM	14	OE1	GLN	1	-12.713	-9.279	2.734	1.00	0.00
ATOM	15	NE2	GLN	1	-12.251	-10.020	0.688	1.00	0.00
ATOM	16	HE21	GLN	1	-12.579	-9.167	0.259	1.00	0.00
ATOM	17	HE22	GLN	1	-11.844	-10.752	0.123	1.00	0.00
ATOM	18	C	GLN	1	-8.805	-12.435	4.121	1.00	0.00
ATOM	19	O	GLN	1	-9.157	-12.915	5.189	1.00	0.00
ATOM	20	N	PRO	2	-7.747	-12.886	3.425	1.00	0.00
ATOM	21	CD	PRO	2	-7.251	-12.362	2.154	1.00	0.00
ATOM	22	HD2	PRO	2	-6.846	-11.368	2.344	1.00	0.00
ATOM	23	HD3	PRO	2	-8.101	-12.284	1.477	1.00	0.00
ATOM	24	CG	PRO	2	-6.197	-13.335	1.651	1.00	0.00
ATOM	25	HG2	PRO	2	-5.232	-12.830	1.610	1.00	0.00
ATOM	26	HG3	PRO	2	-6.471	-13.676	0.652	1.00	0.00

ATOM	27	CB	PRO	2	-6.174	-14.501	2.638	1.00	0.00
ATOM	28	HB2	PRO	2	-5.154	-14.762	2.921	1.00	0.00
ATOM	29	HB3	PRO	2	-6.668	-15.376	2.216	1.00	0.00
ATOM	30	CA	PRO	2	-6.943	-14.008	3.862	1.00	0.00
ATOM	31	HA	PRO	2	-7.613	-14.814	4.161	1.00	0.00
ATOM	32	C	PRO	2	-6.053	-13.642	5.018	1.00	0.00
ATOM	33	O	PRO	2	-5.064	-12.961	4.806	1.00	0.00
ATOM	34	N	GLN	3	-6.399	-14.105	6.223	1.00	0.00
ATOM	35	H	GLN	3	-7.222	-14.683	6.318	1.00	0.00
ATOM	36	CA	GLN	3	-5.631	-13.803	7.412	1.00	0.00
ATOM	37	HA	GLN	3	-5.557	-12.723	7.535	1.00	0.00
ATOM	38	CB	GLN	3	-6.297	-14.391	8.655	1.00	0.00
ATOM	39	HB2	GLN	3	-7.348	-14.580	8.436	1.00	0.00
ATOM	40	HB3	GLN	3	-5.805	-15.331	8.907	1.00	0.00
ATOM	41	CG	GLN	3	-6.188	-13.422	9.840	1.00	0.00
ATOM	42	HG2	GLN	3	-5.742	-13.966	10.673	1.00	0.00
ATOM	43	HG3	GLN	3	-5.558	-12.570	9.584	1.00	0.00
ATOM	44	CD	GLN	3	-7.545	-12.909	10.269	1.00	0.00
ATOM	45	OE1	GLN	3	-8.165	-13.423	11.179	1.00	0.00
ATOM	46	NE2	GLN	3	-8.011	-11.897	9.559	1.00	0.00
ATOM	47	HE21	GLN	3	-8.911	-11.499	9.784	1.00	0.00
ATOM	48	HE22	GLN	3	-7.465	-11.527	8.794	1.00	0.00

ATOM	49	C	GLN	3	-4.249	-14.380	7.288	1.00	0.00
ATOM	50	O	GLN	3	-3.308	-13.803	7.760	1.00	0.00
ATOM	51	N	LYS	4	-4.083	-15.494	6.583	1.00	0.00
ATOM	52	H	LYS	4	-4.899	-15.976	6.234	1.00	0.00
ATOM	53	CA	LYS	4	-2.779	-16.101	6.367	1.00	0.00
ATOM	54	HA	LYS	4	-2.428	-16.580	7.281	1.00	0.00
ATOM	55	CB	LYS	4	-2.886	-17.163	5.269	1.00	0.00
ATOM	56	HB2	LYS	4	-2.054	-17.859	5.380	1.00	0.00
ATOM	57	HB3	LYS	4	-3.826	-17.699	5.398	1.00	0.00
ATOM	58	CG	LYS	4	-2.844	-16.534	3.867	1.00	0.00
ATOM	59	HG2	LYS	4	-3.053	-15.468	3.955	1.00	0.00
ATOM	60	HG3	LYS	4	-1.847	-16.676	3.449	1.00	0.00
ATOM	61	CD	LYS	4	-3.878	-17.181	2.947	1.00	0.00
ATOM	62	HD2	LYS	4	-4.841	-17.203	3.458	1.00	0.00
ATOM	63	HD3	LYS	4	-3.963	-16.586	2.038	1.00	0.00
ATOM	64	CE	LYS	4	-3.462	-18.606	2.585	1.00	0.00
ATOM	65	HE2	LYS	4	-3.261	-18.659	1.515	1.00	0.00
ATOM	66	HE3	LYS	4	-2.559	-18.866	3.137	1.00	0.00
ATOM	67	NZ	LYS	4	-4.538	-19.553	2.930	1.00	0.00
ATOM	68	HZ1	LYS	4	-5.375	-19.313	2.418	1.00	0.00
ATOM	69	HZ2	LYS	4	-4.252	-20.491	2.686	1.00	0.00
ATOM	70	HZ3	LYS	4	-4.724	-19.505	3.921	1.00	0.00

ATOM	71	C	LYS	4	-1.725	-15.029	6.038	1.00	0.00
ATOM	72	O	LYS	4	-0.582	-15.193	6.402	1.00	0.00
ATOM	73	N	CYX	5	-2.169	-13.930	5.402	1.00	0.00
ATOM	74	H	CYX	5	-3.129	-13.897	5.090	1.00	0.00
ATOM	75	CA	CYX	5	-1.330	-12.813	5.126	1.00	0.00
ATOM	76	HA	CYX	5	-0.313	-13.009	5.465	1.00	0.00
ATOM	77	CB	CYX	5	-1.303	-12.568	3.640	1.00	0.00
ATOM	78	HB2	CYX	5	-1.894	-13.332	3.134	1.00	0.00
ATOM	79	HB3	CYX	5	-1.721	-11.585	3.426	1.00	0.00
ATOM	80	SG	CYX	5	0.363	-12.646	2.914	1.00	0.00
ATOM	81	C	CYX	5	-1.842	-11.607	5.844	1.00	0.00
ATOM	82	O	CYX	5	-1.086	-10.850	6.370	1.00	0.00
ATOM	83	N	GLN	6	-3.152	-11.416	5.967	1.00	0.00
ATOM	84	H	GLN	6	-3.766	-12.054	5.480	1.00	0.00
ATOM	85	CA	GLN	6	-3.742	-10.311	6.686	1.00	0.00
ATOM	86	HA	GLN	6	-3.331	-9.371	6.318	1.00	0.00
ATOM	87	CB	GLN	6	-5.259	-10.291	6.491	1.00	0.00
ATOM	88	HB2	GLN	6	-5.577	-11.277	6.153	1.00	0.00
ATOM	89	HB3	GLN	6	-5.728	-10.067	7.449	1.00	0.00
ATOM	90	CG	GLN	6	-5.675	-9.244	5.465	1.00	0.00
ATOM	91	HG2	GLN	6	-4.761	-8.739	5.152	1.00	0.00
ATOM	92	HG3	GLN	6	-6.149	-9.706	4.599	1.00	0.00

ATOM	93	CD	GLN	6	-6.621	-8.216	6.043	1.00	0.00
ATOM	94	OE1	GLN	6	-6.641	-7.917	7.222	1.00	0.00
ATOM	95	NE2	GLN	6	-7.483	-7.698	5.179	1.00	0.00
ATOM	96	HE21	GLN	6	-8.148	-7.004	5.488	1.00	0.00
ATOM	97	HE22	GLN	6	-7.470	-8.000	4.215	1.00	0.00
ATOM	98	C	GLN	6	-3.390	-10.350	8.163	1.00	0.00
ATOM	99	O	GLN	6	-3.635	-9.403	8.891	1.00	0.00
ATOM	100	N	ARG	7	-2.824	-11.458	8.623	1.00	0.00
ATOM	101	H	ARG	7	-2.700	-12.208	7.957	1.00	0.00
ATOM	102	CA	ARG	7	-2.413	-11.678	9.977	1.00	0.00
ATOM	103	HA	ARG	7	-2.818	-10.887	10.608	1.00	0.00
ATOM	104	CB	ARG	7	-2.908	-13.028	10.506	1.00	0.00
ATOM	105	HB2	ARG	7	-3.713	-13.387	9.864	1.00	0.00
ATOM	106	HB3	ARG	7	-2.083	-13.741	10.486	1.00	0.00
ATOM	107	CG	ARG	7	-3.423	-12.886	11.935	1.00	0.00
ATOM	108	HG2	ARG	7	-2.695	-12.319	12.514	1.00	0.00
ATOM	109	HG3	ARG	7	-4.370	-12.346	11.913	1.00	0.00
ATOM	110	CD	ARG	7	-3.633	-14.259	12.583	1.00	0.00
ATOM	111	HD2	ARG	7	-4.627	-14.635	12.338	1.00	0.00
ATOM	112	HD3	ARG	7	-2.881	-14.959	12.217	1.00	0.00
ATOM	113	NE	ARG	7	-3.513	-14.160	14.052	1.00	0.00
ATOM	114	HE	ARG	7	-4.375	-14.068	14.570	1.00	0.00

ATOM 115 CZ ARG 7 -2.371 -14.178 14.723 1.00 0.00

ATOM 116 NH1 ARG 7 -1.229 -14.309 14.089 1.00 0.00

ATOM 117 HH11 ARG 7 -1.221 -14.397 13.083 1.00 0.00

ATOM 118 HH12 ARG 7 -0.364 -14.322 14.610 1.00 0.00

ATOM 119 NH2 ARG 7 -2.369 -14.071 16.030 1.00 0.00

ATOM 120 HH21 ARG 7 -3.243 -13.975 16.527 1.00 0.00

ATOM 121 HH22 ARG 7 -1.494 -14.086 16.533 1.00 0.00

ATOM 122 C ARG 7 -0.912 -11.624 10.078 1.00 0.00

ATOM 123 O ARG 7 -0.436 -11.631 11.186 1.00 0.00

ATOM 124 N GLU 8 -0.216 -11.612 8.954 1.00 0.00

ATOM 125 H GLU 8 -0.714 -11.705 8.080 1.00 0.00

ATOM 126 CA GLU 8 1.229 -11.535 8.915 1.00 0.00

ATOM 127 HA GLU 8 1.631 -11.577 9.927 1.00 0.00

ATOM 128 CB GLU 8 1.798 -12.707 8.115 1.00 0.00

ATOM 129 HB2 GLU 8 0.975 -13.251 7.650 1.00 0.00

ATOM 130 HB3 GLU 8 2.460 -12.319 7.341 1.00 0.00

ATOM 131 CG GLU 8 2.583 -13.654 9.030 1.00 0.00

ATOM 132 HG2 GLU 8 2.239 -13.459 10.045 1.00 0.00

ATOM 133 HG3 GLU 8 2.391 -14.697 8.776 1.00 0.00

ATOM 134 CD GLU 8 4.087 -13.375 8.938 1.00 0.00

ATOM 135 OE1 GLU 8 4.646 -13.639 7.854 1.00 0.00

ATOM 136 OE2 GLU 8 4.643 -12.959 9.973 1.00 0.00

ATOM	137	C	GLU	8	1.705	-10.214	8.328	1.00	0.00
ATOM	138	O	GLU	8	2.773	-9.729	8.644	1.00	0.00
ATOM	139	N	PHE	9	0.846	-9.558	7.560	1.00	0.00
ATOM	140	H	PHE	9	-0.068	-9.968	7.428	1.00	0.00
ATOM	141	CA	PHE	9	1.131	-8.316	6.915	1.00	0.00
ATOM	142	HA	PHE	9	2.169	-8.283	6.585	1.00	0.00
ATOM	143	CB	PHE	9	0.227	-8.180	5.694	1.00	0.00
ATOM	144	HB2	PHE	9	0.702	-8.676	4.847	1.00	0.00
ATOM	145	HB3	PHE	9	-0.744	-8.638	5.885	1.00	0.00
ATOM	146	CG	PHE	9	-0.044	-6.753	5.265	1.00	0.00
ATOM	147	CD1	PHE	9	-0.867	-5.914	6.035	1.00	0.00
ATOM	148	HD1	PHE	9	-1.339	-6.316	6.931	1.00	0.00
ATOM	149	CE1	PHE	9	-1.084	-4.588	5.669	1.00	0.00
ATOM	150	HE1	PHE	9	-1.683	-3.935	6.303	1.00	0.00
ATOM	151	CZ	PHE	9	-0.533	-4.098	4.488	1.00	0.00
ATOM	152	HZ	PHE	9	-0.707	-3.067	4.179	1.00	0.00
ATOM	153	CE2	PHE	9	0.249	-4.941	3.696	1.00	0.00
ATOM	154	HE2	PHE	9	0.661	-4.557	2.763	1.00	0.00
ATOM	155	CD2	PHE	9	0.506	-6.259	4.083	1.00	0.00
ATOM	156	HD2	PHE	9	1.134	-6.897	3.461	1.00	0.00
ATOM	157	C	PHE	9	0.863	-7.203	7.885	1.00	0.00
ATOM	158	O	PHE	9	1.537	-6.193	7.905	1.00	0.00

ATOM	159	N	GLN	10	-0.210	-7.330	8.682	1.00	0.00
ATOM	160	H	GLN	10	-0.799	-8.146	8.587	1.00	0.00
ATOM	161	CA	GLN	10	-0.598	-6.312	9.629	1.00	0.00
ATOM	162	HA	GLN	10	-0.144	-5.361	9.350	1.00	0.00
ATOM	163	CB	GLN	10	-2.121	-6.140	9.652	1.00	0.00
ATOM	164	HB2	GLN	10	-2.417	-5.542	8.791	1.00	0.00
ATOM	165	HB3	GLN	10	-2.584	-7.125	9.586	1.00	0.00
ATOM	166	CG	GLN	10	-2.580	-5.444	10.941	1.00	0.00
ATOM	167	HG2	GLN	10	-2.432	-6.103	11.796	1.00	0.00
ATOM	168	HG3	GLN	10	-1.979	-4.544	11.068	1.00	0.00
ATOM	169	CD	GLN	10	-4.031	-5.049	10.883	1.00	0.00
ATOM	170	OE1	GLN	10	-4.400	-4.052	10.283	1.00	0.00
ATOM	171	NE2	GLN	10	-4.870	-5.864	11.496	1.00	0.00
ATOM	172	HE21	GLN	10	-5.859	-5.659	11.496	1.00	0.00
ATOM	173	HE22	GLN	10	-4.519	-6.689	11.962	1.00	0.00
ATOM	174	C	GLN	10	-0.055	-6.683	11.003	1.00	0.00
ATOM	175	O	GLN	10	-0.072	-5.875	11.930	1.00	0.00
ATOM	176	N	GLN	11	0.414	-7.915	11.161	1.00	0.00
ATOM	177	H	GLN	11	0.370	-8.567	10.390	1.00	0.00
ATOM	178	CA	GLN	11	0.944	-8.378	12.417	1.00	0.00
ATOM	179	HA	GLN	11	0.139	-8.478	13.145	1.00	0.00
ATOM	180	CB	GLN	11	1.613	-9.736	12.241	1.00	0.00

ATOM 181 HB2 GLN 11 1.285 -10.173 11.297 1.00 0.00
ATOM 182 HB3 GLN 11 2.694 -9.598 12.221 1.00 0.00
ATOM 183 CG GLN 11 1.240 -10.673 13.393 1.00 0.00
ATOM 184 HG2 GLN 11 0.421 -10.202 13.936 1.00 0.00
ATOM 185 HG3 GLN 11 0.915 -11.644 13.018 1.00 0.00
ATOM 186 CD GLN 11 2.396 -10.885 14.349 1.00 0.00
ATOM 187 OE1 GLN 11 3.550 -10.924 13.981 1.00 0.00
ATOM 188 NE2 GLN 11 2.056 -11.077 15.613 1.00 0.00
ATOM 189 HE21 GLN 11 2.773 -11.226 16.309 1.00 0.00
ATOM 190 HE22 GLN 11 1.081 -11.075 15.876 1.00 0.00
ATOM 191 C GLN 11 1.932 -7.377 12.985 1.00 0.00
ATOM 192 O GLN 11 1.733 -6.862 14.081 1.00 0.00
ATOM 193 N GLU 12 2.922 -7.008 12.171 1.00 0.00
ATOM 194 H GLU 12 2.992 -7.421 11.252 1.00 0.00
ATOM 195 CA GLU 12 3.966 -6.093 12.596 1.00 0.00
ATOM 196 HA GLU 12 3.519 -5.121 12.805 1.00 0.00
ATOM 197 CB GLU 12 4.692 -6.581 13.859 1.00 0.00
ATOM 198 HB2 GLU 12 5.716 -6.208 13.833 1.00 0.00
ATOM 199 HB3 GLU 12 4.179 -6.173 14.730 1.00 0.00
ATOM 200 CG GLU 12 4.708 -8.110 13.947 1.00 0.00
ATOM 201 HG2 GLU 12 3.809 -8.476 14.443 1.00 0.00
ATOM 202 HG3 GLU 12 4.727 -8.471 12.918 1.00 0.00

ATOM	203	CD	GLU	12	5.934	-8.618	14.683	1.00	0.00
ATOM	204	OE1	GLU	12	6.984	-8.757	14.015	1.00	0.00
ATOM	205	OE2	GLU	12	5.823	-8.809	15.918	1.00	0.00
ATOM	206	C	GLU	12	4.952	-5.853	11.461	1.00	0.00
ATOM	207	O	GLU	12	6.137	-5.617	11.667	1.00	0.00
ATOM	208	N	GLN	13	4.443	-5.870	10.230	1.00	0.00
ATOM	209	H	GLN	13	3.466	-6.091	10.098	1.00	0.00
ATOM	210	CA	GLN	13	5.260	-5.657	9.060	1.00	0.00
ATOM	211	HA	GLN	13	6.004	-4.888	9.266	1.00	0.00
ATOM	212	CB	GLN	13	5.986	-6.946	8.665	1.00	0.00
ATOM	213	HB2	GLN	13	5.407	-7.800	9.018	1.00	0.00
ATOM	214	HB3	GLN	13	6.068	-6.987	7.579	1.00	0.00
ATOM	215	CG	GLN	13	7.382	-6.988	9.282	1.00	0.00
ATOM	216	HG2	GLN	13	7.866	-6.012	9.273	1.00	0.00
ATOM	217	HG3	GLN	13	7.252	-7.319	10.312	1.00	0.00
ATOM	218	CD	GLN	13	8.278	-7.980	8.564	1.00	0.00
ATOM	219	OE1	GLN	13	8.201	-8.205	7.365	1.00	0.00
ATOM	220	NE2	GLN	13	9.215	-8.545	9.308	1.00	0.00
ATOM	221	HE21	GLN	13	9.847	-9.216	8.895	1.00	0.00
ATOM	222	HE22	GLN	13	9.293	-8.303	10.285	1.00	0.00
ATOM	223	C	GLN	13	4.418	-5.134	7.928	1.00	0.00
ATOM	224	O	GLN	13	4.437	-5.670	6.859	1.00	0.00

ATOM	225	N	HIE	14	3.634	-4.088	8.182	1.00	0.00
ATOM	226	H	HIE	14	3.614	-3.723	9.123	1.00	0.00
ATOM	227	CA	HIE	14	2.739	-3.509	7.202	1.00	0.00
ATOM	228	HA	HIE	14	2.131	-4.295	6.754	1.00	0.00
ATOM	229	CB	HIE	14	1.809	-2.480	7.851	1.00	0.00
ATOM	230	HB2	HIE	14	2.271	-1.493	7.856	1.00	0.00
ATOM	231	HB3	HIE	14	0.888	-2.447	7.268	1.00	0.00
ATOM	232	CG	HIE	14	1.410	-2.774	9.267	1.00	0.00
ATOM	233	ND1	HIE	14	0.858	-1.846	10.115	1.00	0.00
ATOM	234	CE1	HIE	14	0.634	-2.431	11.299	1.00	0.00
ATOM	235	HE1	HIE	14	0.170	-1.880	12.117	1.00	0.00
ATOM	236	NE2	HIE	14	1.067	-3.692	11.227	1.00	0.00
ATOM	237	HE2	HIE	14	1.052	-4.364	11.981	1.00	0.00
ATOM	238	CD2	HIE	14	1.538	-3.938	9.966	1.00	0.00
ATOM	239	HD2	HIE	14	1.915	-4.928	9.708	1.00	0.00
ATOM	240	C	HIE	14	3.585	-2.927	6.079	1.00	0.00
ATOM	241	O	HIE	14	4.695	-3.338	5.781	1.00	0.00
ATOM	242	N	LEU	15	3.087	-1.850	5.474	1.00	0.00
ATOM	243	H	LEU	15	2.158	-1.531	5.707	1.00	0.00
ATOM	244	CA	LEU	15	3.790	-1.185	4.401	1.00	0.00
ATOM	245	HA	LEU	15	4.790	-1.604	4.294	1.00	0.00
ATOM	246	CB	LEU	15	3.035	-1.376	3.079	1.00	0.00

ATOM	247	HB2	LEU	15	2.207	-0.673	3.166	1.00	0.00
ATOM	248	HB3	LEU	15	3.672	-1.084	2.244	1.00	0.00
ATOM	249	CG	LEU	15	2.485	-2.784	2.833	1.00	0.00
ATOM	250	HG	LEU	15	1.629	-2.947	3.488	1.00	0.00
ATOM	251	CD1	LEU	15	2.052	-2.951	1.381	1.00	0.00
ATOM	252	HD11	LEU	15	2.907	-2.789	0.725	1.00	0.00
ATOM	253	HD12	LEU	15	1.665	-3.959	1.230	1.00	0.00
ATOM	254	HD13	LEU	15	1.273	-2.225	1.147	1.00	0.00
ATOM	255	CD2	LEU	15	3.470	-3.848	3.225	1.00	0.00
ATOM	256	HD21	LEU	15	3.704	-3.755	4.285	1.00	0.00
ATOM	257	HD22	LEU	15	3.039	-4.831	3.034	1.00	0.00
ATOM	258	HD23	LEU	15	4.383	-3.732	2.640	1.00	0.00
ATOM	259	C	LEU	15	4.017	0.265	4.757	1.00	0.00
ATOM	260	O	LEU	15	3.424	0.763	5.720	1.00	0.00
ATOM	261	N	ARG	16	4.829	0.955	3.955	1.00	0.00
ATOM	262	H	ARG	16	5.244	0.497	3.156	1.00	0.00
ATOM	263	CA	ARG	16	5.138	2.347	4.183	1.00	0.00
ATOM	264	HA	ARG	16	4.279	2.840	4.639	1.00	0.00
ATOM	265	CB	ARG	16	6.339	2.499	5.114	1.00	0.00
ATOM	266	HB2	ARG	16	7.043	1.693	4.908	1.00	0.00
ATOM	267	HB3	ARG	16	6.816	3.458	4.913	1.00	0.00
ATOM	268	CG	ARG	16	5.911	2.440	6.581	1.00	0.00

ATOM	269	HG2	ARG	16	4.840	2.243	6.624	1.00	0.00
ATOM	270	HG3	ARG	16	6.449	1.627	7.069	1.00	0.00
ATOM	271	CD	ARG	16	6.217	3.757	7.297	1.00	0.00
ATOM	272	HD2	ARG	16	6.185	4.577	6.580	1.00	0.00
ATOM	273	HD3	ARG	16	5.476	3.928	8.077	1.00	0.00
ATOM	274	NE	ARG	16	7.556	3.722	7.916	1.00	0.00
ATOM	275	HE	ARG	16	8.193	3.024	7.560	1.00	0.00
ATOM	276	CZ	ARG	16	7.974	4.526	8.876	1.00	0.00
ATOM	277	NH1	ARG	16	7.179	5.454	9.358	1.00	0.00
ATOM	278	HH11	ARG	16	6.243	5.552	8.992	1.00	0.00
ATOM	279	HH12	ARG	16	7.508	6.064	10.092	1.00	0.00
ATOM	280	NH2	ARG	16	9.184	4.402	9.364	1.00	0.00
ATOM	281	HH21	ARG	16	9.800	3.687	9.003	1.00	0.00
ATOM	282	HH22	ARG	16	9.495	5.021	10.098	1.00	0.00
ATOM	283	C	ARG	16	5.460	3.001	2.888	1.00	0.00
ATOM	284	O	ARG	16	4.642	3.736	2.405	1.00	0.00
ATOM	285	N	ALA	17	6.596	2.714	2.290	1.00	0.00
ATOM	286	H	ALA	17	7.256	2.125	2.777	1.00	0.00
ATOM	287	CA	ALA	17	6.983	3.265	1.018	1.00	0.00
ATOM	288	HA	ALA	17	7.140	4.337	1.133	1.00	0.00
ATOM	289	CB	ALA	17	8.299	2.624	0.553	1.00	0.00
ATOM	290	HB1	ALA	17	8.156	1.551	0.424	1.00	0.00

ATOM	291	HB2	ALA	17	8.603	3.065	-0.396	1.00	0.00
ATOM	292	HB3	ALA	17	9.073	2.799	1.300	1.00	0.00
ATOM	293	C	ALA	17	5.859	3.091	-0.022	1.00	0.00
ATOM	294	O	ALA	17	5.761	3.834	-0.989	1.00	0.00
ATOM	295	N	CYX	18	4.920	2.175	0.236	1.00	0.00
ATOM	296	H	CYX	18	5.021	1.617	1.072	1.00	0.00
ATOM	297	CA	CYX	18	3.823	1.901	-0.630	1.00	0.00
ATOM	298	HA	CYX	18	4.110	2.118	-1.659	1.00	0.00
ATOM	299	CB	CYX	18	3.402	0.440	-0.541	1.00	0.00
ATOM	300	HB2	CYX	18	4.212	-0.146	-0.106	1.00	0.00
ATOM	301	HB3	CYX	18	2.515	0.354	0.086	1.00	0.00
ATOM	302	SG	CYX	18	3.040	-0.355	-2.124	1.00	0.00
ATOM	303	C	CYX	18	2.709	2.835	-0.251	1.00	0.00
ATOM	304	O	CYX	18	2.153	3.533	-1.081	1.00	0.00
ATOM	305	N	GLN	19	2.380	2.881	1.037	1.00	0.00
ATOM	306	H	GLN	19	2.813	2.232	1.678	1.00	0.00
ATOM	307	CA	GLN	19	1.365	3.761	1.542	1.00	0.00
ATOM	308	HA	GLN	19	0.647	3.998	0.757	1.00	0.00
ATOM	309	CB	GLN	19	0.630	3.088	2.683	1.00	0.00
ATOM	310	HB2	GLN	19	-0.192	3.740	2.977	1.00	0.00
ATOM	311	HB3	GLN	19	0.229	2.146	2.308	1.00	0.00
ATOM	312	CG	GLN	19	1.519	2.816	3.890	1.00	0.00

ATOM	313	HG2	GLN	19	1.339	1.800	4.243	1.00	0.00
ATOM	314	HG3	GLN	19	2.565	2.920	3.602	1.00	0.00
ATOM	315	CD	GLN	19	1.229	3.785	5.027	1.00	0.00
ATOM	316	OE1	GLN	19	2.115	4.417	5.602	1.00	0.00
ATOM	317	NE2	GLN	19	-0.046	3.906	5.369	1.00	0.00
ATOM	318	HE21	GLN	19	-0.308	4.532	6.116	1.00	0.00
ATOM	319	HE22	GLN	19	-0.750	3.371	4.880	1.00	0.00
ATOM	320	C	GLN	19	1.948	5.088	1.958	1.00	0.00
ATOM	321	O	GLN	19	1.452	5.702	2.873	1.00	0.00
ATOM	322	N	GLN	20	3.031	5.529	1.341	1.00	0.00
ATOM	323	H	GLN	20	3.410	4.961	0.596	1.00	0.00
ATOM	324	CA	GLN	20	3.721	6.746	1.692	1.00	0.00
ATOM	325	HA	GLN	20	3.075	7.340	2.338	1.00	0.00
ATOM	326	CB	GLN	20	5.040	6.487	2.424	1.00	0.00
ATOM	327	HB2	GLN	20	5.598	5.727	1.877	1.00	0.00
ATOM	328	HB3	GLN	20	5.614	7.413	2.446	1.00	0.00
ATOM	329	CG	GLN	20	4.792	6.005	3.861	1.00	0.00
ATOM	330	HG2	GLN	20	3.830	5.495	3.922	1.00	0.00
ATOM	331	HG3	GLN	20	5.590	5.306	4.114	1.00	0.00
ATOM	332	CD	GLN	20	4.804	7.129	4.858	1.00	0.00
ATOM	333	OE1	GLN	20	5.364	8.191	4.655	1.00	0.00
ATOM	334	NE2	GLN	20	4.135	6.918	5.980	1.00	0.00

ATOM	335	HE21	GLN	20	4.106	7.636	6.689	1.00	0.00
ATOM	336	HE22	GLN	20	3.657	6.039	6.121	1.00	0.00
ATOM	337	C	GLN	20	3.985	7.534	0.435	1.00	0.00
ATOM	338	O	GLN	20	3.802	8.742	0.411	1.00	0.00
ATOM	339	N	TRP	21	4.361	6.847	-0.644	1.00	0.00
ATOM	340	H	TRP	21	4.527	5.853	-0.574	1.00	0.00
ATOM	341	CA	TRP	21	4.606	7.496	-1.914	1.00	0.00
ATOM	342	HA	TRP	21	5.369	8.265	-1.796	1.00	0.00
ATOM	343	CB	TRP	21	5.093	6.480	-2.946	1.00	0.00
ATOM	344	HB2	TRP	21	5.595	6.993	-3.766	1.00	0.00
ATOM	345	HB3	TRP	21	5.796	5.807	-2.455	1.00	0.00
ATOM	346	CG	TRP	21	4.038	5.621	-3.562	1.00	0.00
ATOM	347	CD1	TRP	21	3.693	4.352	-3.254	1.00	0.00
ATOM	348	HD1	TRP	21	4.280	3.896	-2.457	1.00	0.00
ATOM	349	NE1	TRP	21	2.647	3.895	-4.034	1.00	0.00
ATOM	350	HE1	TRP	21	2.208	2.988	-3.957	1.00	0.00
ATOM	351	CE2	TRP	21	2.279	4.886	-4.909	1.00	0.00
ATOM	352	CZ2	TRP	21	1.292	4.961	-5.890	1.00	0.00
ATOM	353	HZ2	TRP	21	0.670	4.080	-6.052	1.00	0.00
ATOM	354	CH2	TRP	21	1.159	6.151	-6.603	1.00	0.00
ATOM	355	HH2	TRP	21	0.416	6.236	-7.396	1.00	0.00
ATOM	356	CZ3	TRP	21	1.975	7.255	-6.314	1.00	0.00

ATOM	357	HZ3	TRP	21	1.829	8.196	-6.844	1.00	0.00
ATOM	358	CE3	TRP	21	2.989	7.193	-5.353	1.00	0.00
ATOM	359	HE3	TRP	21	3.620	8.068	-5.201	1.00	0.00
ATOM	360	CD2	TRP	21	3.143	5.999	-4.620	1.00	0.00
ATOM	361	C	TRP	21	3.331	8.190	-2.361	1.00	0.00
ATOM	362	O	TRP	21	3.348	9.260	-2.958	1.00	0.00
ATOM	363	N	ILE	22	2.180	7.590	-2.034	1.00	0.00
ATOM	364	H	ILE	22	2.206	6.706	-1.546	1.00	0.00
ATOM	365	CA	ILE	22	0.891	8.130	-2.405	1.00	0.00
ATOM	366	HA	ILE	22	0.878	8.459	-3.444	1.00	0.00
ATOM	367	CB	ILE	22	-0.204	7.078	-2.189	1.00	0.00
ATOM	368	HB	ILE	22	-0.258	6.830	-1.129	1.00	0.00
ATOM	369	CG2	ILE	22	-1.558	7.622	-2.655	1.00	0.00
ATOM	370	HG21	ILE	22	-1.505	7.869	-3.715	1.00	0.00
ATOM	371	HG22	ILE	22	-2.328	6.866	-2.497	1.00	0.00
ATOM	372	HG23	ILE	22	-1.806	8.517	-2.085	1.00	0.00
ATOM	373	CG1	ILE	22	0.142	5.777	-2.920	1.00	0.00
ATOM	374	HG12	ILE	22	-0.123	5.889	-3.972	1.00	0.00
ATOM	375	HG13	ILE	22	1.215	5.605	-2.832	1.00	0.00
ATOM	376	CD1	ILE	22	-0.613	4.590	-2.330	1.00	0.00
ATOM	377	HD11	ILE	22	-1.686	4.761	-2.418	1.00	0.00
ATOM	378	HD12	ILE	22	-0.345	3.682	-2.872	1.00	0.00

ATOM	379	HD13	ILE	22	-0.349	4.476	-1.279	1.00	0.00
ATOM	380	C	ILE	22	0.644	9.381	-1.586	1.00	0.00
ATOM	381	O	ILE	22	0.006	10.319	-2.040	1.00	0.00
ATOM	382	N	ARG	23	1.166	9.419	-0.354	1.00	0.00
ATOM	383	H	ARG	23	1.625	8.595	0.009	1.00	0.00
ATOM	384	CA	ARG	23	1.066	10.580	0.500	1.00	0.00
ATOM	385	HA	ARG	23	0.028	10.910	0.544	1.00	0.00
ATOM	386	CB	ARG	23	1.536	10.265	1.913	1.00	0.00
ATOM	387	HB2	ARG	23	2.313	9.502	1.864	1.00	0.00
ATOM	388	HB3	ARG	23	1.945	11.171	2.358	1.00	0.00
ATOM	389	CG	ARG	23	0.379	9.757	2.767	1.00	0.00
ATOM	390	HG2	ARG	23	0.531	10.082	3.796	1.00	0.00
ATOM	391	HG3	ARG	23	-0.551	10.179	2.387	1.00	0.00
ATOM	392	CD	ARG	23	0.302	8.236	2.722	1.00	0.00
ATOM	393	HD2	ARG	23	-0.620	7.921	2.233	1.00	0.00
ATOM	394	HD3	ARG	23	1.156	7.833	2.178	1.00	0.00
ATOM	395	NE	ARG	23	0.318	7.691	4.107	1.00	0.00
ATOM	396	HE	ARG	23	-0.466	7.101	4.348	1.00	0.00
ATOM	397	CZ	ARG	23	1.276	7.845	4.985	1.00	0.00
ATOM	398	NH1	ARG	23	2.303	8.598	4.683	1.00	0.00
ATOM	399	HH11	ARG	23	2.346	9.050	3.781	1.00	0.00
ATOM	400	HH12	ARG	23	3.048	8.723	5.353	1.00	0.00

ATOM	401	NH2	ARG	23	1.175	7.325	6.182	1.00	0.00
ATOM	402	HH21	ARG	23	0.350	6.799	6.433	1.00	0.00
ATOM	403	HH22	ARG	23	1.922	7.452	6.849	1.00	0.00
ATOM	404	C	ARG	23	1.881	11.750	-0.046	1.00	0.00
ATOM	405	O	ARG	23	1.667	12.897	0.347	1.00	0.00
ATOM	406	N	GLN	24	2.816	11.459	-0.944	1.00	0.00
ATOM	407	H	GLN	24	3.003	10.493	-1.173	1.00	0.00
ATOM	408	CA	GLN	24	3.622	12.476	-1.566	1.00	0.00
ATOM	409	HA	GLN	24	3.839	13.269	-0.850	1.00	0.00
ATOM	410	CB	GLN	24	4.942	11.879	-2.047	1.00	0.00
ATOM	411	HB2	GLN	24	4.816	10.805	-2.184	1.00	0.00
ATOM	412	HB3	GLN	24	5.212	12.337	-2.999	1.00	0.00
ATOM	413	CG	GLN	24	6.051	12.135	-1.026	1.00	0.00
ATOM	414	HG2	GLN	24	6.768	12.840	-1.446	1.00	0.00
ATOM	415	HG3	GLN	24	5.606	12.562	-0.128	1.00	0.00
ATOM	416	CD	GLN	24	6.785	10.866	-0.646	1.00	0.00
ATOM	417	OE1	GLN	24	6.810	9.897	-1.377	1.00	0.00
ATOM	418	NE2	GLN	24	7.401	10.868	0.515	1.00	0.00
ATOM	419	HE21	GLN	24	7.906	10.047	0.817	1.00	0.00
ATOM	420	HE22	GLN	24	7.367	11.690	1.100	1.00	0.00
ATOM	421	C	GLN	24	2.886	13.134	-2.720	1.00	0.00
ATOM	422	O	GLN	24	3.315	14.163	-3.229	1.00	0.00

ATOM	423	N	GLN	25	1.774	12.516	-3.146	1.00	0.00
ATOM	424	H	GLN	25	1.515	11.622	-2.754	1.00	0.00
ATOM	425	CA	GLN	25	0.964	13.057	-4.210	1.00	0.00
ATOM	426	HA	GLN	25	1.601	13.385	-5.031	1.00	0.00
ATOM	427	CB	GLN	25	-0.003	11.992	-4.736	1.00	0.00
ATOM	428	HB2	GLN	25	0.090	11.094	-4.126	1.00	0.00
ATOM	429	HB3	GLN	25	-1.022	12.372	-4.669	1.00	0.00
ATOM	430	CG	GLN	25	0.319	11.654	-6.191	1.00	0.00
ATOM	431	HG2	GLN	25	1.400	11.519	-6.213	1.00	0.00
ATOM	432	HG3	GLN	25	-0.167	10.739	-6.531	1.00	0.00
ATOM	433	CD	GLN	25	-0.049	12.781	-7.140	1.00	0.00
ATOM	434	OE1	GLN	25	-0.541	13.823	-6.787	1.00	0.00
ATOM	435	NE2	GLN	25	0.101	12.535	-8.424	1.00	0.00
ATOM	436	HE21	GLN	25	-0.128	13.248	-9.102	1.00	0.00
ATOM	437	HE22	GLN	25	0.445	11.634	-8.726	1.00	0.00
ATOM	438	C	GLN	25	0.212	14.299	-3.736	1.00	0.00
ATOM	439	O	GLN	25	-0.401	15.030	-4.502	1.00	0.00
ATOM	440	N	LEU	26	0.223	14.549	-2.430	1.00	0.00
ATOM	441	H	LEU	26	0.646	13.873	-1.810	1.00	0.00
ATOM	442	CA	LEU	26	-0.452	15.677	-1.850	1.00	0.00
ATOM	443	HA	LEU	26	-0.970	16.236	-2.629	1.00	0.00
ATOM	444	CB	LEU	26	-1.483	15.215	-0.809	1.00	0.00

ATOM	445	HB2	LEU	26	-0.968	15.315	0.146	1.00	0.00
ATOM	446	HB3	LEU	26	-2.333	15.896	-0.837	1.00	0.00
ATOM	447	CG	LEU	26	-1.979	13.771	-0.973	1.00	0.00
ATOM	448	HG	LEU	26	-1.864	13.471	-2.015	1.00	0.00
ATOM	449	CD1	LEU	26	-1.179	12.833	-0.086	1.00	0.00
ATOM	450	HD11	LEU	26	-1.294	13.131	0.956	1.00	0.00
ATOM	451	HD12	LEU	26	-1.543	11.813	-0.214	1.00	0.00
ATOM	452	HD13	LEU	26	-0.126	12.880	-0.363	1.00	0.00
ATOM	453	CD2	LEU	26	-3.469	13.670	-0.651	1.00	0.00
ATOM	454	HD21	LEU	26	-4.032	14.313	-1.327	1.00	0.00
ATOM	455	HD22	LEU	26	-3.799	12.638	-0.774	1.00	0.00
ATOM	456	HD23	LEU	26	-3.641	13.986	0.378	1.00	0.00
ATOM	457	C	LEU	26	0.581	16.647	-1.303	1.00	0.00
ATOM	458	O	LEU	26	1.775	16.523	-1.546	1.00	0.00
ATOM	459	N	ALA	27	0.108	17.612	-0.517	1.00	0.00
ATOM	460	H	ALA	27	-0.890	17.711	-0.394	1.00	0.00
ATOM	461	CA	ALA	27	0.979	18.588	0.110	1.00	0.00
ATOM	462	HA	ALA	27	1.875	18.739	-0.492	1.00	0.00
ATOM	463	CB	ALA	27	0.232	19.912	0.212	1.00	0.00
ATOM	464	HB1	ALA	27	-0.663	19.779	0.819	1.00	0.00
ATOM	465	HB2	ALA	27	0.877	20.659	0.675	1.00	0.00
ATOM	466	HB3	ALA	27	-0.053	20.247	-0.786	1.00	0.00

ATOM	467	C	ALA	27	1.455	18.074	1.466	1.00	0.00
ATOM	468	O	ALA	27	1.894	18.841	2.318	1.00	0.00
ATOM	469	N	GLY	28	1.311	16.761	1.699	1.00	0.00
ATOM	470	H	GLY	28	0.884	16.184	0.988	1.00	0.00
ATOM	471	CA	GLY	28	1.740	16.131	2.926	1.00	0.00
ATOM	472	HA2	GLY	28	1.532	16.802	3.759	1.00	0.00
ATOM	473	HA3	GLY	28	1.184	15.203	3.060	1.00	0.00
ATOM	474	C	GLY	28	3.228	15.818	2.902	1.00	0.00
ATOM	475	O	GLY	28	3.706	15.054	3.735	1.00	0.00
ATOM	476	N	SER	29	3.948	16.363	1.921	1.00	0.00
ATOM	477	H	SER	29	3.474	16.906	1.214	1.00	0.00
ATOM	478	CA	SER	29	5.370	16.163	1.790	1.00	0.00
ATOM	479	HA	SER	29	5.822	16.027	2.772	1.00	0.00
ATOM	480	CB	SER	29	5.643	14.917	0.951	1.00	0.00
ATOM	481	HB2	SER	29	5.393	14.050	1.563	1.00	0.00
ATOM	482	HB3	SER	29	4.980	14.954	0.087	1.00	0.00
ATOM	483	OG	SER	29	6.983	14.813	0.512	1.00	0.00
ATOM	484	HG	SER	29	7.088	14.012	-0.008	1.00	0.00
ATOM	485	C	SER	29	6.042	17.370	1.179	1.00	0.00
ATOM	486	O	SER	29	5.396	18.100	0.456	1.00	0.00
ATOM	487	N	PRO	30	7.301	17.614	1.545	1.00	0.00
ATOM	488	CD	PRO	30	8.084	16.803	2.461	1.00	0.00

ATOM	489	HD2	PRO	30	8.254	15.834	1.991	1.00	0.00
ATOM	490	HD3	PRO	30	7.501	16.667	3.372	1.00	0.00
ATOM	491	CG	PRO	30	9.379	17.569	2.708	1.00	0.00
ATOM	492	HG2	PRO	30	10.218	16.977	2.341	1.00	0.00
ATOM	493	HG3	PRO	30	9.494	17.737	3.779	1.00	0.00
ATOM	494	CB	PRO	30	9.260	18.897	1.956	1.00	0.00
ATOM	495	HB2	PRO	30	10.162	19.115	1.384	1.00	0.00
ATOM	496	HB3	PRO	30	9.059	19.718	2.644	1.00	0.00
ATOM	497	CA	PRO	30	8.078	18.710	1.010	1.00	0.00
ATOM	498	HA	PRO	30	7.467	19.611	1.059	1.00	0.00
ATOM	499	C	PRO	30	8.506	18.470	-0.417	1.00	0.00
ATOM	500	O	PRO	30	8.180	17.439	-0.994	1.00	0.00
ATOM	501	N	PHE	31	9.275	19.414	-0.971	1.00	0.00
ATOM	502	H	PHE	31	9.546	20.221	-0.428	1.00	0.00
ATOM	503	CA	PHE	31	9.746	19.316	-2.334	1.00	0.00
ATOM	504	HA	PHE	31	10.001	18.287	-2.588	1.00	0.00
ATOM	505	CB	PHE	31	8.616	19.782	-3.257	1.00	0.00
ATOM	506	HB2	PHE	31	8.548	20.868	-3.315	1.00	0.00
ATOM	507	HB3	PHE	31	8.842	19.380	-4.245	1.00	0.00
ATOM	508	CG	PHE	31	7.233	19.280	-2.891	1.00	0.00
ATOM	509	CD1	PHE	31	6.826	17.991	-3.270	1.00	0.00
ATOM	510	HD1	PHE	31	7.488	17.373	-3.878	1.00	0.00

ATOM	511	CE1	PHE	31	5.575	17.496	-2.871	1.00	0.00
ATOM	512	HE1	PHE	31	5.283	16.478	-3.130	1.00	0.00
ATOM	513	CZ	PHE	31	4.702	18.310	-2.140	1.00	0.00
ATOM	514	HZ	PHE	31	3.710	17.947	-1.869	1.00	0.00
ATOM	515	CE2	PHE	31	5.108	19.599	-1.755	1.00	0.00
ATOM	516	HE2	PHE	31	4.427	20.232	-1.186	1.00	0.00
ATOM	517	CD2	PHE	31	6.382	20.072	-2.100	1.00	0.00
ATOM	518	HD2	PHE	31	6.711	21.052	-1.755	1.00	0.00
ATOM	519	C	PHE	31	10.964	20.196	-2.554	1.00	0.00
ATOM	520	O	PHE	31	11.098	21.240	-1.912	1.00	0.00
ATOM	521	N	SER	32	11.844	19.769	-3.457	1.00	0.00
ATOM	522	H	SER	32	11.704	18.879	-3.915	1.00	0.00
ATOM	523	CA	SER	32	13.042	20.524	-3.773	1.00	0.00
ATOM	524	HA	SER	32	12.943	21.545	-3.406	1.00	0.00
ATOM	525	CB	SER	32	14.271	19.881	-3.112	1.00	0.00
ATOM	526	HB2	SER	32	13.958	18.950	-2.639	1.00	0.00
ATOM	527	HB3	SER	32	15.004	19.664	-3.889	1.00	0.00
ATOM	528	OG	SER	32	14.851	20.731	-2.142	1.00	0.00
ATOM	529	HG	SER	32	15.613	20.297	-1.752	1.00	0.00
ATOM	530	C	SER	32	13.223	20.604	-5.281	1.00	0.00
ATOM	531	O	SER	32	12.864	21.601	-5.907	1.00	0.00
ATOM	532	N	GLU	33	13.776	19.536	-5.865	1.00	0.00

ATOM	533	H	GLU	33	14.104	18.770	-5.295	1.00	0.00
ATOM	534	CA	GLU	33	14.009	19.478	-7.287	1.00	0.00
ATOM	535	HA	GLU	33	14.204	20.480	-7.669	1.00	0.00
ATOM	536	CB	GLU	33	15.219	18.589	-7.601	1.00	0.00
ATOM	537	HB2	GLU	33	15.490	18.042	-6.698	1.00	0.00
ATOM	538	HB3	GLU	33	14.936	17.883	-8.382	1.00	0.00
ATOM	539	CG	GLU	33	16.411	19.416	-8.071	1.00	0.00
ATOM	540	HG2	GLU	33	16.530	19.276	-9.146	1.00	0.00
ATOM	541	HG3	GLU	33	16.217	20.467	-7.861	1.00	0.00
ATOM	542	CD	GLU	33	17.689	18.982	-7.357	1.00	0.00
ATOM	543	OE1	GLU	33	18.070	17.802	-7.534	1.00	0.00
ATOM	544	OE2	GLU	33	18.260	19.835	-6.645	1.00	0.00
ATOM	545	C	GLU	33	12.753	19.007	-8.010	1.00	0.00
ATOM	546	O	GLU	33	11.670	18.913	-7.425	1.00	0.00
ATOM	547	N	ASN	34	12.905	18.682	-9.299	1.00	0.00
ATOM	548	H	ASN	34	13.803	18.807	-9.743	1.00	0.00
ATOM	549	CA	ASN	34	11.822	18.163	-10.087	1.00	0.00
ATOM	550	HA	ASN	34	10.872	18.556	-9.725	1.00	0.00
ATOM	551	CB	ASN	34	11.998	18.578	-11.546	1.00	0.00
ATOM	552	HB2	ASN	34	13.065	18.604	-11.769	1.00	0.00
ATOM	553	HB3	ASN	34	11.507	17.861	-12.204	1.00	0.00
ATOM	554	CG	ASN	34	11.416	19.950	-11.806	1.00	0.00

ATOM	555	OD1	ASN	34	10.348	20.305	-11.336	1.00	0.00
ATOM	556	ND2	ASN	34	12.132	20.726	-12.596	1.00	0.00
ATOM	557	HD21	ASN	34	11.806	21.656	-12.815	1.00	0.00
ATOM	558	HD22	ASN	34	13.003	20.386	-12.979	1.00	0.00
ATOM	559	C	ASN	34	11.779	16.653	-10.006	1.00	0.00
ATOM	560	O	ASN	34	12.825	16.007	-9.949	1.00	0.00
ATOM	561	N	GLN	35	10.579	16.089	-10.098	1.00	0.00
ATOM	562	H	GLN	35	9.761	16.680	-10.140	1.00	0.00
ATOM	563	CA	GLN	35	10.389	14.661	-10.080	1.00	0.00
ATOM	564	HA	GLN	35	10.891	14.215	-10.939	1.00	0.00
ATOM	565	CB	GLN	35	10.967	14.049	-8.804	1.00	0.00
ATOM	566	HB2	GLN	35	11.747	14.710	-8.428	1.00	0.00
ATOM	567	HB3	GLN	35	10.168	13.974	-8.066	1.00	0.00
ATOM	568	CG	GLN	35	11.554	12.665	-9.060	1.00	0.00
ATOM	569	HG2	GLN	35	11.789	12.182	-8.111	1.00	0.00
ATOM	570	HG3	GLN	35	10.816	12.069	-9.597	1.00	0.00
ATOM	571	CD	GLN	35	12.822	12.737	-9.896	1.00	0.00
ATOM	572	OE1	GLN	35	13.864	13.197	-9.473	1.00	0.00
ATOM	573	NE2	GLN	35	12.716	12.246	-11.120	1.00	0.00
ATOM	574	HE21	GLN	35	13.516	12.258	-11.737	1.00	0.00
ATOM	575	HE22	GLN	35	11.836	11.861	-11.432	1.00	0.00
ATOM	576	C	GLN	35	8.922	14.341	-10.213	1.00	0.00

ATOM	577	O	GLN	35	8.078	15.194	-9.934	1.00	0.00
ATOM	578	N	TRP	36	8.626	13.084	-10.592	1.00	0.00
ATOM	579	H	TRP	36	9.368	12.443	-10.835	1.00	0.00
ATOM	580	CA	TRP	36	7.251	12.629	-10.726	1.00	0.00
ATOM	581	HA	TRP	36	6.795	13.087	-11.604	1.00	0.00
ATOM	582	CB	TRP	36	7.200	11.106	-10.887	1.00	0.00
ATOM	583	HB2	TRP	36	7.484	10.821	-11.900	1.00	0.00
ATOM	584	HB3	TRP	36	7.891	10.654	-10.176	1.00	0.00
ATOM	585	CG	TRP	36	5.853	10.490	-10.628	1.00	0.00
ATOM	586	CD1	TRP	36	5.171	10.410	-9.452	1.00	0.00
ATOM	587	HD1	TRP	36	5.678	10.839	-8.588	1.00	0.00
ATOM	588	NE1	TRP	36	3.947	9.783	-9.613	1.00	0.00
ATOM	589	HE1	TRP	36	3.280	9.598	-8.878	1.00	0.00
ATOM	590	CE2	TRP	36	3.796	9.433	-10.934	1.00	0.00
ATOM	591	CZ2	TRP	36	2.768	8.778	-11.613	1.00	0.00
ATOM	592	HZ2	TRP	36	1.888	8.475	-11.046	1.00	0.00
ATOM	593	CH2	TRP	36	2.932	8.550	-12.988	1.00	0.00
ATOM	594	HH2	TRP	36	2.153	8.042	-13.557	1.00	0.00
ATOM	595	CZ3	TRP	36	4.098	8.971	-13.653	1.00	0.00
ATOM	596	HZ3	TRP	36	4.214	8.788	-14.722	1.00	0.00
ATOM	597	CE3	TRP	36	5.137	9.629	-12.982	1.00	0.00
ATOM	598	HE3	TRP	36	6.021	9.922	-13.548	1.00	0.00

ATOM	599	CD2	TRP	36	4.996	9.869	-11.601	1.00	0.00
ATOM	600	C	TRP	36	6.448	13.091	-9.502	1.00	0.00
ATOM	601	O	TRP	36	5.253	13.340	-9.594	1.00	0.00
ATOM	602	N	GLY	37	7.126	13.189	-8.346	1.00	0.00
ATOM	603	H	GLY	37	8.090	12.889	-8.311	1.00	0.00
ATOM	604	CA	GLY	37	6.530	13.707	-7.146	1.00	0.00
ATOM	605	HA2	GLY	37	6.086	14.679	-7.358	1.00	0.00
ATOM	606	HA3	GLY	37	5.754	13.020	-6.807	1.00	0.00
ATOM	607	C	GLY	37	7.563	13.865	-6.048	1.00	0.00
ATOM	608	O	GLY	37	8.105	14.950	-5.852	1.00	0.00
ATOM	609	N	PRO	38	7.862	12.756	-5.354	1.00	0.00
ATOM	610	CD	PRO	38	7.276	11.435	-5.548	1.00	0.00
ATOM	611	HD2	PRO	38	7.618	11.055	-6.511	1.00	0.00
ATOM	612	HD3	PRO	38	6.192	11.549	-5.567	1.00	0.00
ATOM	613	CG	PRO	38	7.751	10.577	-4.377	1.00	0.00
ATOM	614	HG2	PRO	38	8.361	9.758	-4.758	1.00	0.00
ATOM	615	HG3	PRO	38	6.884	10.171	-3.855	1.00	0.00
ATOM	616	CB	PRO	38	8.558	11.495	-3.462	1.00	0.00
ATOM	617	HB2	PRO	38	9.499	11.027	-3.172	1.00	0.00
ATOM	618	HB3	PRO	38	7.991	11.754	-2.568	1.00	0.00
ATOM	619	CA	PRO	38	8.834	12.744	-4.282	1.00	0.00
ATOM	620	HA	PRO	38	8.643	13.597	-3.631	1.00	0.00

ATOM	621	C	PRO	38	10.242	12.826	-4.817	1.00	0.00
ATOM	622	O	PRO	38	10.955	11.838	-4.865	1.00	0.00
ATOM	623	N	GLN	39	10.667	14.015	-5.234	1.00	0.00
ATOM	624	H	GLN	39	10.038	14.804	-5.218	1.00	0.00
ATOM	625	CA	GLN	39	11.994	14.208	-5.771	1.00	0.00
ATOM	626	HA	GLN	39	12.151	13.547	-6.624	1.00	0.00
ATOM	627	CB	GLN	39	12.168	15.659	-6.237	1.00	0.00
ATOM	628	HB2	GLN	39	11.347	15.924	-6.904	1.00	0.00
ATOM	629	HB3	GLN	39	12.160	16.319	-5.370	1.00	0.00
ATOM	630	CG	GLN	39	13.499	15.810	-6.981	1.00	0.00
ATOM	631	HG2	GLN	39	13.630	14.980	-7.676	1.00	0.00
ATOM	632	HG3	GLN	39	13.461	16.747	-7.537	1.00	0.00
ATOM	633	CD	GLN	39	14.690	15.850	-6.044	1.00	0.00
ATOM	634	OE1	GLN	39	14.580	16.233	-4.882	1.00	0.00
ATOM	635	NE2	GLN	39	15.828	15.424	-6.547	1.00	0.00
ATOM	636	HE21	GLN	39	16.661	15.424	-5.976	1.00	0.00
ATOM	637	HE22	GLN	39	15.864	15.098	-7.503	1.00	0.00
ATOM	638	C	GLN	39	13.042	13.842	-4.733	1.00	0.00
ATOM	639	O	GLN	39	14.104	13.325	-5.074	1.00	0.00
ATOM	640	N	GLN	40	12.721	14.090	-3.459	1.00	0.00
ATOM	641	H	GLN	40	11.853	14.556	-3.237	1.00	0.00
ATOM	642	CA	GLN	40	13.618	13.767	-2.380	1.00	0.00

ATOM	643	HA	GLN	40	14.653	13.864	-2.709	1.00	0.00
ATOM	644	CB	GLN	40	13.384	14.725	-1.213	1.00	0.00
ATOM	645	HB2	GLN	40	13.164	15.718	-1.605	1.00	0.00
ATOM	646	HB3	GLN	40	12.537	14.371	-0.626	1.00	0.00
ATOM	647	CG	GLN	40	14.631	14.792	-0.322	1.00	0.00
ATOM	648	HG2	GLN	40	15.458	14.374	-0.897	1.00	0.00
ATOM	649	HG3	GLN	40	14.862	15.822	-0.050	1.00	0.00
ATOM	650	CD	GLN	40	14.465	13.976	0.956	1.00	0.00
ATOM	651	OE1	GLN	40	13.389	13.530	1.335	1.00	0.00
ATOM	652	NE2	GLN	40	15.563	13.815	1.674	1.00	0.00
ATOM	653	HE21	GLN	40	15.528	13.285	2.533	1.00	0.00
ATOM	654	HE22	GLN	40	16.432	14.222	1.360	1.00	0.00
ATOM	655	C	GLN	40	13.445	12.315	-1.941	1.00	0.00
ATOM	656	O	GLN	40	14.168	11.822	-1.085	1.00	0.00
ATOM	657	N	GLY	41	12.464	11.622	-2.520	1.00	0.00
ATOM	658	H	GLY	41	11.873	12.095	-3.188	1.00	0.00
ATOM	659	CA	GLY	41	12.203	10.230	-2.241	1.00	0.00
ATOM	660	HA2	GLY	41	13.135	9.744	-1.953	1.00	0.00
ATOM	661	HA3	GLY	41	11.488	10.159	-1.422	1.00	0.00
ATOM	662	C	GLY	41	11.634	9.524	-3.454	1.00	0.00
ATOM	663	O	GLY	41	10.510	9.065	-3.449	1.00	0.00
ATOM	664	N	PRO	42	12.387	9.454	-4.551	1.00	0.00

ATOM	665	CD	PRO	42	13.728	9.983	-4.689	1.00	0.00
ATOM	666	HD2	PRO	42	14.386	9.406	-4.039	1.00	0.00
ATOM	667	HD3	PRO	42	13.715	11.022	-4.362	1.00	0.00
ATOM	668	CG	PRO	42	14.091	9.842	-6.164	1.00	0.00
ATOM	669	HG2	PRO	42	14.941	9.165	-6.258	1.00	0.00
ATOM	670	HG3	PRO	42	14.362	10.821	-6.558	1.00	0.00
ATOM	671	CB	PRO	42	12.854	9.288	-6.878	1.00	0.00
ATOM	672	HB2	PRO	42	13.123	8.458	-7.531	1.00	0.00
ATOM	673	HB3	PRO	42	12.349	10.059	-7.460	1.00	0.00
ATOM	674	CA	PRO	42	11.937	8.794	-5.763	1.00	0.00
ATOM	675	HA	PRO	42	10.924	9.132	-5.981	1.00	0.00
ATOM	676	C	PRO	42	11.946	7.288	-5.622	1.00	0.00
ATOM	677	O	PRO	42	11.107	6.597	-6.213	1.00	0.00
ATOM	678	N	SER	43	12.881	6.790	-4.820	1.00	0.00
ATOM	679	H	SER	43	13.543	7.421	-4.393	1.00	0.00
ATOM	680	CA	SER	43	13.029	5.375	-4.576	1.00	0.00
ATOM	681	HA	SER	43	12.926	4.832	-5.516	1.00	0.00
ATOM	682	CB	SER	43	14.401	5.056	-3.975	1.00	0.00
ATOM	683	HB2	SER	43	14.299	5.003	-2.891	1.00	0.00
ATOM	684	HB3	SER	43	14.728	4.088	-4.356	1.00	0.00
ATOM	685	OG	SER	43	15.362	6.047	-4.313	1.00	0.00
ATOM	686	HG	SER	43	16.208	5.818	-3.922	1.00	0.00

ATOM	687	C	SER	43	11.913	4.895	-3.655	1.00	0.00
ATOM	688	O	SER	43	12.122	4.584	-2.489	1.00	0.00
ATOM	689	N	LEU	44	10.686	4.850	-4.172	1.00	0.00
ATOM	690	H	LEU	44	10.543	5.158	-5.123	1.00	0.00
ATOM	691	CA	LEU	44	9.541	4.427	-3.414	1.00	0.00
ATOM	692	HA	LEU	44	9.865	3.963	-2.482	1.00	0.00
ATOM	693	CB	LEU	44	8.651	5.624	-3.081	1.00	0.00
ATOM	694	HB2	LEU	44	8.869	6.370	-3.845	1.00	0.00
ATOM	695	HB3	LEU	44	7.607	5.320	-3.158	1.00	0.00
ATOM	696	CG	LEU	44	8.909	6.226	-1.689	1.00	0.00
ATOM	697	HG	LEU	44	9.900	5.921	-1.354	1.00	0.00
ATOM	698	CD1	LEU	44	8.833	7.734	-1.729	1.00	0.00
ATOM	699	HD11	LEU	44	7.842	8.039	-2.064	1.00	0.00
ATOM	700	HD12	LEU	44	9.019	8.134	-0.733	1.00	0.00
ATOM	701	HD13	LEU	44	9.583	8.118	-2.420	1.00	0.00
ATOM	702	CD2	LEU	44	7.922	5.698	-0.670	1.00	0.00
ATOM	703	HD21	LEU	44	8.015	4.614	-0.601	1.00	0.00
ATOM	704	HD22	LEU	44	8.131	6.142	0.303	1.00	0.00
ATOM	705	HD23	LEU	44	6.909	5.956	-0.977	1.00	0.00
ATOM	706	C	LEU	44	8.813	3.400	-4.220	1.00	0.00
ATOM	707	O	LEU	44	8.399	2.432	-3.654	1.00	0.00
ATOM	708	N	ARG	45	8.649	3.572	-5.527	1.00	0.00

ATOM	709	H	ARG	45	9.018	4.413	-5.946	1.00	0.00
ATOM	710	CA	ARG	45	7.975	2.621	-6.380	1.00	0.00
ATOM	711	HA	ARG	45	6.962	2.452	-6.014	1.00	0.00
ATOM	712	CB	ARG	45	7.895	3.142	-7.811	1.00	0.00
ATOM	713	HB2	ARG	45	8.897	3.423	-8.136	1.00	0.00
ATOM	714	HB3	ARG	45	7.517	2.344	-8.451	1.00	0.00
ATOM	715	CG	ARG	45	6.972	4.351	-7.907	1.00	0.00
ATOM	716	HG2	ARG	45	7.481	5.209	-7.468	1.00	0.00
ATOM	717	HG3	ARG	45	6.771	4.548	-8.960	1.00	0.00
ATOM	718	CD	ARG	45	5.644	4.110	-7.168	1.00	0.00
ATOM	719	HD2	ARG	45	5.412	3.045	-7.169	1.00	0.00
ATOM	720	HD3	ARG	45	5.727	4.462	-6.140	1.00	0.00
ATOM	721	NE	ARG	45	4.545	4.832	-7.826	1.00	0.00
ATOM	722	HE	ARG	45	3.806	4.269	-8.223	1.00	0.00
ATOM	723	CZ	ARG	45	4.443	6.146	-7.898	1.00	0.00
ATOM	724	NH1	ARG	45	5.348	6.920	-7.336	1.00	0.00
ATOM	725	HH11	ARG	45	6.128	6.506	-6.845	1.00	0.00
ATOM	726	HH12	ARG	45	5.258	7.924	-7.399	1.00	0.00
ATOM	727	NH2	ARG	45	3.455	6.693	-8.567	1.00	0.00
ATOM	728	HH21	ARG	45	2.774	6.105	-9.026	1.00	0.00
ATOM	729	HH22	ARG	45	3.381	7.699	-8.620	1.00	0.00
ATOM	730	C	ARG	45	8.708	1.322	-6.399	1.00	0.00

ATOM	731	O	ARG	45	8.231	0.372	-5.852	1.00	0.00
ATOM	732	N	GLU	46	9.909	1.285	-6.939	1.00	0.00
ATOM	733	H	GLU	46	10.287	2.125	-7.352	1.00	0.00
ATOM	734	CA	GLU	46	10.704	0.069	-7.000	1.00	0.00
ATOM	735	HA	GLU	46	10.190	-0.681	-7.601	1.00	0.00
ATOM	736	CB	GLU	46	12.068	0.358	-7.638	1.00	0.00
ATOM	737	HB2	GLU	46	12.534	1.190	-7.111	1.00	0.00
ATOM	738	HB3	GLU	46	12.696	-0.528	-7.543	1.00	0.00
ATOM	739	CG	GLU	46	11.910	0.716	-9.121	1.00	0.00
ATOM	740	HG2	GLU	46	11.672	-0.201	-9.661	1.00	0.00
ATOM	741	HG3	GLU	46	11.096	1.430	-9.244	1.00	0.00
ATOM	742	CD	GLU	46	13.201	1.314	-9.677	1.00	0.00
ATOM	743	OE1	GLU	46	13.703	2.274	-9.047	1.00	0.00
ATOM	744	OE2	GLU	46	13.653	0.823	-10.731	1.00	0.00
ATOM	745	C	GLU	46	10.897	-0.518	-5.602	1.00	0.00
ATOM	746	O	GLU	46	11.176	-1.705	-5.461	1.00	0.00
ATOM	747	N	GLN	47	10.712	0.318	-4.568	1.00	0.00
ATOM	748	H	GLN	47	10.533	1.294	-4.753	1.00	0.00
ATOM	749	CA	GLN	47	10.811	-0.106	-3.208	1.00	0.00
ATOM	750	HA	GLN	47	11.577	-0.876	-3.114	1.00	0.00
ATOM	751	CB	GLN	47	11.189	1.068	-2.315	1.00	0.00
ATOM	752	HB2	GLN	47	11.142	1.988	-2.897	1.00	0.00

```
ATOM    753  HB3 GLN    47      10.484   1.126  -1.486  1.00  0.00
ATOM    754  CG  GLN    47      12.609   0.883  -1.766  1.00  0.00
ATOM    755  HG2 GLN    47      12.687  -0.099  -1.298  1.00  0.00
ATOM    756  HG3 GLN    47      13.339   0.959  -2.572  1.00  0.00
ATOM    757  CD  GLN    47      12.924   1.945  -0.723  1.00  0.00
ATOM    758  OE1 GLN    47      13.883   2.694  -0.824  1.00  0.00
ATOM    759  NE2 GLN    47      12.098   1.996   0.311  1.00  0.00
ATOM    760 HE21 GLN    47      12.249   2.677   1.041  1.00  0.00
ATOM    761 HE22 GLN    47      11.321   1.353   0.365  1.00  0.00
ATOM    762  C   GLN    47       9.523  -0.719  -2.752  1.00  0.00
ATOM    763  O   GLN    47       9.573  -1.817  -2.286  1.00  0.00
ATOM    764  N   CYX    48       8.388  -0.063  -2.889  1.00  0.00
ATOM    765  H   CYX    48       8.477   0.865  -3.277  1.00  0.00
ATOM    766  CA  CYX    48       7.088  -0.517  -2.512  1.00  0.00
ATOM    767  HA  CYX    48       7.128  -0.878  -1.484  1.00  0.00
ATOM    768  CB  CYX    48       6.036   0.578  -2.602  1.00  0.00
ATOM    769  HB2 CYX    48       5.733   0.874  -1.598  1.00  0.00
ATOM    770  HB3 CYX    48       6.451   1.439  -3.125  1.00  0.00
ATOM    771  SG  CYX    48       4.501   0.148  -3.459  1.00  0.00
ATOM    772  C   CYX    48       6.712  -1.662  -3.394  1.00  0.00
ATOM    773  O   CYX    48       6.254  -2.667  -2.926  1.00  0.00
ATOM    774  N   CYX    49       6.941  -1.575  -4.696  1.00  0.00
```

ATOM	775	H	CYX	49	7.301	-0.693	-5.030	1.00	0.00
ATOM	776	CA	CYX	49	6.671	-2.610	-5.659	1.00	0.00
ATOM	777	HA	CYX	49	5.593	-2.749	-5.737	1.00	0.00
ATOM	778	CB	CYX	49	7.223	-2.266	-7.049	1.00	0.00
ATOM	779	HB2	CYX	49	8.304	-2.139	-6.990	1.00	0.00
ATOM	780	HB3	CYX	49	6.991	-3.074	-7.743	1.00	0.00
ATOM	781	SG	CYX	49	6.602	-0.735	-7.802	1.00	0.00
ATOM	782	C	CYX	49	7.277	-3.910	-5.140	1.00	0.00
ATOM	783	O	CYX	49	6.827	-4.984	-5.510	1.00	0.00
ATOM	784	N	ASN	50	8.309	-3.814	-4.289	1.00	0.00
ATOM	785	H	ASN	50	8.681	-2.898	-4.084	1.00	0.00
ATOM	786	CA	ASN	50	8.897	-4.943	-3.644	1.00	0.00
ATOM	787	HA	ASN	50	8.632	-5.862	-4.167	1.00	0.00
ATOM	788	CB	ASN	50	10.413	-4.794	-3.660	1.00	0.00
ATOM	789	HB2	ASN	50	10.695	-3.777	-3.933	1.00	0.00
ATOM	790	HB3	ASN	50	10.797	-5.023	-2.666	1.00	0.00
ATOM	791	CG	ASN	50	11.037	-5.750	-4.650	1.00	0.00
ATOM	792	OD1	ASN	50	11.605	-6.774	-4.302	1.00	0.00
ATOM	793	ND2	ASN	50	10.905	-5.414	-5.920	1.00	0.00
ATOM	794	HD21	ASN	50	11.297	-6.006	-6.639	1.00	0.00
ATOM	795	HD22	ASN	50	10.413	-4.568	-6.167	1.00	0.00
ATOM	796	C	ASN	50	8.456	-5.045	-2.215	1.00	0.00

ATOM	797	O	ASN	50	8.218	-6.124	-1.763	1.00	0.00
ATOM	798	N	GLU	51	8.343	-3.956	-1.486	1.00	0.00
ATOM	799	H	GLU	51	8.639	-3.100	-1.931	1.00	0.00
ATOM	800	CA	GLU	51	7.927	-3.901	-0.113	1.00	0.00
ATOM	801	HA	GLU	51	8.509	-4.618	0.466	1.00	0.00
ATOM	802	CB	GLU	51	8.131	-2.505	0.487	1.00	0.00
ATOM	803	HB2	GLU	51	8.704	-2.605	1.409	1.00	0.00
ATOM	804	HB3	GLU	51	8.692	-1.901	-0.226	1.00	0.00
ATOM	805	CG	GLU	51	6.796	-1.828	0.789	1.00	0.00
ATOM	806	HG2	GLU	51	6.253	-1.840	-0.157	1.00	0.00
ATOM	807	HG3	GLU	51	6.215	-2.353	1.547	1.00	0.00
ATOM	808	CD	GLU	51	7.013	-0.424	1.217	1.00	0.00
ATOM	809	OE1	GLU	51	7.278	-0.124	2.398	1.00	0.00
ATOM	810	OE2	GLU	51	6.819	0.416	0.346	1.00	0.00
ATOM	811	C	GLU	51	6.474	-4.359	0.006	1.00	0.00
ATOM	812	O	GLU	51	5.977	-4.586	1.099	1.00	0.00
ATOM	813	N	LEU	52	5.780	-4.484	-1.119	1.00	0.00
ATOM	814	H	LEU	52	6.215	-4.170	-1.975	1.00	0.00
ATOM	815	CA	LEU	52	4.412	-4.896	-1.165	1.00	0.00
ATOM	816	HA	LEU	52	3.988	-4.904	-0.161	1.00	0.00
ATOM	817	CB	LEU	52	3.604	-3.918	-2.031	1.00	0.00
ATOM	818	HB2	LEU	52	2.724	-3.748	-1.411	1.00	0.00

ATOM	819	HB3	LEU	52	4.150	-2.982	-2.150	1.00	0.00
ATOM	820	CG	LEU	52	3.168	-4.433	-3.407	1.00	0.00
ATOM	821	HG	LEU	52	2.801	-3.584	-3.984	1.00	0.00
ATOM	822	CD1	LEU	52	4.297	-5.100	-4.156	1.00	0.00
ATOM	823	HD11	LEU	52	4.664	-5.950	-3.580	1.00	0.00
ATOM	824	HD12	LEU	52	3.936	-5.447	-5.124	1.00	0.00
ATOM	825	HD13	LEU	52	5.107	-4.386	-4.305	1.00	0.00
ATOM	826	CD2	LEU	52	1.968	-5.346	-3.283	1.00	0.00
ATOM	827	HD21	LEU	52	1.139	-4.799	-2.834	1.00	0.00
ATOM	828	HD22	LEU	52	1.676	-5.700	-4.272	1.00	0.00
ATOM	829	HD23	LEU	52	2.223	-6.199	-2.654	1.00	0.00
ATOM	830	C	LEU	52	4.361	-6.352	-1.597	1.00	0.00
ATOM	831	O	LEU	52	3.367	-7.012	-1.351	1.00	0.00
ATOM	832	N	TYR	53	5.420	-6.833	-2.259	1.00	0.00
ATOM	833	H	TYR	53	6.237	-6.246	-2.340	1.00	0.00
ATOM	834	CA	TYR	53	5.470	-8.146	-2.824	1.00	0.00
ATOM	835	HA	TYR	53	4.465	-8.539	-2.977	1.00	0.00
ATOM	836	CB	TYR	53	6.175	-8.067	-4.178	1.00	0.00
ATOM	837	HB2	TYR	53	5.700	-7.261	-4.737	1.00	0.00
ATOM	838	HB3	TYR	53	7.216	-7.810	-3.984	1.00	0.00
ATOM	839	CG	TYR	53	6.113	-9.346	-4.988	1.00	0.00
ATOM	840	CD1	TYR	53	4.874	-9.889	-5.359	1.00	0.00

ATOM	841	HD1	TYR	53	3.961	-9.378	-5.054	1.00	0.00
ATOM	842	CE1	TYR	53	4.798	-11.071	-6.110	1.00	0.00
ATOM	843	HE1	TYR	53	3.834	-11.481	-6.412	1.00	0.00
ATOM	844	CZ	TYR	53	5.988	-11.722	-6.485	1.00	0.00
ATOM	845	OH	TYR	53	5.931	-12.878	-7.195	1.00	0.00
ATOM	846	HH	TYR	53	5.032	-13.158	-7.385	1.00	0.00
ATOM	847	CE2	TYR	53	7.240	-11.189	-6.117	1.00	0.00
ATOM	848	HE2	TYR	53	8.161	-11.695	-6.408	1.00	0.00
ATOM	849	CD2	TYR	53	7.297	-9.997	-5.369	1.00	0.00
ATOM	850	HD2	TYR	53	8.262	-9.578	-5.085	1.00	0.00
ATOM	851	C	TYR	53	6.268	-9.026	-1.915	1.00	0.00
ATOM	852	O	TYR	53	6.055	-10.218	-1.877	1.00	0.00
ATOM	853	N	GLN	54	7.224	-8.463	-1.188	1.00	0.00
ATOM	854	H	GLN	54	7.374	-7.473	-1.322	1.00	0.00
ATOM	855	CA	GLN	54	8.078	-9.163	-0.270	1.00	0.00
ATOM	856	HA	GLN	54	8.704	-9.865	-0.821	1.00	0.00
ATOM	857	CB	GLN	54	8.986	-8.193	0.496	1.00	0.00
ATOM	858	HB2	GLN	54	9.543	-8.761	1.241	1.00	0.00
ATOM	859	HB3	GLN	54	9.682	-7.743	-0.212	1.00	0.00
ATOM	860	CG	GLN	54	8.181	-7.093	1.190	1.00	0.00
ATOM	861	HG2	GLN	54	7.701	-6.444	0.457	1.00	0.00
ATOM	862	HG3	GLN	54	7.418	-7.581	1.797	1.00	0.00

ATOM	863	CD	GLN	54	9.048	-6.244	2.094	1.00	0.00
ATOM	864	OE1	GLN	54	10.214	-5.975	1.820	1.00	0.00
ATOM	865	NE2	GLN	54	8.463	-5.774	3.173	1.00	0.00
ATOM	866	HE21	GLN	54	8.987	-5.200	3.818	1.00	0.00
ATOM	867	HE22	GLN	54	7.492	-5.989	3.352	1.00	0.00
ATOM	868	C	GLN	54	7.228	-9.968	0.701	1.00	0.00
ATOM	869	O	GLN	54	7.675	-10.994	1.198	1.00	0.00
ATOM	870	N	GLU	55	5.993	-9.510	0.936	1.00	0.00
ATOM	871	H	GLU	55	5.722	-8.614	0.556	1.00	0.00
ATOM	872	CA	GLU	55	5.065	-10.196	1.789	1.00	0.00
ATOM	873	HA	GLU	55	5.603	-10.712	2.584	1.00	0.00
ATOM	874	CB	GLU	55	4.101	-9.208	2.418	1.00	0.00
ATOM	875	HB2	GLU	55	3.403	-8.873	1.650	1.00	0.00
ATOM	876	HB3	GLU	55	3.552	-9.720	3.208	1.00	0.00
ATOM	877	CG	GLU	55	4.828	-8.006	3.002	1.00	0.00
ATOM	878	HG2	GLU	55	5.759	-8.401	3.407	1.00	0.00
ATOM	879	HG3	GLU	55	5.052	-7.257	2.243	1.00	0.00
ATOM	880	CD	GLU	55	4.024	-7.373	4.113	1.00	0.00
ATOM	881	OE1	GLU	55	3.084	-7.996	4.640	1.00	0.00
ATOM	882	OE2	GLU	55	4.338	-6.208	4.359	1.00	0.00
ATOM	883	C	GLU	55	4.338	-11.291	1.040	1.00	0.00
ATOM	884	O	GLU	55	3.302	-11.751	1.478	1.00	0.00

ATOM	885	N	ASP	56	4.871	-11.739	-0.086	1.00	0.00
ATOM	886	H	ASP	56	5.760	-11.342	-0.354	1.00	0.00
ATOM	887	CA	ASP	56	4.284	-12.729	-0.941	1.00	0.00
ATOM	888	HA	ASP	56	5.035	-13.148	-1.611	1.00	0.00
ATOM	889	CB	ASP	56	3.709	-13.861	-0.082	1.00	0.00
ATOM	890	HB2	ASP	56	4.399	-13.893	0.761	1.00	0.00
ATOM	891	HB3	ASP	56	2.711	-13.606	0.274	1.00	0.00
ATOM	892	CG	ASP	56	3.672	-15.213	-0.753	1.00	0.00
ATOM	893	OD1	ASP	56	4.106	-15.300	-1.922	1.00	0.00
ATOM	894	OD2	ASP	56	3.117	-16.144	-0.135	1.00	0.00
ATOM	895	C	ASP	56	3.170	-12.110	-1.773	1.00	0.00
ATOM	896	O	ASP	56	2.944	-10.893	-1.800	1.00	0.00
ATOM	897	N	GLN	57	2.406	-12.969	-2.459	1.00	0.00
ATOM	898	H	GLN	57	2.636	-13.953	-2.451	1.00	0.00
ATOM	899	CA	GLN	57	1.290	-12.535	-3.261	1.00	0.00
ATOM	900	HA	GLN	57	1.460	-11.520	-3.620	1.00	0.00
ATOM	901	CB	GLN	57	1.123	-13.459	-4.468	1.00	0.00
ATOM	902	HB2	GLN	57	2.043	-14.025	-4.613	1.00	0.00
ATOM	903	HB3	GLN	57	0.300	-14.148	-4.278	1.00	0.00
ATOM	904	CG	GLN	57	0.824	-12.644	-5.725	1.00	0.00
ATOM	905	HG2	GLN	57	1.433	-11.740	-5.708	1.00	0.00
ATOM	906	HG3	GLN	57	1.055	-13.221	-6.621	1.00	0.00

ATOM	907	CD	GLN	57	-0.630	-12.245	-5.771	1.00	0.00
ATOM	908	OE1	GLN	57	-1.521	-13.043	-5.915	1.00	0.00
ATOM	909	NE2	GLN	57	-0.898	-10.972	-5.671	1.00	0.00
ATOM	910	HE21	GLN	57	-1.858	-10.658	-5.697	1.00	0.00
ATOM	911	HE22	GLN	57	-0.145	-10.307	-5.568	1.00	0.00
ATOM	912	C	GLN	57	0.014	-12.494	-2.447	1.00	0.00
ATOM	913	O	GLN	57	-0.917	-11.752	-2.740	1.00	0.00
ATOM	914	N	VAL	58	-0.026	-13.270	-1.372	1.00	0.00
ATOM	915	H	VAL	58	0.752	-13.890	-1.198	1.00	0.00
ATOM	916	CA	VAL	58	-1.170	-13.337	-0.483	1.00	0.00
ATOM	917	HA	VAL	58	-2.108	-13.327	-1.038	1.00	0.00
ATOM	918	CB	VAL	58	-1.118	-14.612	0.368	1.00	0.00
ATOM	919	HB	VAL	58	-0.779	-14.358	1.372	1.00	0.00
ATOM	920	CG1	VAL	58	-2.507	-15.253	0.445	1.00	0.00
ATOM	921	HG11	VAL	58	-2.846	-15.508	-0.559	1.00	0.00
ATOM	922	HG12	VAL	58	-2.457	-16.158	1.052	1.00	0.00
ATOM	923	HG13	VAL	58	-3.207	-14.551	0.897	1.00	0.00
ATOM	924	CG2	VAL	58	-0.111	-15.621	-0.194	1.00	0.00
ATOM	925	HG21	VAL	58	0.883	-15.175	-0.207	1.00	0.00
ATOM	926	HG22	VAL	58	-0.100	-16.513	0.433	1.00	0.00
ATOM	927	HG23	VAL	58	-0.399	-15.896	-1.209	1.00	0.00
ATOM	928	C	VAL	58	-1.215	-12.077	0.360	1.00	0.00

ATOM	929	O	VAL	58	-2.158	-11.902	1.129	1.00	0.00
ATOM	930	N	CYX	59	-0.230	-11.191	0.184	1.00	0.00
ATOM	931	H	CYX	59	0.509	-11.413	-0.467	1.00	0.00
ATOM	932	CA	CYX	59	-0.155	-9.954	0.900	1.00	0.00
ATOM	933	HA	CYX	59	-0.985	-9.887	1.603	1.00	0.00
ATOM	934	CB	CYX	59	1.135	-9.858	1.669	1.00	0.00
ATOM	935	HB2	CYX	59	1.955	-10.222	1.050	1.00	0.00
ATOM	936	HB3	CYX	59	1.320	-8.819	1.942	1.00	0.00
ATOM	937	SG	CYX	59	1.211	-10.832	3.216	1.00	0.00
ATOM	938	C	CYX	59	-0.266	-8.804	-0.070	1.00	0.00
ATOM	939	O	CYX	59	-0.851	-7.767	0.258	1.00	0.00
ATOM	940	N	VAL	60	0.177	-9.015	-1.318	1.00	0.00
ATOM	941	H	VAL	60	0.645	-9.884	-1.532	1.00	0.00
ATOM	942	CA	VAL	60	0.075	-8.018	-2.359	1.00	0.00
ATOM	943	HA	VAL	60	0.691	-7.137	-2.183	1.00	0.00
ATOM	944	CB	VAL	60	0.466	-8.625	-3.701	1.00	0.00
ATOM	945	HB	VAL	60	0.113	-9.656	-3.736	1.00	0.00
ATOM	946	CG1	VAL	60	-0.147	-7.839	-4.855	1.00	0.00
ATOM	947	HG11	VAL	60	0.206	-6.809	-4.822	1.00	0.00
ATOM	948	HG12	VAL	60	0.147	-8.293	-5.801	1.00	0.00
ATOM	949	HG13	VAL	60	-1.234	-7.854	-4.768	1.00	0.00
ATOM	950	CG2	VAL	60	1.970	-8.722	-3.837	1.00	0.00

ATOM 951 HG21 VAL 60 2.367 -9.352 -3.041 1.00 0.00

ATOM 952 HG22 VAL 60 2.220 -9.159 -4.804 1.00 0.00

ATOM 953 HG23 VAL 60 2.407 -7.726 -3.765 1.00 0.00

ATOM 954 C VAL 60 -1.325 -7.449 -2.389 1.00 0.00

ATOM 955 O VAL 60 -1.523 -6.324 -2.814 1.00 0.00

ATOM 956 N CYX 61 -2.317 -8.224 -1.958 1.00 0.00

ATOM 957 H CYX 61 -2.098 -9.188 -1.749 1.00 0.00

ATOM 958 CA CYX 61 -3.664 -7.802 -1.847 1.00 0.00

ATOM 959 HA CYX 61 -3.767 -7.076 -1.041 1.00 0.00

ATOM 960 CB CYX 61 -4.101 -7.148 -3.150 1.00 0.00

ATOM 961 HB2 CYX 61 -3.336 -7.305 -3.910 1.00 0.00

ATOM 962 HB3 CYX 61 -5.039 -7.592 -3.483 1.00 0.00

ATOM 963 SG CYX 61 -4.373 -5.358 -3.094 1.00 0.00

ATOM 964 C CYX 61 -4.527 -9.000 -1.471 1.00 0.00

ATOM 965 O CYX 61 -4.075 -10.123 -1.571 1.00 0.00

ATOM 966 N PRO 62 -5.776 -8.744 -1.048 1.00 0.00

ATOM 967 CD PRO 62 -6.785 -9.697 -0.631 1.00 0.00

ATOM 968 HD2 PRO 62 -6.431 -10.195 0.272 1.00 0.00

ATOM 969 HD3 PRO 62 -6.909 -10.433 -1.426 1.00 0.00

ATOM 970 CG PRO 62 -8.059 -8.875 -0.390 1.00 0.00

ATOM 971 HG2 PRO 62 -8.343 -8.969 0.658 1.00 0.00

ATOM 972 HG3 PRO 62 -8.856 -9.272 -1.019 1.00 0.00

ATOM	973	CB	PRO	62	-7.746	-7.423	-0.756	1.00	0.00
ATOM	974	HB2	PRO	62	-8.092	-6.713	-0.006	1.00	0.00
ATOM	975	HB3	PRO	62	-8.182	-7.171	-1.723	1.00	0.00
ATOM	976	CA	PRO	62	-6.235	-7.383	-0.844	1.00	0.00
ATOM	977	HA	PRO	62	-6.000	-6.810	-1.741	1.00	0.00
ATOM	978	C	PRO	62	-5.595	-6.739	0.340	1.00	0.00
ATOM	979	O	PRO	62	-5.882	-5.620	0.634	1.00	0.00
ATOM	980	N	THR	63	-4.763	-7.451	1.055	1.00	0.00
ATOM	981	H	THR	63	-4.670	-8.419	0.781	1.00	0.00
ATOM	982	CA	THR	63	-4.028	-6.994	2.208	1.00	0.00
ATOM	983	HA	THR	63	-4.731	-7.087	3.036	1.00	0.00
ATOM	984	CB	THR	63	-2.848	-7.926	2.464	1.00	0.00
ATOM	985	HB	THR	63	-1.939	-7.418	2.142	1.00	0.00
ATOM	986	CG2	THR	63	-2.724	-8.304	3.916	1.00	0.00
ATOM	987	HG21	THR	63	-3.632	-8.813	4.238	1.00	0.00
ATOM	988	HG22	THR	63	-1.870	-8.968	4.047	1.00	0.00
ATOM	989	HG23	THR	63	-2.580	-7.405	4.515	1.00	0.00
ATOM	990	OG1	THR	63	-3.163	-9.149	1.811	1.00	0.00
ATOM	991	HG1	THR	63	-2.447	-9.776	1.942	1.00	0.00
ATOM	992	C	THR	63	-3.558	-5.586	2.035	1.00	0.00
ATOM	993	O	THR	63	-3.873	-4.809	2.912	1.00	0.00
ATOM	994	N	LEU	64	-2.957	-5.237	0.898	1.00	0.00

ATOM 995 H LEU 64 -2.771 -5.962 0.219 1.00 0.00
ATOM 996 CA LEU 64 -2.517 -3.888 0.602 1.00 0.00
ATOM 997 HA LEU 64 -1.649 -3.634 1.210 1.00 0.00
ATOM 998 CB LEU 64 -2.128 -3.783 -0.871 1.00 0.00
ATOM 999 HB2 LEU 64 -1.947 -4.812 -1.181 1.00 0.00
ATOM 1000 HB3 LEU 64 -2.963 -3.375 -1.440 1.00 0.00
ATOM 1001 CG LEU 64 -0.872 -2.942 -1.132 1.00 0.00
ATOM 1002 HG LEU 64 -0.230 -2.982 -0.252 1.00 0.00
ATOM 1003 CD1 LEU 64 -0.118 -3.472 -2.330 1.00 0.00
ATOM 1004 HD11 LEU 64 -0.759 -3.432 -3.211 1.00 0.00
ATOM 1005 HD12 LEU 64 0.770 -2.863 -2.500 1.00 0.00
ATOM 1006 HD13 LEU 64 0.180 -4.504 -2.145 1.00 0.00
ATOM 1007 CD2 LEU 64 -1.226 -1.471 -1.330 1.00 0.00
ATOM 1008 HD21 LEU 64 -1.719 -1.092 -0.435 1.00 0.00
ATOM 1009 HD22 LEU 64 -0.316 -0.900 -1.513 1.00 0.00
ATOM 1010 HD23 LEU 64 -1.896 -1.370 -2.184 1.00 0.00
ATOM 1011 C LEU 64 -3.592 -2.879 0.930 1.00 0.00
ATOM 1012 O LEU 64 -3.278 -1.861 1.535 1.00 0.00
ATOM 1013 N LYS 65 -4.863 -3.209 0.640 1.00 0.00
ATOM 1014 H LYS 65 -5.006 -4.030 0.068 1.00 0.00
ATOM 1015 CA LYS 65 -6.013 -2.416 0.996 1.00 0.00
ATOM 1016 HA LYS 65 -6.069 -1.547 0.340 1.00 0.00

ATOM	1017	CB	LYS	65	-7.314	-3.219	0.857	1.00	0.00
ATOM	1018	HB2	LYS	65	-7.428	-3.525	-0.183	1.00	0.00
ATOM	1019	HB3	LYS	65	-7.249	-4.104	1.490	1.00	0.00
ATOM	1020	CG	LYS	65	-8.517	-2.383	1.275	1.00	0.00
ATOM	1021	HG2	LYS	65	-8.254	-1.329	1.188	1.00	0.00
ATOM	1022	HG3	LYS	65	-9.346	-2.607	0.603	1.00	0.00
ATOM	1023	CD	LYS	65	-8.934	-2.688	2.720	1.00	0.00
ATOM	1024	HD2	LYS	65	-8.119	-3.208	3.223	1.00	0.00
ATOM	1025	HD3	LYS	65	-9.140	-1.751	3.236	1.00	0.00
ATOM	1026	CE	LYS	65	-10.186	-3.566	2.739	1.00	0.00
ATOM	1027	HE2	LYS	65	-10.748	-3.405	1.819	1.00	0.00
ATOM	1028	HE3	LYS	65	-9.887	-4.612	2.802	1.00	0.00
ATOM	1029	NZ	LYS	65	-11.033	-3.226	3.899	1.00	0.00
ATOM	1030	HZ1	LYS	65	-11.311	-2.257	3.840	1.00	0.00
ATOM	1031	HZ2	LYS	65	-11.855	-3.813	3.899	1.00	0.00
ATOM	1032	HZ3	LYS	65	-10.512	-3.376	4.751	1.00	0.00
ATOM	1033	C	LYS	65	-5.832	-1.845	2.384	1.00	0.00
ATOM	1034	O	LYS	65	-6.280	-0.767	2.623	1.00	0.00
ATOM	1035	N	GLN	66	-5.200	-2.541	3.308	1.00	0.00
ATOM	1036	H	GLN	66	-5.012	-3.503	3.064	1.00	0.00
ATOM	1037	CA	GLN	66	-4.846	-2.096	4.605	1.00	0.00
ATOM	1038	HA	GLN	66	-5.733	-1.720	5.115	1.00	0.00

ATOM	1039	CB	GLN	66	-4.250	-3.236	5.437	1.00	0.00
ATOM	1040	HB2	GLN	66	-4.957	-4.065	5.469	1.00	0.00
ATOM	1041	HB3	GLN	66	-3.320	-3.570	4.976	1.00	0.00
ATOM	1042	CG	GLN	66	-3.966	-2.748	6.870	1.00	0.00
ATOM	1043	HG2	GLN	66	-3.688	-3.557	7.546	1.00	0.00
ATOM	1044	HG3	GLN	66	-3.138	-2.044	6.787	1.00	0.00
ATOM	1045	CD	GLN	66	-5.171	-2.017	7.454	1.00	0.00
ATOM	1046	OE1	GLN	66	-5.058	-0.984	8.090	1.00	0.00
ATOM	1047	NE2	GLN	66	-6.359	-2.531	7.148	1.00	0.00
ATOM	1048	HE21	GLN	66	-7.200	-2.097	7.501	1.00	0.00
ATOM	1049	HE22	GLN	66	-6.415	-3.353	6.564	1.00	0.00
ATOM	1050	C	GLN	66	-3.900	-0.922	4.587	1.00	0.00
ATOM	1051	O	GLN	66	-4.291	0.181	4.948	1.00	0.00
ATOM	1052	N	ALA	67	-2.645	-1.145	4.203	1.00	0.00
ATOM	1053	H	ALA	67	-2.346	-2.092	4.016	1.00	0.00
ATOM	1054	CA	ALA	67	-1.669	-0.086	4.101	1.00	0.00
ATOM	1055	HA	ALA	67	-1.450	0.325	5.086	1.00	0.00
ATOM	1056	CB	ALA	67	-0.381	-0.646	3.510	1.00	0.00
ATOM	1057	HB1	ALA	67	-0.582	-1.053	2.519	1.00	0.00
ATOM	1058	HB2	ALA	67	0.360	0.150	3.432	1.00	0.00
ATOM	1059	HB3	ALA	67	0.002	-1.436	4.156	1.00	0.00
ATOM	1060	C	ALA	67	-2.222	1.058	3.258	1.00	0.00

ATOM	1061	O	ALA	67	-1.844	2.200	3.454	1.00	0.00
ATOM	1062	N	ALA	68	-3.199	0.783	2.401	1.00	0.00
ATOM	1063	H	ALA	68	-3.474	-0.182	2.286	1.00	0.00
ATOM	1064	CA	ALA	68	-3.843	1.774	1.592	1.00	0.00
ATOM	1065	HA	ALA	68	-3.153	2.586	1.361	1.00	0.00
ATOM	1066	CB	ALA	68	-4.281	1.121	0.287	1.00	0.00
ATOM	1067	HB1	ALA	68	-4.979	0.312	0.501	1.00	0.00
ATOM	1068	HB2	ALA	68	-4.769	1.863	-0.346	1.00	0.00
ATOM	1069	HB3	ALA	68	-3.409	0.720	-0.230	1.00	0.00
ATOM	1070	C	ALA	68	-5.022	2.392	2.330	1.00	0.00
ATOM	1071	O	ALA	68	-5.394	3.543	2.129	1.00	0.00
ATOM	1072	N	LYS	69	-5.634	1.651	3.242	1.00	0.00
ATOM	1073	H	LYS	69	-5.335	0.690	3.325	1.00	0.00
ATOM	1074	CA	LYS	69	-6.738	2.092	4.045	1.00	0.00
ATOM	1075	HA	LYS	69	-7.440	2.650	3.425	1.00	0.00
ATOM	1076	CB	LYS	69	-7.476	0.899	4.669	1.00	0.00
ATOM	1077	HB2	LYS	69	-7.834	0.250	3.870	1.00	0.00
ATOM	1078	HB3	LYS	69	-6.780	0.346	5.301	1.00	0.00
ATOM	1079	CG	LYS	69	-8.663	1.373	5.511	1.00	0.00
ATOM	1080	HG2	LYS	69	-9.290	0.512	5.745	1.00	0.00
ATOM	1081	HG3	LYS	69	-8.283	1.807	6.436	1.00	0.00
ATOM	1082	CD	LYS	69	-9.494	2.422	4.756	1.00	0.00

ATOM 1083 HD2 LYS 69 -8.951 3.367 4.762 1.00 0.00
ATOM 1084 HD3 LYS 69 -9.626 2.086 3.727 1.00 0.00
ATOM 1085 CE LYS 69 -10.856 2.616 5.405 1.00 0.00
ATOM 1086 HE2 LYS 69 -11.584 1.988 4.891 1.00 0.00
ATOM 1087 HE3 LYS 69 -10.794 2.317 6.451 1.00 0.00
ATOM 1088 NZ LYS 69 -11.278 4.027 5.321 1.00 0.00
ATOM 1089 HZ1 LYS 69 -11.336 4.303 4.351 1.00 0.00
ATOM 1090 HZ2 LYS 69 -12.183 4.134 5.757 1.00 0.00
ATOM 1091 HZ3 LYS 69 -10.604 4.609 5.797 1.00 0.00
ATOM 1092 C LYS 69 -6.227 3.063 5.080 1.00 0.00
ATOM 1093 O LYS 69 -6.913 4.013 5.398 1.00 0.00
ATOM 1094 N SER 70 -5.006 2.877 5.565 1.00 0.00
ATOM 1095 H SER 70 -4.487 2.062 5.271 1.00 0.00
ATOM 1096 CA SER 70 -4.400 3.763 6.535 1.00 0.00
ATOM 1097 HA SER 70 -5.168 4.379 7.003 1.00 0.00
ATOM 1098 CB SER 70 -3.691 2.962 7.619 1.00 0.00
ATOM 1099 HB2 SER 70 -4.407 2.270 8.063 1.00 0.00
ATOM 1100 HB3 SER 70 -2.879 2.398 7.159 1.00 0.00
ATOM 1101 OG SER 70 -3.165 3.811 8.628 1.00 0.00
ATOM 1102 HG SER 70 -2.725 3.280 9.296 1.00 0.00
ATOM 1103 C SER 70 -3.429 4.700 5.843 1.00 0.00
ATOM 1104 O SER 70 -2.625 5.388 6.474 1.00 0.00

ATOM	1105	N	VAL	71	-3.489	4.753	4.507	1.00	0.00
ATOM	1106	H	VAL	71	-4.105	4.123	4.013	1.00	0.00
ATOM	1107	CA	VAL	71	-2.633	5.625	3.731	1.00	0.00
ATOM	1108	HA	VAL	71	-1.576	5.527	3.978	1.00	0.00
ATOM	1109	CB	VAL	71	-2.814	5.341	2.240	1.00	0.00
ATOM	1110	HB	VAL	71	-3.282	4.362	2.127	1.00	0.00
ATOM	1111	CG1	VAL	71	-3.677	6.389	1.583	1.00	0.00
ATOM	1112	HG11	VAL	71	-3.210	7.367	1.695	1.00	0.00
ATOM	1113	HG12	VAL	71	-3.786	6.158	0.523	1.00	0.00
ATOM	1114	HG13	VAL	71	-4.659	6.399	2.055	1.00	0.00
ATOM	1115	CG2	VAL	71	-1.483	5.223	1.526	1.00	0.00
ATOM	1116	HG21	VAL	71	-0.907	4.407	1.963	1.00	0.00
ATOM	1117	HG22	VAL	71	-1.654	5.021	0.468	1.00	0.00
ATOM	1118	HG23	VAL	71	-0.929	6.155	1.632	1.00	0.00
ATOM	1119	C	VAL	71	-2.942	7.068	4.103	1.00	0.00
ATOM	1120	O	VAL	71	-2.232	7.992	3.765	1.00	0.00
ATOM	1121	N	ARG	72	-4.068	7.322	4.763	1.00	0.00
ATOM	1122	H	ARG	72	-4.701	6.557	4.950	1.00	0.00
ATOM	1123	CA	ARG	72	-4.447	8.640	5.193	1.00	0.00
ATOM	1124	HA	ARG	72	-3.569	9.209	5.498	1.00	0.00
ATOM	1125	CB	ARG	72	-5.123	9.369	4.029	1.00	0.00
ATOM	1126	HB2	ARG	72	-5.104	8.726	3.148	1.00	0.00

ATOM 1127 HB3 ARG 72 -6.157 9.585 4.298 1.00 0.00

ATOM 1128 CG ARG 72 -4.393 10.675 3.722 1.00 0.00

ATOM 1129 HG2 ARG 72 -3.461 10.689 4.286 1.00 0.00

ATOM 1130 HG3 ARG 72 -4.172 10.703 2.655 1.00 0.00

ATOM 1131 CD ARG 72 -5.232 11.889 4.098 1.00 0.00

ATOM 1132 HD2 ARG 72 -5.965 12.069 3.311 1.00 0.00

ATOM 1133 HD3 ARG 72 -5.749 11.683 5.035 1.00 0.00

ATOM 1134 NE ARG 72 -4.417 13.096 4.266 1.00 0.00

ATOM 1135 HE ARG 72 -4.464 13.783 3.528 1.00 0.00

ATOM 1136 CZ ARG 72 -3.624 13.333 5.303 1.00 0.00

ATOM 1137 NH1 ARG 72 -3.565 12.476 6.298 1.00 0.00

ATOM 1138 HH11 ARG 72 -4.125 11.636 6.271 1.00 0.00

ATOM 1139 HH12 ARG 72 -2.960 12.661 7.085 1.00 0.00

ATOM 1140 NH2 ARG 72 -2.909 14.428 5.344 1.00 0.00

ATOM 1141 HH21 ARG 72 -2.963 15.091 4.584 1.00 0.00

ATOM 1142 HH22 ARG 72 -2.307 14.604 6.135 1.00 0.00

ATOM 1143 C ARG 72 -5.372 8.547 6.378 1.00 0.00

ATOM 1144 O ARG 72 -4.967 8.889 7.481 1.00 0.00

ATOM 1145 N VAL 73 -6.637 8.180 6.112 1.00 0.00

ATOM 1146 H VAL 73 -6.911 8.026 5.152 1.00 0.00

ATOM 1147 CA VAL 73 -7.649 8.079 7.137 1.00 0.00

ATOM 1148 HA VAL 73 -7.933 9.048 7.547 1.00 0.00

ATOM	1149	CB	VAL	73	-8.897	7.407	6.564	1.00	0.00
ATOM	1150	HB	VAL	73	-9.067	7.802	5.563	1.00	0.00
ATOM	1151	CG1	VAL	73	-8.749	5.894	6.493	1.00	0.00
ATOM	1152	HG11	VAL	73	-8.579	5.497	7.494	1.00	0.00
ATOM	1153	HG12	VAL	73	-9.659	5.458	6.080	1.00	0.00
ATOM	1154	HG13	VAL	73	-7.903	5.640	5.854	1.00	0.00
ATOM	1155	CG2	VAL	73	-10.136	7.772	7.385	1.00	0.00
ATOM	1156	HG21	VAL	73	-10.279	8.852	7.367	1.00	0.00
ATOM	1157	HG22	VAL	73	-11.012	7.282	6.959	1.00	0.00
ATOM	1158	HG23	VAL	73	-10.001	7.441	8.415	1.00	0.00
ATOM	1159	C	VAL	73	-7.068	7.332	8.336	1.00	0.00
ATOM	1160	O	VAL	73	-6.102	6.569	8.213	1.00	0.00
ATOM	1161	N	GLN	74	-7.696	7.521	9.482	1.00	0.00
ATOM	1162	H	GLN	74	-8.471	8.166	9.537	1.00	0.00
ATOM	1163	CA	GLN	74	-7.252	6.876	10.700	1.00	0.00
ATOM	1164	HA	GLN	74	-7.268	5.793	10.571	1.00	0.00
ATOM	1165	CB	GLN	74	-5.822	7.307	11.046	1.00	0.00
ATOM	1166	HB2	GLN	74	-5.338	7.691	10.148	1.00	0.00
ATOM	1167	HB3	GLN	74	-5.859	8.091	11.802	1.00	0.00
ATOM	1168	CG	GLN	74	-5.025	6.114	11.585	1.00	0.00
ATOM	1169	HG2	GLN	74	-5.719	5.427	12.070	1.00	0.00
ATOM	1170	HG3	GLN	74	-4.518	5.600	10.768	1.00	0.00

ATOM	1171	CD	GLN	74	-3.983	6.553	12.604	1.00	0.00
ATOM	1172	OE1	GLN	74	-3.640	7.720	12.733	1.00	0.00
ATOM	1173	NE2	GLN	74	-3.452	5.586	13.330	1.00	0.00
ATOM	1174	HE21	GLN	74	-2.754	5.806	14.026	1.00	0.00
ATOM	1175	HE22	GLN	74	-3.747	4.630	13.186	1.00	0.00
ATOM	1176	C	GLN	74	-8.210	7.178	11.848	1.00	0.00
ATOM	1177	O	GLN	74	-7.814	7.354	12.994	1.00	0.00
ATOM	1178	N	GLY	75	-9.498	7.306	11.516	1.00	0.00
ATOM	1179	H	GLY	75	-9.779	7.201	10.551	1.00	0.00
ATOM	1180	CA	GLY	75	-10.519	7.593	12.501	1.00	0.00
ATOM	1181	HA2	GLY	75	-10.634	6.733	13.161	1.00	0.00
ATOM	1182	HA3	GLY	75	-10.220	8.462	13.086	1.00	0.00
ATOM	1183	C	GLY	75	-11.842	7.881	11.835	1.00	0.00
ATOM	1184	O	GLY	75	-12.830	7.205	12.105	1.00	0.00
ATOM	1185	N	GLN	76	-11.842	8.841	10.897	1.00	0.00
ATOM	1186	H	GLN	76	-10.990	9.347	10.704	1.00	0.00
ATOM	1187	CA	GLN	76	-13.044	9.224	10.186	1.00	0.00
ATOM	1188	HA	GLN	76	-13.356	8.405	9.538	1.00	0.00
ATOM	1189	CB	GLN	76	-14.189	9.547	11.154	1.00	0.00
ATOM	1190	HB2	GLN	76	-13.928	9.182	12.147	1.00	0.00
ATOM	1191	HB3	GLN	76	-14.331	10.627	11.189	1.00	0.00
ATOM	1192	CG	GLN	76	-15.485	8.878	10.689	1.00	0.00

ATOM 1193 HG2 GLN 76 -15.539 8.905 9.601 1.00 0.00

ATOM 1194 HG3 GLN 76 -15.512 7.842 11.027 1.00 0.00

ATOM 1195 CD GLN 76 -16.693 9.604 11.253 1.00 0.00

ATOM 1196 OE1 GLN 76 -17.268 10.471 10.616 1.00 0.00

ATOM 1197 NE2 GLN 76 -17.065 9.239 12.463 1.00 0.00

ATOM 1198 HE21 GLN 76 -17.862 9.682 12.898 1.00 0.00

ATOM 1199 HE22 GLN 76 -16.552 8.518 12.950 1.00 0.00

ATOM 1200 C GLN 76 -12.769 10.412 9.284 1.00 0.00

ATOM 1201 O GLN 76 -12.590 11.539 9.733 1.00 0.00

ATOM 1202 N HID 77 -12.652 10.144 7.985 1.00 0.00

ATOM 1203 H HID 77 -12.636 9.181 7.681 1.00 0.00

ATOM 1204 CA HID 77 -12.418 11.183 6.995 1.00 0.00

ATOM 1205 HA HID 77 -12.587 12.167 7.432 1.00 0.00

ATOM 1206 CB HID 77 -10.969 11.106 6.509 1.00 0.00

ATOM 1207 HB2 HID 77 -10.284 11.271 7.340 1.00 0.00

ATOM 1208 HB3 HID 77 -10.766 10.133 6.060 1.00 0.00

ATOM 1209 CG HID 77 -10.638 12.139 5.466 1.00 0.00

ATOM 1210 ND1 HID 77 -10.018 13.341 5.731 1.00 0.00

ATOM 1211 HD1 HID 77 -9.724 13.663 6.642 1.00 0.00

ATOM 1212 CE1 HID 77 -9.873 14.009 4.570 1.00 0.00

ATOM 1213 HE1 HID 77 -9.392 14.987 4.555 1.00 0.00

ATOM 1214 NE2 HID 77 -10.397 13.269 3.578 1.00 0.00

ATOM	1215	CD2	HID	77	-10.873	12.086	4.114	1.00	0.00
ATOM	1216	HD2	HID	77	-11.328	11.337	3.465	1.00	0.00
ATOM	1217	C	HID	77	-13.450	11.085	5.878	1.00	0.00
ATOM	1218	O	HID	77	-13.339	11.753	4.861	1.00	0.00
ATOM	1219	N	GLY	78	-14.459	10.233	6.063	1.00	0.00
ATOM	1220	H	GLY	78	-14.515	9.710	6.925	1.00	0.00
ATOM	1221	CA	GLY	78	-15.483	10.027	5.071	1.00	0.00
ATOM	1222	HA2	GLY	78	-16.148	9.230	5.406	1.00	0.00
ATOM	1223	HA3	GLY	78	-16.053	10.948	4.952	1.00	0.00
ATOM	1224	C	GLY	78	-14.880	9.636	3.720	1.00	0.00
ATOM	1225	O	GLY	78	-13.657	9.460	3.565	1.00	0.00
ATOM	1226	N	PRO	79	-15.757	9.434	2.736	1.00	0.00
ATOM	1227	CD	PRO	79	-17.204	9.562	2.837	1.00	0.00
ATOM	1228	HD2	PRO	79	-17.440	10.613	2.999	1.00	0.00
ATOM	1229	HD3	PRO	79	-17.536	8.979	3.696	1.00	0.00
ATOM	1230	CG	PRO	79	-17.768	9.029	1.516	1.00	0.00
ATOM	1231	HG2	PRO	79	-18.282	9.838	0.998	1.00	0.00
ATOM	1232	HG3	PRO	79	-18.476	8.228	1.730	1.00	0.00
ATOM	1233	CB	PRO	79	-16.581	8.506	0.698	1.00	0.00
ATOM	1234	HB2	PRO	79	-16.614	8.848	-0.337	1.00	0.00
ATOM	1235	HB3	PRO	79	-16.549	7.416	0.719	1.00	0.00
ATOM	1236	CA	PRO	79	-15.348	9.077	1.395	1.00	0.00

ATOM 1237 HA PRO 79 -14.624 8.265 1.466 1.00 0.00
ATOM 1238 C PRO 79 -14.725 10.245 0.655 1.00 0.00
ATOM 1239 O PRO 79 -15.427 11.095 0.128 1.00 0.00
ATOM 1240 N PHE 80 -13.387 10.284 0.630 1.00 0.00
ATOM 1241 H PHE 80 -12.849 9.549 1.068 1.00 0.00
ATOM 1242 CA PHE 80 -12.674 11.362 -0.019 1.00 0.00
ATOM 1243 HA PHE 80 -13.233 11.751 -0.870 1.00 0.00
ATOM 1244 CB PHE 80 -12.506 12.482 1.008 1.00 0.00
ATOM 1245 HB2 PHE 80 -13.350 12.489 1.698 1.00 0.00
ATOM 1246 HB3 PHE 80 -11.582 12.333 1.566 1.00 0.00
ATOM 1247 CG PHE 80 -12.429 13.859 0.407 1.00 0.00
ATOM 1248 CD1 PHE 80 -13.599 14.500 -0.022 1.00 0.00
ATOM 1249 HD1 PHE 80 -14.560 13.996 0.079 1.00 0.00
ATOM 1250 CE1 PHE 80 -13.536 15.787 -0.581 1.00 0.00
ATOM 1251 HE1 PHE 80 -14.452 16.277 -0.912 1.00 0.00
ATOM 1252 CZ PHE 80 -12.303 16.441 -0.715 1.00 0.00
ATOM 1253 HZ PHE 80 -12.248 17.438 -1.151 1.00 0.00
ATOM 1254 CE2 PHE 80 -11.133 15.799 -0.282 1.00 0.00
ATOM 1255 HE2 PHE 80 -10.172 16.303 -0.381 1.00 0.00
ATOM 1256 CD2 PHE 80 -11.197 14.513 0.277 1.00 0.00
ATOM 1257 HD2 PHE 80 -10.285 14.018 0.612 1.00 0.00
ATOM 1258 C PHE 80 -11.311 10.896 -0.497 1.00 0.00

ATOM	1259	O	PHE	80	-11.015	10.862	-1.680	1.00	0.00
ATOM	1260	N	GLN	81	-10.463	10.499	0.460	1.00	0.00
ATOM	1261	H	GLN	81	-10.769	10.500	1.422	1.00	0.00
ATOM	1262	CA	GLN	81	-9.112	10.083	0.172	1.00	0.00
ATOM	1263	HA	GLN	81	-8.654	10.800	-0.509	1.00	0.00
ATOM	1264	CB	GLN	81	-8.266	10.002	1.436	1.00	0.00
ATOM	1265	HB2	GLN	81	-8.620	9.166	2.040	1.00	0.00
ATOM	1266	HB3	GLN	81	-7.228	9.829	1.151	1.00	0.00
ATOM	1267	CG	GLN	81	-8.363	11.289	2.244	1.00	0.00
ATOM	1268	HG2	GLN	81	-7.509	11.916	1.988	1.00	0.00
ATOM	1269	HG3	GLN	81	-9.286	11.820	2.012	1.00	0.00
ATOM	1270	CD	GLN	81	-8.328	11.007	3.733	1.00	0.00
ATOM	1271	OE1	GLN	81	-8.652	9.944	4.230	1.00	0.00
ATOM	1272	NE2	GLN	81	-7.946	12.014	4.487	1.00	0.00
ATOM	1273	HE21	GLN	81	-7.898	11.900	5.489	1.00	0.00
ATOM	1274	HE22	GLN	81	-7.702	12.896	4.060	1.00	0.00
ATOM	1275	C	GLN	81	-9.181	8.743	-0.483	1.00	0.00
ATOM	1276	O	GLN	81	-8.729	8.658	-1.593	1.00	0.00
ATOM	1277	N	SER	82	-9.795	7.741	0.158	1.00	0.00
ATOM	1278	H	SER	82	-10.194	7.928	1.066	1.00	0.00
ATOM	1279	CA	SER	82	-9.913	6.398	-0.382	1.00	0.00
ATOM	1280	HA	SER	82	-8.982	5.854	-0.222	1.00	0.00

ATOM 1281 CB SER 82 -11.045 5.640 0.304 1.00 0.00
ATOM 1282 HB2 SER 82 -10.782 5.527 1.355 1.00 0.00
ATOM 1283 HB3 SER 82 -11.949 6.242 0.219 1.00 0.00
ATOM 1284 OG SER 82 -11.272 4.372 -0.261 1.00 0.00
ATOM 1285 HG SER 82 -11.991 3.938 0.204 1.00 0.00
ATOM 1286 C SER 82 -10.198 6.444 -1.866 1.00 0.00
ATOM 1287 O SER 82 -9.495 5.820 -2.617 1.00 0.00
ATOM 1288 N THR 83 -11.135 7.290 -2.282 1.00 0.00
ATOM 1289 H THR 83 -11.702 7.765 -1.594 1.00 0.00
ATOM 1290 CA THR 83 -11.457 7.473 -3.680 1.00 0.00
ATOM 1291 HA THR 83 -12.036 6.606 -3.999 1.00 0.00
ATOM 1292 CB THR 83 -12.324 8.722 -3.833 1.00 0.00
ATOM 1293 HB THR 83 -12.149 9.349 -2.959 1.00 0.00
ATOM 1294 CG2 THR 83 -12.027 9.520 -5.099 1.00 0.00
ATOM 1295 HG21 THR 83 -12.202 8.893 -5.974 1.00 0.00
ATOM 1296 HG22 THR 83 -12.680 10.392 -5.141 1.00 0.00
ATOM 1297 HG23 THR 83 -10.987 9.845 -5.088 1.00 0.00
ATOM 1298 OG1 THR 83 -13.639 8.266 -3.957 1.00 0.00
ATOM 1299 HG1 THR 83 -14.230 9.016 -4.057 1.00 0.00
ATOM 1300 C THR 83 -10.202 7.510 -4.542 1.00 0.00
ATOM 1301 O THR 83 -9.877 6.549 -5.241 1.00 0.00
ATOM 1302 N ARG 84 -9.445 8.610 -4.448 1.00 0.00

ATOM	1303	H	ARG	84	-9.725	9.352	-3.824	1.00	0.00
ATOM	1304	CA	ARG	84	-8.248	8.784	-5.217	1.00	0.00
ATOM	1305	HA	ARG	84	-8.445	8.538	-6.261	1.00	0.00
ATOM	1306	CB	ARG	84	-7.756	10.230	-5.140	1.00	0.00
ATOM	1307	HB2	ARG	84	-8.425	10.795	-4.491	1.00	0.00
ATOM	1308	HB3	ARG	84	-6.750	10.238	-4.720	1.00	0.00
ATOM	1309	CG	ARG	84	-7.733	10.871	-6.533	1.00	0.00
ATOM	1310	HG2	ARG	84	-8.762	11.055	-6.841	1.00	0.00
ATOM	1311	HG3	ARG	84	-7.199	11.819	-6.464	1.00	0.00
ATOM	1312	CD	ARG	84	-7.045	9.972	-7.565	1.00	0.00
ATOM	1313	HD2	ARG	84	-6.576	9.129	-7.057	1.00	0.00
ATOM	1314	HD3	ARG	84	-7.785	9.601	-8.275	1.00	0.00
ATOM	1315	NE	ARG	84	-6.010	10.712	-8.306	1.00	0.00
ATOM	1316	HE	ARG	84	-5.678	11.560	-7.869	1.00	0.00
ATOM	1317	CZ	ARG	84	-5.508	10.369	-9.475	1.00	0.00
ATOM	1318	NH1	ARG	84	-5.969	9.316	-10.109	1.00	0.00
ATOM	1319	HH11	ARG	84	-6.711	8.767	-9.698	1.00	0.00
ATOM	1320	HH12	ARG	84	-5.580	9.059	-11.005	1.00	0.00
ATOM	1321	NH2	ARG	84	-4.531	11.068	-10.005	1.00	0.00
ATOM	1322	HH21	ARG	84	-4.164	11.870	-9.514	1.00	0.00
ATOM	1323	HH22	ARG	84	-4.151	10.799	-10.902	1.00	0.00
ATOM	1324	C	ARG	84	-7.177	7.869	-4.698	1.00	0.00

ATOM 1325 O ARG 84 -6.458 7.274 -5.481 1.00 0.00
ATOM 1326 N ILE 85 -7.012 7.800 -3.385 1.00 0.00
ATOM 1327 H ILE 85 -7.547 8.447 -2.824 1.00 0.00
ATOM 1328 CA ILE 85 -6.064 6.946 -2.723 1.00 0.00
ATOM 1329 HA ILE 85 -5.032 7.258 -2.883 1.00 0.00
ATOM 1330 CB ILE 85 -6.346 6.863 -1.217 1.00 0.00
ATOM 1331 HB ILE 85 -7.424 6.905 -1.060 1.00 0.00
ATOM 1332 CG2 ILE 85 -5.798 5.569 -0.629 1.00 0.00
ATOM 1333 HG21 ILE 85 -4.720 5.526 -0.785 1.00 0.00
ATOM 1334 HG22 ILE 85 -6.011 5.535 0.439 1.00 0.00
ATOM 1335 HG23 ILE 85 -6.270 4.717 -1.120 1.00 0.00
ATOM 1336 CG1 ILE 85 -5.804 8.105 -0.488 1.00 0.00
ATOM 1337 HG12 ILE 85 -6.345 8.980 -0.847 1.00 0.00
ATOM 1338 HG13 ILE 85 -5.980 7.983 0.581 1.00 0.00
ATOM 1339 CD1 ILE 85 -4.309 8.292 -0.740 1.00 0.00
ATOM 1340 HD11 ILE 85 -4.133 8.414 -1.809 1.00 0.00
ATOM 1341 HD12 ILE 85 -3.959 9.178 -0.211 1.00 0.00
ATOM 1342 HD13 ILE 85 -3.767 7.417 -0.381 1.00 0.00
ATOM 1343 C ILE 85 -6.058 5.581 -3.382 1.00 0.00
ATOM 1344 O ILE 85 -5.004 4.999 -3.533 1.00 0.00
ATOM 1345 N TYR 86 -7.204 5.124 -3.861 1.00 0.00
ATOM 1346 H TYR 86 -8.019 5.710 -3.751 1.00 0.00

ATOM	1347	CA	TYR	86	-7.352	3.880	-4.547	1.00	0.00
ATOM	1348	HA	TYR	86	-6.666	3.126	-4.161	1.00	0.00
ATOM	1349	CB	TYR	86	-8.778	3.407	-4.308	1.00	0.00
ATOM	1350	HB2	TYR	86	-9.398	4.302	-4.268	1.00	0.00
ATOM	1351	HB3	TYR	86	-9.070	2.800	-5.166	1.00	0.00
ATOM	1352	CG	TYR	86	-8.966	2.606	-3.036	1.00	0.00
ATOM	1353	CD1	TYR	86	-8.654	3.165	-1.785	1.00	0.00
ATOM	1354	HD1	TYR	86	-8.277	4.186	-1.739	1.00	0.00
ATOM	1355	CE1	TYR	86	-8.822	2.425	-0.605	1.00	0.00
ATOM	1356	HE1	TYR	86	-8.543	2.842	0.362	1.00	0.00
ATOM	1357	CZ	TYR	86	-9.307	1.112	-0.689	1.00	0.00
ATOM	1358	OH	TYR	86	-9.519	0.419	0.455	1.00	0.00
ATOM	1359	HH	TYR	86	-9.305	0.922	1.244	1.00	0.00
ATOM	1360	CE2	TYR	86	-9.622	0.533	-1.930	1.00	0.00
ATOM	1361	HE2	TYR	86	-10.000	-0.488	-1.989	1.00	0.00
ATOM	1362	CD2	TYR	86	-9.444	1.286	-3.106	1.00	0.00
ATOM	1363	HD2	TYR	86	-9.677	0.845	-4.076	1.00	0.00
ATOM	1364	C	TYR	86	-7.145	4.047	-6.028	1.00	0.00
ATOM	1365	O	TYR	86	-6.478	3.245	-6.662	1.00	0.00
ATOM	1366	N	GLN	87	-7.650	5.130	-6.611	1.00	0.00
ATOM	1367	H	GLN	87	-8.210	5.758	-6.052	1.00	0.00
ATOM	1368	CA	GLN	87	-7.482	5.427	-8.009	1.00	0.00

ATOM 1369 HA GLN 87 -7.812 4.578 -8.608 1.00 0.00

ATOM 1370 CB GLN 87 -8.306 6.649 -8.400 1.00 0.00

ATOM 1371 HB2 GLN 87 -9.027 6.856 -7.610 1.00 0.00

ATOM 1372 HB3 GLN 87 -7.638 7.503 -8.515 1.00 0.00

ATOM 1373 CG GLN 87 -9.048 6.402 -9.715 1.00 0.00

ATOM 1374 HG2 GLN 87 -8.430 6.463 -10.611 1.00 0.00

ATOM 1375 HG3 GLN 87 -9.423 5.385 -9.602 1.00 0.00

ATOM 1376 CD GLN 87 -10.234 7.343 -9.876 1.00 0.00

ATOM 1377 OE1 GLN 87 -10.284 8.409 -9.273 1.00 0.00

ATOM 1378 NE2 GLN 87 -11.100 7.009 -10.798 1.00 0.00

ATOM 1379 HE21 GLN 87 -11.911 7.589 -10.959 1.00 0.00

ATOM 1380 HE22 GLN 87 -10.953 6.172 -11.345 1.00 0.00

ATOM 1381 C GLN 87 -6.019 5.656 -8.338 1.00 0.00

ATOM 1382 O GLN 87 -5.644 5.754 -9.500 1.00 0.00

ATOM 1383 N ILE 88 -5.154 5.765 -7.344 1.00 0.00

ATOM 1384 H ILE 88 -5.527 5.771 -6.405 1.00 0.00

ATOM 1385 CA ILE 88 -3.741 5.947 -7.516 1.00 0.00

ATOM 1386 HA ILE 88 -3.465 5.947 -8.571 1.00 0.00

ATOM 1387 CB ILE 88 -3.315 7.270 -6.877 1.00 0.00

ATOM 1388 HB ILE 88 -3.658 7.288 -5.843 1.00 0.00

ATOM 1389 CG2 ILE 88 -1.796 7.422 -6.912 1.00 0.00

ATOM 1390 HG21 ILE 88 -1.452 7.404 -7.946 1.00 0.00

ATOM	1391	HG22	ILE	88	-1.515	8.369	-6.453	1.00	0.00
ATOM	1392	HG23	ILE	88	-1.335	6.601	-6.362	1.00	0.00
ATOM	1393	CG1	ILE	88	-3.981	8.449	-7.591	1.00	0.00
ATOM	1394	HG12	ILE	88	-3.438	8.664	-8.511	1.00	0.00
ATOM	1395	HG13	ILE	88	-5.012	8.187	-7.831	1.00	0.00
ATOM	1396	CD1	ILE	88	-3.966	9.689	-6.686	1.00	0.00
ATOM	1397	HD11	ILE	88	-2.936	9.951	-6.446	1.00	0.00
ATOM	1398	HD12	ILE	88	-4.442	10.522	-7.203	1.00	0.00
ATOM	1399	HD13	ILE	88	-4.510	9.474	-5.766	1.00	0.00
ATOM	1400	C	ILE	88	-3.016	4.755	-6.926	1.00	0.00
ATOM	1401	O	ILE	88	-1.833	4.599	-7.139	1.00	0.00
ATOM	1402	N	ALA	89	-3.716	3.894	-6.208	1.00	0.00
ATOM	1403	H	ALA	89	-4.681	4.111	-6.001	1.00	0.00
ATOM	1404	CA	ALA	89	-3.149	2.696	-5.642	1.00	0.00
ATOM	1405	HA	ALA	89	-2.060	2.743	-5.669	1.00	0.00
ATOM	1406	CB	ALA	89	-3.594	2.575	-4.194	1.00	0.00
ATOM	1407	HB1	ALA	89	-4.682	2.517	-4.151	1.00	0.00
ATOM	1408	HB2	ALA	89	-3.166	1.673	-3.756	1.00	0.00
ATOM	1409	HB3	ALA	89	-3.255	3.447	-3.635	1.00	0.00
ATOM	1410	C	ALA	89	-3.549	1.488	-6.472	1.00	0.00
ATOM	1411	O	ALA	89	-3.213	0.354	-6.143	1.00	0.00
ATOM	1412	N	LYS	90	-4.264	1.728	-7.577	1.00	0.00

ATOM 1413 H LYS 90 -4.569 2.673 -7.762 1.00 0.00
ATOM 1414 CA LYS 90 -4.715 0.675 -8.463 1.00 0.00
ATOM 1415 HA LYS 90 -4.668 -0.286 -7.951 1.00 0.00
ATOM 1416 CB LYS 90 -6.165 0.929 -8.897 1.00 0.00
ATOM 1417 HB2 LYS 90 -6.682 -0.031 -8.913 1.00 0.00
ATOM 1418 HB3 LYS 90 -6.627 1.578 -8.153 1.00 0.00
ATOM 1419 CG LYS 90 -6.263 1.579 -10.267 1.00 0.00
ATOM 1420 HG2 LYS 90 -5.680 0.988 -10.974 1.00 0.00
ATOM 1421 HG3 LYS 90 -7.309 1.583 -10.575 1.00 0.00
ATOM 1422 CD LYS 90 -5.739 2.997 -10.242 1.00 0.00
ATOM 1423 HD2 LYS 90 -6.424 3.615 -9.661 1.00 0.00
ATOM 1424 HD3 LYS 90 -4.757 3.000 -9.769 1.00 0.00
ATOM 1425 CE LYS 90 -5.624 3.558 -11.658 1.00 0.00
ATOM 1426 HE2 LYS 90 -5.199 2.789 -12.304 1.00 0.00
ATOM 1427 HE3 LYS 90 -6.622 3.817 -12.012 1.00 0.00
ATOM 1428 NZ LYS 90 -4.766 4.755 -11.689 1.00 0.00
ATOM 1429 HZ1 LYS 90 -3.841 4.515 -11.362 1.00 0.00
ATOM 1430 HZ2 LYS 90 -4.708 5.103 -12.635 1.00 0.00
ATOM 1431 HZ3 LYS 90 -5.160 5.467 -11.092 1.00 0.00
ATOM 1432 C LYS 90 -3.758 0.555 -9.620 1.00 0.00
ATOM 1433 O LYS 90 -3.776 -0.419 -10.354 1.00 0.00
ATOM 1434 N ASN 91 -2.954 1.604 -9.838 1.00 0.00

ATOM	1435	H	ASN	91	-3.051	2.419	-9.250	1.00	0.00
ATOM	1436	CA	ASN	91	-1.972	1.633	-10.885	1.00	0.00
ATOM	1437	HA	ASN	91	-2.293	0.996	-11.710	1.00	0.00
ATOM	1438	CB	ASN	91	-1.789	3.058	-11.410	1.00	0.00
ATOM	1439	HB2	ASN	91	-0.911	3.046	-12.055	1.00	0.00
ATOM	1440	HB3	ASN	91	-2.658	3.376	-11.986	1.00	0.00
ATOM	1441	CG	ASN	91	-1.558	4.049	-10.301	1.00	0.00
ATOM	1442	OD1	ASN	91	-0.916	3.748	-9.324	1.00	0.00
ATOM	1443	ND2	ASN	91	-2.143	5.222	-10.453	1.00	0.00
ATOM	1444	HD21	ASN	91	-2.030	5.934	-9.746	1.00	0.00
ATOM	1445	HD22	ASN	91	-2.701	5.401	-11.276	1.00	0.00
ATOM	1446	C	ASN	91	-0.634	1.110	-10.372	1.00	0.00
ATOM	1447	O	ASN	91	0.362	1.145	-11.086	1.00	0.00
ATOM	1448	N	LEU	92	-0.599	0.707	-9.106	1.00	0.00
ATOM	1449	H	LEU	92	-1.418	0.836	-8.529	1.00	0.00
ATOM	1450	CA	LEU	92	0.583	0.157	-8.498	1.00	0.00
ATOM	1451	HA	LEU	92	1.455	0.752	-8.768	1.00	0.00
ATOM	1452	CB	LEU	92	0.435	0.168	-6.970	1.00	0.00
ATOM	1453	HB2	LEU	92	-0.642	0.185	-6.805	1.00	0.00
ATOM	1454	HB3	LEU	92	0.853	-0.745	-6.545	1.00	0.00
ATOM	1455	CG	LEU	92	1.075	1.386	-6.294	1.00	0.00
ATOM	1456	HG	LEU	92	0.893	2.270	-6.905	1.00	0.00

ATOM	1457	CD1	LEU	92	0.474	1.596	-4.902	1.00	0.00
ATOM	1458	HD11	LEU	92	0.656	0.712	-4.290	1.00	0.00
ATOM	1459	HD12	LEU	92	0.937	2.464	-4.433	1.00	0.00
ATOM	1460	HD13	LEU	92	-0.600	1.761	-4.991	1.00	0.00
ATOM	1461	CD2	LEU	92	2.591	1.242	-6.225	1.00	0.00
ATOM	1462	HD21	LEU	92	2.994	1.151	-7.234	1.00	0.00
ATOM	1463	HD22	LEU	92	3.018	2.120	-5.741	1.00	0.00
ATOM	1464	HD23	LEU	92	2.846	0.351	-5.651	1.00	0.00
ATOM	1465	C	LEU	92	0.839	-1.242	-9.028	1.00	0.00
ATOM	1466	O	LEU	92	1.921	-1.501	-9.513	1.00	0.00
ATOM	1467	N	PRO	93	-0.177	-2.119	-9.017	1.00	0.00
ATOM	1468	CD	PRO	93	-1.521	-1.868	-8.524	1.00	0.00
ATOM	1469	HD2	PRO	93	-1.982	-1.138	-9.189	1.00	0.00
ATOM	1470	HD3	PRO	93	-1.429	-1.442	-7.525	1.00	0.00
ATOM	1471	CG	PRO	93	-2.252	-3.193	-8.521	1.00	0.00
ATOM	1472	HG2	PRO	93	-3.092	-3.142	-9.214	1.00	0.00
ATOM	1473	HG3	PRO	93	-2.623	-3.397	-7.517	1.00	0.00
ATOM	1474	CB	PRO	93	-1.234	-4.251	-8.953	1.00	0.00
ATOM	1475	HB2	PRO	93	-1.645	-4.926	-9.704	1.00	0.00
ATOM	1476	HB3	PRO	93	-0.896	-4.828	-8.093	1.00	0.00
ATOM	1477	CA	PRO	93	-0.065	-3.465	-9.546	1.00	0.00
ATOM	1478	HA	PRO	93	0.852	-3.905	-9.153	1.00	0.00

```
ATOM   1479  C   PRO    93      -0.030  -3.495 -11.064  1.00  0.00
ATOM   1480  O   PRO    93       0.093  -4.562 -11.661  1.00  0.00
ATOM   1481  N   ASN    94      -0.137  -2.312 -11.674  1.00  0.00
ATOM   1482  H   ASN    94      -0.240  -1.473 -11.121  1.00  0.00
ATOM   1483  CA  ASN    94      -0.106  -2.186 -13.101  1.00  0.00
ATOM   1484  HA  ASN    94      -0.543  -3.071 -13.564  1.00  0.00
ATOM   1485  CB  ASN    94      -0.911  -0.960 -13.535  1.00  0.00
ATOM   1486  HB2 ASN    94      -0.681  -0.125 -12.874  1.00  0.00
ATOM   1487  HB3 ASN    94      -0.631  -0.702 -14.556  1.00  0.00
ATOM   1488  CG  ASN    94      -2.400  -1.215 -13.492  1.00  0.00
ATOM   1489  OD1 ASN    94      -2.876  -2.335 -13.425  1.00  0.00
ATOM   1490  ND2 ASN    94      -3.157  -0.133 -13.524  1.00  0.00
ATOM   1491 HD21 ASN    94      -4.163  -0.223 -13.499  1.00  0.00
ATOM   1492 HD22 ASN    94      -2.727   0.779 -13.574  1.00  0.00
ATOM   1493  C   ASN    94       1.323  -2.023 -13.577  1.00  0.00
ATOM   1494  O   ASN    94       1.710  -2.566 -14.604  1.00  0.00
ATOM   1495  N   VAL    95       2.105  -1.250 -12.821  1.00  0.00
ATOM   1496  H   VAL    95       1.709  -0.771 -12.025  1.00  0.00
ATOM   1497  CA  VAL    95       3.498  -1.010 -13.144  1.00  0.00
ATOM   1498  HA  VAL    95       3.759  -1.375 -14.138  1.00  0.00
ATOM   1499  CB  VAL    95       3.787   0.491 -13.082  1.00  0.00
ATOM   1500  HB  VAL    95       3.652   0.831 -12.055  1.00  0.00
```

```
ATOM   1501  CG1 VAL    95       5.214   0.793 -13.535  1.00  0.00
ATOM   1502 HG11 VAL    95       5.349   0.453 -14.562  1.00  0.00
ATOM   1503 HG12 VAL    95       5.393   1.866 -13.481  1.00  0.00
ATOM   1504 HG13 VAL    95       5.919   0.274 -12.886  1.00  0.00
ATOM   1505  CG2 VAL    95       2.785   1.269 -13.947  1.00  0.00
ATOM   1506 HG21 VAL    95       1.773   1.088 -13.583  1.00  0.00
ATOM   1507 HG22 VAL    95       3.006   2.334 -13.891  1.00  0.00
ATOM   1508 HG23 VAL    95       2.863   0.936 -14.982  1.00  0.00
ATOM   1509  C   VAL    95       4.395  -1.801 -12.204  1.00  0.00
ATOM   1510  O   VAL    95       5.602  -1.867 -12.386  1.00  0.00
ATOM   1511  N   CYX    96       3.790  -2.460 -11.220  1.00  0.00
ATOM   1512  H   CYX    96       2.793  -2.348 -11.108  1.00  0.00
ATOM   1513  CA  CYX    96       4.499  -3.252 -10.255  1.00  0.00
ATOM   1514  HA  CYX    96       5.538  -3.348 -10.570  1.00  0.00
ATOM   1515  CB  CYX    96       4.467  -2.627  -8.860  1.00  0.00
ATOM   1516  HB2 CYX    96       3.464  -2.719  -8.443  1.00  0.00
ATOM   1517  HB3 CYX    96       5.177  -3.143  -8.214  1.00  0.00
ATOM   1518  SG  CYX    96       4.888  -0.892  -8.825  1.00  0.00
ATOM   1519  C   CYX    96       3.919  -4.628 -10.275  1.00  0.00
ATOM   1520  O   CYX    96       3.793  -5.162  -9.199  1.00  0.00
ATOM   1521  N   ASN    97       3.542  -5.151 -11.453  1.00  0.00
ATOM   1522  H   ASN    97       3.671  -4.585 -12.279  1.00  0.00
```

ATOM	1523	CA	ASN	97	2.965	-6.469	-11.608	1.00	0.00
ATOM	1524	HA	ASN	97	1.878	-6.394	-11.633	1.00	0.00
ATOM	1525	CB	ASN	97	3.439	-7.117	-12.909	1.00	0.00
ATOM	1526	HB2	ASN	97	4.421	-6.736	-13.189	1.00	0.00
ATOM	1527	HB3	ASN	97	3.501	-8.193	-12.744	1.00	0.00
ATOM	1528	CG	ASN	97	2.483	-6.859	-14.041	1.00	0.00
ATOM	1529	OD1	ASN	97	1.752	-7.729	-14.484	1.00	0.00
ATOM	1530	ND2	ASN	97	2.473	-5.623	-14.490	1.00	0.00
ATOM	1531	HD21	ASN	97	1.857	-5.370	-15.250	1.00	0.00
ATOM	1532	HD22	ASN	97	3.081	-4.933	-14.073	1.00	0.00
ATOM	1533	C	ASN	97	3.361	-7.388	-10.454	1.00	0.00
ATOM	1534	O	ASN	97	4.464	-7.935	-10.448	1.00	0.00
ATOM	1535	N	MET	98	2.492	-7.467	-9.449	1.00	0.00
ATOM	1536	H	MET	98	1.617	-6.965	-9.501	1.00	0.00
ATOM	1537	CA	MET	98	2.792	-8.197	-8.238	1.00	0.00
ATOM	1538	HA	MET	98	3.640	-8.858	-8.420	1.00	0.00
ATOM	1539	CB	MET	98	3.141	-7.262	-7.082	1.00	0.00
ATOM	1540	HB2	MET	98	3.480	-7.862	-6.237	1.00	0.00
ATOM	1541	HB3	MET	98	3.943	-6.596	-7.400	1.00	0.00
ATOM	1542	CG	MET	98	1.930	-6.435	-6.664	1.00	0.00
ATOM	1543	HG2	MET	98	1.066	-6.784	-7.229	1.00	0.00
ATOM	1544	HG3	MET	98	1.756	-6.596	-5.600	1.00	0.00

ATOM 1545 SD MET 98 2.163 -4.690 -6.964 1.00 0.00
ATOM 1546 CE MET 98 0.830 -3.995 -6.030 1.00 0.00
ATOM 1547 HE1 MET 98 0.938 -4.272 -4.981 1.00 0.00
ATOM 1548 HE2 MET 98 0.848 -2.909 -6.123 1.00 0.00
ATOM 1549 HE3 MET 98 -0.118 -4.376 -6.410 1.00 0.00
ATOM 1550 C MET 98 1.617 -9.078 -7.895 1.00 0.00
ATOM 1551 O MET 98 1.546 -9.646 -6.815 1.00 0.00
ATOM 1552 N LYS 99 0.633 -9.158 -8.799 1.00 0.00
ATOM 1553 H LYS 99 0.680 -8.549 -9.604 1.00 0.00
ATOM 1554 CA LYS 99 -0.556 -9.942 -8.607 1.00 0.00
ATOM 1555 HA LYS 99 -0.356 -10.768 -7.924 1.00 0.00
ATOM 1556 CB LYS 99 -1.664 -9.065 -8.005 1.00 0.00
ATOM 1557 HB2 LYS 99 -1.594 -9.116 -6.919 1.00 0.00
ATOM 1558 HB3 LYS 99 -1.507 -8.037 -8.331 1.00 0.00
ATOM 1559 CG LYS 99 -3.056 -9.536 -8.450 1.00 0.00
ATOM 1560 HG2 LYS 99 -2.991 -10.570 -8.790 1.00 0.00
ATOM 1561 HG3 LYS 99 -3.740 -9.475 -7.604 1.00 0.00
ATOM 1562 CD LYS 99 -3.579 -8.649 -9.599 1.00 0.00
ATOM 1563 HD2 LYS 99 -4.296 -7.937 -9.191 1.00 0.00
ATOM 1564 HD3 LYS 99 -2.738 -8.109 -10.033 1.00 0.00
ATOM 1565 CE LYS 99 -4.262 -9.498 -10.690 1.00 0.00
ATOM 1566 HE2 LYS 99 -4.099 -10.552 -10.463 1.00 0.00

```
ATOM   1567  HE3 LYS    99      -5.331  -9.285 -10.678  1.00  0.00
ATOM   1568  NZ  LYS    99      -3.714  -9.192 -12.029  1.00  0.00
ATOM   1569  HZ1 LYS    99      -2.724  -9.390 -12.041  1.00  0.00
ATOM   1570  HZ2 LYS    99      -4.179  -9.762 -12.721  1.00  0.00
ATOM   1571  HZ3 LYS    99      -3.865  -8.216 -12.240  1.00  0.00
ATOM   1572  C   LYS    99      -0.892 -10.633  -9.909  1.00  0.00
ATOM   1573  O   LYS    99      -1.581 -10.100 -10.755  1.00  0.00
ATOM   1574  N   GLN   100      -0.387 -11.825 -10.118  1.00  0.00
ATOM   1575  H   GLN   100       0.256 -12.230  -9.453  1.00  0.00
ATOM   1576  CA  GLN   100      -0.683 -12.566 -11.322  1.00  0.00
ATOM   1577  HA  GLN   100      -0.615 -11.913 -12.192  1.00  0.00
ATOM   1578  CB  GLN   100       0.325 -13.709 -11.487  1.00  0.00
ATOM   1579  HB2 GLN   100       1.234 -13.466 -10.937  1.00  0.00
ATOM   1580  HB3 GLN   100      -0.107 -14.627 -11.087  1.00  0.00
ATOM   1581  CG  GLN   100       0.663 -13.907 -12.969  1.00  0.00
ATOM   1582  HG2 GLN   100       0.796 -12.915 -13.400  1.00  0.00
ATOM   1583  HG3 GLN   100       1.585 -14.478 -13.080  1.00  0.00
ATOM   1584  CD  GLN   100      -0.447 -14.623 -13.729  1.00  0.00
ATOM   1585  OE1 GLN   100      -1.507 -14.932 -13.223  1.00  0.00
ATOM   1586  NE2 GLN   100      -0.183 -14.941 -14.974  1.00  0.00
ATOM   1587 HE21 GLN   100      -0.880 -15.417 -15.529  1.00  0.00
ATOM   1588 HE22 GLN   100       0.716 -14.709 -15.372  1.00  0.00
```

ATOM	1589	C	GLN	100	-2.107	-13.097	-11.284	1.00	0.00
ATOM	1590	O	GLN	100	-2.868	-12.886	-12.221	1.00	0.00
ATOM	1591	N	ILE	101	-2.458	-13.740	-10.170	1.00	0.00
ATOM	1592	H	ILE	101	-1.760	-13.929	-9.465	1.00	0.00
ATOM	1593	CA	ILE	101	-3.775	-14.290	-9.990	1.00	0.00
ATOM	1594	HA	ILE	101	-4.084	-14.903	-10.837	1.00	0.00
ATOM	1595	CB	ILE	101	-3.853	-15.124	-8.698	1.00	0.00
ATOM	1596	HB	ILE	101	-3.427	-16.109	-8.888	1.00	0.00
ATOM	1597	CG2	ILE	101	-3.084	-14.447	-7.574	1.00	0.00
ATOM	1598	HG21	ILE	101	-3.510	-13.462	-7.384	1.00	0.00
ATOM	1599	HG22	ILE	101	-3.152	-15.053	-6.671	1.00	0.00
ATOM	1600	HG23	ILE	101	-2.038	-14.341	-7.862	1.00	0.00
ATOM	1601	CG1	ILE	101	-5.308	-15.399	-8.284	1.00	0.00
ATOM	1602	HG12	ILE	101	-5.327	-16.259	-7.614	1.00	0.00
ATOM	1603	HG13	ILE	101	-5.697	-14.525	-7.763	1.00	0.00
ATOM	1604	CD1	ILE	101	-6.177	-15.690	-9.514	1.00	0.00
ATOM	1605	HD11	ILE	101	-5.788	-16.565	-10.035	1.00	0.00
ATOM	1606	HD12	ILE	101	-7.202	-15.882	-9.198	1.00	0.00
ATOM	1607	HD13	ILE	101	-6.159	-14.831	-10.184	1.00	0.00
ATOM	1608	C	ILE	101	-4.806	-13.159	-10.053	1.00	0.00
ATOM	1609	O	ILE	101	-5.330	-12.825	-11.110	1.00	0.00
ATOM	1610	N	GLY	102	-5.105	-12.544	-8.904	1.00	0.00

ATOM 1611 H GLY 102 -4.612 -12.810 -8.063 1.00 0.00

ATOM 1612 CA GLY 102 -6.103 -11.515 -8.812 1.00 0.00

ATOM 1613 HA2 GLY 102 -5.636 -10.553 -9.023 1.00 0.00

ATOM 1614 HA3 GLY 102 -6.879 -11.712 -9.552 1.00 0.00

ATOM 1615 C GLY 102 -6.731 -11.465 -7.446 1.00 0.00

ATOM 1616 O GLY 102 -7.829 -11.931 -7.211 1.00 0.00

ATOM 1617 N THR 103 -6.018 -10.873 -6.508 1.00 0.00

ATOM 1618 H THR 103 -5.102 -10.526 -6.753 1.00 0.00

ATOM 1619 CA THR 103 -6.451 -10.762 -5.120 1.00 0.00

ATOM 1620 HA THR 103 -7.009 -11.674 -4.906 1.00 0.00

ATOM 1621 CB THR 103 -5.211 -10.725 -4.223 1.00 0.00

ATOM 1622 HB THR 103 -5.227 -9.796 -3.654 1.00 0.00

ATOM 1623 CG2 THR 103 -5.174 -11.910 -3.261 1.00 0.00

ATOM 1624 HG21 THR 103 -5.158 -12.840 -3.830 1.00 0.00

ATOM 1625 HG22 THR 103 -4.279 -11.848 -2.642 1.00 0.00

ATOM 1626 HG23 THR 103 -6.058 -11.890 -2.624 1.00 0.00

ATOM 1627 OG1 THR 103 -4.059 -10.902 -5.047 1.00 0.00

ATOM 1628 HG1 THR 103 -3.269 -10.882 -4.502 1.00 0.00

ATOM 1629 C THR 103 -7.330 -9.538 -4.954 1.00 0.00

ATOM 1630 O THR 103 -8.448 -9.635 -4.450 1.00 0.00

ATOM 1631 N CYX 104 -6.842 -8.379 -5.417 1.00 0.00

ATOM 1632 H CYX 104 -5.883 -8.342 -5.732 1.00 0.00

```
ATOM   1633  CA  CYX   104      -7.604  -7.146  -5.388  1.00  0.00
ATOM   1634  HA  CYX   104      -8.643  -7.358  -5.136  1.00  0.00
ATOM   1635  CB  CYX   104      -7.030  -6.191  -4.337  1.00  0.00
ATOM   1636  HB2 CYX   104      -7.753  -5.403  -4.128  1.00  0.00
ATOM   1637  HB3 CYX   104      -6.820  -6.743  -3.421  1.00  0.00
ATOM   1638  SG  CYX   104      -5.455  -5.344  -4.753  1.00  0.00
ATOM   1639  C   CYX   104      -7.640  -6.544  -6.785  1.00  0.00
ATOM   1640  O   CYX   104      -7.029  -5.517  -7.054  1.00  0.00
ATOM   1641  N   PRO   105      -8.306  -7.221  -7.722  1.00  0.00
ATOM   1642  CD  PRO   105      -9.039  -8.461  -7.525  1.00  0.00
ATOM   1643  HD2 PRO   105      -9.890  -8.246  -6.878  1.00  0.00
ATOM   1644  HD3 PRO   105      -8.374  -9.167  -7.027  1.00  0.00
ATOM   1645  CG  PRO   105      -9.463  -8.940  -8.911  1.00  0.00
ATOM   1646  HG2 PRO   105     -10.552  -8.956  -8.965  1.00  0.00
ATOM   1647  HG3 PRO   105      -9.078  -9.947  -9.074  1.00  0.00
ATOM   1648  CB  PRO   105      -8.867  -7.957  -9.921  1.00  0.00
ATOM   1649  HB2 PRO   105      -9.592  -7.647 -10.673  1.00  0.00
ATOM   1650  HB3 PRO   105      -8.001  -8.399 -10.413  1.00  0.00
ATOM   1651  CA  PRO   105      -8.435  -6.752  -9.088  1.00  0.00
ATOM   1652  HA  PRO   105      -7.443  -6.472  -9.443  1.00  0.00
ATOM   1653  C   PRO   105      -9.372  -5.559  -9.207  1.00  0.00
ATOM   1654  O   PRO   105     -10.320  -5.562  -9.982  1.00  0.00
```

ATOM 1655 N PHE 106 -9.123 -4.534 -8.402 1.00 0.00
ATOM 1656 H PHE 106 -8.323 -4.583 -7.788 1.00 0.00
ATOM 1657 CA PHE 106 -9.929 -3.347 -8.376 1.00 0.00
ATOM 1658 HA PHE 106 -10.965 -3.570 -8.632 1.00 0.00
ATOM 1659 CB PHE 106 -9.887 -2.788 -6.958 1.00 0.00
ATOM 1660 HB2 PHE 106 -10.819 -2.285 -6.701 1.00 0.00
ATOM 1661 HB3 PHE 106 -9.719 -3.615 -6.268 1.00 0.00
ATOM 1662 CG PHE 106 -8.772 -1.787 -6.716 1.00 0.00
ATOM 1663 CD1 PHE 106 -7.448 -2.220 -6.553 1.00 0.00
ATOM 1664 HD1 PHE 106 -7.226 -3.287 -6.598 1.00 0.00
ATOM 1665 CE1 PHE 106 -6.419 -1.301 -6.335 1.00 0.00
ATOM 1666 HE1 PHE 106 -5.387 -1.639 -6.236 1.00 0.00
ATOM 1667 CZ PHE 106 -6.720 0.057 -6.245 1.00 0.00
ATOM 1668 HZ PHE 106 -5.933 0.772 -6.007 1.00 0.00
ATOM 1669 CE2 PHE 106 -8.029 0.503 -6.459 1.00 0.00
ATOM 1670 HE2 PHE 106 -8.248 1.570 -6.442 1.00 0.00
ATOM 1671 CD2 PHE 106 -9.053 -0.415 -6.694 1.00 0.00
ATOM 1672 HD2 PHE 106 -10.072 -0.065 -6.861 1.00 0.00
ATOM 1673 C PHE 106 -9.393 -2.314 -9.320 1.00 0.00
ATOM 1674 O PHE 106 -8.196 -2.347 -9.596 1.00 0.00
ATOM 1675 N ILE 107 -10.254 -1.389 -9.747 1.00 0.00
ATOM 1676 H ILE 107 -11.214 -1.440 -9.438 1.00 0.00

ATOM 1677 CA ILE 107 -9.890 -0.350 -10.693 1.00 0.00

ATOM 1678 HA ILE 107 -9.282 0.428 -10.232 1.00 0.00

ATOM 1679 CB ILE 107 -9.145 -0.927 -11.917 1.00 0.00

ATOM 1680 HB ILE 107 -9.459 -0.386 -12.810 1.00 0.00

ATOM 1681 CG2 ILE 107 -7.625 -0.785 -11.740 1.00 0.00

ATOM 1682 HG21 ILE 107 -7.311 -1.327 -10.848 1.00 0.00

ATOM 1683 HG22 ILE 107 -7.117 -1.197 -12.612 1.00 0.00

ATOM 1684 HG23 ILE 107 -7.368 0.269 -11.635 1.00 0.00

ATOM 1685 CG1 ILE 107 -9.545 -2.394 -12.192 1.00 0.00

ATOM 1686 HG12 ILE 107 -8.834 -3.048 -11.686 1.00 0.00

ATOM 1687 HG13 ILE 107 -10.543 -2.562 -11.788 1.00 0.00

ATOM 1688 CD1 ILE 107 -9.543 -2.698 -13.670 1.00 0.00

ATOM 1689 HD11 ILE 107 -8.545 -2.531 -14.075 1.00 0.00

ATOM 1690 HD12 ILE 107 -9.829 -3.738 -13.828 1.00 0.00

ATOM 1691 HD13 ILE 107 -10.254 -2.046 -14.177 1.00 0.00

ATOM 1692 C ILE 107 -11.122 0.411 -11.131 1.00 0.00

ATOM 1693 O ILE 107 -11.039 1.624 -11.266 1.00 0.00

ATOM 1694 N ALA 108 -12.237 -0.298 -11.343 1.00 0.00

ATOM 1695 H ALA 108 -12.191 -1.306 -11.292 1.00 0.00

ATOM 1696 CA ALA 108 -13.492 0.312 -11.718 1.00 0.00

ATOM 1697 HA ALA 108 -13.331 1.031 -12.521 1.00 0.00

ATOM 1698 CB ALA 108 -14.447 -0.771 -12.217 1.00 0.00

ATOM	1699	HB1	ALA	108	-14.626	-1.494	-11.421	1.00	0.00
ATOM	1700	HB2	ALA	108	-15.392	-0.315	-12.512	1.00	0.00
ATOM	1701	HB3	ALA	108	-14.006	-1.278	-13.075	1.00	0.00
ATOM	1702	C	ALA	108	-14.075	1.094	-10.542	1.00	0.00
ATOM	1703	O	ALA	108	-15.114	0.735	-10.001	1.00	0.00
ATOM	1704	N	ILE	109	-13.386	2.148	-10.134	1.00	0.00
ATOM	1705	H	ILE	109	-12.500	2.316	-10.588	1.00	0.00
ATOM	1706	CA	ILE	109	-13.748	2.979	-9.009	1.00	0.00
ATOM	1707	HA	ILE	109	-14.795	2.858	-8.729	1.00	0.00
ATOM	1708	CB	ILE	109	-12.855	2.695	-7.789	1.00	0.00
ATOM	1709	HB	ILE	109	-12.721	3.618	-7.226	1.00	0.00
ATOM	1710	CG2	ILE	109	-13.499	1.633	-6.884	1.00	0.00
ATOM	1711	HG21	ILE	109	-13.633	0.709	-7.446	1.00	0.00
ATOM	1712	HG22	ILE	109	-12.853	1.445	-6.027	1.00	0.00
ATOM	1713	HG23	ILE	109	-14.468	1.990	-6.537	1.00	0.00
ATOM	1714	CG1	ILE	109	-11.430	2.285	-8.188	1.00	0.00
ATOM	1715	HG12	ILE	109	-10.897	1.951	-7.298	1.00	0.00
ATOM	1716	HG13	ILE	109	-11.487	1.466	-8.905	1.00	0.00
ATOM	1717	CD1	ILE	109	-10.686	3.457	-8.815	1.00	0.00
ATOM	1718	HD11	ILE	109	-10.628	4.276	-8.099	1.00	0.00
ATOM	1719	HD12	ILE	109	-9.679	3.143	-9.090	1.00	0.00
ATOM	1720	HD13	ILE	109	-11.218	3.791	-9.706	1.00	0.00

```
ATOM   1721  C   ILE   109     -13.752   4.454  -9.440  1.00  0.00
ATOM   1722  O   ILE   109     -14.779   5.114  -9.254  1.00  0.00
ATOM   1723  OXT ILE   109     -12.739   4.949  -9.968  1.00  0.00
TER
ATOM   1724  S1  LIG   110      20.756  -8.510  -8.217  1.00  0.00
ATOM   1725  O1  LIG   110      20.927  -9.884  -7.373  1.00  0.00
ATOM   1726  O2  LIG   110      22.108  -8.645  -9.090  1.00  0.00
ATOM   1727  H26 LIG   110      22.877  -8.441  -8.535  1.00  0.00
ATOM   1728  O3  LIG   110      19.659  -8.627  -9.145  1.00  0.00
ATOM   1729  O4  LIG   110      20.869  -7.383  -7.314  1.00  0.00
ATOM   1730  C1  LIG   110      18.704 -15.171  -2.513  1.00  0.00
ATOM   1731  H1  LIG   110      18.901 -15.939  -3.272  1.00  0.00
ATOM   1732  H2  LIG   110      19.641 -15.048  -1.954  1.00  0.00
ATOM   1733  C2  LIG   110      17.611 -15.669  -1.561  1.00  0.00
ATOM   1734  H3  LIG   110      16.674 -15.792  -2.119  1.00  0.00
ATOM   1735  H4  LIG   110      17.413 -14.900  -0.803  1.00  0.00
ATOM   1736  C3  LIG   110      18.354 -13.851  -3.209  1.00  0.00
ATOM   1737  H5  LIG   110      18.159 -13.082  -2.450  1.00  0.00
ATOM   1738  H6  LIG   110      17.416 -13.974  -3.766  1.00  0.00
ATOM   1739  C4  LIG   110      17.959 -16.989  -0.863  1.00  0.00
ATOM   1740  H7  LIG   110      18.896 -16.865  -0.303  1.00  0.00
ATOM   1741  H8  LIG   110      18.158 -17.757  -1.620  1.00  0.00
```

ATOM 1742 C5 LIG 110 19.446 -13.354 -4.164 1.00 0.00

ATOM 1743 H9 LIG 110 20.385 -13.233 -3.609 1.00 0.00

ATOM 1744 H10 LIG 110 19.639 -14.120 -4.925 1.00 0.00

ATOM 1745 C6 LIG 110 16.866 -17.488 0.089 1.00 0.00

ATOM 1746 H11 LIG 110 15.929 -17.610 -0.470 1.00 0.00

ATOM 1747 H12 LIG 110 16.667 -16.719 0.847 1.00 0.00

ATOM 1748 C7 LIG 110 19.094 -12.033 -4.856 1.00 0.00

ATOM 1749 H13 LIG 110 18.154 -12.154 -5.409 1.00 0.00

ATOM 1750 H14 LIG 110 18.906 -11.265 -4.094 1.00 0.00

ATOM 1751 C8 LIG 110 17.213 -18.807 0.787 1.00 0.00

ATOM 1752 H15 LIG 110 17.411 -19.577 0.030 1.00 0.00

ATOM 1753 H16 LIG 110 18.150 -18.685 1.346 1.00 0.00

ATOM 1754 C9 LIG 110 20.192 -11.549 -5.813 1.00 0.00

ATOM 1755 H17 LIG 110 20.371 -12.300 -6.589 1.00 0.00

ATOM 1756 H18 LIG 110 21.133 -11.418 -5.268 1.00 0.00

ATOM 1757 C10 LIG 110 16.120 -19.307 1.739 1.00 0.00

ATOM 1758 H19 LIG 110 15.184 -19.429 1.181 1.00 0.00

ATOM 1759 H20 LIG 110 15.924 -18.538 2.497 1.00 0.00

ATOM 1760 C11 LIG 110 19.821 -10.237 -6.477 1.00 0.00

ATOM 1761 H21 LIG 110 18.912 -10.330 -7.077 1.00 0.00

ATOM 1762 H22 LIG 110 19.697 -9.433 -5.748 1.00 0.00

ATOM 1763 C12 LIG 110 16.477 -20.626 2.431 1.00 0.00

ATOM 1764 H23 LIG 110 17.391 -20.526 3.026 1.00 0.00
ATOM 1765 H24 LIG 110 15.679 -20.953 3.103 1.00 0.00
ATOM 1766 H25 LIG 110 16.644 -21.423 1.701 1.00 0.00
TER
ATOM 1767 S1 LIG 111 17.036 -11.130 26.543 1.00 0.00
ATOM 1768 O1 LIG 111 16.242 -10.699 25.196 1.00 0.00
ATOM 1769 O2 LIG 111 16.152 -10.262 27.579 1.00 0.00
ATOM 1770 H26 LIG 111 16.386 -9.325 27.503 1.00 0.00
ATOM 1771 O3 LIG 111 16.783 -12.515 26.851 1.00 0.00
ATOM 1772 O4 LIG 111 18.383 -10.598 26.488 1.00 0.00
ATOM 1773 C1 LIG 111 14.711 -11.006 17.843 1.00 0.00
ATOM 1774 H1 LIG 111 13.653 -11.177 18.079 1.00 0.00
ATOM 1775 H2 LIG 111 14.835 -9.918 17.774 1.00 0.00
ATOM 1776 C2 LIG 111 15.034 -11.638 16.484 1.00 0.00
ATOM 1777 H3 LIG 111 14.910 -12.727 16.552 1.00 0.00
ATOM 1778 H4 LIG 111 16.093 -11.468 16.248 1.00 0.00
ATOM 1779 C3 LIG 111 15.577 -11.542 18.988 1.00 0.00
ATOM 1780 H5 LIG 111 16.636 -11.369 18.753 1.00 0.00
ATOM 1781 H6 LIG 111 15.455 -12.631 19.055 1.00 0.00
ATOM 1782 C4 LIG 111 14.169 -11.103 15.336 1.00 0.00
ATOM 1783 H7 LIG 111 14.294 -10.015 15.267 1.00 0.00
ATOM 1784 H8 LIG 111 13.111 -11.272 15.572 1.00 0.00

```
ATOM   1785  C5  LIG   111      15.252 -10.913  20.348  1.00  0.00
ATOM   1786  H9  LIG   111      15.372  -9.824  20.281  1.00  0.00
ATOM   1787  H10 LIG   111      14.195 -11.089  20.585  1.00  0.00
ATOM   1788  C6  LIG   111      14.492 -11.735  13.978  1.00  0.00
ATOM   1789  H11 LIG   111      14.368 -12.823  14.047  1.00  0.00
ATOM   1790  H12 LIG   111      15.551 -11.566  13.741  1.00  0.00
ATOM   1791  C7  LIG   111      16.122 -11.449  21.490  1.00  0.00
ATOM   1792  H13 LIG   111      16.003 -12.538  21.554  1.00  0.00
ATOM   1793  H14 LIG   111      17.179 -11.268  21.254  1.00  0.00
ATOM   1794  C8  LIG   111      13.628 -11.201  12.830  1.00  0.00
ATOM   1795  H15 LIG   111      12.568 -11.371  13.064  1.00  0.00
ATOM   1796  H16 LIG   111      13.751 -10.112  12.761  1.00  0.00
ATOM   1797  C9  LIG   111      15.783 -10.816  22.846  1.00  0.00
ATOM   1798  H17 LIG   111      14.736 -11.009  23.100  1.00  0.00
ATOM   1799  H18 LIG   111      15.906  -9.729  22.793  1.00  0.00
ATOM   1800  C10 LIG   111      13.949 -11.832  11.470  1.00  0.00
ATOM   1801  H19 LIG   111      13.826 -12.920  11.541  1.00  0.00
ATOM   1802  H20 LIG   111      15.007 -11.661  11.235  1.00  0.00
ATOM   1803  C11 LIG   111      16.664 -11.358  23.955  1.00  0.00
ATOM   1804  H21 LIG   111      16.538 -12.436  24.081  1.00  0.00
ATOM   1805  H22 LIG   111      17.719 -11.130  23.785  1.00  0.00
ATOM   1806  C12 LIG   111      13.079 -11.292  10.331  1.00  0.00
```

```
ATOM   1807  H23 LIG   111      13.207 -10.211  10.215  1.00  0.00
ATOM   1808  H24 LIG   111      13.335 -11.760   9.377  1.00  0.00
ATOM   1809  H25 LIG   111      12.018 -11.480  10.520  1.00  0.00
TER
ATOM   1810  S1  LIG   112     -19.419  18.689 -26.943  1.00  0.00
ATOM   1811  O1  LIG   112     -19.642  18.067 -25.462  1.00  0.00
ATOM   1812  O2  LIG   112     -18.663  17.413 -27.581  1.00  0.00
ATOM   1813  H26 LIG   112     -17.755  17.369 -27.245  1.00  0.00
ATOM   1814  O3  LIG   112     -20.680  18.804 -27.632  1.00  0.00
ATOM   1815  O4  LIG   112     -18.499  19.805 -26.853  1.00  0.00
ATOM   1816  C1  LIG   112     -22.329  18.085 -18.441  1.00  0.00
ATOM   1817  H1  LIG   112     -22.778  17.100 -18.619  1.00  0.00
ATOM   1818  H2  LIG   112     -21.319  17.893 -18.056  1.00  0.00
ATOM   1819  C2  LIG   112     -23.142  18.826 -17.373  1.00  0.00
ATOM   1820  H3  LIG   112     -24.152  19.018 -17.757  1.00  0.00
ATOM   1821  H4  LIG   112     -22.693  19.812 -17.195  1.00  0.00
ATOM   1822  C3  LIG   112     -22.228  18.843 -19.769  1.00  0.00
ATOM   1823  H5  LIG   112     -21.776  19.828 -19.591  1.00  0.00
ATOM   1824  H6  LIG   112     -23.238  19.037 -20.152  1.00  0.00
ATOM   1825  C4  LIG   112     -23.244  18.070 -16.043  1.00  0.00
ATOM   1826  H7  LIG   112     -22.233  17.879 -15.658  1.00  0.00
ATOM   1827  H8  LIG   112     -23.691  17.084 -16.221  1.00  0.00
```

```
ATOM   1828  C5  LIG   112     -21.417  18.101 -20.837  1.00  0.00
ATOM   1829  H9  LIG   112     -20.407  17.904 -20.455  1.00  0.00
ATOM   1830  H10 LIG   112     -21.870  17.118 -21.019  1.00  0.00
ATOM   1831  C6  LIG   112     -24.057  18.811 -14.976  1.00  0.00
ATOM   1832  H11 LIG   112     -25.067  19.003 -15.361  1.00  0.00
ATOM   1833  H12 LIG   112     -23.609  19.797 -14.798  1.00  0.00
ATOM   1834  C7  LIG   112     -21.316  18.863 -22.163  1.00  0.00
ATOM   1835  H13 LIG   112     -22.326  19.061 -22.544  1.00  0.00
ATOM   1836  H14 LIG   112     -20.858  19.843 -21.982  1.00  0.00
ATOM   1837  C8  LIG   112     -24.159  18.056 -13.646  1.00  0.00
ATOM   1838  H15 LIG   112     -24.609  17.069 -13.823  1.00  0.00
ATOM   1839  H16 LIG   112     -23.149  17.863 -13.261  1.00  0.00
ATOM   1840  C9  LIG   112     -20.507  18.107 -23.226  1.00  0.00
ATOM   1841  H17 LIG   112     -20.969  17.135 -23.429  1.00  0.00
ATOM   1842  H18 LIG   112     -19.495  17.911 -22.858  1.00  0.00
ATOM   1843  C10 LIG   112     -24.973  18.795 -12.578  1.00  0.00
ATOM   1844  H19 LIG   112     -25.982  18.988 -12.963  1.00  0.00
ATOM   1845  H20 LIG   112     -24.523  19.780 -12.400  1.00  0.00
ATOM   1846  C11 LIG   112     -20.417  18.887 -24.524  1.00  0.00
ATOM   1847  H21 LIG   112     -21.404  19.063 -24.959  1.00  0.00
ATOM   1848  H22 LIG   112     -19.900  19.840 -24.390  1.00  0.00
ATOM   1849  C12 LIG   112     -25.069  18.032 -11.254  1.00  0.00
```

```
ATOM   1850  H23 LIG   112     -24.077  17.854 -10.827  1.00  0.00
ATOM   1851  H24 LIG   112     -25.654  18.586 -10.514  1.00  0.00
ATOM   1852  H25 LIG   112     -25.548  17.058 -11.392  1.00  0.00
TER
ATOM   1853  S1  LIG   113     -23.363 -23.592 -11.157  1.00  0.00
ATOM   1854  O1  LIG   113     -22.498 -22.827 -12.296  1.00  0.00
ATOM   1855  O2  LIG   113     -24.788 -23.523 -11.913  1.00  0.00
ATOM   1856  H26 LIG   113     -25.143 -22.622 -11.859  1.00  0.00
ATOM   1857  O3  LIG   113     -22.997 -24.986 -11.103  1.00  0.00
ATOM   1858  O4  LIG   113     -23.404 -22.772  -9.963  1.00  0.00
ATOM   1859  C1  LIG   113     -16.152 -20.238 -15.383  1.00  0.00
ATOM   1860  H1  LIG   113     -16.405 -20.755 -16.317  1.00  0.00
ATOM   1861  H2  LIG   113     -16.598 -19.239 -15.458  1.00  0.00
ATOM   1862  C2  LIG   113     -14.628 -20.103 -15.277  1.00  0.00
ATOM   1863  H3  LIG   113     -14.181 -21.103 -15.202  1.00  0.00
ATOM   1864  H4  LIG   113     -14.374 -19.587 -14.342  1.00  0.00
ATOM   1865  C3  LIG   113     -16.782 -20.987 -14.204  1.00  0.00
ATOM   1866  H5  LIG   113     -16.531 -20.468 -13.269  1.00  0.00
ATOM   1867  H6  LIG   113     -16.333 -21.986 -14.128  1.00  0.00
ATOM   1868  C4  LIG   113     -13.994 -19.354 -16.455  1.00  0.00
ATOM   1869  H7  LIG   113     -14.441 -18.353 -16.529  1.00  0.00
ATOM   1870  H8  LIG   113     -14.249 -19.869 -17.390  1.00  0.00
```

```
ATOM   1871  C5  LIG   113     -18.305 -21.125 -14.310  1.00  0.00
ATOM   1872  H9  LIG   113     -18.755 -20.127 -14.389  1.00  0.00
ATOM   1873  H10 LIG   113     -18.558 -21.647 -15.242  1.00  0.00
ATOM   1874  C6  LIG   113     -12.471 -19.219 -16.350  1.00  0.00
ATOM   1875  H11 LIG   113     -12.025 -20.219 -16.275  1.00  0.00
ATOM   1876  H12 LIG   113     -12.217 -18.703 -15.415  1.00  0.00
ATOM   1877  C7  LIG   113     -18.933 -21.871 -13.128  1.00  0.00
ATOM   1878  H13 LIG   113     -18.481 -22.868 -13.049  1.00  0.00
ATOM   1879  H14 LIG   113     -18.685 -21.346 -12.197  1.00  0.00
ATOM   1880  C8  LIG   113     -11.837 -18.470 -17.527  1.00  0.00
ATOM   1881  H15 LIG   113     -12.090 -18.986 -18.464  1.00  0.00
ATOM   1882  H16 LIG   113     -12.284 -17.470 -17.603  1.00  0.00
ATOM   1883  C9  LIG   113     -20.457 -22.008 -13.250  1.00  0.00
ATOM   1884  H17 LIG   113     -20.714 -22.550 -14.166  1.00  0.00
ATOM   1885  H18 LIG   113     -20.917 -21.017 -13.322  1.00  0.00
ATOM   1886  C10 LIG   113     -10.313 -18.335 -17.424  1.00  0.00
ATOM   1887  H19 LIG   113      -9.868 -19.334 -17.348  1.00  0.00
ATOM   1888  H20 LIG   113     -10.061 -17.819 -16.489  1.00  0.00
ATOM   1889  C11 LIG   113     -21.052 -22.743 -12.064  1.00  0.00
ATOM   1890  H21 LIG   113     -20.660 -23.759 -11.980  1.00  0.00
ATOM   1891  H22 LIG   113     -20.883 -22.206 -11.127  1.00  0.00
ATOM   1892  C12 LIG   113      -9.690 -17.586 -18.605  1.00  0.00
```

ATOM 1893 H23 LIG 113 -10.092 -16.571 -18.686 1.00 0.00

ATOM 1894 H24 LIG 113 -8.605 -17.505 -18.500 1.00 0.00

ATOM 1895 H25 LIG 113 -9.895 -18.097 -19.551 1.00 0.00

TER

ATOM 1896 S1 LIG 114 0.406 12.640 -20.513 1.00 0.00

ATOM 1897 O1 LIG 114 0.467 13.293 -19.030 1.00 0.00

ATOM 1898 O2 LIG 114 1.079 11.222 -20.131 1.00 0.00

ATOM 1899 H26 LIG 114 2.033 11.337 -20.005 1.00 0.00

ATOM 1900 O3 LIG 114 -0.960 12.358 -20.879 1.00 0.00

ATOM 1901 O4 LIG 114 1.290 13.378 -21.392 1.00 0.00

ATOM 1902 C1 LIG 114 -0.943 18.611 -13.907 1.00 0.00

ATOM 1903 H1 LIG 114 -1.385 17.851 -13.251 1.00 0.00

ATOM 1904 H2 LIG 114 0.124 18.648 -13.655 1.00 0.00

ATOM 1905 C2 LIG 114 -1.581 19.972 -13.604 1.00 0.00

ATOM 1906 H3 LIG 114 -2.648 19.936 -13.856 1.00 0.00

ATOM 1907 H4 LIG 114 -1.140 20.734 -14.260 1.00 0.00

ATOM 1908 C3 LIG 114 -1.103 18.174 -15.367 1.00 0.00

ATOM 1909 H5 LIG 114 -0.659 18.934 -16.024 1.00 0.00

ATOM 1910 H6 LIG 114 -2.171 18.140 -15.620 1.00 0.00

ATOM 1911 C4 LIG 114 -1.422 20.413 -12.144 1.00 0.00

ATOM 1912 H7 LIG 114 -0.354 20.451 -11.891 1.00 0.00

ATOM 1913 H8 LIG 114 -1.861 19.652 -11.487 1.00 0.00

ATOM	1914	C5	LIG	114	-0.468	16.812	-15.671	1.00	0.00
ATOM	1915	H9	LIG	114	0.599	16.844	-15.416	1.00	0.00
ATOM	1916	H10	LIG	114	-0.914	16.050	-15.018	1.00	0.00
ATOM	1917	C6	LIG	114	-2.060	21.773	-11.839	1.00	0.00
ATOM	1918	H11	LIG	114	-3.127	21.736	-12.092	1.00	0.00
ATOM	1919	H12	LIG	114	-1.619	22.535	-12.497	1.00	0.00
ATOM	1920	C7	LIG	114	-0.627	16.378	-17.132	1.00	0.00
ATOM	1921	H13	LIG	114	-1.694	16.349	-17.387	1.00	0.00
ATOM	1922	H14	LIG	114	-0.177	17.138	-17.785	1.00	0.00
ATOM	1923	C8	LIG	114	-1.901	22.214	-10.380	1.00	0.00
ATOM	1924	H15	LIG	114	-2.342	21.454	-9.721	1.00	0.00
ATOM	1925	H16	LIG	114	-0.834	22.251	-10.127	1.00	0.00
ATOM	1926	C9	LIG	114	0.007	15.011	-17.422	1.00	0.00
ATOM	1927	H17	LIG	114	-0.451	14.243	-16.790	1.00	0.00
ATOM	1928	H18	LIG	114	1.074	15.034	-17.178	1.00	0.00
ATOM	1929	C10	LIG	114	-2.539	23.575	-10.074	1.00	0.00
ATOM	1930	H19	LIG	114	-3.605	23.537	-10.328	1.00	0.00
ATOM	1931	H20	LIG	114	-2.097	24.334	-10.731	1.00	0.00
ATOM	1932	C11	LIG	114	-0.158	14.611	-18.876	1.00	0.00
ATOM	1933	H21	LIG	114	-1.211	14.525	-19.155	1.00	0.00
ATOM	1934	H22	LIG	114	0.343	15.312	-19.549	1.00	0.00
ATOM	1935	C12	LIG	114	-2.376	24.005	-8.613	1.00	0.00

```
ATOM   1936  H23 LIG   114      -1.318  24.084  -8.341  1.00  0.00
ATOM   1937  H24 LIG   114      -2.839  24.978  -8.430  1.00  0.00
ATOM   1938  H25 LIG   114      -2.838  23.283  -7.934  1.00  0.00
TER
ATOM   1939  S1  LIG   115      11.892  -1.318  17.091  1.00  0.00
ATOM   1940  O1  LIG   115      12.752  -0.186  16.311  1.00  0.00
ATOM   1941  O2  LIG   115      11.579  -0.449  18.416  1.00  0.00
ATOM   1942  H26 LIG   115      12.376  -0.393  18.965  1.00  0.00
ATOM   1943  O3  LIG   115      10.626  -1.526  16.432  1.00  0.00
ATOM   1944  O4  LIG   115      12.763  -2.425  17.429  1.00  0.00
ATOM   1945  C1  LIG   115      16.502   2.615  10.428  1.00  0.00
ATOM   1946  H1  LIG   115      15.876   3.516  10.469  1.00  0.00
ATOM   1947  H2  LIG   115      17.365   2.816  11.076  1.00  0.00
ATOM   1948  C2  LIG   115      16.989   2.403   8.990  1.00  0.00
ATOM   1949  H3  LIG   115      16.127   2.203   8.342  1.00  0.00
ATOM   1950  H4  LIG   115      17.615   1.502   8.949  1.00  0.00
ATOM   1951  C3  LIG   115      15.714   1.426  10.989  1.00  0.00
ATOM   1952  H5  LIG   115      16.341   0.526  10.950  1.00  0.00
ATOM   1953  H6  LIG   115      14.852   1.225  10.338  1.00  0.00
ATOM   1954  C4  LIG   115      17.779   3.590   8.427  1.00  0.00
ATOM   1955  H7  LIG   115      18.643   3.790   9.075  1.00  0.00
ATOM   1956  H8  LIG   115      17.154   4.492   8.469  1.00  0.00
```

ATOM	1957	C5	LIG	115	15.223	1.640	12.425	1.00	0.00
ATOM	1958	H9	LIG	115	16.083	1.843	13.076	1.00	0.00
ATOM	1959	H10	LIG	115	14.593	2.537	12.464	1.00	0.00
ATOM	1960	C6	LIG	115	18.266	3.379	6.989	1.00	0.00
ATOM	1961	H11	LIG	115	17.403	3.179	6.342	1.00	0.00
ATOM	1962	H12	LIG	115	18.891	2.477	6.948	1.00	0.00
ATOM	1963	C7	LIG	115	14.438	0.448	12.983	1.00	0.00
ATOM	1964	H13	LIG	115	13.579	0.243	12.331	1.00	0.00
ATOM	1965	H14	LIG	115	15.070	-0.449	12.949	1.00	0.00
ATOM	1966	C8	LIG	115	19.055	4.566	6.426	1.00	0.00
ATOM	1967	H15	LIG	115	18.431	5.469	6.466	1.00	0.00
ATOM	1968	H16	LIG	115	19.918	4.767	7.075	1.00	0.00
ATOM	1969	C9	LIG	115	13.948	0.675	14.420	1.00	0.00
ATOM	1970	H17	LIG	115	13.298	1.556	14.460	1.00	0.00
ATOM	1971	H18	LIG	115	14.799	0.873	15.079	1.00	0.00
ATOM	1972	C10	LIG	115	19.543	4.356	4.988	1.00	0.00
ATOM	1973	H19	LIG	115	18.680	4.155	4.342	1.00	0.00
ATOM	1974	H20	LIG	115	20.168	3.455	4.948	1.00	0.00
ATOM	1975	C11	LIG	115	13.181	-0.521	14.949	1.00	0.00
ATOM	1976	H21	LIG	115	12.291	-0.728	14.351	1.00	0.00
ATOM	1977	H22	LIG	115	13.807	-1.417	14.995	1.00	0.00
ATOM	1978	C12	LIG	115	20.330	5.548	4.435	1.00	0.00

ATOM	1979	H23	LIG	115	21.218	5.751	5.042	1.00	0.00
ATOM	1980	H24	LIG	115	20.664	5.365	3.410	1.00	0.00
ATOM	1981	H25	LIG	115	19.719	6.456	4.428	1.00	0.00
TER									
ATOM	1982	S1	LIG	116	24.340	1.745	-16.820	1.00	0.00
ATOM	1983	O1	LIG	116	23.954	2.141	-15.296	1.00	0.00
ATOM	1984	O2	LIG	116	24.743	3.229	-17.313	1.00	0.00
ATOM	1985	H26	LIG	116	23.942	3.759	-17.443	1.00	0.00
ATOM	1986	O3	LIG	116	25.551	0.964	-16.843	1.00	0.00
ATOM	1987	O4	LIG	116	23.143	1.310	-17.513	1.00	0.00
ATOM	1988	C1	LIG	116	21.922	0.643	-8.215	1.00	0.00
ATOM	1989	H1	LIG	116	22.779	1.224	-7.855	1.00	0.00
ATOM	1990	H2	LIG	116	21.090	1.353	-8.306	1.00	0.00
ATOM	1991	C2	LIG	116	21.562	-0.426	-7.175	1.00	0.00
ATOM	1992	H3	LIG	116	22.393	-1.136	-7.084	1.00	0.00
ATOM	1993	H4	LIG	116	20.704	-1.008	-7.536	1.00	0.00
ATOM	1994	C3	LIG	116	22.247	0.066	-9.597	1.00	0.00
ATOM	1995	H5	LIG	116	21.387	-0.515	-9.959	1.00	0.00
ATOM	1996	H6	LIG	116	23.077	-0.647	-9.505	1.00	0.00
ATOM	1997	C4	LIG	116	21.235	0.149	-5.792	1.00	0.00
ATOM	1998	H7	LIG	116	20.402	0.859	-5.883	1.00	0.00
ATOM	1999	H8	LIG	116	22.092	0.733	-5.432	1.00	0.00

ATOM	2000	C5	LIG	116	22.609	1.132	-10.636	1.00	0.00
ATOM	2001	H9	LIG	116	21.781	1.847	-10.727	1.00	0.00
ATOM	2002	H10	LIG	116	23.471	1.710	-10.277	1.00	0.00
ATOM	2003	C6	LIG	116	20.876	-0.918	-4.752	1.00	0.00
ATOM	2004	H11	LIG	116	21.708	-1.628	-4.661	1.00	0.00
ATOM	2005	H12	LIG	116	20.018	-1.501	-5.113	1.00	0.00
ATOM	2006	C7	LIG	116	22.931	0.552	-12.018	1.00	0.00
ATOM	2007	H13	LIG	116	23.758	-0.163	-11.925	1.00	0.00
ATOM	2008	H14	LIG	116	22.068	-0.021	-12.378	1.00	0.00
ATOM	2009	C8	LIG	116	20.550	-0.344	-3.369	1.00	0.00
ATOM	2010	H15	LIG	116	21.407	0.239	-3.006	1.00	0.00
ATOM	2011	H16	LIG	116	19.718	0.367	-3.459	1.00	0.00
ATOM	2012	C9	LIG	116	23.298	1.630	-13.047	1.00	0.00
ATOM	2013	H17	LIG	116	24.173	2.193	-12.705	1.00	0.00
ATOM	2014	H18	LIG	116	22.475	2.344	-13.151	1.00	0.00
ATOM	2015	C10	LIG	116	20.190	-1.411	-2.328	1.00	0.00
ATOM	2016	H19	LIG	116	21.022	-2.120	-2.238	1.00	0.00
ATOM	2017	H20	LIG	116	19.333	-1.992	-2.689	1.00	0.00
ATOM	2018	C11	LIG	116	23.604	1.029	-14.405	1.00	0.00
ATOM	2019	H21	LIG	116	24.455	0.344	-14.363	1.00	0.00
ATOM	2020	H22	LIG	116	22.738	0.510	-14.825	1.00	0.00
ATOM	2021	C12	LIG	116	19.867	-0.827	-0.949	1.00	0.00

```
ATOM   2022  H23 LIG   116      19.017  -0.138  -1.002  1.00  0.00
ATOM   2023  H24 LIG   116      19.613  -1.612  -0.232  1.00  0.00
ATOM   2024  H25 LIG   116      20.718  -0.270  -0.546  1.00  0.00
TER
ATOM   2025  S1  LIG   117     -20.265 -13.420  14.722  1.00  0.00
ATOM   2026  O1  LIG   117     -19.130 -12.532  15.466  1.00  0.00
ATOM   2027  O2  LIG   117     -19.710 -14.868  15.174  1.00  0.00
ATOM   2028  H26 LIG   117     -18.919 -15.089  14.660  1.00  0.00
ATOM   2029  O3  LIG   117     -21.539 -13.250  15.375  1.00  0.00
ATOM   2030  O4  LIG   117     -20.120 -13.268  13.288  1.00  0.00
ATOM   2031  C1  LIG   117     -15.970  -6.100  17.736  1.00  0.00
ATOM   2032  H1  LIG   117     -15.948  -6.484  18.764  1.00  0.00
ATOM   2033  H2  LIG   117     -15.021  -6.407  17.277  1.00  0.00
ATOM   2034  C2  LIG   117     -16.043  -4.569  17.769  1.00  0.00
ATOM   2035  H3  LIG   117     -16.991  -4.262  18.229  1.00  0.00
ATOM   2036  H4  LIG   117     -16.066  -4.185  16.741  1.00  0.00
ATOM   2037  C3  LIG   117     -17.132  -6.752  16.979  1.00  0.00
ATOM   2038  H5  LIG   117     -17.153  -6.370  15.949  1.00  0.00
ATOM   2039  H6  LIG   117     -18.080  -6.443  17.437  1.00  0.00
ATOM   2040  C4  LIG   117     -14.881  -3.915  18.526  1.00  0.00
ATOM   2041  H7  LIG   117     -13.932  -4.221  18.066  1.00  0.00
ATOM   2042  H8  LIG   117     -14.857  -4.299  19.553  1.00  0.00
```

```
ATOM   2043  C5  LIG   117     -17.061  -8.283  16.948  1.00  0.00
ATOM   2044  H9  LIG   117     -16.112  -8.593  16.492  1.00  0.00
ATOM   2045  H10 LIG   117     -17.044  -8.666  17.976  1.00  0.00
ATOM   2046  C6  LIG   117     -14.953  -2.384  18.560  1.00  0.00
ATOM   2047  H11 LIG   117     -15.902  -2.078  19.019  1.00  0.00
ATOM   2048  H12 LIG   117     -14.977  -2.000  17.531  1.00  0.00
ATOM   2049  C7  LIG   117     -18.223  -8.931  16.187  1.00  0.00
ATOM   2050  H13 LIG   117     -19.172  -8.618  16.642  1.00  0.00
ATOM   2051  H14 LIG   117     -18.236  -8.551  15.157  1.00  0.00
ATOM   2052  C8  LIG   117     -13.792  -1.730  19.316  1.00  0.00
ATOM   2053  H15 LIG   117     -13.768  -2.112  20.345  1.00  0.00
ATOM   2054  H16 LIG   117     -12.843  -2.036  18.857  1.00  0.00
ATOM   2055  C9  LIG   117     -18.144 -10.463  16.168  1.00  0.00
ATOM   2056  H17 LIG   117     -18.152 -10.852  17.192  1.00  0.00
ATOM   2057  H18 LIG   117     -17.203 -10.784  15.709  1.00  0.00
ATOM   2058  C10 LIG   117     -13.864  -0.199  19.351  1.00  0.00
ATOM   2059  H19 LIG   117     -14.812   0.107  19.809  1.00  0.00
ATOM   2060  H20 LIG   117     -13.886   0.184  18.323  1.00  0.00
ATOM   2061  C11 LIG   117     -19.301 -11.077  15.403  1.00  0.00
ATOM   2062  H21 LIG   117     -20.263 -10.826  15.855  1.00  0.00
ATOM   2063  H22 LIG   117     -19.299 -10.774  14.353  1.00  0.00
ATOM   2064  C12 LIG   117     -12.699   0.445  20.109  1.00  0.00
```

```
ATOM   2065  H23 LIG   117     -11.739   0.185  19.653  1.00  0.00
ATOM   2066  H24 LIG   117     -12.780   1.535  20.114  1.00  0.00
ATOM   2067  H25 LIG   117     -12.672   0.110  21.151  1.00  0.00
TER
ATOM   2068  S1  LIG   118       9.217   9.957  13.878  1.00  0.00
ATOM   2069  O1  LIG   118      10.120  11.134  13.223  1.00  0.00
ATOM   2070  O2  LIG   118       7.783  10.670  13.668  1.00  0.00
ATOM   2071  H26 LIG   118       7.675  11.385  14.313  1.00  0.00
ATOM   2072  O3  LIG   118       9.222   8.792  13.030  1.00  0.00
ATOM   2073  O4  LIG   118       9.513   9.867  15.294  1.00  0.00
ATOM   2074  C1  LIG   118      16.547  14.180  10.788  1.00  0.00
ATOM   2075  H1  LIG   118      16.073  14.386   9.820  1.00  0.00
ATOM   2076  H2  LIG   118      16.363  15.063  11.412  1.00  0.00
ATOM   2077  C2  LIG   118      18.058  14.012  10.585  1.00  0.00
ATOM   2078  H3  LIG   118      18.243  13.129   9.960  1.00  0.00
ATOM   2079  H4  LIG   118      18.533  13.805  11.553  1.00  0.00
ATOM   2080  C3  LIG   118      15.877  12.959  11.428  1.00  0.00
ATOM   2081  H5  LIG   118      16.351  12.753  12.398  1.00  0.00
ATOM   2082  H6  LIG   118      16.065  12.075  10.805  1.00  0.00
ATOM   2083  C4  LIG   118      18.730  15.232   9.945  1.00  0.00
ATOM   2084  H7  LIG   118      18.546  16.116  10.570  1.00  0.00
ATOM   2085  H8  LIG   118      18.255  15.441   8.978  1.00  0.00
```

ATOM	2086	C5	LIG	118	14.367	13.124	11.629	1.00	0.00
ATOM	2087	H9	LIG	118	14.178	14.010	12.250	1.00	0.00
ATOM	2088	H10	LIG	118	13.892	13.326	10.660	1.00	0.00
ATOM	2089	C6	LIG	118	20.240	15.065	9.741	1.00	0.00
ATOM	2090	H11	LIG	118	20.425	14.182	9.117	1.00	0.00
ATOM	2091	H12	LIG	118	20.716	14.857	10.709	1.00	0.00
ATOM	2092	C7	LIG	118	13.701	11.903	12.272	1.00	0.00
ATOM	2093	H13	LIG	118	13.892	11.019	11.652	1.00	0.00
ATOM	2094	H14	LIG	118	14.172	11.705	13.243	1.00	0.00
ATOM	2095	C8	LIG	118	20.913	16.285	9.101	1.00	0.00
ATOM	2096	H15	LIG	118	20.440	16.494	8.132	1.00	0.00
ATOM	2097	H16	LIG	118	20.728	17.169	9.725	1.00	0.00
ATOM	2098	C9	LIG	118	12.188	12.079	12.462	1.00	0.00
ATOM	2099	H17	LIG	118	11.706	12.256	11.495	1.00	0.00
ATOM	2100	H18	LIG	118	11.988	12.956	13.087	1.00	0.00
ATOM	2101	C10	LIG	118	22.424	16.119	8.896	1.00	0.00
ATOM	2102	H19	LIG	118	22.608	15.235	8.274	1.00	0.00
ATOM	2103	H20	LIG	118	22.897	15.912	9.864	1.00	0.00
ATOM	2104	C11	LIG	118	11.557	10.860	13.107	1.00	0.00
ATOM	2105	H21	LIG	118	11.686	9.965	12.494	1.00	0.00
ATOM	2106	H22	LIG	118	11.955	10.678	14.109	1.00	0.00
ATOM	2107	C12	LIG	118	23.086	17.342	8.256	1.00	0.00

```
ATOM   2108  H23 LIG   118      22.948  18.235   8.874  1.00  0.00
ATOM   2109  H24 LIG   118      24.161  17.193   8.126  1.00  0.00
ATOM   2110  H25 LIG   118      22.659  17.553   7.271  1.00  0.00
TER
ATOM   2111  S1  LIG   119       5.357 -20.549   6.966  1.00  0.00
ATOM   2112  O1  LIG   119       4.345 -21.084   8.115  1.00  0.00
ATOM   2113  O2  LIG   119       6.720 -21.101   7.632  1.00  0.00
ATOM   2114  H26 LIG   119       6.965 -20.542   8.386  1.00  0.00
ATOM   2115  O3  LIG   119       5.168 -21.295   5.747  1.00  0.00
ATOM   2116  O4  LIG   119       5.361 -19.100   6.976  1.00  0.00
ATOM   2117  C1  LIG   119      -2.427 -22.343  11.125  1.00  0.00
ATOM   2118  H1  LIG   119      -2.163 -23.399  11.258  1.00  0.00
ATOM   2119  H2  LIG   119      -2.111 -21.833  12.045  1.00  0.00
ATOM   2120  C2  LIG   119      -3.948 -22.217  10.977  1.00  0.00
ATOM   2121  H3  LIG   119      -4.265 -22.727  10.058  1.00  0.00
ATOM   2122  H4  LIG   119      -4.213 -21.160  10.843  1.00  0.00
ATOM   2123  C3  LIG   119      -1.647 -21.771   9.937  1.00  0.00
ATOM   2124  H5  LIG   119      -1.910 -20.713   9.805  1.00  0.00
ATOM   2125  H6  LIG   119      -1.966 -22.280   9.017  1.00  0.00
ATOM   2126  C4  LIG   119      -4.731 -22.787  12.165  1.00  0.00
ATOM   2127  H7  LIG   119      -4.415 -22.276  13.085  1.00  0.00
ATOM   2128  H8  LIG   119      -4.465 -23.844  12.299  1.00  0.00
```

ATOM 2129 C5 LIG 119 -0.127 -21.900 10.083 1.00 0.00
ATOM 2130 H9 LIG 119 0.193 -21.394 11.003 1.00 0.00
ATOM 2131 H10 LIG 119 0.138 -22.957 10.210 1.00 0.00
ATOM 2132 C6 LIG 119 -6.251 -22.663 12.017 1.00 0.00
ATOM 2133 H11 LIG 119 -6.567 -23.172 11.098 1.00 0.00
ATOM 2134 H12 LIG 119 -6.517 -21.605 11.883 1.00 0.00
ATOM 2135 C7 LIG 119 0.650 -21.325 8.894 1.00 0.00
ATOM 2136 H13 LIG 119 0.329 -21.830 7.974 1.00 0.00
ATOM 2137 H14 LIG 119 0.390 -20.266 8.771 1.00 0.00
ATOM 2138 C8 LIG 119 -7.034 -23.232 13.204 1.00 0.00
ATOM 2139 H15 LIG 119 -6.771 -24.290 13.339 1.00 0.00
ATOM 2140 H16 LIG 119 -6.718 -22.723 14.124 1.00 0.00
ATOM 2141 C9 LIG 119 2.171 -21.464 9.050 1.00 0.00
ATOM 2142 H17 LIG 119 2.442 -22.520 9.153 1.00 0.00
ATOM 2143 H18 LIG 119 2.502 -20.957 9.962 1.00 0.00
ATOM 2144 C10 LIG 119 -8.555 -23.108 13.057 1.00 0.00
ATOM 2145 H19 LIG 119 -8.870 -23.617 12.138 1.00 0.00
ATOM 2146 H20 LIG 119 -8.819 -22.052 12.924 1.00 0.00
ATOM 2147 C11 LIG 119 2.914 -20.881 7.864 1.00 0.00
ATOM 2148 H21 LIG 119 2.654 -21.391 6.933 1.00 0.00
ATOM 2149 H22 LIG 119 2.731 -19.808 7.757 1.00 0.00
ATOM 2150 C12 LIG 119 -9.328 -23.681 14.249 1.00 0.00

ATOM 2151 H23 LIG 119 -9.059 -23.169 15.178 1.00 0.00

ATOM 2152 H24 LIG 119 -10.408 -23.575 14.113 1.00 0.00

ATOM 2153 H25 LIG 119 -9.113 -24.745 14.385 1.00 0.00

TER

END

Table L: The number of atoms present in the system of BN-Sum

ATOM 1 N GLN 1 -7.420 -12.315 1.093 1.00 0.00

ATOM 2 H1 GLN 1 -6.489 -12.646 1.302 1.00 0.00

ATOM 3 H2 GLN 1 -7.651 -11.569 1.733 1.00 0.00

ATOM 4 H3 GLN 1 -7.497 -12.168 0.097 1.00 0.00

ATOM 5 CA GLN 1 -8.365 -13.393 1.399 1.00 0.00

ATOM 6 HA GLN 1 -9.067 -13.055 2.161 1.00 0.00

ATOM 7 CB GLN 1 -9.158 -13.809 0.152 1.00 0.00

ATOM 8 HB2 GLN 1 -8.861 -13.175 -0.683 1.00 0.00

ATOM 9 HB3 GLN 1 -8.928 -14.849 -0.082 1.00 0.00

ATOM 10 CG GLN 1 -10.658 -13.662 0.393 1.00 0.00

ATOM 11 HG2 GLN 1 -11.273 -14.469 -0.006 1.00 0.00

ATOM 12 HG3 GLN 1 -10.734 -13.651 1.480 1.00 0.00

ATOM 13 CD GLN 1 -11.183 -12.341 -0.144 1.00 0.00

ATOM 14 OE1 GLN 1 -11.541 -11.440 0.596 1.00 0.00

ATOM 15 NE2 GLN 1 -11.079 -12.181 -1.450 1.00 0.00

ATOM 16 HE21 GLN 1 -11.407 -11.328 -1.879 1.00 0.00
ATOM 17 HE22 GLN 1 -10.672 -12.913 -2.015 1.00 0.00
ATOM 18 C GLN 1 -7.633 -14.596 1.983 1.00 0.00
ATOM 19 O GLN 1 -7.985 -15.076 3.051 1.00 0.00
ATOM 20 N PRO 2 -6.575 -15.047 1.287 1.00 0.00
ATOM 21 CD PRO 2 -6.079 -14.523 0.016 1.00 0.00
ATOM 22 HD2 PRO 2 -5.674 -13.529 0.206 1.00 0.00
ATOM 23 HD3 PRO 2 -6.929 -14.445 -0.661 1.00 0.00
ATOM 24 CG PRO 2 -5.025 -15.496 -0.487 1.00 0.00
ATOM 25 HG2 PRO 2 -4.060 -14.991 -0.528 1.00 0.00
ATOM 26 HG3 PRO 2 -5.299 -15.837 -1.486 1.00 0.00
ATOM 27 CB PRO 2 -5.002 -16.662 0.500 1.00 0.00
ATOM 28 HB2 PRO 2 -3.982 -16.923 0.783 1.00 0.00
ATOM 29 HB3 PRO 2 -5.496 -17.537 0.078 1.00 0.00
ATOM 30 CA PRO 2 -5.771 -16.169 1.724 1.00 0.00
ATOM 31 HA PRO 2 -6.441 -16.975 2.023 1.00 0.00
ATOM 32 C PRO 2 -4.881 -15.803 2.880 1.00 0.00
ATOM 33 O PRO 2 -3.892 -15.122 2.668 1.00 0.00
ATOM 34 N GLN 3 -5.227 -16.266 4.085 1.00 0.00
ATOM 35 H GLN 3 -6.050 -16.844 4.180 1.00 0.00
ATOM 36 CA GLN 3 -4.459 -15.964 5.274 1.00 0.00
ATOM 37 HA GLN 3 -4.385 -14.884 5.397 1.00 0.00

ATOM	38	CB	GLN	3	-5.125	-16.552	6.517	1.00	0.00
ATOM	39	HB2	GLN	3	-6.176	-16.741	6.298	1.00	0.00
ATOM	40	HB3	GLN	3	-4.633	-17.492	6.769	1.00	0.00
ATOM	41	CG	GLN	3	-5.016	-15.583	7.702	1.00	0.00
ATOM	42	HG2	GLN	3	-4.570	-16.127	8.535	1.00	0.00
ATOM	43	HG3	GLN	3	-4.386	-14.731	7.446	1.00	0.00
ATOM	44	CD	GLN	3	-6.373	-15.070	8.131	1.00	0.00
ATOM	45	OE1	GLN	3	-6.993	-15.584	9.041	1.00	0.00
ATOM	46	NE2	GLN	3	-6.839	-14.058	7.421	1.00	0.00
ATOM	47	HE21	GLN	3	-7.739	-13.660	7.646	1.00	0.00
ATOM	48	HE22	GLN	3	-6.293	-13.688	6.656	1.00	0.00
ATOM	49	C	GLN	3	-3.077	-16.541	5.150	1.00	0.00
ATOM	50	O	GLN	3	-2.136	-15.964	5.622	1.00	0.00
ATOM	51	N	LYS	4	-2.911	-17.655	4.445	1.00	0.00
ATOM	52	H	LYS	4	-3.727	-18.137	4.096	1.00	0.00
ATOM	53	CA	LYS	4	-1.607	-18.262	4.229	1.00	0.00
ATOM	54	HA	LYS	4	-1.256	-18.741	5.143	1.00	0.00
ATOM	55	CB	LYS	4	-1.714	-19.324	3.131	1.00	0.00
ATOM	56	HB2	LYS	4	-0.882	-20.020	3.242	1.00	0.00
ATOM	57	HB3	LYS	4	-2.654	-19.860	3.260	1.00	0.00
ATOM	58	CG	LYS	4	-1.672	-18.695	1.729	1.00	0.00
ATOM	59	HG2	LYS	4	-1.881	-17.629	1.817	1.00	0.00

ATOM 60 HG3 LYS 4 -0.675 -18.837 1.311 1.00 0.00
ATOM 61 CD LYS 4 -2.706 -19.342 0.809 1.00 0.00
ATOM 62 HD2 LYS 4 -3.669 -19.364 1.320 1.00 0.00
ATOM 63 HD3 LYS 4 -2.791 -18.747 -0.100 1.00 0.00
ATOM 64 CE LYS 4 -2.290 -20.767 0.447 1.00 0.00
ATOM 65 HE2 LYS 4 -2.089 -20.820 -0.623 1.00 0.00
ATOM 66 HE3 LYS 4 -1.387 -21.027 0.999 1.00 0.00
ATOM 67 NZ LYS 4 -3.366 -21.714 0.792 1.00 0.00
ATOM 68 HZ1 LYS 4 -4.203 -21.474 0.280 1.00 0.00
ATOM 69 HZ2 LYS 4 -3.080 -22.652 0.548 1.00 0.00
ATOM 70 HZ3 LYS 4 -3.552 -21.666 1.783 1.00 0.00
ATOM 71 C LYS 4 -0.553 -17.190 3.900 1.00 0.00
ATOM 72 O LYS 4 0.590 -17.354 4.264 1.00 0.00
ATOM 73 N CYX 5 -0.997 -16.091 3.264 1.00 0.00
ATOM 74 H CYX 5 -1.957 -16.058 2.952 1.00 0.00
ATOM 75 CA CYX 5 -0.158 -14.974 2.988 1.00 0.00
ATOM 76 HA CYX 5 0.859 -15.170 3.327 1.00 0.00
ATOM 77 CB CYX 5 -0.131 -14.729 1.502 1.00 0.00
ATOM 78 HB2 CYX 5 -0.722 -15.493 0.996 1.00 0.00
ATOM 79 HB3 CYX 5 -0.549 -13.746 1.288 1.00 0.00
ATOM 80 SG CYX 5 1.535 -14.807 0.776 1.00 0.00
ATOM 81 C CYX 5 -0.670 -13.768 3.706 1.00 0.00

```
ATOM     82  O   CYX     5       0.086 -13.011   4.232  1.00  0.00
ATOM     83  N   GLN     6      -1.980 -13.577   3.829  1.00  0.00
ATOM     84  H   GLN     6      -2.594 -14.215   3.342  1.00  0.00
ATOM     85  CA  GLN     6      -2.570 -12.472   4.548  1.00  0.00
ATOM     86  HA  GLN     6      -2.159 -11.532   4.180  1.00  0.00
ATOM     87  CB  GLN     6      -4.087 -12.452   4.353  1.00  0.00
ATOM     88  HB2 GLN     6      -4.405 -13.438   4.015  1.00  0.00
ATOM     89  HB3 GLN     6      -4.556 -12.228   5.311  1.00  0.00
ATOM     90  CG  GLN     6      -4.503 -11.405   3.327  1.00  0.00
ATOM     91  HG2 GLN     6      -3.589 -10.900   3.014  1.00  0.00
ATOM     92  HG3 GLN     6      -4.977 -11.867   2.461  1.00  0.00
ATOM     93  CD  GLN     6      -5.449 -10.377   3.905  1.00  0.00
ATOM     94  OE1 GLN     6      -5.469 -10.078   5.084  1.00  0.00
ATOM     95  NE2 GLN     6      -6.311  -9.859   3.041  1.00  0.00
ATOM     96 HE21 GLN     6      -6.976  -9.165   3.350  1.00  0.00
ATOM     97 HE22 GLN     6      -6.298 -10.161   2.077  1.00  0.00
ATOM     98  C   GLN     6      -2.218 -12.511   6.025  1.00  0.00
ATOM     99  O   GLN     6      -2.463 -11.564   6.753  1.00  0.00
ATOM    100  N   ARG     7      -1.652 -13.619   6.485  1.00  0.00
ATOM    101  H   ARG     7      -1.528 -14.369   5.819  1.00  0.00
ATOM    102  CA  ARG     7      -1.241 -13.839   7.839  1.00  0.00
ATOM    103  HA  ARG     7      -1.646 -13.048   8.470  1.00  0.00
```

ATOM	104	CB	ARG	7	-1.736	-15.189	8.368	1.00	0.00
ATOM	105	HB2	ARG	7	-2.541	-15.548	7.726	1.00	0.00
ATOM	106	HB3	ARG	7	-0.911	-15.902	8.348	1.00	0.00
ATOM	107	CG	ARG	7	-2.251	-15.047	9.797	1.00	0.00
ATOM	108	HG2	ARG	7	-1.523	-14.480	10.376	1.00	0.00
ATOM	109	HG3	ARG	7	-3.198	-14.507	9.775	1.00	0.00
ATOM	110	CD	ARG	7	-2.461	-16.420	10.445	1.00	0.00
ATOM	111	HD2	ARG	7	-3.455	-16.796	10.200	1.00	0.00
ATOM	112	HD3	ARG	7	-1.709	-17.120	10.079	1.00	0.00
ATOM	113	NE	ARG	7	-2.341	-16.321	11.914	1.00	0.00
ATOM	114	HE	ARG	7	-3.203	-16.229	12.432	1.00	0.00
ATOM	115	CZ	ARG	7	-1.199	-16.339	12.585	1.00	0.00
ATOM	116	NH1	ARG	7	-0.057	-16.470	11.951	1.00	0.00
ATOM	117	HH11	ARG	7	-0.049	-16.558	10.945	1.00	0.00
ATOM	118	HH12	ARG	7	0.808	-16.483	12.472	1.00	0.00
ATOM	119	NH2	ARG	7	-1.197	-16.232	13.892	1.00	0.00
ATOM	120	HH21	ARG	7	-2.071	-16.136	14.389	1.00	0.00
ATOM	121	HH22	ARG	7	-0.322	-16.247	14.395	1.00	0.00
ATOM	122	C	ARG	7	0.260	-13.785	7.940	1.00	0.00
ATOM	123	O	ARG	7	0.736	-13.792	9.048	1.00	0.00
ATOM	124	N	GLU	8	0.956	-13.773	6.816	1.00	0.00
ATOM	125	H	GLU	8	0.458	-13.866	5.942	1.00	0.00

ATOM	126	CA	GLU	8	2.401	-13.696	6.777	1.00	0.00
ATOM	127	HA	GLU	8	2.803	-13.738	7.789	1.00	0.00
ATOM	128	CB	GLU	8	2.970	-14.868	5.977	1.00	0.00
ATOM	129	HB2	GLU	8	2.147	-15.412	5.512	1.00	0.00
ATOM	130	HB3	GLU	8	3.632	-14.480	5.203	1.00	0.00
ATOM	131	CG	GLU	8	3.755	-15.815	6.892	1.00	0.00
ATOM	132	HG2	GLU	8	3.411	-15.620	7.907	1.00	0.00
ATOM	133	HG3	GLU	8	3.563	-16.858	6.638	1.00	0.00
ATOM	134	CD	GLU	8	5.259	-15.536	6.800	1.00	0.00
ATOM	135	OE1	GLU	8	5.818	-15.800	5.716	1.00	0.00
ATOM	136	OE2	GLU	8	5.815	-15.120	7.835	1.00	0.00
ATOM	137	C	GLU	8	2.877	-12.375	6.190	1.00	0.00
ATOM	138	O	GLU	8	3.945	-11.890	6.506	1.00	0.00
ATOM	139	N	PHE	9	2.018	-11.719	5.422	1.00	0.00
ATOM	140	H	PHE	9	1.104	-12.129	5.290	1.00	0.00
ATOM	141	CA	PHE	9	2.303	-10.477	4.777	1.00	0.00
ATOM	142	HA	PHE	9	3.341	-10.444	4.447	1.00	0.00
ATOM	143	CB	PHE	9	1.399	-10.341	3.556	1.00	0.00
ATOM	144	HB2	PHE	9	1.874	-10.837	2.709	1.00	0.00
ATOM	145	HB3	PHE	9	0.428	-10.799	3.747	1.00	0.00
ATOM	146	CG	PHE	9	1.128	-8.914	3.127	1.00	0.00
ATOM	147	CD1	PHE	9	0.305	-8.075	3.897	1.00	0.00

ATOM 148 HD1 PHE 9 -0.167 -8.477 4.793 1.00 0.00

ATOM 149 CE1 PHE 9 0.088 -6.749 3.531 1.00 0.00

ATOM 150 HE1 PHE 9 -0.511 -6.096 4.165 1.00 0.00

ATOM 151 CZ PHE 9 0.639 -6.259 2.350 1.00 0.00

ATOM 152 HZ PHE 9 0.465 -5.228 2.041 1.00 0.00

ATOM 153 CE2 PHE 9 1.421 -7.102 1.558 1.00 0.00

ATOM 154 HE2 PHE 9 1.833 -6.718 0.625 1.00 0.00

ATOM 155 CD2 PHE 9 1.678 -8.420 1.945 1.00 0.00

ATOM 156 HD2 PHE 9 2.306 -9.058 1.323 1.00 0.00

ATOM 157 C PHE 9 2.035 -9.364 5.747 1.00 0.00

ATOM 158 O PHE 9 2.709 -8.354 5.767 1.00 0.00

ATOM 159 N GLN 10 0.962 -9.491 6.544 1.00 0.00

ATOM 160 H GLN 10 0.373 -10.307 6.449 1.00 0.00

ATOM 161 CA GLN 10 0.574 -8.473 7.491 1.00 0.00

ATOM 162 HA GLN 10 1.028 -7.522 7.212 1.00 0.00

ATOM 163 CB GLN 10 -0.949 -8.301 7.514 1.00 0.00

ATOM 164 HB2 GLN 10 -1.245 -7.703 6.653 1.00 0.00

ATOM 165 HB3 GLN 10 -1.412 -9.286 7.448 1.00 0.00

ATOM 166 CG GLN 10 -1.408 -7.605 8.803 1.00 0.00

ATOM 167 HG2 GLN 10 -1.260 -8.264 9.658 1.00 0.00

ATOM 168 HG3 GLN 10 -0.807 -6.705 8.930 1.00 0.00

ATOM 169 CD GLN 10 -2.859 -7.210 8.745 1.00 0.00

ATOM 170 OE1 GLN 10 -3.228 -6.213 8.145 1.00 0.00
ATOM 171 NE2 GLN 10 -3.698 -8.025 9.358 1.00 0.00
ATOM 172 HE21 GLN 10 -4.687 -7.820 9.358 1.00 0.00
ATOM 173 HE22 GLN 10 -3.347 -8.850 9.824 1.00 0.00
ATOM 174 C GLN 10 1.117 -8.844 8.865 1.00 0.00
ATOM 175 O GLN 10 1.100 -8.036 9.792 1.00 0.00
ATOM 176 N GLN 11 1.586 -10.076 9.023 1.00 0.00
ATOM 177 H GLN 11 1.542 -10.728 8.252 1.00 0.00
ATOM 178 CA GLN 11 2.116 -10.539 10.279 1.00 0.00
ATOM 179 HA GLN 11 1.311 -10.639 11.007 1.00 0.00
ATOM 180 CB GLN 11 2.785 -11.897 10.103 1.00 0.00
ATOM 181 HB2 GLN 11 2.457 -12.334 9.159 1.00 0.00
ATOM 182 HB3 GLN 11 3.866 -11.759 10.083 1.00 0.00
ATOM 183 CG GLN 11 2.412 -12.834 11.255 1.00 0.00
ATOM 184 HG2 GLN 11 1.593 -12.363 11.798 1.00 0.00
ATOM 185 HG3 GLN 11 2.087 -13.805 10.880 1.00 0.00
ATOM 186 CD GLN 11 3.568 -13.046 12.211 1.00 0.00
ATOM 187 OE1 GLN 11 4.722 -13.085 11.843 1.00 0.00
ATOM 188 NE2 GLN 11 3.228 -13.238 13.475 1.00 0.00
ATOM 189 HE21 GLN 11 3.945 -13.387 14.171 1.00 0.00
ATOM 190 HE22 GLN 11 2.253 -13.236 13.738 1.00 0.00
ATOM 191 C GLN 11 3.104 -9.538 10.847 1.00 0.00

ATOM 192 O GLN 11 2.905 -9.023 11.943 1.00 0.00

ATOM 193 N GLU 12 4.094 -9.169 10.033 1.00 0.00

ATOM 194 H GLU 12 4.164 -9.582 9.114 1.00 0.00

ATOM 195 CA GLU 12 5.138 -8.254 10.458 1.00 0.00

ATOM 196 HA GLU 12 4.691 -7.282 10.667 1.00 0.00

ATOM 197 CB GLU 12 5.864 -8.742 11.721 1.00 0.00

ATOM 198 HB2 GLU 12 6.888 -8.369 11.695 1.00 0.00

ATOM 199 HB3 GLU 12 5.351 -8.334 12.592 1.00 0.00

ATOM 200 CG GLU 12 5.880 -10.271 11.809 1.00 0.00

ATOM 201 HG2 GLU 12 4.981 -10.637 12.305 1.00 0.00

ATOM 202 HG3 GLU 12 5.899 -10.632 10.780 1.00 0.00

ATOM 203 CD GLU 12 7.106 -10.779 12.545 1.00 0.00

ATOM 204 OE1 GLU 12 8.156 -10.918 11.877 1.00 0.00

ATOM 205 OE2 GLU 12 6.995 -10.970 13.780 1.00 0.00

ATOM 206 C GLU 12 6.124 -8.014 9.323 1.00 0.00

ATOM 207 O GLU 12 7.309 -7.778 9.529 1.00 0.00

ATOM 208 N GLN 13 5.615 -8.031 8.092 1.00 0.00

ATOM 209 H GLN 13 4.638 -8.252 7.960 1.00 0.00

ATOM 210 CA GLN 13 6.432 -7.818 6.922 1.00 0.00

ATOM 211 HA GLN 13 7.176 -7.049 7.128 1.00 0.00

ATOM 212 CB GLN 13 7.158 -9.107 6.527 1.00 0.00

ATOM 213 HB2 GLN 13 6.579 -9.961 6.880 1.00 0.00

ATOM	214	HB3	GLN	13	7.240	-9.148	5.441	1.00	0.00
ATOM	215	CG	GLN	13	8.554	-9.149	7.144	1.00	0.00
ATOM	216	HG2	GLN	13	9.038	-8.173	7.135	1.00	0.00
ATOM	217	HG3	GLN	13	8.424	-9.480	8.174	1.00	0.00
ATOM	218	CD	GLN	13	9.450	-10.141	6.426	1.00	0.00
ATOM	219	OE1	GLN	13	9.373	-10.366	5.227	1.00	0.00
ATOM	220	NE2	GLN	13	10.387	-10.706	7.170	1.00	0.00
ATOM	221	HE21	GLN	13	11.019	-11.377	6.757	1.00	0.00
ATOM	222	HE22	GLN	13	10.465	-10.464	8.147	1.00	0.00
ATOM	223	C	GLN	13	5.590	-7.295	5.790	1.00	0.00
ATOM	224	O	GLN	13	5.609	-7.831	4.721	1.00	0.00
ATOM	225	N	HIE	14	4.806	-6.249	6.044	1.00	0.00
ATOM	226	H	HIE	14	4.786	-5.884	6.985	1.00	0.00
ATOM	227	CA	HIE	14	3.911	-5.670	5.064	1.00	0.00
ATOM	228	HA	HIE	14	3.303	-6.456	4.616	1.00	0.00
ATOM	229	CB	HIE	14	2.981	-4.641	5.713	1.00	0.00
ATOM	230	HB2	HIE	14	3.443	-3.654	5.718	1.00	0.00
ATOM	231	HB3	HIE	14	2.060	-4.608	5.130	1.00	0.00
ATOM	232	CG	HIE	14	2.582	-4.935	7.129	1.00	0.00
ATOM	233	ND1	HIE	14	2.030	-4.007	7.977	1.00	0.00
ATOM	234	CE1	HIE	14	1.806	-4.592	9.161	1.00	0.00
ATOM	235	HE1	HIE	14	1.342	-4.041	9.979	1.00	0.00

ATOM 236 NE2 HIE 14 2.239 -5.853 9.089 1.00 0.00
ATOM 237 HE2 HIE 14 2.224 -6.525 9.843 1.00 0.00
ATOM 238 CD2 HIE 14 2.710 -6.099 7.828 1.00 0.00
ATOM 239 HD2 HIE 14 3.087 -7.089 7.570 1.00 0.00
ATOM 240 C HIE 14 4.757 -5.088 3.941 1.00 0.00
ATOM 241 O HIE 14 5.867 -5.499 3.643 1.00 0.00
ATOM 242 N LEU 15 4.259 -4.011 3.336 1.00 0.00
ATOM 243 H LEU 15 3.330 -3.692 3.569 1.00 0.00
ATOM 244 CA LEU 15 4.962 -3.346 2.263 1.00 0.00
ATOM 245 HA LEU 15 5.962 -3.765 2.156 1.00 0.00
ATOM 246 CB LEU 15 4.207 -3.537 0.941 1.00 0.00
ATOM 247 HB2 LEU 15 3.379 -2.834 1.028 1.00 0.00
ATOM 248 HB3 LEU 15 4.844 -3.245 0.106 1.00 0.00
ATOM 249 CG LEU 15 3.657 -4.945 0.695 1.00 0.00
ATOM 250 HG LEU 15 2.801 -5.108 1.350 1.00 0.00
ATOM 251 CD1 LEU 15 3.224 -5.112 -0.757 1.00 0.00
ATOM 252 HD11 LEU 15 4.079 -4.950 -1.413 1.00 0.00
ATOM 253 HD12 LEU 15 2.837 -6.120 -0.908 1.00 0.00
ATOM 254 HD13 LEU 15 2.445 -4.386 -0.991 1.00 0.00
ATOM 255 CD2 LEU 15 4.642 -6.009 1.087 1.00 0.00
ATOM 256 HD21 LEU 15 4.876 -5.916 2.147 1.00 0.00
ATOM 257 HD22 LEU 15 4.211 -6.992 0.896 1.00 0.00

ATOM	258	HD23	LEU	15	5.555	-5.893	0.502	1.00	0.00
ATOM	259	C	LEU	15	5.189	-1.896	2.619	1.00	0.00
ATOM	260	O	LEU	15	4.596	-1.398	3.582	1.00	0.00
ATOM	261	N	ARG	16	6.001	-1.206	1.817	1.00	0.00
ATOM	262	H	ARG	16	6.416	-1.664	1.018	1.00	0.00
ATOM	263	CA	ARG	16	6.310	0.186	2.045	1.00	0.00
ATOM	264	HA	ARG	16	5.451	0.679	2.501	1.00	0.00
ATOM	265	CB	ARG	16	7.511	0.338	2.976	1.00	0.00
ATOM	266	HB2	ARG	16	8.215	-0.468	2.770	1.00	0.00
ATOM	267	HB3	ARG	16	7.988	1.297	2.775	1.00	0.00
ATOM	268	CG	ARG	16	7.083	0.279	4.443	1.00	0.00
ATOM	269	HG2	ARG	16	6.012	0.082	4.486	1.00	0.00
ATOM	270	HG3	ARG	16	7.621	-0.534	4.931	1.00	0.00
ATOM	271	CD	ARG	16	7.389	1.596	5.159	1.00	0.00
ATOM	272	HD2	ARG	16	7.357	2.416	4.442	1.00	0.00
ATOM	273	HD3	ARG	16	6.648	1.767	5.939	1.00	0.00
ATOM	274	NE	ARG	16	8.728	1.561	5.778	1.00	0.00
ATOM	275	HE	ARG	16	9.365	0.863	5.422	1.00	0.00
ATOM	276	CZ	ARG	16	9.146	2.365	6.738	1.00	0.00
ATOM	277	NH1	ARG	16	8.351	3.293	7.220	1.00	0.00
ATOM	278	HH11	ARG	16	7.415	3.391	6.854	1.00	0.00
ATOM	279	HH12	ARG	16	8.680	3.903	7.954	1.00	0.00

ATOM 280 NH2 ARG 16 10.356 2.241 7.226 1.00 0.00

ATOM 281 HH21 ARG 16 10.972 1.526 6.865 1.00 0.00

ATOM 282 HH22 ARG 16 10.667 2.860 7.960 1.00 0.00

ATOM 283 C ARG 16 6.632 0.840 0.750 1.00 0.00

ATOM 284 O ARG 16 5.814 1.575 0.267 1.00 0.00

ATOM 285 N ALA 17 7.768 0.553 0.152 1.00 0.00

ATOM 286 H ALA 17 8.428 -0.036 0.639 1.00 0.00

ATOM 287 CA ALA 17 8.155 1.104 -1.120 1.00 0.00

ATOM 288 HA ALA 17 8.312 2.176 -1.005 1.00 0.00

ATOM 289 CB ALA 17 9.471 0.463 -1.585 1.00 0.00

ATOM 290 HB1 ALA 17 9.328 -0.610 -1.714 1.00 0.00

ATOM 291 HB2 ALA 17 9.775 0.904 -2.534 1.00 0.00

ATOM 292 HB3 ALA 17 10.245 0.638 -0.838 1.00 0.00

ATOM 293 C ALA 17 7.031 0.930 -2.160 1.00 0.00

ATOM 294 O ALA 17 6.933 1.673 -3.127 1.00 0.00

ATOM 295 N CYX 18 6.092 0.014 -1.902 1.00 0.00

ATOM 296 H CYX 18 6.193 -0.544 -1.066 1.00 0.00

ATOM 297 CA CYX 18 4.995 -0.260 -2.768 1.00 0.00

ATOM 298 HA CYX 18 5.282 -0.043 -3.797 1.00 0.00

ATOM 299 CB CYX 18 4.574 -1.721 -2.679 1.00 0.00

ATOM 300 HB2 CYX 18 5.384 -2.307 -2.244 1.00 0.00

ATOM 301 HB3 CYX 18 3.687 -1.807 -2.052 1.00 0.00

ATOM	302	SG	CYX	18	4.212	-2.516	-4.262	1.00	0.00
ATOM	303	C	CYX	18	3.881	0.674	-2.389	1.00	0.00
ATOM	304	O	CYX	18	3.325	1.372	-3.219	1.00	0.00
ATOM	305	N	GLN	19	3.552	0.720	-1.101	1.00	0.00
ATOM	306	H	GLN	19	3.985	0.071	-0.460	1.00	0.00
ATOM	307	CA	GLN	19	2.537	1.600	-0.596	1.00	0.00
ATOM	308	HA	GLN	19	1.819	1.837	-1.381	1.00	0.00
ATOM	309	CB	GLN	19	1.802	0.927	0.545	1.00	0.00
ATOM	310	HB2	GLN	19	0.980	1.579	0.839	1.00	0.00
ATOM	311	HB3	GLN	19	1.401	-0.015	0.170	1.00	0.00
ATOM	312	CG	GLN	19	2.691	0.655	1.752	1.00	0.00
ATOM	313	HG2	GLN	19	2.511	-0.361	2.105	1.00	0.00
ATOM	314	HG3	GLN	19	3.737	0.759	1.464	1.00	0.00
ATOM	315	CD	GLN	19	2.401	1.624	2.889	1.00	0.00
ATOM	316	OE1	GLN	19	3.287	2.256	3.464	1.00	0.00
ATOM	317	NE2	GLN	19	1.126	1.745	3.231	1.00	0.00
ATOM	318	HE21	GLN	19	0.864	2.371	3.978	1.00	0.00
ATOM	319	HE22	GLN	19	0.422	1.210	2.742	1.00	0.00
ATOM	320	C	GLN	19	3.120	2.927	-0.180	1.00	0.00
ATOM	321	O	GLN	19	2.624	3.541	0.735	1.00	0.00
ATOM	322	N	GLN	20	4.203	3.368	-0.797	1.00	0.00
ATOM	323	H	GLN	20	4.582	2.800	-1.542	1.00	0.00

ATOM	324	CA	GLN	20	4.893	4.585	-0.446	1.00	0.00
ATOM	325	HA	GLN	20	4.247	5.179	0.200	1.00	0.00
ATOM	326	CB	GLN	20	6.212	4.326	0.286	1.00	0.00
ATOM	327	HB2	GLN	20	6.770	3.566	-0.261	1.00	0.00
ATOM	328	HB3	GLN	20	6.786	5.252	0.308	1.00	0.00
ATOM	329	CG	GLN	20	5.964	3.844	1.723	1.00	0.00
ATOM	330	HG2	GLN	20	5.002	3.334	1.784	1.00	0.00
ATOM	331	HG3	GLN	20	6.762	3.145	1.976	1.00	0.00
ATOM	332	CD	GLN	20	5.976	4.968	2.720	1.00	0.00
ATOM	333	OE1	GLN	20	6.536	6.030	2.517	1.00	0.00
ATOM	334	NE2	GLN	20	5.307	4.757	3.842	1.00	0.00
ATOM	335	HE21	GLN	20	5.278	5.475	4.551	1.00	0.00
ATOM	336	HE22	GLN	20	4.829	3.878	3.983	1.00	0.00
ATOM	337	C	GLN	20	5.157	5.373	-1.703	1.00	0.00
ATOM	338	O	GLN	20	4.974	6.581	-1.727	1.00	0.00
ATOM	339	N	TRP	21	5.533	4.686	-2.782	1.00	0.00
ATOM	340	H	TRP	21	5.699	3.692	-2.712	1.00	0.00
ATOM	341	CA	TRP	21	5.778	5.335	-4.052	1.00	0.00
ATOM	342	HA	TRP	21	6.541	6.104	-3.934	1.00	0.00
ATOM	343	CB	TRP	21	6.265	4.319	-5.084	1.00	0.00
ATOM	344	HB2	TRP	21	6.767	4.832	-5.904	1.00	0.00
ATOM	345	HB3	TRP	21	6.968	3.646	-4.593	1.00	0.00

ATOM	346	CG	TRP	21	5.210	3.460	-5.700	1.00	0.00
ATOM	347	CD1	TRP	21	4.865	2.191	-5.392	1.00	0.00
ATOM	348	HD1	TRP	21	5.452	1.735	-4.595	1.00	0.00
ATOM	349	NE1	TRP	21	3.819	1.734	-6.172	1.00	0.00
ATOM	350	HE1	TRP	21	3.380	0.827	-6.095	1.00	0.00
ATOM	351	CE2	TRP	21	3.451	2.725	-7.047	1.00	0.00
ATOM	352	CZ2	TRP	21	2.464	2.800	-8.028	1.00	0.00
ATOM	353	HZ2	TRP	21	1.842	1.919	-8.190	1.00	0.00
ATOM	354	CH2	TRP	21	2.331	3.990	-8.741	1.00	0.00
ATOM	355	HH2	TRP	21	1.588	4.075	-9.534	1.00	0.00
ATOM	356	CZ3	TRP	21	3.147	5.094	-8.452	1.00	0.00
ATOM	357	HZ3	TRP	21	3.001	6.035	-8.982	1.00	0.00
ATOM	358	CE3	TRP	21	4.161	5.032	-7.491	1.00	0.00
ATOM	359	HE3	TRP	21	4.792	5.907	-7.339	1.00	0.00
ATOM	360	CD2	TRP	21	4.315	3.838	-6.758	1.00	0.00
ATOM	361	C	TRP	21	4.503	6.029	-4.499	1.00	0.00
ATOM	362	O	TRP	21	4.520	7.099	-5.096	1.00	0.00
ATOM	363	N	ILE	22	3.352	5.429	-4.172	1.00	0.00
ATOM	364	H	ILE	22	3.378	4.545	-3.684	1.00	0.00
ATOM	365	CA	ILE	22	2.063	5.969	-4.543	1.00	0.00
ATOM	366	HA	ILE	22	2.050	6.298	-5.582	1.00	0.00
ATOM	367	CB	ILE	22	0.968	4.917	-4.327	1.00	0.00

ATOM 368 HB ILE 22 0.914 4.669 -3.267 1.00 0.00
ATOM 369 CG2 ILE 22 -0.386 5.461 -4.793 1.00 0.00
ATOM 370 HG21 ILE 22 -0.333 5.708 -5.853 1.00 0.00
ATOM 371 HG22 ILE 22 -1.156 4.705 -4.635 1.00 0.00
ATOM 372 HG23 ILE 22 -0.634 6.356 -4.223 1.00 0.00
ATOM 373 CG1 ILE 22 1.314 3.616 -5.058 1.00 0.00
ATOM 374 HG12 ILE 22 1.049 3.728 -6.110 1.00 0.00
ATOM 375 HG13 ILE 22 2.387 3.444 -4.970 1.00 0.00
ATOM 376 CD1 ILE 22 0.559 2.429 -4.468 1.00 0.00
ATOM 377 HD11 ILE 22 -0.514 2.600 -4.556 1.00 0.00
ATOM 378 HD12 ILE 22 0.827 1.521 -5.010 1.00 0.00
ATOM 379 HD13 ILE 22 0.823 2.315 -3.417 1.00 0.00
ATOM 380 C ILE 22 1.816 7.220 -3.724 1.00 0.00
ATOM 381 O ILE 22 1.178 8.158 -4.178 1.00 0.00
ATOM 382 N ARG 23 2.338 7.258 -2.492 1.00 0.00
ATOM 383 H ARG 23 2.797 6.434 -2.129 1.00 0.00
ATOM 384 CA ARG 23 2.238 8.419 -1.638 1.00 0.00
ATOM 385 HA ARG 23 1.200 8.749 -1.594 1.00 0.00
ATOM 386 CB ARG 23 2.708 8.104 -0.225 1.00 0.00
ATOM 387 HB2 ARG 23 3.485 7.341 -0.274 1.00 0.00
ATOM 388 HB3 ARG 23 3.117 9.010 0.220 1.00 0.00
ATOM 389 CG ARG 23 1.551 7.596 0.629 1.00 0.00

ATOM	390	HG2	ARG	23	1.703	7.921	1.658	1.00	0.00
ATOM	391	HG3	ARG	23	0.621	8.018	0.249	1.00	0.00
ATOM	392	CD	ARG	23	1.474	6.075	0.584	1.00	0.00
ATOM	393	HD2	ARG	23	0.552	5.760	0.095	1.00	0.00
ATOM	394	HD3	ARG	23	2.328	5.672	0.040	1.00	0.00
ATOM	395	NE	ARG	23	1.490	5.530	1.969	1.00	0.00
ATOM	396	HE	ARG	23	0.706	4.940	2.210	1.00	0.00
ATOM	397	CZ	ARG	23	2.448	5.684	2.847	1.00	0.00
ATOM	398	NH1	ARG	23	3.475	6.437	2.545	1.00	0.00
ATOM	399	HH11	ARG	23	3.518	6.889	1.643	1.00	0.00
ATOM	400	HH12	ARG	23	4.220	6.562	3.215	1.00	0.00
ATOM	401	NH2	ARG	23	2.347	5.164	4.044	1.00	0.00
ATOM	402	HH21	ARG	23	1.522	4.638	4.295	1.00	0.00
ATOM	403	HH22	ARG	23	3.094	5.291	4.711	1.00	0.00
ATOM	404	C	ARG	23	3.053	9.589	-2.184	1.00	0.00
ATOM	405	O	ARG	23	2.839	10.736	-1.791	1.00	0.00
ATOM	406	N	GLN	24	3.988	9.298	-3.082	1.00	0.00
ATOM	407	H	GLN	24	4.175	8.332	-3.311	1.00	0.00
ATOM	408	CA	GLN	24	4.794	10.315	-3.704	1.00	0.00
ATOM	409	HA	GLN	24	5.011	11.108	-2.988	1.00	0.00
ATOM	410	CB	GLN	24	6.114	9.718	-4.185	1.00	0.00
ATOM	411	HB2	GLN	24	5.988	8.644	-4.322	1.00	0.00

ATOM	412	HB3	GLN	24	6.384	10.176	-5.137	1.00	0.00
ATOM	413	CG	GLN	24	7.223	9.974	-3.164	1.00	0.00
ATOM	414	HG2	GLN	24	7.940	10.679	-3.584	1.00	0.00
ATOM	415	HG3	GLN	24	6.778	10.401	-2.266	1.00	0.00
ATOM	416	CD	GLN	24	7.957	8.705	-2.784	1.00	0.00
ATOM	417	OE1	GLN	24	7.982	7.736	-3.515	1.00	0.00
ATOM	418	NE2	GLN	24	8.573	8.707	-1.623	1.00	0.00
ATOM	419	HE21	GLN	24	9.078	7.886	-1.321	1.00	0.00
ATOM	420	HE22	GLN	24	8.539	9.529	-1.038	1.00	0.00
ATOM	421	C	GLN	24	4.058	10.973	-4.858	1.00	0.00
ATOM	422	O	GLN	24	4.487	12.002	-5.367	1.00	0.00
ATOM	423	N	GLN	25	2.946	10.355	-5.284	1.00	0.00
ATOM	424	H	GLN	25	2.687	9.461	-4.892	1.00	0.00
ATOM	425	CA	GLN	25	2.136	10.896	-6.348	1.00	0.00
ATOM	426	HA	GLN	25	2.773	11.224	-7.169	1.00	0.00
ATOM	427	CB	GLN	25	1.169	9.831	-6.874	1.00	0.00
ATOM	428	HB2	GLN	25	1.262	8.933	-6.264	1.00	0.00
ATOM	429	HB3	GLN	25	0.150	10.211	-6.807	1.00	0.00
ATOM	430	CG	GLN	25	1.491	9.493	-8.329	1.00	0.00
ATOM	431	HG2	GLN	25	2.572	9.358	-8.351	1.00	0.00
ATOM	432	HG3	GLN	25	1.005	8.578	-8.669	1.00	0.00
ATOM	433	CD	GLN	25	1.123	10.620	-9.278	1.00	0.00

ATOM 434 OE1 GLN 25 0.631 11.662 -8.925 1.00 0.00
ATOM 435 NE2 GLN 25 1.273 10.374 -10.562 1.00 0.00
ATOM 436 HE21 GLN 25 1.044 11.087 -11.240 1.00 0.00
ATOM 437 HE22 GLN 25 1.617 9.473 -10.864 1.00 0.00
ATOM 438 C GLN 25 1.384 12.138 -5.874 1.00 0.00
ATOM 439 O GLN 25 0.771 12.869 -6.640 1.00 0.00
ATOM 440 N LEU 26 1.395 12.388 -4.568 1.00 0.00
ATOM 441 H LEU 26 1.818 11.712 -3.948 1.00 0.00
ATOM 442 CA LEU 26 0.720 13.516 -3.988 1.00 0.00
ATOM 443 HA LEU 26 0.202 14.075 -4.767 1.00 0.00
ATOM 444 CB LEU 26 -0.311 13.054 -2.947 1.00 0.00
ATOM 445 HB2 LEU 26 0.204 13.154 -1.992 1.00 0.00
ATOM 446 HB3 LEU 26 -1.161 13.735 -2.975 1.00 0.00
ATOM 447 CG LEU 26 -0.807 11.610 -3.111 1.00 0.00
ATOM 448 HG LEU 26 -0.692 11.310 -4.153 1.00 0.00
ATOM 449 CD1 LEU 26 -0.007 10.672 -2.224 1.00 0.00
ATOM 450 HD11 LEU 26 -0.122 10.970 -1.182 1.00 0.00
ATOM 451 HD12 LEU 26 -0.371 9.652 -2.352 1.00 0.00
ATOM 452 HD13 LEU 26 1.046 10.719 -2.501 1.00 0.00
ATOM 453 CD2 LEU 26 -2.297 11.509 -2.789 1.00 0.00
ATOM 454 HD21 LEU 26 -2.860 12.152 -3.465 1.00 0.00
ATOM 455 HD22 LEU 26 -2.627 10.477 -2.912 1.00 0.00

ATOM	456	HD23	LEU	26	-2.469	11.825	-1.760	1.00	0.00
ATOM	457	C	LEU	26	1.753	14.486	-3.441	1.00	0.00
ATOM	458	O	LEU	26	2.947	14.362	-3.684	1.00	0.00
ATOM	459	N	ALA	27	1.280	15.451	-2.655	1.00	0.00
ATOM	460	H	ALA	27	0.282	15.550	-2.532	1.00	0.00
ATOM	461	CA	ALA	27	2.151	16.427	-2.028	1.00	0.00
ATOM	462	HA	ALA	27	3.047	16.578	-2.630	1.00	0.00
ATOM	463	CB	ALA	27	1.404	17.751	-1.926	1.00	0.00
ATOM	464	HB1	ALA	27	0.509	17.618	-1.319	1.00	0.00
ATOM	465	HB2	ALA	27	2.049	18.498	-1.463	1.00	0.00
ATOM	466	HB3	ALA	27	1.119	18.086	-2.924	1.00	0.00
ATOM	467	C	ALA	27	2.627	15.913	-0.672	1.00	0.00
ATOM	468	O	ALA	27	3.066	16.680	0.180	1.00	0.00
ATOM	469	N	GLY	28	2.483	14.600	-0.439	1.00	0.00
ATOM	470	H	GLY	28	2.056	14.023	-1.150	1.00	0.00
ATOM	471	CA	GLY	28	2.912	13.970	0.788	1.00	0.00
ATOM	472	HA2	GLY	28	2.704	14.641	1.621	1.00	0.00
ATOM	473	HA3	GLY	28	2.356	13.042	0.922	1.00	0.00
ATOM	474	C	GLY	28	4.400	13.657	0.764	1.00	0.00
ATOM	475	O	GLY	28	4.878	12.893	1.597	1.00	0.00
ATOM	476	N	SER	29	5.120	14.202	-0.217	1.00	0.00
ATOM	477	H	SER	29	4.646	14.745	-0.924	1.00	0.00

ATOM	478	CA	SER	29	6.542	14.002	-0.348	1.00	0.00
ATOM	479	HA	SER	29	6.994	13.866	0.634	1.00	0.00
ATOM	480	CB	SER	29	6.815	12.756	-1.187	1.00	0.00
ATOM	481	HB2	SER	29	6.565	11.889	-0.575	1.00	0.00
ATOM	482	HB3	SER	29	6.152	12.793	-2.051	1.00	0.00
ATOM	483	OG	SER	29	8.155	12.652	-1.626	1.00	0.00
ATOM	484	HG	SER	29	8.260	11.851	-2.146	1.00	0.00
ATOM	485	C	SER	29	7.214	15.209	-0.959	1.00	0.00
ATOM	486	O	SER	29	6.568	15.939	-1.682	1.00	0.00
ATOM	487	N	PRO	30	8.473	15.453	-0.593	1.00	0.00
ATOM	488	CD	PRO	30	9.256	14.642	0.323	1.00	0.00
ATOM	489	HD2	PRO	30	9.426	13.673	-0.147	1.00	0.00
ATOM	490	HD3	PRO	30	8.673	14.506	1.234	1.00	0.00
ATOM	491	CG	PRO	30	10.551	15.408	0.570	1.00	0.00
ATOM	492	HG2	PRO	30	11.390	14.816	0.203	1.00	0.00
ATOM	493	HG3	PRO	30	10.666	15.576	1.641	1.00	0.00
ATOM	494	CB	PRO	30	10.432	16.736	-0.182	1.00	0.00
ATOM	495	HB2	PRO	30	11.334	16.954	-0.754	1.00	0.00
ATOM	496	HB3	PRO	30	10.231	17.557	0.506	1.00	0.00
ATOM	497	CA	PRO	30	9.250	16.549	-1.128	1.00	0.00
ATOM	498	HA	PRO	30	8.639	17.450	-1.079	1.00	0.00
ATOM	499	C	PRO	30	9.678	16.309	-2.555	1.00	0.00

ATOM	500	O	PRO	30	9.352	15.278	-3.132	1.00	0.00
ATOM	501	N	PHE	31	10.447	17.253	-3.109	1.00	0.00
ATOM	502	H	PHE	31	10.718	18.060	-2.566	1.00	0.00
ATOM	503	CA	PHE	31	10.918	17.155	-4.472	1.00	0.00
ATOM	504	HA	PHE	31	11.173	16.126	-4.726	1.00	0.00
ATOM	505	CB	PHE	31	9.788	17.621	-5.395	1.00	0.00
ATOM	506	HB2	PHE	31	9.720	18.707	-5.453	1.00	0.00
ATOM	507	HB3	PHE	31	10.014	17.219	-6.383	1.00	0.00
ATOM	508	CG	PHE	31	8.405	17.119	-5.029	1.00	0.00
ATOM	509	CD1	PHE	31	7.998	15.830	-5.408	1.00	0.00
ATOM	510	HD1	PHE	31	8.660	15.212	-6.016	1.00	0.00
ATOM	511	CE1	PHE	31	6.747	15.335	-5.009	1.00	0.00
ATOM	512	HE1	PHE	31	6.455	14.317	-5.268	1.00	0.00
ATOM	513	CZ	PHE	31	5.874	16.149	-4.278	1.00	0.00
ATOM	514	HZ	PHE	31	4.882	15.786	-4.007	1.00	0.00
ATOM	515	CE2	PHE	31	6.280	17.438	-3.893	1.00	0.00
ATOM	516	HE2	PHE	31	5.599	18.071	-3.324	1.00	0.00
ATOM	517	CD2	PHE	31	7.554	17.911	-4.238	1.00	0.00
ATOM	518	HD2	PHE	31	7.883	18.891	-3.893	1.00	0.00
ATOM	519	C	PHE	31	12.136	18.035	-4.692	1.00	0.00
ATOM	520	O	PHE	31	12.270	19.079	-4.050	1.00	0.00
ATOM	521	N	SER	32	13.016	17.608	-5.595	1.00	0.00

ATOM 522 H SER 32 12.876 16.718 -6.053 1.00 0.00
ATOM 523 CA SER 32 14.214 18.363 -5.911 1.00 0.00
ATOM 524 HA SER 32 14.115 19.384 -5.544 1.00 0.00
ATOM 525 CB SER 32 15.443 17.720 -5.250 1.00 0.00
ATOM 526 HB2 SER 32 15.130 16.789 -4.777 1.00 0.00
ATOM 527 HB3 SER 32 16.176 17.503 -6.027 1.00 0.00
ATOM 528 OG SER 32 16.023 18.570 -4.280 1.00 0.00
ATOM 529 HG SER 32 16.785 18.136 -3.890 1.00 0.00
ATOM 530 C SER 32 14.395 18.443 -7.419 1.00 0.00
ATOM 531 O SER 32 14.036 19.440 -8.045 1.00 0.00
ATOM 532 N GLU 33 14.948 17.375 -8.003 1.00 0.00
ATOM 533 H GLU 33 15.276 16.609 -7.433 1.00 0.00
ATOM 534 CA GLU 33 15.181 17.317 -9.425 1.00 0.00
ATOM 535 HA GLU 33 15.376 18.319 -9.807 1.00 0.00
ATOM 536 CB GLU 33 16.391 16.428 -9.739 1.00 0.00
ATOM 537 HB2 GLU 33 16.662 15.881 -8.836 1.00 0.00
ATOM 538 HB3 GLU 33 16.108 15.722 -10.520 1.00 0.00
ATOM 539 CG GLU 33 17.583 17.255 -10.209 1.00 0.00
ATOM 540 HG2 GLU 33 17.702 17.115 -11.284 1.00 0.00
ATOM 541 HG3 GLU 33 17.389 18.306 -9.999 1.00 0.00
ATOM 542 CD GLU 33 18.861 16.821 -9.495 1.00 0.00
ATOM 543 OE1 GLU 33 19.242 15.641 -9.672 1.00 0.00

ATOM	544	OE2	GLU	33	19.432	17.674	-8.783	1.00	0.00
ATOM	545	C	GLU	33	13.925	16.846	-10.148	1.00	0.00
ATOM	546	O	GLU	33	12.842	16.752	-9.563	1.00	0.00
ATOM	547	N	ASN	34	14.077	16.521	-11.437	1.00	0.00
ATOM	548	H	ASN	34	14.975	16.646	-11.881	1.00	0.00
ATOM	549	CA	ASN	34	12.994	16.002	-12.225	1.00	0.00
ATOM	550	HA	ASN	34	12.044	16.395	-11.863	1.00	0.00
ATOM	551	CB	ASN	34	13.170	16.417	-13.684	1.00	0.00
ATOM	552	HB2	ASN	34	14.237	16.443	-13.907	1.00	0.00
ATOM	553	HB3	ASN	34	12.679	15.700	-14.342	1.00	0.00
ATOM	554	CG	ASN	34	12.588	17.789	-13.944	1.00	0.00
ATOM	555	OD1	ASN	34	11.520	18.144	-13.474	1.00	0.00
ATOM	556	ND2	ASN	34	13.304	18.565	-14.734	1.00	0.00
ATOM	557	HD21	ASN	34	12.978	19.495	-14.953	1.00	0.00
ATOM	558	HD22	ASN	34	14.175	18.225	-15.117	1.00	0.00
ATOM	559	C	ASN	34	12.951	14.492	-12.144	1.00	0.00
ATOM	560	O	ASN	34	13.997	13.846	-12.087	1.00	0.00
ATOM	561	N	GLN	35	11.751	13.928	-12.236	1.00	0.00
ATOM	562	H	GLN	35	10.933	14.519	-12.278	1.00	0.00
ATOM	563	CA	GLN	35	11.561	12.500	-12.218	1.00	0.00
ATOM	564	HA	GLN	35	12.063	12.054	-13.077	1.00	0.00
ATOM	565	CB	GLN	35	12.139	11.888	-10.942	1.00	0.00

ATOM	566	HB2	GLN	35	12.919	12.549	-10.566	1.00	0.00
ATOM	567	HB3	GLN	35	11.340	11.813	-10.204	1.00	0.00
ATOM	568	CG	GLN	35	12.726	10.504	-11.198	1.00	0.00
ATOM	569	HG2	GLN	35	12.961	10.021	-10.249	1.00	0.00
ATOM	570	HG3	GLN	35	11.988	9.908	-11.735	1.00	0.00
ATOM	571	CD	GLN	35	13.994	10.576	-12.034	1.00	0.00
ATOM	572	OE1	GLN	35	15.036	11.036	-11.611	1.00	0.00
ATOM	573	NE2	GLN	35	13.888	10.085	-13.258	1.00	0.00
ATOM	574	HE21	GLN	35	14.688	10.097	-13.875	1.00	0.00
ATOM	575	HE22	GLN	35	13.008	9.700	-13.570	1.00	0.00
ATOM	576	C	GLN	35	10.094	12.180	-12.351	1.00	0.00
ATOM	577	O	GLN	35	9.250	13.033	-12.072	1.00	0.00
ATOM	578	N	TRP	36	9.798	10.923	-12.730	1.00	0.00
ATOM	579	H	TRP	36	10.540	10.282	-12.973	1.00	0.00
ATOM	580	CA	TRP	36	8.423	10.468	-12.864	1.00	0.00
ATOM	581	HA	TRP	36	7.967	10.926	-13.742	1.00	0.00
ATOM	582	CB	TRP	36	8.372	8.945	-13.025	1.00	0.00
ATOM	583	HB2	TRP	36	8.656	8.660	-14.038	1.00	0.00
ATOM	584	HB3	TRP	36	9.063	8.493	-12.314	1.00	0.00
ATOM	585	CG	TRP	36	7.025	8.329	-12.766	1.00	0.00
ATOM	586	CD1	TRP	36	6.343	8.249	-11.590	1.00	0.00
ATOM	587	HD1	TRP	36	6.850	8.678	-10.726	1.00	0.00

ATOM	588	NE1	TRP	36	5.119	7.622	-11.751	1.00	0.00
ATOM	589	HE1	TRP	36	4.452	7.437	-11.016	1.00	0.00
ATOM	590	CE2	TRP	36	4.968	7.272	-13.072	1.00	0.00
ATOM	591	CZ2	TRP	36	3.940	6.617	-13.751	1.00	0.00
ATOM	592	HZ2	TRP	36	3.060	6.314	-13.184	1.00	0.00
ATOM	593	CH2	TRP	36	4.104	6.389	-15.126	1.00	0.00
ATOM	594	HH2	TRP	36	3.325	5.881	-15.695	1.00	0.00
ATOM	595	CZ3	TRP	36	5.270	6.810	-15.791	1.00	0.00
ATOM	596	HZ3	TRP	36	5.386	6.627	-16.860	1.00	0.00
ATOM	597	CE3	TRP	36	6.309	7.468	-15.120	1.00	0.00
ATOM	598	HE3	TRP	36	7.193	7.761	-15.686	1.00	0.00
ATOM	599	CD2	TRP	36	6.168	7.708	-13.739	1.00	0.00
ATOM	600	C	TRP	36	7.620	10.930	-11.640	1.00	0.00
ATOM	601	O	TRP	36	6.425	11.179	-11.732	1.00	0.00
ATOM	602	N	GLY	37	8.298	11.028	-10.484	1.00	0.00
ATOM	603	H	GLY	37	9.262	10.728	-10.449	1.00	0.00
ATOM	604	CA	GLY	37	7.702	11.546	-9.284	1.00	0.00
ATOM	605	HA2	GLY	37	7.258	12.518	-9.496	1.00	0.00
ATOM	606	HA3	GLY	37	6.926	10.859	-8.945	1.00	0.00
ATOM	607	C	GLY	37	8.735	11.704	-8.186	1.00	0.00
ATOM	608	O	GLY	37	9.277	12.789	-7.990	1.00	0.00
ATOM	609	N	PRO	38	9.034	10.595	-7.492	1.00	0.00

ATOM	610	CD	PRO	38	8.448	9.274	-7.686	1.00	0.00
ATOM	611	HD2	PRO	38	8.790	8.894	-8.649	1.00	0.00
ATOM	612	HD3	PRO	38	7.364	9.388	-7.705	1.00	0.00
ATOM	613	CG	PRO	38	8.923	8.416	-6.515	1.00	0.00
ATOM	614	HG2	PRO	38	9.533	7.597	-6.896	1.00	0.00
ATOM	615	HG3	PRO	38	8.056	8.010	-5.993	1.00	0.00
ATOM	616	CB	PRO	38	9.730	9.334	-5.600	1.00	0.00
ATOM	617	HB2	PRO	38	10.671	8.866	-5.310	1.00	0.00
ATOM	618	HB3	PRO	38	9.163	9.593	-4.706	1.00	0.00
ATOM	619	CA	PRO	38	10.006	10.583	-6.420	1.00	0.00
ATOM	620	HA	PRO	38	9.815	11.436	-5.769	1.00	0.00
ATOM	621	C	PRO	38	11.414	10.665	-6.955	1.00	0.00
ATOM	622	O	PRO	38	12.127	9.677	-7.003	1.00	0.00
ATOM	623	N	GLN	39	11.839	11.854	-7.372	1.00	0.00
ATOM	624	H	GLN	39	11.210	12.643	-7.356	1.00	0.00
ATOM	625	CA	GLN	39	13.166	12.047	-7.909	1.00	0.00
ATOM	626	HA	GLN	39	13.323	11.386	-8.762	1.00	0.00
ATOM	627	CB	GLN	39	13.340	13.498	-8.375	1.00	0.00
ATOM	628	HB2	GLN	39	12.519	13.763	-9.042	1.00	0.00
ATOM	629	HB3	GLN	39	13.332	14.158	-7.508	1.00	0.00
ATOM	630	CG	GLN	39	14.671	13.649	-9.119	1.00	0.00
ATOM	631	HG2	GLN	39	14.802	12.819	-9.814	1.00	0.00

ATOM 632 HG3 GLN 39 14.633 14.586 -9.675 1.00 0.00
ATOM 633 CD GLN 39 15.862 13.689 -8.182 1.00 0.00
ATOM 634 OE1 GLN 39 15.752 14.072 -7.020 1.00 0.00
ATOM 635 NE2 GLN 39 17.000 13.263 -8.685 1.00 0.00
ATOM 636 HE21 GLN 39 17.833 13.263 -8.114 1.00 0.00
ATOM 637 HE22 GLN 39 17.036 12.937 -9.641 1.00 0.00
ATOM 638 C GLN 39 14.214 11.681 -6.871 1.00 0.00
ATOM 639 O GLN 39 15.276 11.164 -7.212 1.00 0.00
ATOM 640 N GLN 40 13.893 11.929 -5.597 1.00 0.00
ATOM 641 H GLN 40 13.025 12.395 -5.375 1.00 0.00
ATOM 642 CA GLN 40 14.790 11.606 -4.518 1.00 0.00
ATOM 643 HA GLN 40 15.825 11.703 -4.847 1.00 0.00
ATOM 644 CB GLN 40 14.556 12.564 -3.351 1.00 0.00
ATOM 645 HB2 GLN 40 14.336 13.557 -3.743 1.00 0.00
ATOM 646 HB3 GLN 40 13.709 12.210 -2.764 1.00 0.00
ATOM 647 CG GLN 40 15.803 12.631 -2.460 1.00 0.00
ATOM 648 HG2 GLN 40 16.630 12.213 -3.035 1.00 0.00
ATOM 649 HG3 GLN 40 16.034 13.661 -2.188 1.00 0.00
ATOM 650 CD GLN 40 15.637 11.815 -1.182 1.00 0.00
ATOM 651 OE1 GLN 40 14.561 11.369 -0.803 1.00 0.00
ATOM 652 NE2 GLN 40 16.735 11.654 -0.464 1.00 0.00
ATOM 653 HE21 GLN 40 16.700 11.124 0.395 1.00 0.00

ATOM	654	HE22	GLN	40	17.604	12.061	-0.778	1.00	0.00
ATOM	655	C	GLN	40	14.617	10.154	-4.079	1.00	0.00
ATOM	656	O	GLN	40	15.340	9.661	-3.223	1.00	0.00
ATOM	657	N	GLY	41	13.636	9.461	-4.658	1.00	0.00
ATOM	658	H	GLY	41	13.045	9.934	-5.326	1.00	0.00
ATOM	659	CA	GLY	41	13.375	8.069	-4.379	1.00	0.00
ATOM	660	HA2	GLY	41	14.307	7.583	-4.091	1.00	0.00
ATOM	661	HA3	GLY	41	12.660	7.998	-3.560	1.00	0.00
ATOM	662	C	GLY	41	12.806	7.363	-5.592	1.00	0.00
ATOM	663	O	GLY	41	11.682	6.904	-5.587	1.00	0.00
ATOM	664	N	PRO	42	13.559	7.293	-6.689	1.00	0.00
ATOM	665	CD	PRO	42	14.900	7.822	-6.827	1.00	0.00
ATOM	666	HD2	PRO	42	15.558	7.245	-6.177	1.00	0.00
ATOM	667	HD3	PRO	42	14.887	8.861	-6.500	1.00	0.00
ATOM	668	CG	PRO	42	15.263	7.681	-8.302	1.00	0.00
ATOM	669	HG2	PRO	42	16.113	7.004	-8.396	1.00	0.00
ATOM	670	HG3	PRO	42	15.534	8.660	-8.696	1.00	0.00
ATOM	671	CB	PRO	42	14.026	7.127	-9.016	1.00	0.00
ATOM	672	HB2	PRO	42	14.295	6.297	-9.669	1.00	0.00
ATOM	673	HB3	PRO	42	13.521	7.898	-9.598	1.00	0.00
ATOM	674	CA	PRO	42	13.109	6.633	-7.901	1.00	0.00
ATOM	675	HA	PRO	42	12.096	6.971	-8.119	1.00	0.00

ATOM	676	C	PRO	42	13.118	5.127	-7.760	1.00	0.00
ATOM	677	O	PRO	42	12.279	4.436	-8.351	1.00	0.00
ATOM	678	N	SER	43	14.053	4.629	-6.958	1.00	0.00
ATOM	679	H	SER	43	14.715	5.260	-6.531	1.00	0.00
ATOM	680	CA	SER	43	14.201	3.214	-6.714	1.00	0.00
ATOM	681	HA	SER	43	14.098	2.671	-7.654	1.00	0.00
ATOM	682	CB	SER	43	15.573	2.895	-6.113	1.00	0.00
ATOM	683	HB2	SER	43	15.471	2.842	-5.029	1.00	0.00
ATOM	684	HB3	SER	43	15.900	1.927	-6.494	1.00	0.00
ATOM	685	OG	SER	43	16.534	3.886	-6.451	1.00	0.00
ATOM	686	HG	SER	43	17.380	3.657	-6.060	1.00	0.00
ATOM	687	C	SER	43	13.085	2.734	-5.793	1.00	0.00
ATOM	688	O	SER	43	13.294	2.423	-4.627	1.00	0.00
ATOM	689	N	LEU	44	11.858	2.689	-6.310	1.00	0.00
ATOM	690	H	LEU	44	11.715	2.997	-7.261	1.00	0.00
ATOM	691	CA	LEU	44	10.713	2.266	-5.552	1.00	0.00
ATOM	692	HA	LEU	44	11.037	1.802	-4.620	1.00	0.00
ATOM	693	CB	LEU	44	9.823	3.463	-5.219	1.00	0.00
ATOM	694	HB2	LEU	44	10.041	4.209	-5.983	1.00	0.00
ATOM	695	HB3	LEU	44	8.779	3.159	-5.296	1.00	0.00
ATOM	696	CG	LEU	44	10.081	4.065	-3.827	1.00	0.00
ATOM	697	HG	LEU	44	11.072	3.760	-3.492	1.00	0.00

ATOM	698	CD1	LEU	44	10.005	5.573	-3.867	1.00	0.00
ATOM	699	HD11	LEU	44	9.014	5.878	-4.202	1.00	0.00
ATOM	700	HD12	LEU	44	10.191	5.973	-2.871	1.00	0.00
ATOM	701	HD13	LEU	44	10.755	5.957	-4.558	1.00	0.00
ATOM	702	CD2	LEU	44	9.094	3.537	-2.808	1.00	0.00
ATOM	703	HD21	LEU	44	9.187	2.453	-2.739	1.00	0.00
ATOM	704	HD22	LEU	44	9.303	3.981	-1.835	1.00	0.00
ATOM	705	HD23	LEU	44	8.081	3.795	-3.115	1.00	0.00
ATOM	706	C	LEU	44	9.985	1.239	-6.358	1.00	0.00
ATOM	707	O	LEU	44	9.571	0.271	-5.792	1.00	0.00
ATOM	708	N	ARG	45	9.821	1.411	-7.665	1.00	0.00
ATOM	709	H	ARG	45	10.190	2.252	-8.084	1.00	0.00
ATOM	710	CA	ARG	45	9.147	0.460	-8.518	1.00	0.00
ATOM	711	HA	ARG	45	8.134	0.291	-8.152	1.00	0.00
ATOM	712	CB	ARG	45	9.067	0.981	-9.949	1.00	0.00
ATOM	713	HB2	ARG	45	10.069	1.262	-10.274	1.00	0.00
ATOM	714	HB3	ARG	45	8.689	0.183	-10.589	1.00	0.00
ATOM	715	CG	ARG	45	8.144	2.190	-10.045	1.00	0.00
ATOM	716	HG2	ARG	45	8.653	3.048	-9.606	1.00	0.00
ATOM	717	HG3	ARG	45	7.943	2.387	-11.098	1.00	0.00
ATOM	718	CD	ARG	45	6.816	1.949	-9.306	1.00	0.00
ATOM	719	HD2	ARG	45	6.584	0.884	-9.307	1.00	0.00

ATOM 720 HD3 ARG 45 6.899 2.301 -8.278 1.00 0.00
ATOM 721 NE ARG 45 5.717 2.671 -9.964 1.00 0.00
ATOM 722 HE ARG 45 4.978 2.108 -10.361 1.00 0.00
ATOM 723 CZ ARG 45 5.615 3.985 -10.036 1.00 0.00
ATOM 724 NH1 ARG 45 6.520 4.759 -9.474 1.00 0.00
ATOM 725 HH11 ARG 45 7.300 4.345 -8.983 1.00 0.00
ATOM 726 HH12 ARG 45 6.430 5.763 -9.537 1.00 0.00
ATOM 727 NH2 ARG 45 4.627 4.532 -10.705 1.00 0.00
ATOM 728 HH21 ARG 45 3.946 3.944 -11.164 1.00 0.00
ATOM 729 HH22 ARG 45 4.553 5.538 -10.758 1.00 0.00
ATOM 730 C ARG 45 9.880 -0.839 -8.537 1.00 0.00
ATOM 731 O ARG 45 9.403 -1.789 -7.990 1.00 0.00
ATOM 732 N GLU 46 11.081 -0.876 -9.077 1.00 0.00
ATOM 733 H GLU 46 11.459 -0.036 -9.490 1.00 0.00
ATOM 734 CA GLU 46 11.876 -2.092 -9.138 1.00 0.00
ATOM 735 HA GLU 46 11.362 -2.842 -9.739 1.00 0.00
ATOM 736 CB GLU 46 13.240 -1.803 -9.776 1.00 0.00
ATOM 737 HB2 GLU 46 13.706 -0.971 -9.249 1.00 0.00
ATOM 738 HB3 GLU 46 13.868 -2.689 -9.681 1.00 0.00
ATOM 739 CG GLU 46 13.082 -1.445 -11.259 1.00 0.00
ATOM 740 HG2 GLU 46 12.844 -2.362 -11.799 1.00 0.00
ATOM 741 HG3 GLU 46 12.268 -0.731 -11.382 1.00 0.00

ATOM	742	CD	GLU	46	14.373	-0.847	-11.815	1.00	0.00
ATOM	743	OE1	GLU	46	14.875	0.113	-11.185	1.00	0.00
ATOM	744	OE2	GLU	46	14.825	-1.338	-12.869	1.00	0.00
ATOM	745	C	GLU	46	12.069	-2.679	-7.740	1.00	0.00
ATOM	746	O	GLU	46	12.348	-3.866	-7.599	1.00	0.00
ATOM	747	N	GLN	47	11.884	-1.843	-6.706	1.00	0.00
ATOM	748	H	GLN	47	11.705	-0.867	-6.891	1.00	0.00
ATOM	749	CA	GLN	47	11.983	-2.267	-5.346	1.00	0.00
ATOM	750	HA	GLN	47	12.749	-3.037	-5.252	1.00	0.00
ATOM	751	CB	GLN	47	12.361	-1.093	-4.453	1.00	0.00
ATOM	752	HB2	GLN	47	12.314	-0.173	-5.035	1.00	0.00
ATOM	753	HB3	GLN	47	11.656	-1.035	-3.624	1.00	0.00
ATOM	754	CG	GLN	47	13.781	-1.278	-3.904	1.00	0.00
ATOM	755	HG2	GLN	47	13.859	-2.260	-3.436	1.00	0.00
ATOM	756	HG3	GLN	47	14.511	-1.202	-4.710	1.00	0.00
ATOM	757	CD	GLN	47	14.096	-0.216	-2.861	1.00	0.00
ATOM	758	OE1	GLN	47	15.055	0.533	-2.962	1.00	0.00
ATOM	759	NE2	GLN	47	13.270	-0.165	-1.827	1.00	0.00
ATOM	760	HE21	GLN	47	13.421	0.516	-1.097	1.00	0.00
ATOM	761	HE22	GLN	47	12.493	-0.808	-1.773	1.00	0.00
ATOM	762	C	GLN	47	10.695	-2.880	-4.890	1.00	0.00
ATOM	763	O	GLN	47	10.745	-3.978	-4.424	1.00	0.00

ATOM	764	N	CYX	48	9.560	-2.224	-5.027	1.00	0.00
ATOM	765	H	CYX	48	9.649	-1.296	-5.415	1.00	0.00
ATOM	766	CA	CYX	48	8.260	-2.678	-4.650	1.00	0.00
ATOM	767	HA	CYX	48	8.300	-3.039	-3.622	1.00	0.00
ATOM	768	CB	CYX	48	7.208	-1.583	-4.740	1.00	0.00
ATOM	769	HB2	CYX	48	6.905	-1.287	-3.736	1.00	0.00
ATOM	770	HB3	CYX	48	7.623	-0.722	-5.263	1.00	0.00
ATOM	771	SG	CYX	48	5.673	-2.013	-5.597	1.00	0.00
ATOM	772	C	CYX	48	7.884	-3.823	-5.532	1.00	0.00
ATOM	773	O	CYX	48	7.426	-4.828	-5.064	1.00	0.00
ATOM	774	N	CYX	49	8.113	-3.736	-6.834	1.00	0.00
ATOM	775	H	CYX	49	8.473	-2.854	-7.168	1.00	0.00
ATOM	776	CA	CYX	49	7.843	-4.771	-7.797	1.00	0.00
ATOM	777	HA	CYX	49	6.765	-4.910	-7.875	1.00	0.00
ATOM	778	CB	CYX	49	8.395	-4.427	-9.187	1.00	0.00
ATOM	779	HB2	CYX	49	9.476	-4.300	-9.128	1.00	0.00
ATOM	780	HB3	CYX	49	8.163	-5.235	-9.881	1.00	0.00
ATOM	781	SG	CYX	49	7.774	-2.896	-9.940	1.00	0.00
ATOM	782	C	CYX	49	8.449	-6.071	-7.278	1.00	0.00
ATOM	783	O	CYX	49	7.999	-7.145	-7.648	1.00	0.00
ATOM	784	N	ASN	50	9.481	-5.975	-6.427	1.00	0.00
ATOM	785	H	ASN	50	9.853	-5.059	-6.222	1.00	0.00

ATOM	786	CA	ASN	50	10.069	-7.104	-5.782	1.00	0.00
ATOM	787	HA	ASN	50	9.804	-8.023	-6.305	1.00	0.00
ATOM	788	CB	ASN	50	11.585	-6.955	-5.798	1.00	0.00
ATOM	789	HB2	ASN	50	11.867	-5.938	-6.071	1.00	0.00
ATOM	790	HB3	ASN	50	11.969	-7.184	-4.804	1.00	0.00
ATOM	791	CG	ASN	50	12.209	-7.911	-6.788	1.00	0.00
ATOM	792	OD1	ASN	50	12.777	-8.935	-6.440	1.00	0.00
ATOM	793	ND2	ASN	50	12.077	-7.575	-8.058	1.00	0.00
ATOM	794	HD21	ASN	50	12.469	-8.167	-8.777	1.00	0.00
ATOM	795	HD22	ASN	50	11.585	-6.729	-8.305	1.00	0.00
ATOM	796	C	ASN	50	9.628	-7.206	-4.353	1.00	0.00
ATOM	797	O	ASN	50	9.390	-8.285	-3.901	1.00	0.00
ATOM	798	N	GLU	51	9.515	-6.117	-3.624	1.00	0.00
ATOM	799	H	GLU	51	9.811	-5.261	-4.069	1.00	0.00
ATOM	800	CA	GLU	51	9.099	-6.062	-2.251	1.00	0.00
ATOM	801	HA	GLU	51	9.681	-6.779	-1.672	1.00	0.00
ATOM	802	CB	GLU	51	9.303	-4.666	-1.651	1.00	0.00
ATOM	803	HB2	GLU	51	9.876	-4.766	-0.729	1.00	0.00
ATOM	804	HB3	GLU	51	9.864	-4.062	-2.364	1.00	0.00
ATOM	805	CG	GLU	51	7.968	-3.989	-1.349	1.00	0.00
ATOM	806	HG2	GLU	51	7.425	-4.001	-2.295	1.00	0.00
ATOM	807	HG3	GLU	51	7.387	-4.514	-0.591	1.00	0.00

ATOM	808	CD	GLU	51	8.185	-2.585	-0.921	1.00	0.00
ATOM	809	OE1	GLU	51	8.450	-2.285	0.260	1.00	0.00
ATOM	810	OE2	GLU	51	7.991	-1.745	-1.792	1.00	0.00
ATOM	811	C	GLU	51	7.646	-6.520	-2.132	1.00	0.00
ATOM	812	O	GLU	51	7.149	-6.747	-1.039	1.00	0.00
ATOM	813	N	LEU	52	6.952	-6.645	-3.257	1.00	0.00
ATOM	814	H	LEU	52	7.387	-6.331	-4.113	1.00	0.00
ATOM	815	CA	LEU	52	5.584	-7.057	-3.303	1.00	0.00
ATOM	816	HA	LEU	52	5.160	-7.065	-2.299	1.00	0.00
ATOM	817	CB	LEU	52	4.776	-6.079	-4.169	1.00	0.00
ATOM	818	HB2	LEU	52	3.896	-5.909	-3.549	1.00	0.00
ATOM	819	HB3	LEU	52	5.322	-5.143	-4.288	1.00	0.00
ATOM	820	CG	LEU	52	4.340	-6.594	-5.545	1.00	0.00
ATOM	821	HG	LEU	52	3.973	-5.745	-6.122	1.00	0.00
ATOM	822	CD1	LEU	52	5.469	-7.261	-6.294	1.00	0.00
ATOM	823	HD11	LEU	52	5.836	-8.111	-5.718	1.00	0.00
ATOM	824	HD12	LEU	52	5.108	-7.608	-7.262	1.00	0.00
ATOM	825	HD13	LEU	52	6.279	-6.547	-6.443	1.00	0.00
ATOM	826	CD2	LEU	52	3.140	-7.507	-5.421	1.00	0.00
ATOM	827	HD21	LEU	52	2.311	-6.960	-4.972	1.00	0.00
ATOM	828	HD22	LEU	52	2.848	-7.861	-6.410	1.00	0.00
ATOM	829	HD23	LEU	52	3.395	-8.360	-4.792	1.00	0.00

ATOM	830	C	LEU	52	5.533	-8.513	-3.735	1.00	0.00
ATOM	831	O	LEU	52	4.539	-9.173	-3.489	1.00	0.00
ATOM	832	N	TYR	53	6.592	-8.994	-4.397	1.00	0.00
ATOM	833	H	TYR	53	7.409	-8.407	-4.478	1.00	0.00
ATOM	834	CA	TYR	53	6.642	-10.307	-4.962	1.00	0.00
ATOM	835	HA	TYR	53	5.637	-10.700	-5.115	1.00	0.00
ATOM	836	CB	TYR	53	7.347	-10.228	-6.316	1.00	0.00
ATOM	837	HB2	TYR	53	6.872	-9.422	-6.875	1.00	0.00
ATOM	838	HB3	TYR	53	8.388	-9.971	-6.122	1.00	0.00
ATOM	839	CG	TYR	53	7.285	-11.507	-7.126	1.00	0.00
ATOM	840	CD1	TYR	53	6.046	-12.050	-7.497	1.00	0.00
ATOM	841	HD1	TYR	53	5.133	-11.539	-7.192	1.00	0.00
ATOM	842	CE1	TYR	53	5.970	-13.232	-8.248	1.00	0.00
ATOM	843	HE1	TYR	53	5.006	-13.642	-8.550	1.00	0.00
ATOM	844	CZ	TYR	53	7.160	-13.883	-8.623	1.00	0.00
ATOM	845	OH	TYR	53	7.103	-15.039	-9.333	1.00	0.00
ATOM	846	HH	TYR	53	6.204	-15.319	-9.523	1.00	0.00
ATOM	847	CE2	TYR	53	8.412	-13.350	-8.255	1.00	0.00
ATOM	848	HE2	TYR	53	9.333	-13.856	-8.546	1.00	0.00
ATOM	849	CD2	TYR	53	8.469	-12.158	-7.507	1.00	0.00
ATOM	850	HD2	TYR	53	9.434	-11.739	-7.223	1.00	0.00
ATOM	851	C	TYR	53	7.440	-11.187	-4.053	1.00	0.00

ATOM	852	O	TYR	53	7.227	-12.379	-4.015	1.00	0.00
ATOM	853	N	GLN	54	8.396	-10.624	-3.326	1.00	0.00
ATOM	854	H	GLN	54	8.546	-9.634	-3.460	1.00	0.00
ATOM	855	CA	GLN	54	9.250	-11.324	-2.408	1.00	0.00
ATOM	856	HA	GLN	54	9.876	-12.026	-2.959	1.00	0.00
ATOM	857	CB	GLN	54	10.158	-10.354	-1.642	1.00	0.00
ATOM	858	HB2	GLN	54	10.715	-10.922	-0.897	1.00	0.00
ATOM	859	HB3	GLN	54	10.854	-9.904	-2.350	1.00	0.00
ATOM	860	CG	GLN	54	9.353	-9.254	-0.948	1.00	0.00
ATOM	861	HG2	GLN	54	8.873	-8.605	-1.681	1.00	0.00
ATOM	862	HG3	GLN	54	8.590	-9.742	-0.341	1.00	0.00
ATOM	863	CD	GLN	54	10.220	-8.405	-0.044	1.00	0.00
ATOM	864	OE1	GLN	54	11.386	-8.136	-0.318	1.00	0.00
ATOM	865	NE2	GLN	54	9.635	-7.935	1.035	1.00	0.00
ATOM	866	HE21	GLN	54	10.159	-7.361	1.680	1.00	0.00
ATOM	867	HE22	GLN	54	8.664	-8.150	1.214	1.00	0.00
ATOM	868	C	GLN	54	8.400	-12.129	-1.437	1.00	0.00
ATOM	869	O	GLN	54	8.847	-13.155	-0.940	1.00	0.00
ATOM	870	N	GLU	55	7.165	-11.671	-1.202	1.00	0.00
ATOM	871	H	GLU	55	6.894	-10.775	-1.582	1.00	0.00
ATOM	872	CA	GLU	55	6.237	-12.357	-0.349	1.00	0.00
ATOM	873	HA	GLU	55	6.775	-12.873	0.446	1.00	0.00

ATOM 874 CB GLU 55 5.273 -11.369 0.280 1.00 0.00
ATOM 875 HB2 GLU 55 4.575 -11.034 -0.488 1.00 0.00
ATOM 876 HB3 GLU 55 4.724 -11.881 1.070 1.00 0.00
ATOM 877 CG GLU 55 6.000 -10.167 0.864 1.00 0.00
ATOM 878 HG2 GLU 55 6.931 -10.562 1.269 1.00 0.00
ATOM 879 HG3 GLU 55 6.224 -9.418 0.105 1.00 0.00
ATOM 880 CD GLU 55 5.196 -9.534 1.975 1.00 0.00
ATOM 881 OE1 GLU 55 4.256 -10.157 2.502 1.00 0.00
ATOM 882 OE2 GLU 55 5.510 -8.369 2.221 1.00 0.00
ATOM 883 C GLU 55 5.510 -13.452 -1.098 1.00 0.00
ATOM 884 O GLU 55 4.474 -13.912 -0.660 1.00 0.00
ATOM 885 N ASP 56 6.043 -13.900 -2.224 1.00 0.00
ATOM 886 H ASP 56 6.932 -13.503 -2.492 1.00 0.00
ATOM 887 CA ASP 56 5.456 -14.890 -3.079 1.00 0.00
ATOM 888 HA ASP 56 6.207 -15.309 -3.749 1.00 0.00
ATOM 889 CB ASP 56 4.881 -16.022 -2.220 1.00 0.00
ATOM 890 HB2 ASP 56 5.571 -16.054 -1.377 1.00 0.00
ATOM 891 HB3 ASP 56 3.883 -15.767 -1.864 1.00 0.00
ATOM 892 CG ASP 56 4.844 -17.374 -2.891 1.00 0.00
ATOM 893 OD1 ASP 56 5.278 -17.461 -4.060 1.00 0.00
ATOM 894 OD2 ASP 56 4.289 -18.305 -2.273 1.00 0.00
ATOM 895 C ASP 56 4.342 -14.271 -3.911 1.00 0.00

ATOM	896	O	ASP	56	4.116	-13.054	-3.938	1.00	0.00
ATOM	897	N	GLN	57	3.578	-15.130	-4.597	1.00	0.00
ATOM	898	H	GLN	57	3.808	-16.114	-4.589	1.00	0.00
ATOM	899	CA	GLN	57	2.462	-14.696	-5.399	1.00	0.00
ATOM	900	HA	GLN	57	2.632	-13.681	-5.758	1.00	0.00
ATOM	901	CB	GLN	57	2.295	-15.620	-6.606	1.00	0.00
ATOM	902	HB2	GLN	57	3.215	-16.186	-6.751	1.00	0.00
ATOM	903	HB3	GLN	57	1.472	-16.309	-6.416	1.00	0.00
ATOM	904	CG	GLN	57	1.996	-14.805	-7.863	1.00	0.00
ATOM	905	HG2	GLN	57	2.605	-13.901	-7.846	1.00	0.00
ATOM	906	HG3	GLN	57	2.227	-15.382	-8.759	1.00	0.00
ATOM	907	CD	GLN	57	0.542	-14.406	-7.909	1.00	0.00
ATOM	908	OE1	GLN	57	-0.349	-15.204	-8.053	1.00	0.00
ATOM	909	NE2	GLN	57	0.274	-13.133	-7.809	1.00	0.00
ATOM	910	HE21	GLN	57	-0.686	-12.819	-7.835	1.00	0.00
ATOM	911	HE22	GLN	57	1.027	-12.468	-7.706	1.00	0.00
ATOM	912	C	GLN	57	1.186	-14.655	-4.585	1.00	0.00
ATOM	913	O	GLN	57	0.255	-13.913	-4.878	1.00	0.00
ATOM	914	N	VAL	58	1.146	-15.431	-3.510	1.00	0.00
ATOM	915	H	VAL	58	1.924	-16.051	-3.336	1.00	0.00
ATOM	916	CA	VAL	58	0.002	-15.498	-2.621	1.00	0.00
ATOM	917	HA	VAL	58	-0.936	-15.488	-3.176	1.00	0.00

ATOM 918 CB VAL 58 0.054 -16.773 -1.770 1.00 0.00

ATOM 919 HB VAL 58 0.393 -16.519 -0.766 1.00 0.00

ATOM 920 CG1 VAL 58 -1.335 -17.414 -1.693 1.00 0.00

ATOM 921 HG11 VAL 58 -1.674 -17.669 -2.697 1.00 0.00

ATOM 922 HG12 VAL 58 -1.285 -18.319 -1.086 1.00 0.00

ATOM 923 HG13 VAL 58 -2.035 -16.712 -1.241 1.00 0.00

ATOM 924 CG2 VAL 58 1.061 -17.782 -2.332 1.00 0.00

ATOM 925 HG21 VAL 58 2.055 -17.336 -2.345 1.00 0.00

ATOM 926 HG22 VAL 58 1.072 -18.674 -1.705 1.00 0.00

ATOM 927 HG23 VAL 58 0.773 -18.057 -3.347 1.00 0.00

ATOM 928 C VAL 58 -0.043 -14.238 -1.778 1.00 0.00

ATOM 929 O VAL 58 -0.986 -14.063 -1.009 1.00 0.00

ATOM 930 N CYX 59 0.942 -13.352 -1.954 1.00 0.00

ATOM 931 H CYX 59 1.681 -13.574 -2.605 1.00 0.00

ATOM 932 CA CYX 59 1.017 -12.115 -1.238 1.00 0.00

ATOM 933 HA CYX 59 0.187 -12.048 -0.535 1.00 0.00

ATOM 934 CB CYX 59 2.307 -12.019 -0.469 1.00 0.00

ATOM 935 HB2 CYX 59 3.127 -12.383 -1.088 1.00 0.00

ATOM 936 HB3 CYX 59 2.492 -10.980 -0.196 1.00 0.00

ATOM 937 SG CYX 59 2.383 -12.993 1.078 1.00 0.00

ATOM 938 C CYX 59 0.906 -10.965 -2.208 1.00 0.00

ATOM 939 O CYX 59 0.321 -9.928 -1.880 1.00 0.00

```
ATOM    940  N   VAL    60       1.349 -11.176  -3.456  1.00  0.00
ATOM    941  H   VAL    60       1.817 -12.045  -3.670  1.00  0.00
ATOM    942  CA  VAL    60       1.247 -10.179  -4.497  1.00  0.00
ATOM    943  HA  VAL    60       1.863  -9.298  -4.321  1.00  0.00
ATOM    944  CB  VAL    60       1.638 -10.786  -5.839  1.00  0.00
ATOM    945  HB  VAL    60       1.285 -11.817  -5.874  1.00  0.00
ATOM    946  CG1 VAL    60       1.025 -10.000  -6.993  1.00  0.00
ATOM    947 HG11 VAL    60       1.378  -8.970  -6.960  1.00  0.00
ATOM    948 HG12 VAL    60       1.319 -10.454  -7.939  1.00  0.00
ATOM    949 HG13 VAL    60      -0.062 -10.015  -6.906  1.00  0.00
ATOM    950  CG2 VAL    60       3.142 -10.883  -5.975  1.00  0.00
ATOM    951 HG21 VAL    60       3.539 -11.513  -5.179  1.00  0.00
ATOM    952 HG22 VAL    60       3.392 -11.320  -6.942  1.00  0.00
ATOM    953 HG23 VAL    60       3.579  -9.887  -5.903  1.00  0.00
ATOM    954  C   VAL    60      -0.153  -9.610  -4.527  1.00  0.00
ATOM    955  O   VAL    60      -0.351  -8.485  -4.952  1.00  0.00
ATOM    956  N   CYX    61      -1.145 -10.385  -4.096  1.00  0.00
ATOM    957  H   CYX    61      -0.926 -11.349  -3.887  1.00  0.00
ATOM    958  CA  CYX    61      -2.492  -9.963  -3.985  1.00  0.00
ATOM    959  HA  CYX    61      -2.595  -9.237  -3.179  1.00  0.00
ATOM    960  CB  CYX    61      -2.929  -9.309  -5.288  1.00  0.00
ATOM    961  HB2 CYX    61      -2.164  -9.466  -6.048  1.00  0.00
```

ATOM 962 HB3 CYX 61 -3.867 -9.753 -5.621 1.00 0.00
ATOM 963 SG CYX 61 -3.201 -7.519 -5.232 1.00 0.00
ATOM 964 C CYX 61 -3.355 -11.161 -3.609 1.00 0.00
ATOM 965 O CYX 61 -2.903 -12.284 -3.709 1.00 0.00
ATOM 966 N PRO 62 -4.604 -10.905 -3.186 1.00 0.00
ATOM 967 CD PRO 62 -5.613 -11.858 -2.769 1.00 0.00
ATOM 968 HD2 PRO 62 -5.259 -12.356 -1.866 1.00 0.00
ATOM 969 HD3 PRO 62 -5.737 -12.594 -3.564 1.00 0.00
ATOM 970 CG PRO 62 -6.887 -11.036 -2.528 1.00 0.00
ATOM 971 HG2 PRO 62 -7.171 -11.130 -1.480 1.00 0.00
ATOM 972 HG3 PRO 62 -7.684 -11.433 -3.157 1.00 0.00
ATOM 973 CB PRO 62 -6.574 -9.584 -2.894 1.00 0.00
ATOM 974 HB2 PRO 62 -6.920 -8.874 -2.144 1.00 0.00
ATOM 975 HB3 PRO 62 -7.010 -9.332 -3.861 1.00 0.00
ATOM 976 CA PRO 62 -5.063 -9.544 -2.982 1.00 0.00
ATOM 977 HA PRO 62 -4.828 -8.971 -3.879 1.00 0.00
ATOM 978 C PRO 62 -4.423 -8.900 -1.798 1.00 0.00
ATOM 979 O PRO 62 -4.710 -7.781 -1.504 1.00 0.00
ATOM 980 N THR 63 -3.591 -9.612 -1.083 1.00 0.00
ATOM 981 H THR 63 -3.498 -10.580 -1.357 1.00 0.00
ATOM 982 CA THR 63 -2.856 -9.155 0.070 1.00 0.00
ATOM 983 HA THR 63 -3.559 -9.248 0.898 1.00 0.00

ATOM 984 CB THR 63 -1.676 -10.087 0.326 1.00 0.00
ATOM 985 HB THR 63 -0.767 -9.579 0.004 1.00 0.00
ATOM 986 CG2 THR 63 -1.552 -10.465 1.778 1.00 0.00
ATOM 987 HG21 THR 63 -2.460 -10.974 2.100 1.00 0.00
ATOM 988 HG22 THR 63 -0.698 -11.129 1.909 1.00 0.00
ATOM 989 HG23 THR 63 -1.408 -9.566 2.377 1.00 0.00
ATOM 990 OG1 THR 63 -1.991 -11.310 -0.327 1.00 0.00
ATOM 991 HG1 THR 63 -1.275 -11.937 -0.196 1.00 0.00
ATOM 992 C THR 63 -2.386 -7.747 -0.103 1.00 0.00
ATOM 993 O THR 63 -2.701 -6.970 0.774 1.00 0.00
ATOM 994 N LEU 64 -1.785 -7.398 -1.240 1.00 0.00
ATOM 995 H LEU 64 -1.599 -8.123 -1.919 1.00 0.00
ATOM 996 CA LEU 64 -1.345 -6.049 -1.536 1.00 0.00
ATOM 997 HA LEU 64 -0.477 -5.795 -0.928 1.00 0.00
ATOM 998 CB LEU 64 -0.956 -5.944 -3.009 1.00 0.00
ATOM 999 HB2 LEU 64 -0.775 -6.973 -3.319 1.00 0.00
ATOM 1000 HB3 LEU 64 -1.791 -5.536 -3.578 1.00 0.00
ATOM 1001 CG LEU 64 0.300 -5.103 -3.270 1.00 0.00
ATOM 1002 HG LEU 64 0.942 -5.143 -2.390 1.00 0.00
ATOM 1003 CD1 LEU 64 1.054 -5.633 -4.468 1.00 0.00
ATOM 1004 HD11 LEU 64 0.413 -5.593 -5.349 1.00 0.00
ATOM 1005 HD12 LEU 64 1.942 -5.024 -4.638 1.00 0.00

ATOM	1006	HD13	LEU	64	1.352	-6.665	-4.283	1.00	0.00
ATOM	1007	CD2	LEU	64	-0.054	-3.632	-3.468	1.00	0.00
ATOM	1008	HD21	LEU	64	-0.547	-3.253	-2.573	1.00	0.00
ATOM	1009	HD22	LEU	64	0.856	-3.061	-3.651	1.00	0.00
ATOM	1010	HD23	LEU	64	-0.724	-3.531	-4.322	1.00	0.00
ATOM	1011	C	LEU	64	-2.420	-5.040	-1.208	1.00	0.00
ATOM	1012	O	LEU	64	-2.106	-4.022	-0.603	1.00	0.00
ATOM	1013	N	LYS	65	-3.691	-5.370	-1.498	1.00	0.00
ATOM	1014	H	LYS	65	-3.834	-6.191	-2.070	1.00	0.00
ATOM	1015	CA	LYS	65	-4.841	-4.577	-1.142	1.00	0.00
ATOM	1016	HA	LYS	65	-4.897	-3.708	-1.798	1.00	0.00
ATOM	1017	CB	LYS	65	-6.142	-5.380	-1.281	1.00	0.00
ATOM	1018	HB2	LYS	65	-6.256	-5.686	-2.321	1.00	0.00
ATOM	1019	HB3	LYS	65	-6.077	-6.265	-0.648	1.00	0.00
ATOM	1020	CG	LYS	65	-7.345	-4.544	-0.863	1.00	0.00
ATOM	1021	HG2	LYS	65	-7.082	-3.490	-0.950	1.00	0.00
ATOM	1022	HG3	LYS	65	-8.174	-4.768	-1.535	1.00	0.00
ATOM	1023	CD	LYS	65	-7.762	-4.849	0.582	1.00	0.00
ATOM	1024	HD2	LYS	65	-6.947	-5.369	1.085	1.00	0.00
ATOM	1025	HD3	LYS	65	-7.968	-3.912	1.098	1.00	0.00
ATOM	1026	CE	LYS	65	-9.014	-5.727	0.601	1.00	0.00
ATOM	1027	HE2	LYS	65	-9.576	-5.566	-0.319	1.00	0.00

ATOM	1028	HE3	LYS	65	-8.715	-6.773	0.664	1.00	0.00
ATOM	1029	NZ	LYS	65	-9.861	-5.387	1.761	1.00	0.00
ATOM	1030	HZ1	LYS	65	-10.139	-4.418	1.702	1.00	0.00
ATOM	1031	HZ2	LYS	65	-10.683	-5.974	1.761	1.00	0.00
ATOM	1032	HZ3	LYS	65	-9.340	-5.537	2.613	1.00	0.00
ATOM	1033	C	LYS	65	-4.660	-4.006	0.246	1.00	0.00
ATOM	1034	O	LYS	65	-5.108	-2.928	0.485	1.00	0.00
ATOM	1035	N	GLN	66	-4.028	-4.702	1.170	1.00	0.00
ATOM	1036	H	GLN	66	-3.840	-5.664	0.926	1.00	0.00
ATOM	1037	CA	GLN	66	-3.674	-4.257	2.467	1.00	0.00
ATOM	1038	HA	GLN	66	-4.561	-3.881	2.977	1.00	0.00
ATOM	1039	CB	GLN	66	-3.078	-5.397	3.299	1.00	0.00
ATOM	1040	HB2	GLN	66	-3.785	-6.226	3.331	1.00	0.00
ATOM	1041	HB3	GLN	66	-2.148	-5.731	2.838	1.00	0.00
ATOM	1042	CG	GLN	66	-2.794	-4.909	4.732	1.00	0.00
ATOM	1043	HG2	GLN	66	-2.516	-5.718	5.408	1.00	0.00
ATOM	1044	HG3	GLN	66	-1.966	-4.205	4.649	1.00	0.00
ATOM	1045	CD	GLN	66	-3.999	-4.178	5.316	1.00	0.00
ATOM	1046	OE1	GLN	66	-3.886	-3.145	5.952	1.00	0.00
ATOM	1047	NE2	GLN	66	-5.187	-4.692	5.010	1.00	0.00
ATOM	1048	HE21	GLN	66	-6.028	-4.258	5.363	1.00	0.00
ATOM	1049	HE22	GLN	66	-5.243	-5.514	4.426	1.00	0.00

ATOM	1050	C	GLN	66	-2.728	-3.083	2.449	1.00	0.00
ATOM	1051	O	GLN	66	-3.119	-1.980	2.810	1.00	0.00
ATOM	1052	N	ALA	67	-1.473	-3.306	2.065	1.00	0.00
ATOM	1053	H	ALA	67	-1.174	-4.253	1.878	1.00	0.00
ATOM	1054	CA	ALA	67	-0.497	-2.247	1.963	1.00	0.00
ATOM	1055	HA	ALA	67	-0.278	-1.836	2.948	1.00	0.00
ATOM	1056	CB	ALA	67	0.791	-2.807	1.372	1.00	0.00
ATOM	1057	HB1	ALA	67	0.590	-3.214	0.381	1.00	0.00
ATOM	1058	HB2	ALA	67	1.532	-2.011	1.294	1.00	0.00
ATOM	1059	HB3	ALA	67	1.174	-3.597	2.018	1.00	0.00
ATOM	1060	C	ALA	67	-1.050	-1.103	1.120	1.00	0.00
ATOM	1061	O	ALA	67	-0.672	0.039	1.316	1.00	0.00
ATOM	1062	N	ALA	68	-2.027	-1.378	0.263	1.00	0.00
ATOM	1063	H	ALA	68	-2.302	-2.343	0.148	1.00	0.00
ATOM	1064	CA	ALA	68	-2.671	-0.387	-0.546	1.00	0.00
ATOM	1065	HA	ALA	68	-1.981	0.425	-0.777	1.00	0.00
ATOM	1066	CB	ALA	68	-3.109	-1.040	-1.851	1.00	0.00
ATOM	1067	HB1	ALA	68	-3.807	-1.849	-1.637	1.00	0.00
ATOM	1068	HB2	ALA	68	-3.597	-0.298	-2.484	1.00	0.00
ATOM	1069	HB3	ALA	68	-2.237	-1.441	-2.368	1.00	0.00
ATOM	1070	C	ALA	68	-3.850	0.231	0.192	1.00	0.00
ATOM	1071	O	ALA	68	-4.222	1.382	-0.009	1.00	0.00

ATOM	1072	N	LYS	69	-4.462	-0.510	1.104	1.00	0.00
ATOM	1073	H	LYS	69	-4.163	-1.471	1.187	1.00	0.00
ATOM	1074	CA	LYS	69	-5.566	-0.069	1.907	1.00	0.00
ATOM	1075	HA	LYS	69	-6.268	0.489	1.287	1.00	0.00
ATOM	1076	CB	LYS	69	-6.304	-1.262	2.531	1.00	0.00
ATOM	1077	HB2	LYS	69	-6.662	-1.911	1.732	1.00	0.00
ATOM	1078	HB3	LYS	69	-5.608	-1.815	3.163	1.00	0.00
ATOM	1079	CG	LYS	69	-7.491	-0.788	3.373	1.00	0.00
ATOM	1080	HG2	LYS	69	-8.118	-1.649	3.607	1.00	0.00
ATOM	1081	HG3	LYS	69	-7.111	-0.354	4.298	1.00	0.00
ATOM	1082	CD	LYS	69	-8.322	0.261	2.618	1.00	0.00
ATOM	1083	HD2	LYS	69	-7.779	1.206	2.624	1.00	0.00
ATOM	1084	HD3	LYS	69	-8.454	-0.075	1.589	1.00	0.00
ATOM	1085	CE	LYS	69	-9.684	0.455	3.267	1.00	0.00
ATOM	1086	HE2	LYS	69	-10.412	-0.173	2.753	1.00	0.00
ATOM	1087	HE3	LYS	69	-9.622	0.156	4.313	1.00	0.00
ATOM	1088	NZ	LYS	69	-10.106	1.866	3.183	1.00	0.00
ATOM	1089	HZ1	LYS	69	-10.164	2.142	2.213	1.00	0.00
ATOM	1090	HZ2	LYS	69	-11.011	1.973	3.619	1.00	0.00
ATOM	1091	HZ3	LYS	69	-9.432	2.448	3.659	1.00	0.00
ATOM	1092	C	LYS	69	-5.055	0.902	2.942	1.00	0.00
ATOM	1093	O	LYS	69	-5.741	1.852	3.260	1.00	0.00

ATOM	1094	N	SER	70	-3.834	0.716	3.427	1.00	0.00
ATOM	1095	H	SER	70	-3.315	-0.099	3.133	1.00	0.00
ATOM	1096	CA	SER	70	-3.228	1.602	4.397	1.00	0.00
ATOM	1097	HA	SER	70	-3.996	2.218	4.865	1.00	0.00
ATOM	1098	CB	SER	70	-2.519	0.801	5.481	1.00	0.00
ATOM	1099	HB2	SER	70	-3.235	0.109	5.925	1.00	0.00
ATOM	1100	HB3	SER	70	-1.707	0.237	5.021	1.00	0.00
ATOM	1101	OG	SER	70	-1.993	1.650	6.490	1.00	0.00
ATOM	1102	HG	SER	70	-1.553	1.119	7.158	1.00	0.00
ATOM	1103	C	SER	70	-2.257	2.539	3.705	1.00	0.00
ATOM	1104	O	SER	70	-1.453	3.227	4.336	1.00	0.00
ATOM	1105	N	VAL	71	-2.317	2.592	2.369	1.00	0.00
ATOM	1106	H	VAL	71	-2.933	1.962	1.875	1.00	0.00
ATOM	1107	CA	VAL	71	-1.461	3.464	1.593	1.00	0.00
ATOM	1108	HA	VAL	71	-0.404	3.366	1.840	1.00	0.00
ATOM	1109	CB	VAL	71	-1.642	3.180	0.102	1.00	0.00
ATOM	1110	HB	VAL	71	-2.110	2.201	-0.011	1.00	0.00
ATOM	1111	CG1	VAL	71	-2.505	4.228	-0.555	1.00	0.00
ATOM	1112	HG11	VAL	71	-2.038	5.206	-0.443	1.00	0.00
ATOM	1113	HG12	VAL	71	-2.614	3.997	-1.615	1.00	0.00
ATOM	1114	HG13	VAL	71	-3.487	4.238	-0.083	1.00	0.00
ATOM	1115	CG2	VAL	71	-0.311	3.062	-0.612	1.00	0.00

ATOM	1116	HG21	VAL	71	0.265	2.246	-0.175	1.00	0.00
ATOM	1117	HG22	VAL	71	-0.482	2.860	-1.670	1.00	0.00
ATOM	1118	HG23	VAL	71	0.243	3.994	-0.506	1.00	0.00
ATOM	1119	C	VAL	71	-1.770	4.907	1.965	1.00	0.00
ATOM	1120	O	VAL	71	-1.060	5.831	1.627	1.00	0.00
ATOM	1121	N	ARG	72	-2.896	5.161	2.625	1.00	0.00
ATOM	1122	H	ARG	72	-3.529	4.396	2.812	1.00	0.00
ATOM	1123	CA	ARG	72	-3.275	6.479	3.055	1.00	0.00
ATOM	1124	HA	ARG	72	-2.397	7.048	3.360	1.00	0.00
ATOM	1125	CB	ARG	72	-3.951	7.208	1.891	1.00	0.00
ATOM	1126	HB2	ARG	72	-3.932	6.565	1.010	1.00	0.00
ATOM	1127	HB3	ARG	72	-4.985	7.424	2.160	1.00	0.00
ATOM	1128	CG	ARG	72	-3.221	8.514	1.584	1.00	0.00
ATOM	1129	HG2	ARG	72	-2.289	8.528	2.148	1.00	0.00
ATOM	1130	HG3	ARG	72	-3.000	8.542	0.517	1.00	0.00
ATOM	1131	CD	ARG	72	-4.060	9.728	1.960	1.00	0.00
ATOM	1132	HD2	ARG	72	-4.793	9.908	1.173	1.00	0.00
ATOM	1133	HD3	ARG	72	-4.577	9.522	2.897	1.00	0.00
ATOM	1134	NE	ARG	72	-3.245	10.935	2.128	1.00	0.00
ATOM	1135	HE	ARG	72	-3.292	11.622	1.390	1.00	0.00
ATOM	1136	CZ	ARG	72	-2.452	11.172	3.165	1.00	0.00
ATOM	1137	NH1	ARG	72	-2.393	10.315	4.160	1.00	0.00

ATOM 1138 HH11 ARG 72 -2.953 9.475 4.133 1.00 0.00

ATOM 1139 HH12 ARG 72 -1.788 10.500 4.947 1.00 0.00

ATOM 1140 NH2 ARG 72 -1.737 12.267 3.206 1.00 0.00

ATOM 1141 HH21 ARG 72 -1.791 12.930 2.446 1.00 0.00

ATOM 1142 HH22 ARG 72 -1.135 12.443 3.997 1.00 0.00

ATOM 1143 C ARG 72 -4.200 6.386 4.240 1.00 0.00

ATOM 1144 O ARG 72 -3.795 6.728 5.343 1.00 0.00

ATOM 1145 N VAL 73 -5.465 6.019 3.974 1.00 0.00

ATOM 1146 H VAL 73 -5.739 5.865 3.014 1.00 0.00

ATOM 1147 CA VAL 73 -6.477 5.918 4.999 1.00 0.00

ATOM 1148 HA VAL 73 -6.761 6.887 5.409 1.00 0.00

ATOM 1149 CB VAL 73 -7.725 5.246 4.426 1.00 0.00

ATOM 1150 HB VAL 73 -7.895 5.641 3.425 1.00 0.00

ATOM 1151 CG1 VAL 73 -7.577 3.733 4.355 1.00 0.00

ATOM 1152 HG11 VAL 73 -7.407 3.336 5.356 1.00 0.00

ATOM 1153 HG12 VAL 73 -8.487 3.297 3.942 1.00 0.00

ATOM 1154 HG13 VAL 73 -6.731 3.479 3.716 1.00 0.00

ATOM 1155 CG2 VAL 73 -8.964 5.611 5.247 1.00 0.00

ATOM 1156 HG21 VAL 73 -9.107 6.691 5.229 1.00 0.00

ATOM 1157 HG22 VAL 73 -9.840 5.121 4.821 1.00 0.00

ATOM 1158 HG23 VAL 73 -8.829 5.280 6.277 1.00 0.00

ATOM 1159 C VAL 73 -5.896 5.171 6.198 1.00 0.00

ATOM 1160 O VAL 73 -4.930 4.408 6.075 1.00 0.00

ATOM 1161 N GLN 74 -6.524 5.360 7.344 1.00 0.00

ATOM 1162 H GLN 74 -7.299 6.005 7.399 1.00 0.00

ATOM 1163 CA GLN 74 -6.080 4.715 8.562 1.00 0.00

ATOM 1164 HA GLN 74 -6.096 3.632 8.433 1.00 0.00

ATOM 1165 CB GLN 74 -4.650 5.146 8.908 1.00 0.00

ATOM 1166 HB2 GLN 74 -4.166 5.530 8.010 1.00 0.00

ATOM 1167 HB3 GLN 74 -4.687 5.930 9.664 1.00 0.00

ATOM 1168 CG GLN 74 -3.853 3.953 9.447 1.00 0.00

ATOM 1169 HG2 GLN 74 -4.547 3.266 9.932 1.00 0.00

ATOM 1170 HG3 GLN 74 -3.346 3.439 8.630 1.00 0.00

ATOM 1171 CD GLN 74 -2.811 4.392 10.466 1.00 0.00

ATOM 1172 OE1 GLN 74 -2.468 5.559 10.595 1.00 0.00

ATOM 1173 NE2 GLN 74 -2.280 3.425 11.192 1.00 0.00

ATOM 1174 HE21 GLN 74 -1.582 3.645 11.888 1.00 0.00

ATOM 1175 HE22 GLN 74 -2.575 2.469 11.048 1.00 0.00

ATOM 1176 C GLN 74 -7.038 5.017 9.710 1.00 0.00

ATOM 1177 O GLN 74 -6.642 5.193 10.856 1.00 0.00

ATOM 1178 N GLY 75 -8.326 5.145 9.378 1.00 0.00

ATOM 1179 H GLY 75 -8.607 5.040 8.413 1.00 0.00

ATOM 1180 CA GLY 75 -9.347 5.432 10.363 1.00 0.00

ATOM 1181 HA2 GLY 75 -9.462 4.572 11.023 1.00 0.00

ATOM	1182	HA3	GLY	75	-9.048	6.301	10.948	1.00	0.00
ATOM	1183	C	GLY	75	-10.670	5.720	9.697	1.00	0.00
ATOM	1184	O	GLY	75	-11.658	5.044	9.967	1.00	0.00
ATOM	1185	N	GLN	76	-10.670	6.680	8.759	1.00	0.00
ATOM	1186	H	GLN	76	-9.818	7.186	8.566	1.00	0.00
ATOM	1187	CA	GLN	76	-11.872	7.063	8.048	1.00	0.00
ATOM	1188	HA	GLN	76	-12.184	6.244	7.400	1.00	0.00
ATOM	1189	CB	GLN	76	-13.017	7.386	9.016	1.00	0.00
ATOM	1190	HB2	GLN	76	-12.756	7.021	10.009	1.00	0.00
ATOM	1191	HB3	GLN	76	-13.159	8.466	9.051	1.00	0.00
ATOM	1192	CG	GLN	76	-14.313	6.717	8.551	1.00	0.00
ATOM	1193	HG2	GLN	76	-14.367	6.744	7.463	1.00	0.00
ATOM	1194	HG3	GLN	76	-14.340	5.681	8.889	1.00	0.00
ATOM	1195	CD	GLN	76	-15.521	7.443	9.115	1.00	0.00
ATOM	1196	OE1	GLN	76	-16.096	8.310	8.478	1.00	0.00
ATOM	1197	NE2	GLN	76	-15.893	7.078	10.325	1.00	0.00
ATOM	1198	HE21	GLN	76	-16.690	7.521	10.760	1.00	0.00
ATOM	1199	HE22	GLN	76	-15.380	6.357	10.812	1.00	0.00
ATOM	1200	C	GLN	76	-11.597	8.251	7.146	1.00	0.00
ATOM	1201	O	GLN	76	-11.418	9.378	7.595	1.00	0.00
ATOM	1202	N	HID	77	-11.480	7.983	5.847	1.00	0.00
ATOM	1203	H	HID	77	-11.464	7.020	5.543	1.00	0.00

ATOM 1204 CA HID 77 -11.246 9.022 4.857 1.00 0.00

ATOM 1205 HA HID 77 -11.415 10.006 5.294 1.00 0.00

ATOM 1206 CB HID 77 -9.797 8.945 4.371 1.00 0.00

ATOM 1207 HB2 HID 77 -9.112 9.110 5.202 1.00 0.00

ATOM 1208 HB3 HID 77 -9.594 7.972 3.922 1.00 0.00

ATOM 1209 CG HID 77 -9.466 9.978 3.328 1.00 0.00

ATOM 1210 ND1 HID 77 -8.846 11.180 3.593 1.00 0.00

ATOM 1211 HD1 HID 77 -8.552 11.502 4.504 1.00 0.00

ATOM 1212 CE1 HID 77 -8.701 11.848 2.432 1.00 0.00

ATOM 1213 HE1 HID 77 -8.220 12.826 2.417 1.00 0.00

ATOM 1214 NE2 HID 77 -9.225 11.108 1.440 1.00 0.00

ATOM 1215 CD2 HID 77 -9.701 9.925 1.976 1.00 0.00

ATOM 1216 HD2 HID 77 -10.156 9.176 1.327 1.00 0.00

ATOM 1217 C HID 77 -12.278 8.924 3.740 1.00 0.00

ATOM 1218 O HID 77 -12.167 9.592 2.723 1.00 0.00

ATOM 1219 N GLY 78 -13.287 8.072 3.925 1.00 0.00

ATOM 1220 H GLY 78 -13.343 7.549 4.787 1.00 0.00

ATOM 1221 CA GLY 78 -14.311 7.866 2.933 1.00 0.00

ATOM 1222 HA2 GLY 78 -14.976 7.069 3.268 1.00 0.00

ATOM 1223 HA3 GLY 78 -14.881 8.787 2.814 1.00 0.00

ATOM 1224 C GLY 78 -13.708 7.475 1.582 1.00 0.00

ATOM 1225 O GLY 78 -12.485 7.299 1.427 1.00 0.00

ATOM 1226 N PRO 79 -14.585 7.273 0.598 1.00 0.00
ATOM 1227 CD PRO 79 -16.032 7.401 0.699 1.00 0.00
ATOM 1228 HD2 PRO 79 -16.268 8.452 0.861 1.00 0.00
ATOM 1229 HD3 PRO 79 -16.364 6.818 1.558 1.00 0.00
ATOM 1230 CG PRO 79 -16.596 6.868 -0.622 1.00 0.00
ATOM 1231 HG2 PRO 79 -17.110 7.677 -1.140 1.00 0.00
ATOM 1232 HG3 PRO 79 -17.304 6.067 -0.408 1.00 0.00
ATOM 1233 CB PRO 79 -15.409 6.345 -1.440 1.00 0.00
ATOM 1234 HB2 PRO 79 -15.442 6.687 -2.475 1.00 0.00
ATOM 1235 HB3 PRO 79 -15.377 5.255 -1.419 1.00 0.00
ATOM 1236 CA PRO 79 -14.176 6.916 -0.743 1.00 0.00
ATOM 1237 HA PRO 79 -13.452 6.104 -0.672 1.00 0.00
ATOM 1238 C PRO 79 -13.553 8.084 -1.483 1.00 0.00
ATOM 1239 O PRO 79 -14.255 8.934 -2.010 1.00 0.00
ATOM 1240 N PHE 80 -12.215 8.123 -1.508 1.00 0.00
ATOM 1241 H PHE 80 -11.677 7.388 -1.070 1.00 0.00
ATOM 1242 CA PHE 80 -11.502 9.201 -2.157 1.00 0.00
ATOM 1243 HA PHE 80 -12.061 9.590 -3.008 1.00 0.00
ATOM 1244 CB PHE 80 -11.334 10.321 -1.130 1.00 0.00
ATOM 1245 HB2 PHE 80 -12.178 10.328 -0.440 1.00 0.00
ATOM 1246 HB3 PHE 80 -10.410 10.172 -0.572 1.00 0.00
ATOM 1247 CG PHE 80 -11.257 11.698 -1.731 1.00 0.00

ATOM	1248	CD1	PHE	80	-12.427	12.339	-2.160	1.00	0.00
ATOM	1249	HD1	PHE	80	-13.388	11.835	-2.059	1.00	0.00
ATOM	1250	CE1	PHE	80	-12.364	13.626	-2.719	1.00	0.00
ATOM	1251	HE1	PHE	80	-13.280	14.116	-3.050	1.00	0.00
ATOM	1252	CZ	PHE	80	-11.131	14.280	-2.853	1.00	0.00
ATOM	1253	HZ	PHE	80	-11.076	15.277	-3.289	1.00	0.00
ATOM	1254	CE2	PHE	80	-9.961	13.638	-2.420	1.00	0.00
ATOM	1255	HE2	PHE	80	-9.000	14.142	-2.519	1.00	0.00
ATOM	1256	CD2	PHE	80	-10.025	12.352	-1.861	1.00	0.00
ATOM	1257	HD2	PHE	80	-9.113	11.857	-1.526	1.00	0.00
ATOM	1258	C	PHE	80	-10.139	8.735	-2.635	1.00	0.00
ATOM	1259	O	PHE	80	-9.843	8.701	-3.818	1.00	0.00
ATOM	1260	N	GLN	81	-9.291	8.338	-1.678	1.00	0.00
ATOM	1261	H	GLN	81	-9.597	8.339	-0.716	1.00	0.00
ATOM	1262	CA	GLN	81	-7.940	7.922	-1.966	1.00	0.00
ATOM	1263	HA	GLN	81	-7.482	8.639	-2.647	1.00	0.00
ATOM	1264	CB	GLN	81	-7.094	7.841	-0.702	1.00	0.00
ATOM	1265	HB2	GLN	81	-7.448	7.005	-0.098	1.00	0.00
ATOM	1266	HB3	GLN	81	-6.056	7.668	-0.987	1.00	0.00
ATOM	1267	CG	GLN	81	-7.191	9.128	0.106	1.00	0.00
ATOM	1268	HG2	GLN	81	-6.337	9.755	-0.150	1.00	0.00
ATOM	1269	HG3	GLN	81	-8.114	9.659	-0.126	1.00	0.00

ATOM	1270	CD	GLN	81	-7.156	8.846	1.595	1.00	0.00
ATOM	1271	OE1	GLN	81	-7.480	7.783	2.092	1.00	0.00
ATOM	1272	NE2	GLN	81	-6.774	9.853	2.349	1.00	0.00
ATOM	1273	HE21	GLN	81	-6.726	9.739	3.351	1.00	0.00
ATOM	1274	HE22	GLN	81	-6.530	10.735	1.922	1.00	0.00
ATOM	1275	C	GLN	81	-8.009	6.582	-2.621	1.00	0.00
ATOM	1276	O	GLN	81	-7.557	6.497	-3.731	1.00	0.00
ATOM	1277	N	SER	82	-8.623	5.580	-1.980	1.00	0.00
ATOM	1278	H	SER	82	-9.022	5.767	-1.072	1.00	0.00
ATOM	1279	CA	SER	82	-8.741	4.237	-2.520	1.00	0.00
ATOM	1280	HA	SER	82	-7.810	3.693	-2.360	1.00	0.00
ATOM	1281	CB	SER	82	-9.873	3.479	-1.834	1.00	0.00
ATOM	1282	HB2	SER	82	-9.610	3.366	-0.783	1.00	0.00
ATOM	1283	HB3	SER	82	-10.777	4.081	-1.919	1.00	0.00
ATOM	1284	OG	SER	82	-10.100	2.211	-2.399	1.00	0.00
ATOM	1285	HG	SER	82	-10.819	1.777	-1.934	1.00	0.00
ATOM	1286	C	SER	82	-9.026	4.283	-4.004	1.00	0.00
ATOM	1287	O	SER	82	-8.323	3.659	-4.755	1.00	0.00
ATOM	1288	N	THR	83	-9.963	5.129	-4.420	1.00	0.00
ATOM	1289	H	THR	83	-10.530	5.604	-3.732	1.00	0.00
ATOM	1290	CA	THR	83	-10.285	5.312	-5.818	1.00	0.00
ATOM	1291	HA	THR	83	-10.864	4.445	-6.137	1.00	0.00

ATOM	1292	CB	THR	83	-11.152	6.561	-5.971	1.00	0.00
ATOM	1293	HB	THR	83	-10.977	7.188	-5.097	1.00	0.00
ATOM	1294	CG2	THR	83	-10.855	7.359	-7.237	1.00	0.00
ATOM	1295	HG21	THR	83	-11.030	6.732	-8.112	1.00	0.00
ATOM	1296	HG22	THR	83	-11.508	8.231	-7.279	1.00	0.00
ATOM	1297	HG23	THR	83	-9.815	7.684	-7.226	1.00	0.00
ATOM	1298	OG1	THR	83	-12.467	6.105	-6.095	1.00	0.00
ATOM	1299	HG1	THR	83	-13.058	6.855	-6.195	1.00	0.00
ATOM	1300	C	THR	83	-9.030	5.349	-6.680	1.00	0.00
ATOM	1301	O	THR	83	-8.705	4.388	-7.379	1.00	0.00
ATOM	1302	N	ARG	84	-8.273	6.449	-6.586	1.00	0.00
ATOM	1303	H	ARG	84	-8.553	7.191	-5.962	1.00	0.00
ATOM	1304	CA	ARG	84	-7.076	6.623	-7.355	1.00	0.00
ATOM	1305	HA	ARG	84	-7.273	6.377	-8.399	1.00	0.00
ATOM	1306	CB	ARG	84	-6.584	8.069	-7.278	1.00	0.00
ATOM	1307	HB2	ARG	84	-7.253	8.634	-6.629	1.00	0.00
ATOM	1308	HB3	ARG	84	-5.578	8.077	-6.858	1.00	0.00
ATOM	1309	CG	ARG	84	-6.561	8.710	-8.671	1.00	0.00
ATOM	1310	HG2	ARG	84	-7.590	8.894	-8.979	1.00	0.00
ATOM	1311	HG3	ARG	84	-6.027	9.658	-8.602	1.00	0.00
ATOM	1312	CD	ARG	84	-5.873	7.811	-9.703	1.00	0.00
ATOM	1313	HD2	ARG	84	-5.404	6.968	-9.195	1.00	0.00

ATOM	1314	HD3	ARG	84	-6.613	7.440	-10.413	1.00	0.00
ATOM	1315	NE	ARG	84	-4.838	8.551	-10.444	1.00	0.00
ATOM	1316	HE	ARG	84	-4.506	9.399	-10.007	1.00	0.00
ATOM	1317	CZ	ARG	84	-4.336	8.208	-11.613	1.00	0.00
ATOM	1318	NH1	ARG	84	-4.797	7.155	-12.247	1.00	0.00
ATOM	1319	HH11	ARG	84	-5.539	6.606	-11.836	1.00	0.00
ATOM	1320	HH12	ARG	84	-4.408	6.898	-13.143	1.00	0.00
ATOM	1321	NH2	ARG	84	-3.359	8.907	-12.143	1.00	0.00
ATOM	1322	HH21	ARG	84	-2.992	9.709	-11.652	1.00	0.00
ATOM	1323	HH22	ARG	84	-2.979	8.638	-13.040	1.00	0.00
ATOM	1324	C	ARG	84	-6.005	5.708	-6.836	1.00	0.00
ATOM	1325	O	ARG	84	-5.286	5.113	-7.619	1.00	0.00
ATOM	1326	N	ILE	85	-5.840	5.639	-5.523	1.00	0.00
ATOM	1327	H	ILE	85	-6.375	6.286	-4.962	1.00	0.00
ATOM	1328	CA	ILE	85	-4.892	4.785	-4.861	1.00	0.00
ATOM	1329	HA	ILE	85	-3.860	5.097	-5.021	1.00	0.00
ATOM	1330	CB	ILE	85	-5.174	4.702	-3.355	1.00	0.00
ATOM	1331	HB	ILE	85	-6.252	4.744	-3.198	1.00	0.00
ATOM	1332	CG2	ILE	85	-4.626	3.408	-2.767	1.00	0.00
ATOM	1333	HG21	ILE	85	-3.548	3.365	-2.923	1.00	0.00
ATOM	1334	HG22	ILE	85	-4.839	3.374	-1.699	1.00	0.00
ATOM	1335	HG23	ILE	85	-5.098	2.556	-3.258	1.00	0.00

ATOM 1336 CG1 ILE 85 -4.632 5.944 -2.626 1.00 0.00
ATOM 1337 HG12 ILE 85 -5.173 6.819 -2.985 1.00 0.00
ATOM 1338 HG13 ILE 85 -4.808 5.822 -1.557 1.00 0.00
ATOM 1339 CD1 ILE 85 -3.137 6.131 -2.878 1.00 0.00
ATOM 1340 HD11 ILE 85 -2.961 6.253 -3.947 1.00 0.00
ATOM 1341 HD12 ILE 85 -2.787 7.017 -2.349 1.00 0.00
ATOM 1342 HD13 ILE 85 -2.595 5.256 -2.519 1.00 0.00
ATOM 1343 C ILE 85 -4.886 3.420 -5.520 1.00 0.00
ATOM 1344 O ILE 85 -3.832 2.838 -5.671 1.00 0.00
ATOM 1345 N TYR 86 -6.032 2.963 -5.999 1.00 0.00
ATOM 1346 H TYR 86 -6.847 3.549 -5.889 1.00 0.00
ATOM 1347 CA TYR 86 -6.180 1.719 -6.685 1.00 0.00
ATOM 1348 HA TYR 86 -5.494 0.965 -6.299 1.00 0.00
ATOM 1349 CB TYR 86 -7.606 1.246 -6.446 1.00 0.00
ATOM 1350 HB2 TYR 86 -8.226 2.141 -6.406 1.00 0.00
ATOM 1351 HB3 TYR 86 -7.898 0.639 -7.304 1.00 0.00
ATOM 1352 CG TYR 86 -7.794 0.445 -5.174 1.00 0.00
ATOM 1353 CD1 TYR 86 -7.482 1.004 -3.923 1.00 0.00
ATOM 1354 HD1 TYR 86 -7.105 2.025 -3.877 1.00 0.00
ATOM 1355 CE1 TYR 86 -7.650 0.264 -2.743 1.00 0.00
ATOM 1356 HE1 TYR 86 -7.371 0.681 -1.776 1.00 0.00
ATOM 1357 CZ TYR 86 -8.135 -1.049 -2.827 1.00 0.00

ATOM 1358 OH TYR 86 -8.347 -1.742 -1.683 1.00 0.00

ATOM 1359 HH TYR 86 -8.133 -1.239 -0.894 1.00 0.00

ATOM 1360 CE2 TYR 86 -8.450 -1.628 -4.068 1.00 0.00

ATOM 1361 HE2 TYR 86 -8.828 -2.649 -4.127 1.00 0.00

ATOM 1362 CD2 TYR 86 -8.272 -0.875 -5.244 1.00 0.00

ATOM 1363 HD2 TYR 86 -8.505 -1.316 -6.214 1.00 0.00

ATOM 1364 C TYR 86 -5.973 1.886 -8.166 1.00 0.00

ATOM 1365 O TYR 86 -5.306 1.084 -8.800 1.00 0.00

ATOM 1366 N GLN 87 -6.478 2.969 -8.749 1.00 0.00

ATOM 1367 H GLN 87 -7.038 3.597 -8.190 1.00 0.00

ATOM 1368 CA GLN 87 -6.310 3.266 -10.147 1.00 0.00

ATOM 1369 HA GLN 87 -6.640 2.417 -10.746 1.00 0.00

ATOM 1370 CB GLN 87 -7.134 4.488 -10.538 1.00 0.00

ATOM 1371 HB2 GLN 87 -7.855 4.695 -9.748 1.00 0.00

ATOM 1372 HB3 GLN 87 -6.466 5.342 -10.653 1.00 0.00

ATOM 1373 CG GLN 87 -7.876 4.241 -11.853 1.00 0.00

ATOM 1374 HG2 GLN 87 -7.258 4.302 -12.749 1.00 0.00

ATOM 1375 HG3 GLN 87 -8.251 3.224 -11.740 1.00 0.00

ATOM 1376 CD GLN 87 -9.062 5.182 -12.014 1.00 0.00

ATOM 1377 OE1 GLN 87 -9.112 6.248 -11.411 1.00 0.00

ATOM 1378 NE2 GLN 87 -9.928 4.848 -12.936 1.00 0.00

ATOM 1379 HE21 GLN 87 -10.739 5.428 -13.097 1.00 0.00

```
ATOM   1380 HE22 GLN    87      -9.781   4.011 -13.483  1.00  0.00
ATOM   1381  C   GLN    87      -4.847   3.495 -10.476  1.00  0.00
ATOM   1382  O   GLN    87      -4.472   3.593 -11.638  1.00  0.00
ATOM   1383  N   ILE    88      -3.982   3.604  -9.482  1.00  0.00
ATOM   1384  H   ILE    88      -4.355   3.610  -8.543  1.00  0.00
ATOM   1385  CA  ILE    88      -2.569   3.786  -9.654  1.00  0.00
ATOM   1386  HA  ILE    88      -2.293   3.786 -10.709  1.00  0.00
ATOM   1387  CB  ILE    88      -2.143   5.109  -9.015  1.00  0.00
ATOM   1388  HB  ILE    88      -2.486   5.127  -7.981  1.00  0.00
ATOM   1389  CG2 ILE    88      -0.624   5.261  -9.050  1.00  0.00
ATOM   1390 HG21 ILE    88      -0.280   5.243 -10.084  1.00  0.00
ATOM   1391 HG22 ILE    88      -0.343   6.208  -8.591  1.00  0.00
ATOM   1392 HG23 ILE    88      -0.163   4.440  -8.500  1.00  0.00
ATOM   1393  CG1 ILE    88      -2.809   6.288  -9.729  1.00  0.00
ATOM   1394 HG12 ILE    88      -2.266   6.503 -10.649  1.00  0.00
ATOM   1395 HG13 ILE    88      -3.840   6.026  -9.969  1.00  0.00
ATOM   1396  CD1 ILE    88      -2.794   7.528  -8.824  1.00  0.00
ATOM   1397 HD11 ILE    88      -1.764   7.790  -8.584  1.00  0.00
ATOM   1398 HD12 ILE    88      -3.270   8.361  -9.341  1.00  0.00
ATOM   1399 HD13 ILE    88      -3.338   7.313  -7.904  1.00  0.00
ATOM   1400  C   ILE    88      -1.844   2.594  -9.064  1.00  0.00
ATOM   1401  O   ILE    88      -0.661   2.438  -9.277  1.00  0.00
```

ATOM	1402	N	ALA	89	-2.544	1.733	-8.346	1.00	0.00
ATOM	1403	H	ALA	89	-3.509	1.950	-8.139	1.00	0.00
ATOM	1404	CA	ALA	89	-1.977	0.535	-7.780	1.00	0.00
ATOM	1405	HA	ALA	89	-0.888	0.582	-7.807	1.00	0.00
ATOM	1406	CB	ALA	89	-2.422	0.414	-6.332	1.00	0.00
ATOM	1407	HB1	ALA	89	-3.510	0.356	-6.289	1.00	0.00
ATOM	1408	HB2	ALA	89	-1.994	-0.488	-5.894	1.00	0.00
ATOM	1409	HB3	ALA	89	-2.083	1.286	-5.773	1.00	0.00
ATOM	1410	C	ALA	89	-2.377	-0.673	-8.610	1.00	0.00
ATOM	1411	O	ALA	89	-2.041	-1.807	-8.281	1.00	0.00
ATOM	1412	N	LYS	90	-3.092	-0.433	-9.715	1.00	0.00
ATOM	1413	H	LYS	90	-3.397	0.512	-9.900	1.00	0.00
ATOM	1414	CA	LYS	90	-3.543	-1.486	-10.601	1.00	0.00
ATOM	1415	HA	LYS	90	-3.496	-2.447	-10.089	1.00	0.00
ATOM	1416	CB	LYS	90	-4.993	-1.232	-11.035	1.00	0.00
ATOM	1417	HB2	LYS	90	-5.510	-2.192	-11.051	1.00	0.00
ATOM	1418	HB3	LYS	90	-5.455	-0.583	-10.291	1.00	0.00
ATOM	1419	CG	LYS	90	-5.091	-0.582	-12.405	1.00	0.00
ATOM	1420	HG2	LYS	90	-4.508	-1.173	-13.112	1.00	0.00
ATOM	1421	HG3	LYS	90	-6.137	-0.578	-12.713	1.00	0.00
ATOM	1422	CD	LYS	90	-4.567	0.836	-12.380	1.00	0.00
ATOM	1423	HD2	LYS	90	-5.252	1.454	-11.799	1.00	0.00

```
ATOM   1424  HD3 LYS    90      -3.585   0.839 -11.907  1.00  0.00
ATOM   1425  CE  LYS    90      -4.452   1.397 -13.796  1.00  0.00
ATOM   1426  HE2 LYS    90      -4.027   0.628 -14.442  1.00  0.00
ATOM   1427  HE3 LYS    90      -5.450   1.656 -14.150  1.00  0.00
ATOM   1428  NZ  LYS    90      -3.594   2.594 -13.827  1.00  0.00
ATOM   1429  HZ1 LYS    90      -2.669   2.354 -13.500  1.00  0.00
ATOM   1430  HZ2 LYS    90      -3.536   2.942 -14.773  1.00  0.00
ATOM   1431  HZ3 LYS    90      -3.988   3.306 -13.230  1.00  0.00
ATOM   1432  C   LYS    90      -2.586  -1.606 -11.758  1.00  0.00
ATOM   1433  O   LYS    90      -2.604  -2.580 -12.492  1.00  0.00
ATOM   1434  N   ASN    91      -1.782  -0.557 -11.976  1.00  0.00
ATOM   1435  H   ASN    91      -1.879   0.258 -11.388  1.00  0.00
ATOM   1436  CA  ASN    91      -0.800  -0.528 -13.023  1.00  0.00
ATOM   1437  HA  ASN    91      -1.121  -1.165 -13.848  1.00  0.00
ATOM   1438  CB  ASN    91      -0.617   0.897 -13.548  1.00  0.00
ATOM   1439  HB2 ASN    91       0.261   0.885 -14.193  1.00  0.00
ATOM   1440  HB3 ASN    91      -1.486   1.215 -14.124  1.00  0.00
ATOM   1441  CG  ASN    91      -0.386   1.888 -12.439  1.00  0.00
ATOM   1442  OD1 ASN    91       0.256   1.587 -11.462  1.00  0.00
ATOM   1443  ND2 ASN    91      -0.971   3.061 -12.591  1.00  0.00
ATOM   1444 HD21 ASN    91      -0.858   3.773 -11.884  1.00  0.00
ATOM   1445 HD22 ASN    91      -1.529   3.240 -13.414  1.00  0.00
```

ATOM	1446	C	ASN	91	0.538	-1.051	-12.510	1.00	0.00
ATOM	1447	O	ASN	91	1.534	-1.016	-13.224	1.00	0.00
ATOM	1448	N	LEU	92	0.573	-1.454	-11.244	1.00	0.00
ATOM	1449	H	LEU	92	-0.246	-1.325	-10.667	1.00	0.00
ATOM	1450	CA	LEU	92	1.755	-2.004	-10.636	1.00	0.00
ATOM	1451	HA	LEU	92	2.627	-1.409	-10.906	1.00	0.00
ATOM	1452	CB	LEU	92	1.607	-1.993	-9.108	1.00	0.00
ATOM	1453	HB2	LEU	92	0.530	-1.976	-8.943	1.00	0.00
ATOM	1454	HB3	LEU	92	2.025	-2.906	-8.683	1.00	0.00
ATOM	1455	CG	LEU	92	2.247	-0.775	-8.432	1.00	0.00
ATOM	1456	HG	LEU	92	2.065	0.109	-9.043	1.00	0.00
ATOM	1457	CD1	LEU	92	1.646	-0.565	-7.040	1.00	0.00
ATOM	1458	HD11	LEU	92	1.828	-1.449	-6.428	1.00	0.00
ATOM	1459	HD12	LEU	92	2.109	0.303	-6.571	1.00	0.00
ATOM	1460	HD13	LEU	92	0.572	-0.400	-7.129	1.00	0.00
ATOM	1461	CD2	LEU	92	3.763	-0.919	-8.363	1.00	0.00
ATOM	1462	HD21	LEU	92	4.166	-1.010	-9.372	1.00	0.00
ATOM	1463	HD22	LEU	92	4.190	-0.041	-7.879	1.00	0.00
ATOM	1464	HD23	LEU	92	4.018	-1.810	-7.789	1.00	0.00
ATOM	1465	C	LEU	92	2.011	-3.403	-11.166	1.00	0.00
ATOM	1466	O	LEU	92	3.093	-3.662	-11.651	1.00	0.00
ATOM	1467	N	PRO	93	0.995	-4.280	-11.155	1.00	0.00

ATOM	1468	CD	PRO	93	-0.349	-4.029	-10.662	1.00	0.00
ATOM	1469	HD2	PRO	93	-0.810	-3.299	-11.327	1.00	0.00
ATOM	1470	HD3	PRO	93	-0.257	-3.603	-9.663	1.00	0.00
ATOM	1471	CG	PRO	93	-1.080	-5.354	-10.659	1.00	0.00
ATOM	1472	HG2	PRO	93	-1.920	-5.303	-11.352	1.00	0.00
ATOM	1473	HG3	PRO	93	-1.451	-5.558	-9.655	1.00	0.00
ATOM	1474	CB	PRO	93	-0.062	-6.412	-11.091	1.00	0.00
ATOM	1475	HB2	PRO	93	-0.473	-7.087	-11.842	1.00	0.00
ATOM	1476	HB3	PRO	93	0.276	-6.989	-10.231	1.00	0.00
ATOM	1477	CA	PRO	93	1.107	-5.626	-11.684	1.00	0.00
ATOM	1478	HA	PRO	93	2.024	-6.066	-11.291	1.00	0.00
ATOM	1479	C	PRO	93	1.142	-5.656	-13.202	1.00	0.00
ATOM	1480	O	PRO	93	1.265	-6.723	-13.799	1.00	0.00
ATOM	1481	N	ASN	94	1.035	-4.473	-13.812	1.00	0.00
ATOM	1482	H	ASN	94	0.932	-3.634	-13.259	1.00	0.00
ATOM	1483	CA	ASN	94	1.066	-4.347	-15.239	1.00	0.00
ATOM	1484	HA	ASN	94	0.629	-5.232	-15.702	1.00	0.00
ATOM	1485	CB	ASN	94	0.261	-3.121	-15.673	1.00	0.00
ATOM	1486	HB2	ASN	94	0.491	-2.286	-15.012	1.00	0.00
ATOM	1487	HB3	ASN	94	0.541	-2.863	-16.694	1.00	0.00
ATOM	1488	CG	ASN	94	-1.228	-3.376	-15.630	1.00	0.00
ATOM	1489	OD1	ASN	94	-1.704	-4.496	-15.563	1.00	0.00

ATOM	1490	ND2	ASN	94	-1.985	-2.294	-15.662	1.00	0.00
ATOM	1491	HD21	ASN	94	-2.991	-2.384	-15.637	1.00	0.00
ATOM	1492	HD22	ASN	94	-1.555	-1.382	-15.712	1.00	0.00
ATOM	1493	C	ASN	94	2.495	-4.184	-15.715	1.00	0.00
ATOM	1494	O	ASN	94	2.882	-4.727	-16.742	1.00	0.00
ATOM	1495	N	VAL	95	3.277	-3.411	-14.959	1.00	0.00
ATOM	1496	H	VAL	95	2.881	-2.932	-14.163	1.00	0.00
ATOM	1497	CA	VAL	95	4.670	-3.171	-15.282	1.00	0.00
ATOM	1498	HA	VAL	95	4.931	-3.536	-16.276	1.00	0.00
ATOM	1499	CB	VAL	95	4.959	-1.670	-15.220	1.00	0.00
ATOM	1500	HB	VAL	95	4.824	-1.330	-14.193	1.00	0.00
ATOM	1501	CG1	VAL	95	6.386	-1.368	-15.673	1.00	0.00
ATOM	1502	HG11	VAL	95	6.521	-1.708	-16.700	1.00	0.00
ATOM	1503	HG12	VAL	95	6.565	-0.295	-15.619	1.00	0.00
ATOM	1504	HG13	VAL	95	7.091	-1.887	-15.024	1.00	0.00
ATOM	1505	CG2	VAL	95	3.957	-0.892	-16.085	1.00	0.00
ATOM	1506	HG21	VAL	95	2.945	-1.073	-15.721	1.00	0.00
ATOM	1507	HG22	VAL	95	4.178	0.173	-16.029	1.00	0.00
ATOM	1508	HG23	VAL	95	4.035	-1.225	-17.120	1.00	0.00
ATOM	1509	C	VAL	95	5.567	-3.962	-14.342	1.00	0.00
ATOM	1510	O	VAL	95	6.774	-4.028	-14.524	1.00	0.00
ATOM	1511	N	CYX	96	4.962	-4.621	-13.358	1.00	0.00

ATOM	1512	H	CYX	96	3.965	-4.509	-13.246	1.00	0.00
ATOM	1513	CA	CYX	96	5.671	-5.413	-12.393	1.00	0.00
ATOM	1514	HA	CYX	96	6.710	-5.509	-12.708	1.00	0.00
ATOM	1515	CB	CYX	96	5.639	-4.788	-10.998	1.00	0.00
ATOM	1516	HB2	CYX	96	4.636	-4.880	-10.581	1.00	0.00
ATOM	1517	HB3	CYX	96	6.349	-5.304	-10.352	1.00	0.00
ATOM	1518	SG	CYX	96	6.060	-3.053	-10.963	1.00	0.00
ATOM	1519	C	CYX	96	5.091	-6.789	-12.413	1.00	0.00
ATOM	1520	O	CYX	96	4.965	-7.323	-11.337	1.00	0.00
ATOM	1521	N	ASN	97	4.714	-7.312	-13.591	1.00	0.00
ATOM	1522	H	ASN	97	4.843	-6.746	-14.417	1.00	0.00
ATOM	1523	CA	ASN	97	4.137	-8.630	-13.746	1.00	0.00
ATOM	1524	HA	ASN	97	3.050	-8.555	-13.771	1.00	0.00
ATOM	1525	CB	ASN	97	4.611	-9.278	-15.047	1.00	0.00
ATOM	1526	HB2	ASN	97	5.593	-8.897	-15.327	1.00	0.00
ATOM	1527	HB3	ASN	97	4.673	-10.354	-14.882	1.00	0.00
ATOM	1528	CG	ASN	97	3.655	-9.020	-16.179	1.00	0.00
ATOM	1529	OD1	ASN	97	2.924	-9.890	-16.622	1.00	0.00
ATOM	1530	ND2	ASN	97	3.645	-7.784	-16.628	1.00	0.00
ATOM	1531	HD21	ASN	97	3.029	-7.531	-17.388	1.00	0.00
ATOM	1532	HD22	ASN	97	4.253	-7.094	-16.211	1.00	0.00
ATOM	1533	C	ASN	97	4.533	-9.549	-12.592	1.00	0.00

ATOM 1534 O ASN 97 5.636 -10.096 -12.586 1.00 0.00
ATOM 1535 N MET 98 3.664 -9.628 -11.587 1.00 0.00
ATOM 1536 H MET 98 2.789 -9.126 -11.639 1.00 0.00
ATOM 1537 CA MET 98 3.964 -10.358 -10.376 1.00 0.00
ATOM 1538 HA MET 98 4.812 -11.019 -10.558 1.00 0.00
ATOM 1539 CB MET 98 4.313 -9.423 -9.220 1.00 0.00
ATOM 1540 HB2 MET 98 4.652 -10.023 -8.375 1.00 0.00
ATOM 1541 HB3 MET 98 5.115 -8.757 -9.538 1.00 0.00
ATOM 1542 CG MET 98 3.102 -8.596 -8.802 1.00 0.00
ATOM 1543 HG2 MET 98 2.238 -8.945 -9.367 1.00 0.00
ATOM 1544 HG3 MET 98 2.928 -8.757 -7.738 1.00 0.00
ATOM 1545 SD MET 98 3.335 -6.851 -9.102 1.00 0.00
ATOM 1546 CE MET 98 2.002 -6.156 -8.168 1.00 0.00
ATOM 1547 HE1 MET 98 2.110 -6.433 -7.119 1.00 0.00
ATOM 1548 HE2 MET 98 2.020 -5.070 -8.261 1.00 0.00
ATOM 1549 HE3 MET 98 1.054 -6.537 -8.548 1.00 0.00
ATOM 1550 C MET 98 2.789 -11.239 -10.033 1.00 0.00
ATOM 1551 O MET 98 2.718 -11.807 -8.953 1.00 0.00
ATOM 1552 N LYS 99 1.805 -11.319 -10.937 1.00 0.00
ATOM 1553 H LYS 99 1.852 -10.710 -11.742 1.00 0.00
ATOM 1554 CA LYS 99 0.616 -12.103 -10.745 1.00 0.00
ATOM 1555 HA LYS 99 0.816 -12.929 -10.062 1.00 0.00

ATOM 1556 CB LYS 99 -0.492 -11.226 -10.143 1.00 0.00
ATOM 1557 HB2 LYS 99 -0.422 -11.277 -9.057 1.00 0.00
ATOM 1558 HB3 LYS 99 -0.335 -10.198 -10.469 1.00 0.00
ATOM 1559 CG LYS 99 -1.884 -11.697 -10.588 1.00 0.00
ATOM 1560 HG2 LYS 99 -1.819 -12.731 -10.928 1.00 0.00
ATOM 1561 HG3 LYS 99 -2.568 -11.636 -9.742 1.00 0.00
ATOM 1562 CD LYS 99 -2.407 -10.810 -11.737 1.00 0.00
ATOM 1563 HD2 LYS 99 -3.124 -10.098 -11.329 1.00 0.00
ATOM 1564 HD3 LYS 99 -1.566 -10.270 -12.171 1.00 0.00
ATOM 1565 CE LYS 99 -3.090 -11.659 -12.828 1.00 0.00
ATOM 1566 HE2 LYS 99 -2.927 -12.713 -12.601 1.00 0.00
ATOM 1567 HE3 LYS 99 -4.159 -11.446 -12.816 1.00 0.00
ATOM 1568 NZ LYS 99 -2.542 -11.353 -14.167 1.00 0.00
ATOM 1569 HZ1 LYS 99 -1.552 -11.551 -14.179 1.00 0.00
ATOM 1570 HZ2 LYS 99 -3.007 -11.923 -14.859 1.00 0.00
ATOM 1571 HZ3 LYS 99 -2.693 -10.377 -14.378 1.00 0.00
ATOM 1572 C LYS 99 0.280 -12.794 -12.047 1.00 0.00
ATOM 1573 O LYS 99 -0.409 -12.261 -12.893 1.00 0.00
ATOM 1574 N GLN 100 0.785 -13.986 -12.256 1.00 0.00
ATOM 1575 H GLN 100 1.428 -14.391 -11.591 1.00 0.00
ATOM 1576 CA GLN 100 0.489 -14.727 -13.460 1.00 0.00
ATOM 1577 HA GLN 100 0.557 -14.074 -14.330 1.00 0.00

ATOM 1578 CB GLN 100 1.497 -15.870 -13.625 1.00 0.00
ATOM 1579 HB2 GLN 100 2.406 -15.627 -13.075 1.00 0.00
ATOM 1580 HB3 GLN 100 1.065 -16.788 -13.225 1.00 0.00
ATOM 1581 CG GLN 100 1.835 -16.068 -15.107 1.00 0.00
ATOM 1582 HG2 GLN 100 1.968 -15.076 -15.538 1.00 0.00
ATOM 1583 HG3 GLN 100 2.757 -16.639 -15.218 1.00 0.00
ATOM 1584 CD GLN 100 0.725 -16.784 -15.867 1.00 0.00
ATOM 1585 OE1 GLN 100 -0.335 -17.093 -15.361 1.00 0.00
ATOM 1586 NE2 GLN 100 0.989 -17.102 -17.112 1.00 0.00
ATOM 1587 HE21 GLN 100 0.292 -17.578 -17.667 1.00 0.00
ATOM 1588 HE22 GLN 100 1.888 -16.870 -17.510 1.00 0.00
ATOM 1589 C GLN 100 -0.935 -15.258 -13.422 1.00 0.00
ATOM 1590 O GLN 100 -1.696 -15.047 -14.359 1.00 0.00
ATOM 1591 N ILE 101 -1.286 -15.901 -12.308 1.00 0.00
ATOM 1592 H ILE 101 -0.588 -16.090 -11.603 1.00 0.00
ATOM 1593 CA ILE 101 -2.603 -16.451 -12.128 1.00 0.00
ATOM 1594 HA ILE 101 -2.912 -17.064 -12.975 1.00 0.00
ATOM 1595 CB ILE 101 -2.681 -17.285 -10.836 1.00 0.00
ATOM 1596 HB ILE 101 -2.255 -18.270 -11.026 1.00 0.00
ATOM 1597 CG2 ILE 101 -1.912 -16.608 -9.712 1.00 0.00
ATOM 1598 HG21 ILE 101 -2.338 -15.623 -9.522 1.00 0.00
ATOM 1599 HG22 ILE 101 -1.980 -17.214 -8.809 1.00 0.00

ATOM	1600	HG23	ILE	101	-0.866	-16.502	-10.000	1.00	0.00
ATOM	1601	CG1	ILE	101	-4.136	-17.560	-10.422	1.00	0.00
ATOM	1602	HG12	ILE	101	-4.155	-18.420	-9.752	1.00	0.00
ATOM	1603	HG13	ILE	101	-4.525	-16.686	-9.901	1.00	0.00
ATOM	1604	CD1	ILE	101	-5.005	-17.851	-11.652	1.00	0.00
ATOM	1605	HD11	ILE	101	-4.616	-18.726	-12.173	1.00	0.00
ATOM	1606	HD12	ILE	101	-6.030	-18.043	-11.336	1.00	0.00
ATOM	1607	HD13	ILE	101	-4.987	-16.992	-12.322	1.00	0.00
ATOM	1608	C	ILE	101	-3.634	-15.320	-12.191	1.00	0.00
ATOM	1609	O	ILE	101	-4.158	-14.986	-13.248	1.00	0.00
ATOM	1610	N	GLY	102	-3.933	-14.705	-11.042	1.00	0.00
ATOM	1611	H	GLY	102	-3.440	-14.971	-10.201	1.00	0.00
ATOM	1612	CA	GLY	102	-4.931	-13.676	-10.950	1.00	0.00
ATOM	1613	HA2	GLY	102	-4.464	-12.714	-11.161	1.00	0.00
ATOM	1614	HA3	GLY	102	-5.707	-13.873	-11.690	1.00	0.00
ATOM	1615	C	GLY	102	-5.559	-13.626	-9.584	1.00	0.00
ATOM	1616	O	GLY	102	-6.657	-14.092	-9.349	1.00	0.00
ATOM	1617	N	THR	103	-4.846	-13.034	-8.646	1.00	0.00
ATOM	1618	H	THR	103	-3.930	-12.687	-8.891	1.00	0.00
ATOM	1619	CA	THR	103	-5.279	-12.923	-7.258	1.00	0.00
ATOM	1620	HA	THR	103	-5.837	-13.835	-7.044	1.00	0.00
ATOM	1621	CB	THR	103	-4.039	-12.886	-6.361	1.00	0.00

ATOM 1622 HB THR 103 -4.055 -11.957 -5.792 1.00 0.00
ATOM 1623 CG2 THR 103 -4.002 -14.071 -5.399 1.00 0.00
ATOM 1624 HG21 THR 103 -3.986 -15.001 -5.968 1.00 0.00
ATOM 1625 HG22 THR 103 -3.107 -14.009 -4.780 1.00 0.00
ATOM 1626 HG23 THR 103 -4.886 -14.051 -4.762 1.00 0.00
ATOM 1627 OG1 THR 103 -2.887 -13.063 -7.185 1.00 0.00
ATOM 1628 HG1 THR 103 -2.097 -13.043 -6.640 1.00 0.00
ATOM 1629 C THR 103 -6.158 -11.699 -7.092 1.00 0.00
ATOM 1630 O THR 103 -7.276 -11.796 -6.588 1.00 0.00
ATOM 1631 N CYX 104 -5.670 -10.540 -7.555 1.00 0.00
ATOM 1632 H CYX 104 -4.711 -10.503 -7.870 1.00 0.00
ATOM 1633 CA CYX 104 -6.432 -9.307 -7.526 1.00 0.00
ATOM 1634 HA CYX 104 -7.471 -9.519 -7.274 1.00 0.00
ATOM 1635 CB CYX 104 -5.858 -8.352 -6.475 1.00 0.00
ATOM 1636 HB2 CYX 104 -6.581 -7.564 -6.266 1.00 0.00
ATOM 1637 HB3 CYX 104 -5.648 -8.904 -5.559 1.00 0.00
ATOM 1638 SG CYX 104 -4.283 -7.505 -6.891 1.00 0.00
ATOM 1639 C CYX 104 -6.468 -8.705 -8.923 1.00 0.00
ATOM 1640 O CYX 104 -5.857 -7.678 -9.192 1.00 0.00
ATOM 1641 N PRO 105 -7.134 -9.382 -9.860 1.00 0.00
ATOM 1642 CD PRO 105 -7.867 -10.622 -9.663 1.00 0.00
ATOM 1643 HD2 PRO 105 -8.718 -10.407 -9.016 1.00 0.00

ATOM 1644 HD3 PRO 105 -7.202 -11.328 -9.165 1.00 0.00

ATOM 1645 CG PRO 105 -8.291 -11.101 -11.049 1.00 0.00

ATOM 1646 HG2 PRO 105 -9.380 -11.117 -11.103 1.00 0.00

ATOM 1647 HG3 PRO 105 -7.906 -12.108 -11.212 1.00 0.00

ATOM 1648 CB PRO 105 -7.695 -10.118 -12.059 1.00 0.00

ATOM 1649 HB2 PRO 105 -8.420 -9.808 -12.811 1.00 0.00

ATOM 1650 HB3 PRO 105 -6.829 -10.560 -12.551 1.00 0.00

ATOM 1651 CA PRO 105 -7.263 -8.913 -11.226 1.00 0.00

ATOM 1652 HA PRO 105 -6.271 -8.633 -11.581 1.00 0.00

ATOM 1653 C PRO 105 -8.200 -7.720 -11.345 1.00 0.00

ATOM 1654 O PRO 105 -9.148 -7.723 -12.120 1.00 0.00

ATOM 1655 N PHE 106 -7.951 -6.695 -10.540 1.00 0.00

ATOM 1656 H PHE 106 -7.151 -6.744 -9.926 1.00 0.00

ATOM 1657 CA PHE 106 -8.757 -5.508 -10.514 1.00 0.00

ATOM 1658 HA PHE 106 -9.793 -5.731 -10.770 1.00 0.00

ATOM 1659 CB PHE 106 -8.715 -4.949 -9.096 1.00 0.00

ATOM 1660 HB2 PHE 106 -9.647 -4.446 -8.839 1.00 0.00

ATOM 1661 HB3 PHE 106 -8.547 -5.776 -8.406 1.00 0.00

ATOM 1662 CG PHE 106 -7.600 -3.948 -8.854 1.00 0.00

ATOM 1663 CD1 PHE 106 -6.276 -4.381 -8.691 1.00 0.00

ATOM 1664 HD1 PHE 106 -6.054 -5.448 -8.736 1.00 0.00

ATOM 1665 CE1 PHE 106 -5.247 -3.462 -8.473 1.00 0.00

ATOM 1666 HE1 PHE 106 -4.215 -3.800 -8.374 1.00 0.00
ATOM 1667 CZ PHE 106 -5.548 -2.104 -8.383 1.00 0.00
ATOM 1668 HZ PHE 106 -4.761 -1.389 -8.145 1.00 0.00
ATOM 1669 CE2 PHE 106 -6.857 -1.658 -8.597 1.00 0.00
ATOM 1670 HE2 PHE 106 -7.076 -0.591 -8.580 1.00 0.00
ATOM 1671 CD2 PHE 106 -7.881 -2.576 -8.832 1.00 0.00
ATOM 1672 HD2 PHE 106 -8.900 -2.226 -8.999 1.00 0.00
ATOM 1673 C PHE 106 -8.221 -4.475 -11.458 1.00 0.00
ATOM 1674 O PHE 106 -7.024 -4.508 -11.734 1.00 0.00
ATOM 1675 N ILE 107 -9.082 -3.550 -11.885 1.00 0.00
ATOM 1676 H ILE 107 -10.042 -3.601 -11.576 1.00 0.00
ATOM 1677 CA ILE 107 -8.718 -2.511 -12.831 1.00 0.00
ATOM 1678 HA ILE 107 -8.110 -1.733 -12.370 1.00 0.00
ATOM 1679 CB ILE 107 -7.973 -3.088 -14.055 1.00 0.00
ATOM 1680 HB ILE 107 -8.287 -2.547 -14.948 1.00 0.00
ATOM 1681 CG2 ILE 107 -6.453 -2.946 -13.878 1.00 0.00
ATOM 1682 HG21 ILE 107 -6.139 -3.488 -12.986 1.00 0.00
ATOM 1683 HG22 ILE 107 -5.945 -3.358 -14.750 1.00 0.00
ATOM 1684 HG23 ILE 107 -6.196 -1.892 -13.773 1.00 0.00
ATOM 1685 CG1 ILE 107 -8.373 -4.555 -14.330 1.00 0.00
ATOM 1686 HG12 ILE 107 -7.662 -5.209 -13.824 1.00 0.00
ATOM 1687 HG13 ILE 107 -9.371 -4.723 -13.926 1.00 0.00

ATOM 1688 CD1 ILE 107 -8.371 -4.859 -15.808 1.00 0.00
ATOM 1689 HD11 ILE 107 -7.373 -4.692 -16.213 1.00 0.00
ATOM 1690 HD12 ILE 107 -8.657 -5.899 -15.966 1.00 0.00
ATOM 1691 HD13 ILE 107 -9.082 -4.207 -16.315 1.00 0.00
ATOM 1692 C ILE 107 -9.950 -1.750 -13.269 1.00 0.00
ATOM 1693 O ILE 107 -9.867 -0.537 -13.404 1.00 0.00
ATOM 1694 N ALA 108 -11.065 -2.459 -13.481 1.00 0.00
ATOM 1695 H ALA 108 -11.019 -3.467 -13.430 1.00 0.00
ATOM 1696 CA ALA 108 -12.320 -1.849 -13.856 1.00 0.00
ATOM 1697 HA ALA 108 -12.159 -1.130 -14.659 1.00 0.00
ATOM 1698 CB ALA 108 -13.275 -2.932 -14.355 1.00 0.00
ATOM 1699 HB1 ALA 108 -13.454 -3.655 -13.559 1.00 0.00
ATOM 1700 HB2 ALA 108 -14.220 -2.476 -14.650 1.00 0.00
ATOM 1701 HB3 ALA 108 -12.834 -3.439 -15.213 1.00 0.00
ATOM 1702 C ALA 108 -12.903 -1.067 -12.680 1.00 0.00
ATOM 1703 O ALA 108 -13.942 -1.426 -12.139 1.00 0.00
ATOM 1704 N ILE 109 -12.214 -0.013 -12.272 1.00 0.00
ATOM 1705 H ILE 109 -11.328 0.155 -12.726 1.00 0.00
ATOM 1706 CA ILE 109 -12.576 0.818 -11.147 1.00 0.00
ATOM 1707 HA ILE 109 -13.623 0.697 -10.867 1.00 0.00
ATOM 1708 CB ILE 109 -11.683 0.534 -9.927 1.00 0.00
ATOM 1709 HB ILE 109 -11.549 1.457 -9.364 1.00 0.00

ATOM 1710 CG2 ILE 109 -12.327 -0.528 -9.022 1.00 0.00

ATOM 1711 HG21 ILE 109 -12.461 -1.452 -9.584 1.00 0.00

ATOM 1712 HG22 ILE 109 -11.681 -0.716 -8.165 1.00 0.00

ATOM 1713 HG23 ILE 109 -13.296 -0.171 -8.675 1.00 0.00

ATOM 1714 CG1 ILE 109 -10.258 0.124 -10.326 1.00 0.00

ATOM 1715 HG12 ILE 109 -9.725 -0.210 -9.436 1.00 0.00

ATOM 1716 HG13 ILE 109 -10.315 -0.695 -11.043 1.00 0.00

ATOM 1717 CD1 ILE 109 -9.514 1.296 -10.953 1.00 0.00

ATOM 1718 HD11 ILE 109 -9.456 2.115 -10.237 1.00 0.00

ATOM 1719 HD12 ILE 109 -8.507 0.982 -11.228 1.00 0.00

ATOM 1720 HD13 ILE 109 -10.046 1.630 -11.844 1.00 0.00

ATOM 1721 C ILE 109 -12.580 2.293 -11.578 1.00 0.00

ATOM 1722 O ILE 109 -13.607 2.953 -11.392 1.00 0.00

ATOM 1723 OXT ILE 109 -11.567 2.788 -12.106 1.00 0.00

TER

ATOM 1724 S1 LIG 110 18.164 -15.327 -4.429 1.00 0.00

ATOM 1725 O1 LIG 110 20.188 -19.119 -2.802 1.00 0.00

ATOM 1726 O2 LIG 110 16.769 -14.735 -5.050 1.00 0.00

ATOM 1727 O3 LIG 110 18.640 -14.927 -2.923 1.00 0.00

ATOM 1728 N1 LIG 110 17.793 -17.147 -4.501 1.00 0.00

ATOM 1729 H1 LIG 110 16.786 -17.243 -4.596 1.00 0.00

ATOM 1730 N2 LIG 110 19.826 -17.960 -3.613 1.00 0.00

```
ATOM   1731  N3  LIG   110      22.573 -14.285  -8.362  1.00  0.00
ATOM   1732  H10 LIG   110      22.376 -13.880  -9.262  1.00  0.00
ATOM   1733  H11 LIG   110      23.531 -14.496  -8.142  1.00  0.00
ATOM   1734  C1  LIG   110      19.535 -15.046  -5.637  1.00  0.00
ATOM   1735  C2  LIG   110      19.201 -14.487  -6.869  1.00  0.00
ATOM   1736  H2  LIG   110      18.167 -14.249  -7.094  1.00  0.00
ATOM   1737  C3  LIG   110      20.845 -15.361  -5.278  1.00  0.00
ATOM   1738  H3  LIG   110      21.062 -15.819  -4.321  1.00  0.00
ATOM   1739  C4  LIG   110      18.504 -18.077  -3.766  1.00  0.00
ATOM   1740  C5  LIG   110      21.565 -14.537  -7.460  1.00  0.00
ATOM   1741  C6  LIG   110      20.220 -14.231  -7.781  1.00  0.00
ATOM   1742  H4  LIG   110      19.983 -13.792  -8.744  1.00  0.00
ATOM   1743  C7  LIG   110      21.858 -15.106  -6.196  1.00  0.00
ATOM   1744  H5  LIG   110      22.885 -15.349  -5.943  1.00  0.00
ATOM   1745  C8  LIG   110      17.973 -19.250  -3.126  1.00  0.00
ATOM   1746  H6  LIG   110      16.943 -19.565  -3.103  1.00  0.00
ATOM   1747  C9  LIG   110      19.049 -19.863  -2.553  1.00  0.00
ATOM   1748  C10 LIG   110      19.220 -21.098  -1.743  1.00  0.00
ATOM   1749  H7  LIG   110      18.257 -21.597  -1.602  1.00  0.00
ATOM   1750  H8  LIG   110      19.906 -21.796  -2.235  1.00  0.00
ATOM   1751  H9  LIG   110      19.639 -20.863  -0.759  1.00  0.00
TER
```

ATOM	1752	S1	LIG	111	18.119	-14.532	16.073	1.00	0.00
ATOM	1753	O1	LIG	111	15.184	-12.219	13.398	1.00	0.00
ATOM	1754	O2	LIG	111	18.685	-16.026	16.432	1.00	0.00
ATOM	1755	O3	LIG	111	19.049	-13.461	15.271	1.00	0.00
ATOM	1756	N1	LIG	111	16.655	-14.994	15.024	1.00	0.00
ATOM	1757	H1	LIG	111	16.795	-15.955	14.725	1.00	0.00
ATOM	1758	N2	LIG	111	15.865	-12.820	14.541	1.00	0.00
ATOM	1759	N3	LIG	111	15.960	-12.074	21.102	1.00	0.00
ATOM	1760	H10	LIG	111	15.922	-12.601	21.957	1.00	0.00
ATOM	1761	H11	LIG	111	15.636	-11.122	21.122	1.00	0.00
ATOM	1762	C1	LIG	111	17.419	-13.764	17.601	1.00	0.00
ATOM	1763	C2	LIG	111	17.388	-14.544	18.756	1.00	0.00
ATOM	1764	H2	LIG	111	17.750	-15.565	18.730	1.00	0.00
ATOM	1765	C3	LIG	111	16.983	-12.440	17.570	1.00	0.00
ATOM	1766	H3	LIG	111	16.998	-11.874	16.646	1.00	0.00
ATOM	1767	C4	LIG	111	16.040	-14.093	14.175	1.00	0.00
ATOM	1768	C5	LIG	111	16.443	-12.636	19.942	1.00	0.00
ATOM	1769	C6	LIG	111	16.899	-13.976	19.928	1.00	0.00
ATOM	1770	H4	LIG	111	16.870	-14.564	20.840	1.00	0.00
ATOM	1771	C7	LIG	111	16.492	-11.880	18.745	1.00	0.00
ATOM	1772	H5	LIG	111	16.140	-10.854	18.743	1.00	0.00
ATOM	1773	C8	LIG	111	15.501	-14.375	12.873	1.00	0.00

```
ATOM   1774  H6  LIG   111      15.518 -15.320  12.355  1.00  0.00
ATOM   1775  C9  LIG   111      14.978 -13.193  12.438  1.00  0.00
ATOM   1776  C10 LIG   111      14.284 -12.770  11.193  1.00  0.00
ATOM   1777  H7  LIG   111      14.185 -13.614  10.506  1.00  0.00
ATOM   1778  H8  LIG   111      13.284 -12.382  11.416  1.00  0.00
ATOM   1779  H9  LIG   111      14.841 -11.972  10.689  1.00  0.00
TER
ATOM   1780  S1  LIG   112      -6.616  -7.351  15.094  1.00  0.00
ATOM   1781  O1  LIG   112      -6.079 -11.771  16.233  1.00  0.00
ATOM   1782  O2  LIG   112      -6.677  -6.308  13.833  1.00  0.00
ATOM   1783  O3  LIG   112      -7.964  -7.729  15.926  1.00  0.00
ATOM   1784  N1  LIG   112      -5.978  -8.864  14.221  1.00  0.00
ATOM   1785  H1  LIG   112      -6.141  -8.725  13.227  1.00  0.00
ATOM   1786  N2  LIG   112      -5.894 -10.332  16.071  1.00  0.00
ATOM   1787  N3  LIG   112      -2.289  -5.539  18.833  1.00  0.00
ATOM   1788  H10 LIG   112      -1.698  -4.759  18.599  1.00  0.00
ATOM   1789  H11 LIG   112      -2.137  -5.993  19.717  1.00  0.00
ATOM   1790  C1  LIG   112      -5.268  -6.833  16.247  1.00  0.00
ATOM   1791  C2  LIG   112      -4.479  -5.748  15.866  1.00  0.00
ATOM   1792  H2  LIG   112      -4.657  -5.255  14.918  1.00  0.00
ATOM   1793  C3  LIG   112      -5.102  -7.502  17.459  1.00  0.00
ATOM   1794  H3  LIG   112      -5.717  -8.357  17.708  1.00  0.00
```

```
ATOM   1795  C4  LIG   112      -6.085 -10.131  14.763  1.00  0.00
ATOM   1796  C5  LIG   112      -3.276  -5.967  17.975  1.00  0.00
ATOM   1797  C6  LIG   112      -3.481  -5.315  16.734  1.00  0.00
ATOM   1798  H4  LIG   112      -2.858  -4.471  16.461  1.00  0.00
ATOM   1799  C7  LIG   112      -4.100  -7.066  18.320  1.00  0.00
ATOM   1800  H5  LIG   112      -3.947  -7.575  19.266  1.00  0.00
ATOM   1801  C8  LIG   112      -6.363 -11.347  14.050  1.00  0.00
ATOM   1802  H6  LIG   112      -6.555 -11.450  12.994  1.00  0.00
ATOM   1803  C9  LIG   112      -6.342 -12.330  14.996  1.00  0.00
ATOM   1804  C10 LIG   112      -6.551 -13.802  14.956  1.00  0.00
ATOM   1805  H7  LIG   112      -6.760 -14.129  13.935  1.00  0.00
ATOM   1806  H8  LIG   112      -5.665 -14.333  15.320  1.00  0.00
ATOM   1807  H9  LIG   112      -7.393 -14.091  15.595  1.00  0.00
TER
ATOM   1808  S1  LIG   113      16.910   9.419 -18.652  1.00  0.00
ATOM   1809  O1  LIG   113      14.508  12.764 -20.692  1.00  0.00
ATOM   1810  O2  LIG   113      17.081   8.573 -17.261  1.00  0.00
ATOM   1811  O3  LIG   113      16.273   8.719 -19.978  1.00  0.00
ATOM   1812  N1  LIG   113      15.783  10.774 -18.061  1.00  0.00
ATOM   1813  H1  LIG   113      15.363  10.454 -17.193  1.00  0.00
ATOM   1814  N2  LIG   113      15.575  11.972 -20.087  1.00  0.00
ATOM   1815  N3  LIG   113      22.177  11.976 -19.962  1.00  0.00
```

ATOM 1816 H10 LIG 113 22.955 11.906 -19.329 1.00 0.00
ATOM 1817 H11 LIG 113 22.316 12.477 -20.823 1.00 0.00
ATOM 1818 C1 LIG 113 18.513 10.244 -19.063 1.00 0.00
ATOM 1819 C2 LIG 113 19.554 10.115 -18.145 1.00 0.00
ATOM 1820 H2 LIG 113 19.402 9.563 -17.225 1.00 0.00
ATOM 1821 C3 LIG 113 18.647 10.927 -20.271 1.00 0.00
ATOM 1822 H3 LIG 113 17.807 11.028 -20.946 1.00 0.00
ATOM 1823 C4 LIG 113 15.048 11.557 -18.931 1.00 0.00
ATOM 1824 C5 LIG 113 20.963 11.402 -19.663 1.00 0.00
ATOM 1825 C6 LIG 113 20.782 10.696 -18.448 1.00 0.00
ATOM 1826 H4 LIG 113 21.608 10.605 -17.751 1.00 0.00
ATOM 1827 C7 LIG 113 19.876 11.508 -20.565 1.00 0.00
ATOM 1828 H5 LIG 113 20.003 12.051 -21.496 1.00 0.00
ATOM 1829 C8 LIG 113 13.710 12.041 -18.725 1.00 0.00
ATOM 1830 H6 LIG 113 13.073 11.845 -17.878 1.00 0.00
ATOM 1831 C9 LIG 113 13.424 12.785 -19.833 1.00 0.00
ATOM 1832 C10 LIG 113 12.233 13.557 -20.275 1.00 0.00
ATOM 1833 H7 LIG 113 11.440 13.495 -19.526 1.00 0.00
ATOM 1834 H8 LIG 113 12.486 14.611 -20.431 1.00 0.00
ATOM 1835 H9 LIG 113 11.849 13.167 -21.224 1.00 0.00
TER
ATOM 1836 S1 LIG 114 -22.208 16.359 6.968 1.00 0.00

ATOM 1837 O1 LIG 114 -22.398 16.087 11.552 1.00 0.00
ATOM 1838 O2 LIG 114 -21.610 15.641 5.624 1.00 0.00
ATOM 1839 O3 LIG 114 -23.800 16.286 7.311 1.00 0.00
ATOM 1840 N1 LIG 114 -21.249 15.443 8.269 1.00 0.00
ATOM 1841 H1 LIG 114 -20.889 14.598 7.834 1.00 0.00
ATOM 1842 N2 LIG 114 -22.130 16.515 10.182 1.00 0.00
ATOM 1843 N3 LIG 114 -20.304 22.047 7.073 1.00 0.00
ATOM 1844 H10 LIG 114 -19.661 22.388 6.380 1.00 0.00
ATOM 1845 H11 LIG 114 -20.624 22.694 7.774 1.00 0.00
ATOM 1846 C1 LIG 114 -21.611 18.107 7.041 1.00 0.00
ATOM 1847 C2 LIG 114 -20.716 18.521 6.055 1.00 0.00
ATOM 1848 H2 LIG 114 -20.386 17.823 5.294 1.00 0.00
ATOM 1849 C3 LIG 114 -22.087 18.958 8.037 1.00 0.00
ATOM 1850 H3 LIG 114 -22.760 18.591 8.803 1.00 0.00
ATOM 1851 C4 LIG 114 -21.636 15.420 9.596 1.00 0.00
ATOM 1852 C5 LIG 114 -20.736 20.741 7.062 1.00 0.00
ATOM 1853 C6 LIG 114 -20.280 19.842 6.067 1.00 0.00
ATOM 1854 H4 LIG 114 -19.588 20.188 5.306 1.00 0.00
ATOM 1855 C7 LIG 114 -21.643 20.276 8.045 1.00 0.00
ATOM 1856 H5 LIG 114 -21.994 20.955 8.815 1.00 0.00
ATOM 1857 C8 LIG 114 -21.543 14.303 10.496 1.00 0.00
ATOM 1858 H6 LIG 114 -21.182 13.314 10.266 1.00 0.00

ATOM 1859 C9 LIG 114 -22.016 14.765 11.690 1.00 0.00

ATOM 1860 C10 LIG 114 -22.206 14.149 13.029 1.00 0.00

ATOM 1861 H7 LIG 114 -21.881 13.106 13.019 1.00 0.00

ATOM 1862 H8 LIG 114 -21.631 14.687 13.792 1.00 0.00

ATOM 1863 H9 LIG 114 -23.259 14.184 13.327 1.00 0.00

TER

ATOM 1864 S1 LIG 115 -23.262 -3.831 16.484 1.00 0.00

ATOM 1865 O1 LIG 115 -22.882 0.422 18.184 1.00 0.00

ATOM 1866 O2 LIG 115 -23.225 -5.420 16.876 1.00 0.00

ATOM 1867 O3 LIG 115 -24.666 -3.104 16.092 1.00 0.00

ATOM 1868 N1 LIG 115 -22.602 -3.101 18.061 1.00 0.00

ATOM 1869 H1 LIG 115 -22.700 -3.812 18.782 1.00 0.00

ATOM 1870 N2 LIG 115 -22.660 -0.820 17.449 1.00 0.00

ATOM 1871 N3 LIG 115 -19.138 -2.838 12.240 1.00 0.00

ATOM 1872 H10 LIG 115 -18.519 -3.572 11.942 1.00 0.00

ATOM 1873 H11 LIG 115 -19.050 -1.940 11.797 1.00 0.00

ATOM 1874 C1 LIG 115 -21.977 -3.491 15.198 1.00 0.00

ATOM 1875 C2 LIG 115 -21.145 -4.547 14.828 1.00 0.00

ATOM 1876 H2 LIG 115 -21.255 -5.516 15.301 1.00 0.00

ATOM 1877 C3 LIG 115 -21.899 -2.226 14.619 1.00 0.00

ATOM 1878 H3 LIG 115 -22.546 -1.423 14.953 1.00 0.00

ATOM 1879 C4 LIG 115 -22.766 -1.768 18.385 1.00 0.00

```
ATOM   1880  C5  LIG   115     -20.079  -3.055  13.222  1.00  0.00
ATOM   1881  C6  LIG   115     -20.195  -4.326  13.836  1.00  0.00
ATOM   1882  H4  LIG   115     -19.540  -5.134  13.528  1.00  0.00
ATOM   1883  C7  LIG   115     -20.944  -2.012  13.631  1.00  0.00
ATOM   1884  H5  LIG   115     -20.859  -1.033  13.170  1.00  0.00
ATOM   1885  C8  LIG   115     -23.024  -1.230  19.693  1.00  0.00
ATOM   1886  H6  LIG   115     -23.152  -1.785  20.608  1.00  0.00
ATOM   1887  C9  LIG   115     -23.079   0.122  19.520  1.00  0.00
ATOM   1888  C10 LIG   115     -23.315   1.269  20.436  1.00  0.00
ATOM   1889  H7  LIG   115     -23.467   0.914  21.458  1.00  0.00
ATOM   1890  H8  LIG   115     -22.463   1.957  20.428  1.00  0.00
ATOM   1891  H9  LIG   115     -24.199   1.837  20.128  1.00  0.00
TER
ATOM   1892  S1  LIG   116     -15.105 -23.794 -18.853  1.00  0.00
ATOM   1893  O1  LIG   116     -12.427 -22.127 -15.510  1.00  0.00
ATOM   1894  O2  LIG   116     -15.531 -23.609 -20.423  1.00  0.00
ATOM   1895  O3  LIG   116     -14.195 -25.062 -18.382  1.00  0.00
ATOM   1896  N1  LIG   116     -14.155 -22.217 -18.595  1.00  0.00
ATOM   1897  H1  LIG   116     -13.911 -21.863 -19.516  1.00  0.00
ATOM   1898  N2  LIG   116     -13.573 -22.520 -16.325  1.00  0.00
ATOM   1899  N3  LIG   116     -20.063 -23.417 -15.496  1.00  0.00
ATOM   1900  H10 LIG   116     -20.951 -23.193 -15.911  1.00  0.00
```

```
ATOM   1901  H11 LIG   116     -20.038 -23.585 -14.505  1.00  0.00
ATOM   1902  C1  LIG   116     -16.612 -23.651 -17.792  1.00  0.00
ATOM   1903  C2  LIG   116     -17.814 -23.335 -18.425  1.00  0.00
ATOM   1904  H2  LIG   116     -17.836 -23.167 -19.495  1.00  0.00
ATOM   1905  C3  LIG   116     -16.518 -23.894 -16.423  1.00  0.00
ATOM   1906  H3  LIG   116     -15.561 -24.110 -15.964  1.00  0.00
ATOM   1907  C4  LIG   116     -13.280 -22.044 -17.539  1.00  0.00
ATOM   1908  C5  LIG   116     -18.919 -23.493 -16.258  1.00  0.00
ATOM   1909  C6  LIG   116     -18.969 -23.257 -17.654  1.00  0.00
ATOM   1910  H4  LIG   116     -19.917 -23.017 -18.125  1.00  0.00
ATOM   1911  C7  LIG   116     -17.677 -23.810 -15.658  1.00  0.00
ATOM   1912  H5  LIG   116     -17.627 -23.988 -14.589  1.00  0.00
ATOM   1913  C8  LIG   116     -12.024 -21.347 -17.573  1.00  0.00
ATOM   1914  H6  LIG   116     -11.567 -20.878 -18.429  1.00  0.00
ATOM   1915  C9  LIG   116     -11.540 -21.418 -16.300  1.00  0.00
ATOM   1916  C10 LIG   116     -10.304 -20.922 -15.639  1.00  0.00
ATOM   1917  H7  LIG   116      -9.677 -20.387 -16.356  1.00  0.00
ATOM   1918  H8  LIG   116     -10.548 -20.243 -14.813  1.00  0.00
ATOM   1919  H9  LIG   116      -9.726 -21.753 -15.221  1.00  0.00
TER
ATOM   1920  S1  LIG   117      18.930  -1.436  -6.986  1.00  0.00
ATOM   1921  O1  LIG   117      23.213   0.123  -6.397  1.00  0.00
```

ATOM	1922	O2	LIG	117	17.356	-1.142	-6.642	1.00	0.00
ATOM	1923	O3	LIG	117	19.503	-1.226	-8.497	1.00	0.00
ATOM	1924	N1	LIG	117	19.736	-0.216	-5.839	1.00	0.00
ATOM	1925	H1	LIG	117	19.026	0.462	-5.578	1.00	0.00
ATOM	1926	N2	LIG	117	21.994	-0.681	-6.358	1.00	0.00
ATOM	1927	N3	LIG	117	20.312	-6.913	-4.967	1.00	0.00
ATOM	1928	H10	LIG	117	19.644	-7.453	-4.444	1.00	0.00
ATOM	1929	H11	LIG	117	21.211	-7.326	-5.148	1.00	0.00
ATOM	1930	C1	LIG	117	19.391	-3.108	-6.346	1.00	0.00
ATOM	1931	C2	LIG	117	18.425	-3.808	-5.625	1.00	0.00
ATOM	1932	H2	LIG	117	17.453	-3.361	-5.445	1.00	0.00
ATOM	1933	C3	LIG	117	20.653	-3.629	-6.626	1.00	0.00
ATOM	1934	H3	LIG	117	21.386	-3.044	-7.167	1.00	0.00
ATOM	1935	C4	LIG	117	21.048	0.190	-5.995	1.00	0.00
ATOM	1936	C5	LIG	117	20.006	-5.651	-5.423	1.00	0.00
ATOM	1937	C6	LIG	117	18.735	-5.083	-5.164	1.00	0.00
ATOM	1938	H4	LIG	117	17.997	-5.648	-4.604	1.00	0.00
ATOM	1939	C7	LIG	117	20.957	-4.904	-6.159	1.00	0.00
ATOM	1940	H5	LIG	117	21.935	-5.328	-6.358	1.00	0.00
ATOM	1941	C8	LIG	117	21.566	1.513	-5.774	1.00	0.00
ATOM	1942	H6	LIG	117	21.009	2.387	-5.480	1.00	0.00
ATOM	1943	C9	LIG	117	22.904	1.420	-6.027	1.00	0.00

ATOM 1944 C10 LIG 117 24.027 2.393 -5.998 1.00 0.00

ATOM 1945 H7 LIG 117 23.667 3.382 -5.703 1.00 0.00

ATOM 1946 H8 LIG 117 24.801 2.077 -5.290 1.00 0.00

ATOM 1947 H9 LIG 117 24.498 2.473 -6.984 1.00 0.00

TER

ATOM 1948 S1 LIG 118 -4.296 14.715 15.888 1.00 0.00

ATOM 1949 O1 LIG 118 -2.133 18.713 15.209 1.00 0.00

ATOM 1950 O2 LIG 118 -4.174 13.094 15.686 1.00 0.00

ATOM 1951 O3 LIG 118 -4.182 15.378 17.371 1.00 0.00

ATOM 1952 N1 LIG 118 -2.861 15.271 14.845 1.00 0.00

ATOM 1953 H1 LIG 118 -2.257 14.464 14.715 1.00 0.00

ATOM 1954 N2 LIG 118 -3.081 17.603 15.153 1.00 0.00

ATOM 1955 N3 LIG 118 -9.286 16.589 13.135 1.00 0.00

ATOM 1956 H10 LIG 118 -9.848 15.952 12.597 1.00 0.00

ATOM 1957 H11 LIG 118 -9.596 17.543 13.206 1.00 0.00

ATOM 1958 C1 LIG 118 -5.811 15.323 15.022 1.00 0.00

ATOM 1959 C2 LIG 118 -6.547 14.395 14.286 1.00 0.00

ATOM 1960 H2 LIG 118 -6.214 13.365 14.226 1.00 0.00

ATOM 1961 C3 LIG 118 -6.191 16.658 15.153 1.00 0.00

ATOM 1962 H3 LIG 118 -5.578 17.356 15.711 1.00 0.00

ATOM 1963 C4 LIG 118 -2.306 16.531 14.962 1.00 0.00

ATOM 1964 C5 LIG 118 -8.134 16.168 13.760 1.00 0.00

ATOM 1965 C6 LIG 118 -7.711 14.820 13.656 1.00 0.00

ATOM 1966 H4 LIG 118 -8.302 14.114 13.082 1.00 0.00

ATOM 1967 C7 LIG 118 -7.355 17.077 14.516 1.00 0.00

ATOM 1968 H5 LIG 118 -7.667 18.113 14.597 1.00 0.00

ATOM 1969 C8 LIG 118 -0.911 16.864 14.868 1.00 0.00

ATOM 1970 H6 LIG 118 -0.090 16.182 14.719 1.00 0.00

ATOM 1971 C9 LIG 118 -0.856 18.219 15.019 1.00 0.00

ATOM 1972 C10 LIG 118 0.254 19.209 15.033 1.00 0.00

ATOM 1973 H7 LIG 118 1.213 18.708 14.884 1.00 0.00

ATOM 1974 H8 LIG 118 0.122 19.956 14.242 1.00 0.00

ATOM 1975 H9 LIG 118 0.284 19.744 15.989 1.00 0.00

TER

ATOM 1976 S1 LIG 119 12.456 21.961 2.985 1.00 0.00

ATOM 1977 O1 LIG 119 11.838 23.395 7.308 1.00 0.00

ATOM 1978 O2 LIG 119 11.738 21.188 1.733 1.00 0.00

ATOM 1979 O3 LIG 119 12.721 23.568 2.929 1.00 0.00

ATOM 1980 N1 LIG 119 11.234 21.579 4.333 1.00 0.00

ATOM 1981 H1 LIG 119 10.369 21.296 3.880 1.00 0.00

ATOM 1982 N2 LIG 119 12.310 22.698 6.115 1.00 0.00

ATOM 1983 N3 LIG 119 17.523 19.051 4.344 1.00 0.00

ATOM 1984 H10 LIG 119 17.765 18.194 3.878 1.00 0.00

ATOM 1985 H11 LIG 119 18.162 19.407 5.034 1.00 0.00

ATOM	1986	C1	LIG	119	14.006	21.066	3.445	1.00	0.00
ATOM	1987	C2	LIG	119	14.287	19.881	2.767	1.00	0.00
ATOM	1988	H2	LIG	119	13.601	19.510	2.015	1.00	0.00
ATOM	1989	C3	LIG	119	14.853	21.606	4.411	1.00	0.00
ATOM	1990	H3	LIG	119	14.587	22.515	4.936	1.00	0.00
ATOM	1991	C4	LIG	119	11.186	22.297	5.513	1.00	0.00
ATOM	1992	C5	LIG	119	16.356	19.718	4.046	1.00	0.00
ATOM	1993	C6	LIG	119	15.467	19.207	3.069	1.00	0.00
ATOM	1994	H4	LIG	119	15.709	18.285	2.551	1.00	0.00
ATOM	1995	C7	LIG	119	16.029	20.925	4.711	1.00	0.00
ATOM	1996	H5	LIG	119	16.701	21.321	5.464	1.00	0.00
ATOM	1997	C8	LIG	119	10.004	22.668	6.242	1.00	0.00
ATOM	1998	H6	LIG	119	8.982	22.466	5.966	1.00	0.00
ATOM	1999	C9	LIG	119	10.456	23.335	7.343	1.00	0.00
ATOM	2000	C10	LIG	119	9.787	23.988	8.499	1.00	0.00
ATOM	2001	H7	LIG	119	8.702	23.888	8.417	1.00	0.00
ATOM	2002	H8	LIG	119	10.110	23.537	9.444	1.00	0.00
ATOM	2003	H9	LIG	119	10.037	25.054	8.541	1.00	0.00

TER

END

Table M: The number of atoms present in the system of HA-Dic

```
ATOM      1  N   PRO     1       5.137  -6.696  13.483  1.00  0.00
ATOM      2  H2  PRO     1       5.624  -5.814  13.557  1.00  0.00
ATOM      3  H3  PRO     1       5.589  -7.548  13.781  1.00  0.00
ATOM      4  CD  PRO     1       3.871  -6.651  14.277  1.00  0.00
ATOM      5  HD2 PRO     1       3.805  -5.635  14.668  1.00  0.00
ATOM      6  HD3 PRO     1       4.016  -7.348  15.101  1.00  0.00
ATOM      7  CG  PRO     1       2.667  -7.024  13.399  1.00  0.00
ATOM      8  HG2 PRO     1       1.965  -6.190  13.374  1.00  0.00
ATOM      9  HG3 PRO     1       2.173  -7.901  13.816  1.00  0.00
ATOM     10  CB  PRO     1       3.227  -7.322  12.012  1.00  0.00
ATOM     11  HB2 PRO     1       2.676  -6.854  11.196  1.00  0.00
ATOM     12  HB3 PRO     1       3.247  -8.401  11.864  1.00  0.00
ATOM     13  CA  PRO     1       4.666  -6.770  12.056  1.00  0.00
ATOM     14  HA  PRO     1       5.259  -7.530  11.548  1.00  0.00
ATOM     15  C   PRO     1       4.806  -5.429  11.311  1.00  0.00
ATOM     16  O   PRO     1       3.834  -4.824  10.860  1.00  0.00
ATOM     17  N   TYR     2       6.050  -4.994  11.172  1.00  0.00
ATOM     18  H   TYR     2       6.812  -5.544  11.541  1.00  0.00
ATOM     19  CA  TYR     2       6.364  -3.742  10.506  1.00  0.00
ATOM     20  HA  TYR     2       5.488  -3.097  10.443  1.00  0.00
ATOM     21  CB  TYR     2       7.435  -3.033  11.338  1.00  0.00
```

ATOM 22 HB2 TYR 2 7.768 -2.139 10.809 1.00 0.00

ATOM 23 HB3 TYR 2 6.941 -2.743 12.265 1.00 0.00

ATOM 24 CG TYR 2 8.638 -3.911 11.657 1.00 0.00

ATOM 25 CD1 TYR 2 8.625 -4.737 12.801 1.00 0.00

ATOM 26 HD1 TYR 2 7.759 -4.734 13.462 1.00 0.00

ATOM 27 CE1 TYR 2 9.728 -5.564 13.089 1.00 0.00

ATOM 28 HE1 TYR 2 9.744 -6.154 14.005 1.00 0.00

ATOM 29 CZ TYR 2 10.851 -5.552 12.239 1.00 0.00

ATOM 30 OH TYR 2 11.908 -6.372 12.473 1.00 0.00

ATOM 31 HH TYR 2 11.789 -6.924 13.248 1.00 0.00

ATOM 32 CE2 TYR 2 10.843 -4.756 11.084 1.00 0.00

ATOM 33 HE2 TYR 2 11.704 -4.777 10.415 1.00 0.00

ATOM 34 CD2 TYR 2 9.741 -3.936 10.785 1.00 0.00

ATOM 35 HD2 TYR 2 9.745 -3.325 9.882 1.00 0.00

ATOM 36 C TYR 2 6.883 -3.984 9.097 1.00 0.00

ATOM 37 O TYR 2 6.674 -3.135 8.248 1.00 0.00

ATOM 38 N GLY 3 7.470 -5.167 8.862 1.00 0.00

ATOM 39 H GLY 3 7.510 -5.840 9.613 1.00 0.00

ATOM 40 CA GLY 3 8.048 -5.527 7.588 1.00 0.00

ATOM 41 HA2 GLY 3 8.387 -6.562 7.629 1.00 0.00

ATOM 42 HA3 GLY 3 7.291 -5.423 6.810 1.00 0.00

ATOM 43 C GLY 3 9.234 -4.630 7.249 1.00 0.00

ATOM	44	O	GLY	3	9.183	-3.405	7.253	1.00	0.00
ATOM	45	N	ARG	4	10.374	-5.258	6.999	1.00	0.00
ATOM	46	H	ARG	4	10.446	-6.253	7.151	1.00	0.00
ATOM	47	CA	ARG	4	11.573	-4.532	6.592	1.00	0.00
ATOM	48	HA	ARG	4	11.402	-3.456	6.646	1.00	0.00
ATOM	49	CB	ARG	4	12.729	-4.892	7.531	1.00	0.00
ATOM	50	HB2	ARG	4	12.340	-4.993	8.544	1.00	0.00
ATOM	51	HB3	ARG	4	13.160	-5.840	7.210	1.00	0.00
ATOM	52	CG	ARG	4	13.813	-3.804	7.506	1.00	0.00
ATOM	53	HG2	ARG	4	13.653	-3.166	6.636	1.00	0.00
ATOM	54	HG3	ARG	4	13.733	-3.206	8.413	1.00	0.00
ATOM	55	CD	ARG	4	15.207	-4.430	7.429	1.00	0.00
ATOM	56	HD2	ARG	4	15.364	-5.074	8.293	1.00	0.00
ATOM	57	HD3	ARG	4	15.290	-5.020	6.516	1.00	0.00
ATOM	58	NE	ARG	4	16.251	-3.395	7.417	1.00	0.00
ATOM	59	HE	ARG	4	15.934	-2.447	7.271	1.00	0.00
ATOM	60	CZ	ARG	4	17.544	-3.610	7.565	1.00	0.00
ATOM	61	NH1	ARG	4	18.000	-4.821	7.757	1.00	0.00
ATOM	62	HH11	ARG	4	17.358	-5.599	7.792	1.00	0.00
ATOM	63	HH12	ARG	4	18.993	-4.971	7.868	1.00	0.00
ATOM	64	NH2	ARG	4	18.392	-2.618	7.537	1.00	0.00
ATOM	65	HH21	ARG	4	18.057	-1.675	7.400	1.00	0.00

ATOM 66 HH22 ARG 4 19.379 -2.798 7.651 1.00 0.00
ATOM 67 C ARG 4 11.929 -4.797 5.131 1.00 0.00
ATOM 68 O ARG 4 13.013 -4.428 4.700 1.00 0.00
ATOM 69 N GLY 5 11.088 -5.573 4.444 1.00 0.00
ATOM 70 H GLY 5 10.248 -5.911 4.891 1.00 0.00
ATOM 71 CA GLY 5 11.333 -5.951 3.074 1.00 0.00
ATOM 72 HA2 GLY 5 10.507 -6.564 2.713 1.00 0.00
ATOM 73 HA3 GLY 5 11.414 -5.054 2.459 1.00 0.00
ATOM 74 C GLY 5 12.619 -6.742 2.963 1.00 0.00
ATOM 75 O GLY 5 13.363 -6.558 2.015 1.00 0.00
ATOM 76 N ARG 6 12.905 -7.585 3.963 1.00 0.00
ATOM 77 H ARG 6 12.293 -7.599 4.766 1.00 0.00
ATOM 78 CA ARG 6 14.097 -8.425 3.986 1.00 0.00
ATOM 79 HA ARG 6 14.958 -7.849 3.647 1.00 0.00
ATOM 80 CB ARG 6 14.375 -8.933 5.403 1.00 0.00
ATOM 81 HB2 ARG 6 15.139 -9.708 5.350 1.00 0.00
ATOM 82 HB3 ARG 6 14.743 -8.101 6.004 1.00 0.00
ATOM 83 CG ARG 6 13.101 -9.511 6.050 1.00 0.00
ATOM 84 HG2 ARG 6 12.651 -8.744 6.681 1.00 0.00
ATOM 85 HG3 ARG 6 12.402 -9.786 5.260 1.00 0.00
ATOM 86 CD ARG 6 13.425 -10.742 6.896 1.00 0.00
ATOM 87 HD2 ARG 6 14.503 -10.806 7.045 1.00 0.00

```
ATOM     88  HD3 ARG     6      12.929 -10.658   7.863  1.00  0.00
ATOM     89  NE  ARG     6      12.966 -11.975   6.236  1.00  0.00
ATOM     90  HE  ARG     6      12.714 -11.890   5.261  1.00  0.00
ATOM     91  CZ  ARG     6      12.884 -13.162   6.807  1.00  0.00
ATOM     92  NH1 ARG     6      13.199 -13.317   8.069  1.00  0.00
ATOM     93 HH11 ARG     6      13.507 -12.522   8.610  1.00  0.00
ATOM     94 HH12 ARG     6      13.132 -14.230   8.494  1.00  0.00
ATOM     95  NH2 ARG     6      12.465 -14.194   6.129  1.00  0.00
ATOM     96 HH21 ARG     6      12.201 -14.084   5.160  1.00  0.00
ATOM     97 HH22 ARG     6      12.406 -15.097   6.575  1.00  0.00
ATOM     98  C   ARG     6      13.996  -9.592   3.017  1.00  0.00
ATOM     99  O   ARG     6      14.140 -10.734   3.451  1.00  0.00
ATOM    100  N   THR     7      13.658  -9.324   1.772  1.00  0.00
ATOM    101  H   THR     7      13.525  -8.355   1.518  1.00  0.00
ATOM    102  CA  THR     7      13.403 -10.338   0.770  1.00  0.00
ATOM    103  HA  THR     7      14.067 -11.181   0.962  1.00  0.00
ATOM    104  CB  THR     7      11.944 -10.820   0.897  1.00  0.00
ATOM    105  HB  THR     7      11.677 -11.361  -0.011  1.00  0.00
ATOM    106  CG2 THR     7      11.763 -11.740   2.113  1.00  0.00
ATOM    107 HG21 THR     7      12.029 -11.199   3.021  1.00  0.00
ATOM    108 HG22 THR     7      10.724 -12.062   2.173  1.00  0.00
ATOM    109 HG23 THR     7      12.408 -12.612   2.008  1.00  0.00
```

ATOM	110	OG1	THR	7	11.071	-9.721	1.112	1.00	0.00
ATOM	111	HG1	THR	7	10.169	-10.039	1.188	1.00	0.00
ATOM	112	C	THR	7	13.702	-9.782	-0.607	1.00	0.00
ATOM	113	O	THR	7	14.168	-8.654	-0.726	1.00	0.00
ATOM	114	N	GLU	8	13.459	-10.586	-1.632	1.00	0.00
ATOM	115	H	GLU	8	13.163	-11.535	-1.459	1.00	0.00
ATOM	116	CA	GLU	8	13.681	-10.173	-3.011	1.00	0.00
ATOM	117	HA	GLU	8	14.267	-9.254	-3.041	1.00	0.00
ATOM	118	CB	GLU	8	14.452	-11.270	-3.756	1.00	0.00
ATOM	119	HB2	GLU	8	13.997	-12.235	-3.534	1.00	0.00
ATOM	120	HB3	GLU	8	14.392	-11.079	-4.828	1.00	0.00
ATOM	121	CG	GLU	8	15.921	-11.286	-3.322	1.00	0.00
ATOM	122	HG2	GLU	8	16.049	-10.738	-2.389	1.00	0.00
ATOM	123	HG3	GLU	8	16.196	-12.330	-3.171	1.00	0.00
ATOM	124	CD	GLU	8	16.818	-10.674	-4.399	1.00	0.00
ATOM	125	OE1	GLU	8	17.124	-11.405	-5.368	1.00	0.00
ATOM	126	OE2	GLU	8	17.150	-9.476	-4.263	1.00	0.00
ATOM	127	C	GLU	8	12.359	-9.844	-3.703	1.00	0.00
ATOM	128	O	GLU	8	11.280	-9.842	-3.091	1.00	0.00
ATOM	129	N	SER	9	12.449	-9.526	-4.990	1.00	0.00
ATOM	130	H	SER	9	13.364	-9.411	-5.402	1.00	0.00
ATOM	131	CA	SER	9	11.286	-9.257	-5.814	1.00	0.00

ATOM	132	HA	SER	9	10.664	-8.506	-5.327	1.00	0.00
ATOM	133	CB	SER	9	11.690	-8.734	-7.201	1.00	0.00
ATOM	134	HB2	SER	9	11.079	-9.245	-7.945	1.00	0.00
ATOM	135	HB3	SER	9	11.481	-7.664	-7.232	1.00	0.00
ATOM	136	OG	SER	9	13.057	-8.954	-7.484	1.00	0.00
ATOM	137	HG	SER	9	13.262	-8.612	-8.358	1.00	0.00
ATOM	138	C	SER	9	10.392	-10.495	-5.953	1.00	0.00
ATOM	139	O	SER	9	10.744	-11.614	-5.584	1.00	0.00
ATOM	140	N	GLY	10	9.198	-10.302	-6.510	1.00	0.00
ATOM	141	H	GLY	10	8.910	-9.372	-6.782	1.00	0.00
ATOM	142	CA	GLY	10	8.276	-11.408	-6.745	1.00	0.00
ATOM	143	HA2	GLY	10	8.409	-11.772	-7.764	1.00	0.00
ATOM	144	HA3	GLY	10	8.496	-12.210	-6.041	1.00	0.00
ATOM	145	C	GLY	10	6.848	-10.975	-6.563	1.00	0.00
ATOM	146	O	GLY	10	6.258	-10.409	-7.473	1.00	0.00
ATOM	147	N	CYX	11	6.343	-11.078	-5.333	1.00	0.00
ATOM	148	H	CYX	11	6.870	-11.544	-4.608	1.00	0.00
ATOM	149	CA	CYX	11	5.018	-10.605	-5.019	1.00	0.00
ATOM	150	HA	CYX	11	4.300	-11.014	-5.730	1.00	0.00
ATOM	151	CB	CYX	11	4.620	-11.042	-3.619	1.00	0.00
ATOM	152	HB2	CYX	11	5.238	-10.522	-2.887	1.00	0.00
ATOM	153	HB3	CYX	11	3.571	-10.800	-3.447	1.00	0.00

ATOM	154	SG	CYX	11	4.736	-12.800	-3.266	1.00	0.00
ATOM	155	C	CYX	11	4.918	-9.081	-5.164	1.00	0.00
ATOM	156	O	CYX	11	3.831	-8.520	-5.134	1.00	0.00
ATOM	157	N	TYR	12	6.061	-8.414	-5.255	1.00	0.00
ATOM	158	H	TYR	12	6.913	-8.951	-5.179	1.00	0.00
ATOM	159	CA	TYR	12	6.166	-6.996	-5.474	1.00	0.00
ATOM	160	HA	TYR	12	5.420	-6.441	-4.905	1.00	0.00
ATOM	161	CB	TYR	12	7.549	-6.591	-4.987	1.00	0.00
ATOM	162	HB2	TYR	12	7.626	-6.921	-3.951	1.00	0.00
ATOM	163	HB3	TYR	12	8.286	-7.119	-5.592	1.00	0.00
ATOM	164	CG	TYR	12	7.808	-5.109	-5.062	1.00	0.00
ATOM	165	CD1	TYR	12	6.986	-4.217	-4.354	1.00	0.00
ATOM	166	HD1	TYR	12	6.160	-4.603	-3.758	1.00	0.00
ATOM	167	CE1	TYR	12	7.224	-2.833	-4.412	1.00	0.00
ATOM	168	HE1	TYR	12	6.559	-2.142	-3.895	1.00	0.00
ATOM	169	CZ	TYR	12	8.274	-2.337	-5.204	1.00	0.00
ATOM	170	OH	TYR	12	8.539	-1.003	-5.220	1.00	0.00
ATOM	171	HH	TYR	12	7.950	-0.496	-4.657	1.00	0.00
ATOM	172	CE2	TYR	12	9.100	-3.232	-5.917	1.00	0.00
ATOM	173	HE2	TYR	12	9.925	-2.849	-6.519	1.00	0.00
ATOM	174	CD2	TYR	12	8.860	-4.617	-5.852	1.00	0.00
ATOM	175	HD2	TYR	12	9.490	-5.306	-6.415	1.00	0.00

ATOM	176	C	TYR	12	6.033	-6.630	-6.947	1.00	0.00
ATOM	177	O	TYR	12	5.370	-5.668	-7.285	1.00	0.00
ATOM	178	N	GLN	13	6.648	-7.420	-7.820	1.00	0.00
ATOM	179	H	GLN	13	7.192	-8.210	-7.505	1.00	0.00
ATOM	180	CA	GLN	13	6.577	-7.159	-9.242	1.00	0.00
ATOM	181	HA	GLN	13	6.947	-6.157	-9.463	1.00	0.00
ATOM	182	CB	GLN	13	7.442	-8.174	-9.988	1.00	0.00
ATOM	183	HB2	GLN	13	8.492	-7.963	-9.785	1.00	0.00
ATOM	184	HB3	GLN	13	7.198	-9.176	-9.636	1.00	0.00
ATOM	185	CG	GLN	13	7.187	-8.087	-11.498	1.00	0.00
ATOM	186	HG2	GLN	13	6.151	-8.339	-11.724	1.00	0.00
ATOM	187	HG3	GLN	13	7.383	-7.057	-11.797	1.00	0.00
ATOM	188	CD	GLN	13	8.093	-9.003	-12.296	1.00	0.00
ATOM	189	OE1	GLN	13	9.064	-9.563	-11.808	1.00	0.00
ATOM	190	NE2	GLN	13	7.771	-9.216	-13.551	1.00	0.00
ATOM	191	HE21	GLN	13	8.346	-9.819	-14.122	1.00	0.00
ATOM	192	HE22	GLN	13	6.950	-8.775	-13.941	1.00	0.00
ATOM	193	C	GLN	13	5.130	-7.253	-9.709	1.00	0.00
ATOM	194	O	GLN	13	4.661	-6.463	-10.491	1.00	0.00
ATOM	195	N	GLN	14	4.403	-8.263	-9.249	1.00	0.00
ATOM	196	H	GLN	14	4.828	-8.978	-8.677	1.00	0.00
ATOM	197	CA	GLN	14	3.002	-8.408	-9.607	1.00	0.00

ATOM	198	HA	GLN	14	2.885	-8.320	-10.688	1.00	0.00
ATOM	199	CB	GLN	14	2.478	-9.777	-9.168	1.00	0.00
ATOM	200	HB2	GLN	14	1.389	-9.739	-9.147	1.00	0.00
ATOM	201	HB3	GLN	14	2.801	-10.520	-9.897	1.00	0.00
ATOM	202	CG	GLN	14	3.000	-10.159	-7.788	1.00	0.00
ATOM	203	HG2	GLN	14	3.923	-10.718	-7.941	1.00	0.00
ATOM	204	HG3	GLN	14	3.217	-9.245	-7.235	1.00	0.00
ATOM	205	CD	GLN	14	2.062	-10.996	-6.979	1.00	0.00
ATOM	206	OE1	GLN	14	1.305	-11.753	-7.532	1.00	0.00
ATOM	207	NE2	GLN	14	2.096	-10.847	-5.662	1.00	0.00
ATOM	208	HE21	GLN	14	1.479	-11.393	-5.079	1.00	0.00
ATOM	209	HE22	GLN	14	2.739	-10.187	-5.248	1.00	0.00
ATOM	210	C	GLN	14	2.147	-7.298	-9.006	1.00	0.00
ATOM	211	O	GLN	14	1.128	-6.941	-9.566	1.00	0.00
ATOM	212	N	MET	15	2.541	-6.790	-7.844	1.00	0.00
ATOM	213	H	MET	15	3.382	-7.167	-7.432	1.00	0.00
ATOM	214	CA	MET	15	1.863	-5.725	-7.157	1.00	0.00
ATOM	215	HA	MET	15	0.890	-6.075	-6.815	1.00	0.00
ATOM	216	CB	MET	15	2.659	-5.255	-5.956	1.00	0.00
ATOM	217	HB2	MET	15	3.588	-5.823	-5.911	1.00	0.00
ATOM	218	HB3	MET	15	2.888	-4.196	-6.084	1.00	0.00
ATOM	219	CG	MET	15	1.890	-5.451	-4.682	1.00	0.00

ATOM	220	HG2	MET	15	0.881	-5.056	-4.799	1.00	0.00
ATOM	221	HG3	MET	15	1.839	-6.513	-4.445	1.00	0.00
ATOM	222	SD	MET	15	2.710	-4.586	-3.342	1.00	0.00
ATOM	223	CE	MET	15	3.549	-5.964	-2.609	1.00	0.00
ATOM	224	HE1	MET	15	4.223	-6.410	-3.340	1.00	0.00
ATOM	225	HE2	MET	15	4.123	-5.624	-1.747	1.00	0.00
ATOM	226	HE3	MET	15	2.818	-6.706	-2.289	1.00	0.00
ATOM	227	C	MET	15	1.689	-4.536	-8.057	1.00	0.00
ATOM	228	O	MET	15	0.587	-4.039	-8.109	1.00	0.00
ATOM	229	N	GLU	16	2.741	-4.062	-8.716	1.00	0.00
ATOM	230	H	GLU	16	3.652	-4.464	-8.546	1.00	0.00
ATOM	231	CA	GLU	16	2.643	-2.936	-9.641	1.00	0.00
ATOM	232	HA	GLU	16	2.388	-2.024	-9.102	1.00	0.00
ATOM	233	CB	GLU	16	3.995	-2.730	-10.341	1.00	0.00
ATOM	234	HB2	GLU	16	4.096	-1.672	-10.587	1.00	0.00
ATOM	235	HB3	GLU	16	4.787	-3.019	-9.650	1.00	0.00
ATOM	236	CG	GLU	16	4.106	-3.566	-11.625	1.00	0.00
ATOM	237	HG2	GLU	16	3.732	-4.550	-11.345	1.00	0.00
ATOM	238	HG3	GLU	16	3.487	-3.157	-12.424	1.00	0.00
ATOM	239	CD	GLU	16	5.548	-3.689	-12.111	1.00	0.00
ATOM	240	OE1	GLU	16	6.391	-4.256	-11.374	1.00	0.00
ATOM	241	OE2	GLU	16	5.848	-3.143	-13.193	1.00	0.00

ATOM 242 C GLU 16 1.527 -3.154 -10.672 1.00 0.00
ATOM 243 O GLU 16 0.844 -2.209 -11.057 1.00 0.00
ATOM 244 N GLU 17 1.313 -4.419 -11.050 1.00 0.00
ATOM 245 H GLU 17 1.893 -5.152 -10.669 1.00 0.00
ATOM 246 CA GLU 17 0.300 -4.778 -12.010 1.00 0.00
ATOM 247 HA GLU 17 0.297 -4.060 -12.831 1.00 0.00
ATOM 248 CB GLU 17 0.577 -6.175 -12.581 1.00 0.00
ATOM 249 HB2 GLU 17 1.612 -6.451 -12.379 1.00 0.00
ATOM 250 HB3 GLU 17 -0.090 -6.898 -12.113 1.00 0.00
ATOM 251 CG GLU 17 0.338 -6.166 -14.094 1.00 0.00
ATOM 252 HG2 GLU 17 -0.252 -7.042 -14.362 1.00 0.00
ATOM 253 HG3 GLU 17 -0.215 -5.263 -14.354 1.00 0.00
ATOM 254 CD GLU 17 1.663 -6.190 -14.860 1.00 0.00
ATOM 255 OE1 GLU 17 2.551 -5.386 -14.525 1.00 0.00
ATOM 256 OE2 GLU 17 1.774 -7.026 -15.784 1.00 0.00
ATOM 257 C GLU 17 -1.074 -4.748 -11.349 1.00 0.00
ATOM 258 O GLU 17 -2.087 -4.509 -12.006 1.00 0.00
ATOM 259 N ALA 18 -1.134 -5.009 -10.045 1.00 0.00
ATOM 260 H ALA 18 -0.292 -5.323 -9.584 1.00 0.00
ATOM 261 CA ALA 18 -2.346 -4.947 -9.280 1.00 0.00
ATOM 262 HA ALA 18 -3.190 -4.594 -9.873 1.00 0.00
ATOM 263 CB ALA 18 -2.648 -6.359 -8.793 1.00 0.00

ATOM 264 HB1 ALA 18 -1.815 -6.721 -8.191 1.00 0.00
ATOM 265 HB2 ALA 18 -3.556 -6.349 -8.190 1.00 0.00
ATOM 266 HB3 ALA 18 -2.789 -7.017 -9.651 1.00 0.00
ATOM 267 C ALA 18 -2.177 -3.936 -8.171 1.00 0.00
ATOM 268 O ALA 18 -2.083 -4.336 -7.024 1.00 0.00
ATOM 269 N GLU 19 -2.058 -2.647 -8.464 1.00 0.00
ATOM 270 H GLU 19 -2.003 -2.391 -9.440 1.00 0.00
ATOM 271 CA GLU 19 -1.887 -1.602 -7.467 1.00 0.00
ATOM 272 HA GLU 19 -1.092 -1.874 -6.774 1.00 0.00
ATOM 273 CB GLU 19 -1.511 -0.283 -8.148 1.00 0.00
ATOM 274 HB2 GLU 19 -0.464 -0.332 -8.449 1.00 0.00
ATOM 275 HB3 GLU 19 -2.136 -0.157 -9.032 1.00 0.00
ATOM 276 CG GLU 19 -1.714 0.899 -7.211 1.00 0.00
ATOM 277 HG2 GLU 19 -1.904 0.625 -6.174 1.00 0.00
ATOM 278 HG3 GLU 19 -0.778 1.455 -7.275 1.00 0.00
ATOM 279 CD GLU 19 -2.886 1.790 -7.737 1.00 0.00
ATOM 280 OE1 GLU 19 -3.868 1.401 -8.414 1.00 0.00
ATOM 281 OE2 GLU 19 -2.909 2.968 -7.363 1.00 0.00
ATOM 282 C GLU 19 -3.186 -1.541 -6.614 1.00 0.00
ATOM 283 O GLU 19 -4.028 -0.650 -6.597 1.00 0.00
ATOM 284 N MET 20 -3.410 -2.497 -5.758 1.00 0.00
ATOM 285 H MET 20 -2.741 -3.248 -5.665 1.00 0.00

ATOM	286	CA	MET	20	-4.602	-2.538	-4.948	1.00	0.00
ATOM	287	HA	MET	20	-5.382	-1.907	-5.376	1.00	0.00
ATOM	288	CB	MET	20	-5.103	-3.962	-4.903	1.00	0.00
ATOM	289	HB2	MET	20	-6.173	-3.924	-4.699	1.00	0.00
ATOM	290	HB3	MET	20	-4.936	-4.397	-5.889	1.00	0.00
ATOM	291	CG	MET	20	-4.423	-4.807	-3.860	1.00	0.00
ATOM	292	HG2	MET	20	-3.437	-4.371	-3.696	1.00	0.00
ATOM	293	HG3	MET	20	-5.015	-4.719	-2.949	1.00	0.00
ATOM	294	SD	MET	20	-4.242	-6.514	-4.272	1.00	0.00
ATOM	295	CE	MET	20	-2.973	-6.432	-5.493	1.00	0.00
ATOM	296	HE1	MET	20	-2.076	-5.992	-5.057	1.00	0.00
ATOM	297	HE2	MET	20	-2.747	-7.436	-5.852	1.00	0.00
ATOM	298	HE3	MET	20	-3.310	-5.816	-6.328	1.00	0.00
ATOM	299	C	MET	20	-4.255	-2.030	-3.577	1.00	0.00
ATOM	300	O	MET	20	-5.149	-1.695	-2.801	1.00	0.00
ATOM	301	N	LEU	21	-2.955	-2.010	-3.260	1.00	0.00
ATOM	302	H	LEU	21	-2.283	-2.391	-3.911	1.00	0.00
ATOM	303	CA	LEU	21	-2.483	-1.569	-1.971	1.00	0.00
ATOM	304	HA	LEU	21	-3.234	-1.766	-1.207	1.00	0.00
ATOM	305	CB	LEU	21	-1.213	-2.321	-1.610	1.00	0.00
ATOM	306	HB2	LEU	21	-0.546	-1.965	-2.396	1.00	0.00
ATOM	307	HB3	LEU	21	-0.828	-2.010	-0.639	1.00	0.00

ATOM	308	CG	LEU	21	-1.293	-3.860	-1.662	1.00	0.00
ATOM	309	HG	LEU	21	-0.525	-4.248	-0.993	1.00	0.00
ATOM	310	CD1	LEU	21	-2.645	-4.414	-1.256	1.00	0.00
ATOM	311	HD11	LEU	21	-3.413	-4.026	-1.926	1.00	0.00
ATOM	312	HD12	LEU	21	-2.625	-5.502	-1.318	1.00	0.00
ATOM	313	HD13	LEU	21	-2.870	-4.112	-0.234	1.00	0.00
ATOM	314	CD2	LEU	21	-0.843	-4.349	-3.026	1.00	0.00
ATOM	315	HD21	LEU	21	0.185	-4.034	-3.205	1.00	0.00
ATOM	316	HD22	LEU	21	-0.900	-5.437	-3.060	1.00	0.00
ATOM	317	HD23	LEU	21	-1.490	-3.927	-3.796	1.00	0.00
ATOM	318	C	LEU	21	-2.319	-0.061	-1.975	1.00	0.00
ATOM	319	O	LEU	21	-1.747	0.507	-1.058	1.00	0.00
ATOM	320	N	ASN	22	-2.893	0.582	-2.990	1.00	0.00
ATOM	321	H	ASN	22	-3.390	0.056	-3.695	1.00	0.00
ATOM	322	CA	ASN	22	-2.864	2.014	-3.103	1.00	0.00
ATOM	323	HA	ASN	22	-2.073	2.422	-2.474	1.00	0.00
ATOM	324	CB	ASN	22	-2.598	2.418	-4.536	1.00	0.00
ATOM	325	HB2	ASN	22	-1.652	2.157	-5.010	1.00	0.00
ATOM	326	HB3	ASN	22	-3.402	1.839	-4.989	1.00	0.00
ATOM	327	CG	ASN	22	-2.871	3.901	-4.776	1.00	0.00
ATOM	328	OD1	ASN	22	-3.958	4.336	-5.140	1.00	0.00
ATOM	329	ND2	ASN	22	-1.963	4.714	-4.286	1.00	0.00

ATOM 330 HD21 ASN 22 -2.068 5.712 -4.402 1.00 0.00

ATOM 331 HD22 ASN 22 -1.164 4.338 -3.797 1.00 0.00

ATOM 332 C ASN 22 -4.209 2.576 -2.709 1.00 0.00

ATOM 333 O ASN 22 -4.263 3.593 -2.046 1.00 0.00

ATOM 334 N HIE 23 -5.307 1.899 -3.057 1.00 0.00

ATOM 335 H HIE 23 -5.207 1.045 -3.586 1.00 0.00

ATOM 336 CA HIE 23 -6.643 2.361 -2.744 1.00 0.00

ATOM 337 HA HIE 23 -6.749 3.415 -3.003 1.00 0.00

ATOM 338 CB HIE 23 -7.659 1.558 -3.547 1.00 0.00

ATOM 339 HB2 HIE 23 -7.486 0.489 -3.429 1.00 0.00

ATOM 340 HB3 HIE 23 -8.666 1.803 -3.209 1.00 0.00

ATOM 341 CG HIE 23 -7.622 1.839 -5.022 1.00 0.00

ATOM 342 ND1 HIE 23 -8.581 2.554 -5.713 1.00 0.00

ATOM 343 CE1 HIE 23 -8.212 2.588 -7.014 1.00 0.00

ATOM 344 HE1 HIE 23 -8.833 3.111 -7.741 1.00 0.00

ATOM 345 NE2 HIE 23 -7.048 1.909 -7.164 1.00 0.00

ATOM 346 HE2 HIE 23 -6.535 1.765 -8.023 1.00 0.00

ATOM 347 CD2 HIE 23 -6.666 1.435 -5.922 1.00 0.00

ATOM 348 HD2 HIE 23 -5.747 0.858 -5.818 1.00 0.00

ATOM 349 C HIE 23 -6.885 2.218 -1.255 1.00 0.00

ATOM 350 O HIE 23 -7.142 3.225 -0.600 1.00 0.00

ATOM 351 N CYX 24 -6.817 0.980 -0.741 1.00 0.00

ATOM	352	H	CYX	24	-6.687	0.183	-1.348	1.00	0.00
ATOM	353	CA	CYX	24	-6.960	0.743	0.690	1.00	0.00
ATOM	354	HA	CYX	24	-7.959	1.041	1.008	1.00	0.00
ATOM	355	CB	CYX	24	-6.758	-0.731	1.031	1.00	0.00
ATOM	356	HB2	CYX	24	-6.348	-1.251	0.165	1.00	0.00
ATOM	357	HB3	CYX	24	-6.066	-0.819	1.868	1.00	0.00
ATOM	358	SG	CYX	24	-8.257	-1.600	1.493	1.00	0.00
ATOM	359	C	CYX	24	-5.951	1.586	1.458	1.00	0.00
ATOM	360	O	CYX	24	-6.275	2.019	2.542	1.00	0.00
ATOM	361	N	GLY	25	-4.808	1.913	0.852	1.00	0.00
ATOM	362	H	GLY	25	-4.637	1.540	-0.071	1.00	0.00
ATOM	363	CA	GLY	25	-3.809	2.765	1.439	1.00	0.00
ATOM	364	HA2	GLY	25	-3.619	2.436	2.460	1.00	0.00
ATOM	365	HA3	GLY	25	-2.891	2.687	0.856	1.00	0.00
ATOM	366	C	GLY	25	-4.254	4.206	1.463	1.00	0.00
ATOM	367	O	GLY	25	-4.218	4.819	2.513	1.00	0.00
ATOM	368	N	MET	26	-4.793	4.724	0.368	1.00	0.00
ATOM	369	H	MET	26	-4.924	4.098	-0.414	1.00	0.00
ATOM	370	CA	MET	26	-5.238	6.088	0.233	1.00	0.00
ATOM	371	HA	MET	26	-4.387	6.763	0.333	1.00	0.00
ATOM	372	CB	MET	26	-5.883	6.316	-1.131	1.00	0.00
ATOM	373	HB2	MET	26	-5.815	5.395	-1.709	1.00	0.00

ATOM	374	HB3	MET	26	-6.931	6.576	-0.984	1.00	0.00
ATOM	375	CG	MET	26	-5.184	7.438	-1.883	1.00	0.00
ATOM	376	HG2	MET	26	-5.794	8.341	-1.836	1.00	0.00
ATOM	377	HG3	MET	26	-4.214	7.631	-1.425	1.00	0.00
ATOM	378	SD	MET	26	-4.943	6.978	-3.605	1.00	0.00
ATOM	379	CE	MET	26	-3.607	8.113	-4.035	1.00	0.00
ATOM	380	HE1	MET	26	-2.745	7.919	-3.397	1.00	0.00
ATOM	381	HE2	MET	26	-3.326	7.967	-5.078	1.00	0.00
ATOM	382	HE3	MET	26	-3.941	9.141	-3.891	1.00	0.00
ATOM	383	C	MET	26	-6.284	6.393	1.260	1.00	0.00
ATOM	384	O	MET	26	-6.203	7.436	1.864	1.00	0.00
ATOM	385	N	TYR	27	-7.292	5.545	1.426	1.00	0.00
ATOM	386	H	TYR	27	-7.344	4.740	0.819	1.00	0.00
ATOM	387	CA	TYR	27	-8.317	5.709	2.445	1.00	0.00
ATOM	388	HA	TYR	27	-9.037	6.481	2.172	1.00	0.00
ATOM	389	CB	TYR	27	-9.047	4.374	2.544	1.00	0.00
ATOM	390	HB2	TYR	27	-9.482	4.183	1.563	1.00	0.00
ATOM	391	HB3	TYR	27	-8.304	3.608	2.767	1.00	0.00
ATOM	392	CG	TYR	27	-10.135	4.341	3.592	1.00	0.00
ATOM	393	CD1	TYR	27	-11.284	5.132	3.425	1.00	0.00
ATOM	394	HD1	TYR	27	-11.389	5.764	2.542	1.00	0.00
ATOM	395	CE1	TYR	27	-12.300	5.109	4.398	1.00	0.00

ATOM	396	HE1	TYR	27	-13.203	5.703	4.258	1.00	0.00
ATOM	397	CZ	TYR	27	-12.165	4.286	5.536	1.00	0.00
ATOM	398	OH	TYR	27	-13.129	4.272	6.495	1.00	0.00
ATOM	399	HH	TYR	27	-13.861	4.861	6.299	1.00	0.00
ATOM	400	CE2	TYR	27	-11.010	3.502	5.704	1.00	0.00
ATOM	401	HE2	TYR	27	-10.905	2.871	6.586	1.00	0.00
ATOM	402	CD2	TYR	27	-9.991	3.532	4.735	1.00	0.00
ATOM	403	HD2	TYR	27	-9.092	2.930	4.870	1.00	0.00
ATOM	404	C	TYR	27	-7.740	6.058	3.813	1.00	0.00
ATOM	405	O	TYR	27	-8.280	6.896	4.527	1.00	0.00
ATOM	406	N	LEU	28	-6.584	5.481	4.122	1.00	0.00
ATOM	407	H	LEU	28	-6.183	4.819	3.473	1.00	0.00
ATOM	408	CA	LEU	28	-5.904	5.710	5.374	1.00	0.00
ATOM	409	HA	LEU	28	-6.626	6.007	6.135	1.00	0.00
ATOM	410	CB	LEU	28	-5.196	4.447	5.841	1.00	0.00
ATOM	411	HB2	LEU	28	-4.243	4.514	5.315	1.00	0.00
ATOM	412	HB3	LEU	28	-5.021	4.489	6.916	1.00	0.00
ATOM	413	CG	LEU	28	-5.887	3.118	5.485	1.00	0.00
ATOM	414	HG	LEU	28	-6.587	3.301	4.669	1.00	0.00
ATOM	415	CD1	LEU	28	-4.880	2.061	5.061	1.00	0.00
ATOM	416	HD11	LEU	28	-4.180	1.878	5.876	1.00	0.00
ATOM	417	HD12	LEU	28	-5.403	1.137	4.817	1.00	0.00

ATOM	418	HD13	LEU	28	-4.333	2.410	4.185	1.00	0.00
ATOM	419	CD2	LEU	28	-6.750	2.645	6.617	1.00	0.00
ATOM	420	HD21	LEU	28	-7.515	3.393	6.828	1.00	0.00
ATOM	421	HD22	LEU	28	-7.228	1.705	6.342	1.00	0.00
ATOM	422	HD23	LEU	28	-6.135	2.494	7.504	1.00	0.00
ATOM	423	C	LEU	28	-4.927	6.863	5.216	1.00	0.00
ATOM	424	O	LEU	28	-4.711	7.597	6.163	1.00	0.00
ATOM	425	N	MET	29	-4.437	7.121	4.007	1.00	0.00
ATOM	426	H	MET	29	-4.629	6.414	3.312	1.00	0.00
ATOM	427	CA	MET	29	-3.599	8.236	3.634	1.00	0.00
ATOM	428	HA	MET	29	-2.957	8.523	4.467	1.00	0.00
ATOM	429	CB	MET	29	-2.718	7.838	2.455	1.00	0.00
ATOM	430	HB2	MET	29	-2.920	6.797	2.203	1.00	0.00
ATOM	431	HB3	MET	29	-2.966	8.472	1.603	1.00	0.00
ATOM	432	CG	MET	29	-1.247	8.000	2.795	1.00	0.00
ATOM	433	HG2	MET	29	-1.072	7.596	3.791	1.00	0.00
ATOM	434	HG3	MET	29	-0.657	7.442	2.068	1.00	0.00
ATOM	435	SD	MET	29	-0.747	9.724	2.762	1.00	0.00
ATOM	436	CE	MET	29	-0.752	9.972	0.977	1.00	0.00
ATOM	437	HE1	MET	29	-1.751	9.783	0.586	1.00	0.00
ATOM	438	HE2	MET	29	-0.463	10.999	0.751	1.00	0.00
ATOM	439	HE3	MET	29	-0.044	9.286	0.512	1.00	0.00

ATOM	440	C	MET	29	-4.387	9.507	3.335	1.00	0.00
ATOM	441	O	MET	29	-3.853	10.498	2.850	1.00	0.00
ATOM	442	N	LYS	30	-5.695	9.490	3.562	1.00	0.00
ATOM	443	H	LYS	30	-6.118	8.631	3.881	1.00	0.00
ATOM	444	CA	LYS	30	-6.555	10.620	3.296	1.00	0.00
ATOM	445	HA	LYS	30	-6.213	11.156	2.410	1.00	0.00
ATOM	446	CB	LYS	30	-7.985	10.126	3.048	1.00	0.00
ATOM	447	HB2	LYS	30	-8.054	9.072	3.314	1.00	0.00
ATOM	448	HB3	LYS	30	-8.677	10.702	3.662	1.00	0.00
ATOM	449	CG	LYS	30	-8.346	10.303	1.568	1.00	0.00
ATOM	450	HG2	LYS	30	-7.435	10.260	0.970	1.00	0.00
ATOM	451	HG3	LYS	30	-9.017	9.500	1.265	1.00	0.00
ATOM	452	CD	LYS	30	-9.036	11.654	1.350	1.00	0.00
ATOM	453	HD2	LYS	30	-9.944	11.478	0.774	1.00	0.00
ATOM	454	HD3	LYS	30	-9.299	12.059	2.327	1.00	0.00
ATOM	455	CE	LYS	30	-8.151	12.650	0.610	1.00	0.00
ATOM	456	HE2	LYS	30	-8.575	13.649	0.718	1.00	0.00
ATOM	457	HE3	LYS	30	-7.153	12.630	1.048	1.00	0.00
ATOM	458	NZ	LYS	30	-8.065	12.303	-0.832	1.00	0.00
ATOM	459	HZ1	LYS	30	-8.989	12.322	-1.239	1.00	0.00
ATOM	460	HZ2	LYS	30	-7.475	12.973	-1.305	1.00	0.00
ATOM	461	HZ3	LYS	30	-7.672	11.379	-0.933	1.00	0.00

ATOM 462 C LYS 30 -6.456 11.587 4.453 1.00 0.00

ATOM 463 O LYS 30 -6.761 12.751 4.242 1.00 0.00

ATOM 464 N ASN 31 -6.066 11.087 5.629 1.00 0.00

ATOM 465 H ASN 31 -5.938 10.090 5.721 1.00 0.00

ATOM 466 CA ASN 31 -5.829 11.924 6.795 1.00 0.00

ATOM 467 HA ASN 31 -6.244 12.921 6.647 1.00 0.00

ATOM 468 CB ASN 31 -6.519 11.280 8.000 1.00 0.00

ATOM 469 HB2 ASN 31 -6.499 10.193 7.935 1.00 0.00

ATOM 470 HB3 ASN 31 -5.983 11.601 8.893 1.00 0.00

ATOM 471 CG ASN 31 -7.955 11.730 8.112 1.00 0.00

ATOM 472 OD1 ASN 31 -8.358 12.446 9.003 1.00 0.00

ATOM 473 ND2 ASN 31 -8.758 11.342 7.150 1.00 0.00

ATOM 474 HD21 ASN 31 -9.731 11.612 7.169 1.00 0.00

ATOM 475 HD22 ASN 31 -8.399 10.776 6.394 1.00 0.00

ATOM 476 C ASN 31 -4.338 12.077 7.084 1.00 0.00

ATOM 477 O ASN 31 -3.974 12.826 7.979 1.00 0.00

ATOM 478 N LEU 32 -3.513 11.324 6.355 1.00 0.00

ATOM 479 H LEU 32 -3.885 10.646 5.706 1.00 0.00

ATOM 480 CA LEU 32 -2.092 11.392 6.526 1.00 0.00

ATOM 481 HA LEU 32 -1.855 11.632 7.562 1.00 0.00

ATOM 482 CB LEU 32 -1.449 10.056 6.176 1.00 0.00

ATOM 483 HB2 LEU 32 -1.792 9.823 5.167 1.00 0.00

ATOM	484	HB3	LEU	32	-0.363	10.156	6.175	1.00	0.00
ATOM	485	CG	LEU	32	-1.855	8.934	7.122	1.00	0.00
ATOM	486	HG	LEU	32	-2.924	9.006	7.319	1.00	0.00
ATOM	487	CD1	LEU	32	-1.544	7.582	6.505	1.00	0.00
ATOM	488	HD11	LEU	32	-0.474	7.510	6.307	1.00	0.00
ATOM	489	HD12	LEU	32	-1.840	6.791	7.193	1.00	0.00
ATOM	490	HD13	LEU	32	-2.094	7.474	5.570	1.00	0.00
ATOM	491	CD2	LEU	32	-1.150	9.094	8.456	1.00	0.00
ATOM	492	HD21	LEU	32	-1.423	10.052	8.899	1.00	0.00
ATOM	493	HD22	LEU	32	-1.448	8.287	9.124	1.00	0.00
ATOM	494	HD23	LEU	32	-0.071	9.060	8.303	1.00	0.00
ATOM	495	C	LEU	32	-1.532	12.517	5.699	1.00	0.00
ATOM	496	O	LEU	32	-0.471	12.947	6.103	1.00	0.00
ATOM	497	N	GLY	33	-2.245	12.981	4.646	1.00	0.00
ATOM	498	H	GLY	33	-3.129	12.527	4.466	1.00	0.00
ATOM	499	CA	GLY	33	-1.837	14.068	3.768	1.00	0.00
ATOM	500	HA2	GLY	33	-2.360	13.968	2.816	1.00	0.00
ATOM	501	HA3	GLY	33	-2.107	15.017	4.232	1.00	0.00
ATOM	502	C	GLY	33	-0.334	14.052	3.512	1.00	0.00
ATOM	503	O	GLY	33	0.446	14.734	4.168	1.00	0.00
ATOM	504	N	GLU	34	0.072	13.307	2.492	1.00	0.00
ATOM	505	H	GLU	34	-0.585	12.769	1.946	1.00	0.00

ATOM	506	CA	GLU	34	1.488	13.186	2.182	1.00	0.00
ATOM	507	HA	GLU	34	2.066	13.187	3.106	1.00	0.00
ATOM	508	CB	GLU	34	1.773	11.886	1.429	1.00	0.00
ATOM	509	HB2	GLU	34	1.466	11.046	2.051	1.00	0.00
ATOM	510	HB3	GLU	34	1.195	11.883	0.504	1.00	0.00
ATOM	511	CG	GLU	34	3.262	11.759	1.100	1.00	0.00
ATOM	512	HG2	GLU	34	3.407	10.760	0.690	1.00	0.00
ATOM	513	HG3	GLU	34	3.538	12.502	0.351	1.00	0.00
ATOM	514	CD	GLU	34	4.141	11.934	2.339	1.00	0.00
ATOM	515	OE1	GLU	34	3.657	11.679	3.459	1.00	0.00
ATOM	516	OE2	GLU	34	5.299	12.362	2.174	1.00	0.00
ATOM	517	C	GLU	34	1.965	14.395	1.385	1.00	0.00
ATOM	518	O	GLU	34	2.180	14.321	0.173	1.00	0.00
ATOM	519	N	ARG	35	1.974	15.566	2.018	1.00	0.00
ATOM	520	H	ARG	35	1.642	15.616	2.970	1.00	0.00
ATOM	521	CA	ARG	35	2.414	16.791	1.379	1.00	0.00
ATOM	522	HA	ARG	35	3.435	16.674	1.016	1.00	0.00
ATOM	523	CB	ARG	35	1.502	17.128	0.183	1.00	0.00
ATOM	524	HB2	ARG	35	1.209	16.203	-0.312	1.00	0.00
ATOM	525	HB3	ARG	35	0.613	17.642	0.548	1.00	0.00
ATOM	526	CG	ARG	35	2.234	18.024	-0.809	1.00	0.00
ATOM	527	HG2	ARG	35	1.617	18.901	-1.010	1.00	0.00

ATOM	528	HG3	ARG	35	3.178	18.339	-0.365	1.00	0.00
ATOM	529	CD	ARG	35	2.510	17.282	-2.123	1.00	0.00
ATOM	530	HD2	ARG	35	2.521	16.208	-1.938	1.00	0.00
ATOM	531	HD3	ARG	35	1.728	17.519	-2.845	1.00	0.00
ATOM	532	NE	ARG	35	3.809	17.675	-2.691	1.00	0.00
ATOM	533	HE	ARG	35	4.439	18.158	-2.067	1.00	0.00
ATOM	534	CZ	ARG	35	4.221	17.401	-3.912	1.00	0.00
ATOM	535	NH1	ARG	35	3.444	16.777	-4.762	1.00	0.00
ATOM	536	HH11	ARG	35	2.514	16.500	-4.482	1.00	0.00
ATOM	537	HH12	ARG	35	3.779	16.577	-5.694	1.00	0.00
ATOM	538	NH2	ARG	35	5.399	17.801	-4.313	1.00	0.00
ATOM	539	HH21	ARG	35	5.994	18.322	-3.684	1.00	0.00
ATOM	540	HH22	ARG	35	5.709	17.588	-5.251	1.00	0.00
ATOM	541	C	ARG	35	2.394	17.913	2.393	1.00	0.00
ATOM	542	O	ARG	35	3.447	18.428	2.729	1.00	0.00
ATOM	543	N	SER	36	1.189	18.344	2.777	1.00	0.00
ATOM	544	H	SER	36	0.371	17.912	2.372	1.00	0.00
ATOM	545	CA	SER	36	0.997	19.420	3.736	1.00	0.00
ATOM	546	HA	SER	36	1.512	19.192	4.669	1.00	0.00
ATOM	547	CB	SER	36	1.571	20.720	3.166	1.00	0.00
ATOM	548	HB2	SER	36	2.649	20.608	3.051	1.00	0.00
ATOM	549	HB3	SER	36	1.120	20.907	2.191	1.00	0.00

ATOM	550	OG	SER	36	1.302	21.801	4.020	1.00	0.00
ATOM	551	HG	SER	36	1.670	22.605	3.644	1.00	0.00
ATOM	552	C	SER	36	-0.487	19.588	4.028	1.00	0.00
ATOM	553	O	SER	36	-0.936	19.369	5.146	1.00	0.00
ATOM	554	N	GLN	37	-1.269	19.925	2.996	1.00	0.00
ATOM	555	H	GLN	37	-0.854	20.094	2.090	1.00	0.00
ATOM	556	CA	GLN	37	-2.705	20.123	3.145	1.00	0.00
ATOM	557	HA	GLN	37	-3.140	19.301	3.712	1.00	0.00
ATOM	558	CB	GLN	37	-2.975	21.429	3.893	1.00	0.00
ATOM	559	HB2	GLN	37	-2.251	21.543	4.700	1.00	0.00
ATOM	560	HB3	GLN	37	-2.884	22.269	3.203	1.00	0.00
ATOM	561	CG	GLN	37	-4.389	21.400	4.477	1.00	0.00
ATOM	562	HG2	GLN	37	-5.118	21.569	3.684	1.00	0.00
ATOM	563	HG3	GLN	37	-4.556	20.419	4.921	1.00	0.00
ATOM	564	CD	GLN	37	-4.585	22.459	5.550	1.00	0.00
ATOM	565	OE1	GLN	37	-3.683	23.149	5.986	1.00	0.00
ATOM	566	NE2	GLN	37	-5.808	22.628	6.000	1.00	0.00
ATOM	567	HE21	GLN	37	-5.989	23.320	6.713	1.00	0.00
ATOM	568	HE22	GLN	37	-6.561	22.066	5.630	1.00	0.00
ATOM	569	C	GLN	37	-3.387	20.118	1.785	1.00	0.00
ATOM	570	O	GLN	37	-3.774	21.145	1.233	1.00	0.00
ATOM	571	N	VAL	38	-3.406	18.947	1.160	1.00	0.00

ATOM	572	H	VAL	38	-2.896	18.159	1.532	1.00	0.00
ATOM	573	CA	VAL	38	-4.062	18.797	-0.131	1.00	0.00
ATOM	574	HA	VAL	38	-3.849	19.635	-0.796	1.00	0.00
ATOM	575	CB	VAL	38	-3.598	17.506	-0.808	1.00	0.00
ATOM	576	HB	VAL	38	-4.125	17.381	-1.754	1.00	0.00
ATOM	577	CG1	VAL	38	-2.088	17.582	-1.071	1.00	0.00
ATOM	578	HG11	VAL	38	-1.560	17.707	-0.126	1.00	0.00
ATOM	579	HG12	VAL	38	-1.756	16.663	-1.554	1.00	0.00
ATOM	580	HG13	VAL	38	-1.874	18.431	-1.721	1.00	0.00
ATOM	581	CG2	VAL	38	-3.925	16.269	0.037	1.00	0.00
ATOM	582	HG21	VAL	38	-5.002	16.207	0.188	1.00	0.00
ATOM	583	HG22	VAL	38	-3.579	15.374	-0.480	1.00	0.00
ATOM	584	HG23	VAL	38	-3.426	16.347	1.003	1.00	0.00
ATOM	585	C	VAL	38	-5.573	18.868	0.022	1.00	0.00
ATOM	586	O	VAL	38	-6.077	18.889	1.146	1.00	0.00
ATOM	587	N	SER	39	-6.284	18.876	-1.111	1.00	0.00
ATOM	588	H	SER	39	-5.807	18.929	-2.000	1.00	0.00
ATOM	589	CA	SER	39	-7.736	18.900	-1.107	1.00	0.00
ATOM	590	HA	SER	39	-8.085	19.853	-0.709	1.00	0.00
ATOM	591	CB	SER	39	-8.291	18.730	-2.536	1.00	0.00
ATOM	592	HB2	SER	39	-9.125	19.422	-2.656	1.00	0.00
ATOM	593	HB3	SER	39	-7.498	18.993	-3.237	1.00	0.00

ATOM	594	OG	SER	39	-8.733	17.405	-2.797	1.00	0.00
ATOM	595	HG	SER	39	-9.068	17.351	-3.695	1.00	0.00
ATOM	596	C	SER	39	-8.314	17.834	-0.163	1.00	0.00
ATOM	597	O	SER	39	-7.679	16.825	0.184	1.00	0.00
ATOM	598	N	PRO	40	-9.603	17.974	0.151	1.00	0.00
ATOM	599	CD	PRO	40	-10.502	19.024	-0.299	1.00	0.00
ATOM	600	HD2	PRO	40	-10.647	18.903	-1.373	1.00	0.00
ATOM	601	HD3	PRO	40	-10.021	19.983	-0.105	1.00	0.00
ATOM	602	CG	PRO	40	-11.796	18.852	0.491	1.00	0.00
ATOM	603	HG2	PRO	40	-12.607	18.619	-0.199	1.00	0.00
ATOM	604	HG3	PRO	40	-12.021	19.782	1.013	1.00	0.00
ATOM	605	CB	PRO	40	-11.560	17.714	1.480	1.00	0.00
ATOM	606	HB2	PRO	40	-12.395	17.015	1.452	1.00	0.00
ATOM	607	HB3	PRO	40	-11.429	18.083	2.497	1.00	0.00
ATOM	608	CA	PRO	40	-10.277	17.014	1.007	1.00	0.00
ATOM	609	HA	PRO	40	-9.645	16.822	1.873	1.00	0.00
ATOM	610	C	PRO	40	-10.550	15.698	0.273	1.00	0.00
ATOM	611	O	PRO	40	-9.989	15.368	-0.779	1.00	0.00
ATOM	612	N	ARG	41	-11.407	14.866	0.858	1.00	0.00
ATOM	613	H	ARG	41	-11.743	15.088	1.784	1.00	0.00
ATOM	614	CA	ARG	41	-11.828	13.628	0.258	1.00	0.00
ATOM	615	HA	ARG	41	-10.981	13.151	-0.235	1.00	0.00

ATOM 616 CB ARG 41 -12.377 12.674 1.322 1.00 0.00
ATOM 617 HB2 ARG 41 -11.750 12.735 2.211 1.00 0.00
ATOM 618 HB3 ARG 41 -13.395 12.970 1.574 1.00 0.00
ATOM 619 CG ARG 41 -12.379 11.228 0.794 1.00 0.00
ATOM 620 HG2 ARG 41 -11.797 11.208 -0.128 1.00 0.00
ATOM 621 HG3 ARG 41 -11.897 10.598 1.541 1.00 0.00
ATOM 622 CD ARG 41 -13.786 10.705 0.518 1.00 0.00
ATOM 623 HD2 ARG 41 -14.513 11.497 0.700 1.00 0.00
ATOM 624 HD3 ARG 41 -13.858 10.378 -0.520 1.00 0.00
ATOM 625 NE ARG 41 -14.097 9.574 1.388 1.00 0.00
ATOM 626 HE ARG 41 -14.025 8.656 0.975 1.00 0.00
ATOM 627 CZ ARG 41 -14.557 9.676 2.622 1.00 0.00
ATOM 628 NH1 ARG 41 -14.707 10.847 3.194 1.00 0.00
ATOM 629 HH11 ARG 41 -14.468 11.688 2.687 1.00 0.00
ATOM 630 HH12 ARG 41 -15.060 10.903 4.138 1.00 0.00
ATOM 631 NH2 ARG 41 -14.765 8.597 3.323 1.00 0.00
ATOM 632 HH21 ARG 41 -14.573 7.692 2.919 1.00 0.00
ATOM 633 HH22 ARG 41 -15.117 8.672 4.266 1.00 0.00
ATOM 634 C ARG 41 -12.857 13.891 -0.818 1.00 0.00
ATOM 635 O ARG 41 -13.870 13.236 -0.810 1.00 0.00
ATOM 636 N MET 42 -12.609 14.823 -1.725 1.00 0.00
ATOM 637 H MET 42 -11.754 15.358 -1.659 1.00 0.00

ATOM 638 CA MET 42 -13.521 15.114 -2.819 1.00 0.00
ATOM 639 HA MET 42 -14.396 15.654 -2.457 1.00 0.00
ATOM 640 CB MET 42 -12.797 15.988 -3.844 1.00 0.00
ATOM 641 HB2 MET 42 -13.417 16.061 -4.738 1.00 0.00
ATOM 642 HB3 MET 42 -12.658 16.981 -3.417 1.00 0.00
ATOM 643 CG MET 42 -11.429 15.396 -4.218 1.00 0.00
ATOM 644 HG2 MET 42 -10.687 15.801 -3.529 1.00 0.00
ATOM 645 HG3 MET 42 -11.486 14.315 -4.096 1.00 0.00
ATOM 646 SD MET 42 -10.936 15.776 -5.908 1.00 0.00
ATOM 647 CE MET 42 -10.463 17.508 -5.656 1.00 0.00
ATOM 648 HE1 MET 42 -9.658 17.563 -4.923 1.00 0.00
ATOM 649 HE2 MET 42 -10.122 17.934 -6.601 1.00 0.00
ATOM 650 HE3 MET 42 -11.323 18.071 -5.294 1.00 0.00
ATOM 651 C MET 42 -14.009 13.841 -3.522 1.00 0.00
ATOM 652 O MET 42 -15.105 13.836 -4.055 1.00 0.00
ATOM 653 N ARG 43 -13.179 12.780 -3.504 1.00 0.00
ATOM 654 H ARG 43 -12.239 12.911 -3.159 1.00 0.00
ATOM 655 CA ARG 43 -13.539 11.490 -4.029 1.00 0.00
ATOM 656 HA ARG 43 -13.677 11.557 -5.109 1.00 0.00
ATOM 657 CB ARG 43 -12.437 10.464 -3.739 1.00 0.00
ATOM 658 HB2 ARG 43 -11.516 10.982 -3.468 1.00 0.00
ATOM 659 HB3 ARG 43 -12.745 9.818 -2.918 1.00 0.00

ATOM	660	CG	ARG	43	-12.196	9.617	-4.993	1.00	0.00
ATOM	661	HG2	ARG	43	-11.901	8.612	-4.692	1.00	0.00
ATOM	662	HG3	ARG	43	-13.119	9.567	-5.571	1.00	0.00
ATOM	663	CD	ARG	43	-11.087	10.239	-5.854	1.00	0.00
ATOM	664	HD2	ARG	43	-11.123	9.812	-6.857	1.00	0.00
ATOM	665	HD3	ARG	43	-11.236	11.318	-5.914	1.00	0.00
ATOM	666	NE	ARG	43	-9.751	9.976	-5.274	1.00	0.00
ATOM	667	HE	ARG	43	-9.691	9.196	-4.637	1.00	0.00
ATOM	668	CZ	ARG	43	-8.643	10.626	-5.580	1.00	0.00
ATOM	669	NH1	ARG	43	-8.660	11.618	-6.429	1.00	0.00
ATOM	670	HH11	ARG	43	-9.530	11.896	-6.860	1.00	0.00
ATOM	671	HH12	ARG	43	-7.802	12.104	-6.651	1.00	0.00
ATOM	672	NH2	ARG	43	-7.518	10.332	-4.972	1.00	0.00
ATOM	673	HH21	ARG	43	-7.502	9.607	-4.270	1.00	0.00
ATOM	674	HH22	ARG	43	-6.673	10.833	-5.210	1.00	0.00
ATOM	675	C	ARG	43	-14.880	11.045	-3.466	1.00	0.00
ATOM	676	O	ARG	43	-15.123	11.155	-2.272	1.00	0.00
ATOM	677	N	GLU	44	-15.671	10.421	-4.301	1.00	0.00
ATOM	678	H	GLU	44	-15.408	10.340	-5.273	1.00	0.00
ATOM	679	CA	GLU	44	-16.958	9.875	-3.885	1.00	0.00
ATOM	680	HA	GLU	44	-17.175	10.171	-2.859	1.00	0.00
ATOM	681	CB	GLU	44	-18.068	10.407	-4.792	1.00	0.00

ATOM	682	HB2	GLU	44	-19.012	9.965	-4.476	1.00	0.00
ATOM	683	HB3	GLU	44	-18.117	11.490	-4.677	1.00	0.00
ATOM	684	CG	GLU	44	-17.809	10.060	-6.272	1.00	0.00
ATOM	685	HG2	GLU	44	-17.642	10.958	-6.868	1.00	0.00
ATOM	686	HG3	GLU	44	-16.906	9.450	-6.275	1.00	0.00
ATOM	687	CD	GLU	44	-18.968	9.259	-6.870	1.00	0.00
ATOM	688	OE1	GLU	44	-18.978	8.029	-6.640	1.00	0.00
ATOM	689	OE2	GLU	44	-19.847	9.912	-7.479	1.00	0.00
ATOM	690	C	GLU	44	-16.935	8.345	-3.912	1.00	0.00
ATOM	691	O	GLU	44	-17.666	7.703	-3.167	1.00	0.00
ATOM	692	N	GLU	45	-15.983	7.772	-4.657	1.00	0.00
ATOM	693	H	GLU	45	-15.335	8.355	-5.168	1.00	0.00
ATOM	694	CA	GLU	45	-15.876	6.339	-4.803	1.00	0.00
ATOM	695	HA	GLU	45	-16.872	5.900	-4.852	1.00	0.00
ATOM	696	CB	GLU	45	-15.117	5.978	-6.089	1.00	0.00
ATOM	697	HB2	GLU	45	-14.590	6.861	-6.451	1.00	0.00
ATOM	698	HB3	GLU	45	-14.397	5.191	-5.869	1.00	0.00
ATOM	699	CG	GLU	45	-16.098	5.488	-7.169	1.00	0.00
ATOM	700	HG2	GLU	45	-16.343	4.427	-7.190	1.00	0.00
ATOM	701	HG3	GLU	45	-16.973	6.057	-6.855	1.00	0.00
ATOM	702	CD	GLU	45	-15.672	5.940	-8.567	1.00	0.00
ATOM	703	OE1	GLU	45	-15.759	7.157	-8.839	1.00	0.00

ATOM	704	OE2	GLU	45	-15.045	5.105	-9.262	1.00	0.00
ATOM	705	C	GLU	45	-15.177	5.797	-3.567	1.00	0.00
ATOM	706	O	GLU	45	-13.999	6.101	-3.306	1.00	0.00
ATOM	707	N	ASP	46	-15.894	4.964	-2.821	1.00	0.00
ATOM	708	H	ASP	46	-16.839	4.741	-3.098	1.00	0.00
ATOM	709	CA	ASP	46	-15.371	4.361	-1.614	1.00	0.00
ATOM	710	HA	ASP	46	-14.970	5.137	-0.962	1.00	0.00
ATOM	711	CB	ASP	46	-16.472	3.608	-0.861	1.00	0.00
ATOM	712	HB2	ASP	46	-17.347	4.241	-1.008	1.00	0.00
ATOM	713	HB3	ASP	46	-16.660	2.624	-1.290	1.00	0.00
ATOM	714	CG	ASP	46	-16.190	3.467	0.638	1.00	0.00
ATOM	715	OD1	ASP	46	-15.163	4.022	1.107	1.00	0.00
ATOM	716	OD2	ASP	46	-16.936	2.717	1.298	1.00	0.00
ATOM	717	C	ASP	46	-14.261	3.384	-1.987	1.00	0.00
ATOM	718	O	ASP	46	-14.326	2.643	-2.980	1.00	0.00
ATOM	719	N	HID	47	-13.183	3.365	-1.215	1.00	0.00
ATOM	720	H	HID	47	-13.101	4.025	-0.455	1.00	0.00
ATOM	721	CA	HID	47	-12.072	2.461	-1.465	1.00	0.00
ATOM	722	HA	HID	47	-12.311	1.790	-2.290	1.00	0.00
ATOM	723	CB	HID	47	-10.830	3.263	-1.835	1.00	0.00
ATOM	724	HB2	HID	47	-10.652	4.065	-1.118	1.00	0.00
ATOM	725	HB3	HID	47	-9.953	2.618	-1.884	1.00	0.00

ATOM	726	CG	HID	47	-10.960	3.918	-3.192	1.00	0.00
ATOM	727	ND1	HID	47	-11.939	3.618	-4.131	1.00	0.00
ATOM	728	HD1	HID	47	-12.662	2.920	-4.034	1.00	0.00
ATOM	729	CE1	HID	47	-11.754	4.422	-5.195	1.00	0.00
ATOM	730	HE1	HID	47	-12.436	4.376	-6.045	1.00	0.00
ATOM	731	NE2	HID	47	-10.660	5.194	-4.973	1.00	0.00
ATOM	732	CD2	HID	47	-10.150	4.895	-3.718	1.00	0.00
ATOM	733	HD2	HID	47	-9.267	5.418	-3.350	1.00	0.00
ATOM	734	C	HID	47	-11.854	1.613	-0.248	1.00	0.00
ATOM	735	O	HID	47	-11.631	2.168	0.818	1.00	0.00
ATOM	736	N	LYS	48	-11.838	0.292	-0.448	1.00	0.00
ATOM	737	H	LYS	48	-12.019	-0.044	-1.383	1.00	0.00
ATOM	738	CA	LYS	48	-11.631	-0.687	0.611	1.00	0.00
ATOM	739	HA	LYS	48	-10.598	-0.648	0.958	1.00	0.00
ATOM	740	CB	LYS	48	-12.556	-0.386	1.793	1.00	0.00
ATOM	741	HB2	LYS	48	-13.132	0.513	1.571	1.00	0.00
ATOM	742	HB3	LYS	48	-13.235	-1.227	1.933	1.00	0.00
ATOM	743	CG	LYS	48	-11.741	-0.167	3.074	1.00	0.00
ATOM	744	HG2	LYS	48	-11.090	-1.028	3.224	1.00	0.00
ATOM	745	HG3	LYS	48	-11.133	0.730	2.950	1.00	0.00
ATOM	746	CD	LYS	48	-12.657	0.000	4.293	1.00	0.00
ATOM	747	HD2	LYS	48	-12.069	0.390	5.124	1.00	0.00

ATOM	748	HD3	LYS	48	-13.448	0.708	4.044	1.00	0.00
ATOM	749	CE	LYS	48	-13.281	-1.346	4.694	1.00	0.00
ATOM	750	HE2	LYS	48	-12.543	-2.134	4.542	1.00	0.00
ATOM	751	HE3	LYS	48	-13.557	-1.305	5.747	1.00	0.00
ATOM	752	NZ	LYS	48	-14.488	-1.634	3.877	1.00	0.00
ATOM	753	HZ1	LYS	48	-14.232	-1.672	2.900	1.00	0.00
ATOM	754	HZ2	LYS	48	-14.880	-2.521	4.157	1.00	0.00
ATOM	755	HZ3	LYS	48	-15.172	-0.904	4.017	1.00	0.00
ATOM	756	C	LYS	48	-11.863	-2.097	0.096	1.00	0.00
ATOM	757	O	LYS	48	-11.154	-3.020	0.457	1.00	0.00
ATOM	758	N	GLN	49	-12.783	-2.233	-0.854	1.00	0.00
ATOM	759	H	GLN	49	-13.299	-1.424	-1.173	1.00	0.00
ATOM	760	CA	GLN	49	-13.063	-3.503	-1.464	1.00	0.00
ATOM	761	HA	GLN	49	-12.859	-4.299	-0.749	1.00	0.00
ATOM	762	CB	GLN	49	-14.517	-3.602	-1.901	1.00	0.00
ATOM	763	HB2	GLN	49	-14.972	-2.611	-1.876	1.00	0.00
ATOM	764	HB3	GLN	49	-14.565	-3.997	-2.916	1.00	0.00
ATOM	765	CG	GLN	49	-15.273	-4.533	-0.956	1.00	0.00
ATOM	766	HG2	GLN	49	-14.790	-5.509	-0.986	1.00	0.00
ATOM	767	HG3	GLN	49	-15.236	-4.140	0.060	1.00	0.00
ATOM	768	CD	GLN	49	-16.729	-4.698	-1.360	1.00	0.00
ATOM	769	OE1	GLN	49	-17.169	-4.287	-2.414	1.00	0.00

ATOM	770	NE2	GLN	49	-17.511	-5.296	-0.492	1.00	0.00
ATOM	771	HE21	GLN	49	-18.488	-5.432	-0.708	1.00	0.00
ATOM	772	HE22	GLN	49	-17.132	-5.616	0.387	1.00	0.00
ATOM	773	C	GLN	49	-12.200	-3.661	-2.677	1.00	0.00
ATOM	774	O	GLN	49	-11.850	-4.773	-2.931	1.00	0.00
ATOM	775	N	LEU	50	-11.732	-2.611	-3.345	1.00	0.00
ATOM	776	H	LEU	50	-12.050	-1.695	-3.062	1.00	0.00
ATOM	777	CA	LEU	50	-10.837	-2.706	-4.486	1.00	0.00
ATOM	778	HA	LEU	50	-11.251	-3.364	-5.250	1.00	0.00
ATOM	779	CB	LEU	50	-10.666	-1.301	-5.088	1.00	0.00
ATOM	780	HB2	LEU	50	-10.779	-0.608	-4.254	1.00	0.00
ATOM	781	HB3	LEU	50	-9.664	-1.203	-5.508	1.00	0.00
ATOM	782	CG	LEU	50	-11.709	-0.974	-6.171	1.00	0.00
ATOM	783	HG	LEU	50	-12.648	-1.474	-5.934	1.00	0.00
ATOM	784	CD1	LEU	50	-11.937	0.541	-6.224	1.00	0.00
ATOM	785	HD11	LEU	50	-10.999	1.042	-6.462	1.00	0.00
ATOM	786	HD12	LEU	50	-12.676	0.772	-6.992	1.00	0.00
ATOM	787	HD13	LEU	50	-12.299	0.889	-5.257	1.00	0.00
ATOM	788	CD2	LEU	50	-11.259	-1.503	-7.535	1.00	0.00
ATOM	789	HD21	LEU	50	-11.134	-2.584	-7.483	1.00	0.00
ATOM	790	HD22	LEU	50	-12.011	-1.260	-8.286	1.00	0.00
ATOM	791	HD23	LEU	50	-10.311	-1.041	-7.810	1.00	0.00

ATOM	792	C	LEU	50	-9.498	-3.306	-4.054	1.00	0.00
ATOM	793	O	LEU	50	-8.814	-3.900	-4.866	1.00	0.00
ATOM	794	N	CYX	51	-9.159	-3.203	-2.766	1.00	0.00
ATOM	795	H	CYX	51	-9.734	-2.642	-2.154	1.00	0.00
ATOM	796	CA	CYX	51	-7.972	-3.813	-2.221	1.00	0.00
ATOM	797	HA	CYX	51	-7.199	-3.874	-2.987	1.00	0.00
ATOM	798	CB	CYX	51	-7.446	-2.986	-1.068	1.00	0.00
ATOM	799	HB2	CYX	51	-6.360	-3.067	-1.028	1.00	0.00
ATOM	800	HB3	CYX	51	-7.726	-1.942	-1.212	1.00	0.00
ATOM	801	SG	CYX	51	-8.002	-3.369	0.624	1.00	0.00
ATOM	802	C	CYX	51	-8.265	-5.221	-1.783	1.00	0.00
ATOM	803	O	CYX	51	-7.557	-6.100	-2.230	1.00	0.00
ATOM	804	N	CYX	52	-9.371	-5.444	-1.061	1.00	0.00
ATOM	805	H	CYX	52	-9.907	-4.651	-0.738	1.00	0.00
ATOM	806	CA	CYX	52	-9.780	-6.773	-0.637	1.00	0.00
ATOM	807	HA	CYX	52	-8.919	-7.323	-0.257	1.00	0.00
ATOM	808	CB	CYX	52	-10.831	-6.674	0.477	1.00	0.00
ATOM	809	HB2	CYX	52	-11.421	-5.767	0.341	1.00	0.00
ATOM	810	HB3	CYX	52	-11.487	-7.543	0.436	1.00	0.00
ATOM	811	SG	CYX	52	-10.083	-6.657	2.138	1.00	0.00
ATOM	812	C	CYX	52	-10.276	-7.584	-1.839	1.00	0.00
ATOM	813	O	CYX	52	-10.610	-8.751	-1.697	1.00	0.00

ATOM	814	N	MET	53	-10.335	-6.971	-3.020	1.00	0.00
ATOM	815	H	MET	53	-10.012	-6.014	-3.056	1.00	0.00
ATOM	816	CA	MET	53	-10.836	-7.567	-4.229	1.00	0.00
ATOM	817	HA	MET	53	-11.494	-8.398	-3.979	1.00	0.00
ATOM	818	CB	MET	53	-11.626	-6.551	-5.061	1.00	0.00
ATOM	819	HB2	MET	53	-12.587	-6.378	-4.577	1.00	0.00
ATOM	820	HB3	MET	53	-11.063	-5.617	-5.094	1.00	0.00
ATOM	821	CG	MET	53	-11.856	-7.052	-6.476	1.00	0.00
ATOM	822	HG2	MET	53	-11.617	-8.114	-6.524	1.00	0.00
ATOM	823	HG3	MET	53	-12.902	-6.902	-6.744	1.00	0.00
ATOM	824	SD	MET	53	-10.810	-6.156	-7.646	1.00	0.00
ATOM	825	CE	MET	53	-10.486	-7.496	-8.811	1.00	0.00
ATOM	826	HE1	MET	53	-11.428	-7.851	-9.228	1.00	0.00
ATOM	827	HE2	MET	53	-9.847	-7.131	-9.616	1.00	0.00
ATOM	828	HE3	MET	53	-9.986	-8.315	-8.294	1.00	0.00
ATOM	829	C	MET	53	-9.646	-8.072	-5.008	1.00	0.00
ATOM	830	O	MET	53	-9.634	-9.226	-5.402	1.00	0.00
ATOM	831	N	GLN	54	-8.683	-7.196	-5.286	1.00	0.00
ATOM	832	H	GLN	54	-8.739	-6.242	-4.958	1.00	0.00
ATOM	833	CA	GLN	54	-7.524	-7.588	-6.058	1.00	0.00
ATOM	834	HA	GLN	54	-7.840	-8.126	-6.952	1.00	0.00
ATOM	835	CB	GLN	54	-6.734	-6.371	-6.482	1.00	0.00

ATOM	836	HB2	GLN	54	-6.821	-5.620	-5.697	1.00	0.00
ATOM	837	HB3	GLN	54	-5.690	-6.665	-6.588	1.00	0.00
ATOM	838	CG	GLN	54	-7.230	-5.791	-7.792	1.00	0.00
ATOM	839	HG2	GLN	54	-6.482	-5.978	-8.563	1.00	0.00
ATOM	840	HG3	GLN	54	-8.155	-6.306	-8.051	1.00	0.00
ATOM	841	CD	GLN	54	-7.506	-4.296	-7.736	1.00	0.00
ATOM	842	OE1	GLN	54	-6.814	-3.485	-7.161	1.00	0.00
ATOM	843	NE2	GLN	54	-8.558	-3.852	-8.365	1.00	0.00
ATOM	844	HE21	GLN	54	-8.773	-2.865	-8.350	1.00	0.00
ATOM	845	HE22	GLN	54	-9.153	-4.496	-8.865	1.00	0.00
ATOM	846	C	GLN	54	-6.623	-8.470	-5.233	1.00	0.00
ATOM	847	O	GLN	54	-6.009	-9.352	-5.802	1.00	0.00
ATOM	848	N	LEU	55	-6.579	-8.296	-3.910	1.00	0.00
ATOM	849	H	LEU	55	-7.133	-7.566	-3.485	1.00	0.00
ATOM	850	CA	LEU	55	-5.745	-9.119	-3.050	1.00	0.00
ATOM	851	HA	LEU	55	-4.691	-8.986	-3.297	1.00	0.00
ATOM	852	CB	LEU	55	-5.962	-8.699	-1.606	1.00	0.00
ATOM	853	HB2	LEU	55	-6.935	-8.210	-1.645	1.00	0.00
ATOM	854	HB3	LEU	55	-6.023	-9.579	-0.966	1.00	0.00
ATOM	855	CG	LEU	55	-4.916	-7.718	-1.033	1.00	0.00
ATOM	856	HG	LEU	55	-4.366	-7.293	-1.874	1.00	0.00
ATOM	857	CD1	LEU	55	-5.541	-6.583	-0.204	1.00	0.00

ATOM	858	HD11	LEU	55	-6.090	-7.006	0.636	1.00	0.00
ATOM	859	HD12	LEU	55	-4.753	-5.929	0.169	1.00	0.00
ATOM	860	HD13	LEU	55	-6.223	-6.008	-0.831	1.00	0.00
ATOM	861	CD2	LEU	55	-3.858	-8.461	-0.212	1.00	0.00
ATOM	862	HD21	LEU	55	-3.347	-9.184	-0.847	1.00	0.00
ATOM	863	HD22	LEU	55	-3.134	-7.746	0.179	1.00	0.00
ATOM	864	HD23	LEU	55	-4.339	-8.981	0.616	1.00	0.00
ATOM	865	C	LEU	55	-6.120	-10.581	-3.236	1.00	0.00
ATOM	866	O	LEU	55	-5.271	-11.437	-3.090	1.00	0.00
ATOM	867	N	LYS	56	-7.369	-10.859	-3.611	1.00	0.00
ATOM	868	H	LYS	56	-8.075	-10.137	-3.572	1.00	0.00
ATOM	869	CA	LYS	56	-7.777	-12.197	-3.973	1.00	0.00
ATOM	870	HA	LYS	56	-7.459	-12.914	-3.217	1.00	0.00
ATOM	871	CB	LYS	56	-9.300	-12.234	-4.071	1.00	0.00
ATOM	872	HB2	LYS	56	-9.722	-11.986	-3.098	1.00	0.00
ATOM	873	HB3	LYS	56	-9.624	-11.496	-4.806	1.00	0.00
ATOM	874	CG	LYS	56	-9.782	-13.624	-4.497	1.00	0.00
ATOM	875	HG2	LYS	56	-10.085	-13.586	-5.544	1.00	0.00
ATOM	876	HG3	LYS	56	-8.961	-14.332	-4.382	1.00	0.00
ATOM	877	CD	LYS	56	-10.966	-14.073	-3.640	1.00	0.00
ATOM	878	HD2	LYS	56	-10.590	-14.538	-2.730	1.00	0.00
ATOM	879	HD3	LYS	56	-11.568	-13.202	-3.381	1.00	0.00

ATOM	880	CE	LYS	56	-11.829	-15.079	-4.409	1.00	0.00
ATOM	881	HE2	LYS	56	-11.782	-14.851	-5.474	1.00	0.00
ATOM	882	HE3	LYS	56	-11.447	-16.085	-4.236	1.00	0.00
ATOM	883	NZ	LYS	56	-13.236	-14.999	-3.949	1.00	0.00
ATOM	884	HZ1	LYS	56	-13.590	-14.067	-4.110	1.00	0.00
ATOM	885	HZ2	LYS	56	-13.796	-15.665	-4.462	1.00	0.00
ATOM	886	HZ3	LYS	56	-13.280	-15.210	-2.963	1.00	0.00
ATOM	887	C	LYS	56	-7.099	-12.686	-5.250	1.00	0.00
ATOM	888	O	LYS	56	-6.713	-13.841	-5.302	1.00	0.00
ATOM	889	N	ASN	57	-7.063	-11.849	-6.287	1.00	0.00
ATOM	890	H	ASN	57	-7.587	-10.986	-6.256	1.00	0.00
ATOM	891	CA	ASN	57	-6.319	-12.157	-7.491	1.00	0.00
ATOM	892	HA	ASN	57	-6.752	-13.029	-7.980	1.00	0.00
ATOM	893	CB	ASN	57	-6.365	-10.969	-8.473	1.00	0.00
ATOM	894	HB2	ASN	57	-6.669	-10.049	-7.974	1.00	0.00
ATOM	895	HB3	ASN	57	-5.348	-10.858	-8.849	1.00	0.00
ATOM	896	CG	ASN	57	-7.288	-11.210	-9.643	1.00	0.00
ATOM	897	OD1	ASN	57	-7.701	-12.307	-9.969	1.00	0.00
ATOM	898	ND2	ASN	57	-7.704	-10.139	-10.279	1.00	0.00
ATOM	899	HD21	ASN	57	-8.324	-10.234	-11.071	1.00	0.00
ATOM	900	HD22	ASN	57	-7.402	-9.224	-9.974	1.00	0.00
ATOM	901	C	ASN	57	-4.856	-12.506	-7.216	1.00	0.00

ATOM	902	O	ASN	57	-4.255	-13.200	-8.028	1.00	0.00
ATOM	903	N	LEU	58	-4.279	-11.893	-6.176	1.00	0.00
ATOM	904	H	LEU	58	-4.760	-11.134	-5.714	1.00	0.00
ATOM	905	CA	LEU	58	-2.929	-12.196	-5.767	1.00	0.00
ATOM	906	HA	LEU	58	-2.252	-12.139	-6.620	1.00	0.00
ATOM	907	CB	LEU	58	-2.475	-11.174	-4.711	1.00	0.00
ATOM	908	HB2	LEU	58	-3.366	-10.913	-4.140	1.00	0.00
ATOM	909	HB3	LEU	58	-1.757	-11.670	-4.058	1.00	0.00
ATOM	910	CG	LEU	58	-1.852	-9.912	-5.280	1.00	0.00
ATOM	911	HG	LEU	58	-2.606	-9.363	-5.845	1.00	0.00
ATOM	912	CD1	LEU	58	-1.321	-9.028	-4.145	1.00	0.00
ATOM	913	HD11	LEU	58	-0.566	-9.575	-3.580	1.00	0.00
ATOM	914	HD12	LEU	58	-0.876	-8.125	-4.565	1.00	0.00
ATOM	915	HD13	LEU	58	-2.142	-8.753	-3.483	1.00	0.00
ATOM	916	CD2	LEU	58	-0.728	-10.257	-6.212	1.00	0.00
ATOM	917	HD21	LEU	58	-1.109	-10.865	-7.033	1.00	0.00
ATOM	918	HD22	LEU	58	-0.292	-9.341	-6.612	1.00	0.00
ATOM	919	HD23	LEU	58	0.035	-10.815	-5.671	1.00	0.00
ATOM	920	C	LEU	58	-2.795	-13.650	-5.295	1.00	0.00
ATOM	921	O	LEU	58	-3.705	-14.469	-5.391	1.00	0.00
ATOM	922	N	ASP	59	-1.620	-13.980	-4.782	1.00	0.00
ATOM	923	H	ASP	59	-0.874	-13.300	-4.731	1.00	0.00

ATOM	924	CA	ASP	59	-1.368	-15.311	-4.286	1.00	0.00
ATOM	925	HA	ASP	59	-2.045	-16.019	-4.762	1.00	0.00
ATOM	926	CB	ASP	59	0.067	-15.725	-4.596	1.00	0.00
ATOM	927	HB2	ASP	59	0.608	-14.964	-4.034	1.00	0.00
ATOM	928	HB3	ASP	59	0.348	-16.713	-4.233	1.00	0.00
ATOM	929	CG	ASP	59	0.418	-15.609	-6.076	1.00	0.00
ATOM	930	OD1	ASP	59	-0.343	-16.157	-6.892	1.00	0.00
ATOM	931	OD2	ASP	59	1.541	-15.129	-6.335	1.00	0.00
ATOM	932	C	ASP	59	-1.582	-15.338	-2.786	1.00	0.00
ATOM	933	O	ASP	59	-1.252	-14.374	-2.092	1.00	0.00
ATOM	934	N	GLU	60	-1.824	-16.529	-2.243	1.00	0.00
ATOM	935	H	GLU	60	-1.893	-17.345	-2.834	1.00	0.00
ATOM	936	CA	GLU	60	-2.022	-16.696	-0.803	1.00	0.00
ATOM	937	HA	GLU	60	-2.685	-15.916	-0.429	1.00	0.00
ATOM	938	CB	GLU	60	-2.652	-18.062	-0.502	1.00	0.00
ATOM	939	HB2	GLU	60	-3.005	-18.505	-1.433	1.00	0.00
ATOM	940	HB3	GLU	60	-1.900	-18.711	-0.053	1.00	0.00
ATOM	941	CG	GLU	60	-3.831	-17.899	0.466	1.00	0.00
ATOM	942	HG2	GLU	60	-3.549	-17.128	1.184	1.00	0.00
ATOM	943	HG3	GLU	60	-4.726	-17.584	-0.071	1.00	0.00
ATOM	944	CD	GLU	60	-4.114	-19.210	1.208	1.00	0.00
ATOM	945	OE1	GLU	60	-3.501	-19.402	2.283	1.00	0.00

ATOM	946	OE2	GLU	60	-4.903	-20.015	0.669	1.00	0.00
ATOM	947	C	GLU	60	-0.688	-16.577	-0.066	1.00	0.00
ATOM	948	O	GLU	60	-0.625	-16.232	1.109	1.00	0.00
ATOM	949	N	LYS	61	0.412	-16.822	-0.786	1.00	0.00
ATOM	950	H	LYS	61	0.318	-17.199	-1.718	1.00	0.00
ATOM	951	CA	LYS	61	1.737	-16.653	-0.248	1.00	0.00
ATOM	952	HA	LYS	61	1.726	-16.771	0.835	1.00	0.00
ATOM	953	CB	LYS	61	2.663	-17.713	-0.846	1.00	0.00
ATOM	954	HB2	LYS	61	3.569	-17.754	-0.241	1.00	0.00
ATOM	955	HB3	LYS	61	2.153	-18.675	-0.802	1.00	0.00
ATOM	956	CG	LYS	61	3.035	-17.398	-2.301	1.00	0.00
ATOM	957	HG2	LYS	61	2.123	-17.189	-2.861	1.00	0.00
ATOM	958	HG3	LYS	61	3.680	-16.519	-2.318	1.00	0.00
ATOM	959	CD	LYS	61	3.768	-18.580	-2.943	1.00	0.00
ATOM	960	HD2	LYS	61	4.696	-18.221	-3.389	1.00	0.00
ATOM	961	HD3	LYS	61	3.996	-19.316	-2.173	1.00	0.00
ATOM	962	CE	LYS	61	2.897	-19.228	-4.027	1.00	0.00
ATOM	963	HE2	LYS	61	2.240	-18.469	-4.453	1.00	0.00
ATOM	964	HE3	LYS	61	3.544	-19.627	-4.809	1.00	0.00
ATOM	965	NZ	LYS	61	2.079	-20.325	-3.453	1.00	0.00
ATOM	966	HZ1	LYS	61	1.480	-19.954	-2.730	1.00	0.00
ATOM	967	HZ2	LYS	61	1.513	-20.738	-4.181	1.00	0.00

ATOM 968 HZ3 LYS 61 2.688 -21.028 -3.059 1.00 0.00
ATOM 969 C LYS 61 2.224 -15.249 -0.496 1.00 0.00
ATOM 970 O LYS 61 3.290 -14.956 -0.010 1.00 0.00
ATOM 971 N CYX 62 1.542 -14.435 -1.295 1.00 0.00
ATOM 972 H CYX 62 0.758 -14.829 -1.795 1.00 0.00
ATOM 973 CA CYX 62 1.874 -13.048 -1.527 1.00 0.00
ATOM 974 HA CYX 62 2.903 -12.846 -1.231 1.00 0.00
ATOM 975 CB CYX 62 1.728 -12.746 -3.004 1.00 0.00
ATOM 976 HB2 CYX 62 0.777 -13.139 -3.364 1.00 0.00
ATOM 977 HB3 CYX 62 1.757 -11.667 -3.160 1.00 0.00
ATOM 978 SG CYX 62 3.022 -13.494 -4.031 1.00 0.00
ATOM 979 C CYX 62 0.965 -12.154 -0.681 1.00 0.00
ATOM 980 O CYX 62 1.277 -11.002 -0.408 1.00 0.00
ATOM 981 N MET 63 -0.089 -12.692 -0.102 1.00 0.00
ATOM 982 H MET 63 -0.284 -13.671 -0.255 1.00 0.00
ATOM 983 CA MET 63 -1.000 -11.934 0.711 1.00 0.00
ATOM 984 HA MET 63 -1.091 -10.913 0.340 1.00 0.00
ATOM 985 CB MET 63 -2.358 -12.593 0.651 1.00 0.00
ATOM 986 HB2 MET 63 -2.216 -13.665 0.787 1.00 0.00
ATOM 987 HB3 MET 63 -2.957 -12.199 1.471 1.00 0.00
ATOM 988 CG MET 63 -3.074 -12.341 -0.664 1.00 0.00
ATOM 989 HG2 MET 63 -3.717 -11.470 -0.538 1.00 0.00

ATOM 990 HG3 MET 63 -2.322 -12.129 -1.425 1.00 0.00

ATOM 991 SD MET 63 -4.074 -13.746 -1.194 1.00 0.00

ATOM 992 CE MET 63 -4.932 -14.124 0.337 1.00 0.00

ATOM 993 HE1 MET 63 -5.517 -13.259 0.649 1.00 0.00

ATOM 994 HE2 MET 63 -5.596 -14.974 0.181 1.00 0.00

ATOM 995 HE3 MET 63 -4.204 -14.368 1.111 1.00 0.00

ATOM 996 C MET 63 -0.464 -11.882 2.117 1.00 0.00

ATOM 997 O MET 63 -1.042 -12.392 3.034 1.00 0.00

ATOM 998 N CYX 64 0.680 -11.295 2.360 1.00 0.00

ATOM 999 H CYX 64 1.177 -10.912 1.567 1.00 0.00

ATOM 1000 CA CYX 64 1.310 -11.222 3.670 1.00 0.00

ATOM 1001 HA CYX 64 0.736 -10.530 4.286 1.00 0.00

ATOM 1002 CB CYX 64 1.386 -12.581 4.394 1.00 0.00

ATOM 1003 HB2 CYX 64 0.569 -12.657 5.111 1.00 0.00

ATOM 1004 HB3 CYX 64 1.304 -13.386 3.664 1.00 0.00

ATOM 1005 SG CYX 64 2.928 -12.895 5.346 1.00 0.00

ATOM 1006 C CYX 64 2.684 -10.627 3.487 1.00 0.00

ATOM 1007 O CYX 64 2.946 -9.551 4.026 1.00 0.00

ATOM 1008 N PRO 65 3.535 -11.293 2.685 1.00 0.00

ATOM 1009 CD PRO 65 3.276 -12.542 1.981 1.00 0.00

ATOM 1010 HD2 PRO 65 2.537 -12.335 1.207 1.00 0.00

ATOM 1011 HD3 PRO 65 2.860 -13.245 2.702 1.00 0.00

ATOM	1012	CG	PRO	65	4.597	-13.012	1.412	1.00	0.00
ATOM	1013	HG2	PRO	65	4.529	-13.023	0.324	1.00	0.00
ATOM	1014	HG3	PRO	65	4.798	-14.020	1.773	1.00	0.00
ATOM	1015	CB	PRO	65	5.659	-12.046	1.889	1.00	0.00
ATOM	1016	HB2	PRO	65	6.260	-11.740	1.032	1.00	0.00
ATOM	1017	HB3	PRO	65	6.309	-12.463	2.658	1.00	0.00
ATOM	1018	CA	PRO	65	4.895	-10.837	2.448	1.00	0.00
ATOM	1019	HA	PRO	65	5.331	-10.517	3.394	1.00	0.00
ATOM	1020	C	PRO	65	4.906	-9.672	1.465	1.00	0.00
ATOM	1021	O	PRO	65	5.711	-8.762	1.605	1.00	0.00
ATOM	1022	N	ALA	66	3.994	-9.690	0.493	1.00	0.00
ATOM	1023	H	ALA	66	3.369	-10.479	0.412	1.00	0.00
ATOM	1024	CA	ALA	66	3.880	-8.635	-0.464	1.00	0.00
ATOM	1025	HA	ALA	66	4.850	-8.361	-0.881	1.00	0.00
ATOM	1026	CB	ALA	66	2.969	-9.058	-1.596	1.00	0.00
ATOM	1027	HB1	ALA	66	1.992	-9.326	-1.196	1.00	0.00
ATOM	1028	HB2	ALA	66	2.859	-8.234	-2.302	1.00	0.00
ATOM	1029	HB3	ALA	66	3.400	-9.918	-2.108	1.00	0.00
ATOM	1030	C	ALA	66	3.333	-7.422	0.228	1.00	0.00
ATOM	1031	O	ALA	66	3.938	-6.391	0.081	1.00	0.00
ATOM	1032	N	ILE	67	2.251	-7.524	0.995	1.00	0.00
ATOM	1033	H	ILE	67	1.819	-8.432	1.084	1.00	0.00

ATOM	1034	CA	ILE	67	1.652	-6.405	1.701	1.00	0.00
ATOM	1035	HA	ILE	67	1.072	-5.740	1.060	1.00	0.00
ATOM	1036	CB	ILE	67	0.774	-6.908	2.838	1.00	0.00
ATOM	1037	HB	ILE	67	1.313	-7.677	3.389	1.00	0.00
ATOM	1038	CG2	ILE	67	0.414	-5.751	3.790	1.00	0.00
ATOM	1039	HG21	ILE	67	-0.125	-4.980	3.238	1.00	0.00
ATOM	1040	HG22	ILE	67	-0.214	-6.126	4.597	1.00	0.00
ATOM	1041	HG23	ILE	67	1.327	-5.326	4.207	1.00	0.00
ATOM	1042	CG1	ILE	67	-0.456	-7.638	2.265	1.00	0.00
ATOM	1043	HG12	ILE	67	-0.526	-7.401	1.203	1.00	0.00
ATOM	1044	HG13	ILE	67	-0.302	-8.710	2.388	1.00	0.00
ATOM	1045	CD1	ILE	67	-1.748	-7.227	2.964	1.00	0.00
ATOM	1046	HD11	ILE	67	-1.903	-6.155	2.840	1.00	0.00
ATOM	1047	HD12	ILE	67	-2.587	-7.767	2.525	1.00	0.00
ATOM	1048	HD13	ILE	67	-1.678	-7.463	4.025	1.00	0.00
ATOM	1049	C	ILE	67	2.739	-5.507	2.269	1.00	0.00
ATOM	1050	O	ILE	67	2.769	-4.339	1.999	1.00	0.00
ATOM	1051	N	MET	68	3.691	-6.010	3.022	1.00	0.00
ATOM	1052	H	MET	68	3.662	-6.990	3.264	1.00	0.00
ATOM	1053	CA	MET	68	4.790	-5.206	3.534	1.00	0.00
ATOM	1054	HA	MET	68	4.451	-4.556	4.341	1.00	0.00
ATOM	1055	CB	MET	68	5.852	-6.136	4.076	1.00	0.00

ATOM	1056	HB2	MET	68	6.176	-6.798	3.273	1.00	0.00
ATOM	1057	HB3	MET	68	6.697	-5.537	4.416	1.00	0.00
ATOM	1058	CG	MET	68	5.320	-6.968	5.236	1.00	0.00
ATOM	1059	HG2	MET	68	5.529	-6.451	6.172	1.00	0.00
ATOM	1060	HG3	MET	68	4.243	-7.093	5.122	1.00	0.00
ATOM	1061	SD	MET	68	6.108	-8.596	5.266	1.00	0.00
ATOM	1062	CE	MET	68	4.939	-9.459	6.334	1.00	0.00
ATOM	1063	HE1	MET	68	3.950	-9.446	5.875	1.00	0.00
ATOM	1064	HE2	MET	68	5.263	-10.490	6.469	1.00	0.00
ATOM	1065	HE3	MET	68	4.896	-8.962	7.303	1.00	0.00
ATOM	1066	C	MET	68	5.414	-4.321	2.471	1.00	0.00
ATOM	1067	O	MET	68	5.303	-3.103	2.617	1.00	0.00
ATOM	1068	N	MET	69	5.868	-4.942	1.369	1.00	0.00
ATOM	1069	H	MET	69	5.897	-5.951	1.362	1.00	0.00
ATOM	1070	CA	MET	69	6.370	-4.235	0.213	1.00	0.00
ATOM	1071	HA	MET	69	7.244	-3.642	0.482	1.00	0.00
ATOM	1072	CB	MET	69	6.773	-5.236	-0.858	1.00	0.00
ATOM	1073	HB2	MET	69	6.043	-6.045	-0.864	1.00	0.00
ATOM	1074	HB3	MET	69	6.761	-4.729	-1.824	1.00	0.00
ATOM	1075	CG	MET	69	8.153	-5.803	-0.603	1.00	0.00
ATOM	1076	HG2	MET	69	8.191	-6.221	0.402	1.00	0.00
ATOM	1077	HG3	MET	69	8.360	-6.588	-1.331	1.00	0.00

ATOM	1078	SD	MET	69	9.405	-4.506	-0.755	1.00	0.00
ATOM	1079	CE	MET	69	10.823	-5.393	-0.106	1.00	0.00
ATOM	1080	HE1	MET	69	11.014	-6.273	-0.720	1.00	0.00
ATOM	1081	HE2	MET	69	11.698	-4.742	-0.123	1.00	0.00
ATOM	1082	HE3	MET	69	10.621	-5.702	0.919	1.00	0.00
ATOM	1083	C	MET	69	5.390	-3.231	-0.388	1.00	0.00
ATOM	1084	O	MET	69	5.791	-2.203	-0.925	1.00	0.00
ATOM	1085	N	MET	70	4.099	-3.502	-0.296	1.00	0.00
ATOM	1086	H	MET	70	3.827	-4.405	0.063	1.00	0.00
ATOM	1087	CA	MET	70	3.068	-2.605	-0.731	1.00	0.00
ATOM	1088	HA	MET	70	3.114	-2.459	-1.811	1.00	0.00
ATOM	1089	CB	MET	70	1.719	-3.210	-0.379	1.00	0.00
ATOM	1090	HB2	MET	70	1.100	-3.133	-1.273	1.00	0.00
ATOM	1091	HB3	MET	70	1.898	-4.260	-0.151	1.00	0.00
ATOM	1092	CG	MET	70	1.002	-2.551	0.793	1.00	0.00
ATOM	1093	HG2	MET	70	1.758	-2.371	1.557	1.00	0.00
ATOM	1094	HG3	MET	70	0.622	-1.597	0.427	1.00	0.00
ATOM	1095	SD	MET	70	-0.349	-3.490	1.508	1.00	0.00
ATOM	1096	CE	MET	70	-1.255	-2.178	2.294	1.00	0.00
ATOM	1097	HE1	MET	70	-1.569	-1.452	1.543	1.00	0.00
ATOM	1098	HE2	MET	70	-2.134	-2.589	2.790	1.00	0.00
ATOM	1099	HE3	MET	70	-0.619	-1.686	3.030	1.00	0.00

ATOM	1100	C	MET	70	3.219	-1.229	-0.116	1.00	0.00
ATOM	1101	O	MET	70	2.883	-0.242	-0.752	1.00	0.00
ATOM	1102	N	LEU	71	3.645	-1.179	1.149	1.00	0.00
ATOM	1103	H	LEU	71	3.790	-2.050	1.639	1.00	0.00
ATOM	1104	CA	LEU	71	3.797	0.055	1.885	1.00	0.00
ATOM	1105	HA	LEU	71	3.340	0.873	1.328	1.00	0.00
ATOM	1106	CB	LEU	71	3.122	-0.042	3.253	1.00	0.00
ATOM	1107	HB2	LEU	71	3.595	-0.914	3.704	1.00	0.00
ATOM	1108	HB3	LEU	71	3.354	0.844	3.844	1.00	0.00
ATOM	1109	CG	LEU	71	1.616	-0.246	3.215	1.00	0.00
ATOM	1110	HG	LEU	71	1.399	-1.286	2.972	1.00	0.00
ATOM	1111	CD1	LEU	71	1.023	0.090	4.556	1.00	0.00
ATOM	1112	HD11	LEU	71	1.240	1.131	4.799	1.00	0.00
ATOM	1113	HD12	LEU	71	-0.056	-0.057	4.523	1.00	0.00
ATOM	1114	HD13	LEU	71	1.456	-0.558	5.317	1.00	0.00
ATOM	1115	CD2	LEU	71	0.918	0.541	2.112	1.00	0.00
ATOM	1116	HD21	LEU	71	1.311	0.236	1.142	1.00	0.00
ATOM	1117	HD22	LEU	71	-0.154	0.345	2.147	1.00	0.00
ATOM	1118	HD23	LEU	71	1.097	1.607	2.256	1.00	0.00
ATOM	1119	C	LEU	71	5.273	0.381	1.946	1.00	0.00
ATOM	1120	O	LEU	71	5.753	0.925	2.932	1.00	0.00
ATOM	1121	N	ASN	72	6.020	-0.020	0.930	1.00	0.00

ATOM	1122	H	ASN	72	5.581	-0.583	0.215	1.00	0.00
ATOM	1123	CA	ASN	72	7.430	0.270	0.804	1.00	0.00
ATOM	1124	HA	ASN	72	7.750	0.948	1.595	1.00	0.00
ATOM	1125	CB	ASN	72	8.225	-1.024	0.921	1.00	0.00
ATOM	1126	HB2	ASN	72	7.938	-1.819	0.233	1.00	0.00
ATOM	1127	HB3	ASN	72	9.238	-0.693	0.691	1.00	0.00
ATOM	1128	CG	ASN	72	8.204	-1.571	2.326	1.00	0.00
ATOM	1129	OD1	ASN	72	8.038	-2.740	2.591	1.00	0.00
ATOM	1130	ND2	ASN	72	8.543	-0.760	3.295	1.00	0.00
ATOM	1131	HD21	ASN	72	8.541	-1.090	4.249	1.00	0.00
ATOM	1132	HD22	ASN	72	8.805	0.193	3.084	1.00	0.00
ATOM	1133	C	ASN	72	7.731	0.949	-0.518	1.00	0.00
ATOM	1134	O	ASN	72	8.895	1.190	-0.827	1.00	0.00
ATOM	1135	N	GLU	73	6.689	1.200	-1.306	1.00	0.00
ATOM	1136	H	GLU	73	5.770	0.881	-1.035	1.00	0.00
ATOM	1137	CA	GLU	73	6.839	1.832	-2.582	1.00	0.00
ATOM	1138	HA	GLU	73	7.609	1.327	-3.166	1.00	0.00
ATOM	1139	CB	GLU	73	5.510	1.759	-3.354	1.00	0.00
ATOM	1140	HB2	GLU	73	4.748	1.318	-2.712	1.00	0.00
ATOM	1141	HB3	GLU	73	5.207	2.767	-3.637	1.00	0.00
ATOM	1142	CG	GLU	73	5.674	0.897	-4.622	1.00	0.00
ATOM	1143	HG2	GLU	73	6.709	0.768	-4.938	1.00	0.00

ATOM	1144	HG3	GLU	73	5.270	-0.063	-4.301	1.00	0.00
ATOM	1145	CD	GLU	73	4.846	1.432	-5.794	1.00	0.00
ATOM	1146	OE1	GLU	73	3.712	1.893	-5.528	1.00	0.00
ATOM	1147	OE2	GLU	73	5.413	1.502	-6.907	1.00	0.00
ATOM	1148	C	GLU	73	7.336	3.269	-2.426	1.00	0.00
ATOM	1149	O	GLU	73	7.461	3.797	-1.308	1.00	0.00
ATOM	1150	N	PRO	74	7.561	3.961	-3.553	1.00	0.00
ATOM	1151	CD	PRO	74	7.405	3.475	-4.916	1.00	0.00
ATOM	1152	HD2	PRO	74	6.341	3.290	-5.065	1.00	0.00
ATOM	1153	HD3	PRO	74	7.950	2.534	-4.979	1.00	0.00
ATOM	1154	CG	PRO	74	7.949	4.520	-5.863	1.00	0.00
ATOM	1155	HG2	PRO	74	7.144	4.902	-6.492	1.00	0.00
ATOM	1156	HG3	PRO	74	8.722	4.079	-6.493	1.00	0.00
ATOM	1157	CB	PRO	74	8.528	5.613	-4.978	1.00	0.00
ATOM	1158	HB2	PRO	74	8.212	6.597	-5.325	1.00	0.00
ATOM	1159	HB3	PRO	74	9.617	5.565	-4.969	1.00	0.00
ATOM	1160	CA	PRO	74	7.978	5.355	-3.564	1.00	0.00
ATOM	1161	HA	PRO	74	8.798	5.468	-2.856	1.00	0.00
ATOM	1162	C	PRO	74	6.839	6.307	-3.182	1.00	0.00
ATOM	1163	O	PRO	74	6.678	7.377	-3.769	1.00	0.00
ATOM	1164	N	MET	75	6.064	5.938	-2.170	1.00	0.00
ATOM	1165	H	MET	75	6.258	5.057	-1.717	1.00	0.00

ATOM	1166	CA	MET	75	4.956	6.719	-1.696	1.00	0.00
ATOM	1167	HA	MET	75	5.277	7.729	-1.440	1.00	0.00
ATOM	1168	CB	MET	75	3.910	6.806	-2.809	1.00	0.00
ATOM	1169	HB2	MET	75	4.408	6.731	-3.776	1.00	0.00
ATOM	1170	HB3	MET	75	3.201	5.986	-2.701	1.00	0.00
ATOM	1171	CG	MET	75	3.168	8.131	-2.723	1.00	0.00
ATOM	1172	HG2	MET	75	3.424	8.622	-1.784	1.00	0.00
ATOM	1173	HG3	MET	75	3.472	8.764	-3.558	1.00	0.00
ATOM	1174	SD	MET	75	1.373	7.875	-2.792	1.00	0.00
ATOM	1175	CE	MET	75	0.969	9.048	-4.095	1.00	0.00
ATOM	1176	HE1	MET	75	1.498	8.775	-5.008	1.00	0.00
ATOM	1177	HE2	MET	75	-0.105	9.032	-4.280	1.00	0.00
ATOM	1178	HE3	MET	75	1.269	10.050	-3.789	1.00	0.00
ATOM	1179	C	MET	75	4.350	6.075	-0.457	1.00	0.00
ATOM	1180	O	MET	75	3.144	6.124	-0.261	1.00	0.00
ATOM	1181	N	TRP	76	5.161	5.412	0.358	1.00	0.00
ATOM	1182	H	TRP	76	6.128	5.290	0.093	1.00	0.00
ATOM	1183	CA	TRP	76	4.696	4.775	1.569	1.00	0.00
ATOM	1184	HA	TRP	76	3.871	5.342	1.999	1.00	0.00
ATOM	1185	CB	TRP	76	4.207	3.366	1.252	1.00	0.00
ATOM	1186	HB2	TRP	76	4.882	2.934	0.512	1.00	0.00
ATOM	1187	HB3	TRP	76	4.196	2.739	2.143	1.00	0.00

ATOM 1188 CG TRP 76 2.832 3.302 0.666 1.00 0.00

ATOM 1189 CD1 TRP 76 2.452 2.746 -0.501 1.00 0.00

ATOM 1190 HD1 TRP 76 3.227 2.271 -1.103 1.00 0.00

ATOM 1191 NE1 TRP 76 1.096 2.915 -0.678 1.00 0.00

ATOM 1192 HE1 TRP 76 0.597 2.552 -1.478 1.00 0.00

ATOM 1193 CE2 TRP 76 0.538 3.610 0.356 1.00 0.00

ATOM 1194 CZ2 TRP 76 -0.757 4.021 0.635 1.00 0.00

ATOM 1195 HZ2 TRP 76 -1.543 3.791 -0.084 1.00 0.00

ATOM 1196 CH2 TRP 76 -0.969 4.710 1.830 1.00 0.00

ATOM 1197 HH2 TRP 76 -1.979 5.036 2.077 1.00 0.00

ATOM 1198 CZ3 TRP 76 0.068 4.995 2.715 1.00 0.00

ATOM 1199 HZ3 TRP 76 -0.095 5.549 3.639 1.00 0.00

ATOM 1200 CE3 TRP 76 1.361 4.569 2.428 1.00 0.00

ATOM 1201 HE3 TRP 76 2.126 4.774 3.176 1.00 0.00

ATOM 1202 CD2 TRP 76 1.625 3.874 1.230 1.00 0.00

ATOM 1203 C TRP 76 5.800 4.800 2.603 1.00 0.00

ATOM 1204 O TRP 76 6.571 3.852 2.735 1.00 0.00

ATOM 1205 N ILE 77 5.923 5.919 3.320 1.00 0.00

ATOM 1206 H ILE 77 5.268 6.670 3.155 1.00 0.00

ATOM 1207 CA ILE 77 7.025 6.152 4.254 1.00 0.00

ATOM 1208 HA ILE 77 7.625 5.254 4.398 1.00 0.00

ATOM 1209 CB ILE 77 7.940 7.318 3.840 1.00 0.00

ATOM 1210 HB ILE 77 7.520 8.256 4.205 1.00 0.00

ATOM 1211 CG2 ILE 77 9.342 7.115 4.442 1.00 0.00

ATOM 1212 HG21 ILE 77 9.763 6.179 4.076 1.00 0.00

ATOM 1213 HG22 ILE 77 9.988 7.943 4.147 1.00 0.00

ATOM 1214 HG23 ILE 77 9.270 7.081 5.529 1.00 0.00

ATOM 1215 CG1 ILE 77 7.992 7.511 2.323 1.00 0.00

ATOM 1216 HG12 ILE 77 7.006 7.836 1.991 1.00 0.00

ATOM 1217 HG13 ILE 77 8.722 8.291 2.107 1.00 0.00

ATOM 1218 CD1 ILE 77 8.382 6.230 1.584 1.00 0.00

ATOM 1219 HD11 ILE 77 7.652 5.450 1.798 1.00 0.00

ATOM 1220 HD12 ILE 77 8.404 6.422 0.511 1.00 0.00

ATOM 1221 HD13 ILE 77 9.368 5.905 1.915 1.00 0.00

ATOM 1222 C ILE 77 6.436 6.344 5.621 1.00 0.00

ATOM 1223 O ILE 77 5.497 5.672 5.985 1.00 0.00

ATOM 1224 N ARG 78 6.933 7.256 6.426 1.00 0.00

ATOM 1225 H ARG 78 7.715 7.802 6.094 1.00 0.00

ATOM 1226 CA ARG 78 6.406 7.544 7.731 1.00 0.00

ATOM 1227 HA ARG 78 6.628 6.716 8.403 1.00 0.00

ATOM 1228 CB ARG 78 7.018 8.810 8.303 1.00 0.00

ATOM 1229 HB2 ARG 78 7.278 9.482 7.484 1.00 0.00

ATOM 1230 HB3 ARG 78 6.290 9.296 8.953 1.00 0.00

ATOM 1231 CG ARG 78 8.274 8.476 9.105 1.00 0.00

ATOM	1232	HG2	ARG	78	8.263	9.047	10.033	1.00	0.00
ATOM	1233	HG3	ARG	78	8.274	7.411	9.333	1.00	0.00
ATOM	1234	CD	ARG	78	9.531	8.826	8.308	1.00	0.00
ATOM	1235	HD2	ARG	78	9.449	8.417	7.301	1.00	0.00
ATOM	1236	HD3	ARG	78	9.636	9.910	8.251	1.00	0.00
ATOM	1237	NE	ARG	78	10.732	8.269	8.946	1.00	0.00
ATOM	1238	HE	ARG	78	10.582	7.754	9.801	1.00	0.00
ATOM	1239	CZ	ARG	78	11.968	8.421	8.511	1.00	0.00
ATOM	1240	NH1	ARG	78	12.212	9.085	7.411	1.00	0.00
ATOM	1241	HH11	ARG	78	11.448	9.487	6.887	1.00	0.00
ATOM	1242	HH12	ARG	78	13.164	9.193	7.090	1.00	0.00
ATOM	1243	NH2	ARG	78	12.968	7.876	9.154	1.00	0.00
ATOM	1244	HH21	ARG	78	12.793	7.336	9.988	1.00	0.00
ATOM	1245	HH22	ARG	78	13.911	7.999	8.812	1.00	0.00
ATOM	1246	C	ARG	78	4.927	7.784	7.713	1.00	0.00
ATOM	1247	O	ARG	78	4.324	7.365	8.673	1.00	0.00
ATOM	1248	N	MET	79	4.374	8.320	6.629	1.00	0.00
ATOM	1249	H	MET	79	5.003	8.628	5.901	1.00	0.00
ATOM	1250	CA	MET	79	2.952	8.472	6.415	1.00	0.00
ATOM	1251	HA	MET	79	2.569	9.260	7.063	1.00	0.00
ATOM	1252	CB	MET	79	2.651	8.843	4.967	1.00	0.00
ATOM	1253	HB2	MET	79	1.579	8.736	4.801	1.00	0.00

ATOM	1254	HB3	MET	79	2.940	9.883	4.812	1.00	0.00
ATOM	1255	CG	MET	79	3.412	7.949	3.980	1.00	0.00
ATOM	1256	HG2	MET	79	4.134	7.379	4.564	1.00	0.00
ATOM	1257	HG3	MET	79	2.681	7.269	3.543	1.00	0.00
ATOM	1258	SD	MET	79	4.264	8.809	2.675	1.00	0.00
ATOM	1259	CE	MET	79	3.179	8.402	1.315	1.00	0.00
ATOM	1260	HE1	MET	79	2.178	8.779	1.523	1.00	0.00
ATOM	1261	HE2	MET	79	3.556	8.860	0.399	1.00	0.00
ATOM	1262	HE3	MET	79	3.141	7.320	1.191	1.00	0.00
ATOM	1263	C	MET	79	2.210	7.192	6.699	1.00	0.00
ATOM	1264	O	MET	79	1.094	7.246	7.135	1.00	0.00
ATOM	1265	N	ARG	80	2.825	6.024	6.581	1.00	0.00
ATOM	1266	H	ARG	80	3.721	6.025	6.114	1.00	0.00
ATOM	1267	CA	ARG	80	2.276	4.777	6.983	1.00	0.00
ATOM	1268	HA	ARG	80	1.347	4.615	6.436	1.00	0.00
ATOM	1269	CB	ARG	80	3.198	3.616	6.719	1.00	0.00
ATOM	1270	HB2	ARG	80	4.116	3.760	7.289	1.00	0.00
ATOM	1271	HB3	ARG	80	2.709	2.698	7.044	1.00	0.00
ATOM	1272	CG	ARG	80	3.533	3.515	5.235	1.00	0.00
ATOM	1273	HG2	ARG	80	2.703	3.046	4.707	1.00	0.00
ATOM	1274	HG3	ARG	80	3.701	4.514	4.832	1.00	0.00
ATOM	1275	CD	ARG	80	4.792	2.675	5.050	1.00	0.00

ATOM	1276	HD2	ARG	80	4.523	1.635	5.228	1.00	0.00
ATOM	1277	HD3	ARG	80	5.118	2.794	4.016	1.00	0.00
ATOM	1278	NE	ARG	80	5.901	3.030	5.934	1.00	0.00
ATOM	1279	HE	ARG	80	5.845	3.978	6.278	1.00	0.00
ATOM	1280	CZ	ARG	80	6.912	2.294	6.301	1.00	0.00
ATOM	1281	NH1	ARG	80	7.027	1.083	5.847	1.00	0.00
ATOM	1282	HH11	ARG	80	6.333	0.718	5.211	1.00	0.00
ATOM	1283	HH12	ARG	80	7.811	0.513	6.131	1.00	0.00
ATOM	1284	NH2	ARG	80	7.802	2.772	7.134	1.00	0.00
ATOM	1285	HH21	ARG	80	7.702	3.712	7.490	1.00	0.00
ATOM	1286	HH22	ARG	80	8.584	2.199	7.416	1.00	0.00
ATOM	1287	C	ARG	80	1.881	4.801	8.421	1.00	0.00
ATOM	1288	O	ARG	80	1.163	3.915	8.737	1.00	0.00
ATOM	1289	N	ASP	81	2.219	5.750	9.261	1.00	0.00
ATOM	1290	H	ASP	81	2.759	6.493	8.840	1.00	0.00
ATOM	1291	CA	ASP	81	1.889	5.853	10.668	1.00	0.00
ATOM	1292	HA	ASP	81	2.625	5.323	11.272	1.00	0.00
ATOM	1293	CB	ASP	81	1.892	7.323	11.075	1.00	0.00
ATOM	1294	HB2	ASP	81	2.690	7.813	10.515	1.00	0.00
ATOM	1295	HB3	ASP	81	0.934	7.767	10.804	1.00	0.00
ATOM	1296	CG	ASP	81	2.133	7.507	12.570	1.00	0.00
ATOM	1297	OD1	ASP	81	2.389	6.503	13.268	1.00	0.00

ATOM 1298 OD2 ASP 81 2.036 8.670 13.002 1.00 0.00
ATOM 1299 C ASP 81 0.494 5.295 10.949 1.00 0.00
ATOM 1300 O ASP 81 0.318 4.325 11.680 1.00 0.00
ATOM 1301 N GLN 82 -0.485 5.781 10.175 1.00 0.00
ATOM 1302 H GLN 82 -0.280 6.527 9.525 1.00 0.00
ATOM 1303 CA GLN 82 -1.841 5.328 10.285 1.00 0.00
ATOM 1304 HA GLN 82 -2.056 5.127 11.334 1.00 0.00
ATOM 1305 CB GLN 82 -2.841 6.339 9.772 1.00 0.00
ATOM 1306 HB2 GLN 82 -2.326 7.043 9.118 1.00 0.00
ATOM 1307 HB3 GLN 82 -3.612 5.816 9.206 1.00 0.00
ATOM 1308 CG GLN 82 -3.482 7.094 10.923 1.00 0.00
ATOM 1309 HG2 GLN 82 -4.388 7.569 10.546 1.00 0.00
ATOM 1310 HG3 GLN 82 -3.741 6.401 11.723 1.00 0.00
ATOM 1311 CD GLN 82 -2.570 8.169 11.488 1.00 0.00
ATOM 1312 OE1 GLN 82 -2.811 9.352 11.351 1.00 0.00
ATOM 1313 NE2 GLN 82 -1.488 7.769 12.113 1.00 0.00
ATOM 1314 HE21 GLN 82 -0.853 8.451 12.504 1.00 0.00
ATOM 1315 HE22 GLN 82 -1.294 6.782 12.199 1.00 0.00
ATOM 1316 C GLN 82 -2.047 4.044 9.583 1.00 0.00
ATOM 1317 O GLN 82 -2.136 3.067 10.307 1.00 0.00
ATOM 1318 N VAL 83 -2.231 4.062 8.256 1.00 0.00
ATOM 1319 H VAL 83 -2.378 4.979 7.858 1.00 0.00

ATOM 1320 CA VAL 83 -2.304 2.915 7.379 1.00 0.00
ATOM 1321 HA VAL 83 -3.317 2.525 7.285 1.00 0.00
ATOM 1322 CB VAL 83 -1.759 3.189 5.988 1.00 0.00
ATOM 1323 HB VAL 83 -2.608 3.290 5.312 1.00 0.00
ATOM 1324 CG1 VAL 83 -0.921 4.427 5.923 1.00 0.00
ATOM 1325 HG11 VAL 83 -0.071 4.327 6.597 1.00 0.00
ATOM 1326 HG12 VAL 83 -0.560 4.570 4.904 1.00 0.00
ATOM 1327 HG13 VAL 83 -1.520 5.288 6.219 1.00 0.00
ATOM 1328 CG2 VAL 83 -0.995 2.026 5.425 1.00 0.00
ATOM 1329 HG21 VAL 83 -1.650 1.157 5.361 1.00 0.00
ATOM 1330 HG22 VAL 83 -0.629 2.279 4.429 1.00 0.00
ATOM 1331 HG23 VAL 83 -0.150 1.797 6.074 1.00 0.00
ATOM 1332 C VAL 83 -1.583 1.725 7.968 1.00 0.00
ATOM 1333 O VAL 83 -2.166 0.685 8.046 1.00 0.00
ATOM 1334 N MET 84 -0.353 1.813 8.415 1.00 0.00
ATOM 1335 H MET 84 0.108 2.688 8.208 1.00 0.00
ATOM 1336 CA MET 84 0.386 0.803 9.130 1.00 0.00
ATOM 1337 HA MET 84 0.814 0.089 8.427 1.00 0.00
ATOM 1338 CB MET 84 1.513 1.440 9.921 1.00 0.00
ATOM 1339 HB2 MET 84 2.212 1.894 9.218 1.00 0.00
ATOM 1340 HB3 MET 84 1.089 2.214 10.560 1.00 0.00
ATOM 1341 CG MET 84 2.244 0.423 10.776 1.00 0.00

ATOM	1342	HG2	MET	84	1.523	-0.031	11.454	1.00	0.00
ATOM	1343	HG3	MET	84	2.655	-0.342	10.117	1.00	0.00
ATOM	1344	SD	MET	84	3.582	1.159	11.738	1.00	0.00
ATOM	1345	CE	MET	84	2.684	2.590	12.420	1.00	0.00
ATOM	1346	HE1	MET	84	1.840	2.241	13.014	1.00	0.00
ATOM	1347	HE2	MET	84	3.355	3.174	13.050	1.00	0.00
ATOM	1348	HE3	MET	84	2.319	3.214	11.603	1.00	0.00
ATOM	1349	C	MET	84	-0.487	0.011	10.090	1.00	0.00
ATOM	1350	O	MET	84	-0.679	-1.188	9.891	1.00	0.00
ATOM	1351	N	SER	85	-1.051	0.677	11.099	1.00	0.00
ATOM	1352	H	SER	85	-0.757	1.629	11.266	1.00	0.00
ATOM	1353	CA	SER	85	-2.002	0.080	12.015	1.00	0.00
ATOM	1354	HA	SER	85	-1.739	-0.961	12.200	1.00	0.00
ATOM	1355	CB	SER	85	-1.988	0.827	13.338	1.00	0.00
ATOM	1356	HB2	SER	85	-0.962	1.053	13.628	1.00	0.00
ATOM	1357	HB3	SER	85	-2.552	1.756	13.248	1.00	0.00
ATOM	1358	OG	SER	85	-2.587	0.003	14.324	1.00	0.00
ATOM	1359	HG	SER	85	-2.586	0.462	15.167	1.00	0.00
ATOM	1360	C	SER	85	-3.422	0.030	11.435	1.00	0.00
ATOM	1361	O	SER	85	-4.412	0.135	12.146	1.00	0.00
ATOM	1362	N	MET	86	-3.551	-0.274	10.152	1.00	0.00
ATOM	1363	H	MET	86	-2.706	-0.445	9.626	1.00	0.00

ATOM	1364	CA	MET	86	-4.815	-0.360	9.462	1.00	0.00
ATOM	1365	HA	MET	86	-5.599	-0.698	10.139	1.00	0.00
ATOM	1366	CB	MET	86	-5.185	1.010	8.941	1.00	0.00
ATOM	1367	HB2	MET	86	-4.265	1.516	8.646	1.00	0.00
ATOM	1368	HB3	MET	86	-5.821	0.875	8.066	1.00	0.00
ATOM	1369	CG	MET	86	-5.914	1.849	9.970	1.00	0.00
ATOM	1370	HG2	MET	86	-5.285	1.973	10.851	1.00	0.00
ATOM	1371	HG3	MET	86	-6.143	2.828	9.547	1.00	0.00
ATOM	1372	SD	MET	86	-7.455	1.033	10.449	1.00	0.00
ATOM	1373	CE	MET	86	-7.893	2.071	11.854	1.00	0.00
ATOM	1374	HE1	MET	86	-8.012	3.103	11.521	1.00	0.00
ATOM	1375	HE2	MET	86	-8.829	1.720	12.288	1.00	0.00
ATOM	1376	HE3	MET	86	-7.103	2.021	12.603	1.00	0.00
ATOM	1377	C	MET	86	-4.776	-1.329	8.298	1.00	0.00
ATOM	1378	O	MET	86	-5.590	-2.229	8.230	1.00	0.00
ATOM	1379	N	ALA	87	-3.757	-1.292	7.464	1.00	0.00
ATOM	1380	H	ALA	87	-3.123	-0.518	7.600	1.00	0.00
ATOM	1381	CA	ALA	87	-3.505	-2.190	6.385	1.00	0.00
ATOM	1382	HA	ALA	87	-4.431	-2.450	5.872	1.00	0.00
ATOM	1383	CB	ALA	87	-2.561	-1.533	5.382	1.00	0.00
ATOM	1384	HB1	ALA	87	-1.625	-1.274	5.878	1.00	0.00
ATOM	1385	HB2	ALA	87	-2.359	-2.225	4.564	1.00	0.00

```
ATOM   1386  HB3 ALA    87      -3.023  -0.629   4.985  1.00  0.00
ATOM   1387  C   ALA    87      -2.929  -3.480   6.933  1.00  0.00
ATOM   1388  O   ALA    87      -2.530  -4.322   6.164  1.00  0.00
ATOM   1389  N   HIE    88      -2.806  -3.645   8.242  1.00  0.00
ATOM   1390  H   HIE    88      -2.987  -2.853   8.842  1.00  0.00
ATOM   1391  CA  HIE    88      -2.359  -4.869   8.846  1.00  0.00
ATOM   1392  HA  HIE    88      -1.830  -5.488   8.121  1.00  0.00
ATOM   1393  CB  HIE    88      -1.404  -4.524   9.980  1.00  0.00
ATOM   1394  HB2 HIE    88      -0.781  -5.388  10.208  1.00  0.00
ATOM   1395  HB3 HIE    88      -0.772  -3.696   9.658  1.00  0.00
ATOM   1396  CG  HIE    88      -2.046  -4.099  11.280  1.00  0.00
ATOM   1397  ND1 HIE    88      -1.772  -4.654  12.511  1.00  0.00
ATOM   1398  CE1 HIE    88      -2.505  -3.993  13.436  1.00  0.00
ATOM   1399  HE1 HIE    88      -2.410  -4.257  14.489  1.00  0.00
ATOM   1400  NE2 HIE    88      -3.272  -3.059  12.818  1.00  0.00
ATOM   1401  HE2 HIE    88      -3.930  -2.420  13.240  1.00  0.00
ATOM   1402  CD2 HIE    88      -2.993  -3.130  11.467  1.00  0.00
ATOM   1403  HD2 HIE    88      -3.508  -2.470  10.768  1.00  0.00
ATOM   1404  C   HIE    88      -3.546  -5.675   9.311  1.00  0.00
ATOM   1405  O   HIE    88      -3.409  -6.859   9.481  1.00  0.00
ATOM   1406  N   ASN    89      -4.677  -5.035   9.588  1.00  0.00
ATOM   1407  H   ASN    89      -4.686  -4.028   9.502  1.00  0.00
```

ATOM	1408	CA	ASN	89	-5.897	-5.700	9.980	1.00	0.00
ATOM	1409	HA	ASN	89	-5.686	-6.730	10.265	1.00	0.00
ATOM	1410	CB	ASN	89	-6.520	-4.983	11.170	1.00	0.00
ATOM	1411	HB2	ASN	89	-6.285	-3.919	11.123	1.00	0.00
ATOM	1412	HB3	ASN	89	-7.602	-5.116	11.162	1.00	0.00
ATOM	1413	CG	ASN	89	-5.975	-5.540	12.459	1.00	0.00
ATOM	1414	OD1	ASN	89	-6.654	-5.905	13.384	1.00	0.00
ATOM	1415	ND2	ASN	89	-4.673	-5.614	12.546	1.00	0.00
ATOM	1416	HD21	ASN	89	-4.246	-5.978	13.385	1.00	0.00
ATOM	1417	HD22	ASN	89	-4.100	-5.306	11.773	1.00	0.00
ATOM	1418	C	ASN	89	-6.899	-5.675	8.853	1.00	0.00
ATOM	1419	O	ASN	89	-7.757	-6.514	8.833	1.00	0.00
ATOM	1420	N	LEU	90	-6.795	-4.746	7.912	1.00	0.00
ATOM	1421	H	LEU	90	-6.127	-4.001	8.053	1.00	0.00
ATOM	1422	CA	LEU	90	-7.639	-4.697	6.735	1.00	0.00
ATOM	1423	HA	LEU	90	-8.688	-4.641	7.024	1.00	0.00
ATOM	1424	CB	LEU	90	-7.293	-3.453	5.906	1.00	0.00
ATOM	1425	HB2	LEU	90	-6.224	-3.291	6.045	1.00	0.00
ATOM	1426	HB3	LEU	90	-7.494	-3.663	4.855	1.00	0.00
ATOM	1427	CG	LEU	90	-8.056	-2.199	6.329	1.00	0.00
ATOM	1428	HG	LEU	90	-8.079	-2.142	7.417	1.00	0.00
ATOM	1429	CD1	LEU	90	-7.365	-0.951	5.767	1.00	0.00

ATOM	1430	HD11	LEU	90	-7.342	-1.006	4.678	1.00	0.00
ATOM	1431	HD12	LEU	90	-7.915	-0.061	6.073	1.00	0.00
ATOM	1432	HD13	LEU	90	-6.345	-0.896	6.149	1.00	0.00
ATOM	1433	CD2	LEU	90	-9.499	-2.279	5.839	1.00	0.00
ATOM	1434	HD21	LEU	90	-9.982	-3.155	6.270	1.00	0.00
ATOM	1435	HD22	LEU	90	-10.037	-1.381	6.144	1.00	0.00
ATOM	1436	HD23	LEU	90	-9.510	-2.356	4.751	1.00	0.00
ATOM	1437	C	LEU	90	-7.511	-5.978	5.908	1.00	0.00
ATOM	1438	O	LEU	90	-8.514	-6.596	5.574	1.00	0.00
ATOM	1439	N	PRO	91	-6.277	-6.401	5.581	1.00	0.00
ATOM	1440	CD	PRO	91	-5.017	-5.787	5.923	1.00	0.00
ATOM	1441	HD2	PRO	91	-4.906	-5.865	7.004	1.00	0.00
ATOM	1442	HD3	PRO	91	-5.086	-4.736	5.641	1.00	0.00
ATOM	1443	CG	PRO	91	-3.928	-6.511	5.176	1.00	0.00
ATOM	1444	HG2	PRO	91	-3.251	-6.981	5.889	1.00	0.00
ATOM	1445	HG3	PRO	91	-3.373	-5.798	4.564	1.00	0.00
ATOM	1446	CB	PRO	91	-4.616	-7.533	4.317	1.00	0.00
ATOM	1447	HB2	PRO	91	-4.117	-8.490	4.464	1.00	0.00
ATOM	1448	HB3	PRO	91	-4.618	-7.285	3.255	1.00	0.00
ATOM	1449	CA	PRO	91	-6.045	-7.608	4.836	1.00	0.00
ATOM	1450	HA	PRO	91	-6.750	-7.658	4.006	1.00	0.00
ATOM	1451	C	PRO	91	-6.232	-8.811	5.728	1.00	0.00

ATOM	1452	O	PRO	91	-6.746	-9.809	5.282	1.00	0.00
ATOM	1453	N	ILE	92	-5.856	-8.764	6.986	1.00	0.00
ATOM	1454	H	ILE	92	-5.369	-7.949	7.331	1.00	0.00
ATOM	1455	CA	ILE	92	-6.063	-9.874	7.890	1.00	0.00
ATOM	1456	HA	ILE	92	-5.704	-10.833	7.516	1.00	0.00
ATOM	1457	CB	ILE	92	-5.375	-9.555	9.211	1.00	0.00
ATOM	1458	HB	ILE	92	-5.408	-8.477	9.369	1.00	0.00
ATOM	1459	CG2	ILE	92	-6.066	-10.265	10.381	1.00	0.00
ATOM	1460	HG21	ILE	92	-6.033	-11.343	10.223	1.00	0.00
ATOM	1461	HG22	ILE	92	-5.553	-10.018	11.310	1.00	0.00
ATOM	1462	HG23	ILE	92	-7.104	-9.939	10.442	1.00	0.00
ATOM	1463	CG1	ILE	92	-3.885	-9.882	9.132	1.00	0.00
ATOM	1464	HG12	ILE	92	-3.759	-10.624	8.344	1.00	0.00
ATOM	1465	HG13	ILE	92	-3.367	-8.964	8.852	1.00	0.00
ATOM	1466	CD1	ILE	92	-3.302	-10.416	10.428	1.00	0.00
ATOM	1467	HD11	ILE	92	-3.818	-11.333	10.708	1.00	0.00
ATOM	1468	HD12	ILE	92	-2.241	-10.624	10.291	1.00	0.00
ATOM	1469	HD13	ILE	92	-3.427	-9.673	11.216	1.00	0.00
ATOM	1470	C	ILE	92	-7.539	-10.153	8.091	1.00	0.00
ATOM	1471	O	ILE	92	-7.925	-11.311	8.035	1.00	0.00
ATOM	1472	N	GLU	93	-8.340	-9.114	8.288	1.00	0.00
ATOM	1473	H	GLU	93	-7.939	-8.188	8.336	1.00	0.00

ATOM	1474	CA	GLU	93	-9.778	-9.254	8.466	1.00	0.00
ATOM	1475	HA	GLU	93	-9.980	-9.955	9.275	1.00	0.00
ATOM	1476	CB	GLU	93	-10.418	-7.904	8.815	1.00	0.00
ATOM	1477	HB2	GLU	93	-9.893	-7.117	8.272	1.00	0.00
ATOM	1478	HB3	GLU	93	-11.463	-7.922	8.505	1.00	0.00
ATOM	1479	CG	GLU	93	-10.336	-7.627	10.324	1.00	0.00
ATOM	1480	HG2	GLU	93	-9.944	-8.521	10.809	1.00	0.00
ATOM	1481	HG3	GLU	93	-9.664	-6.789	10.509	1.00	0.00
ATOM	1482	CD	GLU	93	-11.720	-7.305	10.890	1.00	0.00
ATOM	1483	OE1	GLU	93	-12.339	-6.342	10.377	1.00	0.00
ATOM	1484	OE2	GLU	93	-12.132	-8.007	11.838	1.00	0.00
ATOM	1485	C	GLU	93	-10.429	-9.804	7.199	1.00	0.00
ATOM	1486	O	GLU	93	-11.528	-10.343	7.247	1.00	0.00
ATOM	1487	N	CYX	94	-9.736	-9.683	6.063	1.00	0.00
ATOM	1488	H	CYX	94	-8.904	-9.109	6.049	1.00	0.00
ATOM	1489	CA	CYX	94	-10.175	-10.276	4.828	1.00	0.00
ATOM	1490	HA	CYX	94	-11.260	-10.379	4.822	1.00	0.00
ATOM	1491	CB	CYX	94	-9.765	-9.383	3.661	1.00	0.00
ATOM	1492	HB2	CYX	94	-8.823	-8.887	3.895	1.00	0.00
ATOM	1493	HB3	CYX	94	-9.642	-9.990	2.764	1.00	0.00
ATOM	1494	SG	CYX	94	-11.027	-8.133	3.264	1.00	0.00
ATOM	1495	C	CYX	94	-9.639	-11.683	4.663	1.00	0.00

ATOM 1496 O CYX 94 -10.091 -12.333 3.727 1.00 0.00

ATOM 1497 N ASN 95 -8.677 -12.108 5.497 1.00 0.00

ATOM 1498 H ASN 95 -8.362 -11.427 6.173 1.00 0.00

ATOM 1499 CA ASN 95 -8.032 -13.410 5.478 1.00 0.00

ATOM 1500 HA ASN 95 -7.591 -13.626 6.451 1.00 0.00

ATOM 1501 CB ASN 95 -9.067 -14.493 5.158 1.00 0.00

ATOM 1502 HB2 ASN 95 -10.012 -14.182 5.602 1.00 0.00

ATOM 1503 HB3 ASN 95 -9.193 -14.609 4.081 1.00 0.00

ATOM 1504 CG ASN 95 -8.675 -15.820 5.742 1.00 0.00

ATOM 1505 OD1 ASN 95 -9.412 -16.416 6.500 1.00 0.00

ATOM 1506 ND2 ASN 95 -7.488 -16.287 5.438 1.00 0.00

ATOM 1507 HD21 ASN 95 -7.188 -17.176 5.811 1.00 0.00

ATOM 1508 HD22 ASN 95 -6.879 -15.755 4.832 1.00 0.00

ATOM 1509 C ASN 95 -6.893 -13.382 4.440 1.00 0.00

ATOM 1510 O ASN 95 -6.534 -14.360 3.808 1.00 0.00

ATOM 1511 N LEU 96 -6.356 -12.228 4.123 1.00 0.00

ATOM 1512 H LEU 96 -6.738 -11.396 4.549 1.00 0.00

ATOM 1513 CA LEU 96 -5.333 -12.074 3.128 1.00 0.00

ATOM 1514 HA LEU 96 -5.504 -12.785 2.320 1.00 0.00

ATOM 1515 CB LEU 96 -5.333 -10.673 2.548 1.00 0.00

ATOM 1516 HB2 LEU 96 -4.919 -10.080 3.363 1.00 0.00

ATOM 1517 HB3 LEU 96 -4.675 -10.606 1.681 1.00 0.00

ATOM	1518	CG	LEU	96	-6.717	-10.137	2.180	1.00	0.00
ATOM	1519	HG	LEU	96	-7.366	-10.206	3.052	1.00	0.00
ATOM	1520	CD1	LEU	96	-6.629	-8.702	1.734	1.00	0.00
ATOM	1521	HD11	LEU	96	-5.980	-8.631	0.861	1.00	0.00
ATOM	1522	HD12	LEU	96	-7.624	-8.339	1.476	1.00	0.00
ATOM	1523	HD13	LEU	96	-6.219	-8.094	2.540	1.00	0.00
ATOM	1524	CD2	LEU	96	-7.418	-10.999	1.150	1.00	0.00
ATOM	1525	HD21	LEU	96	-7.543	-12.007	1.543	1.00	0.00
ATOM	1526	HD22	LEU	96	-8.396	-10.573	0.924	1.00	0.00
ATOM	1527	HD23	LEU	96	-6.820	-11.036	0.239	1.00	0.00
ATOM	1528	C	LEU	96	-4.033	-12.473	3.779	1.00	0.00
ATOM	1529	O	LEU	96	-3.480	-13.489	3.410	1.00	0.00
ATOM	1530	N	MET	97	-3.651	-11.770	4.843	1.00	0.00
ATOM	1531	H	MET	97	-4.222	-10.988	5.130	1.00	0.00
ATOM	1532	CA	MET	97	-2.423	-12.022	5.560	1.00	0.00
ATOM	1533	HA	MET	97	-1.912	-12.889	5.143	1.00	0.00
ATOM	1534	CB	MET	97	-1.508	-10.812	5.435	1.00	0.00
ATOM	1535	HB2	MET	97	-0.537	-11.077	5.852	1.00	0.00
ATOM	1536	HB3	MET	97	-1.397	-10.580	4.375	1.00	0.00
ATOM	1537	CG	MET	97	-2.055	-9.601	6.162	1.00	0.00
ATOM	1538	HG2	MET	97	-2.203	-8.806	5.430	1.00	0.00
ATOM	1539	HG3	MET	97	-3.015	-9.874	6.599	1.00	0.00

ATOM	1540	SD	MET	97	-0.952	-9.008	7.471	1.00	0.00
ATOM	1541	CE	MET	97	0.521	-8.726	6.466	1.00	0.00
ATOM	1542	HE1	MET	97	0.298	-7.992	5.691	1.00	0.00
ATOM	1543	HE2	MET	97	1.327	-8.352	7.098	1.00	0.00
ATOM	1544	HE3	MET	97	0.829	-9.662	6.000	1.00	0.00
ATOM	1545	C	MET	97	-2.652	-12.365	6.997	1.00	0.00
ATOM	1546	O	MET	97	-1.750	-12.096	7.781	1.00	0.00
ATOM	1547	N	SER	98	-3.853	-12.871	7.318	1.00	0.00
ATOM	1548	H	SER	98	-4.537	-12.971	6.581	1.00	0.00
ATOM	1549	CA	SER	98	-4.250	-13.209	8.682	1.00	0.00
ATOM	1550	HA	SER	98	-4.575	-12.306	9.200	1.00	0.00
ATOM	1551	CB	SER	98	-5.393	-14.211	8.688	1.00	0.00
ATOM	1552	HB2	SER	98	-5.667	-14.420	9.721	1.00	0.00
ATOM	1553	HB3	SER	98	-6.244	-13.771	8.168	1.00	0.00
ATOM	1554	OG	SER	98	-5.019	-15.418	8.045	1.00	0.00
ATOM	1555	HG	SER	98	-5.757	-16.031	8.062	1.00	0.00
ATOM	1556	C	SER	98	-3.088	-13.748	9.533	1.00	0.00
ATOM	1557	O	SER	98	-2.492	-13.113	10.391	1.00	0.00
ATOM	1558	N	GLN	99	-2.632	-14.934	9.218	1.00	0.00
ATOM	1559	H	GLN	99	-3.181	-15.505	8.591	1.00	0.00
ATOM	1560	CA	GLN	99	-1.424	-15.496	9.776	1.00	0.00
ATOM	1561	HA	GLN	99	-0.631	-14.755	9.881	1.00	0.00

ATOM	1562	CB	GLN	99	-1.818	-15.986	11.161	1.00	0.00
ATOM	1563	HB2	GLN	99	-2.822	-15.634	11.398	1.00	0.00
ATOM	1564	HB3	GLN	99	-1.801	-17.075	11.179	1.00	0.00
ATOM	1565	CG	GLN	99	-0.837	-15.447	12.188	1.00	0.00
ATOM	1566	HG2	GLN	99	0.010	-16.130	12.242	1.00	0.00
ATOM	1567	HG3	GLN	99	-0.487	-14.457	11.893	1.00	0.00
ATOM	1568	CD	GLN	99	-1.470	-15.347	13.565	1.00	0.00
ATOM	1569	OE1	GLN	99	-2.550	-15.828	13.858	1.00	0.00
ATOM	1570	NE2	GLN	99	-0.794	-14.672	14.467	1.00	0.00
ATOM	1571	HE21	GLN	99	-1.165	-14.571	15.401	1.00	0.00
ATOM	1572	HE22	GLN	99	0.094	-14.256	14.222	1.00	0.00
ATOM	1573	C	GLN	99	-0.855	-16.590	8.875	1.00	0.00
ATOM	1574	O	GLN	99	-0.669	-17.736	9.282	1.00	0.00
ATOM	1575	N	PRO	100	-0.618	-16.267	7.595	1.00	0.00
ATOM	1576	CD	PRO	100	-0.767	-14.971	6.963	1.00	0.00
ATOM	1577	HD2	PRO	100	-0.024	-14.305	7.402	1.00	0.00
ATOM	1578	HD3	PRO	100	-1.766	-14.605	7.198	1.00	0.00
ATOM	1579	CG	PRO	100	-0.561	-15.171	5.480	1.00	0.00
ATOM	1580	HG2	PRO	100	0.292	-14.574	5.157	1.00	0.00
ATOM	1581	HG3	PRO	100	-1.455	-14.840	4.950	1.00	0.00
ATOM	1582	CB	PRO	100	-0.317	-16.649	5.259	1.00	0.00
ATOM	1583	HB2	PRO	100	0.580	-16.767	4.651	1.00	0.00

ATOM	1584	HB3	PRO	100	-1.152	-17.156	4.776	1.00	0.00
ATOM	1585	CA	PRO	100	-0.082	-17.241	6.657	1.00	0.00
ATOM	1586	HA	PRO	100	-0.634	-18.175	6.759	1.00	0.00
ATOM	1587	C	PRO	100	1.400	-17.492	6.938	1.00	0.00
ATOM	1588	O	PRO	100	1.785	-18.612	7.255	1.00	0.00
ATOM	1589	N	CYX	101	2.232	-16.467	6.762	1.00	0.00
ATOM	1590	H	CYX	101	1.880	-15.593	6.397	1.00	0.00
ATOM	1591	CA	CYX	101	3.647	-16.584	7.005	1.00	0.00
ATOM	1592	HA	CYX	101	4.025	-17.512	6.576	1.00	0.00
ATOM	1593	CB	CYX	101	4.385	-15.412	6.360	1.00	0.00
ATOM	1594	HB2	CYX	101	4.400	-14.567	7.049	1.00	0.00
ATOM	1595	HB3	CYX	101	5.408	-15.709	6.127	1.00	0.00
ATOM	1596	SG	CYX	101	3.637	-14.775	4.810	1.00	0.00
ATOM	1597	C	CYX	101	3.869	-16.662	8.508	1.00	0.00
ATOM	1598	O	CYX	101	3.401	-15.791	9.246	1.00	0.00
ATOM	1599	N	GLN	102	4.589	-17.683	8.943	1.00	0.00
ATOM	1600	H	GLN	102	4.887	-18.387	8.283	1.00	0.00
ATOM	1601	CA	GLN	102	4.909	-17.874	10.348	1.00	0.00
ATOM	1602	HA	GLN	102	4.593	-17.004	10.923	1.00	0.00
ATOM	1603	CB	GLN	102	4.187	-19.107	10.890	1.00	0.00
ATOM	1604	HB2	GLN	102	4.508	-19.981	10.323	1.00	0.00
ATOM	1605	HB3	GLN	102	4.452	-19.238	11.939	1.00	0.00

ATOM	1606	CG	GLN	102	2.669	-18.945	10.763	1.00	0.00
ATOM	1607	HG2	GLN	102	2.431	-17.892	10.915	1.00	0.00
ATOM	1608	HG3	GLN	102	2.329	-19.254	9.774	1.00	0.00
ATOM	1609	CD	GLN	102	1.936	-19.763	11.805	1.00	0.00
ATOM	1610	OE1	GLN	102	1.940	-19.460	12.985	1.00	0.00
ATOM	1611	NE2	GLN	102	1.317	-20.845	11.391	1.00	0.00
ATOM	1612	HE21	GLN	102	0.816	-21.420	12.052	1.00	0.00
ATOM	1613	HE22	GLN	102	1.346	-21.096	10.413	1.00	0.00
ATOM	1614	C	GLN	102	6.413	-17.983	10.539	1.00	0.00
ATOM	1615	O	GLN	102	7.175	-17.838	9.578	1.00	0.00
ATOM	1616	N	MET	103	6.827	-18.160	11.792	1.00	0.00
ATOM	1617	H	MET	103	6.149	-18.097	12.538	1.00	0.00
ATOM	1618	CA	MET	103	8.227	-18.360	12.145	1.00	0.00
ATOM	1619	HA	MET	103	8.891	-17.961	11.377	1.00	0.00
ATOM	1620	CB	MET	103	8.479	-17.602	13.452	1.00	0.00
ATOM	1621	HB2	MET	103	7.617	-16.969	13.662	1.00	0.00
ATOM	1622	HB3	MET	103	8.604	-18.324	14.258	1.00	0.00
ATOM	1623	CG	MET	103	9.734	-16.735	13.347	1.00	0.00
ATOM	1624	HG2	MET	103	10.068	-16.727	12.309	1.00	0.00
ATOM	1625	HG3	MET	103	9.485	-15.719	13.655	1.00	0.00
ATOM	1626	SD	MET	103	11.068	-17.366	14.397	1.00	0.00
ATOM	1627	CE	MET	103	11.385	-15.893	15.381	1.00	0.00

```
ATOM   1628  HE1 MET   103      10.481 -15.616  15.923  1.00  0.00
ATOM   1629  HE2 MET   103      12.186 -16.095  16.091  1.00  0.00
ATOM   1630  HE3 MET   103      11.680 -15.073  14.725  1.00  0.00
ATOM   1631  C   MET   103       8.627 -19.846  12.263  1.00  0.00
ATOM   1632  O   MET   103       7.745 -20.611  12.732  1.00  0.00
ATOM   1633  OXT MET   103       9.799 -20.121  11.907  1.00  0.00
TER
ATOM   1634  Cl1 LIG   104      18.896 -14.108  -2.396  1.00  0.00
ATOM   1635  Cl2 LIG   104      19.906 -16.805  -3.021  1.00  0.00
ATOM   1636  P1  LIG   104      17.759 -18.153   0.825  1.00  0.00
ATOM   1637  O1  LIG   104      17.279 -19.620   0.423  1.00  0.00
ATOM   1638  O2  LIG   104      19.075 -18.445   1.659  1.00  0.00
ATOM   1639  O3  LIG   104      18.288 -17.603  -0.635  1.00  0.00
ATOM   1640  O4  LIG   104      16.806 -17.241   1.473  1.00  0.00
ATOM   1641  C1  LIG   104      16.084 -19.834  -0.363  1.00  0.00
ATOM   1642  H1  LIG   104      15.922 -20.910  -0.370  1.00  0.00
ATOM   1643  H2  LIG   104      16.239 -19.473  -1.381  1.00  0.00
ATOM   1644  H3  LIG   104      15.234 -19.330   0.098  1.00  0.00
ATOM   1645  C2  LIG   104      20.136 -19.316   1.203  1.00  0.00
ATOM   1646  H4  LIG   104      20.517 -18.976   0.239  1.00  0.00
ATOM   1647  H5  LIG   104      19.769 -20.340   1.127  1.00  0.00
ATOM   1648  H6  LIG   104      20.916 -19.249   1.958  1.00  0.00
```

```
ATOM   1649  C3  LIG   104      18.275 -16.266  -0.936  1.00  0.00
ATOM   1650  H7  LIG   104      17.679 -15.624  -0.299  1.00  0.00
ATOM   1651  C4  LIG   104      18.948 -15.797  -1.985  1.00  0.00
TER
ATOM   1652  Cl1 LIG   105     -21.281  13.694   4.827  1.00  0.00
ATOM   1653  Cl2 LIG   105     -20.821  14.356   7.662  1.00  0.00
ATOM   1654  P1  LIG   105     -22.621  18.476   6.660  1.00  0.00
ATOM   1655  O1  LIG   105     -23.385  18.827   8.016  1.00  0.00
ATOM   1656  O2  LIG   105     -21.271  19.292   6.813  1.00  0.00
ATOM   1657  O3  LIG   105     -22.211  16.907   6.948  1.00  0.00
ATOM   1658  O4  LIG   105     -23.316  18.664   5.379  1.00  0.00
ATOM   1659  C1  LIG   105     -24.693  18.285   8.307  1.00  0.00
ATOM   1660  H1  LIG   105     -25.030  18.787   9.211  1.00  0.00
ATOM   1661  H2  LIG   105     -24.621  17.210   8.482  1.00  0.00
ATOM   1662  H3  LIG   105     -25.380  18.493   7.486  1.00  0.00
ATOM   1663  C2  LIG   105     -20.438  19.250   7.995  1.00  0.00
ATOM   1664  H4  LIG   105     -20.140  18.222   8.211  1.00  0.00
ATOM   1665  H5  LIG   105     -20.975  19.676   8.843  1.00  0.00
ATOM   1666  H6  LIG   105     -19.564  19.851   7.760  1.00  0.00
ATOM   1667  C3  LIG   105     -22.046  16.017   5.918  1.00  0.00
ATOM   1668  H7  LIG   105     -22.436  16.307   4.950  1.00  0.00
ATOM   1669  C4  LIG   105     -21.452  14.843   6.121  1.00  0.00
```

TER

ATOM 1670 Cl1 LIG 106 12.338 21.479 6.781 1.00 0.00

ATOM 1671 Cl2 LIG 106 13.902 23.854 7.555 1.00 0.00

ATOM 1672 P1 LIG 106 15.880 23.507 3.410 1.00 0.00

ATOM 1673 O1 LIG 106 16.114 25.042 3.041 1.00 0.00

ATOM 1674 O2 LIG 106 17.307 23.066 3.940 1.00 0.00

ATOM 1675 O3 LIG 106 14.949 23.665 4.760 1.00 0.00

ATOM 1676 O4 LIG 106 15.290 22.612 2.405 1.00 0.00

ATOM 1677 C1 LIG 106 15.050 25.860 2.505 1.00 0.00

ATOM 1678 H1 LIG 106 15.513 26.802 2.220 1.00 0.00

ATOM 1679 H2 LIG 106 14.292 26.033 3.271 1.00 0.00

ATOM 1680 H3 LIG 106 14.608 25.380 1.631 1.00 0.00

ATOM 1681 C2 LIG 106 18.060 23.809 4.927 1.00 0.00

ATOM 1682 H4 LIG 106 17.475 23.926 5.840 1.00 0.00

ATOM 1683 H5 LIG 106 18.337 24.784 4.526 1.00 0.00

ATOM 1684 H6 LIG 106 18.949 23.216 5.126 1.00 0.00

ATOM 1685 C3 LIG 106 14.053 22.693 5.120 1.00 0.00

ATOM 1686 H7 LIG 106 13.797 21.957 4.369 1.00 0.00

ATOM 1687 C4 LIG 106 13.510 22.685 6.336 1.00 0.00

TER

ATOM 1688 Cl1 LIG 107 21.486 -10.672 17.327 1.00 0.00

ATOM 1689 Cl2 LIG 107 22.779 -8.035 17.081 1.00 0.00

```
ATOM   1690  P1  LIG   107      22.132  -7.650  21.626  1.00  0.00
ATOM   1691  O1  LIG   107      23.432  -6.834  22.063  1.00  0.00
ATOM   1692  O2  LIG   107      21.031  -6.513  21.543  1.00  0.00
ATOM   1693  O3  LIG   107      22.512  -7.996  20.060  1.00  0.00
ATOM   1694  O4  LIG   107      21.739  -8.837  22.398  1.00  0.00
ATOM   1695  C1  LIG   107      24.704  -7.492  22.262  1.00  0.00
ATOM   1696  H1  LIG   107      25.366  -6.738  22.683  1.00  0.00
ATOM   1697  H2  LIG   107      25.096  -7.842  21.306  1.00  0.00
ATOM   1698  H3  LIG   107      24.594  -8.324  22.959  1.00  0.00
ATOM   1699  C2  LIG   107      21.219  -5.266  20.835  1.00  0.00
ATOM   1700  H4  LIG   107      21.465  -5.457  19.790  1.00  0.00
ATOM   1701  H5  LIG   107      22.009  -4.684  21.311  1.00  0.00
ATOM   1702  H6  LIG   107      20.269  -4.742  20.907  1.00  0.00
ATOM   1703  C3  LIG   107      22.008  -9.108  19.438  1.00  0.00
ATOM   1704  H7  LIG   107      21.572  -9.871  20.070  1.00  0.00
ATOM   1705  C4  LIG   107      22.084  -9.242  18.115  1.00  0.00
TER
ATOM   1706  Cl1 LIG   108      11.246  12.468 -12.877  1.00  0.00
ATOM   1707  Cl2 LIG   108      13.738  11.751 -11.476  1.00  0.00
ATOM   1708  P1  LIG   108      15.645  11.504 -15.661  1.00  0.00
ATOM   1709  O1  LIG   108      17.124  12.025 -15.367  1.00  0.00
ATOM   1710  O2  LIG   108      15.836   9.930 -15.667  1.00  0.00
```

```
ATOM   1711  O3  LIG   108      14.919  11.844 -14.222  1.00  0.00
ATOM   1712  O4  LIG   108      14.914  12.042 -16.816  1.00  0.00
ATOM   1713  C1  LIG   108      17.423  13.436 -15.274  1.00  0.00
ATOM   1714  H1  LIG   108      18.506  13.508 -15.209  1.00  0.00
ATOM   1715  H2  LIG   108      16.965  13.855 -14.377  1.00  0.00
ATOM   1716  H3  LIG   108      17.067  13.957 -16.164  1.00  0.00
ATOM   1717  C2  LIG   108      16.529   9.203 -14.625  1.00  0.00
ATOM   1718  H4  LIG   108      16.068   9.400 -13.656  1.00  0.00
ATOM   1719  H5  LIG   108      17.581   9.489 -14.611  1.00  0.00
ATOM   1720  H6  LIG   108      16.426   8.152 -14.883  1.00  0.00
ATOM   1721  C3  LIG   108      13.574  12.100 -14.150  1.00  0.00
ATOM   1722  H7  LIG   108      13.065  12.320 -15.079  1.00  0.00
ATOM   1723  C4  LIG   108      12.943  12.099 -12.977  1.00  0.00
TER
ATOM   1724  Cl1 LIG   109      22.304 -18.542 -22.331  1.00  0.00
ATOM   1725  Cl2 LIG   109      20.576 -16.227 -21.746  1.00  0.00
ATOM   1726  P1  LIG   109      19.199 -18.596 -18.043  1.00  0.00
ATOM   1727  O1  LIG   109      18.971 -17.425 -16.984  1.00  0.00
ATOM   1728  O2  LIG   109      17.727 -18.876 -18.559  1.00  0.00
ATOM   1729  O3  LIG   109      19.930 -17.759 -19.259  1.00  0.00
ATOM   1730  O4  LIG   109      19.950 -19.792 -17.636  1.00  0.00
ATOM   1731  C1  LIG   109      20.074 -16.846 -16.250  1.00  0.00
```

ATOM 1732 H1 LIG 109 19.625 -16.183 -15.513 1.00 0.00

ATOM 1733 H2 LIG 109 20.713 -16.276 -16.926 1.00 0.00

ATOM 1734 H3 LIG 109 20.646 -17.628 -15.750 1.00 0.00

ATOM 1735 C2 LIG 109 16.823 -17.838 -19.004 1.00 0.00

ATOM 1736 H4 LIG 109 17.273 -17.267 -19.817 1.00 0.00

ATOM 1737 H5 LIG 109 16.574 -17.181 -18.170 1.00 0.00

ATOM 1738 H6 LIG 109 15.933 -18.355 -19.357 1.00 0.00

ATOM 1739 C3 LIG 109 20.798 -18.375 -20.123 1.00 0.00

ATOM 1740 H7 LIG 109 21.178 -19.346 -19.829 1.00 0.00

ATOM 1741 C4 LIG 109 21.168 -17.782 -21.255 1.00 0.00

TER

ATOM 1742 Cl1 LIG 110 -10.189 7.135 -13.254 1.00 0.00

ATOM 1743 Cl2 LIG 110 -7.755 5.717 -12.388 1.00 0.00

ATOM 1744 P1 LIG 110 -6.706 5.443 -16.865 1.00 0.00

ATOM 1745 O1 LIG 110 -5.116 5.569 -16.826 1.00 0.00

ATOM 1746 O2 LIG 110 -6.917 3.878 -16.992 1.00 0.00

ATOM 1747 O3 LIG 110 -7.070 5.801 -15.298 1.00 0.00

ATOM 1748 O4 LIG 110 -7.464 6.251 -17.830 1.00 0.00

ATOM 1749 C1 LIG 110 -4.463 6.854 -16.714 1.00 0.00

ATOM 1750 H1 LIG 110 -3.401 6.662 -16.849 1.00 0.00

ATOM 1751 H2 LIG 110 -4.643 7.278 -15.724 1.00 0.00

ATOM 1752 H3 LIG 110 -4.823 7.530 -17.491 1.00 0.00

```
ATOM   1753  C2  LIG   110      -6.262   2.908 -16.141  1.00  0.00
ATOM   1754  H4  LIG   110      -6.489   3.111 -15.093  1.00  0.00
ATOM   1755  H5  LIG   110      -5.185   2.935 -16.309  1.00  0.00
ATOM   1756  H6  LIG   110      -6.667   1.942 -16.432  1.00  0.00
ATOM   1757  C3  LIG   110      -8.276   6.358 -14.960  1.00  0.00
ATOM   1758  H7  LIG   110      -8.864   6.783 -15.764  1.00  0.00
ATOM   1759  C4  LIG   110      -8.680   6.389 -13.692  1.00  0.00
TER
ATOM   1760  Cl1 LIG   111     -13.456 -25.378 -12.460  1.00  0.00
ATOM   1761  Cl2 LIG   111     -14.972 -23.068 -13.485  1.00  0.00
ATOM   1762  P1  LIG   111     -18.720 -25.234 -11.909  1.00  0.00
ATOM   1763  O1  LIG   111     -19.622 -24.005 -11.438  1.00  0.00
ATOM   1764  O2  LIG   111     -19.329 -25.575 -13.332  1.00  0.00
ATOM   1765  O3  LIG   111     -17.301 -24.457 -12.222  1.00  0.00
ATOM   1766  O4  LIG   111     -18.558 -26.387 -11.013  1.00  0.00
ATOM   1767  C1  LIG   111     -19.412 -23.360 -10.161  1.00  0.00
ATOM   1768  H1  LIG   111     -20.238 -22.662 -10.041  1.00  0.00
ATOM   1769  H2  LIG   111     -18.464 -22.820 -10.168  1.00  0.00
ATOM   1770  H3  LIG   111     -19.426 -24.099  -9.359  1.00  0.00
ATOM   1771  C2  LIG   111     -19.570 -24.584 -14.358  1.00  0.00
ATOM   1772  H4  LIG   111     -18.647 -24.054 -14.597  1.00  0.00
ATOM   1773  H5  LIG   111     -20.335 -23.883 -14.024  1.00  0.00
```

ATOM 1774 H6 LIG 111 -19.919 -25.140 -15.226 1.00 0.00

ATOM 1775 C3 LIG 111 -16.099 -25.108 -12.118 1.00 0.00

ATOM 1776 H7 LIG 111 -16.094 -26.051 -11.585 1.00 0.00

ATOM 1777 C4 LIG 111 -14.990 -24.577 -12.631 1.00 0.00

TER

ATOM 1778 Cl1 LIG 112 -7.156 21.658 19.353 1.00 0.00

ATOM 1779 Cl2 LIG 112 -5.192 19.958 20.748 1.00 0.00

ATOM 1780 P1 LIG 112 -7.642 16.389 19.173 1.00 0.00

ATOM 1781 O1 LIG 112 -6.497 15.308 18.915 1.00 0.00

ATOM 1782 O2 LIG 112 -8.215 15.943 20.581 1.00 0.00

ATOM 1783 O3 LIG 112 -6.726 17.723 19.483 1.00 0.00

ATOM 1784 O4 LIG 112 -8.657 16.617 18.135 1.00 0.00

ATOM 1785 C1 LIG 112 -5.692 15.333 17.714 1.00 0.00

ATOM 1786 H1 LIG 112 -5.099 14.421 17.733 1.00 0.00

ATOM 1787 H2 LIG 112 -5.036 16.205 17.723 1.00 0.00

ATOM 1788 H3 LIG 112 -6.332 15.345 16.830 1.00 0.00

ATOM 1789 C2 LIG 112 -7.385 15.666 21.734 1.00 0.00

ATOM 1790 H4 LIG 112 -6.769 16.534 21.973 1.00 0.00

ATOM 1791 H5 LIG 112 -6.757 14.797 21.538 1.00 0.00

ATOM 1792 H6 LIG 112 -8.075 15.460 22.549 1.00 0.00

ATOM 1793 C3 LIG 112 -7.197 18.983 19.222 1.00 0.00

ATOM 1794 H7 LIG 112 -8.066 19.060 18.580 1.00 0.00

```
ATOM   1795  C4  LIG   112      -6.586  20.056  19.720  1.00  0.00
TER
ATOM   1796  Cl1 LIG   113      18.468  19.675  -6.529  1.00  0.00
ATOM   1797  Cl2 LIG   113      17.233  21.912  -5.060  1.00  0.00
ATOM   1798  P1  LIG   113      15.458  23.178  -9.118  1.00  0.00
ATOM   1799  O1  LIG   113      14.046  23.772  -8.674  1.00  0.00
ATOM   1800  O2  LIG   113      16.337  24.486  -9.286  1.00  0.00
ATOM   1801  O3  LIG   113      15.955  22.504  -7.699  1.00  0.00
ATOM   1802  O4  LIG   113      15.518  22.243 -10.250  1.00  0.00
ATOM   1803  C1  LIG   113      12.915  22.911  -8.407  1.00  0.00
ATOM   1804  H1  LIG   113      12.064  23.573  -8.264  1.00  0.00
ATOM   1805  H2  LIG   113      13.094  22.332  -7.499  1.00  0.00
ATOM   1806  H3  LIG   113      12.736  22.248  -9.254  1.00  0.00
ATOM   1807  C2  LIG   113      16.416  25.532  -8.289  1.00  0.00
ATOM   1808  H4  LIG   113      16.751  25.121  -7.336  1.00  0.00
ATOM   1809  H5  LIG   113      15.442  26.010  -8.176  1.00  0.00
ATOM   1810  H6  LIG   113      17.145  26.243  -8.668  1.00  0.00
ATOM   1811  C3  LIG   113      16.807  21.430  -7.684  1.00  0.00
ATOM   1812  H7  LIG   113      16.934  20.890  -8.614  1.00  0.00
ATOM   1813  C4  LIG   113      17.422  21.064  -6.561  1.00  0.00
TER
END
```

Table N: The number of atoms present in the system of HA-Nap

ATOM	1	N	PRO	1	7.123	-2.899	8.478	1.00	0.00
ATOM	2	H2	PRO	1	7.610	-2.017	8.552	1.00	0.00
ATOM	3	H3	PRO	1	7.575	-3.751	8.776	1.00	0.00
ATOM	4	CD	PRO	1	5.857	-2.854	9.272	1.00	0.00
ATOM	5	HD2	PRO	1	5.791	-1.838	9.663	1.00	0.00
ATOM	6	HD3	PRO	1	6.002	-3.551	10.096	1.00	0.00
ATOM	7	CG	PRO	1	4.653	-3.227	8.394	1.00	0.00
ATOM	8	HG2	PRO	1	3.951	-2.393	8.369	1.00	0.00
ATOM	9	HG3	PRO	1	4.159	-4.104	8.811	1.00	0.00
ATOM	10	CB	PRO	1	5.213	-3.525	7.007	1.00	0.00
ATOM	11	HB2	PRO	1	4.662	-3.057	6.191	1.00	0.00
ATOM	12	HB3	PRO	1	5.233	-4.604	6.859	1.00	0.00
ATOM	13	CA	PRO	1	6.652	-2.973	7.051	1.00	0.00
ATOM	14	HA	PRO	1	7.245	-3.733	6.543	1.00	0.00
ATOM	15	C	PRO	1	6.792	-1.632	6.306	1.00	0.00
ATOM	16	O	PRO	1	5.820	-1.027	5.855	1.00	0.00
ATOM	17	N	TYR	2	8.036	-1.197	6.167	1.00	0.00
ATOM	18	H	TYR	2	8.798	-1.747	6.536	1.00	0.00
ATOM	19	CA	TYR	2	8.350	0.055	5.501	1.00	0.00
ATOM	20	HA	TYR	2	7.474	0.700	5.438	1.00	0.00

ATOM	21	CB	TYR	2	9.421	0.764	6.333	1.00	0.00
ATOM	22	HB2	TYR	2	9.754	1.658	5.804	1.00	0.00
ATOM	23	HB3	TYR	2	8.927	1.054	7.260	1.00	0.00
ATOM	24	CG	TYR	2	10.624	-0.114	6.652	1.00	0.00
ATOM	25	CD1	TYR	2	10.611	-0.940	7.796	1.00	0.00
ATOM	26	HD1	TYR	2	9.745	-0.937	8.457	1.00	0.00
ATOM	27	CE1	TYR	2	11.714	-1.767	8.084	1.00	0.00
ATOM	28	HE1	TYR	2	11.730	-2.357	9.000	1.00	0.00
ATOM	29	CZ	TYR	2	12.837	-1.755	7.234	1.00	0.00
ATOM	30	OH	TYR	2	13.894	-2.575	7.468	1.00	0.00
ATOM	31	HH	TYR	2	13.775	-3.127	8.243	1.00	0.00
ATOM	32	CE2	TYR	2	12.829	-0.959	6.079	1.00	0.00
ATOM	33	HE2	TYR	2	13.690	-0.980	5.410	1.00	0.00
ATOM	34	CD2	TYR	2	11.727	-0.139	5.780	1.00	0.00
ATOM	35	HD2	TYR	2	11.731	0.472	4.877	1.00	0.00
ATOM	36	C	TYR	2	8.869	-0.187	4.092	1.00	0.00
ATOM	37	O	TYR	2	8.660	0.662	3.243	1.00	0.00
ATOM	38	N	GLY	3	9.456	-1.370	3.857	1.00	0.00
ATOM	39	H	GLY	3	9.496	-2.043	4.608	1.00	0.00
ATOM	40	CA	GLY	3	10.034	-1.730	2.583	1.00	0.00
ATOM	41	HA2	GLY	3	10.373	-2.765	2.624	1.00	0.00
ATOM	42	HA3	GLY	3	9.277	-1.626	1.805	1.00	0.00

ATOM	43	C	GLY	3	11.220	-0.833	2.244	1.00	0.00
ATOM	44	O	GLY	3	11.169	0.392	2.248	1.00	0.00
ATOM	45	N	ARG	4	12.360	-1.461	1.994	1.00	0.00
ATOM	46	H	ARG	4	12.432	-2.456	2.146	1.00	0.00
ATOM	47	CA	ARG	4	13.559	-0.735	1.587	1.00	0.00
ATOM	48	HA	ARG	4	13.388	0.341	1.641	1.00	0.00
ATOM	49	CB	ARG	4	14.715	-1.095	2.526	1.00	0.00
ATOM	50	HB2	ARG	4	14.326	-1.196	3.539	1.00	0.00
ATOM	51	HB3	ARG	4	15.146	-2.043	2.205	1.00	0.00
ATOM	52	CG	ARG	4	15.799	-0.007	2.501	1.00	0.00
ATOM	53	HG2	ARG	4	15.639	0.631	1.631	1.00	0.00
ATOM	54	HG3	ARG	4	15.719	0.591	3.408	1.00	0.00
ATOM	55	CD	ARG	4	17.193	-0.633	2.424	1.00	0.00
ATOM	56	HD2	ARG	4	17.350	-1.277	3.288	1.00	0.00
ATOM	57	HD3	ARG	4	17.276	-1.223	1.511	1.00	0.00
ATOM	58	NE	ARG	4	18.237	0.402	2.412	1.00	0.00
ATOM	59	HE	ARG	4	17.920	1.350	2.266	1.00	0.00
ATOM	60	CZ	ARG	4	19.530	0.187	2.560	1.00	0.00
ATOM	61	NH1	ARG	4	19.986	-1.024	2.752	1.00	0.00
ATOM	62	HH11	ARG	4	19.344	-1.802	2.787	1.00	0.00
ATOM	63	HH12	ARG	4	20.979	-1.174	2.863	1.00	0.00
ATOM	64	NH2	ARG	4	20.378	1.179	2.532	1.00	0.00

ATOM 65 HH21 ARG 4 20.043 2.122 2.395 1.00 0.00

ATOM 66 HH22 ARG 4 21.365 0.999 2.646 1.00 0.00

ATOM 67 C ARG 4 13.915 -1.000 0.126 1.00 0.00

ATOM 68 O ARG 4 14.999 -0.631 -0.305 1.00 0.00

ATOM 69 N GLY 5 13.074 -1.776 -0.561 1.00 0.00

ATOM 70 H GLY 5 12.234 -2.114 -0.114 1.00 0.00

ATOM 71 CA GLY 5 13.319 -2.154 -1.931 1.00 0.00

ATOM 72 HA2 GLY 5 12.493 -2.767 -2.292 1.00 0.00

ATOM 73 HA3 GLY 5 13.400 -1.257 -2.546 1.00 0.00

ATOM 74 C GLY 5 14.605 -2.945 -2.042 1.00 0.00

ATOM 75 O GLY 5 15.349 -2.761 -2.990 1.00 0.00

ATOM 76 N ARG 6 14.891 -3.788 -1.042 1.00 0.00

ATOM 77 H ARG 6 14.279 -3.802 -0.239 1.00 0.00

ATOM 78 CA ARG 6 16.083 -4.628 -1.019 1.00 0.00

ATOM 79 HA ARG 6 16.944 -4.052 -1.358 1.00 0.00

ATOM 80 CB ARG 6 16.361 -5.136 0.398 1.00 0.00

ATOM 81 HB2 ARG 6 17.125 -5.911 0.345 1.00 0.00

ATOM 82 HB3 ARG 6 16.729 -4.304 0.999 1.00 0.00

ATOM 83 CG ARG 6 15.087 -5.714 1.045 1.00 0.00

ATOM 84 HG2 ARG 6 14.637 -4.947 1.676 1.00 0.00

ATOM 85 HG3 ARG 6 14.388 -5.989 0.255 1.00 0.00

ATOM 86 CD ARG 6 15.411 -6.945 1.891 1.00 0.00

```
ATOM     87  HD2 ARG     6      16.489  -7.009   2.040  1.00  0.00
ATOM     88  HD3 ARG     6      14.915  -6.861   2.858  1.00  0.00
ATOM     89  NE  ARG     6      14.952  -8.178   1.231  1.00  0.00
ATOM     90  HE  ARG     6      14.700  -8.093   0.256  1.00  0.00
ATOM     91  CZ  ARG     6      14.870  -9.365   1.802  1.00  0.00
ATOM     92  NH1 ARG     6      15.185  -9.520   3.064  1.00  0.00
ATOM     93 HH11 ARG     6      15.493  -8.725   3.605  1.00  0.00
ATOM     94 HH12 ARG     6      15.118 -10.433   3.489  1.00  0.00
ATOM     95  NH2 ARG     6      14.451 -10.397   1.124  1.00  0.00
ATOM     96 HH21 ARG     6      14.187 -10.287   0.155  1.00  0.00
ATOM     97 HH22 ARG     6      14.392 -11.300   1.570  1.00  0.00
ATOM     98  C   ARG     6      15.982  -5.795  -1.988  1.00  0.00
ATOM     99  O   ARG     6      16.126  -6.937  -1.554  1.00  0.00
ATOM    100  N   THR     7      15.644  -5.527  -3.233  1.00  0.00
ATOM    101  H   THR     7      15.511  -4.558  -3.487  1.00  0.00
ATOM    102  CA  THR     7      15.389  -6.541  -4.235  1.00  0.00
ATOM    103  HA  THR     7      16.053  -7.384  -4.043  1.00  0.00
ATOM    104  CB  THR     7      13.930  -7.023  -4.108  1.00  0.00
ATOM    105  HB  THR     7      13.663  -7.564  -5.016  1.00  0.00
ATOM    106  CG2 THR     7      13.749  -7.943  -2.892  1.00  0.00
ATOM    107 HG21 THR     7      14.015  -7.402  -1.984  1.00  0.00
ATOM    108 HG22 THR     7      12.710  -8.265  -2.832  1.00  0.00
```

ATOM 109 HG23 THR 7 14.394 -8.815 -2.997 1.00 0.00
ATOM 110 OG1 THR 7 13.057 -5.924 -3.893 1.00 0.00
ATOM 111 HG1 THR 7 12.155 -6.242 -3.817 1.00 0.00
ATOM 112 C THR 7 15.688 -5.985 -5.612 1.00 0.00
ATOM 113 O THR 7 16.154 -4.857 -5.731 1.00 0.00
ATOM 114 N GLU 8 15.445 -6.789 -6.637 1.00 0.00
ATOM 115 H GLU 8 15.149 -7.738 -6.464 1.00 0.00
ATOM 116 CA GLU 8 15.667 -6.376 -8.016 1.00 0.00
ATOM 117 HA GLU 8 16.253 -5.457 -8.046 1.00 0.00
ATOM 118 CB GLU 8 16.438 -7.473 -8.761 1.00 0.00
ATOM 119 HB2 GLU 8 15.983 -8.438 -8.539 1.00 0.00
ATOM 120 HB3 GLU 8 16.378 -7.282 -9.833 1.00 0.00
ATOM 121 CG GLU 8 17.907 -7.489 -8.327 1.00 0.00
ATOM 122 HG2 GLU 8 18.035 -6.941 -7.394 1.00 0.00
ATOM 123 HG3 GLU 8 18.182 -8.533 -8.176 1.00 0.00
ATOM 124 CD GLU 8 18.804 -6.877 -9.404 1.00 0.00
ATOM 125 OE1 GLU 8 19.110 -7.608 -10.373 1.00 0.00
ATOM 126 OE2 GLU 8 19.136 -5.679 -9.268 1.00 0.00
ATOM 127 C GLU 8 14.345 -6.047 -8.708 1.00 0.00
ATOM 128 O GLU 8 13.266 -6.045 -8.096 1.00 0.00
ATOM 129 N SER 9 14.435 -5.729 -9.995 1.00 0.00
ATOM 130 H SER 9 15.350 -5.614 -10.407 1.00 0.00

ATOM 131 CA SER 9 13.272 -5.460 -10.819 1.00 0.00
ATOM 132 HA SER 9 12.650 -4.709 -10.332 1.00 0.00
ATOM 133 CB SER 9 13.676 -4.937 -12.206 1.00 0.00
ATOM 134 HB2 SER 9 13.065 -5.448 -12.950 1.00 0.00
ATOM 135 HB3 SER 9 13.467 -3.867 -12.237 1.00 0.00
ATOM 136 OG SER 9 15.043 -5.157 -12.489 1.00 0.00
ATOM 137 HG SER 9 15.248 -4.815 -13.363 1.00 0.00
ATOM 138 C SER 9 12.378 -6.698 -10.958 1.00 0.00
ATOM 139 O SER 9 12.730 -7.817 -10.589 1.00 0.00
ATOM 140 N GLY 10 11.184 -6.505 -11.515 1.00 0.00
ATOM 141 H GLY 10 10.896 -5.575 -11.787 1.00 0.00
ATOM 142 CA GLY 10 10.262 -7.611 -11.750 1.00 0.00
ATOM 143 HA2 GLY 10 10.395 -7.975 -12.769 1.00 0.00
ATOM 144 HA3 GLY 10 10.482 -8.413 -11.046 1.00 0.00
ATOM 145 C GLY 10 8.834 -7.178 -11.568 1.00 0.00
ATOM 146 O GLY 10 8.244 -6.612 -12.478 1.00 0.00
ATOM 147 N CYX 11 8.329 -7.281 -10.338 1.00 0.00
ATOM 148 H CYX 11 8.856 -7.747 -9.613 1.00 0.00
ATOM 149 CA CYX 11 7.004 -6.808 -10.024 1.00 0.00
ATOM 150 HA CYX 11 6.286 -7.217 -10.735 1.00 0.00
ATOM 151 CB CYX 11 6.606 -7.245 -8.624 1.00 0.00
ATOM 152 HB2 CYX 11 7.224 -6.725 -7.892 1.00 0.00

ATOM 153 HB3 CYX 11 5.557 -7.003 -8.452 1.00 0.00
ATOM 154 SG CYX 11 6.722 -9.003 -8.271 1.00 0.00
ATOM 155 C CYX 11 6.904 -5.284 -10.169 1.00 0.00
ATOM 156 O CYX 11 5.817 -4.723 -10.139 1.00 0.00
ATOM 157 N TYR 12 8.047 -4.617 -10.260 1.00 0.00
ATOM 158 H TYR 12 8.899 -5.154 -10.184 1.00 0.00
ATOM 159 CA TYR 12 8.152 -3.199 -10.479 1.00 0.00
ATOM 160 HA TYR 12 7.406 -2.644 -9.910 1.00 0.00
ATOM 161 CB TYR 12 9.535 -2.794 -9.992 1.00 0.00
ATOM 162 HB2 TYR 12 9.612 -3.124 -8.956 1.00 0.00
ATOM 163 HB3 TYR 12 10.272 -3.322 -10.597 1.00 0.00
ATOM 164 CG TYR 12 9.794 -1.312 -10.067 1.00 0.00
ATOM 165 CD1 TYR 12 8.972 -0.420 -9.359 1.00 0.00
ATOM 166 HD1 TYR 12 8.146 -0.806 -8.763 1.00 0.00
ATOM 167 CE1 TYR 12 9.210 0.964 -9.417 1.00 0.00
ATOM 168 HE1 TYR 12 8.545 1.655 -8.900 1.00 0.00
ATOM 169 CZ TYR 12 10.260 1.460 -10.209 1.00 0.00
ATOM 170 OH TYR 12 10.525 2.794 -10.225 1.00 0.00
ATOM 171 HH TYR 12 9.936 3.301 -9.662 1.00 0.00
ATOM 172 CE2 TYR 12 11.086 0.565 -10.922 1.00 0.00
ATOM 173 HE2 TYR 12 11.911 0.948 -11.524 1.00 0.00
ATOM 174 CD2 TYR 12 10.846 -0.820 -10.857 1.00 0.00

ATOM	175	HD2	TYR	12	11.476	-1.509	-11.420	1.00	0.00
ATOM	176	C	TYR	12	8.019	-2.833	-11.952	1.00	0.00
ATOM	177	O	TYR	12	7.356	-1.871	-12.290	1.00	0.00
ATOM	178	N	GLN	13	8.634	-3.623	-12.825	1.00	0.00
ATOM	179	H	GLN	13	9.178	-4.413	-12.510	1.00	0.00
ATOM	180	CA	GLN	13	8.563	-3.362	-14.247	1.00	0.00
ATOM	181	HA	GLN	13	8.933	-2.360	-14.468	1.00	0.00
ATOM	182	CB	GLN	13	9.428	-4.377	-14.993	1.00	0.00
ATOM	183	HB2	GLN	13	10.478	-4.166	-14.790	1.00	0.00
ATOM	184	HB3	GLN	13	9.184	-5.379	-14.641	1.00	0.00
ATOM	185	CG	GLN	13	9.173	-4.290	-16.503	1.00	0.00
ATOM	186	HG2	GLN	13	8.137	-4.542	-16.729	1.00	0.00
ATOM	187	HG3	GLN	13	9.369	-3.260	-16.802	1.00	0.00
ATOM	188	CD	GLN	13	10.079	-5.206	-17.301	1.00	0.00
ATOM	189	OE1	GLN	13	11.050	-5.766	-16.813	1.00	0.00
ATOM	190	NE2	GLN	13	9.757	-5.419	-18.556	1.00	0.00
ATOM	191	HE21	GLN	13	10.332	-6.022	-19.127	1.00	0.00
ATOM	192	HE22	GLN	13	8.936	-4.978	-18.946	1.00	0.00
ATOM	193	C	GLN	13	7.116	-3.456	-14.714	1.00	0.00
ATOM	194	O	GLN	13	6.647	-2.666	-15.496	1.00	0.00
ATOM	195	N	GLN	14	6.389	-4.466	-14.254	1.00	0.00
ATOM	196	H	GLN	14	6.814	-5.181	-13.682	1.00	0.00

ATOM	197	CA	GLN	14	4.988	-4.611	-14.612	1.00	0.00
ATOM	198	HA	GLN	14	4.871	-4.523	-15.693	1.00	0.00
ATOM	199	CB	GLN	14	4.464	-5.980	-14.173	1.00	0.00
ATOM	200	HB2	GLN	14	3.375	-5.942	-14.152	1.00	0.00
ATOM	201	HB3	GLN	14	4.787	-6.723	-14.902	1.00	0.00
ATOM	202	CG	GLN	14	4.986	-6.362	-12.793	1.00	0.00
ATOM	203	HG2	GLN	14	5.909	-6.921	-12.946	1.00	0.00
ATOM	204	HG3	GLN	14	5.203	-5.448	-12.240	1.00	0.00
ATOM	205	CD	GLN	14	4.048	-7.199	-11.984	1.00	0.00
ATOM	206	OE1	GLN	14	3.291	-7.956	-12.537	1.00	0.00
ATOM	207	NE2	GLN	14	4.082	-7.050	-10.667	1.00	0.00
ATOM	208	HE21	GLN	14	3.465	-7.596	-10.084	1.00	0.00
ATOM	209	HE22	GLN	14	4.725	-6.390	-10.253	1.00	0.00
ATOM	210	C	GLN	14	4.133	-3.501	-14.011	1.00	0.00
ATOM	211	O	GLN	14	3.114	-3.144	-14.571	1.00	0.00
ATOM	212	N	MET	15	4.527	-2.993	-12.849	1.00	0.00
ATOM	213	H	MET	15	5.368	-3.370	-12.437	1.00	0.00
ATOM	214	CA	MET	15	3.849	-1.928	-12.162	1.00	0.00
ATOM	215	HA	MET	15	2.876	-2.278	-11.820	1.00	0.00
ATOM	216	CB	MET	15	4.645	-1.458	-10.961	1.00	0.00
ATOM	217	HB2	MET	15	5.574	-2.026	-10.916	1.00	0.00
ATOM	218	HB3	MET	15	4.874	-0.399	-11.089	1.00	0.00

ATOM	219	CG	MET	15	3.876	-1.654	-9.687	1.00	0.00
ATOM	220	HG2	MET	15	2.867	-1.259	-9.804	1.00	0.00
ATOM	221	HG3	MET	15	3.825	-2.716	-9.450	1.00	0.00
ATOM	222	SD	MET	15	4.696	-0.789	-8.347	1.00	0.00
ATOM	223	CE	MET	15	5.535	-2.167	-7.614	1.00	0.00
ATOM	224	HE1	MET	15	6.209	-2.613	-8.345	1.00	0.00
ATOM	225	HE2	MET	15	6.109	-1.827	-6.752	1.00	0.00
ATOM	226	HE3	MET	15	4.804	-2.909	-7.294	1.00	0.00
ATOM	227	C	MET	15	3.675	-0.739	-13.062	1.00	0.00
ATOM	228	O	MET	15	2.573	-0.242	-13.114	1.00	0.00
ATOM	229	N	GLU	16	4.727	-0.265	-13.721	1.00	0.00
ATOM	230	H	GLU	16	5.638	-0.667	-13.551	1.00	0.00
ATOM	231	CA	GLU	16	4.629	0.861	-14.646	1.00	0.00
ATOM	232	HA	GLU	16	4.374	1.773	-14.107	1.00	0.00
ATOM	233	CB	GLU	16	5.981	1.067	-15.346	1.00	0.00
ATOM	234	HB2	GLU	16	6.082	2.125	-15.592	1.00	0.00
ATOM	235	HB3	GLU	16	6.773	0.778	-14.655	1.00	0.00
ATOM	236	CG	GLU	16	6.092	0.231	-16.630	1.00	0.00
ATOM	237	HG2	GLU	16	5.718	-0.753	-16.350	1.00	0.00
ATOM	238	HG3	GLU	16	5.473	0.640	-17.429	1.00	0.00
ATOM	239	CD	GLU	16	7.534	0.108	-17.116	1.00	0.00
ATOM	240	OE1	GLU	16	8.377	-0.459	-16.379	1.00	0.00

ATOM	241	OE2	GLU	16	7.834	0.654	-18.198	1.00	0.00
ATOM	242	C	GLU	16	3.513	0.643	-15.677	1.00	0.00
ATOM	243	O	GLU	16	2.830	1.588	-16.062	1.00	0.00
ATOM	244	N	GLU	17	3.299	-0.622	-16.055	1.00	0.00
ATOM	245	H	GLU	17	3.879	-1.355	-15.674	1.00	0.00
ATOM	246	CA	GLU	17	2.286	-0.981	-17.015	1.00	0.00
ATOM	247	HA	GLU	17	2.283	-0.263	-17.836	1.00	0.00
ATOM	248	CB	GLU	17	2.563	-2.378	-17.586	1.00	0.00
ATOM	249	HB2	GLU	17	3.598	-2.654	-17.384	1.00	0.00
ATOM	250	HB3	GLU	17	1.896	-3.101	-17.118	1.00	0.00
ATOM	251	CG	GLU	17	2.324	-2.369	-19.099	1.00	0.00
ATOM	252	HG2	GLU	17	1.734	-3.245	-19.367	1.00	0.00
ATOM	253	HG3	GLU	17	1.771	-1.466	-19.359	1.00	0.00
ATOM	254	CD	GLU	17	3.649	-2.393	-19.865	1.00	0.00
ATOM	255	OE1	GLU	17	4.537	-1.589	-19.530	1.00	0.00
ATOM	256	OE2	GLU	17	3.760	-3.229	-20.789	1.00	0.00
ATOM	257	C	GLU	17	0.912	-0.951	-16.354	1.00	0.00
ATOM	258	O	GLU	17	-0.101	-0.712	-17.011	1.00	0.00
ATOM	259	N	ALA	18	0.852	-1.212	-15.050	1.00	0.00
ATOM	260	H	ALA	18	1.694	-1.526	-14.589	1.00	0.00
ATOM	261	CA	ALA	18	-0.360	-1.150	-14.285	1.00	0.00
ATOM	262	HA	ALA	18	-1.204	-0.797	-14.878	1.00	0.00

ATOM	263	CB	ALA	18	-0.662	-2.562	-13.798	1.00	0.00
ATOM	264	HB1	ALA	18	0.171	-2.924	-13.196	1.00	0.00
ATOM	265	HB2	ALA	18	-1.570	-2.552	-13.195	1.00	0.00
ATOM	266	HB3	ALA	18	-0.803	-3.220	-14.656	1.00	0.00
ATOM	267	C	ALA	18	-0.191	-0.139	-13.176	1.00	0.00
ATOM	268	O	ALA	18	-0.097	-0.539	-12.029	1.00	0.00
ATOM	269	N	GLU	19	-0.072	1.150	-13.469	1.00	0.00
ATOM	270	H	GLU	19	-0.017	1.406	-14.445	1.00	0.00
ATOM	271	CA	GLU	19	0.099	2.195	-12.472	1.00	0.00
ATOM	272	HA	GLU	19	0.894	1.923	-11.779	1.00	0.00
ATOM	273	CB	GLU	19	0.475	3.514	-13.153	1.00	0.00
ATOM	274	HB2	GLU	19	1.522	3.465	-13.454	1.00	0.00
ATOM	275	HB3	GLU	19	-0.150	3.640	-14.037	1.00	0.00
ATOM	276	CG	GLU	19	0.272	4.696	-12.216	1.00	0.00
ATOM	277	HG2	GLU	19	0.082	4.422	-11.179	1.00	0.00
ATOM	278	HG3	GLU	19	1.208	5.252	-12.280	1.00	0.00
ATOM	279	CD	GLU	19	-0.900	5.587	-12.742	1.00	0.00
ATOM	280	OE1	GLU	19	-1.882	5.198	-13.419	1.00	0.00
ATOM	281	OE2	GLU	19	-0.923	6.765	-12.368	1.00	0.00
ATOM	282	C	GLU	19	-1.200	2.256	-11.619	1.00	0.00
ATOM	283	O	GLU	19	-2.042	3.147	-11.602	1.00	0.00
ATOM	284	N	MET	20	-1.424	1.300	-10.763	1.00	0.00

ATOM	285	H	MET	20	-0.755	0.549	-10.670	1.00	0.00
ATOM	286	CA	MET	20	-2.616	1.259	-9.953	1.00	0.00
ATOM	287	HA	MET	20	-3.396	1.890	-10.381	1.00	0.00
ATOM	288	CB	MET	20	-3.117	-0.165	-9.908	1.00	0.00
ATOM	289	HB2	MET	20	-4.187	-0.127	-9.704	1.00	0.00
ATOM	290	HB3	MET	20	-2.950	-0.600	-10.894	1.00	0.00
ATOM	291	CG	MET	20	-2.437	-1.010	-8.865	1.00	0.00
ATOM	292	HG2	MET	20	-1.451	-0.574	-8.701	1.00	0.00
ATOM	293	HG3	MET	20	-3.029	-0.922	-7.954	1.00	0.00
ATOM	294	SD	MET	20	-2.256	-2.717	-9.277	1.00	0.00
ATOM	295	CE	MET	20	-0.987	-2.635	-10.498	1.00	0.00
ATOM	296	HE1	MET	20	-0.090	-2.195	-10.062	1.00	0.00
ATOM	297	HE2	MET	20	-0.761	-3.639	-10.857	1.00	0.00
ATOM	298	HE3	MET	20	-1.324	-2.019	-11.333	1.00	0.00
ATOM	299	C	MET	20	-2.269	1.767	-8.582	1.00	0.00
ATOM	300	O	MET	20	-3.163	2.102	-7.806	1.00	0.00
ATOM	301	N	LEU	21	-0.969	1.787	-8.265	1.00	0.00
ATOM	302	H	LEU	21	-0.297	1.406	-8.916	1.00	0.00
ATOM	303	CA	LEU	21	-0.497	2.228	-6.976	1.00	0.00
ATOM	304	HA	LEU	21	-1.248	2.031	-6.212	1.00	0.00
ATOM	305	CB	LEU	21	0.773	1.476	-6.615	1.00	0.00
ATOM	306	HB2	LEU	21	1.440	1.832	-7.401	1.00	0.00

ATOM	307	HB3	LEU	21	1.158	1.787	-5.644	1.00	0.00
ATOM	308	CG	LEU	21	0.693	-0.063	-6.667	1.00	0.00
ATOM	309	HG	LEU	21	1.461	-0.451	-5.998	1.00	0.00
ATOM	310	CD1	LEU	21	-0.659	-0.617	-6.261	1.00	0.00
ATOM	311	HD11	LEU	21	-1.427	-0.229	-6.931	1.00	0.00
ATOM	312	HD12	LEU	21	-0.639	-1.705	-6.323	1.00	0.00
ATOM	313	HD13	LEU	21	-0.884	-0.315	-5.239	1.00	0.00
ATOM	314	CD2	LEU	21	1.143	-0.552	-8.031	1.00	0.00
ATOM	315	HD21	LEU	21	2.171	-0.237	-8.210	1.00	0.00
ATOM	316	HD22	LEU	21	1.086	-1.640	-8.065	1.00	0.00
ATOM	317	HD23	LEU	21	0.496	-0.130	-8.801	1.00	0.00
ATOM	318	C	LEU	21	-0.333	3.736	-6.980	1.00	0.00
ATOM	319	O	LEU	21	0.239	4.304	-6.063	1.00	0.00
ATOM	320	N	ASN	22	-0.907	4.379	-7.995	1.00	0.00
ATOM	321	H	ASN	22	-1.404	3.853	-8.700	1.00	0.00
ATOM	322	CA	ASN	22	-0.878	5.811	-8.108	1.00	0.00
ATOM	323	HA	ASN	22	-0.087	6.219	-7.479	1.00	0.00
ATOM	324	CB	ASN	22	-0.612	6.215	-9.541	1.00	0.00
ATOM	325	HB2	ASN	22	0.334	5.954	-10.015	1.00	0.00
ATOM	326	HB3	ASN	22	-1.416	5.636	-9.994	1.00	0.00
ATOM	327	CG	ASN	22	-0.885	7.698	-9.781	1.00	0.00
ATOM	328	OD1	ASN	22	-1.972	8.133	-10.145	1.00	0.00

ATOM 329 ND2 ASN 22 0.023 8.511 -9.291 1.00 0.00
ATOM 330 HD21 ASN 22 -0.082 9.509 -9.407 1.00 0.00
ATOM 331 HD22 ASN 22 0.822 8.135 -8.802 1.00 0.00
ATOM 332 C ASN 22 -2.223 6.373 -7.714 1.00 0.00
ATOM 333 O ASN 22 -2.277 7.390 -7.051 1.00 0.00
ATOM 334 N HIE 23 -3.321 5.696 -8.062 1.00 0.00
ATOM 335 H HIE 23 -3.221 4.842 -8.591 1.00 0.00
ATOM 336 CA HIE 23 -4.657 6.158 -7.749 1.00 0.00
ATOM 337 HA HIE 23 -4.763 7.212 -8.008 1.00 0.00
ATOM 338 CB HIE 23 -5.673 5.355 -8.552 1.00 0.00
ATOM 339 HB2 HIE 23 -5.500 4.286 -8.434 1.00 0.00
ATOM 340 HB3 HIE 23 -6.680 5.600 -8.214 1.00 0.00
ATOM 341 CG HIE 23 -5.636 5.636 -10.027 1.00 0.00
ATOM 342 ND1 HIE 23 -6.595 6.351 -10.718 1.00 0.00
ATOM 343 CE1 HIE 23 -6.226 6.385 -12.019 1.00 0.00
ATOM 344 HE1 HIE 23 -6.847 6.908 -12.746 1.00 0.00
ATOM 345 NE2 HIE 23 -5.062 5.706 -12.169 1.00 0.00
ATOM 346 HE2 HIE 23 -4.549 5.562 -13.028 1.00 0.00
ATOM 347 CD2 HIE 23 -4.680 5.232 -10.927 1.00 0.00
ATOM 348 HD2 HIE 23 -3.761 4.655 -10.823 1.00 0.00
ATOM 349 C HIE 23 -4.899 6.015 -6.260 1.00 0.00
ATOM 350 O HIE 23 -5.156 7.022 -5.605 1.00 0.00

ATOM	351	N	CYX	24	-4.831	4.777	-5.746	1.00	0.00
ATOM	352	H	CYX	24	-4.701	3.980	-6.353	1.00	0.00
ATOM	353	CA	CYX	24	-4.974	4.540	-4.315	1.00	0.00
ATOM	354	HA	CYX	24	-5.973	4.838	-3.997	1.00	0.00
ATOM	355	CB	CYX	24	-4.772	3.066	-3.974	1.00	0.00
ATOM	356	HB2	CYX	24	-4.362	2.546	-4.840	1.00	0.00
ATOM	357	HB3	CYX	24	-4.080	2.978	-3.137	1.00	0.00
ATOM	358	SG	CYX	24	-6.271	2.197	-3.512	1.00	0.00
ATOM	359	C	CYX	24	-3.965	5.383	-3.547	1.00	0.00
ATOM	360	O	CYX	24	-4.289	5.816	-2.463	1.00	0.00
ATOM	361	N	GLY	25	-2.822	5.710	-4.153	1.00	0.00
ATOM	362	H	GLY	25	-2.651	5.337	-5.076	1.00	0.00
ATOM	363	CA	GLY	25	-1.823	6.562	-3.566	1.00	0.00
ATOM	364	HA2	GLY	25	-1.633	6.233	-2.545	1.00	0.00
ATOM	365	HA3	GLY	25	-0.905	6.484	-4.149	1.00	0.00
ATOM	366	C	GLY	25	-2.268	8.003	-3.542	1.00	0.00
ATOM	367	O	GLY	25	-2.232	8.616	-2.492	1.00	0.00
ATOM	368	N	MET	26	-2.807	8.521	-4.637	1.00	0.00
ATOM	369	H	MET	26	-2.938	7.895	-5.419	1.00	0.00
ATOM	370	CA	MET	26	-3.252	9.885	-4.772	1.00	0.00
ATOM	371	HA	MET	26	-2.401	10.560	-4.672	1.00	0.00
ATOM	372	CB	MET	26	-3.897	10.113	-6.136	1.00	0.00

ATOM 373 HB2 MET 26 -3.829 9.192 -6.714 1.00 0.00
ATOM 374 HB3 MET 26 -4.945 10.373 -5.989 1.00 0.00
ATOM 375 CG MET 26 -3.198 11.235 -6.888 1.00 0.00
ATOM 376 HG2 MET 26 -3.808 12.138 -6.841 1.00 0.00
ATOM 377 HG3 MET 26 -2.228 11.428 -6.430 1.00 0.00
ATOM 378 SD MET 26 -2.957 10.775 -8.610 1.00 0.00
ATOM 379 CE MET 26 -1.621 11.910 -9.040 1.00 0.00
ATOM 380 HE1 MET 26 -0.759 11.716 -8.402 1.00 0.00
ATOM 381 HE2 MET 26 -1.340 11.764 -10.083 1.00 0.00
ATOM 382 HE3 MET 26 -1.955 12.938 -8.896 1.00 0.00
ATOM 383 C MET 26 -4.298 10.190 -3.745 1.00 0.00
ATOM 384 O MET 26 -4.217 11.233 -3.141 1.00 0.00
ATOM 385 N TYR 27 -5.306 9.342 -3.579 1.00 0.00
ATOM 386 H TYR 27 -5.358 8.537 -4.186 1.00 0.00
ATOM 387 CA TYR 27 -6.331 9.506 -2.560 1.00 0.00
ATOM 388 HA TYR 27 -7.051 10.278 -2.833 1.00 0.00
ATOM 389 CB TYR 27 -7.061 8.171 -2.461 1.00 0.00
ATOM 390 HB2 TYR 27 -7.496 7.980 -3.442 1.00 0.00
ATOM 391 HB3 TYR 27 -6.318 7.405 -2.238 1.00 0.00
ATOM 392 CG TYR 27 -8.149 8.138 -1.413 1.00 0.00
ATOM 393 CD1 TYR 27 -9.298 8.929 -1.580 1.00 0.00
ATOM 394 HD1 TYR 27 -9.403 9.561 -2.463 1.00 0.00

ATOM	395	CE1	TYR	27	-10.314	8.906	-0.607	1.00	0.00
ATOM	396	HE1	TYR	27	-11.217	9.500	-0.747	1.00	0.00
ATOM	397	CZ	TYR	27	-10.179	8.083	0.531	1.00	0.00
ATOM	398	OH	TYR	27	-11.143	8.069	1.490	1.00	0.00
ATOM	399	HH	TYR	27	-11.875	8.658	1.294	1.00	0.00
ATOM	400	CE2	TYR	27	-9.024	7.299	0.699	1.00	0.00
ATOM	401	HE2	TYR	27	-8.919	6.668	1.581	1.00	0.00
ATOM	402	CD2	TYR	27	-8.005	7.329	-0.270	1.00	0.00
ATOM	403	HD2	TYR	27	-7.106	6.727	-0.135	1.00	0.00
ATOM	404	C	TYR	27	-5.754	9.855	-1.192	1.00	0.00
ATOM	405	O	TYR	27	-6.294	10.693	-0.478	1.00	0.00
ATOM	406	N	LEU	28	-4.598	9.278	-0.883	1.00	0.00
ATOM	407	H	LEU	28	-4.197	8.616	-1.532	1.00	0.00
ATOM	408	CA	LEU	28	-3.918	9.507	0.369	1.00	0.00
ATOM	409	HA	LEU	28	-4.640	9.804	1.130	1.00	0.00
ATOM	410	CB	LEU	28	-3.210	8.244	0.836	1.00	0.00
ATOM	411	HB2	LEU	28	-2.257	8.311	0.310	1.00	0.00
ATOM	412	HB3	LEU	28	-3.035	8.286	1.911	1.00	0.00
ATOM	413	CG	LEU	28	-3.901	6.915	0.480	1.00	0.00
ATOM	414	HG	LEU	28	-4.601	7.098	-0.336	1.00	0.00
ATOM	415	CD1	LEU	28	-2.894	5.858	0.056	1.00	0.00
ATOM	416	HD11	LEU	28	-2.194	5.675	0.871	1.00	0.00

ATOM	417	HD12	LEU	28	-3.417	4.934	-0.188	1.00	0.00
ATOM	418	HD13	LEU	28	-2.347	6.207	-0.820	1.00	0.00
ATOM	419	CD2	LEU	28	-4.764	6.442	1.612	1.00	0.00
ATOM	420	HD21	LEU	28	-5.529	7.190	1.823	1.00	0.00
ATOM	421	HD22	LEU	28	-5.242	5.502	1.337	1.00	0.00
ATOM	422	HD23	LEU	28	-4.149	6.291	2.499	1.00	0.00
ATOM	423	C	LEU	28	-2.941	10.660	0.211	1.00	0.00
ATOM	424	O	LEU	28	-2.725	11.394	1.158	1.00	0.00
ATOM	425	N	MET	29	-2.451	10.918	-0.998	1.00	0.00
ATOM	426	H	MET	29	-2.643	10.211	-1.693	1.00	0.00
ATOM	427	CA	MET	29	-1.613	12.033	-1.371	1.00	0.00
ATOM	428	HA	MET	29	-0.971	12.320	-0.538	1.00	0.00
ATOM	429	CB	MET	29	-0.732	11.635	-2.550	1.00	0.00
ATOM	430	HB2	MET	29	-0.934	10.594	-2.802	1.00	0.00
ATOM	431	HB3	MET	29	-0.980	12.269	-3.402	1.00	0.00
ATOM	432	CG	MET	29	0.739	11.797	-2.210	1.00	0.00
ATOM	433	HG2	MET	29	0.914	11.393	-1.214	1.00	0.00
ATOM	434	HG3	MET	29	1.329	11.239	-2.937	1.00	0.00
ATOM	435	SD	MET	29	1.239	13.521	-2.243	1.00	0.00
ATOM	436	CE	MET	29	1.234	13.769	-4.028	1.00	0.00
ATOM	437	HE1	MET	29	0.235	13.580	-4.419	1.00	0.00
ATOM	438	HE2	MET	29	1.523	14.796	-4.254	1.00	0.00

```
ATOM    439  HE3 MET    29       1.942  13.083  -4.493  1.00  0.00
ATOM    440  C   MET    29      -2.401  13.304  -1.670  1.00  0.00
ATOM    441  O   MET    29      -1.867  14.295  -2.155  1.00  0.00
ATOM    442  N   LYS    30      -3.709  13.287  -1.443  1.00  0.00
ATOM    443  H   LYS    30      -4.132  12.428  -1.124  1.00  0.00
ATOM    444  CA  LYS    30      -4.569  14.417  -1.709  1.00  0.00
ATOM    445  HA  LYS    30      -4.227  14.953  -2.595  1.00  0.00
ATOM    446  CB  LYS    30      -5.999  13.923  -1.957  1.00  0.00
ATOM    447  HB2 LYS    30      -6.068  12.869  -1.691  1.00  0.00
ATOM    448  HB3 LYS    30      -6.691  14.499  -1.343  1.00  0.00
ATOM    449  CG  LYS    30      -6.360  14.100  -3.437  1.00  0.00
ATOM    450  HG2 LYS    30      -5.449  14.057  -4.035  1.00  0.00
ATOM    451  HG3 LYS    30      -7.031  13.297  -3.740  1.00  0.00
ATOM    452  CD  LYS    30      -7.050  15.451  -3.655  1.00  0.00
ATOM    453  HD2 LYS    30      -7.958  15.275  -4.231  1.00  0.00
ATOM    454  HD3 LYS    30      -7.313  15.856  -2.678  1.00  0.00
ATOM    455  CE  LYS    30      -6.165  16.447  -4.395  1.00  0.00
ATOM    456  HE2 LYS    30      -6.589  17.446  -4.287  1.00  0.00
ATOM    457  HE3 LYS    30      -5.167  16.427  -3.957  1.00  0.00
ATOM    458  NZ  LYS    30      -6.079  16.100  -5.837  1.00  0.00
ATOM    459  HZ1 LYS    30      -7.003  16.119  -6.244  1.00  0.00
ATOM    460  HZ2 LYS    30      -5.489  16.770  -6.310  1.00  0.00
```

ATOM	461	HZ3	LYS	30	-5.686	15.176	-5.938	1.00	0.00
ATOM	462	C	LYS	30	-4.470	15.384	-0.552	1.00	0.00
ATOM	463	O	LYS	30	-4.775	16.548	-0.763	1.00	0.00
ATOM	464	N	ASN	31	-4.080	14.884	0.624	1.00	0.00
ATOM	465	H	ASN	31	-3.952	13.887	0.716	1.00	0.00
ATOM	466	CA	ASN	31	-3.843	15.721	1.790	1.00	0.00
ATOM	467	HA	ASN	31	-4.258	16.718	1.642	1.00	0.00
ATOM	468	CB	ASN	31	-4.533	15.077	2.995	1.00	0.00
ATOM	469	HB2	ASN	31	-4.513	13.990	2.930	1.00	0.00
ATOM	470	HB3	ASN	31	-3.997	15.398	3.888	1.00	0.00
ATOM	471	CG	ASN	31	-5.969	15.527	3.107	1.00	0.00
ATOM	472	OD1	ASN	31	-6.372	16.243	3.998	1.00	0.00
ATOM	473	ND2	ASN	31	-6.772	15.139	2.145	1.00	0.00
ATOM	474	HD21	ASN	31	-7.745	15.409	2.164	1.00	0.00
ATOM	475	HD22	ASN	31	-6.413	14.573	1.389	1.00	0.00
ATOM	476	C	ASN	31	-2.352	15.874	2.079	1.00	0.00
ATOM	477	O	ASN	31	-1.988	16.623	2.974	1.00	0.00
ATOM	478	N	LEU	32	-1.527	15.121	1.350	1.00	0.00
ATOM	479	H	LEU	32	-1.899	14.443	0.701	1.00	0.00
ATOM	480	CA	LEU	32	-0.106	15.189	1.521	1.00	0.00
ATOM	481	HA	LEU	32	0.131	15.429	2.557	1.00	0.00
ATOM	482	CB	LEU	32	0.537	13.853	1.171	1.00	0.00

ATOM	483	HB2	LEU	32	0.194	13.620	0.162	1.00	0.00
ATOM	484	HB3	LEU	32	1.623	13.953	1.170	1.00	0.00
ATOM	485	CG	LEU	32	0.131	12.731	2.117	1.00	0.00
ATOM	486	HG	LEU	32	-0.938	12.803	2.314	1.00	0.00
ATOM	487	CD1	LEU	32	0.442	11.379	1.500	1.00	0.00
ATOM	488	HD11	LEU	32	1.512	11.307	1.302	1.00	0.00
ATOM	489	HD12	LEU	32	0.146	10.588	2.188	1.00	0.00
ATOM	490	HD13	LEU	32	-0.108	11.271	0.565	1.00	0.00
ATOM	491	CD2	LEU	32	0.836	12.891	3.451	1.00	0.00
ATOM	492	HD21	LEU	32	0.563	13.849	3.894	1.00	0.00
ATOM	493	HD22	LEU	32	0.538	12.084	4.119	1.00	0.00
ATOM	494	HD23	LEU	32	1.915	12.857	3.298	1.00	0.00
ATOM	495	C	LEU	32	0.454	16.314	0.694	1.00	0.00
ATOM	496	O	LEU	32	1.515	16.744	1.098	1.00	0.00
ATOM	497	N	GLY	33	-0.259	16.778	-0.359	1.00	0.00
ATOM	498	H	GLY	33	-1.143	16.324	-0.539	1.00	0.00
ATOM	499	CA	GLY	33	0.149	17.865	-1.237	1.00	0.00
ATOM	500	HA2	GLY	33	-0.374	17.765	-2.189	1.00	0.00
ATOM	501	HA3	GLY	33	-0.121	18.814	-0.773	1.00	0.00
ATOM	502	C	GLY	33	1.652	17.849	-1.493	1.00	0.00
ATOM	503	O	GLY	33	2.432	18.531	-0.837	1.00	0.00
ATOM	504	N	GLU	34	2.058	17.104	-2.513	1.00	0.00

ATOM	505	H	GLU	34	1.401	16.566	-3.059	1.00	0.00
ATOM	506	CA	GLU	34	3.474	16.983	-2.823	1.00	0.00
ATOM	507	HA	GLU	34	4.052	16.984	-1.899	1.00	0.00
ATOM	508	CB	GLU	34	3.759	15.683	-3.576	1.00	0.00
ATOM	509	HB2	GLU	34	3.452	14.843	-2.954	1.00	0.00
ATOM	510	HB3	GLU	34	3.181	15.680	-4.501	1.00	0.00
ATOM	511	CG	GLU	34	5.248	15.556	-3.905	1.00	0.00
ATOM	512	HG2	GLU	34	5.393	14.557	-4.315	1.00	0.00
ATOM	513	HG3	GLU	34	5.524	16.299	-4.654	1.00	0.00
ATOM	514	CD	GLU	34	6.127	15.731	-2.666	1.00	0.00
ATOM	515	OE1	GLU	34	5.643	15.476	-1.546	1.00	0.00
ATOM	516	OE2	GLU	34	7.285	16.159	-2.831	1.00	0.00
ATOM	517	C	GLU	34	3.951	18.192	-3.620	1.00	0.00
ATOM	518	O	GLU	34	4.166	18.118	-4.832	1.00	0.00
ATOM	519	N	ARG	35	3.960	19.363	-2.987	1.00	0.00
ATOM	520	H	ARG	35	3.628	19.413	-2.035	1.00	0.00
ATOM	521	CA	ARG	35	4.400	20.588	-3.626	1.00	0.00
ATOM	522	HA	ARG	35	5.421	20.471	-3.989	1.00	0.00
ATOM	523	CB	ARG	35	3.488	20.925	-4.822	1.00	0.00
ATOM	524	HB2	ARG	35	3.195	20.000	-5.317	1.00	0.00
ATOM	525	HB3	ARG	35	2.599	21.439	-4.457	1.00	0.00
ATOM	526	CG	ARG	35	4.220	21.821	-5.814	1.00	0.00

ATOM	527	HG2	ARG	35	3.603	22.698	-6.015	1.00	0.00
ATOM	528	HG3	ARG	35	5.164	22.136	-5.370	1.00	0.00
ATOM	529	CD	ARG	35	4.496	21.079	-7.128	1.00	0.00
ATOM	530	HD2	ARG	35	4.507	20.005	-6.943	1.00	0.00
ATOM	531	HD3	ARG	35	3.714	21.316	-7.850	1.00	0.00
ATOM	532	NE	ARG	35	5.795	21.472	-7.696	1.00	0.00
ATOM	533	HE	ARG	35	6.425	21.955	-7.072	1.00	0.00
ATOM	534	CZ	ARG	35	6.207	21.198	-8.917	1.00	0.00
ATOM	535	NH1	ARG	35	5.430	20.574	-9.767	1.00	0.00
ATOM	536	HH11	ARG	35	4.500	20.297	-9.487	1.00	0.00
ATOM	537	HH12	ARG	35	5.765	20.374	-10.699	1.00	0.00
ATOM	538	NH2	ARG	35	7.385	21.598	-9.318	1.00	0.00
ATOM	539	HH21	ARG	35	7.980	22.119	-8.689	1.00	0.00
ATOM	540	HH22	ARG	35	7.695	21.385	-10.256	1.00	0.00
ATOM	541	C	ARG	35	4.380	21.710	-2.612	1.00	0.00
ATOM	542	O	ARG	35	5.433	22.225	-2.276	1.00	0.00
ATOM	543	N	SER	36	3.175	22.141	-2.228	1.00	0.00
ATOM	544	H	SER	36	2.357	21.709	-2.633	1.00	0.00
ATOM	545	CA	SER	36	2.983	23.217	-1.269	1.00	0.00
ATOM	546	HA	SER	36	3.498	22.989	-0.336	1.00	0.00
ATOM	547	CB	SER	36	3.557	24.517	-1.839	1.00	0.00
ATOM	548	HB2	SER	36	4.635	24.405	-1.954	1.00	0.00

ATOM	549	HB3	SER	36	3.106	24.704	-2.814	1.00	0.00
ATOM	550	OG	SER	36	3.288	25.598	-0.985	1.00	0.00
ATOM	551	HG	SER	36	3.656	26.402	-1.361	1.00	0.00
ATOM	552	C	SER	36	1.499	23.385	-0.977	1.00	0.00
ATOM	553	O	SER	36	1.050	23.166	0.141	1.00	0.00
ATOM	554	N	GLN	37	0.717	23.722	-2.009	1.00	0.00
ATOM	555	H	GLN	37	1.132	23.891	-2.915	1.00	0.00
ATOM	556	CA	GLN	37	-0.719	23.920	-1.860	1.00	0.00
ATOM	557	HA	GLN	37	-1.154	23.098	-1.293	1.00	0.00
ATOM	558	CB	GLN	37	-0.989	25.226	-1.112	1.00	0.00
ATOM	559	HB2	GLN	37	-0.265	25.340	-0.305	1.00	0.00
ATOM	560	HB3	GLN	37	-0.898	26.066	-1.802	1.00	0.00
ATOM	561	CG	GLN	37	-2.403	25.197	-0.528	1.00	0.00
ATOM	562	HG2	GLN	37	-3.132	25.366	-1.321	1.00	0.00
ATOM	563	HG3	GLN	37	-2.570	24.216	-0.084	1.00	0.00
ATOM	564	CD	GLN	37	-2.599	26.256	0.545	1.00	0.00
ATOM	565	OE1	GLN	37	-1.697	26.946	0.981	1.00	0.00
ATOM	566	NE2	GLN	37	-3.822	26.425	0.995	1.00	0.00
ATOM	567	HE21	GLN	37	-4.003	27.117	1.708	1.00	0.00
ATOM	568	HE22	GLN	37	-4.575	25.863	0.625	1.00	0.00
ATOM	569	C	GLN	37	-1.401	23.915	-3.220	1.00	0.00
ATOM	570	O	GLN	37	-1.788	24.942	-3.772	1.00	0.00

ATOM	571	N	VAL	38	-1.420	22.744	-3.845	1.00	0.00
ATOM	572	H	VAL	38	-0.910	21.956	-3.473	1.00	0.00
ATOM	573	CA	VAL	38	-2.076	22.594	-5.136	1.00	0.00
ATOM	574	HA	VAL	38	-1.863	23.432	-5.801	1.00	0.00
ATOM	575	CB	VAL	38	-1.612	21.303	-5.813	1.00	0.00
ATOM	576	HB	VAL	38	-2.139	21.178	-6.759	1.00	0.00
ATOM	577	CG1	VAL	38	-0.102	21.379	-6.076	1.00	0.00
ATOM	578	HG11	VAL	38	0.426	21.504	-5.131	1.00	0.00
ATOM	579	HG12	VAL	38	0.230	20.460	-6.559	1.00	0.00
ATOM	580	HG13	VAL	38	0.112	22.228	-6.726	1.00	0.00
ATOM	581	CG2	VAL	38	-1.939	20.066	-4.968	1.00	0.00
ATOM	582	HG21	VAL	38	-3.016	20.004	-4.817	1.00	0.00
ATOM	583	HG22	VAL	38	-1.593	19.171	-5.485	1.00	0.00
ATOM	584	HG23	VAL	38	-1.440	20.144	-4.002	1.00	0.00
ATOM	585	C	VAL	38	-3.587	22.665	-4.983	1.00	0.00
ATOM	586	O	VAL	38	-4.091	22.686	-3.859	1.00	0.00
ATOM	587	N	SER	39	-4.298	22.673	-6.116	1.00	0.00
ATOM	588	H	SER	39	-3.821	22.726	-7.005	1.00	0.00
ATOM	589	CA	SER	39	-5.750	22.697	-6.112	1.00	0.00
ATOM	590	HA	SER	39	-6.099	23.650	-5.714	1.00	0.00
ATOM	591	CB	SER	39	-6.305	22.527	-7.541	1.00	0.00
ATOM	592	HB2	SER	39	-7.139	23.219	-7.661	1.00	0.00

ATOM	593	HB3	SER	39	-5.512	22.790	-8.242	1.00	0.00
ATOM	594	OG	SER	39	-6.747	21.202	-7.802	1.00	0.00
ATOM	595	HG	SER	39	-7.082	21.148	-8.700	1.00	0.00
ATOM	596	C	SER	39	-6.328	21.631	-5.168	1.00	0.00
ATOM	597	O	SER	39	-5.693	20.622	-4.821	1.00	0.00
ATOM	598	N	PRO	40	-7.617	21.771	-4.854	1.00	0.00
ATOM	599	CD	PRO	40	-8.516	22.821	-5.304	1.00	0.00
ATOM	600	HD2	PRO	40	-8.661	22.700	-6.378	1.00	0.00
ATOM	601	HD3	PRO	40	-8.035	23.780	-5.110	1.00	0.00
ATOM	602	CG	PRO	40	-9.810	22.649	-4.514	1.00	0.00
ATOM	603	HG2	PRO	40	-10.621	22.416	-5.204	1.00	0.00
ATOM	604	HG3	PRO	40	-10.035	23.579	-3.992	1.00	0.00
ATOM	605	CB	PRO	40	-9.574	21.511	-3.525	1.00	0.00
ATOM	606	HB2	PRO	40	-10.409	20.812	-3.553	1.00	0.00
ATOM	607	HB3	PRO	40	-9.443	21.880	-2.508	1.00	0.00
ATOM	608	CA	PRO	40	-8.291	20.811	-3.998	1.00	0.00
ATOM	609	HA	PRO	40	-7.659	20.619	-3.132	1.00	0.00
ATOM	610	C	PRO	40	-8.564	19.495	-4.732	1.00	0.00
ATOM	611	O	PRO	40	-8.003	19.165	-5.784	1.00	0.00
ATOM	612	N	ARG	41	-9.421	18.663	-4.147	1.00	0.00
ATOM	613	H	ARG	41	-9.757	18.885	-3.221	1.00	0.00
ATOM	614	CA	ARG	41	-9.842	17.425	-4.747	1.00	0.00

ATOM	615	HA	ARG	41	-8.995	16.948	-5.240	1.00	0.00
ATOM	616	CB	ARG	41	-10.391	16.471	-3.683	1.00	0.00
ATOM	617	HB2	ARG	41	-9.764	16.532	-2.794	1.00	0.00
ATOM	618	HB3	ARG	41	-11.409	16.767	-3.431	1.00	0.00
ATOM	619	CG	ARG	41	-10.393	15.025	-4.211	1.00	0.00
ATOM	620	HG2	ARG	41	-9.811	15.005	-5.133	1.00	0.00
ATOM	621	HG3	ARG	41	-9.911	14.395	-3.464	1.00	0.00
ATOM	622	CD	ARG	41	-11.800	14.502	-4.487	1.00	0.00
ATOM	623	HD2	ARG	41	-12.527	15.294	-4.305	1.00	0.00
ATOM	624	HD3	ARG	41	-11.872	14.175	-5.525	1.00	0.00
ATOM	625	NE	ARG	41	-12.111	13.371	-3.617	1.00	0.00
ATOM	626	HE	ARG	41	-12.039	12.453	-4.030	1.00	0.00
ATOM	627	CZ	ARG	41	-12.571	13.473	-2.383	1.00	0.00
ATOM	628	NH1	ARG	41	-12.721	14.644	-1.811	1.00	0.00
ATOM	629	HH11	ARG	41	-12.482	15.485	-2.318	1.00	0.00
ATOM	630	HH12	ARG	41	-13.074	14.700	-0.867	1.00	0.00
ATOM	631	NH2	ARG	41	-12.779	12.394	-1.682	1.00	0.00
ATOM	632	HH21	ARG	41	-12.587	11.489	-2.086	1.00	0.00
ATOM	633	HH22	ARG	41	-13.131	12.469	-0.739	1.00	0.00
ATOM	634	C	ARG	41	-10.871	17.688	-5.823	1.00	0.00
ATOM	635	O	ARG	41	-11.884	17.033	-5.815	1.00	0.00
ATOM	636	N	MET	42	-10.623	18.620	-6.730	1.00	0.00

ATOM 637 H MET 42 -9.768 19.155 -6.664 1.00 0.00
ATOM 638 CA MET 42 -11.535 18.911 -7.824 1.00 0.00
ATOM 639 HA MET 42 -12.410 19.451 -7.462 1.00 0.00
ATOM 640 CB MET 42 -10.811 19.785 -8.849 1.00 0.00
ATOM 641 HB2 MET 42 -11.431 19.858 -9.743 1.00 0.00
ATOM 642 HB3 MET 42 -10.672 20.778 -8.422 1.00 0.00
ATOM 643 CG MET 42 -9.443 19.193 -9.223 1.00 0.00
ATOM 644 HG2 MET 42 -8.701 19.598 -8.534 1.00 0.00
ATOM 645 HG3 MET 42 -9.500 18.112 -9.101 1.00 0.00
ATOM 646 SD MET 42 -8.950 19.573 -10.913 1.00 0.00
ATOM 647 CE MET 42 -8.477 21.305 -10.661 1.00 0.00
ATOM 648 HE1 MET 42 -7.672 21.360 -9.928 1.00 0.00
ATOM 649 HE2 MET 42 -8.136 21.731 -11.606 1.00 0.00
ATOM 650 HE3 MET 42 -9.337 21.868 -10.299 1.00 0.00
ATOM 651 C MET 42 -12.023 17.638 -8.527 1.00 0.00
ATOM 652 O MET 42 -13.119 17.633 -9.060 1.00 0.00
ATOM 653 N ARG 43 -11.193 16.577 -8.509 1.00 0.00
ATOM 654 H ARG 43 -10.253 16.708 -8.164 1.00 0.00
ATOM 655 CA ARG 43 -11.553 15.287 -9.034 1.00 0.00
ATOM 656 HA ARG 43 -11.691 15.354 -10.114 1.00 0.00
ATOM 657 CB ARG 43 -10.451 14.261 -8.744 1.00 0.00
ATOM 658 HB2 ARG 43 -9.530 14.779 -8.473 1.00 0.00

ATOM 659 HB3 ARG 43 -10.759 13.615 -7.923 1.00 0.00
ATOM 660 CG ARG 43 -10.210 13.414 -9.998 1.00 0.00
ATOM 661 HG2 ARG 43 -9.915 12.409 -9.697 1.00 0.00
ATOM 662 HG3 ARG 43 -11.133 13.364 -10.576 1.00 0.00
ATOM 663 CD ARG 43 -9.101 14.036 -10.859 1.00 0.00
ATOM 664 HD2 ARG 43 -9.137 13.609 -11.862 1.00 0.00
ATOM 665 HD3 ARG 43 -9.250 15.115 -10.919 1.00 0.00
ATOM 666 NE ARG 43 -7.765 13.773 -10.279 1.00 0.00
ATOM 667 HE ARG 43 -7.705 12.993 -9.642 1.00 0.00
ATOM 668 CZ ARG 43 -6.657 14.423 -10.585 1.00 0.00
ATOM 669 NH1 ARG 43 -6.674 15.415 -11.434 1.00 0.00
ATOM 670 HH11 ARG 43 -7.544 15.693 -11.865 1.00 0.00
ATOM 671 HH12 ARG 43 -5.816 15.901 -11.656 1.00 0.00
ATOM 672 NH2 ARG 43 -5.532 14.129 -9.977 1.00 0.00
ATOM 673 HH21 ARG 43 -5.516 13.404 -9.275 1.00 0.00
ATOM 674 HH22 ARG 43 -4.687 14.630 -10.215 1.00 0.00
ATOM 675 C ARG 43 -12.894 14.842 -8.471 1.00 0.00
ATOM 676 O ARG 43 -13.137 14.952 -7.277 1.00 0.00
ATOM 677 N GLU 44 -13.685 14.218 -9.306 1.00 0.00
ATOM 678 H GLU 44 -13.422 14.137 -10.278 1.00 0.00
ATOM 679 CA GLU 44 -14.972 13.672 -8.890 1.00 0.00
ATOM 680 HA GLU 44 -15.189 13.968 -7.864 1.00 0.00

ATOM	681	CB	GLU	44	-16.082	14.204	-9.797	1.00	0.00
ATOM	682	HB2	GLU	44	-17.026	13.762	-9.481	1.00	0.00
ATOM	683	HB3	GLU	44	-16.131	15.287	-9.682	1.00	0.00
ATOM	684	CG	GLU	44	-15.823	13.857	-11.277	1.00	0.00
ATOM	685	HG2	GLU	44	-15.656	14.755	-11.873	1.00	0.00
ATOM	686	HG3	GLU	44	-14.920	13.247	-11.280	1.00	0.00
ATOM	687	CD	GLU	44	-16.982	13.056	-11.875	1.00	0.00
ATOM	688	OE1	GLU	44	-16.992	11.826	-11.645	1.00	0.00
ATOM	689	OE2	GLU	44	-17.861	13.709	-12.484	1.00	0.00
ATOM	690	C	GLU	44	-14.949	12.142	-8.917	1.00	0.00
ATOM	691	O	GLU	44	-15.680	11.500	-8.172	1.00	0.00
ATOM	692	N	GLU	45	-13.997	11.569	-9.662	1.00	0.00
ATOM	693	H	GLU	45	-13.349	12.152	-10.173	1.00	0.00
ATOM	694	CA	GLU	45	-13.890	10.136	-9.808	1.00	0.00
ATOM	695	HA	GLU	45	-14.886	9.697	-9.857	1.00	0.00
ATOM	696	CB	GLU	45	-13.131	9.775	-11.094	1.00	0.00
ATOM	697	HB2	GLU	45	-12.604	10.658	-11.456	1.00	0.00
ATOM	698	HB3	GLU	45	-12.411	8.988	-10.874	1.00	0.00
ATOM	699	CG	GLU	45	-14.112	9.285	-12.174	1.00	0.00
ATOM	700	HG2	GLU	45	-14.357	8.224	-12.195	1.00	0.00
ATOM	701	HG3	GLU	45	-14.987	9.854	-11.860	1.00	0.00
ATOM	702	CD	GLU	45	-13.686	9.737	-13.572	1.00	0.00

ATOM 703 OE1 GLU 45 -13.773 10.954 -13.844 1.00 0.00
ATOM 704 OE2 GLU 45 -13.059 8.902 -14.267 1.00 0.00
ATOM 705 C GLU 45 -13.191 9.594 -8.572 1.00 0.00
ATOM 706 O GLU 45 -12.013 9.898 -8.311 1.00 0.00
ATOM 707 N ASP 46 -13.908 8.761 -7.826 1.00 0.00
ATOM 708 H ASP 46 -14.853 8.538 -8.103 1.00 0.00
ATOM 709 CA ASP 46 -13.385 8.158 -6.619 1.00 0.00
ATOM 710 HA ASP 46 -12.984 8.934 -5.967 1.00 0.00
ATOM 711 CB ASP 46 -14.486 7.405 -5.866 1.00 0.00
ATOM 712 HB2 ASP 46 -15.361 8.038 -6.013 1.00 0.00
ATOM 713 HB3 ASP 46 -14.674 6.421 -6.295 1.00 0.00
ATOM 714 CG ASP 46 -14.204 7.264 -4.367 1.00 0.00
ATOM 715 OD1 ASP 46 -13.177 7.819 -3.898 1.00 0.00
ATOM 716 OD2 ASP 46 -14.950 6.514 -3.707 1.00 0.00
ATOM 717 C ASP 46 -12.275 7.181 -6.992 1.00 0.00
ATOM 718 O ASP 46 -12.340 6.440 -7.985 1.00 0.00
ATOM 719 N HID 47 -11.197 7.162 -6.220 1.00 0.00
ATOM 720 H HID 47 -11.115 7.822 -5.460 1.00 0.00
ATOM 721 CA HID 47 -10.086 6.258 -6.470 1.00 0.00
ATOM 722 HA HID 47 -10.325 5.587 -7.295 1.00 0.00
ATOM 723 CB HID 47 -8.844 7.060 -6.840 1.00 0.00
ATOM 724 HB2 HID 47 -8.666 7.862 -6.123 1.00 0.00

```
ATOM    725  HB3 HID    47      -7.967   6.415  -6.889  1.00  0.00
ATOM    726  CG  HID    47      -8.974   7.715  -8.197  1.00  0.00
ATOM    727  ND1 HID    47      -9.953   7.415  -9.136  1.00  0.00
ATOM    728  HD1 HID    47     -10.676   6.717  -9.039  1.00  0.00
ATOM    729  CE1 HID    47      -9.768   8.219 -10.200  1.00  0.00
ATOM    730  HE1 HID    47     -10.450   8.173 -11.050  1.00  0.00
ATOM    731  NE2 HID    47      -8.674   8.991  -9.978  1.00  0.00
ATOM    732  CD2 HID    47      -8.164   8.692  -8.723  1.00  0.00
ATOM    733  HD2 HID    47      -7.281   9.215  -8.355  1.00  0.00
ATOM    734  C   HID    47      -9.868   5.410  -5.253  1.00  0.00
ATOM    735  O   HID    47      -9.645   5.965  -4.187  1.00  0.00
ATOM    736  N   LYS    48      -9.852   4.089  -5.453  1.00  0.00
ATOM    737  H   LYS    48     -10.033   3.753  -6.388  1.00  0.00
ATOM    738  CA  LYS    48      -9.645   3.110  -4.394  1.00  0.00
ATOM    739  HA  LYS    48      -8.612   3.149  -4.047  1.00  0.00
ATOM    740  CB  LYS    48     -10.570   3.411  -3.212  1.00  0.00
ATOM    741  HB2 LYS    48     -11.146   4.310  -3.434  1.00  0.00
ATOM    742  HB3 LYS    48     -11.249   2.570  -3.072  1.00  0.00
ATOM    743  CG  LYS    48      -9.755   3.630  -1.931  1.00  0.00
ATOM    744  HG2 LYS    48      -9.104   2.769  -1.781  1.00  0.00
ATOM    745  HG3 LYS    48      -9.147   4.527  -2.055  1.00  0.00
ATOM    746  CD  LYS    48     -10.671   3.797  -0.712  1.00  0.00
```

ATOM	747	HD2	LYS	48	-10.083	4.187	0.119	1.00	0.00
ATOM	748	HD3	LYS	48	-11.462	4.505	-0.961	1.00	0.00
ATOM	749	CE	LYS	48	-11.295	2.451	-0.311	1.00	0.00
ATOM	750	HE2	LYS	48	-10.557	1.663	-0.463	1.00	0.00
ATOM	751	HE3	LYS	48	-11.571	2.492	0.742	1.00	0.00
ATOM	752	NZ	LYS	48	-12.502	2.163	-1.128	1.00	0.00
ATOM	753	HZ1	LYS	48	-12.246	2.125	-2.105	1.00	0.00
ATOM	754	HZ2	LYS	48	-12.894	1.276	-0.848	1.00	0.00
ATOM	755	HZ3	LYS	48	-13.186	2.893	-0.988	1.00	0.00
ATOM	756	C	LYS	48	-9.877	1.700	-4.909	1.00	0.00
ATOM	757	O	LYS	48	-9.168	0.777	-4.548	1.00	0.00
ATOM	758	N	GLN	49	-10.797	1.564	-5.859	1.00	0.00
ATOM	759	H	GLN	49	-11.313	2.373	-6.178	1.00	0.00
ATOM	760	CA	GLN	49	-11.077	0.294	-6.469	1.00	0.00
ATOM	761	HA	GLN	49	-10.873	-0.502	-5.754	1.00	0.00
ATOM	762	CB	GLN	49	-12.531	0.195	-6.906	1.00	0.00
ATOM	763	HB2	GLN	49	-12.986	1.186	-6.881	1.00	0.00
ATOM	764	HB3	GLN	49	-12.579	-0.200	-7.921	1.00	0.00
ATOM	765	CG	GLN	49	-13.287	-0.736	-5.961	1.00	0.00
ATOM	766	HG2	GLN	49	-12.804	-1.712	-5.991	1.00	0.00
ATOM	767	HG3	GLN	49	-13.250	-0.343	-4.945	1.00	0.00
ATOM	768	CD	GLN	49	-14.743	-0.901	-6.365	1.00	0.00

ATOM	769	OE1	GLN	49	-15.183	-0.490	-7.419	1.00	0.00
ATOM	770	NE2	GLN	49	-15.525	-1.499	-5.497	1.00	0.00
ATOM	771	HE21	GLN	49	-16.502	-1.635	-5.713	1.00	0.00
ATOM	772	HE22	GLN	49	-15.146	-1.819	-4.618	1.00	0.00
ATOM	773	C	GLN	49	-10.214	0.136	-7.682	1.00	0.00
ATOM	774	O	GLN	49	-9.864	-0.976	-7.936	1.00	0.00
ATOM	775	N	LEU	50	-9.746	1.186	-8.350	1.00	0.00
ATOM	776	H	LEU	50	-10.064	2.102	-8.067	1.00	0.00
ATOM	777	CA	LEU	50	-8.851	1.091	-9.491	1.00	0.00
ATOM	778	HA	LEU	50	-9.265	0.433	-10.255	1.00	0.00
ATOM	779	CB	LEU	50	-8.680	2.496	-10.093	1.00	0.00
ATOM	780	HB2	LEU	50	-8.793	3.189	-9.259	1.00	0.00
ATOM	781	HB3	LEU	50	-7.678	2.594	-10.513	1.00	0.00
ATOM	782	CG	LEU	50	-9.723	2.823	-11.176	1.00	0.00
ATOM	783	HG	LEU	50	-10.662	2.323	-10.939	1.00	0.00
ATOM	784	CD1	LEU	50	-9.951	4.338	-11.229	1.00	0.00
ATOM	785	HD11	LEU	50	-9.013	4.839	-11.467	1.00	0.00
ATOM	786	HD12	LEU	50	-10.690	4.569	-11.997	1.00	0.00
ATOM	787	HD13	LEU	50	-10.313	4.686	-10.262	1.00	0.00
ATOM	788	CD2	LEU	50	-9.273	2.294	-12.540	1.00	0.00
ATOM	789	HD21	LEU	50	-9.148	1.213	-12.488	1.00	0.00
ATOM	790	HD22	LEU	50	-10.025	2.537	-13.291	1.00	0.00

ATOM	791	HD23	LEU	50	-8.325	2.756	-12.815	1.00	0.00
ATOM	792	C	LEU	50	-7.512	0.491	-9.059	1.00	0.00
ATOM	793	O	LEU	50	-6.828	-0.103	-9.871	1.00	0.00
ATOM	794	N	CYX	51	-7.173	0.594	-7.771	1.00	0.00
ATOM	795	H	CYX	51	-7.748	1.155	-7.159	1.00	0.00
ATOM	796	CA	CYX	51	-5.986	-0.016	-7.226	1.00	0.00
ATOM	797	HA	CYX	51	-5.213	-0.077	-7.992	1.00	0.00
ATOM	798	CB	CYX	51	-5.460	0.811	-6.073	1.00	0.00
ATOM	799	HB2	CYX	51	-4.374	0.730	-6.033	1.00	0.00
ATOM	800	HB3	CYX	51	-5.740	1.855	-6.217	1.00	0.00
ATOM	801	SG	CYX	51	-6.016	0.428	-4.381	1.00	0.00
ATOM	802	C	CYX	51	-6.279	-1.424	-6.788	1.00	0.00
ATOM	803	O	CYX	51	-5.571	-2.303	-7.235	1.00	0.00
ATOM	804	N	CYX	52	-7.385	-1.647	-6.066	1.00	0.00
ATOM	805	H	CYX	52	-7.921	-0.854	-5.743	1.00	0.00
ATOM	806	CA	CYX	52	-7.794	-2.976	-5.642	1.00	0.00
ATOM	807	HA	CYX	52	-6.933	-3.526	-5.262	1.00	0.00
ATOM	808	CB	CYX	52	-8.845	-2.877	-4.528	1.00	0.00
ATOM	809	HB2	CYX	52	-9.435	-1.970	-4.664	1.00	0.00
ATOM	810	HB3	CYX	52	-9.501	-3.746	-4.569	1.00	0.00
ATOM	811	SG	CYX	52	-8.097	-2.860	-2.867	1.00	0.00
ATOM	812	C	CYX	52	-8.290	-3.787	-6.844	1.00	0.00

ATOM 813 O CYX 52 -8.624 -4.954 -6.702 1.00 0.00
ATOM 814 N MET 53 -8.349 -3.174 -8.025 1.00 0.00
ATOM 815 H MET 53 -8.026 -2.217 -8.061 1.00 0.00
ATOM 816 CA MET 53 -8.850 -3.770 -9.234 1.00 0.00
ATOM 817 HA MET 53 -9.508 -4.601 -8.984 1.00 0.00
ATOM 818 CB MET 53 -9.640 -2.754 -10.066 1.00 0.00
ATOM 819 HB2 MET 53 -10.601 -2.581 -9.582 1.00 0.00
ATOM 820 HB3 MET 53 -9.077 -1.820 -10.099 1.00 0.00
ATOM 821 CG MET 53 -9.870 -3.255 -11.481 1.00 0.00
ATOM 822 HG2 MET 53 -9.631 -4.317 -11.529 1.00 0.00
ATOM 823 HG3 MET 53 -10.916 -3.105 -11.749 1.00 0.00
ATOM 824 SD MET 53 -8.824 -2.359 -12.651 1.00 0.00
ATOM 825 CE MET 53 -8.500 -3.699 -13.816 1.00 0.00
ATOM 826 HE1 MET 53 -9.442 -4.054 -14.233 1.00 0.00
ATOM 827 HE2 MET 53 -7.861 -3.334 -14.621 1.00 0.00
ATOM 828 HE3 MET 53 -8.000 -4.518 -13.299 1.00 0.00
ATOM 829 C MET 53 -7.660 -4.275 -10.013 1.00 0.00
ATOM 830 O MET 53 -7.648 -5.429 -10.407 1.00 0.00
ATOM 831 N GLN 54 -6.697 -3.399 -10.291 1.00 0.00
ATOM 832 H GLN 54 -6.753 -2.445 -9.963 1.00 0.00
ATOM 833 CA GLN 54 -5.538 -3.791 -11.063 1.00 0.00
ATOM 834 HA GLN 54 -5.854 -4.329 -11.957 1.00 0.00

ATOM	835	CB	GLN	54	-4.748	-2.574	-11.487	1.00	0.00
ATOM	836	HB2	GLN	54	-4.835	-1.823	-10.702	1.00	0.00
ATOM	837	HB3	GLN	54	-3.704	-2.868	-11.593	1.00	0.00
ATOM	838	CG	GLN	54	-5.244	-1.994	-12.797	1.00	0.00
ATOM	839	HG2	GLN	54	-4.496	-2.181	-13.568	1.00	0.00
ATOM	840	HG3	GLN	54	-6.169	-2.509	-13.056	1.00	0.00
ATOM	841	CD	GLN	54	-5.520	-0.499	-12.741	1.00	0.00
ATOM	842	OE1	GLN	54	-4.828	0.312	-12.166	1.00	0.00
ATOM	843	NE2	GLN	54	-6.572	-0.055	-13.370	1.00	0.00
ATOM	844	HE21	GLN	54	-6.787	0.932	-13.355	1.00	0.00
ATOM	845	HE22	GLN	54	-7.167	-0.699	-13.870	1.00	0.00
ATOM	846	C	GLN	54	-4.637	-4.673	-10.238	1.00	0.00
ATOM	847	O	GLN	54	-4.023	-5.555	-10.807	1.00	0.00
ATOM	848	N	LEU	55	-4.593	-4.499	-8.915	1.00	0.00
ATOM	849	H	LEU	55	-5.147	-3.769	-8.490	1.00	0.00
ATOM	850	CA	LEU	55	-3.759	-5.322	-8.055	1.00	0.00
ATOM	851	HA	LEU	55	-2.705	-5.189	-8.302	1.00	0.00
ATOM	852	CB	LEU	55	-3.976	-4.902	-6.611	1.00	0.00
ATOM	853	HB2	LEU	55	-4.949	-4.413	-6.650	1.00	0.00
ATOM	854	HB3	LEU	55	-4.037	-5.782	-5.971	1.00	0.00
ATOM	855	CG	LEU	55	-2.930	-3.921	-6.038	1.00	0.00
ATOM	856	HG	LEU	55	-2.380	-3.496	-6.879	1.00	0.00

ATOM	857	CD1	LEU	55	-3.555	-2.786	-5.209	1.00	0.00
ATOM	858	HD11	LEU	55	-4.104	-3.209	-4.369	1.00	0.00
ATOM	859	HD12	LEU	55	-2.767	-2.132	-4.836	1.00	0.00
ATOM	860	HD13	LEU	55	-4.237	-2.211	-5.836	1.00	0.00
ATOM	861	CD2	LEU	55	-1.872	-4.664	-5.217	1.00	0.00
ATOM	862	HD21	LEU	55	-1.361	-5.387	-5.852	1.00	0.00
ATOM	863	HD22	LEU	55	-1.148	-3.949	-4.826	1.00	0.00
ATOM	864	HD23	LEU	55	-2.353	-5.184	-4.389	1.00	0.00
ATOM	865	C	LEU	55	-4.134	-6.784	-8.241	1.00	0.00
ATOM	866	O	LEU	55	-3.285	-7.640	-8.095	1.00	0.00
ATOM	867	N	LYS	56	-5.383	-7.062	-8.616	1.00	0.00
ATOM	868	H	LYS	56	-6.089	-6.340	-8.577	1.00	0.00
ATOM	869	CA	LYS	56	-5.791	-8.400	-8.978	1.00	0.00
ATOM	870	HA	LYS	56	-5.473	-9.117	-8.222	1.00	0.00
ATOM	871	CB	LYS	56	-7.314	-8.437	-9.076	1.00	0.00
ATOM	872	HB2	LYS	56	-7.736	-8.189	-8.103	1.00	0.00
ATOM	873	HB3	LYS	56	-7.638	-7.699	-9.811	1.00	0.00
ATOM	874	CG	LYS	56	-7.796	-9.827	-9.502	1.00	0.00
ATOM	875	HG2	LYS	56	-8.099	-9.789	-10.549	1.00	0.00
ATOM	876	HG3	LYS	56	-6.975	-10.535	-9.387	1.00	0.00
ATOM	877	CD	LYS	56	-8.980	-10.276	-8.645	1.00	0.00
ATOM	878	HD2	LYS	56	-8.604	-10.741	-7.735	1.00	0.00

ATOM 879 HD3 LYS 56 -9.582 -9.405 -8.386 1.00 0.00
ATOM 880 CE LYS 56 -9.843 -11.282 -9.414 1.00 0.00
ATOM 881 HE2 LYS 56 -9.796 -11.054 -10.479 1.00 0.00
ATOM 882 HE3 LYS 56 -9.461 -12.288 -9.241 1.00 0.00
ATOM 883 NZ LYS 56 -11.250 -11.202 -8.954 1.00 0.00
ATOM 884 HZ1 LYS 56 -11.604 -10.270 -9.115 1.00 0.00
ATOM 885 HZ2 LYS 56 -11.810 -11.868 -9.467 1.00 0.00
ATOM 886 HZ3 LYS 56 -11.294 -11.413 -7.968 1.00 0.00
ATOM 887 C LYS 56 -5.113 -8.889 -10.255 1.00 0.00
ATOM 888 O LYS 56 -4.727 -10.044 -10.307 1.00 0.00
ATOM 889 N ASN 57 -5.077 -8.052 -11.292 1.00 0.00
ATOM 890 H ASN 57 -5.601 -7.189 -11.261 1.00 0.00
ATOM 891 CA ASN 57 -4.333 -8.360 -12.496 1.00 0.00
ATOM 892 HA ASN 57 -4.766 -9.232 -12.985 1.00 0.00
ATOM 893 CB ASN 57 -4.379 -7.172 -13.478 1.00 0.00
ATOM 894 HB2 ASN 57 -4.683 -6.252 -12.979 1.00 0.00
ATOM 895 HB3 ASN 57 -3.362 -7.061 -13.854 1.00 0.00
ATOM 896 CG ASN 57 -5.302 -7.413 -14.648 1.00 0.00
ATOM 897 OD1 ASN 57 -5.715 -8.510 -14.974 1.00 0.00
ATOM 898 ND2 ASN 57 -5.718 -6.342 -15.284 1.00 0.00
ATOM 899 HD21 ASN 57 -6.338 -6.437 -16.076 1.00 0.00
ATOM 900 HD22 ASN 57 -5.416 -5.427 -14.979 1.00 0.00

ATOM	901	C	ASN	57	-2.870	-8.709	-12.221	1.00	0.00
ATOM	902	O	ASN	57	-2.269	-9.403	-13.033	1.00	0.00
ATOM	903	N	LEU	58	-2.293	-8.096	-11.181	1.00	0.00
ATOM	904	H	LEU	58	-2.774	-7.337	-10.719	1.00	0.00
ATOM	905	CA	LEU	58	-0.943	-8.399	-10.772	1.00	0.00
ATOM	906	HA	LEU	58	-0.266	-8.342	-11.625	1.00	0.00
ATOM	907	CB	LEU	58	-0.489	-7.377	-9.716	1.00	0.00
ATOM	908	HB2	LEU	58	-1.380	-7.116	-9.145	1.00	0.00
ATOM	909	HB3	LEU	58	0.229	-7.873	-9.063	1.00	0.00
ATOM	910	CG	LEU	58	0.134	-6.115	-10.285	1.00	0.00
ATOM	911	HG	LEU	58	-0.620	-5.566	-10.850	1.00	0.00
ATOM	912	CD1	LEU	58	0.665	-5.231	-9.150	1.00	0.00
ATOM	913	HD11	LEU	58	1.420	-5.778	-8.585	1.00	0.00
ATOM	914	HD12	LEU	58	1.110	-4.328	-9.570	1.00	0.00
ATOM	915	HD13	LEU	58	-0.156	-4.956	-8.488	1.00	0.00
ATOM	916	CD2	LEU	58	1.258	-6.460	-11.217	1.00	0.00
ATOM	917	HD21	LEU	58	0.877	-7.068	-12.038	1.00	0.00
ATOM	918	HD22	LEU	58	1.694	-5.544	-11.617	1.00	0.00
ATOM	919	HD23	LEU	58	2.021	-7.018	-10.676	1.00	0.00
ATOM	920	C	LEU	58	-0.809	-9.853	-10.300	1.00	0.00
ATOM	921	O	LEU	58	-1.719	-10.672	-10.396	1.00	0.00
ATOM	922	N	ASP	59	0.366	-10.183	-9.787	1.00	0.00

ATOM 923 H ASP 59 1.112 -9.503 -9.736 1.00 0.00
ATOM 924 CA ASP 59 0.618 -11.514 -9.291 1.00 0.00
ATOM 925 HA ASP 59 -0.059 -12.222 -9.767 1.00 0.00
ATOM 926 CB ASP 59 2.053 -11.928 -9.601 1.00 0.00
ATOM 927 HB2 ASP 59 2.594 -11.167 -9.039 1.00 0.00
ATOM 928 HB3 ASP 59 2.334 -12.916 -9.238 1.00 0.00
ATOM 929 CG ASP 59 2.404 -11.812 -11.081 1.00 0.00
ATOM 930 OD1 ASP 59 1.643 -12.360 -11.897 1.00 0.00
ATOM 931 OD2 ASP 59 3.527 -11.332 -11.340 1.00 0.00
ATOM 932 C ASP 59 0.404 -11.541 -7.791 1.00 0.00
ATOM 933 O ASP 59 0.734 -10.577 -7.097 1.00 0.00
ATOM 934 N GLU 60 0.162 -12.732 -7.248 1.00 0.00
ATOM 935 H GLU 60 0.093 -13.548 -7.839 1.00 0.00
ATOM 936 CA GLU 60 -0.036 -12.899 -5.808 1.00 0.00
ATOM 937 HA GLU 60 -0.699 -12.119 -5.434 1.00 0.00
ATOM 938 CB GLU 60 -0.666 -14.265 -5.507 1.00 0.00
ATOM 939 HB2 GLU 60 -1.019 -14.708 -6.438 1.00 0.00
ATOM 940 HB3 GLU 60 0.086 -14.914 -5.058 1.00 0.00
ATOM 941 CG GLU 60 -1.845 -14.102 -4.539 1.00 0.00
ATOM 942 HG2 GLU 60 -1.563 -13.331 -3.821 1.00 0.00
ATOM 943 HG3 GLU 60 -2.740 -13.787 -5.076 1.00 0.00
ATOM 944 CD GLU 60 -2.128 -15.413 -3.797 1.00 0.00

ATOM	945	OE1	GLU	60	-1.515	-15.605	-2.722	1.00	0.00
ATOM	946	OE2	GLU	60	-2.917	-16.218	-4.336	1.00	0.00
ATOM	947	C	GLU	60	1.298	-12.780	-5.071	1.00	0.00
ATOM	948	O	GLU	60	1.361	-12.435	-3.896	1.00	0.00
ATOM	949	N	LYS	61	2.398	-13.025	-5.791	1.00	0.00
ATOM	950	H	LYS	61	2.304	-13.402	-6.723	1.00	0.00
ATOM	951	CA	LYS	61	3.723	-12.856	-5.253	1.00	0.00
ATOM	952	HA	LYS	61	3.712	-12.974	-4.170	1.00	0.00
ATOM	953	CB	LYS	61	4.649	-13.916	-5.851	1.00	0.00
ATOM	954	HB2	LYS	61	5.555	-13.957	-5.246	1.00	0.00
ATOM	955	HB3	LYS	61	4.139	-14.878	-5.807	1.00	0.00
ATOM	956	CG	LYS	61	5.021	-13.601	-7.306	1.00	0.00
ATOM	957	HG2	LYS	61	4.109	-13.392	-7.866	1.00	0.00
ATOM	958	HG3	LYS	61	5.666	-12.722	-7.323	1.00	0.00
ATOM	959	CD	LYS	61	5.754	-14.783	-7.948	1.00	0.00
ATOM	960	HD2	LYS	61	6.682	-14.424	-8.394	1.00	0.00
ATOM	961	HD3	LYS	61	5.982	-15.519	-7.178	1.00	0.00
ATOM	962	CE	LYS	61	4.883	-15.431	-9.032	1.00	0.00
ATOM	963	HE2	LYS	61	4.226	-14.672	-9.458	1.00	0.00
ATOM	964	HE3	LYS	61	5.530	-15.830	-9.814	1.00	0.00
ATOM	965	NZ	LYS	61	4.065	-16.528	-8.458	1.00	0.00
ATOM	966	HZ1	LYS	61	3.466	-16.157	-7.735	1.00	0.00

```
ATOM    967  HZ2 LYS    61       3.499 -16.941  -9.186  1.00  0.00
ATOM    968  HZ3 LYS    61       4.674 -17.231  -8.064  1.00  0.00
ATOM    969  C   LYS    61       4.210 -11.452  -5.501  1.00  0.00
ATOM    970  O   LYS    61       5.276 -11.159  -5.015  1.00  0.00
ATOM    971  N   CYX    62       3.528 -10.638  -6.300  1.00  0.00
ATOM    972  H   CYX    62       2.744 -11.032  -6.800  1.00  0.00
ATOM    973  CA  CYX    62       3.860  -9.251  -6.532  1.00  0.00
ATOM    974  HA  CYX    62       4.889  -9.049  -6.236  1.00  0.00
ATOM    975  CB  CYX    62       3.714  -8.949  -8.009  1.00  0.00
ATOM    976  HB2 CYX    62       2.763  -9.342  -8.369  1.00  0.00
ATOM    977  HB3 CYX    62       3.743  -7.870  -8.165  1.00  0.00
ATOM    978  SG  CYX    62       5.008  -9.697  -9.036  1.00  0.00
ATOM    979  C   CYX    62       2.951  -8.357  -5.686  1.00  0.00
ATOM    980  O   CYX    62       3.263  -7.205  -5.413  1.00  0.00
ATOM    981  N   MET    63       1.897  -8.895  -5.107  1.00  0.00
ATOM    982  H   MET    63       1.702  -9.874  -5.260  1.00  0.00
ATOM    983  CA  MET    63       0.986  -8.137  -4.294  1.00  0.00
ATOM    984  HA  MET    63       0.895  -7.116  -4.665  1.00  0.00
ATOM    985  CB  MET    63      -0.372  -8.796  -4.354  1.00  0.00
ATOM    986  HB2 MET    63      -0.230  -9.868  -4.218  1.00  0.00
ATOM    987  HB3 MET    63      -0.971  -8.402  -3.534  1.00  0.00
ATOM    988  CG  MET    63      -1.088  -8.544  -5.669  1.00  0.00
```

```
ATOM    989  HG2 MET    63      -1.731  -7.673  -5.543  1.00  0.00
ATOM    990  HG3 MET    63      -0.336  -8.332  -6.430  1.00  0.00
ATOM    991  SD  MET    63      -2.088  -9.949  -6.199  1.00  0.00
ATOM    992  CE  MET    63      -2.946 -10.327  -4.668  1.00  0.00
ATOM    993  HE1 MET    63      -3.531  -9.462  -4.356  1.00  0.00
ATOM    994  HE2 MET    63      -3.610 -11.177  -4.824  1.00  0.00
ATOM    995  HE3 MET    63      -2.218 -10.571  -3.894  1.00  0.00
ATOM    996  C   MET    63       1.522  -8.085  -2.888  1.00  0.00
ATOM    997  O   MET    63       0.944  -8.595  -1.971  1.00  0.00
ATOM    998  N   CYX    64       2.666  -7.498  -2.645  1.00  0.00
ATOM    999  H   CYX    64       3.163  -7.115  -3.438  1.00  0.00
ATOM   1000  CA  CYX    64       3.296  -7.425  -1.335  1.00  0.00
ATOM   1001  HA  CYX    64       2.722  -6.733  -0.719  1.00  0.00
ATOM   1002  CB  CYX    64       3.372  -8.784  -0.611  1.00  0.00
ATOM   1003  HB2 CYX    64       2.555  -8.860   0.106  1.00  0.00
ATOM   1004  HB3 CYX    64       3.290  -9.589  -1.341  1.00  0.00
ATOM   1005  SG  CYX    64       4.914  -9.098   0.341  1.00  0.00
ATOM   1006  C   CYX    64       4.670  -6.830  -1.518  1.00  0.00
ATOM   1007  O   CYX    64       4.932  -5.754  -0.979  1.00  0.00
ATOM   1008  N   PRO    65       5.521  -7.496  -2.320  1.00  0.00
ATOM   1009  CD  PRO    65       5.262  -8.745  -3.024  1.00  0.00
ATOM   1010  HD2 PRO    65       4.523  -8.538  -3.798  1.00  0.00
```

ATOM 1011 HD3 PRO 65 4.846 -9.448 -2.303 1.00 0.00
ATOM 1012 CG PRO 65 6.583 -9.215 -3.593 1.00 0.00
ATOM 1013 HG2 PRO 65 6.515 -9.226 -4.681 1.00 0.00
ATOM 1014 HG3 PRO 65 6.784 -10.223 -3.232 1.00 0.00
ATOM 1015 CB PRO 65 7.645 -8.249 -3.116 1.00 0.00
ATOM 1016 HB2 PRO 65 8.246 -7.943 -3.973 1.00 0.00
ATOM 1017 HB3 PRO 65 8.295 -8.666 -2.347 1.00 0.00
ATOM 1018 CA PRO 65 6.881 -7.040 -2.557 1.00 0.00
ATOM 1019 HA PRO 65 7.317 -6.720 -1.611 1.00 0.00
ATOM 1020 C PRO 65 6.892 -5.875 -3.540 1.00 0.00
ATOM 1021 O PRO 65 7.697 -4.965 -3.400 1.00 0.00
ATOM 1022 N ALA 66 5.980 -5.893 -4.512 1.00 0.00
ATOM 1023 H ALA 66 5.355 -6.682 -4.593 1.00 0.00
ATOM 1024 CA ALA 66 5.866 -4.838 -5.469 1.00 0.00
ATOM 1025 HA ALA 66 6.836 -4.564 -5.886 1.00 0.00
ATOM 1026 CB ALA 66 4.955 -5.261 -6.601 1.00 0.00
ATOM 1027 HB1 ALA 66 3.978 -5.529 -6.201 1.00 0.00
ATOM 1028 HB2 ALA 66 4.845 -4.437 -7.307 1.00 0.00
ATOM 1029 HB3 ALA 66 5.386 -6.121 -7.113 1.00 0.00
ATOM 1030 C ALA 66 5.319 -3.625 -4.777 1.00 0.00
ATOM 1031 O ALA 66 5.924 -2.594 -4.924 1.00 0.00
ATOM 1032 N ILE 67 4.237 -3.727 -4.010 1.00 0.00

ATOM	1033	H	ILE	67	3.805	-4.635	-3.921	1.00	0.00
ATOM	1034	CA	ILE	67	3.638	-2.608	-3.304	1.00	0.00
ATOM	1035	HA	ILE	67	3.058	-1.943	-3.945	1.00	0.00
ATOM	1036	CB	ILE	67	2.760	-3.111	-2.167	1.00	0.00
ATOM	1037	HB	ILE	67	3.299	-3.880	-1.616	1.00	0.00
ATOM	1038	CG2	ILE	67	2.400	-1.954	-1.215	1.00	0.00
ATOM	1039	HG21	ILE	67	1.861	-1.183	-1.767	1.00	0.00
ATOM	1040	HG22	ILE	67	1.772	-2.329	-0.408	1.00	0.00
ATOM	1041	HG23	ILE	67	3.313	-1.529	-0.798	1.00	0.00
ATOM	1042	CG1	ILE	67	1.530	-3.841	-2.740	1.00	0.00
ATOM	1043	HG12	ILE	67	1.460	-3.604	-3.802	1.00	0.00
ATOM	1044	HG13	ILE	67	1.684	-4.913	-2.617	1.00	0.00
ATOM	1045	CD1	ILE	67	0.238	-3.430	-2.041	1.00	0.00
ATOM	1046	HD11	ILE	67	0.083	-2.358	-2.165	1.00	0.00
ATOM	1047	HD12	ILE	67	-0.601	-3.970	-2.480	1.00	0.00
ATOM	1048	HD13	ILE	67	0.308	-3.666	-0.980	1.00	0.00
ATOM	1049	C	ILE	67	4.725	-1.710	-2.736	1.00	0.00
ATOM	1050	O	ILE	67	4.755	-0.542	-3.006	1.00	0.00
ATOM	1051	N	MET	68	5.677	-2.213	-1.983	1.00	0.00
ATOM	1052	H	MET	68	5.648	-3.193	-1.741	1.00	0.00
ATOM	1053	CA	MET	68	6.776	-1.409	-1.471	1.00	0.00
ATOM	1054	HA	MET	68	6.437	-0.759	-0.664	1.00	0.00

ATOM 1055 CB MET 68 7.838 -2.339 -0.929 1.00 0.00
ATOM 1056 HB2 MET 68 8.162 -3.001 -1.732 1.00 0.00
ATOM 1057 HB3 MET 68 8.683 -1.740 -0.589 1.00 0.00
ATOM 1058 CG MET 68 7.306 -3.171 0.231 1.00 0.00
ATOM 1059 HG2 MET 68 7.515 -2.654 1.167 1.00 0.00
ATOM 1060 HG3 MET 68 6.229 -3.296 0.117 1.00 0.00
ATOM 1061 SD MET 68 8.094 -4.799 0.261 1.00 0.00
ATOM 1062 CE MET 68 6.925 -5.662 1.329 1.00 0.00
ATOM 1063 HE1 MET 68 5.936 -5.649 0.870 1.00 0.00
ATOM 1064 HE2 MET 68 7.249 -6.693 1.464 1.00 0.00
ATOM 1065 HE3 MET 68 6.882 -5.165 2.298 1.00 0.00
ATOM 1066 C MET 68 7.400 -0.524 -2.534 1.00 0.00
ATOM 1067 O MET 68 7.289 0.694 -2.388 1.00 0.00
ATOM 1068 N MET 69 7.854 -1.145 -3.636 1.00 0.00
ATOM 1069 H MET 69 7.883 -2.154 -3.643 1.00 0.00
ATOM 1070 CA MET 69 8.356 -0.438 -4.792 1.00 0.00
ATOM 1071 HA MET 69 9.230 0.155 -4.523 1.00 0.00
ATOM 1072 CB MET 69 8.759 -1.439 -5.863 1.00 0.00
ATOM 1073 HB2 MET 69 8.029 -2.248 -5.869 1.00 0.00
ATOM 1074 HB3 MET 69 8.747 -0.932 -6.829 1.00 0.00
ATOM 1075 CG MET 69 10.139 -2.006 -5.608 1.00 0.00
ATOM 1076 HG2 MET 69 10.177 -2.424 -4.603 1.00 0.00

ATOM	1077	HG3	MET	69	10.346	-2.791	-6.336	1.00	0.00
ATOM	1078	SD	MET	69	11.391	-0.709	-5.760	1.00	0.00
ATOM	1079	CE	MET	69	12.809	-1.596	-5.111	1.00	0.00
ATOM	1080	HE1	MET	69	13.000	-2.476	-5.725	1.00	0.00
ATOM	1081	HE2	MET	69	13.684	-0.945	-5.128	1.00	0.00
ATOM	1082	HE3	MET	69	12.607	-1.905	-4.086	1.00	0.00
ATOM	1083	C	MET	69	7.376	0.566	-5.393	1.00	0.00
ATOM	1084	O	MET	69	7.777	1.594	-5.930	1.00	0.00
ATOM	1085	N	MET	70	6.085	0.295	-5.301	1.00	0.00
ATOM	1086	H	MET	70	5.813	-0.608	-4.942	1.00	0.00
ATOM	1087	CA	MET	70	5.054	1.192	-5.736	1.00	0.00
ATOM	1088	HA	MET	70	5.100	1.338	-6.816	1.00	0.00
ATOM	1089	CB	MET	70	3.705	0.587	-5.384	1.00	0.00
ATOM	1090	HB2	MET	70	3.086	0.664	-6.278	1.00	0.00
ATOM	1091	HB3	MET	70	3.884	-0.463	-5.156	1.00	0.00
ATOM	1092	CG	MET	70	2.988	1.246	-4.212	1.00	0.00
ATOM	1093	HG2	MET	70	3.744	1.426	-3.448	1.00	0.00
ATOM	1094	HG3	MET	70	2.608	2.200	-4.578	1.00	0.00
ATOM	1095	SD	MET	70	1.637	0.307	-3.497	1.00	0.00
ATOM	1096	CE	MET	70	0.731	1.619	-2.711	1.00	0.00
ATOM	1097	HE1	MET	70	0.417	2.345	-3.462	1.00	0.00
ATOM	1098	HE2	MET	70	-0.148	1.208	-2.215	1.00	0.00

ATOM 1099 HE3 MET 70 1.367 2.111 -1.975 1.00 0.00
ATOM 1100 C MET 70 5.205 2.568 -5.121 1.00 0.00
ATOM 1101 O MET 70 4.869 3.555 -5.757 1.00 0.00
ATOM 1102 N LEU 71 5.631 2.618 -3.856 1.00 0.00
ATOM 1103 H LEU 71 5.776 1.747 -3.366 1.00 0.00
ATOM 1104 CA LEU 71 5.783 3.852 -3.120 1.00 0.00
ATOM 1105 HA LEU 71 5.326 4.670 -3.677 1.00 0.00
ATOM 1106 CB LEU 71 5.108 3.755 -1.752 1.00 0.00
ATOM 1107 HB2 LEU 71 5.581 2.883 -1.301 1.00 0.00
ATOM 1108 HB3 LEU 71 5.340 4.641 -1.161 1.00 0.00
ATOM 1109 CG LEU 71 3.602 3.551 -1.790 1.00 0.00
ATOM 1110 HG LEU 71 3.385 2.511 -2.033 1.00 0.00
ATOM 1111 CD1 LEU 71 3.009 3.887 -0.449 1.00 0.00
ATOM 1112 HD11 LEU 71 3.226 4.928 -0.206 1.00 0.00
ATOM 1113 HD12 LEU 71 1.930 3.740 -0.482 1.00 0.00
ATOM 1114 HD13 LEU 71 3.442 3.239 0.312 1.00 0.00
ATOM 1115 CD2 LEU 71 2.904 4.338 -2.893 1.00 0.00
ATOM 1116 HD21 LEU 71 3.297 4.033 -3.863 1.00 0.00
ATOM 1117 HD22 LEU 71 1.832 4.142 -2.858 1.00 0.00
ATOM 1118 HD23 LEU 71 3.083 5.404 -2.749 1.00 0.00
ATOM 1119 C LEU 71 7.259 4.178 -3.059 1.00 0.00
ATOM 1120 O LEU 71 7.739 4.722 -2.073 1.00 0.00

ATOM 1121 N ASN 72 8.006 3.777 -4.075 1.00 0.00

ATOM 1122 H ASN 72 7.567 3.214 -4.790 1.00 0.00

ATOM 1123 CA ASN 72 9.416 4.067 -4.201 1.00 0.00

ATOM 1124 HA ASN 72 9.736 4.745 -3.410 1.00 0.00

ATOM 1125 CB ASN 72 10.211 2.773 -4.084 1.00 0.00

ATOM 1126 HB2 ASN 72 9.924 1.978 -4.772 1.00 0.00

ATOM 1127 HB3 ASN 72 11.224 3.104 -4.314 1.00 0.00

ATOM 1128 CG ASN 72 10.190 2.226 -2.679 1.00 0.00

ATOM 1129 OD1 ASN 72 10.024 1.057 -2.414 1.00 0.00

ATOM 1130 ND2 ASN 72 10.529 3.037 -1.710 1.00 0.00

ATOM 1131 HD21 ASN 72 10.527 2.707 -0.756 1.00 0.00

ATOM 1132 HD22 ASN 72 10.791 3.990 -1.921 1.00 0.00

ATOM 1133 C ASN 72 9.717 4.746 -5.523 1.00 0.00

ATOM 1134 O ASN 72 10.881 4.987 -5.832 1.00 0.00

ATOM 1135 N GLU 73 8.675 4.997 -6.311 1.00 0.00

ATOM 1136 H GLU 73 7.756 4.678 -6.040 1.00 0.00

ATOM 1137 CA GLU 73 8.825 5.629 -7.587 1.00 0.00

ATOM 1138 HA GLU 73 9.595 5.124 -8.171 1.00 0.00

ATOM 1139 CB GLU 73 7.496 5.556 -8.359 1.00 0.00

ATOM 1140 HB2 GLU 73 6.734 5.115 -7.717 1.00 0.00

ATOM 1141 HB3 GLU 73 7.193 6.564 -8.642 1.00 0.00

ATOM 1142 CG GLU 73 7.660 4.694 -9.627 1.00 0.00

ATOM	1143	HG2	GLU	73	8.695	4.565	-9.943	1.00	0.00
ATOM	1144	HG3	GLU	73	7.256	3.734	-9.306	1.00	0.00
ATOM	1145	CD	GLU	73	6.832	5.229	-10.799	1.00	0.00
ATOM	1146	OE1	GLU	73	5.698	5.690	-10.533	1.00	0.00
ATOM	1147	OE2	GLU	73	7.399	5.299	-11.912	1.00	0.00
ATOM	1148	C	GLU	73	9.322	7.066	-7.431	1.00	0.00
ATOM	1149	O	GLU	73	9.447	7.594	-6.313	1.00	0.00
ATOM	1150	N	PRO	74	9.547	7.758	-8.558	1.00	0.00
ATOM	1151	CD	PRO	74	9.391	7.272	-9.921	1.00	0.00
ATOM	1152	HD2	PRO	74	8.327	7.087	-10.070	1.00	0.00
ATOM	1153	HD3	PRO	74	9.936	6.331	-9.984	1.00	0.00
ATOM	1154	CG	PRO	74	9.935	8.317	-10.868	1.00	0.00
ATOM	1155	HG2	PRO	74	9.130	8.699	-11.497	1.00	0.00
ATOM	1156	HG3	PRO	74	10.708	7.876	-11.498	1.00	0.00
ATOM	1157	CB	PRO	74	10.514	9.410	-9.983	1.00	0.00
ATOM	1158	HB2	PRO	74	10.198	10.394	-10.330	1.00	0.00
ATOM	1159	HB3	PRO	74	11.603	9.362	-9.974	1.00	0.00
ATOM	1160	CA	PRO	74	9.964	9.152	-8.569	1.00	0.00
ATOM	1161	HA	PRO	74	10.784	9.265	-7.861	1.00	0.00
ATOM	1162	C	PRO	74	8.825	10.104	-8.187	1.00	0.00
ATOM	1163	O	PRO	74	8.664	11.174	-8.774	1.00	0.00
ATOM	1164	N	MET	75	8.050	9.735	-7.175	1.00	0.00

ATOM	1165	H	MET	75	8.244	8.854	-6.722	1.00	0.00
ATOM	1166	CA	MET	75	6.942	10.516	-6.701	1.00	0.00
ATOM	1167	HA	MET	75	7.263	11.526	-6.445	1.00	0.00
ATOM	1168	CB	MET	75	5.896	10.603	-7.814	1.00	0.00
ATOM	1169	HB2	MET	75	6.394	10.528	-8.781	1.00	0.00
ATOM	1170	HB3	MET	75	5.187	9.783	-7.706	1.00	0.00
ATOM	1171	CG	MET	75	5.154	11.928	-7.728	1.00	0.00
ATOM	1172	HG2	MET	75	5.410	12.419	-6.789	1.00	0.00
ATOM	1173	HG3	MET	75	5.458	12.561	-8.563	1.00	0.00
ATOM	1174	SD	MET	75	3.359	11.672	-7.797	1.00	0.00
ATOM	1175	CE	MET	75	2.955	12.845	-9.100	1.00	0.00
ATOM	1176	HE1	MET	75	3.484	12.572	-10.013	1.00	0.00
ATOM	1177	HE2	MET	75	1.881	12.829	-9.285	1.00	0.00
ATOM	1178	HE3	MET	75	3.255	13.847	-8.794	1.00	0.00
ATOM	1179	C	MET	75	6.336	9.872	-5.462	1.00	0.00
ATOM	1180	O	MET	75	5.130	9.921	-5.266	1.00	0.00
ATOM	1181	N	TRP	76	7.147	9.209	-4.647	1.00	0.00
ATOM	1182	H	TRP	76	8.114	9.087	-4.912	1.00	0.00
ATOM	1183	CA	TRP	76	6.682	8.572	-3.436	1.00	0.00
ATOM	1184	HA	TRP	76	5.857	9.139	-3.006	1.00	0.00
ATOM	1185	CB	TRP	76	6.193	7.163	-3.753	1.00	0.00
ATOM	1186	HB2	TRP	76	6.868	6.731	-4.493	1.00	0.00

ATOM	1187	HB3	TRP	76	6.182	6.536	-2.862	1.00	0.00
ATOM	1188	CG	TRP	76	4.818	7.099	-4.339	1.00	0.00
ATOM	1189	CD1	TRP	76	4.438	6.543	-5.506	1.00	0.00
ATOM	1190	HD1	TRP	76	5.213	6.068	-6.108	1.00	0.00
ATOM	1191	NE1	TRP	76	3.082	6.712	-5.683	1.00	0.00
ATOM	1192	HE1	TRP	76	2.583	6.349	-6.483	1.00	0.00
ATOM	1193	CE2	TRP	76	2.524	7.407	-4.649	1.00	0.00
ATOM	1194	CZ2	TRP	76	1.229	7.818	-4.370	1.00	0.00
ATOM	1195	HZ2	TRP	76	0.443	7.588	-5.089	1.00	0.00
ATOM	1196	CH2	TRP	76	1.017	8.507	-3.175	1.00	0.00
ATOM	1197	HH2	TRP	76	0.007	8.833	-2.928	1.00	0.00
ATOM	1198	CZ3	TRP	76	2.054	8.792	-2.290	1.00	0.00
ATOM	1199	HZ3	TRP	76	1.891	9.346	-1.366	1.00	0.00
ATOM	1200	CE3	TRP	76	3.347	8.366	-2.577	1.00	0.00
ATOM	1201	HE3	TRP	76	4.112	8.571	-1.829	1.00	0.00
ATOM	1202	CD2	TRP	76	3.611	7.671	-3.775	1.00	0.00
ATOM	1203	C	TRP	76	7.786	8.597	-2.402	1.00	0.00
ATOM	1204	O	TRP	76	8.557	7.649	-2.270	1.00	0.00
ATOM	1205	N	ILE	77	7.909	9.716	-1.685	1.00	0.00
ATOM	1206	H	ILE	77	7.254	10.467	-1.850	1.00	0.00
ATOM	1207	CA	ILE	77	9.011	9.949	-0.751	1.00	0.00
ATOM	1208	HA	ILE	77	9.611	9.051	-0.607	1.00	0.00

ATOM 1209 CB ILE 77 9.926 11.115 -1.165 1.00 0.00
ATOM 1210 HB ILE 77 9.506 12.053 -0.800 1.00 0.00
ATOM 1211 CG2 ILE 77 11.328 10.912 -0.563 1.00 0.00
ATOM 1212 HG21 ILE 77 11.749 9.976 -0.929 1.00 0.00
ATOM 1213 HG22 ILE 77 11.974 11.740 -0.858 1.00 0.00
ATOM 1214 HG23 ILE 77 11.256 10.878 0.524 1.00 0.00
ATOM 1215 CG1 ILE 77 9.978 11.308 -2.682 1.00 0.00
ATOM 1216 HG12 ILE 77 8.992 11.633 -3.014 1.00 0.00
ATOM 1217 HG13 ILE 77 10.708 12.088 -2.898 1.00 0.00
ATOM 1218 CD1 ILE 77 10.368 10.027 -3.421 1.00 0.00
ATOM 1219 HD11 ILE 77 9.638 9.247 -3.207 1.00 0.00
ATOM 1220 HD12 ILE 77 10.390 10.219 -4.494 1.00 0.00
ATOM 1221 HD13 ILE 77 11.354 9.702 -3.090 1.00 0.00
ATOM 1222 C ILE 77 8.422 10.141 0.616 1.00 0.00
ATOM 1223 O ILE 77 7.483 9.469 0.980 1.00 0.00
ATOM 1224 N ARG 78 8.919 11.053 1.421 1.00 0.00
ATOM 1225 H ARG 78 9.701 11.599 1.089 1.00 0.00
ATOM 1226 CA ARG 78 8.392 11.341 2.726 1.00 0.00
ATOM 1227 HA ARG 78 8.614 10.513 3.398 1.00 0.00
ATOM 1228 CB ARG 78 9.004 12.607 3.298 1.00 0.00
ATOM 1229 HB2 ARG 78 9.264 13.279 2.479 1.00 0.00
ATOM 1230 HB3 ARG 78 8.276 13.093 3.948 1.00 0.00

ATOM	1231	CG	ARG	78	10.260	12.273	4.100	1.00	0.00
ATOM	1232	HG2	ARG	78	10.249	12.844	5.028	1.00	0.00
ATOM	1233	HG3	ARG	78	10.260	11.208	4.328	1.00	0.00
ATOM	1234	CD	ARG	78	11.517	12.623	3.303	1.00	0.00
ATOM	1235	HD2	ARG	78	11.435	12.214	2.296	1.00	0.00
ATOM	1236	HD3	ARG	78	11.622	13.707	3.246	1.00	0.00
ATOM	1237	NE	ARG	78	12.718	12.066	3.941	1.00	0.00
ATOM	1238	HE	ARG	78	12.568	11.551	4.796	1.00	0.00
ATOM	1239	CZ	ARG	78	13.954	12.218	3.506	1.00	0.00
ATOM	1240	NH1	ARG	78	14.198	12.882	2.406	1.00	0.00
ATOM	1241	HH11	ARG	78	13.434	13.284	1.882	1.00	0.00
ATOM	1242	HH12	ARG	78	15.150	12.990	2.085	1.00	0.00
ATOM	1243	NH2	ARG	78	14.954	11.673	4.149	1.00	0.00
ATOM	1244	HH21	ARG	78	14.779	11.133	4.983	1.00	0.00
ATOM	1245	HH22	ARG	78	15.897	11.796	3.807	1.00	0.00
ATOM	1246	C	ARG	78	6.913	11.581	2.708	1.00	0.00
ATOM	1247	O	ARG	78	6.310	11.162	3.668	1.00	0.00
[truncated: 807,837 more chars]
